# Priming by High Temperature Stress Induces MicroRNA Regulated Heat Shock Modules Indicating Their Involvement in Thermopriming Response in Rice

**DOI:** 10.3390/life11040291

**Published:** 2021-03-29

**Authors:** Akhilesh Kumar Kushawaha, Ambreen Khan, Sudhir Kumar Sopory, Neeti Sanan-Mishra

**Affiliations:** Plant RNAi Biology Group, International Centre for Genetic Engineering and Biotechnology, Aruna Asaf Ali Marg, New Delhi 110067, India; akhilesh2203@gmail.com (A.K.K.); ambreenkhan@icgeb.res.in (A.K.); sopory@icgeb.res.in (S.K.S.)

**Keywords:** high temperature stress, thermo-priming, RNA-seq, microRNA, HSF, HSP, interactome

## Abstract

Rice plants often encounter high temperature stress, but the associated coping strategies are poorly understood. It is known that a prior shorter exposure to high temperature, called thermo-priming, generally results in better adaptation of the plants to subsequent exposure to high temperature stress. High throughput sequencing of transcript and small RNA libraries of rice seedlings primed with short exposure to high temperature followed by high temperature stress and from plants exposed to high temperature without priming was performed. This identified a number of transcripts and microRNAs (miRs) that are induced or down regulated. Among them osa-miR531b, osa-miR5149, osa-miR168a-5p, osa-miR1846d-5p, osa-miR5077, osa-miR156b-3p, osa-miR167e-3p and their respective targets, coding for heat shock activators and repressors, showed differential expression between primed and non-primed plants. These findings were further validated by qRT-PCR. The results indicate that the miR-regulated heat shock proteins (HSPs)/heat shock transcription factors (HSFs) may serve as important regulatory nodes which are induced during thermo-priming for plant survival and development under high temperatures.

## 1. Introduction

Environmental stresses negatively impact plant growth and development by affecting molecular functions and cellular homeostasis [1]. Amongst the abiotic factors, increase in temperature due to global warming is a critical parameter in limiting biological processes and yields in plant [2,3,4]. Transiently increased temperature was found to induce male sterility in wheat, rice and maize [5,6,7]. Exposure to high temperature stress (HTS) causes protein denaturation, elevates lipid peroxidation, disturbs membrane fluidity and generates reactive oxygen species [8,9,10,11,12]. These changes disturb the normal physiological processes and may even lead to plant death.

Plants have an efficient network of receptors, signaling components, enzymes and transcription factors that play a key role in perception and acclimatization to these changes in the environment by rapidly reprogramming genetic machinery [1,12,13]. The response to HTS is mediated by a set of heat shock transcription factors (HSFs) that bind to specific heat shock elements (HSEs) found within the upstream regions of all high temperature (HT) responsive genes [14], including those coding for the heat shock proteins (HSPs) [15,16]. The HSFs are well characterized and have been extensively studied in many plants [17,18,19,20].

Several studies have also indicated that microRNAs (miRs) also play an important role in modifying the plant gene expression in response to abiotic and biotic stresses [21,22,23,24,25]. The miRs act in a sequence specific manner to regulate the expression of transcription factors and other genes at the transcriptional and post-transcriptional levels [21,25]. Small RNA analysis following HTS in Arabidopsis, populus, mustard, wheat, switch grass, cavendish banana, tomato and French beans has displayed the differential expression of numerous conserved and novel miRs [23,24,25,26,27,28,29,30]. Individual miRs may control multiple biological responses in response to diverse stresses. For instance, miR531 plays an important role in drought response and innate immunity in rice [31], biomass improvement in sugarcane [31] and metabolism in switch grass [32]. The miR156 family regulates the auxin signaling pathway, thereby playing a role in lateral root development, leaf morphology, juvenile to vegetative development changes and the overall plant architecture in Arabidopsis, maize, rice and wheat [33,34,35,36]. The miR167 negatively regulates ARF6 and ARF8 gene expression and plays an important role in the flower development, reproductive growth, adventitious rooting and nodulation under the biotic and abiotic stress in plants [37,38]. Recently, it was shown that HTS enhances the synthesis of vitamin E in chloroplast to positively regulate miR398 biogenesis by retrograde signaling, which provides protection to the Arabidopsis plants against increasing environmental temperatures [39].

Priming involves a relatively shorter exposure to abiotic stress, for instance heat and light or chemical treatments like beta-aminobutyric acids, followed by a lag/memory phase before onset of the stress condition. This treatment results in better adaptation of plants when subsequently exposed to corresponding stresses [40]. Recent research into the phenomena and molecular basis of thermo-priming has received increasing attention [40,41,42] as limited exposure to HT helps plants to acquire memory for withstanding subsequent HTS [43,44]. The molecular mechanisms underlying thermo-priming and/or memory are still largely unknown in plants. A role for miR156:SPL has been implicated in HTS memory [45]. We had earlier reported that the miR169:NFY module may be important for integrating stress memory induced during HT priming with light-regulated development [46,47]. In this study, we report the involvement of crucial miR regulated HSF/HSP genetic modules that are affected during thermo-priming in rice.

## 2. Materials and Methods

### 2.1. Plant Materials and Growth Conditions

The heat-susceptible rice variety Pusa Basmati 1 (PB1) was used for this study. Seeds were surface sterilized using 0.1% HgCl_2_ and Teepol for 10 min, followed by washing with distilled water. Seeds were soaked overnight in water and then placed on germination sheets. Seedlings were allowed to grow for two weeks in growth chamber at 28 ± 2 °C. The light intensity of 100 µmol m^−2^ s^−1^ was used with a 16/8-h photoperiod. The mature rice plants were also grown in controlled greenhouse conditions at 28 ± 2 °C under a 16/8 h photoperiod.

### 2.2. Heat Stress Priming and High Temperature Treatments

**Figure 1:**
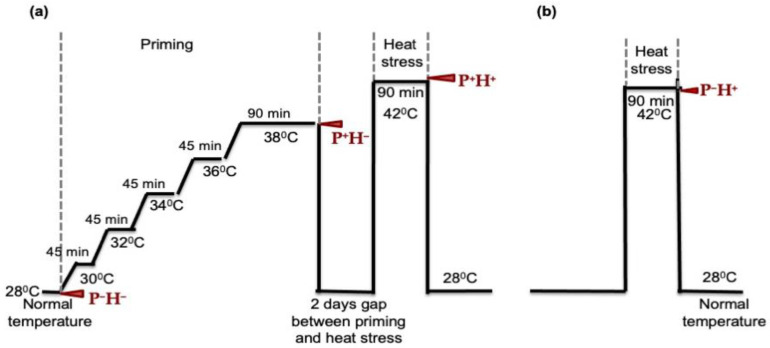
Treatment protocol for high temperature (HT) priming and stress provided to four groups of 15-day-old rice plants. Arrows indicate the time points at which the samples were harvested for each set. (**a**) HT priming was done by gradually increasing the temperature from 28 °C to 38 °C in steps of 45 min each and keeping at 38 °C for 90 min. This was followed by reverting the plants to 28 °C for two days and then providing high temperature stress (HTS) at 42 °C for 90 min. Plants growing at 28 °C served as the control (P^−^H^−^) for each set. The second group was only primed with HT (P^+^H^−^). The third group was primed with HT and then exposed to HTS (P^+^H^+^). (**b**) The fourth group of plants were directly exposed to HTS at 42 °C for 90 min (P^−^H^+^). Each experiment was performed in triplicate using three batches of plants in each group.

The HT priming treatments were provided to rice seedlings as described in Figure 1. Briefly, 15 days old rice seedlings of uniform height were divided into four different groups. One group of plants (P^−^H^−^) was kept at 28 °C to serve as a control. Two groups of plants (P^+^H^−^ and P^+^H^+^) were primed to HTS by exposing them to steadily increasing temperature from 28 °C to 36 °C for 45 min each and then keeping them at 38 °C for 90 min. After 90 min, one group (P^+^H^−^) was harvested. The other group of primed plants (P^+^H^+^) were kept at 28 °C for two days and then exposed to 42 °C for 90 min. The fourth group of plants (P^−^H^+^) were directly exposed to 42 °C for 90 min. After thermo-priming and stress treatments, leaf tissues were harvested in liquid nitrogen and stored at −80 °C. Each experiment was performed using three biological replicates with 15 plants in each set.

Mature plants were divided into four groups. One group was thermo-primed at 38 °C for 90 min before anthesis (PBH) and another group after anthesis (PAH). After priming the plants were returned to normal conditions (28 ± 2 °C, 16/8 h cycle) for two days and then exposed to HTS at 42 °C for 90 min. The remaining two sets were exposed to HTS directly (without priming). These plants represented the sets named as non-primed before anthesis (NBH) and non-primed after anthesis (NAH), respectively. The experiments were performed in triplicates using five mature plants for each treatment.

### 2.3. RNA-seq Data Analysis

Total RNA was extracted from leaf tissues using the Trizol method (Sigma-Aldrich, St. Louis, MO, USA), according to the manufacture’s protocol. The extracted RNA with an RNA integrity number (RIN) of 7.0 was used for mRNA purification, using oligo-dT beads (Tru Seq RNA Sample Preparation Kit, Illumina). The transcriptome libraries were constructed and sequenced on Illumina platform by Bionivid Technology Pvt Ltd., Bangalore, India. The sequence data of all the samples were deposited at NCBI.

For analysis of the sequencing reads (Figure S1), stringent quality control of paired end sequence reads was performed using the NGSQC Tool kit [48]. Paired end sequence reads with a Phred score > Q30 were taken for further analysis. *Oryza sativa* genome from (IRGSP-1.0) was used for read alignment and identification of transcripts. The TopHat [49] pipeline was used for alignment and the Cufflink and Cuffdiff pipelines [50] were used for identification of transcript coding regions followed by quantitation and annotation using default parameters. Treated samples were compared with the control samples by the Cuffdiff data analysis pipeline and Cuffdiff transcripts with log_2_ fold change ≥ 2 and *p*-value ≤ 0.05 were considered as significantly differential expressed. Unsupervised hierarchical clustering of differentially expressed transcripts was done using Cluster 3.0 [51] and visualized using Java Tree View v1.1.6 [52]. The statistical analyses were performed by student’s *t*-test.

### 2.4. Small RNA Data Analysis

Small RNA was enriched using protocol described earlier [53] and an equal amount of samples from three replications were pooled for library preparation. Library preparation and sequencing was outsourced to Bionivid Technology Pvt Ltd. (Bangalore, India). The sequence data of all the samples were deposited at NCBI.

For analysis the sequencing reads (Figure S1), raw data were subjected to QC using the NGSQC Tool kit (v2.3.3). QC-filtered files were subjected to adapter trimming using Cutadapt (v1.10) [54], based on quality score (80% of bases with Phred score ≥ 30) and indicating the sequence TGGAATTCTCGGGTGCCAAGG as the adapter. The output of this filtering was used for downstream analysis. Number of unique tags, their read counts, FASTA sequence and tag length distribution were fetched using the PERL and AWK scripts. Unique tags were subjected to BLASTn against the RFam database with word size (W) 7. The best hits were selected using filtering criteria of 100% query coverage, 2 mismatches and 0 gaps. The unique tags, which did not hit the RFam database were considered for miR analysis. miRDeep2 [55] was used for known miR identification and quantification using default parameters.

Differential expression analysis (treated vs. control) was performed using the DESeq R package [56]. This package provides a method to test for differential expression by using negative binomial distribution. The plant small RNA target Analysis (psRNA) online server [57] was used for target identification. Known miR sequences were used as the query and transcripts from RNA-seq data were used as the target.

### 2.5. Biological Pathways and Gene Ontology Enrichment Analysis

Gene ontologies (GO) and Kyoto Encyclopaedia of Genes and Genomes (KEGG) pathways that harbor significantly expressed transcripts were identified using Blast2GO [58]. Top 10 GO categories were graphically represented using the WEGO tool [59]. GO ontologies and pathways related to HSPs and HTS along with the log_2_ fold change values of transcripts and miRs were used as input for bridge island software (Bionivid Technology Pvt Ltd., Bangalore, India) for identifying key connecting edges. Statistical scores from expression and biological analysis were used as attributes to visualize the network. The output of the Bridge Island Software was used as input to CytoScape V 2.8.3. A force directed spring embedded layout algorithm was used to visualize the network that encompasses biological categories and miRs targeting genes.

### 2.6. qRT-PCR Analysis

Real-time PCR was used to investigate the expression level of miR and its respective targets as described earlier [47]. Primers pairs were designed using primer 3 Web (v.4.1.0) (Table S1). Briefly, total RNA was used to synthesize the cDNA using specific primers with high capacity cDNA kit (Applied Biosystem, USA). qRT-PCR was performed for at least three experimental replicates for each data set using the SYBR Green Master Mix (Applied Biosystem, USA) according to the manufacture’s recommendations. The relative expression level of miRs and targets was calculated using ΔΔCt method [60]. The internal control 18S was used. Standard deviation (SD) values were calculated and plotted.

## 3. Results

To understand the effect of HT priming on rice plants, the seedlings exposed to HTS, as explained in Figure 1, were subjected to RNA-seq for transcriptome and small RNA analysis (Table 1). The data sets obtained using rice seedlings only primed with HT (P^+^H^−^), rice seedlings subjected to HTS after thermo-priming (P^+^H^+^) and rice seedlings subjected to HTS without priming (P^−^H^+^), were compared with seedlings grown under normal conditions (P^−^H^−^).

### 3.1. Differential Expression Patterns Between Primed and Non-Primed Plants

The overall summary of the transcriptome data is presented in Table 1. An average of 25.5 million reads was obtained which provided an average of 24.0 million high quality reads per rice cDNA library (Table 1). Next generation sequencing (NGS) analysis identified a combined total of 5136 DETs in the 3 treatments with respect to P^−^H^−^ (control). When compared with P^−^H^−^, the highest number of transcripts and differentially expressed genes/transcripts (DETs) was seen in P^+^H^+^ (priming followed by HTS), followed by P^−^H^+^ (HTS without priming) and P^+^H^−^ (priming without HTS), respectively. In P^+^H^+^ and P^−^H^+^ a greater number of transcripts were down regulated, while in P^+^H^−^ a higher number of transcripts were up regulated. This shows that priming treatment alone does cause changes in the transcript profiles.

**Figure 2:**
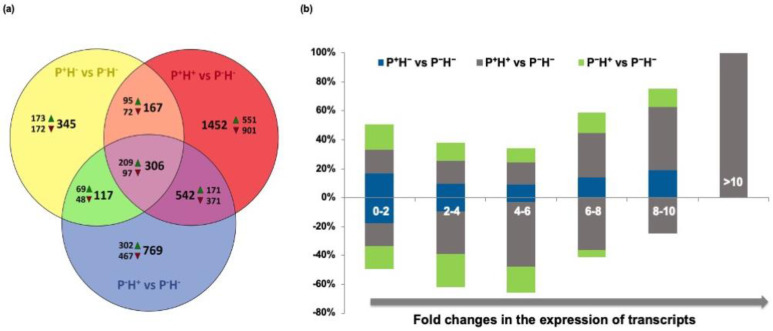
Representation of differentially expressed transcripts (DETs) in datasets obtained from rice seedlings only primed with HT (P^+^H^−^), thermo-priming followed by HTS (P^+^H^+^) and plants exposed directly to HTS without thermo-priming (P^−^H^+^). In each case, the differential expression was calculated with respect to control non-primed plants (P^−^H^−^). (**a**) Venn diagram to show the number of unique and overlapping DETs. The number of transcripts that are up regulated (shown by green triangles) or down regulated (shown by red triangles) at least two-fold are also shown. (**b**) The comparison of fold deregulations with the number of DETs represented as percentage stacked columns.

To identify the global changes in the transcriptome across all four data sets, a Venn diagram of deregulated transcripts was created (Figure 2A). This showed that, among the 935 DETs present in P^+^H^−^, 306 DETs (209 up regulated and 97 down regulated) were common to all data sets. A total of 345 DETs (173 up regulated and 172 down regulated) were specific to P^+^H^−^ while 167 DETs (95 up regulated and 72 down regulated) were common between P^+^H^−^ and P^+^H^+^ (priming followed by HTS) and 117 DETs (69 up regulated and 48 down regulated) were common between P^+^H^−^ and P^−^H^+^.

To understand the alterations in expression profiles, the DETs were categorized on the basis of their fold change values (Figure 2B). It was observed that the percentage of miRs showing up to four-fold change in expression pattern gradually increased from P^+^H^−^ to P^+^H^+^ to P^−^H^+^. In P^+^H^−^ and P^−^H^+^ the transcripts did not show >6-fold and >8-fold down regulation, respectively. In P^+^H^+^, greater number of transcripts showed 4–6-fold down regulation and >8-fold up regulation (Figure 2B).

#### 3.1.1. Effect of High Temperature Stress on Primed Transcript Profiles

To understand the significance of HTS after priming, the profiles of DETs in P^+^H^−^ (priming without HTS) were compared with those in P^+^H^+^ (priming followed by HTS) data sets to observe the changes in expression patterns (Table S2).

This analysis showed that among the 7723 P^+^H^−^ DETs showing up to 2-fold up regulation, 10 transcripts including that coding for HSP17.8 (XP_006647786.1) showed 4–6-fold up regulation in the P^+^H^+^ data sets. Meanwhile, 399 P^+^H^−^ DETs showed 2–4-fold up regulation in P^+^H^+^. These included transcripts coding for HSP70-15 (XP_006643833.1), mitochondrial HSP70 (XP_006661382.1), mitochondrial HSP90-6 (XP_015698315.1), chloroplastic HSP90-5 (XP_004957103.1 and XP_004973760.1), chloroplastic HSP70 (XP_008644594.1), HSFA-9 (XP_015690243.1) and HSFA-4d (XP_006654692.1). Several P^+^H^−^ DETs were also down regulated in P^+^H^+^ as, 6 transcripts showed >6-fold down regulation, 46 transcripts showed 4–6-fold down regulation while 238 transcripts showed 2–4-fold down regulation in P^+^H^+^. A total of 2263 P^+^H^−^ transcripts showed up to 2-fold down regulation in P^+^H^+^. These included HSFA-5 (XP_006647286.1), HSFA-2d (XP_006649439.2), DNAJ-HSP isoform X2 (XP_020403152.1) and class V HSP18.8 (XP_003560151.1).

In P^+^H^−^, 370 transcripts showed 2–4-fold up regulation and within these 120 transcripts were 0–2-fold up regulated, and 41 transcripts were 4–7-fold up regulated in P^+^H^+^. The ones with >4-fold up regulated transcripts included the class I HSP3 (NP_001130454.1), activator of HSP90 ATPase homolog (XP_006659483.1), HSP70-8 (XP_006649645.1), mitochondrial HSP26.2 (XP_015693506.1), mitochondrial HSP70 (XP_014752657.1) and heat-stress-associated (HSA) 32 kDa protein (XP_006656391.1). Among P^+^H^−^ DETs, 13 were 0–2-fold down regulated and 4 DETs were 2–4-fold down regulated in P^+^H^+^.

Within the 43 DETs that were 4–6-fold up regulated in P^+^H^−^, 6-transcripts showed 2–4-fold up regulation, 6 transcripts showed 6–8-fold up regulation and 6-transcripts showed 8–10-fold up regulation in P^+^H^+^ while 3 transcripts showed up to 2-fold up regulation in P^+^H^+^. The >6-fold DETs comprised chloroplastic HSP26.7 (XP_015690159.1), class I HSP17.9 (XP_004984667.1), class I HSP17.9 isoform X2 (XP_006649810.1), class I 16.9kDa HSP3 (XP_006643681.1), HSP70 (XP_006649853.1), class II HSP17.5 (XP_003564488.1), HSP23.2 (XP_021319503.1) and HSP81-1 (XP_006659551.2).

In P^+^H^−^ data sets, 9 DETs showed 6–8-fold up regulation and 3 DETs showed 8–10-fold up regulation. Among these, 1 transcript (NP_001151645.1; coding for ubiquitin-protein ligase CIP8) was up regulated 4–6-fold in P^+^H^+^ while 2 transcripts were up regulated 6–8-fold in P^+^H^+^ and 2 transcripts including HSP83 (XP_006664928.1) were up regulated 8–10-fold in P^+^H^+^.

In P^+^H^−^, 8119 transcripts were up to 2-fold down regulated and many of them showed down regulation in P^+^H^+^ as well. Among these 772 transcripts were 2–4-fold down regulated, 116 genes were 4–6-fold down regulated (including HSP23.2; XP_006664764.1), 15 transcripts were 6–10-fold down regulated. However, 2511 P^+^H^−^ transcripts showed up to 2-fold up regulation and 34 transcripts showed 2–4-fold up regulation (including cognate HSP70; XP_010041882.1) in P^+^H^+^.

From the 376 DETs that were-2–4 fold down regulated in P^+^H^−^, 23 transcripts including chloroplastic small HSP (XP_004983363.1) were not expressed in P^+^H^+^. On the other hand, 39 transcripts showed 4–6-fold down regulation and 5 transcripts showed 6–8-fold down regulation in P^+^H^+^. In contrast, 42 transcripts were 0–2-fold up regulated, and 2 transcripts were 2–4-fold up regulated in P^+^H^+^. A total of 152 transcripts showed up to 2-fold down regulation and this set included HSF C-1b (XP_015689817.1) and HSF C-2b isoform X2 (XP_002438552.1).

Among the DETs, 13 transcripts were 4–6-fold down regulated in P^+^H^−^. A total of 8 of these transcripts showed 2–4-fold down regulation in P^+^H^+^ while 1 transcript (XP_006660483.1; coding for cytochrome P450 containing premnaspirodiene oxygenase) showed >8-fold down regulation.

#### 3.1.2. Effect of Priming on Transcript Profiles

To understand the significance of HT priming on transcripts that co-regulate with HTS response, the profiles of DETs in P^+^H^+^ (priming followed by HTS) were compared with those in P^−^H^+^ (HTS without priming) data sets (Table S2).

This analysis showed that among the 7716 DETs showing up to 2-fold up regulation in P^+^H^+^, 400 DETs including HSP70-2 (XP_006663128.1) and HSP81-1 (XP_004973824.2) showed 2–4-fold up regulated and 9 DETs including HSP17.8 (XP_006647786.1) showed 2–4 fold-up regulation. In P^−^H^+^, 2263 DETs including HSP18.8 (XP_003560151.1), HSF A-2d (XP_006649439.2), HSF-BP1 (XP_006655995.1), HSP-BPX1 (XP_020399314.1) and cognate HSP70-2 (XP_004981251.1) were up to 2-fold down regulated and 238 DETs were 2–4-fold down regulated, while 52 showed >4-fold down regulation.

Among the 370 DETs showing 2–4-fold up regulation in P^+^H^+^, 120 DETs including HSFA-9 (XP_015690243.1), chloroplastic HSP70 (XP_008644594.1) and HSFA-4d (XP_006654692.1) displayed up to 2-fold up regulation in P^−^H^+^, while 42 DETs were >4-fold up regulated in P^−^H^+^ and 17 DETs were down regulated in P^−^H^+.^

A total of 43 DETs showed 4–6-fold up regulation in P^+^H^+^ and 12 DETs showed >6-fold up regulation in P^+^H^+^. This set consisted of a large number of HSPs and HSFs. Among these HSP17.8 (XP_006647786.1) was 2-fold up regulation in P^−^H^+^, while activator of HSP90 ATPase homolog (XP_006659483.1), class III HSP18.6 (XP_004954144.1), HSF B-2a (XP_002446950.1), HSFB-2c (XP_004957414.1), mitochondrial HSP70 (XP_014752657.1), heat-stress-associated (HSA) 32 kDa protein (XP_006656391.1) and class I HSP3 (NP_001130454.1) displayed 2–4-fold up regulation in P^−^H^+^. The HSP81-1 (XP_006659551.2), HSP23.2 (XP_021319503.1), HSF B-2b (XP_006659656.1), HSP17.9 (XP_004984667.1), HSP17.5 (XP_003564488.1), HSP17.9 isoform X2 (XP_006649810.1), cognate HSP70 (XP_006649853.1) and class I 16.9kDa HSP3 (XP_006643681.1) showed 4–6-fold up regulation in P^−^H^+^.

It was seen that 24,351 DETs showed up to 2-fold down regulation in P^+^H^+^ and among these 902 were >2-fold down regulated in P^−^H^+^, while 1766 were up regulated in P^−^H^+^. In P^+^H^+^ 375 DETs showed 2–4-fold down regulation and 468 DETs showed 4–6-fold down regulation among these 91 transcripts were up regulated in P^−^H^+^ including HSF—binding protein 1 (XP_006655995.1).

#### 3.1.3. Comparison of Priming Induced Transcript Profiles with Those in Stressed Tissues

To identify if HT priming and HTS exerted any distinctive effect on the transcripts, the profiles of DETs in P^+^H^−^ (priming without HTS) were compared with those in P^−^H^+^ (HTS without priming) data sets (Table S2).

Comparative analysis of expression pattern of 7723 transcripts showing up to 2-fold up regulation in P^+^H^−^ identified 267 P^−^H^+^ transcripts that were up regulated 2–4-fold and 3 P^−^H^+^ transcripts that were 4–6-fold up regulated. The 2–4-fold up regulated P^−^H^+^ transcripts included HSP90-6 (XP_015698315.1), mitochondrial HSP70 (XP_006661382.1), chloroplastic HSP90-5 (XP_004973760.1), HSP70-2 (XP_006663128.1), 70 kDa HSP-15 isoform X2 (XP_006643833.1) and HSP81-1 (XP_004973824.2). In P^−^H^+^, 6 transcripts were down regulated 4–6-fold, 121 transcripts were down regulated 2–4-fold and 2121 transcripts were down regulated up to 2-fold, including DNAJ HSP isoform X2 (XP_020403152.1) and HSF A-2d (XP_006649439.2).

Among the 370 P^+^H^−^ transcripts showing 2–4-fold up regulation, 146 transcripts showed up to 2-fold up regulation and 16 transcripts showed up to 2-fold down regulation in P^−^H^+^. A total of 19 P^+^H^−^ transcripts were 4–7-fold up regulated in P^−^H^+^, including HSP70-8 (XP_006649645.1) and mitochondrial HSP26.2 (XP_015693506.1).

In P^+^H^−^ data set, 43 transcripts showed 4–6-fold up regulation but 3 of these transcripts were 6–8-fold up regulated in P^−^H^+^, including chloroplastic HSP26.7 (XP_015690159.1) and class I HSP17.9 (XP_004984667.1). While two transcripts showed up to two-fold up regulation and one transcript (XP_004955631.1; coding for early light-inducible protein HV90) was up to two-fold down regulated in P^−^H^+^. A total of 17 P^+^H^−^ transcripts were 2–4-fold up regulated in P^−^H^+^ including HSF B-2c isoform X2 (XP_004957414.1), HSF B-2a (XP_002446950.1) and class III HSP18.6 (XP_004954144.1). Similarly, among the nine transcripts that were 6–8-fold up regulated by priming in P^+^H^−^, one transcript (XP_006653075.1; coding for chloroplastic photosystem II 22 kDa protein) was 2–4-fold up regulated and three transcripts, including HSF B-2b (XP_006659656.1), were 4–6-fold up regulated in P^−^H^+^.

In P^+^H^−^, 8119 transcripts were up to 2-fold down regulated, whereas in P^−^H^+^, 2572 transcripts were up to 2-fold up regulated (including HSP70-16; XP_006657253.1) and 21 transcripts were 2–4-fold up regulated, including cognate HSP70 (XP_010041882.1) and HSP81-1 (XP_022680062.1). Moreover, 618 transcripts showed 2–4-fold down regulation (including XP_006664764.1; 23.2 kDa heat shock protein-like), 47 transcripts showed 4–6-fold down regulation (including XP_006646827.1; 18.9 kDa heat shock protein-like) and 3 transcripts showed 6–8-fold down regulation.

Within the 376 transcripts that were 2–4-fold down regulated in P^+^H^−^, 34 transcripts including HSF C-1b (XP_015689817.1) were up to 2-fold up regulated in P^−^H^+^. A total of 186 transcripts were up to 2-fold down regulated (including chloroplastic small HSP; XP_004983363.1); 24 transcripts were 4–6-fold down regulated in P^−^H^+^.

Among the 13 transcripts that were 4–6-fold down regulated, in P^+^H^−^, 2 transcripts were up to 2-fold down regulated in P^−^H^+^ while 1 transcript (XP_006650831.1; uncharacterised) was 2–4-fold up regulated and 5 transcripts displayed 2–4-fold down regulation in P^−^H^+^.

#### 3.1.4. Functional Categorization of the Priming Induced Gene Expression

To functionally categorize the DETs in the three data sets Gene Ontology (GO) enrichment analysis was performed using transcripts common between two data sets (Figure S2). This analysis identified 171 (4.6%) DETs between P^+^H^−^ and P^+^H^+^, 116 (3.1%) DETs between P^−^H^+^ and P^+^H^−^ and 546 (14.8%) DETs between P^+^H^+^ and P^−^H^+^. It was observed that in all three comparisons the categories of cell, cell part and membrane emerged as the most represented GO terms in the Cellular process. The categories of “catalytic activities” and “binding” and were the most represented GO terms in molecular function while “metabolic process” and “cellular process” were the most represented GO terms in biological process.

**Figure 3:**
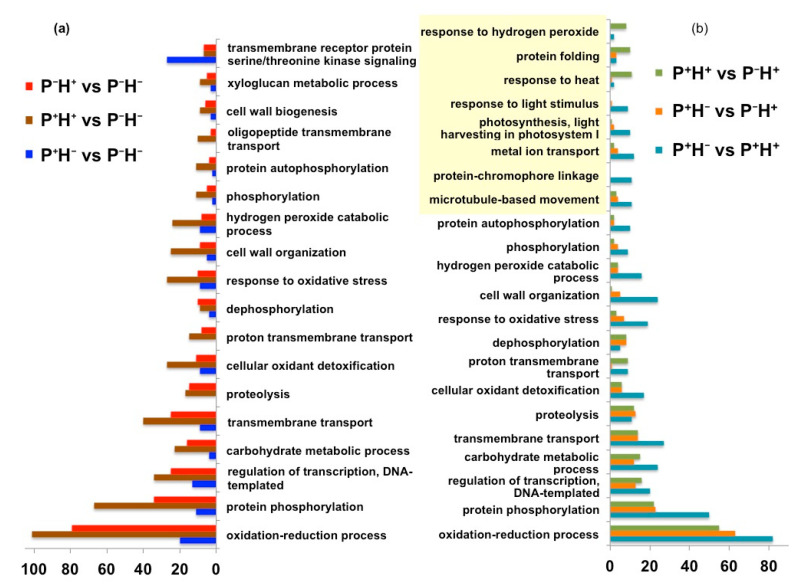
Graphical plot to represent the significantly enriched Gene Ontology (GO) categories in data sets obtained from rice seedlings only primed with HT (P^+^H^−^), thermo-priming followed by HTS (P^+^H^+^) and plants exposed directly to HTS without thermo-priming (P^−^H^+^). (**a**) In each case, the differential expression was calculated with respect to control non-primed plants (P^−^H^−^). (**b**) The differential expression in P^+^H^−^ and P^+^H^+^ was calculated with respect to P^−^H^+^ and in P^+^H^−^ with respect to P^+^H^+^. The unique GO categories appearing in these comparisons are highlighted. The scale represents absolute number of DETs associated with each GO categories.

Analysis of the top 20 significantly regulated pathways (Figure 3a) indicated that the categories “oxidation–reduction process”, “protein phosphorylation” and “regulation of transcription” were maximally represented in the DETs in P^+^H^+^ followed by P^−^H^+^ and P^+^H^−^, respectively, when compared to the control (P^−^H^−^). In P^+^H^−^, the category “serine threonine kinase signaling” was maximally up regulated, when compared with P^−^H^−^. Pairwise comparisons of P^+^H^−^ with P^+^H^+^ and P^−^H^+^, respectively, indicated that the categories “protein chromophore linkage”, “photosynthesis” and “response to light stimulus” were up regulated by priming (P^+^H^+^) and down regulated in response to direct HTS (P^−^H^+^). However, the categories “proton transmembrane transport”, “response to heat” and “response to hydrogen peroxide” were greatly down regulated (Figure 3b). Comparisons of P^+^H^+^ with respect to P^−^H^+^ indicated an up regulation in categories related to “response to heat”, “response to hydrogen peroxide” and “protein folding” (Figure 3b).

### 3.2. Validating the Priming Induced Gene Expression

**Figure 4:**
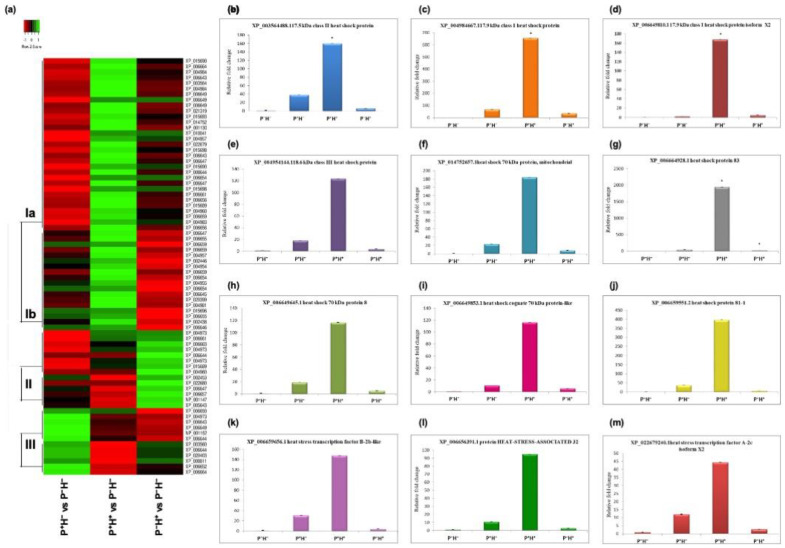
Differential expression of transcripts encoding heat shock factors (HSFs) and heat shock proteins (HSPs). (**a**) Heat map to show the global changes. In each case, the differential expression was calculated with respect to control plants (P^−^H^−^). The DETs were divided into different clades based on the general expression behaviour. (**b**–**m**) The expression patterns of selected transcripts validated by qPCR. The values were normalized with respect to 18S and fold change in expression was plotted (*n* = 3; means ± SD).

RNA-seq analysis indicated that 56 transcripts encoding HSPs and HSFs were up regulated in P^+^H^+^ (Figure 4a). The DETs could be broadly categorized into three clades with clade I including transcripts that are up regulated in P^+^H^+^. In clade Ia the transcripts showed down regulation in P^+^H^−^ followed by P^−^H^+^ while clade Ib contained transcripts that showed down regulation in P^−^H^+^ followed by P^+^H^−^. Clade II includes transcripts that are up regulated by HTS in P^−^H^+^ but down regulated in P^+^H^−^ and P^+^H^+^. Clade III included transcripts that are up regulated in P^+^H^−^ but down regulated in P^+^H^+^ and P^−^H^+^.

The expression of HSP17.5 (XP_003564488.1), HSP17.9 (XP_004984667.1), HSP17.9 isoform X2 (XP_006649810.1) and cognate HSP70 (XP_006649853.1) was 4–6-fold up regulated in P^+^H^−^ and P^−^H^+^ while they were about 8-fold up regulated in P^+^H^+^, with respect to P^−^H^−^. The class III HSP18.6 (XP_004954144.1), mitochondrial HSP70 (XP_014752657.1), HSFA-2c (XP_022679240.1) and HSA32 (XP_006656391.1) displayed 2–4-fold up regulation in P^+^H^−^ and P^−^H^+^ but showed 3–5-fold up regulation in P^+^H^+^. HSP70-8 (XP_006649645.1) HSP81-1 (XP_006659551.2), HSFB-2b (XP_006659656.1) and HSP83 (XP_006664928.1) showed 3–7-fold up regulation in P^+^H^−^ and P^−^H^+^ while they were >8-fold up regulated in P^+^H^+^, with respect to P^−^H^−^ but they were 5–9-fold up regulated in P^+^H^+^. The expression patterns of selected transcripts were validated by qPCR (Figure 4b–m). It was observed that in all cases expression levels were highest in P^+^H^+^ and the overall expression patterns were similar to the results obtained in RNA-seq analysis.

### 3.3. Capturing the miR Regulators in the Priming Response

As post-transcriptional regulation by the miRs also plays an important role in regulating gene expression, small RNA-sequencing of the same samples was therefore also performed. This resulted in an average of 25.4 million reads, which provided an average of 23.7 million high quality reads per library (Table 1). The small RNA sequences were mapped to the rice (*Oryza sativa* L.) genome sequence and filtered on the basis of their size. The small RNAs that were of 21-nt to 24-nt in length were used for identification of known miRs.

Using P^−^H^−^ as control set the log_2_ fold change in miR expression patterns was calculated (Table S3). It was observed that in P^+^H^−^, more than two-fold up regulation occurred in osa-miR2873c, osa-miR812q, osa-miR812r and more than two-fold down regulation occurred in osa-miR1846a-5p, osa-miR1846b-5p (Table 1). Target prediction identified transcripts coding for HSP-DnaJ, putative nucleoporin autopeptidase, calcium/calmodulin dependent serine/threonine protein kinase and CBL-interacting-protein kinase 10 (CIPK10), Villin and aldehyde dehydrogenase as their putative targets.

In P^+^H^+^, six miRs were up regulated (osa-miR2120b-5p, osa-miR397a, osa-miR397b, osa-miR398b, osa-miR408-5p, osa-miR7693-3p) and four miRs were down regulated (osa-miR166k-5p, osa-miR167d-3p, osa-miR1865-3p, osa-miR5153) (Table 1). In P^−^H^+^, only osa-miR2120b-5p showed up regulation and four miRs, namely osa-miR166h-5p, osa-miR166j-5p, osa-miR166k-5p and osa-miR1865-3p, were down regulated (Table 1). The targets for the up regulated miRs included transcripts of D-xylose-proton symporter-like 3, osmotic stress-activated protein kinase, laccase, copper superoxide dismutases (CSD1 and CSD2), cytochrome C oxidase (COX5b), plastocyanin-like protein family and Constans-like protein (CO5). The targets of down regulated miRs included transcripts coding for proteins containing UF630/DUF632 domain, homeobox associated leucine zipper (HD-Zip), MADS-box (OsMADS16), kelch motif and HEAT repeat. The transcripts of midasin protein, lectin receptor kinase, protein phosphatase 2C, 30S ribosomal protein S31, pentatricopeptide, WRKY104, WRKY95, pirin, DnaK, cyclase protein and chloride channel protein were also targeted.

**Figure 5:**
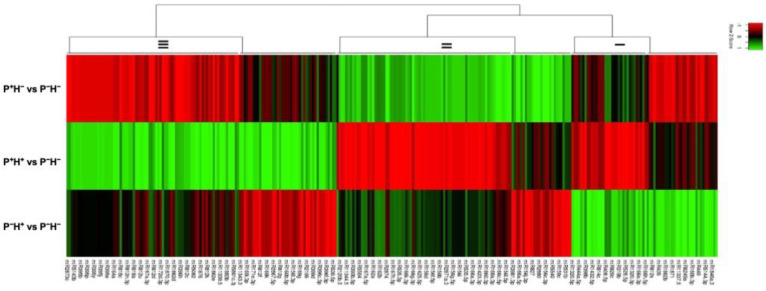
Heat map to show the differential expression of miRs in P^+^H^−^, P^+^H^+^ and P^−^H^+^. In each case, the differential expression was calculated with respect to control non-primed plants (P^−^H^−^). These were divided into different clades as indicated.

A heat map was generated to understand the differential behavior of miRs in the data sets (Figure 5) and this identified three clades of differentially regulated miRs. Clade I included miRs that are down regulated by priming in P^+^H^−^ and P^+^H^+^ but up regulated in plants exposed to direct HTS (P^−^H^+^). Clade II included miRs that are up regulated by priming in P^+^H^−^ but down regulated by HTS in P^+^H^+^ and P^−^H^+^. Clade III included miRs that are down regulated in P^+^H^−^ and P^−^H^+^ but up regulated in plants primed before exposure to HTS (P^+^H^+^).

#### Identifying the Windows of miR and Target Correlations

**Figure 6:**
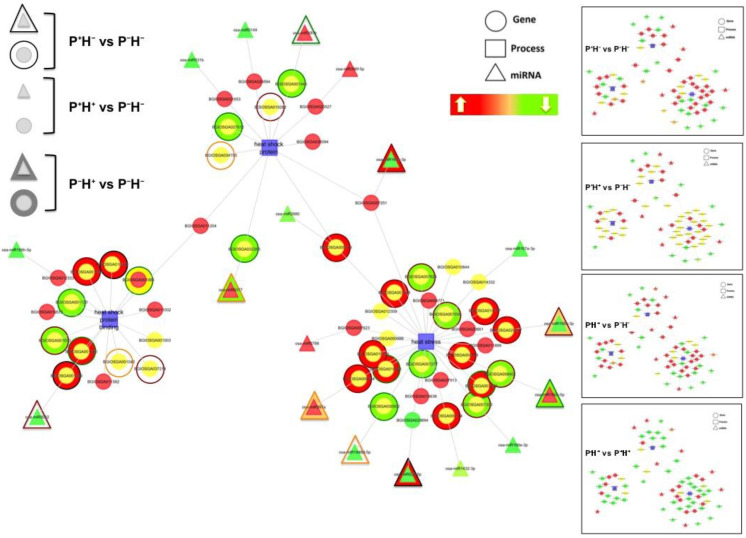
Cytoscape interactome network to connect the GO terms (process) related to heat shock proteins, heat shock proteins (HSP) binding and heat stress (shown as rectangles) with transcripts (shown as circles) and their regulatory miRs (shown as triangles). The transcript and miR nodes are coloured to show the change in their expression. The basic image represents the interactions seen in P^+^H^+^ with respect to P^−^H^−^. The changes in P^−^H^+^ with respect to P^−^H^−^ are represented by the fill color of the larger circles and triangles overlapping the nodes. The changes in P^+^H^−^ with respect to P^−^H^−^ are represented by the color of lines of the larger circles and triangles overlapping the nodes. Individual maps are shown as thumbnails on the right side.

The pool of DETs was sifted to search for transcripts corresponding to HSPs, HSP binding proteins and HSFs. To understand the possible role of miRs in regulating these transcripts, cytoscape interactome networks encompassing selected targets, their biological categories (determined from GO and KEGG analysis) and the rice miRs were created. This identified 18 miRs and 27 proteins showing differential expression in the three data sets (P^+^H^−^, P^+^H^+^ and P^−^H^+^) when compared with P^−^H^−^ (Figure 6). Among the differentially regulated miRs, osa-miR397b and osa-miR5077 targeted HSP20 (BGIOSGA007446) and small HSP20 (HSP20-like) chaperone (BGIOSGA032265) while osa-miR5082 targeted a pleiotropic drug resistance protein (PDR) like ABC transporter (BGIOSGA001229). These were associated with the categories HSP and HSP binding, respectively. The differentially regulated miRs, osa-miR167e, osa-miR156b, osa-miR168a, osa-miR1846d and osa-miR397a, targeted transcripts of NAC domain-containing protein (BGIOSGA014332), HSF29 which is similar to HSFA2b (BGIOSGA025247), HSFB-2c with a probable kinase domain (BGIOSGA008402), HSFA4d (BGIOSGA030902), Glyceraldehyde-3-phosphate dehydrogenase (BGIOSGA008564). The osa-miR167e also targeted the endoplasmic reticulum localized chaperone HSP70 (BGIOSGA007251), which was associated with both HSP and heat stress categories. The osa-miR531b and osa-miR5149 showed down regulation under priming (P^+^H^−^) and HTS (P^−^H^+^). They targeted a small HSF (BGIOSGA021653) and HSP-precursor similar to HSP90-6 (BGIOSGA029594), respectively, that associated with the heat stress category.

**Figure 7:**
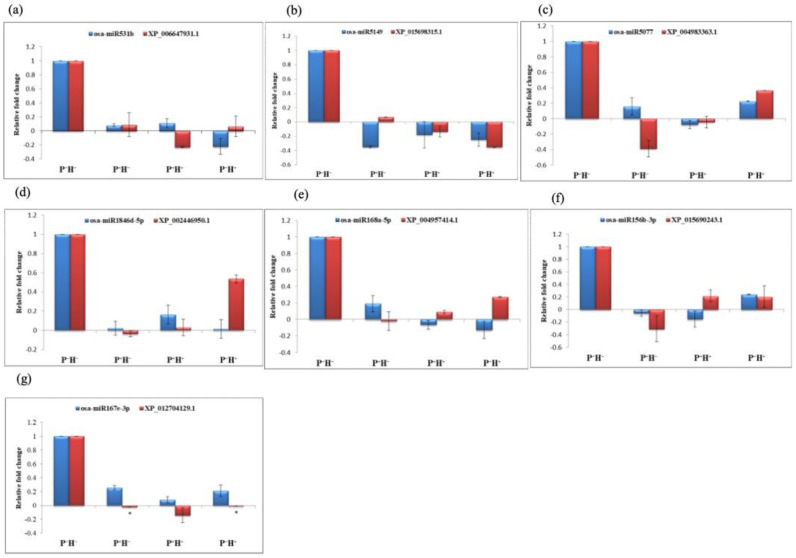
(**a**–**g**) The plot of relative fold change in expression of selected miRs and their targets as quantitated in plants under control conditions (P^−^H^−^), only thermo-primed conditions (P^+^H^−^), thermo-priming followed by HTS (P^+^H^+^) and direct HTS (P^−^H^+^). The expression values were obtained by qRT-PCR and normalized with respect to 18S. For plotting, the fold change was calculated with respect to values in P^−^H^−^. In each case (*n* = 3; means ± SD).

To further confirm these dynamic correlations, the expression patterns of seven differentially regulated miRs and their corresponding targets were validated by q-PCR. This analysis showed qualitative and quantitative changes in the expression patterns (Figure 7). It was seen that in P^+^H^−^, expression of all the miRs was suppressed. In P^+^H^+^ the relative (with respect to P^−^H^+^) expression of osa-miR531b (Figure 7a), osa-miR5149 (Figure 7b), osa-miR1846d-5p (Figure 7d) and osa-miR168a-5p (Figure 7e), was higher and that of osa-miR5077 (Figure 7c), osa-miR156b (Figure 7f) and osa-miR167e (Figure 7g) was lower. Under conditions of P^+^H^−^ and P^+^H^+^ the expression of osa-miR5077 (Figure 7c) and osa-miR168a-5p (Figure 7e) showed opposite behavior.

The conditions in which the miR expression patterns correlated with the target expression were also apparent. Under priming (P^+^H^−^), the changes in levels of osa-miR5149, osa-miR5077, osa-miR1846d-5p, osa-miR168a-5p and osa-miR167e inverse correlated with the changes in their respective transcripts. The miR-target correlations were also seen in P^+^H^+^ and P^−^H^+^ in case of osa-miR531b targeted HSP24.1 (XP_006647931.1) and osa-miR168a-5p targeted HSFB-2c (XP_004957414.1). These results indicate that the miR controlled target nodes are deregulated in response to HT priming. Under HTS other pathways are also activated that may lead to absence of apparent negative correlations of the miRs with their targets. Interestingly osa-miR168a-5p targeted HSFB-2c (XP_004957414.1) emerged as the crucial node regulated by priming and HTS.

### 3.4. Validating the Role of HT Priming

To understand the biological effect of HT priming on rice plants, mature plants were primed with HT in a similar manner (Figure 1) and scored for survival and productivity (Table 2). The results showed that rice plants thermo-primed before anthesis (PBH) or thermo-primed after anthesis (PAH) and then subjected to HTS showed significantly higher grain yields (Table 2) as compared to the non-primed plants directly exposed to HTS before anthesis (NBH) or after anthesis (NAH). Plants grown at normal temperatures and not subjected to thermo-priming or HTS served as the control. Between all sets of plants., no significant change was observed in panicle length, seed size and seed weight. In both non-primed (NBH and NAH) and thermo-primed (PBH and PAH) HTS plants, the number of grain filled panicles were less than the control, but the thermo-primed plants (PBH and PAH) had relatively higher numbers of filled grains as compared to the non-primed plants.

## 4. Discussion

High temperature stress, as a result of global warming, has become a major threat for sustaining plant productivity and hence food security. Plants have the inherent ability to respond to and generate memory of HTS, by initiating changes at the transcriptome, epigenome, metabolome and proteome levels [61]. The HTS responsive genes, miRs, DNA methylation patterns, and histone modifications have been well characterized [5,13,15,62]. It has been shown that the induction of several HSFs and HSPs in response to HT is conserved across species and these proteins act synergistically to counter the effect of stress [17,63,64,65,66].

HSFs bind to specific cis-elements to induce the expression of heat shock genes and thus play a crucial part during plant growth. Plant HSFs are highly conserved and sub-species of *O. japonica* and *O. indica* contain around 25 *hsf* genes. They can be categorized into three conserved classes *viz.* A, B and C based on their structural features [67,68,69]. Class A HSFs are essential for transcriptional activation; however, Class B and C HSFs do not have activator function [69]. Within the class A HSFs, HSFA1a and HSFA1b are important for the initial phase of HTS responsive gene expression [70]. HSFA2 is a heat-inducible transactivator that prolongs acquired thermotolerance by maintaining the expression of HSP genes [71,72]. HSFA3 is regulated by DREB2a and DREB2c and also plays a crucial role in thermotolerance [17,73]. In addition, HSFA4a and HSFA8 act as sensors of the reactive oxygen species (ROS) produced in response to HTS [74]. In plants, class B HSFs are downstream target genes of HSFA1 and they mainly act as transcriptional repressors. The functions of class C HSFs are not very clear. In wheat, overexpression of TaHSFC2a-B resulted in up regulation of HSPs and other heat protection genes to improve thermotolerance [75,76].

HSPs are also ubiquitously conserved and have been categorized into five broad families based on their molecular weight, amino acid homologies and functions. These families are named as HSP100/ClpB, HSP90, HSP70/DnaK, HSP60//GroEL and small HSP family (sHSPs) [77,78]. The *hsp70* and *hsp90* represent highly conserved multi-gene families that have functional roles in all subcellular compartments. Proteins belonging to this family perform chaperone functions under stress conditions and during protein metabolism. The binding of HSP70 to unfolded or denatured proteins prevents aggregation and refolding [79]. The sHsps constitute a large family of nuclear encoded proteins that are maximally expressed under stress conditions [80]. It was shown that overexpression of sHsp 17.7 resulted in enhanced thermotolerance in tomato and rice [81,82].

It has also been shown that a short exposure to HT allows plants to exhibit enhanced thermotolerance by acquiring and storing somatic memory of HTS [8,40,83,84,85,86]. This process is called thermo-priming and it is one of the critical adaptive strategies for generating HT tolerance in plants. Recent studies in Arabidopsis and rice have shown that memory for thermo-priming lasts for several days at normal growth temperatures. This process has adaptive advantages under natural conditions, where there is perpetual heat stress or it keeps recurring [83].

In this study, we have used RNA-seq to map specific changes in transcriptome and miRs during HT priming in rice. To analyze the effect of HT priming, three sets of treatments were given to rice seedlings viz. only HT priming (P^+^H^−^), priming followed by HTS (P^+^H^+^) and direct HTS (P^−^H^+^). Seedlings grown at ambient temperatures served as the control (P^−^H^−^). The experimental conditions used for priming in P^+^H^+^ were chosen to reflect the memory-development phase. NGS analysis identified that priming treatment results in changes in the transcript and miR profiles. When plants were only primed (P^+^H^−^), a greater number of transcripts were up regulated while, in the condition of priming followed by HTS (P^+^H^+^), higher numbers of transcripts were down regulated. Moreover, the fold changes in the expression patterns were generally lower in P^+^H^−^ and larger fluctuations were observed in P^+^H^+^. A large number of the DETs were associated with the redox pathway, protein phosphorylation and regulation of transcription. The transcripts related to photosynthesis and response to light were up regulated by priming in P^+^H^−^ and P^+^H^+^, but down regulated in P^−^H^+^. This showed that the involvement of diverse biological functions such as defense responses, signal transductions and thermotolerance, in HT priming responses may be helping plants to acclimatize to tolerate subsequent high temperatures in the environment.

Among the heat regulated transcripts, the levels of HSP3, HSP17, HSP83, HSP23.2, HSP70s, HSP90s, HSFA-2c, HSFA-9, HSFA-4d, HSF B-2 and HSP18.6 were up regulated by priming in P^+^H^−^ and showed further up regulation in P^+^H^+^. The mitochondrial and chloroplastic HSP70, HSP90, HSP26.2, HSP26.7 and activator of HSP90 ATPase were up regulated under all three treatments. The transcripts of HSFA-5, HSFA-2d, DNAJ-HSP isoform and class V HSP18.8 were up regulated by priming in P^+^H^−^ but were down regulated in P^+^H^+^ and also in P^−^H^+^. Several transcripts were down regulated in P^+^H^−^ and P^+^H^+^ and these included HSP23.2, chloroplastic small HSP, HSF C-1b HSF C-2b and cytochrome P450 containing premnaspirodiene oxygenase. This analysis has helped to elucidate potential HT priming regulated pathways operating during plant adaptation to high temperature stress.

When analyzed further it was found that several class A and class B HSF transcripts were up regulated in primed plants (P^+^H^+^) as compared to the non-primed plants (P^−^H^+^). Earlier studies have revealed a role for HSF in HTS and thermotolerance [63,64,65,66]. It has been shown that under HTS, the HSP70 and HSP90 proteins are induced which interact with class A HSFs [87]. HSFA1 acts as the master activator as it triggers the immediate expression of other transcription factors [17,88,89], including Dehydration Responsive Element Binding protein 2 (DREB2), HSFA2, HSFA7, HSFB and so on. Overexpression of HSFA3 activates the expression of *gols* genes, which in turn regulate the biosynthesis of galactinol to increase oxidative stress tolerance in plants [90]. The downstream HSFBs negatively regulate expression of several heat-inducible HSFs (HSFA2, HSFA7s) and HSPs (e.g., HSP101, HSP70). The HSFAs and HSFBs influence each other, forming a tight regulatory network [87,91]. Other transcription factors, such as MBF1C, NAC, WRKY, bZIP and MYB, are also involved in the regulation of HT responsive genes. MBF1C is a highly conserved transcriptional coactivator and a key regulator of thermotolerance [92]. Similarly, NAC binding sites are present in the promoters of several HSFs e.g., HSFA1b, HSFA6b, HSFA7a, and HSFC1 [20].

It has been shown that the HT-induced stress memory during priming can be regulated by both somatic and trans-generational modes via DNA methylation, histone modifications, alternative splicing and small RNAs [40,93,94,95,96,97,98]. In our study changes in miR expression patterns were also observed and these miRs showed an inverse relationship with their respective targets under the experimental conditions (P^+^H^−^, P^+^H^+^ and P^−^H^+^). The differential regulation of miRs under HTS is known to regulate many processes associated with growth, development and productivity of crops [22,27,28,29,30,46]. The rigorous control is maintained by regulating translation efficiency of key mRNAs including those of transcription factors [27,46,99,100]. Thus, HT priming induced changes in the miR profiles may directly influence HTS tolerance response. The results obtained from small RNA-seq showed that priming (P^+^H^−^) induced the expression of several miR belonging to the osa-miR2873, osa-miR812 families while it down regulated osa-miR1846. HT priming followed by HTS (P^+^H^+^) caused down regulation of osa-miR531, osa-miR156, osa-miR1865, osa-miR5153, osa-miR166 and osa-miR167, while levels of osa-miR2120, osa-miR397, osa-miR398, osa-miR408 and osa-miR7693 were up regulated.

The role of miRs in HT priming was studied by creating cytoscape interactome networks with the transcripts corresponding to HSPs, HSP binding proteins and HSFs. Some of the miRs and their interacting partners were validated by qRT-PCR and the conditions in which they negatively correlated were identified. Cytoscape based interactome analysis identified 18 miRs and 27 proteins that directly correlated with the heat response pathways. Overlapping these networks with their expression profiles indicated a crucial role for these regulatory nodes in governing the response to HT priming. Among these the expression profiles of a few miR target pairs were experimentally validated. These include key HSFs and HSPs targeted by osa-miR156b-3p, osa-miR167e, osa-miR168a-5p, osa-miR1846d-5p, osa-miR531b, osa-miR5077 and osa-miR5149. Under priming (P^+^H^−^) conditions the expression patterns of osa-miR5149, osa-miR5077, osa-miR1846d-5p, osa-miR168a-5p and osa-miR167e inverse correlated with the expression of their respective targets. The osa-miR531b and osa-miR168a-5p negatively regulated their targets in P^+^H^+^ and P^−^H^+^. The results not only supported the findings of the interactome but suggested that regulation of the steady state levels of miRs and their targets is complex and non-stoichiometric.

Earlier studies have shown that expressions of several conserved miRs and their respective targets were deregulated under HTS. It was reported that expression of osa-miR531, a highly conserved miR family, was regulated under different abiotic stress conditions including short duration HTS by signaling through the MAP kinase cascade [26]. Similarly, HT priming generated HTS memory in Arabidopsis through repression of SPL transcripts by inducing transcriptional changes in miR156 isoforms [45]. During recovery from HTS, the SPL levels are restored and this sustains the levels of HT responsive HSFA2 and HSPs [45,101]. In contrast, miR398 is rapidly induced in response to HTS to down regulate the copper/zinc superoxide dismutase gene [102]. This leads to accumulation of ROS, which increases the levels of HSF and HSP. The expression of miR398 is itself under the control of HSFA, thereby constituting a positive feedback loop. A recent study showed that miR167 regulates DNA methylation process during HTS response in crops [103,104,105], indicating a possibility of imprinting playing role in HTS memory. The miR159 acts on the GAMYB transcription factors and miR396 regulates WRKY transcription factors, which have roles in heat tolerance [106,107].

In conclusion, this study is important in identifying the genetic nodes and their regulation by the miRs in response to HT priming. The comparative RNA-seq analysis of rice seedlings primed with HT (P^+^H^−^) and stressed with HT with (P^+^H^+^) and without priming (P^−^H^+^) identified a number of transcripts and miRs that are induced or down regulated. Their analysis allowed identification of crucial regulatory nodes consisting of activators and repressors of the HTS response that are modified by priming. A specific role for osa-miR531a, osa-miR5149, osa-miR168a-5p, osa-miR1846d-5p, osa-miR5077, osa-miR156b-3p, osa-miR167e-3p was recognized as these miRs act on respective HSF or HSP targets to differentially alter gene expression in response to HT priming. These genetic alterations under priming can lead to activation and sustenance of HTS responses for plant survival. Our experiments on thermo-priming the mature plants before and after anthesis indicated that it enables the plants to endure HTS and helps in preventing loss in seed set and productivity by sustaining grain filling. Though direct correlations cannot be drawn between the seedlings and mature plants, this will serve as the basis of future experiments to follow up on the effect of changes in miR expression during priming to plant productivity under HTS. Considering that priming has been predicted to improve plant fitness in complex physiological environments [8,83,85], the findings of this study hold promising results in understanding the genome level changes that are initiated to cope with HTS. The significance of priming regulated miRs remains an important research question and requires a detailed analysis. It will be important to understand the role and mechanism of miRs in integrating priming with HTS memory to regulate plants development and crop yields.

## Figures and Tables

**Table 1:** Summary of RNA-seq transcriptome and small RNA analysis.

	P^−^H^−^	P^+^H^−^	P^+^H^+^	P^−^H^+^
**RNA-seq Data**				
Total number of raw reads (in millions)	25.82	25.57	25.29	25.24
Total high quality reads (in millions)	24.40	24.24	23.89	23.78
Reads aligned to the genome (in millions)	21.97	21.87	21.69	21.20
Percentage alignment	90.04	90.23	90.8	89.14
Number of transcripts	22,417	22,297	22,440	22,412
Total number of differentially expressed transcripts (DETs)		935 ^#^	2467 ^#^	1734 ^#^
Up regulated DETs (FC ≥ 2, *p* ≤ 0.05)		546 ^#^	1026 ^#^	751 ^#^
Down regulated DETs (FC ≤ −2, *p* ≤ 0.05)		389 ^#^	1441 ^#^	983 ^#^
**Small RNA-seq Data**				
Total number of raw reads (in millions)	27.34	24.15	25.17	24.96
Total high quality reads (in millions)	25.51	22.54	23.4	23.29
Number of unique Tags (in millions)	2.18	1.71	2.31	2.23
Number of miRNAs identified	296	294	296	296
Number of DE miRs (FC ≥ 2)		5 ^#^	10 ^#^	5 ^#^
Number of DE miRs (FC ≥ 1.5)		21 ^#^	26 ^#^	15 ^#^

# Compared with respect to seedlings grown under normal conditions (P^−^H^−^); FC: log_2_ Fold Change.

**Table 2:** Agronomic parameters in mature rice plants thermo-primed before anthesis (PBH) or after anthesis (PAH) and then subjected to HTS. These were compared with non-primed plants directly exposed to HTS before anthesis (NBH) or after anthesis (NAH). Plants grown at normal temperatures served as the control.

Traits	Control	PAH	PBH	NAH	NBH
100-grains weight (g)	1.90 ± 0.065	2.10 ± 0.11	2.09 ± 0.068 *	1.78 ± 0.049	1.74 ± 0.057 *
Panicle length (cm)	32.2 ± 2.75	25.8 ± 5.99	29.32 ± 0.59	30.37 ± 2.71	31.65 ± 2.63
Grains per panicle (Filled)	73.5 ± 13.77	48.25 ± 39.16	49.0 ± 13.19 *	21.25 ± 10.21 **	46 ± 18.01 *
Grains per panicle (Empty)	10.5 ± 21.98	21.25 ± 5.90 **	21.75 ± 10.71 **	86.25 ± 21.56	50.5 ± 19.75 **
Seed length (mm)	11.12 ± 0.54	10.96 ± 0.56	11.03 ± 0.60	10.98 ± 0.54	10.84 ± 0.51 **
Seed width (mm)	2.26 ± 0.12	2.20 ± 0.20	2.63 ± 0.28 **	2.53 ± 0.22 **	2.59 ± 0.21 **
Ratio of seed length to seed width	4.90	4.84	4.19	4.33	4.18

*, ** Significance at probability levels of 5% and 1%, respectively (ANOVA single factor).

## Data Availability

The transcriptome and small RNA data generated in this study are publically available at NCBI under the accession numbers SAMN13155778, SAMN13155779, SAMN13155780, SAMN13155781, SAMN13155782, SAMN13155783, SAMN13155784, SAMN13155785.

## Supplementary Materials

The following are available online at https://www.mdpi.com/article/10.3390/life11040291/s1, Figure S1: Bioinformatics workflow used for analyzing the RNA-seq data. It illustrates the stepwise use of tools and databases to identify the (a) transcripts and (b) miRs and their targets, Table S1: List of primers pairs of miRs and their corresponding targets used for qRT-PCR. These were designed using primer3 Web software, Table S2: Table listing the log_2_ fold change values of differentially expressed transcripts calculated for the transcripts in different libraries (P^+^H^−^, P^+^H^+^ and P^−^H^+^) with respect to control non-primed plants (P^−^H^−^), Figure S2: GO enrichment of the terms associated with the differentially expressed transcripts classified as molecular function, cellular components and biological processes. The DETs were searched against the NRDB protein data base using the Blastx program from BLAST2GO. Annotation was done using Blast2GO v5.0 for significantly Up and Down regulated transcripts. (a) P^+^H^−^ vs. P^−^H^−^ (b) P^+^H^+^ vs. P^−^H^−^ and (c) P^−^H^+^ vs. P^−^H^−^, Table S3: Table listing the log_2_ fold change in miR expression patterns calculated for the reads in different libraries (P^+^H^−^, P^+^H^+^ and P^−^H^+^) with respect to control non-primed plants (P^−^H^−^).
