# Supplementary material for: Priming by High Temperature Stress Induces MicroRNA Regulated Heat Shock Modules Indicating Their Involvement in Thermopriming Response in Rice
Source: Life (Basel). 2021 Mar 29;11(4):291. doi: 10.3390/life11040291 (PMC8067039; doi:10.3390/life11040291)
Supplement: Supplementary file 1 [file life-11-00291-s001.pdf]

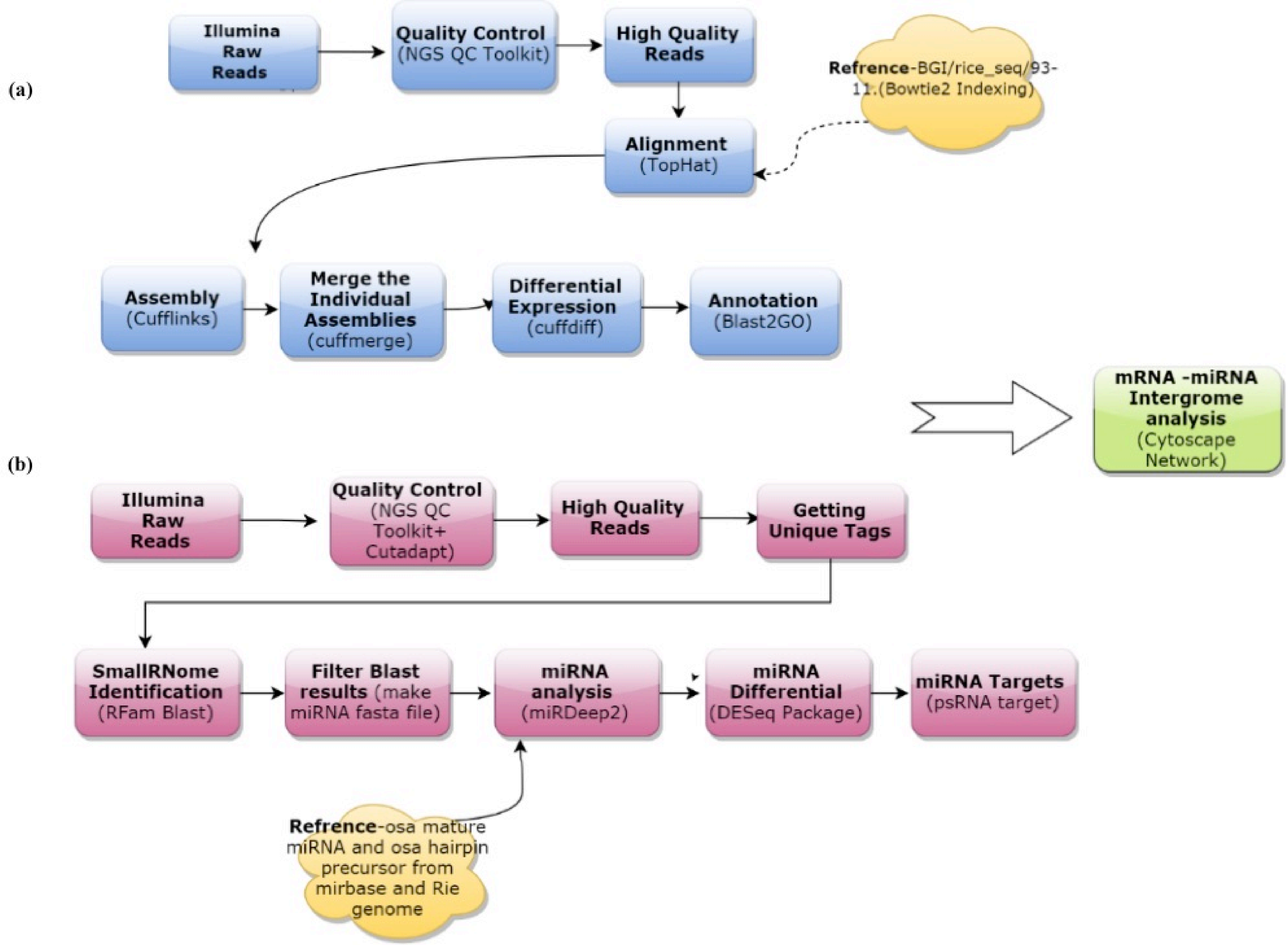

Supplementary Figure 1: Bioinformatics workflow used for analyzing the RNA-seq data. It illustrates the stepwise use of tools and databases to identify the (a) transcripts and (b) miRs and their targets.

Supplementary Fig S2:
GO enrichment of the terms associated with the differentially expressed transcripts classified as molecular function, cellular components and biological processes. The DETs were searched against NRDB protein data base using Blastx program from BLAST2GO. Annotation was done using Blast2GO v5.0 for significantly Up and Down regulated transcripts. (a) P^+H^- vs P^-H^- (b) P^+H^+ vs P^-H^- and (c) P^-H^+ vs P^-H^-.

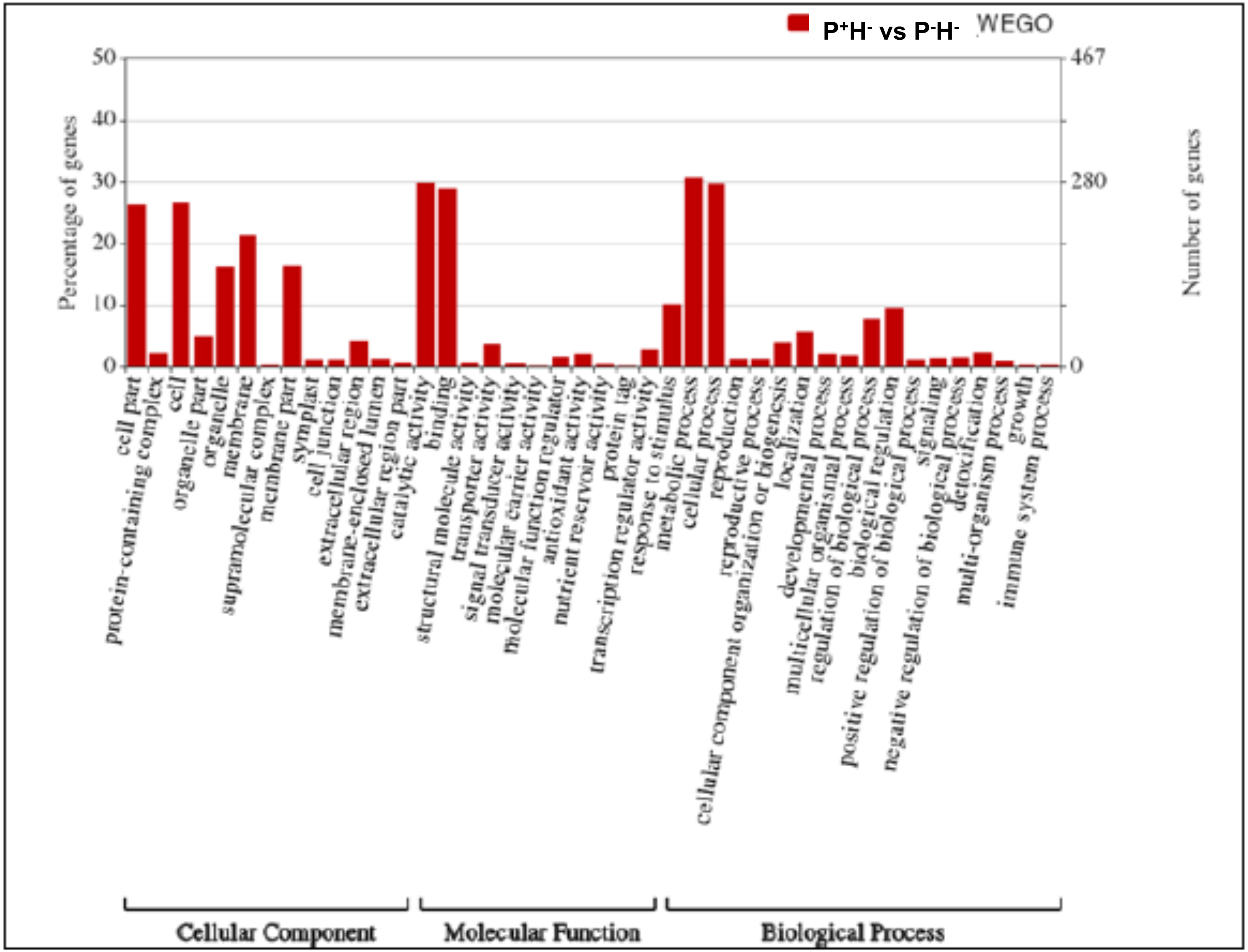

Gene ontology (GO) classification for the DEGs. Transcript sequences were hit against NRDB Protein database using Blastx program from BLAST2GO.

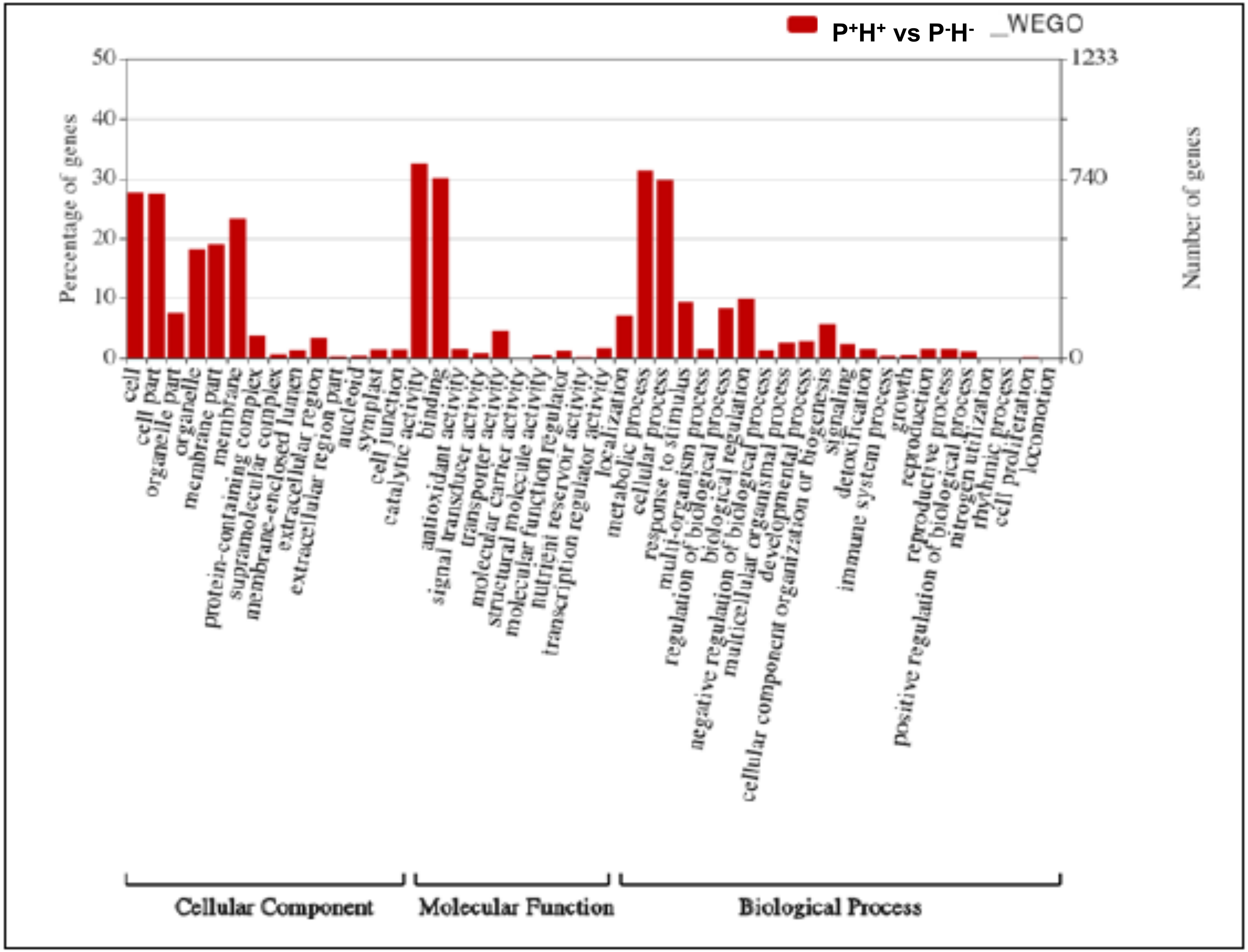

Gene ontology (GO) classification for the DEGs. Transcript sequences were hit against NRDB Protein database using Blastx program from BLAST2GO..

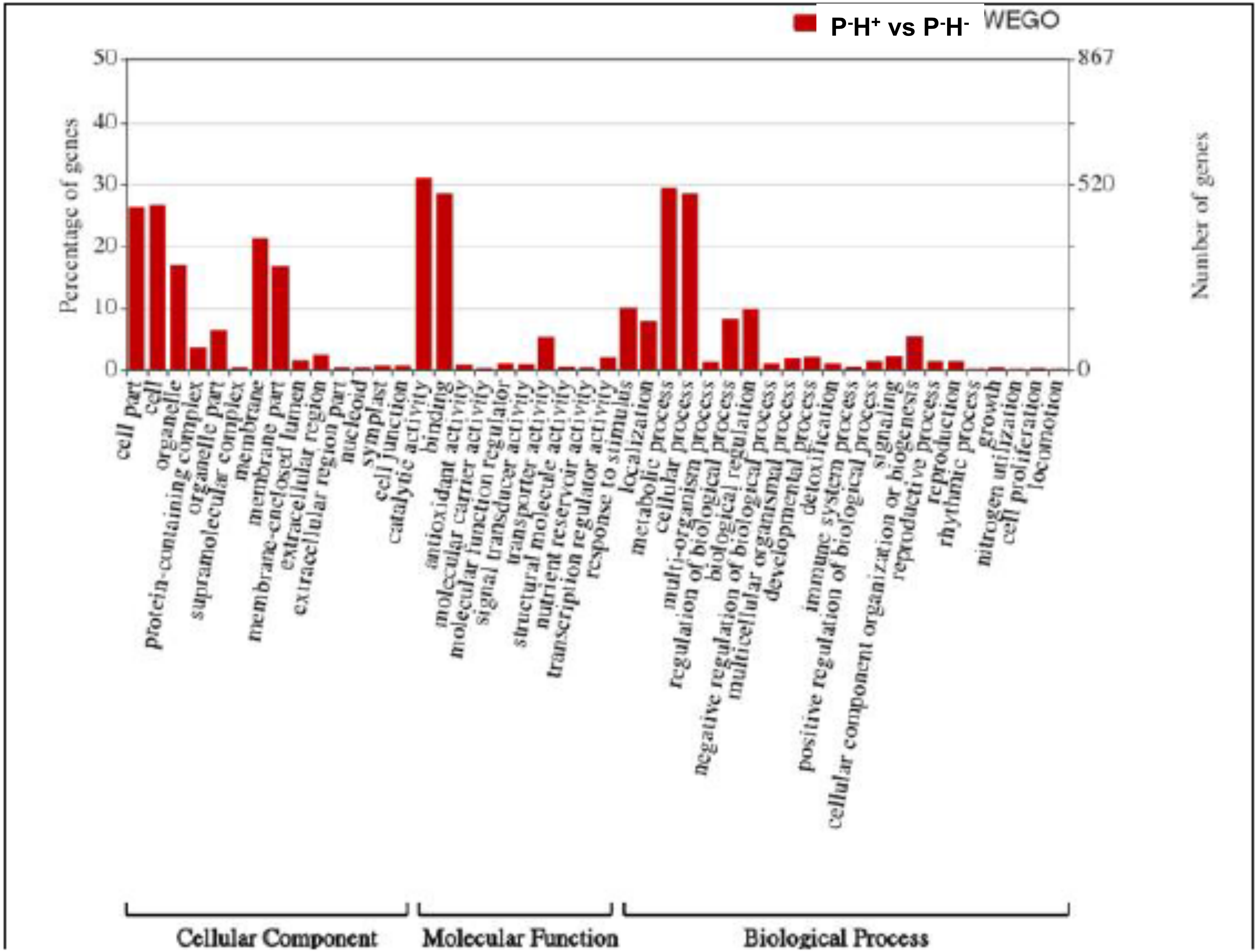

Gene ontology (GO) classification for the DEGs. Transcript sequences were hit against NRDB Protein database using Blastx program from BLAST2GO..

Supplementary Table S1: List of primers pairs of miRs and their corresponding targets used for qRT-PCR. These were designed using primer3 Web software.

miRs

**Primer name**	**Primer sequence (5'-3')**
osa-miR531b(SL)	gTCgTaTCCaGTgCagggTCCgaggTaTTCgCaCTggaTaCgaCCggCaC
osa-miR531b(FP)	gCaCTCgCCggggCTg
osa-miR5149(SL)	gTCgTaTCCagTgCagggTCCgaggTaTTCgCaCTggaTaCgaCTCCCaa
osa-miR5149(FP)	aCgCagaggagCTgTgaCga
osa-miR168a(SL)	gTCgTaTCCagTgCagggTCCgaggTaTTCgCaCTggaTaCgaCgTCCCg
osa-miR168a(FP)	CgCaTCgCTTggTgCaGa
osa-miR1846d-5p(SL)	gTCgTaTCCagTgCagggTCCgaggTaTTCgCaCTggaTaCgaCgagaTC
osa-miR1846d-5p(FP)	gaCTCCCaCCgagCagCC
osa-miR5077(SL)	gTCgTaTCCagTgCagggTCCgaggTaTTCgCaCTggaTaCgaCTggTga
osa-miR5077(FP)	CgCagTTCgCgTCggg
osa-miR156(SL)	gTCgTaTCCagTgCagggTCCgaggTaTTCgCaCTggaTaCgaCgTgCTC
osa-miR156(FP)	CgaCTgaCagaagagagT
osa-miR167i+e-3p(SL)	gTCgTaTCCagTgCagggTCCgaggTaTTCgCaCTggaTaCgaCagTgaa
osa-miR167i+e-3p(FP)	CaCgaCagaTCaTgTTgCagC

miR targets

**Primer name**	**Primer sequence (5'-3')**	Target ID
HSPMITO-FP	CTCAGATGTGTTCCGTGATCC	XP_006647931.1 24.1 kDa heat shock protein, mitochondrial-like
HSPMITO-RP	CCTCCTCGCTCTCCTTCG	
HSP90-6,MITO-FP	GTACCAGGCAGAGGTCAATAGG	XP_015698315.1 heat shock protein 90-6, mitochondrial
HSP90-6,MITO-RP	GACAAGCTCTCGAAGAAACACC	
HSTFB-2cIX2-FP	ACAACAACTTCTCCAGCTTCG	XP_004957414.1 heat stress transcription factor B-2c isoform X2
HSTFB-2cIX2-RP	ATCACACAACAAACGCTTCTCC	
HSTFB-2a-FP	CTGTTCGTCCTCTGTTTCATGG	XP_002446950.1heat stress transcription factor B-2a
HSTFB-2a-RP	ACCGTTGTATGCACTGTCACC	
sHSPChlo-FP	CCACTCTCACCATATCATGTCC	XP_004983363.1small heat shock protein, chloroplastic
sHSPChlo-RP	CTTCTTCGCTTGCTTCTTCG	
HSTFB-2c A-9 FP	CACGGAGTGGTACTTCTTCAGC	XP_015690243.1 heat stress transcription factor A-9
HSTFB-2c A-9 RP	GGTGGCCTTCCAGTAGCC	
HSFA-2aFP	TTCAAGCACAGCAACTTCTCC	XP_012704129.1heat stress transcription factor A-2a
HSFA-2aRP	GACCTTCCTGAACCCGTAGG	
18s FP	CTACGTCCCTGCCCTTTGTACA	INTERNAL PRIMER FORWARD PRIMER
18s RP	ACACTTCACCGGACCATTCAA	INTERNAL PRIMER REVERSE PRIMER

Supplementary Table S2: Table listing the log2 fold change values of differentially expressed transcripts calculated for the transcripts in different libraries (P+H−, P+H+ and P−H+) with respect to control non-primed plants (P−H−),

17025 common eleme	log2FC_P$^+$H$^-$ vs P$^-$H$^-$	log2FC_P$^+$H$^+$ vs P$^-$H$^-$	log2FC_P$^-$H$^+$ vs P$^-$H$^-$	BGI ID	Description
TCONS_00020649	-5.15394	-5.16147	-0.114342	BGIOSGA013373	XP_006656053.1 probable xyloglucan endotransglucosylase/hydrolase protein 26
TCONS_00001859	-5.05957	-2.85023	-2.44994	BGIOSGA013370	XP_006644739.1 nucleobase-ascorbate transporter 2-like
TCONS_00032722	-4.79681	-2.36447	-4.40865	BGIOSGA025892	XP_004983616.1beta-amylase 3, chloroplastic
TCONS_00016486	-4.55562	-2.24076	-4.41295	BGIOSGA005828	XP_003580392.1protein REVEILLE 7-like
TCONS_00013100	-4.46388	-3.458	-1.04732	BGIOSGA009881	XP_008681064.1ubiquitin ligase protein FANCL isoform X3
TCONS_00029723	-4.34353	-8.75028	#NA	BGIOSGA020806	XP_006660483.1 premnaspirodiene oxygenase-like
TCONS_00002735	-4.30705	#NA	-5.4924	BGIOSGA002673	NP_001304895.1ubiquitin-protein ligase/ zinc ion binding protein
TCONS_00014387	-4.27081	-3.06207	-3.8845	BGIOSGA026924	XP_006665080.1 vacuolar amino acid transporter 1-like
TCONS_00011849	-4.2397	-4.83368	-2.84625	BGIOSGA033359	XP_006649694.1 peroxidase N-like
TCONS_00014822	-4.23569	-2.83871	-3.85615	BGIOSGA010119	XP_015691906.1 putative E3 ubiquitin-protein ligase LIN
TCONS_00021317	-4.13361	-3.73154	#NA	BGIOSGA035061	XP_006657297.1 benzyl alcohol O-benzoyltransferase-like
TCONS_00031646	-4.10419	-2.10109	-2.27695	BGIOSGA029546	XP_015693541.1 transcription factor HBP-1b(c38)-like
TCONS_00013461	-4.06618	-4.64925	3.16856	BGIOSGA009524	XP_006650831.1PREDICTED: uncharacterized protein LOC102717884
TCONS_00002675	-3.98483	-1.57548	-2.58624	BGIOSGA031010	XP_015692513.1 serine/threonine-protein kinase HT1-like
TCONS_00035952	-3.98084	0.927297	-4.18109	BGIOSGA023600	XP_015698288.1 AAA-ATPase At3g28580-like
TCONS_00007572	-3.98057	-1.58084	-1.85984	BGIOSGA004032	XP_004951512.1cytochrome P450 734A2
TCONS_00019229	-3.9279	-4.52416	-2.90053	BGIOSGA018120	XP_002441045.2probable membrane-associated kinase regulator 1
TCONS_00013998	-3.92753	-3.10893	-4.65982	BGIOSGA040253	XP_006652179.1 probable aldo-keto reductase 2
TCONS_00015157	-3.90161	-0.101657	-1.50567	#N/A	#N/A
TCONS_00031666	-3.90111	-2.17268	-1.50692	BGIOSGA022006	XP_006662089.1 NAC domain-containing protein 48-like
TCONS_00032753	-3.89963	-1.68911	-1.28819	BGIOSGA014064	XP_021307101.1AT-hook motif nuclear-localized protein 10-like
TCONS_00011702	-3.89943	-1.1748	-0.926124	BGIOSGA023892	XP_015689687.1 alcohol dehydrogenase-like 2
TCONS_00027678	-3.85407	-2.45653	-3.05864	BGIOSGA019255	XP_004974549.2MDIS1-interacting receptor like kinase 2
TCONS_00032681	-3.74526	-5.17113	-4.32558	BGIOSGA008753	XP_006662047.1 expansin-B3
TCONS_00036597	-3.72507	#NA	-1.89997	BGIOSGA006116	XP_015698436.1 protein DETOXIFICATION 21-like isoform X2
TCONS_00002632	-3.71905	-0.615471	#NA	BGIOSGA025988	XP_006654929.1PREDICTED: uncharacterized protein LOC102716972
TCONS_00037180	-3.63275	-1.87816	-0.782173	BGIOSGA017158	XP_021301930.1putative disease resistance protein At3g14460 isoform X1
TCONS_00017282	-3.62656	-4.20749	-3.79786	BGIOSGA008967	XP_015692979.1 germin-like protein 5-1
TCONS_00016703	-3.57722	#NA	#NA	BGIOSGA017524	XP_006653807.2 agmatine coumaroyltransferase-like
TCONS_00010161	-3.56572	0.478281	0.725572	BGIOSGA023851	XP_006650126.1 H/ACA ribonucleoprotein complex subunit 4
TCONS_00010918	-3.54115	-1.55062	-2.03292	BGIOSGA008673	XP_015690458.1PREDICTED: uncharacterized protein LOC102708885
TCONS_00008756	-3.51652	#NA	-3.69878	BGIOSGA004670	XP_006647848.1 protein ODORANT1-like
TCONS_00007180	-3.51347	0.213568	-0.246955	BGIOSGA007317	XP_015688416.1PREDICTED: uncharacterized protein LOC107303489, partial
TCONS_00009831	-3.51308	1.02043	-0.899996	BGIOSGA012383	XP_015691048.1 RNA pseudouridine synthase 5 isoform X3
TCONS_00008220	-3.51101	-0.86352	-1.90511	BGIOSGA037995	XP_010240404.2ent-kaur-16-ene synthase, chloroplastic isoform X2
TCONS_00022721	-3.46823	-3.90214	-2.41666	BGIOSGA020730	XP_008677918.1uncharacterized protein LOC103652737
TCONS_00031719	-3.4575	-4.051	-0.12471	BGIOSGA023614	XP_006661598.1 peroxidase N-like
TCONS_00023837	-3.40994	-2.15362	-0.940847	BGIOSGA021098	XP_015694785.1 cysteine-rich receptor-like protein kinase 10
TCONS_00029983	-3.4073	0.340659	-1.98259	BGIOSGA021589	NP_001152046.1appr-1-p processing enzyme family protein
TCONS_00036757	-3.4069	-1.07485	-0.868117	BGIOSGA034366	XP_015689762.1 GATA transcription factor 9-like
TCONS_00016482	-3.40665	#NA	0.430804	BGIOSGA005831	XP_015691796.1PREDICTED: uncharacterized protein LOC102714284
TCONS_00004249	-3.3976	-1.67144	-1.26995	BGIOSGA025424	XP_006644573.1 salicylate carboxymethyltransferase-like
TCONS_00037602	-3.39642	-3.25789	-3.17975	BGIOSGA026213	XP_006664780.1 purple acid phosphatase 2-like
TCONS_00017895	-3.36695	-2.96335	-2.97723	BGIOSGA014371	XP_004961925.1protein TRANSPARENT TESTA 1
TCONS_00033613	-3.35883	-3.95038	-2.80963	BGIOSGA007444	XP_015698174.1 probable xyloglucan endotransglucosylase/hydrolase
TCONS_00033999	-3.33453	-1.58615	-3.13606	BGIOSGA015557	XP_002465636.1sugar transport protein 13
TCONS_00029473	-3.29964	-3.51425	-5.982	BGIOSGA023639	XP_006660948.1 HMG1/2-like protein
TCONS_00016380	-3.27806	#NA	-3.45138	BGIOSGA014586	XP_006652619.2 acyl-acyl carrier protein thioesterase ATL3, chloroplastic-like
TCONS_00012987	-3.277	-0.691691	-1.86909	BGIOSGA004289	XP_006650430.1 germin-like protein 3-5
TCONS_00021455	-3.25516	-4.24111	-1.86932	BGIOSGA011754	XP_006656549.1 probable polygalacturonase
TCONS_00024346	-3.20415	-3.06861	-2.52368	BGIOSGA025002	XP_006658122.1 peroxidase 70-like
TCONS_00008598	-3.18223	-4.31175	-5.27456	BGIOSGA020501	XP_004953478.1protein REVEILLE 2 isoform X2
TCONS_00006492	-3.17075	-3.06198	-2.92672	BGIOSGA005099	XP_004953169.1auxin response factor 8
TCONS_00015171	-3.17063	-2.10403	-0.431178	BGIOSGA026369	XP_015692307.1PREDICTED: uncharacterized protein LOC102719949 isoform X1

TCONS_00006015	-3.15819	#NA	-2.2693	BGIOSGA027160	XP_014754387.1uncharacterized protein LOC106866147
TCONS_00001307	-3.15298	-2.70843	-2.2824	BGIOSGA006478	XP_023157643.1uncharacterized LOC100279343 isoform X4
TCONS_00012006	-3.15096	-1.38869	-3.28753	BGIOSGA011004	XP_003561776.1beta-glucuronosyltransferase GlcAT14B
TCONS_00006567	-3.13646	#NA	-1.51677	BGIOSGA008735	XP_006647619.2 probable enoyl-CoA hydratase 2, mitochondrial isoform X3
TCONS_00030139	-3.13555	-1.40717	-1.74144	BGIOSGA030941	XP_015696371.1 homeobox-leucine zipper protein HOX11
TCONS_00002586	-3.13548	-2.14423	-0.452114	BGIOSGA034953	XP_015691116.1PREDICTED: uncharacterized protein LOC102718984
TCONS_00023842	-3.13447	-1.56054	-2.32909	BGIOSGA018504	XP_006658641.1 F-box protein At5g49610-like
TCONS_00022936	-3.13437	#NA	-2.32933	BGIOSGA035990	XP_006656508.1 GDSL esterase/lipase At5g55050-like isoform X2
TCONS_00001843	-3.13276	-3.73261	-2.74834	BGIOSGA016791	XP_004972181.1transcription factor PCF7
TCONS_00033054	-3.11009	-5.13364	-5.26209	BGIOSGA032523	XP_024318852.1receptor kinase-like protein Xa21 isoform X1
TCONS_00012852	-3.09955	-4.10695	-1.28857	BGIOSGA011420	XP_006650368.1 predicted GPI-anchored protein 58
TCONS_00015602	-3.08251	-1.63065	-1.61453	#N/A	#N/A
TCONS_00030510	-3.06748	-2.64762	0.286022	BGIOSGA033400	XP_006660955.1 RING-H2 finger protein ATL80-like
TCONS_00016005	-3.03878	-3.50995	-3.03725	BGIOSGA025519	NP_001142653.1uncharacterized protein LOC100274938
TCONS_00022421	-3.03315	-2.89621	-2.01157	BGIOSGA036561	XP_006656162.1 receptor-like protein kinase 5
TCONS_00018434	-3.01305	-2.54346	-0.778386	BGIOSGA005510	XP_006649001.1 chloride channel protein CLC-f-like
TCONS_00036438	-3.00299	0.838057	1.08415	BGIOSGA035820	XP_015698184.1PREDICTED: uncharacterized protein LOC102704106 isoform X2
TCONS_00014965	-2.98871	-2.57058	-3.16148	BGIOSGA007393	XP_002447115.2putative disease resistance protein RGA4 isoform X1
TCONS_00014084	-2.98871	0.599347	0.00844874	#N/A	#N/A
TCONS_00018017	-2.98863	#NA	#NA	BGIOSGA031145	XP_004963692.1uncharacterized protein LOC101753786
TCONS_00020008	-2.98789	-2.2821	-3.16354	BGIOSGA022191	XP_010228038.1uncharacterized protein LOC104581650
TCONS_00035784	-2.98776	-1.24985	-1.16388	BGIOSGA034127	XP_004960738.1transcriptional coactivator YAP1
TCONS_00007820	-2.98749	-2.57211	-4.16454	BGIOSGA037727	XP_015688939.1 calcineurin B-like protein 7
TCONS_00016436	-2.98269	-3.57818	#NA	BGIOSGA005863	XP_024311533.1transcription factor bHLH103 isoform X3
TCONS_00030794	-2.9819	-1.03485	-1.59352	BGIOSGA007204	XP_015690283.1PREDICTED: uncharacterized protein LOC102701624
TCONS_00007103	-2.95091	-3.1254	-4.0015	BGIOSGA009284	XP_006649167.2 carboxyl-terminal-processing peptidase 2, chloroplastic
TCONS_00006546	-2.93087	-3.52042	-2.37858	BGIOSGA037146	XP_006648883.1 B-box zinc finger protein 20-like
TCONS_00009336	-2.92762	0.230372	-0.153999	BGIOSGA022541	XP_015690478.1 FAD-dependent urate hydroxylase-like
TCONS_00016463	-2.92639	-1.9411	-0.541702	BGIOSGA031495	NP_001152262.2transferase, transferring glycosyl groups
TCONS_00028570	-2.92089	-1.66406	-2.04192	#N/A	#N/A
TCONS_00020300	-2.9158	-1.12005	-2.37153	BGIOSGA008043	XP_006656742.2PREDICTED: uncharacterized protein LOC102709091
TCONS_00005744	-2.91222	-3.49999	-1.23113	BGIOSGA021138	XP_006647110.1 peroxidase P7-like
TCONS_00025049	-2.90443	-2.89402	-1.71694	BGIOSGA036271	XP_006654954.1 acetylornithine aminotransferase, mitochondrial
TCONS_00015162	-2.90265	0.567022	0.0606808	BGIOSGA006939	XP_004960085.1chaperone protein dnaJ C76, chloroplastic
TCONS_00028197	-2.87246	-2.92426	-2.68927	BGIOSGA029665	XP_006660223.1 homeobox-leucine zipper protein HOX20-like
TCONS_00025256	-2.86072	-0.214313	0.151172	BGIOSGA032318	XP_006658649.1 cysteine-rich receptor-like protein kinase 15 isoform X1
TCONS_00016684	-2.8565	-2.55505	-1.53838	BGIOSGA037723	XP_014751003.1receptor-like serine/threonine-protein kinase SD1-8
TCONS_00009494	-2.85575	-1.84965	-3.76076	BGIOSGA029806	XP_003558594.1CASP-like protein 2U2
TCONS_00015216	-2.84665	-4.76799	-4.36846	BGIOSGA037882	XP_006653073.1PREDICTED: uncharacterized protein LOC102708432
TCONS_00024498	-2.83169	-0.385808	-1.37388	BGIOSGA024897	XP_006658244.1 transcription factor Maf-like
TCONS_00025835	-2.83169	-0.800845	-1.37388	#N/A	#N/A
TCONS_00028630	-2.83106	-3.3865	#NA	BGIOSGA030145	XP_006644347.1 bidirectional sugar transporter SWEET6b
TCONS_00029037	-2.82684	1.00121	-1.3862	#N/A	#N/A
TCONS_00009788	-2.82275	-0.936379	-2.39657	#N/A	#N/A
TCONS_00009175	-2.81993	-2.39923	-0.988667	BGIOSGA040603	XP_004986011.1uncharacterized protein LOC101778132
TCONS_00035732	-2.81585	#NA	-1.41395	BGIOSGA037216	XP_006663908.1 MADS-box transcription factor 13-like
TCONS_00027250	-2.81572	-1.81956	-0.999237	BGIOSGA032471	XP_006659767.2 exosome complex component RRP41-like
TCONS_00002178	-2.81547	-1.81987	-0.3369	BGIOSGA033298	XP_006645026.1 steroid 5-alpha-reductase DET2
TCONS_00015391	-2.81523	-2.40514	-1.00046	BGIOSGA026343	XP_004975524.1momilactone A synthase
TCONS_00020423	-2.81501	-0.820456	-0.00101326	BGIOSGA003753	XP_006656833.1 protein NRT1/ PTR FAMILY 5.8
TCONS_00004127	-2.8148	3.19423	#NA	BGIOSGA026297	XP_003566966.2lipid phosphate phosphatase 2
TCONS_00026591	-2.81127	-1.60277	-0.0102947	BGIOSGA022575	XP_006659390.1PREDICTED: uncharacterized protein LOC102703053
TCONS_00019583	-2.79138	-3.18378	-2.60653	BGIOSGA020095	XP_006654583.1 cyclin-B1-5 isoform X2
TCONS_00024147	-2.76566	-4.91442	-3.49415	BGIOSGA002711	XP_006645789.1 peroxidase 2-like
TCONS_00034787	-2.76554	-5.36424	-2.37936	BGIOSGA025744	XP_006663415.1 probable apyrase 3
TCONS_00013799	-2.76058	-1.42802	-0.92945	BGIOSGA033262	XP_015697480.1 probable methyltransferase PMT21

transcriptome

TCONS_00038361	-2.76016	#NA	-1.92315	BGIOSGA031454	XP_006663501.1 UPF0481 protein At3g47200-like
TCONS_00032127	-2.75707	0.567248	-1.70854	BGIOSGA032010	XP_015697260.1PREDICTED: uncharacterized protein LOC102703899
TCONS_00002112	-2.75107	-0.844719	-1.53089	BGIOSGA034074	XP_006646452.1 flavonol synthase/flavanone 3-hydroxylase
TCONS_00020662	-2.74553	-1.96341	0.321523	#N/A	#N/A
TCONS_00036568	-2.74302	-5.21891	-2.82045	BGIOSGA034766	XP_006663751.1 BTB/POZ domain-containing protein At3g08570-like
TCONS_00023035	-2.73974	0.19544	-1.85917	BGIOSGA025060	XP_024313239.1uncharacterized protein LOC100833640 isoform X1
TCONS_00024436	-2.73342	-1.49158	-1.42282	BGIOSGA008476	XP_015695169.1 sugar carrier protein C-like
TCONS_00011406	-2.73268	-2.29918	-3.88072	BGIOSGA024937	XP_006650630.1 probable protein phosphatase 2C 34
TCONS_00019325	-2.71984	-1.50755	-2.17607	BGIOSGA017999	XP_003568417.1probable magnesium transporter NIPA4
TCONS_00018234	-2.71856	-0.509167	-0.456774	BGIOSGA012123	XP_006655543.1 probable polygalacturonase At1g80170 isoform X1
TCONS_00016583	-2.71435	-4.52344	-1.68434	BGIOSGA020893	XP_006652790.1 galacturonokinase isoform X1
TCONS_00017233	-2.7109	-1.6349	-0.240679	BGIOSGA001680	XP_004960839.1uncharacterized protein LOC101764560
TCONS_00005586	-2.69856	-2.61988	-1.5199	BGIOSGA013063	XP_006647016.1 premnaspirodiene oxygenase-like
TCONS_00012851	-2.68834	-4.86629	-2.46328	BGIOSGA027900	XP_004982264.1inactive protein RESTRICTED TEV MOVEMENT 2
TCONS_00019277	-2.68032	-3.97046	-3.49964	BGIOSGA002103	XP_015692597.1PREDICTED: uncharacterized protein LOC102712817
TCONS_00032780	-2.68017	-6.5987	-4.19765	BGIOSGA031330	XP_021306528.1uncharacterized protein LOC110431640
TCONS_00004257	-2.67285	0.228275	0.094848	BGIOSGA027387	XP_006659240.1 (DL)-glycerol-3-phosphatase 2 isoform X2
TCONS_00004822	-2.66942	-3.2679	-1.86786	BGIOSGA019901	XP_006645115.1 amino acid permease 8-like
TCONS_00038358	-2.66929	-6.42546	-4.43382	BGIOSGA028484	XP_006663501.1 UPF0481 protein At3g47200-like
TCONS_00009670	-2.66798	-6.26971	-5.87143	BGIOSGA002447	XP_006649724.1 exoglucanase/xylanase-like isoform X2
TCONS_00019551	-2.66187	-1.48932	-3.58881	BGIOSGA017781	XP_015692962.1 fruit bromelain-like
TCONS_00024350	-2.65886	-2.72942	-1.17818	BGIOSGA019131	XP_006658126.1 peroxidase 2-like
TCONS_00033263	-2.65263	-1.37689	-2.62906	BGIOSGA035087	XP_015697624.1PREDICTED: uncharacterized protein LOC102714050
TCONS_00003214	-2.64093	-0.942856	-1.90757	BGIOSGA019094	XP_006643989.2 receptor-like serine/threonine-protein kinase At1g78530
TCONS_00011937	-2.64087	-2.24081	-0.0114848	BGIOSGA011076	XP_015689837.1PREDICTED: uncharacterized protein LOC102700976
TCONS_00000728	-2.64079	-1.60634	-2.76181	BGIOSGA035839	NP_001150894.1ethylene-responsive transcription factor 3
TCONS_00024394	-2.63722	-2.19514	-0.448931	#N/A	#N/A
TCONS_00017627	-2.63586	-3.19662	#NA	BGIOSGA016787	XP_015693111.1 protein DEHYDRATION-INDUCED 19 homolog 2 isoform X1
TCONS_00024629	-2.63442	0.502231	0.543981	BGIOSGA017041	XP_015688878.1 altered inheritance of mitochondria protein 21-like
TCONS_00003065	-2.62868	-4.30724	-2.03384	BGIOSGA021625	XP_006645620.1 peptide-N4-(N-acetyl-beta-glucosaminyl)asparagine amidase A-like
TCONS_00032781	-2.62855	-6.02352	-4.45901	BGIOSGA001542	XP_006645936.2 plastidal glycolate/glycerate translocator 1, chloroplastic
TCONS_00012409	-2.62844	-0.205165	-2.7931	#N/A	#N/A
TCONS_00023133	-2.62748	-1.62138	-0.795527	BGIOSGA025505	XP_004985801.1probable disease resistance protein At5g45440
TCONS_00004429	-2.62664	0.493058	-0.475714	BGIOSGA031189	XP_004970017.1dof zinc finger protein 5
TCONS_00021354	-2.6263	-1.62284	#NA	BGIOSGA031164	XP_006657326.1 peptidyl-prolyl cis-trans isomerase CYP20-1-like
TCONS_00040070	-2.62339	-2.62651	-1.48388	BGIOSGA010437	XP_013443683.1DNA-directed RNA polymerase subunit beta
TCONS_00026168	-2.62244	-0.627718	-0.808186	BGIOSGA008320	XP_020422962.1putative E3 ubiquitin-protein ligase LIN-1
TCONS_00025368	-2.62086	-2.21468	-0.642202	BGIOSGA019008	XP_006657860.1 polygalacturonase inhibitor 1
TCONS_00025219	-2.62019	-1.63055	#NA	BGIOSGA030219	XP_006657778.1 WAT1-related protein At5g64700-like
TCONS_00020891	-2.61983	0.105953	0.272783	BGIOSGA019324	XP_006657059.1 diacylglycerol O-acyltransferase 1-like
TCONS_00006111	-2.61905	-1.63199	-3.49468	BGIOSGA007691	XP_003571092.1isoflavone 2'-hydroxylase
TCONS_00012311	-2.61882	-1.89532	-0.817183	BGIOSGA010686	XP_006651406.1 sugar transporter ERD6-like 16
TCONS_00028029	-2.60797	-1.74982	-2.03786	BGIOSGA003185	XP_015688191.1 mediator-associated protein 2
TCONS_00037603	-2.59789	-4.1967	-3.79684	BGIOSGA036998	XP_021301884.1purple acid phosphatase 2-like
TCONS_00024348	-2.59159	-0.745475	-2.23398	BGIOSGA001846	XP_006658124.1 peroxidase 2-like
TCONS_00024632	-2.59146	-1.36579	-4.3489	#N/A	#N/A
TCONS_00009254	-2.58626	-3.62445	-1.35887	BGIOSGA011784	XP_002468563.2LOW QUALITY PROTEIN: uncharacterized protein LOC8057414
TCONS_00014957	-2.58085	-1.4925	-1.00026	BGIOSGA018909	XP_015691578.1 BAG family molecular chaperone regulator 1-like
TCONS_00014713	-2.56796	-5.16276	-0.853845	BGIOSGA016887	XP_006653652.1 malate dehydrogenase, cytoplasmic-like
TCONS_00024347	-2.5653	-0.219443	-1.41387	BGIOSGA025002	XP_006658123.1 peroxidase 2-like
TCONS_00019155	-2.56498	-4.57255	-2.44095	#N/A	#N/A
TCONS_00023952	-2.56359	0.0940772	0.291319	BGIOSGA005847	XP_006657872.1 40S ribosomal protein S13-2-like
TCONS_00004764	-2.5587	-2.05759	-0.610221	BGIOSGA008303	XP_015698161.1 transcription factor TGA2-like isoform X1
TCONS_00005067	-2.55383	-2.20914	-2.64574	BGIOSGA016634	XP_010240573.2uncharacterized protein LOC100832609 isoform X2
TCONS_00025218	-2.54933	-1.14572	-0.00759874	BGIOSGA002865	XP_014751275.1glucose-6-phosphate/phosphate translocator 2, chloroplastic

transcriptome

TCONS_00017326	-2.54081	-1.74369	-0.00628372	BGIOSGA004116	XP_006654152.1 GDSL esterase/lipase At5g45910-like
TCONS_00029369	-2.53715	-1.08092	-0.646699	BGIOSGA031124	XP_006661461.1 RNA-binding protein 1-like
TCONS_00004005	-2.53241	-0.134261	-0.970502	BGIOSGA014947	XP_006644379.1 mitogen-activated protein kinase kinase kinase 7
TCONS_00007976	-2.53118	0.965792	-0.480271	BGIOSGA006507	XP_003574949.17-dehydrocholesterol reductase
TCONS_00003117	-2.52538	-3.9691	-1.64158	BGIOSGA022347	XP_006648811.2PREDICTED: uncharacterized protein LOC102699290
TCONS_00026182	-2.51524	#NA	#NA	BGIOSGA017422	XP_006659231.1 xyloglucan endotransglucosylase/hydrolase protein 24-like
TCONS_00027907	-2.5137	-2.52311	#NA	BGIOSGA007130	XP_004969731.2wall-associated receptor kinase 3
TCONS_00007967	-2.5122	-4.10995	-2.12456	BGIOSGA006514	XP_002453775.1microtubule-associated serine/threonine-protein kinase 3
TCONS_00036408	-2.51182	-0.940509	0.537458	BGIOSGA027304	XP_022680515.1transcription factor PIF4 isoform X1
TCONS_00025242	-2.51051	-1.52712	-2.71371	BGIOSGA025858	XP_003561395.1ankyrin repeat-containing protein At5g02620
TCONS_00004701	-2.50143	-2.85963	-1.45998	BGIOSGA028723	XP_006662549.1 BEL1-like homeodomain protein 4
TCONS_00028671	-2.50117	-4.01243	-3.35784	BGIOSGA015952	XP_015693578.1PREDICTED: uncharacterized protein LOC102721655
TCONS_00031800	-2.48939	-5.41007	-5.01018	BGIOSGA032490	XP_004983421.1disease resistance protein RGA2
TCONS_00021399	-2.47666	-2.07005	#NA	BGIOSGA006856	XP_015693904.1 transcription factor bHLH93-like
TCONS_00021494	-2.47595	-1.6559	-0.932044	BGIOSGA013307	XP_006655725.1 growth-regulating factor 5
TCONS_00004590	-2.47433	-0.783478	-1.75551	BGIOSGA010850	XP_006644874.2 transcription factor HBP-1b(c1)-like isoform X2
TCONS_00002387	-2.46823	-0.902145	-0.831696	BGIOSGA005022	XP_021319199.1uncharacterized protein LOC8086457
TCONS_00029416	-2.46303	-1.91072	-0.844706	BGIOSGA019564	XP_004953005.1ankyrin repeat and death domain-containing protein 1A
TCONS_00021954	-2.45558	-1.59398	-2.06806	BGIOSGA004890	XP_006656833.1 protein NRT1/ PTR FAMILY 5.8
TCONS_00031401	-2.45491	-4.4693	-3.48478	BGIOSGA031614	XP_006654334.1 AB hydrolase superfamily protein YfhM-like
TCONS_00007948	-2.45111	-1.92899	-1.45894	#N/A	#N/A
TCONS_00001251	-2.44332	-1.11668	-1.38008	BGIOSGA015659	XP_006644329.1 aldehyde dehydrogenase family 2 member C4-like
TCONS_00004588	-2.44279	-1.35552	-0.894224	#N/A	#N/A
TCONS_00008741	-2.44253	-2.55823	-2.64505	BGIOSGA000823	XP_006647834.1 protein NSP-INTERACTING KINASE 1
TCONS_00016273	-2.4413	-2.64123	-5.64521	BGIOSGA006001	XP_015691636.1 phenylalanine ammonia-lyase isoform X1
TCONS_00004343	-2.44073	-1.35823	-0.484224	BGIOSGA013602	XP_002443431.1metal transporter Nramp6
TCONS_00020485	-2.43772	-2.53152	-0.750345	BGIOSGA035939	XP_015693538.1 zinc finger protein CONSTANS-LIKE 16-like
TCONS_00029252	-2.43682	-1.37032	-2.8952	BGIOSGA038518	XP_006660759.1PREDICTED: uncharacterized protein LOC102707798
TCONS_00029270	-2.43647	-0.531235	-0.130544	BGIOSGA005742	XP_015696397.1 3-hydroxy-3-methylglutaryl-coenzyme A reductase 3-like
TCONS_00022408	-2.43396	-1.18778	-3.42719	BGIOSGA020790	XP_002438552.1heat stress transcription factor C-2b isoform X2
TCONS_00026324	-2.43072	-2.37054	-1.33891	#N/A	#N/A
TCONS_00032374	-2.42937	-2.95705	#NA	BGIOSGA007933	XP_006651385.1PREDICTED: uncharacterized protein LOC102702181
TCONS_00003634	-2.42872	-1.95779	-2.51379	#N/A	#N/A
TCONS_00002217	-2.42123	-0.380951	0.052627	#N/A	#N/A
TCONS_00002676	-2.41995	-0.645333	#NA	BGIOSGA016294	XP_024317253.1uncharacterized protein LOC106866428
TCONS_00007198	-2.41756	-3.82692	-1.55431	BGIOSGA020632	XP_004951198.1trihelix transcription factor GTL1-like
TCONS_00012721	-2.41633	-0.649191	-0.544631	#N/A	#N/A
TCONS_00014712	-2.41523	-3.14166	-2.50191	BGIOSGA016886	XP_006652609.1 carotenoid cleavage dioxygenase 7, chloroplastic
TCONS_00002839	-2.41116	0.345139	0.2496	BGIOSGA001568	XP_006643766.1 histone H2B.5
TCONS_00013767	-2.41017	-2.97791	-2.56026	#N/A	#N/A
TCONS_00004951	-2.4091	-0.65721	-1.56297	BGIOSGA031010	XP_004971023.228 kDa ribonucleoprotein, chloroplastic
TCONS_00027016	-2.40861	#NA	#NA	BGIOSGA029066	XP_003572457.2CASP-like protein UU1
TCONS_00016387	-2.40613	-2.53519	-1.32677	BGIOSGA028016	XP_015692154.1 putative glycerol-3-phosphate transporter 1
TCONS_00005342	-2.40572	0.386052	-0.456077	BGIOSGA022179	XP_006646830.1 ADP-ribosylation factor-like isoform X1
TCONS_00033005	-2.40519	-3.98383	-3.57288	#N/A	#N/A
TCONS_00026997	-2.40495	-4.51507	-3.20217	BGIOSGA035957	XP_015695928.1 nucleotide pyrophosphatase/phosphodiesterase-like
TCONS_00004263	-2.40351	-2.24894	-2.16207	BGIOSGA020288	XP_015695521.1 jacalin-related lectin 19
TCONS_00010566	-2.40259	-1.98707	-0.994457	BGIOSGA023196	XP_006650322.1 auxin-responsive protein IAA12-like
TCONS_00031590	-2.40247	-2.98723	-2.57974	BGIOSGA028804	XP_020397541.1probable E3 ubiquitin-protein ligase ATL44
TCONS_00012709	-2.4021	-0.987691	-0.995699	BGIOSGA002703	XP_003562289.1LOB domain-containing protein 37
TCONS_00030438	-2.40082	-1.98931	0.638521	BGIOSGA029364	XP_006660899.1 ribonuclease 1-like
TCONS_00030680	-2.39858	1.56245	-0.00447635	BGIOSGA009637	XP_006650706.1 probable aldehyde oxidase 2
TCONS_00018356	-2.39755	-1.40847	-2.00704	BGIOSGA020408	XP_015693145.1PREDICTED: uncharacterized protein LOC102714924
TCONS_00019509	-2.39673	#NA	-1.59405	BGIOSGA017849	XP_021312803.1protein PIN-LIKES 6
TCONS_00037734	-2.3964	0.0640131	-1.59484	BGIOSGA012231	XP_006651215.1PREDICTED: uncharacterized protein LOC102719475
TCONS_00006714	-2.39634	-1.29452	-3.59499	BGIOSGA029635	XP_006647755.1 abscisic acid 8'-hydroxylase 1

TCONS_00023031	-2.39601	-2.50995	-2.59581	BGIOSGA015758	XP_006658235.1PREDICTED: uncharacterized protein LOC102720064
TCONS_00028246	-2.3888	-3.57517	-1.19809	BGIOSGA027300	XP_015695954.1 probable WRKY transcription factor 2
TCONS_00001001	-2.35812	#NA	-2.51932	#N/A	#N/A
TCONS_00016501	-2.35699	-2.20707	-1.83416	BGIOSGA005820	XP_004976616.1uncharacterized protein LOC101776420
TCONS_00017995	-2.34881	-3.69812	-1.83468	BGIOSGA036092	XP_015692924.1 protein NUCLEAR FUSION DEFECTIVE 4-like
TCONS_00032969	-2.34591	-5.26988	-4.87176	BGIOSGA035372	XP_015691642.1 subtilisin-like protease SBT3.9
TCONS_00010643	-2.34041	1.37713	0.906286	BGIOSGA035696	XP_014752011.1uncharacterized protein LOC100839147
TCONS_00001765	-2.33984	-0.120026	1.17902	BGIOSGA012151	XP_015689817.1 heat stress transcription factor C-1b-like
TCONS_00007545	-2.33798	-0.80816	-2.43589	BGIOSGA017986	XP_006648425.2 subtilisin-like protease SBT5.3
TCONS_00019489	-2.33105	-1.55557	-1.85985	BGIOSGA016990	XP_006654542.1 expansin-A4
TCONS_00005242	-2.32841	-1.51727	-1.68718	BGIOSGA035593	XP_006647067.2 probable LRR receptor-like serine/threonine-protein kinase At3g47570
TCONS_00015348	-2.32068	-1.68152	-2.08054	#N/A	#N/A
TCONS_00029376	-2.31882	-2.53702	-2.50023	BGIOSGA008976	XP_006660877.1 homeobox-leucine zipper protein HOX6-like
TCONS_00008679	-2.31882	-2.61675	-2.0852	BGIOSGA009062	XP_003572869.1basic proline-rich protein
TCONS_00034176	-2.3163	-4.49442	-4.09151	BGIOSGA004404	XP_006663160.1 probable WRKY transcription factor 70
TCONS_00013195	-2.31494	-2.91116	-1.5099	#N/A	#N/A
TCONS_00016489	-2.31493	-0.138591	-2.28755	BGIOSGA012269	XP_006652698.1 probable protein phosphatase 2C 43
TCONS_00021613	-2.31365	0.456454	-0.51309	BGIOSGA008180	XP_006663628.2 glutathione synthetase, chloroplastic-like
TCONS_00035765	-2.31227	-3.6135	-4.77852	BGIOSGA037174	XP_015698837.1 2-alkenal reductase (NADP(+)-dependent)-like
TCONS_00024409	-2.31031	-3.42197	-3.50661	BGIOSGA024410	XP_015694868.1 temperature-sensitive sn-2 acyl-lipid omega-3 desaturase (ferredoxin), chloroplastic-like
TCONS_00011701	-2.30958	-3.07181	-1.99601	BGIOSGA034308	XP_006649531.1 alcohol dehydrogenase-like 7
TCONS_00002094	-2.30711	0.435099	-1.92321	BGIOSGA017481	XP_006644934.1 probable WRKY transcription factor 33
TCONS_00021101	-2.30693	-1.15375	-0.502842	BGIOSGA010162	XP_006656281.1 serine/threonine-protein kinase HT1
TCONS_00017369	-2.30273	-3.47534	-2.21146	BGIOSGA016519	XP_015692517.1 probable aquaporin TIP4-1
TCONS_00028899	-2.29963	-1.89187	-0.640458	BGIOSGA003396	XP_006660583.1 ASC1-like protein 1
TCONS_00029130	-2.29917	-4.21437	-2.4896	BGIOSGA025001	XP_004956981.11-aminocyclopropane-1-carboxylate oxidase 1
TCONS_00032312	-2.29013	-1.97952	-0.100628	BGIOSGA017361	XP_004982999.1putative RING-type E3 ubiquitin transferase C3H69
TCONS_00002590	-2.28247	-1.18558	-0.783652	BGIOSGA012710	XP_006645353.1 cationic peroxidase SPC4-like
TCONS_00020322	-2.28204	-1.27045	-2.44133	BGIOSGA003758	XP_006655897.1PREDICTED: uncharacterized protein LOC102711892
TCONS_00031869	-2.27936	#NA	-0.747659	BGIOSGA032265	XP_004983363.1small heat shock protein, chloroplastic
TCONS_00027115	-2.2789	-1.53731	-1.86431	BGIOSGA029138	XP_015695744.1PREDICTED: uncharacterized protein LOC102716894
TCONS_00015584	-2.27669	-1.86201	-1.86987	BGIOSGA015384	XP_021320517.1G-type lectin S-receptor-like serine/threonine-protein kinase SD2-5
TCONS_00033923	-2.27507	-2.86406	-1.87392	BGIOSGA019098	XP_015697111.1 serine carboxypeptidase-like 13 isoform X2
TCONS_00001417	-2.27441	#NA	-2.13861	BGIOSGA001389	XP_006644401.1 PLASMODESMATA CALLOSE-BINDING PROTEIN 3-like
TCONS_00030318	-2.27375	-4.18765	-2.97674	BGIOSGA003901	XP_006659607.2 RNA pseudouridine synthase 3, mitochondrial
TCONS_00010598	-2.27341	#NA	-0.878054	BGIOSGA037369	XP_006651634.1 probable cyclic nucleotide-gated ion channel 6
TCONS_00000648	-2.27313	-2.28155	-0.141797	BGIOSGA014042	XP_006645753.1 cationic peroxidase 1 isoform X1
TCONS_00026488	-2.27202	-1.54598	-0.881507	BGIOSGA030501	XP_003575299.1serine/threonine-protein phosphatase 6 regulatory ankyrin repeat subunit A
TCONS_00002962	-2.27183	-1.0608	-1.14503	BGIOSGA020673	XP_003569894.1uncharacterized protein LOC100835106
TCONS_00029309	-2.27164	-2.28342	-3.4674	BGIOSGA029487	XP_021302227.1putative F-box/FBD/LRR-repeat protein At4g03220 isoform X1
TCONS_00005291	-2.27109	0.52324	-1.66142	BGIOSGA036416	XP_022680207.1uncharacterized protein LOC101754764 isoform X2
TCONS_00020708	-2.27029	-1.06273	-0.532152	BGIOSGA001307	XP_006656970.2 pentatricopeptide repeat-containing protein At2g03880, mitochondrial
TCONS_00001341	-2.26135	-2.48732	-1.71778	BGIOSGA025370	XP_004969140.1uncharacterized protein LOC101755969
TCONS_00036412	-2.26089	-1.67045	0.065446	BGIOSGA018909	XP_006664181.1 NAC domain-containing protein 21/22-like
TCONS_00021304	-2.24769	-4.74562	-3.17163	BGIOSGA011721	XP_006657289.1 xyloglucan endotransglucosylase/hydrolase protein 22-like
TCONS_00021300	-2.24635	-3.42539	-1.39071	BGIOSGA011559	XP_006656422.2 peroxidase 16-like
TCONS_00004799	-2.2458	-0.80638	-1.40845	BGIOSGA026913	XP_024315516.1G-type lectin S-receptor-like serine/threonine-protein kinase At2g19130
TCONS_00019024	-2.23878	-3.17707	-3.31444	BGIOSGA018337	XP_015697712.1 probable plastid-lipid-associated protein 6, chloroplastic
TCONS_00022105	-2.23757	-0.465586	-1.82029	BGIOSGA037096	XP_006656033.1 zinc finger protein CONSTANS-LIKE 9-like isoform X1
TCONS_00022379	-2.23648	-0.490491	-1.98485	BGIOSGA000795	XP_008659608.1heavy metal-associated isoprenylated plant protein 29 isoform X1
TCONS_00014810	-2.2359	-0.813122	-1.40135	BGIOSGA004933	XP_006652690.1 protein DOWNY MILDEW RESISTANCE 6-like
TCONS_00026784	-2.22866	-0.159247	-3.00448	BGIOSGA023824	XP_003574615.1tuliposide A-converting enzyme 1, chloroplastic

TCONS_00031324	-2.22528	-3.80602	-2.71821	BGIOSGA020284	XP_015697118.1 probable cytokinin riboside 5'-monophosphate phosphoribohydrolase LOGL10 isoform X2
TCONS_00008753	-2.21756	-3.79402	-0.755022	BGIOSGA005679	XP_006647844.2PREDICTED: uncharacterized protein LOC102711043
TCONS_00039308	-2.2167	-4.13876	-3.41769	BGIOSGA039534	XP_012849888.1 retrovirus-related Pol polyprotein from transposon TNT 1-94
TCONS_00025444	-2.21307	-3.61996	-2.41313	BGIOSGA040479	NP_001169643.1uncharacterized LOC100383524
TCONS_00031692	-2.21274	-0.526002	-2.22223	BGIOSGA037904	XP_006662114.1 urea-proton symporter DUR3
TCONS_00005484	-2.21203	-0.25657	0.923921	BGIOSGA007596	XP_006657576.1PREDICTED: uncharacterized protein LOC102720614
TCONS_00020407	-2.20953	-2.99572	-1.51656	#N/A	#N/A
TCONS_00014772	-2.20297	0.196773	-0.0118298	BGIOSGA037563	XP_006652664.1 protein DETOXIFICATION 49-like
TCONS_00019050	-2.20195	-2.06754	-2.66969	BGIOSGA022831	XP_004962463.1receptor-like protein kinase FERONIA
TCONS_00012854	-2.20174	0.909478	-0.0148567	BGIOSGA027267	XP_003570737.1U-box domain-containing protein 44
TCONS_00036563	-2.19843	-4.37661	-5.5587	BGIOSGA004404	XP_006663160.1 probable WRKY transcription factor 70
TCONS_00016964	-2.19692	-1.39972	-1.43021	BGIOSGA018109	XP_015692771.1 serine/arginine-rich splicing factor SR45-like
TCONS_00034181	-2.19188	-5.49366	-2.80975	BGIOSGA027213	XP_006663751.1 BTB/POZ domain-containing protein At3g08570-like
TCONS_00023843	-2.18839	-1.65289	-1.90682	BGIOSGA015857	XP_015694662.1 putative cysteine-rich receptor-like protein kinase 20
TCONS_00013643	-2.18807	-1.66596	-1.61094	BGIOSGA015791	XP_015692110.1PREDICTED: uncharacterized protein LOC102711421
TCONS_00017722	-2.1868	-4.92979	-1.57691	BGIOSGA007571	XP_014660343.1UDP-D-xylose:L-fucose alpha-1,3-D-xylosyltransferase MGP4
TCONS_00016965	-2.18482	-3.54564	-4.46581	BGIOSGA007304	XP_015692446.1 WAT1-related protein At5g64700-like
TCONS_00010423	-2.17714	-3.99696	-1.42065	BGIOSGA013019	XP_006650273.1 formimidoyltransferase-cyclodeaminase-like isoform X2
TCONS_00013198	-2.17485	-2.68381	1.09374	BGIOSGA024956	XP_006650608.1 60S ribosomal protein L13a-4
TCONS_00031321	-2.17485	0.316188	0.274313	BGIOSGA025556	XP_002467074.2uncharacterized protein LOC8067412
TCONS_00022660	-2.16633	-0.694016	0.0727825	#N/A	#N/A
TCONS_00002435	-2.16592	-2.98239	-2.01889	BGIOSGA016097	XP_002456862.1uncharacterized protein LOC8079809
TCONS_00022399	-2.16505	0.76397	-1.25232	#N/A	#N/A
TCONS_00029280	-2.16398	-2.49482	-2.0334	BGIOSGA008063	NP_001151333.1protein brittle-1
TCONS_00014354	-2.16211	-2.34071	-1.59653	BGIOSGA009886	XP_006652346.1 7-deoxyloganetin glucosyltransferase-like
TCONS_00006544	-2.16032	-1.04615	-1.11444	BGIOSGA037060	XP_015688677.1 GATA transcription factor 5-like
TCONS_00032595	-2.15819	1.13842	0.605168	BGIOSGA034536	XP_006662532.1 cytochrome P450 704C1-like
TCONS_00014954	-2.15455	-3.9738	-2.83593	BGIOSGA035533	XP_015692132.1 F-box/kelch-repeat protein At5g60570-like
TCONS_00002034	-2.15362	0.099619	-1.28077	BGIOSGA004667	NP_001149605.1serine-type endopeptidase inhibitor precursor
TCONS_00031966	-2.15268	2.19813	0.301779	BGIOSGA032181	XP_015697115.1 uncharacterized calcium-binding protein At1g02270
TCONS_00011725	-2.15179	-0.709717	-0.285432	BGIOSGA009517	XP_002465702.1uncharacterized protein LOC8054253
TCONS_00004098	-2.15179	-2.12475	-0.115507	#N/A	#N/A
TCONS_00032895	-2.15043	-2.71119	-2.28886	BGIOSGA017330	XP_006664297.2PREDICTED: uncharacterized protein LOC102709420
TCONS_00019536	-2.14969	-2.71201	-0.705785	BGIOSGA034470	XP_015692828.1 histone H3-5-like
TCONS_00029405	-2.14899	-4.29774	-0.418051	BGIOSGA000248	XP_015695001.1 CRIB domain-containing protein RIC4-like isoform X2
TCONS_00021529	-2.14752	#NA	-2.29625	BGIOSGA017662	XP_006655737.2 oligopeptide transporter 7-like
TCONS_00020006	-2.14607	-2.7161	0.0219967	BGIOSGA029432	XP_006656577.1PREDICTED: uncharacterized protein LOC102699792
TCONS_00003751	-2.14527	-1.71704	#NA	BGIOSGA001456	XP_006644287.1 WAT1-related protein At5g64700-like
TCONS_00024653	-2.14497	#NA	-2.30273	BGIOSGA013565	XP_006658323.1 non-specific lipid transfer protein GPI-anchored 2-like
TCONS_00013201	-2.1444	-1.71807	-0.719211	BGIOSGA029269	XP_006650610.1 PLASMODESMATA CALLOSE-BINDING PROTEIN 2-like
TCONS_00008121	-2.14426	0.982205	0.0174086	BGIOSGA026037	XP_015689595.1 protein FAF-like, chloroplastic
TCONS_00012106	-2.14374	-3.30383	0.196656	BGIOSGA011874	XP_006651050.1 peroxidase 44-like
TCONS_00018249	-2.14257	-1.72028	#NA	#N/A	#N/A
TCONS_00027674	-2.14167	-0.914038	-0.311075	BGIOSGA017354	NP_001141770.1fiber protein Fb34 precursor
TCONS_00009969	-2.14157	-1.72151	-2.31131	BGIOSGA000583	XP_006650007.1 WRKY transcription factor 44-like
TCONS_00011670	-2.14139	0.863228	-1.31177	BGIOSGA011338	XP_006649497.1 protein LURP-one-related 6-like
TCONS_00017632	-2.14139	-0.219234	-0.4373	BGIOSGA013688	XP_003566333.1RING-H2 finger protein ATL39
TCONS_00006261	-2.14122	-0.721955	0.687785	BGIOSGA016453	XP_006647372.1 NAC transcription factor 29-like isoform X2
TCONS_00001429	-2.14113	#NA	0.494922	BGIOSGA004059	XP_015696662.1 CBS domain-containing protein CBSX3, mitochondrial-like isoform X1
TCONS_00004141	-2.14079	-1.40055	-0.143351	BGIOSGA015169	XP_012701989.1splicing factor U2af small subunit B
TCONS_00019479	-2.14033	-2.72307	-2.31445	BGIOSGA025417	XP_006655407.1PREDICTED: uncharacterized protein LOC102721368
TCONS_00019373	-2.13996	-0.138558	0.00656142	BGIOSGA036887	XP_006654465.1 RING-H2 finger protein ATL72-like
TCONS_00003111	-2.13989	-4.30857	-3.9005	BGIOSGA018582	XP_006643923.1 bidirectional sugar transporter SWEET3b
TCONS_00036101	-2.13969	-0.238438	-0.550519	#N/A	#N/A
TCONS_00026092	-2.1386	-3.09569	-4.97744	BGIOSGA014345	XP_015695988.1 probable LRR receptor-like serine/threonine-protein kinase At1g56140

TCONS_00023613	-2.1374	-1.1418	-0.234331	#N/A	#N/A
TCONS_00020184	-2.13591	-1.40672	-1.74055	BGIOSGA001050	XP_014752502.1transcription factor SPATULA
TCONS_00030545	-2.13506	-1.72972	-3.91259	BGIOSGA025018	XP_006660983.1 stem bromelain-like
TCONS_00002591	-2.13488	-1.72995	-1.32807	BGIOSGA012703	XP_015688315.1 cationic peroxidase SPC4-like
TCONS_00021939	-2.13478	-3.73007	-3.32831	BGIOSGA026963	XP_006656823.1 cyclin-D1-1-like
TCONS_00012573	-2.13426	0.501935	-2.3296	BGIOSGA037797	XP_006651561.1 protein DETOXIFICATION 40-like
TCONS_00025820	-2.13372	#NA	-1.33095	BGIOSGA035910	XP_015695754.1 kinesin-1-like protein PSS1
TCONS_00030322	-2.13306	-1.50985	-1.18058	BGIOSGA028015	XP_006660806.1 UDP-glucuronate 4-epimerase 6-like
TCONS_00009996	-2.13191	-0.511284	-0.460937	BGIOSGA012550	XP_006650025.1 zinc finger CCCH domain-containing protein 22
TCONS_00008067	-2.1319	-0.478435	-1.76607	BGIOSGA036001	XP_015689429.1PREDICTED: uncharacterized protein At2g33490
TCONS_00039815	-2.13142	1.75065	-0.392824	#N/A	XP_013443004.1senescence-associated protein
TCONS_00005649	-2.13046	-0.695961	-1.70154	BGIOSGA027042	XP_015689281.1 ABC transporter G family member 39
TCONS_00005082	-2.11844	-1.72961	-1.10213	BGIOSGA035359	XP_004971226.1amino-acid permease BAT1 homolog isoform X1
TCONS_00023343	-2.10274	-2.32605	#NA	BGIOSGA002134	XP_010918573.1 U-box domain-containing protein 33-like
TCONS_00031555	-2.10168	-3.06108	-3.46751	BGIOSGA038274	XP_006662555.1 aspartic proteinase nepenthesin-1-like
TCONS_00014186	-2.09663	-3.03003	-0.994607	BGIOSGA008423	XP_004975533.1uncharacterized protein LOC101752917
TCONS_00007692	-2.093	-2.43154	-4.2031	BGIOSGA004483	XP_006647116.1 potassium channel KAT1
TCONS_00009876	-2.08475	0.235573	-0.52062	BGIOSGA012431	XP_006651287.1 CASP-like protein 4B2
TCONS_00019877	-2.07893	-0.529826	-2.9988	BGIOSGA026081	XP_015692977.1 PLASMODESMATA CALLOSE-BINDING PROTEIN 5-like
TCONS_00004262	-2.07868	-2.55445	-2.28146	BGIOSGA000942	XP_006644585.1 anoctamin-like protein Os01g0706700
TCONS_00009603	-2.07696	-0.669824	0.0551751	BGIOSGA012148	XP_004985154.2probable UDP-3-O-acyl-N-acetylglucosamine deacetylase 2, mitochondrial
TCONS_00026634	-2.07506	-0.824215	-1.78603	BGIOSGA010698	XP_003572210.1probable transcription factor RL9
TCONS_00000300	-2.07414	-1.82538	-1.59568	BGIOSGA019621	XP_003569538.4UDP-glycosyltransferase 73C1
TCONS_00033917	-2.07372	-6.82869	-2.25143	BGIOSGA008366	XP_015697930.1PREDICTED: uncharacterized protein LOC102705204 isoform X1
TCONS_00002257	-2.07204	-3.39553	-0.457089	BGIOSGA002935	XP_006646518.2 protein NRT1/ PTR FAMILY 5.10-like
TCONS_00017539	-2.06653	-1.51583	-1.35177	BGIOSGA037152	XP_004962492.1wall-associated receptor kinase-like 20
TCONS_00027911	-2.06576	-0.87719	-1.50492	BGIOSGA002978	XP_002444204.1phosphoenolpyruvate carboxylase 2-like
TCONS_00002191	-2.06494	-1.97212	-1.75527	BGIOSGA017951	XP_006645042.1 auxin transporter-like protein 1
TCONS_00018871	-2.06372	-3.84328	-1.13185	#N/A	#N/A
TCONS_00019606	-2.06213	-1.84701	-1.92903	BGIOSGA017752	XP_004951295.1ribosome production factor 2 homolog
TCONS_00004145	-2.06107	-0.0788943	-0.77581	BGIOSGA036689	XP_006644499.1 putative receptor protein kinase ZmPK1
TCONS_00022521	-2.05346	-0.0215244	-1.86259	BGIOSGA034555	XP_015694450.1 putative receptor protein kinase ZmPK1
TCONS_00023576	-2.05284	-0.387158	-0.309946	BGIOSGA025604	XP_015695411.1 oxidation resistance protein 1 isoform X1
TCONS_00009946	-2.05266	-5.56172	-2.35631	BGIOSGA012830	XP_015690582.1PREDICTED: uncharacterized protein LOC102704685
TCONS_00014170	-2.05264	-1.29357	-0.714545	BGIOSGA035823	XP_006652240.1 probable BOI-related E3 ubiquitin-protein ligase 3
TCONS_00005202	-2.05142	0.125883	-0.313529	BGIOSGA001076	XP_015696091.1 GATA transcription factor 16-like
TCONS_00032013	-2.0488	-4.45391	-0.966014	BGIOSGA029560	XP_015697218.1 UDP-glycosyltransferase 83A1-like
TCONS_00034525	-2.04848	-0.646957	-0.966811	BGIOSGA016309	XP_015697762.1 serine carboxypeptidase-like 42
TCONS_00006246	-2.04651	0.406341	-2.371	BGIOSGA013560	XP_006647362.1 ammonium transporter 3 member 3
TCONS_00027446	-2.04477	-2.8774	-2.12574	BGIOSGA010555	XP_004972725.1nuclear transcription factor Y subunit B-11
TCONS_00033948	-2.04131	-0.315935	-1.00307	#N/A	#N/A
TCONS_00037545	-2.04009	-0.980556	0.296385	BGIOSGA037806	XP_015698183.1PREDICTED: uncharacterized protein LOC102704106 isoform X1
TCONS_00002291	-2.03996	-2.62458	-1.80197	BGIOSGA004929	XP_024316012.1protein DMP7
TCONS_00008151	-2.0377	-5.59899	-2.0071	BGIOSGA010431	XP_021302307.1uncharacterized protein LOC110429892 isoform X1
TCONS_00029277	-2.03737	0.594536	-0.108018	BGIOSGA031020	XP_010238314.1uncharacterized protein LOC100833007 isoform X2
TCONS_00014611	-2.03735	-3.67484	-4.27662	BGIOSGA023490	XP_015691666.1 auxin response factor 10-like
TCONS_00012154	-2.03596	-4.21459	-0.226988	BGIOSGA013101	XP_006649985.1 transcription factor WER-like
TCONS_00034321	-2.03455	-1.04645	-0.356036	BGIOSGA017208	XP_015698112.1 transcription factor HBP-1b(c38)-like
TCONS_00015270	-2.03445	-1.66643	-2.88047	BGIOSGA009834	XP_015691995.1 ATP sulfurylase 2-like
TCONS_00000165	-2.03389	-3.21721	-2.81711	BGIOSGA012138	XP_004968148.1uncharacterized protein LOC101756502
TCONS_00032660	-2.02457	-0.480456	-1.45194	BGIOSGA031513	XP_004983723.1scarecrow-like protein 6 isoform X1
TCONS_00011503	-2.02228	-1.44119	-0.558338	BGIOSGA011523	XP_015691230.1PREDICTED: LOW QUALITY PROTEIN: uncharacterized protein LOC102705985
TCONS_00026911	-2.01957	-3.30606	0.162487	BGIOSGA032653	XP_004973644.1cytochrome P450 76M5
TCONS_00002719	-2.01729	-3.14764	-1.45883	BGIOSGA034403	XP_006643720.2PREDICTED: uncharacterized protein LOC102705568
TCONS_00033091	-2.01719	-1.46886	-1.71587	BGIOSGA004814	XP_003567273.1steroid 5-alpha-reductase DET2
TCONS_00026761	-2.01322	-2.34833	-1.83263	BGIOSGA004875	XP_015695634.1 aspartic proteinase nepenthesin-1-like

TCONS_00021504	-2.01303	-5.93353	-2.72619	BGIOSGA003966	XP_002451310.1aspartyl protease family protein At5g10770
TCONS_00003528	-2.00757	0.0355429	-3.11387	BGIOSGA032278	XP_015688641.1 rapid alkalinization factor-like
TCONS_00015544	-2.0071	#NA	#NA	BGIOSGA010748	NP_001148034.1flowering promoting factor-like 1
TCONS_00011224	-2.00443	-0.239558	-1.00902	BGIOSGA002775	XP_006650798.1 floral homeotic protein APETALA 2-like
TCONS_00004999	-2.00184	0.536802	-0.707983	BGIOSGA027245	XP_006645258.2 disease resistance protein RPP13-like
TCONS_00021570	-1.99768	#NA	-1.55378	#N/A	#N/A
TCONS_00024175	-1.99375	-2.46328	-2.39825	BGIOSGA030892	XP_004958444.1cytochrome P450 709B2
TCONS_00026759	-1.99253	-1.98094	-2.15182	BGIOSGA005531	XP_006660196.1 nitrate reductase [NADH] 1
TCONS_00001533	-1.99161	0.0179515	-1.15416	BGIOSGA004166	XP_006646181.1PREDICTED: uncharacterized protein LOC102713233
TCONS_00019876	-1.99149	-1.69269	-1.15447	#N/A	#N/A
TCONS_00000445	-1.99057	-2.56828	-0.156791	BGIOSGA003380	XP_006645643.1PREDICTED: uncharacterized protein LOC102714248
TCONS_00011794	-1.98939	-3.56973	-3.15977	BGIOSGA011143	XP_014755488.1zinc finger protein 8
TCONS_00011644	-1.98925	-2.49718	-2.31748	BGIOSGA006190	XP_015691058.1 sucrose transport protein SUT1
TCONS_00002935	-1.98887	-1.57037	-2.16107	BGIOSGA002288	XP_004968380.1transcription factor HY5
TCONS_00021214	-1.9884	-2.57097	-3.16226	BGIOSGA007333	XP_021316104.1MADS-box protein ZMM17 isoform X1
TCONS_00011526	-1.98762	-3.57195	-1.57925	BGIOSGA011503	XP_006649350.1 glutathione S-transferase F11-like
TCONS_00003119	-1.98667	-3.57316	-0.581655	BGIOSGA004145	XP_006643927.1 V-type proton ATPase subunit E
TCONS_00015676	-1.98483	0.331412	1.1142	BGIOSGA015303	XP_004975313.1protein CMSS1
TCONS_00022389	-1.98362	-4.16197	-0.951826	BGIOSGA023528	XP_006657042.1 peroxidase P7-like
TCONS_00030062	-1.98343	-1.99229	#NA	BGIOSGA029746	XP_015696617.1 UBP1-associated proteins 1C-like
TCONS_00004104	-1.98284	-2.49053	-3.17616	BGIOSGA036419	XP_006646165.1 G-box-binding factor 3-like
TCONS_00011150	-1.98165	-0.935627	-0.594133	BGIOSGA020192	XP_002466268.1LEAF RUST 10 DISEASE-RESISTANCE LOCUS RECEPTOR-LIKE PROTEIN KINASE-like 1.5
TCONS_00029351	-1.98131	-1.29981	-2.52788	BGIOSGA000865	XP_006664936.2 crocetin glucosyltransferase 2-like
TCONS_00000716	-1.98101	-1.99533	-2.85876	BGIOSGA027183	XP_006645792.1 polygalacturonase At1g48100-like
TCONS_00011516	-1.98065	-0.332821	-0.181586	BGIOSGA010894	XP_015691262.1 probable acyl-activating enzyme 1, peroxisomal
TCONS_00008180	-1.97423	-0.604753	-0.67438	BGIOSGA006259	XP_006647374.1 tRNA (guanine(10)-N2)-methyltransferase homolog
TCONS_00000125	-1.97359	-0.829762	-2.09565	BGIOSGA024868	XP_006645459.2 putative receptor-like protein kinase At4g00960
TCONS_00009335	-1.96912	-6.0216	#NA	BGIOSGA022541	XP_015690478.1 FAD-dependent urate hydroxylase-like
TCONS_00012112	-1.96327	-4.55704	-4.5695	BGIOSGA033804	XP_015689866.1 myb-related protein P-like
TCONS_00019917	-1.95466	-0.522798	0.529637	BGIOSGA001422	XP_006655659.1 vacuolar cation/proton exchanger 1b-like
TCONS_00030466	-1.95302	-2.86422	-2.13727	BGIOSGA002371	NP_001306682.1hypothetical protein
TCONS_00008342	-1.94998	-2.86805	-2.88179	BGIOSGA006080	XP_015689016.1 probable protein phosphatase 2C 21
TCONS_00000079	-1.94348	-1.50529	-2.66864	BGIOSGA011326	XP_006651417.2PREDICTED: uncharacterized protein LOC102712794
TCONS_00036413	-1.9433	-2.47275	-0.695697	BGIOSGA010637	XP_006664181.1 NAC domain-containing protein 21/22-like
TCONS_00035714	-1.94181	-2.57988	-0.890374	BGIOSGA000394	XP_015698432.1 amino acid permease 6-like isoform X2
TCONS_00032776	-1.94025	-2.44047	-3.71573	BGIOSGA032372	XP_004983539.1probable xyloglucan endotransglucosylase/hydrolase protein 28
TCONS_00026270	-1.9395	0.1597	-0.187993	BGIOSGA011183	XP_004973139.1putative receptor-like protein kinase At1g80870
TCONS_00002672	-1.93945	-3.47302	-2.06848	BGIOSGA015756	XP_004967946.1swi5-dependent recombination DNA repair protein 1 homolog
TCONS_00012351	-1.93631	-0.381005	-0.426558	BGIOSGA010640	XP_010228570.1RNA-binding KH domain-containing protein RCF3
TCONS_00020402	-1.93557	-2.42346	-3.07811	BGIOSGA009748	XP_006656810.1 probable mannan synthase 3
TCONS_00030816	-1.93327	-0.916339	-1.55213	BGIOSGA024379	XP_015695317.1PREDICTED: uncharacterized protein LOC102721177
TCONS_00000490	-1.93316	-1.46729	-2.7412	BGIOSGA028533	XP_015698965.1 pentatricopeptide repeat-containing protein At5g25630
TCONS_00000982	-1.93311	0.597897	0.697421	BGIOSGA003586	XP_015698079.1 tRNA (guanine(37)-N1)-methyltransferase 1
TCONS_00024237	-1.93087	-1.95854	-2.60097	BGIOSGA026281	XP_015695132.1PREDICTED: uncharacterized protein LOC102715406
TCONS_00034559	-1.93056	1.4178	-0.701271	BGIOSGA032070	XP_015698105.1 putative disease resistance RPP13-like protein 3
TCONS_00009138	-1.92785	-3.67511	-1.95419	BGIOSGA007353	XP_021305642.1uncharacterized protein LOC110431213 isoform X3
TCONS_00020141	-1.92759	-2.37255	-0.123695	BGIOSGA022338	XP_015694424.1 putative lipase YDR444W isoform X4
TCONS_00009498	-1.91632	-1.6729	-1.83632	BGIOSGA029642	XP_006649565.1 protein indeterminate-domain 2-like
TCONS_00037514	-1.91586	-2.44376	0.141492	BGIOSGA024116	XP_006664184.1 putative serine/threonine-protein kinase
TCONS_00032690	-1.91578	-2.66646	-1.38843	BGIOSGA031419	XP_006662053.1 S-norcoclaurine synthase 1-like
TCONS_00001735	-1.91468	-1.95479	-1.73811	BGIOSGA022182	XP_004969793.1senescence/dehydration-associated protein At4g35985, chloroplastic
TCONS_00003269	-1.91141	-3.63864	-2.53322	BGIOSGA031952	XP_006644026.2 cyclic dof factor 2-like
TCONS_00037782	-1.9015	0.0908466	-0.768984	BGIOSGA030880	XP_015697732.1 probable glucuronosyltransferase Os03g0107900
TCONS_00009224	-1.90118	-3.49451	-0.506732	BGIOSGA031498	XP_002468602.2pre-mRNA-splicing ATP-dependent RNA helicase prp28

transcriptome

TCONS_00034115	-1.89995	0.361919	-1.77282	BGIOSGA002974	XP_006663143.1 fatty acid desaturase DES3-like
TCONS_00021936	-1.89887	0.087543	0.0725067	BGIOSGA001108	XP_002436768.2protein ALTERED XYLOGLUCAN 4
TCONS_00021539	-1.89863	-2.91277	-1.29068	BGIOSGA007655	XP_006656596.1 protein TPX2-like isoform X3
TCONS_00011929	-1.89634	-0.361047	-1.07781	BGIOSGA032182	XP_006649765.1 phosphoenolpyruvate carboxykinase [ATP]
TCONS_00035239	-1.89468	-2.9709	-2.07638	BGIOSGA032824	XP_015697861.1PREDICTED: uncharacterized protein LOC102707256
TCONS_00016063	-1.89277	-2.43588	-1.80534	BGIOSGA014915	XP_006652394.1 cytochrome P450 724B1
TCONS_00028972	-1.88737	-1.57299	-0.0929982	BGIOSGA018135	XP_006661204.1 ABC transporter G family member 17-like
TCONS_00018809	-1.88624	-0.404352	-0.391226	BGIOSGA031532	XP_006654148.2 shaggy-related protein kinase eta-like
TCONS_00034122	-1.88416	-1.09886	-1.73346	BGIOSGA030709	XP_006663711.1 transcription activator GLK2-like
TCONS_00025276	-1.88053	-1.2666	-0.944356	BGIOSGA016189	XP_006657805.1 cysteine-rich receptor-like protein kinase 10
TCONS_00019000	-1.87922	-1.58316	-1.63152	BGIOSGA006117	XP_014757547.1phosphatidate cytidylyltransferase, mitochondrial isoform X3
TCONS_00002855	-1.87658	-1.20721	-1.10943	BGIOSGA018758	XP_006643769.1 serine/arginine-rich splicing factor RS2Z32-like isoform X1
TCONS_00013491	-1.87644	-1.32839	-1.84878	BGIOSGA009499	NP_001308334.1uncharacterized LOC100126915
TCONS_00006148	-1.87323	-1.80638	-2.55505	BGIOSGA007117	XP_015689293.1 tyrosine-sulfated glycopeptide receptor 1-like
TCONS_00020487	-1.87126	-2.04471	-0.119719	BGIOSGA024994	XP_010227773.1protein NRT1/ PTR FAMILY 3.1
TCONS_00038638	-1.87021	-1.88401	-1.8467	BGIOSGA034329	XP_012849888.1 retrovirus-related Pol polyprotein from transposon TNT 1-94
TCONS_00002214	-1.86662	-1.45481	-0.85655	BGIOSGA029710	XP_003567294.1NAC domain-containing protein 90
TCONS_00015203	-1.86484	-1.34589	0.707822	BGIOSGA033437	XP_015691947.1PREDICTED: uncharacterized protein LOC102702841
TCONS_00028165	-1.86236	-2.34939	#NA	#N/A	#N/A
TCONS_00001773	-1.86201	-4.13047	-3.86801	BGIOSGA028061	XP_003567088.1anthocyanidin 5,3-O-glucosyltransferase
TCONS_00036498	-1.86108	-0.568696	0.857627	BGIOSGA032243	XP_002442614.1protein NUCLEAR FUSION DEFECTIVE 4
TCONS_00015827	-1.85783	-5.52545	-4.06415	#N/A	#N/A
TCONS_00004173	-1.8575	-2.41148	-2.21226	BGIOSGA001025	XP_002458314.1two pore calcium channel protein 1
TCONS_00008005	-1.8548	-0.774535	-0.901667	#N/A	#N/A
TCONS_00009521	-1.85361	0.297787	0.297798	BGIOSGA037691	XP_015691073.1 calcium-transporting ATPase 2, plasma membrane-type-like isoform X2
TCONS_00012348	-1.85337	-1.70945	-1.62841	BGIOSGA010641	XP_010228570.1RNA-binding KH domain-containing protein RCF3
TCONS_00012741	-1.85292	-1.36188	#NA	BGIOSGA010225	XP_012704286.1uncharacterized protein LOC101780262 isoform X1
TCONS_00021795	-1.85203	0.636994	#NA	BGIOSGA021720	XP_006655883.1PREDICTED: uncharacterized protein LOC102707779
TCONS_00036049	-1.85203	-1.36301	-1.90845	#N/A	#N/A
TCONS_00005832	-1.85116	-2.36408	-0.325621	BGIOSGA007957	XP_006647156.1 cytochrome c-type biogenesis CcmH-like mitochondrial protein
TCONS_00019936	-1.85116	0.220882	1.41134	BGIOSGA013657	XP_006656565.1 pentatricopeptide repeat-containing protein At1g08070, chloroplastic-like
TCONS_00018426	-1.84958	-2.59438	-1.45431	BGIOSGA029477	XP_015692983.1 inactive protein RESTRICTED TEV MOVEMENT 2-like
TCONS_00020674	-1.84793	#NA	#NA	#N/A	#N/A
TCONS_00012265	-1.84754	-5.8718	-6.25636	BGIOSGA016155	XP_002445531.1alpha-humulene synthase
TCONS_00028039	-1.8475	-0.737179	-0.951323	BGIOSGA011897	XP_006659417.1 protein TIFY 6a-like
TCONS_00007331	-1.84634	-3.6092	-1.09647	BGIOSGA018232	XP_015688452.1 auxin response factor 5
TCONS_00028268	-1.84576	-1.37054	0.0761298	BGIOSGA034437	XP_006648618.1 probable mediator of RNA polymerase II transcription subunit 26c
TCONS_00023498	-1.84507	-1.37132	#NA	BGIOSGA025531	NP_001148038.2PVR3-like protein precursor
TCONS_00031494	-1.84376	-0.372823	-1.92882	BGIOSGA016953	XP_004985957.1uncharacterized protein LOC101757701
TCONS_00020115	-1.84376	-0.050895	-1.92882	#N/A	#N/A
TCONS_00007168	-1.83813	-0.000469832	-0.564233	#N/A	#N/A
TCONS_00001405	-1.83754	-1.60978	-1.45345	BGIOSGA026210	XP_006644396.1 cytochrome P450 72A14-like
TCONS_00022534	-1.83675	-2.47806	-2.01795	BGIOSGA035892	XP_015694996.1 CRIB domain-containing protein RIC4-like isoform X1
TCONS_00029195	-1.83643	-2.14648	-0.963156	BGIOSGA029665	XP_015696875.1 homeobox-leucine zipper protein HOX4 isoform X1
TCONS_00023383	-1.83629	0.361042	-1.41458	BGIOSGA005574	XP_002461715.1zinc transporter 8
TCONS_00020595	-1.83583	-1.38141	0.0515401	BGIOSGA001230	XP_022684423.1putative UPF0496 protein 2
TCONS_00037570	-1.83583	#NA	-1.94846	BGIOSGA032529	NP_001105702.2pathogenesis related protein-5 precursor
TCONS_00021011	-1.8354	#NA	-0.364559	BGIOSGA015157	XP_015693957.1PREDICTED: uncharacterized protein LOC102714932
TCONS_00012675	-1.83499	-0.0603702	-1.95056	#N/A	#N/A
TCONS_00036140	-1.83425	0.0391768	-0.121565	BGIOSGA005643	XP_008776553.1 putative receptor protein kinase ZmPK1
TCONS_00028274	-1.83115	-1.17471	-0.871407	BGIOSGA035483	XP_006659572.1 probable lipoxygenase 8, chloroplastic
TCONS_00024111	-1.83075	-0.386832	-0.376293	#N/A	#N/A
TCONS_00025734	-1.83045	#NA	#NA	BGIOSGA032466	XP_019057696.1 sigma factor binding protein 2, chloroplastic-like
TCONS_00007292	-1.8293	-2.3884	-1.96492	BGIOSGA031097	XP_004951995.1cyclin-dependent protein kinase inhibitor SMR6
TCONS_00029446	-1.8293	#NA	#NA	BGIOSGA031191	XP_006660936.1 auxin-responsive protein SAUR36-like
TCONS_00036916	-1.82903	-1.38869	#NA	#N/A	#N/A
TCONS_00020822	-1.82752	-2.39034	0.61554	BGIOSGA003983	XP_010231417.1uncharacterized protein LOC100826023

transcriptome

TCONS_00027293	-1.82752	-0.302878	-1.38446	BGIOSGA027709	XP_006659091.1 protein LOL4
TCONS_00034222	-1.82706	-0.583499	-0.648664	BGIOSGA034025	XP_004978454.1NDR1/HIN1-like protein 13
TCONS_00004050	-1.82667	-3.01356	-2.75716	BGIOSGA020422	XP_006646136.2 cinnamoyl-CoA reductase 1
TCONS_00009773	-1.82641	#NA	#NA	BGIOSGA005056	XP_015692950.1PREDICTED: uncharacterized protein LOC107304242
TCONS_00014820	-1.82641	-0.39159	-0.97226	BGIOSGA016992	XP_015692056.1 glycine-rich RNA-binding protein 4, mitochondrial-like
TCONS_00033513	-1.82641	0.193372	0.0277401	BGIOSGA034831	XP_006662945.1 HVA22-like protein e
TCONS_00032777	-1.8262	-1.0699	-0.165438	BGIOSGA031334	XP_002464873.1protein HAIKU1
TCONS_00012251	-1.8235	1.60507	0.342283	BGIOSGA007536	XP_015690946.1PREDICTED: uncharacterized protein LOC102707566 isoform X2
TCONS_00033291	-1.82319	#NA	-2.98042	BGIOSGA037771	XP_006659958.1 endo-1,3;1,4-beta-D-glucanase-like
TCONS_00002375	-1.82289	-2.39564	-1.56613	BGIOSGA008548	XP_006645194.1 BAG family molecular chaperone regulator 2-like
TCONS_00008390	-1.82286	-0.370944	-0.722901	BGIOSGA006031	XP_021318470.1H/ACA ribonucleoprotein complex subunit 3-like protein
TCONS_00028287	-1.82275	#NA	-1.98154	BGIOSGA026072	XP_015695816.1PREDICTED: uncharacterized protein LOC102710806
TCONS_00002545	-1.8227	0.57529	-0.476376	BGIOSGA033654	XP_004983450.1uncharacterized protein LOC101760534
TCONS_00032511	-1.82261	1.18898	1.01811	BGIOSGA013451	NP_001169110.1putative acyl-CoA N-acyltransferases (NAT) family protein
TCONS_00036482	-1.82233	-1.81134	0.824764	BGIOSGA029167	XP_010645203.2 serine/threonine-protein phosphatase 7 long form homolog
TCONS_00007283	-1.82156	0.187727	-0.399584	BGIOSGA037903	XP_004952023.1dnaJ homolog subfamily B member 1
TCONS_00008017	-1.82132	-2.98249	-3.57012	BGIOSGA019925	XP_003566211.1postacrosomal sheath WW domain-binding protein
TCONS_00000653	-1.82075	-0.398219	0.0134079	BGIOSGA021794	XP_004967713.1hydroxyproline O-arabinosyltransferase 3
TCONS_00022077	-1.81797	-1.81672	-1.99361	BGIOSGA021403	XP_006656889.1PREDICTED: uncharacterized protein LOC102710884
TCONS_00012378	-1.8177	-3.0541	-2.2838	BGIOSGA010607	XP_006651456.1 metacaspase-1-like
TCONS_00032772	-1.81757	-1.91676	-0.272153	BGIOSGA023234	XP_020402992.1uncharacterized LOC100272480 isoform X1
TCONS_00008032	-1.81738	-1.40242	#NA	BGIOSGA006444	XP_015698481.1 ankyrin repeat and protein kinase domain-containing protein 1-like
TCONS_00016494	-1.81715	-1.20585	-2.24864	BGIOSGA007389	XP_006653709.2 glutamate receptor 3.1
TCONS_00009593	-1.81679	-1.8182	-2.99656	BGIOSGA029772	XP_006649654.1 peroxygenase-like
TCONS_00029537	-1.81667	-1.04196	-0.908836	BGIOSGA016352	XP_008784000.1 protein LITTLE ZIPPER 3
TCONS_00017192	-1.81618	-0.140907	0.203535	#N/A	#N/A
TCONS_00006839	-1.81613	0.296437	#NA	BGIOSGA014803	NP_001131488.1uncharacterized protein LOC100192825
TCONS_00000512	-1.81585	-3.98931	-1.58387	BGIOSGA003086	XP_003565541.1transcription initiation factor TFIID subunit 4
TCONS_00006342	-1.81454	-2.08409	-0.478635	BGIOSGA028630	XP_004984288.1probable mediator of RNA polymerase II transcription subunit 26b isoform X2
TCONS_00011659	-1.81406	-2.08469	0.950816	BGIOSGA032863	XP_015689783.1 AP2-like ethylene-responsive transcription factor ANT
TCONS_00006057	-1.81401	-3.6868	-2.59913	BGIOSGA034465	XP_012703198.15'-3' exoribonuclease 4 isoform X2
TCONS_00026934	-1.81364	-2.25514	-1.4739	BGIOSGA023713	XP_015695765.1 cyclin-dependent kinase B2-1
TCONS_00034250	-1.81299	-1.40797	-1.00604	BGIOSGA034542	XP_006663799.1 dual specificity protein phosphatase 12-like
TCONS_00017428	-1.81297	-0.967422	1.31583	BGIOSGA011074	XP_006655141.1 probable LRR receptor-like serine/threonine-protein kinase At1g56130
TCONS_00028177	-1.81246	#NA	-0.891887	BGIOSGA036039	XP_006659498.1 patatin-like protein 2
TCONS_00012376	-1.81224	-3.40891	-1.13343	BGIOSGA010611	XP_006651456.1 metacaspase-1-like
TCONS_00022811	-1.81128	0.431185	-1.89479	BGIOSGA035672	XP_015693690.1 cysteine-rich receptor-like protein kinase 10
TCONS_00003595	-1.81096	-0.554913	-0.848798	BGIOSGA021325	XP_003566286.1premnaspirodiene oxygenase
TCONS_00026787	-1.81016	-1.96407	-2.15056	BGIOSGA030925	XP_006660211.2PREDICTED: uncharacterized protein LOC102722325
TCONS_00032880	-1.8101	0.647291	-1.27623	BGIOSGA006948	XP_021317511.1protein SCARECROW
TCONS_00017044	-1.81007	-3.73357	-0.3352	BGIOSGA022934	XP_015693238.1 serine/threonine-protein kinase CDL1-like isoform X1
TCONS_00024515	-1.81002	0.0564496	-1.67234	BGIOSGA024500	XP_015694515.1 L-type lectin-domain containing receptor kinase IV.1-like
TCONS_00037521	-1.80942	-1.74108	-3.44784	BGIOSGA021700	XP_006664738.1 homeobox-leucine zipper protein HOX33
TCONS_00026310	-1.80349	-2.89641	-2.64298	BGIOSGA021700	XP_015695446.1 homeobox-leucine zipper protein ROC6-like
TCONS_00037149	-1.80102	-2.48001	-1.34648	BGIOSGA000526	XP_006664012.1 serine/threonine-protein kinase AFC2-like
TCONS_00021465	-1.79843	-1.49677	-0.870706	BGIOSGA019163	XP_009385740.1 phosphoglycerate mutase-like protein AT74H isoform X2
TCONS_00010697	-1.79571	0.0962534	-1.58129	BGIOSGA007264	XP_006651685.1 serine/threonine-protein kinase BLUS1
TCONS_00014832	-1.79383	-1.59892	-1.14106	BGIOSGA012079	XP_002447005.2probable purine permease 11
TCONS_00000353	-1.79256	0.29926	-0.60634	BGIOSGA006340	XP_010239875.160S acidic ribosomal protein P2A
TCONS_00011694	-1.79185	-0.0994926	-1.57321	BGIOSGA021886	XP_006651114.1 transport inhibitor response 1-like protein
TCONS_00032627	-1.78462	-1.5272	-0.844459	BGIOSGA031483	XP_004983795.1putative pentatricopeptide repeat-containing protein At3g25060, mitochondrial
TCONS_00007639	-1.78345	-0.386428	-2.2112	BGIOSGA007810	XP_014756587.1receptor-like protein kinase HSL1 isoform X1
TCONS_00016181	-1.78173	-0.0678718	-1.17466	BGIOSGA023893	XP_006652475.1 cytochrome c oxidase subunit 6b-2-like
TCONS_00021348	-1.77839	-1.25959	-0.305362	BGIOSGA022659	XP_015693721.1 protein NRT1/ PTR FAMILY 8.3-like

TCONS_00002286	-1.77161	-2.68782	-2.39508	BGIOSGA006290	XP_006645124.1 bidirectional sugar transporter SWEET1a
TCONS_00031642	-1.77008	0.603121	0.079244	BGIOSGA022192	XP_015697086.1 junctophilin-1
TCONS_00029363	-1.7696	-0.633818	-0.0546634	BGIOSGA031113	XP_006661453.1 flap endonuclease GEN-like 1
TCONS_00010243	-1.76932	-0.274271	-0.335458	BGIOSGA012806	XP_004984106.1trimethylguanosine synthase
TCONS_00020393	-1.76928	-1.99374	-2.05203	BGIOSGA020835	XP_015693845.1 vacuolar amino acid transporter 1-like isoform X2
TCONS_00025756	-1.76894	-0.817697	-1.36391	BGIOSGA023035	XP_006659001.1 protein trichome birefringence-like 28
TCONS_00021239	-1.76818	-1.21122	-0.680428	#N/A	#N/A
TCONS_00011380	-1.76752	-0.904058	-0.155023	BGIOSGA013993	XP_015691021.1 activating signal cointegrator 1 isoform X3
TCONS_00020013	-1.76661	-4.87156	-2.15173	BGIOSGA022338	XP_002437727.1uncharacterized protein LOC8066922 isoform X1
TCONS_00025396	-1.76493	-0.00745045	-0.442261	BGIOSGA024500	NP_001146046.1putative lectin-like receptor protein kinase family protein precursor
TCONS_00011780	-1.76481	-3.09511	-3.54028	BGIOSGA032693	XP_006649616.1 remorin-like isoform X1
TCONS_00016487	-1.76421	-3.55855	-3.38263	BGIOSGA019252	XP_015691978.1 probable L-type lectin-domain containing receptor kinase S.5
TCONS_00026116	-1.76381	-2.91651	-2.49886	BGIOSGA020032	XP_015695524.1 zinc transporter 4-like
TCONS_00000554	-1.76346	1.1578	-0.983916	BGIOSGA014906	XP_003566323.1probable WRKY transcription factor 31
TCONS_00020877	-1.76339	1.04246	-1.5773	BGIOSGA036861	XP_006656162.1 receptor-like protein kinase 5
TCONS_00011514	-1.76143	0.482146	-0.851781	BGIOSGA031229	XP_015690517.1 NAC domain-containing protein 67 isoform X1
TCONS_00025565	-1.75901	-2.46357	-1.55595	BGIOSGA023869	XP_015695359.1 transcription factor bHLH47-like
TCONS_00019162	-1.75873	0.164913	0.188705	BGIOSGA018191	XP_010231493.1nucleolar protein 16
TCONS_00008599	-1.75535	-1.38775	-1.60975	BGIOSGA020522	XP_021314244.1probable protein phosphatase 2C 25
TCONS_00030093	-1.75277	-2.02315	-1.06723	BGIOSGA001501	XP_006661262.2 beta-1,3-galactosyltransferase 6-like
TCONS_00034921	-1.75272	-1.47068	-0.789838	BGIOSGA025925	XP_006663471.1 bidirectional sugar transporter SWEET14-like
TCONS_00014838	-1.75224	-0.0238148	-0.00441592	BGIOSGA000456	XP_006646494.1PREDICTED: uncharacterized protein LOC102720109, partial
TCONS_00004095	-1.75224	-0.175818	-2.94302	BGIOSGA030346	XP_006664979.1 probable WRKY transcription factor 41
TCONS_00030891	-1.75142	-4.41717	-1.87795	BGIOSGA011225	XP_006661687.2 ubiquitin carboxyl-terminal hydrolase 8-like isoform X2
TCONS_00015817	-1.75125	-4.07133	-2.17041	BGIOSGA030609	XP_004975477.1protein PYRICULARIA ORYZAE RESISTANCE 21
TCONS_00014926	-1.75084	-0.833943	-0.523723	BGIOSGA014776	XP_006450366.140S ribosomal protein S11
TCONS_00005934	-1.74914	-3.68184	-3.8694	BGIOSGA022733	XP_006662214.1PREDICTED: uncharacterized protein LOC102717831
TCONS_00033891	-1.74861	-1.18447	-2.22982	BGIOSGA031216	XP_022684006.1uncharacterized protein LOC101781025
TCONS_00022989	-1.74385	-1.34796	-1.51484	BGIOSGA025021	XP_015695064.1 protein LONGIFOLIA 2
TCONS_00029217	-1.74332	-2.65043	-1.43582	BGIOSGA023697	XP_004957110.1dof zinc finger protein MNB1A
TCONS_00025303	-1.74299	-0.982078	-0.653196	BGIOSGA015857	XP_015694792.1 receptor-like serine/threonine-protein kinase SD1-8 isoform X3
TCONS_00011556	-1.74108	-0.705071	-2.27448	BGIOSGA011852	XP_021311624.1uncharacterized protein LOC8059153 isoform X2
TCONS_00020317	-1.73867	-3.4339	-1.10636	BGIOSGA003394	XP_021319277.1PLAT domain-containing protein 3
TCONS_00021113	-1.73689	#NA	-1.08925	BGIOSGA021325	XP_004965771.2premnaspirodiene oxygenase
TCONS_00010008	-1.73666	-2.07386	-0.937796	BGIOSGA031595	XP_010228674.2uncharacterized protein LOC100840589
TCONS_00011879	-1.7354	-0.326526	0.126177	BGIOSGA005145	XP_004985039.1actin-depolymerizing factor 5
TCONS_00032888	-1.73294	-1.43622	-3.15052	BGIOSGA037382	XP_006662692.1 NAC transcription factor 29-like
TCONS_00030806	-1.72938	-1.13779	-1.6289	BGIOSGA001471	XP_015691994.1 disease resistance protein RGA2-like
TCONS_00029134	-1.72787	-0.217376	-2.89292	#N/A	#N/A
TCONS_00024516	-1.7273	-1.46271	-2.57694	BGIOSGA024874	XP_015694515.1 L-type lectin-domain containing receptor kinase IV.1-like
TCONS_00017064	-1.72598	-1.98944	-0.645956	BGIOSGA016405	XP_015692468.1 cysteine-rich receptor-like protein kinase 2
TCONS_00011538	-1.72027	-1.97302	-1.05959	BGIOSGA022659	XP_006649373.1 protein NRT1/ PTR FAMILY 5.2-like
TCONS_00036246	-1.71995	-1.08465	-1.48249	BGIOSGA035558	XP_015698182.1 putative disease resistance RPP13-like protein 3
TCONS_00017067	-1.71969	-2.3151	-2.91341	BGIOSGA017063	NP_001348186.1WRKY transcription factor 8
TCONS_00024359	-1.71773	-1.51021	-1.3333	BGIOSGA036898	XP_006658139.2 cytochrome P450 714B1-like
TCONS_00003429	-1.71734	-1.93471	-2.13006	BGIOSGA007547	XP_004967515.1protein SENESCENCE-ASSOCIATED GENE 21, mitochondrial
TCONS_00016675	-1.7163	-1.512	-2.33682	BGIOSGA020130	XP_004976940.1receptor-like serine/threonine-protein kinase SD1-8
TCONS_00008076	-1.7134	-2.83711	-3.43798	BGIOSGA006374	XP_021319885.1uncharacterized protein LOC8057879 isoform X7
TCONS_00014163	-1.70827	-0.922237	-0.78579	BGIOSGA008207	XP_006652234.1 40S ribosomal protein S27-like
TCONS_00011126	-1.7056	-3.37388	-2.97291	BGIOSGA016052	XP_006651904.1PREDICTED: uncharacterized protein LOC102719373
TCONS_00012082	-1.70353	-1.94168	-0.542842	BGIOSGA010927	XP_006649923.1 serine/threonine-protein kinase STY46-like
TCONS_00015143	-1.70318	-1.92534	-1.2275	BGIOSGA035881	XP_015691693.1PREDICTED: uncharacterized protein LOC102706745 isoform X1
TCONS_00021652	-1.70269	-3.5277	-2.71485	BGIOSGA032356	XP_006655814.1 disease resistance protein RPM1-like
TCONS_00004561	-1.70235	-2.66489	-1.87848	#N/A	#N/A
TCONS_00003636	-1.69913	-0.853008	-1.43612	BGIOSGA001572	XP_006644203.1 mediator of RNA polymerase II transcription subunit 9
TCONS_00037983	-1.69861	-2.85361	-1.85247	BGIOSGA024656	XP_015698019.1 putative disease resistance RPP13-like protein 3

TCONS_00017277	-1.69857	-1.0974	-1.50461	BGIOSGA001680	XP_024315760.1ABC transporter C family member 8 isoform X1
TCONS_00022922	-1.69807	-0.992227	-1.58962	BGIOSGA024572	XP_015693848.1 homeobox-leucine zipper protein HOX29
TCONS_00034408	-1.69766	-1.97716	-2.22256	BGIOSGA014021	XP_002450415.2ABC transporter G family member 25
TCONS_00012050	-1.69681	-0.740776	-0.446662	BGIOSGA002365	XP_015692414.1 serine/arginine-rich splicing factor RS2Z33 isoform X2
TCONS_00012382	-1.69528	-1.58188	-0.155722	BGIOSGA010604	XP_015690943.1 transcription factor bHLH144-like
TCONS_00004964	-1.69265	1.72412	-3.45251	BGIOSGA021694	XP_002458981.1keratin, type I cytoskeletal 9
TCONS_00030513	-1.69197	-1.84274	-2.44258	BGIOSGA015169	XP_002462810.1phytoene synthase 2, chloroplastic isoform X1
TCONS_00012463	-1.69108	-2.23089	-0.793954	#N/A	#N/A
TCONS_00024982	-1.69089	-2.2781	0.12805	BGIOSGA018546	XP_006657628.1 protein TsetseEP-like
TCONS_00016696	-1.69059	0.440856	0.741299	BGIOSGA014267	XP_006653804.1 regulation of nuclear pre-mRNA domain-containing protein 1A-like isoform X2
TCONS_00027912	-1.6902	0.00792328	-1.12218	BGIOSGA002978	XP_006659319.1 phosphoenolpyruvate carboxylase 2-like
TCONS_00030312	-1.69012	-2.45154	-1.18823	BGIOSGA004422	NP_001151910.1BHLH transcription factor
TCONS_00010303	-1.68876	-1.86576	0.122722	BGIOSGA010533	XP_006650196.1 PI-PLC X domain-containing protein At5g67130
TCONS_00023622	-1.6887	-1.12887	-1.87742	BGIOSGA030346	XP_015694621.1 probable WRKY transcription factor 41
TCONS_00021019	-1.68829	-2.86635	-3.46341	BGIOSGA017810	XP_006656238.1 protein PHR1-LIKE 1
TCONS_00024705	-1.68826	0.133614	0.121488	BGIOSGA011250	XP_015694548.1 probable prolyl 4-hydroxylase 7
TCONS_00017225	-1.68772	-1.94953	-0.879852	BGIOSGA018721	XP_002443222.1F-box protein At5g07610
TCONS_00003072	-1.6877	-1.75162	-1.7279	BGIOSGA001742	XP_003565356.1gibberellin 2-beta-dioxygenase 1
TCONS_00033361	-1.68742	-1.86745	-0.880611	BGIOSGA031352	XP_003562374.1mitochondrial arginine transporter BAC1
TCONS_00034400	-1.68598	-0.141337	1.37636	BGIOSGA007432	XP_015698126.1 probable alkaline/neutral invertase F
TCONS_00038357	-1.6857	-3.13264	-1.41093	BGIOSGA028420	XP_021307139.1uncharacterized protein LOC8080432 isoform X4
TCONS_00016493	-1.68501	-3.45544	-2.47153	BGIOSGA031276	XP_006653708.1 protein S-acyltransferase 21-like
TCONS_00015698	-1.68455	-4.87105	-1.30273	BGIOSGA012979	XP_004973024.1L-gulonolactone oxidase 2
TCONS_00033736	-1.68342	-1.4033	-0.278599	BGIOSGA029435	XP_010235357.1Holliday junction resolvase MOC1, chloroplastic
TCONS_00036960	-1.67902	-2.68686	-1.50615	BGIOSGA002049	XP_006663942.1 isoflavone reductase homolog
TCONS_00006864	-1.67835	-2.52165	-2.60341	BGIOSGA002528	XP_006647882.1 aquaporin NIP2-1
TCONS_00023983	-1.67634	-0.04043	-1.18644	BGIOSGA036099	XP_006657888.1 polyol transporter 5-like
TCONS_00016951	-1.67544	-2.65189	-2.07032	BGIOSGA002565	XP_002439094.2uncharacterized protein LOC8066586
TCONS_00021176	-1.6743	-0.445993	0.223668	BGIOSGA027014	XP_015693948.1 (R)-mandelonitrile lyase-like
TCONS_00019972	-1.6718	-6.2649	-2.10707	BGIOSGA007305	XP_006655695.1 WAT1-related protein At3g30340-like
TCONS_00005000	-1.67005	-4.26711	-1.28134	BGIOSGA000427	XP_006645261.1 probable protein S-acyltransferase 7 isoform X1
TCONS_00013424	-1.66981	-2.05396	-0.726676	BGIOSGA008446	XP_006650797.1 PHD finger protein ALFIN-LIKE 3
TCONS_00011473	-1.66743	-0.640268	-0.133654	BGIOSGA029195	XP_006649297.1 protein DOWNY MILDEW RESISTANCE 6-like isoform X1
TCONS_00004326	-1.6669	-5.81878	-1.27131	BGIOSGA033727	XP_006644653.1 HVA22-like protein f
TCONS_00000668	-1.66686	0.599448	0.00865216	BGIOSGA003255	XP_002455461.1chaperone protein dnaJ 72
TCONS_00025747	-1.66626	-3.16144	-1.37701	BGIOSGA013871	XP_012699366.1uncharacterized protein LOC101779282
TCONS_00000942	-1.66596	-1.90864	-1.66104	BGIOSGA001631	XP_003565764.1probable glutathione S-transferase GSTF1
TCONS_00004917	-1.66495	-1.10733	-1.04249	BGIOSGA012149	XP_006645188.1 two-component response regulator ORR26
TCONS_00014778	-1.66408	-1.25206	-3.16826	BGIOSGA008805	XP_006647681.1 zinc finger CCCH domain-containing protein 17
TCONS_00015483	-1.66399	0.917748	-2.16849	BGIOSGA029313	XP_008780635.2 S-locus-specific glycoprotein S6-like
TCONS_00001938	-1.66134	-0.461972	-1.00517	BGIOSGA012996	XP_022682523.1pentatricopeptide repeat-containing protein At1g09900
TCONS_00014423	-1.66071	-1.03466	-1.15912	#N/A	#N/A
TCONS_00020009	-1.66037	-1.13981	-2.28052	BGIOSGA031674	NP_001143208.1putative DUF594 domain containing family protein
TCONS_00017644	-1.65925	-0.454704	-0.924952	BGIOSGA036473	XP_003568620.1uncharacterized protein LOC100834310
TCONS_00023292	-1.65685	-3.17346	-2.72201	#N/A	#N/A
TCONS_00035567	-1.65606	#NA	#NA	BGIOSGA019168	XP_021316327.1putative cysteine-rich receptor-like protein kinase 32 isoform X1
TCONS_00012163	-1.65492	-2.69107	-2.25091	BGIOSGA010774	XP_006649998.1 CBL-interacting protein kinase 31
TCONS_00019082	-1.65248	-1.68021	-3.40486	BGIOSGA017998	XP_010231524.1protein NRT1/ PTR FAMILY 5.1 isoform X2
TCONS_00024366	-1.65226	-0.416703	-0.417595	BGIOSGA029518	XP_006658973.2 NAC domain-containing protein 2-like
TCONS_00027163	-1.65188	-1.39001	-0.590049	BGIOSGA015660	XP_006660395.2 ABC transporter B family member 1
TCONS_00036573	-1.6503	-1.57539	-1.87661	BGIOSGA018902	XP_006663720.1PREDICTED: uncharacterized protein LOC102720754
TCONS_00032816	-1.64872	-1.74615	-1.10557	BGIOSGA003827	XP_015697528.1 LOB domain-containing protein 12-like
TCONS_00020854	-1.64669	-0.733417	-0.956384	BGIOSGA014214	XP_004965560.1calmodulin-binding receptor kinase CaMRLK
TCONS_00014441	-1.64512	-1.94594	-1.831	BGIOSGA016601	XP_006653520.1 ribonuclease H2 subunit B
TCONS_00037139	-1.64452	-1.98591	-0.955619	BGIOSGA036743	XP_002455454.2uncharacterized protein LOC8074534

TCONS_00013231	-1.6439	-0.289156	-0.714556	BGIOSGA012156	XP_008644470.1allene oxide synthase 1, chloroplastic
TCONS_00009447	-1.64306	0.379583	1.50555	BGIOSGA011995	XP_015690440.1PREDICTED: uncharacterized protein LOC102713805 isoform X1
TCONS_00003100	-1.6427	#NA	-2.1996	BGIOSGA023035	XP_004968629.1protein trichome birefringence-like 41 isoform X1
TCONS_00019543	-1.64232	-1.39564	-2.49574	BGIOSGA035284	XP_003566104.1uncharacterized protein LOC100828480
TCONS_00018382	-1.64079	-1.1913	-0.439882	#N/A	#N/A
TCONS_00034500	-1.63959	-0.657449	-1.6454	BGIOSGA015853	XP_006662814.1 G-type lectin S-receptor-like serine/threonine-protein kinase At2g19130
TCONS_00008803	-1.63937	-2.29383	-2.72961	#N/A	#N/A
TCONS_00004324	-1.63884	-1.84042	-1.78856	BGIOSGA005493	XP_002458431.2subtilisin-like protease SBT5.6
TCONS_00036265	-1.63772	-2.12001	-0.968239	BGIOSGA007251	XP_005643450.1heat shock protein 70
TCONS_00020587	-1.63702	-3.46256	-1.80702	BGIOSGA030919	XP_006658036.1 probable carboxylesterase 18
TCONS_00007031	-1.63627	-2.32449	-2.56927	BGIOSGA009213	XP_004954237.1E3 ubiquitin-protein ligase RNF6
TCONS_00025826	-1.63505	-0.634057	0.289043	BGIOSGA028630	XP_015695479.1 AT-hook motif nuclear-localized protein 1-like
TCONS_00019708	-1.63389	-0.479105	-0.516787	BGIOSGA013739	XP_015692809.1 protein REVERSION-TO-ETHYLENE SENSITIVITY1-like
TCONS_00025217	-1.63284	-3.90364	-3.49503	BGIOSGA032176	XP_015697295.1 proline-rich protein 4-like
TCONS_00015072	-1.632	-0.719721	-4.12646	BGIOSGA010303	XP_004977053.1probable isoaspartyl peptidase/L-asparaginase 2
TCONS_00014654	-1.63174	-3.20036	-4.38105	BGIOSGA008697	XP_024312165.1(+)-neomenthol dehydrogenase-like isoform X2
TCONS_00006687	-1.62964	-2.62604	-0.405953	BGIOSGA027422	NP_001144338.1uncharacterized protein LOC100277236
TCONS_00023195	-1.62904	-1.61948	0.378336	BGIOSGA025237	XP_006658324.1 non-specific lipid-transfer protein-like protein At2g13820
TCONS_00035685	-1.62892	-3.20459	-0.791901	BGIOSGA036535	XP_006663892.1PREDICTED: uncharacterized protein LOC102701400
TCONS_00024026	-1.62822	-3.7904	-1.0567	BGIOSGA002451	XP_006657907.1 zinc finger protein 4-like
TCONS_00008720	-1.62758	#NA	#NA	#N/A	#N/A
TCONS_00011363	-1.62757	-2.22281	-1.90046	BGIOSGA000168	XP_004980988.1S-norcoclaurine synthase 1
TCONS_00025745	-1.62728	-1.59192	-1.7066	BGIOSGA012455	XP_004958775.1BRCT domain-containing protein At4g02110
TCONS_00009812	-1.62606	-0.120644	-0.214144	BGIOSGA012366	XP_015690566.1 elongator complex protein 6 isoform X2
TCONS_00002541	-1.62553	-1.62381	#NA	BGIOSGA023451	XP_004971294.1uncharacterized protein LOC101785677
TCONS_00013943	-1.62519	#NA	-2.80131	BGIOSGA015402	XP_006653261.2 wall-associated receptor kinase 5-like
TCONS_00021337	-1.62465	-2.43721	-1.76882	BGIOSGA030474	XP_015693783.1 protein PHR1-LIKE 1
TCONS_00003090	-1.62455	-3.21001	#NA	BGIOSGA012812	XP_015698331.1 RING-H2 finger protein ATL39-like
TCONS_00028581	-1.62455	-0.673955	-1.03737	BGIOSGA030326	XP_010237337.1PRA1 family protein H isoform X2
TCONS_00012621	-1.6242	-2.21045	-0.21881	BGIOSGA013021	XP_006651573.1 endoribonuclease Dicer homolog 2a
TCONS_00013816	-1.62409	-1.21058	-1.80405	#N/A	#N/A
TCONS_00022787	-1.62368	-1.62615	-1.22012	BGIOSGA031637	XP_010235843.1polyubiquitin 11 isoform X1
TCONS_00017070	-1.62325	-4.21165	-1.48422	BGIOSGA034428	XP_003580557.2trinucleotide repeat-containing gene 18 protein
TCONS_00031020	-1.62308	-3.21187	-3.39155	BGIOSGA032832	XP_008652710.1ribosome-inactivating protein 3-like
TCONS_00019744	-1.62299	-0.890053	-0.221843	BGIOSGA004235	XP_008656010.1serine/arginine repetitive matrix protein 1
TCONS_00026494	-1.62251	#NA	#NA	BGIOSGA039995	XP_006643674.1 glycerophosphodiester phosphodiesterase protein kinase domain-containing GDPDL2-like
TCONS_00016159	-1.62206	-3.62955	-2.01872	BGIOSGA025671	XP_006652458.1 transcription factor bHLH113-like
TCONS_00025409	-1.62165	-2.76622	-3.09967	BGIOSGA031300	XP_006658737.1 protein BZR1 homolog 1
TCONS_00000804	-1.62055	-1.3406	-1.39786	BGIOSGA011559	XP_006644127.1 peroxidase 1-like
TCONS_00021314	-1.6204	-2.95254	0.0312151	BGIOSGA029505	XP_015693402.1 histone H3.v1-like
TCONS_00032695	-1.62027	-1.21542	-2.81359	BGIOSGA000168	XP_006662054.1 S-norcoclaurine synthase 1-like isoform X2
TCONS_00014070	-1.61968	-2.63094	-2.33122	BGIOSGA033672	XP_006657084.1 probable LRR receptor-like serine/threonine-protein kinase At3g47570
TCONS_00009491	-1.61958	-1.47932	-2.40025	BGIOSGA012042	XP_006649555.1 aminotransferase ALD1 homolog
TCONS_00018108	-1.61943	-0.409126	-0.815675	BGIOSGA037764	XP_015692744.1 F-box protein At5g46170-like
TCONS_00011534	-1.61913	-2.63189	-1.49448	BGIOSGA011490	XP_006649364.1 putative magnesium transporter MRS2-H isoform X1
TCONS_00009139	-1.61899	0.527132	-0.890755	BGIOSGA018776	NP_001335008.1tubulin beta-1 chain
TCONS_00004236	-1.61883	-1.63227	-0.357726	BGIOSGA016039	XP_006644564.2 UDP-glycosyltransferase 88B1-like
TCONS_00004097	-1.61874	-0.172955	-0.569458	#N/A	XP_015698979.1 myosin heavy chain, cardiac muscle isoform-like
TCONS_00008575	-1.61752	-1.75945	-1.23543	BGIOSGA033262	XP_015688694.1 probable methyltransferase PMT15
TCONS_00023853	-1.61736	-2.80404	-0.498862	BGIOSGA002624	XP_006657797.1 putative receptor-like protein kinase At4g00960
TCONS_00011395	-1.61703	-2.63453	-1.33617	BGIOSGA011750	XP_010229355.1U-box domain-containing protein 52 isoform X4
TCONS_00005383	-1.61699	-0.820989	-0.843715	BGIOSGA007493	XP_003570912.2putative vacuolar cation/proton exchanger 4 isoform X1
TCONS_00007152	-1.616	-6.05583	-6.38917	BGIOSGA009337	XP_015689158.1PREDICTED: uncharacterized protein LOC102703286
TCONS_00007720	-1.61305	-1.5574	-1.40687	BGIOSGA023032	XP_015688969.1PREDICTED: uncharacterized protein LOC107303578
TCONS_00023306	-1.6125	-2.48317	-1.03469	BGIOSGA013403	XP_006657538.1 UDP-glycosyltransferase 91C1-like
TCONS_00014168	-1.61243	0.00276781	-1.52075	BGIOSGA003427	XP_015692268.1 potassium transporter 1

TCONS_00013478	-1.61018	-4.64501	-2.06224	BGIOSGA027391	XP_003559210.4pectinesterase inhibitor 3
TCONS_00027188	-1.60867	-3.02975	-0.772061	BGIOSGA034464	XP_006659018.1 protein YLS9-like
TCONS_00028811	-1.60843	-0.789176	-0.777163	BGIOSGA030560	XP_004956626.1E3 ubiquitin-protein ligase RDUF1
TCONS_00030441	-1.60842	-2.52107	-0.0106451	BGIOSGA012372	XP_002444620.1probable L-ascorbate peroxidase 4
TCONS_00003311	-1.6081	-1.12882	-2.72384	BGIOSGA001904	XP_006645762.1PREDICTED: uncharacterized protein LOC102704022 isoform X2
TCONS_00014676	-1.60769	-0.647744	-0.319784	BGIOSGA033437	XP_006652591.1 B-box zinc finger protein 20-like
TCONS_00037231	-1.60698	-1.11958	-2.65462	BGIOSGA036142	XP_006664046.1 ureide permease 1-like isoform X1
TCONS_00013209	-1.60607	-2.4859	-1.03591	BGIOSGA025118	XP_015690295.1PREDICTED: uncharacterized protein LOC102705329
TCONS_00011241	-1.60215	-1.54033	-0.0934838	BGIOSGA013841	XP_006650812.1 ribosomal RNA large subunit methyltransferase E
TCONS_00028059	-1.59966	-0.31474	0.689594	BGIOSGA026929	XP_006660155.1PREDICTED: uncharacterized protein LOC102704443
TCONS_00014363	-1.59964	-5.65822	-2.67358	BGIOSGA015557	XP_003579877.1sugar transport protein MST1 isoform X1
TCONS_00015437	-1.59679	-2.21777	-0.770638	BGIOSGA016077	XP_002448612.1malonyl-CoA:anthocyanidin 5-O-glucoside-6''-O-malonyltransferase
TCONS_00030253	-1.59623	-3.71336	-3.31551	BGIOSGA028958	XP_006660764.1PREDICTED: uncharacterized protein LOC102709200
TCONS_00035737	-1.59608	-1.66408	-0.769945	BGIOSGA029398	XP_003576960.1B-box zinc finger protein 23
TCONS_00028317	-1.5957	-0.556772	-1.50467	BGIOSGA033119	XP_006659605.2 methyltransferase-like protein 13
TCONS_00011899	-1.59517	0.542092	0.122041	BGIOSGA011116	XP_015689830.1PREDICTED: uncharacterized protein LOC107303746
TCONS_00025460	-1.59458	-1.78057	-1.26643	BGIOSGA032724	XP_006657912.1 probable protein S-acyltransferase 22 isoform X2
TCONS_00016246	-1.59452	-2.36562	-4.96703	BGIOSGA014729	XP_006652508.1 BRCA1-associated RING domain protein 1-like
TCONS_00006801	-1.59337	-1.40446	0.000871031	BGIOSGA012235	XP_004972995.1ACT domain-containing protein ACR8
TCONS_00006038	-1.59277	-1.46361	-0.825869	BGIOSGA008195	XP_021315851.1putative F-box protein At2g02030 isoform X2
TCONS_00026123	-1.59248	-2.99056	0.168558	BGIOSGA014346	XP_006659921.1 probable LRR receptor-like serine/threonine-protein kinase At1g56130
TCONS_00000977	-1.59176	-0.874071	-0.41624	BGIOSGA013850	XP_003618098.2LRR receptor-like kinase
TCONS_00024502	-1.59075	-2.16938	-1.26094	BGIOSGA013064	XP_008650812.1uncharacterized LOC100191793 isoform X4
TCONS_00022297	-1.589	-3.09997	-1.23038	BGIOSGA030609	XP_003572869.1basic proline-rich protein
TCONS_00015808	-1.58828	-3.84488	-2.0108	BGIOSGA006381	XP_021319043.1trihelix transcription factor ASR3 isoform X1
TCONS_00015951	-1.58798	-1.49373	-1.22825	BGIOSGA017039	XP_003579814.1protein NRT1/ PTR FAMILY 4.3
TCONS_00013245	-1.58777	-2.67455	-1.27606	BGIOSGA005489	XP_018674127.1 L-type lectin-domain containing receptor kinase IV.2-like
TCONS_00014199	-1.58518	-1.95867	-1.4577	BGIOSGA033295	XP_006652261.2PREDICTED: uncharacterized protein LOC102701162
TCONS_00011209	-1.58504	-0.0762754	-0.717659	BGIOSGA013803	XP_015691121.1 E3 ubiquitin-protein ligase RNF126-A-like
TCONS_00026952	-1.58497	-0.908311	-0.458222	BGIOSGA029006	XP_015695666.1 oxysterol-binding protein-related protein 4C-like
TCONS_00029137	-1.58416	-1.44538	-2.27965	BGIOSGA004263	XP_002460315.1aspartic proteinase nepenthesin-1
TCONS_00013206	-1.5835	0.64014	-1.08108	BGIOSGA023706	XP_015690361.1 probable WRKY transcription factor 57
TCONS_00005694	-1.58312	-1.24797	-0.718141	BGIOSGA013173	XP_006648469.1 far upstream element-binding protein 2-like
TCONS_00031184	-1.58302	-3.76874	-1.46305	BGIOSGA024106	XP_002467196.2cation/calcium exchanger 1
TCONS_00029067	-1.58272	-2.69246	-0.245798	BGIOSGA029303	XP_010504143.1 histone H4
TCONS_00029275	-1.58109	-3.17027	-1.9172	BGIOSGA031963	XP_004957239.1uncharacterized protein LOC101783301
TCONS_00008747	-1.58009	-1.24735	0.629834	BGIOSGA038644	XP_004953799.1aldehyde dehydrogenase family 2 member B7, mitochondrial
TCONS_00028284	-1.57877	-0.320461	-0.997607	BGIOSGA000169	XP_002444768.1nucleolar and coiled-body phosphoprotein 1
TCONS_00010870	-1.57847	-1.49551	0.076273	BGIOSGA033465	XP_014661241.1transcription factor bHLH18
TCONS_00028497	-1.57733	-2.91197	-1.2891	BGIOSGA024529	XP_023157675.1uncharacterized LOC100279181 isoform X1
TCONS_00008079	-1.57672	0.0258539	-2.51302	BGIOSGA003122	XP_006647254.1 protein indeterminate-domain 2-like
TCONS_00023847	-1.57642	-1.03864	-0.928782	BGIOSGA024169	XP_006658646.1 putative receptor-like protein kinase At4g00960
TCONS_00022917	-1.57626	-1.09115	-1.66559	BGIOSGA001321	XP_006656496.1 leucine-rich repeat receptor-like kinase protein FLORAL ORGAN NUMBER1
TCONS_00009641	-1.57624	-3.4983	-0.291803	BGIOSGA024901	XP_006651195.2PREDICTED: uncharacterized protein LOC102712793
TCONS_00020048	-1.57499	-0.670801	0.184686	BGIOSGA034929	XP_004964410.1short-chain dehydrogenase TIC 32, chloroplastic
TCONS_00017495	-1.57268	-0.250245	-0.388923	BGIOSGA013374	XP_015692533.1 ankycorbin-like
TCONS_00004398	-1.56908	0.195037	-1.08034	BGIOSGA005308	XP_006646326.1 proline-rich receptor-like protein kinase PERK2
TCONS_00034489	-1.56648	-0.437962	-0.817681	#N/A	#N/A
TCONS_00001956	-1.56282	-0.503898	-1.15361	BGIOSGA029702	XP_009397166.1 pectinesterase-like
TCONS_00030243	-1.56275	-5.95937	-2.38443	BGIOSGA018640	XP_015696533.1 fasciclin-like arabinogalactan protein 7 isoform X2
TCONS_00021385	-1.56203	1.21173	-1.763	BGIOSGA010413	XP_015693712.1 aspartic proteinase-like protein 1
TCONS_00017235	-1.56128	-0.545982	0.607389	BGIOSGA035541	XP_024315058.1cyanidin 3-O-rutinoside 5-O-glucosyltransferase-like
TCONS_00022743	-1.56111	-3.7161	-1.71497	BGIOSGA022397	XP_015695001.1 CRIB domain-containing protein RIC4-like isoform X2

TCONS_00029227	-1.56046	-2.71686	-2.30157	BGIOSGA030969	XP_015696834.1 F-box protein At3g07870-like isoform X2
TCONS_00003549	-1.56009	-3.29916	-2.63775	BGIOSGA034526	XP_006644168.1 acyltransferase-like protein At1g54570, chloroplastic
TCONS_00019453	-1.55891	-0.911354	#NA	BGIOSGA030243	XP_004985170.1beta-hexosaminidase 2
TCONS_00011597	-1.5568	-3.72128	-2.50348	BGIOSGA029496	XP_004985638.1inactive protein RESTRICTED TEV MOVEMENT 2
TCONS_00007118	-1.55671	0.600535	0.273887	BGIOSGA037209	XP_015688766.1 thioredoxin-like 3-2, chloroplastic
TCONS_00007965	-1.55625	-0.512501	-0.788653	BGIOSGA017358	XP_004952430.1leucine-rich repeat extensin-like protein 3
TCONS_00035092	-1.55434	-0.402423	#NA	BGIOSGA002712	XP_008679586.1neurogenic locus notch homolog protein 3
TCONS_00031251	-1.55273	-0.404463	-1.73609	BGIOSGA024748	XP_002467143.1NADPH-dependent aldehyde reductase-like protein, chloroplastic
TCONS_00037053	-1.55244	#NA	-1.32179	BGIOSGA015845	XP_004977747.2uncharacterized protein LOC101761830
TCONS_00029409	-1.55097	-1.80262	-1.25506	BGIOSGA024416	XP_006660895.1 thaumatin-like protein 1b isoform X1
TCONS_00017104	-1.55018	-3.14465	-0.626977	BGIOSGA030479	XP_004960433.2transcription factor APG
TCONS_00028195	-1.55016	0.518285	-1.74251	BGIOSGA017197	XP_015692240.1 anthocyanidin reductase-like
TCONS_00022674	-1.55014	-2.72967	-3.32752	BGIOSGA023383	XP_004965704.1putative GDP-L-fucose synthase 2
TCONS_00015910	-1.54941	-3.14563	-1.52198	BGIOSGA028195	XP_014661246.1BTB/POZ and MATH domain-containing protein 1
TCONS_00017808	-1.54927	0.00620145	0.0853592	BGIOSGA019867	XP_012704671.1transcription termination factor MTERF6, chloroplastic/mitochondrial
TCONS_00006511	-1.54641	-1.71248	-1.48932	BGIOSGA002830	XP_006648863.1 serine carboxypeptidase-like 34
TCONS_00037373	-1.54617	-5.73466	-2.33733	BGIOSGA033598	XP_002443374.2putative disease resistance RPP13-like protein 3
TCONS_00032184	-1.54601	-1.41028	-1.75602	BGIOSGA020893	XP_004983151.1cyclic dof factor 1
TCONS_00000484	-1.54424	0.632138	-2.03969	BGIOSGA003055	XP_015698965.1 pentatricopeptide repeat-containing protein At5g25630
TCONS_00011143	-1.54129	-1.35894	-2.58713	BGIOSGA026213	XP_004981407.1uncharacterized protein LOC101759573
TCONS_00011909	-1.54086	-3.91714	0.629031	#N/A	#N/A
TCONS_00011121	-1.54038	0.161881	0.678263	BGIOSGA024736	XP_024313537.15'-3' exoribonuclease 4-like
TCONS_00020040	-1.54018	-1.06267	-1.07451	BGIOSGA017662	XP_006655737.2 oligopeptide transporter 7-like
TCONS_00022628	-1.53937	0.385639	-0.0910696	BGIOSGA020815	XP_015693843.1 extensin
TCONS_00030226	-1.53857	-1.75749	-1.05822	BGIOSGA022085	XP_003578295.1probable E3 ubiquitin-protein ligase XERICO
TCONS_00012793	-1.53753	-0.014198	0.298638	BGIOSGA031369	XP_015690885.1 phosphatidylinositol N-acetylglucosaminyltransferase subunit P-like
TCONS_00020507	-1.53458	-1.20554	-0.730684	BGIOSGA021591	XP_022681429.1protein trichome birefringence-like 19
TCONS_00025706	-1.53373	-2.27309	-1.73737	BGIOSGA023739	XP_021904043.1potassium transporter 2
TCONS_00032788	-1.5335	-2.08478	-1.33389	BGIOSGA031326	XP_004983522.1CSC1-like protein At1g32090
TCONS_00017264	-1.53246	-0.763963	0.341553	BGIOSGA019306	XP_015689118.1 transmembrane protein 234 homolog
TCONS_00037437	-1.53159	-0.554622	0.22149	BGIOSGA000785	XP_006664152.1 serine/threonine-protein kinase SAPK9
TCONS_00018524	-1.52975	-1.4552	-1.02078	BGIOSGA014747	XP_010231785.1floral homeotic protein APETALA 2 isoform X1
TCONS_00005449	-1.52938	0.65366	-0.441692	BGIOSGA037765	XP_022679979.1uncharacterized protein LOC111256358
TCONS_00002894	-1.52894	-4.0897	-1.34544	BGIOSGA002329	XP_006645526.1 DEAD-box ATP-dependent RNA helicase 18
TCONS_00010789	-1.52812	-0.932888	-1.53228	BGIOSGA001814	XP_004982036.1protein FAF-like, chloroplastic isoform X1
TCONS_00015174	-1.52774	-4.78001	-2.2734	BGIOSGA033201	XP_006653032.1 protochlorophyllide reductase A, chloroplastic
TCONS_00010987	-1.52773	-0.867285	-0.383487	BGIOSGA029560	XP_006650616.2 UDP-glycosyltransferase 83A1-like
TCONS_00033892	-1.52713	-0.686207	-0.364804	BGIOSGA034213	XP_004978094.1uncharacterized protein LOC101754822
TCONS_00018450	-1.52695	-2.64519	-1.71567	BGIOSGA018985	XP_015692868.1 abscisic acid 8'-hydroxylase 2-like
TCONS_00014861	-1.52674	-0.290762	-0.677698	BGIOSGA017039	XP_015691560.1 protein NRT1/ PTR FAMILY 8.2-like
TCONS_00009770	-1.52043	-1.90715	-2.60942	BGIOSGA023852	NP_001150070.1catalytic/ hydrolase
TCONS_00007326	-1.52037	-2.77774	-2.36718	BGIOSGA034967	XP_018684826.1 cycloartenol synthase-like isoform X2
TCONS_00036655	-1.52018	-0.92998	-1.36766	BGIOSGA036644	XP_006663191.1 E3 ubiquitin-protein ligase RING1-like
TCONS_00031397	-1.51972	1.71742	-0.9796	BGIOSGA026092	XP_006662477.2 COBRA-like protein 4
TCONS_00007642	-1.51877	-0.424747	-2.03077	BGIOSGA009487	XP_008645622.2receptor-like protein kinase 5
TCONS_00000832	-1.51857	-1.83221	-0.325991	BGIOSGA015083	XP_006645848.1 neutral/alkaline invertase 1, mitochondrial-like
TCONS_00027303	-1.51832	-0.98141	-0.53944	BGIOSGA027700	NP_001169202.1uncharacterized LOC100383055
TCONS_00002196	-1.51736	-1.93747	-1.59321	BGIOSGA004832	XP_015691946.1 DNA excision repair protein ERCC-8-like
TCONS_00009633	-1.51627	-1.32412	-0.422291	BGIOSGA012177	XP_006651192.1 branched-chain-amino-acid aminotransferase 2, chloroplastic-like
TCONS_00009161	-1.51621	-1.98813	-3.68322	BGIOSGA028075	XP_006650963.1 protein WEAK CHLOROPLAST MOVEMENT UNDER BLUE LIGHT 1-like
TCONS_00031327	-1.51612	-4.10501	-3.69978	BGIOSGA008656	XP_015697114.1 uncharacterized membrane protein At1g06890-like isoform X2
TCONS_00002749	-1.51567	-1.29822	-0.241464	BGIOSGA002468	XP_006643699.1 endonuclease 2
TCONS_00016564	-1.51531	#NA	-3.70181	BGIOSGA007965	XP_006652774.1 xyloglucan endotransglucosylase/hydrolase protein 24-like
TCONS_00008328	-1.51447	0.352339	-0.0600327	BGIOSGA006098	XP_006647470.1 pre-mRNA-splicing factor ISY1 homolog

TCONS_00001432	-1.51367	-1.52314	-2.70588	BGIOSGA027561	XP_006644409.1 protein RAE1-like
TCONS_00000424	-1.51305	#NA	#NA	BGIOSGA002101	XP_006645634.1 GDSL esterase/lipase At5g45910-like
TCONS_00009338	-1.51259	1.04029	-0.184991	BGIOSGA008187	XP_006649421.1 FAD-dependent urate hydroxylase-like
TCONS_00025852	-1.51254	#NA	-1.70869	BGIOSGA016640	XP_006659085.1 kelch repeat-containing protein At3g27220-like
TCONS_00016517	-1.51246	-0.0636985	0.60379	BGIOSGA026486	NP_001335673.1uncharacterized LOC100192517
TCONS_00003009	-1.51213	-1.94012	-0.622235	BGIOSGA020901	XP_015695881.1 protein IQ-DOMAIN 1-like
TCONS_00021660	-1.51185	-2.78847	-0.803505	BGIOSGA027935	XP_006656667.1PREDICTED: uncharacterized protein LOC102704986
TCONS_00001252	-1.51146	-1.52593	-3.71137	BGIOSGA015659	XP_006644329.1 aldehyde dehydrogenase family 2 member C4-like
TCONS_00001748	-1.50932	-1.40234	-0.927823	BGIOSGA004383	XP_006644651.1 probable complex I intermediate-associated protein 30
TCONS_00005847	-1.50925	-0.478074	-0.716813	BGIOSGA016219	XP_015688556.1 putative disease resistance protein RGA4
TCONS_00010117	-1.5079	-0.899454	-1.29834	BGIOSGA035158	XP_003561613.1uncharacterized protein LOC100845964 isoform X2
TCONS_00023551	-1.50583	-0.689638	-2.38386	BGIOSGA015742	XP_015695039.1 RING-H2 finger protein ATL45-like
TCONS_00012939	-1.50574	-0.399489	-1.30516	BGIOSGA035762	XP_015690015.1 ubiquitin-conjugating enzyme 15-like
TCONS_00011324	-1.50574	#NA	#NA	#N/A	#N/A
TCONS_00030212	-1.50574	-1.81453	-0.305157	#N/A	#N/A
TCONS_00026578	-1.50564	-1.25654	-2.3333	BGIOSGA012043	XP_006659385.1 sulfate transporter 1.2-like
TCONS_00000185	-1.50471	-1.11068	-1.44511	BGIOSGA000067	XP_015688275.1 protein NRT1/ PTR FAMILY 8.1-like
TCONS_00013831	-1.5033	-0.871437	-0.775197	BGIOSGA013734	XP_004976559.1histone H1
TCONS_00002953	-1.50305	#NA	-1.31683	BGIOSGA021949	XP_011045355.1 keratin-associated protein 5-5-like
TCONS_00017973	-1.50225	#NA	-4.40483	BGIOSGA038476	XP_015692629.1 pyruvate decarboxylase 1-like
TCONS_00000054	-1.50172	-4.39475	-2.97925	BGIOSGA033309	XP_006654728.1 late embryogenesis abundant protein, group 3
TCONS_00007062	-1.50061	-2.2665	0.382826	BGIOSGA027466	XP_002447096.2myristoylated alanine-rich C-kinase substrate
TCONS_00014969	-1.49968	-4.62841	-2.60922	BGIOSGA017157	XP_006652844.1PREDICTED: uncharacterized protein LOC102709170
TCONS_00011167	-1.49902	-6.2685	-3.54712	BGIOSGA037606	XP_015691197.1 protochlorophyllide-dependent translocon component 52, chloroplastic-like
TCONS_00015507	-1.49801	0.537589	-3.28657	BGIOSGA015478	XP_014751506.1probable cleavage and polyadenylation specificity factor subunit 1 isoform X3
TCONS_00015470	-1.498	-0.354835	-0.59635	BGIOSGA029361	XP_006652120.1 probable L-ascorbate peroxidase 3
TCONS_00011310	-1.49788	-1.96425	-1.03014	BGIOSGA013916	XP_015690195.1 vacuolar protein sorting-associated protein 9A-like isoform X3
TCONS_00016430	-1.49724	-2.27073	-1.5515	BGIOSGA032403	XP_020403774.1uncharacterized LOC103646073 isoform X2
TCONS_00035489	-1.4969	-1.41102	-1.19882	BGIOSGA017808	XP_015698113.1 probable serine/threonine-protein kinase WNK8
TCONS_00014780	-1.49561	-2.34411	-3.55239	BGIOSGA022231	XP_006652667.1 SPX domain-containing membrane protein OsI_17046
TCONS_00018083	-1.49243	0.265899	-1.11524	BGIOSGA036578	XP_003568162.1cyclic nucleotide-gated ion channel 4
TCONS_00037022	-1.49221	-0.79667	0.35547	BGIOSGA027947	XP_015698270.1 E3 ubiquitin-protein ligase RGLG2-like
TCONS_00030355	-1.49184	-2.40699	-1.30367	BGIOSGA029444	XP_006661430.2PREDICTED: uncharacterized protein LOC102722423 isoform X1
TCONS_00005534	-1.49182	-2.26951	-1.19681	BGIOSGA031055	XP_006646951.1 squamosa promoter-binding-like protein 4 isoform X1
TCONS_00036053	-1.49008	-0.96802	-0.985592	BGIOSGA002717	XP_006657487.1 2'-deoxymugineic-acid 2'-dioxygenase-like
TCONS_00023200	-1.48926	-1.45266	-1.11342	BGIOSGA021316	XP_006658327.1 protein tesmin/TSO1-like CXC 2
TCONS_00007675	-1.48867	0.922925	0.089578	BGIOSGA037152	XP_015689003.1 probable LRR receptor-like serine/threonine-protein kinase At2g24230
TCONS_00023487	-1.48792	-1.86546	0.956952	BGIOSGA019803	XP_006657613.2 putative aldehyde oxidase-like protein
TCONS_00010737	-1.48726	0.172214	-0.43048	BGIOSGA010022	XP_004979814.1putative disease resistance RPP13-like protein 1 isoform X1
TCONS_00008777	-1.48615	-0.425316	-0.712316	BGIOSGA012071	XP_015689322.1 formin-like protein 16
TCONS_00001508	-1.48498	-2.50888	0.363497	BGIOSGA032101	XP_006645021.1 laccase-7
TCONS_00009249	-1.4849	-1.87169	0.0397194	BGIOSGA024325	XP_006651010.1 expansin-like A1
TCONS_00019907	-1.48304	-0.975744	0.472374	BGIOSGA001316	XP_006654868.1 hypersensitive-induced response protein 1
TCONS_00005289	-1.483	-0.0610577	-0.730237	BGIOSGA028060	XP_015696591.1 probable acyl-activating enzyme 1, peroxisomal
TCONS_00000665	-1.48104	-1.95455	-0.680352	BGIOSGA018944	XP_006644059.1 cyclic dof factor 2-like
TCONS_00032763	-1.48022	-0.148048	-0.7541	BGIOSGA035191	XP_006662099.1PREDICTED: uncharacterized protein LOC102722052
TCONS_00000393	-1.47978	-0.881728	-1.38721	BGIOSGA002971	XP_004968580.1eukaryotic translation initiation factor 3 subunit D
TCONS_00023854	-1.47952	-1.63454	-1.1138	BGIOSGA028169	XP_006657797.1 putative receptor-like protein kinase At4g00960
TCONS_00011396	-1.47933	-0.726395	-1.53263	BGIOSGA003789	XP_015690373.1 hexose carrier protein HEX6-like
TCONS_00012213	-1.47844	-1.82783	-0.499491	BGIOSGA026398	XP_006650044.1 serine/threonine-protein kinase AtPK2/AtPK19-like
TCONS_00006681	-1.47828	-0.150819	-0.847262	BGIOSGA020859	XP_022680824.1auxin response factor 12
TCONS_00011202	-1.4782	-0.0984744	-0.962981	BGIOSGA013803	XP_015691121.1 E3 ubiquitin-protein ligase RNF126-A-like
TCONS_00008105	-1.47769	-2.61825	-1.97598	BGIOSGA002617	XP_015688618.1 E3 ubiquitin-protein ligase Os03g0188200-like

TCONS_00014734	-1.47534	-3.65667	-0.670525	BGIOSGA030936	XP_003563120.1rop guanine nucleotide exchange factor 3
TCONS_00015944	-1.47519	-1.65686	-4.25586	BGIOSGA015032	XP_002447884.1putative FBD-associated F-box protein At5g38570
TCONS_00026682	-1.47492	-2.33528	-1.93462	BGIOSGA035935	XP_015696557.1 AP2-like ethylene-responsive transcription factor At1g16060
TCONS_00012256	-1.47393	-3.60124	-0.593232	BGIOSGA030609	XP_006650072.1 pollen-specific leucine-rich repeat extensin-like protein 1
TCONS_00033642	-1.47386	-2.07357	-0.7673	BGIOSGA035425	XP_015697692.1 uncharacterized aarF domain-containing protein kinase 1
TCONS_00031648	-1.47381	-1.57056	-1.81753	BGIOSGA033470	XP_002453145.2putative F-box protein At2g02030
TCONS_00002452	-1.47345	-0.57159	-0.559741	BGIOSGA005094	XP_006645268.1 histone-lysine N-methyltransferase, H3 lysine-9 specific SUVH4-like
TCONS_00025849	-1.4734	-1.2131	-1.44459	BGIOSGA013788	XP_006659083.2 uncharacterized membrane protein YuiD-like
TCONS_00030208	-1.47274	-0.437548	-2.38746	BGIOSGA029601	XP_004957213.1putative wall-associated receptor kinase-like 16
TCONS_00007266	-1.47156	-2.15023	-0.523078	BGIOSGA018203	XP_006646808.1 serine/arginine-rich splicing factor RS41 isoform X1
TCONS_00013598	-1.4715	-0.923111	-2.00716	BGIOSGA001846	XP_006652064.1 probable glucuronosyltransferase Os04g0103100
TCONS_00023981	-1.47092	0.394272	0.41317	BGIOSGA011151	XP_003562821.2protein indeterminate-domain 1
TCONS_00016550	-1.46955	-2.95886	-2.04303	BGIOSGA023255	XP_006652759.1 growth-regulating factor 3-like
TCONS_00000368	-1.46831	-0.680312	-0.46114	BGIOSGA036662	XP_006645602.1 cytochrome P450 90D2
TCONS_00026718	-1.46817	-2.04024	-0.741014	BGIOSGA004752	XP_015695985.1 respiratory burst oxidase homolog protein E
TCONS_00008389	-1.4681	-0.734732	-0.463011	BGIOSGA006032	XP_006648841.1 polyribonucleotide nucleotidyltransferase 2, mitochondrial
TCONS_00027632	-1.46734	-2.44707	-1.51245	BGIOSGA022639	XP_006659967.2 protein CHROMATIN REMODELING 35-like
TCONS_00030660	-1.46681	-2.34813	-1.49207	BGIOSGA028616	XP_006661617.1 putative disease resistance RPP13-like protein 3
TCONS_00033967	-1.46565	-0.817335	-0.200245	BGIOSGA008708	XP_015697839.1 pentatricopeptide repeat-containing protein At2g26790, mitochondrial-like
TCONS_00034339	-1.46356	-2.15234	-1.10581	BGIOSGA027813	XP_006662749.1 protein YLS9-like
TCONS_00022386	-1.46279	-2.27724	-2.34325	BGIOSGA021079	XP_006657039.1PREDICTED: uncharacterized protein At1g04910-like
TCONS_00015119	-1.46244	-1.27323	-0.737328	BGIOSGA017298	XP_015691605.1 molybdopterin biosynthesis protein CNX1
TCONS_00028201	-1.46225	-0.957531	-0.636383	BGIOSGA021106	XP_014757041.2uncharacterized protein LOC100840021
TCONS_00023363	-1.46189	0.164005	-1.17786	BGIOSGA025405	XP_006657566.1PREDICTED: uncharacterized protein LOC102717355
TCONS_00026058	-1.4614	-1.91335	#NA	BGIOSGA028128	XP_004951863.1uncharacterized protein LOC101762879
TCONS_00031718	-1.46111	-3.6401	-1.11997	BGIOSGA021572	XP_004983464.1peroxidase A2
TCONS_00023132	-1.4598	-0.108514	-0.85273	BGIOSGA017054	XP_009408527.1 outer envelope membrane protein 7
TCONS_00036575	-1.4598	1.54356	0.884236	BGIOSGA019597	XP_006663758.2PREDICTED: uncharacterized protein LOC102708666
TCONS_00022485	-1.45824	-0.918292	-0.441517	#N/A	#N/A
TCONS_00003647	-1.45763	0.408589	-0.124401	BGIOSGA001558	XP_006665113.1 tetratricopeptide repeat protein 5-like
TCONS_00007134	-1.45715	-0.837716	-1.4819	BGIOSGA015122	XP_015689115.1 U-box domain-containing protein 33-like
TCONS_00005095	-1.45648	-0.882363	-0.127248	BGIOSGA010253	XP_015692139.1 protein OSB2, chloroplastic-like
TCONS_00009083	-1.45617	-0.804746	-1.19214	BGIOSGA005322	XP_015689100.1PREDICTED: uncharacterized protein LOC102704126
TCONS_00014089	-1.45577	0.779708	0.434892	BGIOSGA020673	XP_021321789.1uncharacterized protein LOC8074283
TCONS_00010561	-1.45533	-2.46877	-3.0687	BGIOSGA000727	XP_015691131.1PREDICTED: uncharacterized protein LOC102710677
TCONS_00003272	-1.45382	-1.92498	#NA	#N/A	#N/A
TCONS_00034566	-1.45379	-0.645004	-2.38376	BGIOSGA007130	XP_015697529.1 receptor-like protein kinase HSL1
TCONS_00013791	-1.45111	#NA	-1.45894	#N/A	#N/A
TCONS_00000524	-1.44977	-3.18763	-2.98599	BGIOSGA018446	XP_006643973.1 D-amino-acid transaminase, chloroplastic-like
TCONS_00021542	-1.44951	-0.415273	-0.351641	BGIOSGA014991	XP_010491788.1 histone H3.3
TCONS_00009585	-1.44869	-0.825178	-1.49574	BGIOSGA018258	XP_015691676.1PREDICTED: uncharacterized protein LOC102716210 isoform X3
TCONS_00019654	-1.44849	-1.45119	-1.49274	BGIOSGA009712	XP_006654676.1 reticulon-like protein B1
TCONS_00005885	-1.44743	-0.897159	-0.361369	BGIOSGA014663	XP_002451987.2L-type lectin-domain containing receptor kinase IV.2
TCONS_00028346	-1.44732	0.387576	-0.468152	#N/A	#N/A
TCONS_00008388	-1.44702	-1.43533	-2.54141	BGIOSGA006033	XP_004953115.1serine/threonine protein phosphatase 2A 55 kDa regulatory subunit B beta isoform isoform X1
TCONS_00004006	-1.44648	-2.11593	-0.752738	BGIOSGA001185	XP_006646117.1PREDICTED: uncharacterized protein LOC102715814
TCONS_00014203	-1.445	-0.192571	-1.63896	BGIOSGA013242	XP_006652263.1 glucan endo-1,3-beta-glucosidase 14-like
TCONS_00011495	-1.44497	0.64742	-0.473834	#N/A	#N/A
TCONS_00002721	-1.4449	-4.07258	-2.39923	BGIOSGA030418	XP_006643718.1 protein O-linked-mannose beta-1,4-N-acetylglucosaminyltransferase 2-like
TCONS_00026472	-1.44279	-0.940485	-1.47919	BGIOSGA028541	XP_015695579.1PREDICTED: uncharacterized protein LOC107304721, partial
TCONS_00030477	-1.44279	#NA	-1.47919	BGIOSGA031191	XP_006660936.1 auxin-responsive protein SAUR36-like
TCONS_00017153	-1.44166	-3.19676	-1.96913	BGIOSGA019199	XP_006654058.2 villin-1-like
TCONS_00004734	-1.43953	-3.45303	-2.66467	BGIOSGA017328	XP_006645024.1 reticulon-like protein B12 isoform X2

transcriptome

TCONS_00012882	-1.4394	-3.29308	-2.04081	BGIOSGA035439	XP_002464115.1protein MARD1
TCONS_00013936	-1.43928	-1.08665	0.229254	BGIOSGA024728	XP_006653259.1 probable ribose-5-phosphate isomerase 1
TCONS_00030998	-1.43903	-1.79151	-1.71638	BGIOSGA033780	XP_006665052.2 probable LRR receptor-like serine/threonine-protein kinase At3g47570
TCONS_00036667	-1.4388	-1.2503	-0.777073	BGIOSGA034506	XP_006663811.1 suppressor of mec-8 and unc-52 protein homolog 2
TCONS_00018101	-1.43846	-1.40444	-0.941931	BGIOSGA000727	XP_008648000.1uncharacterized LOC100304390 isoform X1
TCONS_00015849	-1.43766	-1.72355	-1.32572	BGIOSGA003082	XP_006652254.1 serine/threonine-protein kinase D6PK-like
TCONS_00021638	-1.43699	-0.947969	0.313939	BGIOSGA029169	XP_015696453.1 dihydroneopterin aldolase 2-like
TCONS_00032197	-1.4359	-1.92075	-1.73947	BGIOSGA035853	XP_006661768.1 histone-lysine N-methyltransferase ATXR4
TCONS_00011430	-1.43527	#NA	-0.497612	#N/A	#N/A
TCONS_00015728	-1.43468	-0.107879	-2.05251	BGIOSGA035706	XP_006653304.2 wall-associated receptor kinase 2-like
TCONS_00014885	-1.43366	-2.95202	-1.50157	#N/A	#N/A
TCONS_00030715	-1.43347	-1.56125	-0.80692	BGIOSGA030033	XP_015691931.1 UPF0481 protein At3g47200-like
TCONS_00031027	-1.43223	-1.96042	-0.850878	BGIOSGA028420	XP_021307139.1uncharacterized protein LOC8080432 isoform X4
TCONS_00001113	-1.43142	1.95221	1.30027	BGIOSGA030264	XP_004968948.2DEAD-box ATP-dependent RNA helicase 15
TCONS_00011800	-1.43128	0.487831	-1.30547	BGIOSGA030469	XP_006649643.1 sugar transport protein 13
TCONS_00036424	-1.42986	-2.3277	-2.92975	BGIOSGA010161	XP_024319293.1wall-associated receptor kinase 5
TCONS_00025341	-1.42937	-0.372088	-1.51218	BGIOSGA020130	XP_021318493.1E3 ubiquitin-protein ligase PUB23
TCONS_00035251	-1.42937	#NA	-1.51218	#N/A	#N/A
TCONS_00028717	-1.42872	0.364142	-0.928824	BGIOSGA033529	XP_004960354.1chromosome transmission fidelity protein 8
TCONS_00012958	-1.42872	-1.95779	#NA	#N/A	#N/A
TCONS_00014663	-1.42845	-2.07508	-1.77773	BGIOSGA017377	XP_015691317.1 probable LRR receptor-like serine/threonine-protein kinase At1g63430 isoform X2
TCONS_00016065	-1.42809	-1.22153	-0.515352	BGIOSGA014913	XP_006652396.2 protein FATTY ACID EXPORT 1, chloroplastic-like
TCONS_00002260	-1.42807	-1.7274	0.212575	BGIOSGA005586	XP_006646518.2 protein NRT1/ PTR FAMILY 5.10-like
TCONS_00011413	-1.42748	-2.70204	-0.459507	BGIOSGA033413	XP_006649230.1PREDICTED: uncharacterized protein LOC102700872
TCONS_00022690	-1.42747	#NA	-1.51687	BGIOSGA004834	NP_001145346.1uncharacterized LOC100278675 precursor
TCONS_00026173	-1.4273	-4.28752	-1.13079	BGIOSGA023532	XP_015695535.1 xyloglucan endotransglycosylase/hydrolase protein 8
TCONS_00002015	-1.42688	-0.737454	-0.78139	#N/A	#N/A
TCONS_00014351	-1.4263	#NA	-1.5198	BGIOSGA016507	XP_006652342.1 UPF0483 protein AGAP003155-like
TCONS_00025389	-1.4263	0.846866	#NA	#N/A	#N/A
TCONS_00028416	-1.42575	0.188461	-0.775705	BGIOSGA032579	XP_008812461.1 molybdenum cofactor sulfurase 3-like
TCONS_00028279	-1.42517	#NA	-2.52257	BGIOSGA014721	XP_003572368.2beta-glucosidase 28
TCONS_00026928	-1.42517	1.20821	0.0623904	BGIOSGA023829	XP_015694480.1 probable mediator of RNA polymerase II transcription subunit 26b
TCONS_00024408	-1.42512	-1.67929	-1.37485	BGIOSGA026447	XP_015694778.1PREDICTED: uncharacterized protein LOC102719885
TCONS_00019903	-1.42453	-2.81169	-1.34501	BGIOSGA002421	XP_004961036.3transcription factor SRM1
TCONS_00036089	-1.42444	-1.17308	-0.795132	BGIOSGA006744	XP_006664050.1 cyclic pyranopterin monophosphate synthase, mitochondrial
TCONS_00031280	-1.4236	0.0365862	#NA	BGIOSGA020796	XP_008644805.1uncharacterized protein LOC103626189
TCONS_00020475	-1.42323	-1.64529	-0.392101	BGIOSGA022654	XP_015694111.1PREDICTED: uncharacterized protein LOC102699416 isoform X1
TCONS_00012442	-1.42316	-0.541057	0.337385	#N/A	#N/A
TCONS_00025286	-1.4231	0.036056	0.472293	BGIOSGA024140	XP_002462978.1trinucleotide repeat-containing gene 18 protein
TCONS_00036969	-1.4227	-1.35763	-2.01413	BGIOSGA003930	XP_021321955.1disease resistance protein RPM1
TCONS_00006778	-1.42179	-1.52725	-1.90298	BGIOSGA008951	XP_006647814.1 KH domain-containing protein At5g56140-like
TCONS_00016251	-1.42037	#NA	-1.53448	BGIOSGA014723	XP_015692291.1 beta-glucosidase 16-like isoform X1
TCONS_00014619	-1.42037	0.355106	1.63544	#N/A	#N/A
TCONS_00004839	-1.41995	-2.28919	-1.85745	BGIOSGA027558	XP_002438208.2transcription factor TGAL3
TCONS_00011265	-1.41849	-1.01608	-0.1681	BGIOSGA005972	XP_006650842.1PREDICTED: uncharacterized protein LOC102721256
TCONS_00017544	-1.41839	-0.383947	#NA	BGIOSGA033086	XP_004961229.1homeobox-DDT domain protein RLT2
TCONS_00007479	-1.41803	1.83806	0.0446263	BGIOSGA007681	NP_001149805.1CUE domain containing protein
TCONS_00034149	-1.41803	-1.9693	#NA	BGIOSGA040457	XP_006664874.2 biogenesis of lysosome-related organelles complex 1 subunit 1-like
TCONS_00026424	-1.41769	-1.95515	-2.21898	BGIOSGA026350	XP_004972482.1probable serine/threonine-protein kinase At1g54610
TCONS_00004547	-1.41666	1.93612	0.456191	BGIOSGA000661	XP_015688081.1PREDICTED: uncharacterized protein LOC102706146 isoform X1
TCONS_00034427	-1.41648	-1.28902	-1.30575	BGIOSGA034060	XP_004980237.1protein ACCELERATED CELL DEATH 6 isoform X1
TCONS_00028560	-1.41633	0.531381	-0.129593	#N/A	#N/A
TCONS_00025846	-1.41609	-2.23677	-0.240956	BGIOSGA030559	XP_004983906.1zinc finger CCCH domain-containing protein 54
TCONS_00002037	-1.41556	-0.942463	-1.42018	BGIOSGA037085	XP_006644885.1 transcription factor GAMYB

transcriptome

TCONS_00029895	-1.41541	-0.97212	-0.546985	BGIOSGA013188	XP_021308134.1putative leucine-rich repeat receptor-like serine/threonine-protein kinase At2g04300
TCONS_00019339	-1.41514	-1.56446	-2.6206	BGIOSGA025019	XP_006654434.1 potassium channel AKT2 isoform X1
TCONS_00016007	-1.41491	0.158804	-0.693827	BGIOSGA002099	XP_006653479.2 somatic embryogenesis receptor kinase 1-like
TCONS_00002996	-1.41482	#NA	-0.226538	BGIOSGA016346	XP_021319432.1uncharacterized protein LOC8075267
TCONS_00001488	-1.41482	#NA	0.451534	#N/A	#N/A
TCONS_00013885	-1.41477	-0.314852	-2.22037	BGIOSGA035852	XP_006653242.1 wall-associated receptor kinase 5-like
TCONS_00026763	-1.41399	-1.97365	-1.55057	BGIOSGA005241	XP_015695441.1 transcription factor bHLH149-like
TCONS_00012703	-1.41373	-0.388979	0.256116	BGIOSGA010263	XP_015690615.1 F-box-like/WD repeat-containing protein TBL1X
TCONS_00009758	-1.41347	0.466349	0.0330674	#N/A	#N/A
TCONS_00026574	-1.41296	-0.752381	-3.13813	BGIOSGA028636	XP_006652173.1 50S ribosomal protein L27-like
TCONS_00002137	-1.41296	0.832582	0.0317969	#N/A	#N/A
TCONS_00035675	-1.41272	0.609922	#NA	#N/A	#N/A
TCONS_00036550	-1.41225	-1.97556	0.766952	BGIOSGA034623	NP_001151353.2RING-H2 finger protein ATL3F
TCONS_00022896	-1.4118	-0.976067	-2.55612	#N/A	#N/A
TCONS_00035503	-1.41137	1.60841	0.764706	BGIOSGA003050	XP_006664297.2PREDICTED: uncharacterized protein LOC102709420
TCONS_00029208	-1.41116	-0.97679	0.2496	BGIOSGA028881	XP_015696610.1PREDICTED: uncharacterized protein LOC102700366
TCONS_00016402	-1.41096	-1.39206	-0.973315	BGIOSGA014564	XP_015692235.1PREDICTED: uncharacterized protein LOC107304136
TCONS_00004626	-1.41056	-0.392511	0.762639	#N/A	#N/A
TCONS_00012101	-1.4104	-2.18324	-1.737	BGIOSGA036631	XP_006649941.1 probable xyloglucan 6-xylosyltransferase 1
TCONS_00025944	-1.40969	-4.87246	-4.12966	BGIOSGA028019	XP_015695903.1 protein CCA1 isoform X1
TCONS_00008283	-1.40944	-3.34131	-3.20846	BGIOSGA031098	XP_015688626.1 putative UDP-rhamnose:rhamnosyltransferase 1
TCONS_00008482	-1.40918	-1.7474	-1.83258	BGIOSGA005943	XP_015698129.1 magnesium/proton exchanger 1
TCONS_00037082	-1.40771	-2.98077	-1.5665	#N/A	#N/A
TCONS_00007253	-1.4069	-0.981744	-0.568556	BGIOSGA007230	XP_015689670.1 protein Simiate
TCONS_00008991	-1.40641	1.18758	-1.56982	#N/A	#N/A
TCONS_00032568	-1.40594	#NA	-0.834028	BGIOSGA025050	NP_001143412.2uncharacterized LOC100276059
TCONS_00010957	-1.40583	-1.44248	-1.12382	BGIOSGA019740	XP_015690897.1 SRSF protein kinase 2-like
TCONS_00014011	-1.40561	-1.98331	-0.919752	BGIOSGA004029	XP_015691756.1 dCTP pyrophosphatase 1-like
TCONS_00016058	-1.4055	-0.864146	-3.10815	BGIOSGA020163	XP_006653507.1PREDICTED: uncharacterized protein LOC102702466
TCONS_00031794	-1.40499	0.430966	0.0115782	BGIOSGA022235	XP_006656595.1 LRR receptor-like serine/threonine-protein kinase ERL1
TCONS_00036175	-1.4048	-0.814386	-1.45839	BGIOSGA037532	XP_006664083.2PREDICTED: uncharacterized protein LOC102714792
TCONS_00024527	-1.40434	-0.762487	-1.15999	BGIOSGA024864	XP_004956165.1probable UDP-3-O-acylglucosamine N-acyltransferase 2, mitochondrial isoform X1
TCONS_00014408	-1.40425	1.01501	0.594676	#N/A	#N/A
TCONS_00002984	-1.40336	-1.9861	1.96683	BGIOSGA003959	XP_002440727.1probable WRKY transcription factor 50
TCONS_00012777	-1.40336	0.428938	-1.16245	BGIOSGA022197	XP_003578091.1plant UBX domain-containing protein 2
TCONS_00006560	-1.40336	-0.9861	#NA	#N/A	#N/A
TCONS_00022231	-1.40247	#NA	#NA	BGIOSGA018396	XP_015693899.1 B-cell receptor-associated protein 31
TCONS_00026495	-1.40091	-1.67509	-1.98574	BGIOSGA002510	XP_006643674.1 glycerophosphodiester phosphodiesterase protein kinase domain-containing GDPDL2-like
TCONS_00032680	-1.40073	-4.31135	-2.90602	BGIOSGA011669	XP_006662046.1 expansin-B2
TCONS_00012604	-1.40064	-1.18218	-3.58431	BGIOSGA013015	XP_015689958.1 GDSL esterase/lipase At5g55050-like
TCONS_00017626	-1.40064	-0.530104	0.000655194	BGIOSGA019688	XP_006654295.1 CAAX prenyl protease 2
TCONS_00014623	-1.4002	-1.79745	-0.222849	BGIOSGA008665	XP_009385658.1PREDICTED: uncharacterized protein At2g24330
TCONS_00037474	-1.40008	#NA	0.321189	BGIOSGA019301	XP_004963100.1auxin-responsive protein IAA30
TCONS_00004397	-1.3995	-2.72795	-0.587159	BGIOSGA018558	XP_006644710.1 coiled-coil domain-containing protein 25-like
TCONS_00001634	-1.39922	0.00867179	0.0751198	BGIOSGA032331	XP_006646238.1 mitogen-activated protein kinase kinase kinase ANP1-like
TCONS_00030147	-1.39871	-4.31391	-1.74113	BGIOSGA034368	XP_006660677.1 1-aminocyclopropane-1-carboxylate oxidase 1
TCONS_00014332	-1.39868	-0.992012	-3.17416	BGIOSGA032434	XP_006652337.2 non-functional NADPH-dependent codeinone reductase 2-like
TCONS_00004935	-1.39861	-2.19856	-1.24834	BGIOSGA035861	XP_006645201.1PREDICTED: uncharacterized protein LOC102718506
TCONS_00014213	-1.39851	-3.57719	-0.087117	BGIOSGA008366	XP_004975580.1mitochondrial fission protein ELM1 isoform X1
TCONS_00032694	-1.39842	-1.99234	-0.174812	BGIOSGA031416	XP_015697256.1 S-norcoclaurine synthase 1-like isoform X1
TCONS_00011149	-1.39791	1.02692	0.368254	BGIOSGA025317	XP_006650743.1PREDICTED: uncharacterized protein LOC102715558
TCONS_00032146	-1.39782	-0.0112485	-1.32831	BGIOSGA013840	XP_006661741.1 serine/threonine-protein kinase At5g01020-like
TCONS_00002078	-1.39776	-0.993171	-0.212965	BGIOSGA028353	XP_015693672.1 uncharacterized membrane protein At1g06890-like isoform X2
TCONS_00001803	-1.39755	-3.99344	-0.132569	BGIOSGA002928	XP_024315961.1protein IQ-DOMAIN 14 isoform X3
TCONS_00000330	-1.39753	-1.14546	-1.59204	BGIOSGA012348	XP_006643847.1 polynucleotide 5'-hydroxyl-kinase NOL9-like

TCONS_00007784	-1.39731	-1.06086	-1.35159	BGIOSGA000686	XP_006647157.1 transcription factor bHLH133-like
TCONS_00019490	-1.39731	-3.99374	#NA	BGIOSGA029984	XP_004969770.1probable sulfate transporter 3.5
TCONS_00022620	-1.39728	-1.50835	-2.32964	BGIOSGA020821	XP_004965797.1uncharacterized membrane protein At1g16860 isoform X1
TCONS_00033051	-1.39717	-1.11944	-0.370541	BGIOSGA022197	XP_014757850.2uncharacterized protein LOC106866713
TCONS_00029931	-1.39715	-1.73091	-0.32997	BGIOSGA032130	XP_002468294.1UDP-glucose flavonoid 3-O-glucosyltransferase 7
TCONS_00009366	-1.39691	-2.5792	-0.178544	BGIOSGA002256	XP_015690509.1 probable indole-3-pyruvate monooxygenase YUCCA1 isoform X1
TCONS_00005950	-1.39677	-0.409452	-0.48701	BGIOSGA025656	XP_006648586.1 obtusifoliol 14-alpha demethylase-like
TCONS_00021369	-1.39661	-1.10154	-3.40169	BGIOSGA003500	XP_006657340.2 alpha-amylase isozyme 2A-like
TCONS_00011135	-1.39654	-1.52121	-0.542142	BGIOSGA013734	XP_006651910.1 histone H1-like
TCONS_00037442	-1.39641	-0.554295	-0.272889	BGIOSGA023700	XP_006664691.1 cytoplasmic tRNA 2-thiolation protein 2
TCONS_00004000	-1.39624	-2.2402	-1.34733	BGIOSGA026166	XP_006646112.1 hydroquinone glucosyltransferase-like
TCONS_00000302	-1.39619	-5.45458	-2.24745	BGIOSGA034320	XP_021310923.1coleoptile phototropism protein 1
TCONS_00002939	-1.39598	-1.73238	-0.21738	BGIOSGA020418	XP_006643817.1 probable prolyl 4-hydroxylase 6
TCONS_00032579	-1.39595	0.66751	-4.18093	BGIOSGA031535	XP_003571979.2probable trehalase
TCONS_00034104	-1.39591	-4.80285	-3.40341	BGIOSGA000508	XP_006663136.1 laccase-22
TCONS_00036119	-1.39587	1.14741	-1.37376	BGIOSGA033091	XP_004962885.1zinc finger CCCH domain-containing protein 67
TCONS_00015985	-1.39547	-0.551272	-0.519153	BGIOSGA014994	XP_006652341.1 uncharacterized membrane protein At3g27390
TCONS_00027181	-1.39515	-1.05786	-0.860977	BGIOSGA031125	XP_006659015.1 flap endonuclease GEN-like 2
TCONS_00003786	-1.39507	-1.58152	-3.18309	BGIOSGA001425	XP_014757678.1DUF724 domain-containing protein 3 isoform X1
TCONS_00018408	-1.39492	-1.85633	-3.69061	BGIOSGA024641	XP_006655642.1 RNA polymerase sigma factor sigE, chloroplastic/mitochondrial
TCONS_00014281	-1.39461	-0.827209	0.258709	BGIOSGA016910	XP_006652296.1 subtilisin-like protease SBT1.7
TCONS_00014198	-1.39239	-0.396073	-0.789707	BGIOSGA004836	XP_006652261.2PREDICTED: uncharacterized protein LOC102701162
TCONS_00013388	-1.38492	-0.00936339	-0.0329883	BGIOSGA025378	XP_006650770.1 sugar phosphate exchanger 3-like
TCONS_00016502	-1.3828	-0.365112	-1.04305	BGIOSGA022213	XP_015691879.1PREDICTED: uncharacterized protein LOC102718260
TCONS_00030903	-1.38276	-2.19263	-3.23597	BGIOSGA001335	XP_006661699.1 UPF0301 protein TC_0483
TCONS_00002188	-1.38216	0.15043	0.12002	BGIOSGA028586	XP_006645038.1PREDICTED: uncharacterized protein LOC102718225
TCONS_00002709	-1.38173	-1.30099	-1.48502	BGIOSGA002514	XP_006643674.1 glycerophosphodiester phosphodiesterase protein kinase domain-containing GDPDL2-like
TCONS_00027818	-1.38123	-0.599138	-0.654643	BGIOSGA000224	XP_006660046.2 60S ribosomal protein L7a
TCONS_00036964	-1.37871	-3.40003	-0.44009	BGIOSGA000716	XP_015689790.1 ABC transporter B family member 19-like
TCONS_00025723	-1.37856	-1.30311	-1.38595	BGIOSGA023457	XP_015694971.1 NAC domain-containing protein 92-like
TCONS_00019630	-1.37813	-0.360089	-0.389364	BGIOSGA020191	XP_006654648.1 acetyltransferase NSI
TCONS_00006734	-1.37757	-1.81134	-1.97008	BGIOSGA021523	XP_015689062.1 putative receptor-like protein kinase At4g00960
TCONS_00021644	-1.37706	-2.37142	-3.63432	BGIOSGA021769	XP_004964810.13'-N-debenzoyl-2'-deoxytaxol N-benzoyltransferase
TCONS_00021880	-1.37579	-1.62315	-1.63643	BGIOSGA035484	XP_004953948.1ethylene-responsive transcription factor RAP2-4
TCONS_00005035	-1.37552	-0.619178	-0.222555	BGIOSGA018187	XP_006645284.1 lysine-rich arabinogalactan protein 19-like
TCONS_00021197	-1.37322	-0.991544	-0.939963	BGIOSGA000410	XP_006657218.2 protein STRUBBELIG-RECEPTOR FAMILY 3-like
TCONS_00019438	-1.37306	-0.740919	-1.33207	BGIOSGA007812	XP_006654513.1 probable monofunctional riboflavin biosynthesis protein RIBA 3, chloroplastic
TCONS_00000173	-1.37289	-0.61829	-1.09346	BGIOSGA034487	XP_003565134.160S ribosomal protein L26-1
TCONS_00004059	-1.37235	0.876364	-1.08928	BGIOSGA001132	XP_006644424.1 single-stranded DNA-binding protein, mitochondrial
TCONS_00021156	-1.37204	0.120784	-0.0523411	BGIOSGA037013	XP_006656307.1 transmembrane 9 superfamily member 9-like
TCONS_00036516	-1.36992	-1.23097	-2.43912	BGIOSGA014750	XP_015699009.1 caffeoylshikimate esterase-like
TCONS_00007842	-1.36703	-1.35727	-2.32769	BGIOSGA019941	XP_006647194.1 bidirectional sugar transporter SWEET4
TCONS_00033571	-1.36673	-1.8402	-1.7196	BGIOSGA022940	XP_015699017.1 obtusifoliol 14-alpha demethylase
TCONS_00013108	-1.36616	-1.86661	-3.87895	BGIOSGA009622	XP_006650534.1 transcription factor bHLH74-like isoform X2
TCONS_00023387	-1.36536	-1.63191	-0.159325	BGIOSGA014675	XP_015694575.1 AP2-like ethylene-responsive transcription factor TOE3
TCONS_00002612	-1.36493	-2.40613	-1.42773	BGIOSGA005251	XP_006645339.1 probable aspartyl aminopeptidase
TCONS_00014646	-1.36379	-0.944946	-1.52559	BGIOSGA000463	XP_004976316.1RNA polymerase II C-terminal domain phosphatase-like 1 isoform X1
TCONS_00005628	-1.36356	-0.443618	-0.663006	BGIOSGA015622	XP_006648429.2 putative disease resistance RPP13-like protein 3
TCONS_00028684	-1.36272	-0.104156	-0.12181	BGIOSGA030431	XP_015696744.1 SURP and G-patch domain-containing protein 1-like protein
TCONS_00024353	-1.3627	-1.59248	-0.964198	BGIOSGA032557	XP_015694767.1 CBL-interacting protein kinase 29
TCONS_00022568	-1.36258	-1.0114	-0.507131	BGIOSGA007658	XP_015693398.1 protein N-terminal asparagine amidohydrolase
TCONS_00017555	-1.36101	-2.53258	-1.42567	BGIOSGA014673	XP_006660481.1 cyclin-P4-1-like

TCONS_00022923	-1.36063	-0.916064	-0.453626	BGIOSGA024128	XP_024316444.1ubiquitin carboxyl-terminal hydrolase MINDY-1
TCONS_00006316	-1.35937	-0.77917	-2.33574	BGIOSGA004016	XP_006648777.1PREDICTED: uncharacterized protein LOC102711408
TCONS_00023313	-1.35926	0.46677	0.619665	BGIOSGA025356	XP_015695265.1 rRNA biogenesis protein RRP5
TCONS_00018960	-1.3574	-1.26745	-1.67663	BGIOSGA029407	XP_006655145.1 protein HAPLESS 2-A-like
TCONS_00000221	-1.3569	-2.42124	-2.32532	BGIOSGA016058	XP_015694754.1 gamma-glutamyltranspeptidase 3
TCONS_00002077	-1.35679	-5.40388	-2.99798	BGIOSGA029202	XP_006644922.1 expansin-A2
TCONS_00014185	-1.35675	-2.21284	-2.62686	BGIOSGA031527	XP_006652245.1 arogenate dehydratase/prephenate dehydratase 6, chloroplastic-like
TCONS_00011978	-1.35648	-0.953341	-3.1374	BGIOSGA021210	XP_006649824.1 probable protein phosphatase 2C 30
TCONS_00007552	-1.35526	-1.3667	-1.14169	BGIOSGA010085	XP_004951545.1glucan endo-1,3-beta-glucosidase 14
TCONS_00011620	-1.35523	-1.36516	-1.64288	BGIOSGA018193	XP_015691112.1 BEL1-like homeodomain protein 7
TCONS_00031688	-1.35461	-1.25214	-1.30973	BGIOSGA011158	XP_006661721.1 protein NRT1/ PTR FAMILY 8.3-like
TCONS_00019615	-1.3544	1.02895	-0.557558	BGIOSGA023300	XP_004961534.1probable xyloglucan glycosyltransferase 7
TCONS_00034245	-1.35436	0.523783	-0.682115	BGIOSGA027706	XP_006659092.1 60S acidic ribosomal protein P0
TCONS_00028791	-1.35415	-1.21929	-0.821208	BGIOSGA003221	XP_002462243.1L-type lectin-domain containing receptor kinase IX.1
TCONS_00022664	-1.3535	-1.61861	-0.947836	BGIOSGA020776	XP_015693850.1 phospholipase D Z-like
TCONS_00025758	-1.3519	-1.62271	-1.3585	BGIOSGA037594	XP_002463446.1phosphoinositide phospholipase C 2
TCONS_00004648	-1.35136	0.233122	-0.508984	BGIOSGA020043	XP_006644932.1 protein NUCLEAR FUSION DEFECTIVE 4-like
TCONS_00027018	-1.35063	-0.377336	-0.601909	#N/A	#N/A
TCONS_00018985	-1.34998	-0.318886	-1.44235	BGIOSGA036497	XP_008652591.1uncharacterized protein LOC103632616
TCONS_00033199	-1.34987	-1.51961	-0.118634	BGIOSGA034255	XP_021316408.1uncharacterized protein LOC8071576 isoform X1
TCONS_00021315	-1.34895	-2.17627	-0.00828647	BGIOSGA023545	XP_006657294.1 choline monooxygenase, chloroplastic
TCONS_00014755	-1.34892	-0.847204	-0.975193	BGIOSGA003161	XP_021318855.1protein indeterminate-domain 5, chloroplastic
TCONS_00000932	-1.34891	-0.642146	-1.23353	BGIOSGA003583	XP_004965304.1protein DMP2
TCONS_00018284	-1.34715	-0.87602	-1.06137	BGIOSGA013131	XP_006654763.2 T-complex protein 1 subunit beta-like
TCONS_00015089	-1.34705	-0.541871	-1.17883	BGIOSGA014196	XP_006652937.1 G-type lectin S-receptor-like serine/threonine-protein kinase At1g34300
TCONS_00019756	-1.34521	-0.713935	-0.532306	BGIOSGA037085	XP_006654754.1 transcription factor RAX1-like
TCONS_00035701	-1.34518	-0.833336	-0.983117	BGIOSGA004078	XP_006664407.2PREDICTED: uncharacterized protein At1g04910-like
TCONS_00011823	-1.34516	-0.244499	-1.64757	BGIOSGA033518	XP_015691059.1 glucan endo-1,3-beta-glucosidase 7-like isoform X1
TCONS_00012647	-1.34491	-0.843004	-0.960164	BGIOSGA008476	XP_006651548.1 sugar carrier protein C-like
TCONS_00003189	-1.34291	-1.34519	-0.965225	BGIOSGA006602	XP_006643983.1 C2 domain-containing protein At1g53590-like isoform X1
TCONS_00029487	-1.34175	-0.528915	-0.529523	BGIOSGA031241	XP_004957593.1F-box/kelch-repeat protein At5g42360-like
TCONS_00000258	-1.34147	-0.997968	-1.65536	BGIOSGA002838	XP_003565373.1receptor-like protein 9DC3
TCONS_00036019	-1.34008	-1.41227	0.372513	BGIOSGA027441	XP_015695956.1 putative disease resistance protein RGA1
TCONS_00007089	-1.33924	-1.60621	-1.35298	BGIOSGA009270	XP_015689525.1 F-box protein PP2-B1-like
TCONS_00032314	-1.33905	-1.70225	-0.765722	BGIOSGA022026	XP_006662400.1PREDICTED: uncharacterized protein LOC102702693, partial
TCONS_00024392	-1.33682	#NA	#NA	BGIOSGA028580	XP_004958765.3nicotianamine synthase 3
TCONS_00021596	-1.33618	-1.99655	-1.72366	BGIOSGA024466	XP_006655780.1 probable sulfate transporter 3.4
TCONS_00018876	-1.33612	-0.932078	-1.20875	BGIOSGA021657	XP_006655114.1 transcription factor bHLH68-like isoform X1
TCONS_00021899	-1.33602	-0.214743	-1.63774	BGIOSGA033918	XP_006656803.1 BRASSINOSTEROID INSENSITIVE 1-associated receptor kinase 1-like
TCONS_00018518	-1.33529	-0.566493	-0.754898	BGIOSGA033516	XP_015692414.1 serine/arginine-rich splicing factor RS2Z33 isoform X2
TCONS_00007369	-1.33491	-1.0267	-1.21174	BGIOSGA007102	XP_006648317.2 receptor-like protein 2
TCONS_00018500	-1.3346	0.52689	-0.725387	BGIOSGA018933	XP_015692454.1 protein-tyrosine-phosphatase MKP1
TCONS_00000301	-1.33457	0.11037	-0.212583	BGIOSGA019266	XP_003569538.4UDP-glycosyltransferase 73C1
TCONS_00037484	-1.33402	-2.61279	-1.72852	BGIOSGA019937	XP_006664174.1 serine/threonine-protein kinase Nek2
TCONS_00000002	-1.33401	0.109662	0.418293	BGIOSGA033248	XP_006643621.2 monocopper oxidase-like protein SKU5
TCONS_00030541	-1.33358	-1.45126	-1.6966	BGIOSGA028182	XP_006660981.1 potassium transporter 18
TCONS_00028830	-1.33316	0.664123	-2.36806	BGIOSGA020220	XP_015696312.1 probable LRR receptor-like serine/threonine-protein kinase At1g51810
TCONS_00007282	-1.33126	-1.08137	-1.23129	#N/A	XP_006646828.1 dnaJ homolog subfamily B member 1-like
TCONS_00008632	-1.32997	-7.42221	-4.43824	BGIOSGA026145	XP_006647731.1 probable 4-coumarate--CoA ligase 2
TCONS_00014591	-1.3296	-2.5975	-3.68346	BGIOSGA019723	XP_006652510.1 beta-glucosidase 16-like
TCONS_00024117	-1.32956	-2.63111	-1.32935	BGIOSGA010588	XP_015695297.1 protein WEAK CHLOROPLAST MOVEMENT UNDER BLUE LIGHT 1-like
TCONS_00009255	-1.32924	-0.622564	-1.06592	BGIOSGA035417	XP_006649344.1 chitin elicitor-binding protein-like
TCONS_00014757	-1.32872	-0.204878	-0.325099	BGIOSGA022137	XP_022683888.1protein PHR1-LIKE 3 isoform X2

transcriptome

TCONS_00026599	-1.32849	-1.47952	-1.73885	BGIOSGA002943	XP_015691515.1 AP2-like ethylene-responsive transcription factor BBM1
TCONS_00026798	-1.32795	-0.687117	-1.0527	BGIOSGA033119	XP_022683187.1protein DETOXIFICATION 27
TCONS_00000171	-1.3279	-0.779754	-1.60285	BGIOSGA034487	XP_003565134.160S ribosomal protein L26-1
TCONS_00033359	-1.32654	-0.561264	-1.33051	BGIOSGA016128	XP_006662878.1 probable protein phosphatase 2C 75
TCONS_00035107	-1.32638	-0.836262	0.415054	BGIOSGA001532	XP_015697707.1 ABC transporter G family member 48
TCONS_00004902	-1.32613	-2.86663	-1.85347	BGIOSGA033262	XP_006646566.2 probable methyltransferase PMT17
TCONS_00029636	-1.32508	0.742711	-0.289727	BGIOSGA030182	NP_001146226.1putative ARM repeat-containing protein containing family protein
TCONS_00017760	-1.324	-2.63082	-2.46473	BGIOSGA019824	NP_001149610.1dnaJ domain containing protein
TCONS_00003890	-1.32353	-0.275528	-1.09	BGIOSGA031508	XP_002458061.1protein QUIRKY
TCONS_00014975	-1.32249	-3.18574	-1.2458	BGIOSGA017163	XP_021318973.1peroxisomal (S)-2-hydroxy-acid oxidase GLO2
TCONS_00014287	-1.3212	-1.58281	-1.45196	BGIOSGA013697	XP_006652300.1 serine/threonine-protein kinase SAPK7
TCONS_00006792	-1.32104	-2.79695	-2.75878	BGIOSGA027700	XP_015689567.1 putative U-box domain-containing protein 42
TCONS_00037404	-1.32051	-0.816168	-0.660346	BGIOSGA005808	XP_015698749.1 asparagine synthetase domain-containing protein 1-like
TCONS_00018106	-1.32033	-0.401852	-1.75949	BGIOSGA023082	XP_004961554.1protein LURP-one-related 5
TCONS_00028124	-1.31977	-0.227915	-1.52059	BGIOSGA029678	XP_021310336.1F-box/FBD/LRR-repeat protein At1g13570
TCONS_00001658	-1.31951	-1.16209	-2.68815	BGIOSGA013293	XP_015689693.1 transcription factor bHLH13-like
TCONS_00005308	-1.31942	-0.783966	-0.522114	BGIOSGA007418	XP_006646812.1 peroxisome biogenesis protein 16
TCONS_00017063	-1.31876	-3.24066	-3.11645	BGIOSGA013535	XP_002270750.3 probable WRKY transcription factor 65
TCONS_00000709	-1.31872	-2.7869	-4.70731	BGIOSGA005016	XP_006644085.1 kinectin-like isoform X1
TCONS_00016006	-1.31871	-1.72919	-0.660364	BGIOSGA002796	XP_006653478.1 gamma-glutamyltranspeptidase 1-like
TCONS_00015481	-1.31846	0.0219828	-2.4169	BGIOSGA026351	XP_006653215.1 G-type lectin S-receptor-like serine/threonine-protein kinase B120
TCONS_00014935	-1.31727	-0.183741	-1.00995	BGIOSGA017116	XP_006652820.1 myosin heavy chain, non-muscle-like
TCONS_00004698	-1.31683	-0.9791	-1.56096	BGIOSGA035878	NP_001147780.1SLT1 protein
TCONS_00024907	-1.31571	-1.11852	-0.619197	BGIOSGA021823	XP_006655832.2 zinc finger CCCH domain-containing protein 40
TCONS_00035633	-1.31508	-0.63175	-0.332284	BGIOSGA037054	XP_015698227.1 nucleolar protein 6
TCONS_00004390	-1.31507	0.713495	-0.0945423	BGIOSGA000831	XP_010232318.2uncharacterized protein LOC100822531
TCONS_00036524	-1.31497	-2.91112	-4.09479	BGIOSGA002974	XP_006663143.1 fatty acid desaturase DES3-like
TCONS_00031805	-1.31496	-0.40864	-0.287472	BGIOSGA024909	XP_024315700.1uncharacterized protein LOC100840606 isoform X1
TCONS_00002261	-1.31355	-3.49787	-1.77638	BGIOSGA012201	XP_004970732.1protein NRT1/ PTR FAMILY 5.16
TCONS_00015014	-1.31344	-1.91305	-2.61316	BGIOSGA025052	XP_004976919.1organic cation/carnitine transporter 4
TCONS_00005747	-1.31241	-1.20652	-0.445762	BGIOSGA011908	XP_006647111.1 serine/threonine-protein kinase EDR1-like
TCONS_00038416	-1.31147	0.791853	0.322806	#N/A	XP_012440607.1PREDICTED: uncharacterized protein LOC105765883
TCONS_00001596	-1.31026	-0.42961	-0.393014	BGIOSGA017610	XP_006644560.1PREDICTED: uncharacterized protein LOC102719812
TCONS_00004925	-1.30974	-0.616372	-0.325497	#N/A	#N/A
TCONS_00002659	-1.30911	-2.89443	0.185172	BGIOSGA018999	XP_006643637.1 charged multivesicular body protein 5-like isoform X1
TCONS_00035400	-1.30892	-2.20229	-3.787	BGIOSGA026078	XP_006663123.1 zinc finger protein ZAT8-like
TCONS_00034027	-1.30854	-0.69746	-1.59905	BGIOSGA031872	XP_015698166.1 probable serine/threonine-protein kinase DDB_G0271682
TCONS_00009308	-1.30788	-1.74828	-1.27553	BGIOSGA011846	XP_006649391.2 xyloglucan galactosyltransferase KATAMARI1 homolog
TCONS_00005338	-1.30763	-0.744391	-4.79029	BGIOSGA007446	XP_006646827.1 18.9 kDa heat shock protein-like
TCONS_00025721	-1.30744	-2.35415	1.7168	BGIOSGA037114	XP_006658147.1 thioredoxin-like 1-1, chloroplastic
TCONS_00027229	-1.30697	-0.990241	-1.63461	BGIOSGA027773	XP_006659046.1 phytolongin Phyl1.1-like
TCONS_00014988	-1.30643	-0.220869	-0.398726	BGIOSGA017179	XP_006652856.1PREDICTED: uncharacterized protein LOC102713071
TCONS_00034213	-1.30502	-1.04367	-1.967	BGIOSGA008545	XP_006663774.2 probable inactive serine/threonine-protein kinase bub1
TCONS_00011314	-1.30457	0.277683	0.0762708	BGIOSGA013920	XP_006652013.1 protein FAR1-RELATED SEQUENCE 6-like
TCONS_00034158	-1.30455	-0.852339	-0.846491	BGIOSGA024705	XP_006663741.1 probable serine/threonine-protein kinase WNK8
TCONS_00021346	-1.30413	0.0472213	-1.24565	BGIOSGA014940	XP_006656463.1 protein NRT1/ PTR FAMILY 8.3-like isoform X1
TCONS_00018739	-1.3034	-0.906065	-0.538027	BGIOSGA002238	XP_006655062.1 glutamine--tRNA ligase-like
TCONS_00009448	-1.30337	-3.33731	-1.61669	BGIOSGA006260	XP_004985458.1UDP-glucuronate:xylan alpha-glucuronosyltransferase 2 isoform X1
TCONS_00002581	-1.30293	-3.01235	-1.13112	BGIOSGA005224	XP_012702038.1uncharacterized protein LOC101770868
TCONS_00002106	-1.30278	-0.902416	-1.28383	BGIOSGA017466	XP_006644950.1 protein trichome birefringence-like 28 isoform X1
TCONS_00033053	-1.30261	-3.15436	-1.50926	BGIOSGA019644	XP_012703819.1putative receptor-like protein kinase At3g47110
TCONS_00021462	-1.30256	-2.248	-1.80486	BGIOSGA000752	XP_003568127.1probable xyloglucan glycosyltransferase 7
TCONS_00022812	-1.3022	-0.746315	-1.3054	BGIOSGA020589	XP_006656409.1 probable cadmium/zinc-transporting ATPase HMA1, chloroplastic

TCONS_00035864	-1.30079	-0.64753	-0.938062	BGIOSGA028688	XP_006664495.1 disease resistance protein RPP13-like
TCONS_00025265	-1.30044	1.03515	1.09852	BGIOSGA029105	XP_004978571.1uncharacterized protein LOC101772377
TCONS_00016591	-1.29955	-0.783425	-0.683769	BGIOSGA014373	XP_010240390.1uncharacterized protein LOC100823437
TCONS_00022352	-1.29917	-2.40702	1.4364	BGIOSGA024234	XP_006656133.2 probable protein phosphatase 2C 56
TCONS_00019938	-1.29845	-2.17331	-2.77775	BGIOSGA011508	XP_003567058.2cytochrome P450 93G2
TCONS_00035513	-1.29723	-2.89489	#NA	BGIOSGA034037	XP_015698692.1 alpha-L-arabinofuranosidase 1-like isoform X1
TCONS_00002995	-1.29712	-2.63199	0.353312	BGIOSGA020215	XP_006643859.1 hexokinase-8
TCONS_00019844	-1.29637	0.174419	0.268999	BGIOSGA010624	XP_006654826.1 3-ketoacyl-CoA synthase 11-like
TCONS_00025687	-1.29588	-3.41116	-2.2347	BGIOSGA004585	XP_015695247.1 probable indole-3-acetic acid-amido synthetase GH3.11 isoform X1
TCONS_00020101	-1.29352	-1.55532	-0.481631	BGIOSGA013226	XP_006655783.1 translation factor GUF1 homolog, mitochondrial
TCONS_00015988	-1.29128	-0.240976	-1.45927	BGIOSGA002133	XP_006653472.2 WEB family protein At4g27595, chloroplastic-like
TCONS_00033135	-1.29025	-0.969401	-0.882744	BGIOSGA034980	XP_015697589.1 leucine-rich repeat receptor protein kinase EMS1-like
TCONS_00016567	-1.29012	-0.421945	-1.00305	BGIOSGA014400	XP_006652778.1 plant intracellular Ras-group-related LRR protein 1
TCONS_00028194	-1.28963	-1.36332	-2.54611	BGIOSGA020859	XP_006652866.1 anthocyanidin reductase-like
TCONS_00002588	-1.28947	-1.30898	-0.436253	BGIOSGA023665	XP_015694413.1 cationic peroxidase SPC4-like
TCONS_00011311	-1.28836	-3.83058	-2.49122	BGIOSGA013917	XP_006650878.1PREDICTED: uncharacterized protein YacP
TCONS_00015070	-1.28778	-0.921462	-0.399844	BGIOSGA016844	XP_015691802.1 protein IRX15-LIKE-like
TCONS_00036628	-1.28773	0.848043	-0.477974	BGIOSGA027706	XP_006659092.1 60S acidic ribosomal protein P0
TCONS_00037060	-1.28668	-2.97939	-2.48684	BGIOSGA018917	XP_006663977.1 ABC transporter G family member 11-like
TCONS_00007441	-1.28653	-0.983727	-0.433353	BGIOSGA002134	XP_003570450.1protein STRUBBELIG-RECEPTOR FAMILY 3 isoform X1
TCONS_00022866	-1.28588	-1.50554	-1.6225	BGIOSGA020578	XP_024318988.1disease resistance RPP8-like protein 3
TCONS_00029501	-1.2857	-3.4548	-2.28725	BGIOSGA031275	XP_006661535.1 thioredoxin H4-like
TCONS_00029878	-1.28523	-2.67913	-0.870328	BGIOSGA025669	XP_003576440.1retinol dehydrogenase 13
TCONS_00018839	-1.28439	-6.57548	-2.08374	BGIOSGA032475	XP_010925838.1 BURP domain-containing protein 6-like
TCONS_00031448	-1.2843	-0.22223	-0.486457	BGIOSGA033266	XP_006661948.1 probable serine protease EDA2
TCONS_00011512	-1.28396	-2.01666	-0.169802	BGIOSGA024325	XP_006651010.1 expansin-like A1
TCONS_00030348	-1.28378	-0.605332	-0.788548	BGIOSGA026706	XP_015696785.1 beta-glucosidase 32-like isoform X1
TCONS_00000166	-1.28367	-4.80467	-1.42624	BGIOSGA002736	XP_006643730.1 reticulon-like protein B9
TCONS_00034182	-1.28113	-1.50184	-2.09867	BGIOSGA036750	XP_006662682.2PREDICTED: uncharacterized protein LOC102702048
TCONS_00024525	-1.28092	-0.900049	-2.2508	BGIOSGA017850	XP_006657428.1 amino acid permease 6
TCONS_00005728	-1.28066	0.0636746	-0.226274	BGIOSGA008980	XP_006648483.2PREDICTED: uncharacterized protein LOC102714349, partial
TCONS_00026873	-1.27759	-1.58648	-0.677248	BGIOSGA005718	XP_006660248.1 uncharacterized WD repeat-containing protein C2A9.03
TCONS_00015221	-1.27706	#NA	-3.45389	BGIOSGA017402	XP_006653915.2PREDICTED: uncharacterized protein LOC102717896
TCONS_00012202	-1.27702	#NA	-4.11616	BGIOSGA026400	XP_006650032.2 cytochrome P450 714B2
TCONS_00028103	-1.27675	-2.27697	0.793248	BGIOSGA029700	XP_006662238.1 AT-rich interactive domain-containing protein 4-like
TCONS_00017779	-1.27635	-0.029101	-0.324112	BGIOSGA034421	XP_006654377.1 fructose-bisphosphate aldolase cytoplasmic isozyme
TCONS_00032885	-1.27576	-2.27823	#NA	BGIOSGA034712	XP_015698595.1 probable apyrase 3
TCONS_00012273	-1.27558	-0.0539412	0.2337	BGIOSGA010733	XP_006650080.1 probable nucleolar protein 5-2
TCONS_00028479	-1.27465	-1.59674	-3.22801	BGIOSGA025929	XP_006659711.2 protein DETOXIFICATION 34-like
TCONS_00031613	-1.27423	-3.86512	-1.13904	BGIOSGA011178	XP_006662048.1 expansin-B4
TCONS_00019642	-1.274	0.311802	0.105214	BGIOSGA017715	XP_006654657.1 periodic tryptophan protein 2 homolog
TCONS_00015289	-1.27358	-2.28098	-1.14067	BGIOSGA037594	XP_015691353.1 WEB family protein At2g17940-like
TCONS_00023496	-1.27315	-6.08393	-3.22599	BGIOSGA030205	XP_006657617.1 ABC transporter G family member 11-like
TCONS_00021287	-1.27308	-1.73946	-1.85379	BGIOSGA033580	XP_015693690.1 cysteine-rich receptor-like protein kinase 10
TCONS_00019500	-1.27293	-0.407324	-2.14228	BGIOSGA033176	XP_006655419.2PREDICTED: uncharacterized protein LOC102701452, partial
TCONS_00035043	-1.27289	-1.86681	-0.879356	BGIOSGA037826	XP_015697694.1PREDICTED: uncharacterized protein LOC102717943
TCONS_00006683	-1.27269	-1.2821	-3.46481	BGIOSGA008853	XP_015688702.1 E3 ubiquitin-protein ligase RNF14-like
TCONS_00009533	-1.27181	1.70674	-0.244599	BGIOSGA024937	XP_006649600.2 probable protein phosphatase 2C 29
TCONS_00012034	-1.27171	-1.1266	-0.865748	BGIOSGA010973	XP_015690859.1PREDICTED: uncharacterized protein LOC102710481 isoform X2
TCONS_00031624	-1.27083	-1.23197	-0.732447	BGIOSGA030676	XP_006662061.1 8-amino-7-oxononanoate synthase-like
TCONS_00032083	-1.27046	-1.64748	-0.0553025	BGIOSGA018449	XP_012698264.1probable glucan 1,3-beta-glucosidase A
TCONS_00005621	-1.27033	-1.2687	-1.00406	BGIOSGA030764	XP_015688495.1 MLO-like protein 1
TCONS_00027296	-1.27008	-0.577888	-0.785004	BGIOSGA036674	XP_006659092.1 60S acidic ribosomal protein P0
TCONS_00006958	-1.26963	-2.54899	-0.887424	BGIOSGA009142	XP_015688875.1 fatty-acid-binding protein 2-like isoform X1
TCONS_00007115	-1.26928	-0.648955	0.0292623	BGIOSGA009299	XP_015688763.1 pentatricopeptide repeat-containing protein At1g31430

TCONS_00023268	-1.26916	0.553452	-1.18011	BGIOSGA009208	XP_006658357.1 plasma membrane ATPase 1-like
TCONS_00021341	-1.26844	-0.446385	-0.72145	BGIOSGA023568	XP_006657319.1 putative tRNA (cytidine(32)/guanosine(34)-2'-O)-methyltransferase
TCONS_00000291	-1.268	-1.39848	-1.30906	BGIOSGA019257	NP_001142251.1lipase/lipooxygenase, PLAT/LH2
TCONS_00002506	-1.268	-0.989198	-1.1452	#N/A	#N/A
TCONS_00030162	-1.26631	-0.647962	-3.38255	BGIOSGA025034	XP_006660692.1 tuliposide A-converting enzyme b1, amyloplastic-like
TCONS_00026835	-1.26561	-0.511349	-0.794056	BGIOSGA001540	XP_015696192.1 transcription factor BIM1-like
TCONS_00015060	-1.26541	-0.702698	-0.382861	#N/A	#N/A
TCONS_00024573	-1.26535	-0.365112	-2.91265	#N/A	#N/A
TCONS_00004347	-1.26531	-1.41579	-1.18491	BGIOSGA000867	XP_006659755.1 NADPH-dependent diflavin oxidoreductase 1
TCONS_00025848	-1.26519	1.42882	-0.63991	BGIOSGA014548	XP_004972971.1lysine histidine transporter 2
TCONS_00009796	-1.26364	-0.867451	-0.18077	BGIOSGA009555	XP_015691148.1PREDICTED: uncharacterized protein LOC102720963, partial
TCONS_00016092	-1.26273	-1.43817	-0.564831	BGIOSGA006136	XP_006652410.2 copper methylamine oxidase
TCONS_00028429	-1.26242	-2.55766	-2.5508	BGIOSGA010524	XP_006660360.1 cytochrome P450 78A9-like
TCONS_00000078	-1.26226	-0.774686	-1.6785	BGIOSGA002652	XP_006643675.1 probable receptor-like protein kinase At1g67000
TCONS_00010925	-1.26203	-0.654657	-1.20857	BGIOSGA013493	XP_014752663.1transcription factor BHLH148
TCONS_00018251	-1.26124	-0.738721	-0.786269	BGIOSGA004259	XP_015692685.1 ABC transporter B family member 5-like
TCONS_00027135	-1.26104	-0.929631	0.836921	BGIOSGA030826	XP_006650246.1 auxin-repressed 12.5 kDa protein
TCONS_00017617	-1.26095	-3.21932	0.718201	BGIOSGA001545	XP_002465233.1universal stress protein PHOS34
TCONS_00005619	-1.26058	0.0597679	0.685205	BGIOSGA020720	XP_006647022.1 probable copper-transporting ATPase HMA5
TCONS_00022696	-1.26041	-1.7107	-1.3716	BGIOSGA020745	XP_006656328.1 TBC1 domain family member 5 homolog B-like
TCONS_00018722	-1.26005	-1.23494	-0.774712	BGIOSGA034766	XP_006654102.1 auxin-responsive protein IAA15
TCONS_00004265	-1.25979	-4.31569	-2.59264	BGIOSGA005375	XP_021311299.1transcription factor bHLH84
TCONS_00001522	-1.25927	-4.31634	-0.915853	BGIOSGA004953	XP_015689492.1 mannan endo-1,4-beta-mannosidase 1
TCONS_00039755	-1.25919	1.82813	0.161069	#N/A	XP_013443002.1senescence-associated protein
TCONS_00010878	-1.25786	-0.617679	-0.671419	BGIOSGA013065	XP_006650533.1 pentatricopeptide repeat-containing protein At3g49730
TCONS_00021735	-1.25781	0.13967	-0.55027	BGIOSGA009206	XP_017625336.1 60S ribosomal protein L39
TCONS_00024549	-1.25687	-0.795792	-0.336811	BGIOSGA030536	XP_010228421.1rust resistance kinase Lr10
TCONS_00027801	-1.25636	-0.640075	-1.36404	BGIOSGA027960	XP_006661582.1 basic blue protein-like
TCONS_00010009	-1.25594	-2.56556	-2.04034	BGIOSGA012425	XP_015691298.1 probable inactive receptor kinase At5g58300
TCONS_00030642	-1.25538	-1.16771	-0.926246	BGIOSGA032349	XP_015696920.1 putative disease resistance protein RGA3
TCONS_00035566	-1.25471	-3.37734	-1.93894	BGIOSGA036469	NP_001278782.1jasmonate-induced protein-like
TCONS_00031237	-1.25431	-0.524824	-1.89292	BGIOSGA011454	XP_015697499.1 putative transporter arsB
TCONS_00002461	-1.25427	-1.06119	-0.750612	BGIOSGA005104	XP_004970117.1protein SPA, chloroplastic
TCONS_00023215	-1.25388	-2.92359	-0.829061	BGIOSGA034552	XP_003577876.1protein ZINC INDUCED FACILITATOR-LIKE 1 isoform X2
TCONS_00003869	-1.2526	0.0977829	-4.25635	BGIOSGA001346	XP_015691251.1 myosin-2 heavy chain-like
TCONS_00013339	-1.25253	-1.99114	-2.1469	BGIOSGA024718	XP_008681053.1plant Tudor-like RNA-binding protein isoform X1
TCONS_00007776	-1.25201	-0.409918	-0.00987964	BGIOSGA000780	XP_015689066.1 protein tesmin/TSO1-like CXC 5 isoform X1
TCONS_00025381	-1.25133	3.05554	0.264061	BGIOSGA021329	XP_006657865.2 protein TIME FOR COFFEE
TCONS_00035010	-1.2502	-2.94882	-1.45069	BGIOSGA033847	NP_001159208.1virulent strain associated lipoprotein
TCONS_00006596	-1.24923	-1.10966	-2.06133	BGIOSGA000547	XP_015688686.1 scarecrow-like protein 6
TCONS_00015369	-1.24852	-0.704771	-1.57361	BGIOSGA022542	XP_012702710.1uncharacterized protein LOC101768343
TCONS_00022566	-1.24836	-1.34749	0.710163	BGIOSGA011643	XP_015694255.1 ribose-phosphate pyrophosphokinase 2, chloroplastic
TCONS_00036529	-1.24831	-0.767558	-1.50713	BGIOSGA036798	XP_006662647.2 probable phytol kinase 2, chloroplastic
TCONS_00011356	-1.24768	0.102344	-1.51098	BGIOSGA013969	XP_004981004.1putative gamma-glutamylcyclotransferase At3g02910
TCONS_00027514	-1.24626	-3.25558	-2.43823	BGIOSGA006596	XP_002445184.1putative F-box/LRR-repeat protein 23
TCONS_00012712	-1.24575	-0.0728712	-1.80363	BGIOSGA011300	XP_003562286.1zinc finger protein 1
TCONS_00034331	-1.24548	-2.38716	-1.96202	BGIOSGA036312	XP_010025545.1 histone H3.2
TCONS_00005255	-1.24462	-2.3881	#NA	BGIOSGA007368	XP_012837152.1 peptidyl-prolyl cis-trans isomerase 1
TCONS_00036637	-1.24461	-3.57958	-3.76426	BGIOSGA012782	XP_006662724.1 agmatine coumaroyltransferase-2-like
TCONS_00027456	-1.24447	-1.33626	-1.77038	BGIOSGA019942	XP_006659885.2 transcription factor TGA6-like
TCONS_00002704	-1.24414	-2.72402	-1.75607	BGIOSGA002733	XP_006645443.1 glycerophosphodiester phosphodiesterase protein kinase domain-containing GDPDL2-like
TCONS_00004745	-1.2441	-1.84326	-1.18056	BGIOSGA032900	XP_006645036.1 glycerol-3-phosphate 2-O-acyltransferase 6-like
TCONS_00006713	-1.24396	-0.6128	-1.41434	BGIOSGA008882	XP_006647754.1PREDICTED: uncharacterized protein LOC102708688
TCONS_00001544	-1.24336	-0.121721	0.341181	BGIOSGA038169	XP_006644497.1 putative receptor protein kinase ZmPK1
TCONS_00008252	-1.24307	-2.69255	-3.08	BGIOSGA035404	XP_002452347.1protein NRT1/ PTR FAMILY 6.4
TCONS_00009475	-1.24259	-1.42458	-1.78525	BGIOSGA024273	XP_006649538.1 ras-related protein RABH1e-like
TCONS_00020994	-1.24241	-0.774987	-1.59975	BGIOSGA028488	XP_006656219.1 phospholipase D alpha 2-like
TCONS_00002455	-1.24184	0.533604	-0.401277	BGIOSGA008657	XP_003564986.1auxin response factor 4

transcriptome

TCONS_00024265	-1.24149	-3.60347	-1.39927	BGIOSGA019808	XP_006658911.1 methyltransferase-like protein 13
TCONS_00009716	-1.24124	#NA	-2.97279	BGIOSGA033832	XP_006649781.2 probable phospholipase A2 homolog 2 isoform X2
TCONS_00017894	-1.24032	-1.07973	-0.969161	BGIOSGA027558	XP_015693200.1 transcription factor HBP-1b(c38)-like isoform X6
TCONS_00022934	-1.23823	-1.39529	1.34151	BGIOSGA020511	XP_015694234.1 thiosulfate sulfurtransferase 16, chloroplastic-like
TCONS_00031727	-1.23749	-2.90141	-2.47128	BGIOSGA036981	XP_006661601.1 protein NRT1/ PTR FAMILY 8.3-like
TCONS_00006900	-1.23629	-2.27067	-0.840077	#N/A	XP_006647921.1PREDICTED: uncharacterized protein LOC102710376
TCONS_00030199	-1.23618	-0.53575	-0.759453	BGIOSGA014453	XP_006661334.1 E3 ubiquitin-protein ligase RING1-like
TCONS_00024357	-1.23602	-0.889593	-0.570589	BGIOSGA010794	XP_006658136.1 serine/threonine-protein kinase AtPK2/AtPK19-like
TCONS_00011464	-1.23579	0.186743	0.286426	BGIOSGA011424	XP_002465969.1heavy metal-associated isoprenylated plant protein 30
TCONS_00003322	-1.23514	-0.8001	-1.13644	BGIOSGA036673	XP_008657261.1protein ZINC INDUCED FACILITATOR-LIKE 1
TCONS_00035920	-1.23507	-1.81415	0.333506	BGIOSGA005528	XP_006664005.1 probable tyrosine-protein phosphatase At1g05000
TCONS_00006780	-1.23507	-1.39911	-1.40346	BGIOSGA021835	XP_015693150.1 auxin-responsive protein IAA8-like isoform X2
TCONS_00016833	-1.23497	-0.229305	0.40365	BGIOSGA014132	XP_003579457.1probable carboxylesterase Os04g0669600
TCONS_00004067	-1.23369	-1.3467	-0.255739	BGIOSGA032833	XP_006644432.1 molybdate transporter 2
TCONS_00009036	-1.23351	0.163742	0.177588	BGIOSGA005371	XP_006649166.2 diphthamide biosynthesis protein 1
TCONS_00011059	-1.23289	-1.21587	-0.385633	BGIOSGA013658	XP_006651872.1PREDICTED: uncharacterized protein LOC102708513
TCONS_00016377	-1.23254	1.24159	0.0558471	BGIOSGA014589	XP_004976442.1probable plastid-lipid-associated protein 11
TCONS_00012820	-1.23242	-1.81746	-1.99509	BGIOSGA010146	XP_006651633.1 protein ROOT PRIMORDIUM DEFECTIVE 1-like
TCONS_00020312	-1.23206	-2.08095	-3.99599	BGIOSGA027060	XP_006655895.1 anthocyanin regulatory C1 protein-like
TCONS_00011058	-1.23151	2.82525	0.909537	BGIOSGA001014	XP_006656565.1 pentatricopeptide repeat-containing protein At1g08070, chloroplastic-like
TCONS_00025660	-1.23143	2.71904	0.563226	#N/A	#N/A
TCONS_00017009	-1.23112	-0.404062	-0.413374	BGIOSGA015659	XP_004960492.1uncharacterized protein LOC101760917 isoform X1
TCONS_00034269	-1.23091	-0.596852	-0.593884	BGIOSGA001660	XP_006660824.1 acyltransferase-like protein At1g54570, chloroplastic
TCONS_00037560	-1.23063	-4.40468	-2.99956	BGIOSGA033877	XP_006664764.1 23.2 kDa heat shock protein-like
TCONS_00024454	-1.22977	-0.820808	#NA	BGIOSGA002245	XP_006658215.1 probable WRKY transcription factor 57
TCONS_00007252	-1.22964	-0.236012	0.904854	BGIOSGA023625	XP_006648244.1 serine/threonine-protein kinase STY46-like
TCONS_00016172	-1.22957	-0.442544	-0.569238	BGIOSGA029852	XP_004976089.1(+)-neomenthol dehydrogenase
TCONS_00004252	-1.22933	-3.82137	-2.68088	BGIOSGA011837	XP_006644576.1 myb-related protein Hv33-like
TCONS_00027077	-1.22876	0.789347	-0.682304	BGIOSGA024205	XP_015695711.1 thaumatin-like protein 1
TCONS_00027070	-1.22866	-1.59982	-1.00448	BGIOSGA029066	XP_006659657.1 probable 6-phosphogluconolactonase 3, chloroplastic
TCONS_00005344	-1.22863	-1.23728	-0.00453631	BGIOSGA018630	XP_023157549.1uncharacterized protein LOC103646630
TCONS_00036570	-1.22838	-0.533055	-0.889689	BGIOSGA034604	XP_006663163.2PREDICTED: uncharacterized protein LOC102714866
TCONS_00036286	-1.22803	-1.82301	-2.42108	BGIOSGA038054	XP_015698505.1 factor of DNA methylation 5-like isoform X5
TCONS_00032183	-1.22773	-0.246142	-0.342013	BGIOSGA031953	XP_006661763.1PREDICTED: uncharacterized protein LOC102712663
TCONS_00015068	-1.2276	-1.39395	-1.63691	BGIOSGA028838	XP_015691597.1 floral homeotic protein APETALA 2-like
TCONS_00021963	-1.22745	-1.99366	-1.78509	BGIOSGA015677	XP_006655959.1 anthranilate O-methyltransferase 3-like
TCONS_00017622	-1.22724	-1.40897	-1.22374	BGIOSGA019684	XP_010231509.1exonuclease mut-7 homolog isoform X2
TCONS_00007124	-1.22701	-0.71536	-1.03486	BGIOSGA015886	XP_015688768.1 ethylene receptor 3
TCONS_00010064	-1.22694	-1.39795	-0.596052	BGIOSGA012608	XP_015689890.1 lysine-specific demethylase JMJ25
TCONS_00030155	-1.22651	-0.222252	-1.09226	BGIOSGA009762	XP_015696756.1 nuclear pore complex protein NUP58
TCONS_00016521	-1.22615	-1.97737	-0.873195	BGIOSGA008356	XP_015692065.1PREDICTED: uncharacterized protein LOC102700137
TCONS_00018583	-1.22613	0.718927	-1.20339	BGIOSGA007670	XP_006654970.1 WRKY transcription factor 6-like
TCONS_00030124	-1.22597	-1.96999	-2.49657	BGIOSGA026880	NP_001147572.1flavonoid 3-monooxygenase
TCONS_00011716	-1.22564	-1.37261	-1.13234	BGIOSGA011294	XP_003558608.3non-specific lipid-transfer protein-like protein At5g64080
TCONS_00003787	-1.22547	-0.734385	-1.83828	BGIOSGA035293	XP_004968917.1methylesterase 7
TCONS_00027305	-1.22499	-1.02013	-0.383269	BGIOSGA014281	XP_019709040.1 mediator of RNA polymerase II transcription subunit 21-like
TCONS_00001941	-1.22499	-2.16706	-1.35416	BGIOSGA023866	XP_022682731.1G-type lectin S-receptor-like serine/threonine-protein kinase B120
TCONS_00025395	-1.22497	0.00322656	-1.53967	BGIOSGA024500	XP_015694893.1 L-type lectin-domain containing receptor kinase IV.1-like
TCONS_00015002	-1.22469	-0.000354087	-0.464105	BGIOSGA021995	XP_006653797.2 protein SDA1 homolog
TCONS_00012944	-1.22448	-2.0201	-0.522944	BGIOSGA006044	XP_015690017.1 protein argonaute 12-like
TCONS_00032618	-1.22368	-0.67748	-2.08596	BGIOSGA032289	XP_012698474.1homeobox protein BEL1 homolog
TCONS_00012568	-1.22246	-2.2323	-0.265492	BGIOSGA008183	XP_006648628.1PREDICTED: uncharacterized protein LOC102707377

TCONS_00004928	-1.22175	-1.34571	-0.903426	BGIOSGA000293	XP_004970978.1TBC1 domain family member 13
TCONS_00036155	-1.22138	-1.12379	-0.704409	BGIOSGA037514	XP_003575781.1uncharacterized protein LOC100843284
TCONS_00018784	-1.22056	-1.81197	-1.82315	BGIOSGA029609	XP_006654140.1 NDR1/HIN1-Like protein 3-like
TCONS_00016148	-1.22045	-0.610337	-0.513326	BGIOSGA004987	XP_015692101.1 CMP-sialic acid transporter 4
TCONS_00027295	-1.21946	-0.949767	-1.3244	BGIOSGA027707	XP_006659796.1 2-succinylbenzoate--CoA ligase, chloroplastic/peroxisomal
TCONS_00032588	-1.21894	0.122727	-0.482114	BGIOSGA025777	XP_004983871.1arogenate dehydratase 3, chloroplastic
TCONS_00012100	-1.21843	-0.905106	-1.42067	BGIOSGA019641	XP_004984898.1uncharacterized protein LOC101756085
TCONS_00002702	-1.21775	-1.86759	-2.16855	BGIOSGA002514	XP_006643674.1 glycerophosphodiester phosphodiesterase protein kinase domain-containing GDPDL2-like
TCONS_00013781	-1.21686	-2.46289	-1.90258	BGIOSGA029433	XP_015695813.1 disease resistance protein RPM1-like
TCONS_00035406	-1.21613	0.384116	0.118233	BGIOSGA016943	XP_006663131.1 RHOMBOID-like protein 2
TCONS_00023794	-1.21608	-1.06159	-0.330395	BGIOSGA038018	XP_006657773.2 septum-promoting GTP-binding protein 1
TCONS_00018501	-1.21593	-1.503	-1.07679	BGIOSGA023259	XP_006654923.1 beta-hexosaminidase 1
TCONS_00001631	-1.21585	-2.28415	-1.14029	BGIOSGA004264	XP_006644567.1 transcription initiation factor TFIID subunit 1 isoform X2
TCONS_00026340	-1.21396	-0.802474	-0.769242	BGIOSGA007832	XP_015695941.1 putative chloride channel-like protein CLC-g
TCONS_00031003	-1.21389	-0.417204	-1.64415	BGIOSGA032811	XP_021304334.1uncharacterized protein LOC8155683
TCONS_00016160	-1.21388	-0.0256536	-0.377135	BGIOSGA014819	XP_006650351.1 7-methylguanosine phosphate-specific 5'-nucleotidase A
TCONS_00007178	-1.21354	-0.0769993	-1.35224	BGIOSGA007319	XP_006648191.1 protein disulfide isomerase-like 1-4
TCONS_00022406	-1.21348	-9.5481	-4.61026	BGIOSGA007839	XP_006656153.1 protein HEADING DATE 3A-like isoform X1
TCONS_00001918	-1.21337	-1.75581	-0.0955063	BGIOSGA004554	XP_006646387.1 aspartic proteinase-like protein 2
TCONS_00003574	-1.21334	-1.85847	-0.555433	BGIOSGA021669	XP_015688299.1 putative 12-oxophytodienoate reductase 10
TCONS_00029682	-1.21311	0.30137	-0.144442	BGIOSGA026593	XP_006660471.1 60S ribosomal protein L17
TCONS_00024118	-1.21191	-0.793442	-1.10918	BGIOSGA026153	XP_015694949.1 polcalcin Phl p 7-like
TCONS_00032121	-1.21169	-0.867729	-1.04249	BGIOSGA026567	XP_006661729.1 probable calcium-binding protein CML8 isoform X2
TCONS_00025240	-1.21107	-0.622473	-0.225429	BGIOSGA035543	XP_006663641.1 disease resistance protein RPM1-like
TCONS_00010895	-1.21103	-0.997302	-2.02281	BGIOSGA025928	XP_015690056.1 BEL1-like homeodomain protein 4
TCONS_00014307	-1.21082	-0.660192	-1.32759	BGIOSGA000812	XP_015692249.1 auxin response factor 9
TCONS_00010029	-1.2108	-1.58008	-2.03968	BGIOSGA023572	XP_015691231.1 putative potassium transporter 8
TCONS_00032262	-1.21021	-1.02332	-0.312997	BGIOSGA037671	XP_014617947.1TATA-box-binding protein isoform X4
TCONS_00009147	-1.21001	-2.79434	-0.38661	BGIOSGA030324	XP_006649239.1 UPF0548 protein At2g17695 isoform X2
TCONS_00036441	-1.20987	-2.07994	-1.86985	BGIOSGA004251	XP_022681241.1probable LRR receptor-like serine/threonine-protein kinase At3g47570
TCONS_00029172	-1.20956	-0.369401	-1.51489	BGIOSGA024334	XP_006661320.1 probable carboxylesterase 2
TCONS_00014294	-1.20942	0.285967	-0.869282	BGIOSGA003960	XP_015692345.1 amino-acid permease BAT1 homolog isoform X2
TCONS_00028069	-1.20942	-2.46126	-3.2843	BGIOSGA029762	XP_006659441.1 ATP-dependent 6-phosphofructokinase 5, chloroplastic-like
TCONS_00018781	-1.20903	-0.411125	-1.4108	BGIOSGA034776	XP_015692900.1 IQ domain-containing protein IQM5-like
TCONS_00023393	-1.20884	0.398245	0.0985456	BGIOSGA016863	XP_024313537.15'-3' exoribonuclease 4-like
TCONS_00022119	-1.20869	-0.0955709	-0.804963	BGIOSGA021386	XP_006656026.1 39S ribosomal protein L46, mitochondrial
TCONS_00014009	-1.20844	0.142588	-0.251725	BGIOSGA016317	XP_015691740.1 40S ribosomal protein S27-like
TCONS_00035859	-1.20799	-1.46306	-0.965914	BGIOSGA036821	XP_004978420.1probable LRR receptor-like serine/threonine-protein kinase At1g67720
TCONS_00023640	-1.20725	-1.65428	-3.26069	BGIOSGA033341	XP_015695308.1 probable glutathione S-transferase GSTU1
TCONS_00015506	-1.20705	-1.6479	-1.51413	BGIOSGA015478	XP_015691984.1 probable cleavage and polyadenylation specificity factor subunit 1 isoform X3
TCONS_00014913	-1.20697	1.35068	-1.99509	BGIOSGA037498	XP_004976775.2vacuolar-sorting receptor 7
TCONS_00010705	-1.20619	-1.99182	-1.07424	BGIOSGA013334	XP_015690574.1 ankyrin repeat and protein kinase domain-containing protein 1-like isoform X1
TCONS_00006859	-1.20515	-0.804933	-2.17909	BGIOSGA009039	XP_006647878.1 photosynthetic NDH subunit of subcomplex B 4, chloroplastic
TCONS_00027482	-1.20504	-0.713154	-0.698571	BGIOSGA035456	XP_004972692.1CRS2-associated factor 2, mitochondrial
TCONS_00034219	-1.20459	-1.79095	-0.759569	BGIOSGA031869	XP_015698533.1PREDICTED: uncharacterized protein LOC102714873
TCONS_00009085	-1.20166	0.524993	#NA	BGIOSGA039322	XP_008811187.1PREDICTED: uncharacterized protein LOC103722404 isoform X2
TCONS_00026460	-1.20155	0.110541	-1.61355	BGIOSGA030488	XP_006659322.1 protein SAR DEFICIENT 1-like
TCONS_00032440	-1.20072	-2.42761	-1.2585	BGIOSGA016743	XP_004982832.1E3 ubiquitin-protein ligase MARCH11 isoform X2
TCONS_00000331	-1.20045	-1.28987	-0.224602	BGIOSGA023883	XP_004968493.1uncharacterized protein LOC101754097
TCONS_00008965	-1.19916	0.147355	-0.23763	BGIOSGA005439	XP_015688839.1 DEAD-box ATP-dependent RNA helicase 1
TCONS_00016846	-1.19832	-2.4945	-1.32599	BGIOSGA007604	XP_015691612.1 auxin response factor 12
TCONS_00032616	-1.19772	-0.439696	-0.649235	BGIOSGA030546	XP_015697436.1 probable receptor-like protein kinase At5g24010

transcriptome

TCONS_00032599	-1.1974	-3.07154	-1.91848	BGIOSGA005165	XP_006661977.1 glutathione S-transferase U17-like
TCONS_00035325	-1.19737	-0.469007	-0.685006	BGIOSGA004118	XP_006655331.1 pentatricopeptide repeat-containing protein At4g14050, mitochondrial
TCONS_00014340	-1.19722	1.10826	-1.37026	BGIOSGA016502	XP_021318037.1zeaxanthin epoxidase, chloroplastic isoform X2
TCONS_00037549	-1.19696	-0.257119	-1.10333	BGIOSGA015452	XP_015698821.1PREDICTED: uncharacterized protein LOC102704661
TCONS_00005042	-1.19676	-0.853835	-0.233907	BGIOSGA001423	XP_015699238.1 esterase PIR7B-like
TCONS_00019740	-1.19655	#NA	-0.693878	BGIOSGA004244	XP_006654744.1 reticulon-like protein B9
TCONS_00033237	-1.19601	-0.499367	-1.13659	BGIOSGA035071	XP_015698017.1 ankyrin repeat protein SKIP35-like
TCONS_00036580	-1.19595	-2.10278	-1.69538	BGIOSGA036742	XP_006663761.1 protein LURP-one-related 5-like
TCONS_00025499	-1.19589	-2.5179	-2.11057	BGIOSGA000502	XP_004971717.1aspartic proteinase CDR1
TCONS_00031558	-1.19559	-0.848417	-1.0272	BGIOSGA025905	XP_004983798.1protein PLASTID MOVEMENT IMPAIRED 1
TCONS_00031701	-1.19554	0.751034	-0.218392	BGIOSGA025073	XP_003573755.1protein PHLOEM PROTEIN 2-LIKE A10
TCONS_00005995	-1.19502	-1.16161	-0.8172	BGIOSGA020647	XP_006647242.2PREDICTED: uncharacterized protein At4g22758
TCONS_00030271	-1.19495	-1.98429	-0.845447	BGIOSGA026136	XP_015696852.1 AT-hook motif nuclear-localized protein 10-like
TCONS_00005108	-1.19489	-2.10413	0.208843	BGIOSGA005178	XP_015695873.1 transcription factor bHLH100-like
TCONS_00013503	-1.19434	-1.33438	-1.10758	BGIOSGA036525	XP_006652004.1 serine/threonine-protein kinase pakF-like
TCONS_00011816	-1.19429	-0.350248	-1.20585	BGIOSGA030715	XP_015689822.1 alpha,alpha-trehalose-phosphate synthase [UDP-forming] 6-like
TCONS_00012795	-1.19407	-0.408095	-1.70442	BGIOSGA032532	XP_004982339.1homeobox-leucine zipper protein HOX32
TCONS_00028088	-1.19389	-5.34777	-2.34592	BGIOSGA017855	XP_006659458.1 receptor-like protein kinase
TCONS_00025976	-1.19349	-1.78398	0.620385	BGIOSGA025176	XP_008811288.1 putative deoxyribonuclease TATDN1 isoform X2
TCONS_00029639	-1.19327	-3.17832	-3.44288	BGIOSGA031408	XP_010942234.1 zinc finger protein CONSTANS-LIKE 2-like
TCONS_00027228	-1.19325	-1.28612	-2.16954	BGIOSGA027774	XP_006659045.1 putative methylesterase 14, chloroplastic
TCONS_00013579	-1.19266	2.35248	1.15437	BGIOSGA009396	XP_006652050.1 pollen-specific leucine-rich repeat extensin-like protein 3
TCONS_00000687	-1.19242	-0.648021	0.0490259	BGIOSGA003277	XP_015688261.1 procyclic form-specific polypeptide B-alpha-like
TCONS_00011748	-1.19233	-0.137735	-0.534494	BGIOSGA010240	XP_006649590.1 cyclin-D5-3 isoform X1
TCONS_00019354	-1.19191	-2.39507	-1.86298	BGIOSGA031270	XP_003566212.1protein NRT1/ PTR FAMILY 5.10 isoform X1
TCONS_00006657	-1.19188	-0.300581	-1.70555	BGIOSGA008826	XP_006648943.1PREDICTED: uncharacterized protein LOC102719840
TCONS_00029000	-1.19184	0.0331551	-1.362	BGIOSGA012046	XP_006660626.1 sugar transport protein 7
TCONS_00002422	-1.19131	-0.649217	-1.38503	BGIOSGA017264	XP_006645243.2PREDICTED: uncharacterized protein At4g15970-like
TCONS_00027653	-1.19087	-1.78727	-2.445	BGIOSGA034537	XP_006650308.1 protein DMR6-LIKE OXYGENASE 2-like
TCONS_00029656	-1.19078	-1.66195	-2.18925	#N/A	#N/A
TCONS_00013854	-1.19063	-2.58931	-2.89173	BGIOSGA002791	XP_006643761.1 very-long-chain (3R)-3-hydroxyacyl-CoA dehydratase PASTICCINO 2A
TCONS_00025864	-1.19034	-2.52491	-1.70937	BGIOSGA009613	XP_015695714.1 probable beta-1,3-galactosyltransferase 20
TCONS_00015529	-1.19015	-3.11011	-2.70984	BGIOSGA021442	XP_002448065.2UDP-glycosyltransferase 92A1
TCONS_00033393	-1.18971	-1.42334	-1.47002	BGIOSGA015344	XP_002451211.1tropinone reductase homolog At5g06060
TCONS_00031096	-1.18934	-3.12219	-2.29912	BGIOSGA032914	XP_015697374.1 acylamino-acid-releasing enzyme-like
TCONS_00005641	-1.18922	-2.78934	-3.12716	BGIOSGA007756	XP_004951515.1(6-4)DNA photolyase
TCONS_00009188	-1.18842	-0.794909	-0.72433	BGIOSGA029410	XP_006649271.1 F-box protein PP2-A13-like
TCONS_00000468	-1.18827	0.550498	-2.29943	BGIOSGA025262	XP_015694473.1 S-type anion channel SLAH3-like isoform X2
TCONS_00008542	-1.18756	-0.545611	-1.05105	BGIOSGA014569	XP_006647648.1 pre-mRNA-splicing ATP-dependent RNA helicase prp28-like
TCONS_00008164	-1.18706	-0.391162	-0.895279	BGIOSGA026164	XP_006647363.1 serine/threonine-protein kinase SAPK6
TCONS_00025717	-1.18604	-1.86917	-1.31933	BGIOSGA017784	XP_015695159.1 probable inactive receptor kinase At5g58300
TCONS_00007161	-1.18545	-3.29632	-0.791188	BGIOSGA010570	XP_006648176.2 sucrose synthase 6
TCONS_00014337	-1.18538	-1.54176	-1.59365	BGIOSGA022765	XP_015691778.1 aspartic proteinase nepenthesin-1-like
TCONS_00036764	-1.18511	-2.33203	-1.61658	BGIOSGA036550	XP_021302175.1uncharacterized protein LOC8076284
TCONS_00025226	-1.18424	-0.868305	-5.46726	BGIOSGA032229	XP_024310766.1bisdemethoxycurcumin synthase
TCONS_00003364	-1.184	-1.19751	-0.6352	BGIOSGA001769	XP_022682683.1S-adenosylmethionine synthase 3
TCONS_00018894	-1.18359	-0.915678	-1.29639	BGIOSGA024453	XP_006659155.1 topless-related protein 1-like
TCONS_00036518	-1.18357	-1.84161	-1.5009	BGIOSGA009459	XP_004978420.1probable LRR receptor-like serine/threonine-protein kinase At1g67720
TCONS_00004821	-1.18276	-5.16554	-1.689	BGIOSGA019907	XP_006645114.1PREDICTED: uncharacterized protein LOC102717020
TCONS_00034435	-1.18239	-0.464592	-1.60102	BGIOSGA034374	XP_004978893.1DNA polymerase delta catalytic subunit
TCONS_00030716	-1.18228	-2.17764	-1.4071	BGIOSGA013634	XP_006661867.1 homeobox-leucine zipper protein HOX9
TCONS_00000040	-1.18216	-1.15965	-1.00285	BGIOSGA008490	XP_006649199.2PREDICTED: uncharacterized protein LOC102705229, partial
TCONS_00030435	-1.18204	-1.03877	-0.21982	BGIOSGA019255	XP_006660896.1 protein NUCLEAR FUSION DEFECTIVE 4-like

TCONS_00022681	-1.1812	-1.48995	-1.42938	BGIOSGA003756	XP_004965689.1zinc finger protein CONSTANS-LIKE 5
TCONS_00016028	-1.18117	-1.76894	-1.60013	BGIOSGA006930	XP_006652380.1 vacuolar iron transporter 1.1
TCONS_00008281	-1.18063	-2.51049	-2.74223	BGIOSGA033878	XP_003559503.1UDP-glycosyltransferase 91D1
TCONS_00014497	-1.17952	-2.36556	-2.15957	BGIOSGA033575	XP_008678533.1amino acid permease 4
TCONS_00036567	-1.17945	-2.85147	-3.17488	BGIOSGA000267	XP_015698058.1 mannan endo-1,4-beta-mannosidase 8-like
TCONS_00014831	-1.17941	-0.0458613	-0.921223	BGIOSGA008838	XP_006652710.1 probable purine permease 11
TCONS_00016632	-1.17868	0.277237	0.352928	BGIOSGA018383	XP_006652837.2 probable receptor-like protein kinase At1g30570
TCONS_00021535	-1.17821	-1.11512	-1.16938	BGIOSGA022015	XP_006655741.1PREDICTED: uncharacterized protein LOC102714192
TCONS_00023354	-1.17808	-1.92586	-1.08658	BGIOSGA025396	XP_015694488.1 pentatricopeptide repeat-containing protein At1g80270, mitochondrial-like
TCONS_00013192	-1.17663	0.167264	-1.07712	BGIOSGA008563	XP_015690283.1PREDICTED: uncharacterized protein LOC102701624
TCONS_00006592	-1.17639	-0.395811	0.88722	BGIOSGA016897	XP_006647639.1 vesicle-associated protein 1-3-like isoform X1
TCONS_00020135	-1.17619	0.670828	-0.852473	BGIOSGA022336	XP_006655797.1 ubiquinone biosynthesis O-methyltransferase, mitochondrial
TCONS_00002540	-1.17577	-0.773913	-0.447411	BGIOSGA022767	XP_006646688.2 putative amidohydrolase YtcJ
TCONS_00002104	-1.17487	-0.408836	-0.855747	BGIOSGA009738	XP_006644945.1 malate dehydrogenase, chloroplastic-like
TCONS_00024363	-1.17386	#NA	-1.51148	BGIOSGA007813	XP_004959896.1protein PYRICULARIA ORYZAE RESISTANCE 21-like
TCONS_00026069	-1.17358	-2.25846	-1.59591	BGIOSGA028154	XP_010237549.1uncharacterized protein LOC100825130
TCONS_00024334	-1.17326	-1.9184	-2.73726	BGIOSGA004761	XP_004958670.1UDP-glucuronic acid decarboxylase 2
TCONS_00010000	-1.17316	0.738234	-0.546745	BGIOSGA005545	XP_015690746.1 probable leucine-rich repeat receptor-like protein kinase At5g49770
TCONS_00013811	-1.17309	0.898954	0.0894168	BGIOSGA031261	XP_019703496.1 probable E3 ubiquitin-protein ligase HERC2 isoform X2
TCONS_00024197	-1.17286	-0.674394	-0.375284	BGIOSGA026235	XP_006658026.1 probable protein arginine N-methyltransferase 3
TCONS_00005250	-1.17281	0.372846	-0.182768	BGIOSGA007942	XP_015688422.1PREDICTED: uncharacterized protein LOC102700965
TCONS_00015037	-1.17278	-2.60941	-1.57951	BGIOSGA006743	XP_006652895.1PREDICTED: uncharacterized protein LOC102702651
TCONS_00004970	-1.17147	-1.89281	-1.86115	BGIOSGA034018	XP_006645228.2 zinc finger CCCH domain-containing protein 12
TCONS_00037091	-1.17128	-0.500879	-0.954331	BGIOSGA011389	XP_006663996.1 beta-galactosidase 15 isoform X1
TCONS_00004707	-1.16962	#NA	-3.89474	BGIOSGA032102	XP_006644994.1 laccase-12/13-like
TCONS_00020589	-1.16912	0.771849	0.0923152	BGIOSGA019532	XP_015693343.1 pentatricopeptide repeat-containing protein At4g38150-like
TCONS_00019474	-1.16854	-0.189222	-0.866995	BGIOSGA012992	XP_006654536.1 exocyst complex component EXO70B1
TCONS_00025725	-1.16831	-1.08052	-1.26329	BGIOSGA007162	XP_006658148.1 myb family transcription factor APL-like isoform X2
TCONS_00037508	-1.16769	-1.1075	-0.660836	BGIOSGA010178	XP_004982353.1F-box/LRR-repeat protein At3g48880
TCONS_00000610	-1.16739	-3.19151	-0.966631	BGIOSGA003194	XP_015692846.1PREDICTED: uncharacterized protein LOC102719442
TCONS_00005106	-1.1673	-4.20727	-1.99007	BGIOSGA005183	XP_015688054.1 beta-galactosidase isoform X1
TCONS_00034261	-1.16704	-0.724927	0.248101	BGIOSGA034532	XP_003576573.1putative gamma-glutamylcyclotransferase At3g02910
TCONS_00003597	-1.16691	-3.70878	-1.65442	BGIOSGA011557	XP_015693322.1 peroxidase 24-like
TCONS_00032805	-1.16665	-2.55109	-2.21547	BGIOSGA003414	XP_003577897.2protein DWARF 53
TCONS_00020288	-1.16633	-0.694016	-0.249146	#N/A	#N/A
TCONS_00005438	-1.16562	-1.43476	-2.8483	BGIOSGA032811	XP_006646887.2 probable protein phosphatase 2C 10
TCONS_00006112	-1.16552	-0.397235	-2.44398	BGIOSGA003427	XP_015688518.1PREDICTED: uncharacterized protein LOC102710934
TCONS_00035007	-1.16518	-2.51831	-2.53318	BGIOSGA007944	XP_006662995.1 heparan-alpha-glucosaminide N-acetyltransferase-like isoform X1
TCONS_00033767	-1.16501	1.13252	-0.626726	BGIOSGA011244	XP_006663572.1 notchless protein homolog
TCONS_00002715	-1.16469	-1.44722	-2.04502	BGIOSGA002639	XP_006643727.1 glycerophosphodiester phosphodiesterase protein kinase domain-containing GDPDL2-like isoform X2
TCONS_00021595	-1.16462	-1.11189	-1.10753	BGIOSGA019382	XP_006655779.1 pre-mRNA-splicing factor 38B-like
TCONS_00017899	-1.16459	-1.68617	-2.21272	BGIOSGA004822	XP_006654479.2 cytochrome P450 94C1-like
TCONS_00017681	-1.16435	0.525037	-0.950278	BGIOSGA019743	XP_006655255.1 protein BONZAI 3-like
TCONS_00013708	-1.16429	-5.51943	-1.69409	BGIOSGA015865	XP_020401269.1myosin heavy chain, striated muscle
TCONS_00022587	-1.16427	-0.79401	-0.336682	BGIOSGA029524	XP_006656267.1 cinnamoyl-CoA reductase 2-like
TCONS_00023639	-1.1642	-1.47515	-2.5356	BGIOSGA025664	XP_015694625.1 DNA-directed primase/polymerase protein
TCONS_00028200	-1.16408	-0.645966	0.165227	BGIOSGA014952	XP_006659514.1 blue copper protein-like
TCONS_00006391	-1.16326	-0.687838	-0.422458	BGIOSGA006079	XP_015689345.1 telomere length regulation protein TEL2 homolog
TCONS_00022342	-1.16108	-0.699831	-0.677209	BGIOSGA021131	XP_015694426.1PREDICTED: LOW QUALITY PROTEIN: uncharacterized protein LOC102722408
TCONS_00021023	-1.16108	-2.28479	-2.84713	BGIOSGA037112	XP_015695071.1PREDICTED: uncharacterized protein LOC107304639
TCONS_00000462	-1.16082	-0.201108	-0.399671	BGIOSGA003037	XP_015692248.1 GTPase Der
TCONS_00012440	-1.16081	-2.66144	-0.179658	BGIOSGA028946	NP_001130701.1EF hand family protein
TCONS_00007990	-1.16056	6.03003E-05	0.643451	#N/A	#N/A
TCONS_00003789	-1.16045	-0.954222	-2.16311	BGIOSGA020483	XP_006644271.1 vacuolar cation/proton exchanger 1a
TCONS_00001213	-1.16023	-0.662589	-2.68559	BGIOSGA037676	XP_006646035.2 probable protein phosphatase 2C 6

TCONS_00017406	-1.15981	-3.50006	-1.98712	BGIOSGA019474	XP_006654179.1 putative leucine-rich repeat receptor-like protein kinase At2g19210 isoform X1
TCONS_00007907	-1.15958	0.105932	-0.680912	#N/A	#N/A
TCONS_00027227	-1.15903	-1.74038	-0.96213	BGIOSGA027775	XP_006659044.1 survival of motor neuron-related-splicing factor 30 isoform X1
TCONS_00005245	-1.159	-1.31084	-2.81814	BGIOSGA009326	XP_003574998.1cytochrome P450 89A2
TCONS_00034374	-1.15864	0.104948	-1.68323	BGIOSGA037056	NP_001168481.1uncharacterized LOC100382258
TCONS_00022901	-1.15827	-0.919181	-0.0762556	BGIOSGA002171	XP_006657341.1PREDICTED: uncharacterized protein LOC102717169
TCONS_00008166	-1.15796	0.173285	-0.176837	BGIOSGA000406	XP_006648725.1PREDICTED: uncharacterized protein LOC102718151
TCONS_00027302	-1.15704	-0.808383	-1.46523	BGIOSGA027701	XP_006659802.1 probable sugar phosphate/phosphate translocator At3g14410
TCONS_00024323	-1.15651	#NA	-2.27349	BGIOSGA005952	XP_006658953.2 formin BNI1-like isoform X2
TCONS_00029553	-1.15636	-0.554323	-0.864295	BGIOSGA009094	XP_006661011.1 peptidyl-prolyl cis-trans isomerase CYP19-3 isoform X1
TCONS_00033009	-1.15629	-1.14961	-1.17278	BGIOSGA008794	XP_006662753.2 transcription factor LHW-like
TCONS_00002718	-1.15531	-1.68556	-2.60861	BGIOSGA001576	XP_004968129.1uncharacterized protein LOC101783535
TCONS_00004061	-1.15476	-1.06279	-0.885918	BGIOSGA019167	XP_006644427.1 mitogen-activated protein kinase 16
TCONS_00004992	-1.15436	-1.52401	-0.946035	BGIOSGA000227	XP_006645254.1 F-box only protein 6
TCONS_00003141	-1.15431	-1.58121	-1.59753	BGIOSGA002087	XP_006643939.1PREDICTED: uncharacterized protein LOC102700836
TCONS_00009429	-1.15402	-1.01528	-1.2732	BGIOSGA028973	XP_008649396.1lipoxygenase 9 isoform X1
TCONS_00034483	-1.15369	1.1134	0.0708026	BGIOSGA034250	XP_006663282.1 F-box/FBD/LRR-repeat protein At5g22660-like isoform X1
TCONS_00006050	-1.15362	-0.437936	-0.526558	BGIOSGA024624	XP_006647300.1 40S ribosomal protein S15a-1
TCONS_00000082	-1.15335	-1.25604	-1.94043	BGIOSGA002652	XP_006643675.1 probable receptor-like protein kinase At1g67000
TCONS_00035452	-1.15268	#NA	-0.698221	BGIOSGA004476	XP_006644104.1 probable receptor-like protein kinase At5g18500
TCONS_00003395	-1.15202	-3.33649	-2.08901	BGIOSGA016787	XP_006644108.1 UPF0481 protein At3g47200-like
TCONS_00014662	-1.15177	-0.301182	-0.172091	BGIOSGA016825	XP_010240178.1O-glucosyltransferase rumi homolog
TCONS_00028225	-1.15142	0.970187	0.263151	BGIOSGA019220	XP_006659525.1 translation initiation factor IF-2-like
TCONS_00003494	-1.15133	-2.13244	-1.2036	BGIOSGA001710	NP_001306658.1TPR repeat
TCONS_00012855	-1.15069	0.0964481	-0.288205	BGIOSGA014199	XP_006651653.1 probable WRKY transcription factor 2
TCONS_00009519	-1.15031	-1.56601	0.446835	BGIOSGA037929	XP_006649588.1 probable strigolactone esterase D14
TCONS_00030701	-1.14922	-0.524901	-0.104314	BGIOSGA034126	XP_006661624.1 E3 ubiquitin-protein ligase At3g02290-like
TCONS_00015179	-1.14839	-2.33978	-1.7702	BGIOSGA005008	XP_006653884.1 E3 ubiquitin-protein ligase RNF8-like
TCONS_00007873	-1.14772	0.45571	-1.29575	BGIOSGA005335	XP_006651645.2PREDICTED: LOW QUALITY PROTEIN: uncharacterized protein LOC102704603
TCONS_00030308	-1.14752	0.224161	0.703745	BGIOSGA029500	XP_006660800.1 mimitin, mitochondrial
TCONS_00019137	-1.14695	0.0922662	-2.2977	BGIOSGA001387	XP_002440938.1transcription initiation factor TFIID subunit 15b
TCONS_00023048	-1.14695	#NA	-0.712737	BGIOSGA009167	XP_006657419.1 pathogenesis-related protein 1-like
TCONS_00009346	-1.14649	-1.31291	-0.440037	BGIOSGA001134	XP_006649427.1 cell number regulator 13
TCONS_00034228	-1.14574	-2.71649	0.869158	BGIOSGA003363	XP_006663179.2PREDICTED: uncharacterized protein LOC102721030
TCONS_00012821	-1.14574	-0.909133	-0.715805	BGIOSGA025639	XP_004964061.2sigma intracellular receptor 2
TCONS_00032796	-1.14569	-0.318961	0.444828	BGIOSGA024561	XP_010907063.1 THO complex subunit 4A
TCONS_00006796	-1.14554	-1.20459	-1.73469	BGIOSGA030644	XP_015689111.1 ASC1-like protein 2
TCONS_00010350	-1.14512	0.0901351	0.282613	BGIOSGA012926	XP_015689942.1 lysine-specific demethylase JMJ25-like
TCONS_00016700	-1.14454	-2.71791	-1.30382	BGIOSGA014269	XP_024311529.1uncharacterized protein LOC100840279
TCONS_00000133	-1.1445	2.07217	1.62327	#N/A	#N/A
TCONS_00035022	-1.14426	-1.87024	-0.538985	BGIOSGA033835	XP_015697889.1 armadillo repeat-containing protein 7
TCONS_00017247	-1.14413	2.13959	#NA	BGIOSGA019289	NP_001144377.1uncharacterized LOC100277300
TCONS_00002688	-1.144	1.86641	-0.135269	BGIOSGA014512	XP_006643666.1PREDICTED: uncharacterized protein LOC102712757
TCONS_00029943	-1.14337	-2.71931	#NA	BGIOSGA032361	XP_006646025.1 putative cyclin-dependent kinase F-2
TCONS_00031960	-1.14337	#NA	-2.30678	#N/A	#N/A
TCONS_00006604	-1.14299	-0.535351	-0.810926	BGIOSGA020319	XP_006647651.1 U-box domain-containing protein 33-like
TCONS_00001129	-1.14265	-0.608137	-3.36588	BGIOSGA017996	XP_021313622.1protein NRT1/ PTR FAMILY 6.2
TCONS_00030425	-1.14253	-1.21265	-0.467131	BGIOSGA029377	XP_015696680.1 lon protease homolog 2, peroxisomal isoform X2
TCONS_00003321	-1.1424	-0.620227	-0.123273	BGIOSGA010243	XP_010227418.1exportin-7-A isoform X1
TCONS_00005180	-1.14194	0.300919	-0.594658	BGIOSGA027933	XP_006646734.2 pentatricopeptide repeat-containing protein At1g11290, chloroplastic-like
TCONS_00028487	-1.14148	#NA	#NA	BGIOSGA014148	XP_006660396.1 putative kinase-like protein TMKL1
TCONS_00033742	-1.14148	#NA	#NA	BGIOSGA033680	XP_015698117.1 beta-carotene isomerase D27, chloroplastic
TCONS_00026356	-1.1413	1.27815	0.688006	#N/A	#N/A
TCONS_00011774	-1.14099	-0.928144	-0.939092	BGIOSGA011231	XP_015691150.1 pre-mRNA-processing factor 39 isoform X1
TCONS_00016820	-1.14096	-1.30724	-1.19738	BGIOSGA008737	XP_006652981.1 ubiquitin carboxyl-terminal hydrolase 3-like isoform X1

TCONS_00009084	-1.14088	0.216222	-1.31307	BGIOSGA015052	XP_004954492.1transcription factor TCP7-like
TCONS_00004686	-1.14071	-0.263149	-1.72852	BGIOSGA005997	XP_015690345.1 dirigent protein 25-like
TCONS_00036723	-1.14056	-2.0309	-1.57691	BGIOSGA014059	XP_004977335.1NDR1/HIN1-like protein 1
TCONS_00001518	-1.14056	-0.40085	#NA	BGIOSGA030618	XP_015689474.1 CASP-like protein 4A3
TCONS_00029554	-1.14048	#NA	2.07824	BGIOSGA011329	XP_006661578.1 ethylene-responsive transcription factor 1B-like
TCONS_00014710	-1.14018	-2.72325	-0.992899	BGIOSGA002718	XP_004976435.1ethylene-responsive transcription factor ERF039
TCONS_00030875	-1.14011	-1.35411	-0.993081	#N/A	#N/A
TCONS_00006597	-1.1401	-1.33128	-1.1276	BGIOSGA019501	XP_015689452.1 scarecrow-like protein 6
TCONS_00028276	-1.14007	-3.09682	-0.864287	BGIOSGA029544	XP_006659573.1 beta-glucosidase 27
TCONS_00030868	-1.13969	-1.72386	-2.31605	BGIOSGA032672	XP_015697394.1PREDICTED: uncharacterized protein LOC102718870 isoform X3
TCONS_00031647	-1.13969	0.112637	-0.663977	BGIOSGA033470	XP_002453434.2putative F-box protein At2g02030 isoform X1
TCONS_00024853	-1.1395	0.320282	0.207015	BGIOSGA024521	XP_002459624.1NDR1/HIN1-like protein 2
TCONS_00023214	-1.13914	-1.85421	-0.266297	BGIOSGA036677	XP_003576348.1protein ZINC INDUCED FACILITATOR-LIKE 1 isoform X1
TCONS_00026946	-1.1384	-0.222992	-0.203805	BGIOSGA033119	XP_004972995.1ACT domain-containing protein ACR8
TCONS_00030407	-1.13778	-3.72628	-0.818336	BGIOSGA029394	XP_015696765.1PREDICTED: uncharacterized protein LOC102717925
TCONS_00016350	-1.13748	0.443264	-1.58462	BGIOSGA035269	XP_003580265.1ELMO domain-containing protein A
TCONS_00005386	-1.13742	-2.68504	-2.62953	BGIOSGA020577	XP_006646870.1 squamosa promoter-binding-like protein 3
TCONS_00027684	-1.13732	-0.852397	-0.321994	BGIOSGA026210	XP_024371671.1BUD13 homolog
TCONS_00015402	-1.13724	0.935999	0.485164	BGIOSGA028391	XP_006652114.1PREDICTED: uncharacterized protein KIAA0930 homolog
TCONS_00010673	-1.13712	-1.40518	-1.32248	#N/A	#N/A
TCONS_00031983	-1.13625	-0.85375	0.412303	BGIOSGA031740	XP_006647129.1 bifunctional riboflavin kinase/FMN phosphatase-like
TCONS_00026621	-1.13616	-1.34982	-1.47689	BGIOSGA017241	XP_002444347.1uncharacterized protein LOC8070096
TCONS_00002284	-1.13601	-2.92726	-1.60994	BGIOSGA018021	XP_006645120.1 UDP-glucuronate:xylan alpha-glucuronosyltransferase 1-like
TCONS_00005100	-1.13596	0.558603	0.716482	BGIOSGA034990	XP_015688309.1 flowering time control protein FY
TCONS_00005869	-1.13488	-0.61447	-0.158145	BGIOSGA014550	XP_015688827.1 GDSL esterase/lipase At2g42990-like isoform X1
TCONS_00028969	-1.1348	-3.73004	#NA	BGIOSGA034213	XP_006661202.2 probable cinnamyl alcohol dehydrogenase 8A
TCONS_00036939	-1.13466	1.07713	-1.65055	BGIOSGA028494	XP_022685166.1putative receptor-like protein kinase At4g00960 isoform X1
TCONS_00029030	-1.13455	-1.43268	-1.24142	BGIOSGA029336	XP_015696557.1 AP2-like ethylene-responsive transcription factor At1g16060
TCONS_00027012	-1.13437	0.105913	-0.492831	BGIOSGA029062	XP_006659634.2 glycerophosphodiester phosphodiesterase GDPD1, chloroplastic-like
TCONS_00035450	-1.13437	0.893903	1.60927	BGIOSGA036893	XP_010227211.1uncharacterized protein LOC104581408
TCONS_00008956	-1.13381	-3.31625	-2.91567	BGIOSGA005744	XP_003570444.1putative F-box/FBD/LRR-repeat protein At5g56810
TCONS_00021561	-1.13363	-1.27208	-0.746194	BGIOSGA006535	XP_006655761.2 probable protein transport Sec1b
TCONS_00036517	-1.13316	0.125877	0.191246	BGIOSGA020261	XP_024312256.1WRKY transcription factor 44 isoform X2
TCONS_00004375	-1.13309	-0.147233	-0.332496	BGIOSGA020213	XP_015699322.1PREDICTED: LOW QUALITY PROTEIN: uncharacterized protein LOC102708860
TCONS_00024054	-1.13302	-3.56168	-0.466957	BGIOSGA010527	XP_015695378.1 probable 6-phosphogluconolactonase 2
TCONS_00022041	-1.1329	-1.10017	-1.06994	BGIOSGA037798	XP_006655993.2PREDICTED: uncharacterized protein LOC102716696, partial
TCONS_00001178	-1.13282	-0.23004	0.169328	BGIOSGA011837	XP_015689076.1 protein indeterminate-domain 7
TCONS_00034058	-1.13253	-0.484968	-1.33387	BGIOSGA036445	XP_006663115.1 probable LRR receptor-like serine/threonine-protein kinase At3g47570
TCONS_00029463	-1.13252	0.396371	-0.633464	BGIOSGA031215	XP_006661506.2 protein DETOXIFICATION 45, chloroplastic-like
TCONS_00030257	-1.13241	-1.51066	-1.45971	BGIOSGA035042	XP_006660768.1 IRK-interacting protein
TCONS_00009009	-1.13189	-0.885706	-0.409458	BGIOSGA027089	XP_002454747.1wall-associated receptor kinase 2
TCONS_00023698	-1.13189	-2.92635	-2.01354	BGIOSGA034983	XP_022680073.1protein ACCELERATED CELL DEATH 6-like
TCONS_00009081	-1.13182	0.266215	-0.528264	BGIOSGA016399	XP_006649194.1 pentatricopeptide repeat-containing protein At5g38730
TCONS_00030392	-1.13181	-0.596301	-0.265269	BGIOSGA019515	XP_015696483.1 protein HAPLESS 2-B-like isoform X1
TCONS_00014259	-1.13154	-0.033708	-0.429441	BGIOSGA029512	XP_006652286.1 probable helicase MAGATAMA 3
TCONS_00007788	-1.13132	-1.73442	-0.449336	BGIOSGA017098	XP_015688554.1 ent-copalyl diphosphate synthase 1, chloroplastic
TCONS_00017427	-1.12998	-1.64947	-1.13627	BGIOSGA035854	XP_015693222.1 probable LRR receptor-like serine/threonine-protein kinase At1g56140
TCONS_00020546	-1.12894	-1.3293	-3.3041	BGIOSGA029855	XP_015694302.1 UDP-glycosyltransferase 708A6-like
TCONS_00027784	-1.12859	-0.140763	-2.15825	BGIOSGA030233	XP_015695461.1 protein DCL, chloroplastic
TCONS_00002206	-1.12568	1.0824	-1.21046	BGIOSGA018439	XP_015692120.1 acidic endochitinase-like
TCONS_00028999	-1.12519	-1.17687	-1.47563	BGIOSGA004730	XP_015696824.1 probable methyltransferase PMT2
TCONS_00007501	-1.12453	0.0109692	-0.0042213	BGIOSGA020308	XP_015688487.1 putative multidrug resistance protein
TCONS_00032806	-1.12447	-1.3082	-1.41356	BGIOSGA023035	NP_001168546.1putative DUF231 domain containing family protein

TCONS_00011787	-1.12417	-0.556487	-0.0281122	BGIOSGA012883	XP_015690765.1 ninja-family protein Os03g0214200-like
TCONS_00022734	-1.12408	-1.29276	-0.772887	BGIOSGA007732	XP_015693671.1 copper-transporting ATPase RAN1-like
TCONS_00012962	-1.12399	-1.40362	-1.01599	BGIOSGA004890	XP_015690889.1 protein NRT1/ PTR FAMILY 2.11
TCONS_00018054	-1.12321	-1.80978	-0.586928	BGIOSGA000640	XP_004961641.1uncharacterized protein LOC101768497
TCONS_00023240	-1.12117	-1.42574	-1.60029	BGIOSGA011249	XP_006658339.1 histone H1-like
TCONS_00030074	-1.12078	-1.68256	-1.66291	BGIOSGA029729	XP_015696354.1 WAT1-related protein At4g30420-like
TCONS_00020132	-1.12074	-2.51447	-1.51826	BGIOSGA031400	XP_006656647.1 putative dual specificity protein phosphatase DSP8
TCONS_00004338	-1.12026	-2.23107	-2.2745	BGIOSGA020237	XP_006644660.1 protein STICHEL-like 4
TCONS_00013429	-1.11967	0.165202	0.131796	BGIOSGA012352	XP_015690127.1PREDICTED: uncharacterized protein LOC102712626, partial
TCONS_00005883	-1.11933	-0.843737	0.203523	BGIOSGA014663	XP_002451987.2L-type lectin-domain containing receptor kinase IV.2
TCONS_00009843	-1.11898	-3.37844	-1.16998	BGIOSGA001107	XP_006649908.1 protein PMR5-like
TCONS_00018696	-1.11799	0.451472	-0.012055	BGIOSGA003969	XP_006655038.1 extensin-2-like
TCONS_00026121	-1.1178	-1.71287	-1.8135	BGIOSGA039516	NP_001151098.1uncharacterized LOC100272871 precursor
TCONS_00003180	-1.11756	-2.06578	-2.05721	BGIOSGA002043	XP_004967344.1probable transcription factor GLK2
TCONS_00036094	-1.11742	-1.23007	-0.697736	BGIOSGA009740	XP_010090180.140S ribosomal protein S29
TCONS_00037888	-1.11735	-1.12605	-1.09892	BGIOSGA024932	XP_022684565.1uncharacterized protein LOC111258131
TCONS_00023104	-1.11694	-1.46158	-0.700164	BGIOSGA017139	XP_015694524.1 transcription factor PIF5-like
TCONS_00013184	-1.11574	-1.16648	-1.32018	BGIOSGA027199	XP_014752433.1oligopeptide transporter 3
TCONS_00009252	-1.11555	1.50271	-1.67664	BGIOSGA023633	XP_004985822.1chitinase 11
TCONS_00020702	-1.11548	1.01692	0.0552188	BGIOSGA040463	XP_006656078.2 1,4-alpha-glucan-branching enzyme 3, chloroplastic/amyloplastic
TCONS_00000018	-1.11535	0.218941	-0.880979	BGIOSGA004895	XP_006645424.1 protein NRT1/ PTR FAMILY 6.1
TCONS_00005041	-1.1151	-4.46976	-2.45306	BGIOSGA000172	XP_006646655.2 probable esterase PIR7A
TCONS_00018954	-1.11491	-0.735589	-0.895162	BGIOSGA007670	XP_004962519.1serine carboxypeptidase 2
TCONS_00024655	-1.11466	-2.34767	0.0619622	BGIOSGA002254	XP_008652078.1protein MARD1 isoform X2
TCONS_00016136	-1.11439	-1.76055	0.517165	BGIOSGA038668	XP_008668638.1malate synthase, glyoxysomal isoform X1
TCONS_00007361	-1.11394	-0.59886	-0.144604	BGIOSGA007123	XP_004951806.1uncharacterized protein C23H3.12c
TCONS_00002671	-1.11378	0.236199	0.852311	BGIOSGA012103	XP_004967945.1G patch domain-containing protein 4
TCONS_00004783	-1.11356	-0.241264	-1.06852	BGIOSGA000431	XP_006645076.1 sm-like protein LSM3A
TCONS_00016552	-1.11332	-2.76653	-1.01201	BGIOSGA014415	NP_001147488.1EF hand family protein
TCONS_00022092	-1.11328	-0.317248	-2.79492	BGIOSGA008065	XP_006656011.1PREDICTED: uncharacterized protein LOC102722220
TCONS_00001813	-1.11319	-2.70247	0.38438	BGIOSGA018806	XP_015692703.1PREDICTED: uncharacterized protein LOC102712863
TCONS_00030577	-1.11301	-0.0356608	-0.155706	BGIOSGA032412	XP_006661585.1 U-box domain-containing protein 33-like
TCONS_00007645	-1.1128	0.101866	0.0561249	BGIOSGA000897	XP_006647092.1 40S ribosomal protein S24-1
TCONS_00025727	-1.11274	-0.818768	-1.0154	BGIOSGA013100	XP_004958734.1ETHYLENE INSENSITIVE 3-like 1 protein
TCONS_00004755	-1.11192	-0.973242	-1.8555	BGIOSGA001188	XP_006645045.1 GABA transporter 1
TCONS_00013647	-1.11149	-2.94942	-3.4347	BGIOSGA035372	XP_015691642.1 subtilisin-like protease SBT3.9
TCONS_00016657	-1.11085	0.807272	-2.18415	BGIOSGA029405	XP_006652859.1 homeobox-leucine zipper protein ROC2
TCONS_00012438	-1.11045	-0.221783	-0.821599	BGIOSGA005411	XP_015690351.1 protein ELF4-LIKE 3-like
TCONS_00009685	-1.11027	0.0969534	-1.48813	BGIOSGA008975	XP_004985008.1ACT domain-containing protein ACR8
TCONS_00016082	-1.11026	-1.15301	-0.465272	BGIOSGA011222	XP_006652404.2 beta-glucosidase 10-like
TCONS_00024180	-1.11026	-2.27269	-1.86106	BGIOSGA026218	XP_006658010.1PREDICTED: uncharacterized protein LOC102703412 isoform X1
TCONS_00032774	-1.11006	0.539615	0.330538	BGIOSGA031335	XP_002464873.1protein HAIKU1
TCONS_00012211	-1.10984	-0.742693	-0.784129	#N/A	#N/A
TCONS_00019608	-1.10964	0.496264	0.225079	BGIOSGA035877	XP_015692774.1 CRC domain-containing protein TSO1-like
TCONS_00025923	-1.10963	-1.48696	-0.734822	BGIOSGA016851	XP_006659117.1 sphingoid long-chain bases kinase 1-like
TCONS_00007243	-1.10844	-1.05852	-0.275506	BGIOSGA007244	XP_006648236.2 autophagy protein 5
TCONS_00031329	-1.10839	-2.03682	-0.921198	BGIOSGA016282	XP_006661866.2 auxin response factor 22
TCONS_00002222	-1.10836	-1.15507	-0.733109	#N/A	#N/A
TCONS_00026994	-1.10822	0.694994	-0.46848	BGIOSGA016388	XP_006660323.1 exocyst complex component EXO70A1-like
TCONS_00032593	-1.10738	-1.04682	-1.98992	BGIOSGA013093	XP_006661975.1 subtilisin-like protease SBT1.7
TCONS_00030771	-1.10722	-3.57944	-3.17901	BGIOSGA036323	XP_010937386.1 ABC transporter G family member 11-like isoform X2
TCONS_00012277	-1.1072	0.168408	0.286082	BGIOSGA000206	XP_006650085.1 nucleolar protein 56-like
TCONS_00014470	-1.10713	-1.0856	-0.943231	BGIOSGA016629	XP_006653540.1 anaphase-promoting complex subunit 2
TCONS_00005852	-1.10707	-1.58995	-2.324	BGIOSGA014643	XP_021315034.1putative elongation factor TypA-like SVR3, chloroplastic isoform X1
TCONS_00023988	-1.10703	0.290208	-0.793687	BGIOSGA012030	XP_006657889.1 chitin-inducible gibberellin-responsive protein 2
TCONS_00029263	-1.10693	-2.28742	-1.69315	BGIOSGA028960	XP_015696820.1 OTU domain-containing protein At3g57810
TCONS_00013510	-1.10682	0.717736	-0.443039	BGIOSGA006820	XP_006650875.1 shaggy-related protein kinase kappa-like
TCONS_00027379	-1.10673	-4.47881	-4.05918	#N/A	#N/A
TCONS_00026588	-1.1066	-1.80763	-0.656975	BGIOSGA030637	XP_015695767.1 quinolone resistance protein NorA-like

TCONS_00007980	-1.10647	-0.933695	0.959162	BGIOSGA006504	XP_006647307.1 putative glutathione-specific gamma-glutamylcyclotransferase 2
TCONS_00032597	-1.10591	-0.981137	-0.835569	BGIOSGA016958	XP_006662532.1 cytochrome P450 704C1-like
TCONS_00029991	-1.10577	-1.58126	-0.960194	BGIOSGA030695	XP_015696332.1 malonyl-CoA decarboxylase, mitochondrial
TCONS_00000382	-1.10558	-0.416122	1.21357	BGIOSGA002237	XP_015696384.1 B-box zinc finger protein 22
TCONS_00002784	-1.1049	-2.86437	-1.87454	BGIOSGA021550	XP_006644049.1PREDICTED: uncharacterized protein LOC102708379
TCONS_00000362	-1.10477	-0.894654	-1.45331	BGIOSGA002275	XP_006643871.1 protein indeterminate-domain 11-like
TCONS_00029085	-1.104	-0.483582	-0.564198	BGIOSGA030833	XP_004956923.1probable polygalacturonase
TCONS_00033406	-1.10393	-1.80921	-0.672052	BGIOSGA019094	XP_006662874.1 receptor like protein kinase S.2 isoform X1
TCONS_00032222	-1.10383	-0.373869	-0.761727	BGIOSGA000264	XP_004983193.1uncharacterized protein LOC101753379
TCONS_00002639	-1.10311	-1.52881	-1.97461	BGIOSGA005902	NP_001152540.1ring zinc finger protein
TCONS_00019504	-1.10297	-1.11931	-0.418236	BGIOSGA004696	XP_006654546.1 mucin-5AC-like
TCONS_00004979	-1.10252	-0.922519	-2.53943	BGIOSGA015921	XP_006645238.1 probable auxin efflux carrier component 6
TCONS_00027021	-1.10241	-0.120011	0.479465	BGIOSGA029230	XP_014755420.1O-fucosyltransferase 13
TCONS_00032148	-1.10218	-1.05597	-1.35528	BGIOSGA031991	XP_015697270.1 stress enhanced protein 1, chloroplastic-like
TCONS_00029485	-1.10188	#NA	-3.88203	BGIOSGA008700	XP_006661521.2 cytosolic sulfotransferase 5-like
TCONS_00009749	-1.1012	-1.86903	-0.883727	BGIOSGA011936	XP_006649815.1PREDICTED: uncharacterized protein LOC102704597
TCONS_00021224	-1.10113	-2.03871	-1.07729	BGIOSGA000184	XP_006656365.1 probable potassium transporter 13
TCONS_00016588	-1.10077	-0.596554	-0.845259	BGIOSGA014378	XP_006652797.1 organic cation/carnitine transporter 7
TCONS_00031771	-1.10069	-0.346115	-0.382492	BGIOSGA009263	XP_006662187.2 putative F-box protein At5g38390
TCONS_00017287	-1.10069	1.06106	-0.30003	BGIOSGA025423	XP_006654141.1 zinc transporter 7
TCONS_00029300	-1.10064	-0.832557	-0.585419	BGIOSGA031042	XP_021310719.1tRNA-splicing endonuclease subunit Sen54
TCONS_00026390	-1.09965	-0.549053	-0.887557	BGIOSGA028917	XP_006660049.2 probable galacturonosyltransferase 4
TCONS_00002762	-1.09947	-1.31139	0.138088	BGIOSGA002456	XP_006643691.1 Bowman-Birk type wound-induced proteinase inhibitor WIP1-like
TCONS_00025804	-1.09906	-0.844056	-3.0786	BGIOSGA008983	XP_006659055.1 MADS-box transcription factor 26
TCONS_00022938	-1.09839	0.88474	-1.56146	BGIOSGA010816	XP_004966508.1NAC domain-containing protein 67
TCONS_00008676	-1.09828	-2.03602	-1.26889	BGIOSGA005754	XP_006647784.2PREDICTED: uncharacterized protein LOC102717316 isoform X1
TCONS_00013812	-1.09768	0.368334	-1.08208	BGIOSGA020375	XP_024318798.1ultraviolet-B receptor UVR8
TCONS_00007569	-1.09683	-0.310026	-0.882624	BGIOSGA003750	XP_015689511.1 TATA-binding protein-associated factor 2N
TCONS_00032896	-1.09566	-1.32227	-0.765195	BGIOSGA012529	XP_015698179.1 ELMO domain-containing protein C-like
TCONS_00017328	-1.09566	-1.51066	-0.804201	BGIOSGA035349	XP_006655093.1 GDSL esterase/lipase At1g28580-like
TCONS_00023946	-1.09531	-2.11228	-0.839632	BGIOSGA022624	NP_001145964.1primary amine oxidase precursor
TCONS_00006406	-1.09526	-2.52739	-1.49184	BGIOSGA019564	XP_004953005.1ankyrin repeat and death domain-containing protein 1A
TCONS_00009845	-1.09412	-1.98808	-2.08375	BGIOSGA017466	XP_006649910.1 protein trichome birefringence-like 28
TCONS_00014826	-1.09393	0.154591	-0.367904	BGIOSGA002819	XP_003580431.1receptor-like protein kinase FERONIA
TCONS_00025481	-1.09393	0.753543	-0.391002	BGIOSGA023950	XP_015694897.1 ribosome biogenesis protein WDR12 homolog
TCONS_00000491	-1.09345	-0.794547	-0.39219	BGIOSGA008955	XP_006643959.1 putative ubiquitin-conjugating enzyme E2 38 isoform X3
TCONS_00005806	-1.093	-1.17363	-1.50878	BGIOSGA028948	XP_015689079.1 probable WRKY transcription factor 27
TCONS_00021378	-1.09225	-0.932866	-0.545734	BGIOSGA000909	XP_015694314.1PREDICTED: uncharacterized protein LOC102721372
TCONS_00022843	-1.0919	-0.211543	-0.341598	BGIOSGA032394	NP_001149719.1cell Division Protein AAA ATPase family
TCONS_00012898	-1.09156	-1.32744	-0.637885	BGIOSGA029492	XP_008681422.1putative O-glycosyl hydrolase family 17 protein isoform X1
TCONS_00007202	-1.091	-1.79764	-0.928782	BGIOSGA029457	XP_021320637.1putative F-box/FBD/LRR-repeat protein At5g56810
TCONS_00009227	-1.09097	-1.86067	-0.44851	BGIOSGA011183	XP_004985897.1probable serine/threonine-protein kinase At1g01540
TCONS_00020080	-1.09017	-0.426557	-0.0809113	BGIOSGA022260	XP_006656614.1 GDP-mannose 4,6 dehydratase 1-like
TCONS_00025979	-1.0898	-0.97601	-1.51669	BGIOSGA023713	XP_004972751.1ABC transporter G family member 14
TCONS_00036739	-1.08963	-2.19432	-1.67977	BGIOSGA035796	XP_006663847.1 deoxyhypusine hydroxylase-A
TCONS_00024492	-1.08935	-2.04071	-2.10276	BGIOSGA015286	XP_024312030.1receptor kinase-like protein Xa21
TCONS_00033121	-1.08931	-0.252258	-0.570359	BGIOSGA034971	XP_015697582.1 DNA topoisomerase 2-binding protein 1-A
TCONS_00005301	-1.08862	-0.0462544	0.215884	BGIOSGA011515	XP_015688994.1 putative HVA22-like protein g
TCONS_00024845	-1.08817	-0.40245	-1.22511	BGIOSGA024524	XP_006658442.2 IAA-amino acid hydrolase ILR1-like 7
TCONS_00001722	-1.08809	-0.720676	-0.883285	BGIOSGA027322	XP_015689967.1 putative disease resistance protein RGA3
TCONS_00019186	-1.08799	-3.63848	-0.90431	BGIOSGA031741	XP_015693018.1 lichenase-2-like
TCONS_00030080	-1.08774	-3.14844	-0.7957	BGIOSGA028313	XP_015696356.1 probable xyloglucan glycosyltransferase 2
TCONS_00007413	-1.08769	0.0214612	0.245703	BGIOSGA007061	XP_006646931.1 ribonuclease H2 subunit C
TCONS_00012963	-1.086	-1.78021	-0.546112	BGIOSGA005707	XP_004982145.1formin-like protein 14

TCONS_00017793	-1.08552	0.115467	-0.333153	BGIOSGA030818	XP_015692598.1 gibberellin receptor GID1
TCONS_00001697	-1.08481	-0.683579	0.291576	BGIOSGA016328	XP_015689737.1 alpha-amylase 3, chloroplastic
TCONS_00008902	-1.08475	-2.4945	-1.33621	BGIOSGA029411	XP_006647990.1 zinc finger protein 8-like
TCONS_00007544	-1.08475	-3.15674	-2.69054	#N/A	#N/A
TCONS_00032226	-1.08435	-2.46068	-2.54621	BGIOSGA016731	XP_010234737.1protein indeterminate-domain 12 isoform X1
TCONS_00026426	-1.08388	-1.56818	-1.36678	BGIOSGA007286	XP_021317531.1cysteine-rich receptor-like protein kinase 15 isoform X2
TCONS_00036487	-1.08324	0.526483	-0.661019	BGIOSGA024868	XP_002442601.1receptor-like protein kinase HAIKU2
TCONS_00037288	-1.08288	-0.628318	-0.783153	BGIOSGA036080	XP_006664067.1PREDICTED: uncharacterized protein LOC102710077
TCONS_00007377	-1.08282	0.707322	-0.286945	BGIOSGA007100	XP_006646901.1 tyrosine-sulfated glycopeptide receptor 1-like
TCONS_00010233	-1.08209	-2.51884	-2.27858	BGIOSGA033417	XP_006651467.1 patatin-like protein 1
TCONS_00014937	-1.08181	-2.32032	-3.31549	BGIOSGA028648	XP_015692319.1PREDICTED: uncharacterized protein LOC102702749
TCONS_00033344	-1.0808	-0.986899	0.228266	#N/A	#N/A
TCONS_00020975	-1.08038	-1.46468	-0.769817	BGIOSGA009218	XP_006657104.1 probable protein phosphatase 2C 57
TCONS_00015237	-1.08035	-1.06852	-2.04843	BGIOSGA031737	XP_015691344.1PREDICTED: uncharacterized protein LOC102714825
TCONS_00030115	-1.08029	-1.40258	-2.35797	BGIOSGA001038	XP_006660660.1 protein TIFY 10c-like
TCONS_00011927	-1.08009	-2.93033	-0.678304	BGIOSGA032709	XP_006649762.2 beta-galactosidase 6
TCONS_00016842	-1.07997	-0.303069	-1.0334	BGIOSGA014122	XP_006653004.1 polyamine oxidase 3-like
TCONS_00025924	-1.07951	-2.98853	#NA	BGIOSGA028002	XP_006659119.1 ABC transporter B family member 19-like
TCONS_00007786	-1.07948	-1.44811	-1.84892	BGIOSGA006697	XP_014754981.1probable leucine-rich repeat receptor-like protein kinase At1g35710 isoform X1
TCONS_00031945	-1.07923	-2.12147	-1.15951	#N/A	XP_002443140.2nuclear mitotic apparatus protein 1
TCONS_00014902	-1.07917	0.835744	-2.70413	#N/A	#N/A
TCONS_00017094	-1.07842	-2.51707	-3.04187	BGIOSGA004259	XP_015693237.1 ABC transporter B family member 21-like
TCONS_00033573	-1.07829	-0.973643	-0.824345	BGIOSGA001222	XP_023157963.1uncharacterized LOC100278526 isoform X2
TCONS_00016558	-1.07809	-1.104	-1.05164	BGIOSGA004742	XP_015692104.1 serine/threonine-protein kinase minibrain-like isoform X2
TCONS_00014630	-1.07752	-0.00778011	-1.39185	BGIOSGA014629	XP_004976283.1probable metal-nicotianamine transporter YSL12
TCONS_00003341	-1.0773	0.178597	#NA	BGIOSGA006968	XP_004967647.1bromodomain adjacent to zinc finger domain protein 2B-like
TCONS_00031573	-1.07715	-0.669582	0.659724	BGIOSGA027939	XP_003574275.1protein STRICTOSIDINE SYNTHASE-LIKE 6
TCONS_00016283	-1.07686	-0.519782	0.274917	BGIOSGA033224	XP_003581442.1probable metal-nicotianamine transporter YSL13
TCONS_00025314	-1.07601	-1.31166	-1.04986	BGIOSGA016398	NP_001105655.2S-domain class receptor-like kinase 3 precursor
TCONS_00034554	-1.0754	-0.186367	1.1888	BGIOSGA034269	XP_006663306.1 probable non-inhibitory serpin-Z9
TCONS_00020459	-1.07537	-1.12894	-0.550294	BGIOSGA027385	XP_015696028.1 protein CHROMATIN REMODELING 35-like
TCONS_00018228	-1.07506	#NA	-1.27146	BGIOSGA013046	XP_004961359.1transcription factor BHLH089
TCONS_00011277	-1.07481	-1.5615	-0.272087	BGIOSGA012475	XP_006650853.1 tRNA:m(4)X modification enzyme TRM13 homolog
TCONS_00014245	-1.07477	-0.672577	-0.594104	BGIOSGA018741	XP_006652282.1 G-type lectin S-receptor-like serine/threonine-protein kinase At2g19130
TCONS_00035424	-1.07437	-1.0032	-0.663045	BGIOSGA006184	XP_006662472.2 probable trans-2-enoyl-CoA reductase, mitochondrial
TCONS_00004737	-1.07364	-1.55723	-1.11958	BGIOSGA027890	XP_015699263.1 calmodulin-binding receptor-like cytoplasmic kinase 2
TCONS_00013850	-1.0735	-1.5178	-1.20124	BGIOSGA025978	XP_002446210.1primary amine oxidase
TCONS_00011437	-1.07312	-1.18922	-1.16522	BGIOSGA021106	XP_015690736.1 transmembrane and coiled-coil domain-containing protein 4-like
TCONS_00030472	-1.07262	-0.588139	-0.691993	BGIOSGA031203	XP_006660926.1 auxin-responsive protein SAUR36-like
TCONS_00013024	-1.072	-0.901708	-0.406626	BGIOSGA028077	XP_006650461.1 probable LRR receptor-like serine/threonine-protein kinase At5g10290
TCONS_00019701	-1.07179	-0.480109	-1.44371	BGIOSGA012080	XP_004961376.1probable protein phosphatase 2C 50
TCONS_00034327	-1.07164	-1.54439	-2.78527	BGIOSGA024522	XP_004978713.1probable F-box protein At2g36090
TCONS_00030021	-1.07145	-1.0876	-0.340534	#N/A	#N/A
TCONS_00024333	-1.06958	-2.17622	-0.142727	BGIOSGA008524	XP_006658112.1PREDICTED: uncharacterized protein LOC102708450
TCONS_00004402	-1.06945	-0.39226	0.00355902	BGIOSGA001062	XP_006644713.1 60S ribosomal protein L18a
TCONS_00024656	-1.06932	-1.15515	-0.434225	BGIOSGA011409	XP_006657495.1 type I inositol polyphosphate 5-phosphatase 4-like
TCONS_00029819	-1.06864	0.0933733	-0.910074	BGIOSGA024631	XP_015696879.1 sugar transport protein 14
TCONS_00021672	-1.06829	-0.165491	-1.14988	BGIOSGA031459	XP_015694294.1 U-box domain-containing protein 57-like
TCONS_00008200	-1.06767	0.259498	-0.330184	BGIOSGA006237	XP_006647395.1 vesicle transport protein SFT2B
TCONS_00004078	-1.06756	-4.29636	-2.51676	BGIOSGA004123	XP_006646158.1 phospholipase A1-II 4-like
TCONS_00026845	-1.06752	-0.578721	-2.62555	BGIOSGA004041	XP_006660244.1 oligopeptide transporter 5-like
TCONS_00016503	-1.06746	-0.178679	-0.510528	BGIOSGA006138	XP_015691870.1PREDICTED: uncharacterized protein LOC102718540
TCONS_00021049	-1.06746	-3.17868	-2.73292	BGIOSGA013657	XP_006656565.1 pentatricopeptide repeat-containing protein At1g08070, chloroplastic-like
TCONS_00004680	-1.06721	-0.407382	-0.637155	BGIOSGA017893	XP_015690324.1 zinc finger protein CONSTANS-LIKE 2-like
TCONS_00028385	-1.0671	-0.384441	0.432955	BGIOSGA029461	XP_006660330.1 zinc finger protein CONSTANS-LIKE 15-like
TCONS_00010666	-1.067	0.366933	-0.425827	#N/A	#N/A

TCONS_00025672	-1.06631	-1.52141	-1.90242	BGIOSGA012614	XP_002463352.1zinc finger protein CONSTANS-LIKE 13
TCONS_00009150	-1.06615	0.14175	0.0711755	BGIOSGA011675	XP_015690942.1 cyclin-dependent protein kinase inhibitor EL2
TCONS_00013913	-1.06592	-1.74837	-1.0492	BGIOSGA026786	XP_009416161.1 tropinone reductase homolog At5g06060-like isoform X1
TCONS_00004733	-1.06524	0.207553	-1.92125	BGIOSGA000481	XP_003564618.1O-fucosyltransferase 19
TCONS_00000481	-1.06448	-1.55766	-3.64391	BGIOSGA026880	XP_003565894.2 cytochrome P450 71A1-like
TCONS_00035496	-1.06436	-3.63212	-2.40183	BGIOSGA018892	XP_015698595.1 probable apyrase 3
TCONS_00016512	-1.06402	0.804329	-2.15963	BGIOSGA017014	XP_004978197.1uncharacterized protein LOC101780625
TCONS_00023399	-1.06374	-2.66242	-1.89052	BGIOSGA005776	XP_006658425.1 DIMBOA UDP-glucosyltransferase BX8-like isoform X1
TCONS_00018642	-1.06325	-0.97424	-0.671198	BGIOSGA035689	XP_015693330.1 ATP-dependent DNA helicase Q-like SIM isoform X2
TCONS_00007251	-1.06224	0.154747	-0.526285	BGIOSGA032793	XP_006646795.1 pentatricopeptide repeat-containing protein At2g17033
TCONS_00002345	-1.06206	-1.6302	-1.16218	BGIOSGA037183	XP_002456788.1uncharacterized protein LOC8078680
TCONS_00030368	-1.0616	-2.65504	-2.66732	BGIOSGA026557	XP_015696797.1 inositol-tetrakisphosphate 1-kinase 6
TCONS_00006158	-1.06154	-2.18526	0.837364	#N/A	#N/A
TCONS_00024688	-1.06142	-2.88238	-2.54742	BGIOSGA022343	XP_015694827.1PREDICTED: uncharacterized protein ycf45
TCONS_00014184	-1.06096	0.143906	0.928678	BGIOSGA016339	XP_015691442.1 uridylate kinase
TCONS_00002647	-1.06064	-1.41286	-1.41159	BGIOSGA018919	XP_006645412.1 protein NETWORKED 2D-like isoform X1
TCONS_00022771	-1.06053	-0.0164114	1.70933	BGIOSGA030778	XP_012700796.1N-alpha-acetyltransferase 38-A, NatC auxiliary subunit
TCONS_00037433	-1.06045	-0.094622	-1.45562	BGIOSGA017471	XP_006664149.1 BTB/POZ domain-containing protein NPY4-like
TCONS_00006883	-1.05887	-1.85112	-1.08911	BGIOSGA009061	XP_006647899.1 aspartic proteinase-like protein 1
TCONS_00004903	-1.05814	-1.7346	-2.02847	BGIOSGA000318	XP_015688134.1PREDICTED: uncharacterized protein At3g49140
TCONS_00003061	-1.05795	-3.65963	-2.26132	BGIOSGA004187	XP_006645618.1 probable LRR receptor-like serine/threonine-protein kinase MRH1 isoform X2
TCONS_00033587	-1.05748	-1.51904	-2.4754	BGIOSGA006365	XP_006662968.1 nudix hydrolase 13, mitochondrial-like
TCONS_00013544	-1.05726	-1.1544	0.428801	BGIOSGA028831	XP_006650901.1PREDICTED: uncharacterized protein At4g14100-like
TCONS_00022593	-1.05671	-0.725906	-0.684496	BGIOSGA021330	XP_006657157.1 potassium transporter 10
TCONS_00014260	-1.05554	-2.64703	-1.53271	BGIOSGA016421	XP_006652286.1 probable helicase MAGATAMA 3
TCONS_00007306	-1.05506	1.20291	0.343981	BGIOSGA007176	XP_015689396.1 structural maintenance of chromosomes protein 3
TCONS_00019327	-1.05401	-0.505294	-0.426589	BGIOSGA014023	XP_006654428.1 NAC domain-containing protein 82-like isoform X1
TCONS_00027494	-1.05383	-1.52366	-1.45898	BGIOSGA027516	XP_006659017.1 SWR1 complex subunit 2
TCONS_00032784	-1.05355	-0.380483	-0.148651	BGIOSGA001480	XP_006662108.1 CSC1-like protein At1g32090
TCONS_00001842	-1.05314	0.390776	-0.0681711	BGIOSGA003742	XP_015692724.1 protein NUCLEAR FUSION DEFECTIVE 6, chloroplastic/mitochondrial-like isoform X4
TCONS_00029002	-1.05267	-0.525123	-1.43676	BGIOSGA020704	XP_006661230.1PREDICTED: uncharacterized protein LOC102705282
TCONS_00012236	-1.05142	-1.64325	-0.877054	BGIOSGA012583	XP_002467895.1uroporphyrinogen decarboxylase
TCONS_00005696	-1.05007	-2.02157	-0.91812	BGIOSGA018650	XP_004951412.1pollen-specific protein SF21
TCONS_00021648	-1.0492	-1.55308	-1.67594	BGIOSGA012682	XP_015694076.1 GDSL esterase/lipase At5g45910-like
TCONS_00000324	-1.04881	0.378411	0.873999	BGIOSGA002899	XP_006645574.1 probable U3 small nucleolar RNA-associated protein 7
TCONS_00014833	-1.04695	-1.82364	-0.959979	BGIOSGA017004	XP_006653713.2 probable purine permease 11
TCONS_00025500	-1.04695	-4.27797	-4.2	BGIOSGA020957	XP_006657931.1 COBRA-like protein 6
TCONS_00035465	-1.04662	-0.744864	-1.24061	BGIOSGA020050	XP_006662672.1 non-specific lipid-transfer protein 1
TCONS_00022438	-1.04604	-0.272241	-1.63103	BGIOSGA022324	XP_006656171.1 cysteine synthase-like
TCONS_00006773	-1.04547	-1.60371	-2.37103	BGIOSGA012007	XP_006647807.1 protein ASPARTIC PROTEASE IN GUARD CELL 1-like
TCONS_00031737	-1.04498	0.058962	-1.75495	BGIOSGA000752	XP_015694508.1 putative xyloglucan glycosyltransferase 10
TCONS_00018035	-1.04486	-2.2035	#NA	BGIOSGA016608	NP_001136636.1uncharacterized LOC100216764 precursor
TCONS_00007145	-1.0448	-2.30047	-0.60586	#N/A	#N/A
TCONS_00009660	-1.04443	-1.63914	-2.06019	#N/A	#N/A
TCONS_00016958	-1.04423	0.164884	-1.81438	BGIOSGA000359	XP_015699178.1 probable phosphoribosylformylglycinamidine synthase, chloroplastic/mitochondrial
TCONS_00036324	-1.04371	-0.777901	-1.2372	BGIOSGA020191	XP_006664153.2 probable disease resistance protein At4g33300
TCONS_00036322	-1.04317	0.0149768	-0.053754	BGIOSGA009539	XP_015698687.1 nucleobase-ascorbate transporter 3 isoform X1
TCONS_00026238	-1.04263	0.27372	-1.13641	BGIOSGA008406	XP_010235105.1uncharacterized protein LOC104583707
TCONS_00000005	-1.04204	0.68059	0.595581	BGIOSGA033994	XP_006662936.1 40S ribosomal protein S5
TCONS_00038473	-1.04191	-1.86705	-1.84704	#N/A	#N/A
TCONS_00020728	-1.04168	-1.92062	-3.03018	BGIOSGA008889	XP_004965389.1protein RADIALIS-like 3
TCONS_00001350	-1.04027	-0.378451	0.392907	BGIOSGA003982	XP_006646092.1 protein disulfide-isomerase LQY1, chloroplastic-like
TCONS_00015025	-1.04024	-1.07879	-0.653936	BGIOSGA027558	XP_015692188.1 transcription factor TGA4-like
TCONS_00028388	-1.03993	-1.25546	-0.697151	BGIOSGA029459	XP_006660331.1 ADP-ribosylation factor GTPase-activating protein AGD3-like
TCONS_00026103	-1.03988	0.256925	-0.190315	BGIOSGA027844	XP_003570916.1disease resistance protein RPP13

TCONS_00030111	-1.03977	-3.20978	-2.80245	BGIOSGA022065	XP_003578169.1uncharacterized protein LOC100827723
TCONS_00028752	-1.03917	-1.37583	-1.2813	BGIOSGA030674	XP_006660533.1 nucleobase-ascorbate transporter 2-like
TCONS_00029624	-1.03778	0.369896	-0.707872	BGIOSGA009935	XP_002466586.1coatomer subunit alpha-3
TCONS_00010972	-1.03759	-1.62758	-0.222944	BGIOSGA010891	XP_006650598.1 meiotic recombination protein SPO11-1
TCONS_00009251	-1.03598	-0.460547	0.588551	BGIOSGA019382	XP_002468567.1probable serine/threonine-protein kinase At4g35230
TCONS_00009495	-1.03571	-2.3153	-2.71008	BGIOSGA027743	XP_006649563.1 putative polyol transporter 1
TCONS_00031326	-1.03532	-4.38532	-1.73554	BGIOSGA018736	XP_006661865.2 uncharacterized membrane protein At1g06890-like isoform X1
TCONS_00023626	-1.03516	-0.893681	-2.39892	BGIOSGA034105	XP_006658538.2 5-pentadecatrienyl resorcinol O-methyltransferase-like
TCONS_00002689	-1.03507	0.199317	0.12442	BGIOSGA002530	XP_006643669.1PREDICTED: uncharacterized protein LOC102713580
TCONS_00012576	-1.03505	1.27611	-1.81422	BGIOSGA032106	XP_002466898.1protein DETOXIFICATION 40
TCONS_00014521	-1.03493	-3.21589	-1.81452	BGIOSGA004971	XP_006652464.1 cytochrome b561 and DOMON domain-containing protein At3g07570-like
TCONS_00020705	-1.03469	-0.724243	-1.07909	BGIOSGA037206	XP_015693418.1PREDICTED: uncharacterized protein LOC102716602
TCONS_00018097	-1.03453	-2.43996	-2.58502	BGIOSGA032703	XP_015693220.1 jacalin-related lectin 3-like
TCONS_00009337	-1.03441	-0.216549	-1.00845	BGIOSGA024282	XP_014752004.1monooxygenase 2
TCONS_00014422	-1.03437	0.306963	-0.49398	BGIOSGA030974	XP_006653509.1 ATP-dependent 6-phosphofructokinase 2
TCONS_00035315	-1.03405	-1.99461	-2.40166	BGIOSGA011754	XP_006663646.1 probable polygalacturonase
TCONS_00020517	-1.03402	-1.01541	-1.43827	BGIOSGA009622	XP_015694093.1 transcription factor bHLH49
TCONS_00006772	-1.03393	-0.714653	-1.23204	BGIOSGA022041	XP_015688719.1 protein ASPARTIC PROTEASE IN GUARD CELL 2-like
TCONS_00007370	-1.03368	-1.89553	-1.35817	BGIOSGA011629	XP_015689441.1 tyrosine-sulfated glycopeptide receptor 1-like
TCONS_00033254	-1.03363	-1.93493	0.632851	BGIOSGA021554	XP_006662915.1 anthranilate O-methyltransferase 2-like
TCONS_00034729	-1.03347	-1.83472	-2.57312	BGIOSGA003157	XP_006643998.1 shaggy-related protein kinase alpha
TCONS_00032401	-1.0334	0.336772	-0.496376	BGIOSGA031716	XP_006662430.2 phytosulfokine receptor 1-like
TCONS_00002359	-1.03306	-1.77767	-4.04153	BGIOSGA000067	XP_006645185.1 protein NRT1/ PTR FAMILY 8.1-like
TCONS_00035607	-1.0328	-3.21858	#NA	BGIOSGA029105	XP_003577030.3cationic amino acid transporter 6, chloroplastic
TCONS_00009914	-1.03247	-1.21898	-0.474818	BGIOSGA015434	NP_001144260.1uncharacterized LOC100277134
TCONS_00020511	-1.03234	-2.90161	-2.13608	BGIOSGA002299	XP_015693546.1 protein NSP-INTERACTING KINASE 1-like
TCONS_00020646	-1.03167	-1.21999	-0.999451	BGIOSGA012562	XP_015694280.1 receptor-like protein kinase HERK 1
TCONS_00018341	-1.031	-2.52286	-1.01686	BGIOSGA008058	XP_010235741.1uncharacterized protein LOC100846116 isoform X2
TCONS_00009160	-1.03023	-1.38346	-1.1908	BGIOSGA002186	XP_006649248.1 homeobox-leucine zipper protein HOX10
TCONS_00009761	-1.02921	-0.116898	-0.0194555	BGIOSGA015860	XP_006651247.1 RNA polymerase sigma factor sigB-like
TCONS_00007042	-1.02801	-3.04301	-2.55066	BGIOSGA023962	XP_006648064.1 heparanase-like protein 3
TCONS_00024817	-1.02778	-1.08712	-1.88024	BGIOSGA036789	XP_002437684.1uncharacterized protein LOC8066879
TCONS_00037135	-1.02774	-3.7201	-2.10139	BGIOSGA037332	NP_001266910.1fatty acid alpha-dioxygenase
TCONS_00027894	-1.02755	0.230538	0.425755	BGIOSGA027106	XP_006659456.1 30S ribosomal protein S17
TCONS_00035378	-1.02706	-0.75564	0.06447	BGIOSGA030535	XP_003565888.1probable WRKY transcription factor 41
TCONS_00033797	-1.02651	-0.731781	-3.22476	BGIOSGA034804	XP_003577385.1mannose-6-phosphate isomerase 1
TCONS_00002168	-1.02648	-1.04559	-1.87252	BGIOSGA012197	XP_006645009.1 aldo-keto reductase family 4 member C9-like isoform X1
TCONS_00013972	-1.02632	-2.01196	-1.66015	BGIOSGA010917	XP_006656087.2 7-hydroxymethyl chlorophyll a reductase, chloroplastic
TCONS_00016592	-1.02572	0.381769	0.173152	BGIOSGA035274	XP_021319511.1uncharacterized protein LOC8072320 isoform X1
TCONS_00008639	-1.02537	-1.67876	0.0525419	BGIOSGA010646	XP_006647741.1 polyamine transporter PUT1 isoform X1
TCONS_00015785	-1.02513	-0.274637	-0.415744	BGIOSGA015198	XP_006653331.1 mediator of RNA polymerase II transcription subunit 30-like
TCONS_00011835	-1.02433	-0.912312	0.109867	BGIOSGA031252	XP_006649679.1 squalene monooxygenase-like
TCONS_00013498	-1.02409	0.499049	-0.609172	BGIOSGA005643	XP_006650869.1 G-type lectin S-receptor-like serine/threonine-protein kinase At1g34300 isoform X1
TCONS_00016279	-1.02402	-2.03047	-2.21157	BGIOSGA005990	XP_015692195.1PREDICTED: uncharacterized protein LOC102711878 isoform X2
TCONS_00017746	-1.02169	-0.788464	-0.0999919	BGIOSGA019808	XP_004962183.1methyltransferase-like protein 13
TCONS_00017917	-1.02096	-2.45829	-1.5904	BGIOSGA019958	XP_006654491.1 probable anion transporter 2, chloroplastic
TCONS_00000052	-1.02069	-0.79857	-2.80751	BGIOSGA012808	XP_004967963.1transcription factor bHLH168
TCONS_00022908	-1.02065	-0.345344	-0.21527	BGIOSGA032500	XP_002439025.1probable serine/threonine-protein kinase PBL19
TCONS_00030173	-1.0196	-1.85523	-1.00564	BGIOSGA014767	XP_015696469.1 probable carboxylesterase 2
TCONS_00033122	-1.01879	-1.89434	-1.71632	BGIOSGA034360	XP_006662794.2 probable NAD kinase 2, chloroplastic isoform X2
TCONS_00006566	-1.01857	-0.879579	-0.951256	BGIOSGA004589	XP_010235782.2phospholipase A1-Ibeta2, chloroplastic
TCONS_00031414	-1.01851	-2.62729	-1.48882	BGIOSGA036042	XP_004982697.1calcium uniporter protein 2, mitochondrial
TCONS_00021236	-1.01763	-0.170855	-0.607714	BGIOSGA002704	XP_015693719.1 probable alpha-glucosidase Os06g0675700

transcriptome

TCONS_00013602	-1.01718	-0.940354	-1.04031	BGIOSGA000248	XP_006653096.1PREDICTED: uncharacterized protein LOC102716494 isoform X2
TCONS_00025692	-1.01702	0.31709	-1.15731	BGIOSGA008455	XP_015695234.1 serine/arginine-rich-splicing factor SR34 isoform X1
TCONS_00013208	-1.01662	-0.69393	-1.17107	BGIOSGA024943	XP_006650619.1 lamin-like protein
TCONS_00002546	-1.01654	-0.274111	-0.750181	BGIOSGA033646	XP_009417414.1 disease resistance RPP13-like protein 4
TCONS_00019750	-1.0165	0.688835	0.0553763	BGIOSGA004231	XP_006654750.1 guanine nucleotide-binding protein subunit beta-like protein B
TCONS_00015838	-1.0163	-3.04047	-1.15705	BGIOSGA006349	XP_006653368.1PREDICTED: uncharacterized protein At4g22758-like, partial
TCONS_00010254	-1.016	-4.68878	-2.66398	BGIOSGA012819	XP_015690546.1 protein GOS9-like
TCONS_00004501	-1.01517	-0.478668	-0.351531	BGIOSGA017612	XP_004970165.1uncharacterized protein LOC101784875
TCONS_00003359	-1.01472	-2.09474	-1.2874	BGIOSGA004259	XP_015698032.1 ABC transporter B family member 21-like
TCONS_00008419	-1.01415	-2.40226	-1.0646	BGIOSGA006001	XP_006647543.1 phenylalanine ammonia-lyase-like
TCONS_00014800	-1.01308	-1.29797	0.166116	BGIOSGA004385	XP_006653698.2 GDSL esterase/lipase At5g41890
TCONS_00006106	-1.01302	-0.347463	-0.35486	BGIOSGA015231	XP_006653313.2 60S ribosomal protein L35
TCONS_00004471	-1.01279	-0.595817	-1.15809	BGIOSGA037885	XP_006644767.1 protein CROWDED NUCLEI 4-like
TCONS_00034588	-1.01265	-1.50374	-1.20448	BGIOSGA029627	XP_008670701.1probable carboxylesterase 18
TCONS_00030075	-1.01229	#NA	-0.667454	BGIOSGA029730	XP_015696355.1 WAT1-related protein At4g30420-like
TCONS_00001760	-1.01211	-3.39981	-1.40488	BGIOSGA004396	XP_004981337.2uncharacterized protein LOC101764708
TCONS_00037561	-1.01207	0.387189	0.164522	BGIOSGA035800	XP_003577207.1protein SINE1
TCONS_00015883	-1.01199	-2.67181	-1.46573	BGIOSGA015103	XP_022683883.1protein SET DOMAIN GROUP 41 isoform X2
TCONS_00013827	-1.01193	-0.815064	-0.530855	BGIOSGA016004	XP_006644202.1 putative methyltransferase C9orf114 isoform X1
TCONS_00012218	-1.01071	-1.35149	-1.53928	BGIOSGA033241	XP_015690635.1 valine--tRNA ligase, mitochondrial 1-like
TCONS_00002785	-1.00983	-2.90614	-1.37943	BGIOSGA002432	XP_004968147.2L-Ala-D/L-amino acid epimerase
TCONS_00007332	-1.00898	-0.433842	-0.382641	BGIOSGA023558	XP_006648300.1 L-arabinokinase
TCONS_00008275	-1.00844	-4.6965	-2.49026	BGIOSGA008935	XP_006647434.1 fimbrin-5-like
TCONS_00026623	-1.00834	-1.08981	-1.09953	BGIOSGA028693	NP_001183889.1uncharacterized LOC100502482
TCONS_00014850	-1.00804	-3.09281	-2.25878	BGIOSGA008119	XP_006652726.1 WEB family protein At5g16730, chloroplastic
TCONS_00014773	-1.00791	-0.259629	-0.604551	BGIOSGA016948	XP_006652665.1 RING-H2 finger protein ATL68-like
TCONS_00010053	-1.00778	-3.94431	-2.24009	BGIOSGA000247	XP_006651362.2 bidirectional sugar transporter SWEET16
TCONS_00032818	-1.00748	0.501249	-1.67953	BGIOSGA026049	XP_002441676.1protein NUCLEAR FUSION DEFECTIVE 4
TCONS_00010245	-1.00741	-1.49138	-0.663026	BGIOSGA010584	XP_006651476.1PREDICTED: uncharacterized protein C630.12
TCONS_00017832	-1.00618	0.128573	0.135414	BGIOSGA000369	XP_006654418.1 serine/arginine repetitive matrix protein 1-like
TCONS_00033841	-1.00599	-0.237599	-0.304727	BGIOSGA037430	XP_006663048.1 DNA repair protein RAD51 homolog A
TCONS_00011608	-1.00561	-0.738267	-1.26141	BGIOSGA011286	XP_006649437.1 sulfate transporter 3.1-like
TCONS_00028045	-1.00545	-2.25299	-1.40928	BGIOSGA026945	XP_009351719.2 nucleolin 1
TCONS_00001847	-1.0049	-2.82393	-1.4229	BGIOSGA004482	XP_006644728.1PREDICTED: uncharacterized protein LOC102722258
TCONS_00032155	-1.00454	-1.82438	0.514816	BGIOSGA037582	XP_010665455.1 F-box/kelch-repeat protein At3g23880
TCONS_00026764	-1.00429	-0.446186	-1.80774	BGIOSGA001294	XP_015695635.1 radial spoke head 1 homolog
TCONS_00004759	-1.00356	-0.732511	-0.809553	BGIOSGA034536	XP_006645049.1 cytochrome P450 94C1
TCONS_00028364	-1.00345	-2.41072	-1.01145	BGIOSGA003538	XP_006660316.2PREDICTED: uncharacterized protein LOC102708176
TCONS_00018972	-1.00334	0.174113	-1.20436	BGIOSGA018393	XP_024313771.1putative pentatricopeptide repeat-containing protein At1g19290
TCONS_00021228	-1.00326	-1.12472	-0.138406	BGIOSGA038150	XP_015694341.1 BTB/POZ domain-containing protein At1g04390
TCONS_00037758	-1.00315	-1.3124	-0.26359	BGIOSGA021667	XP_004975716.1putative 12-oxophytodienoate reductase 13 isoform X1
TCONS_00022134	-1.0031	-0.44046	0.134436	BGIOSGA021369	XP_006653019.2 protein S-acyltransferase 11
TCONS_00004374	-1.00294	-1.01461	-0.592824	BGIOSGA000846	XP_006646313.1 tryptophan--tRNA ligase, chloroplastic/mitochondrial
TCONS_00002727	-1.00285	0.710828	-0.0205652	BGIOSGA020894	XP_006643712.1 eukaryotic translation initiation factor 3 subunit A
TCONS_00037056	-1.00219	1.23961	0.71957	BGIOSGA035211	XP_006663976.1 serine hydroxymethyltransferase 4-like
TCONS_00028249	-1.00176	-1.06299	-1.12037	BGIOSGA006495	XP_006659550.1 probable protein phosphatase 2C 66
TCONS_00024311	-1.00149	-0.712	-0.951687	BGIOSGA025380	XP_006658087.2 DNA polymerase zeta catalytic subunit
TCONS_00018219	-1.00148	-1.48215	0.224458	BGIOSGA020278	XP_015693167.1 probable ADP,ATP carrier protein At5g56450
TCONS_00006722	-1.00144	-1.53135	-1.42992	BGIOSGA001223	XP_006647771.1PREDICTED: uncharacterized protein LOC102713601
TCONS_00026100	-1.00024	-3.16775	-0.882849	BGIOSGA019490	XP_016648188.1 probable LRR receptor-like serine/threonine-protein kinase At1g56140
TCONS_00011300	-0.998929	0.847065	-1.46089	BGIOSGA003118	XP_006650870.1 ammonium transporter 3 member 2
TCONS_00005917	-0.997927	0.688192	0.562312	BGIOSGA000047	XP_002468590.1cytochrome c oxidase assembly protein COX19
TCONS_00026164	-0.997699	0.191122	-0.558016	BGIOSGA017067	XP_004972521.160S ribosomal protein L7-3
TCONS_00009853	-0.997309	0.112965	0.51087	BGIOSGA004886	XP_006649915.2 transcription factor bHLH140

transcriptome

TCONS_00036177	-0.997215	-2.56052	-2.13994	BGIOSGA037532	XP_006664083.2PREDICTED: uncharacterized protein LOC102714792
TCONS_00003826	-0.996838	-2.41012	-0.493213	BGIOSGA034932	XP_004969001.1transcription factor bHLH87
TCONS_00029779	-0.996712	-0.262631	0.165919	BGIOSGA016118	XP_006661092.1 flowering time control protein FPA
TCONS_00002931	-0.996386	0.163308	-0.226221	BGIOSGA007407	XP_006643814.1 phospholipase D alpha 1
TCONS_00006602	-0.996182	0.102952	-0.426796	BGIOSGA008769	XP_015688847.1 adenine nucleotide transporter BT1, chloroplastic/amyloplastic/mitochondrial isoform X2
TCONS_00027560	-0.995918	-0.102553	-1.14324	#N/A	#N/A
TCONS_00032901	-0.993968	-0.734228	0.767847	BGIOSGA036931	XP_015698767.1 pentatricopeptide repeat-containing protein At5g43790-like isoform X5
TCONS_00036410	-0.993861	-0.866024	-0.93779	BGIOSGA013851	XP_022680515.1transcription factor PIF4 isoform X1
TCONS_00033738	-0.992783	0.312516	-0.706118	BGIOSGA025246	XP_006663003.1 ETO1-like protein 1
TCONS_00028466	-0.992127	0.313882	-0.41723	BGIOSGA031298	XP_015695696.1PREDICTED: uncharacterized protein At2g27730, mitochondrial-like, partial
TCONS_00009584	-0.992126	-2.6122	-0.858236	#N/A	XP_010238423.1uncharacterized protein LOC104584713
TCONS_00029001	-0.991737	-1.48886	0.0856244	BGIOSGA030758	XP_006661229.1 endoribonuclease Dicer homolog 4-like isoform X1
TCONS_00030833	-0.991682	-0.853941	-1.19127	BGIOSGA037797	XP_006661673.2 protein DETOXIFICATION 16-like
TCONS_00006783	-0.991464	1.23407	-0.547014	BGIOSGA021399	XP_015689666.1 zinc finger protein CONSTANS-LIKE 9 isoform X1
TCONS_00030108	-0.991168	-0.647337	-0.310931	#N/A	XP_021314494.1uncharacterized protein LOC8072443
TCONS_00004457	-0.991046	-1.12647	-1.12393	BGIOSGA028069	XP_004970074.1clp protease adapter protein ClpF, chloroplastic
TCONS_00015881	-0.990609	-0.653411	-0.964672	BGIOSGA005484	XP_003579736.2RNA-binding motif protein, X-linked-like-3
TCONS_00010601	-0.989953	-0.109603	-0.158347	BGIOSGA025179	XP_008446394.1 transcription elongation factor spt5-like
TCONS_00006365	-0.988873	0.014588	0.463424	#N/A	#N/A
TCONS_00022950	-0.988867	-1.50916	-0.95942	BGIOSGA020497	XP_006657373.1 protease 2
TCONS_00002337	-0.98804	-0.17997	-0.711007	BGIOSGA004979	XP_006645167.1PREDICTED: uncharacterized protein LOC102708665
TCONS_00002092	-0.987782	0.0387149	-0.667252	BGIOSGA034639	XP_015688323.1PREDICTED: uncharacterized protein LOC102702810
TCONS_00022691	-0.98751	-0.508112	-0.660378	BGIOSGA028256	XP_006656322.2 villin-4-like isoform X1
TCONS_00015333	-0.987492	0.597816	-2.16454	BGIOSGA015653	XP_015691362.1 probable DNA double-strand break repair Rad50 ATPase
TCONS_00008428	-0.987428	-0.669206	-0.232485	BGIOSGA035852	XP_006647551.1 phytosulfokine receptor 1-like
TCONS_00030474	-0.987403	-1.33888	-0.499557	BGIOSGA012392	XP_006660925.1 auxin-responsive protein SAUR36-like
TCONS_00007566	-0.987303	-1.57235	#NA	BGIOSGA006917	XP_004951526.1F-box/kelch-repeat protein At1g55270
TCONS_00011871	-0.987073	-1.10796	-0.356066	BGIOSGA017012	XP_015698346.1 protein misato homolog 1
TCONS_00025772	-0.987003	-1.4028	-4.16577	BGIOSGA011347	XP_004972397.1casparian strip membrane protein 2
TCONS_00035311	-0.985779	-1.72628	-0.905799	BGIOSGA033603	XP_015697736.1 alkylated DNA repair protein alkB homolog 8
TCONS_00031104	-0.984594	-0.726596	-0.193164	BGIOSGA033702	XP_006661778.1 GDP-mannose 3,5-epimerase 1
TCONS_00021225	-0.984427	-0.715597	0.20617	BGIOSGA023449	XP_004966296.2twinkle homolog protein, chloroplastic/mitochondrial isoform X1
TCONS_00028973	-0.984398	-2.57602	-1.17228	BGIOSGA015228	XP_003579585.1protein XRI1 isoform X2
TCONS_00004082	-0.984128	-0.769009	-3.17295	BGIOSGA001104	XP_012701771.1uncharacterized protein LOC101769092 isoform X2
TCONS_00024747	-0.98407	-1.57644	-0.365741	BGIOSGA030778	XP_006664896.1 leucine-rich repeat receptor-like tyrosine-protein kinase PXC3
TCONS_00016661	-0.983545	0.00786343	0.217916	BGIOSGA014300	XP_006652862.1 APO protein 4, mitochondrial
TCONS_00003164	-0.983497	-1.25523	-1.05904	BGIOSGA002056	XP_015688094.1 protein DEFECTIVE IN MERISTEM SILENCING 3
TCONS_00028434	-0.983474	-2.57719	-3.17458	BGIOSGA024777	XP_006659659.2 probable carboxylesterase 8
TCONS_00009358	-0.983361	0.775522	-0.601733	BGIOSGA032942	XP_015691182.1 formin-like protein 20
TCONS_00005454	-0.9828	-1.47431	-1.14306	BGIOSGA007563	XP_006649634.1 GDP-mannose transporter GONST3 isoform X2
TCONS_00002739	-0.982486	-1.11977	-0.311462	BGIOSGA002484	XP_006664941.1 Bowman-Birk type bran trypsin inhibitor-like
TCONS_00019669	-0.98248	-2.25651	-2.59208	BGIOSGA001676	XP_015688234.1 indole-3-pyruvate monooxygenase YUCCA6-like
TCONS_00006232	-0.982321	-0.659596	-0.57125	BGIOSGA010030	XP_006647356.2 myosin-8-like
TCONS_00026716	-0.981725	-0.485298	-0.0919027	BGIOSGA006729	NP_001309038.1uncharacterized LOC100192502
TCONS_00033720	-0.9817	-0.772068	-0.00905277	BGIOSGA021101	XP_015698120.1PREDICTED: uncharacterized protein LOC102709967 isoform X1
TCONS_00025809	-0.981606	-0.284921	-0.786894	BGIOSGA010816	XP_006659059.1 transcription factor JUNGBRUNNEN 1-like
TCONS_00024108	-0.981592	-3.65867	-1.52174	BGIOSGA026143	XP_004978851.1dirigent protein 21
TCONS_00023125	-0.981583	-0.120139	-0.304799	BGIOSGA018187	XP_020396804.1transcription factor PCF6
TCONS_00023296	-0.981338	-0.813623	-0.464564	BGIOSGA008021	XP_015694551.1 F-box protein CPR30-like
TCONS_00011828	-0.981217	#NA	-1.59521	BGIOSGA014541	XP_006649669.1 probable receptor-like serine/threonine-protein kinase At4g34500
TCONS_00031875	-0.981151	-0.585241	-0.427567	BGIOSGA032256	XP_006366028.1PREDICTED: uncharacterized protein LOC102601474
TCONS_00005746	-0.981043	-1.18793	-1.72117	BGIOSGA005164	XP_006648492.1PREDICTED: uncharacterized protein LOC102717409
TCONS_00016438	-0.980766	-0.836841	-0.779705	BGIOSGA025669	XP_015692045.1 protein argonaute 1B isoform X2
TCONS_00010603	-0.979498	0.640205	-0.377058	BGIOSGA037825	XP_003561753.1protein DYAD
TCONS_00021331	-0.978845	-0.555525	-0.932263	BGIOSGA033901	XP_006656453.2 auxin response factor 19
TCONS_00012065	-0.977572	-0.603793	2.68641	BGIOSGA021489	XP_006651276.2 asparagine synthetase [glutamine-hydrolyzing] 1

transcriptome

TCONS_00013469	-0.976931	0.86205	-0.526896	BGIOSGA018395	XP_006650837.1 bifunctional epoxide hydrolase 2-like isoform X2
TCONS_00013165	-0.97572	-0.339844	-0.123205	BGIOSGA009819	XP_006650576.1 tRNA (guanine-N(7)-)-methyltransferase non-catalytic subunit wdr4
TCONS_00006578	-0.975572	-0.903443	-0.320839	BGIOSGA014816	XP_006647627.1 dnaJ protein homolog 2-like isoform X2
TCONS_00006720	-0.97509	-3.68143	-1.54552	BGIOSGA022921	XP_015689596.1 protein RADIALIS-like 3
TCONS_00018417	-0.975059	-1.83632	0.189532	BGIOSGA020470	XP_015692831.1 RNA polymerase sigma factor sigC
TCONS_00027451	-0.975045	0.421054	0.640536	BGIOSGA030419	XP_015696148.1 DNA polymerase I A, chloroplastic-like
TCONS_00022462	-0.974773	-0.119887	0.287962	BGIOSGA007798	XP_006657074.2 U-box domain-containing protein 34
TCONS_00025902	-0.974658	-0.997912	-2.03367	BGIOSGA002232	XP_003573373.1transcription factor MYBS3
TCONS_00026566	-0.974215	-1.10294	-1.66873	BGIOSGA027900	XP_006662182.1 F-box/LRR-repeat protein 4-like
TCONS_00014920	-0.973787	-3.15198	-1.87539	BGIOSGA032093	XP_006653760.1 36.4 kDa proline-rich protein-like
TCONS_00023641	-0.973501	-2.25799	-2.0045	BGIOSGA033358	XP_015694626.1 protein CURVATURE THYLAKOID 1B, chloroplastic
TCONS_00025531	-0.973295	-1.16596	0.669804	BGIOSGA019930	XP_015694870.1 ethylene-responsive transcription factor ERF073-like
TCONS_00001394	-0.972831	-4.5134	-2.11524	BGIOSGA019208	XP_006646123.1 kinesin-13A isoform X1
TCONS_00027183	-0.972498	-0.446123	-1.26622	BGIOSGA027816	XP_015695830.1 uncharacterized N-acetyltransferase ycf52
TCONS_00016307	-0.971687	-1.2201	-1.24644	BGIOSGA026416	XP_024312165.1(+)-neomenthol dehydrogenase-like isoform X2
TCONS_00005757	-0.970199	-0.232946	-0.31026	BGIOSGA007870	XP_006648498.1PREDICTED: uncharacterized protein LOC102719650
TCONS_00011034	-0.969598	-1.08103	-0.0529487	BGIOSGA031108	XP_006651853.1 protein PHLOEM PROTEIN 2-LIKE A10-like
TCONS_00031097	-0.969424	0.396631	-0.735238	BGIOSGA032920	XP_015697098.1 chitinase 2-like
TCONS_00005561	-0.969269	-0.371241	0.263542	BGIOSGA003306	XP_015688476.1 probable WRKY transcription factor 60
TCONS_00028049	-0.968882	-0.223631	-0.408677	BGIOSGA036512	XP_006659425.1 dihydrolipoyllysine-residue acetyltransferase component 4 of pyruvate dehydrogenase complex, chloroplastic
TCONS_00022885	-0.968631	-0.674789	1.2447	BGIOSGA020559	XP_015693405.1 WAT1-related protein At3g45870-like
TCONS_00027352	-0.968053	-0.250432	-0.656079	BGIOSGA027646	XP_006659118.2 HEAT repeat-containing protein 6
TCONS_00015764	-0.967819	-2.5234	-2.59449	BGIOSGA006417	XP_012703058.1F-box/kelch-repeat protein At1g67480
TCONS_00009355	-0.967712	-0.794126	-0.228361	BGIOSGA012049	XP_004985625.1protein SUPPRESSOR OF GENE SILENCING 3 homolog
TCONS_00031548	-0.96666	-1.65934	-5.1162	BGIOSGA030297	XP_006662008.1 aspartic proteinase nepenthesin-1-like
TCONS_00000817	-0.966256	-1.516	-0.294552	BGIOSGA003405	XP_006644131.1 craniofacial development protein 1
TCONS_00002686	-0.966206	-2.40644	-1.182	BGIOSGA016276	XP_006643662.1 peptidyl-prolyl cis-trans isomerase CYP26-2, chloroplastic
TCONS_00014379	-0.965331	-1.3819	-1.07474	BGIOSGA008481	XP_015692123.1PREDICTED: uncharacterized protein LOC102712799
TCONS_00037656	-0.96527	-2.41174	-1.23585	BGIOSGA030520	XP_015689456.1 26S proteasome non-ATPase regulatory subunit 2 homolog A-like
TCONS_00000313	-0.965015	-0.344858	-0.820162	BGIOSGA002887	XP_015695852.1 putative disease resistance protein RGA3
TCONS_00005305	-0.964749	0.411014	0.182857	BGIOSGA006261	XP_015688435.1 UTP:RNA uridylyltransferase 1
TCONS_00007710	-0.964509	-0.206945	1.06315	BGIOSGA032313	XP_010233742.1putative F-box protein At3g16210
TCONS_00021042	-0.964374	-1.08286	-0.199069	BGIOSGA016289	XP_002437294.1uncharacterized protein LOC8083462
TCONS_00010157	-0.964345	0.398427	0.00522099	BGIOSGA023845	XP_006650124.1 pyruvate dehydrogenase (acetyl-transferring) kinase, mitochondrial-like isoform X1
TCONS_00031674	-0.964274	0.306229	-0.537133	BGIOSGA032547	XP_012698381.1 transcription factor MYC2-like
TCONS_00016296	-0.963702	-0.327697	-0.525334	BGIOSGA005968	XP_015691524.1 ferredoxin-thioredoxin reductase, variable chain
TCONS_00010859	-0.963627	-2.73162	0.128076	BGIOSGA037808	NP_001150853.1glucan endo-1,3-beta-glucosidase 7 precursor
TCONS_00002351	-0.963347	-0.42856	-1.23144	BGIOSGA033344	XP_002456792.2transcription factor bHLH128
TCONS_00020788	-0.963113	-0.0207675	-0.0695921	BGIOSGA030330	XP_006656996.1 protein DGCR14
TCONS_00001139	-0.963085	-1.26893	-0.58681	BGIOSGA003764	XP_006644265.1PREDICTED: uncharacterized protein LOC102703434
TCONS_00034100	-0.962436	0.340136	1.65817	BGIOSGA035743	XP_004978278.1scarecrow-like protein 33
TCONS_00036170	-0.962242	0.356487	-0.209294	#N/A	#N/A
TCONS_00034569	-0.961991	-0.341353	-0.16547	BGIOSGA035884	XP_006663315.2 PHD finger protein At1g33420-like
TCONS_00001325	-0.961503	-0.795568	-1.99817	BGIOSGA004607	XP_014755012.1amino-acid permease BAT1 homolog isoform X2
TCONS_00011376	-0.961315	-0.779651	-0.511667	BGIOSGA001314	XP_006652045.1 probable cytokinin riboside 5'-monophosphate phosphoribohydrolase LOGL5
TCONS_00034419	-0.960265	-1.0105	-1.16891	BGIOSGA036542	XP_006662779.1 protein-tyrosine-phosphatase PTP1-like isoform X1
TCONS_00000557	-0.959474	-1.32121	-1.4596	BGIOSGA020406	XP_006643991.1 cytosolic isocitrate dehydrogenase [NADP]-like
TCONS_00013982	-0.95889	-1.14302	-1.92392	BGIOSGA021442	XP_002462695.1UDP-glycosyltransferase 85A5
TCONS_00029483	-0.958744	-1.16602	-1.87383	BGIOSGA031237	XP_006660956.1 uncharacterized transporter YBR287W-like
TCONS_00013081	-0.957389	-1.09222	-1.90739	BGIOSGA025996	XP_015690044.1 vacuolar protein sorting-associated protein 62-like
TCONS_00036096	-0.957225	-1.47203	-1.26057	BGIOSGA009740	XP_009405891.2 40S ribosomal protein S29
TCONS_00034372	-0.956353	-2.30795	-1.15719	BGIOSGA034766	XP_004978774.1abscisic stress-ripening protein 3

TCONS_00019467	-0.955465	-1.8641	-3.18071	BGIOSGA027689	XP_006654530.1 protein trichome birefringence-like 28
TCONS_00011572	-0.955237	-2.31994	-2.92027	BGIOSGA003321	XP_015690286.1 WAT1-related protein At2g39510-like
TCONS_00023929	-0.955117	-0.673849	0.181001	BGIOSGA005071	XP_015694899.1 probable arabinosyltransferase ARAD1
TCONS_00007742	-0.95464	0.722803	0.352224	BGIOSGA031010	NP_001336967.1uncharacterized LOC100383799 isoform 1
TCONS_00000348	-0.954199	-0.747341	-1.15586	BGIOSGA002924	XP_006643857.1 probable 2-oxoglutarate-dependent dioxygenase At3g49630 isoform X1
TCONS_00030252	-0.954112	-0.991428	0.0019048	#N/A	#N/A
TCONS_00037552	-0.953327	-0.281474	-0.539548	BGIOSGA021834	XP_006664757.1PREDICTED: uncharacterized protein LOC102721330
TCONS_00024621	-0.95327	-0.541967	-2.45856	#N/A	#N/A
TCONS_00022974	-0.953031	-1.01518	-0.672155	BGIOSGA025008	XP_006658201.1 photosynthetic NDH subunit of lumenal location 3, chloroplastic
TCONS_00010754	-0.952988	-1.39395	-1.51485	BGIOSGA013378	XP_006650437.2 nuclear transcription factor Y subunit A-4-like
TCONS_00022838	-0.952588	-0.279795	-0.652908	BGIOSGA039024	XP_006657287.2 protein STAR1 isoform X2
TCONS_00001376	-0.951836	-0.705101	-0.786951	BGIOSGA011606	XP_015697882.1 probable polygalacturonase
TCONS_00013241	-0.951273	-1.54449	-0.293613	BGIOSGA010900	XP_003558752.1transcription factor MYB4
TCONS_00008591	-0.951148	0.431106	-0.589378	BGIOSGA005837	XP_006647692.1 protein TRANSPARENT TESTA GLABRA 1-like
TCONS_00015972	-0.951148	-1.77142	-0.867997	#N/A	XP_014660848.2F-box protein At5g03100
TCONS_00012407	-0.950959	#NA	#NA	BGIOSGA026145	XP_004984090.1F-box only protein 13
TCONS_00016042	-0.950936	-2.86684	0.236065	BGIOSGA025174	XP_003579927.1peroxidase 31
TCONS_00021031	-0.950802	-1.86701	-0.657352	BGIOSGA012642	XP_015693889.1 zinc finger protein WIP2
TCONS_00035437	-0.950756	-2.58108	-2.05586	BGIOSGA000250	XP_003577897.2protein DWARF 53
TCONS_00006307	-0.950052	-1.12954	-1.57001	BGIOSGA015502	XP_004956311.1ent-copalyl diphosphate synthase 2 isoform X2
TCONS_00008245	-0.950013	0.714745	0.295883	BGIOSGA032386	XP_006648784.1 cell number regulator 2-like isoform X1
TCONS_00001413	-0.949993	-0.224175	-0.379253	BGIOSGA009879	NP_001105724.2siroheme uroporphyrinogen methyltransferase 1
TCONS_00021538	-0.949755	-2.10627	-1.8601	BGIOSGA016958	XP_006655746.1 cytochrome P450 704C1
TCONS_00000757	-0.949706	-0.568711	-0.71559	BGIOSGA003147	XP_006645808.1 heavy metal-associated isoprenylated plant protein 26-like
TCONS_00009772	-0.949555	-1.56863	-2.00677	BGIOSGA005358	XP_006651249.1 telomere repeat-binding protein 5-like
TCONS_00018153	-0.949555	-1.13268	-2.48415	BGIOSGA007831	XP_008649914.1uncharacterized protein LOC103630644
TCONS_00022957	-0.949307	-3.11116	-1.72357	BGIOSGA012281	XP_006657378.2 sulfoquinovosyl transferase SQD2-like
TCONS_00004661	-0.949298	-0.73141	-0.652473	BGIOSGA020026	XP_006644948.1PREDICTED: uncharacterized protein LOC102716267
TCONS_00013423	-0.949208	-1.93613	-2.12074	BGIOSGA021588	XP_004981275.1protein trichome birefringence-like 33 isoform X1
TCONS_00024127	-0.948839	-1.32563	-1.11898	BGIOSGA005974	XP_015694729.1 GLABRA2 expression modulator-like
TCONS_00035423	-0.948807	-1.51325	-0.803251	BGIOSGA015373	XP_024311120.1phototropin-1A isoform X4
TCONS_00013313	-0.948698	0.42161	0.402235	BGIOSGA025523	XP_006650706.1 probable aldehyde oxidase 2
TCONS_00014326	-0.948613	0.415636	-0.0106984	BGIOSGA030461	XP_015691988.1 peptidyl-prolyl cis-trans isomerase FKBP53
TCONS_00004357	-0.947919	-1.33459	-0.986418	BGIOSGA002938	XP_006644676.1 ATP-dependent 6-phosphofructokinase 6-like
TCONS_00028956	-0.947721	-0.467695	#NA	BGIOSGA006930	XP_006660605.1 vacuolar iron transporter 1.2
TCONS_00021717	-0.947678	-0.118317	-1.04611	BGIOSGA032863	XP_006655844.1 putative clathrin assembly protein At5g35200
TCONS_00019413	-0.946592	-0.866151	-0.491423	BGIOSGA038061	XP_006654493.1 phosphatidylinositol:ceramide inositolphosphotransferase-like isoform X1
TCONS_00017020	-0.9465	-7.19434	-2.15341	BGIOSGA019825	XP_006653984.1 125 kDa kinesin-related protein-like
TCONS_00015917	-0.946399	-1.29861	-0.883529	BGIOSGA006274	XP_015692355.1PREDICTED: uncharacterized protein LOC102714282 isoform X2
TCONS_00000708	-0.946273	-2.74371	-2.15151	BGIOSGA030133	XP_006644084.1 CBL-interacting protein kinase 1
TCONS_00014912	-0.944515	-0.731553	1.07664	BGIOSGA022759	XP_006652802.1PREDICTED: uncharacterized protein LOC102721266
TCONS_00011631	-0.944294	-1.5347	0.155106	BGIOSGA011379	XP_006649462.1PREDICTED: uncharacterized protein LOC102722193
TCONS_00019717	-0.943931	-0.824139	-1.41079	BGIOSGA004297	XP_006654725.2 60S ribosomal protein L28-1-like
TCONS_00011744	-0.94367	-0.259817	-0.624115	BGIOSGA011262	XP_006649584.1 dual-specificity RNA methyltransferase RlmN
TCONS_00012243	-0.943554	-0.748707	-3.60904	BGIOSGA025361	XP_006650061.1 phylloplanin
TCONS_00004328	-0.942196	0.0771322	-0.426127	BGIOSGA001265	XP_006644655.1 cytochrome P450 72A15-like
TCONS_00029084	-0.941866	-1.61759	-1.05674	BGIOSGA023300	XP_006651080.1 probable mannan synthase 4
TCONS_00008241	-0.941806	-1.52935	-0.00882715	BGIOSGA030748	XP_006647414.1PREDICTED: uncharacterized protein LOC102704395 isoform X1
TCONS_00022841	-0.941578	-3.11773	-0.664839	BGIOSGA007965	XP_004966138.1probable xyloglucan endotransglucosylase/hydrolase protein 25
TCONS_00032554	-0.941463	-1.602	-1.67883	BGIOSGA015066	XP_006662507.2 kinesin-II 85 kDa subunit-like
TCONS_00002738	-0.941385	-1.98931	-1.58387	BGIOSGA003024	XP_006664941.1 Bowman-Birk type bran trypsin inhibitor-like
TCONS_00032564	-0.941372	1.10926	0.0482775	BGIOSGA026540	XP_002464376.1trihelix transcription factor GTL1 isoform X1
TCONS_00005530	-0.940843	-1.90254	-0.777871	BGIOSGA020724	XP_006648357.1 haloacid dehalogenase-like hydrolase domain-containing protein 3
TCONS_00020818	-0.940765	-1.40514	-0.585419	BGIOSGA006757	XP_015694209.1 cyclic nucleotide-gated ion channel 1-like isoform X2
TCONS_00002266	-0.940744	0.3678	0.171131	BGIOSGA023450	XP_006645095.1 patellin-3-like
TCONS_00032493	-0.94033	0.565509	-0.00420296	BGIOSGA005967	XP_021302280.1ALG-2 interacting protein X
TCONS_00024756	-0.939979	-2.50506	-1.7276	BGIOSGA024619	XP_010234976.1exocyst complex component EXO70E2

transcriptome

TCONS_00021218	-0.939558	-0.657441	0.350517	BGIOSGA007063	XP_015693726.1 OTU domain-containing protein DDB_G0284757-like
TCONS_00027668	-0.939161	-0.998648	-1.02426	BGIOSGA009182	XP_015696108.1 probable E3 ubiquitin-protein ligase XBOS35 isoform X3
TCONS_00034867	-0.939095	-1.38433	-0.778042	BGIOSGA034453	XP_006663448.1PREDICTED: uncharacterized protein LOC102714137
TCONS_00022661	-0.938619	-1.63526	-0.660023	BGIOSGA029907	XP_008659852.2protein POLAR LOCALIZATION DURING ASYMMETRIC DIVISION AND REDISTRIBUTION
TCONS_00001127	-0.938608	#NA	#NA	BGIOSGA006914	XP_006644277.1 zinc finger Ran-binding domain-containing protein 2-like
TCONS_00024913	-0.938349	-0.512021	-0.448473	BGIOSGA024474	XP_015694465.1 zinc transporter 5 isoform X2
TCONS_00013517	-0.938227	0.676742	-0.113757	BGIOSGA024490	XP_008669094.1late embryogenesis abundant protein group 2 isoform X1
TCONS_00030205	-0.937697	-1.16516	-0.808761	BGIOSGA032427	XP_021309449.1putative wall-associated receptor kinase-like 16
TCONS_00014682	-0.937544	-2.44426	-2.20911	BGIOSGA025077	XP_006653626.1 uncharacterized membrane protein At1g16860-like
TCONS_00011264	-0.937362	0.264919	-0.681915	BGIOSGA009241	XP_006650841.1 GATA transcription factor 15
TCONS_00014578	-0.936814	1.18483	-0.103381	BGIOSGA016742	XP_015694539.1 BTB/POZ domain-containing protein At5g41330-like
TCONS_00028163	-0.936589	-0.673433	-1.13636	BGIOSGA001926	XP_003572289.1abscisic acid 8'-hydroxylase 2
TCONS_00005830	-0.936535	-1.12237	-0.973298	BGIOSGA007954	XP_015688551.1 zinc finger Ran-binding domain-containing protein 2-like
TCONS_00014495	-0.936238	-0.456233	-0.0158684	BGIOSGA002733	XP_015692102.1 PTI1-like tyrosine-protein kinase 3
TCONS_00010990	-0.936197	-0.488406	-1.86374	BGIOSGA000697	XP_010229614.1cyclic nucleotide-gated ion channel 2 isoform X1
TCONS_00019852	-0.935785	0.216482	0.486943	BGIOSGA006142	XP_006655618.1 probable 2-oxoglutarate-dependent dioxygenase AOP1
TCONS_00033719	-0.935635	-1.15717	-1.40651	BGIOSGA001614	XP_015698121.1PREDICTED: uncharacterized protein LOC102709967 isoform X2
TCONS_00032261	-0.935516	-0.601797	-0.941148	BGIOSGA017253	XP_015697141.1 probable receptor-like protein kinase At5g56460
TCONS_00013082	-0.934344	0.325273	-0.0479314	BGIOSGA009900	XP_004981950.1calcium load-activated calcium channel
TCONS_00031672	-0.934105	-0.028041	-0.323156	BGIOSGA034622	XP_004983564.1abscisic acid receptor PYR1
TCONS_00028924	-0.933584	-3.10194	-2.10874	BGIOSGA001431	XP_006490151.18-amino-7-oxononanoate synthase
TCONS_00019548	-0.933578	-2.10604	-0.571212	BGIOSGA017051	XP_006654580.1 protein PHR1-LIKE 1-like
TCONS_00006745	-0.933044	0.652264	-0.695059	BGIOSGA010956	XP_015689276.1 RNA-binding protein 24-like isoform X1
TCONS_00019770	-0.932841	-1.03515	0.0646105	BGIOSGA017586	XP_020394454.1uncharacterized LOC100274886 isoform X1
TCONS_00029325	-0.932134	-1.27742	-0.380628	BGIOSGA029074	XP_015696734.1 chitin elicitor receptor kinase 1-like
TCONS_00001326	-0.932005	-0.934014	-0.112702	BGIOSGA029256	XP_015693383.1PREDICTED: uncharacterized protein LOC102704385
TCONS_00028406	-0.931422	-0.0602834	-0.998683	BGIOSGA003179	NP_001140822.1protein MTD1
TCONS_00002916	-0.931344	-1.19628	-0.3693	BGIOSGA019251	XP_015699306.1 tryptophan aminotransferase-related protein 1-like
TCONS_00026060	-0.931192	0.123798	-0.551644	BGIOSGA001116	XP_015695518.1 transcription factor GTE7-like
TCONS_00002720	-0.931166	-1.74362	-1.40668	BGIOSGA002507	XP_015692142.1 protein O-linked-mannose beta-1,4-N-acetylglucosaminyltransferase 2-like
TCONS_00019308	-0.930361	-2.06422	-1.31335	BGIOSGA004945	XP_006654414.1 protein root UVB sensitive 6-like
TCONS_00027462	-0.930345	-3.15091	-3.13479	BGIOSGA022577	XP_015695513.1 zinc finger Ran-binding domain-containing protein 2-like
TCONS_00012443	-0.930278	1.74354	-1.38005	BGIOSGA023817	XP_006651497.1PREDICTED: uncharacterized protein LOC102716029
TCONS_00006171	-0.929982	0.980965	0.689602	BGIOSGA018768	XP_003575046.1inositol polyphosphate multikinase IPK2
TCONS_00005101	-0.929704	-0.801791	-1.19976	BGIOSGA004822	XP_004971279.1cytochrome P450 94A2
TCONS_00032903	-0.929181	-3.42677	-1.44231	BGIOSGA034037	XP_015698011.1 alpha-L-arabinofuranosidase 1-like isoform X2
TCONS_00007497	-0.928933	#NA	-2.12036	BGIOSGA008300	XP_015688486.1 RPM1-interacting protein 4-like
TCONS_00020910	-0.928351	0.415007	0.100585	BGIOSGA023115	XP_015693619.1PREDICTED: uncharacterized protein LOC102720612
TCONS_00009672	-0.928295	-1.40819	1.35918	BGIOSGA015371	XP_021307440.1thaumatin-like protein 1b
TCONS_00028587	-0.927986	-1.65794	-1.07943	BGIOSGA030331	XP_006660438.2 sulfate transporter 4.1, chloroplastic-like
TCONS_00030166	-0.927779	-1.52431	-0.386262	BGIOSGA029537	XP_003574625.1uncharacterized protein LOC100834941
TCONS_00015896	-0.92769	-3.10939	-3.22298	BGIOSGA027805	XP_006653405.1 PI-PLC X domain-containing protein At5g67130-like
TCONS_00016498	-0.926598	0.287218	-1.07013	BGIOSGA028771	XP_002448408.2rRNA 2'-O-methyltransferase fibrillarin
TCONS_00037266	-0.926544	-0.7889	-5.08976	BGIOSGA030346	XP_004962897.1polyol transporter 5
TCONS_00010833	-0.926494	-0.223366	-0.640979	BGIOSGA018041	XP_006651750.1 protein POLLEN DEFECTIVE IN GUIDANCE 1
TCONS_00005843	-0.926228	-2.45963	-2.29601	BGIOSGA022418	NP_001152138.1leucoanthocyanidin dioxygenase
TCONS_00031400	-0.926077	-2.28045	-1.11363	BGIOSGA031614	XP_006654334.1 AB hydrolase superfamily protein YfhM-like
TCONS_00003078	-0.925875	-0.78974	-0.712895	BGIOSGA006796	XP_015688137.1 potassium channel KAT2 isoform X1
TCONS_00025800	-0.925874	-0.467153	-0.328435	BGIOSGA027864	XP_006659750.1 far upstream element-binding protein 2-like
TCONS_00014562	-0.925543	0.473619	-0.406987	BGIOSGA035916	XP_006652485.1 adenine phosphoribosyltransferase 2-like
TCONS_00030821	-0.925472	-0.861526	-1.49772	BGIOSGA022624	XP_006649028.1 dynamin-2A-like
TCONS_00004377	-0.925069	-0.0770549	-0.33637	BGIOSGA000843	XP_006646315.1 golgin-84
TCONS_00035485	-0.924744	1.19201	-0.422758	BGIOSGA036899	XP_006663164.2 bifunctional epoxide hydrolase 2-like

TCONS_00028934	-0.924366	0.202542	0.00728588	BGIOSGA025290	XP_006660596.1 chromatin remodeling protein EBS
TCONS_00031192	-0.923031	-1.49447	-0.186096	BGIOSGA033012	XP_006662379.1 inactive protein RESTRICTED TEV MOVEMENT 2-like
TCONS_00024901	-0.922983	-2.31878	0.167036	#N/A	#N/A
TCONS_00036587	-0.922658	-1.77599	-0.924425	BGIOSGA034587	XP_006662812.1 pentatricopeptide repeat-containing protein At4g18975, chloroplastic isoform X2
TCONS_00032516	-0.922399	-2.78632	-0.799376	BGIOSGA031606	XP_004982691.1glutamyl-tRNA reductase 2
TCONS_00017452	-0.921928	-3.84356	-1.06567	BGIOSGA000084	XP_004955595.1uncharacterized protein LOC101778584
TCONS_00003163	-0.921913	-1.89971	-0.154212	BGIOSGA010719	XP_015688294.1 E3 ubiquitin-protein ligase SINAT3-like
TCONS_00021003	-0.921035	-1.82328	-2.56533	BGIOSGA014738	XP_002437256.1zinc finger protein AEBP2
TCONS_00035377	-0.919699	0.726534	-0.217013	BGIOSGA036370	XP_015698062.1 putative disease resistance RPP13-like protein 2 isoform X2
TCONS_00004207	-0.919697	0.00915952	-1.04027	BGIOSGA029386	XP_015697623.1 probable serine/threonine-protein kinase At1g18390 isoform X1
TCONS_00009871	-0.919466	-0.30612	-1.0882	BGIOSGA003683	XP_006649934.2 pentatricopeptide repeat-containing protein At1g62350
TCONS_00028741	-0.919461	#NA	0.274033	#N/A	#N/A
TCONS_00024905	-0.918966	-0.467242	-1.24263	BGIOSGA010141	XP_015691433.1 E3 ubiquitin protein ligase DRIP1-like
TCONS_00000336	-0.918105	-3.2634	-1.05164	BGIOSGA027939	XP_015696513.1 plant cysteine oxidase 2-like
TCONS_00012518	-0.917881	-0.606694	-0.437128	BGIOSGA010465	XP_006650236.1 allene oxide cyclase 3, chloroplastic-like
TCONS_00000242	-0.917838	-0.827625	-1.89483	BGIOSGA011469	XP_015698452.1 receptor-like protein kinase HERK 1
TCONS_00016940	-0.917787	-1.40132	-1.46683	BGIOSGA019011	XP_015692409.1 ras-related protein RABC1-like
TCONS_00030268	-0.917754	-1.46835	-1.1464	BGIOSGA012010	XP_006660270.1 uncharacterized abhydrolase domain-containing protein DDB_G0269086-like
TCONS_00018660	-0.917521	0.207942	0.0875917	BGIOSGA018775	XP_004977085.1dihydrolipoyl dehydrogenase 1, chloroplastic
TCONS_00011146	-0.917443	-1.65163	-0.0484659	#N/A	#N/A
TCONS_00001799	-0.917314	-1.31051	-0.102311	BGIOSGA021835	XP_006644688.1 auxin-responsive protein IAA6-like isoform X3
TCONS_00029315	-0.917041	-0.0832413	0.340634	BGIOSGA018101	XP_004957290.1uncharacterized protein LOC101771681
TCONS_00020516	-0.916245	-0.438562	0.060202	BGIOSGA022686	XP_015694091.1 histone-lysine N-methyltransferase EZ1
TCONS_00005012	-0.915632	-0.511631	-1.95825	BGIOSGA010727	XP_004971151.1transcription factor ICE1
TCONS_00005622	-0.915169	-0.528838	0.608295	BGIOSGA020904	XP_015689613.1PREDICTED: uncharacterized protein LOC102719462
TCONS_00006917	-0.914755	-0.503598	-0.592963	BGIOSGA014996	XP_006647957.1 galactoside 2-alpha-L-fucosyltransferase-like
TCONS_00001035	-0.914583	-0.2248	-3.90781	BGIOSGA032366	XP_021314501.1MDIS1-interacting receptor like kinase 2
TCONS_00020770	-0.914072	-5.31291	-1.90909	BGIOSGA005771	XP_006664961.1 transcription factor bHLH83-like
TCONS_00010570	-0.914009	0.441899	-0.324282	BGIOSGA029795	XP_003578091.1plant UBX domain-containing protein 2
TCONS_00036105	-0.913696	-1.31523	-0.193351	BGIOSGA013425	XP_006650479.1 putative disease resistance RPP13-like protein 3
TCONS_00018595	-0.912861	-0.158265	-1.25493	BGIOSGA018840	NP_001151868.1uncharacterized protein LOC100285504
TCONS_00006358	-0.912861	-2.48019	-3.06228	BGIOSGA037215	NP_001145028.1uncharacterized protein LOC100278203
TCONS_00011142	-0.912544	-1.72987	-2.32792	BGIOSGA025295	XP_003559293.1SEC12-like protein 1
TCONS_00009284	-0.912325	-0.447573	-0.743763	BGIOSGA004822	XP_006649380.1 alkane hydroxylase MAH1-like
TCONS_00004960	-0.912212	-0.858019	-1.38683	BGIOSGA016888	XP_006645221.1 transcription factor bHLH77 isoform X2
TCONS_00024667	-0.912124	-0.326157	-0.141309	BGIOSGA007474	XP_006657501.1 phosphopantetheine adenylyltransferase 1
TCONS_00000134	-0.911923	0.743141	1.54311	BGIOSGA036561	XP_006643693.1 ethylene-responsive transcription factor ERF036-like
TCONS_00032792	-0.911652	-1.71813	-0.683589	BGIOSGA007417	XP_006662113.1 cyclin-dependent kinase E-1
TCONS_00017148	-0.911323	-0.792811	-0.915913	BGIOSGA002664	XP_015692482.1 beta-glucuronosyltransferase GlcAT14A-like
TCONS_00031296	-0.911041	0.834074	-1.33857	BGIOSGA032396	XP_006661849.1 protein NRT1/ PTR FAMILY 5.2-like
TCONS_00018808	-0.910958	-2.89743	#NA	BGIOSGA018613	XP_004969848.1E3 ubiquitin-protein ligase MARCH2
TCONS_00024557	-0.910886	1.2136	0.0333615	BGIOSGA024834	XP_015694525.1PREDICTED: uncharacterized protein LOC102705264, partial
TCONS_00012375	-0.910795	-0.16199	-1.01717	BGIOSGA010609	XP_004984160.1metacaspase-1 isoform X2
TCONS_00021044	-0.910637	0.892218	-0.332648	BGIOSGA023275	XP_015693766.1 putative disease resistance protein RGA3
TCONS_00005537	-0.910101	-1.93391	-1.011	BGIOSGA006138	XP_004951742.1uncharacterized protein LOC101780165
TCONS_00036530	-0.909934	1.82017	0.178219	BGIOSGA030709	XP_006663145.1 transcription activator GLK2-like
TCONS_00024097	-0.909207	0.68097	-1.83367	BGIOSGA034685	XP_015694723.1 inter alpha-trypsin inhibitor, heavy chain 4
TCONS_00002897	-0.909194	-1.65608	-0.751244	BGIOSGA030129	XP_009776295.1 HORMA domain-containing protein 1-like isoform X2
TCONS_00004856	-0.907707	-1.26235	-0.386242	BGIOSGA006660	XP_003564797.1CLP protease regulatory subunit CLPX1, mitochondrial
TCONS_00006802	-0.907458	-1.30556	-1.55293	BGIOSGA029665	XP_006647837.1 homeobox-leucine zipper protein HOX16 isoform X1
TCONS_00001583	-0.907184	-0.592566	-1.80581	BGIOSGA036429	XP_004969565.1uncharacterized protein LOC101755436
TCONS_00027533	-0.906969	-3.3079	-1.89054	BGIOSGA002597	XP_006659208.1 NAC domain-containing protein 21/22-like
TCONS_00014333	-0.906828	0.644612	-0.834307	BGIOSGA012197	XP_006652337.2 non-functional NADPH-dependent codeinone reductase 2-like

TCONS_00007770	-0.906547	-2.19718	-0.588614	BGIOSGA018450	XP_006647151.1 D-amino-acid transaminase, chloroplastic-like
TCONS_00019532	-0.906339	-2.78814	-2.04713	BGIOSGA028511	XP_021303324.1myb-related protein 2
TCONS_00009126	-0.906239	-1.03856	-0.503196	BGIOSGA011652	XP_006649225.1 phosphatidylserine decarboxylase proenzyme 1, mitochondrial
TCONS_00018596	-0.906117	0.351402	-0.690559	BGIOSGA013417	XP_015692477.1 putative E3 ubiquitin-protein ligase RF298
TCONS_00019264	-0.906017	0.0391423	-0.435484	BGIOSGA003645	XP_015693233.1 probable manganese-transporting ATPase PDR2
TCONS_00035214	-0.905698	0.000916764	0.332812	BGIOSGA025868	XP_006663053.1 transmembrane protein 45A-like
TCONS_00026670	-0.905322	-0.123546	-1.54854	BGIOSGA021382	XP_003574544.1uncharacterized protein LOC100835247
TCONS_00015235	-0.905304	-1.72605	-0.57622	BGIOSGA017425	XP_006653090.2PREDICTED: uncharacterized protein LOC102713267
TCONS_00007478	-0.90528	-1.30943	0.105843	BGIOSGA003430	XP_006646987.1 pyrophosphate-energized vacuolar membrane proton pump
TCONS_00005353	-0.90492	-1.29785	-1.78877	BGIOSGA007464	XP_006646833.1 extracellular matrix-binding protein ebh
TCONS_00037198	-0.904895	-0.0923552	1.12516	BGIOSGA040665	XP_006664027.1PREDICTED: uncharacterized protein LOC102720933
TCONS_00003176	-0.904666	-0.906126	-0.457669	BGIOSGA015451	XP_004967353.1uncharacterized protein LOC101783380
TCONS_00011193	-0.904425	0.509589	0.501365	BGIOSGA013795	XP_015690523.1 RNA-binding protein 8A-like isoform X2
TCONS_00031984	-0.903984	-2.35612	-0.793314	BGIOSGA018063	XP_006661686.1 WAT1-related protein At4g08290-like
TCONS_00017927	-0.903787	-0.772401	-1.91527	BGIOSGA019971	XP_015692905.1 transcription factor ILR3-like
TCONS_00011127	-0.903519	0.531301	0.0536617	BGIOSGA025265	XP_006650730.1 guanylate-binding protein 4-like
TCONS_00024421	-0.9031	0.717364	0.613526	BGIOSGA017386	XP_003561539.1L-type lectin-domain containing receptor kinase I.9
TCONS_00015192	-0.902806	-3.02436	-1.66986	BGIOSGA017374	XP_006653043.1 putative threonine aspartase
TCONS_00026084	-0.902752	-1.97005	-1.34947	#N/A	#N/A
TCONS_00038000	-0.902638	-1.76846	-2.10771	BGIOSGA007923	XP_006653789.1 putative disease resistance protein RGA4
TCONS_00031302	-0.902096	-1.55476	-0.893022	BGIOSGA019760	XP_015697192.1 desumoylating isopeptidase 1-like isoform X2
TCONS_00019409	-0.901415	-1.27183	-1.09111	BGIOSGA034875	XP_006654489.1 transcription factor DIVARICATA-like
TCONS_00013921	-0.901249	-0.787781	-0.229863	#N/A	#N/A
TCONS_00024418	-0.901002	0.183908	-0.0157013	BGIOSGA016279	XP_006658183.1 2-oxoglutarate dehydrogenase, mitochondrial-like
TCONS_00011400	-0.900914	-1.28481	0.21421	BGIOSGA011624	XP_015690822.1PREDICTED: uncharacterized protein LOC107303929
TCONS_00005193	-0.9001	-1.44716	-0.634248	BGIOSGA005774	XP_015699260.1 soluble inorganic pyrophosphatase 4-like
TCONS_00025712	-0.898907	0.295985	0.208561	BGIOSGA015085	NP_001169741.1uncharacterized LOC100383622
TCONS_00032930	-0.898662	0.00642726	0.245748	BGIOSGA007036	XP_002448955.1probable calcium-transporting ATPase 9, plasma membrane-type
TCONS_00012263	-0.898601	-3.90122	0.617796	BGIOSGA006524	XP_015689893.1 beta-caryophyllene synthase-like
TCONS_00019914	-0.898268	-0.9599	-1.12556	BGIOSGA003070	XP_006654877.1 casein kinase I isoform delta-like isoform X1
TCONS_00009784	-0.897789	0.200106	-0.997483	BGIOSGA002794	XP_006649852.1PREDICTED: uncharacterized protein LOC102715376
TCONS_00026986	-0.897783	-3.81304	-1.27272	BGIOSGA029036	XP_006659594.1 UBX domain-containing protein 1-like
TCONS_00026836	-0.897363	-0.265135	-0.209634	BGIOSGA030854	XP_015696049.1 dof zinc finger protein MNB1A-like
TCONS_00008834	-0.897315	-4.0752	-2.57263	BGIOSGA025750	XP_003570606.1protein EXORDIUM
TCONS_00023770	-0.897051	-0.57135	-0.481797	BGIOSGA022733	XP_015695260.1 polyadenylate-binding protein RBP45-like
TCONS_00008744	-0.896919	-3.9535	-2.81578	BGIOSGA029490	XP_010240461.1uncharacterized protein LOC100838048 isoform X1
TCONS_00021691	-0.896408	-1.07331	-0.604337	BGIOSGA011902	XP_006655833.1 probable receptor-like protein kinase At5g15080 isoform X1
TCONS_00004499	-0.89621	0.0305038	-0.747142	BGIOSGA000721	XP_002458585.2WD repeat-containing protein 13
TCONS_00013107	-0.896109	-0.842076	-1.24119	BGIOSGA010316	XP_006651771.1PREDICTED: uncharacterized protein LOC102722948
TCONS_00002699	-0.896083	-1.18478	-0.473962	BGIOSGA023803	XP_002455809.1rust resistance kinase Lr10
TCONS_00019955	-0.895366	-2.05055	-1.24629	BGIOSGA016901	XP_004964284.1scarecrow-like protein 6
TCONS_00009065	-0.895042	-1.18777	-1.01775	BGIOSGA013400	XP_006648146.1 phosphatidylinositol 4-phosphate 5-kinase 9
TCONS_00029595	-0.894872	-0.389791	-0.831195	BGIOSGA014766	XP_003576878.1polyadenylate-binding protein 2
TCONS_00033106	-0.894563	-0.91219	0.307405	BGIOSGA009693	XP_006662789.1 aldehyde dehydrogenase family 3 member H1-like
TCONS_00003868	-0.893738	-0.904143	-1.88094	BGIOSGA022838	NP_001146074.1submergence induced protein SI397
TCONS_00029360	-0.893649	0.178083	0.985522	BGIOSGA031108	XP_006660856.1 DEAD-box ATP-dependent RNA helicase 7
TCONS_00007683	-0.892937	-0.951325	0.34999	BGIOSGA002978	XP_006647114.1 phosphoenolpyruvate carboxylase 1
TCONS_00033691	-0.892718	-1.52799	-1.01601	BGIOSGA035466	XP_021317288.1uncharacterized protein LOC8062623 isoform X2
TCONS_00016511	-0.892134	0.484025	-0.184171	BGIOSGA024352	XP_015694619.1 heterogeneous nuclear ribonucleoprotein R-like
TCONS_00029332	-0.891479	0.685606	0.113792	BGIOSGA013956	XP_003578418.1DNA repair RAD52-like protein 2, chloroplastic
TCONS_00011331	-0.891111	-1.30215	-0.926055	BGIOSGA013941	XP_006650893.1 RPM1-interacting protein 4-like
TCONS_00019524	-0.89066	-1.40741	-1.78497	BGIOSGA005545	XP_024315081.1probable leucine-rich repeat receptor-like protein kinase At5g49770

TCONS_00014510	-0.890649	-0.789704	-1.92099	BGIOSGA035813	XP_015689994.1PREDICTED: uncharacterized protein LOC102703201
TCONS_00002252	-0.889885	-1.23806	-1.24319	BGIOSGA004889	XP_006646517.2 bifunctional aspartate aminotransferase and glutamate/aspartate-prephenate aminotransferase
TCONS_00015923	-0.889702	-0.204368	0.146397	BGIOSGA015060	XP_008669391.1acyl-coenzyme A thioesterase 13
TCONS_00018285	-0.889674	0.159905	-0.0882428	BGIOSGA020339	XP_006654760.1 60S ribosomal protein L35a-2
TCONS_00019011	-0.889464	-0.65782	-0.798324	BGIOSGA015693	XP_006654243.2PREDICTED: uncharacterized protein LOC102706192, partial
TCONS_00012970	-0.889095	-0.545712	0.022391	BGIOSGA034003	XP_022679265.1putative disease resistance RPP13-like protein 1
TCONS_00008282	-0.88904	-4.05779	-3.10039	BGIOSGA000124	XP_004956139.1lecithin-cholesterol acyltransferase-like 1
TCONS_00030467	-0.888647	-3.07388	0.325189	BGIOSGA016911	XP_004957527.1rop guanine nucleotide exchange factor 1
TCONS_00034043	-0.888228	#NA	-4.26081	BGIOSGA016659	NP_001131192.1retrotransposon protein precursor
TCONS_00023356	-0.887848	-0.34488	-1.43158	BGIOSGA017579	XP_006649647.1 tubulin alpha-2 chain
TCONS_00003843	-0.887625	0.888861	0.251681	BGIOSGA037949	NP_001168737.1uncharacterized LOC100382529
TCONS_00005944	-0.887022	-0.336252	-0.389279	BGIOSGA008078	XP_006647214.1 motile sperm domain-containing protein 2 isoform X1
TCONS_00002093	-0.88694	-3.63278	#NA	BGIOSGA004721	XP_019706214.1 heavy metal-associated isoprenylated plant protein 33-like isoform X1
TCONS_00017843	-0.886872	-1.55684	-2.06065	BGIOSGA037215	XP_004962033.1amino acid permease 3
TCONS_00018306	-0.886236	-2.03646	-1.55351	BGIOSGA001046	XP_015693178.1 auxin response factor 15 isoform X2
TCONS_00000334	-0.885978	-0.686778	-0.172052	BGIOSGA023194	XP_006643851.1 3'-N-debenzoyl-2'-deoxytaxol N-benzoyltransferase-like
TCONS_00014944	-0.885727	-0.51303	-1.6573	BGIOSGA017127	XP_006644010.2 dynein light chain 1, cytoplasmic
TCONS_00006954	-0.885712	-0.944115	-1.59493	BGIOSGA033172	XP_006648000.1 myosin-15-like
TCONS_00009920	-0.885466	0.52641	0.0709739	BGIOSGA002350	XP_006649971.2 DEAD-box ATP-dependent RNA helicase 24
TCONS_00012055	-0.885295	-0.0173466	-1.76797	BGIOSGA010953	XP_015690400.1 cytoplasmic phosphatidylinositol transfer protein 1-like isoform X2
TCONS_00033155	-0.885285	-0.930332	0.073909	BGIOSGA006261	XP_015697591.1 mitogen-activated protein kinase kinase kinase YODA-like
TCONS_00013622	-0.885224	-0.212996	0.250085	BGIOSGA033864	XP_003580626.1probable carbohydrate esterase At4g34215
TCONS_00034249	-0.885079	#NA	#NA	BGIOSGA034543	XP_019103799.1PREDICTED: uncharacterized protein LOC104889492 isoform X2
TCONS_00036515	-0.884682	0.17282	-2.33169	BGIOSGA028948	XP_006664796.1PREDICTED: uncharacterized protein LOC102710366
TCONS_00033133	-0.884654	-0.768489	-0.962779	BGIOSGA014910	XP_006662801.1 amino acid permease 3-like
TCONS_00016575	-0.884637	-0.943885	-0.290839	BGIOSGA014394	XP_006653742.1 putative DNA ligase 4
TCONS_00020451	-0.884326	1.19749	-1.01263	BGIOSGA017591	XP_015693822.1 MACPF domain-containing protein At1g14780-like isoform X1
TCONS_00026656	-0.884257	-2.64587	-3.52738	BGIOSGA007420	XP_015695614.1 probable mannan synthase 11
TCONS_00001169	-0.884191	0.980829	0.472459	BGIOSGA039766	XP_002455819.1carbamoyl-phosphate synthase large chain, chloroplastic
TCONS_00033724	-0.884158	0.306147	0.0426051	BGIOSGA035498	XP_006663547.1 putative H/ACA ribonucleoprotein complex subunit 1-like protein 1
TCONS_00035897	-0.883822	0.308796	-0.309798	BGIOSGA021176	XP_003576639.115-cis-zeta-carotene isomerase, chloroplastic
TCONS_00011337	-0.883645	-1.03757	-1.94198	BGIOSGA005210	XP_012698059.14-hydroxyphenylacetaldehyde oxime monooxygenase
TCONS_00002018	-0.883402	-0.828716	-0.382915	BGIOSGA036680	XP_015688330.1 dihydropyrimidinase
TCONS_00023196	-0.883265	0.682818	0.168304	BGIOSGA025238	XP_002462003.1uncharacterized protein LOC8061708
TCONS_00033198	-0.883265	0.26778	-1.8317	BGIOSGA034254	XP_021316408.1uncharacterized protein LOC8071576 isoform X1
TCONS_00033226	-0.883259	-1.10931	-1.72838	BGIOSGA031703	XP_006662847.1 non-lysosomal glucosylceramidase
TCONS_00026271	-0.882784	0.356071	-2.00882	BGIOSGA028354	XP_015696025.1 photosynthetic NDH subunit of subcomplex B 1, chloroplastic
TCONS_00035100	-0.882165	-0.470338	-3.7962	BGIOSGA000177	XP_006663556.1PREDICTED: uncharacterized protein LOC102705580, partial
TCONS_00013385	-0.882084	-0.797518	-0.15785	BGIOSGA016673	XP_004981338.1remorin 4.1
TCONS_00016748	-0.881904	0.513105	-0.878454	BGIOSGA029174	XP_006652923.1 oryzain alpha chain
TCONS_00006595	-0.881509	-1.22555	-0.34873	BGIOSGA008762	XP_020408903.1rho guanine nucleotide exchange factor isoform X1
TCONS_00017859	-0.881173	0.241333	0.526917	BGIOSGA000410	XP_004961980.1amidophosphoribosyltransferase, chloroplastic
TCONS_00008588	-0.880693	0.196336	-0.945574	BGIOSGA014504	XP_006648938.1 scarecrow-like protein 8
TCONS_00029443	-0.880555	-4.92719	-2.52104	BGIOSGA029334	XP_006660929.1 auxin-responsive protein SAUR36-like
TCONS_00029599	-0.880505	0.854105	1.17927	BGIOSGA030221	XP_006661031.1 PI-PLC X domain-containing protein At5g67130
TCONS_00036571	-0.880155	-2.808	-1.67464	BGIOSGA018902	XP_006663720.1PREDICTED: uncharacterized protein LOC102720754
TCONS_00012988	-0.879725	-0.422808	-1.23779	BGIOSGA001992	XP_006650431.1 germin-like protein 3-6
TCONS_00036838	-0.879215	-0.884494	-0.31494	#N/A	#N/A
TCONS_00032389	-0.878518	0.0962799	-0.16894	BGIOSGA011828	XP_006661841.1 60S ribosomal protein L21-2
TCONS_00002136	-0.878066	-1.49568	-0.207276	#N/A	#N/A
TCONS_00026724	-0.87757	-0.255877	-1.30182	BGIOSGA008273	XP_022683600.1exocyst complex component EXO70B1
TCONS_00009963	-0.877186	0.586091	-0.684288	BGIOSGA034560	XP_006649997.1 calmodulin
TCONS_00011426	-0.876913	0.843336	0.399361	BGIOSGA008836	XP_015690439.1 40S ribosomal protein S17-like
TCONS_00005610	-0.876747	-1.28199	0.335964	BGIOSGA017313	XP_006648412.2 zinc finger CCCH domain-containing protein 14
TCONS_00036082	-0.87644	-0.328387	#NA	#N/A	#N/A

TCONS_00029625	-0.876229	-2.12933	0.305849	BGIOSGA002716	XP_006664800.1PREDICTED: uncharacterized protein LOC102717582
TCONS_00036290	-0.87612	#NA	-2.53208	BGIOSGA037649	XP_012700358.1uncharacterized protein LOC101786827 isoform X3
TCONS_00004627	-0.875987	-0.140691	0.267711	BGIOSGA014196	XP_006644909.2 probable LRR receptor-like serine/threonine-protein kinase At1g06840 isoform X1
TCONS_00017846	-0.875547	-0.653722	-1.55772	BGIOSGA017544	XP_006654427.1 bidirectional sugar transporter SWEET1b isoform X1
TCONS_00022867	-0.875471	-2.12625	-1.36376	BGIOSGA007499	XP_015694405.1 squamosa promoter-binding-like protein 12
TCONS_00007182	-0.874775	-0.612552	-0.287481	BGIOSGA015602	XP_006648197.1 vacuolar amino acid transporter 1-like isoform X2
TCONS_00004417	-0.874088	0.331445	0.0478606	BGIOSGA000805	XP_006644726.1PREDICTED: uncharacterized protein At4g18490 isoform X3
TCONS_00011440	-0.873942	-1.47609	-0.630571	BGIOSGA035153	XP_002465995.2trihelix transcription factor GTL1
TCONS_00005640	-0.873564	-0.161811	-0.300857	BGIOSGA029181	XP_006647052.1 peptidyl-prolyl cis-trans isomerase CYP95 isoform X1
TCONS_00004160	-0.873513	-0.293596	0.050676	BGIOSGA009466	XP_006646193.1PREDICTED: uncharacterized protein LOC102717671 isoform X2
TCONS_00032733	-0.873274	0.251633	#NA	#N/A	#N/A
TCONS_00008097	-0.873239	-2.21744	-1.5127	BGIOSGA034211	XP_006647323.2 heparan-alpha-glucosaminide N-acetyltransferase-like isoform X1
TCONS_00004185	-0.872945	-2.2166	-2.30075	BGIOSGA020330	XP_006646207.1 glutamate synthase 1 [NADH], chloroplastic isoform X1
TCONS_00029486	-0.872717	-0.445206	-0.218547	BGIOSGA016952	XP_015692233.1 DNA-directed RNA polymerase IV subunit 1-like
TCONS_00026140	-0.872313	-0.15841	-0.208249	BGIOSGA035883	XP_006659944.1 26S proteasome non-ATPase regulatory subunit 1 homolog A-like
TCONS_00011000	-0.872289	-0.472012	-1.07264	BGIOSGA004030	XP_006650627.1 isoflavone 2'-hydroxylase-like
TCONS_00008926	-0.87221	-0.351994	-0.337703	BGIOSGA020076	XP_015690933.1 probable NOT transcription complex subunit VIP2 isoform X2
TCONS_00021352	-0.872007	-2.70214	-1.19722	BGIOSGA023580	XP_010227454.1type 2 DNA topoisomerase 6 subunit B-like isoform X1
TCONS_00028440	-0.871923	-0.358245	-0.386828	BGIOSGA033906	XP_006659667.1 acyl carrier protein 3, chloroplastic-like
TCONS_00009470	-0.871883	-1.12583	-1.24391	BGIOSGA027293	XP_006649529.1 homeobox-leucine zipper protein HOX13-like
TCONS_00036272	-0.871755	#NA	0.724703	BGIOSGA035740	XP_004962696.1metallothionein-like protein 4B
TCONS_00014202	-0.870803	-1.49739	-2.01665	BGIOSGA027798	XP_004975561.1uncharacterized protein LOC101763320
TCONS_00016993	-0.870645	-0.924101	-0.33104	BGIOSGA000344	XP_015692770.1 pectin acetylesterase 5-like
TCONS_00021007	-0.870584	-2.7886	-2.16992	BGIOSGA009697	XP_006656225.1 phosphatidylcholine:diacylglycerol cholinephosphotransferase 1-like
TCONS_00014661	-0.870204	-0.934687	-1.02056	BGIOSGA016826	XP_006652571.1 O-glucosyltransferase rumi-like isoform X2
TCONS_00026486	-0.869806	-2.46948	-1.33311	BGIOSGA009539	XP_006659347.2 nucleobase-ascorbate transporter 2-like
TCONS_00023636	-0.869729	-1.19655	-1.48531	BGIOSGA025660	XP_015690188.1PREDICTED: uncharacterized protein LOC102704689 isoform X2
TCONS_00022452	-0.8697	-1.32968	-0.530609	BGIOSGA038041	XP_006657069.1 ent-kaurene oxidase 2
TCONS_00019942	-0.869607	-0.352764	0.151358	BGIOSGA007263	XP_006655675.2PREDICTED: uncharacterized protein LOC102718557 isoform X1
TCONS_00008996	-0.869373	-1.23658	-1.05241	BGIOSGA014738	XP_004964732.1adenylyltransferase and sulfurtransferase MOCS3-1
TCONS_00011496	-0.86926	-0.62056	-0.451226	BGIOSGA006243	XP_015691083.1 MLO protein homolog 1
TCONS_00031328	-0.868979	-0.466227	-0.864525	BGIOSGA003704	XP_008791558.1 protein ENHANCED DISEASE RESISTANCE 2-like isoform X3
TCONS_00030192	-0.868854	#NA	1.13267	#N/A	#N/A
TCONS_00001210	-0.868562	-1.35556	-1.33618	BGIOSGA007810	XP_022682778.1U-box domain-containing protein 35
TCONS_00025315	-0.868551	-1.35344	-0.851495	BGIOSGA024113	XP_015694460.1 cell number regulator 2
TCONS_00027253	-0.868401	0.796028	0.102894	BGIOSGA025984	XP_015688778.1 40S ribosomal protein S13-2
TCONS_00027145	-0.868352	0.472828	-0.245329	BGIOSGA034819	XP_006659696.1 40S ribosomal protein S25-4
TCONS_00021871	-0.868268	-3.57685	-1.95151	BGIOSGA009080	XP_003564073.1protein EXORDIUM
TCONS_00017119	-0.868207	-1.45102	-0.319258	BGIOSGA003571	XP_006654032.1 LEC14B protein-like
TCONS_00005062	-0.86811	1.09064	-0.345573	BGIOSGA000153	XP_015699149.1 putative disease resistance protein RGA3
TCONS_00012269	-0.867918	-1.69804	-1.19363	BGIOSGA004986	XP_006651374.1 nuclear pore complex protein NUP93A-like
TCONS_00013829	-0.867482	2.56482	2.71426	#N/A	#N/A
TCONS_00030250	-0.86691	0.412901	0.0389202	BGIOSGA007303	XP_006660762.1 60S ribosomal protein L9
TCONS_00039373	-0.866146	#NA	#NA	BGIOSGA028277	XP_014756540.1endo-1,3;1,4-beta-D-glucanase isoform X2
TCONS_00030667	-0.86573	-0.410117	-2.44323	BGIOSGA001826	XP_015697273.1 putative disease resistance protein RGA4
TCONS_00000814	-0.865638	-2.2609	-1.52755	BGIOSGA019130	XP_015688277.1 peroxidase 1-like
TCONS_00017310	-0.865073	-0.601098	-1.90746	BGIOSGA037146	XP_003568756.1B-box zinc finger protein 22
TCONS_00015290	-0.865014	0.23395	-0.141005	BGIOSGA015692	NP_001344787.1uncharacterized LOC111365147
TCONS_00015769	-0.864541	-1.59462	0.608742	BGIOSGA000232	XP_006652207.1 SNF1-related protein kinase regulatory subunit gamma-1
TCONS_00022759	-0.864515	0.0307665	-1.46334	BGIOSGA020695	XP_015694194.1PREDICTED: uncharacterized protein LOC102708629 isoform X2
TCONS_00013727	-0.864373	-1.46943	-1.03546	BGIOSGA005151	XP_022684357.1disease resistance protein RGA2 isoform X1
TCONS_00024567	-0.863879	-2.16733	-0.5998	BGIOSGA009777	XP_006657442.1 probable receptor-like protein kinase At1g30570 isoform X2
TCONS_00017951	-0.863861	-0.414066	-0.399395	BGIOSGA028952	XP_006655385.1PREDICTED: uncharacterized protein LOC102714099
TCONS_00032957	-0.863318	-0.742763	0.0894341	BGIOSGA033836	XP_015697555.1 putative NAC domain-containing protein 94

transcriptome

TCONS_00003158	-0.863283	-0.538448	-0.910653	BGIOSGA030049	XP_006643959.1 putative ubiquitin-conjugating enzyme E2 38 isoform X3
TCONS_00024332	-0.863198	0.820986	0.304865	BGIOSGA026373	XP_006658111.1 F-box/kelch-repeat protein SKIP6-like
TCONS_00010396	-0.862953	0.0761483	0.0507715	BGIOSGA010420	XP_004982552.1uncharacterized protein LOC101773486
TCONS_00030872	-0.862729	-1.48411	-2.11761	BGIOSGA040597	XP_008785647.1 heavy metal-associated isoprenylated plant protein 3-like
TCONS_00010365	-0.862722	-3.48328	-2.56681	BGIOSGA000168	XP_006651534.1 protein SRG1-like
TCONS_00005355	-0.862356	-0.98682	-0.88319	BGIOSGA032966	XP_006659718.1 mitochondrial import receptor subunit TOM9-2-like
TCONS_00017136	-0.861526	-2.70408	-1.35538	BGIOSGA027832	XP_006654994.1 O-glucosyltransferase rumi homolog
TCONS_00011640	-0.861167	-1.35102	-0.886076	#N/A	#N/A
TCONS_00036520	-0.861112	-1.37179	-1.25439	BGIOSGA009459	XP_004978420.1probable LRR receptor-like serine/threonine-protein kinase At1g67720
TCONS_00015647	-0.860954	-1.97307	-1.74291	BGIOSGA040514	XP_004961357.1jacalin-related lectin 19 isoform X2
TCONS_00012267	-0.860204	-1.31952	-1.87349	BGIOSGA031779	XP_006650078.1 E3 ubiquitin-protein ligase MIEL1 isoform X1
TCONS_00020314	-0.860124	-0.555027	-0.260226	BGIOSGA022509	XP_006655896.2 omega-amidase, chloroplastic-like
TCONS_00034467	-0.860069	-2.5551	-1.79088	BGIOSGA027987	XP_004978928.1uncharacterized protein LOC101780233
TCONS_00030922	-0.859818	-1.64586	-0.38807	BGIOSGA032112	XP_006653400.1 cellulose synthase-like protein H2
TCONS_00029377	-0.859609	-0.960188	-2.59667	BGIOSGA029050	XP_006660875.1 cytochrome P450 78A9-like
TCONS_00007514	-0.859454	-0.311984	0.0031188	BGIOSGA032873	XP_004951609.2 protein FLX-like 2
TCONS_00019260	-0.859203	1.29277	-0.178138	BGIOSGA022835	XP_003568474.1uncharacterized protein LOC100841736 isoform X2
TCONS_00005721	-0.858715	-2.75025	-3.04713	BGIOSGA035197	NP_001152657.1heat shock factor protein 1
TCONS_00011785	-0.858706	-0.457275	0.0802173	BGIOSGA011220	XP_008658017.1retrovirus-related Pol polyprotein LINE-1 isoform X1
TCONS_00002780	-0.85843	0.47288	-0.521066	BGIOSGA016434	XP_015697597.1 probable serine/threonine-protein kinase At1g18390
TCONS_00027657	-0.858359	-0.233026	-1.17843	BGIOSGA027354	XP_006659250.1 chaperone protein ClpB3, mitochondrial-like
TCONS_00018104	-0.858222	-0.223364	-0.013123	BGIOSGA000731	XP_006654622.2 pseudouridylate synthase 7 homolog
TCONS_00016585	-0.858151	-0.123376	1.47618	BGIOSGA014382	XP_006652793.2 probable nucleoredoxin 3
TCONS_00029226	-0.857866	-0.795365	-1.53999	BGIOSGA021419	XP_015696749.1 serine/threonine-protein kinase D6PK-like
TCONS_00034628	-0.857827	-1.35552	0.690738	BGIOSGA034191	XP_006662916.1 protein FAM136A-like
TCONS_00007147	-0.857827	-2.94049	-0.894224	#N/A	#N/A
TCONS_00022788	-0.857827	1.05951	0.690738	#N/A	#N/A
TCONS_00034758	-0.85678	-0.871475	0.103223	BGIOSGA035414	XP_015697688.1 transmembrane protein 87B
TCONS_00017000	-0.85675	0.0256977	-1.79908	BGIOSGA012828	XP_008648635.1cation/H(+) antiporter 20
TCONS_00036527	-0.855641	0.417991	-0.0888682	BGIOSGA036800	XP_006663709.1 mRNA turnover protein 4 homolog
TCONS_00019071	-0.855026	-1.49726	-1.61333	BGIOSGA035186	XP_015692847.1 adenine/guanine permease AZG1-like
TCONS_00031920	-0.854948	-1.49974	-1.60057	BGIOSGA006364	XP_002440185.1probable glucuronosyltransferase Os01g0926600
TCONS_00037776	-0.854585	-0.615763	-0.684055	BGIOSGA017425	XP_006653090.2PREDICTED: uncharacterized protein LOC102713267
TCONS_00026567	-0.85443	-0.932515	-0.472781	BGIOSGA029967	XP_021321317.1protein ACCELERATED CELL DEATH 6
TCONS_00005633	-0.854137	-1.45064	-0.927542	BGIOSGA015549	XP_015688386.1 proteoglycan 4-like
TCONS_00021524	-0.853973	-0.721711	-1.10961	BGIOSGA022026	XP_015694190.1 probable transcriptional regulator SLK2
TCONS_00011708	-0.853482	-1.00236	-1.00363	BGIOSGA012779	XP_006649535.1 calmodulin-binding transcription activator 1-like
TCONS_00031315	-0.853378	0.578013	-0.372217	BGIOSGA014526	XP_006664819.1 probable methyltransferase PMT3
TCONS_00029481	-0.853031	-1.45624	-3.0918	BGIOSGA022352	NP_001142968.2uncharacterized LOC100275421
TCONS_00012344	-0.852939	-0.0160444	-1.13996	BGIOSGA029028	NP_001152250.1cationic amino acid transporter
TCONS_00027457	-0.852938	1.44292	-0.280054	BGIOSGA017449	XP_006644307.1 chromatin modification-related protein EAF1 B-like isoform X1
TCONS_00019918	-0.852783	0.298624	-1.75592	BGIOSGA010344	XP_006655660.1PREDICTED: uncharacterized protein LOC102706200
TCONS_00036326	-0.852432	-0.113978	-0.0968168	BGIOSGA017012	XP_015698346.1 protein misato homolog 1
TCONS_00004840	-0.85239	-0.501279	-1.14375	BGIOSGA000379	XP_006645131.1 NAD(P)H-quinone oxidoreductase subunit N, chloroplastic
TCONS_00005036	-0.852029	-1.36301	#NA	BGIOSGA006598	XP_002459042.1flowering-promoting factor 1-like protein 2
TCONS_00012293	-0.851967	0.508084	-0.396199	BGIOSGA026299	XP_015690831.1 protein HUA2-LIKE 3-like isoform X2
TCONS_00032674	-0.851626	-1.76013	-1.05299	BGIOSGA017996	XP_006662041.1 protein NRT1/ PTR FAMILY 6.3-like
TCONS_00035145	-0.851613	-1.32372	-1.07935	BGIOSGA017141	XP_015698142.1 F-box/kelch-repeat protein SKIP11-like
TCONS_00012481	-0.851157	-1.36408	0.674379	BGIOSGA010501	XP_021308267.1U-box domain-containing protein 57
TCONS_00018091	-0.850984	0.547151	0.290332	BGIOSGA020147	XP_014754606.1protein RNA-directed DNA methylation 3 isoform X2
TCONS_00035320	-0.850488	-1.66578	-2.31363	BGIOSGA005940	XP_015698129.1 magnesium/proton exchanger 1
TCONS_00036858	-0.850384	0.129616	0.43871	BGIOSGA036467	XP_015698758.1 putative disease resistance protein RGA1
TCONS_00020564	-0.850369	0.0442621	-0.529237	BGIOSGA023243	XP_006656035.1 C2 and GRAM domain-containing protein At1g03370
TCONS_00003181	-0.85031	0.219851	-1.91265	BGIOSGA002041	XP_004967342.1protein NUCLEAR FUSION DEFECTIVE 6, chloroplastic/mitochondrial isoform X2

TCONS_00011776	-0.850072	0.224986	-0.574748	BGIOSGA034621	XP_006649612.2PREDICTED: uncharacterized protein LOC102717136 isoform X2
TCONS_00037380	-0.849729	-2.12778	-0.785743	BGIOSGA017818	XP_006664115.1 myb-related protein MYBAS2 isoform X1
TCONS_00002722	-0.849573	-3.2992	-4.22278	BGIOSGA008108	XP_004968121.1uncharacterized protein LOC101780825
TCONS_00030471	-0.849492	#NA	-0.914657	#N/A	#N/A
TCONS_00022194	-0.849476	-0.880495	-1.39619	BGIOSGA021478	XP_006656056.1PREDICTED: uncharacterized protein LOC102711074
TCONS_00021491	-0.84905	0.136924	-0.588928	BGIOSGA027602	XP_015694094.1 60S ribosomal protein L13-1-like
TCONS_00015993	-0.848483	-2.52594	-1.489	BGIOSGA014984	XP_006652353.1 sugar transport protein 5-like
TCONS_00023568	-0.847931	-0.367978	0.666467	#N/A	#N/A
TCONS_00034619	-0.847631	-0.332582	0.151555	BGIOSGA032544	XP_015697425.1 heterogeneous nuclear ribonucleoprotein R-like
TCONS_00015230	-0.847524	-0.889713	-1.71376	BGIOSGA033131	XP_006653085.1 probable methyltransferase PMT26
TCONS_00021199	-0.84752	-1.84235	0.448765	BGIOSGA023418	XP_015694457.1PREDICTED: uncharacterized protein LOC102722586
TCONS_00018463	-0.847185	0.631138	0.0796651	BGIOSGA018972	XP_015692444.1 methionine S-methyltransferase-like
TCONS_00022156	-0.847185	0.2161	-0.920335	BGIOSGA026715	XP_004965340.160S ribosomal protein L31
TCONS_00016943	-0.846687	0.868446	-0.607376	BGIOSGA023101	XP_017620756.1 putative phospholipid-transporting ATPase 9
TCONS_00027922	-0.846462	-1.36972	#NA	BGIOSGA027097	XP_015695839.1 protein tesmin/TSO1-like CXC 7
TCONS_00005379	-0.846206	-0.244883	-1.24578	BGIOSGA007490	XP_006646866.1 guanine nucleotide-binding protein subunit gamma 2-like
TCONS_00021945	-0.846074	-1.17517	-0.35478	BGIOSGA021574	XP_015694427.1 protein NLRC3
TCONS_00033745	-0.846014	-1.04079	-1.51796	BGIOSGA035431	XP_006663011.1 disease resistance protein RPP13-like
TCONS_00019058	-0.845935	-0.762061	-1.73218	BGIOSGA028965	XP_006654260.1 trans-cinnamate 4-monooxygenase-like
TCONS_00034312	-0.845564	0.867194	-0.501172	BGIOSGA034193	XP_006662734.1 60S ribosomal protein L26-1-like
TCONS_00036963	-0.845187	-1.31621	-0.973802	BGIOSGA031273	XP_004968848.1wall-associated receptor kinase 2
TCONS_00019285	-0.845144	-1.15443	-2.88302	BGIOSGA020096	XP_015692890.1 WAT1-related protein At5g07050-like
TCONS_00034455	-0.845072	#NA	0.88179	BGIOSGA007792	XP_022685016.1uncharacterized protein LOC111258248
TCONS_00002658	-0.84502	-1.5086	0.484896	BGIOSGA007226	XP_015688279.1 charged multivesicular body protein 5-like isoform X2
TCONS_00031508	-0.844559	0.689456	-0.916412	BGIOSGA010153	XP_002467615.1probable glutathione S-transferase GSTU6
TCONS_00004687	-0.844405	#NA	#NA	#N/A	#N/A
TCONS_00003113	-0.844173	-1.73123	-0.0322865	BGIOSGA031504	XP_015692237.1 jasmonic acid-amido synthetase JAR2-like
TCONS_00031569	-0.844081	-1.82928	2.076	BGIOSGA024325	XP_006662562.1 expansin-like A2
TCONS_00019122	-0.843974	-0.223249	0.175074	BGIOSGA026446	XP_006655221.2 probable AMP deaminase
TCONS_00027921	-0.843757	0.627177	#NA	#N/A	#N/A
TCONS_00006766	-0.843735	-0.298933	-0.0979506	BGIOSGA018983	XP_006647804.1 protein MEI2-like 2
TCONS_00003456	-0.84362	-1.81754	-1.70618	BGIOSGA030088	XP_021312225.1S-adenosylmethionine synthase 1
TCONS_00024301	-0.843549	-1.04418	-1.11019	BGIOSGA022793	XP_006658080.1 sodium/hydrogen exchanger 2
TCONS_00021900	-0.843544	-2.61846	-1.97309	BGIOSGA009055	XP_015693515.1 shikimate kinase 2, chloroplastic
TCONS_00018598	-0.843126	-1.9585	-1.19342	BGIOSGA018836	XP_015692982.1PREDICTED: uncharacterized protein LOC102710496
TCONS_00022580	-0.842512	-0.637255	0.483126	BGIOSGA033939	XP_015698206.1 scarecrow-like protein 3
TCONS_00002701	-0.842226	0.00306535	-3.18384	BGIOSGA035756	XP_006643674.1 glycerophosphodiester phosphodiesterase protein kinase domain-containing GDPDL2-like
TCONS_00015215	-0.842158	-3.39335	-2.13102	BGIOSGA015831	XP_006653071.1 probable receptor-like protein kinase At1g49730
TCONS_00022179	-0.841915	0.362082	-0.196427	BGIOSGA006710	XP_006656046.1PREDICTED: uncharacterized protein LOC102708251
TCONS_00004323	-0.841915	-1.37488	1.38854	BGIOSGA019401	XP_015696719.1PREDICTED: uncharacterized protein LOC102722439
TCONS_00025022	-0.841465	-3.48422	-1.81926	BGIOSGA013998	XP_003563185.1COP1-interacting protein 7
TCONS_00022295	-0.841459	-1.96577	-2.10753	BGIOSGA019251	XP_003560049.1 probable indole-3-acetic acid-amido synthetase GH3.7
TCONS_00023318	-0.841389	-1.37163	-0.932325	BGIOSGA028533	XP_015694869.1PREDICTED: uncharacterized protein LOC102710711
TCONS_00009499	-0.841333	-1.37553	-0.934835	#N/A	#N/A
TCONS_00005111	-0.841304	-1.50941	-1.43227	BGIOSGA005190	XP_006645380.1 probable NADPH:quinone oxidoreductase 1
TCONS_00010661	-0.841301	-1.13484	-0.924545	BGIOSGA007803	XP_002464100.1BOI-related E3 ubiquitin-protein ligase 1
TCONS_00000020	-0.841124	-1.02205	-0.942581	BGIOSGA012308	XP_006647280.2PREDICTED: uncharacterized protein At1g76660, partial
TCONS_00016462	-0.840759	-6.57138	-3.85096	BGIOSGA014509	XP_014751254.1ferric reduction oxidase 2
TCONS_00035078	-0.84061	0.0502039	-0.0735898	BGIOSGA000738	XP_015697994.1 ubiquitin carboxyl-terminal hydrolase 12-like
TCONS_00000399	-0.840212	1.94518	-0.93761	#N/A	XP_006643893.2 SCAR-like protein 2
TCONS_00009919	-0.839673	1.71013	-0.131589	BGIOSGA012473	XP_015691275.1 ubiquitin-conjugating enzyme E2 32 isoform X2
TCONS_00030717	-0.839673	#NA	#NA	BGIOSGA032533	XP_003573732.3putative lipid-transfer protein DIR1
TCONS_00037746	-0.839673	-0.377336	0.0610564	BGIOSGA040691	XP_003560362.2wall-associated receptor kinase 3
TCONS_00025076	-0.839147	0.20706	#NA	#N/A	#N/A
TCONS_00011628	-0.838933	-0.250806	0.724426	BGIOSGA034488	XP_021314174.1IQ domain-containing protein IQM1 isoform X2

transcriptome

TCONS_00023523	-0.838635	-1.37845	1.05849	BGIOSGA012449	XP_014755546.1anaphase-promoting complex subunit 11
TCONS_00019152	-0.838635	0.791474	0.643451	BGIOSGA018201	XP_002440974.1mitochondrial zinc maintenance protein 1, mitochondrial
TCONS_00016500	-0.838635	#NA	-1.94151	BGIOSGA028771	XP_006653710.1 lateral signaling target protein 2 homolog
TCONS_00008658	-0.838428	-2.15352	-0.0842862	BGIOSGA031008	XP_006647763.1 transcription factor bHLH63-like
TCONS_00009312	-0.838312	-1.72617	-0.497489	BGIOSGA011852	XP_002456220.1uncharacterized protein LOC8059153 isoform X1
TCONS_00013644	-0.837961	-0.573284	1.31539	BGIOSGA015796	XP_015692155.1 pyruvate dehydrogenase E1 component subunit alpha-3, chloroplastic
TCONS_00010469	-0.837647	0.620505	-0.943946	#N/A	#N/A
TCONS_00012588	-0.837245	-0.390409	-2.89394	BGIOSGA010386	XP_006650265.1 potassium transporter 27
TCONS_00004229	-0.837173	#NA	#NA	BGIOSGA036432	XP_004967389.2uncharacterized protein LOC101762180
TCONS_00010213	-0.837173	-1.37999	#NA	#N/A	#N/A
TCONS_00021357	-0.837151	-1.38435	-0.463663	BGIOSGA023059	XP_006656476.1 arogenate dehydrogenase 2, chloroplastic-like
TCONS_00020044	-0.837101	0.717857	0.323796	BGIOSGA035300	XP_006656591.1 nucleolar protein 10
TCONS_00011485	-0.836711	-2.96544	#NA	BGIOSGA011541	XP_014752791.1uncharacterized protein LOC100835806
TCONS_00020078	-0.836711	-1.38048	0.0537405	BGIOSGA019020	XP_015693955.1 auxin-induced protein 15A-like
TCONS_00032778	-0.836486	-0.525008	-0.598848	BGIOSGA009411	XP_015697348.1 inactive poly [ADP-ribose] polymerase RCD1
TCONS_00027064	-0.836263	1.4264	#NA	BGIOSGA025845	XP_004974082.1traB domain-containing protein
TCONS_00014667	-0.836263	-1.38095	#NA	#N/A	#N/A
TCONS_00001318	-0.835404	-0.464322	-0.419007	BGIOSGA003952	XP_006644348.1PREDICTED: uncharacterized protein LOC102704370
TCONS_00001995	-0.835404	-0.38186	#NA	BGIOSGA004629	XP_006644855.1 E3 ubiquitin-protein ligase RING1-like
TCONS_00019403	-0.835367	0.800808	0.140865	BGIOSGA007612	XP_002441194.2uncharacterized protein LOC8061745
TCONS_00013800	-0.835212	-1.02084	-0.700635	BGIOSGA004215	XP_006662117.1PREDICTED: uncharacterized protein LOC102704180
TCONS_00001964	-0.83505	0.0905336	-0.891405	BGIOSGA030974	XP_008674940.2subtilisin-like protease SBT3.5
TCONS_00028876	-0.835008	-0.548134	-0.941816	BGIOSGA008993	XP_003576467.1probable trehalose-phosphate phosphatase 7
TCONS_00020070	-0.83499	-2.3823	0.0494435	BGIOSGA007405	XP_004983138.1uncharacterized protein At4g22758
TCONS_00011177	-0.83499	1.11247	1.67747	#N/A	#N/A
TCONS_00011944	-0.8348	-2.21431	-1.89338	BGIOSGA001814	XP_006649783.1 protein FAF-like, chloroplastic
TCONS_00007874	-0.834586	1.20224	-0.951567	BGIOSGA011322	XP_020202243.1plasma membrane ATPase 3-like
TCONS_00018243	-0.834453	-1.18961	-0.571382	BGIOSGA007471	XP_015693208.1 phosphatidylinositol/phosphatidylcholine transfer protein SFH8-like isoform X4
TCONS_00001490	-0.834239	-1.75701	-0.773282	BGIOSGA020396	XP_006644451.1 CTP synthase-like
TCONS_00019944	-0.834191	-0.383143	#NA	BGIOSGA003441	XP_015693976.1 zinc finger protein CONSTANS-LIKE 12-like
TCONS_00009604	-0.834071	-0.530804	0.0127873	BGIOSGA000304	XP_006649660.1 two-component response regulator ORR21
TCONS_00018223	-0.833806	-1.38355	-0.953515	BGIOSGA017651	XP_010239928.1uncharacterized protein LOC100835989
TCONS_00003386	-0.83343	#NA	#NA	BGIOSGA030848	XP_004983817.1uncharacterized protein LOC101782683
TCONS_00010463	-0.83343	0.715589	0.145081	#N/A	#N/A
TCONS_00016882	-0.833068	-1.86286	-1.59854	BGIOSGA014081	XP_006653035.1 tetraspanin-19
TCONS_00019847	-0.833065	#NA	#NA	BGIOSGA014538	XP_006647666.1 amino acid transporter ANTL1-like
TCONS_00001373	-0.832978	-0.476206	-0.650096	BGIOSGA021125	XP_006644374.2 DEAD-box ATP-dependent RNA helicase 26
TCONS_00008976	-0.832709	#NA	#NA	BGIOSGA009223	XP_008780610.1PREDICTED: uncharacterized protein Os02g0798400-like
TCONS_00025133	-0.832362	0.199875	-0.957149	BGIOSGA024272	XP_014752362.2protein FAR1-RELATED SEQUENCE 5 isoform X3
TCONS_00029204	-0.832362	#NA	#NA	BGIOSGA026772	XP_006660734.1 EG45-like domain containing protein
TCONS_00011421	-0.832362	-2.23308	-1.31972	BGIOSGA033398	XP_006649242.1 arsenate reductase 2.2
TCONS_00013415	-0.832308	0.689032	0.201789	BGIOSGA037765	XP_003561529.3uncharacterized protein LOC100837842 isoform X1
TCONS_00025461	-0.832283	-0.00457375	-0.294579	BGIOSGA028363	XP_015694710.1 formin-like protein 5
TCONS_00037363	-0.832249	0.0147317	0.0917652	BGIOSGA036007	XP_015698422.1 eukaryotic translation initiation factor 5B
TCONS_00002736	-0.832236	-2.25705	-2.25149	BGIOSGA002072	XP_006643706.1 Bowman-Birk type bran trypsin inhibitor-like
TCONS_00030602	-0.832023	-1.38545	1.04199	BGIOSGA018470	XP_015692744.1 F-box protein At5g46170-like
TCONS_00033369	-0.832023	-0.537455	0.179497	#N/A	#N/A
TCONS_00005153	-0.831988	-0.89322	-0.748921	BGIOSGA000051	XP_015692546.1 coiled-coil domain-containing protein R3HCC1L
TCONS_00005340	-0.831905	-2.87605	-0.488903	BGIOSGA036419	XP_015689336.1 transcription factor HBP-1a-like isoform X1
TCONS_00034217	-0.831609	-2.8596	-2.99043	BGIOSGA030934	XP_006663775.2 protein DETOXIFICATION 21-like isoform X1
TCONS_00009368	-0.83151	-3.99137	-0.653806	BGIOSGA003234	XP_002468461.1uncharacterized protein LOC8059848
TCONS_00019249	-0.83137	-2.55608	0.598327	BGIOSGA018099	XP_006654367.1 cyclin-P3-1
TCONS_00008601	-0.83137	-0.649191	-0.544631	#N/A	#N/A
TCONS_00005979	-0.831177	-1.4807	-1.07845	BGIOSGA008112	XP_006665082.1 transcription factor bHLH121-like isoform X1
TCONS_00005625	-0.830868	-1.6666	-1.07358	BGIOSGA005713	XP_015688804.1 ADP-ribosylation factor GTPase-activating protein AGD12-like
TCONS_00024034	-0.830748	-0.216907	0.286672	BGIOSGA029883	XP_006664931.1 glyoxylate/hydroxypyruvate reductase HPR3-like

TCONS_00037111	-0.830748	-1.1238	-0.113259	BGIOSGA031394	XP_004957941.1probable histone H2A.1
TCONS_00032984	-0.830446	-0.480267	-1.15467	BGIOSGA029239	XP_006659696.1 40S ribosomal protein S25-4
TCONS_00009128	-0.830446	-2.09765	-2.54699	BGIOSGA035703	XP_006649226.1 DNA-directed RNA polymerases II, IV and V subunit 9A-like
TCONS_00004004	-0.829956	-0.729222	-1.7952	BGIOSGA023020	XP_006646115.1PREDICTED: uncharacterized protein LOC102715261
TCONS_00010061	-0.82962	-0.358904	-1.15938	BGIOSGA010090	XP_015690277.1 40S ribosomal protein S21
TCONS_00029797	-0.829578	-0.873523	-2.77158	BGIOSGA012549	XP_006650025.1 zinc finger CCCH domain-containing protein 22
TCONS_00020245	-0.829556	-0.735162	-0.123311	BGIOSGA022433	XP_015694123.1 ABC transporter C family member 13
TCONS_00006694	-0.828569	0.284731	-0.126924	BGIOSGA008890	XP_006648956.2 ATPase family AAA domain-containing protein 3-B-like
TCONS_00031031	-0.828298	-2.07824	-2.4202	BGIOSGA032847	XP_014661364.1uncharacterized protein LOC101785497
TCONS_00019514	-0.828285	-0.689513	-0.758992	BGIOSGA012556	XP_006654555.1 2,3-bisphosphoglycerate-independent phosphoglycerate mutase-like
TCONS_00028241	-0.828225	-1.1803	-0.0950098	BGIOSGA006399	XP_003572341.2WPP domain-interacting protein 1
TCONS_00036025	-0.828182	-2.67936	-1.527	BGIOSGA030133	XP_015698718.1 serine/threonine-protein kinase D6PKL1-like
TCONS_00030883	-0.82753	-3.09942	-3.77734	BGIOSGA021484	XP_006665050.1 flavonoid 3'-monooxygenase
TCONS_00003041	-0.827522	-0.493434	-0.154978	BGIOSGA006828	XP_006643882.2 aquaporin NIP1-2-like
TCONS_00001654	-0.827522	0.609659	0.352506	#N/A	#N/A
TCONS_00021951	-0.82729	0.931329	0.351915	BGIOSGA026961	XP_002436775.2U-box domain-containing protein 39
TCONS_00020637	-0.82729	-0.3906	#NA	BGIOSGA027641	XP_004965358.1uncharacterized protein LOC101756485
TCONS_00029089	-0.827063	0.194109	-0.311629	BGIOSGA004530	XP_020404094.1copper transporter 5.1
TCONS_00024896	-0.82699	-1.12078	-0.641673	BGIOSGA004969	XP_006657600.1 putative metallophosphoesterase At3g03305 isoform X1
TCONS_00024835	-0.826655	-1.24912	0.32038	BGIOSGA007905	XP_015694799.1 probable polygalacturonase
TCONS_00020549	-0.826623	#NA	#NA	BGIOSGA025435	XP_002468294.1UDP-glucose flavonoid 3-O-glucosyltransferase 7
TCONS_00036264	-0.826474	-1.28718	-2.15167	BGIOSGA035731	XP_004962706.1osmotin-like protein
TCONS_00011860	-0.825791	-0.54429	-0.421285	BGIOSGA018443	XP_006651204.1PREDICTED: uncharacterized protein LOC102715561
TCONS_00014675	-0.825675	-0.306559	-0.868854	BGIOSGA008714	XP_015692212.1 GATA transcription factor 5-like
TCONS_00001636	-0.825593	#NA	-0.974327	BGIOSGA019837	XP_003569627.1membrane protein PM19L
TCONS_00014413	-0.825398	#NA	0.688147	BGIOSGA007446	XP_006652389.1 MLP-like protein 423
TCONS_00000089	-0.825237	0.133211	-0.264719	BGIOSGA016925	XP_006665126.1PREDICTED: uncharacterized protein LOC102715819
TCONS_00019439	-0.824658	-0.879003	0.122846	BGIOSGA004769	XP_006654515.1 50S ribosomal protein L17-like
TCONS_00000523	-0.824482	#NA	#NA	BGIOSGA016473	XP_006643970.1 CASP-like protein 1C1-1
TCONS_00033597	-0.82414	1.66472	-2.56297	BGIOSGA019486	XP_002464185.1selenoprotein H
TCONS_00011424	-0.823812	-0.0726318	0.343089	BGIOSGA015010	XP_002466011.1uncharacterized protein LOC8078025
TCONS_00018351	-0.823495	0.0904951	-2.30157	#N/A	#N/A
TCONS_00012441	-0.823367	-0.151921	0.00420052	BGIOSGA031746	XP_015690456.1 zinc transporter 2-like
TCONS_00006023	-0.822833	-2.3569	-0.797242	BGIOSGA030697	XP_003536688.1ubiquitin receptor RAD23c
TCONS_00006143	-0.82261	-2.39598	#NA	BGIOSGA029245	XP_004974189.1uncharacterized protein LOC101772750
TCONS_00018260	-0.822471	-1.65918	-0.567208	BGIOSGA005018	XP_024315680.1uncharacterized protein LOC100838382
TCONS_00008852	-0.822471	#NA	1.60272	BGIOSGA028839	XP_006647953.1PREDICTED: uncharacterized protein LOC102720304
TCONS_00001310	-0.821812	-2.9819	-0.983917	BGIOSGA003110	XP_002455911.1uncharacterized protein LOC8082509
TCONS_00008986	-0.821687	-0.66012	-2.5692	BGIOSGA013021	XP_006650279.1 cyclin-B1-2
TCONS_00035755	-0.821687	1.82531	0.752732	#N/A	#N/A
TCONS_00003993	-0.821564	-0.275245	-0.824081	BGIOSGA001200	XP_020400600.1uncharacterized LOC103631680 isoform X1
TCONS_00023545	-0.821564	-1.07531	-1.98455	#N/A	#N/A
TCONS_00037408	-0.821327	-1.7557	-1.376	BGIOSGA035958	XP_006664130.1PREDICTED: uncharacterized protein LOC102705124
TCONS_00026824	-0.820645	-4.20571	-0.624296	BGIOSGA003805	XP_015695843.1PREDICTED: uncharacterized protein LOC102718014
TCONS_00003850	-0.820433	-2.39861	-0.615432	BGIOSGA026208	XP_003569243.1NDR1/HIN1-like protein 13
TCONS_00020987	-0.820329	-3.9837	#NA	BGIOSGA002288	XP_006657109.2 transcription factor HY5
TCONS_00017520	-0.820227	0.338102	-1.57288	BGIOSGA010742	XP_006652182.1 (+)-germacrene D synthase-like
TCONS_00028500	-0.81974	0.237965	-0.351717	BGIOSGA026457	XP_006659720.1 RNA-binding protein 42-like isoform X2
TCONS_00012362	-0.81974	-0.524996	-1.57411	BGIOSGA037627	XP_004984201.2uncharacterized protein LOC101757961
TCONS_00023936	-0.819571	-0.361023	-0.0104558	BGIOSGA037843	XP_015695270.1 zinc finger CCCH domain-containing protein 50
TCONS_00000289	-0.819319	-0.912355	-2.20434	BGIOSGA033119	XP_006645543.1 phosphoenolpyruvate/phosphate translocator 3, chloroplastic-like
TCONS_00001742	-0.819287	-1.13699	-3.31222	BGIOSGA000895	XP_006644647.1 CASP-like protein 3A1
TCONS_00037025	-0.819287	0.0405461	0.450286	BGIOSGA014856	XP_006652425.1 mitochondrial inner membrane protease ATP23
TCONS_00005733	-0.819115	0.921687	-0.99072	#N/A	#N/A
TCONS_00020418	-0.818867	-0.178159	0.00865216	BGIOSGA022603	NP_001146539.1nucleic acid binding protein
TCONS_00011906	-0.818827	-1.30307	-2.16895	BGIOSGA032230	XP_006649748.1PREDICTED: uncharacterized protein LOC102709156
TCONS_00022414	-0.81855	0.599053	-1.57711	BGIOSGA002188	XP_002438556.2uncharacterized protein LOC8076704
TCONS_00014718	-0.818251	#NA	-1.9929	BGIOSGA016892	XP_006652616.1 cyclin-P2-1
TCONS_00036405	-0.818251	0.335643	0.229494	BGIOSGA037773	XP_006664731.1 WD repeat-containing protein 75

TCONS_00019246	-0.818178	-3.45727	-3.04893	BGIOSGA004804	XP_006654363.1PREDICTED: uncharacterized protein LOC102717800
TCONS_00016447	-0.818177	-2.45766	-1.6083	BGIOSGA004975	XP_015691546.1 protein IQ-DOMAIN 14-like
TCONS_00028119	-0.818107	#NA	0.229131	BGIOSGA007378	XP_006659476.2 germin-like protein 8-13
TCONS_00018810	-0.818107	0.335462	-1.99326	BGIOSGA031595	XP_006654148.2 shaggy-related protein kinase eta-like
TCONS_00001540	-0.817763	-0.32271	-0.0294591	BGIOSGA023494	XP_006644491.1 ras-related protein RABA1f
TCONS_00009426	-0.817697	-1.08009	-1.40933	BGIOSGA005180	XP_004985498.1germin-like protein 3-1
TCONS_00038972	-0.817503	#NA	-0.994779	BGIOSGA038135	XP_006657694.1 serine carboxypeptidase-like
TCONS_00010523	-0.817378	2.04236	0.294413	BGIOSGA020338	XP_015691206.1 T-complex protein 1 subunit beta
TCONS_00029066	-0.817256	#NA	#NA	BGIOSGA000566	XP_006647829.1 sodium-coupled neutral amino acid transporter 1 isoform X2
TCONS_00020301	-0.817136	-0.495837	-0.995699	BGIOSGA014480	XP_006655890.1PREDICTED: uncharacterized protein LOC102709653
TCONS_00036263	-0.817136	-1.40273	-1.58066	BGIOSGA023474	XP_004962706.1osmotin-like protein
TCONS_00034186	-0.817078	0.552804	-1.28535	BGIOSGA022298	XP_006663758.2PREDICTED: uncharacterized protein LOC102708666
TCONS_00023654	-0.817058	-1.00387	-0.449719	BGIOSGA019408	NP_001147782.1cyclopropane fatty acid synthase
TCONS_00003328	-0.81702	-1.81791	-2.99599	BGIOSGA026234	XP_006644068.1 eukaryotic translation initiation factor 6-2-like
TCONS_00027112	-0.816906	#NA	-2.99628	BGIOSGA034849	XP_015696185.1PREDICTED: uncharacterized protein LOC107303388
TCONS_00002061	-0.81679	0.0495443	-0.542756	BGIOSGA017849	XP_021312803.1protein PIN-LIKES 6
TCONS_00014928	-0.816686	-1.59594	-0.826903	BGIOSGA022872	XP_015691698.1 MADS-box transcription factor 31-like isoform X1
TCONS_00007129	-0.815973	-1.25152	-1.11157	BGIOSGA005130	XP_006648142.1 putative transporter arsB
TCONS_00031582	-0.81581	-3.40441	-0.999019	BGIOSGA007965	XP_006662567.1 xyloglucan endotransglucosylase/hydrolase protein 31-like
TCONS_00004138	-0.81568	-1.40457	1.00066	BGIOSGA008319	XP_021313026.1splicing factor U2af small subunit B
TCONS_00001585	-0.815595	-0.925512	-0.584519	BGIOSGA004218	XP_006644531.1 protein prenyltransferase alpha subunit repeat-containing protein 1-B
TCONS_00016565	-0.815512	-1.40478	-0.718582	#N/A	#N/A
TCONS_00009694	-0.815431	-4.46378	1.48546	#N/A	#N/A
TCONS_00012784	-0.815391	-1.66797	-0.777674	BGIOSGA006938	XP_004982353.1F-box/LRR-repeat protein At3g48880
TCONS_00006217	-0.815196	0.764741	0.699888	BGIOSGA029120	XP_003575090.1protein MKS1
TCONS_00031316	-0.815121	-1.89071	-2.22313	BGIOSGA024987	XP_006664818.1 protein CUP-SHAPED COTYLEDON 1-like
TCONS_00019301	-0.815011	0.842509	-2.00101	BGIOSGA028630	XP_012700131.1LOB domain-containing protein 6
TCONS_00001994	-0.814869	-0.668632	-2.00137	BGIOSGA004630	XP_006644855.1 E3 ubiquitin-protein ligase RING1-like
TCONS_00027686	-0.8148	-1.82072	0.168386	BGIOSGA034994	XP_015695553.1PREDICTED: uncharacterized protein LOC102703056
TCONS_00009631	-0.814601	-1.05801	-1.00204	BGIOSGA026955	XP_004985119.1homeobox-leucine zipper protein HOX19 isoform X2
TCONS_00032379	-0.814601	-1.40594	-2.00204	BGIOSGA028620	XP_006661838.1 hypersensitive-induced response protein 1-like
TCONS_00010796	-0.81458	0.140169	-0.0127793	BGIOSGA011897	XP_006650473.1 DELLA protein SLR1
TCONS_00009156	-0.814536	-1.92059	0.260837	BGIOSGA028985	XP_021302193.1cell division control protein 2 homolog
TCONS_00004803	-0.814463	-0.676682	-0.297203	BGIOSGA000412	XP_003564737.13-phosphoinositide-dependent protein kinase 2
TCONS_00002177	-0.814232	1.82626	1.13455	BGIOSGA004813	XP_015699226.1 fructokinase-like 1, chloroplastic
TCONS_00024296	-0.814145	-2.40651	1.69727	BGIOSGA035354	XP_002463347.1momilactone A synthase
TCONS_00003896	-0.814033	-0.111197	-2.14095	BGIOSGA030592	XP_006644319.2 hypersensitive-induced response protein 1-like
TCONS_00029171	-0.81382	-3.40692	-2.00398	BGIOSGA019134	XP_003578245.12-hydroxyisoflavanone dehydratase
TCONS_00025947	-0.813795	0.429547	0.580919	BGIOSGA028023	XP_006659147.1 dnaJ homolog subfamily C member 2
TCONS_00000913	-0.813601	-0.82839	-0.543212	BGIOSGA019732	XP_015699241.1 40S ribosomal protein S4-like isoform X3
TCONS_00034093	-0.813525	-3.05937	-0.820289	BGIOSGA012244	XP_006663127.1PREDICTED: uncharacterized protein LOC102702225
TCONS_00031481	-0.813502	-0.489786	-1.36734	BGIOSGA016943	XP_008659873.1RHOMBOID-like protein 2
TCONS_00002500	-0.813502	0.293116	1.7367	BGIOSGA017363	XP_006645312.2 glucan endo-1,3-beta-glucosidase, acidic isoform-like
TCONS_00025713	-0.813455	-0.0405999	-2.93089	BGIOSGA014615	XP_006658134.1 WRKY transcription factor SUSIBA2-like
TCONS_00013712	-0.813213	-1.89311	-0.642919	BGIOSGA032160	XP_004975056.3zinc finger protein ZAT5-like
TCONS_00018534	-0.81315	-0.085839	-0.83572	BGIOSGA006747	XP_015693246.1PREDICTED: uncharacterized protein LOC102700143
TCONS_00012695	-0.813089	-0.0859165	-0.783405	BGIOSGA023984	XP_006651592.2PREDICTED: uncharacterized protein LOC102711962
TCONS_00032973	-0.813068	-1.40787	-0.59081	BGIOSGA019723	NP_001105567.1root cap periphery gene 2 precursor
TCONS_00011976	-0.812893	-0.789182	-0.491711	BGIOSGA004796	XP_015691139.1 probable mannose-1-phosphate guanylyltransferase 1
TCONS_00031042	-0.812893	-0.408091	-0.491711	BGIOSGA034002	XP_015697750.1 putative disease resistance protein RGA4
TCONS_00031473	-0.812782	0.106342	0.709647	BGIOSGA019185	XP_022681533.1uncharacterized protein LOC101778128 isoform X2
TCONS_00008939	-0.812745	0.430442	0.559698	BGIOSGA009705	XP_003570262.1mitogen-activated protein kinase kinase 5
TCONS_00023894	-0.812745	-0.145243	-0.328577	BGIOSGA024312	XP_002460844.1uncharacterized protein LOC8081736 isoform X1
TCONS_00028415	-0.812675	0.0322066	-0.0700185	BGIOSGA029420	XP_006659652.1 transcription factor PCF2
TCONS_00010207	-0.812657	-0.408388	-2.42191	BGIOSGA036770	XP_003561584.1probable trehalose-phosphate phosphatase 9

TCONS_00016701	-0.812347	-2.06086	-4.46707	BGIOSGA014262	XP_019710597.1 transcription termination factor MTERF6, chloroplastic/mitochondrial-like
TCONS_00009270	-0.812332	-0.176851	-0.527049	BGIOSGA006899	XP_015690852.1 probable protein phosphatase 2C 28
TCONS_00009070	-0.812332	-1.55564	-1.87441	BGIOSGA027894	XP_006648153.1 AT-hook motif nuclear-localized protein 9-like
TCONS_00021545	-0.812316	-5.40882	-2.00771	BGIOSGA031062	XP_006655752.1 NAC domain-containing protein 43-like
TCONS_00020050	-0.812271	-0.224449	0.682491	BGIOSGA006092	XP_006656659.2 GDSL esterase/lipase At5g45910-like
TCONS_00017540	-0.81203	-1.82421	-4.00842	BGIOSGA037494	XP_006655193.2 putative mannan endo-1,4-beta-mannosidase 5
TCONS_00004829	-0.811787	0.0869432	-2.49445	BGIOSGA025756	XP_006645122.1 protein trichome berefringence-like 7
TCONS_00037284	-0.811635	-0.599335	-1.21103	BGIOSGA036091	XP_006664066.1 guanylate kinase 1
TCONS_00010544	-0.811613	0.442741	0.280052	BGIOSGA012439	XP_004968836.1pentatricopeptide repeat-containing protein At5g66500, mitochondrial
TCONS_00002309	-0.811558	0.327194	0.520923	BGIOSGA022297	XP_015695832.1 NAC transcription factor ONAC010
TCONS_00001188	-0.811558	1.53364	-0.388103	BGIOSGA035894	XP_010231939.1putative transcription factor bHLH041
TCONS_00022440	-0.811536	-1.23987	1.03475	BGIOSGA021021	XP_006656171.1 cysteine synthase-like
TCONS_00007537	-0.811493	0.360666	0.0320695	BGIOSGA006946	XP_006648423.1 ultraviolet-B receptor UVR8
TCONS_00001590	-0.811483	-0.35924	-3.81713	BGIOSGA035541	XP_021312281.1limonoid UDP-glucosyltransferase
TCONS_00026805	-0.81135	-0.410032	0.463827	BGIOSGA032055	XP_015695729.1 probable glucan 1,3-beta-glucosidase A
TCONS_00017174	-0.811264	-0.0762402	0.305586	BGIOSGA027666	XP_006644248.1PREDICTED: uncharacterized protein LOC102721967 isoform X1
TCONS_00023731	-0.811217	-2.4102	-2.5954	BGIOSGA032130	XP_015698251.1 anthocyanidin 3-O-glucosyltransferase 2-like
TCONS_00002605	-0.811199	0.17474	0.989522	BGIOSGA000858	XP_006645346.2 ABC transporter D family member 1-like
TCONS_00005390	-0.811199	-1.62703	-1.43674	BGIOSGA029776	XP_004951929.1aspartate--tRNA ligase 2, cytoplasmic
TCONS_00011113	-0.81119	2.0492	-1.81786	BGIOSGA012138	XP_006650710.1 glucan endo-1,3-beta-glucosidase 8-like
TCONS_00015078	-0.811127	-1.41031	0.512907	BGIOSGA033111	XP_014750974.1ubiquitin thioesterase otubain-like isoform X2
TCONS_00031451	-0.81105	-3.21776	-2.49627	BGIOSGA028809	XP_003574177.1cytochrome P450 89A2
TCONS_00031827	-0.810984	-5.93405	-2.72722	BGIOSGA032747	XP_015691385.1 cytochrome P450 76C1-like
TCONS_00009137	-0.810896	0.699022	-0.451798	BGIOSGA008764	XP_006650951.2 scarecrow-like protein 6
TCONS_00034216	-0.810866	-4.87007	-4.47073	BGIOSGA030934	XP_006663775.2 protein DETOXIFICATION 21-like isoform X1
TCONS_00004472	-0.810727	-0.734705	-0.557926	BGIOSGA034468	XP_004970093.2lysosomal Pro-X carboxypeptidase
TCONS_00026074	-0.81072	-0.437296	0.149494	BGIOSGA034333	XP_002445194.1putative F-box/LRR-repeat protein At5g41840 isoform X1
TCONS_00006786	-0.810474	-2.27989	-2.40459	BGIOSGA033446	XP_006647820.1 protein kinase G11A-like
TCONS_00006042	-0.81022	0.116928	-0.543408	BGIOSGA017591	XP_006647301.1 MACPF domain-containing protein At1g14780-like
TCONS_00014990	-0.810166	0.147908	-1.4536	BGIOSGA007922	XP_006653789.1 putative disease resistance protein RGA4
TCONS_00028816	-0.810156	0.0447888	0.160666	BGIOSGA028608	XP_004956634.1proton pump-interactor BIP103
TCONS_00002889	-0.810151	-0.919685	-0.883781	BGIOSGA002324	XP_006645526.1 DEAD-box ATP-dependent RNA helicase 18
TCONS_00037143	-0.810146	-2.41154	-0.691148	BGIOSGA024901	XP_006650657.1PREDICTED: uncharacterized protein LOC102714997
TCONS_00015310	-0.810027	-1.06377	-0.88784	BGIOSGA012264	XP_010228842.2dynamin-related protein 4C
TCONS_00005604	-0.809966	1.83616	0.334404	BGIOSGA023433	XP_006648408.2 protein LYK5
TCONS_00001607	-0.809937	-2.28627	-2.15109	BGIOSGA021705	XP_003567011.1LEAF RUST 10 DISEASE-RESISTANCE LOCUS RECEPTOR-LIKE PROTEIN KINASE-like 2.5
TCONS_00002714	-0.809929	-0.836314	-0.276646	BGIOSGA002521	XP_015699285.1 probable receptor-like protein kinase At1g67000
TCONS_00017026	-0.809925	-0.685996	-0.906706	BGIOSGA018135	XP_004960311.1ABC transporter G family member 16
TCONS_00033351	-0.80979	-1.34786	-0.666031	BGIOSGA035179	XP_004979200.1probable pre-mRNA-splicing factor ATP-dependent RNA helicase DEAH4
TCONS_00011994	-0.809678	#NA	-3.6923	BGIOSGA004808	XP_015691244.1 laccase-10
TCONS_00016114	-0.809636	-1.9442	-0.133749	BGIOSGA010051	XP_003581361.1probable galactinol--sucrose galactosyltransferase 2
TCONS_00015435	-0.809558	0.909647	-1.01452	BGIOSGA015262	XP_004975176.1G-type lectin S-receptor-like serine/threonine-protein kinase LECRK4
TCONS_00036241	-0.809549	-1.18367	0.24484	BGIOSGA020820	XP_003579150.1rhodanese-like domain-containing protein 6 isoform X4
TCONS_00030394	-0.809526	1.16717	-0.889074	BGIOSGA031527	XP_015696802.1 homeobox-leucine zipper protein ROC6-like
TCONS_00019768	-0.809362	-0.0205206	-0.95742	BGIOSGA017588	XP_015692915.1 probable ubiquitin-conjugating enzyme E2 24 isoform X1
TCONS_00035215	-0.809299	0.51598	1.64694	BGIOSGA033611	XP_015697721.1 glycine-rich domain-containing protein 1-like
TCONS_00009005	-0.809204	0.392926	-0.734587	BGIOSGA005402	XP_006648078.1 clathrin interactor EPSIN 2
TCONS_00022952	-0.809155	-1.17033	-0.621854	BGIOSGA033796	XP_006655356.1 ABC transporter G family member 28-like
TCONS_00026933	-0.808772	-2.46988	-1.32114	BGIOSGA028735	XP_006660274.1 psbP domain-containing protein 3, chloroplastic
TCONS_00004284	-0.808404	-1.1824	-0.518555	BGIOSGA009390	XP_015697975.1 myosin-17-like isoform X2
TCONS_00035242	-0.807794	-0.575077	-0.486448	BGIOSGA009211	XP_015697726.1 splicing factor U2af large subunit A
TCONS_00005001	-0.80764	0.0892643	-1.24028	BGIOSGA031229	XP_006645262.1 probable serine/threonine-protein kinase At1g54610 isoform X1
TCONS_00033202	-0.807514	-1.99047	-1.02448	BGIOSGA035037	XP_006662833.1 disease resistance protein RPM1-like
TCONS_00006223	-0.806928	-1.55113	0.419245	BGIOSGA010557	XP_015689218.1 serine decarboxylase 1

TCONS_00005547	-0.804843	-1.0573	-0.590345	#N/A	XP_008460445.1 FGGY carbohydrate kinase domain-containing protein
TCONS_00028840	-0.804344	-0.669494	-0.561266	BGIOSGA036737	XP_006661159.1 probable receptor-like protein kinase At5g15080
TCONS_00029334	-0.804044	-0.43454	0.66471	BGIOSGA031082	XP_015696421.1 U11/U12 small nuclear ribonucleoprotein 35 kDa protein
TCONS_00023345	-0.803977	-1.09617	-1.63268	BGIOSGA025386	XP_015694561.1 mediator of RNA polymerase II transcription subunit 33A-like
TCONS_00014689	-0.80376	0.27752	0.494079	BGIOSGA004029	XP_006653630.1 leiomodin-2-like
TCONS_00011905	-0.803679	-0.231347	-1.01026	BGIOSGA034638	XP_006649746.1PREDICTED: uncharacterized protein LOC102708596
TCONS_00014923	-0.803585	0.156925	-0.455302	BGIOSGA027460	XP_006450366.140S ribosomal protein S11
TCONS_00028874	-0.802798	0.512201	0.10122	BGIOSGA032849	XP_006660575.1 ethylene-responsive transcription factor RAP2-13
TCONS_00029266	-0.802777	-0.874823	-0.801253	BGIOSGA031014	XP_006660769.2PREDICTED: uncharacterized protein LOC102710811
TCONS_00032167	-0.802706	-1.63835	-0.268658	BGIOSGA007941	XP_006644747.1 glutelin type-A 1
TCONS_00008618	-0.802117	0.242868	0.0879694	BGIOSGA006963	NP_001183696.1PLATZ transcription factor
TCONS_00016618	-0.801967	-1.66054	0.16233	BGIOSGA025878	XP_015692044.1 probable LRR receptor-like serine/threonine-protein kinase At1g56140
TCONS_00020190	-0.801803	-0.913932	-0.327204	BGIOSGA022381	XP_006655827.1 PTI1-like tyrosine-protein kinase At3g15890 isoform X1
TCONS_00002821	-0.801099	1.56337	0.156767	#N/A	XP_013442996.1senescence-associated protein
TCONS_00032498	-0.800727	0.03661	-0.191113	BGIOSGA031619	XP_006661904.2 DNA-directed RNA polymerase I subunit 2 isoform X1
TCONS_00000472	-0.799572	-1.19545	-1.18836	BGIOSGA016437	XP_015699180.1 proline-rich receptor-like protein kinase PERK1
TCONS_00014503	-0.799251	-0.500881	-0.934296	BGIOSGA023261	XP_006653556.1 DNA-directed RNA polymerase III subunit 1
TCONS_00031838	-0.799245	-2.98426	-3.17004	BGIOSGA012214	XP_004970732.1protein NRT1/ PTR FAMILY 5.16
TCONS_00037090	-0.799155	-2.01798	-0.720922	BGIOSGA010891	XP_015698667.1 beta-galactosidase 15 isoform X2
TCONS_00005857	-0.798712	0.335481	0.198428	BGIOSGA003522	XP_006647174.1 muscle M-line assembly protein unc-89-like isoform X1
TCONS_00007150	-0.798619	-0.386813	-0.697094	BGIOSGA035748	XP_006648160.2 sucrose transport protein SUT4 isoform X2
TCONS_00035686	-0.798086	-0.208617	-1.41582	BGIOSGA037104	XP_015698451.1 histone deacetylase 14 isoform X3
TCONS_00010042	-0.79776	0.00815909	-0.562166	BGIOSGA012593	XP_006650059.1 spidroin-2
TCONS_00012710	-0.79726	-1.44427	-0.00340521	BGIOSGA012076	XP_006650278.1 BTB/POZ domain-containing protein NPY4-like isoform X2
TCONS_00014273	-0.796957	-0.12518	-0.413242	BGIOSGA013898	XP_006653403.1 nicotinate phosphoribosyltransferase 2-like isoform X2
TCONS_00031852	-0.796838	0.117176	0.0701722	BGIOSGA034201	XP_015697425.1 heterogeneous nuclear ribonucleoprotein R-like
TCONS_00032308	-0.795517	0.184415	-0.0582969	BGIOSGA033266	XP_015697399.1 probable inorganic phosphate transporter 1-8
TCONS_00022356	-0.795478	-4.59603	-2.19623	BGIOSGA032386	XP_006658218.1 flavin-containing monooxygenase FMO GS-OX-like 9
TCONS_00014750	-0.795429	-0.311676	-0.985319	BGIOSGA008782	XP_006653679.1 HMG1/2-like protein
TCONS_00008579	-0.79517	-0.295738	0.171906	BGIOSGA025984	XP_015688778.1 40S ribosomal protein S13-2
TCONS_00032301	-0.795102	-1.09779	-1.78003	BGIOSGA023394	XP_015697003.1 probable receptor-like protein kinase At3g55450
TCONS_00011289	-0.79438	0.705191	-0.874593	BGIOSGA020449	NP_001150798.1harpin-induced protein
TCONS_00022732	-0.792567	-0.504225	-1.21266	BGIOSGA005499	XP_015694354.1 probable allantoate deiminase isoform X2
TCONS_00035253	-0.792008	-1.07192	0.0994931	BGIOSGA033654	XP_006663071.1 armadillo repeat-containing protein 8
TCONS_00008797	-0.791153	-2.92668	-0.274111	BGIOSGA011757	XP_004953880.1auxin efflux carrier component 1a
TCONS_00019546	-0.79095	-1.32074	1.45828	BGIOSGA017812	XP_006655437.2 SNF1-related protein kinase regulatory subunit beta-1-like
TCONS_00004443	-0.790944	-0.225793	0.14688	BGIOSGA006708	XP_015689950.1 protein tesmin/TSO1-like CXC 5
TCONS_00034694	-0.790733	-2.74452	-1.96623	BGIOSGA021652	XP_015689597.1 F-box protein FBW2-like
TCONS_00005949	-0.790031	-2.84655	-3.22337	BGIOSGA001230	XP_004954858.2mitogen-activated protein kinase kinase kinase 18
TCONS_00036590	-0.78983	0.139661	-0.088429	BGIOSGA034584	XP_003577865.140S ribosomal protein S16
TCONS_00010090	-0.789762	-2.01696	-0.295363	BGIOSGA005872	XP_002467852.1U4/U6 small nuclear ribonucleoprotein PRP4-like protein
TCONS_00028622	-0.789469	-0.306833	-0.170556	BGIOSGA007345	XP_006660465.2 beta-fructofuranosidase, insoluble isoenzyme 7-like
TCONS_00002194	-0.789396	-1.37958	-1.67932	BGIOSGA017953	NP_001152198.1starch binding domain containing protein
TCONS_00030608	-0.789298	-1.44848	-0.720961	BGIOSGA032433	NP_001148246.1NAD(P)H-dependent oxidoreductase
TCONS_00037004	-0.789136	-1.13992	-0.527525	BGIOSGA033568	XP_015698266.1 putative disease resistance RPP13-like protein 3
TCONS_00014727	-0.788872	0.236738	-0.555358	BGIOSGA008764	XP_002446917.1scarecrow-like protein 6
TCONS_00037409	-0.788729	-2.18522	-0.884194	BGIOSGA029048	XP_015698401.1 probable inactive purple acid phosphatase 1
TCONS_00031559	-0.788351	-1.11328	-1.043	BGIOSGA034009	XP_004983796.1monosaccharide-sensing protein 2
TCONS_00009162	-0.787127	0.10244	-0.779902	BGIOSGA037013	XP_006649250.2PREDICTED: uncharacterized protein LOC102706362
TCONS_00006281	-0.786564	-0.971905	-0.605664	BGIOSGA012442	XP_006647388.1 calmodulin-binding protein 60 D-like isoform X1
TCONS_00026174	-0.786456	-3.92075	-2.38308	BGIOSGA019835	XP_014756540.1endo-1,3;1,4-beta-D-glucanase isoform X2
TCONS_00005328	-0.785835	-3.43351	-0.977574	BGIOSGA007436	XP_006646821.1PREDICTED: uncharacterized protein LOC102716930
TCONS_00012914	-0.785607	-3.39707	-1.51907	BGIOSGA001873	XP_006650400.1 CSC1-like protein RXW8 isoform X2

TCONS_00021025	-0.785416	-4.04796	-3.49652	BGIOSGA022041	XP_003563633.1aspartyl protease family protein At5g10770
TCONS_00018679	-0.78532	-1.25937	-0.492869	BGIOSGA028598	XP_015693299.1 acyl-coenzyme A oxidase 4, peroxisomal-like
TCONS_00026800	-0.784743	-0.101557	-0.240175	BGIOSGA003815	XP_006659506.1 14-3-3-like protein GF14-A
TCONS_00005953	-0.784588	-0.621072	0.332595	BGIOSGA008090	NP_001150485.2senescence-associated protein
TCONS_00018007	-0.784461	0.984074	-0.763925	BGIOSGA026954	XP_010231260.1serine/threonine-protein kinase STN8, chloroplastic
TCONS_00010591	-0.784242	0.605183	-0.432359	BGIOSGA015291	XP_006650340.1 beclin-1-like protein
TCONS_00005013	-0.783909	-0.277532	0.584176	BGIOSGA000205	XP_006646640.1 coiled-coil domain-containing protein SCD2
TCONS_00003010	-0.78374	0.606555	0.0574509	BGIOSGA002207	XP_006645596.1 regulatory protein NPR1
TCONS_00003073	-0.783633	0.0904886	-2.54104	BGIOSGA035843	XP_004968601.1cationic amino acid transporter 5
TCONS_00007825	-0.783104	-0.784395	0.205394	BGIOSGA000363	XP_006647186.2 CLP protease regulatory subunit CLPX1, mitochondrial
TCONS_00015509	-0.783104	-0.306668	-0.0973247	BGIOSGA021985	XP_010239737.1probable protein transport Sec1a
TCONS_00002211	-0.782887	-1.22989	-1.32455	BGIOSGA004845	XP_006646498.1PREDICTED: uncharacterized protein LOC102721231
TCONS_00014924	-0.782398	-1.10017	-0.228641	BGIOSGA038236	XP_006657844.2PREDICTED: uncharacterized protein LOC102704159 isoform X1
TCONS_00033646	-0.782305	-0.931107	-1.5337	BGIOSGA024454	XP_006663522.2 putative disease resistance RPP13-like protein 3
TCONS_00023745	-0.781999	-0.29708	0.0471038	BGIOSGA012138	XP_006657748.1 glucan endo-1,3-beta-glucosidase 1-like
TCONS_00011462	-0.781898	-3.3785	-2.44694	BGIOSGA033641	XP_006649290.1PREDICTED: uncharacterized protein LOC102718154
TCONS_00011759	-0.78177	-3.60615	-1.14719	BGIOSGA025045	XP_006649595.1PREDICTED: uncharacterized protein LOC102712511
TCONS_00036256	-0.781209	0.425202	-0.368272	BGIOSGA037612	XP_021301510.160S ribosomal protein L8
TCONS_00026020	-0.781033	-5.50038	-2.78132	BGIOSGA020093	XP_006659181.1 G-type lectin S-receptor-like serine/threonine-protein kinase B120 isoform X2
TCONS_00018625	-0.780791	-1.98746	-1.11072	BGIOSGA018809	XP_015693323.1 probable calcium-binding protein CML22
TCONS_00006082	-0.780789	-1.66718	-1.44968	BGIOSGA031102	XP_015688599.1 UDP-glycosyltransferase 85A2-like
TCONS_00009203	-0.780754	-0.933764	-0.782478	BGIOSGA001492	XP_006649287.1 callose synthase 9
TCONS_00000747	-0.780706	-3.83312	-1.62276	BGIOSGA025705	XP_006644114.1 myb-related protein P-like
TCONS_00037465	-0.780604	0.291281	-0.296007	BGIOSGA013733	XP_006664169.1 protein WRKY1
TCONS_00028192	-0.780496	-0.876026	-0.954246	BGIOSGA003217	NP_001150903.2surfeit locus protein 5
TCONS_00036732	-0.780154	0.412064	-0.446515	BGIOSGA036583	XP_006664358.1PREDICTED: uncharacterized protein LOC102707638
TCONS_00013327	-0.780128	-1.81381	-0.977193	BGIOSGA027730	XP_006651762.1PREDICTED: uncharacterized protein LOC102720419 isoform X1
TCONS_00003978	-0.779284	0.785923	0.000980655	BGIOSGA001936	XP_015688143.1 UBP1-associated protein 2C-like
TCONS_00011814	-0.779182	-0.442559	-1.64313	BGIOSGA009926	XP_006649656.1 glutamine synthetase cytosolic isozyme 1-2
TCONS_00005891	-0.778879	0.893516	0.544923	BGIOSGA017759	XP_004951287.1zinc finger CCCH domain-containing protein 15
TCONS_00034528	-0.778855	-0.305294	-0.661589	BGIOSGA034291	XP_015697831.1 probable ubiquitin-like-specific protease 2B
TCONS_00008614	-0.778824	-0.380193	-0.736233	BGIOSGA018822	XP_006647708.1 protein PHOTOPERIOD-INDEPENDENT EARLY FLOWERING 1
TCONS_00020873	-0.778805	-0.0558383	-0.977989	BGIOSGA001201	XP_015694346.1 amino acid permease 3-like isoform X1
TCONS_00005038	-0.778508	-0.32359	-1.0759	BGIOSGA019178	XP_006646651.1 glutathione S-transferase 3
TCONS_00032130	-0.778448	-0.208027	0.538523	BGIOSGA032006	XP_006661734.1 pumilio homolog 23
TCONS_00029319	-0.778204	-3.76555	-1.36365	BGIOSGA027394	XP_015696416.1 double-stranded RNA-binding protein 5-like
TCONS_00010975	-0.778047	-1.01871	-0.842023	BGIOSGA035305	XP_002466438.1dentin sialophosphoprotein
TCONS_00003020	-0.777646	-0.478125	-0.184951	BGIOSGA018330	XP_015697354.1 putative pentatricopeptide repeat-containing protein At1g17630
TCONS_00013536	-0.777609	-1.44314	-1.12218	BGIOSGA008080	XP_006650891.1 B-cell receptor-associated protein 31-like
TCONS_00011388	-0.777323	-1.44486	-1.01405	BGIOSGA027358	XP_003564169.1F-box protein At4g00755
TCONS_00018495	-0.777188	-1.28321	-1.95332	BGIOSGA036338	XP_006653973.1 soluble inorganic pyrophosphatase 4-like
TCONS_00037618	-0.776426	-0.888072	-1.44659	BGIOSGA014093	XP_006664797.1 sucrose transport protein SUT2
TCONS_00004387	-0.775897	0.110671	-0.306709	BGIOSGA018746	XP_006644701.1 VQ motif-containing protein 4-like
TCONS_00011873	-0.775735	-1.4077	-1.74879	BGIOSGA029386	XP_006649715.1 probable protein kinase At2g41970
TCONS_00013371	-0.77565	0.535022	0.0562995	BGIOSGA027932	XP_004981378.1hydroxyproline O-galactosyltransferase GALT6
TCONS_00033734	-0.775274	-0.337318	0.13187	BGIOSGA035486	XP_002448574.2putative F-box protein At2g33200
TCONS_00030769	-0.77516	-0.899781	-0.552071	BGIOSGA003728	XP_006661651.1 disease resistance protein RPM1-like
TCONS_00028389	-0.775157	-1.242	-0.484867	BGIOSGA026574	XP_006659639.2 ubiquitin thioesterase otubain-like
TCONS_00031231	-0.775035	-0.0188877	-0.36717	BGIOSGA033049	XP_004986535.1AT-rich interactive domain-containing protein 1
TCONS_00002574	-0.774958	1.09049	0.655968	BGIOSGA005217	XP_006646704.2 serine/arginine-rich splicing factor SR45-like
TCONS_00000041	-0.774652	-0.769138	-1.0819	BGIOSGA002610	XP_006648166.1PREDICTED: uncharacterized protein LOC102709903
TCONS_00005975	-0.773978	-1.23707	-0.049668	BGIOSGA007479	XP_004952341.1uncharacterized protein LOC101783666 isoform X3
TCONS_00012530	-0.773643	0.856962	0.379071	BGIOSGA026044	XP_006651529.2 mitotic checkpoint protein BUB3.1-like
TCONS_00004769	-0.773488	-0.643549	-1.1317	BGIOSGA000446	XP_006645062.1 protein ABHD11
TCONS_00007109	-0.773428	-0.877491	-0.735442	BGIOSGA009136	XP_006648114.1 myosin-8-like

TCONS_00007166	-0.773369	-0.50578	-0.445503	BGIOSGA022076	XP_006648179.1 callose synthase 3-like
TCONS_00022768	-0.773072	-0.922624	-1.88956	BGIOSGA025476	XP_006656371.1 metal transporter Nramp3
TCONS_00025103	-0.772883	-1.01302	-0.610435	BGIOSGA024298	XP_006658564.1 protein RFT1 homolog
TCONS_00007215	-0.77247	-0.828298	-1.39151	BGIOSGA032300	XP_015688800.1 diphosphomevalonate decarboxylase MVD1-like
TCONS_00017050	-0.772093	-0.577769	-0.598366	BGIOSGA013090	XP_015693017.1 tetraspanin-6 isoform X4
TCONS_00004942	-0.772086	-0.370553	0.0870702	BGIOSGA002198	XP_015690720.1 DEAD-box ATP-dependent RNA helicase 30
TCONS_00010871	-0.772045	-1.27822	-1.07603	BGIOSGA025398	XP_021321458.1tubulin alpha-1 chain
TCONS_00019344	-0.77197	-0.762642	0.270058	BGIOSGA019650	XP_006654435.1 patellin-3-like
TCONS_00032567	-0.771923	-1.47131	-2.59482	BGIOSGA003944	XP_004982603.1methionine gamma-lyase
TCONS_00023091	-0.77154	-1.4254	-0.919375	BGIOSGA036206	XP_006657433.2 probable UDP-arabinose 4-epimerase 1
TCONS_00018472	-0.771207	-2.21676	-1.43771	BGIOSGA018963	XP_014755776.1putative F-box/LRR-repeat protein At5g02700
TCONS_00018041	-0.771123	0.781508	0.102002	BGIOSGA001903	XP_006654572.1 60S ribosomal protein L30-like
TCONS_00006804	-0.77061	-1.04661	-0.623294	BGIOSGA005122	XP_015689328.1 potassium transporter 25 isoform X1
TCONS_00023862	-0.770575	-1.29155	-1.23283	BGIOSGA000803	XP_006657803.1 beta-amylase
TCONS_00004662	-0.770268	-0.809677	-0.916701	BGIOSGA003285	XP_003564530.12-oxoglutarate-dependent dioxygenase DAO
TCONS_00004828	-0.770149	-1.95505	-1.14077	BGIOSGA005479	XP_006645121.1 probable pectinesterase/pectinesterase inhibitor 51
TCONS_00019397	-0.769804	-0.732566	0.165193	BGIOSGA003012	XP_006655367.2PREDICTED: uncharacterized protein LOC102707966
TCONS_00019890	-0.769606	-1.20483	0.0220302	BGIOSGA030241	XP_004977990.1UPF0481 protein At3g47200
TCONS_00037436	-0.768921	0.546378	1.21387	BGIOSGA022315	XP_006664698.2PREDICTED: uncharacterized protein LOC102701782, partial
TCONS_00003374	-0.768901	-4.22741	-2.04285	BGIOSGA012710	XP_006644090.1 peroxidase 2-like
TCONS_00025807	-0.768763	-1.39837	-0.778668	BGIOSGA018725	XP_006659056.1 fructokinase-2
TCONS_00010015	-0.7683	1.217	-0.115577	BGIOSGA004699	XP_024313564.1protein ENHANCED DISEASE RESISTANCE 4
TCONS_00014514	-0.76802	0.151312	-0.693543	BGIOSGA024790	XP_020393134.1inverted formin-2-like isoform X1
TCONS_00012315	-0.768015	-1.20683	-0.4113	BGIOSGA026245	XP_015691174.1 stomatal closure-related actin-binding protein 1-like
TCONS_00032758	-0.76789	-2.28599	-3.95849	BGIOSGA011300	XP_006662096.1 mitochondrial carnitine/acylcarnitine carrier-like protein
TCONS_00007973	-0.767862	-0.408193	-0.245201	#N/A	#N/A
TCONS_00032430	-0.767723	-0.0518938	-1.04759	BGIOSGA031687	XP_006662443.2PREDICTED: uncharacterized protein LOC102717187
TCONS_00020160	-0.767472	-0.68758	-0.436994	BGIOSGA022357	XP_004964789.1uncharacterized protein LOC101762969
TCONS_00029236	-0.767463	0.678773	-0.743555	BGIOSGA014063	XP_004957138.1nuclear transcription factor Y subunit C-6
TCONS_00036548	-0.767333	-1.886	1.68432	BGIOSGA019635	XP_006662667.1 CBL-interacting protein kinase 15-like
TCONS_00025767	-0.767014	-0.661556	-0.529587	BGIOSGA027828	XP_006659012.1 branchpoint-bridging protein isoform X2
TCONS_00001801	-0.766221	1.66193	-0.600147	BGIOSGA037768	XP_022682700.1receptor-like protein kinase 5
TCONS_00035032	-0.76579	-0.356903	0.255705	BGIOSGA035354	XP_006663519.1 phosphatidate cytidylyltransferase, mitochondrial
TCONS_00034210	-0.765662	0.482189	0.0832744	BGIOSGA036719	XP_003577865.140S ribosomal protein S16
TCONS_00007982	-0.765069	-0.220309	0.423836	BGIOSGA033958	XP_006647304.1 rRNA-processing protein UTP23 homolog
TCONS_00005181	-0.764439	1.59698	0.41876	BGIOSGA000023	XP_003565161.1transcription factor PCL1
TCONS_00019765	-0.763942	0.00600175	-1.28151	BGIOSGA008199	XP_006655579.2 MACPF domain-containing protein NSL1-like
TCONS_00028144	-0.763912	-2.85385	-2.45705	BGIOSGA005531	XP_004973655.1nitrate reductase [NADH]
TCONS_00019737	-0.763446	-1.85337	-1.22116	BGIOSGA017623	XP_004961329.1serine/threonine-protein kinase STN7, chloroplastic
TCONS_00006971	-0.7631	-0.552059	0.220638	BGIOSGA005481	XP_006648013.1 chaperone protein DnaJ-like
TCONS_00013330	-0.763015	-0.40931	-0.257854	BGIOSGA009654	XP_006651903.1 folate-biopterin transporter 1, chloroplastic-like
TCONS_00019110	-0.762752	-0.318545	0.12421	BGIOSGA018251	XP_015692556.1 cytosolic endo-beta-N-acetylglucosaminidase 1-like
TCONS_00017535	-0.762617	0.161715	-0.296287	BGIOSGA024011	XP_021312343.1ethylene-responsive transcription factor CRF3
TCONS_00023712	-0.762461	-0.499804	-0.408604	BGIOSGA009277	XP_015695104.1PREDICTED: uncharacterized protein LOC102716704
TCONS_00019487	-0.762428	0.458072	-0.125123	BGIOSGA004709	XP_002441262.140S ribosomal protein S26
TCONS_00009105	-0.762393	-1.69787	-0.42387	BGIOSGA026323	XP_006648181.1 alpha-1,3-mannosyl-glycoprotein 2-beta-N-acetylglucosaminyltransferase
TCONS_00013793	-0.762245	-0.0812255	-0.777972	BGIOSGA015918	XP_015691378.1 vacuolar protein sorting-associated protein 54, chloroplastic
TCONS_00028521	-0.762191	-1.0204	-0.476383	BGIOSGA019452	XP_015690470.1 aldose 1-epimerase-like
TCONS_00001637	-0.761961	-0.884346	-1.088	BGIOSGA004271	XP_006644570.1PREDICTED: uncharacterized protein LOC102722438
TCONS_00013340	-0.761878	-0.423014	-0.0601649	BGIOSGA025258	XP_006650729.1 60S ribosomal protein L4-like
TCONS_00006349	-0.761353	-1.11742	-0.0864415	BGIOSGA008511	XP_015689425.1 protein FLUORESCENT IN BLUE LIGHT, chloroplastic-like isoform X1
TCONS_00025684	-0.761249	0.440031	0.18956	BGIOSGA023713	XP_006658092.1 potassium transporter 7
TCONS_00036356	-0.760967	0.139529	-1.4794	BGIOSGA014279	XP_004963056.1wall-associated receptor kinase-like 21 isoform X2
TCONS_00031604	-0.760667	0.124464	0.143865	BGIOSGA033419	XP_008786987.1 NADH dehydrogenase [ubiquinone] 1 beta subcomplex subunit 8, mitochondrial-like
TCONS_00036522	-0.760664	-0.460996	-1.01785	BGIOSGA017337	XP_006664264.1 protein transport protein Sec61 subunit beta
TCONS_00004868	-0.760596	-1.53336	-1.99817	BGIOSGA033579	XP_015691692.1 G-type lectin S-receptor-like serine/threonine-protein kinase At2g19130
TCONS_00000890	-0.760495	0.44634	-0.414013	BGIOSGA018783	XP_003568901.1E3 ubiquitin-protein ligase RFI2

TCONS_00021026	-0.760476	-0.650545	-0.693633	BGIOSGA019860	XP_015693723.1 65-kDa microtubule-associated protein 1
TCONS_00019462	-0.760382	-1.17342	-0.233342	BGIOSGA014554	XP_003568266.1calcium-dependent protein kinase 16
TCONS_00012983	-0.760287	1.65437	0.70608	BGIOSGA018559	XP_006651716.1PREDICTED: uncharacterized protein LOC102703671
TCONS_00019402	-0.760218	0.291272	-0.1415	BGIOSGA017591	XP_006655580.1 mediator of RNA polymerase II transcription subunit 13
TCONS_00024657	-0.760173	-1.25641	-0.117942	BGIOSGA021729	XP_004955608.2leucine-rich repeat extensin-like protein 4
TCONS_00015232	-0.76006	-3.03655	-1.46565	BGIOSGA006176	XP_015691632.1 proteinaceous RNase P 1, chloroplastic/mitochondrial-like
TCONS_00006272	-0.760052	-2.05228	-0.820407	BGIOSGA008430	XP_006647378.2 zinc finger CCCH domain-containing protein 16
TCONS_00036362	-0.759437	1.48761	0.347142	BGIOSGA013169	XP_006664168.1 flowering locus K homology domain
TCONS_00031541	-0.759121	1.28858	-0.806784	BGIOSGA013995	XP_006662003.1 protein DOWNY MILDEW RESISTANCE 6-like
TCONS_00004176	-0.758936	-0.528735	-0.483879	BGIOSGA020340	XP_004975378.160S ribosomal protein L37a-1
TCONS_00012490	-0.758929	-1.38568	0.660161	BGIOSGA012094	XP_021307472.1RING finger protein 222
TCONS_00037323	-0.758907	0.446182	-0.21162	BGIOSGA001685	XP_006664621.2 auxilin-related protein 2-like
TCONS_00013638	-0.758726	-0.123761	-0.403421	BGIOSGA036535	XP_006652078.1 amidase 1-like
TCONS_00029080	-0.758496	-0.462834	0.270369	BGIOSGA010761	XP_004956902.2dormancy-associated protein homolog 3 isoform X2
TCONS_00011087	-0.758486	0.0891099	0.105547	BGIOSGA032755	XP_006650697.1 probable tRNA (guanine(26)-N(2))-dimethyltransferase 1
TCONS_00019530	-0.758158	-1.64946	-2.007	BGIOSGA037853	XP_006664231.1PREDICTED: uncharacterized protein LOC102709977
TCONS_00036843	-0.758096	0.81274	-0.576008	BGIOSGA037137	XP_015698758.1 putative disease resistance protein RGA1
TCONS_00011880	-0.757402	-1.16678	-0.192297	BGIOSGA011131	XP_015691289.1 integrator complex subunit 9 isoform X2
TCONS_00035212	-0.757291	-1.01573	0.320626	BGIOSGA026134	XP_015698020.1 oligouridylate-binding protein 1B-like
TCONS_00030164	-0.757237	-1.88085	-1.47791	BGIOSGA034236	XP_006660692.1 tuliposide A-converting enzyme b1, amyloplastic-like
TCONS_00026080	-0.757193	-1.68703	-1.12266	BGIOSGA004315	XP_017696093.1 peroxisomal (S)-2-hydroxy-acid oxidase-like
TCONS_00029177	-0.757139	-0.881569	-0.575927	BGIOSGA002398	XP_006660712.2PREDICTED: uncharacterized protein LOC102717367 isoform X1
TCONS_00013427	-0.756485	-2.7298	-0.649704	BGIOSGA022587	XP_002457242.1aspartic proteinase CDR1
TCONS_00012379	-0.756162	0.233269	0.303691	BGIOSGA017894	XP_006650152.1 metacaspase-1-like
TCONS_00003249	-0.755399	-2.08893	0.514665	#N/A	#N/A
TCONS_00030515	-0.755226	0.027027	-0.733027	BGIOSGA005528	XP_006660959.1 protein indeterminate-domain 5, chloroplastic-like
TCONS_00016617	-0.755035	-0.375849	0.35094	BGIOSGA025878	XP_006653769.1 probable LRR receptor-like serine/threonine-protein kinase At1g56140
TCONS_00010307	-0.754827	-5.05381	-2.19438	BGIOSGA009790	XP_006650197.1 COBRA-like protein 5
TCONS_00010246	-0.75436	1.58946	1.53486	BGIOSGA017261	XP_015690176.1 vacuolar cation/proton exchanger 2 isoform X2
TCONS_00015057	-0.75397	-2.24753	-1.84857	BGIOSGA027224	XP_015692300.1 synaptotagmin-5-like
TCONS_00016177	-0.753602	-2.47039	-4.65684	BGIOSGA007128	XP_006652472.1 endoglucanase 12
TCONS_00002652	-0.753011	-1.65672	-1.20414	BGIOSGA005292	XP_015693860.1 mitochondrial uncoupling protein 1-like
TCONS_00017008	-0.752979	-1.8977	-0.500675	BGIOSGA010658	XP_009421350.1 myb-related protein MYBAS1 isoform X2
TCONS_00008104	-0.752776	-1.47137	-0.688988	BGIOSGA004697	XP_006648685.1 serine/threonine-protein kinase CTR1-like
TCONS_00005323	-0.752732	-0.909001	-1.21254	BGIOSGA007432	XP_002451453.1light-harvesting complex-like protein 3 isotype 1, chloroplastic
TCONS_00036777	-0.752661	0.199103	0.642983	BGIOSGA036828	XP_021317073.1uncharacterized protein LOC8079950 isoform X3
TCONS_00018414	-0.752623	-0.799517	-1.47995	BGIOSGA017626	XP_003566864.1probable inactive receptor kinase At2g26730
TCONS_00031330	-0.75224	-1.05947	-0.92932	BGIOSGA035845	XP_006661867.1 homeobox-leucine zipper protein HOX9
TCONS_00010181	-0.752201	-1.51371	-0.474592	BGIOSGA012742	XP_003557837.1protein DGS1, mitochondrial
TCONS_00027699	-0.752005	-0.53472	-0.870707	BGIOSGA027306	XP_008678441.1uncharacterized protein LOC100277149
TCONS_00023978	-0.75192	-2.47168	-1.52878	BGIOSGA008619	XP_015691515.1 AP2-like ethylene-responsive transcription factor BBM1
TCONS_00015918	-0.751694	-0.411419	-0.204257	BGIOSGA015068	XP_006653419.1 threonylcarbamoyladenosine tRNA methylthiotransferase
TCONS_00001351	-0.751025	-0.699581	-0.97029	BGIOSGA021863	XP_006646093.2 GDSL esterase/lipase At1g28570
TCONS_00012270	-0.750966	0.358232	0.0672917	BGIOSGA035194	XP_004984389.1probable serine/threonine-protein kinase At1g54610
TCONS_00001573	-0.750955	-1.1182	-0.993459	BGIOSGA024746	XP_015688083.1PREDICTED: uncharacterized protein LOC102707720 isoform X2
TCONS_00033112	-0.750461	0.0670484	0.0525607	BGIOSGA034957	XP_006662793.1 probable anion transporter 6 isoform X1
TCONS_00010124	-0.750343	0.700525	0.408579	BGIOSGA037764	XP_006651408.2 serine/threonine-protein kinase At5g01020-like
TCONS_00004184	-0.750247	1.52782	-0.584303	BGIOSGA019301	XP_012701665.1uncharacterized protein LOC105914466
TCONS_00025485	-0.750193	-0.46233	-2.01225	BGIOSGA020770	XP_006657922.1 vestitone reductase-like
TCONS_00001719	-0.74978	-2.23337	-0.404802	BGIOSGA017672	XP_014751474.1disease resistance protein RGA2
TCONS_00021720	-0.749504	-2.30966	0.0601785	BGIOSGA023962	XP_004954253.1heparanase-like protein 3
TCONS_00034095	-0.749481	-0.233744	-0.834386	BGIOSGA000816	XP_006663130.1 probable GTP-binding protein OBGM, mitochondrial
TCONS_00022514	-0.749465	-0.410641	-0.011301	BGIOSGA020850	XP_015694107.1 pentatricopeptide repeat-containing protein At1g31430-like
TCONS_00031631	-0.748997	-1.12744	-1.53602	BGIOSGA011425	XP_004983636.1serine/threonine-protein kinase D6PK

TCONS_00008734	-0.748671	-1.55863	-0.755562	BGIOSGA037735	XP_006647826.1 dof zinc finger protein DOF5.4-like
TCONS_00009035	-0.748652	-0.989426	-1.39145	BGIOSGA029934	XP_006648101.1 nicotinamide/nicotinic acid mononucleotide adenylyltransferase
TCONS_00016901	-0.748205	-1.71613	-0.569157	BGIOSGA009861	XP_006653051.1 AT-hook motif nuclear-localized protein 9-like
TCONS_00023861	-0.747813	-2.96109	-4.73919	BGIOSGA011829	XP_003562968.2beta-amylase isoform X1
TCONS_00011605	-0.747774	-2.12897	-2.14671	BGIOSGA011410	XP_015691136.1 probable RNA helicase SDE3
TCONS_00001346	-0.747774	0.506191	0.0744347	BGIOSGA021951	XP_006644360.2 cytochrome c oxidase subunit 5b-1, mitochondrial-like
TCONS_00017550	-0.746485	-1.71861	-1.87198	BGIOSGA014441	XP_002440874.1protein ACTIVITY OF BC1 COMPLEX KINASE 3, chloroplastic
TCONS_00029635	-0.74629	-0.239719	-0.265581	BGIOSGA030183	XP_006660445.1 histone-lysine N-methyltransferase ATX2-like isoform X1
TCONS_00002533	-0.746246	-0.704492	-1.38864	BGIOSGA005176	XP_015695557.1 uridine 5'-monophosphate synthase-like
TCONS_00022653	-0.745928	0.690893	-0.179358	BGIOSGA020789	XP_003560349.1transcription initiation factor TFIID subunit 1 isoform X2
TCONS_00019930	-0.745879	0.338893	-0.20466	BGIOSGA017430	XP_015693261.1 translation initiation factor IF-2-like isoform X2
TCONS_00002411	-0.745779	-1.07249	-0.591246	BGIOSGA005054	XP_006646610.1PREDICTED: uncharacterized protein LOC102708210
TCONS_00035427	-0.745779	-1.51501	-0.93917	BGIOSGA036834	XP_006663701.1 protein TONNEAU 1a-like
TCONS_00030674	-0.745343	0.368967	-0.151754	BGIOSGA020362	XP_006662164.1PREDICTED: uncharacterized protein LOC102721866
TCONS_00005139	-0.745026	-0.847159	-0.593111	BGIOSGA000033	XP_010231908.1uncharacterized protein LOC104582790
TCONS_00021250	-0.744996	0.134792	-0.822469	BGIOSGA031637	XP_009404996.1 polyubiquitin 11
TCONS_00006178	-0.744766	-0.130826	-0.577304	BGIOSGA015180	XP_012703589.1chaperone protein ClpD1, chloroplastic
TCONS_00005326	-0.744585	0.524342	0.0388925	BGIOSGA012688	XP_015688441.1 calcium-dependent protein kinase 16-like
TCONS_00030001	-0.744569	-0.935168	-0.989445	BGIOSGA036028	XP_015696663.1 protein trichome birefringence-like 14 isoform X1
TCONS_00030564	-0.744537	-0.37422	-0.592929	BGIOSGA034819	XP_006660997.1 40S ribosomal protein S25-2-like
TCONS_00012430	-0.744483	-0.688512	-0.17125	BGIOSGA034685	XP_006650182.1PREDICTED: uncharacterized protein LOC102717967
TCONS_00022182	-0.744359	-1.0599	-0.288723	BGIOSGA002976	XP_006656051.1 RNA-binding protein EWS-like
TCONS_00000169	-0.743996	-3.80265	-0.595661	BGIOSGA034934	XP_002457394.1protein ASPARTIC PROTEASE IN GUARD CELL 1
TCONS_00018539	-0.743716	-0.231709	1.05814	BGIOSGA018898	XP_006653998.1 isovaleryl-CoA dehydrogenase, mitochondrial
TCONS_00033032	-0.743677	0.301734	-0.408478	BGIOSGA020348	XP_006662761.1 tubby-like F-box protein 13
TCONS_00014214	-0.743606	0.924779	-0.156051	BGIOSGA029619	XP_006652267.1 OTU domain-containing protein 5
TCONS_00031614	-0.743602	-2.70241	-1.53122	BGIOSGA027617	XP_006653885.1 RNA polymerase II transcription factor B subunit 2
TCONS_00014450	-0.743567	-0.949672	-0.155084	BGIOSGA005228	XP_004971348.1uncharacterized protein LOC101772585
TCONS_00007406	-0.74318	-0.802653	-0.580036	BGIOSGA007067	XP_006646924.1 RNA-binding protein 5 isoform X1
TCONS_00014779	-0.743153	-2.28784	-2.2693	BGIOSGA012822	XP_004976547.1uncharacterized protein LOC101779547
TCONS_00017275	-0.743153	0.467046	-0.394832	#N/A	#N/A
TCONS_00005364	-0.743052	1.00352	-3.14231	BGIOSGA007475	XP_015689397.1 L-aspartate oxidase, chloroplastic
TCONS_00005811	-0.742848	0.750497	0.764078	BGIOSGA007933	XP_015688887.1PREDICTED: uncharacterized protein LOC102705885
TCONS_00011385	-0.74265	-1.88974	-1.3161	BGIOSGA024383	XP_003557289.1COP1-interacting protein 7
TCONS_00027252	-0.74259	0.579327	0.2149	BGIOSGA025984	XP_015688778.1 40S ribosomal protein S13-2
TCONS_00032961	-0.742251	-0.862441	-0.241376	BGIOSGA034794	XP_006664335.1 peptidyl-prolyl cis-trans isomerase FKBP42
TCONS_00010413	-0.742051	-0.753863	-0.485474	BGIOSGA005787	XP_004972759.1probable polyamine transporter At1g31830
TCONS_00016839	-0.740865	-0.374529	0.36817	BGIOSGA028530	XP_006653002.1 probable arabinosyltransferase ARAD1 isoform X1
TCONS_00005862	-0.740554	-0.00201341	-0.28533	BGIOSGA021608	XP_015691777.1 putative pentatricopeptide repeat-containing protein At5g08490
TCONS_00014437	-0.740305	-0.758045	-1.19035	BGIOSGA002732	XP_015691330.1 G-type lectin S-receptor-like serine/threonine-protein kinase RLK1
TCONS_00017122	-0.740262	0.437087	0.732331	BGIOSGA019171	XP_006654988.1 uncharacterized CRM domain-containing protein At3g25440, chloroplastic
TCONS_00016105	-0.739743	-0.295357	0.0661068	BGIOSGA006128	XP_015692141.1 isocitrate dehydrogenase [NAD] regulatory subunit 1, mitochondrial-like
TCONS_00029905	-0.739478	-0.498776	0.371734	BGIOSGA029901	XP_015696532.1 THO complex subunit 6 isoform X2
TCONS_00035457	-0.739234	-0.822699	-1.25336	BGIOSGA028217	XP_019078711.1 ethanolamine-phosphate cytidylyltransferase isoform X2
TCONS_00037360	-0.739212	-0.955176	-0.793985	BGIOSGA001316	XP_006664100.1 RNA pseudouridine synthase 1
TCONS_00018777	-0.739048	-1.01197	0.790569	BGIOSGA007818	XP_006654138.1 pollen-specific protein SF21-like
TCONS_00002204	-0.738903	-0.325017	-0.739937	BGIOSGA029185	XP_015689347.1 60S ribosomal protein L10a-like
TCONS_00017750	-0.738751	1.31334	-0.0373499	BGIOSGA019812	XP_024314920.1leucine-rich repeat receptor-like serine/threonine-protein kinase At2g14510 isoform X2
TCONS_00010962	-0.738732	-1.68792	0.590051	BGIOSGA013562	XP_015690733.1 beta-glucosidase BoGH3B-like
TCONS_00021662	-0.738583	-0.707736	-0.280774	#N/A	#N/A
TCONS_00036312	-0.738473	-0.0715848	-0.301056	BGIOSGA004012	XP_006664145.1 E3 ubiquitin-protein ligase SDIR1-like
TCONS_00000601	-0.738362	-1.01283	-0.448171	BGIOSGA009933	XP_015688191.1 mediator-associated protein 2
TCONS_00018656	-0.738328	-2.80732	-0.651898	BGIOSGA017544	XP_015692844.1 GATA transcription factor 23-like

TCONS_00003778	-0.737763	-0.703244	-1.08709	BGIOSGA030676	XP_015688188.1 8-amino-7-oxononanoate synthase-like isoform X1
TCONS_00010333	-0.737763	-0.582152	-0.420509	BGIOSGA038782	XP_006650204.2 mediator of RNA polymerase II transcription subunit 8
TCONS_00009960	-0.737337	-0.206494	0.577392	BGIOSGA023689	XP_003558036.1SNF1-related protein kinase regulatory subunit beta-1
TCONS_00023753	-0.73716	-0.381663	-0.375708	BGIOSGA010995	XP_006657753.2 bromodomain-containing protein DDB_G0270170
TCONS_00001081	-0.737119	-0.820316	0.114738	BGIOSGA013718	XP_004968887.1uncharacterized protein LOC101756105 isoform X2
TCONS_00007636	-0.736986	-0.525544	-1.07205	BGIOSGA006845	XP_006647086.1 early nodulin-93-like
TCONS_00017007	-0.736849	-0.28812	-0.64343	BGIOSGA019053	XP_006654918.1PREDICTED: uncharacterized protein LOC102713910
TCONS_00027920	-0.736485	-2.65904	-0.800734	BGIOSGA027072	XP_015695839.1 protein tesmin/TSO1-like CXC 7
TCONS_00014789	-0.736426	-0.0943348	0.177093	BGIOSGA016962	XP_015692140.1 ankyrin repeat and zinc finger domain-containing protein 1
TCONS_00021320	-0.736321	0.00921013	-2.08256	BGIOSGA039369	XP_006657300.2 probable aminodeoxychorismate synthase, chloroplastic
TCONS_00033705	-0.736309	-0.904221	-0.0210107	BGIOSGA035235	XP_015697699.1 zinc finger CCHC domain-containing protein 7-like
TCONS_00036728	-0.736122	-1.62975	-1.26106	BGIOSGA022575	XP_022684940.1BEL1-like homeodomain protein 1
TCONS_00021842	-0.736076	-0.437167	-0.988158	BGIOSGA033403	XP_015694383.1PREDICTED: uncharacterized protein LOC102718283
TCONS_00003046	-0.735171	-0.235671	-0.218368	BGIOSGA023073	XP_003565213.2protein BZR1 homolog 2
TCONS_00023342	-0.73482	-1.9534	-1.02469	BGIOSGA025384	XP_022682008.1exocyst complex component EXO70B1-like
TCONS_00013016	-0.734678	-1.50535	-0.670402	BGIOSGA037334	XP_015691246.1 pinin
TCONS_00031817	-0.734637	-2.57833	-2.59187	BGIOSGA035157	XP_006660837.1 solute carrier family 35 member F1-like
TCONS_00031611	-0.734559	-0.0418579	-0.399566	BGIOSGA014584	XP_006662042.1 putative glucuronosyltransferase PGSIP7 isoform X1
TCONS_00003642	-0.734548	-1.75244	0.244477	BGIOSGA025677	XP_006645926.1 metal transporter Nramp4 isoform X1
TCONS_00016761	-0.734437	-0.799441	-0.177475	BGIOSGA014203	XP_015691600.1 golgin candidate 2
TCONS_00015198	-0.733821	0.493802	-0.875322	BGIOSGA013850	XP_015692209.1 probable serine/threonine-protein kinase At5g41260
TCONS_00001141	-0.73371	-1.66813	-1.42841	BGIOSGA005949	XP_015698906.1 vacuolar-processing enzyme
TCONS_00038791	-0.733402	-1.5518	-1.41596	BGIOSGA034298	XP_024310899.1probable LRR receptor-like serine/threonine-protein kinase At4g29180 isoform X2
TCONS_00004122	-0.733394	-0.29592	0.21785	BGIOSGA020374	XP_006646178.2PREDICTED: putative uncharacterized protein DDB_G0277255 isoform X1
TCONS_00036388	-0.733135	-1.49305	-1.01569	BGIOSGA006359	XP_006664166.1 gamma-tubulin complex component 5-like isoform X1
TCONS_00007285	-0.733105	-3.45107	-1.43675	BGIOSGA007199	XP_015688989.1 protein LOW PSII ACCUMULATION 3, chloroplastic
TCONS_00029767	-0.732977	-2.10857	-0.130728	BGIOSGA023596	XP_021318845.1probable serine/threonine-protein kinase PIX13
TCONS_00002381	-0.73278	#NA	-1.35585	BGIOSGA005015	XP_006645197.1 proline transporter 1-like
TCONS_00012297	-0.732552	-0.638443	-0.123102	BGIOSGA020904	XP_006650100.1PREDICTED: uncharacterized protein LOC102716943 isoform X1
TCONS_00029216	-0.73244	-0.966803	-0.776958	BGIOSGA035063	XP_015696741.1 transcription factor BIM2
TCONS_00019949	-0.732011	-0.472282	-0.942637	BGIOSGA022133	XP_015694095.1 membrane protein of ER body-like protein isoform X2
TCONS_00008640	-0.731384	-0.686997	-2.50642	BGIOSGA009203	XP_006647744.1 protein Brevis radix-like 2
TCONS_00006835	-0.731227	0.893116	-0.430371	BGIOSGA009009	XP_015689549.1 ATP-dependent RNA helicase DEAH13
TCONS_00022813	-0.731167	-1.00342	-1.12104	BGIOSGA020637	XP_006657282.1 DNA repair protein REV1 isoform X1
TCONS_00018002	-0.731031	-3.30107	-3.14793	BGIOSGA035187	XP_014660333.1non-specific lipid-transfer protein 1-like
TCONS_00000420	-0.730756	-5.25016	-2.00779	BGIOSGA018768	XP_015691735.1 GDSL esterase/lipase At5g45910-like
TCONS_00027803	-0.730356	0.633635	0.660956	BGIOSGA029619	XP_012698550.14-hydroxybenzoate polyprenyltransferase, mitochondrial
TCONS_00011493	-0.729664	-0.689164	-1.51068	BGIOSGA011533	XP_021310221.1DNA polymerase delta small subunit isoform X2
TCONS_00036532	-0.729105	-0.859634	-1.40027	BGIOSGA002158	XP_006662650.1 probable LRR receptor-like serine/threonine-protein kinase MRH1
TCONS_00003027	-0.72885	-1.2505	-1.68127	BGIOSGA009908	XP_015688305.1 monoglyceride lipase
TCONS_00005143	-0.72883	-0.0812795	-1.66809	BGIOSGA016432	XP_006645354.1 40S ribosomal protein S10-like
TCONS_00024437	-0.728578	0.943951	-0.190685	BGIOSGA022229	XP_003557694.2sugar transport protein MST3
TCONS_00006666	-0.728497	-0.250058	0.130286	BGIOSGA008835	XP_006647705.1 probable dolichyl pyrophosphate Glc1Man9GlcNAc2 alpha-1,3-glucosyltransferase
TCONS_00011022	-0.728443	0.209545	-0.000315788	BGIOSGA037704	XP_003558930.1uncharacterized protein LOC100837825
TCONS_00014396	-0.728431	-0.224629	-0.10459	BGIOSGA002981	XP_006652359.1 14-3-3-like protein GF14-B
TCONS_00019232	-0.728132	-0.850091	-1.09267	BGIOSGA001576	XP_004962186.1uncharacterized protein LOC101784684
TCONS_00007139	-0.728121	-0.512522	-1.08224	BGIOSGA004365	XP_002453074.1caffeic acid 3-O-methyltransferase
TCONS_00001202	-0.727004	0.172566	-1.01475	BGIOSGA032709	XP_006646030.1 beta-galactosidase 2
TCONS_00026553	-0.726918	-1.5194	-0.050659	BGIOSGA001311	XP_006660106.1PREDICTED: uncharacterized protein LOC102709021
TCONS_00029546	-0.726753	1.76542	0.0737805	BGIOSGA034249	XP_015696477.1 U-box domain-containing protein 33-like isoform X1
TCONS_00011162	-0.726633	0.20535	-2.03735	BGIOSGA025344	XP_006651930.1 squalene synthase-like
TCONS_00003347	-0.72653	-2.2238	-0.483525	BGIOSGA022512	XP_015688295.1 transcription factor APG-like
TCONS_00021604	-0.726358	-1.31946	0.281423	#N/A	#N/A
TCONS_00003266	-0.725698	-1.64392	-0.579734	BGIOSGA001949	XP_020405021.1uncharacterized protein LOC100191545 isoform X2

TCONS_00013603	-0.725447	-4.08553	-0.657589	BGIOSGA001229	XP_006652072.1PREDICTED: uncharacterized protein LOC102710580
TCONS_00024073	-0.725298	-0.179342	0.0588517	BGIOSGA026107	XP_003562710.1protein bem46
TCONS_00031375	-0.725253	-0.678903	-0.25803	BGIOSGA025437	XP_015697036.1 alpha-galactosidase 1-like
TCONS_00013459	-0.725038	0.269118	0.184558	BGIOSGA027861	XP_015690999.1 protein-lysine methyltransferase METTL21B
TCONS_00008163	-0.724854	0.689215	0.162212	BGIOSGA006278	XP_006647360.1 exosome complex component RRP45A-like isoform X2
TCONS_00014312	-0.724727	-1.15901	-1.03375	BGIOSGA012442	XP_004975725.1calmodulin-binding protein 60 D isoform X2
TCONS_00004079	-0.724721	-2.82402	-1.20069	BGIOSGA021585	XP_006644448.1 protein trichome birefringence-like 19
TCONS_00024403	-0.724622	-1.29615	-1.34651	BGIOSGA036804	XP_015695174.1 ubiquitin-activating enzyme E1 3-like isoform X2
TCONS_00014973	-0.723895	0.550938	-0.692248	BGIOSGA017154	XP_003579311.1putative disease resistance protein At3g14460
TCONS_00030105	-0.723685	0.859874	-0.903446	BGIOSGA028554	XP_006660655.1 E3 ubiquitin-protein ligase RNF14-like
TCONS_00004805	-0.723609	-0.268431	-1.40099	BGIOSGA034888	XP_006645091.1 amidophosphoribosyltransferase, chloroplastic-like
TCONS_00021448	-0.723489	-1.13441	-0.296844	BGIOSGA022101	XP_006656545.1 dihydrolipoyllysine-residue acetyltransferase component 2 of pyruvate dehydrogenase complex, mitochondrial-like
TCONS_00025095	-0.723355	-1.33084	-1.11154	BGIOSGA005888	XP_004957703.1FT-interacting protein 1
TCONS_00013258	-0.723075	0.364509	-0.89326	BGIOSGA015415	XP_015690092.1 proline-rich receptor-like protein kinase PERK4
TCONS_00035600	-0.722974	-0.851521	-0.583299	BGIOSGA000169	XP_012703259.1uncharacterized protein LOC101782771
TCONS_00036250	-0.722775	0.533932	0.556023	BGIOSGA037609	XP_006664111.1 protein MODIFIER OF SNC1 1
TCONS_00005114	-0.722247	-0.379452	-0.706306	BGIOSGA030998	XP_015691020.1 protein LATERAL ROOT PRIMORDIUM 1
TCONS_00000909	-0.7222	-0.464141	-0.719338	BGIOSGA003498	XP_006647964.1 ferredoxin--nitrite reductase, chloroplastic
TCONS_00035636	-0.721994	0.437435	0.220028	BGIOSGA034880	XP_006662762.2 60S ribosomal protein L3-like
TCONS_00036867	-0.721674	-1.05526	-1.10867	BGIOSGA002841	XP_006663910.1 receptor-like protein 12
TCONS_00025081	-0.721329	-0.693309	0.832198	BGIOSGA014047	XP_003563136.1serine/threonine-protein kinase STY17
TCONS_00034839	-0.72131	-2.29341	-0.337974	BGIOSGA037011	XP_006663435.1 cysteine-rich receptor-like protein kinase 42
TCONS_00008655	-0.721272	-2.71575	-1.23023	BGIOSGA005773	XP_006647761.1PREDICTED: uncharacterized protein LOC102710665
TCONS_00003668	-0.721152	0.210306	-0.00288269	BGIOSGA001539	XP_006645938.1 nicotinamide adenine dinucleotide transporter 2, mitochondrial-like
TCONS_00006156	-0.720789	-0.728751	-0.910675	BGIOSGA022663	XP_015697715.1PREDICTED: uncharacterized protein LOC102712867
TCONS_00013182	-0.720692	1.37874	-0.0948345	BGIOSGA009803	XP_006651826.1 transcriptional adapter ADA2
TCONS_00024119	-0.720591	-1.53392	-0.53814	BGIOSGA026154	XP_015695063.1 calcium-binding protein CML38-like
TCONS_00006940	-0.720535	1.41389	0.101518	BGIOSGA018799	XP_006647976.1 1-aminocyclopropane-1-carboxylate oxidase
TCONS_00020221	-0.720334	0.381086	0.279718	BGIOSGA021342	XP_006655830.1 proteasome subunit alpha type-4-2
TCONS_00012910	-0.720278	-4.31436	-0.742021	BGIOSGA027985	XP_006651678.1 homeobox protein knotted-1-like 4 isoform X2
TCONS_00029212	-0.72002	-0.144759	0.994304	BGIOSGA014151	XP_015696518.1 transcription factor bHLH49
TCONS_00028348	-0.719963	-0.403942	-0.498528	BGIOSGA018585	XP_015695674.1 RPM1-interacting protein 4
TCONS_00004412	-0.719941	0.482258	-0.829109	BGIOSGA020500	XP_014754463.1callose synthase 11
TCONS_00012188	-0.719841	0.578175	0.583394	BGIOSGA029518	XP_015689877.1 NAC domain-containing protein 100-like
TCONS_00023763	-0.719526	-0.797902	-0.381169	BGIOSGA006747	XP_022680353.1uncharacterized protein LOC101755892
TCONS_00023893	-0.718934	-0.792489	0.254646	BGIOSGA023659	XP_002462239.2uncharacterized protein LOC8055533
TCONS_00034124	-0.718727	-0.286564	-0.856898	BGIOSGA018227	XP_006662650.1 probable LRR receptor-like serine/threonine-protein kinase MRH1
TCONS_00026622	-0.718571	0.505387	-0.308763	BGIOSGA025290	XP_006659406.1 chromatin remodeling protein EBS-like isoform X2
TCONS_00002079	-0.718362	-0.168352	-0.751081	BGIOSGA003376	XP_002441262.140S ribosomal protein S26
TCONS_00003565	-0.718054	0.0267279	-1.42643	BGIOSGA001640	XP_001420315.1predicted protein
TCONS_00017506	-0.7176	-0.298517	-1.92935	BGIOSGA037588	XP_015692537.1 probable linoleate 9S-lipoxygenase 5
TCONS_00021264	-0.716967	-1.00511	-1.01892	BGIOSGA023490	XP_006656399.1 auxin response factor 18
TCONS_00020911	-0.716863	-0.870564	-0.524803	BGIOSGA009241	XP_003563721.1GATA transcription factor 21
TCONS_00022897	-0.716804	1.0092	-0.0792396	BGIOSGA020547	XP_006657335.2 protein FAM63B
TCONS_00035741	-0.716323	0.792203	0.0402422	BGIOSGA023433	XP_015698242.1 probable leucine-rich repeat receptor-like protein kinase At5g49770
TCONS_00018188	-0.715954	-0.350758	0.00262994	BGIOSGA020471	XP_015693113.1 plasminogen activator inhibitor 1 RNA-binding protein-like isoform X1
TCONS_00029098	-0.715141	-0.495745	-0.243314	BGIOSGA000481	XP_003578192.1O-fucosyltransferase 35
TCONS_00031791	-0.714791	-0.349001	-0.941268	BGIOSGA007774	XP_002450002.2disease resistance protein RPP13
TCONS_00036077	-0.714746	-0.197678	0.0376944	BGIOSGA037441	XP_015698406.1 glycine-rich RNA-binding protein blt801
TCONS_00026313	-0.71453	-0.481484	0.0161494	BGIOSGA011746	XP_010234555.2homeobox protein knotted-1-like 13 isoform X1
TCONS_00007737	-0.714375	-0.513869	1.0623	BGIOSGA037452	XP_006664050.1 cyclic pyranopterin monophosphate synthase, mitochondrial
TCONS_00028680	-0.714367	-1.60759	-0.874264	BGIOSGA030429	XP_015696744.1 SURP and G-patch domain-containing protein 1-like protein
TCONS_00007025	-0.714326	0.742422	0.334253	BGIOSGA021784	XP_017625336.1 60S ribosomal protein L39
TCONS_00009104	-0.71429	0.085113	0.394482	BGIOSGA005297	XP_006648178.1 outer envelope pore protein 21, chloroplastic-like
TCONS_00032353	-0.714229	-1.0672	1.76384	BGIOSGA010737	XP_006661828.1 E3 ubiquitin-protein ligase MIEL1-like

TCONS_00000084	-0.7142	-2.341	-0.4143	BGIOSGA006565	XP_006643724.1 WAT1-related protein At3g18200-like
TCONS_00013454	-0.713943	-0.187908	-1.70785	BGIOSGA020093	XP_015690743.1 calmodulin-binding receptor-like cytoplasmic kinase 2 isoform X3
TCONS_00009328	-0.713757	-0.178844	0.142021	BGIOSGA022199	XP_006651048.1 WD repeat-containing protein 3
TCONS_00014088	-0.713517	-1.51409	-1.10288	BGIOSGA016239	XP_003581133.1uncharacterized protein At3g27210 isoform X1
TCONS_00026817	-0.71298	0.605299	0.145944	BGIOSGA028878	XP_006659517.1PREDICTED: uncharacterized protein LOC102716893
TCONS_00012198	-0.712958	-0.829985	-1.19612	BGIOSGA010808	XP_015690806.1 protein stum-like
TCONS_00035681	-0.712937	-0.261778	-0.492751	BGIOSGA037100	XP_004985978.1uncharacterized protein LOC101765276 isoform X2
TCONS_00030036	-0.712836	-3.51582	1.34525	BGIOSGA030755	XP_010238156.1uncharacterized protein LOC104584676
TCONS_00017948	-0.711979	0.367782	-0.910162	BGIOSGA006433	XP_015692856.1 protein GPR107
TCONS_00028917	-0.711913	-1.70474	1.53553	BGIOSGA009606	XP_006661184.1 probable acyl-activating enzyme 17, peroxisomal
TCONS_00001877	-0.711794	-0.104421	-0.0325479	BGIOSGA004511	XP_006644762.1PREDICTED: uncharacterized protein LOC102708939
TCONS_00001982	-0.711756	-1.24902	-1.48893	BGIOSGA004616	XP_024315087.1disease resistance protein At4g27190
TCONS_00035049	-0.711669	-0.479414	-1.04028	BGIOSGA004260	XP_006662979.1 myb-related protein 306-like
TCONS_00028055	-0.711508	-1.85813	-0.555253	BGIOSGA017615	XP_015695611.1 NAC transcription factor 29
TCONS_00001937	-0.710504	-1.56312	-0.996497	BGIOSGA000701	XP_015691453.1 putative disease resistance RPP13-like protein 1
TCONS_00018326	-0.710353	1.56815	-1.46815	BGIOSGA025611	XP_004961187.1protein RADIALIS-like 3
TCONS_00027722	-0.710281	-0.643661	-0.888635	BGIOSGA027284	XP_003573223.1mediator of RNA polymerase II transcription subunit 15a
TCONS_00001085	-0.709771	-0.0162754	0.390195	BGIOSGA006640	XP_006644291.1 bidirectional sugar transporter SWEET2a
TCONS_00021544	-0.709608	-0.474743	-1.21567	BGIOSGA015766	XP_004964433.1arginine decarboxylase 1
TCONS_00001296	-0.709554	-0.653987	-0.998441	BGIOSGA017460	XP_015688063.1 inactive protein kinase SELMODRAFT_444075-like
TCONS_00029186	-0.709346	0.22197	-1.75401	BGIOSGA001420	XP_006660720.2 glutathione transferase GST 23-like
TCONS_00011192	-0.708765	0.23153	0.561339	BGIOSGA025387	XP_006651941.1 protein phosphatase 2C 70-like
TCONS_00002547	-0.708344	-0.668734	-1.63392	BGIOSGA000093	XP_006645379.1 probable NADPH:quinone oxidoreductase 2
TCONS_00004130	-0.708244	-0.405232	-1.70109	BGIOSGA025053	XP_003566969.1GDSL esterase/lipase At4g10955
TCONS_00030491	-0.708159	-0.0844034	-0.502753	BGIOSGA014220	XP_006660944.1 bromodomain-containing protein 4B-like
TCONS_00025862	-0.707784	-2.07942	-1.64552	BGIOSGA017137	XP_015696057.1 probable UDP-arabinose 4-epimerase 3 isoform X2
TCONS_00014506	-0.707456	0.345513	-1.11066	BGIOSGA024505	XP_006652455.1 probable calcium-binding protein CML22
TCONS_00023202	-0.707079	0.27381	0.0858804	BGIOSGA025244	XP_015695266.1 MMS19 nucleotide excision repair protein homolog
TCONS_00013888	-0.706898	0.0609562	-0.0615123	#N/A	#N/A
TCONS_00009058	-0.706452	0.533772	0.00864633	BGIOSGA032526	XP_004954416.1protein KINESIN LIGHT CHAIN-RELATED 2
TCONS_00030828	-0.706277	-1.3008	-0.384053	BGIOSGA017413	XP_021302658.1uncharacterized protein LOC8077899 isoform X2
TCONS_00028038	-0.706237	-2.49342	-1.59372	BGIOSGA026953	XP_015696094.1 THO complex subunit 4D-like isoform X2
TCONS_00026859	-0.706218	-2.47296	-1.05034	BGIOSGA013792	XP_006659534.1 probable galactinol--sucrose galactosyltransferase 1
TCONS_00010971	-0.706112	-0.678113	-0.0505063	BGIOSGA012476	XP_006650597.1 phytochrome C
TCONS_00007506	-0.70564	-1.00462	-0.516419	BGIOSGA004239	XP_003571064.1alpha/beta hydrolase domain-containing protein 17C
TCONS_00008563	-0.705335	-1.34219	-1.19956	BGIOSGA013699	XP_015689391.1 protein argonaute 1A isoform X2
TCONS_00014108	-0.70529	1.7702	0.342192	BGIOSGA016263	XP_006644320.2 branchpoint-bridging protein
TCONS_00006788	-0.705283	-0.226763	-0.249015	BGIOSGA008963	XP_015688723.1 tRNA methyltransferase 10 homolog A
TCONS_00028289	-0.705195	-0.236266	-0.501891	BGIOSGA026690	XP_006659578.1 plant intracellular Ras-group-related LRR protein 4
TCONS_00009334	-0.70517	#NA	#NA	BGIOSGA011881	XP_015690478.1 FAD-dependent urate hydroxylase-like
TCONS_00027658	-0.704777	1.95605	-1.38777	BGIOSGA036847	XP_008662693.1protein SMAX1-like
TCONS_00011758	-0.70475	-0.661898	-0.180564	BGIOSGA011247	XP_015690542.1 FAD-linked sulfhydryl oxidase ERV1
TCONS_00033661	-0.70462	-0.065549	0.250222	BGIOSGA035443	XP_006662980.1 actin-related protein 2/3 complex subunit 5A-like
TCONS_00002989	-0.70448	-1.27521	-1.1279	BGIOSGA030173	XP_015694374.1PREDICTED: uncharacterized protein LOC102710794
TCONS_00007682	-0.704348	-0.156384	-1.54083	BGIOSGA035541	XP_006648495.1 hydroquinone glucosyltransferase-like
TCONS_00027030	-0.704322	-1.38334	0.166801	BGIOSGA013958	XP_004974059.1ankyrin repeat domain-containing protein 2A
TCONS_00031880	-0.704275	-1.41023	-1.0105	BGIOSGA024599	XP_008672768.1thioredoxin superfamily protein isoform X1
TCONS_00004756	-0.703883	-4.11116	-2.38998	BGIOSGA019949	XP_008672666.1uncharacterized LOC100278681 isoform X1
TCONS_00007425	-0.703554	-0.650395	-0.470904	BGIOSGA034461	XP_006648354.1 copper-transporting ATPase RAN1-like
TCONS_00031228	-0.703309	1.22384	0.00844874	BGIOSGA028838	XP_015697008.1PREDICTED: uncharacterized protein LOC102702414
TCONS_00034842	-0.703283	-0.997414	-0.648974	BGIOSGA037268	XP_006662931.1 zinc finger CCCH domain-containing protein 63
TCONS_00032697	-0.703275	-2.30457	-0.0695512	BGIOSGA019401	XP_015697078.1PREDICTED: uncharacterized protein LOC102718872, partial

TCONS_00035646	-0.703256	-0.0784973	0.0380946	BGIOSGA037067	XP_006663862.1 RNA cytidine acetyltransferase 1-like
TCONS_00005906	-0.703162	0.316657	0.405805	BGIOSGA004832	XP_015691946.1 DNA excision repair protein ERCC-8-like
TCONS_00019456	-0.702568	-0.516964	-0.619992	BGIOSGA000537	XP_004961800.1TBC1 domain family member 10B
TCONS_00006808	-0.70249	-0.40168	-0.186259	BGIOSGA021662	XP_006647842.1 MADS-box transcription factor 57 isoform X2
TCONS_00016862	-0.702386	0.863095	0.241801	BGIOSGA009838	XP_008668693.1probable acyl-activating enzyme 5, peroxisomal
TCONS_00019525	-0.702282	0.857848	1.22132	BGIOSGA014947	XP_006655431.1 probable leucine-rich repeat receptor-like protein kinase At5g49770
TCONS_00034519	-0.702262	-1.10261	-0.52277	BGIOSGA028326	XP_015697970.1 putative disease resistance protein RGA4
TCONS_00001199	-0.702228	-0.679585	0.379104	BGIOSGA017450	XP_004969422.1protein HIR1
TCONS_00013311	-0.701899	0.0827448	0.252833	BGIOSGA038334	XP_006650706.1 probable aldehyde oxidase 2
TCONS_00010356	-0.70183	-1.68708	-0.497879	BGIOSGA012938	XP_015698679.1 mechanosensitive ion channel protein 2, chloroplastic-like isoform X2
TCONS_00037929	-0.701346	-0.571981	-0.485512	BGIOSGA032375	XP_015697254.1PREDICTED: uncharacterized protein LOC102715977
TCONS_00017164	-0.701024	0.123006	-0.491183	BGIOSGA026380	XP_002439318.160S ribosomal protein L18-3
TCONS_00010970	-0.700897	-2.40369	-0.495107	BGIOSGA031506	XP_015690342.1 sulfated surface glycoprotein 185 isoform X1
TCONS_00005234	-0.700607	-0.673458	-4.60419	BGIOSGA016364	XP_006646767.1 sucrose:sucrose 1-fructosyltransferase-like
TCONS_00037510	-0.700198	-1.46518	0.248016	BGIOSGA037764	XP_006664184.1 putative serine/threonine-protein kinase
TCONS_00030857	-0.700192	-0.554033	-0.370236	BGIOSGA032184	XP_015697115.1 uncharacterized calcium-binding protein At1g02270
TCONS_00012086	-0.700026	0.859386	0.313939	BGIOSGA010926	XP_006649924.1 gamma-interferon-inducible lysosomal thiol reductase-like isoform X1
TCONS_00023326	-0.699954	-1.62065	-0.598062	BGIOSGA023374	XP_006657544.1 transcription initiation factor TFIID subunit 5-like isoform X2
TCONS_00002148	-0.69949	-0.152153	#NA	BGIOSGA004776	XP_006644991.1 elicitor-responsive protein 1 isoform X1
TCONS_00025092	-0.699254	-0.0390077	-0.605912	BGIOSGA024307	XP_006657698.1 putative disease resistance protein RGA3
TCONS_00031923	-0.698942	-1.4957	-1.20032	BGIOSGA032222	XP_006662227.2 pentatricopeptide repeat-containing protein MRL1, chloroplastic
TCONS_00035689	-0.698887	-0.728455	0.995381	BGIOSGA037107	XP_006663888.1 2-oxoisovalerate dehydrogenase subunit alpha 2, mitochondrial
TCONS_00030022	-0.697574	-0.763138	-1.52233	BGIOSGA029784	XP_015696250.1 sprT-like domain-containing protein Spartan isoform X2
TCONS_00019051	-0.697332	0.4103	-0.269122	BGIOSGA006841	XP_006654257.1 receptor-like protein kinase FERONIA
TCONS_00023543	-0.697197	-0.533897	-1.13236	BGIOSGA004871	XP_006658496.1 sterol 3-beta-glucosyltransferase UGT80A2-like
TCONS_00036488	-0.696768	-0.336728	-1.13729	BGIOSGA019255	XP_015698378.1 receptor-like protein kinase HSL1
TCONS_00008462	-0.696552	-1.25814	-0.638917	BGIOSGA033028	XP_006647582.1 putative serine/threonine-protein kinase
TCONS_00021234	-0.696294	-0.58495	-0.271828	BGIOSGA007073	XP_006656375.1PREDICTED: uncharacterized protein LOC102711268
TCONS_00005631	-0.696286	-1.99448	-0.201728	BGIOSGA007743	XP_015688370.1 putative F-box protein At2g02030
TCONS_00005844	-0.696033	0.352787	0.0795843	BGIOSGA037981	XP_002451101.2putative disease resistance RPP13-like protein 2
TCONS_00013129	-0.695936	-0.960951	-0.432392	BGIOSGA020596	XP_004981867.1GATA transcription factor 19
TCONS_00006140	-0.695833	-0.902318	-0.818367	BGIOSGA008298	XP_015688836.1 RNA pseudouridine synthase 7
TCONS_00012595	-0.695777	0.8496	-0.156585	BGIOSGA023918	XP_006659162.1 40S ribosomal protein S3-3
TCONS_00002940	-0.695712	-1.67327	-0.508001	BGIOSGA010192	XP_021312743.1protein kinase PINOID 2
TCONS_00011401	-0.695652	-1.26822	-0.00599613	BGIOSGA025660	XP_015690188.1PREDICTED: uncharacterized protein LOC102704689 isoform X2
TCONS_00021760	-0.695506	0.362097	-1.42605	BGIOSGA036186	XP_002437987.1UDP-glycosyltransferase 73B4
TCONS_00008967	-0.69549	-1.67355	-0.203695	BGIOSGA005437	XP_006648047.2 pentatricopeptide repeat-containing protein At5g27460
TCONS_00018218	-0.695441	-1.07813	-1.06468	BGIOSGA001353	XP_006654716.1PREDICTED: uncharacterized protein LOC102700993 isoform X3
TCONS_00030704	-0.695307	0.112819	-1.18143	BGIOSGA034891	XP_022685043.1serine/threonine-protein kinase-like protein At1g28390
TCONS_00010438	-0.695202	-1.90682	-0.673766	BGIOSGA013030	XP_006650301.1 probable cytokinin riboside 5'-monophosphate phosphoribohydrolase LOGL5
TCONS_00023165	-0.694798	1.78569	-0.277169	BGIOSGA025203	XP_006657474.1 nucleolar protein 58-like
TCONS_00029039	-0.694264	-0.915869	-0.85273	BGIOSGA004301	XP_006644589.1 glycylpeptide N-tetradecanoyltransferase 1
TCONS_00016541	-0.693834	-0.947959	-1.03527	BGIOSGA016114	XP_006653729.1 wall-associated receptor kinase 3-like
TCONS_00011486	-0.693296	-1.76257	-0.739945	BGIOSGA011540	NP_001148819.1cell growth defect factor 2
TCONS_00037511	-0.693108	0.577768	1.79758	BGIOSGA024833	XP_006664735.1 DEAD-box ATP-dependent RNA helicase 9
TCONS_00011285	-0.692919	-0.555977	1.09205	BGIOSGA013888	XP_015691179.1 electron transfer flavoprotein subunit alpha, mitochondrial
TCONS_00024487	-0.692675	-0.0246661	0.198265	BGIOSGA006781	XP_008780672.2PREDICTED: uncharacterized protein LOC103700519, partial
TCONS_00023650	-0.692668	-0.278154	-0.0191491	BGIOSGA025674	XP_006657685.2 protein GRIP isoform X1
TCONS_00013376	-0.692533	-0.4489	-0.637912	BGIOSGA000279	XP_022679087.1DEAD-box ATP-dependent RNA helicase 37
TCONS_00036092	-0.692434	-0.314069	0.824962	BGIOSGA037455	XP_015698800.1 protein TRIGALACTOSYLDIACYLGLYCEROL 5, chloroplastic-like
TCONS_00008697	-0.691621	-1.35996	-0.218203	BGIOSGA024411	XP_015689348.1 fatty acid desaturase DES2
TCONS_00007791	-0.691261	-0.623444	-1.2882	BGIOSGA006691	XP_006647168.1PREDICTED: uncharacterized protein LOC102702532

TCONS_00016619	-0.690886	-0.838015	-0.0606923	BGIOSGA018413	XP_006653769.1 probable LRR receptor-like serine/threonine-protein kinase At1g56140
TCONS_00036804	-0.690655	1.05802	-0.43295	BGIOSGA010359	XP_015698780.1 leucine-rich repeat receptor-like tyrosine-protein kinase PXC3
TCONS_00007207	-0.690509	-0.959183	-1.51687	#N/A	#N/A
TCONS_00010392	-0.690343	-0.850751	0.828726	BGIOSGA012986	XP_006650251.1PREDICTED: uncharacterized protein LOC102713610
TCONS_00016163	-0.690266	-0.0303907	0.695342	BGIOSGA016881	XP_015690808.1 dnaJ protein homolog
TCONS_00005027	-0.690064	-0.0985767	0.987268	BGIOSGA000191	XP_002459034.1thiamine pyrophosphokinase 1 isoform X1
TCONS_00036578	-0.690064	-0.957221	-0.552097	BGIOSGA034600	XP_015698575.1 protein LURP-one-related 8-like
TCONS_00013564	-0.690036	-1.0173	-0.520857	BGIOSGA008700	XP_006650922.1 probable xyloglucan endotransglucosylase/hydrolase protein 30
TCONS_00035782	-0.689999	-1.23052	-0.0459505	BGIOSGA019250	XP_006662491.1 NAD-dependent malic enzyme 59 kDa isoform, mitochondrial
TCONS_00027140	-0.689944	-0.28733	-0.152372	BGIOSGA038407	XP_006659691.1 60S ribosomal protein L10a
TCONS_00003897	-0.689856	0.687579	1.08728	BGIOSGA034270	XP_003569281.1probable serine/threonine-protein kinase PIX7
TCONS_00010735	-0.689851	-1.43556	-1.5985	BGIOSGA000718	XP_006650423.1 inositol hexakisphosphate and diphosphoinositol-pentakisphosphate kinase-like isoform X1
TCONS_00012706	-0.68982	-1.05706	-0.289672	BGIOSGA010260	XP_010025208.1 putative protease Do-like 14
TCONS_00014987	-0.689742	0.344935	-0.552901	BGIOSGA011960	XP_006652855.1 polyadenylate-binding protein RBP45-like
TCONS_00013248	-0.689244	0.901007	0.366826	BGIOSGA004136	XP_006651859.2 probable LRR receptor-like serine/threonine-protein kinase At2g16250
TCONS_00012593	-0.688801	-0.0988414	-0.510768	BGIOSGA027602	XP_006659160.1 60S ribosomal protein L13-1
TCONS_00021392	-0.688484	-0.378335	-0.216579	BGIOSGA033658	XP_003571225.1uncharacterized protein LOC106866486
TCONS_00005077	-0.687925	-0.281851	-0.656963	BGIOSGA028215	XP_006645316.1 glucan endo-1,3-beta-glucosidase GII-like
TCONS_00025569	-0.687768	1.65655	0.342648	BGIOSGA022508	XP_004958408.1transcription factor MYB30
TCONS_00014156	-0.687144	0.539635	-0.074545	BGIOSGA016310	XP_002436358.1dolichyl-diphosphooligosaccharide--protein glycosyltransferase subunit DAD1
TCONS_00031071	-0.687024	-3.86795	-4.27391	BGIOSGA037494	XP_010234705.1protein PHYTOCHROME KINASE SUBSTRATE 1
TCONS_00001874	-0.686991	1.08621	1.85529	BGIOSGA004507	XP_020405458.1uncharacterized LOC100382829 isoform X1
TCONS_00030960	-0.686712	-1.20054	-0.373772	BGIOSGA010538	XP_015694452.1 probable galacturonosyltransferase 3 isoform X2
TCONS_00033132	-0.686705	0.411367	-0.411657	BGIOSGA025495	XP_006663272.1 lipase-like PAD4
TCONS_00030703	-0.68654	0.0383332	-0.380289	BGIOSGA032294	XP_003572032.1F-box/LRR-repeat protein At2g43260-like
TCONS_00016130	-0.686476	0.0632604	0.411611	BGIOSGA023609	XP_004976006.1elongator complex protein 3
TCONS_00016814	-0.686446	0.506364	-0.767546	BGIOSGA030954	XP_008661900.1transfactor isoform X2
TCONS_00032615	-0.686149	-1.54675	-0.841742	BGIOSGA031497	XP_015697435.1 probable RNA-binding protein 18
TCONS_00014905	-0.686071	-0.0848765	0.268051	BGIOSGA017087	XP_015691570.1PREDICTED: LOW QUALITY PROTEIN: uncharacterized protein LOC102711153
TCONS_00013312	-0.685367	0.78567	0.520812	BGIOSGA025523	XP_006650706.1 probable aldehyde oxidase 2
TCONS_00000480	-0.685282	-1.34328	-0.944003	BGIOSGA026880	XP_002457084.2cytochrome P450 71A1
TCONS_00007046	-0.685134	-0.226468	-1.0118	BGIOSGA007045	XP_015688907.1 putative clathrin assembly protein At5g35200
TCONS_00034383	-0.685088	-1.44717	-1.57322	BGIOSGA033955	XP_006662768.1 CBS domain-containing protein CBSCBSPB3-like
TCONS_00023026	-0.685069	-1.41157	0.239455	BGIOSGA025627	XP_004955370.1transcription factor NIGTH1
TCONS_00023772	-0.684478	-1.36865	-2.25045	BGIOSGA025812	XP_006658614.1 condensin-2 complex subunit H2
TCONS_00006947	-0.684319	-0.239079	-0.585713	BGIOSGA021701	XP_006647986.1 probable cyclic nucleotide-gated ion channel 20, chloroplastic
TCONS_00005683	-0.684226	-0.887194	-0.94054	BGIOSGA021001	XP_006647077.1 elongation factor 1-gamma 2
TCONS_00001491	-0.684219	-0.500433	-0.197417	BGIOSGA004132	XP_006644453.1 actin-related protein 2/3 complex subunit 2A
TCONS_00002690	-0.68406	-0.0676742	-0.0278865	BGIOSGA003623	XP_006644461.1PREDICTED: uncharacterized protein LOC102713683
TCONS_00007927	-0.683898	0.313751	-0.432869	BGIOSGA006765	XP_006647232.1 SNAP25 homologous protein SNAP33-like
TCONS_00003043	-0.683882	2.27785	0.654969	BGIOSGA018619	XP_003565474.1flocculation protein FLO11
TCONS_00011926	-0.683824	1.03363	-1.53342	#N/A	#N/A
TCONS_00000486	-0.683757	0.174222	0.690933	BGIOSGA007951	XP_006643942.1 target of Myb protein 1-like
TCONS_00032380	-0.683682	0.0926766	-0.0361925	BGIOSGA033210	XP_006661840.1 WD repeat-containing protein 70
TCONS_00000710	-0.68339	-0.844516	-0.282561	BGIOSGA003313	XP_006644087.1 squamosa promoter-binding-like protein 1 isoform X2
TCONS_00018432	-0.68327	-0.550457	-1.08785	BGIOSGA020487	NP_001151864.1CCT motif family protein
TCONS_00005617	-0.683009	-0.898584	-0.704125	BGIOSGA027823	XP_006647021.2 zinc transporter ZIP12
TCONS_00017140	-0.682981	-0.244137	-0.0856749	BGIOSGA022823	XP_015693070.1 probable chromatin-remodeling complex ATPase chain
TCONS_00031728	-0.68285	-0.820803	-1.05102	BGIOSGA031434	XP_015697434.1 protein NRT1/ PTR FAMILY 8.3-like isoform X2
TCONS_00016900	-0.682715	-0.781232	-1.72402	BGIOSGA016161	XP_008656972.1oxalate--CoA ligase isoform X2
TCONS_00023439	-0.682583	#NA	-2.12149	#N/A	#N/A
TCONS_00009044	-0.68214	-0.853598	-0.770893	BGIOSGA005361	XP_006648117.1 actin-related protein 2/3 complex subunit 1B-like
TCONS_00009184	-0.68193	0.549352	1.32965	BGIOSGA011712	XP_015689743.1 mitochondrial inner membrane protein OXA1-like
TCONS_00007041	-0.681803	-0.968513	-1.12344	#N/A	#N/A
TCONS_00025432	-0.680999	-1.08944	-0.842815	BGIOSGA023996	XP_003562794.1protein EFFECTOR OF TRANSCRIPTION 2
TCONS_00005490	-0.680869	-0.119576	-0.743523	BGIOSGA022468	XP_006646917.1 auxin response factor 6

transcriptome

TCONS_00009500	-0.680831	-1.29378	-0.310083	BGIOSGA012050	XP_006649566.1 J domain-containing protein required for chloroplast accumulation response 1 isoform X2
TCONS_00029549	-0.680785	0.0313205	-1.2212	BGIOSGA003264	XP_006661007.1 probable anion transporter 4, chloroplastic
TCONS_00009045	-0.680763	-0.941055	-0.707792	BGIOSGA004190	XP_006648119.1 auxin-responsive protein IAA10 isoform X1
TCONS_00018693	-0.680528	-0.796366	-0.393455	BGIOSGA008656	XP_006655034.1 uncharacterized membrane protein At1g06890-like
TCONS_00016985	-0.679818	-0.867277	-0.653483	BGIOSGA019033	XP_015692894.1PREDICTED: uncharacterized protein LOC107304226
TCONS_00025966	-0.679624	-0.096882	-1.2448	BGIOSGA004431	XP_020400588.1uncharacterized protein LOC100278423 isoform X1
TCONS_00012271	-0.679574	-0.554805	-0.510419	#N/A	#N/A
TCONS_00011596	-0.679091	-0.596559	-1.45481	BGIOSGA009625	XP_015691283.1 ABC transporter G family member 22-like isoform X1
TCONS_00008299	-0.678983	-0.528321	0.0664274	BGIOSGA006129	XP_006647449.1PREDICTED: uncharacterized protein LOC102715451
TCONS_00000514	-0.678749	-0.502326	0.169414	BGIOSGA003088	XP_002457057.1heme-binding protein 2
TCONS_00018614	-0.67848	0.0671065	0.658256	BGIOSGA018820	XP_015692814.1 E3 ubiquitin-protein ligase SIS3 isoform X2
TCONS_00026034	-0.678437	0.6385	-1.07025	BGIOSGA017826	XP_004972697.1homeobox-leucine zipper protein ROC1
TCONS_00022286	-0.678391	-0.61073	-0.244764	BGIOSGA020524	XP_006656104.1 G-type lectin S-receptor-like serine/threonine-protein kinase SD2-5
TCONS_00010906	-0.678373	-0.287737	-1.28493	BGIOSGA009837	XP_004981852.1ATP synthase delta chain, chloroplastic
TCONS_00022316	-0.678346	-2.90447	-0.793726	BGIOSGA007918	XP_006656116.2PREDICTED: uncharacterized protein At5g41620-like
TCONS_00010955	-0.678164	0.0892042	-0.61129	BGIOSGA013556	XP_006650584.1 PHD finger protein ING2 isoform X2
TCONS_00025863	-0.677858	-0.235786	-1.13343	BGIOSGA019301	XP_006659090.1PREDICTED: uncharacterized protein LOC102703418
TCONS_00000117	-0.677713	-1.71409	-2.43868	BGIOSGA002690	XP_006645453.1 multicopper oxidase LPR1-like
TCONS_00019985	-0.677693	-0.0406115	0.153254	BGIOSGA022171	XP_006655705.1 5'-methylthioadenosine/S-adenosylhomocysteine nucleosidase 2-like
TCONS_00013574	-0.677027	#NA	-1.55057	BGIOSGA030041	XP_006652046.1PREDICTED: uncharacterized protein LOC102714724
TCONS_00020321	-0.676978	-1.95421	-0.897281	BGIOSGA005507	XP_004964928.1kinesin-like protein KIN-7D, chloroplastic isoform X1
TCONS_00018514	-0.676923	0.312728	-0.944513	BGIOSGA018921	NP_001150247.1glycine-rich protein A3
TCONS_00015927	-0.676846	-0.402178	0.507542	BGIOSGA006261	XP_015691463.1 mitogen-activated protein kinase kinase kinase YODA-like
TCONS_00035491	-0.676828	0.248905	-0.9674	BGIOSGA006948	NP_001168484.1protein SCARECROW
TCONS_00027367	-0.676803	-0.776892	-0.888748	BGIOSGA010945	XP_003573480.1protein NRT1/ PTR FAMILY 6.3
TCONS_00001267	-0.676787	-1.26882	-1.73977	BGIOSGA034661	XP_015695087.1 probable strigolactone esterase DAD2
TCONS_00010112	-0.676762	0.163562	0.185726	#N/A	#N/A
TCONS_00005636	-0.676701	-0.883027	0.15504	BGIOSGA012839	XP_003571443.1SPX domain-containing protein 1
TCONS_00010380	-0.676115	-3.78174	-1.64723	BGIOSGA025868	XP_006650248.1 receptor-like serine/threonine-protein kinase SD1-8
TCONS_00029159	-0.676007	-1.13463	-0.5122	BGIOSGA030911	XP_006660702.1 calmodulin-like protein 1
TCONS_00013894	-0.67582	1.2059	1.62857	BGIOSGA033968	XP_006663461.1 putative disease resistance RPP13-like protein 1
TCONS_00004724	-0.675794	-1.79416	-1.26054	BGIOSGA031494	XP_015688150.1 BEL1-like homeodomain protein 9
TCONS_00021222	-0.675781	-0.731348	-0.0489727	BGIOSGA023443	XP_006657238.1 peptidyl-prolyl cis-trans isomerase CYP59
TCONS_00008078	-0.675391	-1.13096	-2.10374	BGIOSGA006372	XP_015689629.1 probable magnesium transporter NIPA8
TCONS_00014668	-0.675184	-0.602884	-0.844281	BGIOSGA014156	XP_003580229.1beta-fructofuranosidase 1
TCONS_00009419	-0.675159	0.898629	0.036934	BGIOSGA011972	XP_006649494.1 elongation factor 1-alpha
TCONS_00005213	-0.674851	-2.26928	-4.2246	BGIOSGA008154	XP_006648199.2 GDSL esterase/lipase EXL3-like
TCONS_00020701	-0.674753	-2.40889	-3.00786	BGIOSGA040503	NP_001144651.1uncharacterized LOC100277676
TCONS_00016167	-0.674496	-1.76046	1.20057	BGIOSGA008588	XP_006652461.1PREDICTED: uncharacterized protein LOC102713263
TCONS_00036643	-0.67399	0.344906	1.10469	BGIOSGA034531	XP_006664849.1 mitochondrial acidic protein MAM33
TCONS_00031214	-0.673697	0.342845	-0.232268	BGIOSGA013750	XP_006661799.2 ABC transporter G family member 28-like
TCONS_00001450	-0.673496	-0.340083	0.876556	BGIOSGA025813	XP_015693815.1 AAA-ATPase At3g50940-like isoform X1
TCONS_00027071	-0.673464	-1.41051	-0.426087	BGIOSGA029396	XP_015696047.1 GTPase-activating protein gyp7-like
TCONS_00020566	-0.673224	-0.598889	-0.184833	BGIOSGA022744	XP_006656030.1 formin-binding protein 4 isoform X2
TCONS_00021488	-0.673189	-0.317749	-0.364031	BGIOSGA030428	XP_002437714.1uncharacterized membrane protein At1g75140
TCONS_00016395	-0.673134	-2.12286	-2.93039	BGIOSGA026759	XP_006652626.1 cis-zeatin O-glucosyltransferase 1-like
TCONS_00033916	-0.672737	-1.47283	-0.729909	BGIOSGA032285	XP_015697728.1 F-box/LRR-repeat protein 4-like
TCONS_00012373	-0.672479	-0.756349	-1.5621	BGIOSGA034236	XP_003561581.1copper-transporting ATPase PAA1, chloroplastic isoform X2
TCONS_00014785	-0.672352	0.798697	0.752372	BGIOSGA004637	XP_006652670.1 cytochrome P450 704C1-like
TCONS_00028958	-0.671972	-0.242366	-0.563388	#N/A	#N/A
TCONS_00036091	-0.671905	-0.537627	-0.183216	BGIOSGA004722	XP_006664581.2 probable WRKY transcription factor 3
TCONS_00024844	-0.67176	0.242783	-0.197096	BGIOSGA024529	XP_006652114.1PREDICTED: uncharacterized protein KIAA0930 homolog
TCONS_00034215	-0.671689	-1.78007	-2.07297	BGIOSGA030934	XP_006663775.2 protein DETOXIFICATION 21-like isoform X1

TCONS_00025990	-0.67168	-0.124182	-0.621169	BGIOSGA037204	XP_004979650.1putative disease resistance protein At1g50180
TCONS_00014147	-0.671649	-0.757317	-0.827243	BGIOSGA021885	XP_003579655.1glycerophosphodiester phosphodiesterase GDPD2
TCONS_00037608	-0.671164	-0.369794	-0.250199	BGIOSGA017996	XP_006664784.2 protein NRT1/ PTR FAMILY 2.11-like
TCONS_00018773	-0.671131	0.406677	-0.956735	BGIOSGA026934	XP_015692935.1 NAC domain-containing protein 73-like isoform X2
TCONS_00021927	-0.671038	-1.98043	-3.15072	BGIOSGA004442	XP_015693214.1 pseudo histidine-containing phosphotransfer protein 5
TCONS_00031474	-0.67095	-0.136693	0.135926	BGIOSGA019185	XP_022681533.1uncharacterized protein LOC101778128 isoform X2
TCONS_00022815	-0.670864	-2.21778	-3.30365	BGIOSGA001741	XP_006657280.1 IAA-amino acid hydrolase ILR1-like 6
TCONS_00013138	-0.670737	-1.21397	0.180947	BGIOSGA009847	XP_006650556.1 CBS domain-containing protein CBSX3, mitochondrial
TCONS_00022902	-0.670454	-0.343769	-1.024	BGIOSGA020541	XP_006657342.1 UPF0183 protein At3g51130
TCONS_00031912	-0.670375	-0.207807	-0.406071	BGIOSGA032230	NP_001304792.1uncharacterized LOC100839725
TCONS_00013289	-0.669953	-1.96259	-2.10347	BGIOSGA000019	XP_003559356.1zinc finger protein GIS
TCONS_00033179	-0.669742	-0.371956	-0.629223	BGIOSGA037126	XP_006662832.2PREDICTED: uncharacterized protein At4g06598-like
TCONS_00013255	-0.669567	-1.90672	-3.53427	BGIOSGA009733	XP_002463811.1putative S-adenosyl-L-methionine-dependent methyltransferase Mvan_0910 isoform X4
TCONS_00012212	-0.669439	0.339584	-0.83285	BGIOSGA010796	XP_006651346.1PREDICTED: uncharacterized protein LOC102714000
TCONS_00035883	-0.669006	-0.495833	-0.225033	BGIOSGA002235	XP_006663963.1 serine/threonine-protein kinase tricorner-like
TCONS_00025462	-0.668961	0.135539	0.219051	BGIOSGA026350	XP_006657913.1 probable serine/threonine-protein kinase At1g54610 isoform X1
TCONS_00013783	-0.668953	-1.40752	0.0688341	BGIOSGA001216	XP_004974538.1senescence-specific cysteine protease SAG39
TCONS_00027459	-0.668802	-0.19621	-0.166006	BGIOSGA037091	XP_008676865.1putative rnase H family protein isoform X4
TCONS_00007259	-0.668778	-1.03303	-1.2735	BGIOSGA007223	XP_015688434.1PREDICTED: uncharacterized protein LOC102710761
TCONS_00034824	-0.668642	-0.295258	-0.934398	#N/A	#N/A
TCONS_00018291	-0.66858	-0.0890478	0.141286	BGIOSGA020344	XP_006655582.1 phenylalanine--tRNA ligase beta subunit, cytoplasmic
TCONS_00009882	-0.668186	0.796638	0.631589	BGIOSGA004612	XP_015691299.1 WD repeat-containing protein 43
TCONS_00009756	-0.668149	0.131398	-1.97703	BGIOSGA031552	XP_003558255.1uncharacterized protein LOC100836685
TCONS_00007208	-0.668132	0.744394	-0.167668	BGIOSGA011129	XP_022679979.1uncharacterized protein LOC111256358
TCONS_00027486	-0.667734	-1.86916	-1.36527	BGIOSGA008383	XP_006659901.1 probable disease resistance protein RXW24L
TCONS_00020358	-0.66764	0.646676	-0.398368	BGIOSGA021834	XP_008649239.2nascent polypeptide-associated complex subunit alpha, muscle-specific form
TCONS_00034292	-0.667366	-1.57497	-0.821757	#N/A	#N/A
TCONS_00008573	-0.667114	0.632757	-0.438352	BGIOSGA014527	XP_006647677.1 homeobox-leucine zipper protein ROC5
TCONS_00008338	-0.666829	-1.65074	-0.982887	BGIOSGA024890	XP_015689002.1 chitinase 6 isoform X1
TCONS_00018271	-0.66672	-0.658262	0.413799	BGIOSGA001010	XP_015692727.1 protein DETOXIFICATION 14-like isoform X1
TCONS_00025128	-0.666401	0.122698	0.774011	BGIOSGA024276	XP_006658571.1PREDICTED: uncharacterized protein LOC102722974
TCONS_00005350	-0.665784	-0.732581	-1.4034	BGIOSGA007459	XP_015688444.1PREDICTED: uncharacterized protein LOC102718805, partial
TCONS_00033730	-0.665767	0.488114	0.697358	BGIOSGA037750	XP_006662999.1 protein ROOT HAIR DEFECTIVE 3 homolog 2
TCONS_00003396	-0.665375	0.235007	-0.165018	BGIOSGA021400	XP_004982036.1protein FAF-like, chloroplastic isoform X1
TCONS_00030568	-0.665375	1.23501	-2.16502	BGIOSGA030654	XP_006661009.1 ACT domain-containing protein ACR10
TCONS_00002209	-0.665202	-1.24689	-0.292181	BGIOSGA030800	XP_006645060.1 glucan endo-1,3-beta-glucosidase 14-like
TCONS_00030607	-0.664243	-0.305891	-1.09934	BGIOSGA012197	XP_002467392.1probable NAD(P)H-dependent oxidoreductase 1
TCONS_00025596	-0.664127	0.513535	-0.360788	BGIOSGA023837	XP_015695129.1 thymidylate kinase
TCONS_00008838	-0.664017	0.326113	-0.0202045	BGIOSGA018572	XP_006647942.1 universal stress protein Sll1388
TCONS_00015502	-0.663782	-3.64485	-1.247	BGIOSGA010570	XP_006652128.1 sucrose synthase 7
TCONS_00025380	-0.66361	-0.945771	-0.752152	BGIOSGA024052	XP_006657865.2 protein TIME FOR COFFEE
TCONS_00002313	-0.663428	-0.98985	-3.16989	BGIOSGA011445	XP_004970852.1LOB domain-containing protein 6
TCONS_00011781	-0.663135	-1.70531	-1.20121	BGIOSGA035949	XP_004985228.1probable galacturonosyltransferase 14
TCONS_00030091	-0.662784	-1.06279	-1.95627	BGIOSGA035936	XP_015696647.1 zinc finger protein 2-like
TCONS_00037165	-0.662565	-0.62837	-0.809472	BGIOSGA016997	XP_015698295.1 bidirectional sugar transporter SWEET13-like
TCONS_00018107	-0.662464	0.0522998	0.177616	BGIOSGA020162	XP_006654628.2 ubiquitin carboxyl-terminal hydrolase 25
TCONS_00025768	-0.662326	-1.36901	-0.880621	BGIOSGA027830	XP_006659725.1 protein odr-4 homolog
TCONS_00025701	-0.661766	0.329979	1.34953	BGIOSGA021572	XP_006658128.1 peroxidase P7-like
TCONS_00015264	-0.661642	-3.57707	-2.17434	BGIOSGA033976	NP_001183315.1legume lectins beta domain containing protein
TCONS_00013349	-0.661248	0.347231	0.242414	BGIOSGA009634	XP_006650737.1 40S ribosomal protein S15
TCONS_00012282	-0.660235	-0.421748	-0.475929	BGIOSGA010723	XP_006650090.1 beta-adaptin-like protein C
TCONS_00027615	-0.660068	-1.63322	-0.896584	BGIOSGA011034	XP_006645004.1 probable mannose-1-phosphate guanylyltransferase 3
TCONS_00019771	-0.660027	0.804602	0.143582	BGIOSGA031285	XP_006654770.1 ribosomal L1 domain-containing protein 1-like

TCONS_00003663	-0.659866	-0.354079	-1.44087	BGIOSGA001542	XP_006645936.2 plastidal glycolate/glycerate translocator 1, chloroplastic
TCONS_00018233	-0.659833	0.0353639	1.05958	BGIOSGA020290	XP_006654729.1 lys-63-specific deubiquitinase BRCC36-like isoform X1
TCONS_00027008	-0.659749	-1.08028	-0.123992	BGIOSGA009879	XP_006659627.2PREDICTED: uncharacterized protein LOC102702588 isoform X1
TCONS_00027256	-0.659701	-2.57951	-1.17915	BGIOSGA001310	XP_004972995.1ACT domain-containing protein ACR8
TCONS_00012130	-0.659599	-1.69093	-2.0699	BGIOSGA010878	XP_006651313.2 protein high chlorophyll fluorescent 107
TCONS_00013895	-0.659384	-0.000435969	-0.39388	#N/A	#N/A
TCONS_00017812	-0.659124	0.464157	0.906883	BGIOSGA022123	XP_006654408.1 glutathione gamma-glutamylcysteinyltransferase 1-like
TCONS_00012090	-0.65902	-0.00275054	-0.687928	BGIOSGA001394	XP_006649932.1PREDICTED: uncharacterized protein At5g01610-like
TCONS_00002497	-0.658901	-0.0886644	-1.18113	BGIOSGA007803	XP_006645310.1 cytokinin dehydrogenase 4
TCONS_00008183	-0.658629	0.287037	-1.41627	BGIOSGA015045	XP_006647375.1 DDT domain-containing protein DDB_G0282237-like
TCONS_00005155	-0.658388	-0.628232	-2.01987	BGIOSGA000049	XP_015695364.1PREDICTED: uncharacterized protein LOC102722642
TCONS_00002298	-0.658374	-2.22099	-1.4133	BGIOSGA004937	XP_006646543.1 abhydrolase domain-containing protein C22H12.03
TCONS_00019836	-0.658355	1.1813	0.276954	BGIOSGA020396	XP_006654818.1 CTP synthase-like isoform X2
TCONS_00030046	-0.658124	-0.929416	-0.990403	BGIOSGA020219	XP_006660625.1 ATP-dependent 6-phosphofructokinase 5, chloroplastic-like
TCONS_00003278	-0.658042	-0.244532	-0.0306399	BGIOSGA001937	XP_015688147.1PREDICTED: uncharacterized protein LOC102722266
TCONS_00023856	-0.657926	-0.98589	-0.942528	BGIOSGA027694	XP_006658657.1 putative receptor-like protein kinase At4g00960
TCONS_00016888	-0.65778	-0.412	-0.540041	BGIOSGA014077	XP_006653038.1 choline transporter-like protein 2
TCONS_00002035	-0.657652	0.31408	0.121597	BGIOSGA034571	XP_015688272.1 histone-lysine N-methyltransferase, H3 lysine-9 specific SUVH1-like
TCONS_00032528	-0.657505	-1.01152	-0.896995	BGIOSGA031586	XP_006662498.1 SET and MYND domain-containing protein 4
TCONS_00029621	-0.656595	-0.480308	-2.47653	BGIOSGA020422	XP_006661038.1 cinnamoyl-CoA reductase 1-like
TCONS_00033239	-0.656573	-0.975095	-0.60087	BGIOSGA025398	XP_010941981.2 tubulin alpha chain
TCONS_00035438	-0.656572	-1.15172	-0.342745	BGIOSGA023035	NP_001168546.1putative DUF231 domain containing family protein
TCONS_00003798	-0.656385	0.472618	-1.3531	#N/A	#N/A
TCONS_00011972	-0.65569	-1.52721	-1.46358	BGIOSGA011038	XP_015690181.1 protein DA1-related 2 isoform X1
TCONS_00025335	-0.655411	1.04801	-0.840416	BGIOSGA026500	XP_006657831.1 sugar transport protein 1-like
TCONS_00012338	-0.655143	-0.405326	0.202964	BGIOSGA004037	XP_003557869.1protein COP1 SUPPRESSOR 2
TCONS_00007325	-0.655064	-0.285768	-0.0626347	BGIOSGA007160	XP_006648297.1 protein arginine N-methyltransferase 5
TCONS_00028018	-0.654772	-0.690609	-0.857789	BGIOSGA009203	XP_006659398.1 homeobox-leucine zipper protein HOX5-like
TCONS_00029435	-0.654658	0.487745	-0.636975	BGIOSGA003286	XP_015696731.1 phospholipase D delta
TCONS_00032202	-0.654601	-0.0679213	-0.31839	BGIOSGA030138	XP_008664970.1phospholipid-transporting ATPase 3 isoform X1
TCONS_00036629	-0.654529	-0.969331	-1.39264	BGIOSGA036677	XP_006662714.1 protein ZINC INDUCED FACILITATOR-LIKE 1-like isoform X3
TCONS_00036565	-0.654086	-0.722208	-1.30451	BGIOSGA008170	XP_006662678.1PREDICTED: uncharacterized protein LOC102700746
TCONS_00002287	-0.653817	-0.592108	-0.0290418	#N/A	#N/A
TCONS_00007454	-0.653698	-0.870844	-1.18662	BGIOSGA036907	XP_006646963.1 CBL-interacting serine/threonine-protein kinase 21
TCONS_00002320	-0.653619	-4.51315	-2.97455	BGIOSGA020928	XP_006645151.1 2-alkenal reductase (NADP(+)-dependent)-like
TCONS_00009169	-0.653458	0.21472	-0.0165465	BGIOSGA011696	XP_012704294.1WD repeat-containing protein 6 isoform X1
TCONS_00013458	-0.653355	-1.25306	-0.354113	BGIOSGA011056	XP_015690133.1 anthranilate synthase alpha subunit 1, chloroplastic
TCONS_00013520	-0.652968	-0.751468	0.00421905	BGIOSGA009460	XP_006650882.1 poly(ADP-ribose) glycohydrolase 1-like
TCONS_00034206	-0.65285	-0.862315	-0.797217	BGIOSGA036723	XP_006662812.1 pentatricopeptide repeat-containing protein At4g18975, chloroplastic isoform X2
TCONS_00013628	-0.652612	0.150562	0.0990565	BGIOSGA015778	XP_006652080.2 G patch domain-containing protein 8
TCONS_00017120	-0.652377	1.02953	0.0105877	BGIOSGA010144	XP_006654987.2 serine/threonine-protein phosphatase BSL1 homolog
TCONS_00007151	-0.652254	0.0517698	-0.0850635	BGIOSGA007917	XP_006649191.1 splicing factor 3B subunit 2
TCONS_00020709	-0.652254	-1.44448	-0.483158	BGIOSGA022904	XP_006656969.1 UPF0261 protein RA0729
TCONS_00026391	-0.651947	-1.44487	-1.53122	BGIOSGA028917	XP_006660049.2 probable galacturonosyltransferase 4
TCONS_00020624	-0.651753	-2.12635	-0.639037	#N/A	#N/A
TCONS_00026175	-0.651549	-4.31226	-2.90781	BGIOSGA028248	XP_003573587.1endo-1,3;1,4-beta-D-glucanase isoform X1
TCONS_00018075	-0.651435	-3.91184	-1.92628	BGIOSGA021017	XP_006654605.1 scarecrow-like protein 32
TCONS_00025789	-0.651029	-0.139763	-0.637782	BGIOSGA027850	XP_006659032.1 chromatin assembly factor 1 subunit FAS2 homolog
TCONS_00009648	-0.650716	0.994145	-0.290631	BGIOSGA016495	XP_006649699.1 glutamate decarboxylase 1-like
TCONS_00016538	-0.650453	0.348307	0.471325	BGIOSGA014430	NP_001149203.2seed specific protein Bn15D1B
TCONS_00017095	-0.64996	-0.729862	-0.332276	BGIOSGA019149	XP_003568982.1diacylglycerol O-acyltransferase 3, cytosolic
TCONS_00002064	-0.649502	-0.302905	0.0350021	BGIOSGA004695	XP_006644908.1 WUSCHEL-related homeobox 8
TCONS_00008684	-0.649322	-0.455064	-0.0139631	BGIOSGA003109	XP_015688712.1 probable L-type lectin-domain containing receptor kinase S.5

TCONS_00019767	-0.649282	-0.649217	0.388167	BGIOSGA004180	XP_014754714.1putative ubiquitin-conjugating enzyme E2 38
TCONS_00016245	-0.649265	#NA	-1.48054	BGIOSGA014730	XP_006652507.1 actin-related protein 2/3 complex subunit 2B-like isoform X1
TCONS_00014671	-0.649263	-0.477237	-1.65747	BGIOSGA020093	XP_006652581.1 cysteine-rich repeat secretory protein 15-like
TCONS_00011786	-0.649247	-1.46717	-0.950066	BGIOSGA001236	XP_006649628.1 mitochondrial substrate carrier family protein E
TCONS_00029823	-0.649247	-0.592701	-2.65051	BGIOSGA039286	XP_006661118.1 UDP-glucose 4-epimerase 4 isoform X1
TCONS_00033702	-0.649245	-0.883464	-0.147727	BGIOSGA035481	XP_015697802.1 sialyltransferase-like protein 5 isoform X2
TCONS_00036292	-0.64902	-0.699852	-0.335072	BGIOSGA023244	XP_006279124.2pumilio homolog 2
TCONS_00017325	-0.648507	-0.793152	-0.966874	BGIOSGA035843	XP_002439444.1uncharacterized protein LOC8055740
TCONS_00014380	-0.648339	-0.301079	0.50848	BGIOSGA016537	XP_003579892.1aldose 1-epimerase
TCONS_00017490	-0.648159	-0.00910744	0.594728	BGIOSGA019557	XP_006655241.1 U3 small nucleolar RNA-associated protein 25
TCONS_00035456	-0.647941	-2.03202	-1.29802	BGIOSGA030403	XP_006662689.1 vacuolar protein sorting-associated protein 32 homolog 2-like isoform X3
TCONS_00024218	-0.647877	-0.744523	-0.531131	BGIOSGA026258	XP_015694751.1 SAC3 family protein B
TCONS_00035981	-0.64739	-1.97352	-1.09084	BGIOSGA000228	XP_021316803.1probable kinase CHARK
TCONS_00029023	-0.647195	-1.12212	-0.420341	BGIOSGA036929	XP_006660634.1PREDICTED: uncharacterized protein LOC102716526
TCONS_00001530	-0.646928	-0.678987	-0.629962	BGIOSGA019167	XP_006644479.1 mitogen-activated protein kinase 8 isoform X1
TCONS_00021364	-0.646866	-0.723501	-0.928569	BGIOSGA020791	XP_006656481.1 lysophospholipid acyltransferase LPEAT2
TCONS_00027251	-0.646839	-2.33273	-0.631934	BGIOSGA023467	XP_015695705.1 beta-1,6-galactosyltransferase GALT31A-like
TCONS_00002929	-0.64648	-0.411753	-0.816042	BGIOSGA035976	XP_015699198.1 DEAD-box ATP-dependent RNA helicase 14 isoform X2
TCONS_00001744	-0.646329	-1.01792	-1.53965	BGIOSGA004380	XP_024314621.1protein SPA1-RELATED 4
TCONS_00024331	-0.646163	-0.776071	0.139495	BGIOSGA009806	XP_006658108.1 universal stress protein YxiE-like
TCONS_00018488	-0.646016	0.0812701	0.549248	BGIOSGA005279	XP_015693306.1 E3 ubiquitin-protein ligase XB3
TCONS_00009062	-0.645901	-0.137737	-0.608734	BGIOSGA004297	XP_006654725.2 60S ribosomal protein L28-1-like
TCONS_00016613	-0.645672	-1.24927	-1.15683	BGIOSGA009923	XP_015692331.1 protein FAR1-RELATED SEQUENCE 5-like
TCONS_00016950	-0.64565	-1.17897	-0.962915	BGIOSGA005079	XP_020405759.1uncharacterized protein LOC100276184
TCONS_00011120	-0.645619	-0.258456	-0.0887166	BGIOSGA008212	XP_015690174.1 5'-3' exoribonuclease 4-like
TCONS_00007503	-0.645491	-0.0739187	-0.539613	BGIOSGA034041	XP_002453448.1lycopene beta cyclase, chloroplastic
TCONS_00002641	-0.645489	#NA	-1.16514	BGIOSGA005282	XP_015699261.1 glycine-rich RNA-binding protein 2, mitochondrial-like
TCONS_00037175	-0.64532	0.577439	-0.481673	BGIOSGA001046	XP_015698650.1 auxin response factor 24 isoform X1
TCONS_00017513	-0.645198	0.0705825	-0.685856	BGIOSGA026122	XP_015693347.1 probable leucine-rich repeat receptor-like protein kinase At5g49770
TCONS_00022758	-0.644935	-1.59857	-0.507479	BGIOSGA023452	XP_015693731.1PREDICTED: uncharacterized protein LOC107304379
TCONS_00035059	-0.644652	-1.01352	-0.807694	BGIOSGA037794	XP_010233689.2wall-associated receptor kinase 2
TCONS_00007657	-0.644611	0.451963	0.39012	BGIOSGA006829	XP_006647101.1 E3 ubiquitin-protein ligase CCNB1IP1 homolog isoform X2
TCONS_00029556	-0.644428	-0.938654	-0.778282	BGIOSGA031320	XP_006661584.1 protein SCAI isoform X2
TCONS_00009850	-0.644415	-2.65774	-2.14212	BGIOSGA014428	XP_020396980.1mitogen-activated protein kinase kinase kinase 18
TCONS_00006132	-0.644045	-0.982679	-0.703667	BGIOSGA001241	XP_006647270.2 ATP-binding cassette sub-family F member 1-like
TCONS_00032278	-0.64404	-0.4124	-0.171262	BGIOSGA011896	XP_015697475.1 protein RRP6-like 2
TCONS_00028922	-0.643979	-0.856413	-0.215629	#N/A	#N/A
TCONS_00011466	-0.643794	-0.319587	-0.781839	BGIOSGA001841	XP_004985934.1peroxidase 5
TCONS_00029354	-0.642844	-0.97888	-0.971373	BGIOSGA000531	XP_006660851.1 ankyrin repeat-containing protein At5g02620-like
TCONS_00009286	-0.642792	-3.19756	-2.51173	BGIOSGA004637	XP_006649381.2 alkane hydroxylase MAH1-like
TCONS_00028028	-0.642759	-0.900502	-0.775798	BGIOSGA016375	XP_003574493.1U-box domain-containing protein 41
TCONS_00036865	-0.642407	-1.23887	-3.00767	BGIOSGA002841	XP_021321650.1receptor-like protein 12
TCONS_00010258	-0.642396	-1.6093	-2.09846	BGIOSGA009164	XP_015690836.1 probable LIM domain-containing serine/threonine-protein kinase DDB_G0287001 isoform X1
TCONS_00015596	-0.642345	-0.382298	0.242089	#N/A	#N/A
TCONS_00030290	-0.642119	-0.266202	-0.892911	BGIOSGA017636	XP_006660786.1 chaperone protein dnaJ 10-like
TCONS_00022894	-0.641826	-0.239603	-1.68718	BGIOSGA009203	XP_002437580.1F-box/kelch-repeat protein SKIP25
TCONS_00023299	-0.641606	0.085081	-1.16889	BGIOSGA013758	XP_002459511.1protein YLS3
TCONS_00031676	-0.641546	-2.1905	-0.437979	BGIOSGA033495	XP_015697290.1PREDICTED: uncharacterized protein LOC107305141
TCONS_00016837	-0.641506	-0.226709	0.0050453	BGIOSGA019422	XP_006652996.1 oryzain beta chain isoform X2
TCONS_00033825	-0.640767	-0.0185411	-0.0117293	BGIOSGA030618	XP_006663591.1 pentatricopeptide repeat-containing protein At4g19890
TCONS_00002181	-0.640333	-1.21071	-0.806443	BGIOSGA018756	XP_015698995.1 coronatine-insensitive protein homolog 1a
TCONS_00026024	-0.640306	-0.0190545	-0.474837	BGIOSGA028096	XP_006659183.2 histone-lysine N-methyltransferase ATXR3
TCONS_00018325	-0.640067	-0.15187	0.777535	BGIOSGA020377	XP_015693320.1 putative rRNA methyltransferase
TCONS_00001802	-0.639717	-2.19244	1.32296	#N/A	#N/A
TCONS_00000641	-0.63948	0.617274	-0.0149018	BGIOSGA007044	XP_006645749.1 alpha-glucosidase YihQ-like

TCONS_00000504	-0.639476	-0.893312	0.184664	BGIOSGA030049	XP_015699227.1 putative ubiquitin-conjugating enzyme E2 38
TCONS_00000576	-0.639447	0.300071	-0.601016	BGIOSGA018612	XP_006643998.1 shaggy-related protein kinase alpha
TCONS_00010768	-0.639434	1.17243	-0.015014	BGIOSGA006958	XP_006650444.1 probable linoleate 9S-lipoxygenase 4
TCONS_00019120	-0.639358	-1.02072	-2.43613	BGIOSGA005536	XP_015696589.1 NAD(P)H-quinone oxidoreductase subunit L, chloroplastic
TCONS_00019091	-0.639048	-0.6082	-0.30677	BGIOSGA018278	XP_006656002.1 guanine nucleotide-binding protein alpha-1 subunit isoform X1
TCONS_00022830	-0.639022	-3.94477	-2.16592	BGIOSGA020616	XP_006656416.1PREDICTED: uncharacterized protein LOC102722587
TCONS_00029274	-0.638811	-2.02795	-2.44625	BGIOSGA026643	XP_006660787.1 chitinase-like protein 1
TCONS_00004715	-0.638799	-3.86911	-1.1469	BGIOSGA011556	XP_004970553.1aspartic proteinase PCS1
TCONS_00013329	-0.63845	0.138857	-0.306297	BGIOSGA025935	XP_015695274.1 40S ribosomal protein S18
TCONS_00003894	-0.638395	-3.92865	-0.93896	BGIOSGA034998	XP_006646046.1 enolase-phosphatase E1-like
TCONS_00012427	-0.638286	-0.109678	-0.83616	BGIOSGA026133	XP_006650179.1 protein TIFY 10a
TCONS_00025496	-0.63817	-0.174047	0.429712	BGIOSGA020888	XP_015694896.1 ninja-family protein Os07g0602900-like
TCONS_00026296	-0.638127	-0.00910724	-1.14729	#N/A	#N/A
TCONS_00019733	-0.638062	0.878284	-1.06532	BGIOSGA029249	XP_003571225.1uncharacterized protein LOC106866486
TCONS_00002570	-0.637974	-0.109651	-0.0844368	BGIOSGA022755	XP_015688317.1 uncharacterized CRM domain-containing protein At3g25440, chloroplastic
TCONS_00025718	-0.63792	0.191224	0.624985	BGIOSGA012549	XP_006658144.1 zinc finger CCCH domain-containing protein 53 isoform X1
TCONS_00036288	-0.63788	#NA	-0.885326	BGIOSGA000018	XP_008676028.1uncharacterized protein LOC103652167
TCONS_00005282	-0.637478	0.311382	-0.135073	BGIOSGA009690	XP_006646791.1 probable serine/threonine-protein kinase NAK
TCONS_00027546	-0.637241	-2.10762	-2.24468	BGIOSGA017116	XP_015696198.1 probable gamma-aminobutyrate transaminase 3, mitochondrial
TCONS_00015124	-0.637216	0.505295	-0.448931	BGIOSGA017303	XP_015691815.1 U11/U12 small nuclear ribonucleoprotein 25 kDa protein
TCONS_00009777	-0.637183	0.877175	0.602341	BGIOSGA012329	XP_015691141.1 palmitoyltransferase ZDHHC17-like isoform X1
TCONS_00020718	-0.636938	0.610906	-0.350033	BGIOSGA022914	XP_006656933.1 phosphomannomutase/phosphoglucomutase
TCONS_00005842	-0.63693	0.681004	-0.0800081	BGIOSGA033125	XP_006647162.1 casein kinase I-like
TCONS_00026087	-0.636891	-0.760623	-1.20574	BGIOSGA007605	XP_015695762.1 putative serine/threonine-protein kinase
TCONS_00029174	-0.635563	-1.23178	0.276389	BGIOSGA030922	XP_006660709.2 patatin-like protein 2
TCONS_00029657	-0.635465	-0.357913	-0.510007	BGIOSGA030163	XP_004956392.2protein RDM16
TCONS_00009798	-0.635348	-0.718695	-1.06487	BGIOSGA025240	XP_015689846.1 ankyrin repeat-containing protein At3g12360-like
TCONS_00036162	-0.635304	-0.876653	-0.0957651	BGIOSGA037518	XP_008661756.1uncharacterized LOC100277005 isoform X5
TCONS_00001783	-0.634958	-0.0940811	-0.585092	BGIOSGA004421	XP_006644674.1 protein NUCLEAR FUSION DEFECTIVE 6, chloroplastic/mitochondrial
TCONS_00000439	-0.63494	-1.31168	-1.32169	BGIOSGA012010	XP_004968636.1transcription factor bHLH30
TCONS_00031066	-0.634882	-0.217985	-0.635274	BGIOSGA037290	XP_006661759.2PREDICTED: uncharacterized protein LOC102711566, partial
TCONS_00022552	-0.634331	-2.36665	-1.32521	BGIOSGA037065	XP_004965891.1uncharacterized protein LOC101776480
TCONS_00022887	-0.634264	-0.528239	-0.408821	BGIOSGA020557	XP_015693422.1 uncharacterized zinc finger CCHC domain-containing protein At4g19190
TCONS_00020002	-0.634239	-0.194124	-0.195066	BGIOSGA025077	XP_015694053.1 cyclin-dependent kinase F-4-like
TCONS_00016064	-0.633978	-1.12832	-2.101	BGIOSGA006154	XP_006652395.1 small nuclear ribonucleoprotein Sm D1-like
TCONS_00010546	-0.633914	-0.379707	-0.58085	BGIOSGA013161	XP_015689978.1 probable E3 ubiquitin-protein ligase ARI1
TCONS_00005927	-0.633885	-1.48078	-0.657261	BGIOSGA020392	XP_021318584.1uncharacterized protein LOC8072962 isoform X4
TCONS_00009472	-0.633554	#NA	-2.78015	BGIOSGA012020	XP_015699097.1 L-galactono-1,4-lactone dehydrogenase 2, mitochondrial-like
TCONS_00004303	-0.633172	0.518981	0.218223	BGIOSGA001035	XP_014753726.1trihelix transcription factor ASIL1
TCONS_00003567	-0.633022	0.396289	-0.53147	BGIOSGA007259	XP_015699215.1 probable chromatin-remodeling complex ATPase chain
TCONS_00008278	-0.632995	-5.05185	-3.14291	BGIOSGA006148	XP_002455208.1lecithin-cholesterol acyltransferase-like 1
TCONS_00024944	-0.632949	-0.945279	0.0344368	BGIOSGA030034	XP_006660667.1 transcription initiation factor TFIID subunit 15 isoform X1
TCONS_00002796	-0.632453	-0.894621	-0.81535	BGIOSGA031094	XP_006643740.1 transcription factor DIVARICATA
TCONS_00023995	-0.632152	-0.0542588	0.160366	BGIOSGA001698	XP_004958151.1cell wall protein IFF6
TCONS_00007568	-0.632144	-0.108696	-0.557554	BGIOSGA006915	XP_015689304.1PREDICTED: uncharacterized protein LOC102711041
TCONS_00005968	-0.631458	0.590823	0.172151	BGIOSGA002716	XP_006647208.1PREDICTED: uncharacterized protein LOC102715175
TCONS_00016324	-0.631058	-1.42514	-1.65283	BGIOSGA019208	XP_006652585.1 kinesin-like protein NACK1
TCONS_00000571	-0.630989	0.0428035	-0.0124558	BGIOSGA003151	XP_006643996.2 5'-nucleotidase domain-containing protein 4
TCONS_00006364	-0.630557	-1.58286	-0.964545	BGIOSGA001469	XP_006644339.1 serine/threonine-protein phosphatase 7 long form homolog
TCONS_00023087	-0.630496	-2.35788	-1.05096	BGIOSGA010099	XP_006657432.1 switch-associated protein 70-like
TCONS_00015755	-0.630396	-0.0328974	1.29931	BGIOSGA016224	XP_008669528.1dnaJ homolog subfamily C member 2
TCONS_00013316	-0.630288	-0.751181	-0.0172473	BGIOSGA019294	XP_004955561.1ubiquitin-conjugating enzyme E2 2
TCONS_00016806	-0.630249	0.108677	-0.748378	BGIOSGA014160	NP_001136882.1putative TLD family protein
TCONS_00024330	-0.629884	-0.597307	-0.344386	BGIOSGA026371	XP_003562473.1uncharacterized protein LOC100836840 isoform X2

TCONS_00004566	-0.629684	-0.3641	-0.0617146	BGIOSGA000649	XP_012698367.1disease resistance RPP13-like protein 4 isoform X1
TCONS_00007987	-0.629582	-0.618204	-0.946788	BGIOSGA000683	XP_006648631.2 probable protein phosphatase 2C 14
TCONS_00021470	-0.628812	0.683827	0.0749332	BGIOSGA035907	XP_006655709.1 extra-large guanine nucleotide-binding protein 3-like
TCONS_00019105	-0.62796	-1.11726	-0.039957	BGIOSGA018255	XP_006664953.1 BTB/POZ domain-containing protein At2g30600
TCONS_00024066	-0.627761	-0.607248	-0.30414	BGIOSGA007371	XP_015694718.1 uncharacterized ATP-dependent helicase YprA-like
TCONS_00017839	-0.627721	-0.298673	0.103605	BGIOSGA019899	XP_015692077.1PREDICTED: uncharacterized protein LOC102715293
TCONS_00008402	-0.627434	0.160553	-1.4148	BGIOSGA031709	XP_006647531.1 protein ECERIFERUM 1-like isoform X2
TCONS_00010295	-0.627154	-1.04962	-0.94156	BGIOSGA012860	XP_006650193.1PREDICTED: uncharacterized protein LOC102721060
TCONS_00009006	-0.626609	0.0958553	-0.500784	BGIOSGA014748	NP_001168683.1putative RING zinc finger and VWF domain family protein
TCONS_00029454	-0.626421	0.669478	-0.52421	BGIOSGA029134	XP_006661502.1PREDICTED: uncharacterized protein LOC102720909
TCONS_00028314	-0.626214	-1.39341	-1.23312	BGIOSGA037060	XP_006660289.1 probable mitochondrial adenine nucleotide transporter BTL1
TCONS_00009054	-0.62616	-2.01996	-1.40883	BGIOSGA009393	XP_015689572.1 OTU domain-containing protein 5-A-like
TCONS_00014472	-0.626124	-1.75751	-0.980296	BGIOSGA016631	XP_006652432.1 glucose-6-phosphate 1-dehydrogenase, cytoplasmic isoform
TCONS_00019460	-0.62611	-0.660777	-0.526849	BGIOSGA004746	XP_006654525.1PREDICTED: uncharacterized protein At4g08330, chloroplastic-like
TCONS_00001977	-0.625905	-1.2083	0.2005	BGIOSGA028042	XP_015695712.1 succinate dehydrogenase subunit 6, mitochondrial
TCONS_00007274	-0.625698	-2.0003	-0.684235	BGIOSGA015687	NP_001142221.1uncharacterized LOC100274389
TCONS_00015223	-0.625696	-0.863784	-0.402488	BGIOSGA010605	XP_006653076.1 serine/threonine-protein kinase SAPK5
TCONS_00037817	-0.625694	-1.03976	-0.135586	BGIOSGA012775	XP_006657979.1PREDICTED: uncharacterized protein LOC102718470
TCONS_00000597	-0.625606	0.250754	0.00710117	#N/A	#N/A
TCONS_00009836	-0.624987	1.82617	0.0857137	BGIOSGA012388	XP_006649905.1 protein YIPF5 homolog
TCONS_00012876	-0.624795	-1.2097	-2.80229	BGIOSGA011128	XP_006650379.1 alpha-amylase/trypsin inhibitor-like
TCONS_00016795	-0.624754	0.220016	-0.193247	BGIOSGA014172	XP_004960168.1protein HEAT INTOLERANT 4
TCONS_00028216	-0.624733	-0.624815	-1.21749	BGIOSGA014266	XP_015695647.1 basic leucine zipper 63
TCONS_00010980	-0.624706	-0.434796	-0.491974	BGIOSGA028002	XP_003559326.1ABC transporter B family member 25
TCONS_00006809	-0.624605	-2.76501	-0.137349	BGIOSGA014401	XP_006647843.1 basic blue protein-like
TCONS_00031540	-0.624551	-2.15938	-0.99555	BGIOSGA007056	XP_006662002.1 MADS-box transcription factor 56
TCONS_00017522	-0.624491	-1.98769	-0.481126	BGIOSGA028413	XP_006659271.1 protein MEMO1 isoform X1
TCONS_00006269	-0.624184	0.229086	-0.333718	BGIOSGA031015	XP_006648743.1 auxin response factor 7
TCONS_00007570	-0.624041	-0.888725	0.00317214	BGIOSGA006913	NP_001147153.1TPR domain containing protein
TCONS_00011003	-0.624041	-0.210653	0.195817	#N/A	#N/A
TCONS_00009922	-0.623989	0.5587	0.147722	BGIOSGA013571	XP_006651316.1 phytochrome B
TCONS_00031580	-0.623981	-0.423987	-1.30684	BGIOSGA011600	XP_006662024.1 methylsterol monooxygenase 1-1-like
TCONS_00006731	-0.623905	0.367218	-1.14432	BGIOSGA023617	XP_015688710.1 protein MIZU-KUSSEI 1-like
TCONS_00010888	-0.623779	-3.25281	-1.82045	BGIOSGA034079	XP_015690053.1 serine carboxypeptidase-like 7
TCONS_00018170	-0.62365	0.500473	-0.0882322	BGIOSGA020229	XP_004958492.1photosystem I assembly factor PSA3, chloroplastic
TCONS_00031406	-0.623363	-1.70279	-0.845407	BGIOSGA033218	XP_003574135.1protein NEOXANTHIN-DEFICIENT 1
TCONS_00019886	-0.623264	-2.33673	-2.31112	BGIOSGA002089	XP_015693309.1 peroxisomal biogenesis factor 6-like
TCONS_00015213	-0.623112	0.191019	0.4744	BGIOSGA017393	XP_015691830.1PREDICTED: uncharacterized protein LOC102716213
TCONS_00002787	-0.622946	0.902883	0.511538	BGIOSGA003128	XP_004968150.1leucine-rich repeat extensin-like protein 2
TCONS_00018327	-0.622886	-0.569535	-0.207598	BGIOSGA022478	XP_006654804.1 sugar transporter ERD6-like 4
TCONS_00027940	-0.622875	-0.912423	-2.01665	#N/A	#N/A
TCONS_00034472	-0.622867	0.903338	-0.0701526	BGIOSGA021783	XP_004978936.1serine/threonine-protein kinase WNK4
TCONS_00032826	-0.622391	-0.574135	-0.308848	BGIOSGA034658	XP_015698190.1 F-box/LRR-repeat protein 3-like
TCONS_00024940	-0.622224	-2.21295	-0.108282	BGIOSGA018206	XP_006657622.1 S-adenosylmethionine carrier 1, chloroplastic/mitochondrial-like isoform X2
TCONS_00033623	-0.62219	0.371966	0.693692	BGIOSGA005045	XP_015697685.1 RNA-binding protein MEX3D-like
TCONS_00012589	-0.621465	0.0118327	-1.73339	BGIOSGA016324	XP_006650266.1 probable potassium transporter 16
TCONS_00036433	-0.621381	-0.834798	-0.416025	BGIOSGA022276	XP_004963204.1pyruvate dehydrogenase E1 component subunit beta-3, chloroplastic isoform X2
TCONS_00021634	-0.621361	0.785957	0.511056	BGIOSGA004652	XP_024312594.1uncharacterized protein LOC100836720 isoform X3
TCONS_00015936	-0.62128	1.03378	0.773889	BGIOSGA015047	XP_015691465.1 zinc finger CCCH domain-containing protein 28
TCONS_00018039	-0.621175	-0.711776	-0.396297	BGIOSGA009619	XP_006654567.1 probable prolyl 4-hydroxylase 4
TCONS_00011542	-0.621099	0.91491	0.0953663	BGIOSGA016059	XP_006649382.1 cysteine-rich repeat secretory protein 3-like
TCONS_00023377	-0.62105	-0.44725	-0.506828	BGIOSGA030496	XP_006660481.1 cyclin-P4-1-like
TCONS_00036242	-0.620912	-0.612708	0.0430128	BGIOSGA003042	XP_006664105.1 probable Xaa-Pro aminopeptidase 3 isoform X1
TCONS_00028210	-0.62088	-4.21465	-0.812068	BGIOSGA022244	XP_003572314.1protein EXORDIUM-like 2
TCONS_00026062	-0.620505	-4.21512	-1.813	BGIOSGA020680	XP_006659203.2 solanesyl-diphosphate synthase 1, mitochondrial-like isoform X2

TCONS_00028976	-0.620176	0.0827455	-0.431222	BGIOSGA030734	XP_006661210.1PREDICTED: uncharacterized protein LOC102722702, partial
TCONS_00007113	-0.619861	-0.30105	-0.249814	BGIOSGA021936	XP_006648118.1 probable voltage-gated potassium channel subunit beta
TCONS_00001611	-0.6198	0.511297	-0.22717	BGIOSGA017621	XP_006644547.1 protein PLASTID MOVEMENT IMPAIRED 2-like
TCONS_00005283	-0.619582	-1.47932	-1.5929	BGIOSGA007397	XP_006648238.1 sialyltransferase-like protein 4
TCONS_00032452	-0.619426	-0.80709	0.488105	BGIOSGA016006	XP_006661874.1 probable N-succinyldiaminopimelate aminotransferase DapC isoform X1
TCONS_00019748	-0.619361	-1.82779	-0.767515	BGIOSGA004232	XP_015692776.1PREDICTED: uncharacterized protein LOC102722302
TCONS_00007828	-0.61928	-0.172271	-0.0611501	BGIOSGA024903	XP_015688560.1PREDICTED: uncharacterized protein LOC102718991, partial
TCONS_00010713	-0.619231	-0.000346168	-0.710404	BGIOSGA012630	XP_006651689.1 protein SABRE
TCONS_00013782	-0.618891	-0.0472288	-0.817	BGIOSGA030682	XP_003581017.1UDP-glycosyltransferase 79 isoform X1
TCONS_00026157	-0.618628	-3.43988	-4.625	BGIOSGA027415	XP_006659227.1 uncharacterized membrane protein At1g16860-like
TCONS_00008970	-0.618579	-0.663811	-0.642609	BGIOSGA005434	XP_006649134.1 probable serine/threonine-protein kinase vps15
TCONS_00011348	-0.618428	-0.171094	0.150743	BGIOSGA013959	XP_015690750.1 pantoate--beta-alanine ligase
TCONS_00017454	-0.618125	-1.21812	0.596144	BGIOSGA019529	XP_015692441.1PREDICTED: uncharacterized protein LOC102721450
TCONS_00035704	-0.618089	-0.487768	-0.312628	BGIOSGA010698	XP_006664409.2 probable thiamine biosynthetic bifunctional enzyme, chloroplastic
TCONS_00022858	-0.617791	0.56211	0.0199797	BGIOSGA020586	XP_006656445.1PREDICTED: uncharacterized protein LOC102706948
TCONS_00024576	-0.617746	-0.472249	-1.1243	BGIOSGA024816	XP_006657447.1 CSC1-like protein ERD4
TCONS_00016935	-0.617373	-0.826742	-0.820747	BGIOSGA008099	XP_003576973.2protein CHROMATIN REMODELING 24 isoform X2
TCONS_00024750	-0.616973	-0.307729	-0.153292	BGIOSGA008206	XP_006657549.1 40S ribosomal protein S15a-1-like
TCONS_00013815	-0.616792	-0.870639	-1.85694	BGIOSGA026351	XP_006653215.1 G-type lectin S-receptor-like serine/threonine-protein kinase B120
TCONS_00021984	-0.616765	0.337127	-0.435221	BGIOSGA030466	XP_006656846.2 potassium channel KOR1
TCONS_00006164	-0.616744	0.146387	-1.22333	BGIOSGA021793	XP_006647247.1 acyl-coenzyme A thioesterase 13-like
TCONS_00029083	-0.616703	-0.344301	-0.90567	BGIOSGA033427	XP_015696475.1 probable ethanolamine kinase
TCONS_00018279	-0.616669	-0.963245	-0.690942	BGIOSGA020333	XP_006655576.2PREDICTED: uncharacterized protein LOC102702382 isoform X1
TCONS_00013872	-0.616323	-0.383874	-0.249864	BGIOSGA016052	XP_015691390.1PREDICTED: uncharacterized protein LOC102716587, partial
TCONS_00018227	-0.615096	-1.29928	-1.11535	BGIOSGA008655	XP_015692680.1 probable cytokinin riboside 5'-monophosphate phosphoribohydrolase LOGL7
TCONS_00019454	-0.615032	-1.64768	-2.16663	#N/A	#N/A
TCONS_00000060	-0.614852	-0.427418	0.904459	BGIOSGA030366	XP_006643659.2 acetyl-CoA acetyltransferase, cytosolic 1-like
TCONS_00031289	-0.614757	-0.116448	0.136769	BGIOSGA033102	XP_015697017.1 eukaryotic translation initiation factor isoform 4E-2
TCONS_00029292	-0.614559	-0.347683	-0.0398383	BGIOSGA029026	XP_006660801.1 60S ribosomal protein L32-1
TCONS_00003282	-0.614287	-1.83286	-0.221753	BGIOSGA001930	XP_006644032.1 uncharacterized isomerase BH0283-like
TCONS_00004673	-0.613904	-0.752138	-0.684334	BGIOSGA005376	XP_015699056.1 protein IQ-DOMAIN 1-like
TCONS_00013565	-0.613902	-0.741368	-0.148679	BGIOSGA031332	XP_015690833.1 probable inactive poly [ADP-ribose] polymerase SRO1
TCONS_00030323	-0.613894	-0.248341	-0.315855	BGIOSGA029485	XP_006660807.1 E3 ubiquitin-protein ligase RNF4-like
TCONS_00011498	-0.613603	-1.07549	-0.673661	BGIOSGA030712	XP_006649327.2PREDICTED: uncharacterized protein At4g15970-like
TCONS_00029568	-0.613537	-0.195823	0.0406503	BGIOSGA030250	XP_006660411.1 pre-mRNA-splicing factor 38
TCONS_00014150	-0.613437	0.0768054	-0.282357	BGIOSGA031064	XP_006652222.1PREDICTED: uncharacterized protein At2g33490-like isoform X1
TCONS_00037452	-0.613419	-0.363357	-0.446727	BGIOSGA036542	XP_006664699.1 extra-large guanine nucleotide-binding protein 1
TCONS_00035283	-0.613251	-2.30031	-0.899407	BGIOSGA027379	XP_015698144.1 kinesin-like protein KIFC3 isoform X2
TCONS_00016555	-0.612813	-1.04962	-1.20005	BGIOSGA005783	XP_004976700.1trihelix transcription factor GT-3b
TCONS_00011366	-0.61281	-0.640224	-0.112341	BGIOSGA013980	XP_006650927.1 ATP-dependent DNA helicase 2 subunit KU80
TCONS_00004787	-0.612691	-0.975307	-0.644966	BGIOSGA006726	XP_006665041.1 protein LIKE COV 2-like
TCONS_00027973	-0.61255	-0.448456	-0.794803	BGIOSGA027022	XP_022683438.1uncharacterized protein LOC101777506
TCONS_00016642	-0.612128	-0.520461	-0.759029	BGIOSGA014319	XP_006652847.1 probable polyamine oxidase 2
TCONS_00023367	-0.612042	0.911896	0.424833	BGIOSGA037532	XP_024313333.1uncharacterized protein LOC100833961
TCONS_00017029	-0.611961	0.216729	-0.0719591	BGIOSGA011648	XP_003566634.1calcium-transporting ATPase 4, endoplasmic reticulum-type
TCONS_00008585	-0.611779	-0.730253	-1.39264	BGIOSGA014398	XP_006647688.2 protein REVEILLE 6 isoform X1
TCONS_00011753	-0.611346	-0.0488746	0.18736	BGIOSGA011252	XP_006649591.1 activating signal cointegrator 1 complex subunit 2
TCONS_00008406	-0.61094	-2.03545	-0.72482	BGIOSGA006016	XP_006647535.1 putative phosphatidylinositol N-acetylglucosaminyltransferase subunit C
TCONS_00019706	-0.610492	-1.54064	-0.464912	BGIOSGA017654	XP_015693273.1 beta-galactosidase 8 isoform X1
TCONS_00021451	-0.610303	-0.366669	-1.19389	BGIOSGA020708	XP_006655684.1 protein REVEILLE 6-like
TCONS_00023170	-0.610271	-2.70771	-1.2458	BGIOSGA019255	XP_015695093.1 probable inactive leucine-rich repeat receptor-like protein kinase At3g03770
TCONS_00006051	-0.610249	0.382842	-0.183648	BGIOSGA016317	XP_003574974.140S ribosomal protein S27
TCONS_00016301	-0.609917	-0.748284	0.705812	BGIOSGA014668	XP_006652553.1 vicilin-like seed storage protein At2g18540
TCONS_00029545	-0.609506	-0.083918	-0.813619	BGIOSGA013642	XP_006661002.2 U-box domain-containing protein 33-like isoform X1

TCONS_00022698	-0.609495	-0.928753	0.0371217	BGIOSGA014389	XP_006656330.1PREDICTED: uncharacterized protein LOC102722867
TCONS_00004576	-0.609359	-0.53124	-0.453743	BGIOSGA025216	XP_006644858.1 probable sugar phosphate/phosphate translocator At2g25520
TCONS_00002553	-0.609224	-0.959989	-0.363621	BGIOSGA037096	XP_006646693.1 protein CLT1, chloroplastic
TCONS_00009296	-0.608739	-0.795683	-0.947397	BGIOSGA015572	XP_006651027.2 ABC transporter C family member 13 isoform X1
TCONS_00023867	-0.608356	0.775439	1.14627	BGIOSGA027683	XP_004957941.1probable histone H2A.1
TCONS_00030549	-0.608244	-0.203458	-0.301168	BGIOSGA029254	XP_003578662.1myosin heavy chain, embryonic smooth muscle isoform
TCONS_00018610	-0.60823	-0.547016	-0.0992803	BGIOSGA018824	XP_006654985.1 putative membrane-bound O-acyltransferase C24H6.01c isoform X1
TCONS_00012380	-0.608069	0.241354	-0.432793	BGIOSGA026165	XP_006650153.1 40S ribosomal protein S6
TCONS_00006066	-0.607918	-1.86934	-0.661931	BGIOSGA026802	XP_015688595.1 phenolic glucoside malonyltransferase 1-like
TCONS_00026857	-0.607697	1.02436	0.715764	BGIOSGA022245	XP_010235410.1uncharacterized protein LOC100835952 isoform X2
TCONS_00011341	-0.607404	-1.50372	-0.320497	BGIOSGA000207	XP_015690804.1 aspartokinase 1, chloroplastic isoform X1
TCONS_00013582	-0.606856	-0.873364	0.0875974	BGIOSGA005350	NP_001148776.1cysteine-type peptidase
TCONS_00006989	-0.60684	-0.265387	0.146728	BGIOSGA005913	XP_004954166.1putative lipase C4A8.10 isoform X1
TCONS_00014767	-0.606826	0.798844	-1.24634	BGIOSGA023035	XP_015692284.1 RHOMBOID-like protein 2 isoform X2
TCONS_00020424	-0.606702	-0.755386	-1.15036	BGIOSGA025349	XP_008659068.1O-fucosyltransferase 15
TCONS_00009518	-0.606674	0.630548	0.0867393	BGIOSGA012066	XP_015689807.1PREDICTED: uncharacterized protein LOC102709629
TCONS_00008555	-0.606423	-1.40812	-0.899415	BGIOSGA020820	XP_006647662.1 GDSL esterase/lipase At5g55050-like
TCONS_00001736	-0.606142	-0.245962	0.34424	BGIOSGA004441	XP_006644644.1 NADP-dependent malic enzyme isoform X2
TCONS_00018055	-0.606018	0.137471	-0.439764	BGIOSGA012874	XP_015693065.1 transcription elongation factor SPT6 isoform X1
TCONS_00037885	-0.605819	-2.66195	#NA	#N/A	#N/A
TCONS_00023036	-0.605625	0.864752	-0.972439	BGIOSGA015758	XP_006657411.1PREDICTED: uncharacterized protein LOC102721288 isoform X1
TCONS_00011250	-0.604909	-1.3031	-0.251087	BGIOSGA032613	XP_015690130.1 DIMBOA UDP-glucosyltransferase BX8-like
TCONS_00022856	-0.60488	0.418283	-0.364817	BGIOSGA004077	XP_008228256.1 eukaryotic initiation factor 4A-11
TCONS_00015799	-0.604744	-0.096819	-0.0265702	BGIOSGA018793	XP_006652225.1 transport inhibitor response 1-like protein Os04g0395600
TCONS_00014145	-0.604582	-1.65144	-1.71133	BGIOSGA022703	XP_015692379.1 serine/threonine-protein kinase CDL1-like
TCONS_00031226	-0.604016	-0.654625	-0.46976	BGIOSGA011444	XP_003573986.1uncharacterized protein LOC100844091 isoform X1
TCONS_00000252	-0.603755	-1.63242	-1.11702	BGIOSGA003419	XP_003568700.3serine carboxypeptidase II-1
TCONS_00006785	-0.603537	-0.0898146	-0.584624	BGIOSGA031224	XP_015696862.1 25S rRNA (cytosine-C(5))-methyltransferase nop2 isoform X2
TCONS_00024378	-0.603503	-1.11222	-0.71399	BGIOSGA010840	XP_004958758.1nitrate regulatory gene2 protein isoform X1
TCONS_00003489	-0.603452	-2.24212	-0.669196	BGIOSGA008185	XP_015697242.1 cleavage and polyadenylation specificity factor subunit 6-like isoform X3
TCONS_00019544	-0.603399	-3.97725	0.0261742	BGIOSGA017814	XP_006655436.2 probable calcium-binding protein CML9
TCONS_00027327	-0.603397	-0.246557	-0.342255	#N/A	#N/A
TCONS_00013527	-0.603305	-0.0286217	-0.405847	BGIOSGA036472	XP_012704142.1pirin-like protein isoform X4
TCONS_00018315	-0.602952	0.433449	0.0692018	BGIOSGA000819	XP_006654794.1 60S ribosomal protein L18a-like
TCONS_00003130	-0.602841	-0.429842	-0.396437	BGIOSGA002094	XP_015699137.1PREDICTED: uncharacterized protein LOC102718414, partial
TCONS_00020129	-0.602764	-0.258029	-0.372674	BGIOSGA027765	XP_006655791.1 shikimate O-hydroxycinnamoyltransferase-like
TCONS_00024072	-0.602588	-1.13957	-0.913869	BGIOSGA016408	XP_006658796.1PREDICTED: uncharacterized protein LOC102703598
TCONS_00016704	-0.602107	-0.951327	-1.296	BGIOSGA022286	XP_006653807.2 agmatine coumaroyltransferase-like
TCONS_00009793	-0.602081	-0.690276	-1.61372	BGIOSGA012350	XP_006649867.1 nitric oxide synthase-interacting protein
TCONS_00006621	-0.602053	-1.14054	-1.0886	BGIOSGA022749	XP_022678981.1uncharacterized protein LOC101778835
TCONS_00035542	-0.602012	0.710901	1.40413	BGIOSGA005135	XP_002442692.1probable calcium-transporting ATPase 9, plasma membrane-type
TCONS_00025523	-0.601877	0.38549	0.225354	BGIOSGA007652	XP_002453367.160S ribosomal protein L27a-2
TCONS_00030190	-0.601869	-1.23406	-1.11036	BGIOSGA029612	NP_001131978.1Purine permease 3
TCONS_00016740	-0.601527	-1.41817	-0.159519	BGIOSGA034432	XP_003580694.1ethylene-responsive transcription factor RAP2-1
TCONS_00024791	-0.601171	-2.65629	-0.254752	BGIOSGA009570	XP_006657572.1 transcription elongation factor TFIIS-like
TCONS_00014205	-0.600997	-1.19115	-1.05963	BGIOSGA029643	XP_015691820.1 alpha carbonic anhydrase 1, chloroplastic-like
TCONS_00010135	-0.600942	-0.145363	-0.236276	BGIOSGA009416	XP_006650115.1 peptidyl-prolyl cis-trans isomerase PASTICCINO1
TCONS_00035168	-0.600708	-0.130016	-0.735641	BGIOSGA007976	XP_006663042.1 probable LRR receptor-like serine/threonine-protein kinase At5g10290
TCONS_00022470	-0.60067	-0.174489	-0.192452	BGIOSGA032430	XP_015694334.1 protein NRT1/ PTR FAMILY 4.6-like isoform X3
TCONS_00028489	-0.600642	0.560898	0.204535	BGIOSGA026468	XP_006660397.2 mediator of RNA polymerase II transcription subunit 15a
TCONS_00011735	-0.600397	-0.797206	-0.521953	BGIOSGA020777	XP_021309013.1methyltransferase-like protein 2
TCONS_00014887	-0.600259	-0.698938	-0.274908	BGIOSGA013733	XP_015691793.1 probable WRKY transcription factor 17
TCONS_00019941	-0.60018	-0.10066	-0.399479	BGIOSGA022125	XP_015694285.1 U-box domain-containing protein 12 isoform X1

TCONS_00027764	-0.600149	-1.20358	-0.654197	BGIOSGA017150	XP_014756325.2putative disease resistance protein RGA3
TCONS_00001038	-0.600002	-0.775095	-0.887718	BGIOSGA003638	XP_006644230.1 probable protein Pop3
TCONS_00010404	-0.599632	-0.336301	0.385295	BGIOSGA012993	NP_001151230.2DTW domain containing protein
TCONS_00012827	-0.599585	0.832234	-0.386649	BGIOSGA026297	XP_015690423.1 nuclear transcription factor Y subunit A-10-like
TCONS_00005424	-0.599386	0.745697	0.0402743	BGIOSGA000187	XP_015689456.1 26S proteasome non-ATPase regulatory subunit 2 homolog A-like
TCONS_00012961	-0.599321	1.13604	-0.620155	#N/A	#N/A
TCONS_00003970	-0.599211	-1.03778	-1.63346	BGIOSGA001220	XP_015688304.1 DNA (cytosine-5)-methyltransferase isoform X2
TCONS_00031079	-0.599163	0.67544	-0.239171	BGIOSGA034501	XP_002436652.140S ribosomal protein S17-3
TCONS_00016341	-0.599109	-1.20211	0.0234799	BGIOSGA007430	XP_006653632.1 histone-lysine N-methyltransferase, H3 lysine-9 specific SUVH5-like
TCONS_00005706	-0.59891	-0.334956	-0.206764	BGIOSGA021041	XP_015689421.1 nuclear poly(A) polymerase 4-like isoform X1
TCONS_00002324	-0.598813	-1.39767	-0.482952	BGIOSGA005848	XP_015688924.1PREDICTED: LOW QUALITY PROTEIN: uncharacterized protein LOC102711226
TCONS_00028574	-0.597659	-0.606777	-0.204365	BGIOSGA005936	XP_004966549.1leucine-rich repeat protein 1
TCONS_00036506	-0.597405	-0.929639	-0.108197	BGIOSGA013365	XP_006664791.2 HIPL1 protein
TCONS_00029989	-0.597299	-0.762267	-0.177525	BGIOSGA029833	XP_006648810.1PREDICTED: uncharacterized protein LOC102722839
TCONS_00031664	-0.597296	-0.893666	-0.0117123	BGIOSGA000482	XP_006662620.1 pyruvate kinase isozyme G, chloroplastic-like
TCONS_00030254	-0.596639	0.138354	-0.403378	BGIOSGA030621	XP_002462562.1uncharacterized protein LOC8064843
TCONS_00000562	-0.596235	-2.98581	-2.57691	BGIOSGA030479	XP_010230692.1uncharacterized protein LOC104582533
TCONS_00030578	-0.596113	1.45642	0.0666743	BGIOSGA020093	XP_006661587.1 G-type lectin S-receptor-like serine/threonine-protein kinase At5g24080
TCONS_00027655	-0.596006	0.598863	-0.577489	BGIOSGA009769	XP_002455932.1ATP synthase subunit epsilon, mitochondrial
TCONS_00031698	-0.595833	0.230636	-0.00346171	BGIOSGA040471	XP_006662126.1 serine carboxypeptidase-like 6
TCONS_00008134	-0.595602	0.744272	0.461916	BGIOSGA036595	XP_015688813.1 transcription factor E2FA-like
TCONS_00016311	-0.595552	-0.12696	-1.01553	BGIOSGA028590	XP_014751505.1probable ascorbate-specific transmembrane electron transporter 2
TCONS_00028137	-0.595477	-1.3747	-0.84715	BGIOSGA032818	XP_003574599.1homeobox-leucine zipper protein HOX27
TCONS_00004758	-0.595475	-0.169981	-0.941498	BGIOSGA017011	XP_006646494.1PREDICTED: uncharacterized protein LOC102720109, partial
TCONS_00014121	-0.595387	0.350254	-0.0139631	BGIOSGA012970	XP_006652213.1PREDICTED: uncharacterized protein LOC102711249
TCONS_00006305	-0.595207	0.158813	-2.15797	BGIOSGA030097	XP_006647334.1 ent-cassadiene C2-hydroxylase-like
TCONS_00032113	-0.595113	-0.40931	0.0486505	BGIOSGA036736	XP_006659222.1 ethanolamine-phosphate cytidylyltransferase-like
TCONS_00007561	-0.595039	-0.382307	-0.428446	BGIOSGA016487	XP_015688500.1 nuclear pore complex protein NUP205
TCONS_00020144	-0.595002	-0.443304	0.395335	BGIOSGA022342	XP_015694352.1 protein IQ-DOMAIN 1
TCONS_00015247	-0.594929	0.147274	-1.59489	BGIOSGA015734	XP_015691322.1 UNC93-like protein 1 isoform X1
TCONS_00000038	-0.594665	-3.68389	-4.97231	BGIOSGA009581	XP_006648169.2PREDICTED: uncharacterized protein LOC102710760
TCONS_00019026	-0.59433	-1.25679	-1.08394	BGIOSGA026572	XP_006654247.1 pentatricopeptide repeat-containing protein At5g16860
TCONS_00008375	-0.59424	-0.147026	0.022938	BGIOSGA036622	XP_006647513.1 UDP-galactose transporter 2-like
TCONS_00021401	-0.594135	-1.66653	-1.99722	BGIOSGA024325	XP_006657358.1 expansin-like A4
TCONS_00014548	-0.593891	-1.06964	-0.979174	BGIOSGA009838	XP_003580080.1magnesium transporter MRS2-C
TCONS_00014651	-0.593785	0.751817	-0.66272	BGIOSGA017371	XP_006652558.1 elicitor-responsive protein 3
TCONS_00019306	-0.593691	0.368527	0.0605571	BGIOSGA018039	XP_006654413.2 uncharacterized hydrolase YugF
TCONS_00016697	-0.593596	-0.15004	-0.171276	#N/A	#N/A
TCONS_00014638	-0.593435	-0.375492	-0.958376	BGIOSGA016795	XP_006652541.1PREDICTED: uncharacterized protein LOC102715104
TCONS_00008994	-0.592792	0.213646	-0.515143	BGIOSGA005413	XP_010673301.1 40S ribosomal protein S30
TCONS_00006630	-0.592738	-0.0422335	-0.167463	BGIOSGA024373	XP_008790519.1 ORM1-like protein 3
TCONS_00029017	-0.592729	0.46919	0.87373	BGIOSGA002553	XP_006660631.2 probable E3 ubiquitin-protein ligase RNF144A-B
TCONS_00019912	-0.592598	-0.571426	-1.2507	BGIOSGA013688	XP_006654874.1 translation initiation factor IF-2-like
TCONS_00001721	-0.592559	-1.49114	-0.837642	BGIOSGA034856	XP_022682212.1putative disease resistance protein RGA3 isoform X2
TCONS_00005614	-0.592403	-1.38419	-0.722121	BGIOSGA007727	XP_006648418.1PREDICTED: uncharacterized protein LOC102717774
TCONS_00016540	-0.592301	-0.88393	-4.43997	BGIOSGA021829	XP_024312090.1wall-associated receptor kinase 2
TCONS_00024746	-0.592256	0.750675	-0.0289251	BGIOSGA022708	XP_010228237.1uncharacterized protein LOC100842709
TCONS_00028242	-0.592064	-1.00101	-1.83728	BGIOSGA037477	XP_006659540.1 tricin synthase 1
TCONS_00006531	-0.591428	-2.40692	-0.341014	BGIOSGA035676	XP_015689021.1 myb-related protein 308
TCONS_00021421	-0.591326	-0.117543	-0.446236	BGIOSGA023647	XP_003563221.1lysM and putative peptidoglycan-binding domain-containing protein 1
TCONS_00017585	-0.591178	1.15967	0.81556	BGIOSGA029757	XP_006655211.1 probable WRKY transcription factor 26
TCONS_00014344	-0.591118	-0.501022	0.511053	BGIOSGA010409	XP_003579863.1polyadenylate-binding protein RBP47
TCONS_00023971	-0.590656	-2.62362	-1.81827	BGIOSGA025235	XP_006657880.1 glutamic acid-rich protein-like
TCONS_00005650	-0.590483	-0.260572	-0.544896	BGIOSGA020942	XP_006648446.1 death-inducer obliterator 1-like

TCONS_00030344	-0.590481	0.136635	-0.247144	BGIOSGA026575	XP_006660829.1 ADP-ribosylation factor GTPase-activating protein AGD3-like
TCONS_00018323	-0.590374	-0.557056	-0.11129	BGIOSGA019151	XP_006654801.2 aspartic proteinase oryzasin-1 isoform X1
TCONS_00000311	-0.589777	-0.135984	-0.133609	BGIOSGA029989	XP_006643827.1 E3 ubiquitin-protein ligase At1g12760-like
TCONS_00011335	-0.589734	-2.99402	-1.59315	BGIOSGA028365	XP_015690473.1PREDICTED: uncharacterized protein At4g15970-like
TCONS_00019891	-0.589558	0.00576033	-1.78623	BGIOSGA021766	XP_006655641.2 transcription factor FAMA
TCONS_00009588	-0.589322	-0.527897	-0.952737	BGIOSGA012967	XP_006649651.1 G-type lectin S-receptor-like serine/threonine-protein kinase At2g19130
TCONS_00035654	-0.589204	-0.101968	0.084385	BGIOSGA003782	NP_001151680.1protein transport protein Sec61 beta subunit
TCONS_00025819	-0.589202	0.441529	-0.448985	BGIOSGA019946	XP_006659065.1 60S acidic ribosomal protein P1-like
TCONS_00023781	-0.589018	-1.99492	-1.13549	BGIOSGA025820	XP_006657767.1 cytochrome P450 716B1-like
TCONS_00025784	-0.588881	-0.747164	-0.385804	BGIOSGA004006	XP_002443718.2probable polygalacturonase
TCONS_00004123	-0.588788	-2.18785	-4.59549	BGIOSGA003388	XP_006644477.1 probable receptor-like protein kinase At2g42960
TCONS_00027884	-0.5887	1.98196	-2.27378	BGIOSGA033064	XP_006659329.1PREDICTED: uncharacterized protein LOC102706867 isoform X2
TCONS_00009502	-0.588281	-0.452563	-0.360254	BGIOSGA021038	XP_006649568.1 deSI-like protein At4g17486
TCONS_00037460	-0.587927	-0.848958	-0.730948	BGIOSGA035899	XP_015698672.1 plastidial lipoyltransferase 2-like
TCONS_00017776	-0.587601	-1.23941	0.226397	BGIOSGA019841	XP_004962146.1uncharacterized protein LOC101767296
TCONS_00008432	-0.587554	-1.27017	-0.716074	BGIOSGA014691	XP_015689587.1PREDICTED: uncharacterized protein LOC102721239
TCONS_00011840	-0.587489	0.367734	-0.0379774	BGIOSGA000262	XP_015690251.1 putative F-box protein At1g23770
TCONS_00019980	-0.587275	2.06785	0.465862	BGIOSGA022167	XP_006655711.1 6-phosphogluconate dehydrogenase, decarboxylating 1
TCONS_00002868	-0.587108	-0.242429	0.487832	BGIOSGA002351	XP_006643776.1 NF-X1-type zinc finger protein NFXL2
TCONS_00011286	-0.586818	-0.056068	-0.506366	BGIOSGA022466	XP_015690779.1 rab9 effector protein with kelch motifs
TCONS_00015122	-0.586687	-0.900866	-0.0349134	BGIOSGA017301	XP_015692090.1 protein RCC2 homolog
TCONS_00002006	-0.58631	-1.74323	-3.48666	BGIOSGA011824	XP_006644863.2 cytochrome P450 94B3-like
TCONS_00034402	-0.586194	-0.158683	-0.0556814	BGIOSGA009191	XP_003577782.1protein TPLATE
TCONS_00025394	-0.584885	-4.11161	-2.03471	BGIOSGA025397	XP_004958113.1beta-hexosaminidase 2
TCONS_00022057	-0.584702	-4.76663	-1.66533	BGIOSGA025988	XP_006654929.1PREDICTED: uncharacterized protein LOC102716972
TCONS_00002290	-0.584153	0.972784	-0.186049	BGIOSGA020176	XP_006645129.1 probable magnesium transporter NIPA4
TCONS_00039483	-0.584115	-1.35829	0.346936	BGIOSGA012955	XP_024318541.1uncharacterized protein LOC100837437
TCONS_00012975	-0.58406	-1.47263	-0.982028	BGIOSGA014167	XP_015690025.1 1,4-dihydroxy-2-naphthoyl-CoA thioesterase 1
TCONS_00032583	-0.583807	-1.7265	-0.603006	BGIOSGA015928	XP_009400717.1 probable methyltransferase PMT15
TCONS_00026908	-0.58375	0.15088	-0.412828	BGIOSGA019606	XP_004973867.1transcription factor bHLH130 isoform X1
TCONS_00016469	-0.583474	-1.68329	-0.349105	BGIOSGA011442	XP_006652689.1 E3 ubiquitin-protein ligase EL5-like
TCONS_00030740	-0.58309	-0.742914	-0.00707942	BGIOSGA003756	XP_010233742.1putative F-box protein At3g16210
TCONS_00033591	-0.583057	-0.248635	-0.291564	BGIOSGA026573	XP_006663493.1 retinoblastoma-related protein 2 isoform X1
TCONS_00006128	-0.582934	-1.15885	-1.11785	BGIOSGA005079	XP_003575013.1uncharacterized protein LOC100827089
TCONS_00011902	-0.582841	-2.63273	-0.185785	BGIOSGA007687	XP_015690356.1 geraniol 8-hydroxylase-like
TCONS_00026973	-0.582763	0.0331809	-0.514795	BGIOSGA029496	XP_015695699.1 60S ribosomal protein L32-1-like
TCONS_00002126	-0.582725	1.51699	0.339164	BGIOSGA030813	XP_010504143.1 histone H4
TCONS_00030519	-0.582318	0.432991	-0.960083	BGIOSGA029273	XP_021309842.1probable anion transporter 5, chloroplastic
TCONS_00014747	-0.582281	-0.712905	0.26222	BGIOSGA016923	XP_021307322.1probable F-box protein At4g22165 isoform X3
TCONS_00039100	-0.582179	-1.4557	-0.554138	BGIOSGA032669	XP_006657081.2 probable LRR receptor-like serine/threonine-protein kinase At3g47570
TCONS_00011448	-0.582176	-4.34592	-1.62144	BGIOSGA022177	XP_015691075.1 short-chain dehydrogenase TIC 32, chloroplastic
TCONS_00027452	-0.582159	-0.921212	-0.685068	BGIOSGA017210	XP_015696148.1 DNA polymerase I A, chloroplastic-like
TCONS_00019910	-0.581618	-0.482752	-0.662777	BGIOSGA017659	XP_006654873.1 probable protein phosphatase 2C 53
TCONS_00037579	-0.58137	-0.372088	#NA	#N/A	#N/A
TCONS_00000371	-0.580927	-1.26205	-0.831269	BGIOSGA002947	XP_006643874.1PREDICTED: uncharacterized protein LOC102703429
TCONS_00031695	-0.580614	-0.556319	-0.238173	BGIOSGA036001	XP_015697148.1 protein TIC 62, chloroplastic
TCONS_00017521	-0.580542	-0.728959	-0.519022	BGIOSGA019592	XP_015698993.1 cathepsin B-like
TCONS_00037580	-0.580532	0.0336538	1.21829	BGIOSGA035780	XP_015698375.1 pyridine nucleotide-disulfide oxidoreductase domain-containing protein 2
TCONS_00019298	-0.580478	1.29407	-0.148437	BGIOSGA028172	XP_015693342.1 probably inactive leucine-rich repeat receptor-like protein kinase At5g48380
TCONS_00023472	-0.580353	0.859367	0.520492	BGIOSGA025621	XP_021308380.1putative disease resistance RPP13-like protein 1
TCONS_00000046	-0.579699	-1.61207	-0.405187	BGIOSGA002613	XP_015692925.1 probable glycosyltransferase At3g07620 isoform X3
TCONS_00016727	-0.579537	-0.652943	-0.410503	BGIOSGA014234	XP_015691332.1PREDICTED: uncharacterized protein LOC102708241 isoform X1
TCONS_00002432	-0.579339	-1.11214	-0.630599	BGIOSGA005074	XP_010234976.1exocyst complex component EXO70E2
TCONS_00002678	-0.578829	-0.273065	-0.122722	BGIOSGA002543	XP_015688096.1 protein EXECUTER 2, chloroplastic

TCONS_00007618	-0.578706	-0.217219	-0.30646	BGIOSGA023119	XP_006648462.1 putative GATA transcription factor 22
TCONS_00006357	-0.578559	-0.180546	0.270073	BGIOSGA036238	XP_002452366.1glycerophosphodiester phosphodiesterase GDPDL3
TCONS_00027024	-0.578482	-0.613495	-0.363465	BGIOSGA029313	XP_015695685.1 chitin elicitor receptor kinase 1-like
TCONS_00003781	-0.578428	-0.410211	-0.730678	BGIOSGA031397	XP_006663383.1 F-box/LRR-repeat protein 4
TCONS_00022559	-0.578228	-1.44956	-1.92757	BGIOSGA027024	XP_015693849.1 transcription factor TGA2-like isoform X2
TCONS_00025661	-0.578175	0.0689121	-0.591873	BGIOSGA033777	XP_006658929.2 tyrosine-specific transport protein-like
TCONS_00014484	-0.578139	-0.309105	0.430873	BGIOSGA008570	XP_006652438.1 cyclin-dependent kinase G-2 isoform X1
TCONS_00004644	-0.578107	-3.49596	-0.772614	BGIOSGA017723	XP_006644925.1 transcription repressor OFP1-like
TCONS_00016944	-0.577856	-0.987703	-0.667598	BGIOSGA002547	XP_006654891.1 peroxisomal membrane protein PEX14-like isoform X2
TCONS_00027013	-0.577414	-0.260032	-0.749867	BGIOSGA033343	XP_006659635.1 NAC domain-containing protein 83-like
TCONS_00004679	-0.577405	-1.45633	0.193262	BGIOSGA000536	XP_003564555.1dirigent protein 17
TCONS_00017822	-0.577177	1.54925	1.06239	BGIOSGA012061	XP_003568440.1uncharacterized protein LOC100831239
TCONS_00021943	-0.577102	-0.50671	-0.0904909	BGIOSGA035284	XP_006656826.1 mucin-5AC-like
TCONS_00001247	-0.576937	-1.31941	-0.378811	BGIOSGA000526	XP_006646056.1 serine/threonine-protein kinase AFC3
TCONS_00036502	-0.576885	-0.797055	-0.290212	BGIOSGA037871	XP_015698383.1PREDICTED: uncharacterized protein YtfP, partial
TCONS_00018740	-0.576752	-1.07398	0.735371	BGIOSGA018677	XP_015692751.1PREDICTED: uncharacterized protein LOC102713629 isoform X2
TCONS_00023467	-0.576734	-3.17576	-1.77601	BGIOSGA025501	XP_015694596.1 LRR receptor-like serine/threonine-protein kinase ERECTA
TCONS_00015474	-0.576638	-0.451336	-1.45352	BGIOSGA012604	XP_006665010.1PREDICTED: uncharacterized protein LOC102710368
TCONS_00000332	-0.576131	-0.203347	0.0542817	BGIOSGA019295	XP_006643849.1 FACT complex subunit SSRP1-A
TCONS_00036775	-0.576113	-1.69983	1.43827	BGIOSGA036542	XP_006664386.2 protein-tyrosine-phosphatase PTP1-like isoform X1
TCONS_00018803	-0.57607	-0.955343	-1.28535	BGIOSGA012310	XP_004960601.1aquaporin NIP1-3
TCONS_00037518	-0.575613	-0.379972	-1.24338	BGIOSGA035847	XP_006664189.1PREDICTED: uncharacterized protein LOC102721410
TCONS_00036542	-0.57525	0.281813	-0.578038	BGIOSGA034637	XP_006664872.1 pentatricopeptide repeat-containing protein At2g38420, mitochondrial-like
TCONS_00012980	-0.575113	1.28401	-0.0266033	BGIOSGA010010	XP_006651712.1PREDICTED: uncharacterized protein LOC102702550, partial
TCONS_00014754	-0.575099	1.39508	0.421589	BGIOSGA007259	XP_015692381.1 protein CHROMATIN REMODELING 19
TCONS_00010979	-0.574985	-0.251952	-0.176254	BGIOSGA009943	XP_010229723.1serine/threonine-protein kinase dst1
TCONS_00032624	-0.574929	0.394216	0.0663522	BGIOSGA026547	XP_003574263.1uncharacterized protein LOC100825224
TCONS_00036296	-0.574889	-2.05521	-0.0150262	BGIOSGA013293	XP_004962981.1polyadenylate-binding protein, cytoplasmic and nuclear isoform X1
TCONS_00017190	-0.574846	-1.64101	-2.02056	BGIOSGA019234	XP_004977104.1probable GTP diphosphokinase CRSH1, chloroplastic
TCONS_00005182	-0.57482	1.2548	0.512051	BGIOSGA032004	XP_004960534.1pumilio homolog 1
TCONS_00027182	-0.574705	-0.861805	-1.85361	BGIOSGA018004	XP_006659727.2 ascorbate-specific transmembrane electron transporter 1-like
TCONS_00006446	-0.574468	-0.313632	0.818721	BGIOSGA036589	XP_006647501.1 phosphatidyl-N-methylethanolamine N-methyltransferase
TCONS_00024678	-0.574454	0.525479	-0.52433	BGIOSGA009634	XP_006650737.1 40S ribosomal protein S15
TCONS_00002466	-0.574113	-0.655257	-2.29025	BGIOSGA018894	XP_004971167.1tropinone reductase homolog At5g06060
TCONS_00037338	-0.573746	-0.402492	0.0835307	BGIOSGA019666	XP_021307843.1putative disease resistance RPP13-like protein 3
TCONS_00014899	-0.573613	-0.184836	0.324461	BGIOSGA017080	XP_006653748.1 zinc finger CCCH domain-containing protein 13
TCONS_00010886	-0.573228	-2.70288	#NA	#N/A	#N/A
TCONS_00012477	-0.573228	1.51951	0.393664	#N/A	#N/A
TCONS_00028340	-0.572904	-0.950673	-1.18589	BGIOSGA029493	XP_006660305.2PREDICTED: uncharacterized protein LOC102704831 isoform X1
TCONS_00014745	-0.572763	-1.87744	0.0904648	BGIOSGA037389	XP_003581527.1protein S-acyltransferase 10
TCONS_00008876	-0.572494	-0.222899	-1.00477	BGIOSGA021607	XP_006647969.1 pentatricopeptide repeat-containing protein At1g05750, chloroplastic
TCONS_00011914	-0.571418	-0.236833	-0.172636	BGIOSGA035213	XP_015690230.1 long chain base biosynthesis protein 1a
TCONS_00002712	-0.571329	-0.669391	-0.419338	BGIOSGA002522	XP_015692190.1 HBS1-like protein isoform X2
TCONS_00011494	-0.571241	0.379799	-2.69757	BGIOSGA011532	XP_004985869.1uncharacterized protein LOC101755828 isoform X3
TCONS_00002028	-0.571214	-1.4043	-0.899875	BGIOSGA037746	XP_007146213.1hypothetical protein PHAVU_006G021800g
TCONS_00022881	-0.57103	-1.45022	-0.49348	BGIOSGA024837	XP_022684357.1disease resistance protein RGA2 isoform X1
TCONS_00014964	-0.571027	0.257377	0.0274266	BGIOSGA027515	XP_006652843.1 nucleolin 2-like
TCONS_00023703	-0.570949	-0.9217	0.496801	BGIOSGA035322	XP_004957773.1putative casein kinase II subunit beta-4 isoform X3
TCONS_00034123	-0.570875	-0.86267	-0.843084	BGIOSGA036806	XP_006663712.1 SAM50-like protein SPAC17C9.06
TCONS_00002251	-0.570833	-2.24851	-1.04739	BGIOSGA037065	XP_006645086.1 zinc finger protein STOP1 homolog
TCONS_00014417	-0.570792	-0.380627	-1.42505	BGIOSGA016580	XP_015692303.1 agmatine deiminase
TCONS_00036253	-0.570259	-0.91171	-0.91041	BGIOSGA037612	XP_015698674.1PREDICTED: uncharacterized protein LOC102700851
TCONS_00017178	-0.569971	-0.435982	-0.971419	BGIOSGA003417	XP_006654077.1 Golgi apparatus membrane protein-like protein ECHIDNA

transcriptome

TCONS_00034935	-0.569675	1.29336	0.529155	BGIOSGA012493	XP_006649980.1 probable pre-mRNA-splicing factor ATP-dependent RNA helicase DEAH2
TCONS_00011917	-0.569667	-1.78999	0.350151	BGIOSGA005376	XP_006651224.1 agamous-like MADS-box protein AGL29
TCONS_00005292	-0.569439	-1.05034	-1.25687	BGIOSGA021704	XP_006646794.1 choline/ethanolaminephosphotransferase 1-like
TCONS_00034381	-0.569177	-1.96404	-1.66506	BGIOSGA014876	XP_022685187.1filament-like plant protein 6
TCONS_00016165	-0.568658	-0.385808	-1.28077	BGIOSGA014814	XP_006652465.1 transcription factor RF2a-like
TCONS_00014444	-0.568658	-1.12277	0.719226	#N/A	#N/A
TCONS_00016829	-0.568336	0.876878	0.303366	BGIOSGA014135	XP_015691719.1PREDICTED: uncharacterized protein LOC107304046
TCONS_00002236	-0.568269	-1.20895	-0.0959578	BGIOSGA017983	XP_006665040.1 polypyrimidine tract-binding protein homolog 3-like
TCONS_00026352	-0.568059	0.603406	0.252756	BGIOSGA009669	XP_015695564.1 pentatricopeptide repeat-containing protein At5g65560-like
TCONS_00030668	-0.568011	0.12492	-1.04002	BGIOSGA032495	XP_014756261.1disease resistance protein RGA2
TCONS_00009186	-0.567896	-0.687099	-1.71649	BGIOSGA033295	XP_015691099.1PREDICTED: uncharacterized protein LOC102711510 isoform X2
TCONS_00030119	-0.567848	-0.0579256	0.46591	BGIOSGA007788	XP_015696659.1 aldehyde dehydrogenase family 7 member A1
TCONS_00021796	-0.567762	-0.83688	-1.11504	BGIOSGA028430	XP_015690871.1 25.3 kDa vesicle transport protein
TCONS_00012460	-0.567487	-1.37898	-0.0745346	BGIOSGA010525	XP_006650199.1 protein SPIRAL1-like 1
TCONS_00008071	-0.567449	-1.28923	-0.798491	BGIOSGA002221	XP_021316288.1trihelix transcription factor ASR3
TCONS_00028916	-0.567412	0.197805	1.30101	BGIOSGA013832	NP_001152332.1catalytic/ ligase
TCONS_00016973	-0.56741	-1.54484	#NA	BGIOSGA005263	XP_006653956.1 protein DEHYDRATION-INDUCED 19 homolog 6-like
TCONS_00024945	-0.56741	0.179964	0.111483	BGIOSGA033292	XP_003571978.2probable S-adenosyl-L-methionine-dependent RNA methyltransferase RSM22, mitochondrial
TCONS_00011866	-0.567393	-0.330576	-0.0342109	BGIOSGA011142	XP_015690962.1 bZIP transcription factor 16-like isoform X1
TCONS_00033562	-0.567252	0.759234	0.913662	BGIOSGA000205	XP_006663480.1 coiled-coil domain-containing protein SCD2-like
TCONS_00036534	-0.567131	-0.896897	0.41031	BGIOSGA030362	XP_022680397.1uncharacterized protein LOC111256472
TCONS_00011172	-0.566907	-1.56476	-1.69681	BGIOSGA013775	XP_006650759.1PREDICTED: uncharacterized protein LOC102720414
TCONS_00038886	-0.566838	0.2621	-0.371189	BGIOSGA000749	XP_010240583.2exocyst complex component EXO70A1 isoform X1
TCONS_00037291	-0.566827	-0.207217	0.377533	BGIOSGA019930	XP_006664071.1 probable histone H2AXb
TCONS_00029192	-0.566534	0.193005	-1.1793	BGIOSGA030939	XP_006660727.1 queuine tRNA-ribosyltransferase-like
TCONS_00016595	-0.566375	-0.672317	-0.415544	BGIOSGA024242	XP_010914336.1 potassium transporter 7-like isoform X2
TCONS_00002127	-0.566066	-0.472679	-0.446556	BGIOSGA017906	XP_015699313.1 SAC3 family protein A isoform X1
TCONS_00024115	-0.565271	-0.365331	0.883815	BGIOSGA026149	XP_006657963.1 vacuolar protein sorting-associated protein 27 isoform X1
TCONS_00014399	-0.56524	0.534268	-0.243892	BGIOSGA016562	XP_015692369.1 putative pentatricopeptide repeat-containing protein At1g09680
TCONS_00031095	-0.565097	-1.48038	-1.19129	BGIOSGA032915	XP_006661776.2 acylamino-acid-releasing enzyme-like
TCONS_00024661	-0.565041	0.622102	-0.160593	BGIOSGA016475	XP_006657498.1 BI1-like protein
TCONS_00004995	-0.565038	0.234683	-0.159735	BGIOSGA000225	XP_015699196.1PREDICTED: uncharacterized protein LOC102713792 isoform X1
TCONS_00030245	-0.56476	-0.970684	-0.892624	BGIOSGA032130	XP_002441414.1anthocyanidin 3-O-glucosyltransferase 2
TCONS_00016382	-0.56471	0.292287	-0.0101037	BGIOSGA033425	XP_006652620.1 putative glucuronosyltransferase PGSIP8 isoform X1
TCONS_00019994	-0.564425	-0.881792	1.11725	BGIOSGA009624	XP_006648157.1 DEAD-box ATP-dependent RNA helicase 48
TCONS_00034610	-0.564297	-1.00952	-0.64711	BGIOSGA034207	XP_014757471.1protein AUXIN-REGULATED GENE INVOLVED IN ORGAN SIZE
TCONS_00032354	-0.564229	-0.369987	0.256885	BGIOSGA024245	XP_015697010.1 midasin
TCONS_00000402	-0.564105	-0.0645108	-0.747567	BGIOSGA004447	XP_006643893.2 SCAR-like protein 2
TCONS_00003168	-0.563928	0.048662	-0.249051	BGIOSGA025368	XP_006643964.1 transcription factor bHLH13
TCONS_00004600	-0.563541	-0.709083	-0.557793	BGIOSGA035556	XP_015698047.1 putative disease resistance RPP13-like protein 3 isoform X1
TCONS_00000055	-0.563486	-1.12868	-1.01572	BGIOSGA002621	XP_006643656.2 deoxynucleoside triphosphate triphosphohydrolase SAMHD1 homolog
TCONS_00010506	-0.563374	-3.03545	-1.29419	BGIOSGA013113	XP_015689969.1 serpin-ZXA
TCONS_00023176	-0.563374	-1.29462	-0.347259	BGIOSGA013882	XP_006657484.1 glucan endo-1,3-beta-glucosidase 8
TCONS_00018278	-0.563231	0.533654	0.445631	BGIOSGA020339	XP_006654760.1 60S ribosomal protein L35a-2
TCONS_00014210	-0.562752	-0.910534	-0.958243	BGIOSGA029643	XP_015691820.1 alpha carbonic anhydrase 1, chloroplastic-like
TCONS_00000584	-0.562583	-0.204629	0.51139	BGIOSGA003169	XP_015688106.1PREDICTED: uncharacterized protein LOC102715257
TCONS_00021696	-0.562566	0.447324	0.434712	BGIOSGA024479	XP_006655832.2 zinc finger CCCH domain-containing protein 40
TCONS_00003519	-0.562268	0.446499	0.446476	BGIOSGA036044	XP_015688216.1 auxilin-related protein 2-like
TCONS_00018465	-0.562253	-0.846716	-0.40813	BGIOSGA024746	XP_006653950.1PREDICTED: uncharacterized protein LOC102711699 isoform X1
TCONS_00028775	-0.562232	-0.622687	-0.576474	BGIOSGA020592	XP_006656439.2 26S proteasome non-ATPase regulatory subunit 2 homolog A-like
TCONS_00024680	-0.561877	0.136563	1.60061	BGIOSGA024704	XP_015695276.1PREDICTED: uncharacterized protein LOC102707324 isoform X1
TCONS_00019390	-0.561804	-0.371942	0.211198	BGIOSGA010085	XP_006654473.2 glucan endo-1,3-beta-glucosidase 14
TCONS_00016418	-0.561757	-0.995706	-2.12003	BGIOSGA018471	XP_006652643.1 lysine histidine transporter-like 8

TCONS_00035989	-0.561539	-1.04225	-0.360743	BGIOSGA001288	XP_006664011.1PREDICTED: uncharacterized protein LOC102716362
TCONS_00000438	-0.561395	-0.247312	-0.52401	BGIOSGA017955	XP_006660456.1 protein ROS1-like
TCONS_00030400	-0.561275	0.454018	-1.2995	#N/A	#N/A
TCONS_00011446	-0.561153	-0.58633	0.539651	BGIOSGA000670	XP_006650975.1 bifunctional fucokinase/fucose pyrophosphorylase
TCONS_00002451	-0.560866	0.515881	-0.723306	BGIOSGA009433	XP_006645267.2 random slug protein 5-like
TCONS_00024963	-0.560782	-0.274947	-0.247988	BGIOSGA007434	XP_006664838.1 calcium-dependent protein kinase 16-like
TCONS_00011562	-0.560555	0.707012	0.0331987	BGIOSGA033055	XP_015690718.1 putative transporter arsB isoform X1
TCONS_00028553	-0.560546	-0.900985	-0.742818	BGIOSGA030299	XP_006664916.1 structural maintenance of chromosomes protein 6B-like
TCONS_00037185	-0.559715	-1.34212	-0.450041	BGIOSGA036192	XP_006664020.1 L-galactose dehydrogenase
TCONS_00035492	-0.559683	-0.900273	-1.2005	BGIOSGA000595	XP_004980118.1calcium uniporter protein 6, mitochondrial
TCONS_00012981	-0.559575	-2.59238	-0.515326	BGIOSGA010010	XP_006651712.1PREDICTED: uncharacterized protein LOC102702550, partial
TCONS_00010326	-0.559535	-1.73518	0.163842	#N/A	#N/A
TCONS_00017613	-0.55948	-0.453911	-0.76638	BGIOSGA019674	XP_006655223.1 anaphase-promoting complex subunit 1
TCONS_00002402	-0.559461	-0.322064	-0.426393	BGIOSGA033336	XP_015688181.1 protein YLS7-like
TCONS_00029820	-0.559354	-2.23041	-0.38834	BGIOSGA029994	XP_006661117.1 nudix hydrolase 20, chloroplastic-like
TCONS_00029362	-0.559352	-0.702013	-0.488986	BGIOSGA011729	XP_006660857.1 hydroxymethylglutaryl-CoA synthase-like
TCONS_00008945	-0.559226	-0.452771	-1.57336	BGIOSGA031253	XP_004954179.1cyclic nucleotide-gated ion channel 17
TCONS_00003250	-0.559189	-1.29882	0.34681	BGIOSGA003300	XP_002457586.1protein SENESCENCE-ASSOCIATED GENE 21, mitochondrial
TCONS_00017124	-0.559141	-0.762309	-0.0648388	BGIOSGA019172	XP_006654040.2 nucleolar MIF4G domain-containing protein 1
TCONS_00005736	-0.559046	-0.291615	0.150694	BGIOSGA000783	XP_006648489.2 aspartate aminotransferase, mitochondrial-like
TCONS_00008864	-0.558935	-0.159594	-0.881023	BGIOSGA005548	XP_006647965.2PREDICTED: uncharacterized protein LOC102699842, partial
TCONS_00009490	-0.558915	0.746794	0.251028	BGIOSGA016456	XP_006651129.1 cytochrome b561 and DOMON domain-containing protein At4g12980-like
TCONS_00028810	-0.558238	-0.997332	-1.6963	BGIOSGA030561	XP_003578003.1sulfite exporter TauE/SafE family protein 3
TCONS_00036918	-0.558069	-0.0716418	-0.425841	BGIOSGA036407	XP_003578739.1exocyst complex component 5
TCONS_00022855	-0.557436	-0.447777	-0.948846	BGIOSGA020639	XP_006656442.1 putative inactive cadmium/zinc-transporting ATPase HMA3
TCONS_00003058	-0.557142	-0.279474	-0.213267	BGIOSGA029757	XP_006643888.2 shaggy-related protein kinase eta-like
TCONS_00002783	-0.557092	0.601013	-0.310101	#N/A	#N/A
TCONS_00022106	-0.556993	0.833551	0.274613	BGIOSGA021398	XP_006656029.1 protein translocase subunit SecA
TCONS_00027637	-0.556993	-1.55111	-1.50299	BGIOSGA037345	XP_015695540.1PREDICTED: uncharacterized protein LOC107304708
TCONS_00017131	-0.556896	0.60077	2.33326	BGIOSGA001630	XP_004960283.1probable glutathione S-transferase GSTF1
TCONS_00034742	-0.556869	0.136502	-1.81733	BGIOSGA015955	XP_006662877.1 ABC transporter G family member 28-like
TCONS_00007964	-0.556705	-0.0209534	-1.50372	BGIOSGA006517	XP_021307930.1U4/U6.U5 small nuclear ribonucleoprotein 27 kDa protein
TCONS_00036619	-0.556248	1.33905	0.286136	BGIOSGA034563	XP_006662707.1 glutamine--fructose-6-phosphate aminotransferase [isomerizing] 2-like
TCONS_00006447	-0.556226	-0.214315	0.102116	BGIOSGA022042	XP_003572710.2calcium uptake protein, mitochondrial
TCONS_00001495	-0.556213	-2.24479	-0.685282	BGIOSGA004137	XP_006646160.2 probable plastid-lipid-associated protein 14, chloroplastic
TCONS_00014315	-0.556033	-1.7708	-3.14695	BGIOSGA016479	XP_015691473.1 ferric reduction oxidase 7, chloroplastic-like
TCONS_00006165	-0.55588	0.34703	0.624957	#N/A	#N/A
TCONS_00000295	-0.55579	-0.496924	-1.13589	BGIOSGA019320	XP_015699259.1 ABC transporter C family member 3-like
TCONS_00018287	-0.555774	-0.545054	1.51691	BGIOSGA020341	XP_015693180.1PREDICTED: uncharacterized protein LOC102715305
TCONS_00019534	-0.555752	-0.741689	-2.65452	BGIOSGA003020	XP_015692641.1 non-specific lipid transfer protein GPI-anchored 2-like
TCONS_00023935	-0.555689	-0.221921	0.597261	BGIOSGA025966	XP_006657852.2 nodulation receptor kinase-like
TCONS_00013097	-0.555671	-0.983188	-1.04966	BGIOSGA009884	XP_002463955.1sulfite exporter TauE/SafE family protein 4
TCONS_00016961	-0.555594	-1.25633	-0.556144	BGIOSGA019012	XP_006653948.1 tubulin-folding cofactor E
TCONS_00027373	-0.555593	1.11314	-0.0130282	BGIOSGA037365	XP_006659135.1 protein PAF1 homolog
TCONS_00003095	-0.555492	-0.784449	0.647385	BGIOSGA002167	XP_006643903.1 GDSL esterase/lipase At5g45920-like
TCONS_00003742	-0.555172	0.353846	-0.103391	BGIOSGA007462	XP_024315768.1DNA damage-repair/toleration protein DRT102
TCONS_00017863	-0.555144	-2.72334	#NA	BGIOSGA014836	XP_015688131.1 protein PHYTOCHROME KINASE SUBSTRATE 4
TCONS_00025546	-0.555098	-0.866929	-0.41037	BGIOSGA011302	XP_015695237.1 calmodulin-binding transcription activator 1-like isoform X1
TCONS_00032822	-0.555002	1.92034	0.491988	BGIOSGA009635	XP_006663719.1 PRA1 family protein B2-like
TCONS_00036758	-0.554979	0.474386	0.0438047	BGIOSGA036558	XP_015698755.1 V-type proton ATPase 16 kDa proteolipid subunit-like
TCONS_00034188	-0.554932	0.861354	-0.145617	BGIOSGA004690	XP_006663759.1 protein LURP-one-related 8-like
TCONS_00026788	-0.554932	-1.46985	-0.846057	BGIOSGA028850	XP_006659496.1 uncharacterized oxidoreductase YoxD isoform X1
TCONS_00008350	-0.554796	-1.13882	0.610114	BGIOSGA000828	XP_008643956.1putative integrin-linked protein kinase family protein isoform X1

TCONS_00018310	-0.554729	-0.308827	0.00587402	BGIOSGA015674	XP_002440250.1protein N-lysine methyltransferase METTL21A
TCONS_00014330	-0.554668	0.914302	-0.894	BGIOSGA028973	XP_010239962.1putative lipoxygenase 5
TCONS_00009497	-0.554559	-0.389854	-2.27119	BGIOSGA012048	XP_006651134.2 vicilin-like seed storage protein At2g18540
TCONS_00018513	-0.554512	2.75908	0.321145	#N/A	#N/A
TCONS_00005572	-0.554396	-1.92216	-0.8179	BGIOSGA017000	XP_006646991.2 premnaspirodiene oxygenase-like
TCONS_00004497	-0.554378	-1.50431	-1.18466	BGIOSGA019168	XP_015694266.1 jacalin-related lectin 3
TCONS_00035419	-0.554132	-1.65767	-0.563898	BGIOSGA026403	XP_003558758.1mediator of RNA polymerase II transcription subunit 34 isoform X1
TCONS_00015904	-0.554039	-0.335075	-0.788549	BGIOSGA006279	NP_001147920.1neutral/alkaline invertase
TCONS_00009445	-0.553752	-1.07142	-1.22586	BGIOSGA014282	XP_006649512.2 protein STRUBBELIG-RECEPTOR FAMILY 7-like
TCONS_00019644	-0.553641	0.445194	0.0564545	BGIOSGA014099	XP_006654660.1 dolichyl-diphosphooligosaccharide--protein glycosyltransferase subunit STT3A
TCONS_00015526	-0.553461	-1.00819	-0.97467	BGIOSGA015454	XP_015692124.1 NAD-dependent protein deacetylase SRT1 isoform X1
TCONS_00033867	-0.553442	-3.72549	-3.31928	BGIOSGA035572	XP_004979775.1protein PYRICULARIA ORYZAE RESISTANCE 21
TCONS_00023707	-0.552956	-1.14115	0.849428	BGIOSGA025746	XP_006657721.1 expansin-like B1
TCONS_00024391	-0.552661	-1.14006	-0.00548615	BGIOSGA012465	XP_004958766.1probable glutamyl endopeptidase, chloroplastic isoform X1
TCONS_00007355	-0.552636	-1.34327	-0.417301	BGIOSGA022934	XP_022684723.1probable LRR receptor-like serine/threonine-protein kinase At1g05700
TCONS_00004991	-0.552548	-0.287087	-0.20537	BGIOSGA009018	XP_006645253.2 probable histidine kinase 3
TCONS_00009614	-0.552223	-2.7487	-2.82408	BGIOSGA009133	XP_006649663.1 metal tolerance protein 4
TCONS_00011391	-0.5522	-0.580222	-0.271758	BGIOSGA035370	XP_006650938.1 protein TORNADO 2-like
TCONS_00037031	-0.551871	0.231016	-0.153996	BGIOSGA036344	XP_006663957.2 quinolinate synthase, chloroplastic
TCONS_00016269	-0.551581	-0.514431	-0.708846	BGIOSGA005999	XP_006652522.1 phenylalanine ammonia-lyase-like
TCONS_00022074	-0.551573	-3.92343	-1.75122	BGIOSGA002857	XP_003563952.1O-fucosyltransferase 34
TCONS_00000411	-0.551411	1.35165	-1.01221	BGIOSGA025960	XP_003565462.1sodium/calcium exchanger NCL1
TCONS_00019950	-0.551256	0.159269	-0.324737	BGIOSGA022105	XP_006655680.2 NADH dehydrogenase (ubiquinone) complex I, assembly factor 6
TCONS_00038183	-0.551197	-1.72833	#NA	BGIOSGA002510	XP_014754607.2 rust resistance kinase Lr10
TCONS_00008644	-0.55102	-0.665826	-0.6347	BGIOSGA014409	XP_015689291.1 dual specificity tyrosine-phosphorylation-regulated kinase 1B-like isoform X2
TCONS_00022351	-0.550999	0.271419	0.762086	BGIOSGA015367	XP_006656133.2 probable protein phosphatase 2C 56
TCONS_00009193	-0.550972	0.934349	0.089592	BGIOSGA035393	XP_015691285.1 probable glycosidase crf1
TCONS_00004866	-0.550956	-2.45459	-0.862629	BGIOSGA000354	XP_010232674.1DNA-directed RNA polymerase III subunit RPC4 isoform X1
TCONS_00000296	-0.55066	-1.43355	0.462274	BGIOSGA003689	XP_015699356.1 monothiol glutaredoxin-S4, mitochondrial
TCONS_00005037	-0.550635	-0.0660756	-0.589318	BGIOSGA014110	XP_021310923.1coleoptile phototropism protein 1
TCONS_00005739	-0.55061	-3.899	-1.32634	BGIOSGA010666	XP_003571154.1peroxidase P7
TCONS_00016819	-0.550444	-1.99232	-0.589792	BGIOSGA017320	XP_006652980.1 hyoscyamine 6-dioxygenase-like
TCONS_00031905	-0.550421	-0.586352	-1.00489	#N/A	#N/A
TCONS_00016710	-0.550331	-3.72942	-0.519684	BGIOSGA014252	XP_006648582.1PREDICTED: uncharacterized protein LOC102706077
TCONS_00029176	-0.550179	-2.23212	-0.556899	BGIOSGA031661	XP_006660711.1 polyadenylate-binding protein RBP47-like
TCONS_00011132	-0.549802	0.00030272	0.0701982	BGIOSGA009990	XP_006651908.2 E3 ubiquitin protein ligase DRIP2-like
TCONS_00003132	-0.549765	-1.08628	1.13099	BGIOSGA002092	XP_003565805.13-methyl-2-oxobutanoate hydroxymethyltransferase 1, mitochondrial
TCONS_00023041	-0.549729	2.26982	2.11441	BGIOSGA029777	XP_006658240.1PREDICTED: uncharacterized protein LOC102721465
TCONS_00001366	-0.549554	2.05096	1.256	BGIOSGA006706	XP_006646049.2 pentatricopeptide repeat-containing protein At4g36680, mitochondrial-like
TCONS_00026791	-0.549439	-0.31551	0.173248	BGIOSGA011644	XP_003574635.1protein transport protein sec31
TCONS_00002120	-0.549359	0.633175	-0.0919712	BGIOSGA012245	XP_006644966.1 V-type proton ATPase subunit a1
TCONS_00019946	-0.549316	0.416692	-0.150464	BGIOSGA022132	XP_006655676.1 peroxisomal acyl-coenzyme A oxidase 1-like
TCONS_00002200	-0.549313	1.75656	-0.230029	BGIOSGA016731	XP_006646495.1 sialyltransferase-like protein 1
TCONS_00018156	-0.549237	#NA	-2.91472	BGIOSGA006676	XP_006654665.1 probable pectinesterase 53
TCONS_00005486	-0.549207	-1.18869	-1.49029	BGIOSGA018019	XP_006646915.1 probable beta-1,3-galactosyltransferase 2
TCONS_00002245	-0.549149	-1.50852	-1.04046	BGIOSGA005815	XP_006647706.1 phosphatidylinositol glycan anchor biosynthesis class U protein-like
TCONS_00033018	-0.549023	-1.73107	-0.593317	BGIOSGA037038	XP_008679695.1nodulation-signaling pathway 2 protein
TCONS_00032455	-0.548965	-0.71818	-0.894319	BGIOSGA031662	XP_006662455.2PREDICTED: uncharacterized protein LOC102721679, partial
TCONS_00008630	-0.548934	-1.25527	-0.658352	BGIOSGA027474	XP_024314258.1uncharacterized protein LOC100840408
TCONS_00028703	-0.548878	-0.643791	-1.16072	BGIOSGA016488	XP_006661083.2 peptidyl-prolyl cis-trans isomerase FKBP43-like isoform X1
TCONS_00001683	-0.548635	#NA	-3.91621	BGIOSGA026329	XP_024313850.1uncharacterized protein LOC112270208
TCONS_00028819	-0.548396	-0.653857	-1.25383	BGIOSGA030568	XP_006661144.1 probable histone acetyltransferase type B catalytic subunit isoform X1
TCONS_00028510	-0.548326	-2.83148	-0.715336	BGIOSGA029013	XP_006651552.2 RNA pseudouridine synthase 4, mitochondrial-like
TCONS_00010281	-0.548323	0.575249	0.0349826	BGIOSGA023910	XP_002463173.160S ribosomal protein L27a-3

TCONS_00002390	-0.548298	0.320832	-0.148181	BGIOSGA005026	XP_015698304.1 dolichyl-diphosphooligosaccharide--protein glycosyltransferase subunit 2
TCONS_00034112	-0.547672	-0.0589976	-0.576194	BGIOSGA038661	XP_006664262.1PREDICTED: uncharacterized protein LOC102719542
TCONS_00009386	-0.547625	0.45426	1.18658	BGIOSGA031942	XP_015689776.1 IQ domain-containing protein IQM2-like
TCONS_00028895	-0.547464	0.726401	0.617608	BGIOSGA030639	XP_006660581.2 MND1-interacting protein 1-like
TCONS_00018858	-0.547406	0.378973	0.385983	BGIOSGA010008	XP_010231332.1uncharacterized protein LOC100845944
TCONS_00027794	-0.547139	-0.0238847	-0.322861	BGIOSGA028437	XP_015696027.1 exocyst complex component SEC8 isoform X2
TCONS_00035667	-0.547117	0.91039	0.337433	BGIOSGA034932	XP_003578845.1SWI/SNF complex subunit SWI3C
TCONS_00004408	-0.546997	-1.51122	-1.54174	BGIOSGA007153	XP_006646331.2 auxin response factor 3
TCONS_00025685	-0.546785	-0.663492	-0.850382	BGIOSGA005535	XP_006658950.1 pentatricopeptide repeat-containing protein At4g32430, mitochondrial
TCONS_00018018	-0.546685	-0.762204	0.439303	BGIOSGA005483	XP_002439976.1uncharacterized protein LOC8077017
TCONS_00030984	-0.546491	0.785124	0.23231	BGIOSGA033570	XP_015697929.1 disease resistance protein RPP13-like
TCONS_00002677	-0.546402	-0.734362	0.155101	BGIOSGA002544	XP_008646447.1AP-5 complex subunit beta-1
TCONS_00022146	-0.546371	-2.01194	-1.70456	BGIOSGA021358	XP_006656922.2 protein WEAK CHLOROPLAST MOVEMENT UNDER BLUE LIGHT 1-like
TCONS_00013066	-0.546182	0.773158	-0.051891	BGIOSGA014054	XP_015690041.1 receptor protein kinase TMK1-like
TCONS_00013413	-0.545836	-1.93423	-0.890896	BGIOSGA009569	XP_015690685.1 U-box domain-containing protein 62-like
TCONS_00001806	-0.545656	-1.14201	0.158406	BGIOSGA021596	XP_015688329.1 pseudo histidine-containing phosphotransfer protein 1
TCONS_00023331	-0.545573	0.608444	0.778663	BGIOSGA013783	XP_004981345.140S ribosomal protein S2-3
TCONS_00006872	-0.545507	-2.11793	-2.02519	BGIOSGA000885	XP_006647886.1 HVA22-like protein a
TCONS_00020193	-0.545318	-1.4299	-0.737048	BGIOSGA022384	XP_004964739.1probable LRR receptor-like serine/threonine-protein kinase At1g56130 isoform X1
TCONS_00008132	-0.545237	-3.59032	-2.16724	BGIOSGA016610	XP_004952661.1uncharacterized protein LOC101753026
TCONS_00015234	-0.545119	-1.00229	-1.59066	BGIOSGA017420	XP_015691673.1 probable thylakoidal processing peptidase 2, chloroplastic isoform X2
TCONS_00009598	-0.545104	-3.18782	-1.73882	BGIOSGA028353	XP_015689819.1 probable inactive receptor kinase At1g48480
TCONS_00028848	-0.544937	-1.73689	-1.53933	BGIOSGA028621	XP_015696321.1 aminopeptidase M1-D
TCONS_00021761	-0.544532	-0.648058	-1.11971	BGIOSGA021759	XP_006655866.1 putative UDP-sugar transporter DDB_G0278631
TCONS_00002797	-0.544363	-1.17095	-0.572724	BGIOSGA012302	XP_002455216.1mitochondrial substrate carrier family protein B
TCONS_00006891	-0.544271	-0.143371	-0.0651788	BGIOSGA031767	XP_006647912.1PREDICTED: uncharacterized protein LOC102707374
TCONS_00031469	-0.54419	0.464702	-0.507138	BGIOSGA020303	XP_006661962.1 STE20/SPS1-related proline-alanine-rich protein kinase isoform X2
TCONS_00022963	-0.544164	-0.298018	-1.40805	BGIOSGA006370	XP_015694500.1 potassium transporter 22-like
TCONS_00025135	-0.544118	-0.357277	-0.631551	BGIOSGA012622	XP_006657726.1 protein CHROMATIN REMODELING 4
TCONS_00022772	-0.543977	-1.49727	-0.213857	BGIOSGA022468	XP_006656378.1 auxin response factor 17
TCONS_00018063	-0.543938	-0.420267	-0.360696	BGIOSGA025787	XP_015692796.1 putative E3 ubiquitin-protein ligase RING1a
TCONS_00015172	-0.543909	0.0660186	0.180206	BGIOSGA027327	NP_001152519.1fiber protein Fb34 precursor
TCONS_00009616	-0.543806	-0.0357621	-0.144576	BGIOSGA032403	NP_001333756.1uncharacterized LOC103633237
TCONS_00006852	-0.543558	1.42111	-0.166686	BGIOSGA009033	XP_015689268.1PREDICTED: uncharacterized protein LOC102719081
TCONS_00016241	-0.543254	-0.86563	-0.38527	BGIOSGA014733	XP_015692280.1PREDICTED: LOW QUALITY PROTEIN: uncharacterized protein LOC102703674
TCONS_00014555	-0.543227	-0.336448	-0.814435	BGIOSGA028311	XP_015692376.1 protein NUCLEAR FUSION DEFECTIVE 4-like
TCONS_00003417	-0.542911	-0.175968	0.930214	BGIOSGA016998	XP_006651596.1 LOB domain-containing protein 37-like
TCONS_00030985	-0.542787	-0.955068	-0.618601	BGIOSGA032792	XP_006661725.1 pollen-specific protein C13
TCONS_00028347	-0.5423	-0.92018	-0.711375	BGIOSGA026620	XP_015695894.1 E3 ubiquitin-protein ligase RNF4-like isoform X1
TCONS_00006495	-0.542112	0.0121497	-0.945125	BGIOSGA013999	NP_001105026.1aquaporin PIP2-4
TCONS_00018053	-0.542051	0.330255	-1.09112	BGIOSGA030796	XP_003568197.1probable sugar phosphate/phosphate translocator At2g25520
TCONS_00003290	-0.541485	0.0722447	0.275168	BGIOSGA003227	XP_015688154.1 rab proteins geranylgeranyltransferase component A 1
TCONS_00001821	-0.541404	-0.942727	0.37852	BGIOSGA009753	XP_006644705.1PREDICTED: uncharacterized protein LOC102715348 isoform X1
TCONS_00027473	-0.541368	-2.34525	-2.12243	BGIOSGA026917	XP_015696152.1 cinnamoyl-CoA reductase 1-like
TCONS_00019132	-0.541271	-4.06598	0.370505	BGIOSGA020322	XP_006654290.1 E3 ubiquitin-protein ligase MARCH5-like
TCONS_00003008	-0.541255	-1.43987	-1.36761	BGIOSGA022272	XP_006643870.2PREDICTED: uncharacterized protein LOC102702325, partial
TCONS_00031740	-0.541198	0.25679	-0.645787	BGIOSGA010923	XP_015697361.1 barley B recombinant-like protein A
TCONS_00001550	-0.540972	-0.0118692	-0.51999	BGIOSGA017565	XP_006644502.2 homeobox-DDT domain protein RLT2-like
TCONS_00004201	-0.540361	-1.67715	-0.243471	BGIOSGA001001	XP_003569588.1caffeoylshikimate esterase
TCONS_00026541	-0.539876	-0.193108	-0.370575	BGIOSGA028608	XP_006659373.1 proton pump-interactor 1-like
TCONS_00008208	-0.539594	-1.97527	-0.385977	BGIOSGA007547	XP_006648759.2 homeobox-leucine zipper protein HOX7-like

TCONS_00004197	-0.539017	-0.975998	-0.222353	BGIOSGA002077	XP_006644557.2 arginine/serine-rich coiled-coil protein 2 isoform X2
TCONS_00010868	-0.538532	-1.09449	-0.530395	BGIOSGA013490	XP_006651765.2 lariat debranching enzyme
TCONS_00012844	-0.538381	-0.947281	-0.110465	BGIOSGA025269	XP_002464148.1cationic amino acid transporter 2, vacuolar isoform X2
TCONS_00023210	-0.538359	0.121631	0.0134829	BGIOSGA025251	XP_006657500.1 peptidyl-prolyl cis-trans isomerase CYP63-like
TCONS_00007387	-0.53823	-0.936476	-0.30371	BGIOSGA007089	XP_006646905.2 DExH-box ATP-dependent RNA helicase DExH11
TCONS_00021134	-0.537931	-0.387645	-0.45654	BGIOSGA007004	XP_006656294.1 pyrophosphate-energized vacuolar membrane proton pump-like
TCONS_00026466	-0.537886	-1.13757	0.131752	BGIOSGA026888	XP_006662238.1 AT-rich interactive domain-containing protein 4-like
TCONS_00037297	-0.537851	-0.245578	0.430732	BGIOSGA018818	XP_015698691.1 VIN3-like protein 1 isoform X2
TCONS_00005024	-0.537675	-0.857733	0.193294	BGIOSGA033850	XP_006645281.2 heparan-alpha-glucosaminide N-acetyltransferase-like
TCONS_00027396	-0.537536	-0.263351	0.361189	BGIOSGA005435	XP_015695775.1 protein ABHD17C
TCONS_00007153	-0.537529	-0.770503	-1.06473	BGIOSGA009338	XP_006649193.1 testis-expressed sequence 2 protein-like
TCONS_00020901	-0.537414	-0.692366	-0.275405	BGIOSGA017626	XP_003563730.1L-ascorbate oxidase
TCONS_00022249	-0.536973	-1.35408	-1.30662	#N/A	#N/A
TCONS_00025515	-0.53697	-1.66584	-1.37321	BGIOSGA005501	XP_015694843.1 nuclear transcription factor Y subunit A-7-like isoform X2
TCONS_00014825	-0.536912	-0.888302	-0.411029	BGIOSGA031484	XP_008663453.1RING-H2 finger protein ATL74
TCONS_00036236	-0.536902	-1.32885	-1.17871	BGIOSGA007734	XP_006664642.1 pentatricopeptide repeat-containing protein At2g31400, chloroplastic
TCONS_00037393	-0.536893	0.263251	0.37921	BGIOSGA007844	XP_015698727.1 protein spotted leaf 11 isoform X1
TCONS_00005348	-0.536611	0.797022	-0.725637	BGIOSGA007461	NP_001168856.1uncharacterized LOC100382661
TCONS_00004594	-0.536536	-1.81945	-0.790566	BGIOSGA014380	XP_015690221.1 probable LRR receptor-like serine/threonine-protein kinase At1g51810
TCONS_00005347	-0.536497	-0.377717	-0.308762	BGIOSGA031300	XP_006646832.2 beta-amylase 8-like
TCONS_00021593	-0.536486	-1.97702	-1.23635	BGIOSGA003978	XP_004964513.1putative cytochrome c oxidase subunit 5b-like
TCONS_00016593	-0.5362	-0.0280425	0.249343	BGIOSGA013268	XP_015692339.1PREDICTED: uncharacterized protein LOC102720699 isoform X1
TCONS_00004227	-0.536185	0.565071	0.544348	BGIOSGA000977	XP_015697301.1 symplekin
TCONS_00025604	-0.536165	-1.20736	-0.914528	BGIOSGA018055	XP_015694480.1 probable mediator of RNA polymerase II transcription subunit 26b
TCONS_00025325	-0.536107	0.0800664	-0.455951	BGIOSGA024103	XP_006657824.1 equilibrative nucleotide transporter 3-like
TCONS_00003799	-0.536058	0.0508155	-0.0477318	BGIOSGA013895	XP_006646005.1 IAA-amino acid hydrolase ILR1-like 1
TCONS_00030203	-0.536053	1.07634	1.74036	BGIOSGA025174	XP_006660732.1 peroxidase 17
TCONS_00027130	-0.536039	-0.167719	0.379097	BGIOSGA031276	XP_006660378.2 peroxisome biogenesis protein 1
TCONS_00021263	-0.535827	0.0208935	-0.936458	BGIOSGA023489	XP_006656398.1 synaptotagmin-3-like isoform X1
TCONS_00004650	-0.535752	-0.734001	-0.0298498	BGIOSGA018268	XP_006646440.2 target of Myb protein 1-like
TCONS_00027778	-0.535454	0.593241	-0.492106	BGIOSGA027218	XP_006659291.1 protein SMG7 isoform X2
TCONS_00035697	-0.535248	-2.44911	-5.18657	BGIOSGA032966	XP_006663882.1 carboxyvinyl-carboxyphosphonate phosphorylmutase, chloroplastic
TCONS_00002412	-0.534917	0.214014	-0.574985	BGIOSGA027144	XP_006645229.1 leucine-rich repeat receptor protein kinase MSP1
TCONS_00018082	-0.534832	1.80394	-0.136122	BGIOSGA008702	XP_004961595.1universal stress protein PHOS32
TCONS_00032100	-0.534778	-0.9496	0.253213	BGIOSGA032036	XP_006662307.1 pyridoxine/pyridoxamine 5'-phosphate oxidase 1, chloroplastic isoform X2
TCONS_00023515	-0.534741	-1.50944	-1.26044	BGIOSGA031237	XP_006660957.1 uncharacterized transporter YBR287W-like
TCONS_00001526	-0.534692	-1.13492	-0.913821	BGIOSGA001281	XP_006644476.1PREDICTED: uncharacterized protein LOC102718499
TCONS_00036661	-0.534145	-2.56467	-0.689739	BGIOSGA036651	XP_021311068.1uncharacterized protein LOC8062346
TCONS_00009669	-0.534075	0.0903205	-0.303742	BGIOSGA012214	XP_003561826.1acyl-CoA-binding domain-containing protein 5 isoform X1
TCONS_00009121	-0.533903	-0.657872	-0.261501	BGIOSGA023974	NP_001336929.1uncharacterized LOC100278565
TCONS_00030499	-0.533821	-1.38195	-0.664988	BGIOSGA029309	XP_023157456.1DNA mismatch repair protein MLH3 isoform X3
TCONS_00013403	-0.533582	-0.879277	-0.309096	BGIOSGA009577	XP_015690124.1 probable DNA gyrase subunit A, chloroplastic/mitochondrial
TCONS_00027972	-0.533089	-0.716643	-0.0727411	BGIOSGA028641	XP_022683438.1uncharacterized protein LOC101777506
TCONS_00020807	-0.53284	0.596634	0.764879	BGIOSGA012762	XP_003567668.1exocyst complex component SEC15B
TCONS_00019714	-0.532813	-0.7115	-0.30379	BGIOSGA034934	XP_021302483.1serine/arginine repetitive matrix protein 1 isoform X3
TCONS_00004489	-0.532712	-2.28589	-0.356785	BGIOSGA020159	XP_022680410.1pseudouridylate synthase 7 homolog isoform X1
TCONS_00005229	-0.532634	-1.6467	-0.745511	BGIOSGA007339	XP_006659776.2 RNA-binding protein 48-like isoform X1
TCONS_00024935	-0.53262	-4.21565	-0.229086	BGIOSGA012992	NP_001141382.1uncharacterized LOC100273473
TCONS_00000346	-0.532393	-1.80413	-1.4871	BGIOSGA027197	XP_003565447.1zinc finger CCCH domain-containing protein 17
TCONS_00028950	-0.531955	-1.01382	-0.00380758	BGIOSGA009342	XP_024318842.1lysine-specific demethylase JMJ25 isoform X1
TCONS_00009824	-0.531655	-0.172474	0.137759	BGIOSGA012377	XP_006651270.2 DDB1- and CUL4-associated factor 8
TCONS_00022583	-0.531263	0.122907	-0.544816	BGIOSGA004912	XP_015693641.1 RNA-binding protein 38-like
TCONS_00012994	-0.530956	0.0217289	-0.32239	BGIOSGA017236	XP_006650436.1 chloride channel protein CLC-d

TCONS_00011166	-0.530876	-4.21785	-1.49644	BGIOSGA013772	XP_006651935.1 protochlorophyllide-dependent translocon component 52, chloroplastic-like
TCONS_00005190	-0.530818	0.388966	0.261602	BGIOSGA000014	XP_006645401.1PREDICTED: uncharacterized protein LOC102706703 isoform X1
TCONS_00021146	-0.530627	-0.86905	-1.26192	BGIOSGA023367	XP_004965731.150S ribosomal protein L35, chloroplastic
TCONS_00005760	-0.530537	2.43278	1.28932	BGIOSGA007873	XP_024314853.1probable splicing factor 3A subunit 1
TCONS_00037042	-0.530028	-2.11998	-1.38814	BGIOSGA036337	XP_004952076.1phosphoglucan, water dikinase, chloroplastic
TCONS_00011946	-0.529792	0.429897	-0.151674	BGIOSGA009117	XP_006651233.1 mitogen-activated protein kinase kinase kinase 1-like
TCONS_00011470	-0.529713	-0.664983	-0.865741	BGIOSGA033156	XP_015690751.1 endoribonuclease Dicer homolog 1
TCONS_00031606	-0.529681	-0.679413	-1.55968	BGIOSGA031457	XP_006662585.1 U-box domain-containing protein 33-like isoform X1
TCONS_00017255	-0.529632	-0.602602	-1.05193	BGIOSGA019300	XP_015693384.1 protein CHUP1, chloroplastic
TCONS_00010337	-0.529629	-0.151657	-1.43448	BGIOSGA035954	XP_006650207.1 diacylglycerol kinase 1 isoform X1
TCONS_00028942	-0.529255	-0.392298	-0.420935	BGIOSGA025290	XP_006660596.1 chromatin remodeling protein EBS
TCONS_00035436	-0.529196	-1.05706	-1.07018	BGIOSGA037885	XP_015698707.1PREDICTED: uncharacterized protein LOC102718415
TCONS_00022418	-0.529104	-0.378165	0.457322	BGIOSGA019930	XP_002438562.1tRNA A64-2'-O-ribosylphosphate transferase
TCONS_00025254	-0.528314	-1.25116	1.67629	BGIOSGA008904	XP_012699403.1cysteine-rich receptor-like protein kinase 6
TCONS_00019792	-0.528264	-0.0204103	1.83701	BGIOSGA004181	XP_006654783.1 protein DEHYDRATION-INDUCED 19
TCONS_00008835	-0.527556	0.0787435	-0.0735404	BGIOSGA022553	XP_015689371.1 SNW/SKI-interacting protein
TCONS_00004742	-0.5275	-0.574337	-0.624073	BGIOSGA021811	XP_006646487.1 transcription factor DIVARICATA-like
TCONS_00023519	-0.526938	0.18575	-0.39011	BGIOSGA025554	XP_015698427.1 xylulose kinase isoform X1
TCONS_00004089	-0.526914	-0.994088	-1.21698	BGIOSGA020656	XP_006644455.1 cytosolic isocitrate dehydrogenase [NADP]-like
TCONS_00003719	-0.526855	-0.351374	-0.298125	BGIOSGA007885	XP_006645969.2 putative callose synthase 6
TCONS_00008615	-0.526594	-0.764879	1.16439	BGIOSGA023574	XP_015688374.1 protein NRT1/ PTR FAMILY 7.2-like
TCONS_00009683	-0.526451	0.878364	-0.659447	BGIOSGA026406	XP_006649740.1PREDICTED: uncharacterized protein LOC102706732
TCONS_00015935	-0.526399	-0.383694	0.0738214	BGIOSGA024269	XP_015691464.1 glycolipid transfer protein 2
TCONS_00008827	-0.526302	-0.180283	0.672688	BGIOSGA000497	XP_002452533.2probable pectin methyltransferase QUA2
TCONS_00003467	-0.526225	0.491497	0.0273972	BGIOSGA001754	XP_022682343.1F-box protein SKIP23-like
TCONS_00020095	-0.526183	-2.64912	-1.47357	BGIOSGA003221	XP_010228098.1wall-associated receptor kinase 3 isoform X1
TCONS_00032682	-0.526112	-0.691008	-2.8133	BGIOSGA019157	XP_015697075.1 transcription factor UNE10-like
TCONS_00020671	-0.525924	-0.182495	0.28954	BGIOSGA011793	XP_015694103.1PREDICTED: uncharacterized protein LOC102712736
TCONS_00020648	-0.525688	-2.55946	-0.717961	BGIOSGA013183	XP_006656052.1 auxin-responsive protein IAA21
TCONS_00006033	-0.525512	-0.862803	-1.09822	BGIOSGA008185	XP_006665033.1 high mobility group B protein 15-like
TCONS_00019321	-0.525208	0.0762991	-0.648568	BGIOSGA027060	XP_006654423.1 probable receptor-like protein kinase At1g11050
TCONS_00017576	-0.525159	-2.25385	-0.471359	BGIOSGA035755	XP_004962423.1protein NRT1/ PTR FAMILY 2.7
TCONS_00007010	-0.525111	0.0633371	0.398903	BGIOSGA034406	XP_002450408.1protein TPLATE
TCONS_00033081	-0.524998	-1.38334	-1.85027	BGIOSGA022473	XP_015697509.1 myb-related protein 308
TCONS_00000519	-0.524948	-0.497213	-0.841855	BGIOSGA036199	XP_006645691.1 auxin response factor 1
TCONS_00022433	-0.524524	-2.44703	-1.26481	BGIOSGA002213	XP_006656170.1 pollen-specific protein SF21-like isoform X1
TCONS_00015109	-0.524463	-0.632555	0.313878	BGIOSGA017289	XP_006652952.1 protein PELOTA 1
TCONS_00030686	-0.524454	-0.166062	-0.157548	BGIOSGA013958	XP_006660835.1 ankyrin repeat domain-containing protein 2A-like
TCONS_00022023	-0.524447	-0.0495945	-1.24503	BGIOSGA013611	XP_014753035.1uncharacterized protein LOC100837969
TCONS_00008596	-0.523831	-1.78557	-1.33891	BGIOSGA029303	XP_010504143.1 histone H4
TCONS_00002568	-0.523702	0.0987798	0.218219	BGIOSGA002538	XP_003565105.2ribosomal RNA-processing protein 12
TCONS_00032797	-0.523429	-0.143593	0.0263472	BGIOSGA029174	XP_015698258.1PREDICTED: uncharacterized protein LOC102701320, partial
TCONS_00037449	-0.523349	-1.40814	-1.59134	BGIOSGA029539	XP_006664158.2PREDICTED: uncharacterized protein LOC102712487
TCONS_00028199	-0.523334	-1.77415	1.77782	BGIOSGA026829	XP_006659512.1 basic blue protein-like
TCONS_00023874	-0.522803	-2.42983	-1.8193	BGIOSGA025905	XP_015695418.1 BTB/POZ domain-containing protein At3g22104
TCONS_00034405	-0.522785	0.281241	-0.328836	BGIOSGA034405	XP_006662774.1 DExH-box ATP-dependent RNA helicase DExH10
TCONS_00017683	-0.522637	-1.69522	-0.937556	BGIOSGA030088	XP_006654325.1 RNA-binding protein BRN1-like
TCONS_00033358	-0.522634	-0.309503	-1.77597	BGIOSGA023337	XP_010237180.1premnaspirodiene oxygenase isoform X2
TCONS_00024407	-0.522496	0.0229882	-0.469506	BGIOSGA024675	XP_006658174.1 probable AMP deaminase
TCONS_00019284	-0.522378	-2.13523	-1.70053	BGIOSGA025304	XP_006654395.1 1-deoxy-D-xylulose-5-phosphate synthase 1, chloroplastic isoform X4
TCONS_00036720	-0.52214	0.535893	-0.594334	BGIOSGA015126	XP_015698223.1 transcription factor E2FA-like
TCONS_00021486	-0.521919	-0.908353	-0.526474	BGIOSGA022063	XP_006655719.1PREDICTED: uncharacterized protein LOC102706663
TCONS_00021798	-0.521635	-0.444122	-0.161174	BGIOSGA009147	XP_006655885.1 polyadenylate-binding protein-interacting protein 7
TCONS_00009516	-0.521493	-1.18808	-0.180895	BGIOSGA026517	XP_021302227.1putative F-box/FBD/LRR-repeat protein At4g03220 isoform X1
TCONS_00011861	-0.521314	0.419378	0.0854493	BGIOSGA003655	XP_015689828.1 type IV inositol polyphosphate 5-phosphatase 9

TCONS_00027022	-0.521208	0.553208	0.938075	BGIOSGA016314	XP_006659640.2 glycine--tRNA ligase, mitochondrial 1-like
TCONS_00035509	-0.520977	0.807969	-0.102613	BGIOSGA001894	XP_015699121.1 calmodulin-binding protein 25-like
TCONS_00005895	-0.52089	-0.875114	-0.467662	BGIOSGA006624	XP_021313205.1tyrosine aminotransferase isoform X1
TCONS_00034357	-0.520869	-1.22754	-0.398165	BGIOSGA034446	XP_006662758.1 autophagy-related protein 13b-like
TCONS_00022502	-0.520866	-0.364361	-1.171	BGIOSGA020951	XP_006656197.2 two-on-two hemoglobin-3
TCONS_00009563	-0.520753	-0.691141	-0.259825	BGIOSGA006849	XP_006651167.1 protein RIK isoform X1
TCONS_00015021	-0.519957	-0.903901	-0.218626	BGIOSGA027555	XP_006652883.1 double-strand break repair protein MRE11 isoform X1
TCONS_00021909	-0.519668	-0.568905	-1.54483	BGIOSGA011458	XP_006655938.1 aquaporin NIP2-2
TCONS_00021414	-0.519663	-1.33277	-0.323214	BGIOSGA008813	XP_015693717.1PREDICTED: uncharacterized protein LOC102701461
TCONS_00015941	-0.51955	-0.57716	0.688597	BGIOSGA015042	XP_006653432.1 nuclear speckle splicing regulatory protein 1-like
TCONS_00033476	-0.519515	0.394401	1.08102	BGIOSGA011671	XP_021314058.1plasma membrane ATPase isoform X2
TCONS_00033180	-0.519503	0.03046	-0.711705	BGIOSGA034641	XP_006663720.1PREDICTED: uncharacterized protein LOC102720754
TCONS_00002262	-0.519294	-1.44564	0.641045	BGIOSGA000524	XP_015690519.1 protein NRT1/ PTR FAMILY 5.10-like
TCONS_00010442	-0.519055	-0.0690059	0.60634	BGIOSGA013035	XP_002466864.1FRIGIDA-like protein 3
TCONS_00017764	-0.519009	-2.93748	0.593958	BGIOSGA008904	XP_004962160.1probable serine/threonine-protein kinase PBL7
TCONS_00010615	-0.518808	-0.665235	0.0654383	BGIOSGA013238	XP_006651650.1 protein FAR1-RELATED SEQUENCE 5-like isoform X2
TCONS_00018311	-0.518604	-0.343288	-0.240335	BGIOSGA031012	XP_015693290.1 splicing factor U2af small subunit B
TCONS_00030456	-0.518045	0.344194	0.224907	BGIOSGA011386	XP_006660916.1 H/ACA ribonucleoprotein complex subunit 1
TCONS_00034899	-0.517636	-0.51813	0.391473	BGIOSGA016888	XP_006663465.2 putative squamosa promoter-binding-like protein 19
TCONS_00005002	-0.517564	-0.405011	-0.0246984	BGIOSGA000218	XP_006645263.1 ubiquitin-conjugating enzyme E2 27
TCONS_00036683	-0.517296	0.403867	-0.162045	BGIOSGA034487	XP_006662734.1 60S ribosomal protein L26-1-like
TCONS_00011865	-0.517234	-0.296246	0.695322	BGIOSGA027891	XP_022685589.1zinc finger protein 4
TCONS_00025777	-0.516567	-0.487788	-0.610376	BGIOSGA020180	XP_006659019.1 tubby-like F-box protein 12 isoform X2
TCONS_00027740	-0.516476	0.0653639	-0.376965	BGIOSGA027261	XP_006660015.1 pentatricopeptide repeat-containing protein At4g19440, chloroplastic-like
TCONS_00027151	-0.516346	0.444597	0.39767	BGIOSGA007449	XP_006660387.1 serine carboxypeptidase-like 51
TCONS_00021858	-0.516339	-0.166135	0.315714	BGIOSGA021661	XP_015693794.1 mediator of RNA polymerase II transcription subunit 6
TCONS_00000417	-0.516247	-1.07137	-0.669876	BGIOSGA026217	XP_010230614.1uncharacterized protein LOC100833563
TCONS_00030191	-0.516162	0.287359	-0.792787	BGIOSGA029614	NP_001131978.1Purine permease 3
TCONS_00026732	-0.516119	0.588085	-0.886614	BGIOSGA030846	XP_003574588.1probable GTP diphosphokinase RSH2, chloroplastic
TCONS_00018613	-0.516059	0.203669	0.71835	BGIOSGA018821	XP_006654036.1 DNA repair helicase XPD
TCONS_00018860	-0.515909	0.562925	-0.222374	BGIOSGA036323	XP_015698945.1 ABC transporter G family member 12-like isoform X1
TCONS_00004448	-0.515487	0.538044	0.8222	BGIOSGA020174	XP_015691196.1 mitochondrial import inner membrane translocase subunit TIM50-like
TCONS_00032650	-0.515417	-0.544888	-0.0410155	BGIOSGA021847	XP_015697388.1 tetratricopeptide repeat protein SKI3
TCONS_00002063	-0.515344	1.67537	0.655832	BGIOSGA013816	XP_006644907.1 KH domain-containing protein SPIN1-like
TCONS_00033470	-0.515274	-1.64665	0.298107	BGIOSGA014614	XP_004979270.1ELMO domain-containing protein A isoform X1
TCONS_00032860	-0.514979	-0.55969	-1.3708	BGIOSGA034679	XP_006662672.1 non-specific lipid-transfer protein 1
TCONS_00000715	-0.514746	-0.127258	0.274643	BGIOSGA031532	XP_006644095.1 glycogen synthase kinase-3 homolog MsK-1-like
TCONS_00008553	-0.514602	-0.895314	0.714178	BGIOSGA014552	XP_006647661.1 ganglioside-induced differentiation-associated protein 2-like
TCONS_00029550	-0.51436	-0.413221	-0.268557	BGIOSGA011673	XP_014757943.1uncharacterized protein LOC100832504
TCONS_00018787	-0.514123	0.319168	-1.38016	BGIOSGA006676	XP_003564938.1PRA1 family protein F3
TCONS_00009198	-0.513945	-1.46345	-1.5303	BGIOSGA016953	XP_006649273.1 dihydrodipicolinate reductase-like protein CRR1, chloroplastic isoform X1
TCONS_00013994	-0.513934	0.738449	0.355336	BGIOSGA016140	XP_006652178.1 importin beta-like SAD2
TCONS_00004406	-0.513844	-0.41646	-0.0989256	BGIOSGA010255	XP_015688108.1 transcription factor VOZ1
TCONS_00004411	-0.5138	-0.558321	-0.327491	BGIOSGA003708	XP_004969987.1uncharacterized protein At4g28440
TCONS_00020562	-0.513701	1.4452	-0.0999049	BGIOSGA022733	XP_015693553.1PREDICTED: uncharacterized protein LOC102717260
TCONS_00003493	-0.513244	-0.488522	-1.76054	BGIOSGA021201	XP_006645860.1 LYR motif-containing protein At3g19508
TCONS_00026819	-0.513104	-0.698353	0.451643	BGIOSGA028876	XP_006659517.1PREDICTED: uncharacterized protein LOC102716893
TCONS_00013601	-0.512997	0.441118	-0.14739	BGIOSGA015754	XP_006652071.2 zinc finger CCCH domain-containing protein 24
TCONS_00026285	-0.512484	-1.52464	-1.3869	BGIOSGA028368	XP_006659255.2 reticulon-like protein B21 isoform X2
TCONS_00016016	-0.512462	0.500491	0.750283	BGIOSGA006189	XP_006652370.1 NAP1-related protein 1
TCONS_00036327	-0.512383	0.488263	-0.239795	BGIOSGA012069	XP_006664154.1 calcium-transporting ATPase 1, plasma membrane-type
TCONS_00006381	-0.512301	-0.67687	-0.27632	BGIOSGA027714	XP_006647456.2 actin-related protein 3 isoform X2
TCONS_00028734	-0.512203	0.137975	-0.902167	BGIOSGA028529	XP_015696602.1 protein SAR DEFICIENT 1-like

TCONS_00010237	-0.512167	0.590442	-0.709611	BGIOSGA010905	XP_006651470.1 probable protein phosphatase 2C 12
TCONS_00005426	-0.51192	-0.272124	0.0492175	BGIOSGA000186	XP_006646881.1 pathogenesis-related homeodomain protein
TCONS_00031409	-0.511906	1.41803	0.0786924	BGIOSGA033223	NP_001143149.1hypothetical protein
TCONS_00013637	-0.511902	0.234943	-0.189327	BGIOSGA015793	XP_015691355.1 tRNA-dihydrouridine(47) synthase [NAD(P)(+)]-like
TCONS_00011444	-0.511375	0.117826	-0.463641	BGIOSGA023363	XP_006649262.2 AAA-ATPase At2g46620
TCONS_00031076	-0.511362	-0.198203	0.0201282	BGIOSGA006869	XP_002467261.1putative serine/threonine-protein phosphatase PP2A-4 catalytic subunit
TCONS_00033030	-0.51124	-0.218294	-0.158296	BGIOSGA004862	XP_006662759.1 actin-7
TCONS_00004155	-0.511231	-0.818392	0.610004	BGIOSGA020354	XP_014660809.2 mediator of RNA polymerase II transcription subunit 15-like
TCONS_00019935	-0.510955	-0.684516	0.844255	BGIOSGA022119	XP_015694340.1 protein-lysine methyltransferase METTL21D
TCONS_00019087	-0.510861	-0.719322	-0.464911	BGIOSGA018107	XP_006654272.1 TOM1-like protein 2
TCONS_00007783	-0.510763	-0.580449	-0.801606	BGIOSGA006700	XP_015688553.1 probable tocopherol cyclase, chloroplastic
TCONS_00006270	-0.510434	-1.38509	-0.294184	BGIOSGA006255	XP_006647377.1 RING-H2 finger protein ATL58-like
TCONS_00004321	-0.510434	-1.06316	-0.957149	BGIOSGA021834	XP_004969818.1uncharacterized protein LOC101753132
TCONS_00005807	-0.510388	-1.36762	-1.43408	BGIOSGA007928	XP_006647138.2PREDICTED: uncharacterized protein LOC102716377
TCONS_00025343	-0.510018	-0.893585	-0.73438	BGIOSGA033172	XP_006657835.1 myosin-1 isoform X2
TCONS_00006396	-0.50998	-1.81619	-0.445796	BGIOSGA008552	XP_015689345.1 telomere length regulation protein TEL2 homolog
TCONS_00017756	-0.509826	0.108148	0.00121974	BGIOSGA011758	XP_006654361.1 DUF21 domain-containing protein At4g14240
TCONS_00007527	-0.509775	-0.165653	-0.469177	BGIOSGA031963	XP_006648417.1 TBCC domain-containing protein 1-like
TCONS_00031596	-0.509741	-1.03775	-0.66497	BGIOSGA011613	XP_006662032.1PREDICTED: uncharacterized protein LOC102702041
TCONS_00022669	-0.509482	0.235629	-0.0420267	BGIOSGA023944	XP_006657196.1 vestitone reductase-like
TCONS_00020835	-0.509249	-1.75587	-1.31249	BGIOSGA012591	XP_004965538.1E3 ubiquitin-protein ligase Os06g0535400
TCONS_00000883	-0.509218	-0.614969	-0.223517	BGIOSGA014781	XP_012702637.160S ribosomal protein L23A
TCONS_00023135	-0.508867	-0.140799	-0.185608	BGIOSGA013394	XP_006657456.1 ethylene-insensitive protein 2-like
TCONS_00000335	-0.508739	0.488452	0.376712	BGIOSGA002911	XP_006643852.2 nuclear distribution protein nudF-like
TCONS_00015144	-0.508234	0.504693	1.13556	BGIOSGA036952	XP_006652987.1 F-box/kelch-repeat protein At5g15710
TCONS_00020122	-0.507577	-1.71521	-0.761714	BGIOSGA012481	XP_015693413.1 GDSL esterase/lipase APG-like
TCONS_00011333	-0.507571	0.526225	-0.924981	BGIOSGA013947	XP_004981047.1uncharacterized protein At4g15970
TCONS_00011475	-0.507112	0.618711	0.313204	BGIOSGA031491	XP_006649301.1PREDICTED: uncharacterized protein LOC102721525
TCONS_00016433	-0.507045	1.6681	0.503704	BGIOSGA029244	XP_015692316.1 amino acid transporter ANTL2-like
TCONS_00018250	-0.506762	0.839299	0.104463	BGIOSGA034439	XP_006654741.1 histone-lysine N-methyltransferase, H3 lysine-9 specific SUVH3-like
TCONS_00027495	-0.506567	0.320604	0.355486	BGIOSGA022518	XP_006659905.1 nucleolin 1
TCONS_00032185	-0.506459	-0.446761	-0.764574	BGIOSGA006965	XP_006662343.1 probable mannan synthase 2
TCONS_00007725	-0.506454	-0.740702	-0.943467	BGIOSGA028733	XP_015688543.1 protein TSS
TCONS_00023128	-0.506449	0.901998	-1.41816	BGIOSGA038447	XP_006657453.1 peroxisomal (S)-2-hydroxy-acid oxidase GLO5
TCONS_00017424	-0.506321	0.331897	-0.0148325	BGIOSGA018691	XP_004955547.1eukaryotic translation initiation factor 3 subunit I
TCONS_00003682	-0.50614	-1.81279	-0.478457	BGIOSGA001522	XP_006645946.1PREDICTED: uncharacterized protein LOC102705398
TCONS_00022993	-0.506137	-0.10412	-0.345501	BGIOSGA025025	XP_006657403.2 nipped-B-like protein B
TCONS_00030698	-0.505998	-1.4421	0.148958	BGIOSGA032518	XP_003573733.2uncharacterized protein LOC100841347
TCONS_00016641	-0.505831	-1.22015	-0.0619284	#N/A	#N/A
TCONS_00021205	-0.505753	-0.943466	-0.00742874	BGIOSGA023426	XP_006657228.2 thioredoxin O, mitochondrial
TCONS_00012580	-0.505633	-0.685025	-0.594842	BGIOSGA019930	XP_015690849.1 vacuolar protein-sorting-associated protein 37 homolog 1-like isoform X2
TCONS_00027881	-0.505593	-0.778906	-1.49549	#N/A	#N/A
TCONS_00038397	-0.505419	-2.23925	-0.974032	BGIOSGA005995	XP_002448198.2heavy metal-associated isoprenylated plant protein 34
TCONS_00020598	-0.505362	-0.805637	0.837342	BGIOSGA023829	XP_015694480.1 probable mediator of RNA polymerase II transcription subunit 26b
TCONS_00032770	-0.505288	0.8296	0.187645	BGIOSGA008174	XP_006662634.1 inositol-tetrakisphosphate 1-kinase 5
TCONS_00007378	-0.505037	-0.408564	-0.53729	BGIOSGA007099	XP_015688460.1 translation factor GUF1 homolog, chloroplastic
TCONS_00014645	-0.504772	0.109151	-0.0928314	BGIOSGA017355	XP_006653617.1 WD repeat-containing protein 44-like
TCONS_00021581	-0.504741	-0.247115	-0.588539	BGIOSGA028368	XP_003557199.1nudix hydrolase 19, chloroplastic
TCONS_00005542	-0.504361	0.165677	-0.0379639	BGIOSGA030433	XP_009414815.1 60S ribosomal protein L27a-3-like
TCONS_00011016	-0.504322	-1.14644	0.211373	BGIOSGA001368	XP_010229377.1transcription factor HHO5
TCONS_00013204	-0.504317	-0.38983	0.182768	BGIOSGA009783	XP_006650612.1PREDICTED: uncharacterized protein LOC102702080
TCONS_00003333	-0.504282	-4.26186	-1.271	BGIOSGA001883	XP_002457676.1lamin-like protein
TCONS_00028087	-0.504106	#NA	-2.36788	BGIOSGA015763	XP_006664931.1 glyoxylate/hydroxypyruvate reductase HPR3-like
TCONS_00022859	-0.504072	-0.461065	-1.50748	BGIOSGA002073	XP_006657304.1 probable cation transporter HKT9
TCONS_00012628	-0.504061	0.278425	0.312557	BGIOSGA010345	XP_015691313.1 lysine--tRNA ligase
TCONS_00029811	-0.503468	-1.99469	-0.494937	BGIOSGA030004	XP_004956563.1glutathione S-transferase T3

TCONS_00010939	-0.503215	-1.67825	-0.858626	BGIOSGA027472	XP_006650520.1PREDICTED: uncharacterized protein LOC102721532
TCONS_00008011	-0.503095	1.03037	0.378609	BGIOSGA032485	XP_015688595.1 phenolic glucoside malonyltransferase 1-like
TCONS_00019698	-0.502959	-0.401456	0.0660943	BGIOSGA021436	XP_006655530.1 delta-1-pyrroline-5-carboxylate dehydrogenase 12A1, mitochondrial
TCONS_00031171	-0.502895	0.174814	0.210481	BGIOSGA035727	XP_015697000.1 E3 ubiquitin-protein ligase RBBP6-like
TCONS_00009064	-0.502676	-1.58065	-0.944353	BGIOSGA002819	XP_006648137.1 calmodulin-binding receptor-like cytoplasmic kinase 2
TCONS_00030557	-0.5024	-0.311068	-0.303914	BGIOSGA029101	XP_003559281.1uncharacterized protein LOC100826907
TCONS_00004630	-0.502226	-0.480296	0.11444	BGIOSGA000587	XP_006644915.1 probable inactive serine/threonine-protein kinase scy1
TCONS_00024362	-0.502139	-1.80372	0.179853	BGIOSGA028853	XP_015695406.1 probable galacturonosyltransferase 7 isoform X1
TCONS_00013137	-0.501928	-0.328172	-0.564051	BGIOSGA014680	XP_006651794.1 GEM-like protein 1
TCONS_00005550	-0.50165	-0.568012	-0.629752	BGIOSGA010489	XP_003563375.1protein transport protein Sec61 subunit gamma
TCONS_00023376	-0.501567	-0.394932	-1.97964	BGIOSGA013269	XP_006657574.1PREDICTED: uncharacterized protein LOC102720060
TCONS_00009082	-0.501567	-0.9355	-0.979645	BGIOSGA021467	XP_006648162.1 signal peptidase complex catalytic subunit SEC11C-like isoform X1
TCONS_00036507	-0.501435	-0.731426	-0.129347	BGIOSGA013366	XP_006664792.2 beta-glucuronosyltransferase GlcAT14A
TCONS_00033842	-0.501399	-0.510618	-1.16271	BGIOSGA037430	XP_006663048.1 DNA repair protein RAD51 homolog A
TCONS_00019002	-0.501352	-0.189993	-0.706608	BGIOSGA021293	XP_015693103.1 proline-, glutamic acid- and leucine-rich protein 1-like
TCONS_00001369	-0.501341	0.529184	0.637319	BGIOSGA003999	XP_003566889.1centromere protein C isoform X3
TCONS_00000387	-0.501115	-1.54302	-0.820316	BGIOSGA002963	XP_006645616.1 uncharacterized WD repeat-containing protein alr2800
TCONS_00020096	-0.500578	0.125778	0.222279	BGIOSGA022276	XP_006656624.2 protein arginine N-methyltransferase PRMT10
TCONS_00012577	-0.500431	1.31962	-0.61342	BGIOSGA035894	XP_003562350.1protein DETOXIFICATION 40
TCONS_00014471	-0.500167	0.288022	0.154675	BGIOSGA026762	XP_006652430.1 26S proteasome non-ATPase regulatory subunit 6
TCONS_00034679	-0.500007	0.17259	-0.0559988	#N/A	#N/A
TCONS_00022424	-0.499909	-0.0919145	0.436571	BGIOSGA021041	XP_006656163.1 nuclear poly(A) polymerase 4 isoform X2
TCONS_00022773	-0.499892	-0.179939	-0.474565	BGIOSGA009399	XP_006657250.1 solanesyl-diphosphate synthase 1, mitochondrial
TCONS_00026297	-0.499884	0.330986	-1.02972	#N/A	#N/A
TCONS_00002292	-0.49988	-0.595875	-0.264432	BGIOSGA020627	XP_006646536.1 probable receptor-like protein kinase At1g11050
TCONS_00008268	-0.499787	0.904419	0.872926	BGIOSGA014918	XP_004952921.1heavy metal-associated isoprenylated plant protein 47
TCONS_00028189	-0.499625	-0.30478	-0.819689	BGIOSGA026796	XP_006659504.1 signal recognition particle receptor subunit alpha
TCONS_00024969	-0.499474	-1.99043	-3.17103	BGIOSGA031554	XP_006664959.1 protein CDI-like
TCONS_00005797	-0.499395	0.509364	-2.98515	BGIOSGA007912	XP_004951276.1uncharacterized protein LOC101764619
TCONS_00008339	-0.49919	-0.135652	0.377125	BGIOSGA006086	XP_004953030.1eukaryotic translation initiation factor 2A
TCONS_00033195	-0.499108	-0.804071	-0.74436	BGIOSGA021845	XP_015697771.1 putative disease resistance RPP13-like protein 2
TCONS_00010377	-0.499048	0.689515	-0.986031	#N/A	#N/A
TCONS_00036984	-0.498971	-1.94983	-1.72251	BGIOSGA007558	XP_006663954.1 ribulose bisphosphate carboxylase small chain, chloroplastic-like
TCONS_00035046	-0.498935	-2.10858	-1.02696	BGIOSGA033808	XP_006663526.2 putative wall-associated receptor kinase-like 16
TCONS_00021014	-0.498504	0.601389	-0.180046	BGIOSGA030362	XP_006656231.1 protein transport protein SFT2
TCONS_00004225	-0.498099	-1.24434	-0.805211	BGIOSGA020926	XP_006644542.1 peptidyl-tRNA hydrolase, mitochondrial
TCONS_00012896	-0.498002	-0.0773015	-1.4037	BGIOSGA010087	XP_006650392.1 deoxyuridine 5'-triphosphate nucleotidohydrolase
TCONS_00015194	-0.497908	-1.37072	0.10402	BGIOSGA017376	XP_015692401.1 tRNA-specific adenosine deaminase 1 isoform X1
TCONS_00007072	-0.497722	-0.354987	-0.223317	BGIOSGA003070	XP_015689626.1 diphthine--ammonia ligase
TCONS_00028485	-0.497682	-0.592115	0.523096	BGIOSGA005326	XP_006659713.1 ABC transporter F family member 1 isoform X1
TCONS_00010911	-0.497638	0.525745	-0.393618	#N/A	XP_015690060.1 serine/threonine-protein kinase SMG1-like
TCONS_00016871	-0.497567	-1.38678	0.272474	BGIOSGA003668	XP_004960078.1transcription factor SRM1
TCONS_00026734	-0.497236	0.153264	-0.962937	BGIOSGA021674	XP_006659475.1 12-oxophytodienoate reductase 7
TCONS_00010576	-0.496977	-1.97153	-0.211051	BGIOSGA031257	XP_006650328.1 organic cation/carnitine transporter 7-like
TCONS_00009401	-0.49656	-0.294471	-0.726089	BGIOSGA035533	XP_006649477.1 F-box/kelch-repeat protein At1g74510-like
TCONS_00027149	-0.496536	0.127821	0.109617	BGIOSGA031309	XP_006659702.1 protein HESO1-like
TCONS_00020218	-0.496513	-1.53468	-0.529438	BGIOSGA022405	XP_015694301.1PREDICTED: uncharacterized protein LOC102714471
TCONS_00001717	-0.496399	-3.22749	-0.855377	BGIOSGA026004	XP_015696661.1 chlorophyll a-b binding protein of LHCII type 1-like isoform X2
TCONS_00028411	-0.496098	0.300463	-0.158249	BGIOSGA026552	XP_015695998.1PREDICTED: uncharacterized protein LOC102719038
TCONS_00002437	-0.496071	-0.389182	0.289228	BGIOSGA025045	XP_006645249.1 CBS domain-containing protein CBSCBSPB1-like
TCONS_00002083	-0.49597	-2.40177	0.176138	BGIOSGA034486	XP_006647656.1 cytochrome P450 86A2-like
TCONS_00010263	-0.495389	0.0569312	-1.12078	#N/A	#N/A
TCONS_00029264	-0.495051	0.813481	-0.238196	BGIOSGA002301	XP_004957179.1transcription factor bHLH130 isoform X2

TCONS_00004873	-0.494922	-1.81813	-2.99642	BGIOSGA000351	XP_015695030.1 putative exosome complex component rrp40
TCONS_00022748	-0.494876	0.633019	0.642166	BGIOSGA000676	XP_006657236.1 conserved oligomeric Golgi complex subunit 7
TCONS_00027114	-0.494705	-1.40337	-1.412	BGIOSGA026516	XP_015695745.1PREDICTED: uncharacterized protein LOC107303368
TCONS_00033643	-0.494686	-0.571586	-0.298461	BGIOSGA007007	XP_015697693.1 probable protein S-acyltransferase 23
TCONS_00011079	-0.494519	-0.0690934	-1.02297	BGIOSGA013679	NP_001149840.1deoxyribodipyrimidine photolyase
TCONS_00023852	-0.494328	-0.0244654	0.158078	BGIOSGA001458	XP_006658649.1 cysteine-rich receptor-like protein kinase 15 isoform X1
TCONS_00003947	-0.49425	0.0554897	0.809256	BGIOSGA027024	NP_001144724.2uncharacterized LOC100277766 precursor
TCONS_00029654	-0.494202	#NA	-0.413256	BGIOSGA030175	XP_006660441.1 probable 2-oxoglutarate-dependent dioxygenase At3g49630
TCONS_00013724	-0.493807	-0.704716	0.229427	BGIOSGA024506	XP_015691370.1 ethylene receptor 2
TCONS_00006393	-0.493601	-0.560112	-0.812939	BGIOSGA024440	XP_015689444.1 glucose-6-phosphate 1-dehydrogenase, cytoplasmic isoform-like
TCONS_00021756	-0.493383	-0.346052	-0.112089	BGIOSGA009196	XP_006655862.1PREDICTED: uncharacterized protein LOC102701454
TCONS_00021839	-0.493358	-0.344564	-0.366217	BGIOSGA021679	XP_015693500.1 alternative NAD(P)H-ubiquinone oxidoreductase C1, chloroplastic/mitochondrial
TCONS_00002729	-0.493172	1.05075	0.674705	BGIOSGA002493	XP_006645447.1PREDICTED: uncharacterized protein LOC102722913 isoform X1
TCONS_00020693	-0.493047	-3.40546	-2.0011	BGIOSGA007133	XP_006659038.2 alcohol dehydrogenase
TCONS_00017138	-0.492905	-0.161891	-0.120371	BGIOSGA019184	XP_004960274.1anaphase-promoting complex subunit 7 isoform X1
TCONS_00031102	-0.492804	1.45221	0.805647	BGIOSGA032918	XP_015697107.1 chitinase 2-like
TCONS_00033266	-0.492462	-1.06499	-0.69246	BGIOSGA029043	XP_006647152.1 uridine kinase-like protein 3
TCONS_00021499	-0.492246	-4.40648	-2.0031	#N/A	#N/A
TCONS_00001078	-0.492203	-2.72627	-1.49669	BGIOSGA006986	XP_006644296.1 ABC transporter B family member 4-like
TCONS_00016734	-0.492078	-1.34779	-0.833592	BGIOSGA014229	XP_006652915.1 ubiquitin carboxyl-terminal hydrolase 22
TCONS_00011573	-0.491997	-3.40679	-1.41876	BGIOSGA002203	XP_002465848.1RING-H2 finger protein ATL1
TCONS_00015962	-0.491997	-0.79208	-1.35986	BGIOSGA015020	XP_015692165.1 putative 12-oxophytodienoate reductase 11 isoform X2
TCONS_00027147	-0.491909	0.43682	0.544038	BGIOSGA029191	XP_006659699.1 dihydroxy-acid dehydratase, chloroplastic
TCONS_00027544	-0.491741	0.708365	0.16557	BGIOSGA028177	XP_003573527.1equilibrative nucleotide transporter 1
TCONS_00024280	-0.491274	-0.435681	0.625804	BGIOSGA026324	XP_003563386.1nucleolar protein 56
TCONS_00030479	-0.491216	#NA	-1.63054	BGIOSGA031202	XP_006660929.1 auxin-responsive protein SAUR36-like
TCONS_00025346	-0.491196	-0.0486486	1.07749	BGIOSGA024084	XP_006657841.1 probable ADP-ribosylation factor GTPase-activating protein AGD5 isoform X2
TCONS_00001905	-0.490866	-0.215595	0.139369	BGIOSGA040479	XP_015690078.1 probable alpha-mannosidase I MNS4
TCONS_00006450	-0.490849	-0.0918535	0.274882	BGIOSGA008618	XP_002452442.1nucleotide-sugar uncharacterized transporter 3
TCONS_00023468	-0.490732	-1.52898	-0.873715	BGIOSGA026141	XP_008651509.1senescence-associated-like protein isoform X2
TCONS_00008619	-0.490692	-1.78351	0.97137	BGIOSGA005805	XP_006647717.2 dnaJ homolog subfamily B member 14-like
TCONS_00019307	-0.490289	-0.553566	-1.12524	BGIOSGA035427	XP_006654413.2 uncharacterized hydrolase YugF
TCONS_00023023	-0.490199	-0.0871278	-0.423221	BGIOSGA014413	XP_021307823.1putative F-box/FBD/LRR-repeat protein At4g03220
TCONS_00028615	-0.489997	-0.140426	-0.310721	BGIOSGA008103	XP_006660457.1 protein C2-DOMAIN ABA-RELATED 11
TCONS_00031924	-0.489871	0.290972	0.991007	BGIOSGA016117	XP_010918979.1 protein MICRORCHIDIA 2 isoform X2
TCONS_00012901	-0.489583	-1.33996	-0.821881	BGIOSGA004417	XP_006650394.1 protein TRIGALACTOSYLDIACYLGLYCEROL 3, chloroplastic
TCONS_00008721	-0.489459	#NA	-1.39388	BGIOSGA015772	XP_006657780.1 isocitrate lyase
TCONS_00035644	-0.489383	0.290359	-0.822573	BGIOSGA020673	XP_004977257.1zinc finger protein STAR3
TCONS_00004350	-0.489383	1.39727	-0.0102002	BGIOSGA026166	XP_006644668.1 UDP-glycosyltransferase 88F4-like
TCONS_00005061	-0.489317	-1.08824	-0.486802	BGIOSGA000156	XP_006645300.1 basic 7S globulin 2-like
TCONS_00025260	-0.489226	0.321525	0.781968	BGIOSGA016358	XP_006657798.2 glucan endo-1,3-beta-glucosidase 12-like
TCONS_00037657	-0.488981	-0.582322	-0.125541	BGIOSGA000187	XP_006646880.1 DNA-binding protein BIN4
TCONS_00005142	-0.488785	0.67663	1.23625	BGIOSGA033440	XP_006646715.1 putative box C/D snoRNA protein SPCC613.07
TCONS_00034917	-0.488744	0.589116	0.895111	BGIOSGA035892	XP_015697667.1PREDICTED: uncharacterized protein LOC102719052, partial
TCONS_00031263	-0.488623	-0.80632	-0.322202	BGIOSGA033081	XP_006661832.1PREDICTED: uncharacterized protein LOC102712664 isoform X1
TCONS_00024163	-0.48858	-2.63507	-1.64652	BGIOSGA026200	XP_004958426.1uncharacterized protein LOC101776974
TCONS_00025386	-0.48854	-0.352246	-0.349318	BGIOSGA021135	XP_006657876.1 CMP-sialic acid transporter 2 isoform X1
TCONS_00034106	-0.48843	0.0377245	-1.05937	BGIOSGA018401	XP_015697857.1 probable pyridoxal 5'-phosphate synthase subunit PDX1.1
TCONS_00028321	-0.487636	1.38028	0.0850259	BGIOSGA026650	XP_006660294.1 isoamylase 1, chloroplastic
TCONS_00007280	-0.487422	0.471991	0.38035	BGIOSGA015965	XP_006648272.2 ribose-phosphate pyrophosphokinase 1, chloroplastic
TCONS_00012249	-0.487368	-0.0632317	0.0181964	BGIOSGA001682	XP_006650065.1 putative ubiquitin-like-specific protease 1B
TCONS_00018902	-0.486969	-1.9414	-0.822905	BGIOSGA028032	XP_006645169.1 50S ribosomal protein L13

TCONS_00002750	-0.486965	0.0608507	0.0317969	BGIOSGA002467	XP_021313139.1protein MAO HUZI 4, chloroplastic
TCONS_00009901	-0.486633	0.949041	0.442456	BGIOSGA023677	XP_004987034.1BRCT domain-containing protein At4g02110 isoform X2
TCONS_00016174	-0.486618	-1.52132	0.338441	BGIOSGA014802	XP_004976091.1serine/arginine-rich splicing factor 6
TCONS_00022248	-0.48596	-1.50413	-0.291058	BGIOSGA008184	XP_015694087.1 protein MICRORCHIDIA 6-like isoform X2
TCONS_00028561	-0.485802	-0.920677	-0.0301167	BGIOSGA030210	XP_015696597.1 ankyrin-1-like
TCONS_00026892	-0.485441	0.136354	0.0630786	BGIOSGA008519	XP_015696041.1 glycerophosphodiester phosphodiesterase GDPDL3
TCONS_00036267	-0.484905	-1.18528	-0.0615964	BGIOSGA037628	XP_015698334.1PREDICTED: uncharacterized protein LOC107305378, partial
TCONS_00015952	-0.484849	0.127051	0.279142	BGIOSGA015028	XP_006653436.2 cleft lip and palate transmembrane protein 1 homolog
TCONS_00003431	-0.484587	-0.0060928	0.0171337	BGIOSGA012337	XP_006645818.1 UDP-glucuronic acid decarboxylase 2
TCONS_00001705	-0.484369	-0.353357	-1.20812	BGIOSGA004338	XP_006644625.1 alliin lyase-like
TCONS_00012129	-0.484192	-0.635012	-0.768098	BGIOSGA010880	XP_006649970.1PREDICTED: uncharacterized protein LOC102701065
TCONS_00001531	-0.484159	0.469285	0.211032	BGIOSGA004078	XP_023156542.1ribokinase isoform X1
TCONS_00016877	-0.484094	-1.53901	-0.671132	BGIOSGA014434	XP_015692380.1 HBS1-like protein
TCONS_00015792	-0.483869	0.89865	0.623598	BGIOSGA015190	XP_006652218.1 membrane-anchored ubiquitin-fold protein 4
TCONS_00005019	-0.483376	0.550885	1.35786	BGIOSGA006075	XP_010232773.1carbonyl reductase [NADPH] 1
TCONS_00013518	-0.483327	-1.68035	-0.105727	#N/A	#N/A
TCONS_00018617	-0.483181	-0.920371	-0.950266	BGIOSGA018817	XP_015692754.1 pentatricopeptide repeat-containing protein At4g35850, mitochondrial
TCONS_00033572	-0.483051	-0.235208	1.61379	BGIOSGA032512	XP_006665027.1 alpha-mannosidase-like
TCONS_00013571	-0.482827	-0.250564	0.126347	BGIOSGA009405	XP_015691016.1PREDICTED: uncharacterized protein LOC102723037
TCONS_00029611	-0.482821	-0.893422	0.112551	BGIOSGA006444	XP_015696597.1 ankyrin-1-like
TCONS_00028647	-0.482796	-0.2976	-0.596942	BGIOSGA030399	XP_003581007.1uncharacterized protein LOC100842516
TCONS_00020268	-0.482579	-0.121871	-0.436199	BGIOSGA023208	XP_004954152.126S proteasome regulatory subunit 7A
TCONS_00015045	-0.482566	-2.10981	-0.43623	BGIOSGA035289	XP_006652901.2 3-oxoacyl-[acyl-carrier-protein] synthase III, chloroplastic
TCONS_00029508	-0.482367	-1.41416	-0.22295	BGIOSGA023425	XP_006660976.1 ALA-interacting subunit 1-like
TCONS_00034575	-0.482007	-0.838322	-0.0289479	BGIOSGA034241	XP_015697607.1 glycerophosphodiester phosphodiesterase GDPD4
TCONS_00036446	-0.481996	-0.56081	-0.151397	BGIOSGA014650	XP_006664216.1 protein YABBY 6
TCONS_00031468	-0.481908	0.274659	0.478606	BGIOSGA011701	XP_004982595.1protein HLB1
TCONS_00028400	-0.481827	1.1221	0.618136	BGIOSGA000219	XP_003606833.1transmembrane protein, putative
TCONS_00028302	-0.481729	0.191391	0.0138025	BGIOSGA007545	XP_006660282.2 serrate RNA effector molecule-like
TCONS_00007547	-0.481671	-1.12512	0.152638	BGIOSGA007743	XP_006647036.1 peptidyl-prolyl cis-trans isomerase FKBP16-1, chloroplastic
TCONS_00014424	-0.481671	-0.219368	0.042356	BGIOSGA032170	XP_006653510.1 ras-related protein Rab-2-B
TCONS_00030565	-0.481603	-1.11255	-0.780843	BGIOSGA022642	XP_006661570.1PREDICTED: uncharacterized protein LOC102718868
TCONS_00013187	-0.481478	-2.02104	-3.12454	BGIOSGA025019	XP_015690716.1 two pore potassium channel a isoform X1
TCONS_00005965	-0.481458	-2.75195	-1.37738	BGIOSGA008101	XP_015689400.1 RHOMBOID-like protein 9, chloroplastic
TCONS_00004922	-0.481078	-0.147153	-0.969768	BGIOSGA000299	XP_006645192.2PREDICTED: uncharacterized protein LOC102715626 isoform X1
TCONS_00020261	-0.480717	1.12696	0.558673	BGIOSGA013446	XP_004961229.1homeobox-DDT domain protein RLT2
TCONS_00009551	-0.480444	-1.43873	-0.737537	BGIOSGA037665	XP_015691287.1 heparanase-like protein 2 isoform X2
TCONS_00011982	-0.480079	0.491842	0.288525	BGIOSGA027456	XP_015690223.1 tropinone reductase homolog At5g06060-like
TCONS_00034221	-0.47991	-2.21674	-1.6324	BGIOSGA018773	XP_015698574.1PREDICTED: uncharacterized protein LOC102710259
TCONS_00007149	-0.479866	1.06527	0.237708	BGIOSGA009334	XP_006648159.1 probable prolyl 4-hydroxylase 3
TCONS_00020120	-0.479711	-0.356388	-1.76267	BGIOSGA025373	XP_004964857.1uncharacterized protein LOC101757286
TCONS_00014898	-0.479545	0.617506	0.162483	BGIOSGA003409	XP_004976753.1probable ATP-dependent RNA helicase YTHDC2 isoform X4
TCONS_00011677	-0.479336	0.600045	0.150923	BGIOSGA012825	NP_001149295.140S ribosomal protein SA
TCONS_00007229	-0.47925	0.202978	0.287543	BGIOSGA019187	XP_006648224.1 ATP-dependent helicase BRM
TCONS_00036759	-0.47913	-0.434764	-0.322948	BGIOSGA010816	XP_006663859.1 fatty acid amide hydrolase-like
TCONS_00025248	-0.478993	0.985102	-0.764452	BGIOSGA021835	XP_002462959.1aspartic proteinase nepenthesin-1
TCONS_00026427	-0.478897	0.278177	0.459312	BGIOSGA021589	XP_006659342.1 transcription initiation factor IIE subunit alpha
TCONS_00013951	-0.478763	0.34068	0.427735	#N/A	#N/A
TCONS_00001058	-0.478344	0.0378101	0.277188	BGIOSGA003675	XP_002436514.1DNA-damage-repair/toleration protein DRT111, chloroplastic
TCONS_00020628	-0.478344	-2.42022	-0.962565	BGIOSGA030471	XP_006659170.1 pyrophosphate--fructose 6-phosphate 1-phosphotransferase subunit alpha-like isoform X2
TCONS_00009310	-0.478288	-2.36168	-0.805938	BGIOSGA011850	XP_015690435.1 beta-glucuronosyltransferase GlcAT14A-like
TCONS_00000213	-0.477763	0.436491	#NA	BGIOSGA002790	XP_006643761.1 very-long-chain (3R)-3-hydroxyacyl-CoA dehydratase PASTICCINO 2A
TCONS_00036765	-0.477736	0.606576	-0.624784	BGIOSGA034908	XP_015698230.1 dystrophia myotonica WD repeat-containing protein-like
TCONS_00034425	-0.477575	-0.400885	-0.298764	BGIOSGA034384	XP_006662785.1 F-box/WD-40 repeat-containing protein At3g52030
TCONS_00013919	-0.477451	-2.35561	-1.43316	BGIOSGA016094	XP_004960459.1protein GOS9

TCONS_00025914	-0.477406	-1.6102	-0.0347571	BGIOSGA027987	XP_015695707.1 transcription factor MYB1R1-like isoform X2
TCONS_00020233	-0.477214	-0.745192	-0.0495362	BGIOSGA019076	XP_015694292.1PREDICTED: LOW QUALITY PROTEIN: uncharacterized protein LOC102718371
TCONS_00036762	-0.477098	0.0998627	-0.155789	BGIOSGA003268	XP_006644067.2 pentatricopeptide repeat-containing protein At1g80270, mitochondrial-like
TCONS_00009022	-0.477094	0.456189	0.077872	BGIOSGA024393	XP_004954316.1ankyrin repeat domain-containing protein 13C-B
TCONS_00021247	-0.477021	1.34438	-0.513357	BGIOSGA031637	XP_009618235.1 polyubiquitin-like isoform X1
TCONS_00029645	-0.476738	0.626941	0.33851	BGIOSGA034120	XP_006660444.2 DNA-directed RNA polymerase 1B, mitochondrial-like
TCONS_00004880	-0.476684	-0.0111213	-0.410857	BGIOSGA027875	XP_004970880.1fructokinase-1
TCONS_00025522	-0.476625	-1.29471	-0.494619	BGIOSGA028795	XP_006658804.2 probable GTP diphosphokinase RSH3, chloroplastic
TCONS_00016338	-0.476251	-0.221869	-1.94328	BGIOSGA007382	XP_021319113.1probable metal-nicotianamine transporter YSL16
TCONS_00020872	-0.476225	-0.0842986	0.0192181	BGIOSGA000897	XP_002437145.140S ribosomal protein S24-1
TCONS_00037050	-0.476177	-0.208019	-0.0964884	BGIOSGA037267	XP_006663975.1 phosphatidylinositol 3,4,5-trisphosphate 3-phosphatase and protein-tyrosine-phosphatase PTEN2A-like
TCONS_00019987	-0.476017	0.869398	-0.489455	BGIOSGA014439	XP_006655704.1 binding partner of ACD11 1-like
TCONS_00031309	-0.475964	1.0146	0.569124	BGIOSGA033284	XP_008681005.1uncharacterized LOC100192009 isoform X1
TCONS_00007279	-0.475839	-0.0121864	-0.794826	BGIOSGA007204	XP_004952032.4phosphatidate cytidylyltransferase 4, chloroplastic
TCONS_00029945	-0.475682	-0.0666863	0.242456	BGIOSGA011194	XP_006660589.1 probable alpha,alpha-trehalose-phosphate synthase [UDP-forming] 9
TCONS_00025933	-0.475679	0.184887	0.22232	BGIOSGA028006	XP_006659124.1 DNA topoisomerase 1-like
TCONS_00013777	-0.475671	-1.38059	-1.07735	BGIOSGA015538	XP_022683954.1uncharacterized protein LOC101758198
TCONS_00007809	-0.475406	-0.0127316	-0.255332	BGIOSGA032637	XP_006648549.1PREDICTED: uncharacterized protein LOC102715371
TCONS_00037341	-0.475331	-0.154262	-0.0499231	BGIOSGA033900	NP_001148142.2triacylglycerol lipase precursor
TCONS_00023152	-0.475258	-2.61976	-0.421052	BGIOSGA025189	XP_010228374.1uncharacterized LOC104581721 isoform X1
TCONS_00014995	-0.475202	0.629755	-0.10221	BGIOSGA027539	XP_010493589.1 polyubiquitin-like
TCONS_00025321	-0.474894	-0.698996	0.0551309	BGIOSGA002431	XP_021307999.1histone-lysine N-methyltransferase 2E
TCONS_00036810	-0.474789	-0.339237	-1.22878	BGIOSGA038481	XP_003578817.1thioredoxin M-type, chloroplastic
TCONS_00011988	-0.474355	1.22473	0.134385	BGIOSGA019978	XP_004984930.1uncharacterized protein LOC101768536
TCONS_00034048	-0.474106	0.599925	-0.0961341	BGIOSGA002293	XP_006663114.1 DEAD-box ATP-dependent RNA helicase 40-like
TCONS_00019998	-0.473998	0.865204	0.200616	BGIOSGA030637	XP_006656565.1 pentatricopeptide repeat-containing protein At1g08070, chloroplastic-like
TCONS_00025413	-0.473966	-0.475591	-0.876781	BGIOSGA024014	XP_006657883.1 UDP-galactose transporter 2-like
TCONS_00014008	-0.473593	-0.91816	-0.211709	BGIOSGA016157	XP_006652181.1 prolyl 4-hydroxylase 1
TCONS_00002842	-0.473318	0.634013	0.393635	BGIOSGA001568	XP_006643766.1 histone H2B.5
TCONS_00033197	-0.472951	-0.248584	-0.373763	BGIOSGA034034	XP_015698140.1 disease resistance protein RPP13-like
TCONS_00007554	-0.472894	1.14082	0.190973	BGIOSGA008905	XP_002453497.1EIN3-binding F-box protein 1
TCONS_00036556	-0.471815	-5.52146	-3.40434	BGIOSGA034619	XP_006663745.1 probable chalcone--flavonone isomerase 3 isoform X1
TCONS_00026902	-0.471652	-2.92684	-1.56566	BGIOSGA029552	XP_004973859.1uncharacterized protein LOC101774238
TCONS_00013015	-0.471524	-0.106491	-0.232009	BGIOSGA018780	XP_015690840.1 probable ethylene response sensor 1
TCONS_00009159	-0.471452	-1.14886	-0.516611	BGIOSGA031196	XP_015691044.1 probable cytokinin riboside 5'-monophosphate phosphoribohydrolase LOGL3
TCONS_00026668	-0.471366	-1.42246	-0.242621	BGIOSGA018187	XP_006659442.1 protein ENHANCED DISEASE RESISTANCE 2
TCONS_00004502	-0.471181	-0.17345	-0.231501	BGIOSGA013359	XP_015699359.1 inositol hexakisphosphate and diphosphoinositol-pentakisphosphate kinase 2-like
TCONS_00030720	-0.471118	-1.0182	-0.128407	BGIOSGA004599	XP_015691642.1 subtilisin-like protease SBT3.9
TCONS_00029086	-0.47105	0.0998406	-0.957875	BGIOSGA006945	NP_001141336.1Photosystem I chlorophyll a/b-binding protein 6 chloroplastic
TCONS_00010349	-0.470793	-0.553486	-0.841054	BGIOSGA012925	XP_002465062.1uncharacterized protein LOC8066230
TCONS_00012049	-0.470639	1.08583	-0.179116	BGIOSGA029675	XP_006649891.1 mitogen-activated protein kinase 5
TCONS_00021704	-0.470636	-1.38875	-0.672388	BGIOSGA021819	XP_015693465.1 ALBINO3-like protein 2, chloroplastic
TCONS_00020768	-0.470613	0.42824	0.0939755	BGIOSGA002630	XP_006656110.1 protein decapping 5-like
TCONS_00010184	-0.470456	-1.25785	-1.69425	BGIOSGA012746	XP_006651435.1PREDICTED: uncharacterized protein At4g17910 isoform X1
TCONS_00018896	-0.470273	-0.292632	0.379183	BGIOSGA002054	XP_006654174.1 WPP domain-interacting tail-anchored protein 1-like isoform X2
TCONS_00010781	-0.469986	-0.527269	-0.509752	BGIOSGA030597	XP_006650464.1 uncharacterized membrane protein At4g09580
TCONS_00005191	-0.469975	-1.53927	-1.45322	BGIOSGA019042	XP_006645404.1 pectin acetylesterase 5-like
TCONS_00006461	-0.46937	-0.678238	0.765878	BGIOSGA008633	XP_006647523.1 two-component response regulator-like PRR1
TCONS_00028254	-0.469008	-2.51121	-0.832755	BGIOSGA007841	XP_004973827.1wall-associated receptor kinase 3 isoform X1
TCONS_00019351	-0.468936	-4.76699	-1.90705	BGIOSGA023574	XP_006655351.1 protein NRT1/ PTR FAMILY 5.10-like
TCONS_00002383	-0.468882	-1.78667	-0.247431	BGIOSGA000285	XP_015694821.1 histone deacetylase HDT2-like
TCONS_00032571	-0.468852	0.42273	0.0927381	BGIOSGA022550	XP_006662516.1 DNA excision repair protein ERCC-1

TCONS_00024666	-0.468817	-0.862662	-1.1342	BGIOSGA035835	XP_003577366.12-methyl-6-phytyl-1,4-hydroquinone methyltransferase 2, chloroplastic
TCONS_00020068	-0.468227	0.90521	-0.416659	#N/A	#N/A
TCONS_00016442	-0.468215	-0.381522	-0.801627	BGIOSGA024828	XP_004976523.1dof zinc finger protein 1 isoform X2
TCONS_00006663	-0.468169	-0.921864	-0.864801	BGIOSGA008832	XP_006648945.1PREDICTED: uncharacterized protein LOC102720683
TCONS_00032188	-0.468081	-0.540414	-1.20936	BGIOSGA031946	XP_006661765.1 pectinesterase 31
TCONS_00003126	-0.468079	-1.39132	-0.805175	BGIOSGA030655	XP_006645650.2 probable inactive receptor kinase At4g23740
TCONS_00031435	-0.468067	0.202478	0.26992	BGIOSGA033249	XP_004982657.1protein GRAVITROPIC IN THE LIGHT 1
TCONS_00002022	-0.467993	0.444997	-0.414378	BGIOSGA033918	XP_002456508.1leucine-rich repeat protein 1
TCONS_00019012	-0.467979	-0.38308	-0.501397	BGIOSGA018351	XP_006654241.1 WD repeat-containing protein 91 homolog isoform X1
TCONS_00006667	-0.467589	-1.00624	-0.552304	BGIOSGA016999	XP_002454457.2E3 ubiquitin-protein ligase ATL4
TCONS_00011842	-0.467549	-0.342095	-0.622701	BGIOSGA008842	XP_015691163.1 U-box domain-containing protein 30
TCONS_00033956	-0.467268	-3.44716	-1.46371	BGIOSGA037524	XP_006663087.1 acyl-CoA-binding domain-containing protein 5
TCONS_00005324	-0.467248	-1.66357	-0.401113	BGIOSGA018483	XP_015688383.1PREDICTED: uncharacterized protein LOC102701975
TCONS_00036306	-0.467188	-0.693896	-0.0906933	BGIOSGA009784	XP_006657402.1 protein LEO1 homolog
TCONS_00032716	-0.46708	0.109549	-1.37771	BGIOSGA024281	XP_015697100.1 nucleoside diphosphate kinase 1
TCONS_00032258	-0.466703	-0.830705	-3.61497	BGIOSGA030724	XP_015696163.1 putative cinnamyl alcohol dehydrogenase 5
TCONS_00016563	-0.466645	-2.92681	-3.58708	BGIOSGA014401	XP_006652772.2 xyloglucan endotransglucosylase/hydrolase protein 24-like
TCONS_00012394	-0.466056	0.221309	-1.46302	BGIOSGA010591	XP_006650164.1 nodulation protein H-like isoform X1
TCONS_00012862	-0.465979	-0.388874	-0.591644	BGIOSGA012570	XP_006651657.1 protein MPE1-like
TCONS_00029614	-0.465926	1.04698	0.180233	#N/A	#N/A
TCONS_00005230	-0.46591	0.642977	-0.333114	BGIOSGA022100	XP_006646765.2 protein transport protein SEC16A homolog
TCONS_00013573	-0.465771	1.3387	-0.357	BGIOSGA009404	XP_006650931.1 protein EXPORTIN 1A-like
TCONS_00017386	-0.465578	-0.886933	-0.0194184	BGIOSGA002066	XP_006654172.1 cyclin-A1-4
TCONS_00028959	-0.465385	-0.0906267	1.86193	BGIOSGA029692	XP_006661198.1 alpha,alpha-trehalose-phosphate synthase [UDP-forming] 6-like
TCONS_00010424	-0.465269	-1.54522	-1.14304	BGIOSGA007996	XP_015689959.1 translation initiation factor IF-2
TCONS_00010860	-0.465165	0.220187	0.579165	BGIOSGA013481	NP_001146378.2uncharacterized protein LOC100279956
TCONS_00011083	-0.465027	-2.86745	#NA	BGIOSGA009051	XP_002466344.1transcription factor PCF6
TCONS_00007097	-0.465006	-0.39102	-0.438968	BGIOSGA027921	XP_006649162.1 60S ribosomal protein L37-3-like
TCONS_00009976	-0.464905	-1.19767	-0.808451	BGIOSGA012530	XP_006658056.1 magnesium-chelatase subunit ChlH, chloroplastic
TCONS_00034105	-0.464903	-1.38365	-0.89809	BGIOSGA037915	XP_006663137.1 GDSL esterase/lipase CPRD49 isoform X1
TCONS_00003531	-0.464896	-1.19505	-0.957151	BGIOSGA015052	XP_015692115.1 protein LOW PSII ACCUMULATION 1, chloroplastic
TCONS_00012385	-0.464439	1.28831	0.117932	BGIOSGA006854	XP_003557774.1uncharacterized protein LOC100846339
TCONS_00026974	-0.464246	-1.01045	-0.0412446	BGIOSGA021302	XP_015695672.1 transcription factor bHLH78-like
TCONS_00005008	-0.464156	-2.68138	-1.56686	BGIOSGA004976	XP_006645269.1PREDICTED: uncharacterized protein LOC102713692 isoform X2
TCONS_00016730	-0.463938	-1.17851	-0.283847	BGIOSGA008696	XP_006652914.1 uncharacterized vacuolar membrane protein YML018C
TCONS_00028980	-0.463634	0.739606	0.231417	BGIOSGA012144	XP_015696504.1 putative receptor-like protein kinase At4g00960
TCONS_00001584	-0.463604	-1.08713	-0.352299	BGIOSGA004218	XP_006644531.1 protein prenyltransferase alpha subunit repeat-containing protein 1-B
TCONS_00004226	-0.46357	0.858636	0.338173	BGIOSGA000978	XP_010232178.1atherin isoform X1
TCONS_00036449	-0.463394	-1.51805	-0.814871	BGIOSGA026733	XP_006660251.1 COP9 signalosome complex subunit 6a-like
TCONS_00018456	-0.463358	0.2484	0.613324	BGIOSGA018980	XP_004960932.1HMG box-containing protein 4
TCONS_00011046	-0.463103	-0.14689	-0.7743	BGIOSGA013643	XP_015690089.1PREDICTED: uncharacterized protein LOC102718346, partial
TCONS_00023880	-0.463067	-0.0293815	0.473408	BGIOSGA034875	XP_002460832.1uncharacterized protein LOC8084102
TCONS_00033783	-0.463029	#NA	-0.429668	BGIOSGA036743	XP_002442752.1protein LURP-one-related 8
TCONS_00002584	-0.462923	-1.81869	-0.699628	#N/A	XP_015699208.1 replication protein A 70 kDa DNA-binding subunit D-like isoform X2
TCONS_00004244	-0.462716	1.35253	0.781107	BGIOSGA009460	XP_004969649.1golgin subfamily A member 2
TCONS_00030353	-0.462638	-0.740871	-1.46073	BGIOSGA029446	XP_004957338.1uncharacterized protein LOC101756299
TCONS_00031659	-0.462545	-0.0632168	-0.321967	BGIOSGA012749	XP_006662085.1 probable methyltransferase PMT2
TCONS_00020771	-0.462338	-1.29647	-0.0746112	BGIOSGA020192	XP_006664960.2 cysteine-rich receptor-like protein kinase 10
TCONS_00001056	-0.462268	-0.615763	-1.78359	BGIOSGA040621	XP_004968851.1transcription factor MYBS1
TCONS_00016913	-0.462222	-0.577521	-0.189158	BGIOSGA014050	XP_015691745.1 UPF0235 protein C15orf40 homolog
TCONS_00021865	-0.46218	-0.444764	-0.34333	BGIOSGA021655	XP_006655917.1 probable DNA helicase MCM9
TCONS_00011887	-0.461912	1.44272	-0.199528	BGIOSGA029119	XP_004985025.1chaperone protein dnaJ 11, chloroplastic
TCONS_00005513	-0.461813	-0.17105	0.489956	BGIOSGA007066	XP_006646926.1 cullin-associated NEDD8-dissociated protein 1
TCONS_00007177	-0.461403	0.408579	1.73618	#N/A	#N/A
TCONS_00003420	-0.46139	-0.872019	-1.26511	BGIOSGA018213	XP_015688249.1 phospholipase D zeta 1-like
TCONS_00011941	-0.460569	-0.239094	0.294413	BGIOSGA011072	XP_010228846.1protein DENND6B

TCONS_00029644	-0.460487	-1.60158	-1.19891	BGIOSGA030166	XP_006660441.1 probable 2-oxoglutarate-dependent dioxygenase At3g49630
TCONS_00011971	-0.460299	-1.21427	-1.22745	BGIOSGA011041	XP_006649816.1PREDICTED: uncharacterized protein LOC102704874
TCONS_00020883	-0.460089	-3.93941	-2.53959	BGIOSGA019239	XP_015693949.1 solute carrier family 40 member 1-like isoform X1
TCONS_00026222	-0.459945	1.25597	0.449991	BGIOSGA028302	XP_006659971.1 cell wall integrity protein scw1
TCONS_00011724	-0.459569	-1.8297	-1.81682	BGIOSGA021834	XP_002465703.1low affinity sulfate transporter 3
TCONS_00021136	-0.459503	-0.0992756	-0.155321	BGIOSGA037365	XP_015694441.1 protein S-acyltransferase 24-like
TCONS_00014529	-0.459425	-1.13234	-0.904464	BGIOSGA022657	XP_004976103.1zinc finger protein CONSTANS-LIKE 3
TCONS_00016692	-0.459093	0.137961	0.0874937	BGIOSGA014271	XP_006653800.2 heterogeneous nuclear ribonucleoprotein U-like protein 1
TCONS_00029988	-0.459008	-0.574279	0.00785533	BGIOSGA036546	XP_015697446.1 ABC transporter G family member 51
TCONS_00006147	-0.458993	-1.72552	-0.550958	BGIOSGA008305	XP_015689654.1PREDICTED: uncharacterized protein LOC102706720 isoform X1
TCONS_00011918	-0.45898	0.957038	0.714153	BGIOSGA029864	XP_015691022.1 protein MOS2-like
TCONS_00030365	-0.458941	-1.44616	-1.42179	BGIOSGA015895	XP_006660848.1 disease resistance protein RPM1-like
TCONS_00037533	-0.458904	0.0382345	-0.322851	BGIOSGA035833	XP_006664204.1 putative hydrolase C777.06c
TCONS_00001916	-0.458852	-0.196684	0.162742	BGIOSGA007377	XP_012700429.1uncharacterized protein LOC101753625
TCONS_00033707	-0.458713	-1.18201	0.216502	BGIOSGA013751	XP_015697863.1 F-box only protein 7-like isoform X1
TCONS_00008464	-0.458691	-1.24214	-0.691114	BGIOSGA014667	XP_015688674.1 E3 ubiquitin-protein ligase RMA1-like
TCONS_00000674	-0.458568	-0.473316	-1.03299	BGIOSGA026823	XP_012472885.1 calmodulin-like
TCONS_00023741	-0.458558	-0.252319	0.591041	BGIOSGA015356	XP_004957811.1protein FLX-like 1
TCONS_00006730	-0.458415	0.490305	0.083705	BGIOSGA008902	XP_006647780.1 calpain-type cysteine protease ADL1
TCONS_00025639	-0.458407	-0.824198	-0.0697887	BGIOSGA023787	XP_015694478.1PREDICTED: uncharacterized protein LOC102715777
TCONS_00017648	-0.458374	-0.276292	-0.244036	BGIOSGA006323	XP_006654305.2 caffeoylshikimate esterase isoform X2
TCONS_00019906	-0.458237	0.942396	0.818445	BGIOSGA015140	XP_006654865.1 probable esterase KAI2
TCONS_00023260	-0.458172	-0.565758	0.168399	BGIOSGA039314	XP_006658350.2 methylmalonate-semialdehyde dehydrogenase [acylating], mitochondrial
TCONS_00023786	-0.457934	-0.0388681	-0.0382019	BGIOSGA007413	XP_006657769.1 succinate dehydrogenase subunit 3-2, mitochondrial
TCONS_00013434	-0.457894	0.766379	-0.794955	BGIOSGA005913	XP_006650808.1 protein phosphatase 2C 35
TCONS_00029241	-0.457608	-0.492627	-0.180289	BGIOSGA018655	XP_006660750.1 protein ASPARTIC PROTEASE IN GUARD CELL 1-like
TCONS_00002405	-0.457362	0.00490843	-0.112533	BGIOSGA005044	XP_006645226.1 selenium-binding protein 1-like
TCONS_00000972	-0.45726	-0.295125	-0.136756	BGIOSGA003572	XP_006644190.1 nuclear pore complex protein NUP98A-like isoform X1
TCONS_00029273	-0.457236	-1.25863	-1.27432	BGIOSGA028998	XP_006660784.1PREDICTED: uncharacterized protein LOC102714857
TCONS_00030041	-0.45723	-0.455831	-0.911071	BGIOSGA000410	XP_008652702.1uncharacterized protein LOC103632738
TCONS_00004190	-0.457204	0.644097	-0.173629	BGIOSGA022960	XP_015688308.1 protein DETOXIFICATION 12-like
TCONS_00021137	-0.457176	0.212938	-0.0774592	BGIOSGA023361	XP_006656295.1 U3 small nucleolar RNA-associated protein 21 homolog
TCONS_00018678	-0.457057	-1.72747	-0.589482	BGIOSGA002348	XP_006654083.1 E3 ubiquitin-protein ligase MBR2-like isoform X1
TCONS_00018854	-0.456986	-0.144759	-0.479627	BGIOSGA018563	XP_006664912.1 post-GPI attachment to proteins factor 3
TCONS_00031221	-0.456801	-0.333175	0.455793	BGIOSGA011438	XP_015697006.1 protein GDAP2 homolog
TCONS_00010515	-0.456734	-1.90863	0.287581	BGIOSGA005135	XP_006651602.2 calcium-transporting ATPase 2, plasma membrane-type-like
TCONS_00012546	-0.456599	-0.62716	-1.09532	BGIOSGA006287	XP_021313437.1uncharacterized protein LOC8069552 isoform X3
TCONS_00034993	-0.456545	1.23535	-0.154219	BGIOSGA017894	NP_001169036.1uncharacterized protein LOC100382872
TCONS_00004598	-0.456445	-0.786483	-1.482	BGIOSGA000618	XP_006644883.1 probable arabinosyltransferase ARAD1
TCONS_00024406	-0.456298	-0.0463287	-0.0685692	BGIOSGA010939	XP_006659000.1 importin-11
TCONS_00017537	-0.456142	-1.70221	-1.24565	BGIOSGA018383	XP_003568697.1receptor-like protein kinase FERONIA
TCONS_00016465	-0.456058	-1.20821	-0.339873	BGIOSGA022460	XP_015691835.1 E3 ubiquitin-protein ligase RHA1B-like
TCONS_00035425	-0.455906	-1.11718	-0.695112	BGIOSGA036838	XP_006663701.1 protein TONNEAU 1a-like
TCONS_00017795	-0.455904	1.81397	0.00766292	BGIOSGA015425	XP_015693221.1 squamosa promoter-binding-like protein 9
TCONS_00018521	-0.455852	0.286246	-0.726842	BGIOSGA018913	XP_015692459.1 thylakoid membrane protein TERC, chloroplastic
TCONS_00017623	-0.455464	-0.813588	-0.0607132	BGIOSGA020546	XP_006654293.1 nicotinamide adenine dinucleotide transporter 1, chloroplastic-like
TCONS_00017370	-0.455327	-3.51988	-2.9715	BGIOSGA019435	XP_006654168.1PREDICTED: uncharacterized protein LOC102706101
TCONS_00004823	-0.455287	-1.1469	-2.65376	BGIOSGA037101	XP_004970810.1amino acid permease 4
TCONS_00030537	-0.455226	0.862962	-0.160931	BGIOSGA029268	XP_015696732.1 protein SUPPRESSOR OF FRI 4
TCONS_00038888	-0.454983	0.405263	0.685339	BGIOSGA000749	XP_010240583.2exocyst complex component EXO70A1 isoform X1
TCONS_00010397	-0.454933	1.5183	-0.994779	BGIOSGA014643	XP_006650257.1 elongation factor G, mitochondrial
TCONS_00025005	-0.45482	0.978922	0.906766	BGIOSGA024394	XP_006658508.1 ribosome biogenesis protein BOP1 homolog
TCONS_00013949	-0.454696	-0.304773	0.350403	BGIOSGA032220	XP_015691647.1 protein MICRORCHIDIA 7 isoform X1
TCONS_00007548	-0.454517	-1.95739	-2.01493	BGIOSGA027931	NP_001150620.1F-box domain containing protein

transcriptome

TCONS_00017497	-0.454367	0.626881	-0.111714	BGIOSGA019569	XP_015692536.1PREDICTED: uncharacterized protein LOC102712995
TCONS_00011924	-0.454187	0.101419	-0.376873	BGIOSGA011088	XP_015689836.1 chorismate synthase 2, chloroplastic
TCONS_00002088	-0.454174	-0.216951	-0.005383	BGIOSGA013279	NP_001167967.1uncharacterized protein LOC100381683
TCONS_00012610	-0.453983	-0.300536	0.150376	BGIOSGA001307	XP_006651570.2 pentatricopeptide repeat-containing protein At3g24000, mitochondrial-like
TCONS_00007176	-0.453942	-0.0253811	-0.88167	BGIOSGA038191	XP_021315347.1epimerase family protein SDR39U1 homolog, chloroplastic isoform X2
TCONS_00025794	-0.453906	-1.07921	-1.76289	BGIOSGA002927	XP_006659042.1 auxin-responsive protein IAA25-like isoform X2
TCONS_00011144	-0.453901	0.464109	-0.247792	BGIOSGA020785	XP_006651915.1 cactin
TCONS_00000277	-0.453816	-0.924985	0.547711	#N/A	#N/A
TCONS_00035616	-0.453768	-0.553632	-0.584398	BGIOSGA034846	XP_006664356.1 transcription factor LHW-like
TCONS_00006952	-0.453746	-0.683987	-0.430953	BGIOSGA009134	XP_006647993.1PREDICTED: uncharacterized protein LOC102707465 isoform X1
TCONS_00003667	-0.453659	0.414049	0.68366	BGIOSGA029631	XP_003567758.1probable protein phosphatase 2C 3 isoform X2
TCONS_00031597	-0.453565	-1.14501	-0.739072	BGIOSGA026271	XP_015697432.1 mediator of RNA polymerase II transcription subunit 12-like isoform X2
TCONS_00013893	-0.453373	1.06968	0.242139	BGIOSGA005951	XP_004975218.1WRKY transcription factor WRKY51
TCONS_00013542	-0.453292	-1.1792	-0.775058	BGIOSGA001322	NP_001130540.1putative DUF2301 domain protein
TCONS_00006777	-0.453239	-1.05115	-1.2072	BGIOSGA000378	XP_006647812.2 probable amino acid permease 7
TCONS_00004174	-0.453025	-4.21203	-1.80691	BGIOSGA001024	XP_015688292.1 transcription factor-like protein DPB isoform X1
TCONS_00008247	-0.453025	0.488405	0.822445	BGIOSGA006181	XP_006648785.1 CTL-like protein DDB_G0274487
TCONS_00018590	-0.452703	-1.12498	-0.559788	BGIOSGA018846	XP_015692843.1PREDICTED: uncharacterized protein LOC102708524
TCONS_00020915	-0.452697	-1.49533	0.19697	BGIOSGA018014	XP_006656180.1 beta-galactosidase 9-like
TCONS_00005825	-0.452634	-0.403756	0.365958	BGIOSGA007949	XP_006647150.1 gamma-secretase subunit APH1-like
TCONS_00030457	-0.452546	0.0259843	0.33622	BGIOSGA014652	XP_006660917.2 aspartic proteinase-like protein 1
TCONS_00033088	-0.451888	-0.441502	-0.496187	BGIOSGA037090	XP_006663263.1 SWI/SNF complex subunit SWI3C isoform X1
TCONS_00026375	-0.451852	-1.37848	-0.386489	BGIOSGA015377	XP_015696837.1PREDICTED: uncharacterized protein LOC102722801 isoform X2
TCONS_00033443	-0.451849	-0.741514	-0.233725	BGIOSGA026603	XP_015698125.1 GDT1-like protein 3
TCONS_00011653	-0.451392	-1.79043	-0.0512906	BGIOSGA011355	XP_002452410.2uncharacterized protein LOC8056495 isoform X1
TCONS_00024206	-0.45084	0.540093	1.18765	BGIOSGA003982	XP_004958497.1probable carboxylesterase 18
TCONS_00019061	-0.450664	-0.215016	0.887649	BGIOSGA025325	XP_024041499.1tRNA (guanine(26)-N(2))-dimethyltransferase isoform X2
TCONS_00032630	-0.450524	-0.818728	-1.82987	BGIOSGA006243	XP_006662559.1 MLO-like protein 9
TCONS_00018525	-0.450478	-1.4079	0.27421	BGIOSGA018910	XP_006653986.1 DNA repair protein RAD51 homolog 2
TCONS_00035594	-0.450471	0.190796	-0.228412	BGIOSGA034831	XP_003574083.1uncharacterized protein LOC100821945
TCONS_00006879	-0.450317	0.0615811	-1.16248	BGIOSGA021627	XP_004955213.1transcription termination factor MTERF6, chloroplastic/mitochondrial
TCONS_00022976	-0.450301	-1.08006	-1.39301	BGIOSGA025010	XP_006657394.1 probable serine/threonine-protein kinase DDB_G0278509
TCONS_00002420	-0.450248	-0.241536	-1.26363	BGIOSGA005062	XP_015698734.1 haloalkane dehalogenase 2 isoform X1
TCONS_00015671	-0.449801	0.693635	0.040397	#N/A	#N/A
TCONS_00006154	-0.449725	0.25235	0.181424	BGIOSGA008067	XP_006647256.1 protein MEI2-like 4
TCONS_00032006	-0.449686	-0.262051	-0.291654	BGIOSGA000791	XP_015698964.1 phosphatidate cytidylyltransferase 1-like
TCONS_00023327	-0.449557	-4.21641	0.271925	BGIOSGA018731	XP_006657546.1PREDICTED: uncharacterized protein LOC102711713
TCONS_00000039	-0.449355	1.48378	0.827819	BGIOSGA027363	XP_006648168.1 pentatricopeptide repeat-containing protein At1g61870, mitochondrial-like
TCONS_00005251	-0.449247	-1.06543	-2.72745	BGIOSGA026711	XP_003570794.1linolenate hydroperoxide lyase, chloroplastic
TCONS_00030067	-0.449122	1.97287	-0.646688	BGIOSGA016708	XP_003578136.1putrescine hydroxycinnamoyltransferase 1
TCONS_00017099	-0.449007	-1.90045	0.385938	BGIOSGA010029	XP_015692474.1PREDICTED: uncharacterized protein LOC102707121
TCONS_00006776	-0.448934	0.812553	-0.272759	BGIOSGA005431	XP_006647811.1 transmembrane 9 superfamily member 12-like
TCONS_00007949	-0.448828	-0.15285	0.22979	BGIOSGA006531	XP_015688583.1 conserved oligomeric Golgi complex subunit 4
TCONS_00008186	-0.448623	-0.115976	-1.49679	BGIOSGA004909	XP_015689466.1 chloride channel protein CLC-c-like
TCONS_00004072	-0.448354	0.0835489	-1.33725	BGIOSGA016610	XP_006646148.1 potassium channel AKT1 isoform X2
TCONS_00027211	-0.448273	-1.00857	-0.689429	BGIOSGA003715	XP_006659029.1 indole-2-monooxygenase-like
TCONS_00033297	-0.448136	-2.51193	-1.05575	BGIOSGA020982	XP_004979163.1protein NRT1/ PTR FAMILY 4.5
TCONS_00007944	-0.448062	-0.100646	-0.410211	BGIOSGA021986	XP_006647243.1 probable protein transport Sec1a
TCONS_00037136	-0.447595	-3.3673	-2.14376	BGIOSGA037333	NP_001266910.1fatty acid alpha-dioxygenase
TCONS_00037541	-0.44746	-0.823849	-1.10761	BGIOSGA035824	XP_006664751.2 K(+) efflux antiporter 3, chloroplastic
TCONS_00016084	-0.447318	-1.93435	1.11681	BGIOSGA017830	XP_020214401.1beta-glucosidase 24-like
TCONS_00016578	-0.44728	-0.190653	-0.595314	BGIOSGA014391	XP_006652786.1 putative RNA methyltransferase At5g10620
TCONS_00035869	-0.446944	-0.421408	-0.326757	BGIOSGA026299	XP_015698268.1 pentatricopeptide repeat-containing protein At3g46610-like
TCONS_00025737	-0.446767	-1.02991	-0.128417	BGIOSGA023685	XP_015694806.1 importin subunit alpha-4
TCONS_00016155	-0.446728	-1.00119	-0.999796	BGIOSGA014734	XP_006652450.1 inositol transporter 1

TCONS_00003377	-0.446684	-0.220527	-0.530218	BGIOSGA019128	XP_006644092.1 probable protein phosphatase 2C 2
TCONS_00035731	-0.446129	-0.351018	-1.47104	#N/A	#N/A
TCONS_00008052	-0.446063	-0.201279	0.573142	BGIOSGA003024	XP_006648665.1 extensin
TCONS_00019472	-0.445889	-0.739462	0.378799	BGIOSGA017880	XP_015693287.1PREDICTED: uncharacterized protein LOC102720798 isoform X1
TCONS_00021420	-0.445876	0.186399	0.227579	BGIOSGA022730	XP_006656524.1 tRNA dimethylallyltransferase 9
TCONS_00026523	-0.445794	1.22485	0.632657	BGIOSGA028589	XP_006660096.1 protein Dr1 homolog
TCONS_00028392	-0.445769	-0.0342655	0.271985	BGIOSGA026572	XP_014756044.1pentatricopeptide repeat-containing protein At2g22410, mitochondrial
TCONS_00019798	-0.445759	-0.316249	0.40626	BGIOSGA017560	XP_006654788.2PREDICTED: uncharacterized protein LOC102721553
TCONS_00000743	-0.445611	-1.07752	-1.20234	BGIOSGA020246	XP_015688318.1PREDICTED: uncharacterized protein LOC102719635
TCONS_00029977	-0.445457	0.772649	0.372535	BGIOSGA026961	XP_004956731.1U-box domain-containing protein 39
TCONS_00003964	-0.445367	0.452044	-0.478475	BGIOSGA001226	XP_006644358.1 protein YIPF1 homolog
TCONS_00022204	-0.445328	-1.32426	-0.365815	BGIOSGA022397	XP_004965431.1probable transcription factor GLK1
TCONS_00005514	-0.445026	-0.390426	-0.411236	BGIOSGA031106	XP_006646937.2 pentatricopeptide repeat-containing protein At2g35130
TCONS_00025587	-0.445012	-1.69355	-2.65434	BGIOSGA012718	XP_015694786.1 pyruvate dehydrogenase (acetyl-transferring) kinase, mitochondrial-like isoform X1
TCONS_00010872	-0.445009	-1.00282	-0.930893	BGIOSGA005517	XP_006650525.1 inositol-tetrakisphosphate 1-kinase 3
TCONS_00012725	-0.444825	-0.679039	0.0705189	BGIOSGA001893	XP_015699151.1 exportin-7-A isoform X1
TCONS_00025886	-0.444678	-2.90986	-0.842918	BGIOSGA027961	XP_015695488.1 blue copper protein-like
TCONS_00027775	-0.444571	-0.417537	0.0942115	#N/A	#N/A
TCONS_00026513	-0.444465	-0.515694	-0.157826	BGIOSGA018994	XP_006660094.2 probable phospholipid-transporting ATPase 8
TCONS_00016206	-0.444412	0.622463	0.619488	BGIOSGA020957	XP_003580095.1polyadenylate-binding protein 2
TCONS_00009397	-0.444379	-1.35704	-0.624293	BGIOSGA031952	XP_015690912.1 cyclic dof factor 1-like
TCONS_00021528	-0.444143	0.0986147	0.234695	BGIOSGA019454	XP_015694199.1 calcium-dependent protein kinase 34
TCONS_00005941	-0.444068	-0.791144	-0.278561	BGIOSGA006581	XP_010234161.1fatty-acid-binding protein 3, chloroplastic
TCONS_00014201	-0.443866	-0.521908	-0.444631	BGIOSGA008356	XP_003581199.1protein BIC1
TCONS_00028582	-0.443864	#NA	0.845378	BGIOSGA036057	XP_006664082.1 golgin candidate 6
TCONS_00017055	-0.443521	1.22526	-0.376872	BGIOSGA031869	XP_008656525.1putative serine/threonine-protein kinase-like protein CCR3
TCONS_00037342	-0.443516	-0.405813	0.0821097	BGIOSGA036086	XP_006664088.1 trafficking protein particle complex II-specific subunit 130 homolog isoform X2
TCONS_00016760	-0.443211	-0.263093	0.0370934	BGIOSGA012043	XP_006652928.2 probable sulfate transporter 3.3
TCONS_00017185	-0.443103	0.973124	0.094887	BGIOSGA027531	XP_006654079.1 ubiquitin-40S ribosomal protein S27a
TCONS_00009457	-0.443066	-0.722032	-1.3824	BGIOSGA023496	XP_024313762.1transmembrane protein 131
TCONS_00034865	-0.441967	0.42921	0.0362196	BGIOSGA002574	XP_006662936.1 40S ribosomal protein S5
TCONS_00027006	-0.441816	-0.252791	-0.338154	BGIOSGA011914	XP_015695792.1 ACT domain-containing protein ACR6-like
TCONS_00037970	-0.441743	0.701992	-0.896777	BGIOSGA035180	XP_006664993.1 protein KTI12 homolog
TCONS_00011782	-0.441701	0.714878	1.11764	BGIOSGA011223	XP_006649619.1 probable leucine-rich repeat receptor-like protein kinase At1g35710
TCONS_00003851	-0.441648	-0.0763936	-0.0414643	BGIOSGA001363	XP_015699277.1 DENN domain and WD repeat-containing protein SCD1
TCONS_00017202	-0.441615	-2.13595	-0.355904	BGIOSGA002851	XP_015692837.1PREDICTED: LOW QUALITY PROTEIN: uncharacterized protein LOC102704801
TCONS_00018952	-0.441511	-4.80497	-0.901664	BGIOSGA040670	XP_006654183.1 random slug protein 5-like isoform X2
TCONS_00015036	-0.441374	-0.886178	-0.110777	BGIOSGA017218	XP_006652894.1 probable 20S rRNA accumulation protein 4
TCONS_00028813	-0.440751	0.447283	-0.433512	BGIOSGA029313	XP_006661143.2 probable LRR receptor-like serine/threonine-protein kinase At1g07650
TCONS_00036430	-0.440688	-0.688496	-0.924406	BGIOSGA025162	XP_010237011.1transcription factor PCF8
TCONS_00014748	-0.440172	0.825427	0.074314	BGIOSGA005729	XP_006665126.1PREDICTED: uncharacterized protein LOC102715819
TCONS_00036484	-0.439995	2.55856	0.547807	BGIOSGA037492	XP_006664231.1PREDICTED: uncharacterized protein LOC102709977
TCONS_00001259	-0.439861	0.473464	-0.182055	BGIOSGA008512	XP_002455879.1protein SYS1 homolog
TCONS_00023949	-0.439578	0.0877144	0.36235	BGIOSGA024188	XP_021311360.1uncharacterized protein LOC8061824
TCONS_00016211	-0.439274	-0.438843	-0.443956	BGIOSGA012519	XP_006653582.2 probable leucine-rich repeat receptor-like protein kinase At5g63930
TCONS_00016699	-0.43923	0.647519	0.294398	BGIOSGA018800	XP_002448602.1N-terminal acetyltransferase A complex catalytic subunit NAA10
TCONS_00010619	-0.439212	0.52466	-0.343099	BGIOSGA012138	XP_015690460.1 glucan endo-1,3-beta-glucosidase 8-like
TCONS_00015423	-0.438959	-0.391826	-0.128985	BGIOSGA000082	XP_015692142.1 protein O-linked-mannose beta-1,4-N-acetylglucosaminyltransferase 2-like
TCONS_00006704	-0.438953	0.679326	0.230366	BGIOSGA017042	XP_006652746.1 60S ribosomal protein L12-1
TCONS_00031598	-0.438739	-2.11348	0.0395287	BGIOSGA016965	XP_002451500.1uncharacterized protein LOC8057532
TCONS_00020576	-0.438418	-0.594892	-0.103104	BGIOSGA022753	XP_015693918.1 GPI-anchored protein LORELEI-like isoform X1
TCONS_00001844	-0.438394	-1.40302	-0.858773	BGIOSGA026441	NP_001148308.2RING-H2 finger protein ATL2K
TCONS_00006007	-0.437961	-0.875877	0.157498	BGIOSGA008153	XP_006647319.1 putative disease resistance RPP13-like protein 1
TCONS_00029255	-0.437858	-0.281775	-0.901661	BGIOSGA028950	XP_006660760.1 tonoplast dicarboxylate transporter-like
TCONS_00033615	-0.437375	-1.39006	-0.253357	BGIOSGA033854	XP_023157350.1F-box/FBD/LRR-repeat protein At1g13570-like isoform X1

TCONS_00029158	-0.437308	-0.901035	-0.0765521	BGIOSGA011997	XP_006660701.1 probable LRR receptor-like serine/threonine-protein kinase At1g67720
TCONS_00029331	-0.43725	-0.829423	-0.471653	BGIOSGA031079	XP_006660832.1 hsp70 nucleotide exchange factor FES1
TCONS_00013640	-0.437195	0.974236	-0.0210566	BGIOSGA000898	XP_006647249.1 elongation factor 2-like
TCONS_00030109	-0.43691	-1.64749	-0.990088	BGIOSGA029696	XP_022682261.1uncharacterized protein LOC101768145
TCONS_00034291	-0.436869	0.571632	0.870449	BGIOSGA036642	XP_015697824.1 meiotic recombination protein DMC1 homolog
TCONS_00009279	-0.436859	0.329988	-0.138477	BGIOSGA009276	XP_010940715.1 60S ribosomal protein L23
TCONS_00002427	-0.436477	0.32227	0.194195	BGIOSGA005070	XP_015690781.1 alpha-glucosidase 2-like
TCONS_00007659	-0.436358	-3.72753	-0.323295	BGIOSGA002176	XP_006647103.1 aquaporin NIP1-1
TCONS_00002421	-0.436323	-0.0848853	-0.190972	BGIOSGA010464	XP_006645240.1PREDICTED: uncharacterized protein At3g27210
TCONS_00033408	-0.436321	0.0831883	-0.416986	BGIOSGA010822	XP_015698081.1 phospholipid-transporting ATPase 2-like isoform X2
TCONS_00010983	-0.43621	0.408022	-0.374325	BGIOSGA001590	XP_006650608.1 60S ribosomal protein L13a-4
TCONS_00020951	-0.436197	-1.40503	-1.55467	BGIOSGA014071	XP_006657095.1 probable LRR receptor-like serine/threonine-protein kinase At1g14390
TCONS_00004643	-0.43612	-0.949043	1.31181	BGIOSGA013657	XP_006656565.1 pentatricopeptide repeat-containing protein At1g08070, chloroplastic-like
TCONS_00008767	-0.436109	-0.417065	-2.25975	BGIOSGA014182	XP_004953842.1glutamine synthetase cytosolic isozyme 1-1
TCONS_00000699	-0.436047	-1.22304	-0.606737	BGIOSGA009237	XP_015692529.1 auxin-responsive protein IAA4-like
TCONS_00002253	-0.435907	-2.06558	-0.425555	BGIOSGA004740	XP_014754480.1protein NRT1/ PTR FAMILY 5.10
TCONS_00024797	-0.43562	-1.01712	-1.92974	BGIOSGA009564	XP_006658415.1 protein LURP-one-related 15-like
TCONS_00004514	-0.435561	-0.69019	-0.028369	BGIOSGA000699	XP_006644804.1 sphingosine kinase 2-like
TCONS_00007524	-0.435545	-0.314493	0.832642	BGIOSGA023272	XP_015689063.1 non-structural maintenance of chromosomes element 4 homolog A-like
TCONS_00005092	-0.434894	-0.263122	-0.221895	BGIOSGA039225	XP_004971243.1U-box domain-containing protein 43
TCONS_00012096	-0.434853	-0.978181	-0.642899	BGIOSGA013820	XP_006651288.1 protein SCAR2-like
TCONS_00007975	-0.434789	-1.03848	-0.809887	BGIOSGA006508	XP_006647309.2 7-dehydrocholesterol reductase
TCONS_00000527	-0.43451	-0.467077	-0.0475051	BGIOSGA003098	XP_015697724.1 DDB1- and CUL4-associated factor 13
TCONS_00002224	-0.434356	-0.329391	-0.258527	BGIOSGA036588	XP_006646502.1 transcription factor LHW-like
TCONS_00014099	-0.434351	0.123869	0.322289	BGIOSGA005460	XP_006652209.1 vacuolar protein-sorting-associated protein 11 homolog
TCONS_00030409	-0.434198	0.913606	0.34376	BGIOSGA029392	NP_001131256.2uncharacterized LOC100192569
TCONS_00028800	-0.433426	0.969218	-2.45611	BGIOSGA014279	XP_006661137.1 probable kinase CHARK
TCONS_00036953	-0.433425	-2.69141	-1.59163	BGIOSGA037500	XP_006663945.2 glutathione synthetase, chloroplastic-like
TCONS_00035467	-0.433277	-1.03604	-1.33813	BGIOSGA000316	XP_006663744.1 probable non-specific lipid-transfer protein 3
TCONS_00002066	-0.433276	-0.208086	0.00503361	BGIOSGA001037	XP_014754699.1ubiquitin-conjugating enzyme E2-17 kDa isoform X1
TCONS_00014582	-0.432893	-0.438368	-1.72585	BGIOSGA015013	XP_006652499.1PREDICTED: uncharacterized protein LOC102701719
TCONS_00013331	-0.432784	-0.712782	-1.92995	BGIOSGA005739	XP_006650720.1PREDICTED: uncharacterized protein LOC102708783
TCONS_00016083	-0.432608	-4.01648	-1.90806	BGIOSGA011222	XP_006652404.2 beta-glucosidase 10-like
TCONS_00017912	-0.432565	-1.09117	-1.32987	BGIOSGA007188	XP_006655369.2 beta-glucosidase BoGH3B-like isoform X2
TCONS_00018919	-0.432177	-0.480367	-0.328543	BGIOSGA018437	XP_015691155.1 switch 2
TCONS_00020713	-0.432147	-0.953825	0.494703	BGIOSGA009239	XP_022679633.1quinone oxidoreductase PIG3
TCONS_00016554	-0.43209	-1.18399	-0.061958	BGIOSGA030174	NP_001146059.1putative putrescine-binding domain family protein
TCONS_00023476	-0.431906	-0.841235	-0.939056	BGIOSGA035998	XP_006664107.1 psbP domain-containing protein 1, chloroplastic
TCONS_00006892	-0.431764	-0.970645	-0.125166	BGIOSGA027012	XP_004953951.1PLAT domain-containing protein 3
TCONS_00014339	-0.431691	-0.375065	0.0590287	BGIOSGA028483	XP_015691477.1 mitochondrial phosphate carrier protein 3, mitochondrial-like
TCONS_00001914	-0.431611	-0.759146	-0.717399	BGIOSGA028580	XP_008652974.1putative cysteine-rich receptor-like protein kinase 35
TCONS_00014274	-0.431496	0.299226	-0.0354791	#N/A	#N/A
TCONS_00007045	-0.431426	-0.148771	-0.145695	BGIOSGA009229	XP_006649138.1 N-alpha-acetyltransferase 35, NatC auxiliary subunit isoform X1
TCONS_00001170	-0.431362	-0.487668	0.337069	BGIOSGA003797	XP_006646017.2 carbamoyl-phosphate synthase large chain, chloroplastic
TCONS_00028303	-0.43089	-1.10422	0.303835	BGIOSGA034215	XP_006660283.1PREDICTED: uncharacterized protein LOC102721105, partial
TCONS_00022330	-0.430721	-1.9555	#NA	BGIOSGA009516	XP_015694249.1 cell number regulator 4-like isoform X2
TCONS_00016485	-0.430721	-0.955498	0.0761298	BGIOSGA029303	XP_010504143.1 histone H4
TCONS_00018467	-0.430667	-0.343335	-0.164054	BGIOSGA036558	XP_009337683.1 V-type proton ATPase 16 kDa proteolipid subunit-like
TCONS_00036558	-0.430413	-0.928458	0.151327	BGIOSGA032100	XP_006664280.1 vacuolar-sorting receptor 1-like
TCONS_00025927	-0.430355	-0.959905	-0.422515	BGIOSGA016851	XP_006659117.1 sphingoid long-chain bases kinase 1-like
TCONS_00033922	-0.430223	0.0510876	-0.338403	BGIOSGA018537	XP_006663628.2 glutathione synthetase, chloroplastic-like
TCONS_00021374	-0.43022	0.218921	0.402521	BGIOSGA023602	XP_002441742.1UV radiation resistance-associated gene protein
TCONS_00027317	-0.430036	-0.471627	-0.44121	BGIOSGA007014	XP_015695490.1 U-box domain-containing protein 8-like
TCONS_00005417	-0.430034	1.04371	1.48947	#N/A	#N/A
TCONS_00020745	-0.430034	0.365641	-0.510528	#N/A	#N/A

TCONS_00034995	-0.430024	1.34258	0.0173735	BGIOSGA017692	XP_003577502.1respiratory burst oxidase homolog protein B
TCONS_00037320	-0.430021	1.15825	1.53357	BGIOSGA036047	XP_006664085.1 calmodulin-binding protein 60 D-like isoform X1
TCONS_00022662	-0.429906	1.62337	-0.441535	BGIOSGA005812	XP_006657194.1 WRKY transcription factor 18-like
TCONS_00007522	-0.429874	0.171213	-0.199784	BGIOSGA017105	XP_006648411.1 brefeldin A-inhibited guanine nucleotide-exchange protein 1
TCONS_00019142	-0.42972	0.297361	-1.62257	BGIOSGA026862	XP_003568623.1probable protein phosphatase 2C 48
TCONS_00011176	-0.429657	0.664455	0.120486	BGIOSGA028281	XP_006650761.1 cytochrome b-c1 complex subunit 7-like
TCONS_00011743	-0.429413	-1.19635	-0.29721	BGIOSGA008192	XP_006649583.1 mitochondrial outer membrane protein porin 5
TCONS_00029574	-0.429182	1.14428	-0.0653154	BGIOSGA026430	XP_012699597.2 transcription factor MYB44-like
TCONS_00005052	-0.429077	-1.00558	0.0241627	BGIOSGA035641	XP_006645298.1 lipase-like
TCONS_00000973	-0.428819	-0.314544	0.083636	BGIOSGA008872	XP_024314430.1probable LRR receptor-like serine/threonine-protein kinase RKF3 isoform X1
TCONS_00037544	-0.428544	0.358181	0.427008	BGIOSGA010143	XP_006664755.1 GTPase LSG1-2-like
TCONS_00009713	-0.428153	0.895768	-0.174779	BGIOSGA011394	XP_015691176.1 myosin-binding protein 2 isoform X2
TCONS_00016929	-0.428044	1.8989	0.496995	BGIOSGA014035	XP_006653918.1 DDB1- and CUL4-associated factor homolog 1
TCONS_00021473	-0.427662	-0.399561	-0.257469	BGIOSGA001042	XP_006655702.1 callose synthase 10
TCONS_00036595	-0.427549	-2.13552	-1.74577	BGIOSGA036712	XP_006663775.2 protein DETOXIFICATION 21-like isoform X1
TCONS_00021493	-0.42742	-0.314687	-0.621447	BGIOSGA037907	XP_006655722.1PREDICTED: uncharacterized protein LOC102708063 isoform X1
TCONS_00037416	-0.4273	-1.0299	-0.471918	BGIOSGA032254	XP_015698733.1PREDICTED: uncharacterized protein LOC102707360
TCONS_00000703	-0.427251	0.923419	-0.365645	BGIOSGA017876	XP_002455489.1probable WRKY transcription factor 47 isoform X1
TCONS_00012390	-0.427247	-0.326841	0.277277	BGIOSGA037065	XP_006650163.1 serine carboxypeptidase-like 51
TCONS_00001133	-0.427244	-0.645064	-0.29612	BGIOSGA016821	XP_021310739.1NAD(P)-specific glutamate dehydrogenase isoform X4
TCONS_00015453	-0.427096	0.605284	-0.17753	BGIOSGA011835	XP_006653199.1 ABC transporter C family member 4-like
TCONS_00003966	-0.427066	0.268017	-0.118788	BGIOSGA001224	XP_002458145.1uncharacterized protein LOC8054370
TCONS_00017512	-0.426986	0.228946	-1.01625	BGIOSGA001474	XP_021317352.1disease resistance protein RGA2
TCONS_00001250	-0.426877	-2.50041	-2.81792	BGIOSGA023186	XP_006644328.1 aldehyde dehydrogenase family 2 member C4-like
TCONS_00003849	-0.426774	-1.36036	-0.351403	BGIOSGA001365	XP_015699230.1 glyoxylate/succinic semialdehyde reductase 2, chloroplastic
TCONS_00005360	-0.426713	-0.806951	-0.592549	BGIOSGA005715	XP_006646841.1 phosphatidylinositol/phosphatidylcholine transfer protein SFH8-like
TCONS_00031185	-0.426674	0.214434	0.158064	BGIOSGA013366	XP_006661790.1 beta-glucuronosyltransferase GlcAT14A-like
TCONS_00032787	-0.425985	-0.0644566	-0.441453	BGIOSGA031323	XP_006651970.1PREDICTED: uncharacterized protein LOC102715103
TCONS_00033578	-0.425707	0.317743	-0.396415	BGIOSGA033889	XP_006663488.2PREDICTED: uncharacterized protein LOC102705674
TCONS_00024160	-0.425606	-0.0945747	-0.925495	BGIOSGA026197	XP_004958420.1dnaJ homolog subfamily C member 16
TCONS_00011178	-0.42545	-0.787131	-0.315447	BGIOSGA004226	XP_006651936.1PREDICTED: uncharacterized protein LOC102704790
TCONS_00025581	-0.425113	0.794248	0.658251	BGIOSGA018806	XP_003569731.1H/ACA ribonucleoprotein complex subunit 4
TCONS_00031249	-0.424886	-0.484417	0.239179	BGIOSGA007330	XP_006661824.1 protein ENHANCED DISEASE RESISTANCE 2-like isoform X1
TCONS_00022947	-0.424835	-2.06882	-2.02931	BGIOSGA035794	XP_004966531.1phytoene synthase, chloroplastic
TCONS_00004198	-0.424709	-0.774899	-0.20165	BGIOSGA001004	XP_006644556.1 IST1-like protein
TCONS_00022960	-0.424612	-0.68122	-0.40729	BGIOSGA024995	XP_015694499.1 putative pentatricopeptide repeat-containing protein At1g74580
TCONS_00008286	-0.424571	0.623882	0.117612	BGIOSGA014901	XP_003575257.160S ribosomal protein L6-3
TCONS_00023189	-0.424491	-0.532971	-0.796375	BGIOSGA011412	XP_003557517.1uncharacterized protein LOC100844809
TCONS_00036234	-0.424488	0.39857	-1.09536	BGIOSGA037593	XP_006664642.1 pentatricopeptide repeat-containing protein At2g31400, chloroplastic
TCONS_00009446	-0.424478	-0.428463	-0.563016	BGIOSGA008626	XP_006649514.1 phytoene dehydrogenase, chloroplastic/chromoplastic
TCONS_00016971	-0.424261	-0.139177	0.0827345	BGIOSGA005248	XP_006654902.1PREDICTED: uncharacterized protein LOC102708898
TCONS_00027777	-0.42411	-2.54783	-3.11017	BGIOSGA017615	XP_004971901.1uncharacterized protein LOC101779376
TCONS_00016198	-0.424045	-0.30548	-0.285907	BGIOSGA027460	XP_004976135.140S ribosomal protein S11
TCONS_00033273	-0.424039	-1.3485	-1.78774	BGIOSGA004786	XP_006662908.2 tetrapyrrole-binding protein, chloroplastic
TCONS_00008518	-0.424009	-0.333866	-0.130859	BGIOSGA005910	XP_006648902.1 ceramide kinase
TCONS_00027797	-0.423834	-3.64717	-0.68251	BGIOSGA028891	XP_006659292.1 fasciclin-like arabinogalactan protein 2
TCONS_00000471	-0.423471	0.0686828	0.185254	BGIOSGA029994	XP_003565871.1probable chlorophyll(ide) b reductase NYC1, chloroplastic
TCONS_00018578	-0.423309	-1.19523	-3.18529	BGIOSGA018857	XP_015693136.1 kinesin-1 heavy chain-like
TCONS_00029285	-0.423122	0.715769	0.709511	BGIOSGA031028	XP_004957252.1KH domain-containing protein HEN4
TCONS_00027171	-0.423097	1.03606	-1.52771	#N/A	#N/A
TCONS_00000375	-0.423036	-0.466479	-0.302491	BGIOSGA002950	XP_015691748.1 phosphoribosylaminoimidazole carboxylase, chloroplastic
TCONS_00009125	-0.422881	-0.547149	-0.692598	BGIOSGA031409	XP_006649224.1 cell number regulator 6

TCONS_00024276	-0.422853	-1.26619	-1.05714	BGIOSGA026322	XP_006658069.1 adenosine deaminase-like protein
TCONS_00028285	-0.422642	1.0911	0.134981	BGIOSGA034947	XP_006659574.1PREDICTED: uncharacterized protein LOC102709670
TCONS_00033086	-0.422405	0.410977	-1.66975	BGIOSGA031464	XP_003577768.1short-chain dehydrogenase TIC 32, chloroplastic
TCONS_00025270	-0.422186	0.467913	-1.30826	BGIOSGA025873	XP_006658656.1 cysteine-rich receptor-like protein kinase 10
TCONS_00012026	-0.421907	0.409505	-0.246669	BGIOSGA018625	XP_004984768.1ABC transporter B family member 6
TCONS_00004151	-0.421858	-1.17681	-1.54931	BGIOSGA028797	XP_010930421.1 putative 12-oxophytodienoate reductase 4 isoform X4
TCONS_00017101	-0.421674	#NA	-0.531222	BGIOSGA001865	XP_010231745.1uncharacterized protein LOC104582761
TCONS_00031386	-0.421616	-0.362641	0.338177	BGIOSGA031632	XP_006661902.1 KH domain-containing protein At4g18375-like
TCONS_00003932	-0.421401	0.0207331	-0.174432	BGIOSGA000883	XP_004969232.1cytochrome P450 CYP72A219
TCONS_00030286	-0.421231	-1.36287	0.335195	BGIOSGA002409	XP_021319375.1ubiquitin recognition factor in ER-associated degradation protein 1-like
TCONS_00017654	-0.421207	-1.1808	-2.86857	BGIOSGA005107	XP_006655238.1 beta-glucosidase 19-like
TCONS_00016050	-0.420923	0.329834	0.432763	BGIOSGA029454	XP_006653501.1PREDICTED: uncharacterized protein LOC102700798 isoform X4
TCONS_00035530	-0.420862	-0.57278	-0.592367	BGIOSGA001923	XP_015698006.1 hydroxymethylglutaryl-CoA lyase, mitochondrial-like
TCONS_00002116	-0.420366	0.265839	-2.27145	BGIOSGA001560	XP_004970463.1uncharacterized protein LOC101765839
TCONS_00012539	-0.420243	-0.464535	-0.535844	BGIOSGA010444	XP_006651539.1PREDICTED: uncharacterized protein LOC102709548 isoform X2
TCONS_00019884	-0.420067	1.96916	0.443357	BGIOSGA002090	XP_006654847.1 late embryogenesis abundant protein Lea14-A-like
TCONS_00034576	-0.419932	0.456898	-1.03122	BGIOSGA014125	XP_008678023.1probable sucrose-phosphate synthase 5
TCONS_00021597	-0.419684	0.0335929	-0.77755	BGIOSGA035035	XP_004964527.1coatomer subunit beta'-1
TCONS_00022765	-0.419585	-2.0739	-1.84282	BGIOSGA002704	XP_015693719.1 probable alpha-glucosidase Os06g0675700
TCONS_00022419	-0.419548	0.35424	1.04843	BGIOSGA034318	XP_006663348.1 F-box/LRR-repeat protein At5g02910-like
TCONS_00024129	-0.41918	0.355026	0.114376	BGIOSGA010606	XP_004958354.140S ribosomal protein S6
TCONS_00018045	-0.418948	-0.155616	0.295788	BGIOSGA020091	XP_010231224.1putative disease resistance protein RGA3
TCONS_00029640	-0.418927	-1.38058	-3.06722	BGIOSGA030180	XP_010942234.1 zinc finger protein CONSTANS-LIKE 2-like
TCONS_00021987	-0.41858	0.0238036	0.343563	BGIOSGA037876	XP_006655969.1 microtubule-associated protein 70-3 isoform X1
TCONS_00034119	-0.4181	0.177015	0.0512437	BGIOSGA036810	XP_006663709.1 mRNA turnover protein 4 homolog
TCONS_00025342	-0.417806	-2.73104	-1.81564	BGIOSGA027663	XP_006657833.1 organic cation/carnitine transporter 2-like
TCONS_00037063	-0.417715	-4.95419	-2.88243	BGIOSGA021563	XP_004973447.1acetylserotonin O-methyltransferase 2
TCONS_00003034	-0.417651	-1.25099	-2.79947	BGIOSGA020205	XP_006643879.1 serine/threonine-protein kinase HT1-like
TCONS_00032651	-0.417539	-0.477514	-0.882897	BGIOSGA021847	XP_006662030.1 U-box domain-containing protein 33-like isoform X1
TCONS_00016892	-0.417088	0.246074	0.0428014	BGIOSGA017340	XP_006653870.1 anamorsin homolog 1
TCONS_00032628	-0.416655	-0.385808	0.0411536	#N/A	#N/A
TCONS_00014492	-0.416366	-0.599664	0.0672375	BGIOSGA016653	XP_015692260.1 U-box domain-containing protein 6-like
TCONS_00032807	-0.416181	1.5728	-0.062934	BGIOSGA036849	XP_006663706.1 clathrin heavy chain 1-like
TCONS_00013281	-0.416167	-0.620627	-0.994759	BGIOSGA024823	XP_006650686.1 ferredoxin--NADP reductase, root isozyme, chloroplastic
TCONS_00024325	-0.416164	-0.48303	0.0285377	BGIOSGA026368	XP_015695255.1 probable histone-arginine methyltransferase CARM1 isoform X2
TCONS_00021643	-0.415879	-1.88688	-1.79065	BGIOSGA006045	XP_010238726.1uncharacterized protein LOC100837604 isoform X2
TCONS_00010177	-0.415714	-0.255507	-0.354731	BGIOSGA009256	XP_006650134.1 polypyrimidine tract-binding protein homolog 1
TCONS_00024628	-0.415409	1.83523	-0.546985	#N/A	#N/A
TCONS_00026655	-0.41517	-1.85385	-1.5177	BGIOSGA026192	XP_006659433.1 extracellular ribonuclease LE-like
TCONS_00034170	-0.414935	-2.85453	-2.96166	BGIOSGA036763	XP_006663745.1 probable chalcone--flavonone isomerase 3 isoform X1
TCONS_00004923	-0.414794	-0.0683854	0.349051	BGIOSGA000298	XP_015688115.1 vacuolar protein sorting-associated protein 53 A isoform X2
TCONS_00006174	-0.414701	-0.906445	0.773603	BGIOSGA005411	XP_006648681.1 adenylyltransferase and sulfurtransferase MOCS3-2
TCONS_00034844	-0.414675	-0.936953	-0.356213	BGIOSGA022395	XP_008659242.1RNA polymerase II transcription factor B subunit 3
TCONS_00025289	-0.414635	-0.107454	0.437247	BGIOSGA020149	XP_003562954.160S ribosomal protein L38
TCONS_00018684	-0.414541	0.0269416	-1.54918	#N/A	#N/A
TCONS_00002954	-0.414494	-0.799036	0.472399	BGIOSGA019301	XP_006643825.1 serine/threonine protein phosphatase 2A 57 kDa regulatory subunit B' iota isoform-like
TCONS_00037617	-0.414416	-0.818316	-0.712282	BGIOSGA035749	XP_006664256.1 sodium/hydrogen exchanger 8
TCONS_00024005	-0.414404	-0.552452	-0.345806	BGIOSGA010449	XP_006658752.1 interferon-related developmental regulator 2-like
TCONS_00035801	-0.414264	-1.19575	-0.450348	BGIOSGA021914	XP_006656635.1PREDICTED: uncharacterized protein LOC102718464
TCONS_00001399	-0.414125	-0.139877	-1.88129	BGIOSGA001171	XP_006646124.1 cytochrome P450 72A13-like isoform X2
TCONS_00007367	-0.413926	-0.0791633	-0.848014	BGIOSGA007108	XP_006646900.1 tyrosine-sulfated glycopeptide receptor 1-like
TCONS_00016621	-0.413766	-1.24434	-1.46138	BGIOSGA019492	XP_006653769.1 probable LRR receptor-like serine/threonine-protein kinase At1g56140

transcriptome

TCONS_00022807	-0.413727	1.48549	2.1492	BGIOSGA030948	XP_002438913.1EG45-like domain containing protein
TCONS_00022535	-0.413479	-1.38515	-0.805916	BGIOSGA035473	XP_006656223.1 RNA polymerase II degradation factor 1-like
TCONS_00004178	-0.413085	-0.47108	-0.65448	BGIOSGA020335	XP_006644525.1 cytochrome b561 and DOMON domain-containing protein At3g61750-like
TCONS_00018277	-0.412964	-1.23781	-0.678696	BGIOSGA013026	XP_015692696.1PREDICTED: uncharacterized protein LOC107304204
TCONS_00005159	-0.412912	-0.449785	0.388721	BGIOSGA000045	XP_006646726.1 protein phosphatase 1 regulatory subunit SDS22
TCONS_00025091	-0.412791	-0.869476	-1.14763	BGIOSGA016911	XP_006657697.1 rop guanine nucleotide exchange factor 3-like
TCONS_00032919	-0.412721	-1.97504	-1.55378	#N/A	#N/A
TCONS_00026000	-0.412484	#NA	#NA	#N/A	#N/A
TCONS_00013524	-0.412252	-0.3906	0.445024	BGIOSGA024477	XP_006650887.1PREDICTED: uncharacterized protein LOC102710858
TCONS_00028675	-0.412178	-1.03153	-0.347015	BGIOSGA024808	XP_006660492.1 DNA topoisomerase 6 subunit B
TCONS_00012174	-0.411803	-1.97607	0.0288398	BGIOSGA009369	XP_015689874.1 ABSCISIC ACID-INSENSITIVE 5-like protein 2
TCONS_00007721	-0.411748	0.0276037	0.305388	BGIOSGA024435	XP_015689619.1 spermine synthase-like
TCONS_00009037	-0.411371	1.8308	-0.557222	BGIOSGA005370	XP_006648103.1 exosome complex component CSL4
TCONS_00016560	-0.411189	-1.196	-1.08609	BGIOSGA020752	XP_002448471.1uncharacterized protein LOC8072288
TCONS_00015565	-0.411161	-0.861312	0.638642	BGIOSGA038270	XP_015691799.1 probable ribonuclease P/MRP protein subunit POP5 isoform X2
TCONS_00009740	-0.410915	-0.574959	0.673298	BGIOSGA005958	XP_006649807.1 LIM domain-containing protein WLIM2a
TCONS_00011033	-0.410754	0.344679	-1.55879	BGIOSGA022508	XP_002466388.1uncharacterized protein LOC8084189
TCONS_00029902	-0.410363	-1.31422	-0.663815	BGIOSGA037670	XP_004956654.1F-box/SPRY domain-containing protein 1
TCONS_00028859	-0.410361	-1.97769	#NA	BGIOSGA015169	XP_002460130.1heavy metal-associated isoprenylated plant protein 23
TCONS_00027606	-0.41035	-0.590022	-0.0810352	BGIOSGA006448	XP_021315203.1ankyrin repeat, PH and SEC7 domain containing protein secG
TCONS_00026262	-0.409909	-1.23553	-0.409656	BGIOSGA029172	XP_006659262.1PREDICTED: uncharacterized protein LOC102709863
TCONS_00009388	-0.40962	0.828816	1.10131	BGIOSGA027836	XP_015690387.1 receptor homology region, transmembrane domain- and RING domain-containing protein 1
TCONS_00007268	-0.409496	-0.344944	-0.0438336	BGIOSGA007215	XP_015688437.1PREDICTED: uncharacterized protein LOC102713243
TCONS_00025619	-0.409444	0.0212581	-0.562098	BGIOSGA025803	XP_003562563.1probable protein phosphatase 2C 65 isoform X1
TCONS_00011096	-0.409347	-0.732807	-0.219904	BGIOSGA009683	XP_015690101.1 nuclear pore complex protein NUP1-like
TCONS_00022126	-0.409302	-3.40997	-1.91044	BGIOSGA022767	XP_004965303.1aspartyl protease family protein At5g10770
TCONS_00011325	-0.409197	-1.15551	0.395729	BGIOSGA013935	XP_006665035.1 cyclin-dependent kinase F-3 isoform X2
TCONS_00003045	-0.409057	-0.21672	-0.246641	BGIOSGA023075	XP_006643882.2 aquaporin NIP1-2-like
TCONS_00011044	-0.409038	0.0313484	-0.751446	BGIOSGA015066	XP_015690199.1 kinesin-like protein KIF19 isoform X1
TCONS_00001788	-0.408941	-0.853864	-0.224908	BGIOSGA017050	XP_015691389.1 clathrin interactor EPSIN 1-like isoform X1
TCONS_00009560	-0.408857	0.534261	-0.105102	BGIOSGA032159	XP_006649626.1 hornerin-like
TCONS_00027858	-0.408809	0.336956	0.517183	BGIOSGA030071	XP_006659346.1 enhancer of polycomb-like protein 1
TCONS_00025387	-0.408706	-0.376549	-0.682512	BGIOSGA024040	XP_015695365.1 glutamyl-tRNA reductase-binding protein, chloroplastic
TCONS_00013580	-0.408612	-0.162399	0.258581	BGIOSGA009395	XP_006652051.2 ABC transporter G family member 3
TCONS_00012908	-0.408547	-0.200952	-0.621915	BGIOSGA037004	XP_006664348.2PREDICTED: uncharacterized protein LOC102703742 isoform X1
TCONS_00001903	-0.408322	0.803814	-0.205044	BGIOSGA004536	XP_006646378.2 dymeclin
TCONS_00006795	-0.408303	0.479356	0.0199634	BGIOSGA008968	XP_004953778.1basic leucine zipper 8
TCONS_00019916	-0.408212	1.48531	0.162567	BGIOSGA001439	XP_002441637.1B2 protein isoform X1
TCONS_00002302	-0.407968	-0.654822	-0.498151	BGIOSGA025413	XP_006645141.1 KH domain-containing protein At1g09660/At1g09670-like
TCONS_00030180	-0.407887	-1.18639	-0.728255	BGIOSGA020761	XP_015696385.1 ubiquitin carboxyl-terminal hydrolase 16-like
TCONS_00006277	-0.407857	0.189322	-1.56613	BGIOSGA034843	XP_004952758.1monooxygenase 1
TCONS_00014153	-0.407713	1.54279	-0.566498	#N/A	#N/A
TCONS_00012290	-0.407692	-0.355561	-0.00416172	BGIOSGA026315	XP_015689899.1 rho GTPase-activating protein 7-like
TCONS_00012183	-0.407528	0.550124	-0.471069	BGIOSGA001895	XP_003558003.1phospholipid-transporting ATPase 1
TCONS_00014808	-0.407487	-0.905434	-0.539289	BGIOSGA016979	XP_006652685.2 BTB/POZ domain-containing protein At1g63850
TCONS_00019296	-0.407253	-0.0296811	-0.120259	BGIOSGA025386	XP_006654405.2 probable mediator of RNA polymerase II transcription subunit 26b
TCONS_00028292	-0.407122	-0.289995	0.0520362	BGIOSGA007052	XP_015696036.1 AT-hook motif nuclear-localized protein 10
TCONS_00036103	-0.406902	-1.98174	0.431444	#N/A	#N/A
TCONS_00000128	-0.406669	-1.29851	-1.82962	BGIOSGA002700	XP_006643697.1 extended synaptotagmin-1
TCONS_00030737	-0.40665	-1.69826	-3.09276	BGIOSGA007410	XP_006661641.1 peptidyl-prolyl cis-trans isomerase
TCONS_00037124	-0.40665	-0.74104	-0.941165	BGIOSGA036247	NP_001148486.1DNA binding protein
TCONS_00027659	-0.406515	-0.555639	-0.209505	BGIOSGA025018	NP_001105685.1cysteine proteinase 1 precursor
TCONS_00006250	-0.406496	-0.407018	-0.320559	BGIOSGA018968	XP_006648726.1 uncharacterized zinc finger protein At4g06634

TCONS_00027481	-0.406316	0.54801	0.672315	BGIOSGA027530	XP_015695514.1 polyadenylation and cleavage factor homolog 4-like
TCONS_00015180	-0.406258	-1.12743	-0.491231	BGIOSGA033427	XP_006653885.1 RNA polymerase II transcription factor B subunit 2
TCONS_00005925	-0.406077	0.435809	-0.515625	BGIOSGA001979	XP_014755520.1oligosaccharyltransferase complex subunit ostc
TCONS_00025884	-0.405899	-1.00524	-0.833719	BGIOSGA027956	XP_006659098.1 polycomb group protein FIE2
TCONS_00020482	-0.405826	-2.98305	-0.571276	BGIOSGA012396	XP_015690126.1 protein trichome birefringence-like 33
TCONS_00002922	-0.405823	-1.75328	-1.23081	BGIOSGA037652	XP_006643812.2 protein NSP-INTERACTING KINASE 3 isoform X1
TCONS_00015626	-0.405819	-1.08585	-0.82792	BGIOSGA002830	XP_004975273.1serine carboxypeptidase 1
TCONS_00016131	-0.405646	-0.287574	-0.00449007	BGIOSGA025510	XP_006652431.1 serine/threonine protein phosphatase 2A 57 kDa regulatory subunit B' theta isoform-like
TCONS_00035142	-0.4055	-1.98344	-0.987135	BGIOSGA035536	XP_006663027.1 probable calcium-binding protein CML45
TCONS_00008761	-0.4055	1.79791	-0.25017	#N/A	#N/A
TCONS_00026422	-0.405409	-1.37242	-0.794985	BGIOSGA007471	XP_018681243.1 phosphatidylinositol/phosphatidylcholine transfer protein SFH13-like isoform X2
TCONS_00000308	-0.405234	-2.188	-0.759259	BGIOSGA002883	XP_006645560.1 tudor domain-containing protein 3 isoform X2
TCONS_00027846	-0.405104	0.493693	-0.431362	BGIOSGA008340	XP_004964325.1cellulose synthase-like protein D2
TCONS_00015243	-0.405098	0.370534	0.897841	BGIOSGA016407	XP_006664915.1 G-type lectin S-receptor-like serine/threonine-protein kinase At2g19130
TCONS_00023718	-0.40499	0.716368	-2.57338	BGIOSGA029037	XP_015694890.1 protein trichome birefringence-like 13
TCONS_00020046	-0.404958	-1.08078	-1.91848	BGIOSGA004722	XP_006656592.1 probable E3 ubiquitin-protein ligase XBOS36
TCONS_00007716	-0.404786	0.0928921	-0.279886	BGIOSGA033517	XP_006647123.1 serine/arginine-rich SC35-like splicing factor SCL30
TCONS_00013631	-0.404702	#NA	-1.57411	BGIOSGA017399	NP_001309935.1uncharacterized protein LOC103641406
TCONS_00015142	-0.404596	-1.70358	-1.37849	BGIOSGA017322	XP_006652983.1 actin-related protein 8
TCONS_00020601	-0.404469	0.309346	-0.306572	BGIOSGA031626	XP_002453753.2atherin isoform X1
TCONS_00025177	-0.403902	-3.20987	-3.97999	BGIOSGA006807	XP_006657749.1 anthocyanidin 3-O-glucosyltransferase 2-like
TCONS_00036122	-0.403788	-0.0337692	-1.97203	BGIOSGA037479	XP_015698607.1PREDICTED: uncharacterized protein LOC107305435
TCONS_00026746	-0.403669	0.353087	0.545276	BGIOSGA033400	XP_006660191.2 RING-H2 zinc finger protein RHA4a-like
TCONS_00035613	-0.403669	0.934851	-1.21414	BGIOSGA037350	XP_006664354.2 putative HVA22-like protein g
TCONS_00035960	-0.403361	-0.401137	-0.355097	BGIOSGA023949	XP_006663984.1 probable histone H2A.1
TCONS_00003497	-0.403249	-0.586183	-0.677427	BGIOSGA001705	XP_015699317.1 glycine-rich domain-containing protein 1
TCONS_00002213	-0.403069	0.335462	0.0998484	BGIOSGA000155	XP_022682596.1proline-rich receptor-like protein kinase PERK9 isoform X1
TCONS_00031313	-0.402929	-0.664715	-0.993614	BGIOSGA002127	XP_012701221.1GDSL esterase/lipase At5g62930 isoform X1
TCONS_00016179	-0.402929	0.150861	0.743352	BGIOSGA022183	XP_006652473.1 6,7-dimethyl-8-ribityllumazine synthase, chloroplastic
TCONS_00015900	-0.402793	-1.12432	-0.716423	BGIOSGA005583	XP_015691819.1 acetyltransferase At1g77540
TCONS_00012663	-0.402695	0.620588	0.718497	BGIOSGA032439	XP_006651582.1 nucleolar protein 14
TCONS_00003059	-0.402594	0.235327	0.0835458	#N/A	#N/A
TCONS_00006965	-0.402526	-0.835296	-0.60745	BGIOSGA016792	XP_015688365.1 polyadenylate-binding protein-interacting protein 7-like
TCONS_00013974	-0.402466	-0.402265	-0.772387	BGIOSGA035287	XP_006660481.1 cyclin-P4-1-like
TCONS_00036551	-0.402466	1.53633	2.27824	BGIOSGA036904	XP_003574085.1pentatricopeptide repeat-containing protein At1g61870, mitochondrial
TCONS_00021816	-0.402261	0.140006	-0.436334	BGIOSGA009113	XP_015694146.1PREDICTED: uncharacterized protein LOC102713003 isoform X1
TCONS_00002438	-0.402042	-0.909003	-1.15834	BGIOSGA032809	XP_006645250.2 calmodulin-binding transcription activator 4-like isoform X1
TCONS_00008251	-0.401957	-0.412732	-0.107184	BGIOSGA014944	XP_003575233.1cellulose synthase-like protein D2
TCONS_00031403	-0.40184	-0.919881	-1.06622	BGIOSGA033215	XP_003574132.1trafficking protein particle complex subunit 13
TCONS_00020596	-0.40149	-1.98846	0.195421	BGIOSGA000053	XP_015691456.1 putative UPF0496 protein 2
TCONS_00024070	-0.40136	-0.266866	-0.0905351	BGIOSGA026104	XP_006657944.1 acidic leucine-rich nuclear phosphoprotein 32-related protein 1
TCONS_00004653	-0.40133	-1.48416	-1.50294	BGIOSGA020039	XP_015690280.1 protein RETICULATA-RELATED 5, chloroplastic-like
TCONS_00023671	-0.401046	-0.0630253	-1.5833	BGIOSGA025695	XP_010238379.2zinc finger MYND domain-containing protein 15 isoform X1
TCONS_00029498	-0.401039	-0.765185	-0.722519	BGIOSGA027350	XP_006660970.1 probable cyclic nucleotide-gated ion channel 17
TCONS_00006130	-0.400984	0.130908	0.906142	BGIOSGA016276	XP_006647273.1PREDICTED: uncharacterized protein LOC102709990
TCONS_00007775	-0.400953	-4.5741	-0.361139	BGIOSGA029664	XP_002440001.1cytochrome P450 98A1
TCONS_00016147	-0.400772	1.06952	0.918518	BGIOSGA014828	XP_006652443.1 ribosome-recycling factor
TCONS_00008673	-0.400504	-0.353566	-0.00887261	BGIOSGA005757	XP_015689270.1 protein CROWDED NUCLEI 1-like
TCONS_00032866	-0.400434	-1.40484	0.322063	BGIOSGA036891	XP_004977391.1methylesterase 17
TCONS_00017018	-0.399629	-0.990817	-0.00187413	BGIOSGA019065	XP_006654927.1 nudix hydrolase 9
TCONS_00010282	-0.399614	0.316345	1.02415	BGIOSGA023912	XP_006651490.1 transcription initiation factor TFIID subunit 9
TCONS_00006841	-0.399602	-1.75926	-2.00379	BGIOSGA018887	XP_006647861.1 endoglucanase 7
TCONS_00002454	-0.399499	-0.575943	-0.587159	BGIOSGA004136	XP_006645271.2 inactive leucine-rich repeat receptor-like protein kinase CORYNE
TCONS_00005700	-0.399436	-0.99106	-3.17228	BGIOSGA001283	XP_003571132.1inorganic pyrophosphatase 2
TCONS_00015464	-0.399343	0.511323	0.149419	BGIOSGA015555	XP_006652118.2PREDICTED: uncharacterized protein LOC102702648

TCONS_00008969	-0.399283	-1.25429	-1.17266	BGIOSGA001234	XP_006648051.1 protein ABHD17B
TCONS_00017391	-0.399253	-0.832862	0.388417	BGIOSGA010719	XP_002440840.1E3 ubiquitin-protein ligase SINAT5
TCONS_00022217	-0.399253	-0.0244578	0.0402596	BGIOSGA021281	XP_006656065.2 uncharacterized exonuclease domain-containing protein At3g15140
TCONS_00017811	-0.398997	0.491001	0.194357	BGIOSGA026556	XP_003568448.1putative disease resistance protein RGA1
TCONS_00022842	-0.398897	0.349033	-1.00043	BGIOSGA009449	XP_015694104.1 DNA-directed RNA polymerases II, IV and V subunit 8B-like
TCONS_00001586	-0.398809	-0.184497	-0.418952	BGIOSGA002203	XP_006646212.1 transcription repressor MYB6-like
TCONS_00028245	-0.398681	-0.58602	-1.66319	BGIOSGA035813	XP_012702094.1uncharacterized protein LOC101753989
TCONS_00026171	-0.398607	-0.981218	0.267094	BGIOSGA016633	XP_006653543.1PREDICTED: uncharacterized protein LOC102713626, partial
TCONS_00005034	-0.398583	-0.992137	0.28503	BGIOSGA016484	XP_003562968.2beta-amylase isoform X1
TCONS_00003782	-0.398535	-1.67027	-0.00459581	BGIOSGA001430	XP_006644276.1PREDICTED: uncharacterized protein LOC102706509 isoform X1
TCONS_00025345	-0.398441	-0.226781	0.547712	BGIOSGA024085	XP_015695051.1PREDICTED: uncharacterized protein LOC102718855
TCONS_00003684	-0.39835	0.814924	0.146947	BGIOSGA001519	XP_006644233.1 F-box/kelch-repeat protein At5g43190-like
TCONS_00002635	-0.39835	-0.43989	-0.0374772	BGIOSGA021559	NP_001147221.1plant-specific domain TIGR01615 family protein
TCONS_00011105	-0.39824	-0.1181	-1.59029	BGIOSGA004547	XP_024317267.1probable carboxylesterase 15 isoform X2
TCONS_00013866	-0.398197	-0.992624	-0.590399	BGIOSGA038704	XP_014661014.1probable long-chain-alcohol O-fatty-acyltransferase 1
TCONS_00037118	-0.398175	0.180186	-0.441074	BGIOSGA017113	XP_010237281.1probable signal peptidase complex subunit 1
TCONS_00003819	-0.397853	0.289364	0.911948	BGIOSGA021953	XP_006646014.2 protein EARLY FLOWERING 3-like
TCONS_00037122	-0.397799	#NA	-5.05082	BGIOSGA014665	XP_006654645.1 O-methyltransferase ZRP4-like
TCONS_00032562	-0.397781	0.0068521	-0.591431	BGIOSGA016434	XP_004982611.1probable receptor-like protein kinase At4g10390
TCONS_00033480	-0.397744	-0.955719	-0.591522	BGIOSGA035274	XP_015697666.1 alpha-ketoglutarate-dependent dioxygenase alkB
TCONS_00012960	-0.397742	0.337058	-0.196374	BGIOSGA008396	XP_015690727.1 myosin-17-like
TCONS_00020497	-0.397708	1.32869	0.215743	BGIOSGA022669	XP_004965202.1uncharacterized protein LOC101760541
TCONS_00016631	-0.397655	0.109787	-0.0247032	BGIOSGA019500	XP_006652835.1 putative serine/threonine-protein kinase
TCONS_00011996	-0.39762	0.474996	-0.120525	BGIOSGA014627	XP_006649840.1 protein kinase APK1B, chloroplastic-like isoform X1
TCONS_00027372	-0.397568	-0.713307	0.848614	BGIOSGA027629	XP_015695820.1 meiotic recombination protein SPO11-2 isoform X2
TCONS_00017176	-0.397485	-1.99352	-2.59217	BGIOSGA018602	XP_015692487.1 gibberellin 2-beta-dioxygenase 6-like
TCONS_00004177	-0.397485	-0.0083177	-0.288012	BGIOSGA028720	XP_003569564.1serine/arginine repetitive matrix protein 1
TCONS_00030038	-0.397468	-0.119072	0.373028	BGIOSGA029770	XP_006660620.1 activating signal cointegrator 1
TCONS_00032638	-0.397436	#NA	-0.422362	BGIOSGA036535	XP_015697066.1 transcription factor bHLH93-like
TCONS_00023457	-0.397414	-1.05736	-0.919299	BGIOSGA032077	XP_006657608.1PREDICTED: uncharacterized protein LOC102705544 isoform X2
TCONS_00006415	-0.39734	-0.363652	-0.126859	BGIOSGA004987	XP_006647485.1 CMP-sialic acid transporter 5
TCONS_00030426	-0.397325	3.01751	1.45183	BGIOSGA013750	XP_015696435.1 probable receptor-like protein kinase At5g47070
TCONS_00005189	-0.397089	-1.84202	-1.30364	BGIOSGA002861	XP_015696335.1 probable serine/threonine-protein kinase NAK
TCONS_00026333	-0.396988	-0.865254	-0.236572	BGIOSGA028414	XP_004978471.1triphosphate tunel metalloenzyme 3
TCONS_00026619	-0.396946	-1.11191	-0.759182	BGIOSGA030677	XP_006659403.1PREDICTED: uncharacterized protein LOC102706958
TCONS_00020976	-0.396811	-1.2574	-2.59383	BGIOSGA023198	XP_003560615.2uncharacterized protein LOC100839355
TCONS_00009363	-0.396799	0.994301	0.428504	BGIOSGA001322	XP_006651063.1 protein jagged-1-like isoform X1
TCONS_00011567	-0.396643	0.207055	-0.456745	BGIOSGA036001	XP_022679297.1capping protein, Arp2/3 and myosin-I linker protein 3
TCONS_00032783	-0.39662	-1.12014	-1.85734	BGIOSGA011778	XP_006662642.2 4-coumarate--CoA ligase-like 2
TCONS_00029082	-0.396585	-0.80774	-2.41056	BGIOSGA001663	XP_003578168.1respiratory burst oxidase homolog protein E
TCONS_00033407	-0.396553	-0.879215	-2.59447	BGIOSGA020673	XP_004970253.1protein RICE SALT SENSITIVE 3 isoform X1
TCONS_00023001	-0.396531	-3.14672	-1.16157	BGIOSGA025034	XP_006658217.1 flavin-containing monooxygenase FMO GS-OX-like 8
TCONS_00012005	-0.396523	-0.918201	0.0554176	BGIOSGA011004	XP_006651251.1 beta-glucuronosyltransferase GlcAT14A-like
TCONS_00018664	-0.396236	-2.3736	-0.540809	BGIOSGA031478	XP_015692730.1 serine carboxypeptidase-like 33
TCONS_00030317	-0.396116	-0.509815	-0.91748	BGIOSGA028353	XP_015696666.1 probable glucan endo-1,3-beta-glucosidase A6
TCONS_00009496	-0.395898	1.86247	-2.18105	BGIOSGA028895	XP_006649564.1 polyol transporter 5-like
TCONS_00034862	-0.395711	-0.051246	-0.55629	BGIOSGA033995	XP_010237729.1O-fucosyltransferase 1
TCONS_00016953	-0.395641	-0.410876	0.277744	BGIOSGA021227	XP_006653941.1 formin-like protein 14
TCONS_00005973	-0.395574	-0.0445779	-0.680219	BGIOSGA002503	XP_015698872.1 protein O-linked-mannose beta-1,4-N-acetylglucosaminyltransferase 2-like
TCONS_00012272	-0.395561	0.735865	0.98374	BGIOSGA010732	XP_006650080.1 probable nucleolar protein 5-2
TCONS_00030361	-0.395548	0.103581	0.502581	BGIOSGA005021	XP_006661435.1 3beta-hydroxysteroid-dehydrogenase/decarboxylase isoform 2
TCONS_00025691	-0.395491	-1.20796	-0.517308	BGIOSGA023732	XP_006658107.1 probable E3 ubiquitin-protein ligase BAH1-like 1

TCONS_00019805	-0.395455	0.849757	0.312302	BGIOSGA017550	XP_010231016.1eukaryotic translation initiation factor 3 subunit D
TCONS_00011851	-0.395455	-1.73304	-0.164226	BGIOSGA033506	XP_006649697.1 protein NRT1/ PTR FAMILY 8.3-like
TCONS_00013299	-0.395401	0.588823	0.26662	BGIOSGA013696	XP_015690101.1 nuclear pore complex protein NUP1-like
TCONS_00037607	-0.395378	-1.78972	-1.29782	BGIOSGA017996	XP_006664784.2 protein NRT1/ PTR FAMILY 2.11-like
TCONS_00020593	-0.395343	-0.130229	0.0333059	BGIOSGA002129	XP_003563908.3mannan endo-1,4-beta-mannosidase 6
TCONS_00007225	-0.395326	0.00376686	-2.81989	BGIOSGA032365	XP_006646775.1 wall-associated receptor kinase 2-like
TCONS_00016127	-0.395245	0.536699	0.615291	BGIOSGA014849	XP_006653536.1 kelch domain-containing protein 4
TCONS_00036892	-0.395114	-0.286007	0.586398	BGIOSGA036428	XP_015698840.1 protein CARMIL isoform X1
TCONS_00013440	-0.394879	-0.826869	-1.83307	BGIOSGA011074	NP_001147835.2lectin-like receptor kinase 7 precursor
TCONS_00037010	-0.394644	-1.71078	-2.00254	BGIOSGA028008	XP_015698269.1 oxysterol-binding protein-related protein 1D-like
TCONS_00006964	-0.394624	-0.774721	-0.52123	#N/A	XP_015689588.1PREDICTED: uncharacterized protein LOC102711129 isoform X3
TCONS_00033200	-0.394573	-0.0429819	-0.692468	BGIOSGA034264	XP_021317317.1disease resistance protein RPM1
TCONS_00004144	-0.394512	1.15405	0.0399012	BGIOSGA036689	XP_006644498.1 putative receptor protein kinase ZmPK1
TCONS_00027060	-0.394495	0.0529407	0.285253	BGIOSGA029110	XP_004974083.1sorbitol dehydrogenase
TCONS_00023527	-0.394297	-1.19017	-1.48456	BGIOSGA025560	XP_002459755.2poly [ADP-ribose] polymerase 1
TCONS_00030764	-0.394234	-0.269682	-1.51273	BGIOSGA034259	XP_015698934.1 disease resistance protein RPM1-like
TCONS_00014161	-0.393788	0.272746	0.0605694	BGIOSGA002822	NP_001105561.1cytochrome b-c1 complex subunit Rieske, mitochondrial precursor
TCONS_00020871	-0.393725	-0.0623706	0.0582157	BGIOSGA024975	XP_012701148.2kinesin-like protein KIN-14M isoform X1
TCONS_00012953	-0.393603	-1.01807	-0.973173	BGIOSGA010036	XP_010231508.1GATA transcription factor 18 isoform X1
TCONS_00025188	-0.393203	1.19004	-0.594109	BGIOSGA025793	XP_021308049.1ATP synthase subunit gamma, chloroplastic
TCONS_00003542	-0.392992	-1.22567	0.0172986	BGIOSGA030829	XP_006644164.1 respiratory burst oxidase homolog protein B
TCONS_00028945	-0.392756	-0.92808	0.0650124	BGIOSGA017576	XP_003578076.1protein EDS1L isoform X1
TCONS_00006293	-0.392387	0.628926	-0.186917	BGIOSGA021964	XP_006648760.1PREDICTED: uncharacterized protein LOC102705507
TCONS_00019241	-0.392321	-1.49903	-0.89934	BGIOSGA002154	XP_006655292.1 peptide-N4-(N-acetyl-beta-glucosaminyl)asparagine amidase A
TCONS_00014277	-0.392275	0.488915	-0.262731	BGIOSGA016432	XP_006652294.1 40S ribosomal protein S10
TCONS_00027714	-0.391726	-1.11615	-0.103228	BGIOSGA033572	XP_015695750.1 succinate dehydrogenase assembly factor 1, mitochondrial isoform X2
TCONS_00002149	-0.391632	0.657616	-1.60873	BGIOSGA013862	XP_006644992.1 scarecrow-like protein 9
TCONS_00031099	-0.391622	-1.32281	-1.22302	BGIOSGA032920	XP_015697374.1 acylamino-acid-releasing enzyme-like
TCONS_00035586	-0.391458	-0.492668	-0.772122	BGIOSGA016731	XP_004978373.1sialyltransferase-like protein 2
TCONS_00031388	-0.391422	-0.619823	-0.0568851	BGIOSGA017353	XP_020408564.1uncharacterized protein LOC100284779 isoform X2
TCONS_00021449	-0.391369	-0.224706	0.053192	BGIOSGA007340	XP_006656547.1 protein transport protein SEC16B homolog
TCONS_00008740	-0.391079	-0.0223608	-1.3471	BGIOSGA005694	XP_004953779.1serine protease SPPA, chloroplastic
TCONS_00020398	-0.390825	-0.573266	-0.0739055	BGIOSGA000832	XP_015688184.1 homeobox protein HOX1A isoform X2
TCONS_00018100	-0.39061	0.0440455	-0.547343	BGIOSGA000725	XP_006654620.1 MATH domain-containing protein At5g43560-like
TCONS_00032626	-0.390593	-1.28565	-0.735762	BGIOSGA015052	XP_002465010.1RING-H2 finger protein ATL80
TCONS_00024607	-0.39049	0.521426	-0.0742443	BGIOSGA011765	XP_002461493.1calcium-dependent protein kinase 17
TCONS_00018491	-0.390236	-1.01718	0.172757	BGIOSGA018942	XP_006654190.1PREDICTED: uncharacterized protein LOC102712167 isoform X1
TCONS_00018847	-0.390164	0.00194368	-0.304361	BGIOSGA012162	XP_003568732.1proline-rich receptor-like protein kinase PERK1 isoform X1
TCONS_00014703	-0.389732	0.115934	0.349038	BGIOSGA016877	XP_006653644.1PREDICTED: uncharacterized protein At3g06530
TCONS_00018059	-0.38949	-1.86316	-1.41544	BGIOSGA005155	XP_015692829.1 putative glucan endo-1,3-beta-glucosidase GVI
TCONS_00020630	-0.38946	-0.521577	0.0194785	BGIOSGA022812	XP_006656043.2 diacylglycerol O-acyltransferase 2
TCONS_00016584	-0.389056	0.256851	0.282773	BGIOSGA028216	XP_006652792.1 26S proteasome non-ATPase regulatory subunit 1 homolog A-like
TCONS_00019415	-0.388956	-0.0793057	-1.77919	BGIOSGA019196	XP_006654494.1 splicing factor 1
TCONS_00004613	-0.388834	0.561061	0.659732	BGIOSGA017960	XP_006644895.1 F-box protein SKIP24 isoform X1
TCONS_00011532	-0.388819	0.366756	0.344331	BGIOSGA032530	XP_004985790.1putative aconitate hydratase, cytoplasmic
TCONS_00000049	-0.388743	-0.0274367	-0.10743	BGIOSGA002615	XP_006661048.1 prolyl endopeptidase-like
TCONS_00005515	-0.388729	-2.54061	-1.90561	BGIOSGA007624	XP_006646940.1 glycine cleavage system H protein 2, mitochondrial-like
TCONS_00000008	-0.3886	-0.712404	-1.09182	BGIOSGA022903	XP_015699138.1 putative pentatricopeptide repeat-containing protein At2g01510
TCONS_00006041	-0.38854	0.581575	-0.188559	BGIOSGA016140	XP_003574969.1importin beta-like SAD2
TCONS_00011085	-0.388194	1.35296	-1.1364	BGIOSGA017164	XP_006650695.1 peroxisomal (S)-2-hydroxy-acid oxidase GLO1 isoform X1
TCONS_00038038	-0.387847	-0.691783	-0.215009	BGIOSGA035211	XP_006663976.1 serine hydroxymethyltransferase 4-like
TCONS_00002980	-0.387768	0.121439	-0.200286	BGIOSGA002239	XP_002455041.1phosducin-like protein 3
TCONS_00012627	-0.387696	0.0166258	0.147662	BGIOSGA010346	XP_006651575.1 paladin

transcriptome

TCONS_00002511	-0.387571	0.343634	-0.207098	BGIOSGA005146	XP_021313101.1NAC domain-containing protein 48-like
TCONS_00034562	-0.387362	0.252303	0.0178346	BGIOSGA032568	XP_022684857.1disease resistance protein RPM1
TCONS_00004340	-0.38728	-1.91836	-3.06077	BGIOSGA003137	XP_006646291.1 pathogen-related protein
TCONS_00008776	-0.387255	-0.237472	-0.915462	BGIOSGA007683	XP_006649028.1 dynamin-2A-like
TCONS_00021033	-0.387189	-0.882268	0.631783	BGIOSGA006954	XP_006656245.1 zinc finger A20 and AN1 domain-containing stress-associated protein 8
TCONS_00010893	-0.386954	1.48123	0.0784274	BGIOSGA026386	XP_006650540.1 leishmanolysin homolog
TCONS_00017704	-0.386944	-0.399299	0.190309	BGIOSGA037065	XP_003566307.1calmodulin binding protein PICBP
TCONS_00016180	-0.38689	0.252427	0.719986	BGIOSGA006070	XP_006653562.1 la protein 1
TCONS_00019943	-0.386731	-0.446895	-0.548178	BGIOSGA007266	XP_006656537.2PREDICTED: uncharacterized protein LOC102711075
TCONS_00036166	-0.386697	-0.165639	-0.614925	BGIOSGA031717	XP_015698810.1 conserved oligomeric Golgi complex subunit 8
TCONS_00021902	-0.386492	0.632573	1.02988	BGIOSGA001038	XP_012701130.1spastin isoform X1
TCONS_00003060	-0.386475	-1.90937	-1.53209	BGIOSGA017870	XP_006643890.1 CBL-interacting protein kinase 5
TCONS_00030376	-0.386463	0.0168343	-0.489061	BGIOSGA029422	XP_006660855.2 probable sodium/metabolite cotransporter BASS5, chloroplastic
TCONS_00033286	-0.385911	-4.01939	-2.44434	BGIOSGA019828	XP_003573587.1endo-1,3;1,4-beta-D-glucanase isoform X1
TCONS_00019225	-0.385901	-1.31005	-0.764651	BGIOSGA018123	XP_015693278.1 carbon catabolite repressor protein 4 homolog 4
TCONS_00014178	-0.385828	0.0389019	-1.63815	BGIOSGA016334	XP_002446391.2acetyl-coenzyme A synthetase, chloroplastic/glyoxysomal
TCONS_00032514	-0.385174	-0.900375	0.495861	BGIOSGA031608	XP_015697042.1 thylakoid lumenal 17.4 kDa protein, chloroplastic
TCONS_00036740	-0.384986	-0.239072	-0.0935242	BGIOSGA025996	XP_015698726.1 tubby-like F-box protein 14
TCONS_00034390	-0.384696	0.851087	0.387702	BGIOSGA001884	XP_006662772.1 coatomer subunit beta-1
TCONS_00027633	-0.384426	-0.111256	0.178331	BGIOSGA027384	XP_006659241.1PREDICTED: uncharacterized protein LOC102703601
TCONS_00036513	-0.383984	-0.297068	0.0801637	BGIOSGA021528	XP_006664255.1 microtubule-associated protein 70-1
TCONS_00035216	-0.383819	-1.44248	-2.5098	BGIOSGA033307	XP_015697901.1 stress enhanced protein 1, chloroplastic-like isoform X1
TCONS_00025357	-0.38378	0.235531	0.202298	BGIOSGA016128	XP_015695302.1 probable protein phosphatase 2C 64
TCONS_00018611	-0.383768	0.523496	1.32874	BGIOSGA018823	XP_004960466.1ribosome biogenesis protein NOP53
TCONS_00009720	-0.383634	0.388954	0.119036	BGIOSGA014480	XP_015690363.1 partner of Y14 and mago isoform X2
TCONS_00010460	-0.3836	-1.95386	-0.702054	BGIOSGA019995	XP_006650299.1PREDICTED: uncharacterized protein LOC102704686
TCONS_00024306	-0.383457	0.330302	0.0337762	BGIOSGA006631	XP_012699221.1probable serine/threonine-protein kinase At1g54610
TCONS_00020049	-0.383304	-0.172898	0.188697	BGIOSGA016954	XP_006655742.1 SPX domain-containing membrane protein Os06g0129400 isoform X1
TCONS_00018071	-0.383295	-3.73469	-2.66701	BGIOSGA023014	XP_006655455.1 peroxidase 1-like
TCONS_00005788	-0.383188	-1.28064	0.241772	BGIOSGA006847	XP_006647127.1 abscisic acid receptor PYL8-like
TCONS_00014169	-0.383101	-0.856831	-0.181865	BGIOSGA007063	XP_006652239.1 OTU domain-containing protein DDB_G0284757
TCONS_00016068	-0.382792	0.832401	-0.48102	BGIOSGA001201	XP_004975924.1probable amino acid permease 7 isoform X2
TCONS_00018295	-0.382434	0.623157	-0.359485	BGIOSGA034862	XP_006654779.1 tubby-like F-box protein 10
TCONS_00018827	-0.382361	-1.55217	0.341764	BGIOSGA002114	XP_006654155.1PREDICTED: uncharacterized protein LOC102701914
TCONS_00010720	-0.382083	-3.08008	-2.70052	BGIOSGA013344	XP_015690018.1 magnesium transporter MRS2-A, chloroplastic
TCONS_00005984	-0.381543	-0.712027	-0.25475	BGIOSGA008116	XP_006647236.1 cyclin-T1-2-like
TCONS_00004086	-0.381461	0.743581	-0.00434889	BGIOSGA001105	XP_006658273.1PREDICTED: uncharacterized protein LOC102709386
TCONS_00009760	-0.380842	-0.207193	0.274767	BGIOSGA012308	XP_006647280.2PREDICTED: uncharacterized protein At1g76660, partial
TCONS_00005765	-0.380754	-1.16791	0.848668	BGIOSGA022587	XP_006657995.2 probable mannan synthase 7
TCONS_00026720	-0.380719	0.421504	0.126021	BGIOSGA014038	XP_006653070.1 cationic peroxidase SPC4-like
TCONS_00028740	-0.380697	0.92434	-0.305918	BGIOSGA036103	XP_021906424.1 putative disease resistance protein At3g14460
TCONS_00019993	-0.380685	-1.33675	0.71805	BGIOSGA022081	XP_004964337.1F-box protein At2g26160 isoform X1
TCONS_00006110	-0.3806	-0.880885	-1.43398	BGIOSGA013604	XP_015688603.1 isoflavone 2'-hydroxylase-like
TCONS_00017652	-0.38058	0.745091	-0.160708	BGIOSGA008421	XP_006654311.1 pentatricopeptide repeat-containing protein At1g10270-like
TCONS_00009241	-0.380487	-0.20845	0.831721	BGIOSGA014124	XP_006649330.1 probable adenylate kinase 1, chloroplastic
TCONS_00006640	-0.380436	0.189964	0.175372	BGIOSGA016952	XP_006647681.1 zinc finger CCCH domain-containing protein 17
TCONS_00034680	-0.380429	-1.77018	-1.77642	BGIOSGA004783	XP_006663364.1 probable glutathione peroxidase 2
TCONS_00018973	-0.379846	-0.146423	-0.303954	BGIOSGA008318	XP_006654212.1 peroxisome biogenesis protein 2
TCONS_00006743	-0.379727	0.726083	-0.172751	BGIOSGA020304	XP_006647789.1 thioredoxin reductase NTRB
TCONS_00006643	-0.379631	0.0421051	-1.68934	BGIOSGA024641	XP_015689649.1 bifunctional dTDP-4-dehydrorhamnose 3,5-epimerase/dTDP-4-dehydrorhamnose reductase isoform X1
TCONS_00010514	-0.379451	-0.890788	0.0481739	BGIOSGA013124	XP_006651602.2 calcium-transporting ATPase 2, plasma membrane-type-like
TCONS_00005298	-0.379104	-1.21716	-1.16218	BGIOSGA005721	XP_006646798.1 ubiquitin-conjugating enzyme E2 28-like
TCONS_00036411	-0.379018	-0.444753	0.492846	BGIOSGA037777	XP_015698704.1 protein RRNAD1
TCONS_00015979	-0.37869	-1.39055	-3.1066	BGIOSGA015002	XP_006653461.1 putative glutathione-specific gamma-glutamylcyclotransferase 2

TCONS_00031361	-0.378663	-0.721407	-0.519612	BGIOSGA037141	XP_004982773.1probable protein arginine N-methyltransferase 6.2
TCONS_00005585	-0.378274	-0.18218	0.56544	BGIOSGA007693	XP_015689617.1 DNA ligase 1
TCONS_00020496	-0.378079	-1.69442	-1.57562	BGIOSGA017849	XP_015693542.1 aspartic proteinase Asp1-like
TCONS_00012062	-0.377928	-2.65959	-3.49194	BGIOSGA024410	XP_015689854.1 omega-3 fatty acid desaturase, chloroplastic-like
TCONS_00004008	-0.377804	-0.402265	0.0380109	BGIOSGA020455	XP_006646118.1 probable protein ABIL1
TCONS_00033004	-0.377496	-0.823152	0.196219	BGIOSGA029381	XP_006662751.1 two-component response regulator-like APRR3
TCONS_00013219	-0.377197	-0.0888835	0.417693	BGIOSGA009768	XP_006650637.1PREDICTED: uncharacterized protein LOC102709259
TCONS_00016375	-0.376837	-1.05534	-0.489891	BGIOSGA014547	XP_003580282.1O-fucosyltransferase 29 isoform X2
TCONS_00008625	-0.376775	-0.947141	-0.989457	BGIOSGA005799	XP_004953528.1serine/threonine-protein kinase/endoribonuclease IRE1 isoform X1
TCONS_00008531	-0.376494	0.0896019	0.414056	BGIOSGA005897	XP_006647636.2 peroxisome biogenesis protein 19-1-like
TCONS_00018476	-0.37649	-0.115637	-0.749076	BGIOSGA038236	NP_001141005.1lysine ketoglutarate reductase trans-splicing 1
TCONS_00004941	-0.376321	-0.0204833	1.0546	BGIOSGA000280	XP_006645205.1 neuroguidin
TCONS_00013711	-0.37612	-1.1351	-0.14265	BGIOSGA030026	XP_006652095.1 polycomb group protein EMBRYONIC FLOWER 2-like isoform X1
TCONS_00007295	-0.376	0.670864	0.504198	BGIOSGA007187	XP_006660402.2 ras GTPase-activating protein-binding protein 2
TCONS_00027809	-0.375963	0.113667	-0.4855	BGIOSGA001806	XP_006661616.1 cation-chloride cotransporter 1 isoform X1
TCONS_00020102	-0.375878	-1.64075	-0.76931	BGIOSGA022283	XP_006656627.1 carboxypeptidase SOL1
TCONS_00023709	-0.375325	-1.46437	-0.310997	BGIOSGA025749	XP_015695371.1 protein FAR1-RELATED SEQUENCE 6-like isoform X1
TCONS_00011145	-0.375194	-0.0825353	0.000871031	#N/A	#N/A
TCONS_00035664	-0.375029	0.847038	0.361045	BGIOSGA031249	XP_006664390.1 transmembrane 9 superfamily member 1-like
TCONS_00017037	-0.374652	-0.288449	-0.79276	BGIOSGA020997	XP_006653991.1 tetraspanin-3
TCONS_00004355	-0.374422	-0.980779	0.183539	BGIOSGA009861	XP_015699165.1 protein farnesyltransferase subunit beta
TCONS_00037399	-0.37417	0.656457	1.00066	BGIOSGA033517	XP_006647123.1 serine/arginine-rich SC35-like splicing factor SCL30
TCONS_00034622	-0.374088	-3.59816	-1.69622	BGIOSGA035074	XP_006662860.1 ankyrin repeat-containing protein At3g12360-like
TCONS_00004646	-0.373906	-0.289731	0.528469	BGIOSGA011273	XP_015691471.1 U-box domain-containing protein 16-like
TCONS_00014357	-0.373715	0.41923	0.841245	BGIOSGA029032	XP_015696817.1PREDICTED: uncharacterized protein LOC102718134, partial
TCONS_00026584	-0.373443	-2.41647	-3.38684	BGIOSGA012770	XP_006660121.1 probable trehalose-phosphate phosphatase 6
TCONS_00000013	-0.373426	0.97285	-0.693124	BGIOSGA002584	XP_015691360.1 thylakoid lumenal 17.9 kDa protein, chloroplastic
TCONS_00013877	-0.373356	-1.72488	-0.247674	BGIOSGA016056	XP_006652152.1PREDICTED: uncharacterized protein LOC102717057
TCONS_00005257	-0.373313	0.110421	-0.383581	BGIOSGA009278	XP_006646776.1 60S ribosomal protein L37-1-like
TCONS_00024420	-0.372982	-1.60968	-1.30013	BGIOSGA010151	XP_015695221.1 proline transporter 1
TCONS_00019571	-0.372911	-0.942273	-1.12898	BGIOSGA028119	XP_006654597.2PREDICTED: uncharacterized protein LOC102715941, partial
TCONS_00024170	-0.3728	-3.12183	-0.569001	BGIOSGA017057	XP_006658006.1 pectin acetylesterase 12-like isoform X1
TCONS_00030359	-0.372787	0.601756	0.178096	BGIOSGA004940	XP_015696740.1 flowering time control protein FPA
TCONS_00004836	-0.372731	0.349991	0.97333	BGIOSGA019903	XP_006645128.1 F-box protein SKP2A-like
TCONS_00037558	-0.372706	-0.933106	-0.0462298	BGIOSGA035805	XP_015698471.1 plant cysteine oxidase 2-like
TCONS_00026810	-0.372689	-0.599484	-0.779475	BGIOSGA028870	XP_003574665.1trihelix transcription factor ASIL1
TCONS_00002394	-0.372524	0.0943172	-0.411454	BGIOSGA005030	XP_006645207.2 snRNA-activating protein complex subunit isoform X1
TCONS_00012057	-0.372518	-0.67625	-0.255331	BGIOSGA010951	XP_006649896.1 protein disulfide isomerase-like 5-1
TCONS_00026197	-0.372483	-0.554903	-0.0403372	BGIOSGA034239	XP_015696084.1 RNA polymerase sigma factor sigF, chloroplastic
TCONS_00028248	-0.372481	-0.538537	1.19502	BGIOSGA026732	XP_006659549.1 pre-mRNA-splicing factor CWC25 homolog
TCONS_00007030	-0.372419	0.718997	-0.0589819	BGIOSGA009212	XP_015688980.1 alpha-1,6-mannosyl-glycoprotein 2-beta-N-acetylglucosaminyltransferase
TCONS_00018221	-0.372249	0.221693	0.399376	BGIOSGA013345	XP_012700357.2glycosyltransferase family protein 64 protein C5
TCONS_00000152	-0.372125	0.157947	0.556535	BGIOSGA002732	XP_006645476.1 probable receptor-like protein kinase At1g67000
TCONS_00011815	-0.372048	0.0282075	0.281603	BGIOSGA011195	XP_006649657.1 protein VAC14 homolog isoform X2
TCONS_00002707	-0.371919	-1.45898	-2.7659	BGIOSGA002517	XP_015695305.1 probable receptor-like protein kinase At1g67000
TCONS_00013148	-0.371677	-0.589475	-0.225902	BGIOSGA031473	XP_004981821.1magnesium transporter MRS2-I
TCONS_00006337	-0.371654	-0.483562	-0.476333	BGIOSGA008500	XP_006408496.2 GPI ethanolamine phosphate transferase 1
TCONS_00022805	-0.371593	-0.45867	0.168542	BGIOSGA018156	XP_012701107.1probable methyltransferase PMT9
TCONS_00018759	-0.371498	-0.30959	-0.060788	BGIOSGA021040	XP_015692753.1 stem 28 kDa glycoprotein-like
TCONS_00005907	-0.371404	-1.73117	-0.0503738	BGIOSGA004832	XP_015691946.1 DNA excision repair protein ERCC-8-like
TCONS_00016192	-0.371354	0.0415347	-0.323583	BGIOSGA015068	XP_006657561.1 60S ribosomal protein L23A-like
TCONS_00004754	-0.371191	-1.89494	-0.816437	BGIOSGA000461	XP_012702009.2probable 3-deoxy-D-manno-octulosonic acid transferase, mitochondrial

TCONS_00021030	-0.371136	-0.346738	-0.197925	BGIOSGA016665	XP_006656244.2 DNA-directed RNA polymerase I subunit 1
TCONS_00008751	-0.371127	1.62021	0.671825	BGIOSGA022668	XP_015689245.1 aspartic proteinase Asp1-like
TCONS_00026858	-0.371052	-1.52371	-1.41929	BGIOSGA027808	XP_006659533.1 BTB/POZ domain-containing protein At1g55760
TCONS_00035617	-0.370644	-0.189926	0.760336	BGIOSGA022378	XP_006663840.1 protein FAR1-RELATED SEQUENCE 5-like
TCONS_00027254	-0.370432	1.73139	0.164916	BGIOSGA030912	XP_006659067.1 probable calcium-binding protein CML7
TCONS_00026645	-0.370008	0.117261	0.0925232	BGIOSGA030844	XP_006660148.1PREDICTED: uncharacterized protein LOC102702211, partial
TCONS_00033801	-0.370003	0.11211	-0.365204	BGIOSGA012517	XP_006665135.1 histone-lysine N-methyltransferase, H3 lysine-9 specific SUVH3-like
TCONS_00011739	-0.369913	0.773994	-0.0826276	BGIOSGA011268	XP_015690948.1 peptidyl-prolyl cis-trans isomerase CYP65
TCONS_00022160	-0.369896	-1.13652	-0.0739978	BGIOSGA008923	XP_006656038.1PREDICTED: uncharacterized protein LOC102706012
TCONS_00020575	-0.369679	0.0542513	-0.0652852	BGIOSGA019348	XP_006657621.1 RNA polymerase I termination factor isoform X1
TCONS_00029167	-0.369679	-1.07157	-1.5057	BGIOSGA034755	XP_015696813.1 2-hydroxyisoflavanone dehydratase-like
TCONS_00004232	-0.369622	-0.606978	-0.867286	BGIOSGA000973	XP_006644535.2 putative D-cysteine desulfhydrase 2, mitochondrial
TCONS_00009696	-0.36945	0.365908	0.223658	BGIOSGA020192	XP_004984992.3receptor-like serine/threonine-protein kinase At2g45590
TCONS_00030339	-0.369447	0.350014	0.0171129	BGIOSGA026571	XP_006660819.1 probable protein S-acyltransferase 7
TCONS_00005578	-0.36923	0.662909	-0.867764	BGIOSGA017000	XP_006646990.1 ent-cassadiene C2-hydroxylase-like
TCONS_00013264	-0.36912	-0.148733	0.251293	BGIOSGA009723	XP_015690835.1 protein FAR1-RELATED SEQUENCE 5-like
TCONS_00024651	-0.369081	0.663771	1.00649	BGIOSGA015223	XP_004955594.1FRIGIDA-like protein 4b
TCONS_00021415	-0.369068	-1.05222	-0.843326	BGIOSGA023641	XP_006656520.1PREDICTED: uncharacterized protein LOC102705442
TCONS_00012416	-0.368974	0.303911	0.132287	BGIOSGA028448	XP_015698980.1 probable LRR receptor-like serine/threonine-protein kinase At1g74360
TCONS_00017097	-0.368943	0.402103	0.204847	BGIOSGA001860	XP_006654022.1 aspartic proteinase
TCONS_00025916	-0.368916	0.0437658	0.630424	BGIOSGA013649	XP_003573503.1uncharacterized protein At1g01500
TCONS_00030417	-0.368169	-1.246	-0.0679465	BGIOSGA030000	XP_006660882.1 polygalacturonate 4-alpha-galacturonosyltransferase-like
TCONS_00024372	-0.368074	0.618953	-1.77867	BGIOSGA017809	XP_006658150.1 monoacylglycerol lipase abhd6-B
TCONS_00025710	-0.368016	-0.66175	-0.591584	BGIOSGA030559	XP_006658132.1 B3 domain-containing protein Os07g0679700
TCONS_00001629	-0.367962	1.8889	-2.33149	BGIOSGA030887	XP_002450636.1aspartic proteinase nepenthesin-1
TCONS_00023216	-0.367911	0.659453	0.251262	BGIOSGA009645	XP_015695090.1 60S ribosomal protein L4-1-like
TCONS_00011236	-0.367628	0.536674	-0.530294	BGIOSGA037214	XP_004981251.1heat shock cognate 70 kDa protein 2
TCONS_00029383	-0.367612	-0.785908	-0.485364	BGIOSGA035935	XP_006661467.1 cytochrome P450 71A1-like
TCONS_00015197	-0.367592	-4.05408	-1.65433	BGIOSGA008765	XP_006653047.1 protein QUIRKY
TCONS_00009695	-0.367507	0.398899	-0.788364	BGIOSGA004748	XP_006649752.1 V-type proton ATPase subunit a3-like
TCONS_00005329	-0.36744	0.43927	0.595094	BGIOSGA007437	XP_015689562.1 Golgi SNAP receptor complex member 1-2
TCONS_00029305	-0.367108	-0.581131	-0.375276	BGIOSGA004045	XP_006660813.1 uridine kinase-like protein 1, chloroplastic
TCONS_00022988	-0.367061	0.184986	0.191978	BGIOSGA009789	XP_015695065.1PREDICTED: uncharacterized protein LOC102713103 isoform X2
TCONS_00024413	-0.367018	-0.0104051	0.617267	BGIOSGA026451	XP_015694469.1PREDICTED: uncharacterized protein LOC102702674
TCONS_00005178	-0.36696	0.852018	0.898693	BGIOSGA006860	XP_015688059.1 pentatricopeptide repeat-containing protein At3g48250, chloroplastic
TCONS_00015416	-0.366831	0.01378	0.052223	BGIOSGA015957	XP_015691872.1 F-box protein At3g57580-like
TCONS_00006673	-0.36682	1.46851	0.297955	BGIOSGA017612	XP_003570132.1probable protein phosphatase 2C 26
TCONS_00035426	-0.366726	-2.14666	-0.790568	BGIOSGA015373	XP_006664257.1 phototropin-1A
TCONS_00028126	-0.366724	-1.24781	0.201504	BGIOSGA026865	XP_021310336.1F-box/FBD/LRR-repeat protein At1g13570
TCONS_00026708	-0.366681	-1.06436	-1.06806	BGIOSGA014468	NP_001183875.1uncharacterized LOC100275491
TCONS_00009817	-0.366446	1.26912	1.35736	BGIOSGA012370	XP_008654433.1uncharacterized protein LOC100275370
TCONS_00011450	-0.36636	-1.14436	-2.6519	BGIOSGA033295	XP_003558974.1uncharacterized protein LOC100824638 isoform X2
TCONS_00006787	-0.366338	-0.734102	-0.618896	BGIOSGA005701	XP_006647822.1 cellulose synthase-like protein E2
TCONS_00007069	-0.366305	-2.3792	0.0211737	BGIOSGA022956	XP_006649157.1 phosphate transporter PHO1-2-like
TCONS_00015038	-0.366075	0.109794	-0.058892	BGIOSGA027577	XP_010240509.1DNA-directed RNA polymerases IV and V subunit 2
TCONS_00002353	-0.365928	-0.696975	0.0590746	BGIOSGA004993	XP_006646570.2 GDP-L-galactose phosphorylase 2-like
TCONS_00033933	-0.365884	-3.45192	-0.0953643	BGIOSGA009297	XP_015697821.1 protein tas-like isoform X1
TCONS_00036603	-0.365353	0.513059	0.432916	BGIOSGA034578	XP_015698195.1 actin cytoskeleton-regulatory complex protein pan1-like
TCONS_00010625	-0.365178	0.173088	0.549952	BGIOSGA013247	XP_006650370.1 ferredoxin
TCONS_00002089	-0.364783	2.54105	-0.219402	BGIOSGA004716	XP_006644929.1PREDICTED: uncharacterized protein LOC102711204
TCONS_00029009	-0.364724	-1.86842	-1.29755	BGIOSGA021312	XP_015696343.1 putative CBL-interacting protein kinase 27
TCONS_00016061	-0.364567	-1.88831	-1.79838	BGIOSGA006159	XP_002447998.1heavy metal-associated isoprenylated plant protein 16

TCONS_00028342	-0.364447	-0.642424	0.188412	BGIOSGA026623	XP_015696217.1 glutamic acid-rich protein-like
TCONS_00012063	-0.364421	-0.399067	-0.495835	BGIOSGA034095	XP_015691076.1 phosphoinositide phosphatase SAC8 isoform X1
TCONS_00000212	-0.364066	-0.518109	0.222846	BGIOSGA002786	XP_015699211.1 geranylgeranyl transferase type-1 subunit beta isoform X1
TCONS_00015832	-0.364024	-3.28433	0.115754	BGIOSGA033850	XP_004975514.1heparan-alpha-glucosaminide N-acetyltransferase isoform X1
TCONS_00001973	-0.364016	-0.595204	-0.104044	BGIOSGA027987	XP_006644840.1 probable 3-hydroxybutyryl-CoA dehydrogenase
TCONS_00014334	-0.364003	-2.5474	-1.46926	BGIOSGA015005	XP_006652338.1 glutamate decarboxylase 4-like
TCONS_00025568	-0.363924	-0.537779	-0.0368989	BGIOSGA024150	XP_006658858.2 putative receptor-like protein kinase At4g00960
TCONS_00009653	-0.363473	0.0637489	-0.0104839	BGIOSGA004800	XP_006649702.1 probable NAD(P)H-dependent oxidoreductase 1
TCONS_00020148	-0.363109	-2.06518	-0.719932	BGIOSGA021863	XP_006656658.1 GDSL esterase/lipase At5g45910-like isoform X2
TCONS_00008817	-0.363071	-0.498881	-0.0601018	BGIOSGA005610	XP_006647907.1 random slug protein 5-like
TCONS_00014688	-0.363021	0.999808	0.256236	BGIOSGA016860	XP_015692112.1PREDICTED: uncharacterized protein LOC102719669 isoform X2
TCONS_00009170	-0.362859	1.12057	0.0354351	BGIOSGA008708	XP_010229321.1AP-2 complex subunit alpha-2 isoform X1
TCONS_00036883	-0.362851	-0.233492	0.472272	BGIOSGA028831	XP_015698245.1 la-related protein 6B-like
TCONS_00004895	-0.362755	-4.06489	-2.07953	BGIOSGA028714	XP_015690661.1 formin-like protein 1
TCONS_00000372	-0.362645	-3.53051	-2.42156	BGIOSGA021834	XP_014753811.1lachrymatory-factor synthase
TCONS_00016915	-0.362638	-0.51212	0.216766	BGIOSGA024321	XP_006653907.1 serine/threonine-protein kinase STY8-like
TCONS_00021645	-0.362538	-0.62646	-2.65845	#N/A	#N/A
TCONS_00022722	-0.362535	-1.06834	-0.441495	BGIOSGA020729	XP_006656339.1 endonuclease V
TCONS_00008985	-0.362287	-1.72361	-0.315542	BGIOSGA009292	XP_015689508.1 NADH dehydrogenase [ubiquinone] 1 alpha subcomplex subunit 9, mitochondrial isoform X2
TCONS_00033271	-0.362114	0.0351969	-0.719032	BGIOSGA035581	XP_010237767.1putative disease resistance RPP13-like protein 3
TCONS_00013479	-0.362032	-1.19387	1.30102	BGIOSGA003352	NP_001333705.1uncharacterized LOC103625736 precursor
TCONS_00014771	-0.36172	-0.431938	0.449557	BGIOSGA033298	XP_006652663.1 F-box protein PP2-A13-like
TCONS_00010593	-0.361705	0.181176	-0.108901	BGIOSGA013212	XP_006650342.2 pyruvate dehydrogenase E1 component subunit beta-4, chloroplastic-like
TCONS_00029311	-0.36161	0.356661	0.407213	BGIOSGA031054	XP_015696896.1 TITAN-like protein isoform X3
TCONS_00001572	-0.361575	-0.688884	-0.243035	BGIOSGA004204	XP_006644518.1 UPF0678 fatty acid-binding protein-like protein At1g79260
TCONS_00033044	-0.361527	1.0532	-0.101197	BGIOSGA037056	XP_006662762.2 60S ribosomal protein L3-like
TCONS_00013141	-0.361321	-0.51925	0.582421	BGIOSGA009845	XP_006650558.1 magnesium-dependent phosphatase 1-like
TCONS_00025640	-0.361308	0.41363	0.454685	BGIOSGA023786	XP_003562532.1uncharacterized protein LOC100829367
TCONS_00036715	-0.361238	-0.570278	0.654219	BGIOSGA036598	XP_015698409.1 TLD domain-containing protein 1
TCONS_00015054	-0.361207	-0.24685	0.362964	BGIOSGA009993	XP_006652911.1 putative chloride channel-like protein CLC-g
TCONS_00012531	-0.361029	-1.59106	-1.066	BGIOSGA018680	XP_006651528.1 interferon-related developmental regulator 2-like
TCONS_00013621	-0.360747	0.017255	-0.234635	BGIOSGA013477	XP_006652083.1 SWI/SNF complex subunit SWI3D-like isoform X2
TCONS_00004802	-0.360476	0.0842728	0.635174	BGIOSGA008212	XP_006645089.1 5'-3' exoribonuclease 3 isoform X1
TCONS_00000094	-0.360104	-0.660373	0.0369813	BGIOSGA004228	XP_006643710.1 protein-lysine N-methyltransferase mettl10-like
TCONS_00005873	-0.360032	-0.471806	-0.283933	BGIOSGA008005	XP_015688857.1 UV-stimulated scaffold protein A homolog
TCONS_00026942	-0.360008	-0.26396	0.373805	BGIOSGA028992	XP_021308234.1titin homolog
TCONS_00017774	-0.359707	-0.881597	-0.292843	BGIOSGA019839	XP_015693270.1 protein ABCI12, chloroplastic
TCONS_00006031	-0.359475	-0.263403	-0.286229	BGIOSGA008183	XP_006648628.1PREDICTED: uncharacterized protein LOC102707377
TCONS_00027214	-0.359368	1.01993	-0.223315	BGIOSGA012075	XP_006659031.1PREDICTED: uncharacterized protein LOC102709862
TCONS_00009228	-0.359349	0.0721994	0.676869	BGIOSGA013305	XP_006649308.1 DUF21 domain-containing protein At2g14520-like
TCONS_00011587	-0.359232	-1.85614	-2.0792	BGIOSGA020692	XP_015696882.1 sufE-like protein 1, chloroplastic/mitochondrial
TCONS_00003342	-0.359202	-0.929361	-1.48004	BGIOSGA010070	XP_006650401.1 CSC1-like protein At1g10090 isoform X3
TCONS_00016665	-0.359002	0.342247	0.497262	BGIOSGA014297	XP_015692247.1 histone-lysine N-methyltransferase ATXR2
TCONS_00030489	-0.358765	-0.920053	0.916948	BGIOSGA027713	XP_015696611.1 U11/U12 small nuclear ribonucleoprotein 31 kDa protein
TCONS_00026815	-0.358521	0.183061	0.074142	BGIOSGA002918	XP_015696216.1 nitronate monooxygenase
TCONS_00012665	-0.358504	-0.0840599	-0.371031	BGIOSGA022773	XP_015693346.1 60S ribosomal protein L15 isoform X3
TCONS_00016845	-0.358498	0.416352	0.581004	BGIOSGA018712	XP_006653006.1 zinc finger CCCH domain-containing protein 32
TCONS_00007701	-0.358466	-1.8168	0.328141	BGIOSGA013693	XP_002453610.1E3 ubiquitin-protein ligase ATL31
TCONS_00006981	-0.35839	0.0229834	-0.446514	BGIOSGA009163	XP_015689408.1 guanine nucleotide exchange factor subunit Rich-like
TCONS_00006820	-0.35835	-0.249609	0.69659	BGIOSGA008995	NP_001151430.1SET domain containing protein
TCONS_00017337	-0.357804	0.301278	0.577475	BGIOSGA003024	XP_012699764.1uncharacterized protein LOC101781173
TCONS_00033268	-0.357765	-0.593578	-0.0576052	BGIOSGA035091	XP_015697515.1 diphthamide biosynthesis protein 2
TCONS_00000894	-0.357555	-1.04509	-0.379832	BGIOSGA000168	XP_006644153.1 protein SRG1-like
TCONS_00037616	-0.357539	-0.341961	-0.598952	BGIOSGA009944	XP_015698637.1 serine/threonine-protein kinase prpf4B-like

transcriptome

TCONS_00000572	-0.357398	-0.209952	0.521929	BGIOSGA003153	XP_006645712.1 protein TRIGALACTOSYLDIACYLGLYCEROL 4, chloroplastic
TCONS_00020189	-0.357376	-0.301311	1.59981	BGIOSGA012581	XP_010227995.1ethylene-responsive transcription factor RAP2-1
TCONS_00002917	-0.35735	0.2627	0.0217996	BGIOSGA018733	XP_006643803.1 vegetative cell wall protein gp1-like
TCONS_00025965	-0.357182	-1.2337	-0.733796	BGIOSGA018909	XP_006659164.1 RNA polymerase sigma factor sigA
TCONS_00026176	-0.357137	-4.04568	-1.74477	BGIOSGA028249	XP_006659959.1 endo-1,3;1,4-beta-D-glucanase-like
TCONS_00025555	-0.357136	-1.85274	-0.940301	BGIOSGA005203	XP_015695249.1 probable inactive receptor kinase At5g10020
TCONS_00012275	-0.357003	-0.340795	0.0377972	BGIOSGA001033	XP_006651378.1 tubby-like F-box protein 6
TCONS_00027283	-0.356664	-0.0935983	0.508896	BGIOSGA027719	XP_006659082.1 transcription factor GTE9-like
TCONS_00036494	-0.356548	0.243147	-0.436778	BGIOSGA015087	XP_002442608.1uncharacterized protein LOC8065246 isoform X2
TCONS_00007611	-0.356497	-1.87788	-0.622339	BGIOSGA004437	XP_006647075.1 U-box domain-containing protein 52 isoform X1
TCONS_00025771	-0.35627	0.926681	-0.400904	BGIOSGA021893	XP_006659728.1 O-glucosyltransferase rumi-like
TCONS_00009811	-0.355227	-0.411438	0.155254	BGIOSGA000537	XP_015690699.1 EVI5-like protein isoform X2
TCONS_00012768	-0.354995	-0.252304	0.594153	BGIOSGA000727	XP_015690228.1 E3 ubiquitin protein ligase DRIP2
TCONS_00013773	-0.354788	-3.81305	-3.87915	BGIOSGA015539	XP_021319877.1cysteine-rich receptor-like protein kinase 15 isoform X1
TCONS_00028533	-0.354601	-0.123441	0.44903	BGIOSGA015795	XP_003576858.1F-box protein At1g47056
TCONS_00035428	-0.354552	0.424646	-0.757121	BGIOSGA036839	XP_006665005.1PREDICTED: uncharacterized protein LOC102708769 isoform X2
TCONS_00015903	-0.354506	0.151504	0.675784	BGIOSGA019450	XP_015692143.1 DExH-box ATP-dependent RNA helicase DExH7, chloroplastic-like isoform X1
TCONS_00005786	-0.354388	-0.826899	-0.630501	BGIOSGA000006	XP_004951289.1probable protein phosphatase 2C 13 isoform X1
TCONS_00026920	-0.354363	0.63744	-0.196688	BGIOSGA013500	XP_022683389.1G patch domain-containing protein 11
TCONS_00016828	-0.354363	0.400401	0.165882	BGIOSGA013934	XP_015692302.1 ubiquinol oxidase 4, chloroplastic/chromoplastic
TCONS_00000251	-0.354281	-1.13697	0.0179167	BGIOSGA002829	XP_006643773.1 importin subunit alpha-2
TCONS_00029308	-0.354237	-2.2443	-0.149407	BGIOSGA016610	XP_021302228.1putative F-box/FBD/LRR-repeat protein At4g03220 isoform X2
TCONS_00014997	-0.354165	-1.08528	0.828354	BGIOSGA011557	XP_006652863.1 peroxidase 1
TCONS_00006973	-0.353848	-0.733668	-0.978358	BGIOSGA021738	XP_015688782.1 phosphoinositide phosphatase SAC2-like isoform X1
TCONS_00009992	-0.353727	-0.660958	-0.695706	BGIOSGA012547	XP_015690797.1 guanine nucleotide exchange factor SPIKE 1 isoform X2
TCONS_00012132	-0.353677	0.780112	-0.099909	BGIOSGA010877	XP_006651314.2 FIP1[III]-like protein
TCONS_00013286	-0.353594	-0.966236	-0.329247	BGIOSGA024814	XP_008665555.1uncharacterized LOC103644136 isoform X1
TCONS_00008701	-0.353304	1.34653	0.398124	BGIOSGA016925	XP_015689433.1PREDICTED: uncharacterized protein LOC102711409
TCONS_00009301	-0.353124	-0.342427	1.09091	BGIOSGA011840	XP_006649281.2 UDP-galactose/UDP-glucose transporter 2-like isoform X1
TCONS_00031742	-0.352713	-0.825612	-0.290224	BGIOSGA022207	XP_006662143.2 U1 small nuclear ribonucleoprotein 70 kDa
TCONS_00004462	-0.352653	0.114453	0.384031	BGIOSGA035050	XP_015689984.1 serpin-Z1-like
TCONS_00013549	-0.352374	-0.966349	-0.702192	BGIOSGA009426	XP_015690748.1 putative ATPase N2B
TCONS_00031034	-0.352349	0.46891	0.160082	BGIOSGA032850	XP_003573851.1mediator of RNA polymerase II transcription subunit 15a
TCONS_00031358	-0.352302	-0.269675	-0.566226	BGIOSGA009136	XP_006661886.1 myosin-1-like
TCONS_00009077	-0.352285	-0.0668165	0.0376084	BGIOSGA026471	XP_006648156.1 ABC transporter F family member 3
TCONS_00009423	-0.351943	0.939086	-0.263381	BGIOSGA037417	XP_006649495.1 ylmG homolog protein 2, chloroplastic
TCONS_00019982	-0.351881	0.180253	-0.0859386	BGIOSGA035272	XP_004971196.16-phosphogluconate dehydrogenase, decarboxylating 1
TCONS_00032553	-0.351594	0.0391596	0.135911	BGIOSGA012289	XP_006661952.1 SH3 domain-containing protein 2-like
TCONS_00013397	-0.351488	-0.00969343	0.543421	BGIOSGA009587	XP_006650777.1 INO80 complex subunit B-like
TCONS_00018222	-0.351365	-0.0680815	0.651796	BGIOSGA020280	XP_006654720.1PREDICTED: uncharacterized protein At4g29660-like
TCONS_00027388	-0.351071	-2.67965	-3.46678	BGIOSGA033427	XP_022683193.1protein LSD1
TCONS_00035687	-0.350727	-0.756146	-0.898687	BGIOSGA037105	XP_006663889.1 proteasome assembly chaperone 4
TCONS_00002429	-0.350715	0.112018	-0.165079	BGIOSGA019930	XP_006645246.1 exocyst complex component EXO70B1-like
TCONS_00015092	-0.350346	0.0730256	0.131337	BGIOSGA006572	XP_006652940.1 plasma membrane ATPase
TCONS_00002997	-0.350314	-2.07546	-1.23081	BGIOSGA021040	XP_015695178.1 stem 28 kDa glycoprotein-like
TCONS_00032144	-0.350263	0.448422	-2.25092	BGIOSGA002488	XP_021306721.1shewanella-like protein phosphatase 1
TCONS_00006168	-0.349916	-1.03219	-0.148668	BGIOSGA006391	XP_015689019.1 ubiquitin thioesterase otubain-like
TCONS_00035026	-0.349773	0.337232	1.51471	BGIOSGA007551	XP_015698057.1 adagio-like protein 3
TCONS_00007365	-0.349551	-0.644817	-0.329439	BGIOSGA020630	XP_015688457.1 tyrosine-sulfated glycopeptide receptor 1-like
TCONS_00037346	-0.349383	-0.468412	-0.012163	BGIOSGA009435	XP_006664095.1 CTP synthase isoform X1
TCONS_00023705	-0.349264	1.18096	0.37996	BGIOSGA007697	XP_006658577.1 regulator of nonsense transcripts 1 homolog
TCONS_00029297	-0.349245	-3.97024	-2.39222	BGIOSGA016214	XP_021308070.1chloroplast envelope quinone oxidoreductase homolog
TCONS_00027854	-0.34917	-0.0199212	-0.0896498	BGIOSGA027151	XP_003573495.1general transcription and DNA repair factor IIH subunit TFB1-1
TCONS_00010867	-0.349058	0.0778598	0.535174	BGIOSGA013488	XP_006651764.1 FIP1[V]-like protein
TCONS_00028448	-0.348988	-0.286748	-1.04341	BGIOSGA031833	XP_015695695.1 protein indeterminate-domain 5, chloroplastic-like

TCONS_00020723	-0.348972	1.14329	-0.0871981	#N/A	#N/A
TCONS_00021998	-0.348913	-1.93165	-2.108	BGIOSGA021519	XP_006656852.2 probable peroxygenase 4
TCONS_00008181	-0.348767	0.0578145	0.611191	BGIOSGA033237	XP_015689638.1 ran-binding protein 10-like
TCONS_00008022	-0.348726	0.873842	0.0958533	BGIOSGA000263	XP_006647294.2 probable UDP-N-acetylglucosamine--peptide N-acetylglucosaminyltransferase SEC
TCONS_00004899	-0.348695	-0.0069112	-0.581874	BGIOSGA007515	XP_006645172.1PREDICTED: uncharacterized protein LOC102710074
TCONS_00010973	-0.348561	-2.64539	-6.15457	BGIOSGA015726	XP_015690070.1 senescence-specific cysteine protease SAG39-like
TCONS_00025463	-0.348431	-0.137438	-0.221685	BGIOSGA010657	XP_015694711.1 probable WRKY transcription factor 3
TCONS_00008808	-0.348295	-0.18087	0.0865715	BGIOSGA005622	XP_006647891.1 RINT1-like protein MAG2 isoform X1
TCONS_00025690	-0.348288	-0.38379	0.276726	BGIOSGA023733	XP_006658106.1 ribosome biogenesis protein NSA2 homolog
TCONS_00036026	-0.348248	-0.459748	0.336145	BGIOSGA016920	XP_006664555.2PREDICTED: uncharacterized protein LOC102701966
TCONS_00025086	-0.348159	-0.649785	-0.259448	BGIOSGA028394	XP_015694914.1 DNA-directed RNA polymerase III subunit RPC8
TCONS_00005758	-0.348066	-0.864848	-0.58579	BGIOSGA026801	XP_015689386.1 ubiquitin carboxyl-terminal hydrolase 15-like
TCONS_00016121	-0.347831	0.5975	-0.110725	BGIOSGA006120	XP_006652425.1 mitochondrial inner membrane protease ATP23
TCONS_00001422	-0.347578	-0.957938	-0.452615	BGIOSGA030262	XP_015696900.1PREDICTED: uncharacterized protein LOC102720903
TCONS_00034802	-0.347535	-0.0158524	0.763	BGIOSGA034482	XP_015694853.1 probable serine/threonine-protein kinase WNK3 isoform X3
TCONS_00014865	-0.347374	0.394761	0.0900558	BGIOSGA008871	NP_001150452.160S ribosomal protein L12
TCONS_00001249	-0.347325	0.754301	-1.22844	BGIOSGA038934	XP_006644328.1 aldehyde dehydrogenase family 2 member C4-like
TCONS_00013013	-0.347282	1.24755	0.809263	#N/A	#N/A
TCONS_00000956	-0.347229	-1.09997	0.555595	BGIOSGA018630	XP_004968778.1NPL4-like protein
TCONS_00036604	-0.347122	-3.10227	-3.19263	BGIOSGA037315	XP_015698574.1PREDICTED: uncharacterized protein LOC102710259
TCONS_00033321	-0.346751	-1.45942	0.665941	BGIOSGA014272	XP_006664858.2 uncharacterized HIT-like protein MT1300
TCONS_00007071	-0.346614	-1.1474	-0.128794	BGIOSGA009253	XP_006648084.1 ABC transporter I family member 19-like
TCONS_00010725	-0.346612	-0.318667	0.0438466	BGIOSGA006188	XP_006651699.2PREDICTED: uncharacterized protein LOC102721634
TCONS_00005504	-0.346459	-1.48019	-0.0942611	BGIOSGA007618	XP_015689217.1 urease accessory protein F
TCONS_00000421	-0.346441	-0.37909	-0.807534	BGIOSGA019363	XP_006643906.1 GDSL esterase/lipase At5g45910-like
TCONS_00036706	-0.346315	-2.12522	-2.6789	BGIOSGA016081	XP_004978707.1protein FANTASTIC FOUR 3 isoform X2
TCONS_00036882	-0.346087	-0.971589	-0.260062	BGIOSGA036438	XP_006663913.1 protein ENHANCED DOWNY MILDEW 2-like
TCONS_00020440	-0.346032	0.335496	0.256356	BGIOSGA025978	XP_006655965.1 dynamin-2A-like
TCONS_00010353	-0.345963	-1.11228	-0.180002	BGIOSGA007484	XP_006650224.1 restin homolog
TCONS_00030571	-0.345657	-0.895496	-0.519851	BGIOSGA004174	XP_008670030.1sn1-specific diacylglycerol lipase beta
TCONS_00028007	-0.345626	-1.65364	-0.55842	BGIOSGA029876	XP_006660585.1 mitochondrial pyruvate carrier 1-like
TCONS_00009066	-0.345624	0.430381	-0.378178	BGIOSGA005338	XP_006649182.1 50S ribosomal protein L9, chloroplastic-like
TCONS_00035700	-0.345604	-0.0216046	0.510639	BGIOSGA004649	XP_006663879.2PREDICTED: uncharacterized protein LOC102721786
TCONS_00034178	-0.345442	-0.248106	-0.990278	BGIOSGA008170	XP_006662678.1PREDICTED: uncharacterized protein LOC102700746
TCONS_00019046	-0.34544	0.510086	0.515087	BGIOSGA021611	XP_004962468.1RING finger and transmembrane domain-containing protein 2
TCONS_00032747	-0.345252	-0.760886	-0.754196	BGIOSGA031361	XP_006662090.1 aluminum-activated malate transporter 12
TCONS_00002442	-0.345214	-0.0330033	0.267395	BGIOSGA029386	XP_003564965.1transcription factor TCP20
TCONS_00022516	-0.345091	-0.244796	-0.152764	BGIOSGA007758	XP_006656206.1 serine-threonine kinase receptor-associated protein-like
TCONS_00014950	-0.345065	0.356567	0.149393	BGIOSGA017129	XP_006653772.1 imidazoleglycerol-phosphate dehydratase
TCONS_00015401	-0.345051	-1.61879	-0.349682	BGIOSGA024529	XP_006652114.1PREDICTED: uncharacterized protein KIAA0930 homolog
TCONS_00011306	-0.345034	-0.0568586	-0.397658	BGIOSGA015439	XP_008644344.1uncharacterized protein LOC103625729
TCONS_00010098	-0.344859	0.00466708	-0.909642	BGIOSGA028256	XP_006650096.1 villin-3-like
TCONS_00010288	-0.344753	0.392365	0.479972	BGIOSGA012851	XP_015691211.1PREDICTED: uncharacterized protein LOC102714907
TCONS_00036535	-0.344256	-0.366998	-0.988455	BGIOSGA024552	XP_004964776.1uncharacterized protein LOC101758371 isoform X3
TCONS_00014279	-0.344234	0.59295	-0.897317	BGIOSGA020041	XP_006652295.1PREDICTED: uncharacterized protein LOC102711611
TCONS_00016937	-0.344063	-0.300349	1.29491	BGIOSGA004901	XP_004959920.1probable magnesium transporter NIPA4
TCONS_00007236	-0.344043	-0.0596209	-0.778968	BGIOSGA011520	XP_006646781.1 catalase isozyme A-like
TCONS_00018654	-0.343996	-1.8348	-0.476179	BGIOSGA003468	XP_021311031.1peroxisomal fatty acid beta-oxidation multifunctional protein MFP2
TCONS_00021578	-0.343531	-0.0393765	-0.0284449	BGIOSGA014074	XP_006655773.1 U-box domain-containing protein 35
TCONS_00030744	-0.34336	-0.805269	-1.3207	BGIOSGA026423	XP_006662193.1 receptor-like protein kinase
TCONS_00024823	-0.34326	0.646171	-0.122126	BGIOSGA025444	XP_006657582.1 F-box/LRR-repeat protein At2g43260-like
TCONS_00029439	-0.343153	-0.299274	-0.303116	BGIOSGA008531	XP_006660922.2 serine/threonine-protein kinase STY46-like
TCONS_00027165	-0.343064	-0.0785295	0.0136594	BGIOSGA029211	XP_004974256.1uncharacterized protein LOC101763437
TCONS_00004614	-0.342877	1.64569	2.44171	BGIOSGA013553	XP_006644896.1 cysteine synthase-like

TCONS_00029164	-0.342809	-1.57316	-2.35926	BGIOSGA023458	XP_002460345.1uncharacterized protein LOC8083228
TCONS_00004965	-0.342626	0.460538	-0.0756828	BGIOSGA000257	XP_006645224.1 AP-4 complex subunit epsilon
TCONS_00016023	-0.342518	-0.861723	1.68339	BGIOSGA011168	XP_008673565.1lecithin-cholesterol acyltransferase-like 1
TCONS_00020187	-0.342356	-1.09445	-0.747958	BGIOSGA029575	XP_006655824.1 caffeoyl-CoA O-methyltransferase 1
TCONS_00016497	-0.342353	-0.251796	-0.202025	BGIOSGA014471	XP_006652704.1PREDICTED: uncharacterized protein LOC102717425
TCONS_00038020	-0.342184	2.91784	-0.802864	BGIOSGA037482	XP_004964100.1probable L-type lectin-domain containing receptor kinase S.7
TCONS_00006982	-0.342147	-0.261639	-0.629878	BGIOSGA037714	XP_021314671.1probable LIM domain-containing serine/threonine-protein kinase DDB_G0286997
TCONS_00010682	-0.342072	-1.94029	-0.317714	BGIOSGA025662	XP_006650403.1 growth-regulating factor 9
TCONS_00027146	-0.342034	-0.0275723	-0.0317314	BGIOSGA029190	XP_006659697.1 probable UDP-N-acetylglucosamine--peptide N-acetylglucosaminyltransferase SPINDLY
TCONS_00029296	-0.34195	-2.3435	-2.10726	BGIOSGA031057	XP_004957270.1quinone-oxidoreductase QR1, chloroplastic
TCONS_00011130	-0.341886	-1.43294	-0.176426	BGIOSGA013730	XP_015690876.1 rRNA-processing protein EFG1-like
TCONS_00002655	-0.34183	-0.113092	-0.710053	BGIOSGA002566	XP_015699139.1 snurportin-1
TCONS_00020513	-0.341692	-1.31158	-0.992391	BGIOSGA020760	XP_012701063.1zinc finger protein HD1 isoform X1
TCONS_00019177	-0.341642	0.722138	-0.126132	BGIOSGA033091	XP_003575421.1ribosomal RNA-processing protein 14-C
TCONS_00002679	-0.341638	0.657088	0.0914333	BGIOSGA018992	XP_006645435.1 paired amphipathic helix protein Sin3-like 4
TCONS_00011518	-0.341578	-0.547195	0.998472	BGIOSGA011510	XP_006649347.1 probable serine acetyltransferase 2
TCONS_00035870	-0.34153	-0.9531	-0.345777	BGIOSGA037244	XP_006663949.1 ultraviolet-B receptor UVR8 isoform X1
TCONS_00004881	-0.341477	-0.635391	-0.709332	BGIOSGA000338	NP_001336778.1uncharacterized LOC100194187
TCONS_00028336	-0.341388	-0.291045	-1.49524	BGIOSGA029499	XP_006659600.1 cytochrome b561 and DOMON domain-containing protein At4g12980-like
TCONS_00003643	-0.341356	-1.48108	-1.09531	BGIOSGA004206	XP_004973073.1probable purine permease 4
TCONS_00002990	-0.341263	-0.650738	0.22119	BGIOSGA004441	XP_006643858.1 NADP-dependent malic enzyme, chloroplastic
TCONS_00008872	-0.341172	-0.420558	0.0919567	BGIOSGA030888	XP_006647967.1 transmembrane emp24 domain-containing protein p24delta3-like
TCONS_00024100	-0.341065	-0.755902	0.569122	#N/A	#N/A
TCONS_00002159	-0.340718	-1.41788	-2.18688	BGIOSGA011032	XP_006645000.1 probable protein phosphatase 2C 9
TCONS_00023902	-0.340695	1.66942	0.81013	BGIOSGA006218	XP_015694683.1 zinc finger and BTB domain-containing protein 47-like
TCONS_00003660	-0.340644	0.701309	0.32715	BGIOSGA001544	XP_015692902.1PREDICTED: uncharacterized protein LOC102714046
TCONS_00015598	-0.340068	-0.533353	0.66072	BGIOSGA017458	XP_006653260.1PREDICTED: uncharacterized protein LOC102714286, partial
TCONS_00021841	-0.339734	-0.363286	-0.353065	BGIOSGA021676	NP_001152650.1acylaminoacyl-peptidase 1
TCONS_00025748	-0.339658	-0.0204955	0.0369985	BGIOSGA028870	XP_015695134.1PREDICTED: uncharacterized protein LOC102715960
TCONS_00028190	-0.339456	-0.876953	0.251284	BGIOSGA029195	XP_006660221.1 protein DMR6-LIKE OXYGENASE 2-like
TCONS_00037011	-0.339411	-1.13034	-3.29816	#N/A	#N/A
TCONS_00016107	-0.339375	-1.25869	-0.200342	BGIOSGA005687	NP_001152152.1F-box domain containing protein
TCONS_00002111	-0.339083	-1.16615	-0.816256	BGIOSGA008829	XP_008654305.1uncharacterized LOC100192661 isoform X1
TCONS_00005099	-0.339022	-0.572997	-0.0923812	BGIOSGA040355	XP_002459105.1proline synthase co-transcribed bacterial homolog protein isoform X1
TCONS_00024450	-0.338971	0.764312	0.401973	BGIOSGA021736	XP_006655879.1 nucleolar GTP-binding protein 1-like
TCONS_00024443	-0.338883	-1.71591	0.400938	BGIOSGA024962	XP_006657398.1 GTPase activating protein 1-like
TCONS_00030959	-0.338653	-0.97077	-0.713734	BGIOSGA038425	XP_002465702.1uncharacterized protein LOC8054253
TCONS_00034310	-0.338501	-5.02486	-2.45513	BGIOSGA018646	XP_006663216.1 IQ domain-containing protein IQM3-like
TCONS_00003161	-0.338464	-1.12457	-0.453888	BGIOSGA021718	XP_002466320.125.3 kDa vesicle transport protein
TCONS_00026804	-0.338291	0.197796	-0.310193	BGIOSGA028866	XP_006660225.1PREDICTED: uncharacterized protein LOC102702682
TCONS_00017807	-0.338112	-0.325703	1.12762	BGIOSGA019866	XP_006654401.1PREDICTED: uncharacterized protein LOC102704805 isoform X1
TCONS_00003766	-0.338022	1.53859	0.117796	BGIOSGA017445	XP_006644279.1 B2 protein
TCONS_00032390	-0.337946	-0.270672	-0.142606	BGIOSGA023856	XP_015697476.1PREDICTED: uncharacterized protein LOC102716070
TCONS_00006488	-0.337924	-0.483291	-1.44409	BGIOSGA036578	XP_006647545.1 putative cyclic nucleotide-gated ion channel 15
TCONS_00032647	-0.337823	-0.0230371	-0.204517	BGIOSGA034929	XP_002464991.1short-chain dehydrogenase TIC 32, chloroplastic
TCONS_00013462	-0.337707	-0.865735	0.542947	BGIOSGA025448	XP_006650833.1 transmembrane protein 184B
TCONS_00003741	-0.337609	-0.167549	-0.370663	BGIOSGA001467	XP_006645973.2 protein kinase and PP2C-like domain-containing protein
TCONS_00025670	-0.337514	0.239607	0.411385	BGIOSGA023753	XP_015694995.1 protein NETWORKED 3A-like
TCONS_00024983	-0.337498	-0.261622	1.26572	BGIOSGA018725	XP_006654096.1 phospholipase D alpha 1-like
TCONS_00021243	-0.337463	-0.668778	0.391233	BGIOSGA035713	XP_006657253.1 heat shock 70 kDa protein 16
TCONS_00010909	-0.337345	0.00295355	-0.468703	BGIOSGA013522	XP_006651798.1 vacuolar protein sorting-associated protein 51 homolog
TCONS_00011622	-0.337152	0.945154	0.44818	BGIOSGA011388	XP_006651069.2PREDICTED: uncharacterized protein LOC102718817, partial

TCONS_00014633	-0.337006	-1.50088	-0.312765	BGIOSGA016790	XP_006652538.2 probable sphingolipid transporter spinster homolog 2
TCONS_00020638	-0.336995	-1.60904	-0.136188	BGIOSGA021352	XP_010233889.1jasmonate O-methyltransferase
TCONS_00035649	-0.336831	1.11974	-0.137087	BGIOSGA022937	XP_006664379.2 putative CCA tRNA nucleotidyltransferase 2
TCONS_00005589	-0.336623	-0.0177587	-0.213856	BGIOSGA029411	XP_006647015.1 kynurenine formamidase-like
TCONS_00009527	-0.336512	-1.5745	-1.04374	BGIOSGA012075	XP_015689726.1 membrane-associated progesterone-binding protein 4 isoform X1
TCONS_00036237	-0.336472	-0.0153695	-0.405966	BGIOSGA019427	XP_015698772.1 dual specificity protein kinase shkD
TCONS_00014247	-0.33645	0.242628	0.0620548	BGIOSGA016784	XP_015691452.1 exocyst complex component SEC5A-like
TCONS_00007347	-0.336307	-0.931839	-0.137894	BGIOSGA026597	XP_004971131.1uncharacterized protein LOC101786617
TCONS_00009656	-0.336296	-1.68624	-1.54219	BGIOSGA033828	XP_006651205.1 purple acid phosphatase 4-like
TCONS_00011370	-0.33629	-0.683934	0.557207	BGIOSGA018032	XP_015690320.1 gibberellin 20 oxidase 1-like
TCONS_00014823	-0.335888	-0.290469	-0.0875805	BGIOSGA013791	XP_004976604.1calcium-dependent protein kinase 13
TCONS_00010659	-0.335668	-0.588437	-0.287019	BGIOSGA033598	XP_021321955.1disease resistance protein RPM1
TCONS_00017021	-0.335632	-0.280154	0.239469	BGIOSGA019068	XP_021313506.1protein SENSITIVITY TO RED LIGHT REDUCED 1
TCONS_00009163	-0.335136	0.808744	0.97003	BGIOSGA011690	XP_006649251.1 THO complex subunit 1
TCONS_00026833	-0.335064	-5.4508	-0.239995	BGIOSGA029754	XP_006659524.1 DNA-3-methyladenine glycosylase isoform X2
TCONS_00028706	-0.334779	-0.410199	-0.248593	BGIOSGA030467	XP_015696242.1PREDICTED: uncharacterized protein LOC102702035
TCONS_00032393	-0.334697	0.251687	0.231746	BGIOSGA011818	XP_010940715.1 60S ribosomal protein L23
TCONS_00013944	-0.334455	1.26708	-1.14276	BGIOSGA010394	XP_006653261.2 wall-associated receptor kinase 5-like
TCONS_00019913	-0.334343	-0.102687	-0.335163	BGIOSGA027556	XP_015693012.1 chromatin modification-related protein EAF1 B-like isoform X1
TCONS_00005829	-0.334156	0.513394	0.45285	BGIOSGA003468	XP_006647155.1 peroxisomal fatty acid beta-oxidation multifunctional protein
TCONS_00036144	-0.33387	-2.79991	-1.28461	BGIOSGA018537	XP_006664070.1 glutathione synthetase, chloroplastic-like
TCONS_00005474	-0.333861	-1.01749	-0.0856576	BGIOSGA020662	XP_015688777.1 TATA-binding protein-associated factor BTAF1 isoform X1
TCONS_00017249	-0.333752	-0.127696	-1.2886	BGIOSGA002908	XP_006655061.1 FACT complex subunit SSRP1-B
TCONS_00023258	-0.333751	-0.862377	-1.20635	BGIOSGA025299	XP_006657521.1 protein CHUP1, chloroplastic-like
TCONS_00019150	-0.333726	0.268017	-0.482509	BGIOSGA004761	XP_006654306.2 UDP-glucuronic acid decarboxylase 2
TCONS_00015837	-0.333425	-0.790635	0.0998225	BGIOSGA016337	XP_015692185.1PREDICTED: uncharacterized protein LOC102720039
TCONS_00028139	-0.333364	-0.275938	-1.11939	BGIOSGA011585	XP_006659485.1 glutamate decarboxylase-like
TCONS_00001638	-0.333299	-4.03532	-3.41118	BGIOSGA016997	XP_006644571.1 bidirectional sugar transporter SWEET2b
TCONS_00013538	-0.333281	-0.235202	-0.837251	BGIOSGA009445	XP_003562360.1conserved oligomeric Golgi complex subunit 2
TCONS_00019400	-0.333089	0.0497707	-0.448297	BGIOSGA034864	XP_015693170.1 pleckstrin homology domain-containing protein 1-like
TCONS_00024466	-0.332835	-0.316913	-0.00383215	BGIOSGA024938	XP_006658223.1 suppressor of RPS4-RLD 1
TCONS_00000205	-0.332599	0.371592	0.162656	BGIOSGA002777	XP_015697918.1 E3 ubiquitin-protein ligase PRT6 isoform X1
TCONS_00027688	-0.332553	-2.34004	-2.14425	BGIOSGA027318	XP_006659265.1PREDICTED: uncharacterized protein LOC102710719
TCONS_00011916	-0.332362	-1.39019	-0.446826	BGIOSGA013353	XP_015691178.1 phosphomevalonate kinase
TCONS_00025683	-0.332168	-0.899908	0.314207	BGIOSGA023740	XP_006658091.1 PRKR-interacting protein 1 homolog
TCONS_00009222	-0.332146	0.790858	-0.0810499	BGIOSGA011137	XP_006649303.2 probable receptor-like protein kinase At5g24010
TCONS_00022117	-0.332077	0.293729	-0.246318	BGIOSGA023246	XP_002461133.1protein transport protein SFT2
TCONS_00026025	-0.332072	0.0609972	-0.129879	BGIOSGA014296	XP_006659184.1 helicase-like transcription factor CHR28
TCONS_00009515	-0.332066	-0.134169	0.473845	BGIOSGA012063	XP_006653258.1 elongator complex protein 5-like
TCONS_00037298	-0.33171	-0.402728	-0.0696998	BGIOSGA030216	XP_006664075.1 DNA-directed RNA polymerase I subunit rpa43-like isoform X1
TCONS_00013441	-0.331675	-1.47435	-0.454655	BGIOSGA009543	XP_006651969.1 D-xylose-proton symporter-like 3, chloroplastic
TCONS_00016344	-0.331572	-1.3758	-0.679384	BGIOSGA014623	XP_021307180.1GATA transcription factor 26
TCONS_00001368	-0.331479	0.66737	0.769258	BGIOSGA003998	XP_003569364.1uncharacterized protein LOC100828895 isoform X2
TCONS_00023159	-0.331418	-0.53744	0.654685	BGIOSGA007546	XP_006658297.1 serine/threonine-protein kinase RIO1-like
TCONS_00018375	-0.331313	-0.265727	-0.996693	BGIOSGA032818	XP_008656066.1zinc-finger homeodomain protein 6
TCONS_00007260	-0.331278	-0.165956	0.347394	BGIOSGA007222	XP_006646805.1 histone deacetylase complex subunit SAP18
TCONS_00017719	-0.331249	0.371643	0.352983	BGIOSGA003238	XP_003580147.1hydroxyproline O-arabinosyltransferase 3
TCONS_00032840	-0.330857	-0.0032405	-0.735602	BGIOSGA036729	XP_015698025.1PREDICTED: uncharacterized protein LOC102711575 isoform X1
TCONS_00025197	-0.330774	-0.461195	0.0715494	BGIOSGA002329	XP_006657765.1 DEAD-box ATP-dependent RNA helicase 32
TCONS_00028983	-0.330705	-0.588394	-0.0440995	BGIOSGA009957	NP_001145912.1putative RING zinc finger domain superfamily protein
TCONS_00032711	-0.330665	-0.39135	-1.08719	BGIOSGA003670	XP_006662604.2 transcription factor DIVARICATA-like
TCONS_00031603	-0.330472	-1.4043	-0.735762	#N/A	#N/A
TCONS_00020556	-0.330381	-0.171284	-1.88902	BGIOSGA008065	XP_006656899.1PREDICTED: uncharacterized protein LOC102714472

TCONS_00019700	-0.330302	1.03354	0.77204	BGIOSGA022304	XP_006651417.2PREDICTED: uncharacterized protein LOC102712794
TCONS_00032735	-0.330085	-2.14175	-0.736729	BGIOSGA031371	XP_015697216.1 VAN3-binding protein-like isoform X2
TCONS_00002145	-0.330045	-0.00794624	-0.480491	BGIOSGA020798	XP_006646471.1 epsin-2-like
TCONS_00006770	-0.330045	-1.1219	0.232795	BGIOSGA021371	XP_015688717.1 protein-tyrosine-phosphatase IBR5-like
TCONS_00003596	-0.330005	0.0989674	-0.068048	BGIOSGA001609	XP_006644186.1 cyclin-L1-1
TCONS_00030609	-0.329846	1.55839	-1.32229	BGIOSGA032433	XP_002467392.1probable NAD(P)H-dependent oxidoreductase 1
TCONS_00016938	-0.32976	-0.939547	-0.509747	BGIOSGA014026	XP_015691338.1 acyl-coenzyme A thioesterase 13-like
TCONS_00031773	-0.328807	-0.557982	-0.276636	BGIOSGA019510	XP_006650285.1PREDICTED: uncharacterized protein LOC102700605
TCONS_00026448	-0.328772	-0.942369	0.846158	BGIOSGA040512	XP_004973271.1bZIP transcription factor 11 isoform X1
TCONS_00032908	-0.328591	-0.172303	-0.176383	BGIOSGA009356	XP_015689584.1 heterogeneous nuclear ribonucleoprotein H3 isoform X2
TCONS_00018282	-0.328584	-6.17204	-1.83182	BGIOSGA016684	XP_006654762.1 cytochrome b561 and DOMON domain-containing protein At3g61750-like
TCONS_00006487	-0.328432	-0.852365	-0.337782	BGIOSGA014705	XP_006647543.1 phenylalanine ammonia-lyase-like
TCONS_00036818	-0.328342	-3.72891	-1.74107	BGIOSGA025174	XP_006663875.2 peroxidase 43
TCONS_00029400	-0.327703	0.89477	0.672376	BGIOSGA003811	XP_006661480.1 E3 ubiquitin ligase BIG BROTHER-related-like
TCONS_00017721	-0.327642	1.47966	0.372664	BGIOSGA001350	XP_004954076.1UDP-D-xylose:L-fucose alpha-1,3-D-xylosyltransferase MGP4
TCONS_00006664	-0.327278	-3.19771	-1.78419	BGIOSGA034079	XP_021301678.1serine carboxypeptidase-like 2
TCONS_00014475	-0.327046	0.208052	0.75821	BGIOSGA016635	XP_006653544.1 trihelix transcription factor GT-1-like
TCONS_00031303	-0.32692	-3.14574	-0.744603	BGIOSGA029906	XP_004982889.13-ketoacyl-CoA synthase 12
TCONS_00008210	-0.326815	-0.315802	-1.59286	BGIOSGA010378	XP_006647402.1 beta-1,3-galactosyltransferase 7-like
TCONS_00027430	-0.326715	-0.373591	-0.745514	BGIOSGA033795	XP_004979650.1putative disease resistance protein At1g50180
TCONS_00011768	-0.326703	-1.14435	-1.5127	BGIOSGA014476	XP_006649604.1 probable protein S-acyltransferase 22
TCONS_00017911	-0.326631	-0.76936	-0.221495	BGIOSGA012284	XP_003568339.1coronatine-insensitive protein homolog 1b isoform X2
TCONS_00006627	-0.326149	-0.694751	0.269504	BGIOSGA008792	XP_006647672.1 DNA-directed RNA polymerase III subunit RPC10-like
TCONS_00018641	-0.326045	-0.548585	-0.550371	BGIOSGA015185	XP_006654054.1 transport inhibitor response 1-like protein Os05g0150500
TCONS_00019575	-0.325971	-1.13691	-0.814992	BGIOSGA001299	XP_006654600.1 traB domain-containing protein-like
TCONS_00037359	-0.325969	0.428197	-0.493302	BGIOSGA033678	XP_006664098.1 GTP-binding protein SAR1A
TCONS_00005587	-0.325933	-1.56202	-1.52465	BGIOSGA034023	XP_004951642.1premnaspirodiene oxygenase
TCONS_00031254	-0.325804	0.50026	0.633543	BGIOSGA033072	XP_006662411.1 protein fluG
TCONS_00007184	-0.325783	0.36291	-1.39694	BGIOSGA007314	XP_006646754.1 glycerate dehydrogenase
TCONS_00006410	-0.32568	-1.056	0.262883	BGIOSGA008572	XP_003572693.1transcription termination factor MTERF5, chloroplastic
TCONS_00011196	-0.32568	-0.19264	0.0879617	BGIOSGA013798	XP_015690121.1 magnesium-chelatase subunit ChlD, chloroplastic
TCONS_00037100	-0.325534	0.0489455	0.034375	BGIOSGA011620	XP_015698706.1PREDICTED: uncharacterized protein LOC102710931 isoform X1
TCONS_00012237	-0.3252	0.173419	1.11065	BGIOSGA026140	XP_004984440.1sucrose synthase 4
TCONS_00019220	-0.325067	-1.2495	0.775413	BGIOSGA009124	XP_006647980.1 N-alpha-acetyltransferase 40
TCONS_00010763	-0.325065	0.713848	0.902888	BGIOSGA013388	XP_006651725.1 pescadillo homolog
TCONS_00000904	-0.32484	1.25763	-0.0419256	BGIOSGA003490	XP_006644159.1 probable phosphoinositide phosphatase SAC9
TCONS_00027194	-0.324811	-0.825609	0.359154	BGIOSGA028914	XP_002444863.1BTB/POZ domain-containing protein At1g21780
TCONS_00034994	-0.324789	-0.00546481	-0.493532	BGIOSGA033859	XP_015697680.1 glutamyl-tRNA(Gln) amidotransferase subunit A
TCONS_00030272	-0.324431	0.665419	1.28527	BGIOSGA029533	XP_015696871.1 uncharacterized transporter YBR287W-like isoform X2
TCONS_00018466	-0.324404	-0.681985	0.24949	BGIOSGA008411	XP_015692445.1 uncharacterized zinc finger protein At4g06634-like
TCONS_00014129	-0.32403	-0.665167	-0.857251	BGIOSGA007382	XP_003579626.1probable metal-nicotianamine transporter YSL6
TCONS_00018478	-0.32397	1.14006	0.570042	BGIOSGA038052	XP_006653961.2 pumilio homolog 1-like
TCONS_00008511	-0.323897	-0.664721	-0.0176565	BGIOSGA010460	XP_006647621.1 transcription initiation factor TFIID subunit 4b-like
TCONS_00018462	-0.323866	-1.31963	-2.67209	BGIOSGA008585	XP_006653945.1 methionine S-methyltransferase-like
TCONS_00021650	-0.323807	-1.79602	-0.83475	BGIOSGA035432	XP_006655813.1 disease resistance protein RPM1-like
TCONS_00002276	-0.32376	0.334428	0.64893	BGIOSGA004914	XP_006645112.1 PHD finger protein At1g33420
TCONS_00000095	-0.323698	-0.0321119	0.0551874	BGIOSGA027971	XP_002457471.1beta-glucuronosyltransferase GlcAT14A
TCONS_00021277	-0.323677	-0.155475	0.290335	BGIOSGA023502	XP_006656405.1 RNA polymerase I-specific transcription initiation factor RRN3 isoform X1
TCONS_00033008	-0.323507	-0.926428	-1.02752	BGIOSGA016384	XP_006663233.1 digalactosyldiacylglycerol synthase 1, chloroplastic-like
TCONS_00018444	-0.323502	-0.775309	-0.620963	BGIOSGA016403	XP_015693117.1 proline-rich receptor-like protein kinase PERK8
TCONS_00034335	-0.323476	-1.90045	0.0139688	BGIOSGA031769	XP_006663228.1PREDICTED: uncharacterized protein LOC102712306
TCONS_00033082	-0.323396	1.12762	0.347702	BGIOSGA018263	XP_006662781.2 transmembrane 9 superfamily member 1
TCONS_00026444	-0.323395	0.0615951	-1.72307	BGIOSGA030103	XP_006661098.1 UPF0481 protein At3g47200-like
TCONS_00009985	-0.323351	-0.919919	-0.536482	BGIOSGA030474	XP_006650017.1 myb family transcription factor APL-like

TCONS_00010902	-0.322889	0.413457	0.156338	BGIOSGA013517	XP_003560086.1WD repeat-containing protein PCN
TCONS_00003548	-0.322868	-1.17704	-1.64265	BGIOSGA001659	XP_006644167.1 acyltransferase-like protein At1g54570, chloroplastic isoform X2
TCONS_00035974	-0.322762	-0.36695	-0.398331	BGIOSGA036257	XP_010276527.1 FACT complex subunit SPT16-like
TCONS_00018157	-0.32273	0.528148	0.211464	BGIOSGA017675	XP_006654666.2 hexokinase-5
TCONS_00018453	-0.322556	-0.462424	-0.433265	BGIOSGA008067	XP_021303011.1protein MEI2-like 3 isoform X2
TCONS_00028384	-0.322528	0.663175	-0.552323	BGIOSGA033343	XP_006659635.1 NAC domain-containing protein 83-like
TCONS_00017509	-0.322469	-0.662639	-0.675181	BGIOSGA019583	XP_015692927.1PREDICTED: uncharacterized protein LOC102707030 isoform X2
TCONS_00028020	-0.322335	-0.924216	-0.393547	BGIOSGA026971	XP_006659399.2 RNA-binding protein 25 isoform X3
TCONS_00030391	-0.322274	-0.722311	-0.436118	BGIOSGA026539	XP_006660864.1 plant UBX domain-containing protein 8-like
TCONS_00012133	-0.322239	0.487776	0.444776	BGIOSGA015175	NP_001141020.1uncharacterized LOC100273099 precursor
TCONS_00018261	-0.321944	-1.48821	-0.800605	BGIOSGA023202	XP_006655556.1 probable E3 ubiquitin-protein ligase HIP1
TCONS_00004778	-0.321864	-0.716292	-0.206352	BGIOSGA032853	XP_006645072.1 putative UDP-rhamnose:rhamnosyltransferase 1
TCONS_00021345	-0.321844	-0.818598	-1.78227	BGIOSGA012582	XP_006656463.1 protein NRT1/ PTR FAMILY 8.3-like isoform X1
TCONS_00003669	-0.321809	0.396098	0.109889	BGIOSGA001538	XP_004967459.1trafficking protein particle complex subunit 1
TCONS_00010992	-0.321773	-0.893682	-0.884284	BGIOSGA013595	XP_006650620.1PREDICTED: uncharacterized protein LOC102704320
TCONS_00002133	-0.321406	-0.751008	-0.771448	BGIOSGA025786	XP_006644976.2 UDP-glucuronic acid decarboxylase 1-like
TCONS_00002247	-0.321322	-0.321045	-0.172927	BGIOSGA017925	XP_003564717.1ammonium transporter 3 member 1
TCONS_00008126	-0.321315	0.456779	0.0677385	BGIOSGA007344	XP_004952654.140S ribosomal protein S14
TCONS_00014565	-0.321262	0.194894	0.765523	BGIOSGA031186	XP_015691516.1 2,3-dimethylmalate lyase-like
TCONS_00039715	-0.321152	-0.121729	-2.56421	BGIOSGA025089	XP_004968783.1pathogenesis-related protein PRMS
TCONS_00017425	-0.321135	-2.3729	-1.27638	BGIOSGA028167	XP_006654184.1 probable LRR receptor-like serine/threonine-protein kinase At1g56140 isoform X2
TCONS_00001963	-0.321112	0.403823	-1.63274	BGIOSGA004598	XP_006646403.1 probable nucleoredoxin 2
TCONS_00019038	-0.32088	0.0494192	-0.503532	BGIOSGA026402	XP_006655186.2 mediator of RNA polymerase II transcription subunit 33A-like
TCONS_00004931	-0.320705	0.749699	0.820518	BGIOSGA000070	XP_006646587.1 E3 ubiquitin-protein ligase RGLG2-like
TCONS_00030411	-0.320545	-1.17295	-0.46809	BGIOSGA004895	XP_015696433.1PREDICTED: uncharacterized protein LOC102710330
TCONS_00002475	-0.320485	0.182167	0.231175	BGIOSGA005119	XP_006645287.1 homeobox protein LUMINIDEPENDENS
TCONS_00033949	-0.320425	-0.184667	-0.567457	BGIOSGA035543	XP_015697734.1 disease resistance protein RPM1-like
TCONS_00009103	-0.320358	-1.76174	-0.536206	BGIOSGA007293	XP_015689185.1 RING-H2 finger protein ATL80-like
TCONS_00036920	-0.320321	-0.924705	-0.797025	BGIOSGA036405	XP_006663925.1 probable GPI-anchored adhesin-like protein PGA55
TCONS_00025116	-0.32028	-0.249003	0.564466	BGIOSGA024286	XP_006657712.1 ranBP2-type zinc finger protein At1g67325-like
TCONS_00019821	-0.320266	0.560245	-0.593508	BGIOSGA017534	XP_006654813.1 protein SUPPRESSOR OF PHYA-105 1-like isoform X1
TCONS_00032497	-0.320217	-0.083645	0.104455	BGIOSGA031620	XP_015697467.1 nucleolar complex protein 2 homolog
TCONS_00028243	-0.320115	-1.0259	-0.97045	BGIOSGA030918	XP_015696187.1 tricin synthase 2-like
TCONS_00017112	-0.319997	-0.243645	0.0297998	BGIOSGA016586	XP_015692439.1 phosphoglycerate mutase-like protein AT74H
TCONS_00005502	-0.319618	0.268934	0.240093	BGIOSGA005497	XP_008645280.1auxin-responsive protein SAUR71
TCONS_00012913	-0.319513	-1.00773	0.554601	#N/A	#N/A
TCONS_00029494	-0.319467	0.193246	0.00144237	BGIOSGA023378	XP_007011925.1 transmembrane 9 superfamily member 10
TCONS_00015538	-0.319463	-0.168483	-0.624166	BGIOSGA026459	XP_014751494.1probable carbohydrate esterase At4g34215
TCONS_00001925	-0.319147	-0.557549	-0.41433	BGIOSGA024057	XP_006644800.2 zinc-metallopeptidase, peroxisomal-like
TCONS_00020590	-0.31899	1.77845	0.909116	BGIOSGA022774	XP_006656017.1 protein HOMOLOG OF MAMMALIAN LYST-INTERACTING PROTEIN 5
TCONS_00013029	-0.318757	0.378498	-0.468635	BGIOSGA034510	XP_006650470.1 phosphatidylinositol 4-phosphate 5-kinase 1
TCONS_00006939	-0.318741	-0.26936	-0.138463	BGIOSGA015003	XP_015689357.1PREDICTED: uncharacterized protein LOC102716577
TCONS_00009488	-0.318478	-0.844003	-0.742009	BGIOSGA018015	XP_002468345.1mitochondrial import inner membrane translocase subunit TIM22-3
TCONS_00020886	-0.318403	0.408773	0.49499	BGIOSGA011537	XP_015694407.1 K(+) efflux antiporter 4-like
TCONS_00036941	-0.31836	-0.144957	-0.241306	BGIOSGA021765	XP_004960778.1serine carboxypeptidase 1
TCONS_00004638	-0.318178	0.0818461	-0.228332	BGIOSGA000579	XP_006644920.1 probable receptor-like protein kinase At3g55450
TCONS_00019089	-0.318144	0.226139	-0.764473	#N/A	#N/A
TCONS_00028041	-0.318087	0.312332	-0.75743	BGIOSGA027837	XP_006659418.2 putative 1-phosphatidylinositol-3-phosphate 5-kinase FAB1C
TCONS_00015954	-0.318026	0.643757	0.187738	BGIOSGA028422	XP_006652320.1 chloride channel protein CLC-c-like
TCONS_00009078	-0.318	-0.412998	-0.670796	BGIOSGA009330	XP_006648158.1 plant intracellular Ras-group-related LRR protein 6
TCONS_00007035	-0.317708	0.277268	0.0915022	BGIOSGA009220	XP_006648062.1PREDICTED: uncharacterized protein LOC102703655
TCONS_00017969	-0.317696	0.364264	0.476395	BGIOSGA033329	XP_006654528.1 ammonium transporter 2 member 1

TCONS_00022684	-0.317438	-0.957075	-0.125582	BGIOSGA028277	XP_004965684.1probable 4-coumarate--CoA ligase 4
TCONS_00003572	-0.317262	-0.723169	-0.566549	BGIOSGA003539	XP_006645900.2 glutathione S-transferase 4-like
TCONS_00010873	-0.317137	-0.668088	-0.15284	BGIOSGA013497	XP_014753213.1protein starmaker
TCONS_00004705	-0.317088	0.413492	-0.718587	BGIOSGA000513	XP_015699311.1 ribonuclease II, chloroplastic/mitochondrial
TCONS_00029165	-0.316536	-0.412258	-0.769367	BGIOSGA028844	XP_002460346.1protein transport protein SEC23 isoform X2
TCONS_00028926	-0.316448	-2.98541	-0.768749	BGIOSGA037744	XP_003578066.1uncharacterized protein LOC100821553
TCONS_00033154	-0.316422	1.05311	0.520196	BGIOSGA030303	XP_006662811.1 transcriptional corepressor SEUSS
TCONS_00001485	-0.316399	-0.400152	0.424673	BGIOSGA002951	XP_006644446.1 ketol-acid reductoisomerase, chloroplastic-like
TCONS_00024101	-0.31623	0.947422	-0.853791	BGIOSGA010559	XP_006657953.1 protein TIFY 10b-like
TCONS_00032752	-0.316194	-0.970982	-0.699439	BGIOSGA031357	XP_006662093.1 3-hydroxyisobutyryl-CoA hydrolase 1-like
TCONS_00004021	-0.316099	-0.694962	-0.626856	BGIOSGA001173	XP_006644393.1 transcription factor bHLH144-like
TCONS_00007718	-0.316049	0.714726	0.431444	BGIOSGA006772	XP_015689604.1PREDICTED: uncharacterized protein LOC102700308
TCONS_00003237	-0.315952	0.715193	-0.670313	BGIOSGA001976	XP_024315978.1peptidyl-prolyl cis-trans isomerase CYP21-3, mitochondrial-like
TCONS_00008231	-0.315725	-0.457757	-0.0719006	BGIOSGA014986	XP_006647407.1 ubiquitin carboxyl-terminal hydrolase 4-like
TCONS_00001110	-0.315576	0.291665	0.0763251	BGIOSGA002288	XP_004968951.1DEAD-box ATP-dependent RNA helicase 40 isoform X1
TCONS_00010388	-0.315288	0.389818	-1.1859	#N/A	#N/A
TCONS_00035840	-0.315072	-0.695983	-0.306951	BGIOSGA019404	XP_006662093.1 3-hydroxyisobutyryl-CoA hydrolase 1-like
TCONS_00001852	-0.315034	-0.52596	0.043458	BGIOSGA004487	XP_006646338.1 DNA polymerase eta
TCONS_00026128	-0.315005	-1.47924	-0.369477	BGIOSGA028204	XP_006665186.2PREDICTED: uncharacterized protein LOC102710555
TCONS_00023355	-0.314925	0.751784	-1.28755	BGIOSGA017101	XP_006651173.1 beta-hexosaminidase 2
TCONS_00031341	-0.314844	0.245926	-0.188411	#N/A	XP_015697032.1 protein EXECUTER 1, chloroplastic
TCONS_00020518	-0.314804	-1.58562	-2.04182	BGIOSGA031544	XP_006656000.1 sodium-coupled neutral amino acid transporter 1
TCONS_00029003	-0.31461	-1.08231	-0.119651	BGIOSGA028741	XP_006660627.1 probable CCR4-associated factor 1 homolog 7
TCONS_00023947	-0.314504	-1.70981	-1.03645	BGIOSGA025979	NP_001335846.1uncharacterized LOC100275698
TCONS_00037024	-0.314001	-1.30486	0.415303	BGIOSGA036348	XP_006663952.2 coilin
TCONS_00001775	-0.313931	1.43334	0.0111566	BGIOSGA024238	XP_003567088.1anthocyanidin 5,3-O-glucosyltransferase
TCONS_00033995	-0.313821	2.43321	0.487323	BGIOSGA019108	XP_024311164.1putative serine/threonine-protein kinase-like protein CCR3
TCONS_00030437	-0.313678	0.869077	3.47222	BGIOSGA029365	XP_006660898.1 ribonuclease 1-like
TCONS_00025792	-0.313624	-0.12956	-0.0348484	BGIOSGA008276	XP_006659037.2 phosphoinositide phosphatase SAC1 isoform X1
TCONS_00015178	-0.313498	0.214342	-0.454347	BGIOSGA027880	XP_006653881.1 clathrin light chain 1
TCONS_00023980	-0.313483	-0.457996	0.234147	BGIOSGA021762	XP_006657885.1PREDICTED: uncharacterized protein LOC102716429 isoform X1
TCONS_00026434	-0.313483	-0.977494	0.484682	BGIOSGA022810	XP_015695955.1 pyrophosphate--fructose 6-phosphate 1-phosphotransferase subunit alpha-like
TCONS_00026954	-0.312941	-0.27172	-0.217617	BGIOSGA029008	XP_006660287.1 TBC1 domain family member 5 homolog A
TCONS_00008006	-0.312509	0.183471	0.249809	BGIOSGA006472	XP_006647298.1 polyadenylate-binding protein-interacting protein 4-like
TCONS_00001804	-0.312501	0.981937	0.443422	BGIOSGA000855	XP_015691752.1 probable glutamate carboxypeptidase 2
TCONS_00011332	-0.312294	0.522919	-0.641976	BGIOSGA007340	XP_015689701.1 putative pentatricopeptide repeat-containing protein At1g77010, mitochondrial
TCONS_00006909	-0.312199	-0.346919	0.367442	BGIOSGA009091	XP_002452868.1uncharacterized protein LOC8078905
TCONS_00033598	-0.312133	-0.376148	-0.632722	BGIOSGA013570	XP_015697679.1 leucine-rich repeat extensin-like protein 3
TCONS_00032416	-0.312056	0.0445729	0.0944024	BGIOSGA006017	XP_004982881.1long-chain-alcohol oxidase FAO2
TCONS_00028897	-0.311947	-0.35223	-0.390238	BGIOSGA030641	XP_006660582.2 CD2 antigen cytoplasmic tail-binding protein 2
TCONS_00020179	-0.311849	-0.134343	-0.467266	BGIOSGA022370	XP_006656675.2 putative esterase YitV
TCONS_00019720	-0.311642	-0.258883	-0.532983	BGIOSGA023241	XP_006654726.1 putative aminoacrylate hydrolase RutD isoform X1
TCONS_00035495	-0.311489	-0.694994	0.433287	BGIOSGA033337	XP_004978487.1glutathione transferase GST 23
TCONS_00018230	-0.311451	-0.524605	-0.0202868	BGIOSGA020287	XP_004961358.1uncharacterized protein LOC101762134
TCONS_00024360	-0.31142	-1.32494	-2.66519	BGIOSGA030761	XP_003565395.2probable calcium-binding protein CML24
TCONS_00021217	-0.311181	-1.83788	-1.68911	BGIOSGA023439	XP_015693773.1 disease resistance protein RGA2-like
TCONS_00022871	-0.311171	-1.29608	-2.21986	BGIOSGA007489	XP_021306094.1uncharacterized protein LOC8064953
TCONS_00011788	-0.311075	-1.48199	-0.742786	BGIOSGA011035	XP_006649631.1 digalactosyldiacylglycerol synthase 2, chloroplastic-like
TCONS_00002310	-0.311017	-0.367474	-0.912545	BGIOSGA008421	XP_006645146.1 MLO-like protein 1
TCONS_00015901	-0.310825	-0.645497	0.521608	BGIOSGA015087	XP_006652299.1 golgin subfamily A member 6-like protein 22
TCONS_00032102	-0.310681	-1.10941	-1.20825	BGIOSGA031071	XP_008665991.1ent-kaurenoic acid oxidase
TCONS_00011999	-0.310402	0.174152	0.374844	BGIOSGA000384	XP_006649844.1 putative clathrin assembly protein At2g25430
TCONS_00019796	-0.310189	1.03787	0.826849	BGIOSGA006622	XP_006654786.1 guanylate-binding protein 1

TCONS_00030051	-0.310125	-1.36473	-2.67077	BGIOSGA010627	XP_006661234.1 probable WRKY transcription factor 60
TCONS_00024178	-0.310055	0.706961	0.407917	BGIOSGA009949	XP_002461092.1probable transcription factor At3g04930
TCONS_00000870	-0.309862	2.9011	1.91523	BGIOSGA003424	XP_015696817.1PREDICTED: uncharacterized protein LOC102718134, partial
TCONS_00035830	-0.309701	0.644512	0.784327	BGIOSGA037227	XP_015698255.1PREDICTED: uncharacterized protein LOC102699363
TCONS_00023713	-0.309554	-1.03282	-0.754505	BGIOSGA018911	XP_015695196.1 peptide-N(4)-(N-acetyl-beta-glucosaminyl)asparagine amidase isoform X1
TCONS_00030635	-0.309386	-1.6193	-0.226321	BGIOSGA032465	XP_003573740.1uncharacterized protein LOC100843787 isoform X2
TCONS_00007130	-0.309335	-0.401558	0.741419	BGIOSGA007020	XP_006648143.1 ubiquitin carboxyl-terminal hydrolase 2 isoform X1
TCONS_00018903	-0.309204	0.00401191	-0.60835	BGIOSGA018454	XP_006645169.1 50S ribosomal protein L13
TCONS_00009610	-0.309111	0.226686	0.335481	BGIOSGA030546	XP_006649661.1 wall-associated receptor kinase-like 14
TCONS_00037313	-0.309033	-0.823393	-0.605157	BGIOSGA013803	XP_006664082.1 golgin candidate 6
TCONS_00015151	-0.308962	-1.99229	-0.415104	BGIOSGA017331	XP_008668661.1probable phytol kinase, chloroplastic isoform X1
TCONS_00011819	-0.30889	0.959252	0.928594	BGIOSGA031143	XP_015697827.1 pentatricopeptide repeat-containing protein At5g47360
TCONS_00001445	-0.30887	-0.957786	-1.27674	BGIOSGA004092	XP_015697684.1 carbonic anhydrase, chloroplastic-like isoform X1
TCONS_00009097	-0.308587	0.233092	0.37858	BGIOSGA033046	XP_004954520.1large ribosomal RNA subunit accumulation protein YCED homolog 1, chloroplastic
TCONS_00006849	-0.307869	-0.214866	-0.597488	BGIOSGA003624	XP_006647870.1 chaperone protein dnaJ 16-like
TCONS_00000029	-0.307793	0.819595	0.99727	BGIOSGA006007	XP_006646752.2 something about silencing protein 10-like
TCONS_00008118	-0.307766	-0.38412	0.307295	BGIOSGA004289	XP_006647340.1 germin-like protein 2-4
TCONS_00019794	-0.307677	0.17145	-0.175007	BGIOSGA004180	XP_004961229.1homeobox-DDT domain protein RLT2
TCONS_00009243	-0.307585	-0.0777196	-0.138757	BGIOSGA025787	XP_014753056.1bromodomain-containing protein DDB_G0270170
TCONS_00018038	-0.307254	0.262811	0.466185	BGIOSGA024731	XP_006654566.1 type I inositol polyphosphate 5-phosphatase 10-like
TCONS_00019018	-0.307201	1.19276	-0.206816	BGIOSGA019322	XP_006655175.2 lysine-specific demethylase JMJ18-like
TCONS_00007851	-0.306883	0.122659	-0.182259	BGIOSGA006630	XP_006648568.1 protein DEHYDRATION-INDUCED 19 homolog 4
TCONS_00031730	-0.306813	-1.99901	-0.600051	BGIOSGA039447	XP_006661603.1 protein NRT1/ PTR FAMILY 8.3-like
TCONS_00010857	-0.306771	-0.249857	0.481602	BGIOSGA015774	XP_015691052.1 SWI/SNF complex subunit SWI3D-like
TCONS_00006159	-0.306734	0.886424	0.920497	BGIOSGA000898	XP_006647249.1 elongation factor 2-like
TCONS_00009428	-0.306639	0.865249	-0.105024	BGIOSGA007130	XP_006651097.1 serine/threonine-protein kinase At5g01020-like
TCONS_00025633	-0.3066	0.883362	0.0675747	BGIOSGA023793	XP_002463309.2CTL-like protein DDB_G0274487
TCONS_00021358	-0.30642	-0.418945	-0.263771	BGIOSGA023586	XP_006656477.1 F-box protein SKIP31
TCONS_00017162	-0.306213	0.59119	-0.0357403	BGIOSGA014638	XP_006654061.1 kinesin-13A
TCONS_00019645	-0.30618	-0.521076	0.180058	BGIOSGA017712	XP_002441401.1probable peptidyl-tRNA hydrolase 2 isoform X2
TCONS_00001239	-0.30574	0.0947324	0.526627	BGIOSGA036884	XP_006644321.1 coiled-coil domain-containing protein 94 homolog
TCONS_00031602	-0.305671	-1.25179	-2.11739	BGIOSGA007713	XP_006662580.1 proline dehydrogenase 2, mitochondrial-like
TCONS_00032573	-0.305554	0.88112	-0.473599	BGIOSGA025490	XP_003574208.1mitochondrial import inner membrane translocase subunit TIM23-1
TCONS_00035497	-0.305387	-2.95627	-0.898012	BGIOSGA022006	XP_006663768.1 NAC transcription factor 29-like
TCONS_00021668	-0.305357	0.378378	1.01133	#N/A	#N/A
TCONS_00034469	-0.305352	0.709425	0.200555	BGIOSGA030880	XP_006663277.1 protein disulfide isomerase-like 1-1
TCONS_00022222	-0.305203	-0.688539	-0.442715	BGIOSGA021275	XP_015693913.1 60S ribosomal protein L18a-like protein isoform X1
TCONS_00032625	-0.305191	-1.31059	0.529987	BGIOSGA030813	XP_010504143.1 histone H4
TCONS_00017211	-0.304982	2.60031	0.372957	BGIOSGA006599	XP_002439361.2universal stress protein PHOS32
TCONS_00018266	-0.304802	1.11466	-0.212458	BGIOSGA016888	XP_004961312.1formin-like protein 18
TCONS_00006569	-0.304731	-2.04404	-0.645787	BGIOSGA030133	XP_015689303.1 serine/threonine-protein kinase D6PK
TCONS_00005799	-0.304565	-1.57459	0.186738	BGIOSGA004144	XP_010234116.2F-box/kelch-repeat protein At3g61590
TCONS_00028370	-0.304447	0.503798	0.271007	BGIOSGA030132	XP_006659589.1 60S ribosomal protein L17-like
TCONS_00023532	-0.304375	0.993829	-0.419985	BGIOSGA035871	XP_015694830.1PREDICTED: uncharacterized protein LOC102711362 isoform X1
TCONS_00023242	-0.304254	-0.486898	0.183848	BGIOSGA025283	XP_006657510.2 ATP-dependent DNA helicase 2 subunit KU70
TCONS_00017351	-0.304213	-0.070504	-1.79891	BGIOSGA019217	XP_006654073.1 DNA-directed RNA polymerase V subunit 7-like
TCONS_00019275	-0.303853	-1.18693	-0.988958	BGIOSGA018074	XP_006655309.1 cytochrome P450 734A1-like
TCONS_00015750	-0.303825	-3.20849	-2.79988	BGIOSGA017685	XP_003579586.1ER lumen protein-retaining receptor
TCONS_00037376	-0.303824	-0.184353	-0.56618	BGIOSGA009643	XP_015698426.1 GDSL esterase/lipase At4g28780-like
TCONS_00021496	-0.303768	1.31759	0.521819	BGIOSGA003229	XP_004964368.1uncharacterized protein LOC101766488
TCONS_00002915	-0.303721	-0.281663	0.225944	BGIOSGA002305	XP_006643802.1 protein RST1
TCONS_00014189	-0.303689	-0.538704	0.130682	BGIOSGA016344	XP_006652253.1 histone deacetylase 9
TCONS_00030787	-0.303677	-0.383143	0.401085	BGIOSGA012238	XP_006661660.1 chloride conductance regulatory protein ICln

TCONS_00008925	-0.303506	-0.326442	-0.0894918	BGIOSGA000593	XP_006649101.1PREDICTED: uncharacterized protein LOC102722745, partial
TCONS_00029087	-0.303432	0.530662	-0.453042	BGIOSGA030663	XP_004956927.1exocyst complex component EXO70B1
TCONS_00034774	-0.303394	-0.418958	0.783994	BGIOSGA009845	XP_006663399.1 magnesium-dependent phosphatase 1-like
TCONS_00033381	-0.30334	1.21288	-0.231832	BGIOSGA028843	XP_004979229.1protein transport protein SEC23
TCONS_00001459	-0.303308	-1.60119	-2.12403	BGIOSGA004100	XP_006644431.1 probable sodium/metabolite cotransporter BASS2, chloroplastic
TCONS_00020151	-0.303089	0.263692	0.240035	BGIOSGA018639	XP_006655808.1 probable beta-1,3-galactosyltransferase 14
TCONS_00007502	-0.303027	-0.15208	-0.464942	BGIOSGA004783	XP_015688487.1 putative multidrug resistance protein
TCONS_00009629	-0.302821	-1.41313	-1.78406	BGIOSGA013495	XP_006649675.1 inositol-tetrakisphosphate 1-kinase 2 isoform X1
TCONS_00001911	-0.302757	-1.19466	-0.380568	BGIOSGA012263	XP_015697834.1 myosin-binding protein 1-like
TCONS_00022932	-0.302667	-0.043967	-1.00929	BGIOSGA020513	XP_015694211.1 DDB1- and CUL4-associated factor 8
TCONS_00010024	-0.302418	-0.187106	0.529308	BGIOSGA012578	XP_006651351.2 nardilysin
TCONS_00022350	-0.302346	0.172366	0.437368	BGIOSGA004003	XP_021305342.1DEAD-box ATP-dependent RNA helicase 31
TCONS_00009619	-0.302316	-0.574676	0.0789409	BGIOSGA012164	XP_006649667.1 DNA polymerase I isoform X1
TCONS_00031588	-0.30213	-0.183135	-0.148243	BGIOSGA014016	XP_006662571.1 syndetin
TCONS_00009422	-0.301916	0.957032	0.649439	BGIOSGA011972	XP_006649494.1 elongation factor 1-alpha
TCONS_00001525	-0.301878	-0.786	-0.442215	BGIOSGA004156	XP_006646179.1 carboxyl-terminal-processing peptidase 1, chloroplastic
TCONS_00021437	-0.301875	-0.61815	-0.804795	BGIOSGA027623	XP_010228157.1protein NTM1-like 9
TCONS_00018400	-0.301852	0.110944	-0.667333	BGIOSGA020452	NP_001278572.1harpin-induced protein
TCONS_00033153	-0.30172	0.261001	0.351791	BGIOSGA034338	XP_006662811.1 transcriptional corepressor SEUSS
TCONS_00011275	-0.301702	#NA	-0.635286	BGIOSGA019760	XP_006645841.2 probable glycerol-3-phosphate acyltransferase 3
TCONS_00034156	-0.301593	-1.93031	2.96617	BGIOSGA007029	XP_006663738.1 CBL-interacting protein kinase 15
TCONS_00003998	-0.301563	-0.223887	0.426457	BGIOSGA026927	XP_015697870.1 polypyrimidine tract-binding protein homolog 1-like
TCONS_00025065	-0.301562	-0.605284	-0.259278	BGIOSGA012735	XP_006658541.1PREDICTED: uncharacterized protein LOC102711548
TCONS_00019442	-0.301428	-0.772457	-0.303395	BGIOSGA008732	XP_006654518.1 ras-related protein RABE1c
TCONS_00022317	-0.301411	0.334271	0.224941	BGIOSGA039058	XP_003558495.1probable methionine--tRNA ligase
TCONS_00006274	-0.301346	0.413612	1.06661	BGIOSGA001181	XP_015689141.1 molybdopterin synthase sulfur carrier subunit
TCONS_00025934	-0.301304	0.573697	0.733433	BGIOSGA028007	XP_004972817.1sm-like protein LSM2
TCONS_00005560	-0.301237	0.685478	0.138484	BGIOSGA028355	XP_006646980.1 cinnamoyl-CoA reductase 2-like
TCONS_00013139	-0.301169	-0.417773	-0.378854	BGIOSGA009846	XP_015690983.1 E3 ubiquitin-protein ligase HOS1 isoform X3
TCONS_00008997	-0.300953	0.00218289	-0.983942	BGIOSGA022382	XP_006648071.1 AP-1 complex subunit gamma-2-like
TCONS_00020629	-0.300858	0.569117	-0.0524337	BGIOSGA022811	XP_006656042.1PREDICTED: uncharacterized protein At2g39795, mitochondrial-like
TCONS_00027015	-0.300685	-1.45993	-0.679574	BGIOSGA034765	XP_006659636.1PREDICTED: uncharacterized protein LOC102704999 isoform X2
TCONS_00016230	-0.300638	-0.619546	-0.53775	BGIOSGA014744	XP_006653591.1 endoribonuclease Dicer homolog 4 isoform X1
TCONS_00010143	-0.300499	-1.33273	-0.56818	BGIOSGA030711	XP_015691200.1 pyrrolidone-carboxylate peptidase-like
TCONS_00010762	-0.300483	0.778373	0.344545	BGIOSGA037475	XP_006651722.2 zinc finger CCCH domain-containing protein 24
TCONS_00002607	-0.300019	-0.936669	-0.749676	BGIOSGA030372	XP_006645342.1 beta-fructofuranosidase, insoluble isoenzyme 4 isoform X1
TCONS_00010978	-0.299725	-0.473613	-0.047158	BGIOSGA013581	XP_021304987.1cleavage stimulation factor subunit 50
TCONS_00037176	-0.299104	-1.2518	-3.39838	BGIOSGA019551	XP_006654233.1 high-light-induced protein, chloroplastic
TCONS_00030390	-0.299048	-2.19958	-0.140591	BGIOSGA029409	XP_006660863.1 E3 ubiquitin ligase BIG BROTHER-like
TCONS_00023998	-0.299023	0.31665	-0.196145	#N/A	#N/A
TCONS_00032450	-0.298999	-0.327064	-0.046416	BGIOSGA031669	XP_015697175.1PREDICTED: uncharacterized protein LOC102701300
TCONS_00005116	-0.298882	1.18276	-0.101712	BGIOSGA029456	XP_006645376.1 non-specific phospholipase C2-like
TCONS_00031639	-0.298837	-0.140794	-0.305395	BGIOSGA014171	XP_003574347.1trihelix transcription factor ASIL2
TCONS_00017678	-0.298814	-1.08237	-0.868994	BGIOSGA021098	XP_006654323.1PREDICTED: uncharacterized protein LOC102706193
TCONS_00000114	-0.298666	0.0826444	-0.156665	BGIOSGA002684	XP_006643704.1 probable ubiquitin-conjugating enzyme E2 23 isoform X2
TCONS_00035767	-0.29864	-2.84307	-5.31534	BGIOSGA034194	XP_006664438.1 2-alkenal reductase (NADP(+)-dependent)-like
TCONS_00028431	-0.298631	-4.70413	-0.470523	BGIOSGA031126	XP_015696048.1 kinesin-like protein NACK2 isoform X1
TCONS_00016612	-0.298579	0.162666	-0.592321	BGIOSGA013371	XP_015695774.1 50S ribosomal protein L18
TCONS_00029802	-0.298524	0.468003	-0.0688973	BGIOSGA030017	XP_006661108.1 disease resistance protein RGA2-like
TCONS_00000525	-0.298516	-0.0991838	-0.435091	BGIOSGA018990	NP_001146919.1OB-fold nucleic acid binding domain containing protein
TCONS_00011111	-0.298341	-0.127615	1.12873	BGIOSGA000770	XP_006651895.2 probable zinc metalloprotease EGY1, chloroplastic
TCONS_00027351	-0.298339	0.667221	0.140605	BGIOSGA027647	XP_006659835.2 transmembrane protein 120 homolog
TCONS_00005223	-0.298302	#NA	-1.8317	#N/A	#N/A

TCONS_00024486	-0.29825	-1.75155	0.32323	BGIOSGA002144	XP_006658233.1PREDICTED: uncharacterized protein LOC102719504
TCONS_00034584	-0.298208	-0.967659	0.140282	BGIOSGA025264	XP_002449290.1probable carboxylesterase 18
TCONS_00005309	-0.297966	1.17772	0.361627	BGIOSGA025075	XP_006648257.1 eukaryotic translation initiation factor 3 subunit C-like
TCONS_00034953	-0.297878	0.675824	0.0178432	BGIOSGA023559	XP_015697998.1 auxin response factor 23
TCONS_00015469	-0.297502	-0.498178	-0.499599	BGIOSGA015547	XP_006652119.1 vacuolar protein-sorting-associated protein 33 homolog
TCONS_00004694	-0.297471	-4.63155	-1.90885	BGIOSGA029235	XP_002458734.1protein UPSTREAM OF FLC
TCONS_00028414	-0.297174	-0.0616341	-0.317165	BGIOSGA026546	XP_006659651.1 probable protein NAP1
TCONS_00018244	-0.297131	1.91234	1.13761	BGIOSGA019910	XP_015693381.1 FHA domain-containing protein DDL
TCONS_00001009	-0.296907	-0.271886	-0.686935	BGIOSGA011424	XP_006644215.1 heavy metal-associated isoprenylated plant protein 26-like
TCONS_00016752	-0.296878	-0.047335	-0.116769	BGIOSGA014206	XP_006653829.1 small RNA degrading nuclease 1-like isoform X1
TCONS_00025719	-0.296847	0.472	0.0896054	BGIOSGA010812	XP_015694772.1 probable apyrase 2
TCONS_00037557	-0.296813	0.666815	1.01453	BGIOSGA031853	XP_006664762.1 cationic amino acid transporter 2, vacuolar-like
TCONS_00000531	-0.296775	-0.0603707	0.317749	BGIOSGA037794	XP_006643977.1 receptor-like protein kinase HSL1
TCONS_00021193	-0.296623	-0.510733	-0.109248	BGIOSGA018533	XP_006656323.1 glutaredoxin-C8
TCONS_00018655	-0.296519	-0.088428	-0.294534	BGIOSGA009973	XP_006654065.1 probable ethylene response sensor 2
TCONS_00015359	-0.296233	0.0549477	-0.233841	BGIOSGA015617	XP_006652099.1 AT-rich interactive domain-containing protein 2-like
TCONS_00010022	-0.296182	1.36683	0.144545	BGIOSGA015296	XP_006651349.1 pentatricopeptide repeat-containing protein At1g09900-like
TCONS_00028944	-0.296175	0.159014	-0.121216	BGIOSGA035892	XP_002460179.1uncharacterized protein LOC8055789
TCONS_00006599	-0.296157	0.247722	0.152911	BGIOSGA012246	XP_006662433.2 UDP-glucose:glycoprotein glucosyltransferase
TCONS_00011476	-0.296048	0.221948	-0.0480269	BGIOSGA011549	XP_006649305.1 50S ribosomal protein L5, chloroplastic
TCONS_00027007	-0.29574	-0.897284	0.462153	BGIOSGA030107	XP_015690801.1PREDICTED: uncharacterized protein LOC102721819 isoform X1
TCONS_00007006	-0.295725	-0.0894248	-2.49813	BGIOSGA007661	XP_015689520.1 serine/threonine-protein kinase fray2 isoform X2
TCONS_00038376	-0.295701	-0.685542	-0.584138	BGIOSGA032568	XP_022684857.1disease resistance protein RPM1
TCONS_00002065	-0.295632	-1.34436	-0.597893	BGIOSGA006347	XP_006644911.1 probable inactive receptor kinase At5g58300
TCONS_00005481	-0.295377	-1.35916	-0.352238	BGIOSGA018032	XP_006648341.1PREDICTED: uncharacterized protein LOC102715370
TCONS_00000978	-0.295053	-1.1463	-0.181066	BGIOSGA003581	XP_015697898.1 piezo-type mechanosensitive ion channel homolog isoform X3
TCONS_00009266	-0.294724	0.109992	0.486663	BGIOSGA011803	XP_006649356.1 24-methylenesterol C-methyltransferase 2
TCONS_00002230	-0.294638	0.289358	0.0497079	BGIOSGA027835	XP_015688049.1 tubby-like F-box protein 3
TCONS_00000823	-0.294578	-4.75974	-1.51105	BGIOSGA000357	XP_015688480.1 GDSL esterase/lipase At1g09390-like
TCONS_00002711	-0.294163	-0.229108	-1.37651	BGIOSGA012967	XP_006645443.1 glycerophosphodiester phosphodiesterase protein kinase domain-containing GDPDL2-like
TCONS_00026415	-0.293753	0.0969404	-0.056373	BGIOSGA003622	XP_015695946.1 protein ENHANCED DOWNY MILDEW 2-like isoform X2
TCONS_00016932	-0.2937	-1.30281	-0.218597	BGIOSGA020488	XP_006653081.1 UDP-galactose transporter 1-like
TCONS_00032986	-0.293579	0.240187	-0.133753	BGIOSGA012081	XP_006662741.1 ribonuclease H2 subunit A isoform X1
TCONS_00004558	-0.293473	-1.06215	-0.184206	BGIOSGA020111	XP_006644841.1 putative E3 ubiquitin-protein ligase RING1a
TCONS_00004508	-0.293451	0.303867	-0.388474	BGIOSGA028097	XP_015699033.1 helicase-like transcription factor CHR28 isoform X1
TCONS_00008070	-0.29344	-0.811351	-0.378646	BGIOSGA006382	XP_015689579.1 RING finger protein 10
TCONS_00001988	-0.293324	-2.99639	-2.70743	BGIOSGA004624	XP_015693543.1 nucleotide pyrophosphatase/phosphodiesterase-like
TCONS_00005735	-0.293213	-0.91346	-1.51443	BGIOSGA021073	XP_003572164.1berberine bridge enzyme-like 23
TCONS_00004946	-0.293179	-0.48983	0.156215	BGIOSGA000275	NP_001132810.2putative ACR family protein
TCONS_00031384	-0.293155	-0.144518	-0.0107412	BGIOSGA033196	XP_006662473.1 E3 ubiquitin-protein ligase Hakai
TCONS_00019819	-0.293122	-1.43527	-0.148654	BGIOSGA007532	XP_006647563.1PREDICTED: uncharacterized protein LOC102699931
TCONS_00010926	-0.293115	0.68451	0.700535	BGIOSGA024007	XP_015691263.1 protein WRKY1-like
TCONS_00030687	-0.293089	1.03839	1.83279	BGIOSGA017121	XP_015696420.1 phospholipase A(1) LCAT3
TCONS_00018634	-0.292908	-1.84999	-0.939058	BGIOSGA022793	XP_006654045.1 sodium/hydrogen exchanger 2-like
TCONS_00015523	-0.292798	0.415024	-0.162716	BGIOSGA015458	XP_006652142.1 ER membrane protein complex subunit 8/9 homolog
TCONS_00009577	-0.292601	0.180704	-0.415826	BGIOSGA012128	XP_006649644.1 protein OBERON 4-like
TCONS_00020512	-0.292516	-0.566922	-0.771531	BGIOSGA006821	XP_004965216.1LRR receptor kinase SERL2
TCONS_00027249	-0.292513	-0.335803	0.276035	BGIOSGA027752	XP_014756366.1putative vacuolar protein sorting-associated protein 13A isoform X1
TCONS_00020766	-0.292461	-0.689279	0.176003	BGIOSGA022964	NP_001149074.1nucleic acid binding protein
TCONS_00013938	-0.29243	-0.411727	0.165076	BGIOSGA008711	XP_015692250.1 dnaJ protein ERDJ2A
TCONS_00002476	-0.292421	-1.5792	-0.71855	BGIOSGA005120	XP_006646654.1 psbP domain-containing protein 7, chloroplastic
TCONS_00004573	-0.292381	0.951393	-0.154011	BGIOSGA000641	XP_006644853.2PREDICTED: uncharacterized protein LOC102712310
TCONS_00007769	-0.292029	-0.254119	0.962705	BGIOSGA024573	XP_006647152.1 uridine kinase-like protein 3
TCONS_00006142	-0.292023	-0.0588389	-0.719762	BGIOSGA033105	XP_006648669.2 serine/threonine-protein kinase CDL1
TCONS_00015845	-0.291882	-0.990935	-1.95829	BGIOSGA020506	XP_015692161.1 1,4-alpha-glucan-branching enzyme 1, chloroplastic/amyloplastic-like

TCONS_00029355	-0.291478	-1.32839	#NA	BGIOSGA031104	XP_022679765.1cytochrome c oxidase assembly factor 5
TCONS_00008349	-0.291417	0.0102634	0.198105	BGIOSGA014801	XP_021303370.1LETM1 and EF-hand domain-containing protein 1, mitochondrial
TCONS_00013280	-0.291415	-0.643377	-0.466068	BGIOSGA024824	XP_006651884.2 WPP domain-associated protein-like
TCONS_00011682	-0.291368	-1.41497	0.51157	BGIOSGA011327	XP_015696269.1 SAP-like protein BP-73
TCONS_00014308	-0.291348	-1.21514	-1.37462	BGIOSGA015027	XP_006652319.2PREDICTED: uncharacterized protein LOC102718642 isoform X1
TCONS_00005527	-0.291341	0.116366	0.866173	BGIOSGA007636	XP_015689414.1 cytochrome P450 97B2, chloroplastic
TCONS_00032634	-0.29118	0.958202	0.144434	BGIOSGA015170	XP_015691446.1 ubiquitin domain-containing protein DSK2a-like
TCONS_00017416	-0.29105	-1.45186	0.338163	BGIOSGA035852	XP_015693281.1 probable LRR receptor-like serine/threonine-protein kinase At1g56140
TCONS_00016194	-0.290943	-0.763135	-0.625724	BGIOSGA006057	XP_009390559.1 BTB/POZ domain-containing protein At2g24240
TCONS_00028257	-0.290637	0.695225	0.139485	BGIOSGA026721	XP_006659561.1 serine--glyoxylate aminotransferase
TCONS_00002156	-0.290546	0.609604	0.914232	BGIOSGA009303	XP_006644998.1 pumilio homolog 1-like isoform X1
TCONS_00030133	-0.290459	0.310197	-0.0263708	BGIOSGA031979	XP_022680184.1 mitochondrial substrate carrier family protein J-like
TCONS_00022543	-0.290441	-0.940146	-0.313306	BGIOSGA010699	XP_006656239.1PREDICTED: uncharacterized protein LOC102719683
TCONS_00003209	-0.290275	0.826766	0.270657	BGIOSGA002008	XP_015699249.1 probable histone acetyltransferase HAC-like 3 isoform X3
TCONS_00024930	-0.290035	-1.10908	0.287719	BGIOSGA024456	XP_010231242.14-hydroxyphenylacetaldehyde oxime monooxygenase
TCONS_00013319	-0.290024	0.392816	-0.486601	BGIOSGA009665	XP_006651897.1 replication factor C subunit 5
TCONS_00026027	-0.289874	0.669094	1.84771	#N/A	#N/A
TCONS_00018281	-0.289786	-0.649111	-1.72812	BGIOSGA001019	NP_001339548.1uncharacterized LOC100273076
TCONS_00028325	-0.28954	-0.954828	-0.794703	BGIOSGA026646	XP_006659603.1PREDICTED: uncharacterized protein LOC102719139
TCONS_00022697	-0.289164	-0.962497	-4.09964	BGIOSGA020744	XP_002438796.1cryptochrome DASH, chloroplastic/mitochondrial
TCONS_00024089	-0.289126	1.23431	0.78007	BGIOSGA036025	XP_015695366.1 exportin-T
TCONS_00007790	-0.288675	0.137473	-0.57293	BGIOSGA034263	XP_006655122.1 disease resistance protein RPP13-like
TCONS_00003312	-0.288572	0.0427182	0.174192	BGIOSGA000610	XP_006644054.1 60S ribosomal protein L30 isoform X1
TCONS_00006100	-0.288482	0.448821	0.239539	BGIOSGA033107	XP_022679774.1uncharacterized protein LOC101754006
TCONS_00004483	-0.288373	0.249838	0.0786545	BGIOSGA033768	XP_015699161.1 ubiquitin carboxyl-terminal hydrolase 13-like isoform X2
TCONS_00008214	-0.288131	0.371758	-0.201903	BGIOSGA029631	XP_003575194.1probable protein phosphatase 2C 15
TCONS_00019431	-0.288116	-1.34066	0.067494	BGIOSGA017922	XP_015692742.1PREDICTED: uncharacterized protein LOC102711533
TCONS_00006193	-0.28803	-0.408344	0.137609	BGIOSGA033294	XP_006648690.1PREDICTED: uncharacterized protein LOC102707561
TCONS_00017845	-0.287532	0.664469	0.63831	BGIOSGA000382	XP_006654426.1 F-box protein SKP2A-like
TCONS_00001749	-0.287522	-0.986555	-0.0759007	BGIOSGA017230	XP_015693009.1PREDICTED: LOW QUALITY PROTEIN: uncharacterized protein LOC102722719
TCONS_00028102	-0.287017	0.0601951	-0.179042	BGIOSGA029700	XP_012702565.1AT-rich interactive domain-containing protein 4 isoform X1
TCONS_00027365	-0.286926	0.260621	0.978096	BGIOSGA027637	XP_006659126.1 U3 small nucleolar ribonucleoprotein protein IMP4
TCONS_00003471	-0.286876	-0.414496	-0.505035	BGIOSGA022582	XP_015699345.1 dihydrolipoyl dehydrogenase 1, mitochondrial-like
TCONS_00021281	-0.286793	-0.0137324	-0.0529045	#N/A	#N/A
TCONS_00027268	-0.286613	0.0793024	-0.201046	BGIOSGA020329	XP_006659077.1 serine/threonine protein phosphatase 2A 57 kDa regulatory subunit B' theta isoform-like
TCONS_00000782	-0.286207	0.0438713	-0.421912	BGIOSGA003379	XP_006645823.2 aarF domain-containing protein kinase 4
TCONS_00024299	-0.28613	-0.974902	0.155317	BGIOSGA038680	XP_004958606.1momilactone A synthase
TCONS_00034560	-0.285967	-0.878892	-0.786193	BGIOSGA027332	XP_021317317.1disease resistance protein RPM1
TCONS_00028073	-0.285377	0.876978	-0.0880561	BGIOSGA014531	XP_006659447.1 stromal cell-derived factor 2-like protein
TCONS_00027461	-0.285354	-0.81646	-0.790108	BGIOSGA017202	XP_006659180.1 diacylglycerol kinase 5-like
TCONS_00014824	-0.285348	-0.562483	-0.728475	BGIOSGA016995	XP_015692223.1PREDICTED: uncharacterized protein LOC102716679
TCONS_00021322	-0.28528	-0.364467	0.237633	BGIOSGA023552	XP_015693697.1 subtilisin-like protease SBT2.6
TCONS_00008913	-0.285222	0.167181	-0.132877	BGIOSGA005496	XP_015689363.1PREDICTED: uncharacterized protein LOC102708496 isoform X1
TCONS_00028453	-0.285186	0.657333	0.19955	BGIOSGA024746	XP_006659679.1PREDICTED: uncharacterized protein LOC102717458
TCONS_00020334	-0.285162	-1.4738	-0.224581	BGIOSGA007258	XP_006655900.1 WAT1-related protein At1g21890-like
TCONS_00013642	-0.284822	0.328644	-0.440466	BGIOSGA012717	XP_006653119.2 pentatricopeptide repeat-containing protein At3g57430, chloroplastic
TCONS_00008036	-0.284774	-0.499937	0.22743	BGIOSGA030977	XP_006648647.1 galacturonosyltransferase 8
TCONS_00027205	-0.284693	-0.250743	-1.61386	BGIOSGA014032	XP_006659027.1 UDP-galactose transporter 1-like
TCONS_00009823	-0.284491	0.328307	-0.580958	BGIOSGA018909	XP_006651269.1 probable sugar phosphate/phosphate translocator At3g11320
TCONS_00019861	-0.28416	0.162486	0.64995	BGIOSGA037752	XP_006654831.1 serine/threonine-protein kinase HT1-like
TCONS_00035074	-0.284042	-1.21121	-0.91874	BGIOSGA009680	XP_006662974.1 E3 ubiquitin-protein ligase RHF2A
TCONS_00021541	-0.283976	1.1899	-0.576842	BGIOSGA022009	XP_004964428.1histone H1

TCONS_00032172	-0.283891	-0.340022	0.454602	#N/A	#N/A
TCONS_00007819	-0.283781	-0.151145	-0.361989	BGIOSGA006667	XP_006647180.1 dipeptidyl aminopeptidase 4
TCONS_00017210	-0.283747	-0.596383	-0.198315	BGIOSGA019255	XP_015692888.1 probable LRR receptor-like serine/threonine-protein kinase At4g26540
TCONS_00009345	-0.283653	0.498022	0.536723	BGIOSGA020760	XP_015691094.1 la-related protein 1B-like
TCONS_00003895	-0.283583	0.165423	-0.550238	BGIOSGA000917	XP_006646048.1 mitochondrial outer membrane protein porin 3
TCONS_00012867	-0.283361	0.0437796	0.235189	BGIOSGA028779	XP_002464123.1protein VASP homolog
TCONS_00026248	-0.283295	-1.45273	-1.04508	BGIOSGA028327	XP_006659266.1 lactation elevated protein 1-like
TCONS_00015786	-0.283269	-1.66057	-1.49761	BGIOSGA006404	XP_002447708.1U6 snRNA-associated Sm-like protein LSm6
TCONS_00032838	-0.283058	-1.7698	-1.36717	BGIOSGA023719	XP_006662662.1 peroxidase 4-like
TCONS_00010778	-0.283057	0.590472	0.209898	BGIOSGA019747	XP_006650459.1 eukaryotic peptide chain release factor subunit 1-3
TCONS_00002458	-0.28301	0.815098	0.14729	BGIOSGA005102	XP_015688251.1 unconventional prefoldin RPB5 interactor 1
TCONS_00014130	-0.282795	0.00422253	-0.393514	BGIOSGA037505	XP_008798980.1 probable metal-nicotianamine transporter YSL6
TCONS_00009405	-0.28252	-0.67911	-0.368206	BGIOSGA031966	XP_004985529.1uncharacterized protein LOC101762981
TCONS_00014602	-0.28246	0.265144	0.114932	BGIOSGA008647	XP_006653601.1 inositol phosphorylceramide glucuronosyltransferase 1-like
TCONS_00020612	-0.282332	-0.209364	0.586924	BGIOSGA022796	XP_006654192.1 nuclear poly(A) polymerase 1-like
TCONS_00022852	-0.282238	0.555689	-0.0514523	BGIOSGA000187	XP_006656439.2 26S proteasome non-ATPase regulatory subunit 2 homolog A-like
TCONS_00025006	-0.282182	0.465931	0.218789	BGIOSGA024393	XP_003563194.1ankyrin repeat domain-containing protein 13B
TCONS_00029648	-0.282176	-0.38725	-0.253688	BGIOSGA030171	XP_015696271.1 auxin transport protein BIG
TCONS_00007523	-0.282162	0.111339	0.0724074	BGIOSGA037972	XP_021314179.1citrate synthase 4, mitochondrial
TCONS_00002624	-0.28203	-0.636041	-1.2589	BGIOSGA019023	XP_006645332.2 protein DEHYDRATION-INDUCED 19 homolog 5
TCONS_00015022	-0.281984	1.00712	0.286318	BGIOSGA035948	XP_006652884.1 probable galacturonosyltransferase 10
TCONS_00014991	-0.281905	-0.45907	-0.214777	BGIOSGA007819	XP_006652858.2PREDICTED: uncharacterized protein LOC102713621
TCONS_00025448	-0.28178	0.378186	0.362752	BGIOSGA023981	XP_002463116.1aspartic proteinase CDR1
TCONS_00018579	-0.281457	-1.25652	-1.60192	BGIOSGA001476	XP_006654967.1 CBL-interacting protein kinase 17
TCONS_00027817	-0.281436	0.0207107	0.493882	BGIOSGA027185	XP_006659298.1 nuclear inhibitor of protein phosphatase 1
TCONS_00000777	-0.28133	-0.547652	-0.798004	BGIOSGA019714	XP_006644124.1PREDICTED: uncharacterized protein LOC102706140
TCONS_00029375	-0.281184	0.356409	0.447949	BGIOSGA031127	XP_003578489.130S ribosomal protein S31, mitochondrial
TCONS_00004624	-0.281043	-1.1436	0.0459037	BGIOSGA005483	XP_015688199.1PREDICTED: uncharacterized protein LOC102699464
TCONS_00003260	-0.280966	-0.16296	-0.0991502	BGIOSGA001955	XP_006644015.1 FAD synthase-like
TCONS_00014583	-0.280792	0.424416	-2.57021	BGIOSGA019047	XP_006652500.1 ammonium transporter 1 member 1
TCONS_00012884	-0.280749	-0.622381	-0.312907	BGIOSGA025119	XP_015691087.1 switch-associated protein 70-like
TCONS_00034386	-0.280702	0.589801	0.750379	BGIOSGA034420	XP_010237897.2integrator complex subunit 3
TCONS_00014481	-0.280602	1.45221	0.280705	BGIOSGA020627	XP_006653547.1 probable inactive receptor kinase At1g27190
TCONS_00024340	-0.280446	-0.154698	0.735016	BGIOSGA026381	XP_006658117.1 probable acyl-CoA dehydrogenase IBR3
TCONS_00020703	-0.280268	-0.476771	-0.414942	BGIOSGA040525	XP_006656078.2 1,4-alpha-glucan-branching enzyme 3, chloroplastic/amyloplastic
TCONS_00031065	-0.280141	0.231955	-0.413461	BGIOSGA036737	XP_006661758.1 protein kinase 2B, chloroplastic
TCONS_00023900	-0.280124	-0.164938	-0.68867	BGIOSGA037599	XP_006657823.1 cytochrome b6-f complex iron-sulfur subunit, chloroplastic
TCONS_00001222	-0.279949	0.259244	0.660027	BGIOSGA003850	XP_006646040.2 small subunit processome component 20 homolog
TCONS_00022051	-0.279821	0.0641621	0.0989053	BGIOSGA021465	XP_006655996.1 importin-9
TCONS_00009108	-0.279426	-0.254678	0.0337233	BGIOSGA006186	XP_006648186.1 protein FD-like
TCONS_00030863	-0.279394	0.563524	-0.478513	BGIOSGA014527	XP_006662259.1 protein DETOXIFICATION 42-like
TCONS_00016085	-0.279362	-4.44362	-2.22571	BGIOSGA037299	XP_006652404.2 beta-glucosidase 10-like
TCONS_00010618	-0.279351	0.146099	-0.483254	BGIOSGA037830	XP_004982260.1cation/calcium exchanger 1
TCONS_00017445	-0.279298	-0.682522	0.677907	BGIOSGA028941	XP_006659555.1 phosphoribosylglycinamide formyltransferase, chloroplastic isoform X1
TCONS_00027581	-0.27903	-0.345383	-0.351229	BGIOSGA024746	XP_004972553.1YTH domain-containing family protein 2
TCONS_00018202	-0.278897	-1.30341	-0.823591	BGIOSGA000913	XP_006654708.1 protein IQ-DOMAIN 31-like
TCONS_00012480	-0.278826	-1.71923	-0.423406	BGIOSGA010499	XP_010230025.2 U-box domain-containing protein 57
TCONS_00002419	-0.278815	-0.616184	-2.02203	BGIOSGA025562	XP_015690763.1PREDICTED: uncharacterized protein LOC102709336
TCONS_00011765	-0.278735	0.0966115	0.152127	BGIOSGA011240	XP_006649601.1 protein RBL
TCONS_00000762	-0.278634	-0.123288	-0.112208	BGIOSGA026454	XP_015699292.1PREDICTED: uncharacterized protein LOC102722811 isoform X1
TCONS_00020458	-0.278561	-0.500409	-0.0173983	BGIOSGA012622	XP_015694040.1 chromatin structure-remodeling complex protein SYD isoform X4
TCONS_00003516	-0.278318	-1.06831	-0.731323	BGIOSGA011580	XP_006661554.1 delta(14)-sterol reductase
TCONS_00020142	-0.27814	-0.556487	-0.241785	BGIOSGA011277	XP_003564311.1protein sip5
TCONS_00011707	-0.278064	-0.162807	-0.197898	BGIOSGA011303	XP_006649534.2PREDICTED: uncharacterized protein LOC102719279
TCONS_00027634	-0.277926	0.129331	0.198049	BGIOSGA032735	XP_006659242.1PREDICTED: uncharacterized protein LOC102703886
TCONS_00016054	-0.27786	-1.41011	-0.0293565	BGIOSGA014923	XP_015692342.1 protection of telomeres protein 1b-like

transcriptome

TCONS_00016664	-0.277618	-0.0600298	0.10641	BGIOSGA014298	XP_015692239.1PREDICTED: uncharacterized protein LOC102699586 isoform X2
TCONS_00007327	-0.277568	-0.812141	0.145354	BGIOSGA034139	XP_006646871.1 cycloartenol synthase
TCONS_00013519	-0.27752	0.0952657	0.359977	BGIOSGA008587	XP_006650881.1 putative RNA-binding protein Luc7-like 2
TCONS_00034794	-0.277393	#NA	#NA	#N/A	#N/A
TCONS_00037886	-0.277393	-1.34939	0.701773	#N/A	#N/A
TCONS_00005822	-0.277373	-1.54365	-0.740536	BGIOSGA007946	XP_006647149.2 transcription factor MYB28-like isoform X2
TCONS_00030878	-0.277249	-0.508165	-0.354884	BGIOSGA032677	XP_006661697.1PREDICTED: uncharacterized protein LOC102717002
TCONS_00012150	-0.277202	1.55734	0.0760181	BGIOSGA016345	XP_006649984.1 neutral/alkaline invertase 1, mitochondrial
TCONS_00016954	-0.277069	0.194372	-0.0263253	BGIOSGA019005	XP_006653942.1 transforming growth factor-beta receptor-associated protein 1
TCONS_00020365	-0.276639	0.157781	-0.061656	BGIOSGA037116	NP_001132153.2uncharacterized LOC100193572
TCONS_00013199	-0.276568	-0.540232	-0.285206	BGIOSGA027771	XP_006650607.1PREDICTED: uncharacterized protein LOC102700413
TCONS_00016515	-0.276453	-0.872526	0.503718	BGIOSGA029607	XP_006652722.1 E3 ubiquitin-protein ligase Os04g0590900-like
TCONS_00025795	-0.276437	-0.154656	-0.245611	BGIOSGA022888	XP_015696195.1 bromodomain-containing factor 1
TCONS_00007411	-0.276321	-1.2732	-0.885399	BGIOSGA016325	XP_006646930.1 OTU domain-containing protein DDB_G0284757-like
TCONS_00034267	-0.276081	1.01251	-0.108317	BGIOSGA038562	XP_006664317.2 cytochrome c-type biogenesis ccda-like chloroplastic protein 1
TCONS_00011045	-0.276073	-1.52224	-0.74824	BGIOSGA031303	XP_006650665.1 leucine-rich repeat receptor-like serine/threonine-protein kinase BAM1
TCONS_00005674	-0.275915	-4.26939	-2.28584	BGIOSGA020994	XP_015688972.1 U-box domain-containing protein 34-like
TCONS_00034432	-0.275892	0.818448	0.269968	BGIOSGA027623	NP_001308992.1putative NAC domain transcription factor superfamily protein
TCONS_00006441	-0.275479	0.704451	0.122831	BGIOSGA034914	XP_003575334.1protein TAB2 homolog, chloroplastic
TCONS_00013745	-0.275438	0.78441	-0.113484	BGIOSGA002922	XP_006653179.1PREDICTED: uncharacterized protein LOC102699860
TCONS_00029193	-0.275388	-0.648983	-0.0978175	BGIOSGA026787	XP_006660729.1 dicarboxylate transporter 2.1, chloroplastic-like
TCONS_00005317	-0.275286	1.23775	-0.288415	BGIOSGA007427	XP_003573769.1protein LOW PSII ACCUMULATION 2, chloroplastic
TCONS_00036682	-0.275268	0.21637	-0.922713	BGIOSGA032896	XP_015698830.1 IQ domain-containing protein IQM3-like
TCONS_00019440	-0.275234	-0.558392	-1.08272	BGIOSGA017913	XP_006654516.1 very-long-chain (3R)-3-hydroxyacyl-CoA dehydratase 2
TCONS_00024150	-0.275217	-0.224695	-0.802335	BGIOSGA017730	XP_006658854.1 myb-like protein Q
TCONS_00001929	-0.275138	-0.323948	-0.663258	BGIOSGA014087	XP_006646391.1 katanin p80 WD40 repeat-containing subunit B1 homolog
TCONS_00029781	-0.275125	-0.233145	0.258413	BGIOSGA024445	XP_006660667.1 transcription initiation factor TFIID subunit 15 isoform X1
TCONS_00011923	-0.275099	0.053295	0.154117	BGIOSGA001977	XP_006649759.1 topless-related protein 1-like
TCONS_00005198	-0.274777	-0.0123211	-0.506984	BGIOSGA010981	XP_006645409.1 ABC transporter B family member 20-like
TCONS_00033404	-0.27432	-2.59897	0.526764	BGIOSGA009108	XP_006662873.1PREDICTED: uncharacterized protein LOC102718029
TCONS_00016033	-0.274313	-0.0209766	0.143229	BGIOSGA028697	XP_004975879.1betaine aldehyde dehydrogenase 1
TCONS_00001927	-0.274283	-0.148271	-0.205253	BGIOSGA014196	XP_015694229.1 L-type lectin-domain containing receptor kinase IX.1-like
TCONS_00000825	-0.274266	-1.86507	-0.138957	BGIOSGA003415	XP_006645844.1 GDSL esterase/lipase LIP-4-like
TCONS_00021152	-0.274198	-0.192625	0.429915	BGIOSGA025368	XP_006656305.1 transcription initiation factor TFIID subunit 5
TCONS_00000568	-0.274195	-0.436691	0.147093	BGIOSGA003148	NP_001144560.1uncharacterized protein LOC100277564
TCONS_00024124	-0.27403	0.00931441	-0.0263074	BGIOSGA026159	XP_006658826.1 AP-4 complex subunit mu
TCONS_00028229	-0.273848	0.213373	-0.337536	BGIOSGA028441	XP_003574688.1heterogeneous nuclear ribonucleoprotein A0 isoform X2
TCONS_00001298	-0.273693	-1.15308	-1.46231	BGIOSGA028917	XP_003569313.1transcription factor MYBS3
TCONS_00008572	-0.273546	-0.313076	0.602336	BGIOSGA025652	XP_003570086.1E3 ubiquitin-protein ligase At1g63170
TCONS_00034305	-0.273493	0.330347	-0.00338046	BGIOSGA034498	XP_022683907.1DDT domain-containing protein PTM
TCONS_00020460	-0.27341	0.359341	0.423743	BGIOSGA022640	XP_006655977.2 exocyst complex component EXO70A1-like isoform X1
TCONS_00027974	-0.273272	0.331588	-0.249563	BGIOSGA038651	XP_015693091.1 putative disease resistance RPP13-like protein 2 isoform X2
TCONS_00027169	-0.273262	-0.880395	-3.33022	BGIOSGA029219	XP_006660403.1 protein PROTON GRADIENT REGULATION 5, chloroplastic
TCONS_00012139	-0.273076	-0.807681	-0.800883	BGIOSGA008412	XP_004977032.1uncharacterized protein LOC101770369
TCONS_00032471	-0.272897	-2.45834	-3.3813	BGIOSGA007412	XP_015697281.1 S-(+)-linalool synthase, chloroplastic-like
TCONS_00005880	-0.272553	0.0405205	0.164322	BGIOSGA038236	XP_015689574.1PREDICTED: uncharacterized protein LOC102709338 isoform X1
TCONS_00031705	-0.27246	-1.25854	-1.5019	BGIOSGA013495	XP_006662130.1 inositol-tetrakisphosphate 1-kinase 1
TCONS_00016496	-0.272401	0.717536	-0.880562	BGIOSGA005824	XP_008668978.1uncharacterized protein At4g15970
TCONS_00016087	-0.272389	-2.04952	-1.30456	BGIOSGA011222	XP_006652406.1 beta-glucosidase 13
TCONS_00030896	-0.272343	0.380428	-0.00623661	BGIOSGA011673	XP_003577058.1uncharacterized protein LOC100837604 isoform X1
TCONS_00031733	-0.272129	-0.64373	-1.23924	BGIOSGA021117	XP_006661605.2 cell number regulator 10-like
TCONS_00002117	-0.27196	-0.524359	-1.69741	BGIOSGA004744	XP_006644959.1PREDICTED: uncharacterized protein LOC102719339

TCONS_00024757	-0.271905	-0.698126	-1.46676	BGIOSGA024620	XP_010234976.1exocyst complex component EXO70E2
TCONS_00033046	-0.271857	2.01453	0.233565	BGIOSGA015096	XP_020407531.1leucine-rich repeat receptor protein kinase EMS1
TCONS_00017481	-0.271829	-0.211706	0.0643918	BGIOSGA017940	XP_006654199.2 phosphatidylinositol:ceramide inositolphosphotransferase-like
TCONS_00024345	-0.271818	1.10253	-0.896777	BGIOSGA026385	XP_015691194.1 mannose-1-phosphate guanyltransferase alpha
TCONS_00013221	-0.271394	0.403597	-0.202766	BGIOSGA001432	XP_006650640.1 mitogen-activated protein kinase kinase kinase YODA-like
TCONS_00009728	-0.271277	-0.727153	-0.0473251	BGIOSGA029198	XP_003558293.1uncharacterized TPR repeat-containing protein At1g05150
TCONS_00023914	-0.271251	-0.246217	-0.0243783	BGIOSGA023709	XP_006658693.1 B3 domain-containing protein Os07g0563300
TCONS_00016685	-0.271101	-1.06171	0.168684	BGIOSGA036785	XP_006652875.1 G-type lectin S-receptor-like serine/threonine-protein kinase At1g11410
TCONS_00001500	-0.271063	-1.93146	-0.785694	BGIOSGA017925	XP_006646161.1 probable protein phosphatase 2C 8
TCONS_00032652	-0.270984	0.474111	0.394387	BGIOSGA031458	XP_015697308.1 translocase of chloroplast 132, chloroplastic-like
TCONS_00007154	-0.270887	-0.93636	-1.1866	BGIOSGA001837	XP_006648164.1PREDICTED: uncharacterized protein LOC102709148
TCONS_00024339	-0.270788	0.813316	0.312487	BGIOSGA019210	XP_006658116.1 60S ribosomal protein L18-3-like
TCONS_00010853	-0.270693	0.188426	0.645781	BGIOSGA013478	XP_006651758.2 eIF-2-alpha kinase activator GCN1
TCONS_00021240	-0.270686	-0.869584	0.0327274	BGIOSGA008744	XP_015692476.1 anaphase-promoting complex subunit 8-like
TCONS_00033021	-0.270621	-1.2847	-0.753725	BGIOSGA034856	XP_006662757.1 disease resistance protein RGA2-like
TCONS_00007453	-0.270125	-0.467289	-0.00819812	BGIOSGA016474	XP_006646962.1 calmodulin-binding protein 60 B-like
TCONS_00023161	-0.270121	0.409641	0.0572161	BGIOSGA013504	XP_015695236.1 ubiquitin carboxyl-terminal hydrolase 12-like
TCONS_00020845	-0.269992	0.772084	0.523631	BGIOSGA023052	XP_006657030.1PREDICTED: uncharacterized protein LOC102704430
TCONS_00030299	-0.269944	-0.774888	0.121269	BGIOSGA029509	XP_008668192.1lipid binding protein isoform X1
TCONS_00008371	-0.269942	-0.492007	0.473249	BGIOSGA014772	XP_015689232.1 F-box/LRR-repeat protein 14-like
TCONS_00016403	-0.269833	-4.3595	#NA	BGIOSGA005973	XP_008663515.1uncharacterized protein LOC103641989
TCONS_00023687	-0.269701	0.757412	0.33946	BGIOSGA023962	XP_006657710.1 photosynthetic NDH subunit of subcomplex B 3, chloroplastic
TCONS_00036744	-0.269678	-0.390178	0.461564	BGIOSGA027811	XP_006664362.1 serine/threonine-protein kinase STY8-like
TCONS_00026580	-0.269507	-0.705478	-0.662165	BGIOSGA028648	XP_006664057.1PREDICTED: uncharacterized protein LOC102705771
TCONS_00020434	-0.269479	-0.370843	-0.963759	BGIOSGA022618	XP_006656839.2 alanine--tRNA ligase, chloroplastic/mitochondrial
TCONS_00017989	-0.269416	0.950171	-0.633263	BGIOSGA000561	XP_021303488.1PRA1 family protein B2-like
TCONS_00034486	-0.269178	0.539457	0.237001	BGIOSGA003374	XP_004967389.2uncharacterized protein LOC101762180
TCONS_00026676	-0.269145	-0.816766	-0.611468	BGIOSGA028743	XP_006659448.1 NADP-dependent glyceraldehyde-3-phosphate dehydrogenase
TCONS_00002865	-0.269092	-0.549284	-1.30522	BGIOSGA002354	XP_006643775.1 protein PHLOEM PROTEIN 2-LIKE A1-like
TCONS_00036192	-0.269042	0.1581	-0.251435	BGIOSGA040690	XP_015698324.1PREDICTED: uncharacterized protein LOC102701322
TCONS_00012259	-0.268926	0.00267633	0.188851	BGIOSGA024065	XP_006651369.2PREDICTED: uncharacterized protein LOC102720970
TCONS_00016853	-0.268883	#NA	#NA	BGIOSGA014111	XP_006653866.1 uracil-DNA glycosylase, mitochondrial
TCONS_00029081	-0.268782	-0.446436	0.393357	BGIOSGA025024	XP_006660657.1 2Fe-2S ferredoxin-like isoform X2
TCONS_00035693	-0.268739	-1.65854	-1.52221	BGIOSGA034518	XP_003578818.1BTB/POZ domain-containing protein At2g13690
TCONS_00021106	-0.26866	-0.769761	-0.933207	BGIOSGA023330	XP_006657168.1PREDICTED: uncharacterized protein LOC102707322
TCONS_00000317	-0.268586	0.847065	0.0264991	BGIOSGA002891	XP_006643832.1PREDICTED: uncharacterized protein LOC102715614 isoform X2
TCONS_00008652	-0.268556	0.223092	0.0682122	BGIOSGA005775	XP_006647759.1PREDICTED: uncharacterized protein LOC102710093
TCONS_00003017	-0.268549	1.05285	-0.433203	BGIOSGA018635	XP_003564938.1PRA1 family protein F3
TCONS_00025637	-0.268531	-0.784214	-1.36446	BGIOSGA009199	XP_006658912.2 serine carboxypeptidase II-3-like
TCONS_00024052	-0.268354	-2.14043	-0.44816	BGIOSGA033506	XP_015694479.1 protein NRT1/ PTR FAMILY 8.3-like
TCONS_00037300	-0.268306	-0.020601	-0.426582	BGIOSGA036068	XP_006664605.1PREDICTED: uncharacterized protein LOC102718980
TCONS_00003000	-0.268192	-0.404573	1.13311	BGIOSGA013472	XP_004968503.1transcription factor RAX2
TCONS_00009443	-0.26809	-0.521477	-0.271297	BGIOSGA032005	XP_006649507.1 phosphoinositide phosphatase SAC2 isoform X2
TCONS_00024711	-0.267987	0.0124263	-0.391209	BGIOSGA010340	XP_006657534.2 phosphoacetylglucosamine mutase
TCONS_00014533	-0.267907	-1.07914	-0.500239	BGIOSGA008607	XP_015691734.1 S-adenosylmethionine decarboxylase proenzyme
TCONS_00019834	-0.267437	-0.0624018	0.633198	BGIOSGA017522	XP_004961159.1uncharacterized protein LOC101784147 isoform X1
TCONS_00014545	-0.267284	-1.11062	-0.0850402	BGIOSGA018841	XP_006652479.1 shikimate O-hydroxycinnamoyltransferase
TCONS_00036205	-0.267144	-0.600292	-0.276954	BGIOSGA024350	XP_002442300.2F-box/LRR-repeat protein 3
TCONS_00033968	-0.26677	-0.791714	-0.273418	BGIOSGA000267	XP_004979897.1WD repeat-containing protein VIP3
TCONS_00004654	-0.266755	0.528222	-0.425821	BGIOSGA020038	XP_015690280.1 protein RETICULATA-RELATED 5, chloroplastic-like
TCONS_00016270	-0.266566	0.114901	-0.051317	#N/A	#N/A

TCONS_00030605	-0.266256	1.31881	-0.278344	BGIOSGA016493	XP_006661608.1 probable NAD(P)H-dependent oxidoreductase 2
TCONS_00002343	-0.266165	-0.607203	0.493675	BGIOSGA004984	XP_015690666.1PREDICTED: uncharacterized protein LOC102710354, partial
TCONS_00021683	-0.265891	-1.90645	-1.59811	BGIOSGA037144	XP_004964753.2F-box protein At5g67140 isoform X1
TCONS_00000280	-0.265801	-0.532167	0.0655614	BGIOSGA008965	XP_003569202.1O-fucosyltransferase 3 isoform X1
TCONS_00010993	-0.26573	0.0356949	-0.6063	BGIOSGA025427	XP_017696773.1 mitotic checkpoint protein BUB3.3
TCONS_00037578	-0.265729	-0.622408	-0.662427	BGIOSGA035781	XP_015698645.1 ATP-dependent RNA helicase DEAH12, chloroplastic
TCONS_00024275	-0.265561	0.301056	0.359752	BGIOSGA026320	XP_006658922.2 WD repeat-containing protein 55
TCONS_00009421	-0.26529	0.669166	0.00643091	BGIOSGA011972	XP_006649494.1 elongation factor 1-alpha
TCONS_00006761	-0.265114	-0.0863254	-0.0194424	BGIOSGA035659	XP_006647802.1 ADP,ATP carrier protein, mitochondrial
TCONS_00015199	-0.26493	-0.392231	0.196872	BGIOSGA017379	XP_015692209.1 probable serine/threonine-protein kinase At5g41260
TCONS_00015080	-0.264883	-0.45831	-1.53234	BGIOSGA017085	XP_015692168.1 protein TIFY 3-like isoform X2
TCONS_00017635	-0.264777	-0.0507537	0.43474	BGIOSGA023344	XP_006654300.1 ferrochelatase-2, chloroplastic
TCONS_00008284	-0.264516	-1.47959	-1.33845	BGIOSGA025243	XP_006647436.1 serine/threonine-protein kinase Nek6
TCONS_00002642	-0.264403	-0.876984	0.572272	BGIOSGA009975	XP_010232888.1transcription factor MYB59 isoform X2
TCONS_00037374	-0.264401	0.753651	-0.223297	BGIOSGA004756	XP_004962744.1protein NPG1
TCONS_00027937	-0.264298	-0.943169	-0.629174	BGIOSGA000253	XP_004973329.1UDP-glucose 4-epimerase 2
TCONS_00021840	-0.264092	-0.988329	0.426375	BGIOSGA021678	XP_006655909.1 RNA-binding protein 5
TCONS_00000092	-0.264039	1.3621	0.417944	BGIOSGA002661	XP_015688076.1 fruit protein pKIWI502-like
TCONS_00002172	-0.263962	0.779488	-1.43934	BGIOSGA037749	XP_006645016.1PREDICTED: uncharacterized protein LOC102711654
TCONS_00013132	-0.263948	0.830527	0.359399	BGIOSGA029345	XP_006650551.1 ATP-dependent RNA helicase A-like
TCONS_00013513	-0.263738	-2.36706	-0.331642	#N/A	#N/A
TCONS_00035907	-0.263738	-0.367061	-1.9166	#N/A	#N/A
TCONS_00004218	-0.263267	-1.43623	-0.386307	BGIOSGA019297	XP_008655107.1protein binding protein isoform X1
TCONS_00020420	-0.263088	-0.0614465	0.291621	BGIOSGA022606	XP_015693526.1 ribosomal RNA small subunit methyltransferase G
TCONS_00003588	-0.262968	0.36008	1.00492	BGIOSGA001615	XP_006644180.1 guanine nucleotide-binding protein-like NSN1
TCONS_00023939	-0.262968	0.216984	0.88886	BGIOSGA025971	XP_014752830.1protein N-lysine methyltransferase METTL21A
TCONS_00030826	-0.262897	0.176449	0.168561	BGIOSGA017709	XP_003573835.1receptor-like serine/threonine-protein kinase At2g45590
TCONS_00016522	-0.262883	-0.860778	0.301677	BGIOSGA013408	XP_006652728.1 uncharacterized membrane protein At4g09580-like
TCONS_00030419	-0.262694	0.125379	-1.49231	BGIOSGA029383	XP_015696434.1 protein STAY-GREEN, chloroplastic-like
TCONS_00000738	-0.262677	0.224398	-0.671351	BGIOSGA019676	XP_006644111.1 protein FAM91A1
TCONS_00012786	-0.262645	1.38089	0.823066	BGIOSGA035855	XP_015690721.1 protein LOL3
TCONS_00030156	-0.262417	1.1545	-0.0136711	BGIOSGA029646	XP_015696373.1 dipeptidyl aminopeptidase BI
TCONS_00019782	-0.262265	-0.214645	-0.0900412	BGIOSGA017577	XP_006654775.1 golgin candidate 5
TCONS_00013279	-0.262134	-2.59751	-1.27989	BGIOSGA013598	XP_004981515.1patatin-like protein 3
TCONS_00036171	-0.262092	-0.0576401	0.14698	BGIOSGA034322	XP_003575725.1lysine-specific demethylase 6B
TCONS_00029535	-0.262043	0.316662	0.249963	BGIOSGA026086	XP_006660993.2 protein HIRA
TCONS_00023353	-0.262027	-1.14294	0.260917	#N/A	#N/A
TCONS_00003522	-0.262015	-0.00883421	-0.670568	BGIOSGA015573	XP_006645881.1 ABC transporter C family member 4-like
TCONS_00025957	-0.261812	0.825483	-0.864196	BGIOSGA011089	XP_006659155.1 topless-related protein 1-like
TCONS_00001055	-0.261633	0.33049	-0.740065	BGIOSGA031396	XP_003565794.1transcription factor MYBS1
TCONS_00018240	-0.261492	-3.31354	-1.10298	BGIOSGA004364	XP_006654733.1 myb-related protein Hv33-like
TCONS_00030789	-0.261333	-0.954312	-0.757711	BGIOSGA032597	XP_006651590.1 GPI ethanolamine phosphate transferase 3 isoform X1
TCONS_00004133	-0.261304	0.71997	0.59067	BGIOSGA001060	XP_006644490.1 persulfide dioxygenase ETHE1 homolog, mitochondrial
TCONS_00014925	-0.261202	0.411323	-0.0374114	BGIOSGA006962	XP_006658699.1 brefeldin A-inhibited guanine nucleotide-exchange protein 5
TCONS_00001920	-0.261005	-0.398611	-0.572363	BGIOSGA004556	XP_004970170.1uncharacterized protein At1g27050
TCONS_00021563	-0.260989	0.411008	-0.262098	BGIOSGA001316	XP_006655762.1 hypersensitive-induced response protein 4
TCONS_00004117	-0.260984	-0.759596	-0.104249	BGIOSGA001077	XP_006644472.1 1,4-dihydroxy-2-naphthoyl-CoA synthase, peroxisomal
TCONS_00006387	-0.26078	-1.04992	-0.276253	BGIOSGA016624	XP_015689678.1 uncharacterized membrane protein At3g27390 isoform X1
TCONS_00020529	-0.260456	-0.563704	-0.430724	BGIOSGA024796	XP_006665105.2 calcium/calmodulin-regulated receptor-like kinase 2
TCONS_00016791	-0.260109	#NA	-1.92557	BGIOSGA013065	XP_009394490.1 V-type proton ATPase subunit e1-like
TCONS_00017525	-0.260104	-1.35434	-2.59343	BGIOSGA036743	XP_004962480.1protein LURP-one-related 8
TCONS_00023097	-0.260083	-0.974452	-1.58232	BGIOSGA009712	XP_006657435.1 reticulon-like protein B8
TCONS_00002910	-0.259959	-0.882337	0.29951	BGIOSGA010536	XP_003569192.1uncharacterized protein LOC100828088
TCONS_00009968	-0.259948	-2.31549	0.173288	BGIOSGA004234	XP_004984553.1F-box protein At5g46170
TCONS_00001423	-0.2598	-1.38942	-0.147966	BGIOSGA024540	XP_015698857.1 glucose-1-phosphate adenylyltransferase large subunit 1, chloroplastic/amyloplastic-like
TCONS_00010798	-0.25973	0.542217	-0.10736	BGIOSGA034231	XP_004982024.1putative F-box protein At1g65770
TCONS_00029322	-0.259723	-0.184685	0.019923	BGIOSGA016301	XP_006660822.1PREDICTED: uncharacterized protein At2g33490-like
TCONS_00011805	-0.259663	0.462118	0.103147	BGIOSGA031921	XP_015690996.1 3-ketoacyl-CoA synthase 6-like

TCONS_00019587	-0.259628	0.269069	0.35805	BGIOSGA017771	XP_006654609.1 H/ACA ribonucleoprotein complex non-core subunit NAF1-like
TCONS_00017175	-0.259489	-0.441599	0.0232873	BGIOSGA019220	XP_015688736.1PREDICTED: uncharacterized protein LOC102721967 isoform X2
TCONS_00016151	-0.259489	-1.40918	0.0846878	BGIOSGA035126	XP_015691805.1 protein NRT1/ PTR FAMILY 4.5-like
TCONS_00001004	-0.259329	-0.447587	-0.386261	BGIOSGA002152	XP_010237340.1ribosome quality control complex subunit 2 isoform X2
TCONS_00009832	-0.259234	-1.33454	-0.161056	BGIOSGA031780	XP_006649900.1 beta-taxilin isoform X2
TCONS_00003729	-0.259222	-0.316404	-0.642955	BGIOSGA011542	XP_006645971.2 CBL-interacting protein kinase 8
TCONS_00024181	-0.259128	-1.22906	-1.27235	BGIOSGA020919	XP_006658013.1 CBL-interacting protein kinase 21
TCONS_00006425	-0.25912	-1.0274	-1.35265	BGIOSGA014811	XP_006647480.1 B-box zinc finger protein 25-like
TCONS_00030346	-0.258984	-1.73174	0.0282962	BGIOSGA007296	XP_004976560.1putative FBD-associated F-box protein At1g61330
TCONS_00013786	-0.258984	1.35572	0.331365	BGIOSGA015589	XP_003581017.1UDP-glycosyltransferase 79 isoform X1
TCONS_00003157	-0.258859	-0.215322	0.318345	BGIOSGA030346	XP_015699234.1 cyclin-A1-1-like
TCONS_00013051	-0.258852	1.29543	0.107606	BGIOSGA030200	XP_006650487.1 coatomer subunit alpha-3 isoform X1
TCONS_00023780	-0.2588	-0.938423	0.315642	BGIOSGA025818	XP_006657767.1 cytochrome P450 716B1-like
TCONS_00017366	-0.258747	0.457331	0.197934	BGIOSGA019431	XP_006654166.1 ER membrane protein complex subunit 1
TCONS_00011599	-0.258671	0.551427	1.28218	BGIOSGA015820	XP_004985631.1YTH domain-containing family protein 2 isoform X3
TCONS_00029804	-0.258667	-0.278351	-1.68345	BGIOSGA027094	XP_006660529.1 GDP-mannose transporter GONST4-like
TCONS_00021800	-0.258609	-0.512016	0.109131	BGIOSGA021714	XP_006655887.1 phosphopantothenoylcysteine decarboxylase
TCONS_00001758	-0.258164	1.43382	-0.930389	BGIOSGA028952	XP_015689810.1 protein Mpv17
TCONS_00010882	-0.257874	-0.423279	0.828889	BGIOSGA028838	XP_006651772.1 DEAD-box ATP-dependent RNA helicase 16
TCONS_00003120	-0.257667	-2.67451	-0.170385	BGIOSGA012010	XP_002454916.1uncharacterized protein LOC8079483
TCONS_00001048	-0.257454	-0.688986	0.198039	BGIOSGA003655	NP_001147064.1heat shock protein binding protein
TCONS_00011987	-0.257044	-0.888324	0.092927	BGIOSGA011027	XP_006649830.2 serine/arginine repetitive matrix protein 1
TCONS_00017397	-0.256968	-0.745709	-0.300824	BGIOSGA031001	NP_001141460.1triose phosphate/phosphate translocator
TCONS_00002737	-0.256538	-1.66056	-1.11069	BGIOSGA002477	XP_006643706.1 Bowman-Birk type bran trypsin inhibitor-like
TCONS_00027177	-0.256445	-0.0780742	0.291703	BGIOSGA027822	XP_003573239.1probable E3 ubiquitin ligase SUD1
TCONS_00025063	-0.256331	-0.206555	-0.0217345	BGIOSGA001275	XP_015694486.1 ubiquitin-related modifier 1 homolog isoform X1
TCONS_00019539	-0.256323	-0.777078	-0.215833	BGIOSGA020163	XP_006654574.1 F-box protein At4g18380-like
TCONS_00002108	-0.256295	-0.456049	-0.877931	BGIOSGA029411	XP_006644951.1 BAG family molecular chaperone regulator 4
TCONS_00021004	-0.256096	-0.943909	0.409331	BGIOSGA023235	XP_006657118.2 flocculation protein FLO11
TCONS_00037444	-0.256027	-0.776604	0.985743	BGIOSGA008617	XP_004963031.1adenine phosphoribosyltransferase 1
TCONS_00003836	-0.255959	-0.752829	0.439125	BGIOSGA005794	XP_006646018.2 IQ domain-containing protein IQM1
TCONS_00005909	-0.255839	1.41142	1.17975	BGIOSGA038519	XP_015688567.1PREDICTED: uncharacterized protein LOC102701059
TCONS_00025935	-0.255612	-1.22389	-1.36348	BGIOSGA036360	XP_006659844.1 oxysterol-binding protein-related protein 2A-like
TCONS_00002921	-0.255249	#NA	-0.93761	BGIOSGA037754	NP_001144755.2uncharacterized protein LOC100277806 precursor
TCONS_00013038	-0.255048	0.129075	-0.10357	BGIOSGA026216	XP_021307245.1probable transcription factor At3g04930
TCONS_00034180	-0.25503	-1.90988	-1.61239	BGIOSGA036054	XP_006663750.1 mannan endo-1,4-beta-mannosidase 8-like
TCONS_00004162	-0.254951	-0.396873	-0.428066	BGIOSGA007864	XP_006644507.1 serine/threonine-protein kinase EDR1
TCONS_00029699	-0.25471	-0.411821	-0.628331	BGIOSGA017207	XP_020396114.1putative phototropic-resoponsive NPH3 family protein isoform X1
TCONS_00011273	-0.254665	0.243605	0.912939	BGIOSGA029530	XP_006651988.1 galactokinase
TCONS_00024622	-0.254515	-1.26921	-2.08858	BGIOSGA024543	XP_006658306.1 indole-3-acetaldehyde oxidase
TCONS_00008297	-0.254115	-0.697326	-0.695098	BGIOSGA014880	XP_006647448.1 beta-1,4-mannosyl-glycoprotein 4-beta-N-acetylglucosaminyltransferase-like
TCONS_00037540	-0.254039	0.6511	0.544023	BGIOSGA013109	XP_004982319.1translation initiation factor eIF-2B subunit delta isoform X2
TCONS_00024890	-0.253898	-0.255272	-0.608668	BGIOSGA024492	XP_006657604.1 phosphatidylinositol N-acetylglucosaminyltransferase gpi3 subunit
TCONS_00002275	-0.253683	0.423393	-0.273307	BGIOSGA018019	XP_015690549.1 mitotic spindle checkpoint protein MAD1-like
TCONS_00036404	-0.253679	0.804844	1.13753	BGIOSGA002869	XP_006664731.1 WD repeat-containing protein 75
TCONS_00020074	-0.253672	-2.37845	-0.941512	BGIOSGA022254	XP_015694298.1 WASH complex subunit 7-like isoform X1
TCONS_00004896	-0.253337	0.393717	0.068593	BGIOSGA000325	XP_006645171.1 sister chromatid cohesion 1 protein 4
TCONS_00003123	-0.253195	-0.264041	0.0670473	BGIOSGA033196	XP_015699250.1 GDSL esterase/lipase At5g45910-like isoform X1
TCONS_00014805	-0.253132	0.386417	-1.13796	BGIOSGA016975	XP_006652681.1 beta-carotene hydroxylase 2, chloroplastic-like
TCONS_00030691	-0.253114	-1.73375	-0.0999994	BGIOSGA017125	XP_006661627.2 alpha-mannosidase-like isoform X2
TCONS_00016388	-0.253038	0.00599506	-0.435234	BGIOSGA009830	XP_006653659.1 BEACH domain-containing protein C2

transcriptome

TCONS_00025330	-0.252874	-1.76924	-0.458665	BGIOSGA024097	XP_006658689.1 putative PAP-specific phosphatase, mitochondrial
TCONS_00027401	-0.252816	-0.0193258	-0.288334	BGIOSGA015307	XP_008654255.1putative RING/U-box superfamily protein isoform X1
TCONS_00036255	-0.25279	0.101304	-0.580563	BGIOSGA037614	XP_010237137.1diaminopimelate epimerase, chloroplastic
TCONS_00006662	-0.252732	1.01917	-0.0216973	BGIOSGA008831	XP_006660416.1 aspartate--tRNA ligase 2, cytoplasmic-like
TCONS_00019305	-0.252616	0.249803	-1.44896	BGIOSGA009203	XP_006655332.1 NAC domain-containing protein 72-like
TCONS_00002026	-0.252589	-0.371719	-0.449027	BGIOSGA040169	XP_014754758.1L-2-hydroxyglutarate dehydrogenase, mitochondrial
TCONS_00002982	-0.252294	-1.9398	0.656969	BGIOSGA002236	XP_004968480.1protein TWIN LOV 1 isoform X1
TCONS_00017942	-0.252293	-0.0840464	-0.284106	BGIOSGA028845	XP_015693062.1 60S ribosomal protein L36-3-like
TCONS_00005381	-0.252279	-0.525237	-0.187357	BGIOSGA020575	XP_006648296.2PREDICTED: uncharacterized protein LOC102701420, partial
TCONS_00033900	-0.252198	-0.869354	-3.07273	BGIOSGA023345	XP_002437334.1premnaspirodiene oxygenase
TCONS_00023938	-0.252193	-0.0744465	0.159088	BGIOSGA025970	XP_006658709.1PREDICTED: uncharacterized protein LOC102723071 isoform X1
TCONS_00001945	-0.251939	-0.0600477	-1.13838	BGIOSGA009810	XP_006644813.1 NAD(P)H dehydrogenase (quinone) FQR1-like isoform X1
TCONS_00008255	-0.251938	-0.938769	-0.634636	BGIOSGA006173	XP_015689620.1PREDICTED: uncharacterized protein LOC102708588 isoform X1
TCONS_00001594	-0.251898	1.03293	-0.118706	BGIOSGA017608	XP_006644561.1 guanine nucleotide-binding protein subunit beta-like protein A
TCONS_00031107	-0.25176	0.467912	0.283579	#N/A	#N/A
TCONS_00024328	-0.251749	-1.38048	-0.361297	BGIOSGA019120	XP_015698300.1 UPF0481 protein At3g47200-like
TCONS_00027390	-0.251666	0.48121	-0.382708	BGIOSGA016673	XP_003573458.1zinc finger CCCH domain-containing protein 56
TCONS_00005206	-0.251664	-0.36908	0.0271958	BGIOSGA021508	XP_015699015.1 E3 ubiquitin-protein ligase listerin isoform X1
TCONS_00006790	-0.251648	-2.07334	-0.451356	BGIOSGA012849	XP_003570464.2O-fucosyltransferase 34 isoform X2
TCONS_00032538	-0.251433	-0.696499	0.980696	BGIOSGA026632	XP_004982651.121 kDa protein
TCONS_00024179	-0.251317	-0.718114	-0.412932	BGIOSGA026217	XP_006658872.1 SWI/SNF-related matrix-associated actin-dependent regulator of chromatin subfamily A-like protein 1
TCONS_00031372	-0.251084	-0.648985	-0.161238	BGIOSGA033185	XP_006661893.1 G patch domain-containing protein 11-like
TCONS_00030505	-0.251015	-0.251289	-0.409297	BGIOSGA030813	XP_010504143.1 histone H4
TCONS_00031353	-0.250982	-0.755351	0.212907	BGIOSGA027679	NP_001278609.160S ribosomal protein L37
TCONS_00027238	-0.250765	-0.0252108	-0.448578	BGIOSGA002863	XP_004972482.1probable serine/threonine-protein kinase At1g54610
TCONS_00001557	-0.250729	-2.16761	0.444077	BGIOSGA002158	XP_006644509.2 probable LRR receptor-like serine/threonine-protein kinase MRH1
TCONS_00007870	-0.250438	-0.073214	0.38133	BGIOSGA006606	XP_004952337.1negative regulator of systemic acquired resistance SNI1 isoform X1
TCONS_00009120	-0.250367	-0.515692	0.483476	BGIOSGA011646	XP_006649219.2 DNA repair endonuclease UVH1
TCONS_00010721	-0.250337	-0.65995	-1.01402	BGIOSGA020279	XP_015690019.1 glycosyltransferase family protein 64 protein C5-like
TCONS_00000017	-0.250088	0.00983755	-0.164683	BGIOSGA027407	XP_015688092.1 protein CHROMATIN REMODELING 8
TCONS_00033048	-0.24997	1.31687	0.570042	BGIOSGA034883	XP_006663244.1 sec1 family domain-containing protein MIP3
TCONS_00002280	-0.249812	-0.341989	-0.271101	BGIOSGA036331	XP_015688257.1PREDICTED: uncharacterized protein LOC102718127 isoform X1
TCONS_00032786	-0.249623	-1.8973	-0.518607	BGIOSGA031322	XP_006662110.1 probable NADH dehydrogenase [ubiquinone] 1 alpha subcomplex subunit 12
TCONS_00016789	-0.249557	0.45651	0.204464	BGIOSGA024100	XP_006652953.1 probable serine/threonine-protein kinase abkC
TCONS_00011483	-0.24953	-0.517994	0.141653	BGIOSGA001476	XP_006649310.1 CBL-interacting protein kinase 9 isoform X1
TCONS_00000410	-0.249197	-0.341621	0.317657	BGIOSGA018122	XP_015692375.1 probable pre-mRNA-splicing factor ATP-dependent RNA helicase DEAH9
TCONS_00008376	-0.249171	0.0799161	-0.675243	BGIOSGA031634	XP_006647516.1PREDICTED: uncharacterized protein LOC102709809
TCONS_00008190	-0.249128	-0.977286	-1.17196	BGIOSGA015018	XP_015689481.1 putative 12-oxophytodienoate reductase 8 isoform X1
TCONS_00019894	-0.248826	-4.75758	-1.13696	BGIOSGA019365	XP_003565827.1TPR repeat-containing thioredoxin TTL1
TCONS_00035439	-0.248749	0.594946	-0.307815	BGIOSGA036849	XP_006663706.1 clathrin heavy chain 1-like
TCONS_00037002	-0.248671	-0.610129	-0.208713	BGIOSGA036372	XP_006663933.2 DNA-(apurinic or apyrimidinic site) lyase
TCONS_00010253	-0.248399	-4.66846	-3.38585	BGIOSGA021330	XP_015689927.1 horcolin-like
TCONS_00001494	-0.248377	-0.379305	-0.14022	BGIOSGA002118	XP_006644459.2 histone-lysine N-methyltransferase ATX5
TCONS_00023264	-0.248129	-0.610811	-1.72463	BGIOSGA020095	XP_002443182.1uncharacterized protein LOC8064065
TCONS_00009166	-0.247989	-1.32573	-0.464278	BGIOSGA018319	XP_015690693.1 DNA (cytosine-5)-methyltransferase DRM2-like
TCONS_00008716	-0.247904	0.719245	0.29415	BGIOSGA028492	XP_015689568.1 phosphatidylinositol/phosphatidylcholine transfer protein SFH9-like isoform X1
TCONS_00031734	-0.247891	-0.166699	0.115141	BGIOSGA028031	XP_006661606.1 transmembrane 9 superfamily member 3-like
TCONS_00004704	-0.247815	-0.305861	0.0262054	BGIOSGA019988	XP_003564587.1protein ESMERALDA 1 isoform X1
TCONS_00004536	-0.247684	-0.688295	-1.08594	BGIOSGA000679	XP_006644820.2 autophagy-related protein 18f-like
TCONS_00015761	-0.247626	-0.0458742	0.463263	BGIOSGA015219	XP_006652202.2 violaxanthin de-epoxidase, chloroplastic

TCONS_00017469	-0.247191	-0.43337	0.32962	BGIOSGA019541	XP_020403209.1uncharacterized protein LOC100277740 isoform X1
TCONS_00009018	-0.247149	0.387957	0.594875	BGIOSGA027055	XP_003572932.1leucine-rich repeat extensin-like protein 1
TCONS_00030566	-0.246772	0.247417	0.346593	BGIOSGA029238	XP_006661571.1 cleavage and polyadenylation specificity factor subunit 2
TCONS_00008538	-0.24673	-0.385808	-0.958846	BGIOSGA019048	XP_004953378.1actin-depolymerizing factor 1
TCONS_00029399	-0.246493	-0.237288	0.933642	BGIOSGA017628	XP_006660890.1 transcription initiation factor IIB
TCONS_00008135	-0.246361	0.139877	0.446351	BGIOSGA005419	XP_006647348.1 pyrophosphate-energized membrane proton pump 3
TCONS_00032413	-0.246235	-0.763674	-1.48136	BGIOSGA035060	XP_006661858.2 non-lysosomal glucosylceramidase-like isoform X1
TCONS_00001740	-0.246166	-0.155098	0.0227176	BGIOSGA029961	XP_006646277.1 ABC transporter G family member 38
TCONS_00004959	-0.245222	-0.936388	-0.34276	BGIOSGA015046	XP_006646603.1 probable UDP-N-acetylglucosamine--peptide N-acetylglucosaminyltransferase SEC
TCONS_00002060	-0.245122	-0.352369	0.629786	BGIOSGA012556	XP_006644904.1 2,3-bisphosphoglycerate-independent phosphoglycerate mutase
TCONS_00036048	-0.245064	0.912037	0.261354	BGIOSGA025038	XP_015698661.1 ubiquitin carboxyl-terminal hydrolase 12-like isoform X2
TCONS_00033741	-0.244923	-0.140195	-0.565855	BGIOSGA036010	XP_006663006.1 ADP-ribosylation factor-like protein 5
TCONS_00027736	-0.244914	-0.673467	-0.499294	BGIOSGA027268	XP_006660014.1PREDICTED: uncharacterized protein LOC102712285, partial
TCONS_00013247	-0.244625	-0.754448	-0.529921	BGIOSGA037458	XP_015690960.1 TSL-kinase interacting protein 1
TCONS_00021427	-0.244577	-1.36234	-0.385328	BGIOSGA023651	XP_006657372.1PREDICTED: uncharacterized protein LOC102703408
TCONS_00028141	-0.244378	-0.55386	-0.566048	BGIOSGA008850	XP_015696181.1 IAA-alanine resistance protein 1 isoform X1
TCONS_00020208	-0.244335	-0.438404	0.736654	BGIOSGA037984	XP_015694097.1 uncharacterized WD repeat-containing protein C2A9.03-like isoform X1
TCONS_00007159	-0.243975	-0.463894	-0.57014	BGIOSGA009345	XP_006648173.1 zinc finger CCCH domain-containing protein 19-like
TCONS_00010787	-0.243975	-0.196122	0.351227	BGIOSGA036360	XP_021302013.1oxysterol-binding protein-related protein 1C
TCONS_00028861	-0.243802	1.19598	1.20365	BGIOSGA030611	XP_024316004.1SNF1-related protein kinase regulatory subunit beta-3 isoform X2
TCONS_00019893	-0.24363	-0.794465	-0.47123	BGIOSGA011530	XP_006654852.1 probable protein phosphatase 2C 52
TCONS_00010261	-0.243569	0.669109	-0.101683	BGIOSGA031910	XP_006650178.1 40S ribosomal protein SA-like
TCONS_00015267	-0.243551	-0.868249	-0.525247	BGIOSGA015715	XP_004975002.1uncharacterized RNA-binding protein C17H9.04c
TCONS_00037092	-0.243546	0.490237	-0.430584	BGIOSGA035264	XP_006663994.2 protein transport protein Sec24-like CEF
TCONS_00013460	-0.243542	1.93267	3.52492	BGIOSGA012231	XP_006650830.1PREDICTED: uncharacterized protein LOC102717604
TCONS_00029342	-0.243419	-0.39371	-0.111473	BGIOSGA031090	XP_006661432.1 translation initiation factor IF-2 isoform X1
TCONS_00013294	-0.243406	0.751831	0.337178	BGIOSGA016678	NP_001149996.1expp1 protein precursor
TCONS_00025099	-0.243397	-0.884876	0.130622	BGIOSGA029381	XP_006657704.2 beta-ureidopropionase
TCONS_00007824	-0.243393	-0.158073	-0.242837	BGIOSGA011414	XP_006647185.1 E3 ubiquitin-protein ligase SINAT5
TCONS_00006315	-0.243387	-0.918119	-0.947983	BGIOSGA008480	XP_003575213.2protein ACTIVITY OF BC1 COMPLEX KINASE 8, chloroplastic
TCONS_00005358	-0.243317	0.271974	-0.00235405	BGIOSGA007469	XP_015689488.1PREDICTED: uncharacterized protein LOC102722366
TCONS_00036839	-0.243265	-1.64974	-0.270234	BGIOSGA036471	XP_015692950.1PREDICTED: uncharacterized protein LOC107304242
TCONS_00000403	-0.243189	-0.0183137	-0.0504528	BGIOSGA034231	XP_006645621.2 dol-P-Man:Man(6)GlcNAc(2)-PP-Dol alpha-1,2-mannosyltransferase
TCONS_00024491	-0.243004	0.505866	0.27618	BGIOSGA024905	XP_015695299.1PREDICTED: uncharacterized protein LOC102719784 isoform X1
TCONS_00033553	-0.24296	-1.95087	-0.673093	BGIOSGA035343	XP_015697799.1PREDICTED: uncharacterized protein LOC102699349
TCONS_00009736	-0.242846	0.715275	0.0926063	BGIOSGA023312	XP_006651241.1PREDICTED: uncharacterized protein LOC102704044
TCONS_00033289	-0.242814	-0.867225	0.159363	BGIOSGA008042	XP_015696174.1 pentatricopeptide repeat-containing protein At2g37320-like
TCONS_00035009	-0.242796	0.194885	0.533681	BGIOSGA033848	XP_006663508.2 protein AUXIN RESPONSE 4
TCONS_00000657	-0.242771	-0.318857	0.0388387	BGIOSGA019751	XP_006644051.2PREDICTED: uncharacterized protein LOC102708842
TCONS_00005188	-0.242699	0.250618	-0.300697	BGIOSGA000016	XP_006646741.1 probable dual-specificity RNA methyltransferase RlmN
TCONS_00016004	-0.242695	-2.20132	-0.893982	BGIOSGA006945	XP_015692116.1 chlorophyll a-b binding protein CP24, chloroplastic
TCONS_00022274	-0.24239	-0.331059	-0.357714	BGIOSGA033285	XP_006656094.1 serine/threonine-protein kinase fray2 isoform X1
TCONS_00034101	-0.242327	0.599557	0.910254	BGIOSGA036958	XP_004978278.1scarecrow-like protein 33
TCONS_00017603	-0.241831	0.268768	-0.0443759	BGIOSGA020949	XP_006654286.1 ERBB-3 BINDING PROTEIN 1
TCONS_00027488	-0.241688	1.2037	1.18969	BGIOSGA011492	XP_015695984.1 putative aconitate hydratase, cytoplasmic
TCONS_00002235	-0.241577	0.526249	-0.726088	BGIOSGA017981	XP_002456695.1protein YIPF6 homolog
TCONS_00007889	-0.241446	-1.39159	-0.97226	BGIOSGA001229	XP_006648597.1 ABC transporter G family member 40 isoform X1
TCONS_00003013	-0.241446	-1.39159	-1.97226	BGIOSGA017605	XP_019707609.1PREDICTED: uncharacterized protein LOC109506141
TCONS_00026794	-0.241446	#NA	-0.97226	BGIOSGA028855	XP_002444531.2SH3 domain-containing protein C23A1.17
TCONS_00014795	-0.241242	0.0260001	0.103689	BGIOSGA029790	XP_002451500.1uncharacterized protein LOC8057532
TCONS_00021920	-0.241208	0.787778	-0.0814008	BGIOSGA036398	XP_015694362.1PREDICTED: uncharacterized protein At5g39570 isoform X2
TCONS_00027798	-0.241031	-0.392059	0.348614	#N/A	#N/A

TCONS_00006993	-0.240819	1.47736	0.27612	BGIOSGA030807	XP_006648027.2 glutamate receptor 2.7-like
TCONS_00009134	-0.240816	-2.8941	-1.26188	BGIOSGA001825	XP_006649227.1 cortical cell-delineating protein-like
TCONS_00004893	-0.240739	-0.599636	-0.72919	BGIOSGA000327	XP_004970898.1ribonuclease 2
TCONS_00036491	-0.24063	-0.807548	0.262713	BGIOSGA015169	XP_006664234.1PREDICTED: uncharacterized protein LOC102710832
TCONS_00008715	-0.24063	-0.618071	-0.494334	BGIOSGA030041	XP_006647809.1 GPI-anchored protein LORELEI-like
TCONS_00017177	-0.240614	0.467003	0.202685	BGIOSGA008077	XP_006654075.1 E3 ubiquitin-protein ligase UPL6
TCONS_00036543	-0.24058	0.470334	-0.146294	BGIOSGA019154	XP_012454889.1 regulatory-associated protein of TOR 1-like isoform X2
TCONS_00020570	-0.240454	0.670972	0.404774	BGIOSGA022748	XP_006656912.1 proteasome activator subunit 4
TCONS_00006875	-0.240436	0.192232	-0.389855	#N/A	#N/A
TCONS_00026367	-0.240377	-0.435051	-0.131675	BGIOSGA014766	XP_006659302.1 polyadenylate-binding protein 2-like
TCONS_00008287	-0.24034	1.00334	0.847188	BGIOSGA006143	XP_006647438.1 ribosome biogenesis protein BRX1-like
TCONS_00016543	-0.240324	0.391963	-0.149724	BGIOSGA005789	XP_006652749.2PREDICTED: uncharacterized protein At1g51745-like isoform X1
TCONS_00016663	-0.240172	0.286939	0.463074	BGIOSGA028099	XP_015692325.1 zinc finger BED domain-containing protein RICESLEEPER 1-like isoform X1
TCONS_00006444	-0.239929	-0.994564	-1.3155	BGIOSGA038732	XP_003575337.1hydroxycinnamoyltransferase 2
TCONS_00026881	-0.239881	0.0197879	0.44261	BGIOSGA028937	XP_006659547.1 peroxisome biogenesis protein 5 isoform X1
TCONS_00003543	-0.239875	1.13019	1.11123	BGIOSGA001662	XP_006644166.1 dephospho-CoA kinase
TCONS_00011689	-0.239813	0.609414	0.817008	BGIOSGA011320	XP_006649519.1 origin of replication complex subunit 2
TCONS_00029218	-0.239695	-0.877306	0.130436	BGIOSGA030960	XP_003578287.1protein MICROTUBULE BINDING PROTEIN 2C
TCONS_00011585	-0.239519	1.51311	0.0228642	BGIOSGA011875	XP_015690314.1 ribosomal large subunit pseudouridine synthase B isoform X1
TCONS_00009486	-0.239519	-0.393779	1.3921	BGIOSGA028983	XP_015695743.1 geranylgeranyl transferase type-2 subunit beta-like
TCONS_00035696	-0.239464	-0.952819	-0.520148	BGIOSGA037111	XP_006663884.1 small RNA degrading nuclease 5
TCONS_00003989	-0.239427	0.0991269	0.0616139	BGIOSGA025210	XP_006644372.1 N-alpha-acetyltransferase 16, NatA auxiliary subunit
TCONS_00001993	-0.239347	-2.97894	-2.1475	BGIOSGA017801	XP_004970278.1basic leucine zipper 23
TCONS_00033383	-0.239242	0.257989	0.153356	BGIOSGA020149	XP_003562954.160S ribosomal protein L38
TCONS_00017242	-0.238936	2.08613	1.19675	BGIOSGA019284	XP_004979658.1acyl-coenzyme A oxidase 2, peroxisomal
TCONS_00019368	-0.238873	-0.854271	-0.646085	BGIOSGA004865	XP_006655360.1 fructose-1,6-bisphosphatase, cytosolic-like
TCONS_00019411	-0.238849	-1.39456	0.828516	BGIOSGA011050	XP_006654492.1PREDICTED: uncharacterized protein LOC102707588
TCONS_00037379	-0.238849	-0.809597	-0.393877	BGIOSGA018911	XP_006664651.2 UNC93-like protein 3 isoform X1
TCONS_00033784	-0.238838	-0.535926	-0.475597	BGIOSGA035638	XP_006663025.1PREDICTED: uncharacterized protein LOC102718497
TCONS_00018328	-0.238826	-0.633469	-0.286734	BGIOSGA014371	XP_006655599.1 sugar transporter ERD6-like 6
TCONS_00029151	-0.238788	-0.171863	-0.744497	BGIOSGA000790	XP_015696881.1 heat stress transcription factor B-1
TCONS_00005303	-0.238533	0.0525273	0.418905	BGIOSGA025827	XP_006657769.1 succinate dehydrogenase subunit 3-2, mitochondrial
TCONS_00008516	-0.23835	-3.83537	-0.734112	BGIOSGA039122	XP_006647625.1 putative kinase-like protein TMKL1
TCONS_00016909	-0.23806	0.092214	-0.337293	BGIOSGA001890	XP_006653058.1 receptor protein kinase TMK1-like
TCONS_00034345	-0.237908	0.0650707	-1.13346	BGIOSGA034831	XP_020408426.1BEL1-like homeodomain protein 1 isoform X1
TCONS_00026951	-0.237788	-1.39581	0.0184645	BGIOSGA027887	XP_006660281.1 LOB domain-containing protein 24-like
TCONS_00004269	-0.237749	-1.89387	-0.705057	BGIOSGA016744	XP_015699083.1 receptor-like serine/threonine-protein kinase ALE2 isoform X11
TCONS_00026003	-0.237657	-0.465879	-1.08448	BGIOSGA021625	XP_006659883.1 somatic embryogenesis receptor kinase 2-like
TCONS_00004288	-0.23763	-1.22539	-0.393087	BGIOSGA000919	XP_006646262.1 branchpoint-bridging protein
TCONS_00033003	-0.237414	-0.152615	0.151241	BGIOSGA034839	XP_006662748.1 cyclin-T1-3
TCONS_00005472	-0.237413	-0.354081	0.867405	BGIOSGA022163	XP_010233840.1uncharacterized protein LOC100827700
TCONS_00004287	-0.237239	-0.612196	0.273408	BGIOSGA003048	XP_006644616.1 ras-related protein Rab7
TCONS_00030638	-0.237239	-0.00415011	-0.282492	BGIOSGA032467	XP_004975169.1F-box protein At5g49610
TCONS_00008754	-0.237098	-0.449504	-0.467037	BGIOSGA028838	XP_014661075.1zinc finger protein CONSTANS-LIKE 16 isoform X1
TCONS_00033182	-0.237088	0.401793	-0.179462	BGIOSGA010802	XP_002453584.160S ribosomal protein L10-1
TCONS_00018057	-0.237038	0.439488	0.764271	BGIOSGA013123	XP_006654591.1 probable calcium-transporting ATPase 6, plasma membrane-type
TCONS_00024015	-0.236926	0.0143964	0.44085	BGIOSGA017583	XP_006657904.1 WD repeat-containing protein 82-B
TCONS_00026768	-0.236674	-5.83748	-1.5318	BGIOSGA028830	XP_015696218.1PREDICTED: uncharacterized protein LOC102719792
TCONS_00021173	-0.236361	0.25455	-0.79251	BGIOSGA004903	XP_002438777.1protein GLUTAMINE DUMPER 5
TCONS_00012822	-0.236289	0.735177	0.167945	BGIOSGA035823	XP_015690690.1 serine/threonine-protein phosphatase BSL2 homolog
TCONS_00003388	-0.236284	-0.321902	-1.14897	BGIOSGA001820	XP_006644103.1 ATP-citrate synthase beta chain protein 1
TCONS_00006623	-0.236246	0.347173	-0.0455626	BGIOSGA022584	XP_004976512.1protein indeterminate-domain 5, chloroplastic
TCONS_00005121	-0.236211	-0.524054	-0.815371	BGIOSGA015952	XP_004971312.1protein O-linked-mannose beta-1,4-N-acetylglucosaminyltransferase 2
TCONS_00001076	-0.23613	0.0444822	-0.632678	BGIOSGA003695	XP_006645966.1 condensin complex subunit 2

TCONS_00026640	-0.235977	-0.0980681	0.0590244	BGIOSGA028708	XP_015695608.1 F-box protein SKIP28
TCONS_00023518	-0.2359	-0.594401	-0.417741	BGIOSGA011101	XP_006664839.1 synaptotagmin-5
TCONS_00023141	-0.235682	0.409003	0.598097	BGIOSGA025176	XP_006657457.1 putative deoxyribonuclease TATDN1
TCONS_00023267	-0.235575	-0.779572	-0.624565	BGIOSGA025315	XP_003566629.1bifunctional phosphatase IMPL2, chloroplastic isoform X2
TCONS_00027595	-0.23547	-2.39861	0.334527	BGIOSGA034965	XP_009343889.1 BTB/POZ and MATH domain-containing protein 3-like isoform X1
TCONS_00021481	-0.235459	-0.0459035	-0.236596	BGIOSGA022070	XP_015694074.1PREDICTED: uncharacterized protein LOC102705438
TCONS_00011525	-0.235412	0.184767	-0.494514	BGIOSGA011493	XP_015689756.1 pre-mRNA-splicing factor cwf23
TCONS_00005446	-0.235333	-0.375745	0.0509088	BGIOSGA033486	XP_008353740.1 ketol-acid reductoisomerase, chloroplastic-like
TCONS_00034470	-0.235316	-0.465792	0.371368	BGIOSGA034347	XP_006662806.1 zinc finger CCHC domain-containing protein 10-like
TCONS_00037372	-0.235235	0.352613	0.369249	BGIOSGA025511	XP_006664648.1 nuclear export mediator factor NEMF
TCONS_00031404	-0.235164	-0.816044	-0.284165	BGIOSGA027116	XP_015697040.1 CBS domain-containing protein CBSX5-like
TCONS_00035110	-0.234968	-0.814267	0.0113327	BGIOSGA033702	XP_015697941.1 GDP-mannose 3,5-epimerase 2 isoform X2
TCONS_00023246	-0.234884	-1.22569	-0.730713	BGIOSGA022440	XP_015694832.1 AUGMIN subunit 3-like isoform X1
TCONS_00026166	-0.234849	-1.57898	1.09805	#N/A	#N/A
TCONS_00013300	-0.234642	-5.29257	-2.30759	BGIOSGA009681	XP_002463768.1fasciclin-like arabinogalactan protein 16
TCONS_00025189	-0.234501	-0.663544	-0.322078	BGIOSGA025792	XP_015694650.1 protein RRP6-like 3
TCONS_00026943	-0.234358	-0.569276	-0.653953	BGIOSGA019689	XP_006659584.2 squamosa promoter-binding-like protein 15
TCONS_00020366	-0.234325	-1.03903	-0.29639	BGIOSGA005585	XP_006655919.1 polyadenylate-binding protein 2-like
TCONS_00032865	-0.234153	-1.49335	-0.99072	BGIOSGA017997	XP_006658042.1 pre-mRNA-splicing factor SYF1
TCONS_00010775	-0.234151	-0.843389	0.0560246	BGIOSGA005339	XP_006650453.1 phosphatidylinositol 4-phosphate 5-kinase 1 isoform X1
TCONS_00010802	-0.234146	1.26555	0.404469	BGIOSGA013426	XP_006650481.1PREDICTED: uncharacterized protein LOC102710004
TCONS_00017690	-0.234012	0.228121	-0.0432021	BGIOSGA003242	XP_006654330.2PREDICTED: uncharacterized protein LOC102708337
TCONS_00018317	-0.233954	-0.160864	-0.0760061	BGIOSGA016586	XP_006654796.1 urease accessory protein G
TCONS_00003704	-0.233739	0.573934	-0.151119	BGIOSGA008525	XP_006644339.1 serine/threonine-protein phosphatase 7 long form homolog
TCONS_00012657	-0.233665	-1.26335	-3.31388	BGIOSGA010811	XP_003568127.1probable xyloglucan glycosyltransferase 7
TCONS_00000400	-0.233615	-0.196515	-0.326091	BGIOSGA002977	XP_006643893.2 SCAR-like protein 2
TCONS_00020495	-0.233613	0.636786	0.495584	BGIOSGA022665	XP_006655991.2 autophagy-related protein 2
TCONS_00011850	-0.233347	-0.473578	-0.224558	BGIOSGA009904	XP_006649696.1 protein NRT1/ PTR FAMILY 8.3-like isoform X1
TCONS_00023191	-0.233318	0.971319	0.0992934	BGIOSGA009655	XP_015695274.1 40S ribosomal protein S18
TCONS_00025567	-0.233085	-0.519829	-0.398559	BGIOSGA007810	XP_006657992.1 putative receptor-like protein kinase At4g00960
TCONS_00003488	-0.23306	-0.823867	-0.625149	BGIOSGA023071	XP_015688128.1PREDICTED: uncharacterized protein LOC102712596
TCONS_00030543	-0.233024	-0.811638	0.206935	BGIOSGA029261	XP_006365432.1 peroxisome biogenesis protein 1
TCONS_00027909	-0.232936	0.405588	-0.671859	BGIOSGA027088	XP_006659320.1 putative methyltransferase NSUN6
TCONS_00009952	-0.232844	0.434764	0.0637829	BGIOSGA011417	XP_006649989.1PREDICTED: uncharacterized protein LOC102707011 isoform X1
TCONS_00025530	-0.232704	0.896684	0.304296	BGIOSGA011331	XP_006657956.2 40S ribosomal protein Sa-2-like
TCONS_00026658	-0.232669	-2.06107	-0.947151	BGIOSGA012745	XP_006659435.2 probable apyrase 6
TCONS_00029633	-0.232545	-0.176595	0.062449	BGIOSGA030188	XP_006660433.1 WPP domain-associated protein-like
TCONS_00016623	-0.232426	-0.0794173	-0.225588	BGIOSGA014340	XP_003580553.1peroxisome biogenesis protein 6 isoform X1
TCONS_00010930	-0.2324	0.950478	-0.0845437	BGIOSGA012085	XP_004981818.1protein transport protein sec23-1
TCONS_00034878	-0.232115	-0.595447	0.911044	BGIOSGA033453	XP_006654102.1 auxin-responsive protein IAA15
TCONS_00009274	-0.23184	-0.879633	-0.979928	BGIOSGA031707	XP_015691111.1 tetratricopeptide repeat protein 7A
TCONS_00001469	-0.231828	0.645313	0.472887	BGIOSGA035770	XP_006644437.1 WD repeat-containing protein 43
TCONS_00012635	-0.231814	-1.97653	-0.805662	BGIOSGA009004	XP_006652053.1PREDICTED: uncharacterized protein LOC102717235
TCONS_00004494	-0.231771	-0.358137	-1.76447	BGIOSGA000726	XP_006644789.1PREDICTED: uncharacterized protein LOC102716826
TCONS_00012668	-0.231533	0.831535	-0.607471	BGIOSGA017292	XP_006650286.1 UDP-arabinopyranose mutase 1
TCONS_00002326	-0.231343	-0.19042	-1.07368	BGIOSGA004968	XP_006645154.1 BTB/POZ and TAZ domain-containing protein 3
TCONS_00017484	-0.231301	0.214317	-0.617661	BGIOSGA019550	XP_006654234.1 transcription initiation factor IIA subunit 1-like
TCONS_00011377	-0.231163	0.700594	1.24911	BGIOSGA013991	XP_012698312.1transducin beta-like protein 2
TCONS_00023664	-0.231028	-0.584752	-0.179141	BGIOSGA033392	XP_006657692.1 NEP1-interacting protein 2-like isoform X2
TCONS_00036680	-0.231028	-1.2887	0.274449	BGIOSGA036632	XP_015698216.1 E3 ubiquitin-protein ligase EL5-like
TCONS_00019318	-0.230991	-0.314991	0.0682052	BGIOSGA004931	XP_010231359.1ubinuclein-1 isoform X3
TCONS_00036835	-0.230975	-0.48282	0.366737	BGIOSGA036474	XP_006664414.1 protein SUPPRESSOR OF GENE SILENCING 3 homolog

transcriptome

TCONS_00022200	-0.230435	0.168094	-0.0353331	BGIOSGA008021	XP_015694102.1 probable pre-mRNA-splicing factor ATP-dependent RNA helicase DEAH5 isoform X2
TCONS_00025989	-0.230389	-1.42845	-0.650581	BGIOSGA028064	XP_006659169.2 haloacid dehalogenase-like hydrolase domain-containing protein 3
TCONS_00031443	-0.230389	-2.40499	-3.00017	BGIOSGA033261	XP_021307167.1uncharacterized protein LOC8065863
TCONS_00024914	-0.230369	-0.762275	-0.892844	BGIOSGA024356	XP_024314303.1uncharacterized protein LOC104582301
TCONS_00024627	-0.230362	-0.861443	-0.546377	BGIOSGA005216	XP_015695327.1 MACPF domain-containing protein At4g24290-like
TCONS_00001743	-0.23027	-0.230346	0.113112	BGIOSGA004379	XP_006646279.1 ultraviolet-B receptor UVR8
TCONS_00011361	-0.230258	0.657873	0.391999	BGIOSGA022733	XP_006650926.1 outer envelope pore protein 24, chloroplastic
TCONS_00013271	-0.230174	-1.43914	1.29531	BGIOSGA009715	XP_015690095.1 ribosome-binding ATPase YchF
TCONS_00030186	-0.230085	0.00966539	-2.00092	BGIOSGA005769	XP_006660717.1 zinc-finger homeodomain protein 1
TCONS_00014384	-0.230027	-0.196667	-1.62438	BGIOSGA027913	XP_006652373.1 glyceraldehyde-3-phosphate dehydrogenase A, chloroplastic
TCONS_00028908	-0.229838	-4.40568	-1.19418	BGIOSGA023312	XP_006661179.1 RING-H2 finger protein ATL46-like
TCONS_00009771	-0.22981	0.593082	0.0580072	BGIOSGA012322	XP_006649839.1 oxysterol-binding protein-related protein 3C-like
TCONS_00003193	-0.229594	0.0329453	-0.140998	BGIOSGA002022	XP_006645699.1 probable folate-biopterin transporter 4 isoform X1
TCONS_00000623	-0.229491	-2.4475	-0.885266	BGIOSGA003206	XP_006644027.1PREDICTED: uncharacterized protein LOC102701863
TCONS_00013814	-0.22924	-1.08451	-0.418065	BGIOSGA029680	XP_003581081.2probable LRR receptor-like serine/threonine-protein kinase At3g47570
TCONS_00011540	-0.229228	-1.40122	-0.496902	BGIOSGA011485	XP_006649378.1PREDICTED: uncharacterized protein LOC102719843
TCONS_00031484	-0.229099	-1.0143	0.153124	BGIOSGA016946	XP_006661972.1 F-box protein PP2-A13-like
TCONS_00033901	-0.229092	-2.62979	-3.60456	BGIOSGA013536	XP_002437334.1premnaspirodiene oxygenase
TCONS_00019590	-0.228755	0.0824732	-0.292332	BGIOSGA017769	XP_004961588.1sulfite reductase [ferredoxin], chloroplastic
TCONS_00012893	-0.228716	-0.0285292	0.678562	BGIOSGA012605	XP_015690277.1 40S ribosomal protein S21
TCONS_00030335	-0.228681	0.845414	0.671635	BGIOSGA000224	XP_006660046.2 60S ribosomal protein L7a
TCONS_00000892	-0.228569	-0.138022	-0.135869	BGIOSGA030174	XP_015693720.1 poly [ADP-ribose] polymerase 2-A isoform X1
TCONS_00016934	-0.228563	0.236562	-0.078714	BGIOSGA014030	XP_006653084.1PREDICTED: uncharacterized protein LOC102711527
TCONS_00024810	-0.228516	0.592647	-0.267864	BGIOSGA007636	XP_004981250.1dof zinc finger protein DOF2.4
TCONS_00008916	-0.228371	0.108291	-0.418327	BGIOSGA005013	XP_004954115.1subtilisin-like protease SBT1.4
TCONS_00036633	-0.228026	-0.913205	0.17838	BGIOSGA034547	XP_006663799.1 dual specificity protein phosphatase 12-like
TCONS_00009829	-0.22793	-0.530082	-0.47427	BGIOSGA036062	XP_006649898.1PREDICTED: uncharacterized protein LOC102704684
TCONS_00037447	-0.227409	1.19578	0.967176	BGIOSGA007378	XP_021317519.1uncharacterized protein LOC8076351 isoform X1
TCONS_00020383	-0.227385	0.650115	0.729329	BGIOSGA009998	XP_006656798.1PREDICTED: uncharacterized protein LOC102703693, partial
TCONS_00012525	-0.227116	0.184024	0.519822	BGIOSGA017281	XP_006650232.1 ABC transporter F family member 4
TCONS_00010482	-0.227068	-2.08725	-1.10153	BGIOSGA013086	XP_006650284.1 cytochrome P450 85A1
TCONS_00027999	-0.227001	-0.409261	-2.00859	BGIOSGA015274	XP_004959674.1F-box protein At2g26160
TCONS_00007216	-0.226988	0.448704	-0.549187	BGIOSGA016824	NP_001183350.2uncharacterized protein LOC100501758
TCONS_00024266	-0.22655	0.584327	-0.174732	BGIOSGA035389	XP_006658058.2 protein transport protein SEC31 homolog B
TCONS_00008735	-0.22651	1.2585	-0.687875	BGIOSGA030958	XP_006647828.2 transcription factor BIM2-like isoform X2
TCONS_00014612	-0.226489	0.290535	0.00509624	BGIOSGA030526	XP_015692282.1 pentatricopeptide repeat-containing protein At1g77360, mitochondrial-like
TCONS_00025650	-0.226375	0.580257	0.51807	BGIOSGA012622	XP_006658068.1 protein CHROMATIN REMODELING 5 isoform X2
TCONS_00015924	-0.226359	-1.41007	-2.42521	BGIOSGA006264	XP_015692131.1 protein disulfide isomerase-like 1-2
TCONS_00026139	-0.2262	0.642843	-0.0470935	BGIOSGA000133	XP_004972551.1glucan endo-1,3-beta-glucosidase 6
TCONS_00025351	-0.226182	0.107558	0.159313	BGIOSGA024080	XP_006657846.1PREDICTED: uncharacterized protein LOC102704628
TCONS_00029122	-0.226147	0.941826	1.27179	BGIOSGA029662	XP_015698225.1 F-box protein At3g07870-like
TCONS_00017076	-0.226037	-0.17487	-0.502247	BGIOSGA006973	XP_006654012.1 probable protein phosphatase 2C 47
TCONS_00036745	-0.225945	-0.487359	-0.22412	BGIOSGA034877	XP_006663851.1 protein FAM135B-like isoform X1
TCONS_00003764	-0.225881	0.759256	0.211035	BGIOSGA001441	XP_006645990.1PREDICTED: uncharacterized protein LOC102722172 isoform X1
TCONS_00008479	-0.2258	0.25481	0.130463	BGIOSGA033193	XP_015688676.1 autophagy-related protein 13a
TCONS_00026083	-0.225643	-0.352074	-0.137475	BGIOSGA034318	XP_015695725.1 F-box/LRR-repeat protein At1g55660-like isoform X2
TCONS_00001701	-0.225476	-0.352284	-1.72285	BGIOSGA013714	XP_015688192.1 type IV inositol polyphosphate 5-phosphatase 3 isoform X1
TCONS_00030779	-0.225424	0.910686	-0.597446	BGIOSGA012056	XP_015697457.1 nucleolin-like isoform X3
TCONS_00027358	-0.225368	0.57054	0.548094	BGIOSGA028034	XP_006659839.1 pentatricopeptide repeat-containing protein At4g16835, mitochondrial
TCONS_00030329	-0.225278	-1.04886	-0.913308	BGIOSGA016744	XP_015696485.1 nucleotide pyrophosphatase/phosphodiesterase-like
TCONS_00036500	-0.225189	-0.546467	0.168266	BGIOSGA037869	XP_006664786.2 mediator of RNA polymerase II transcription subunit 17
TCONS_00007197	-0.225069	0.551786	0.793995	BGIOSGA007299	XP_015694814.1 U3 small nucleolar RNA-associated protein 6 homolog
TCONS_00029520	-0.224959	0.465163	0.204792	BGIOSGA014476	XP_006660982.1 probable protein S-acyltransferase 19

TCONS_00006311	-0.224884	0.432832	-0.708961	BGIOSGA016517	XP_004952853.1cryptochrome-1 isoform X1
TCONS_00034097	-0.224865	0.721912	-0.183788	BGIOSGA018951	XP_006663689.1 scarecrow-like protein 34
TCONS_00014078	-0.224781	0.784348	1.25895	BGIOSGA016229	XP_015691419.1 methyltransferase-like protein 13
TCONS_00020541	-0.224677	-0.286696	-0.58433	BGIOSGA030368	XP_015693829.1 RPM1-interacting protein 4-like isoform X2
TCONS_00002164	-0.224353	-1.63601	-0.141768	BGIOSGA011034	XP_006645004.1 probable mannose-1-phosphate guanylyltransferase 3
TCONS_00030831	-0.224282	-1.07403	-0.736349	BGIOSGA027830	XP_006659725.1 protein odr-4 homolog
TCONS_00024173	-0.22404	0.423524	-0.550227	BGIOSGA021755	XP_006658008.1 cytochrome P450 709B2-like
TCONS_00015355	-0.223966	-1.79939	-1.94431	BGIOSGA000001	XP_006653160.1 bifunctional L-3-cyanoalanine synthase/cysteine synthase 1, mitochondrial
TCONS_00036906	-0.223925	0.1153	-0.0887454	BGIOSGA007448	XP_006663922.1 transcription factor HBP-1a-like
TCONS_00037472	-0.223893	-0.201445	-0.215404	BGIOSGA020656	NP_001310439.1uncharacterized LOC103640569
TCONS_00005665	-0.223875	-0.80175	-0.900821	BGIOSGA007787	XP_004951470.2receptor kinase-like protein Xa21
TCONS_00002293	-0.223872	-0.560157	-0.323885	BGIOSGA017686	XP_003567336.1ubinuclein-1 isoform X2
TCONS_00030313	-0.222971	0.174228	-0.124391	BGIOSGA029026	XP_006660801.1 60S ribosomal protein L32-1
TCONS_00000299	-0.222932	-0.362705	-1.16376	BGIOSGA004408	XP_003569538.4UDP-glycosyltransferase 73C1
TCONS_00030315	-0.222795	0.155304	0.312858	BGIOSGA029026	XP_006660801.1 60S ribosomal protein L32-1
TCONS_00006756	-0.222769	0.357357	0.654444	BGIOSGA008925	XP_006647797.1 outer envelope pore protein 16-3, chloroplastic/mitochondrial-like
TCONS_00014732	-0.222694	-1.28305	-0.218748	BGIOSGA016909	XP_015692234.1 acyl-coenzyme A thioesterase 8 isoform X2
TCONS_00004535	-0.222678	0.155731	-0.375648	BGIOSGA000681	XP_003564374.2sulfite exporter TauE/SafE family protein 5
TCONS_00014976	-0.222483	-0.268451	0.158511	BGIOSGA017165	XP_006653780.2 signal recognition particle 14 kDa protein
TCONS_00002557	-0.222451	-0.204824	-0.0979724	BGIOSGA019788	XP_006646694.1 probable NAD kinase 1
TCONS_00005215	-0.22245	-1.76123	-0.786301	BGIOSGA021612	XP_015688962.1 amino acid permease 3-like
TCONS_00016205	-0.222412	-0.232467	-0.7575	BGIOSGA029629	XP_015692349.1PREDICTED: uncharacterized protein LOC107304147
TCONS_00007529	-0.222279	-0.295201	0.288534	BGIOSGA005288	XP_006645415.1 dnaJ protein P58IPK homolog
TCONS_00005201	-0.222039	-0.14318	-0.00258457	BGIOSGA020522	XP_010941352.1 probable protein phosphatase 2C 79 isoform X2
TCONS_00015220	-0.22168	-0.100006	0.490989	BGIOSGA017400	XP_006653074.1 small glutamine-rich tetratricopeptide repeat-containing protein
TCONS_00011182	-0.221227	1.02429	0.190487	BGIOSGA025573	XP_006650764.1 40S ribosomal protein S2-4-like
TCONS_00020316	-0.22095	-0.014649	-0.127775	BGIOSGA022511	NP_001148368.2gb protein
TCONS_00001829	-0.220835	-1.61091	-0.528405	BGIOSGA017552	XP_010102136.1hypothetical protein L484_021370
TCONS_00018386	-0.22073	0.616616	0.345062	BGIOSGA020438	XP_006654843.1PREDICTED: uncharacterized protein LOC102715021
TCONS_00008968	-0.220671	-0.93841	-0.195277	BGIOSGA012149	XP_006648050.1 two-component response regulator ORR23
TCONS_00011580	-0.220477	0.332045	-0.0663437	BGIOSGA006908	XP_006649413.1 cell division control protein 48 homolog E-like
TCONS_00000007	-0.220038	-0.500611	-0.387656	BGIOSGA002576	XP_006643625.1 sphingosine-1-phosphate lyase
TCONS_00011418	-0.219997	0.182589	0.222611	BGIOSGA011606	XP_015691213.1 SKP1-like protein 21
TCONS_00020959	-0.219945	0.331201	-2.12657	#N/A	#N/A
TCONS_00000271	-0.219799	-0.69686	-1.00483	BGIOSGA002851	XP_015691963.1 probable staphylococcal-like nuclease CAN1
TCONS_00023257	-0.219767	-1.79211	-2.27068	BGIOSGA025298	XP_006657520.1 peptidyl-prolyl cis-trans isomerase FKBP16-4, chloroplastic
TCONS_00003763	-0.219757	-0.00467575	-0.0376802	BGIOSGA032220	XP_015688978.1 protein MICRORCHIDIA 6-like
TCONS_00031744	-0.219545	-1.62175	-1.03029	BGIOSGA011145	XP_006662146.1PREDICTED: uncharacterized protein LOC102714587
TCONS_00006675	-0.219397	0.564327	0.0630083	BGIOSGA008843	XP_002454449.1AP-2 complex subunit mu
TCONS_00024037	-0.219348	-0.65796	0.81447	BGIOSGA026075	XP_006657915.1 EID1-like F-box protein 2
TCONS_00010239	-0.219313	-0.349962	0.130164	BGIOSGA026265	XP_006650168.1 CLK4-associating serine/arginine rich protein
TCONS_00014585	-0.219248	0.268342	0.29005	BGIOSGA016749	XP_015692372.1 putative G3BP-like protein isoform X2
TCONS_00032124	-0.219198	0.0592673	0.0232237	BGIOSGA032011	XP_003564341.1red chlorophyll catabolite reductase isoform X2
TCONS_00030009	-0.218953	-0.477909	-0.902256	BGIOSGA013197	XP_015696468.1 TPR repeat-containing thioredoxin TDX
TCONS_00004280	-0.218848	-1.31742	-1.06625	BGIOSGA014577	XP_004969732.1cytidine deaminase 1
TCONS_00016596	-0.218815	0.40654	0.214309	BGIOSGA014369	XP_006653757.1 probable RNA-binding protein 19
TCONS_00037480	-0.218744	-2.01857	-1.61837	BGIOSGA028533	XP_004963109.1CBL-interacting protein kinase 4
TCONS_00015224	-0.218716	-0.203948	0.536662	BGIOSGA035129	XP_006653077.2 protein NRT1/ PTR FAMILY 5.10-like
TCONS_00017266	-0.218704	0.118276	-0.125474	BGIOSGA019308	XP_006654130.1 nodulin homeobox
TCONS_00013272	-0.218462	-0.103782	0.141375	BGIOSGA009714	XP_006650681.1 probable ribose-5-phosphate isomerase 4, chloroplastic isoform X1
TCONS_00027166	-0.218419	1.09349	1.0834	BGIOSGA022008	XP_003573227.1monothiol glutaredoxin-S10
TCONS_00009729	-0.218355	1.02319	-0.211957	BGIOSGA024122	XP_006649796.1 putative serine/threonine-protein kinase
TCONS_00036309	-0.21832	-0.201476	-0.0344584	BGIOSGA011183	XP_015698528.1 probable polyamine transporter At3g19553
TCONS_00019934	-0.218288	0.0635626	0.0347082	BGIOSGA022119	XP_015694145.1PREDICTED: uncharacterized protein LOC102710214 isoform X3
TCONS_00026707	-0.217839	0.255645	-0.204802	BGIOSGA028770	XP_006660180.1 conserved oligomeric Golgi complex subunit 3 isoform X1
TCONS_00026792	-0.217577	-0.689401	-0.980636	BGIOSGA028854	XP_006659500.1PREDICTED: uncharacterized protein LOC102711723 isoform X2

TCONS_00019469	-0.21756	-1.23942	-0.824482	BGIOSGA010931	XP_008649737.1protein MALE DISCOVERER 2 isoform X3
TCONS_00007354	-0.217237	-0.650959	-0.615542	BGIOSGA023521	XP_006646892.1PREDICTED: uncharacterized protein LOC102713517
TCONS_00000506	-0.217092	-0.4052	0.414783	BGIOSGA003079	XP_006647767.1 chromatin modification-related protein MEAF6
TCONS_00030434	-0.216856	-0.313591	-0.0643987	BGIOSGA029368	XP_015696717.1 DNA-(apurinic or apyrimidinic site) lyase 2 isoform X1
TCONS_00001790	-0.216655	-1.31888	-0.344906	BGIOSGA002188	XP_006644678.1 putative dual specificity protein phosphatase DSP8
TCONS_00039529	-0.216627	-0.393979	0.674503	BGIOSGA039356	YP_008815761.1photosystem I assembly protein ycf4 (plastid)
TCONS_00035008	-0.216525	0.728182	0.0765922	BGIOSGA033849	XP_008679455.1transportin MOS14
TCONS_00014430	-0.216468	-0.0153887	1.612	BGIOSGA016588	XP_006652402.1PREDICTED: uncharacterized protein LOC102719483
TCONS_00013212	-0.216415	-5.1156	-0.658884	BGIOSGA027891	XP_006650629.1 peamaclein-like
TCONS_00013716	-0.216364	0.234503	-0.297459	BGIOSGA007562	XP_015692158.1 ATG8-interacting protein 1-like
TCONS_00006121	-0.216341	-0.849468	0.0178537	#N/A	XP_006647275.2 ubiquitin-like modifier-activating enzyme 5
TCONS_00005605	-0.216324	-0.167686	0.118644	BGIOSGA007718	XP_006646996.2PREDICTED: uncharacterized protein LOC102719552
TCONS_00003327	-0.216277	-0.0198186	0.319895	BGIOSGA013187	XP_006644066.1 ERI1 exoribonuclease 2-like
TCONS_00009212	-0.216209	0.211167	0.651998	BGIOSGA027975	XP_006649295.1 nascent polypeptide-associated complex subunit alpha-like protein 1
TCONS_00015434	-0.216163	0.14712	-0.066106	BGIOSGA000853	XP_006652106.1 G-type lectin S-receptor-like serine/threonine-protein kinase RLK1
TCONS_0000511	-0.215632	-1.56653	-0.720331	BGIOSGA029435	XP_008806757.1 probable pectinesterase/pectinesterase inhibitor 34
TCONS_00020791	-0.215559	0.765821	0.936902	BGIOSGA022989	XP_006656998.1PREDICTED: uncharacterized protein LOC102717626
TCONS_00026989	-0.215508	0.360387	1.04293	BGIOSGA027180	XP_006659592.1 FHA domain-containing protein FHA2 isoform X1
TCONS_00012563	-0.215497	-0.571107	-0.032066	BGIOSGA010411	XP_006650263.1 purple acid phosphatase 18 isoform X1
TCONS_00023916	-0.215256	0.84113	0.300052	BGIOSGA025949	XP_015695376.1 elongator complex protein 1
TCONS_00021746	-0.215145	-1.29184	-0.55689	BGIOSGA009200	XP_006655855.2 ubiquitin carboxyl-terminal hydrolase 23
TCONS_00011952	-0.215107	1.87309	1.10495	BGIOSGA011059	XP_006649791.1 splicing factor 3A subunit 2
TCONS_00002660	-0.214866	-0.465992	0.0224206	BGIOSGA002561	XP_006663462.2 probable 3-beta-hydroxysteroid-Delta(8),Delta(7)-isomerase
TCONS_00000794	-0.214715	-0.698734	-0.111845	BGIOSGA003386	XP_003567621.1protein FAM192A
TCONS_00003310	-0.214514	-0.259068	0.516085	BGIOSGA015647	XP_006644052.1 protein argonaute 4A
TCONS_00004334	-0.214331	-0.153345	0.123271	BGIOSGA016023	XP_015695404.1 F-box/FBD/LRR-repeat protein At1g13570-like isoform X1
TCONS_00032710	-0.214317	-0.543699	-1.40328	BGIOSGA024221	XP_006662604.2 transcription factor DIVARICATA-like
TCONS_00034198	-0.214253	-1.87516	-1.17714	BGIOSGA030403	XP_006662689.1 vacuolar protein sorting-associated protein 32 homolog 2-like isoform X3
TCONS_00021906	-0.214187	-0.262608	-1.15699	BGIOSGA021322	XP_015693851.1 sphinganine C4-monooxygenase 1-like
TCONS_00002936	-0.213864	0.75665	-0.736695	BGIOSGA002288	XP_004968380.1transcription factor HY5
TCONS_00016513	-0.21367	0.459593	0.312045	BGIOSGA021330	XP_006652719.1 AT-hook motif nuclear-localized protein 10-like
TCONS_00011323	-0.213546	0.0634631	0.629723	BGIOSGA023266	XP_022683262.1uncharacterized protein LOC101757873 isoform X1
TCONS_00010848	-0.213439	2.48903	0.137971	BGIOSGA021785	XP_015691127.1 glutamate decarboxylase 1
TCONS_00022640	-0.213417	0.413448	0.850876	BGIOSGA030213	NP_001149805.1CUE domain containing protein
TCONS_00016115	-0.21335	0.721227	0.695069	BGIOSGA029175	XP_006652424.1 protein-L-isoaspartate O-methyltransferase
TCONS_00003390	-0.213026	-0.490733	0.205663	BGIOSGA006860	XP_003567580.1pentatricopeptide repeat-containing protein At4g01990, mitochondrial
TCONS_00011998	-0.212987	-0.00172698	2.51994	BGIOSGA004688	XP_004984730.1E3 ubiquitin-protein ligase XB3
TCONS_00017692	-0.212762	-0.884804	-0.452236	BGIOSGA019753	XP_015692578.1 peptide chain release factor PrfB3, chloroplastic
TCONS_00017968	-0.212686	-0.639837	0.250805	BGIOSGA006455	XP_008656382.1UTP--glucose-1-phosphate uridylyltransferase 3, chloroplastic
TCONS_00006020	-0.212634	0.0877975	-0.372765	BGIOSGA034610	XP_010237945.1uncharacterized protein LOC100822671
TCONS_00008294	-0.212347	1.83539	0.573016	BGIOSGA014885	XP_006647446.1 protein NBR1 homolog
TCONS_00026095	-0.21222	-1.23837	-0.234077	BGIOSGA038523	XP_015695987.1 probable LRR receptor-like serine/threonine-protein kinase At1g56140
TCONS_00023924	-0.212052	-1.09648	-0.0390198	BGIOSGA025957	XP_015694689.1 peptidyl-prolyl cis-trans isomerase CYP37, chloroplastic
TCONS_00009245	-0.212001	-0.555561	-0.438156	BGIOSGA030655	XP_015689753.1 probable serine/threonine-protein kinase Cx32, chloroplastic
TCONS_00025766	-0.211957	1.27071	0.330316	BGIOSGA010705	XP_015695979.1 ENHANCER OF AG-4 protein 2-like
TCONS_00028678	-0.211878	-0.169036	-0.884597	BGIOSGA000451	XP_015696803.1 transcription factor TGA2-like isoform X1
TCONS_00014310	-0.211777	-0.512414	-0.916904	BGIOSGA019011	NP_001336775.1uncharacterized LOC100194096
TCONS_00007258	-0.211524	0.0389268	-0.11985	BGIOSGA008530	XP_010233736.1bromodomain-containing protein 4 isoform X2
TCONS_00029269	-0.211137	-0.291522	-0.848956	BGIOSGA020364	XP_006660779.1 splicing factor U2af small subunit A
TCONS_00022053	-0.210887	0.383063	-1.43057	BGIOSGA012168	XP_004965227.1putative U-box domain-containing protein 42
TCONS_00011261	-0.210265	0.0603732	-1.0092	BGIOSGA004154	XP_021314601.1mannan endo-1,4-beta-mannosidase 3
TCONS_00022849	-0.210175	0.579308	0.397007	BGIOSGA009856	XP_015693694.1 serrate RNA effector molecule-like
TCONS_00026890	-0.210092	-0.153654	0.0360839	BGIOSGA028947	XP_003574734.1mediator of RNA polymerase II transcription subunit 15
TCONS_00025393	-0.210041	-0.246639	0.484718	BGIOSGA013396	XP_006658727.1 importin-5

TCONS_00024013	-0.209949	-1.15056	0.842045	BGIOSGA025841	XP_004958181.1LOB domain-containing protein 37
TCONS_00036279	-0.20992	-0.636717	-0.304465	BGIOSGA037637	XP_006664657.1 cleavage stimulation factor subunit 77 isoform X3
TCONS_00022906	-0.209918	-0.210085	-1.26128	BGIOSGA012283	XP_004966442.1WAS protein family homolog 2
TCONS_00034253	-0.20974	0.115169	0.279554	BGIOSGA036634	XP_006662722.1 proteasome-associated protein ECM29 homolog isoform X1
TCONS_00009357	-0.209327	0.780815	0.908634	BGIOSGA017091	XP_015690892.1 serine/threonine-protein kinase EDR1-like
TCONS_00005564	-0.209003	-0.988261	-1.30168	BGIOSGA000247	XP_015688396.1PREDICTED: uncharacterized protein LOC102715728
TCONS_00024374	-0.208586	0.0770389	0.39367	BGIOSGA008697	XP_006658151.1 short-chain dehydrogenase/reductase 2b-like isoform X1
TCONS_00004231	-0.208518	-1.75676	-1.66111	BGIOSGA004282	XP_006644535.2 putative D-cysteine desulfhydrase 2, mitochondrial
TCONS_00000214	-0.208316	-0.934386	-0.432297	BGIOSGA002793	XP_006643761.1 very-long-chain (3R)-3-hydroxyacyl-CoA dehydratase PASTICCINO 2A
TCONS_00005330	-0.208308	0.436754	0.00143095	#N/A	XP_006646825.2 ER membrane protein complex subunit 2
TCONS_00028318	-0.208151	-0.204325	-0.123912	BGIOSGA035840	XP_006659604.2 auxin response factor 21
TCONS_00017655	-0.208141	-0.467842	-0.951387	BGIOSGA003041	XP_006655239.1 beta-glucosidase 20-like
TCONS_00012715	-0.208137	-0.639297	-0.0240491	BGIOSGA000111	XP_015689971.1 protein OSB1, mitochondrial-like
TCONS_00000210	-0.20807	0.429836	-0.444861	BGIOSGA030551	XP_006643766.1 histone H2B.5
TCONS_00005373	-0.207637	-0.29274	-0.305194	BGIOSGA007484	XP_006646857.2 WD repeat-containing protein 11-like
TCONS_00011945	-0.207622	1.31409	0.393086	BGIOSGA020300	XP_015691220.1 importin-4 isoform X2
TCONS_00022980	-0.207542	-1.7672	-0.496121	BGIOSGA035005	NP_001151705.1disease resistance response protein 206
TCONS_00010985	-0.207302	-0.0796989	-0.326509	BGIOSGA030717	XP_024313641.1UDP-glycosyltransferase 83A1-like
TCONS_00010961	-0.207202	-1.49578	-0.593649	BGIOSGA013561	XP_015690732.1 beta-glucosidase BoGH3B-like
TCONS_00022010	-0.207106	-0.526464	0.152936	BGIOSGA029492	XP_006655983.1 putative 1-phosphatidylinositol-3-phosphate 5-kinase FAB1C
TCONS_00011375	-0.207062	-2.77744	-1.10307	BGIOSGA015440	XP_004980973.1probable carbohydrate esterase At4g34215
TCONS_00011963	-0.206952	-1.07053	0.432953	BGIOSGA017943	XP_006649805.1PREDICTED: uncharacterized protein LOC102701899
TCONS_00009440	-0.20692	-0.600211	-1.40839	BGIOSGA011988	XP_015691106.1PREDICTED: uncharacterized protein LOC102710767
TCONS_00031445	-0.206879	0.18273	-0.158744	BGIOSGA000319	XP_015697366.1 probable methyltransferase PMT18
TCONS_00021769	-0.206765	-0.20546	0.719387	BGIOSGA026866	XP_015694027.1 glutamate receptor 2.8-like
TCONS_00029946	-0.206602	-0.655033	-0.353554	BGIOSGA019299	XP_015696330.1 potassium transporter 23
TCONS_00005782	-0.206542	-1.35221	-0.893821	BGIOSGA019126	XP_003572041.1GDSL esterase/lipase At5g45910
TCONS_00024079	-0.206506	-0.349119	0.379816	BGIOSGA026275	XP_015694825.1 armadillo repeat-containing protein LFR
TCONS_00001297	-0.206474	-1.46725	-0.311012	BGIOSGA027867	XP_004967657.1putative disease resistance RPP13-like protein 3
TCONS_00002038	-0.206397	0.386798	-0.27878	BGIOSGA004672	XP_014754963.1dentin sialophosphoprotein
TCONS_00019889	-0.206076	-0.581218	0.137031	BGIOSGA017473	XP_006654848.1 mitochondrial adenine nucleotide transporter ADNT1
TCONS_00017866	-0.205916	0.357117	-0.0415053	BGIOSGA009826	XP_006654445.1PREDICTED: uncharacterized protein At5g49945-like
TCONS_00016226	-0.205818	-0.219221	-0.236103	BGIOSGA011834	XP_015692358.1PREDICTED: uncharacterized protein LOC102705158
TCONS_00011095	-0.205679	1.87484	-0.0729453	BGIOSGA013695	XP_015690101.1 nuclear pore complex protein NUP1-like
TCONS_00003432	-0.205574	1.88663	0.443954	BGIOSGA009184	XP_006645818.1 UDP-glucuronic acid decarboxylase 2
TCONS_00028338	-0.205346	-0.712775	-1.39826	BGIOSGA008711	XP_006659598.1 ras-related protein RABA5c-like
TCONS_00023973	-0.205242	0.462143	0.268788	BGIOSGA030566	XP_006658733.1 probable succinyl-CoA ligase [ADP-forming] subunit alpha, mitochondrial
TCONS_00010466	-0.205061	-1.16568	0.419272	BGIOSGA034994	XP_006650297.1PREDICTED: uncharacterized protein LOC102704131
TCONS_00030258	-0.20494	1.32853	1.47109	BGIOSGA022251	XP_012698798.1lysine-specific demethylase JMJ30 isoform X1
TCONS_00007909	-0.204856	-0.902017	-0.182328	BGIOSGA025403	XP_002464098.2ADP-ribosylation factor-like protein 2 isoform X2
TCONS_00016649	-0.204694	0.441751	-0.538546	BGIOSGA024983	XP_004976873.1CAP-Gly domain-containing linker protein 1
TCONS_00004770	-0.204619	0.582551	0.655215	BGIOSGA000445	XP_006646499.1 sorting nexin 1
TCONS_00009238	-0.204613	-0.170771	-1.1938	BGIOSGA020719	XP_006649326.2 glyceraldehyde-3-phosphate dehydrogenase GAPB, chloroplastic
TCONS_00006895	-0.204559	-0.993874	0.40714	BGIOSGA009073	XP_006647914.1PREDICTED: uncharacterized protein LOC102708495 isoform X1
TCONS_00015540	-0.204536	-0.635663	-0.756079	BGIOSGA015437	XP_006652149.2 tRNA-dihydrouridine(20/20a) synthase-like
TCONS_00005131	-0.204524	-1.35144	-0.678988	BGIOSGA000591	XP_015698512.1 probable LRR receptor-like serine/threonine-protein kinase At1g06840
TCONS_00010194	-0.204515	0.286179	-0.312861	BGIOSGA012760	XP_006651442.1 tRNA pseudouridine(38/39) synthase
TCONS_00003965	-0.204456	-0.501821	0.267504	BGIOSGA001225	XP_006646087.1 carbon catabolite repressor protein 4 homolog 6
TCONS_00024069	-0.204327	-1.30837	-0.177263	BGIOSGA022383	XP_015694433.1 dehydrodolichyl diphosphate synthase 6
TCONS_00004013	-0.204207	-0.0893749	0.0290967	BGIOSGA001179	XP_015699114.1 neutral ceramidase
TCONS_00029108	-0.204188	0.490861	0.440057	BGIOSGA030861	XP_015696370.1 gamma-tubulin complex component 3
TCONS_00028174	-0.204154	0.446189	0.242637	BGIOSGA037557	XP_006659497.1 patatin-like protein 2
TCONS_00006251	-0.204126	0.237009	-0.868933	BGIOSGA008412	XP_015689532.1 transmembrane 9 superfamily member 1-like

TCONS_00035715	-0.203958	-0.342555	-0.00312523	BGIOSGA013887	XP_015698666.1 translocase of chloroplast 90, chloroplastic
TCONS_00029116	-0.203699	-0.598642	-0.714071	BGIOSGA028182	XP_006660672.2 probable potassium transporter 17
TCONS_00003224	-0.203458	-4.20954	-0.965473	BGIOSGA034439	XP_006643995.1 germin-like protein 5-1
TCONS_00028651	-0.203316	-0.192851	-0.103103	BGIOSGA030403	XP_006660478.1 protein NEDD1
TCONS_00022781	-0.203245	-0.501299	-0.0162033	BGIOSGA019282	XP_006656381.2PREDICTED: uncharacterized protein LOC102712822
TCONS_00003470	-0.203129	-0.0316871	0.316668	BGIOSGA003405	XP_004968668.1ubiquitin-40S ribosomal protein S27a-1
TCONS_00011436	-0.203024	-0.233303	-0.558523	BGIOSGA011588	XP_014753998.1uncharacterized protein LOC100833370
TCONS_00001242	-0.203013	-3.49164	-1.7641	BGIOSGA004334	XP_015695850.1 putative methyltransferase DDB_G0268948
TCONS_00030874	-0.202821	-0.1474	-1.0209	BGIOSGA032678	XP_024318204.1uncharacterized protein LOC100834118
TCONS_00003553	-0.202652	0.882664	0.228448	#N/A	#N/A
TCONS_00028300	-0.202543	1.68135	1.06622	BGIOSGA013123	XP_006659616.1 calcium-transporting ATPase 8, plasma membrane-type-like isoform X2
TCONS_00026346	-0.202461	0.659598	0.824272	BGIOSGA007680	XP_006659287.1 probable sucrose-phosphate synthase 4
TCONS_00030052	-0.202326	-0.982587	-0.892288	BGIOSGA006096	XP_014758812.1CBL-interacting protein kinase 16
TCONS_00010016	-0.202319	0.690177	-0.611177	BGIOSGA021216	XP_006650046.1 phospholipid-transporting ATPase 1-like
TCONS_00002961	-0.202278	0.373745	0.308603	BGIOSGA018693	XP_015688186.1 MAR-binding filament-like protein 1
TCONS_00018237	-0.202271	0.327488	0.242826	BGIOSGA013619	XP_006654397.1 RAN GTPase-activating protein 2-like
TCONS_00024842	-0.202195	1.26189	0.803396	BGIOSGA024527	XP_006658440.1 pentatricopeptide repeat-containing protein At3g06920
TCONS_00009317	-0.202042	-1.56469	-1.45015	BGIOSGA011860	XP_006649399.1PREDICTED: uncharacterized protein SYNPCC7002_A1590
TCONS_00021898	-0.201923	-0.0424619	-0.556041	BGIOSGA021631	XP_021304238.1transcription termination factor MTERF15, mitochondrial isoform X1
TCONS_00010555	-0.201751	-0.957563	-0.337583	BGIOSGA008210	XP_006650318.1 calcineurin B-like protein 3
TCONS_00013171	-0.201734	-0.867967	-0.196382	BGIOSGA009813	XP_015690066.1 photosystem II D1 precursor processing protein PSB27-H2, chloroplastic
TCONS_00031002	-0.201672	-1.18806	0.343717	BGIOSGA012780	NP_001149884.1catalytic/ hydrolase
TCONS_00021568	-0.201643	-1.71743	-1.60006	BGIOSGA011877	XP_006656612.1 armadillo repeat-containing kinesin-like protein 2
TCONS_00013134	-0.201569	-0.149253	-0.0633534	BGIOSGA020076	XP_015690933.1 probable NOT transcription complex subunit VIP2 isoform X2
TCONS_00003317	-0.201516	0.216288	0.581914	BGIOSGA001897	XP_006645765.2 glucosidase 2 subunit beta
TCONS_00036976	-0.201503	-0.94314	-2.58628	BGIOSGA020439	XP_015698395.1 probable solanesyl-diphosphate synthase 3, chloroplastic
TCONS_00008254	-0.201357	-0.885295	-0.295951	BGIOSGA014926	XP_006647427.1 dentin sialophosphoprotein-like
TCONS_00015164	-0.201347	0.896692	0.442532	BGIOSGA017346	XP_006653876.1 dihydroorotate dehydrogenase (quinone), mitochondrial
TCONS_00021231	-0.201344	-0.493754	0.0390853	BGIOSGA037776	XP_015693718.1 NAC domain-containing protein 21/22-like
TCONS_00036999	-0.201333	-0.373919	0.189705	BGIOSGA007498	XP_006663935.1 zinc finger CCCH domain-containing protein 65 isoform X1
TCONS_00029731	-0.201186	0.224618	0.310612	BGIOSGA028977	XP_006660486.1 protein EMSY-LIKE 3-like
TCONS_00013378	-0.201126	-1.07261	-1.20088	BGIOSGA009606	XP_006651932.1 probable acyl-activating enzyme 18, peroxisomal
TCONS_00016029	-0.201087	0.657982	-0.147929	BGIOSGA009914	XP_006653489.1 anthranilate synthase beta subunit 1, chloroplastic
TCONS_00011766	-0.200874	0.336994	-0.0574418	BGIOSGA014312	XP_006649602.1 metal transporter Nramp2
TCONS_00021351	-0.200757	0.339176	0.63004	BGIOSGA035109	XP_004966382.1mitogen-activated protein kinase 12
TCONS_00027168	-0.200744	-0.306306	-0.241624	BGIOSGA033093	XP_015695925.1 ribokinase
TCONS_00001668	-0.200685	-0.33635	-0.601904	BGIOSGA014717	XP_006644591.1 casein kinase I isoform delta-like isoform X1
TCONS_00003770	-0.200654	-0.673367	-0.383233	BGIOSGA019520	XP_006645993.1 ribonuclease 3-like protein 2
TCONS_00007028	-0.20055	0.190872	-0.238602	BGIOSGA009210	XP_006648056.1 transcriptional regulatory protein AlgP-like
TCONS_00007936	-0.200257	-1.2472	-0.392459	BGIOSGA023116	XP_015692636.1PREDICTED: uncharacterized protein LOC107304196
TCONS_00037551	-0.200101	0.779853	0.00095938	BGIOSGA035814	XP_006664215.1 methyl-CpG-binding domain-containing protein 11-like isoform X1
TCONS_00017039	-0.199803	-1.10429	-0.920094	BGIOSGA017711	XP_006653992.1 protein IQ-DOMAIN 1
TCONS_00003983	-0.199562	-0.638019	-1.15603	BGIOSGA008609	XP_006644368.1 3'-N-debenzoyl-2'-deoxytaxol N-benzoyltransferase-like
TCONS_00016807	-0.199432	0.0510836	-0.255222	BGIOSGA017305	XP_022680720.1peptidyl-prolyl cis-trans isomerase NIMA-interacting 1
TCONS_00033418	-0.199354	0.134705	-1.37051	BGIOSGA018007	XP_006662926.1 serine hydroxymethyltransferase 4
TCONS_00028478	-0.199325	-0.181794	-0.293153	BGIOSGA040675	XP_006659708.1 puromycin-sensitive aminopeptidase isoform X1
TCONS_00019947	-0.199323	-0.775541	0.142522	BGIOSGA022131	XP_006656540.1 glycine--tRNA ligase, chloroplastic/mitochondrial 2
TCONS_00021467	-0.199319	-2.14115	-4.90546	BGIOSGA022084	XP_015694337.1 haloacid dehalogenase-like hydrolase domain-containing protein Sgpp
TCONS_00007203	-0.199268	0.431821	0.0719651	BGIOSGA019732	XP_006646768.1 40S ribosomal protein S4
TCONS_00036358	-0.199115	-0.531414	0.237574	BGIOSGA013632	XP_015698784.1 protein DA1-related 1-like
TCONS_00017855	-0.199081	-0.712004	0.674379	BGIOSGA000405	XP_006654439.1 deoxycytidine kinase-like
TCONS_00004395	-0.198984	-1.92702	-0.3471	BGIOSGA018089	XP_015688107.1 probable WRKY transcription factor 65
TCONS_00000004	-0.198974	-0.480615	-0.059669	BGIOSGA007677	XP_006643623.1 R3H domain-containing protein 1-like
TCONS_00025646	-0.198973	-2.76023	-2.5894	BGIOSGA023780	XP_006658064.1 haloacid dehalogenase-like hydrolase domain-containing protein 3

TCONS_00035637	-0.198763	-0.597922	0.20284	BGIOSGA037057	XP_015698228.1 5-formyltetrahydrofolate cyclo-ligase-like protein COG0212
TCONS_00004978	-0.198661	0.298956	-2.23083	BGIOSGA034240	XP_015688130.1 probable sucrose-phosphate synthase 1
TCONS_00007988	-0.198654	0.083624	0.48854	BGIOSGA006493	XP_006648632.1 riboflavin biosynthesis protein PYRR, chloroplastic isoform X1
TCONS_00016471	-0.198535	0.0497247	-0.5268	BGIOSGA005836	XP_006652691.1 mitochondrial import inner membrane translocase subunit Tim13-like
TCONS_00008841	-0.198485	-0.796915	-0.190401	BGIOSGA005575	XP_006647944.1 armadillo repeat-containing protein 6 isoform X2
TCONS_00017060	-0.198471	-0.0189175	0.520319	BGIOSGA030955	XP_006654002.2 glutamate--cysteine ligase B, chloroplastic
TCONS_00033859	-0.198237	-1.58808	-0.667106	BGIOSGA027080	XP_015697811.1 protein ELF4-LIKE 4-like
TCONS_00011136	-0.198015	-1.40804	0.208979	BGIOSGA025284	XP_006651911.1 protein WVD2-like 1
TCONS_00008682	-0.197983	0.0566029	0.110921	BGIOSGA005748	XP_022679424.1 galactan beta-1,4-galactosyltransferase GALS1-like
TCONS_00033362	-0.197887	0.484606	-0.105543	BGIOSGA016174	XP_006650241.2 ervatamin-C-like
TCONS_00008091	-0.197835	0.112438	0.0984261	BGIOSGA006361	XP_015689538.1 protein BONZAI 1-like
TCONS_00023965	-0.197829	-0.498179	-0.144715	BGIOSGA009901	XP_004958115.1vacuolar protein sorting-associated protein 62
TCONS_00026865	-0.197712	0.562391	-0.105982	BGIOSGA023434	XP_002445949.1nuclear transcription factor Y subunit C-6
TCONS_00013223	-0.197694	0.558617	-0.551109	BGIOSGA029649	XP_015690086.1 vegetative cell wall protein gp1-like
TCONS_00002633	-0.197668	-0.596875	0.212659	BGIOSGA004058	XP_006646740.2 AP3-complex subunit beta-A
TCONS_00022884	-0.197553	-0.273459	0.870572	BGIOSGA020818	XP_006656474.1 probable isoprenylcysteine alpha-carbonyl methylesterase ICMEL1 isoform X1
TCONS_00012800	-0.197295	-1.20117	-1.29287	BGIOSGA027705	XP_006650337.1 BTB/POZ domain-containing protein At1g30440
TCONS_00026937	-0.197218	-1.51623	-1.10723	BGIOSGA028988	XP_004973897.1serine/threonine-protein phosphatase 7
TCONS_00005801	-0.19702	0.122961	1.42266	BGIOSGA021163	XP_004951268.1BTB/POZ domain-containing protein POB1 isoform X1
TCONS_00025132	-0.196422	0.620161	0.238645	BGIOSGA012025	XP_010238113.1ras-related protein RABH1b
TCONS_00006499	-0.196324	0.0247585	0.410922	BGIOSGA016779	XP_006647553.1PREDICTED: uncharacterized protein At2g24330-like
TCONS_00008160	-0.196206	-0.217104	-1.02987	BGIOSGA036558	XP_006647358.1 V-type proton ATPase 16 kDa proteolipid subunit-like
TCONS_00006672	-0.196152	-0.850181	-0.267234	BGIOSGA017015	XP_024318338.1uncharacterized protein LOC100830018
TCONS_00001370	-0.196141	-1.45012	0.693434	BGIOSGA004000	XP_006646105.1 psbP domain-containing protein 5, chloroplastic
TCONS_00005632	-0.19609	-0.387545	-0.28741	BGIOSGA020913	XP_006647039.2 SEC14-like protein 1
TCONS_00006726	-0.196082	0.160193	-0.500712	BGIOSGA008896	XP_006647775.1 RING-box protein 1
TCONS_00007255	-0.196038	0.504967	0.276196	BGIOSGA030523	XP_006648247.1 bifunctional protein FolD 4, chloroplastic
TCONS_00012031	-0.196014	0.25316	0.102353	BGIOSGA019077	XP_002465447.1calcium-transporting ATPase 4, endoplasmic reticulum-type
TCONS_00022090	-0.195758	-0.887578	-0.586018	BGIOSGA008736	XP_003563941.1protein kinase G11A
TCONS_00005835	-0.195714	-0.625148	-0.658545	BGIOSGA007961	XP_006647158.1 NADPH:adrenodoxin oxidoreductase, mitochondrial
TCONS_00016079	-0.195655	0.382748	0.280533	BGIOSGA006144	XP_006652403.1 60S ribosomal protein L6-3-like
TCONS_00009287	-0.195113	0.0756195	-1.29507	BGIOSGA014738	XP_006649383.1PREDICTED: uncharacterized protein LOC102721906
TCONS_00000283	-0.19493	-0.731644	-0.109018	BGIOSGA002860	XP_015688240.1 angiogenic factor with G patch and FHA domains 1 isoform X1
TCONS_00026628	-0.194927	-0.156229	0.570253	BGIOSGA014941	XP_015695605.1 betaine aldehyde dehydrogenase 2
TCONS_00024862	-0.194385	-1.00715	0.970958	BGIOSGA032270	XP_015689772.1 3-ketoacyl-CoA synthase 20-like
TCONS_00036348	-0.194277	0.108821	-1.03214	BGIOSGA025602	XP_015698717.1PREDICTED: uncharacterized protein LOC102712766
TCONS_00024425	-0.194175	-0.908351	-0.096847	BGIOSGA024984	XP_015694991.1 probable tetraacyldisaccharide 4'-kinase, mitochondrial
TCONS_00003997	-0.194158	0.350308	-0.423195	BGIOSGA015423	XP_002458167.2ATP-dependent zinc metalloprotease FTSH 9, chloroplastic/mitochondrial
TCONS_00012266	-0.194149	0.252486	-0.0560346	BGIOSGA000164	XP_015690949.1 lipase-like isoform X1
TCONS_00020210	-0.193874	-0.0644593	-0.738495	BGIOSGA005164	XP_014660472.1probable calcium-binding protein CML30
TCONS_00010605	-0.19383	-0.263945	-0.449271	BGIOSGA015356	XP_015689997.1 myosin-binding protein 7-like
TCONS_00036605	-0.193708	1.44897	-0.000551291	BGIOSGA001074	XP_004977445.1NDR1/HIN1-like protein 13
TCONS_00025079	-0.19367	-0.405232	-1.37916	BGIOSGA033384	XP_006657686.1 expansin-like A3 isoform X2
TCONS_00025326	-0.193596	-1.30904	-0.907708	BGIOSGA024103	XP_012698871.1equilibrative nucleotide transporter 3 isoform X2
TCONS_00004057	-0.193416	-0.905729	-1.46419	#N/A	#N/A
TCONS_00017645	-0.193411	0.666748	-0.0519672	BGIOSGA019707	XP_006655234.1PREDICTED: uncharacterized protein LOC102711535
TCONS_00003923	-0.193226	-1.19961	0.415005	BGIOSGA005566	XP_006649069.1 cytoplasmic tRNA 2-thiolation protein 1
TCONS_00020882	-0.193224	-1.76757	1.3501	BGIOSGA023090	XP_010227607.1uncharacterized protein LOC100843946
TCONS_00011546	-0.193121	-0.757602	-0.114459	BGIOSGA011477	NP_001140304.1uncharacterized LOC100272349
TCONS_00018616	-0.19298	-0.270789	-0.214583	BGIOSGA036072	XP_015693327.1 VIN3-like protein 1
TCONS_00024388	-0.192923	0.215312	-0.0025084	BGIOSGA011155	XP_015695040.1 transcription factor MYB108-like
TCONS_00008115	-0.192809	-0.412379	1.20768	BGIOSGA006335	XP_015688619.1 poly [ADP-ribose] polymerase 3
TCONS_00019557	-0.192679	-1.55657	-1.14868	BGIOSGA020526	XP_020394449.1putative bZIP transcription factor superfamily protein isoform X1
TCONS_00036161	-0.192652	0.0451059	0.556977	BGIOSGA037517	XP_015698701.1 protein RTF1 homolog

TCONS_00020145	-0.192418	0.139897	-0.964107	BGIOSGA036462	XP_015694431.1PREDICTED: uncharacterized protein ycf45
TCONS_00004720	-0.192298	-0.00712677	0.122277	BGIOSGA000496	XP_015688218.1 transcription initiation factor TFIID subunit 12b isoform X2
TCONS_00000378	-0.192132	0.550598	0.504537	BGIOSGA002953	XP_002457170.2nuclear pore complex protein NUP35
TCONS_00009586	-0.192084	-0.407235	-0.897681	BGIOSGA032938	XP_006649653.2 homocysteine S-methyltransferase 1
TCONS_00033437	-0.19206	0.140223	0.201796	BGIOSGA029039	XP_010237731.1FHA domain-containing protein FHA2
TCONS_00022641	-0.191864	0.0911764	0.1528	BGIOSGA020804	XP_006657182.1 ELKS/Rab6-interacting/CAST family member 1
TCONS_00007818	-0.191709	-0.0284817	-0.295705	BGIOSGA021301	XP_003572668.1phosphatidylinositol 4-kinase gamma 4
TCONS_00002047	-0.191688	0.525503	0.404275	BGIOSGA004681	XP_022682190.1 probable ADP-ribosylation factor GTPase-activating protein AGD14
TCONS_00022951	-0.191252	0.325017	0.433587	BGIOSGA020496	XP_006656529.2PREDICTED: uncharacterized protein LOC102707973
TCONS_00013576	-0.191076	0.0912039	0.471997	BGIOSGA009400	XP_006652048.1PREDICTED: uncharacterized protein LOC102715565
TCONS_00015812	-0.191067	#NA	-3.70756	BGIOSGA015170	XP_004975474.1protein PYRICULARIA ORYZAE RESISTANCE 21
TCONS_00021276	-0.190802	-0.0365807	-0.537118	BGIOSGA001794	XP_006656404.2 probable glucuronosyltransferase Os06g0687900
TCONS_00000003	-0.19076	0.381424	0.267349	BGIOSGA016906	XP_004967891.1uncharacterized protein LOC101753816
TCONS_00011088	-0.190695	-0.422756	0.134966	BGIOSGA001345	XP_006650698.1 cullin-4
TCONS_00005170	-0.190652	-0.302123	-0.123625	BGIOSGA000035	XP_015688132.1 UDP-D-apiose/UDP-D-xylose synthase
TCONS_00009356	-0.190624	-0.202623	-0.46073	BGIOSGA011905	XP_006649432.1 pentatricopeptide repeat-containing protein At2g30780-like
TCONS_00008100	-0.190555	0.212759	0.727673	BGIOSGA004544	NP_001146865.1SAM domain family protein
TCONS_00018653	-0.190329	0.0838532	-0.553781	BGIOSGA018782	XP_006654063.1 mediator of RNA polymerase II transcription subunit 28
TCONS_00023970	-0.190302	-0.448716	0.048127	BGIOSGA007921	XP_015694700.1 glucan endo-1,3-beta-glucosidase 4-like
TCONS_00022427	-0.190293	-0.631844	0.525134	BGIOSGA012052	XP_015694434.1 deSI-like protein At4g17486 isoform X2
TCONS_00001666	-0.190263	1.627	1.16492	BGIOSGA004301	XP_006644589.1 glycylpeptide N-tetradecanoyltransferase 1
TCONS_00018712	-0.190105	-2.42843	-2.29266	BGIOSGA033504	XP_006654097.1 probable cellulose synthase A catalytic subunit 1 [UDP-forming]
TCONS_00029385	-0.190093	0.480074	-0.0619131	BGIOSGA033267	XP_015692007.1 zinc finger CCCH domain-containing protein 19-like isoform X2
TCONS_00022567	-0.190047	0.557257	0.859894	BGIOSGA017313	XP_006657138.1 zinc finger CCCH domain-containing protein 44
TCONS_00009225	-0.190026	-3.11027	0.53779	BGIOSGA013966	XP_015690910.1 probable polygalacturonase
TCONS_00029142	-0.189984	0.0072444	-0.61575	BGIOSGA030893	XP_015696563.1 RHOMBOID-like protein 10, chloroplastic
TCONS_00007395	-0.189926	0.0657021	-0.0113901	BGIOSGA007344	XP_006646913.1 40S ribosomal protein S14
TCONS_00029397	-0.18972	-0.788723	-0.251464	BGIOSGA029569	XP_008811147.1 heat shock protein 81-1-like
TCONS_00028327	-0.189392	-0.579289	0.915353	BGIOSGA026652	XP_003574772.1putative L-ascorbate peroxidase 6 isoform X1
TCONS_00034730	-0.189325	-0.356259	-0.624407	BGIOSGA010944	XP_006662879.1 phosphoinositide phosphatase SAC7-like
TCONS_00023644	-0.189312	-1.99398	-0.935468	BGIOSGA025666	XP_015694628.1 probable plastid-lipid-associated protein 12, chloroplastic
TCONS_00004809	-0.189209	0.143981	0.393753	BGIOSGA006274	XP_015688156.1PREDICTED: uncharacterized protein LOC102705495
TCONS_00005375	-0.189169	0.807728	0.0938002	BGIOSGA007486	XP_015689426.1 splicing factor 3B subunit 3-like
TCONS_00022958	-0.188939	-0.435285	-0.378821	BGIOSGA024993	XP_006665128.1PREDICTED: uncharacterized protein LOC102716375
TCONS_00037013	-0.188563	0.344906	-0.821311	BGIOSGA035829	XP_006663950.1 50S ribosomal protein HLP, mitochondrial-like
TCONS_00010637	-0.188556	-0.173512	-0.491374	BGIOSGA010106	XP_012704366.1uncharacterized protein LOC101779182, partial
TCONS_00010795	-0.188467	-0.567243	-1.00394	BGIOSGA032579	NP_001151096.1integral membrane protein DUF6 containing protein
TCONS_00030860	-0.188322	-0.0492919	-0.280619	BGIOSGA032665	XP_003581121.1ubiquitin-fold modifier-conjugating enzyme 1
TCONS_00001592	-0.188294	0.346992	0.373051	BGIOSGA010684	XP_004969576.1EEF1A lysine methyltransferase 2
TCONS_00006970	-0.18829	0.895596	0.737459	BGIOSGA009153	XP_003570699.2RRP12-like protein
TCONS_00016334	-0.188283	-0.468597	0.570964	BGIOSGA027744	XP_015692334.1 trihelix transcription factor GTL1-like isoform X2
TCONS_00019032	-0.188157	0.371215	0.0610923	BGIOSGA029400	XP_006664965.1PREDICTED: uncharacterized protein LOC102715817 isoform X1
TCONS_00008609	-0.188147	-0.660866	-1.47853	BGIOSGA022026	XP_003570124.1BOI-related E3 ubiquitin-protein ligase 1
TCONS_00030213	-0.188012	-0.305439	-0.656214	BGIOSGA029596	XP_015696792.1 pentatricopeptide repeat-containing protein At1g76280 isoform X1
TCONS_00023324	-0.188007	0.00672255	-0.358135	BGIOSGA025365	XP_015694557.1 zeta-carotene desaturase, chloroplastic/chromoplastic
TCONS_00014331	-0.187874	0.135789	0.686067	BGIOSGA015612	XP_006652336.2 D-galacturonate reductase-like
TCONS_00011015	-0.187851	-0.269879	0.580076	BGIOSGA016402	XP_006650641.1 upstream activation factor subunit spp27-like
TCONS_00004450	-0.187747	0.0326748	-0.0534779	BGIOSGA000772	XP_006644750.1PREDICTED: uncharacterized protein C9orf85 homolog
TCONS_00006131	-0.187576	0.272083	0.578083	BGIOSGA016278	NP_001151761.2acetolactate synthase 1, chloroplastic
TCONS_00035656	-0.187526	0.126608	-0.0199428	BGIOSGA019988	XP_003578848.1O-fucosyltransferase 9 isoform X2
TCONS_00037336	-0.187491	-0.274798	0.0514915	BGIOSGA036032	XP_002265489.2 universal stress protein PHOS34

TCONS_00026527	-0.187408	2.19159	1.02376	BGIOSGA040488	XP_015696060.1 serine/arginine-rich splicing factor SR45a-like isoform X2
TCONS_00030025	-0.187406	0.448041	0.919432	BGIOSGA033437	XP_006660614.1 U4/U6.U5 tri-snRNP-associated protein 2-like
TCONS_00025111	-0.187404	-0.248721	0.594819	BGIOSGA008866	XP_006658566.1 calmodulin-binding transcription activator 6-like
TCONS_00007666	-0.187141	0.556958	-0.48249	BGIOSGA009469	XP_006647108.1 shaggy-related protein kinase eta
TCONS_00015020	-0.18688	-0.0720513	0.874869	BGIOSGA012521	XP_008661703.1uncharacterized protein LOC100275650 isoform X6
TCONS_00018155	-0.186633	0.261865	0.72856	BGIOSGA000845	XP_015693357.1 protein cereblon-like
TCONS_00023436	-0.186525	-1.3224	-1.67528	BGIOSGA002599	XP_003557327.1probable cellulose synthase A catalytic subunit 6 [UDP-forming]
TCONS_00013997	-0.186242	-0.807309	-1.79979	BGIOSGA040253	XP_006652179.1 probable aldo-keto reductase 2
TCONS_00009398	-0.186217	-0.502778	0.222586	BGIOSGA011948	XP_006649470.1 3-dehydroquinate synthase homolog isoform X2
TCONS_00025020	-0.186112	-0.166717	0.381575	BGIOSGA035060	XP_015695224.1 non-lysosomal glucosylceramidase-like isoform X2
TCONS_00037264	-0.186002	1.1112	-0.00344921	BGIOSGA036114	XP_003576025.1F-box protein At3g12350
TCONS_00016801	-0.185973	0.236927	-1.5413	BGIOSGA014165	XP_015691606.1 GTP cyclohydrolase 1
TCONS_00004973	-0.185707	-1.75177	-1.12166	BGIOSGA000245	XP_015690767.1 protein XRI1-like isoform X4
TCONS_00007241	-0.185535	-0.324712	-0.534129	BGIOSGA006640	XP_006646786.1 DNA-directed RNA polymerase II subunit 4
TCONS_00012165	-0.185518	-1.90922	-0.388923	BGIOSGA034729	XP_015690842.1 alpha-L-arabinofuranosidase 1-like isoform X1
TCONS_00027517	-0.18547	-1.28179	-0.00477158	BGIOSGA027506	XP_022683089.1putative F-box/LRR-repeat protein 22
TCONS_00020610	-0.185391	0.342666	0.281273	BGIOSGA022795	XP_006656013.1 E3 ubiquitin-protein ligase makorin
TCONS_00022874	-0.185303	-0.503819	-0.319101	BGIOSGA007712	XP_021305681.1pentatricopeptide repeat-containing protein At1g74600, chloroplastic
TCONS_00006737	-0.185299	0.888041	0.0016151	BGIOSGA008908	XP_015689382.1 heat shock 70 kDa protein 17
TCONS_00000063	-0.185215	0.480614	-0.28874	BGIOSGA022966	XP_006645438.1 protein decapping 5-like
TCONS_00004455	-0.185176	0.206751	0.14727	BGIOSGA000771	XP_006644751.1PREDICTED: uncharacterized protein LOC102705673
TCONS_00004998	-0.185142	1.31503	0.545948	BGIOSGA036278	XP_006645258.2 disease resistance protein RPP13-like
TCONS_00022614	-0.185049	0.907127	1.02179	BGIOSGA035350	XP_006657164.1 plant UBX domain-containing protein 4-like
TCONS_00017195	-0.184985	0.0658462	-0.0188087	BGIOSGA019237	XP_015693174.1 arginine--tRNA ligase, cytoplasmic-like isoform X1
TCONS_00009425	-0.184984	0.462083	0.722235	BGIOSGA011976	XP_006649496.1 tubulin--tyrosine ligase-like protein 12
TCONS_00021784	-0.184915	0.326986	-0.0582828	BGIOSGA009155	XP_015694201.1 phosphoinositide phosphatase SAC3-like isoform X1
TCONS_00029088	-0.184643	-0.301104	0.21035	BGIOSGA035158	NP_001333458.1uncharacterized LOC103632816
TCONS_00034378	-0.18452	0.306307	-0.131175	BGIOSGA018529	XP_015697571.1PREDICTED: uncharacterized protein LOC102717843, partial
TCONS_00010222	-0.184476	-0.202898	-0.280485	BGIOSGA012786	XP_006651458.1 F-box protein At1g78280
TCONS_00026962	-0.184384	0.341457	0.651876	BGIOSGA030263	XP_006659607.2 RNA pseudouridine synthase 3, mitochondrial
TCONS_00018416	-0.184071	-0.154629	0.936444	BGIOSGA001256	XP_015693305.1 transmembrane protein 33 homolog
TCONS_00034949	-0.183856	0.632919	-0.347551	BGIOSGA037317	XP_006661053.1 E3 ubiquitin-protein ligase UPL1-like
TCONS_00029525	-0.183796	-0.298396	-0.00269946	BGIOSGA031281	XP_006660990.2 nuclear pore complex protein GP210 isoform X2
TCONS_00002680	-0.183788	-0.210599	0.0372283	BGIOSGA002541	XP_021310910.1uncharacterized protein LOC8079087 isoform X1
TCONS_00028306	-0.183788	0.0662409	0.905984	BGIOSGA031025	XP_006659613.1 30S ribosomal protein S16
TCONS_00036223	-0.183459	0.309972	-1.5984	BGIOSGA011066	XP_021301302.1calmodulin-binding protein 60 D
TCONS_00030289	-0.183371	0.600764	0.537163	BGIOSGA037382	XP_015696399.1 NAC domain-containing protein 16-like
TCONS_00034061	-0.183326	0.545113	-0.621789	BGIOSGA015551	XP_006663115.1 probable LRR receptor-like serine/threonine-protein kinase At3g47570
TCONS_00017265	-0.183298	0.0450792	0.454491	BGIOSGA019307	XP_006654129.2 sister-chromatid cohesion protein 3
TCONS_00005639	-0.18328	-0.0621577	0.174224	BGIOSGA026511	XP_003571494.1cyanidin 3-O-rutinoside 5-O-glucosyltransferase
TCONS_00024210	-0.183173	-0.660123	-0.0902827	BGIOSGA026249	XP_006658891.1 cytochrome P450 78A4-like
TCONS_00010946	-0.183002	-0.825468	-0.184917	BGIOSGA013548	XP_015690063.1 autophagy-related protein 16
TCONS_00007296	-0.182994	-2.54794	0.52966	BGIOSGA024510	XP_006646836.1 metal transporter NRAT1
TCONS_00007362	-0.182969	0.609432	0.601326	BGIOSGA022189	XP_006646896.1 DNA-directed RNA polymerase V subunit 1
TCONS_00034501	-0.182924	-0.314183	-1.16271	BGIOSGA034322	XP_003577269.1probable LRR receptor-like serine/threonine-protein kinase At3g47570
TCONS_00026950	-0.182861	-1.91936	-0.42869	BGIOSGA016462	NP_001149948.1KID-containing protein
TCONS_00007119	-0.182847	0.264395	-0.149495	BGIOSGA000020	XP_006649176.1 pumilio homolog 1-like
TCONS_00005828	-0.1825	0.337736	1.01133	BGIOSGA037028	XP_006647154.1PREDICTED: uncharacterized protein LOC102721614
TCONS_00023341	-0.182412	-0.102749	0.109592	BGIOSGA025382	XP_006658391.2 DNA-binding protein EMBP-1-like
TCONS_00013798	-0.18232	0.0617553	0.170796	BGIOSGA022835	XP_003566733.2glycosyltransferase-like At2g41451
TCONS_00023408	-0.182034	1.07412	0.162162	BGIOSGA024545	XP_004955928.1F-box/kelch-repeat protein At3g23880
TCONS_00013368	-0.181873	0.250816	0.262685	BGIOSGA011932	XP_015690115.1PREDICTED: uncharacterized protein LOC102701437, partial
TCONS_00022889	-0.181849	-0.226117	0.925035	BGIOSGA026306	XP_003563543.1uncharacterized protein LOC100829885
TCONS_00006555	-0.181798	-0.625201	-0.922153	BGIOSGA008721	XP_006648884.1 ATP-dependent zinc metalloprotease FTSH 7, chloroplastic

TCONS_00011862	-0.181346	-0.318876	0.429903	BGIOSGA032555	XP_006651206.1PREDICTED: uncharacterized protein LOC102716394
TCONS_00005759	-0.181105	-0.172049	0.568875	BGIOSGA002151	XP_015688535.1 polyadenylate-binding protein-interacting protein 8-like
TCONS_00020083	-0.180963	-0.0581791	0.762139	BGIOSGA002885	XP_006655769.1PREDICTED: uncharacterized protein LOC102721844 isoform X2
TCONS_00020131	-0.180789	-0.43868	0.142324	BGIOSGA022331	XP_006655795.1 DNA repair protein RadA homolog
TCONS_00004862	-0.180713	-0.503343	0.560522	BGIOSGA000358	XP_006646549.1 serine/threonine-protein kinase rio2
TCONS_00014125	-0.180458	0.161296	-0.124699	BGIOSGA038099	XP_006652215.1 2-oxoglutarate dehydrogenase, mitochondrial-like
TCONS_00017296	-0.180333	-1.24993	#NA	BGIOSGA004895	XP_002439433.2 lachrymatory-factor synthase
TCONS_00003972	-0.180332	-0.0744623	-0.168059	BGIOSGA001967	XP_006644362.1PREDICTED: uncharacterized protein LOC102708475
TCONS_00006408	-0.180292	0.76193	-0.278134	BGIOSGA016643	XP_015689305.1 cyclin-dependent kinase G-1
TCONS_00019566	-0.180203	-0.495999	-0.0750079	BGIOSGA035871	XP_015693332.1 glyoxysomal processing protease, glyoxysomal
TCONS_00011403	-0.179738	-0.107015	-0.316645	BGIOSGA036283	XP_006663986.1PREDICTED: uncharacterized protein LOC102709889 isoform X1
TCONS_00014352	-0.179596	-0.893409	-1.12858	BGIOSGA006529	XP_010491788.1 histone H3.3
TCONS_00011358	-0.179544	-0.384596	-0.305581	BGIOSGA031910	XP_006652039.1 heat stress transcription factor A-1
TCONS_00005018	-0.179533	-3.57287	-0.581098	BGIOSGA000199	XP_015699164.1 serine/threonine-protein kinase CDL1-like isoform X2
TCONS_00018397	-0.179517	0.201571	0.305718	BGIOSGA020449	XP_020394635.1uncharacterized LOC100381615 isoform X1
TCONS_00019377	-0.179098	-0.113993	0.690832	BGIOSGA026536	NP_001131692.1uncharacterized LOC100193052
TCONS_00004447	-0.178951	-0.804016	-0.694975	BGIOSGA000775	XP_006644746.1PREDICTED: uncharacterized protein LOC102704013
TCONS_00029024	-0.178947	-6.39026	-0.993183	BGIOSGA025923	XP_006660635.1 cellulose synthase A catalytic subunit 9 [UDP-forming]
TCONS_00002597	-0.178834	0.125365	-0.197461	BGIOSGA014051	XP_004971373.2PH, RCC1 and FYVE domains-containing protein 1 isoform X2
TCONS_00031287	-0.178751	0.108627	-0.0320443	BGIOSGA033101	XP_006661848.1 peroxisome biogenesis protein 12 isoform X1
TCONS_00022982	-0.17831	-0.197156	0.200018	BGIOSGA002861	XP_006657396.1 phytosulfokine receptor 2
TCONS_00036748	-0.17807	-0.746801	0.519389	BGIOSGA025477	XP_004977302.1exocyst complex component EXO70B1
TCONS_00025288	-0.178007	-0.0426866	-0.291573	BGIOSGA024138	XP_015694676.1 peptide chain release factor PrfB1, chloroplastic-like
TCONS_00007337	-0.177781	0.530102	0.0344177	BGIOSGA008208	XP_006646877.2 splicing factor 3B subunit 1
TCONS_00002983	-0.177729	0.0801952	-0.134947	BGIOSGA002235	XP_015699272.1 serine/threonine-protein kinase 38
TCONS_00000350	-0.177617	-0.537312	-0.169233	BGIOSGA014832	XP_022682500.1clavaminate synthase-like protein At3g21360
TCONS_00010250	-0.177581	-0.253415	0.15099	BGIOSGA012812	XP_004984095.1E3 ubiquitin-protein ligase EL5
TCONS_00009226	-0.177525	-0.0939102	1.23499	BGIOSGA011756	XP_015691154.1 PIN2/TERF1-interacting telomerase inhibitor 1 isoform X2
TCONS_00036361	-0.177427	-0.36463	0.0868871	BGIOSGA000725	XP_006664165.1 MATH domain-containing protein At5g43560-like
TCONS_00025797	-0.1773	0.69305	0.8047	BGIOSGA027861	XP_006656946.1 50S ribosomal protein L30
TCONS_00034471	-0.177242	0.592447	0.26048	BGIOSGA003061	XP_006662807.1 TOM1-like protein 2
TCONS_00009221	-0.177238	1.00756	-1.2347	BGIOSGA012861	XP_006649302.1 homeobox protein BEL1 homolog
TCONS_00002578	-0.177065	0.58786	0.0641475	BGIOSGA005221	XP_004971334.1probable gamma-secretase subunit PEN-2
TCONS_00001466	-0.176924	-0.32582	0.279093	BGIOSGA004104	XP_003569452.1uncharacterized protein LOC100830426
TCONS_00018134	-0.176878	-0.265191	-0.542363	BGIOSGA031780	XP_006654649.1 ras-related protein Rab7 isoform X2
TCONS_00023063	-0.17677	-0.867673	-0.492007	BGIOSGA033444	XP_006661064.1 hexose carrier protein HEX6-like
TCONS_00014941	-0.176755	2.89812	2.10517	BGIOSGA017119	XP_006653765.2 protein argonaute 2-like
TCONS_00006256	-0.176752	0.175656	-0.17732	BGIOSGA008417	XP_006647368.2PREDICTED: uncharacterized protein LOC102714703
TCONS_00016922	-0.176744	0.00856177	0.63433	BGIOSGA016419	XP_006653070.1 cationic peroxidase SPC4-like
TCONS_00005918	-0.176517	-0.852992	0.402082	BGIOSGA008053	XP_010234565.1uncharacterized protein LOC100822445
TCONS_00030225	-0.176477	-0.594874	0.330594	BGIOSGA029582	XP_002462536.1uncharacterized protein LOC8063750
TCONS_00025873	-0.176416	1.70859	0.148088	BGIOSGA027945	XP_006659801.1PREDICTED: uncharacterized protein LOC102706676 isoform X2
TCONS_00000609	-0.176409	-0.0959215	-0.286011	BGIOSGA003193	XP_015688046.1PREDICTED: uncharacterized protein LOC102699906 isoform X2
TCONS_00012696	-0.176102	-1.00361	-0.594809	BGIOSGA013095	XP_002461077.2uncharacterized protein LOC8079369
TCONS_00015826	-0.176072	-1.25532	-0.174697	BGIOSGA035124	XP_006652240.1 probable BOI-related E3 ubiquitin-protein ligase 3
TCONS_00023184	-0.175819	-0.395875	-2.38732	BGIOSGA025224	XP_003557525.1protein MET1, chloroplastic
TCONS_00013105	-0.175773	-0.875287	-0.138462	BGIOSGA010318	XP_015690048.1 GTP-binding protein At3g49725, chloroplastic
TCONS_00031786	-0.175773	-0.0196773	0.276576	#N/A	#N/A
TCONS_00033040	-0.175425	-0.118632	1.03315	BGIOSGA034876	XP_006647316.1PREDICTED: uncharacterized protein LOC102700210
TCONS_00018338	-0.175125	-0.00275677	-0.338327	BGIOSGA020389	XP_015693307.1 common plant regulatory factor 1-like
TCONS_00023244	-0.175036	-0.300074	-0.360873	BGIOSGA013735	XP_006657512.1 protein WVD2-like 1
TCONS_00021808	-0.174683	0.0648576	-1.27125	BGIOSGA007401	XP_006655893.1 choline/ethanolaminephosphotransferase 1-like
TCONS_00017344	-0.174669	-1.62482	-2.27129	BGIOSGA002531	XP_015698944.1PREDICTED: uncharacterized protein LOC102718237
TCONS_00000711	-0.174587	1.66881	1.77582	BGIOSGA012235	XP_006644089.1 protein ROOT PRIMORDIUM DEFECTIVE 1

TCONS_00012131	-0.17456	-0.534758	0.406517	BGIOSGA010877	XP_006649970.1PREDICTED: uncharacterized protein LOC102701065
TCONS_00014162	-0.174534	-0.0873326	0.0994736	BGIOSGA032223	XP_006652232.2 probable glucuronosyltransferase Os04g0398600
TCONS_00020267	-0.174502	-0.179453	0.170253	BGIOSGA022457	XP_006656727.1 pre-mRNA-splicing factor ATP-dependent RNA helicase DEAH10
TCONS_00012350	-0.174161	-1.31662	0.266821	BGIOSGA026659	XP_006650136.2 probable mannan synthase 5
TCONS_00007779	-0.174146	0.688308	-1.15989	BGIOSGA006383	XP_022680572.1galactoside 2-alpha-L-fucosyltransferase isoform X2
TCONS_00005129	-0.174135	0.253969	-0.579518	BGIOSGA000075	XP_015691084.1 phosphatidylserine decarboxylase proenzyme 2
TCONS_00004721	-0.174122	-1.11172	0.679284	BGIOSGA030356	XP_006645005.1 CASP-like protein 5B1
TCONS_00016767	-0.174037	0.474937	0.244014	#N/A	#N/A
TCONS_00018791	-0.174029	-0.405133	-0.850241	BGIOSGA010300	XP_023156864.1uncharacterized protein LOC103638887
TCONS_00014302	-0.173862	0.174857	0.94911	BGIOSGA004260	XP_006653425.1 probable pectinesterase/pectinesterase inhibitor 51
TCONS_00008638	-0.173803	0.679775	-1.536	BGIOSGA021182	XP_006648963.1 ubiquinol oxidase 1a, mitochondrial-like
TCONS_00008425	-0.173751	-1.25824	-1.18045	BGIOSGA004358	XP_006644638.1 putative disease resistance protein RGA3 isoform X1
TCONS_00013550	-0.173746	-2.31072	0.550688	BGIOSGA009425	XP_010232041.1uncharacterized protein At5g65660 isoform X2
TCONS_00030216	-0.173578	0.576118	-0.180874	BGIOSGA010814	XP_014757402.1O-fucosyltransferase 34
TCONS_00005274	-0.173525	-0.671406	-0.477236	BGIOSGA011520	XP_006646781.1 catalase isozyme A-like
TCONS_00021716	-0.173447	-0.571393	1.4423	BGIOSGA021805	XP_004964716.1ran-binding protein 9
TCONS_00024602	-0.173399	0.0969359	-0.0747578	BGIOSGA037907	XP_006655722.1PREDICTED: uncharacterized protein LOC102708063 isoform X1
TCONS_00022870	-0.173145	-1.07846	-0.321323	BGIOSGA020574	XP_015694256.1 delta-aminolevulinic acid dehydratase, chloroplastic
TCONS_00016634	-0.172767	0.438784	-0.756507	BGIOSGA014328	XP_015691991.1 ABC transporter C family member 2-like
TCONS_00003225	-0.172608	-0.36503	-1.07397	BGIOSGA017549	XP_015688306.1PREDICTED: uncharacterized protein LOC102713223
TCONS_00015081	-0.172435	0.0661431	-0.319752	BGIOSGA007496	XP_006652933.1 vacuolar cation/proton exchanger 3
TCONS_00017903	-0.172432	-0.347771	0.183414	BGIOSGA019947	XP_006655366.2PREDICTED: uncharacterized protein LOC102707680
TCONS_00024329	-0.172376	-0.0552663	-0.0690254	BGIOSGA010755	XP_015695239.1PREDICTED: uncharacterized protein LOC102705092
TCONS_00024282	-0.172357	0.504667	-0.451406	BGIOSGA023908	XP_006658072.1 elongation factor 1-beta
TCONS_00032932	-0.172228	0.036361	0.238257	BGIOSGA015044	XP_006660823.1PREDICTED: uncharacterized protein LOC102703243
TCONS_00017734	-0.171881	-0.0911792	-0.334569	BGIOSGA019795	XP_006654353.1 probable folate-biopterin transporter 7
TCONS_00005480	-0.171844	-0.475647	-1.24173	BGIOSGA033648	XP_006647006.1 disease resistance protein RPM1-like
TCONS_00019104	-0.171597	-0.302764	1.75508	BGIOSGA018256	XP_006654275.2 phagocyte signaling-impaired protein
TCONS_00005172	-0.171512	-0.935796	0.52797	BGIOSGA000064	XP_024314581.1uncharacterized protein LOC100835523, partial
TCONS_00025096	-0.171327	-0.66549	0.0270892	BGIOSGA024303	XP_006658559.2 E3 ubiquitin protein ligase RIN2-like
TCONS_00016412	-0.171208	-0.360962	-0.498509	BGIOSGA023366	XP_003580318.2lysine-specific histone demethylase 1 homolog 3
TCONS_00006845	-0.171037	-1.25644	-0.539955	BGIOSGA028870	XP_015689320.1 DExH-box ATP-dependent RNA helicase DExH15 chloroplastic isoform X1
TCONS_00019618	-0.170794	0.179592	-0.0894483	BGIOSGA033125	XP_006654631.1 serine/threonine-protein kinase tricorner-like
TCONS_00021594	-0.170757	-0.0804144	1.00986	BGIOSGA002264	XP_006655777.1 probable 1-deoxy-D-xylulose-5-phosphate synthase, chloroplastic isoform X1
TCONS_00027099	-0.170715	-1.22592	0.287947	BGIOSGA034849	XP_015696038.1PREDICTED: uncharacterized protein LOC102715781
TCONS_00029197	-0.170632	0.0573979	0.30289	BGIOSGA030942	XP_004957070.1protein SCAI homolog isoform X1
TCONS_00031293	-0.17054	-2.56452	-1.75698	BGIOSGA024994	XP_003571861.1protein NRT1/ PTR FAMILY 5.2
TCONS_00016178	-0.17048	-0.915987	-0.0419907	BGIOSGA037451	XP_006664049.1 ubiquitin-like-conjugating enzyme ATG10
TCONS_00016843	-0.170462	-1.13041	-0.438421	BGIOSGA014123	XP_006653005.1 probable polyamine oxidase 4
TCONS_00017166	-0.170454	-1.49805	-0.454794	BGIOSGA004652	XP_006654067.2 heparan-alpha-glucosaminide N-acetyltransferase-like
TCONS_00002046	-0.170449	0.159493	0.475422	BGIOSGA020256	XP_010232480.1 probable ADP-ribosylation factor GTPase-activating protein AGD14
TCONS_00008564	-0.170352	0.0922205	0.518577	BGIOSGA005860	XP_006647671.1PREDICTED: uncharacterized protein LOC102708409
TCONS_00027543	-0.170209	-0.990238	-0.405443	BGIOSGA027468	XP_015695749.1 equilibrative nucleotide transporter 1-like
TCONS_00010334	-0.170151	1.14668	0.349039	BGIOSGA012907	XP_004983991.140S ribosomal protein S19
TCONS_00001682	-0.169989	-1.37447	-0.851138	BGIOSGA037727	XP_006644608.1 protein REVERSION-TO-ETHYLENE SENSITIVITY1-like
TCONS_00010730	-0.169955	0.684006	1.276	BGIOSGA013353	XP_003559565.1protein FLOURY ENDOSPERM 6, chloroplastic
TCONS_00009665	-0.169907	-0.792332	-0.28912	BGIOSGA032465	XP_006649714.1PREDICTED: uncharacterized protein LOC102722938 isoform X1
TCONS_00037127	-0.169862	0.2474	0.57156	BGIOSGA036245	XP_006663982.1 proline--tRNA ligase, cytoplasmic
TCONS_00023434	-0.169779	-0.211893	-0.0986998	BGIOSGA008199	XP_006658445.1 protein CIA1-like
TCONS_00025726	-0.169729	0.398212	0.122937	BGIOSGA023695	XP_015695181.1 nuclear pore complex protein NUP214
TCONS_00019113	-0.169581	-2.70541	-1.58365	BGIOSGA018251	XP_015692556.1 cytosolic endo-beta-N-acetylglucosaminidase 1-like
TCONS_00033791	-0.169561	1.35728	0.427032	BGIOSGA018806	XP_003577389.1DEAD-box ATP-dependent RNA helicase 52C
TCONS_00015160	-0.169443	0.0842735	0.638581	BGIOSGA021369	XP_006653019.2 protein S-acyltransferase 11

TCONS_00000064	-0.169366	-0.619774	-0.0917683	BGIOSGA007547	XP_023157643.1uncharacterized LOC100279343 isoform X4
TCONS_00008093	-0.169332	0.70392	0.740599	BGIOSGA015162	XP_015689224.1 staphylococcal nuclease domain-containing protein 1-like
TCONS_00033087	-0.169253	0.474824	-1.30531	#N/A	#N/A
TCONS_00035668	-0.169168	-0.756226	0.485973	BGIOSGA012507	XP_004977226.1aspartic proteinase Asp1
TCONS_00012120	-0.168816	0.0651971	-1.23859	BGIOSGA006341	XP_015689868.1 probable glycosyltransferase 4
TCONS_00000930	-0.168788	-2.76773	0.155623	BGIOSGA025034	XP_006658218.1 flavin-containing monooxygenase FMO GS-OX-like 9
TCONS_00018224	-0.168703	0.249178	0.088229	BGIOSGA020282	XP_006654721.2 SWR1-complex protein 4
TCONS_00028847	-0.168685	0.0334102	-1.17081	BGIOSGA007797	XP_015696320.1 aminopeptidase M1-C
TCONS_00020895	-0.168284	0.0634496	0.216818	BGIOSGA006855	XP_004966062.1heterogeneous nuclear ribonucleoprotein A3 homolog 2
TCONS_00012871	-0.168062	-0.173872	0.168754	BGIOSGA013258	XP_015691677.1 probable periplasmic serine endoprotease DegP-like isoform X4
TCONS_00036385	-0.167751	0.337755	0.853763	BGIOSGA032771	XP_015698359.1 methylcrotonoyl-CoA carboxylase subunit alpha, mitochondrial
TCONS_00002998	-0.16765	-1.73044	-0.396163	BGIOSGA034037	XP_015688178.1 protein ATAF2 isoform X2
TCONS_00000502	-0.167341	-0.26699	0.603773	BGIOSGA003075	XP_006645681.1 zinc phosphodiesterase ELAC protein 2 isoform X1
TCONS_00013055	-0.167298	0.955396	0.352606	BGIOSGA037819	XP_006650489.2 probable serine/threonine protein kinase IREH1
TCONS_00021219	-0.167263	0.122303	-0.156222	BGIOSGA023441	XP_006656363.1 molybdenum cofactor sulfurase
TCONS_00004470	-0.167043	-0.261566	0.172045	BGIOSGA005632	XP_006646359.1 probable xyloglucan glycosyltransferase 1
TCONS_00037596	-0.166808	-0.640544	-0.617206	BGIOSGA013510	XP_006664240.1 BEL1-like homeodomain protein 7
TCONS_00034797	-0.166787	-3.38361	-0.746223	BGIOSGA031094	XP_021302775.1DIMBOA UDP-glucosyltransferase BX9-like
TCONS_00000844	-0.166654	0.304405	-0.550798	BGIOSGA033678	XP_012702006.1GTP-binding protein SAR1A
TCONS_00016235	-0.166615	0.482624	0.29189	BGIOSGA017179	XP_015691318.1 DEAD-box ATP-dependent RNA helicase 13
TCONS_00026870	-0.166571	-2.24127	0.235543	BGIOSGA030981	XP_006659539.1 phosphatidylinositol/phosphatidylcholine transfer protein SFH6
TCONS_00017498	-0.166464	0.45286	0.392494	BGIOSGA019571	XP_006654235.1 dolichyl-diphosphooligosaccharide--protein glycosyltransferase subunit 1A
TCONS_00019297	-0.1663	0.471111	0.0954723	BGIOSGA032316	XP_006654406.1 putative disease resistance protein RGA3
TCONS_00009180	-0.16619	0.00522513	0.446393	BGIOSGA014189	XP_015690279.1 molybdate-anion transporter-like
TCONS_00014268	-0.165985	-0.592151	0.868358	BGIOSGA031245	XP_006652292.1 histone-lysine N-methyltransferase ASHH1
TCONS_00007293	-0.165955	-0.139831	0.0366416	BGIOSGA007189	XP_006646834.1 anthranilate phosphoribosyltransferase
TCONS_00008709	-0.165809	0.231709	-0.156281	BGIOSGA028930	XP_006649001.1 chloride channel protein CLC-f-like
TCONS_00027230	-0.165778	0.223904	-0.487316	BGIOSGA013505	XP_006659748.1PREDICTED: uncharacterized protein LOC102714034
TCONS_00032904	-0.165767	-1.84698	-1.48172	BGIOSGA010845	XP_015698010.1 alpha-L-arabinofuranosidase 1-like isoform X1
TCONS_00023185	-0.165505	-0.0243587	-0.272474	BGIOSGA018634	XP_004955583.1casein kinase 1-like protein HD16 isoform X2
TCONS_00023747	-0.165397	-1.17563	-0.475292	BGIOSGA013147	XP_006658603.1 rab3 GTPase-activating protein catalytic subunit
TCONS_00032795	-0.165147	0.379466	-0.205866	BGIOSGA022057	XP_006662116.1PREDICTED: uncharacterized protein LOC102703901
TCONS_00018999	-0.165087	0.228922	-0.345256	BGIOSGA032771	XP_006654231.1 acetyl-CoA carboxylase 2
TCONS_00000967	-0.164838	-1.80936	-0.0807543	BGIOSGA015881	NP_001151260.1stress regulated protein
TCONS_00002623	-0.164733	-0.716405	-1.07886	BGIOSGA005262	XP_020405928.1uncharacterized protein LOC100303930
TCONS_00031566	-0.164731	-0.1753	0.276081	BGIOSGA033380	XP_006662560.1 probable protein phosphatase 2C 47
TCONS_00015630	-0.164503	-1.54189	-1.14255	BGIOSGA001516	XP_006644236.1 putative disease resistance protein RGA3 isoform X2
TCONS_00023676	-0.16444	0.888814	0.331122	BGIOSGA025701	XP_015694963.1 predicted GPI-anchored protein 58
TCONS_00009316	-0.16442	-0.210038	-0.2759	BGIOSGA011859	XP_015691254.1 pheophorbide a oxygenase, chloroplastic
TCONS_00019365	-0.164299	-0.758713	0.207724	BGIOSGA004873	XP_006654459.1 polypyrimidine tract-binding protein homolog 3-like isoform X1
TCONS_00022431	-0.16406	1.4498	0.549074	BGIOSGA001561	XP_006656169.1 T-complex protein 1 subunit epsilon-like
TCONS_00008477	-0.164026	-0.65843	0.3595	BGIOSGA003766	XP_003569976.1vacuolar-processing enzyme beta-isozyme 1
TCONS_00000077	-0.16375	0.350255	-0.144402	BGIOSGA024205	XP_006643673.1 G-type lectin S-receptor-like serine/threonine-protein kinase SD2-5
TCONS_00033405	-0.163603	-1.71656	0.239102	BGIOSGA015855	XP_006662873.1PREDICTED: uncharacterized protein LOC102718029
TCONS_00028296	-0.163421	-0.374439	-0.0964598	BGIOSGA026683	XP_002444756.1transcription termination factor MTERF6, chloroplastic/mitochondrial
TCONS_00019030	-0.163388	-0.985606	0.215572	BGIOSGA034637	XP_006664872.1 pentatricopeptide repeat-containing protein At2g38420, mitochondrial-like
TCONS_00029125	-0.162893	0.373333	0.324434	BGIOSGA030875	XP_024310200.1uncharacterized protein LOC100838037 isoform X1
TCONS_00023147	-0.162747	-0.269004	-0.353863	BGIOSGA013694	XP_006657458.1 putative phosphatidylglycerol/phosphatidylinositol transfer protein DDB_G0282179
TCONS_00028266	-0.16269	-0.891155	-0.808979	BGIOSGA019255	XP_006660260.1 DNA-damage-repair/toleration protein DRT100-like
TCONS_00011319	-0.162316	1.03809	1.04598	BGIOSGA002795	XP_006652015.1 ALBINO3-like protein 1, chloroplastic
TCONS_00031449	-0.162203	0.458242	0.145192	BGIOSGA003758	XP_006662506.2 zinc finger CCCH domain-containing protein 19-like

TCONS_00020578	-0.162155	1.21251	0.277152	BGIOSGA005214	XP_015696844.1 uncharacterized CRM domain-containing protein At3g25440, chloroplastic isoform X2
TCONS_00022920	-0.162108	-0.200173	-0.261047	BGIOSGA033393	XP_006656500.1 probable protein phosphatase 2C 60
TCONS_00022086	-0.162066	1.55742	-0.406918	BGIOSGA017709	XP_006656893.1 leucine-rich repeat receptor-like serine/threonine/tyrosine-protein kinase SOBIR1
TCONS_00011806	-0.162009	-0.0802381	-0.302645	BGIOSGA011200	XP_015690965.1PREDICTED: uncharacterized protein LOC102706926
TCONS_00012276	-0.161972	-0.167948	-0.890692	BGIOSGA010728	XP_006650083.1 E3 ubiquitin-protein ligase RING1-like
TCONS_00021332	-0.161942	-0.592148	0.838405	BGIOSGA023560	XP_002437547.1protein APEM9
TCONS_00032819	-0.161937	-1.98116	-2.66468	BGIOSGA034655	XP_020403558.1uncharacterized LOC100501783 isoform X1
TCONS_00002140	-0.161693	1.23091	0.992937	BGIOSGA017914	XP_006644980.1 50S ribosomal protein L17-like
TCONS_00014216	-0.161601	0.108091	-2.26087	BGIOSGA016371	XP_015691866.1 glycine-rich RNA-binding protein 4, mitochondrial isoform X1
TCONS_00005475	-0.161551	-0.410956	0.0681594	BGIOSGA022638	XP_015688777.1 TATA-binding protein-associated factor BTAF1 isoform X1
TCONS_00024068	-0.161336	-0.309584	0.139587	BGIOSGA026102	XP_006657942.1PREDICTED: uncharacterized protein LOC102707787
TCONS_00019342	-0.161292	-0.315487	-0.305675	BGIOSGA030346	XP_010910914.1 serine hydroxymethyltransferase 7-like
TCONS_00024025	-0.161075	0.470094	-0.262172	#N/A	#N/A
TCONS_00024793	-0.161067	0.476555	0.115666	BGIOSGA025155	XP_015694782.1 40S ribosomal protein S12-like
TCONS_00004676	-0.16102	0.385199	-0.154636	BGIOSGA009558	XP_006654522.1 40S ribosomal protein S23
TCONS_00016073	-0.160964	-0.0950168	-0.235905	BGIOSGA014907	XP_015692053.1PREDICTED: uncharacterized protein LOC102718921
TCONS_00034721	-0.160904	0.00603335	-0.00403165	BGIOSGA034107	XP_024314657.1uncharacterized protein LOC100822022
TCONS_00036485	-0.160615	1.13123	2.67558	BGIOSGA019389	XP_015698376.1 glycine-rich RNA-binding protein GRP1A-like
TCONS_00025361	-0.16056	-0.074608	0.0814676	BGIOSGA025656	XP_006658707.1PREDICTED: uncharacterized protein At4g19900
TCONS_00009893	-0.160378	0.71347	0.801576	BGIOSGA012445	XP_024311582.1peroxisomal membrane protein 11-3
TCONS_00005066	-0.160223	-0.820488	0.36846	BGIOSGA018172	XP_004971209.1tyrosine-protein phosphatase non-receptor type 23
TCONS_00030575	-0.16019	1.07678	-0.0178456	BGIOSGA026471	XP_015696838.1 ABC transporter F family member 1-like
TCONS_00029527	-0.16015	-0.40893	-0.645364	BGIOSGA017586	XP_010910663.1 arogenate dehydratase/prephenate dehydratase 6, chloroplastic-like
TCONS_00025602	-0.160007	-1.65907	-1.03645	#N/A	XP_015699208.1 replication protein A 70 kDa DNA-binding subunit D-like isoform X2
TCONS_00036393	-0.159938	0.122514	-0.473161	BGIOSGA036536	XP_006664178.1 eukaryotic translation initiation factor 2 subunit gamma
TCONS_00006937	-0.159927	0.607863	-0.23088	BGIOSGA009963	XP_015689484.1 mitogen-activated protein kinase kinase kinase 1-like
TCONS_00005827	-0.159917	-0.252899	-0.864184	BGIOSGA020042	XP_015689545.1 TOM1-like protein 2
TCONS_00014683	-0.159903	-0.561016	-2.23928	BGIOSGA016856	XP_002446872.1uncharacterized protein LOC8083337
TCONS_00024847	-0.159651	1.48353	0.353399	BGIOSGA003314	XP_002459628.1IAA-amino acid hydrolase ILR1-like 9
TCONS_00004264	-0.159459	0.537734	-1.23204	BGIOSGA034410	XP_006646253.1 IAA-amino acid hydrolase ILR1-like 2
TCONS_00021522	-0.159394	-0.94621	-0.849278	BGIOSGA028899	XP_015694186.1 oligopeptide transporter 7-like
TCONS_00023739	-0.159374	-0.32002	0.947966	BGIOSGA025772	XP_006657743.1 transcription factor GTE9-like
TCONS_00036699	-0.159324	0.640657	-1.00998	BGIOSGA036611	XP_024317377.1 pentatricopeptide repeat-containing protein At1g09820-like
TCONS_00011248	-0.159179	0.129474	0.600648	BGIOSGA013847	XP_015690709.1 RNA-binding protein 28 isoform X1
TCONS_00027891	-0.159095	0.367351	-0.0105455	BGIOSGA004248	NP_001336948.1uncharacterized LOC100382599
TCONS_00004849	-0.159018	-0.503714	-0.281353	BGIOSGA000370	XP_015691366.1PREDICTED: uncharacterized protein LOC102700194
TCONS_00020182	-0.15901	0.974127	0.68982	BGIOSGA022373	XP_010228003.1subtilisin-like protease SBT6.1
TCONS_00019346	-0.158864	-1.54355	-0.272224	BGIOSGA027817	XP_015692608.1 carboxymethylenebutenolidase homolog
TCONS_00023071	-0.158839	-0.361943	-0.970937	BGIOSGA024973	XP_006657430.1PREDICTED: uncharacterized protein LOC102702861 isoform X1
TCONS_00030968	-0.15836	0.200546	0.480597	BGIOSGA032777	XP_006661718.1PREDICTED: uncharacterized protein LOC102699989
TCONS_00024761	-0.158129	0.0706819	-0.390298	BGIOSGA019815	XP_014753334.1nitrate regulatory gene2 protein
TCONS_00015133	-0.158043	-0.352945	0.370147	BGIOSGA020883	XP_015692062.1 zinc finger CCCH domain-containing protein 31
TCONS_00021815	-0.158023	0.425396	0.362818	BGIOSGA025296	XP_006655898.2 putative transcription elongation factor SPT5 homolog 1
TCONS_00004877	-0.157987	-2.00578	-1.83717	BGIOSGA019042	XP_015690627.1 pectin acetylesterase 5-like
TCONS_00001288	-0.157972	0.235648	-0.101755	BGIOSGA006852	XP_015697908.1 serine/threonine protein phosphatase 2A 55 kDa regulatory subunit B beta isoform-like isoform X1
TCONS_00003357	-0.157726	0.0848283	0.265137	BGIOSGA036803	XP_004967622.1aspartic proteinase
TCONS_00035655	-0.157716	-0.641548	0.418161	BGIOSGA037079	XP_006664381.2PREDICTED: uncharacterized protein LOC102715439
TCONS_00015019	-0.157569	-0.158136	-0.238483	BGIOSGA000693	XP_006652876.1 diacylglycerol kinase 5-like
TCONS_00028092	-0.157483	-0.270224	-0.422431	BGIOSGA017255	XP_002445584.1D-3-phosphoglycerate dehydrogenase 2, chloroplastic
TCONS_00004678	-0.157423	-1.02706	-0.520853	BGIOSGA012365	XP_006644969.1 TBC1 domain family member 8B
TCONS_00020890	-0.157386	-0.122521	-0.00573539	BGIOSGA003919	XP_010227595.1serine/threonine protein phosphatase 2A 55 kDa regulatory subunit B alpha isoform isoform X2

TCONS_00009041	-0.157305	-1.23271	-1.75688	BGIOSGA005362	XP_008677475.1ABC(yeast) homolog 1 isoform X1
TCONS_00009321	-0.157223	0.689494	0.0369021	BGIOSGA030304	XP_006649404.1 calmodulin-binding receptor-like cytoplasmic kinase 2
TCONS_00023583	-0.157153	-2.84154	-1.28664	BGIOSGA035928	XP_002461109.2disease resistance RPP8-like protein 3
TCONS_00033676	-0.15711	-1.01257	0.00952138	BGIOSGA018472	XP_021314164.1achilleol B synthase-like isoform X3
TCONS_00016284	-0.157081	-0.0510912	0.0667797	BGIOSGA014686	XP_003580169.2large ribosomal RNA subunit accumulation protein YCED homolog 2, chloroplastic
TCONS_00012044	-0.157004	0.506615	-0.416971	BGIOSGA010963	XP_006649886.1 50S ribosomal protein L10, chloroplastic-like
TCONS_00014122	-0.156918	0.103129	0.0494435	BGIOSGA026527	XP_003574549.1transcription factor MYB30
TCONS_00014916	-0.156825	-1.85481	-1.54665	BGIOSGA034013	XP_006652803.2 ent-kaur-16-ene synthase, chloroplastic isoform X1
TCONS_00024222	-0.156817	-0.239077	0.0450336	BGIOSGA026263	XP_003562559.1BSD domain-containing protein 1
TCONS_00001385	-0.156546	-0.52714	0.301704	BGIOSGA023898	XP_006644383.1 calcium-dependent protein kinase 3-like
TCONS_00024739	-0.156538	0.345036	-0.264772	BGIOSGA024637	XP_006658384.1 DNA damage-binding protein 1
TCONS_00034088	-0.156499	-0.662841	-0.874364	BGIOSGA037624	XP_002464589.1osmotin-like protein
TCONS_00022627	-0.156349	-0.405038	0.336772	BGIOSGA007697	NP_001150336.1cyclase precursor
TCONS_00021130	-0.156236	0.767563	-0.247541	BGIOSGA023352	XP_006656286.1 ESCRT-related protein CHMP1
TCONS_00001538	-0.156194	-0.30282	-0.167874	BGIOSGA004169	XP_006646183.1 protein VACUOLELESS1
TCONS_00000086	-0.156183	0.476996	-0.308057	BGIOSGA015550	XP_015698231.1 DExH-box ATP-dependent RNA helicase DExH3-like
TCONS_00009943	-0.156057	0.301819	0.267886	BGIOSGA033915	XP_006649980.1 probable pre-mRNA-splicing factor ATP-dependent RNA helicase DEAH2
TCONS_00019902	-0.155902	-0.644618	-1.12282	BGIOSGA010354	XP_006654863.1 AAA-ATPase At3g50940-like
TCONS_00030347	-0.155861	-0.330735	-1.08053	BGIOSGA015096	XP_006661423.1 beta-glucosidase 31
TCONS_00017106	-0.155638	0.39053	-1.19107	BGIOSGA000169	XP_004960436.1uncharacterized protein LOC101774563
TCONS_00034329	-0.155569	-0.0176673	-1.28775	BGIOSGA037596	XP_015698016.1 ABC transporter C family member 10-like
TCONS_00013492	-0.155504	-0.329342	-0.815558	BGIOSGA037529	XP_006650861.1 acyl-CoA-binding domain-containing protein 4-like
TCONS_00004610	-0.155501	-1.86922	-0.0540125	BGIOSGA005107	XP_006644890.2 beta-glucosidase 2-like
TCONS_00016914	-0.155355	-0.301725	0.843186	BGIOSGA021607	XP_006653061.1 putative pentatricopeptide repeat-containing protein At3g15130
TCONS_00015997	-0.15524	-3.65437	-2.25096	BGIOSGA005593	XP_006652354.2PREDICTED: uncharacterized protein LOC102706094
TCONS_00004967	-0.155098	0.693053	0.24129	BGIOSGA005200	XP_015699140.1 THO complex subunit 2 isoform X1
TCONS_00038356	-0.154993	-1.20366	-0.858975	BGIOSGA024171	XP_002453153.2probable nucleolar protein 5-2
TCONS_00008403	-0.154961	-0.490601	0.455966	BGIOSGA006019	XP_006647532.1 E3 ubiquitin-protein ligase PRT1-like
TCONS_00002317	-0.154806	-2.65491	-0.608178	BGIOSGA016395	XP_010239693.1G-type lectin S-receptor-like serine/threonine-protein kinase At2g19130
TCONS_00010075	-0.154783	0.343043	0.983914	BGIOSGA012620	XP_003557836.1nuclear/nucleolar GTPase 2
TCONS_00029957	-0.154781	1.08652	0.140221	BGIOSGA016852	XP_006661183.1 homeobox-leucine zipper protein HOX25-like
TCONS_00017515	-0.154749	-3.30247	-0.539355	BGIOSGA034485	XP_006655183.2 hippocampus abundant transcript 1 protein-like
TCONS_00018565	-0.154548	-1.10937	-0.297053	BGIOSGA001018	XP_015693140.1 long chain acyl-CoA synthetase 1
TCONS_00006298	-0.154537	0.829547	1.47391	BGIOSGA017178	XP_015689340.1 polyadenylate-binding protein RBP47-like isoform X2
TCONS_00018526	-0.154358	0.431983	-0.00522119	BGIOSGA012376	XP_004960337.1probable sugar phosphate/phosphate translocator At3g11320
TCONS_00004867	-0.154358	1.01128	0.876134	BGIOSGA036382	XP_006645148.2 rhodanese-like domain-containing protein 4A, chloroplastic
TCONS_00034385	-0.154312	-0.30673	-1.09577	BGIOSGA019844	XP_006662769.1 fructose-bisphosphate aldolase, chloroplastic
TCONS_00030601	-0.154272	-1.84192	-1.41726	BGIOSGA011659	XP_006662140.2 protein NRT1/ PTR FAMILY 8.3-like
TCONS_00004688	-0.154262	-0.340638	-0.51916	BGIOSGA002461	XP_006646465.1 metal tolerance protein 5
TCONS_00018095	-0.154012	1.56241	-0.487584	BGIOSGA025454	XP_006654617.1 calreticulin-3-like
TCONS_00005538	-0.153849	-1.56711	-1.39754	BGIOSGA014385	XP_006646953.1 probable cation transporter HKT6
TCONS_00002459	-0.153654	0.669258	-0.879904	BGIOSGA033885	XP_006646641.1PREDICTED: uncharacterized protein LOC102718238
TCONS_00024652	-0.153621	-1.70774	0.0411536	BGIOSGA020215	XP_006663021.1 non-specific phospholipase C4-like
TCONS_00032906	-0.153477	-1.0644	1.36726	BGIOSGA021840	XP_015698694.1 putative receptor protein kinase ZmPK1 isoform X1
TCONS_00016523	-0.15281	0.37012	0.649153	BGIOSGA014445	XP_015691559.1 transducin beta-like protein 3
TCONS_00004672	-0.152598	-0.022076	0.804027	BGIOSGA017401	XP_006644963.1 coatomer subunit delta-3
TCONS_00035364	-0.152573	0.014699	-1.67261	BGIOSGA024778	XP_022679613.1cysteine-rich receptor-like protein kinase 10
TCONS_00009046	-0.15256	0.224285	0.154611	BGIOSGA033076	XP_006649171.1 3-ketoacyl-CoA thiolase 2, peroxisomal
TCONS_00033869	-0.152228	-0.0954736	0.208963	BGIOSGA010054	XP_006663061.2 vacuolar protein sorting-associated protein 26A-like
TCONS_00020004	-0.151986	0.699837	0.48725	BGIOSGA013577	XP_004964371.1multiple organellar RNA editing factor 2, chloroplastic
TCONS_00005673	-0.151276	0.699886	1.05915	BGIOSGA028621	XP_006648455.1 aminopeptidase M1-A
TCONS_00016322	-0.151019	0.649504	0.0592803	BGIOSGA005947	XP_015691528.1 autophagy-related protein 13a-like
TCONS_00014752	-0.150938	0.487648	-1.05716	BGIOSGA016039	XP_006652647.1 cis-zeatin O-glucosyltransferase 1-like
TCONS_00014839	-0.150827	0.427545	0.782512	BGIOSGA037690	XP_015698346.1 protein misato homolog 1

TCONS_00008578	-0.150815	0.014355	0.572318	BGIOSGA007844	XP_015688924.1PREDICTED: LOW QUALITY PROTEIN: uncharacterized protein LOC102711226
TCONS_00008612	-0.150269	0.80874	-0.0859384	BGIOSGA031377	XP_003570129.1serine/arginine repetitive matrix protein 1
TCONS_00001754	-0.1502	0.0323188	0.913618	BGIOSGA021890	XP_003569726.1uncharacterized protein LOC100836957
TCONS_00001136	-0.150083	0.0430531	0.862992	BGIOSGA003761	XP_006646001.2 probable zinc protease PqqL
TCONS_00016656	-0.150083	-0.973	0.156029	BGIOSGA029488	XP_004976560.1putative FBD-associated F-box protein At1g61330
TCONS_00008113	-0.150054	-2.91474	-2.15763	BGIOSGA006337	XP_006647337.1 copper transport protein ATX1
TCONS_00036429	-0.149745	1.82689	1.93186	BGIOSGA037798	XP_003577345.1p21-activated protein kinase-interacting protein 1-like
TCONS_00012864	-0.149706	0.785405	0.869513	BGIOSGA010112	XP_006650375.1PREDICTED: uncharacterized protein LOC102702737 isoform X1
TCONS_00001187	-0.149687	0.313827	0.353832	BGIOSGA003817	XP_006644304.1 protein ROOT HAIR DEFECTIVE 3
TCONS_00016880	-0.149509	-0.437246	1.92598	BGIOSGA009831	XP_006653034.1 stem-specific protein TSJT1
TCONS_00023742	-0.149454	0.35265	-0.350632	BGIOSGA034240	XP_006657746.1 mitotic checkpoint serine/threonine-protein kinase BUB1
TCONS_00031290	-0.149213	-7.30782	-1.68193	BGIOSGA025466	XP_015697370.1 cellulose synthase A catalytic subunit 7 [UDP-forming]
TCONS_00036813	-0.148926	0.731239	-2.07331	BGIOSGA036501	XP_006663878.1 GDP-L-galactose phosphorylase 2-like
TCONS_00007081	-0.148729	1.1811	1.33971	BGIOSGA009264	XP_006648090.2 translation initiation factor eIF-2B subunit epsilon
TCONS_00006629	-0.148683	0.568136	-1.0946	BGIOSGA034846	XP_006647675.1 transcription factor bHLH157-like
TCONS_00025002	-0.148641	-0.300894	-0.840431	BGIOSGA027385	XP_015696028.1 protein CHROMATIN REMODELING 35-like
TCONS_00034487	-0.148614	-0.824782	0.653428	BGIOSGA034330	XP_006663283.1 F-box/LRR-repeat protein At3g59190-like
TCONS_00021724	-0.148415	0.75993	0.726935	BGIOSGA008730	XP_015693921.1 putative glycerol-3-phosphate transporter 5
TCONS_00026733	-0.148402	-0.295738	-0.280953	BGIOSGA028796	XP_006659473.1PREDICTED: uncharacterized protein LOC102704077
TCONS_00014674	-0.14828	-0.0269127	-1.60856	BGIOSGA016844	XP_006652588.1 NAD(P)H-quinone oxidoreductase subunit M, chloroplastic
TCONS_00005307	-0.148113	-0.0171348	-0.277312	BGIOSGA006838	XP_015689262.1 cyclin-dependent kinase A-2
TCONS_00014117	-0.148113	-0.899032	0.172885	BGIOSGA025856	XP_008807445.1PREDICTED: uncharacterized protein LOC103719813 isoform X1
TCONS_00014550	-0.148074	-0.230008	0.0901647	BGIOSGA016713	XP_015692184.1 gamma-tubulin complex component 2 isoform X2
TCONS_00022096	-0.147994	0.648371	1.01868	BGIOSGA035933	XP_006656799.1 cytochrome P450 76C2-like
TCONS_00013023	-0.147634	-1.53651	0.53236	BGIOSGA020209	XP_006650460.1 beta-glucosidase 8 isoform X2
TCONS_00032099	-0.147519	0.683335	0.17191	BGIOSGA031125	XP_006662306.1 ras-related protein Rab11D-like
TCONS_00017748	-0.14745	-0.0847983	0.665497	BGIOSGA011506	XP_015693312.1 SNF2 domain-containing protein CLASSY 3-like
TCONS_00002519	-0.147408	-0.458596	-0.426621	BGIOSGA029476	XP_002456925.1scarecrow-like protein 3
TCONS_00034236	-0.147053	1.5883	0.861077	BGIOSGA036685	XP_006662707.1 glutamine--fructose-6-phosphate aminotransferase [isomerizing] 2-like
TCONS_00018953	-0.147025	-2.09651	-1.05762	BGIOSGA018410	XP_006654182.1 random slug protein 5-like
TCONS_00005556	-0.146891	0.196179	0.127554	BGIOSGA005227	XP_015689393.1 U2 snRNP-associated SURP motif-containing protein-like isoform X1
TCONS_00014489	-0.146609	1.47167	0.791731	BGIOSGA023462	XP_006653553.1 transcription factor bHLH128-like
TCONS_00022953	-0.146548	-0.031386	0.226343	BGIOSGA020494	XP_006656531.2 lysine-specific demethylase 5B-B
TCONS_00035011	-0.14637	0.433182	0.777257	BGIOSGA011984	XP_006649456.1 protein ROOT PRIMORDIUM DEFECTIVE 1-like
TCONS_00011975	-0.146269	-0.575086	0.344293	BGIOSGA011217	XP_006649822.1 digalactosyldiacylglycerol synthase 2, chloroplastic-like
TCONS_00006245	-0.146255	1.67511	0.342571	BGIOSGA003439	XP_006648723.2 protein disulfide isomerase-like 5-3
TCONS_00021441	-0.146157	-0.206588	-0.161258	BGIOSGA033474	XP_006655678.1 probable methyltransferase PMT17 isoform X1
TCONS_00007722	-0.146041	-0.331539	-0.383046	BGIOSGA017229	XP_006647126.1 cyclic nucleotide-gated ion channel 1
TCONS_00019215	-0.14594	0.453149	-0.288211	BGIOSGA000098	XP_003568548.1PH, RCC1 and FYVE domains-containing protein 1
TCONS_00008874	-0.145903	0.60563	0.0215748	BGIOSGA022526	XP_010236068.1STOREKEEPER protein
TCONS_00013500	-0.145881	-0.713495	0.319136	BGIOSGA009484	XP_015690203.1 ubiquinone biosynthesis monooxygenase COQ6, mitochondrial isoform X2
TCONS_00023818	-0.145757	0.309124	0.799723	BGIOSGA037056	XP_006657781.1 thiamine thiazole synthase 2, chloroplastic
TCONS_00017345	-0.145664	-0.260091	0.366171	BGIOSGA006958	XP_010231335.1neurofilament medium polypeptide
TCONS_00006718	-0.145599	-0.540634	0.262014	BGIOSGA025336	XP_004953612.1tubby-like F-box protein 5
TCONS_00017524	-0.14547	-0.474078	-0.110582	BGIOSGA000544	XP_015689608.1 coatomer subunit delta-1 isoform X1
TCONS_00010633	-0.145298	0.394706	-0.526657	BGIOSGA037843	XP_008644627.2 atherin
TCONS_00031203	-0.144796	0.239796	0.298956	BGIOSGA034370	XP_006661796.2 RNA-binding protein 39-like
TCONS_00026842	-0.144662	0.603271	0.0932537	BGIOSGA030966	XP_006659528.1 serine/threonine-protein kinase D6PK-like
TCONS_00009609	-0.144631	0.0517551	0.391622	BGIOSGA012154	XP_006651187.1 conserved oligomeric Golgi complex subunit 6
TCONS_00012081	-0.144484	-0.122501	0.0320834	BGIOSGA005710	XP_006654023.1 DNA-directed RNA polymerases I and III subunit rpac1
TCONS_00000207	-0.144461	2.10138	0.409655	BGIOSGA037366	XP_002462619.2uncharacterized protein LOC8058965
TCONS_00016132	-0.144398	0.0348038	-0.043016	BGIOSGA006108	XP_003575297.1cytosolic Fe-S cluster assembly factor NBP35

TCONS_00022375	-0.144319	-0.075052	0.30128	BGIOSGA024906	XP_006657033.1PREDICTED: uncharacterized protein LOC102705262
TCONS_00026156	-0.144263	0.603693	1.21904	BGIOSGA014392	XP_006659226.1PREDICTED: uncharacterized protein LOC102722514
TCONS_00012070	-0.144257	-0.0216637	-0.00448405	BGIOSGA010938	XP_004984863.1pyruvate decarboxylase 2
TCONS_00009272	-0.144102	0.310746	-0.442636	BGIOSGA011810	XP_004985783.1uncharacterized protein LOC101755008
TCONS_00002752	-0.144042	0.468157	-0.143798	BGIOSGA002466	XP_006643698.1 transmembrane protein 214-A-like
TCONS_00017884	-0.143741	-2.71886	-1.30584	#N/A	#N/A
TCONS_00011255	-0.143712	0.0541536	-0.116411	#N/A	XP_006650827.1 zinc finger CCCH domain-containing protein 25
TCONS_00016832	-0.143649	0.494037	1.25735	BGIOSGA014131	XP_003580782.1probable carboxylesterase Os04g0669500
TCONS_00025728	-0.143615	-0.719014	1.50119	BGIOSGA023693	XP_006658982.1 E3 ubiquitin-protein ligase RHA2A-like
TCONS_00026163	-0.143472	1.39675	0.0305949	BGIOSGA038424	XP_012700639.1exocyst complex component EXO70B1
TCONS_00019929	-0.143407	0.578689	0.585523	BGIOSGA006432	XP_006655666.1 structural maintenance of chromosomes protein 5
TCONS_00013087	-0.143316	-0.783787	-0.78459	BGIOSGA009927	XP_023157350.1F-box/FBD/LRR-repeat protein At1g13570-like isoform X1
TCONS_00015648	-0.143309	-1.22147	0.537657	BGIOSGA015337	XP_006653273.1 aspartic proteinase-like protein 2
TCONS_00025543	-0.142969	-0.728766	-1.99109	BGIOSGA031387	XP_015694732.1 phospho-2-dehydro-3-deoxyheptonate aldolase 2, chloroplastic
TCONS_00014160	-0.142958	-0.380647	0.516662	BGIOSGA029072	XP_006653348.1 glycine--tRNA ligase, mitochondrial 1-like
TCONS_00004984	-0.142895	0.0575303	-0.143213	BGIOSGA015212	XP_006645242.1 dynamin-related protein 3B-like isoform X1
TCONS_00000380	-0.142853	-0.109532	0.335326	BGIOSGA026350	XP_002457168.1probable serine/threonine-protein kinase At1g54610
TCONS_00037152	-0.142698	-0.317743	0.200005	BGIOSGA013281	XP_006664875.1 glioma tumor suppressor candidate region gene 1 protein
TCONS_00021742	-0.142512	-0.306999	0.29686	BGIOSGA018822	XP_015694099.1 CHD3-type chromatin-remodeling factor PICKLE-like
TCONS_00023252	-0.142502	-0.570978	-0.636853	BGIOSGA025294	XP_015695166.1 probable D-2-hydroxyglutarate dehydrogenase, mitochondrial
TCONS_00030151	-0.142496	0.506829	-0.943507	BGIOSGA006225	XP_006660681.2 beta-1,3-galactosyltransferase 7-like
TCONS_00000181	-0.14248	0.151093	0.761791	BGIOSGA004696	XP_006643736.2 mucin-5AC-like
TCONS_00023015	-0.142429	-1.82132	0.0937405	BGIOSGA011246	XP_015695059.1PREDICTED: uncharacterized protein LOC107304628
TCONS_00005211	-0.142351	0.0139658	0.449941	BGIOSGA007323	XP_003570759.1translation initiation factor IF3-1, mitochondrial
TCONS_00017788	-0.142242	-0.0015212	-0.45062	BGIOSGA019851	XP_006654386.1 autophagy-related protein 18h-like
TCONS_00010296	-0.142109	-0.469408	-0.897046	BGIOSGA020407	XP_002467695.1nuclear transcription factor Y subunit B-8
TCONS_00032785	-0.142009	-0.883292	-1.31929	BGIOSGA031322	XP_006662109.1 D-xylose-proton symporter-like 2
TCONS_00031447	-0.142001	-0.0902321	0.594959	BGIOSGA023970	XP_006661946.1 probable serine protease EDA2
TCONS_00019317	-0.141996	-0.350169	-0.125046	BGIOSGA018031	XP_006655338.1PREDICTED: uncharacterized protein LOC102722018
TCONS_00009547	-0.141966	-1.69842	-0.723006	BGIOSGA012092	XP_006649611.1PREDICTED: uncharacterized protein LOC102716853
TCONS_00002872	-0.141948	-0.425955	1.37078	BGIOSGA018751	XP_006645523.1 acyl-coenzyme A oxidase 4, peroxisomal-like
TCONS_00036359	-0.14168	-1.47822	0.809881	BGIOSGA037726	XP_006664164.1 phosphatidylinositol-glycan biosynthesis class X protein isoform X1
TCONS_00009640	-0.14165	0.729693	0.374682	BGIOSGA033422	XP_006649688.2 probable E3 ubiquitin-protein ligase ARI2
TCONS_00028996	-0.141392	0.978705	1.38867	BGIOSGA030754	XP_021319724.1uncharacterized protein LOC8074706 isoform X1
TCONS_00018637	-0.141342	0.646028	0.709231	BGIOSGA003530	XP_006654051.1 cullin-1-like
TCONS_00031675	-0.141339	0.100513	-0.630877	BGIOSGA033494	XP_015697092.1 probable plastid-lipid-associated protein 3, chloroplastic
TCONS_00009055	-0.141307	0.76537	-0.173602	BGIOSGA031832	XP_006648129.1 probable receptor-like protein kinase At5g47070
TCONS_00003521	-0.141285	0.44315	0.424753	BGIOSGA001682	XP_006645880.1 ubiquitin-like-specific protease ESD4
TCONS_00034873	-0.141244	-0.345358	0.365976	BGIOSGA028447	XP_006662940.1 probable bifunctional methylthioribulose-1-phosphate dehydratase/enolase-phosphatase E1
TCONS_00015168	-0.141195	-1.0341	0.192229	BGIOSGA017350	XP_006653878.1 probable protein arginine N-methyltransferase 6.1
TCONS_00017456	-0.141129	0.403468	-0.248303	BGIOSGA019531	XP_015693280.1PREDICTED: uncharacterized protein LOC102705435 isoform X2
TCONS_00012402	-0.141011	-1.21294	-0.569307	BGIOSGA007114	XP_006650171.1 protein STRUBBELIG-RECEPTOR FAMILY 7
TCONS_00024271	-0.140998	-0.793598	-0.121886	BGIOSGA011079	XP_015694758.1 rho GTPase-activating protein 7-like
TCONS_00007407	-0.140901	0.276375	0.30077	BGIOSGA007625	XP_006646926.1 cullin-associated NEDD8-dissociated protein 1
TCONS_00006863	-0.140895	-0.938533	-0.659651	BGIOSGA003209	XP_002454287.1geranylgeranyl diphosphate reductase, chloroplastic
TCONS_00004640	-0.140876	-0.885876	0.737556	BGIOSGA036953	XP_002458689.1RING-H2 finger protein ATL74
TCONS_00008173	-0.140811	0.670794	0.283994	BGIOSGA034095	XP_006647370.1 phosphoinositide phosphatase SAC7-like
TCONS_00023588	-0.140664	-4.82351	-2.04809	BGIOSGA024383	XP_003563185.1COP1-interacting protein 7
TCONS_00037865	-0.140174	-1.35448	-0.673663	BGIOSGA026308	XP_006658904.2PREDICTED: uncharacterized protein LOC102712743
TCONS_00030102	-0.140151	-0.220906	-0.272727	BGIOSGA028535	XP_006661267.1 AT-rich interactive domain-containing protein 4

TCONS_00011722	-0.139964	-0.430739	0.55253	BGIOSGA011288	XP_006649556.1 ribosome biogenesis protein TSR3 homolog isoform X1
TCONS_00006635	-0.139964	-1.13856	-0.730404	BGIOSGA016948	XP_006647680.1 RING-H2 finger protein ATL2-like
TCONS_00009106	-0.139849	1.28614	0.363985	BGIOSGA031140	XP_015689360.1 protein ELC-like
TCONS_00034353	-0.139837	-0.597956	-0.0506168	BGIOSGA033992	XP_006663448.1PREDICTED: uncharacterized protein LOC102714137
TCONS_00035540	-0.139662	-0.309474	-0.513653	BGIOSGA034759	XP_006664846.1 heterogeneous nuclear ribonucleoprotein R-like
TCONS_00007619	-0.139518	0.881768	0.73696	BGIOSGA014370	XP_006648463.2 serine/threonine-protein kinase EDR1
TCONS_00036220	-0.139496	-0.020725	-0.654193	BGIOSGA037577	XP_015698329.1 calmodulin-binding protein 60 C-like
TCONS_00034279	-0.139495	-0.608635	-0.20107	BGIOSGA007988	XP_006663193.1 DNA-directed RNA polymerase V subunit 5A
TCONS_00036801	-0.139395	0.277168	0.374015	BGIOSGA000394	XP_015698423.1 amino acid permease 3
TCONS_00032487	-0.139306	0.18254	-2.31702	BGIOSGA027785	XP_021307824.1protein Rf1, mitochondrial
TCONS_00010370	-0.139182	-0.177868	0.610941	BGIOSGA026734	XP_020400022.1probable WRKY transcription factor 4 isoform X2
TCONS_00032918	-0.138948	-5.04673	-4.63985	BGIOSGA034535	XP_004978619.1phosphoglycerate mutase-like protein 4
TCONS_00024446	-0.138834	1.51402	1.24538	BGIOSGA017281	XP_006657401.1 60S ribosomal protein L13a-2-like
TCONS_00013494	-0.138819	-0.100574	-0.26468	BGIOSGA009493	XP_006650865.1 glycine-rich RNA-binding protein RZ1A isoform X2
TCONS_00000006	-0.138813	-0.198139	0.521841	BGIOSGA018977	NP_001169346.1putative DUF1664 domain family protein
TCONS_00025364	-0.138806	-1.01094	-0.490835	BGIOSGA024066	XP_006657851.1 exocyst complex component EXO84C
TCONS_00015860	-0.138792	0.486084	-0.32499	BGIOSGA007344	XP_004952654.140S ribosomal protein S14
TCONS_00022097	-0.138736	-0.272266	-0.929114	BGIOSGA022740	XP_006656905.1PREDICTED: uncharacterized protein LOC102716700
TCONS_00006679	-0.138722	1.41094	1.5357	BGIOSGA008848	XP_006647718.1 ubiquitin carboxyl-terminal hydrolase 21
TCONS_00001138	-0.138395	0.217911	0.0571769	BGIOSGA003763	XP_006649567.1 serine--tRNA ligase
TCONS_00033411	-0.138213	-0.233009	0.320845	BGIOSGA012312	XP_015698145.1 RNA polymerase sigma factor sigB
TCONS_00031620	-0.138166	-0.601481	-0.303221	BGIOSGA005812	XP_006662057.1PREDICTED: uncharacterized protein LOC102709773
TCONS_00012078	-0.138068	-2.25893	-0.813858	BGIOSGA006959	XP_015689695.1 probable inactive receptor-like protein kinase At3g56050 isoform X2
TCONS_00013541	-0.138011	1.27401	0.738629	BGIOSGA009435	XP_003562405.1ankyrin repeat and SAM domain-containing protein 6
TCONS_00037179	-0.138001	-0.0309277	0.87261	BGIOSGA036195	XP_006664559.1 DEAD-box ATP-dependent RNA helicase 28
TCONS_00016606	-0.137831	-0.709581	-0.361321	BGIOSGA028185	XP_006652815.1 probable potassium transporter 11
TCONS_00001114	-0.137807	0.0113344	0.369412	BGIOSGA003735	XP_006644282.1 DEAD-box ATP-dependent RNA helicase 15
TCONS_00034490	-0.137646	0.103786	-0.19322	BGIOSGA022079	XP_006662810.1 extra-large guanine nucleotide-binding protein 3
TCONS_00020552	-0.137559	-3.80237	-1.19302	BGIOSGA026166	XP_006656895.1 UDP-glycosyltransferase 708A6-like
TCONS_00011279	-0.137533	-0.814355	-0.782312	BGIOSGA004006	XP_006650856.1 probable polygalacturonase isoform X3
TCONS_00017042	-0.13747	0.698768	0.551845	BGIOSGA013466	XP_006653995.1 E3 SUMO-protein ligase SIZ1 isoform X2
TCONS_00001209	-0.137439	-0.880494	-0.0351629	BGIOSGA003830	XP_006644312.2 lycopene epsilon cyclase, chloroplastic
TCONS_00032563	-0.137319	-1.1419	-1.73703	BGIOSGA038478	XP_006661957.1 protein CDI-like
TCONS_00024514	-0.137201	1.55839	0.765175	BGIOSGA024883	XP_021304390.1L-type lectin-domain containing receptor kinase IV.1
TCONS_00036998	-0.137163	-1.34855	0.128277	BGIOSGA003481	XP_019078959.1PREDICTED: uncharacterized protein LOC104881002
TCONS_00009183	-0.137152	-1.10344	-0.975299	BGIOSGA002764	XP_006649265.1 pyridoxine/pyridoxamine 5'-phosphate oxidase 2
TCONS_00008921	-0.137025	-1.49093	-0.593388	BGIOSGA005487	XP_015689310.1 ATP-dependent DNA helicase Q-like 3
TCONS_00010958	-0.136976	0.495092	0.0181862	BGIOSGA013559	XP_006651820.1 binding partner of ACD11 1
TCONS_00024968	-0.136652	-3.25751	-3.97093	BGIOSGA022241	XP_012474758.1 granule-bound starch synthase 1, chloroplastic/amyloplastic-like isoform X3
TCONS_00013026	-0.136488	1.17339	-0.212104	BGIOSGA009117	XP_004982050.1mitogen-activated protein kinase kinase kinase 1
TCONS_00020394	-0.136465	-0.920591	0.48323	BGIOSGA028034	XP_015694021.1 protein RKD2-like
TCONS_00022616	-0.136306	1.57621	0.0345179	BGIOSGA003899	XP_006656279.1PREDICTED: uncharacterized protein LOC102707685
TCONS_00003986	-0.136303	-0.315834	0.42447	BGIOSGA001204	XP_006644369.1 zinc finger CCCH domain-containing protein 8-like
TCONS_00001362	-0.136247	0.600824	0.657568	BGIOSGA003993	XP_003569359.1SCY1-like protein 2
TCONS_00026497	-0.135903	-3.462	-5.85854	BGIOSGA034394	NP_001159086.2dirigent-like protein pDIR3
TCONS_00001395	-0.135827	-2.14379	-3.32571	BGIOSGA010844	XP_006644390.1 heat stress transcription factor C-1a-like
TCONS_00028841	-0.135723	-3.72888	-1.74101	BGIOSGA001316	XP_021310531.1hypersensitive-induced response protein-like protein 2
TCONS_00011764	-0.135622	-1.29605	0.851696	BGIOSGA013616	XP_006649599.1 casein kinase II subunit alpha-like
TCONS_00004413	-0.135528	1.13963	0.143424	BGIOSGA000809	XP_006644724.1 basic salivary proline-rich protein 1 isoform X1
TCONS_00001396	-0.135503	0.467036	0.190164	BGIOSGA004026	XP_006644391.1 beta-adaptin-like protein A
TCONS_00000315	-0.135499	-0.327833	0.393966	BGIOSGA019275	XP_015695924.1 ABC transporter F family member 4
TCONS_00035889	-0.13533	0.17202	-0.99508	BGIOSGA037263	XP_006664509.1 chloroplast sensor kinase, chloroplastic
TCONS_00015227	-0.13532	0.376664	-0.865031	BGIOSGA001322	XP_015691630.1 protein QUIRKY-like

TCONS_00018454	-0.135271	1.85551	0.920833	BGIOSGA009622	XP_015692858.1 transcription factor bHLH77-like
TCONS_00014650	-0.135227	-1.28894	-1.81263	BGIOSGA005655	XP_002446838.1probable galacturonosyltransferase-like 3
TCONS_00037542	-0.135142	0.755818	0.184491	BGIOSGA010144	XP_006664752.2 serine/threonine-protein phosphatase BSL2 homolog
TCONS_00007381	-0.135099	-0.559744	-1.1576	BGIOSGA017252	XP_004951837.1protein IRREGULAR XYLEM 15
TCONS_00025236	-0.135098	-0.62505	-1.34115	BGIOSGA030042	XP_006657785.1PREDICTED: uncharacterized protein LOC102710713
TCONS_00027724	-0.135062	-0.386498	0.053884	BGIOSGA027281	XP_006659267.1 tobamovirus multiplication protein 2A
TCONS_00006896	-0.135058	0.206148	0.497542	BGIOSGA021646	XP_006647916.1 nuclear receptor coactivator 7-like
TCONS_00015225	-0.134998	0.62433	0.485208	BGIOSGA017411	XP_015691629.1 transportin-1
TCONS_00019350	-0.134937	0.518054	0.479431	BGIOSGA018827	XP_006654442.2 probable magnesium transporter NIPA6
TCONS_00008823	-0.134819	-0.391904	-0.616741	BGIOSGA011841	XP_006647917.1 DIS3-like exonuclease 2
TCONS_00016175	-0.134807	-0.802035	0.118748	BGIOSGA006077	XP_006652470.1 LETM1 and EF-hand domain-containing protein 1, mitochondrial-like
TCONS_00026117	-0.134802	1.645	0.671741	BGIOSGA028189	XP_006659933.1 GTP-binding protein ERG
TCONS_00000451	-0.13462	0.165892	-0.0229009	BGIOSGA003026	XP_006645646.1 GDT1-like protein 1, chloroplastic
TCONS_00030023	-0.13454	-0.58642	-0.798329	BGIOSGA029783	XP_006660613.1 haloacid dehalogenase-like hydrolase domain-containing protein Sgpp
TCONS_00007665	-0.134458	-0.717508	0.252801	BGIOSGA021624	XP_003571896.1probable LRR receptor-like serine/threonine-protein kinase At5g45780
TCONS_00021629	-0.134434	0.143962	-1.4547	BGIOSGA018154	XP_015693857.1PREDICTED: uncharacterized protein LOC102699695
TCONS_00025942	-0.134291	-0.631152	-0.67745	BGIOSGA021882	XP_006659134.1 mitogen-activated protein kinase 2
TCONS_00033810	-0.134231	-0.846554	-2.25145	BGIOSGA034056	XP_006663035.2 ABC transporter F family member 5-like
TCONS_00010335	-0.134005	0.763107	0.303384	BGIOSGA012907	XP_004983991.140S ribosomal protein S19
TCONS_00030060	-0.133995	-1.88844	-0.942145	BGIOSGA029749	XP_006661241.2 centromere-associated protein E
TCONS_00001665	-0.133989	-0.309767	0.366839	BGIOSGA035841	XP_006644588.2 single myb histone 3 isoform X2
TCONS_00034837	-0.133972	-0.316051	0.0847213	BGIOSGA035236	XP_003571252.1putative F-box protein At2g33200
TCONS_00001609	-0.13395	0.254774	0.942986	BGIOSGA004245	XP_015698119.1 DNA repair helicase XPB1-like
TCONS_00033158	-0.133905	-0.0531004	-0.128848	BGIOSGA003294	XP_004978786.1F-box/LRR-repeat protein At3g26922
TCONS_00031644	-0.133769	-0.610551	0.0868578	BGIOSGA033459	XP_006661941.2 E3 ubiquitin-protein ligase BRE1-like 2
TCONS_00002518	-0.133752	-0.56144	0.0383736	BGIOSGA005159	XP_015692173.1 crossover junction endonuclease MUS81
TCONS_00023673	-0.133703	1.66089	0.57591	BGIOSGA029238	XP_015695433.1PREDICTED: uncharacterized protein LOC102709009
TCONS_00038844	-0.133597	0.641393	-0.206915	BGIOSGA034010	XP_006663111.1 putative disease resistance RPP13-like protein 3 isoform X1
TCONS_00008736	-0.133552	-1.50922	-0.59439	BGIOSGA022688	XP_006647829.1 sodium-coupled neutral amino acid transporter 1 isoform X2
TCONS_00000416	-0.133379	0.295648	0.25318	BGIOSGA014321	XP_015688112.1 transcription factor GTE4 isoform X1
TCONS_00026185	-0.133361	0.424917	0.263433	BGIOSGA012647	XP_006659234.1 villin-4-like isoform X2
TCONS_00019392	-0.133298	-1.41001	-0.162058	BGIOSGA013217	XP_004961927.1cell division protein FtsZ homolog 2-2, chloroplastic isoform X2
TCONS_00001355	-0.133288	-0.329849	-0.817434	BGIOSGA019906	XP_006646096.1 protein trichome birefringence-like 3
TCONS_00008374	-0.133105	1.10891	1.48141	BGIOSGA032439	XP_006648834.1 cell surface glycoprotein 1-like
TCONS_00021673	-0.133047	-0.58386	0.599708	BGIOSGA021846	XP_006656677.1 proline iminopeptidase
TCONS_00001101	-0.132978	-2.3173	-0.917737	BGIOSGA003721	XP_015688958.1 putative pentatricopeptide repeat-containing protein At1g64310
TCONS_00019468	-0.132872	0.785377	0.988891	BGIOSGA026596	XP_006654531.1 sorting and assembly machinery component 50 homolog A-like
TCONS_00011575	-0.132817	1.11664	0.921439	BGIOSGA011186	XP_006649410.1 inorganic phosphate transporter 1-1
TCONS_00030218	-0.132727	2.00431	0.49668	BGIOSGA026755	XP_006661346.1 RNA-binding protein cabeza-like
TCONS_00021778	-0.132625	-4.38434	-1.37041	BGIOSGA021744	XP_006655873.1PREDICTED: uncharacterized protein LOC102704708 isoform X2
TCONS_00011399	-0.132573	-1.73285	-0.874343	BGIOSGA011625	XP_013448283.1chromodomain helicase-DNA-binding-like protein
TCONS_00017529	-0.132564	-0.183861	0.931723	BGIOSGA019599	XP_015693325.1 quinone oxidoreductase-like protein 2
TCONS_00025917	-0.132396	-0.826178	0.596526	BGIOSGA037240	NP_001145703.1putative RING zinc finger domain superfamily protein
TCONS_00012826	-0.132309	0.404326	0.38804	BGIOSGA018275	XP_015689993.1 GTPase LSG1-1-like
TCONS_00024447	-0.131991	-0.477237	-0.130096	BGIOSGA009784	XP_006657402.1 protein LEO1 homolog
TCONS_00022948	-0.131951	-0.00885334	-0.260191	BGIOSGA020498	XP_006657369.1PREDICTED: uncharacterized protein LOC102702575 isoform X1
TCONS_00029386	-0.131888	-0.11763	0.132674	BGIOSGA020542	XP_015696547.1PREDICTED: uncharacterized protein LOC102710051
TCONS_00029877	-0.131886	-0.926353	0.522513	BGIOSGA030562	XP_006660555.1 probable NADH kinase
TCONS_00027709	-0.13142	-0.5119	0.0326167	BGIOSGA030546	XP_004973145.1probable LRR receptor-like serine/threonine-protein kinase At2g24230
TCONS_00003031	-0.131249	-0.536568	-2.17657	BGIOSGA006838	XP_002454995.1homeobox-leucine zipper protein HOX29
TCONS_00034369	-0.131105	-1.13332	-0.78752	BGIOSGA026712	XP_006648618.1 probable mediator of RNA polymerase II transcription subunit 26c
TCONS_00011023	-0.130954	-1.42219	-1.17982	BGIOSGA007547	NP_001147411.1plastoquinol-plastocyanin reductase
TCONS_00036090	-0.130907	0.111226	0.786759	BGIOSGA025428	XP_006664051.1 eukaryotic translation initiation factor 5A-2

TCONS_00025574	-0.130646	-0.752101	-0.794752	BGIOSGA002240	XP_015695126.1 glucan endo-1,3-beta-glucosidase 4
TCONS_00017736	-0.130555	-0.884259	-0.670208	BGIOSGA019797	XP_006655278.2 probable transmembrane GTPase FZO-like, chloroplastic
TCONS_00035677	-0.13054	0.231616	-0.0577398	BGIOSGA037096	XP_022682920.1uncharacterized protein LOC111257425
TCONS_00011896	-0.130225	0.14463	-0.121852	BGIOSGA027117	XP_006649733.1 brefeldin A-inhibited guanine nucleotide-exchange protein 2
TCONS_00014443	-0.130092	-0.216465	-0.215774	BGIOSGA008529	XP_004975960.1plant intracellular Ras-group-related LRR protein 3
TCONS_00011497	-0.130083	0.102804	0.143809	BGIOSGA011530	XP_006651002.1 exosome complex exonuclease RRP44 homolog A
TCONS_00013156	-0.129913	0.00528871	-0.190571	BGIOSGA014577	XP_010230072.1protein SPIRRIG
TCONS_00036347	-0.129827	-0.563449	0.22905	BGIOSGA018027	XP_015698653.1PREDICTED: uncharacterized protein LOC102702624 isoform X2
TCONS_00011347	-0.129449	-0.367144	-1.76728	BGIOSGA029082	XP_006650906.1 ankyrin repeat domain-containing protein 2A-like isoform X2
TCONS_00014221	-0.129383	-0.707562	-0.479797	BGIOSGA037029	XP_006652271.1 putative HVA22-like protein g
TCONS_00005793	-0.129344	-0.893603	-0.435682	BGIOSGA007908	XP_003572147.1protein THYLAKOID RHODANESE-LIKE, chloroplastic
TCONS_00009561	-0.129337	-0.0746019	0.566007	BGIOSGA012109	XP_021313687.1recQ-mediated genome instability protein 1-like
TCONS_00006626	-0.128965	0.815435	0.638386	BGIOSGA031351	XP_014756870.1probable N6-adenosine-methyltransferase MT-A70-like
TCONS_00007607	-0.128913	0.383264	1.53088	BGIOSGA013766	XP_002453535.1serine/threonine-protein phosphatase PP2A-1 catalytic subunit
TCONS_00028923	-0.128836	0.901744	-0.197168	BGIOSGA030501	XP_006660592.1 nucleobase-ascorbate transporter 6-like
TCONS_00025793	-0.128795	-1.05125	-0.416931	BGIOSGA007133	XP_006659038.2 alcohol dehydrogenase
TCONS_00008162	-0.128645	-0.0176677	0.307794	BGIOSGA007432	XP_006647359.1 cytosolic invertase 1
TCONS_00001128	-0.128563	0.666155	1.0384	BGIOSGA003752	XP_015699136.1 chaperone protein dnaJ 49
TCONS_00011958	-0.128473	0.645736	0.741801	BGIOSGA011054	XP_006649797.1 BTB/POZ domain-containing protein At3g09030
TCONS_00032352	-0.128238	-2.771	0.806904	BGIOSGA033298	XP_006661827.2 ras-related protein RABC2a-like
TCONS_00018673	-0.128174	-0.181431	-0.0946822	BGIOSGA002366	XP_015698986.1 calponin homology domain-containing protein DDB_G0272472-like
TCONS_00009332	-0.12808	-0.0702911	0.0280992	BGIOSGA011876	XP_021301803.1uncharacterized protein LOC8063279 isoform X1
TCONS_00022432	-0.128065	-0.165258	0.18088	BGIOSGA021031	XP_015694156.1 protein split ends
TCONS_00021112	-0.127981	-0.146528	0.451663	BGIOSGA023335	XP_015694289.1 E3 ubiquitin-protein ligase KEG isoform X2
TCONS_00007275	-0.127979	0.859546	0.130333	BGIOSGA007208	XP_004952049.13-isopropylmalate dehydratase large subunit, chloroplastic isoform X1
TCONS_00010314	-0.127911	0.119335	0.0243662	BGIOSGA000749	XP_015689938.1 ninja-family protein Os03g0419100
TCONS_00024953	-0.127896	0.249667	0.130539	BGIOSGA024441	XP_002461868.1zinc finger protein VAR3, chloroplastic
TCONS_00005957	-0.12785	0.109329	-0.483083	BGIOSGA020262	XP_006655700.2 cytochrome P450 88A1-like
TCONS_00032701	-0.127548	-0.22281	-0.219818	BGIOSGA031410	XP_006662599.1 autophagy-related protein 3
TCONS_00004299	-0.127497	-0.148393	0.152343	#N/A	#N/A
TCONS_00019081	-0.127179	-1.1536	-0.423147	BGIOSGA030138	XP_015692047.1 phospholipid-transporting ATPase 2-like isoform X3
TCONS_00028843	-0.127129	-0.860694	-0.434593	BGIOSGA030595	XP_010238089.1isochorismate synthase 1, chloroplastic isoform X2
TCONS_00005006	-0.126904	-0.777822	0.113569	BGIOSGA016316	XP_003564973.1probable glucuronosyltransferase Os01g0926600
TCONS_00035863	-0.126842	-0.13767	-0.0880775	BGIOSGA037235	XP_015698265.1 DExH-box ATP-dependent RNA helicase DExH9
TCONS_00013529	-0.126815	-1.52711	-0.559933	BGIOSGA009453	XP_006665086.2 probable acyl-activating enzyme 16, chloroplastic
TCONS_00017198	-0.126492	-0.0118237	-0.0348392	BGIOSGA012407	XP_006655022.2 6-phosphofructo-2-kinase/fructose-2,6-bisphosphatase
TCONS_00002228	-0.126485	-1.69251	-4.00557	BGIOSGA011043	XP_006645074.1 fructose-1,6-bisphosphatase, cytosolic
TCONS_00001917	-0.126457	1.01532	0.451545	BGIOSGA004553	XP_015690113.1 conserved oligomeric Golgi complex subunit 1
TCONS_00015984	-0.12625	0.514106	0.211125	BGIOSGA012802	XP_004975776.1probable protein phosphatase 2C 40
TCONS_00012250	-0.126128	0.404292	0.20852	BGIOSGA026370	XP_006650067.2PREDICTED: uncharacterized protein LOC102707566 isoform X1
TCONS_00004938	-0.126041	-0.899805	-1.3464	BGIOSGA006774	XP_006646591.1 glycine-rich cell wall structural protein 1.8-like
TCONS_00028441	-0.126033	0.496744	0.355281	BGIOSGA026523	XP_006660365.1 probable 26S proteasome non-ATPase regulatory subunit 3
TCONS_00018026	-0.12602	0.19582	0.916824	BGIOSGA000594	XP_006654557.1 transmembrane protein 56-B-like
TCONS_00011901	-0.125974	-1.14026	-1.3433	BGIOSGA026530	XP_015690356.1 geraniol 8-hydroxylase-like
TCONS_00036734	-0.125729	-0.18049	0.197019	BGIOSGA036632	XP_006663841.1 probable serine/threonine-protein kinase WNK9
TCONS_00022930	-0.125722	-0.788556	-0.511788	BGIOSGA020515	XP_006656503.2PREDICTED: uncharacterized protein LOC102700347
TCONS_00028473	-0.125466	-2.12921	-1.36247	BGIOSGA029235	XP_015696157.1 serine/arginine repetitive matrix protein 1-like isoform X1
TCONS_00034094	-0.12539	3.25547	2.95811	BGIOSGA003641	XP_010041882.1 heat shock 70 kDa protein cognate 1-like
TCONS_00006479	-0.125377	0.385374	0.0827186	BGIOSGA016761	XP_006648850.1 inositol phosphorylceramide glucuronosyltransferase 1-like
TCONS_00030305	-0.12528	0.406431	0.457764	BGIOSGA022549	XP_002462611.2uncharacterized protein LOC8054763
TCONS_00008700	-0.125199	0.499506	0.128463	BGIOSGA005730	XP_006647798.1 clustered mitochondria protein isoform X1
TCONS_00030058	-0.125177	-0.56021	-0.437186	BGIOSGA000703	XP_006660633.1 BTB/POZ domain-containing protein NPY1-like

TCONS_00001836	-0.125173	0.654124	0.95927	BGIOSGA008536	XP_006646330.1 GTP-binding protein TypA/BipA homolog isoform X1
TCONS_00034189	-0.124881	-0.989695	-0.547148	BGIOSGA000190	XP_015698575.1 protein LURP-one-related 8-like
TCONS_00000521	-0.124844	-0.937308	-0.617429	BGIOSGA010052	XP_006643969.2 protein NLP3
TCONS_00025162	-0.124839	0.0141912	0.401346	BGIOSGA024251	XP_006658595.1 WD repeat-containing protein DDB_G0290555
TCONS_00034033	-0.124834	-0.342417	-0.238263	BGIOSGA022090	XP_006663107.1 splicing factor U2af large subunit B isoform X2
TCONS_00032728	-0.124742	-1.65633	-1.6128	BGIOSGA031379	XP_006662080.1 chlorophyllide a oxygenase, chloroplastic
TCONS_00024550	-0.124528	-0.438332	-0.2332	BGIOSGA024840	XP_015694468.1 oxygen-evolving enhancer protein 2, chloroplastic-like
TCONS_00036853	-0.124486	1.08524	-0.550569	BGIOSGA040681	YP_008815755.1NADH-plastoquinone oxidoreductase subunit K (plastid)
TCONS_00023802	-0.124403	0.314433	0.0414745	BGIOSGA025837	XP_006657777.1 protein disulfide isomerase-like 5-4
TCONS_00030260	-0.124073	-0.200919	0.246999	BGIOSGA016530	XP_015696822.1 ETHYLENE INSENSITIVE 3-like 3 protein
TCONS_00013116	-0.124012	0.124222	0.0669056	BGIOSGA013360	XP_006651781.1 calcium-transporting ATPase 3, endoplasmic reticulum-type
TCONS_00035040	-0.123865	-0.759846	-0.583328	BGIOSGA036188	XP_006662984.1 protein yippee-like At4g27745
TCONS_00020660	-0.123851	-0.16982	-0.360118	BGIOSGA033536	XP_015693568.1 probable LRR receptor-like serine/threonine-protein kinase At3g47570
TCONS_00031236	-0.123706	0.674571	0.352252	BGIOSGA011451	XP_010234783.1methyltransferase-like protein 1
TCONS_00024012	-0.123553	-1.19844	0.791447	BGIOSGA011785	XP_006654944.1 probable glucuronosyltransferase Os05g0123100
TCONS_00031887	-0.123466	-0.862763	-0.353981	BGIOSGA000124	XP_002457173.1lecithin-cholesterol acyltransferase-like 1
TCONS_00004495	-0.123409	-0.681585	0.234325	BGIOSGA037728	XP_006644794.1 MATH domain-containing protein At5g43560-like isoform X2
TCONS_00016919	-0.123357	-1.64614	-0.613433	BGIOSGA014043	XP_006653063.1PREDICTED: uncharacterized protein LOC102705811 isoform X1
TCONS_00015489	-0.123252	-0.114336	-2.96043	BGIOSGA017108	XP_006652134.1 probable cinnamyl alcohol dehydrogenase 6
TCONS_00033512	-0.123124	0.160999	0.429216	BGIOSGA035306	XP_006663466.1 zinc finger protein 511
TCONS_00015079	-0.122788	-1.46244	-1.45439	BGIOSGA019120	XP_015691689.1 endonuclease 2-like
TCONS_00000266	-0.122671	-0.240414	-0.283468	BGIOSGA002847	XP_006645528.1 Golgi to ER traffic protein 4 homolog
TCONS_00011635	-0.122632	-0.190618	0.350271	BGIOSGA021438	XP_014752695.1O-fucosyltransferase 3
TCONS_00034596	-0.122618	-0.949087	-0.173544	BGIOSGA002391	XP_015697829.1 putative disease resistance protein RGA4
TCONS_00013499	-0.122566	0.519056	0.0601703	BGIOSGA009485	XP_006650873.1 E3 ubiquitin-protein ligase RNF170-like
TCONS_00010077	-0.1225	0.187283	-0.253699	BGIOSGA022638	XP_015690894.1 DNA helicase INO80
TCONS_00025595	-0.122386	-0.688741	-0.296776	BGIOSGA012702	XP_006658023.1 pyrrolidone-carboxylate peptidase
TCONS_00024225	-0.122252	-0.22687	0.728279	BGIOSGA029161	XP_006658049.1 DEAD-box ATP-dependent RNA helicase 57
TCONS_00010846	-0.122161	0.16225	-0.208032	BGIOSGA019093	XP_015698585.1 E3 SUMO-protein ligase SIZ2-like
TCONS_00007032	-0.122134	1.213	0.735971	BGIOSGA019817	XP_015688758.1PREDICTED: uncharacterized protein LOC102708695
TCONS_00010225	-0.122087	-0.375673	-0.300342	BGIOSGA012790	XP_004984152.1uric acid degradation bifunctional protein TTL
TCONS_00008886	-0.122042	0.429568	0.140863	BGIOSGA005524	XP_006647978.1PREDICTED: uncharacterized protein LOC102703462 isoform X3
TCONS_00005882	-0.121932	-0.0425089	-1.65983	BGIOSGA024887	XP_004986219.1L-type lectin-domain containing receptor kinase IV.1
TCONS_00037348	-0.121866	0.504742	-0.0689584	BGIOSGA019053	XP_006664096.1 transmembrane protein 205-like
TCONS_00013155	-0.121848	-0.386226	-0.581201	BGIOSGA014083	XP_003559774.1stem-specific protein TSJT1
TCONS_00029555	-0.121765	-0.512884	0.0287321	BGIOSGA005351	XP_006661579.2 P-loop NTPase domain-containing protein LPA1 homolog
TCONS_00006706	-0.12174	-0.779203	0.0802173	BGIOSGA017049	XP_015688982.1 cell division cycle 20.2, cofactor of APC complex-like
TCONS_00031739	-0.121399	-0.835028	-0.336535	BGIOSGA010923	XP_015697361.1 barley B recombinant-like protein A
TCONS_00018264	-0.121117	0.684098	-1.1854	BGIOSGA014196	XP_020395231.1LEAF RUST 10 DISEASE-RESISTANCE LOCUS RECEPTOR-LIKE PROTEIN KINASE-like 1.2
TCONS_00000187	-0.121037	-0.938071	0.618389	BGIOSGA010055	XP_006643748.1 glucose-induced degradation protein 4 homolog isoform X1
TCONS_00001644	-0.121004	-0.454369	-0.135345	BGIOSGA004278	XP_006644575.1PREDICTED: uncharacterized protein LOC102700283 isoform X1
TCONS_00017664	-0.121003	-3.39738	-3.98485	#N/A	#N/A
TCONS_00016796	-0.120997	0.48325	0.154157	BGIOSGA014171	XP_006653845.1 serine/arginine repetitive matrix protein 2
TCONS_00036936	-0.120854	-0.304206	-0.633668	BGIOSGA040203	YP_005090186.1ribulose-1,5-bisphosphate carboxylase/oxygenase large subunit (chloroplast)
TCONS_00016817	-0.120643	-0.758853	-0.295791	BGIOSGA019067	XP_006652979.2 kinesin-like calmodulin-binding protein homolog
TCONS_00020357	-0.120492	-0.796944	-1.58441	BGIOSGA023024	XP_004964885.12-hydroxyisoflavanone dehydratase
TCONS_00024254	-0.12048	2.00148	1.54096	BGIOSGA037243	XP_015695168.1 protein HUA2-LIKE 2-like
TCONS_00014391	-0.120437	-2.27553	-1.06592	BGIOSGA014332	XP_015691663.1 NAC domain-containing protein 92-like
TCONS_00033665	-0.120314	-0.938787	0.183333	#N/A	#N/A
TCONS_00024038	-0.120265	-0.434596	-0.0818307	BGIOSGA026076	XP_015695434.1 DNA annealing helicase and endonuclease ZRANB3
TCONS_00000461	-0.120013	-0.00439638	-0.276059	BGIOSGA026210	XP_006643930.2 SMR domain-containing protein At5g58720-like
TCONS_00020615	-0.11987	-0.175576	0.434147	BGIOSGA022799	XP_006656040.1 CDK5RAP3-like protein

transcriptome

TCONS_00017977	-0.119808	0.200405	1.0999	BGIOSGA022398	XP_015692630.1PREDICTED: uncharacterized protein LOC102717994
TCONS_00027889	-0.11963	-0.724422	0.263328	BGIOSGA002737	XP_015695736.1 lipid phosphate phosphatase 2-like
TCONS_00029796	-0.119473	-0.12216	0.0349043	BGIOSGA030019	XP_024310935.1ethylene-responsive transcription factor ERF118
TCONS_00013646	-0.119308	-0.25903	-0.416648	BGIOSGA015686	XP_006653121.2 receptor-like protein 2
TCONS_00016841	-0.119275	-0.208604	-0.126345	BGIOSGA011771	XP_006653003.1 probable adenylate kinase 6, chloroplastic
TCONS_00014653	-0.119152	-0.700289	-0.763464	BGIOSGA008015	XP_014661017.1L-type lectin-domain containing receptor kinase IV.1
TCONS_00017959	-0.118808	0.168474	0.655374	BGIOSGA000548	XP_015692627.1 selT-like protein
TCONS_00036314	-0.118678	-0.0743595	0.468801	BGIOSGA013103	XP_015698339.1PREDICTED: uncharacterized protein LOC102719168, partial
TCONS_00024929	-0.118618	-1.22398	-0.866075	BGIOSGA009442	XP_006657620.1 B-cell receptor-associated protein 31-like
TCONS_00035106	-0.118512	-0.336292	0.217937	BGIOSGA005758	XP_006663009.1 TBC1 domain family member 15-like
TCONS_00015231	-0.118321	1.35475	0.182496	BGIOSGA017417	XP_021318722.1proteinaceous RNase P 1, chloroplastic/mitochondrial isoform X5
TCONS_00014666	-0.118271	0.0584802	0.159254	BGIOSGA018900	XP_015697132.1PREDICTED: uncharacterized protein LOC102707437 isoform X3
TCONS_00028543	-0.11819	0.376707	0.534509	BGIOSGA030289	XP_006663966.1 CCAAT/enhancer-binding protein zeta
TCONS_00013561	-0.118058	0.365521	-0.0269651	BGIOSGA009414	XP_006650920.1 exosome complex exonuclease RRP46 homolog
TCONS_00025493	-0.11792	-0.602629	-0.93035	BGIOSGA023942	XP_015695425.1 uncharacterized hydrolase YugF-like
TCONS_00027034	-0.117898	-0.162876	-0.147477	BGIOSGA015425	XP_006659645.2 biotin synthase
TCONS_00019367	-0.117761	0.842742	0.671378	BGIOSGA017392	XP_006654462.1 tubby-like F-box protein 8
TCONS_00016409	-0.117693	0.879761	-0.0519696	BGIOSGA005883	XP_006652633.1 mitogen-activated protein kinase kinase kinase YODA-like
TCONS_00022984	-0.117581	-0.292736	0.134683	BGIOSGA025015	XP_006658208.2 DDB1- and CUL4-associated factor 4
TCONS_00005444	-0.117544	0.713031	-0.467615	BGIOSGA020618	XP_002451556.1putative adagio-like protein 2
TCONS_00005936	-0.117493	0.217581	0.291114	BGIOSGA018983	XP_006647220.1 protein MEI2-like 5
TCONS_00010289	-0.117285	-1.14623	-0.831695	BGIOSGA023919	XP_015691250.1 nuclear transcription factor Y subunit A-3-like
TCONS_00027485	-0.117128	-0.811309	-0.0772836	BGIOSGA016505	XP_015696143.1 polyadenylate-binding protein RBP45-like
TCONS_00025732	-0.117082	-0.423942	0.266011	BGIOSGA013543	XP_015695099.1 UPF0483 protein CG5412-like
TCONS_00007432	-0.117064	-0.402265	-0.187424	BGIOSGA021804	XP_015689133.1 myb family transcription factor APL
TCONS_00022746	-0.116934	1.00529	-0.0163605	BGIOSGA020711	XP_015694239.1PREDICTED: uncharacterized protein LOC102706205 isoform X2
TCONS_00016420	-0.116746	-0.540041	-0.445517	BGIOSGA005866	XP_006652644.1 protein LAZ1-like
TCONS_00008364	-0.11661	0.123865	1.08999	BGIOSGA006061	XP_015689491.1 eukaryotic translation initiation factor isoform 4G-2-like
TCONS_00032159	-0.116572	-0.675795	-0.264524	BGIOSGA011968	XP_006661747.1 general transcription factor IIE subunit 2
TCONS_00035000	-0.116571	-1.19731	-0.486509	BGIOSGA035394	XP_023157350.1F-box/FBD/LRR-repeat protein At1g13570-like isoform X1
TCONS_00007220	-0.116479	-0.895115	-1.03251	BGIOSGA031848	XP_006646771.2 GDSL esterase/lipase At5g45950-like
TCONS_00019273	-0.116317	0.411788	-1.3202	BGIOSGA018078	XP_015692595.1 pyruvate, phosphate dikinase 1, chloroplastic
TCONS_00037581	-0.116227	1.26123	2.71747	BGIOSGA019389	XP_015698376.1 glycine-rich RNA-binding protein GRP1A-like
TCONS_00016078	-0.116128	1.04786	1.48754	BGIOSGA014902	XP_003579954.2uncharacterized protein LOC100838142
TCONS_00030405	-0.116012	-0.373501	-0.0799784	BGIOSGA029115	XP_006660874.1 GTPase-activating protein gyp7-like
TCONS_00028988	-0.115938	-0.0477968	0.844848	BGIOSGA021201	XP_006660619.1 pentatricopeptide repeat-containing protein At2g36730
TCONS_00001246	-0.115767	-0.496382	-1.37314	BGIOSGA029125	XP_004969045.1light-harvesting complex-like protein OHP2, chloroplastic
TCONS_00031229	-0.115723	0.0852958	0.817027	BGIOSGA033047	XP_015696903.1PREDICTED: uncharacterized protein LOC102702975 isoform X1
TCONS_00022218	-0.115438	0.1544	-0.0974398	BGIOSGA021279	XP_006656066.2 acyl-coenzyme A oxidase 3, peroxisomal-like
TCONS_00018666	-0.115326	-3.6675	-1.19177	BGIOSGA018768	XP_006655015.1 GDSL esterase/lipase At1g09390-like
TCONS_00004689	-0.115317	-0.662249	-0.411236	BGIOSGA003876	XP_006658054.1 serine/threonine-protein kinase AFC1-like
TCONS_00020159	-0.115014	-0.410462	0.378618	BGIOSGA022356	XP_015693456.1 protein-tyrosine sulfotransferase
TCONS_00034340	-0.114913	0.262117	-0.94618	BGIOSGA014056	XP_006662750.1 exocyst complex component EXO70A1-like
TCONS_00000314	-0.11489	0.282271	0.0348277	BGIOSGA002888	NP_001149760.1syntaxin 32
TCONS_00005079	-0.114868	0.0204008	0.0967797	BGIOSGA014071	XP_006645319.2 DNA repair protein recA homolog 2, mitochondrial
TCONS_00026674	-0.114865	-0.0487151	1.55855	BGIOSGA009832	XP_006659446.2 probable CCR4-associated factor 1 homolog 7
TCONS_00016821	-0.114836	0.114282	1.05827	BGIOSGA030459	NP_001148222.1ubiquitin-conjugating enzyme E2-17 kDa
TCONS_00007389	-0.114788	-0.260361	-0.0211147	BGIOSGA035923	XP_006646907.1 CBL-interacting protein kinase 26
TCONS_00003148	-0.114775	0.381599	-1.51698	BGIOSGA007727	XP_006645677.1 transcription factor bHLH68-like isoform X2
TCONS_00025130	-0.114705	0.365692	-0.705684	BGIOSGA024275	XP_006658575.1 pentatricopeptide repeat-containing protein At4g17616
TCONS_00026412	-0.114514	0.703198	-0.360091	BGIOSGA030776	XP_020399314.1heat shock protein binding protein isoform X1
TCONS_00019507	-0.1145	-0.0597337	0.623299	BGIOSGA013816	XP_006655420.1 KH domain-containing protein SPIN1-like

TCONS_00031714	-0.114483	0.226925	0.146293	BGIOSGA014539	XP_006661595.1PREDICTED: uncharacterized protein LOC102707710 isoform X1
TCONS_00017341	-0.11443	-2.82063	-3.43577	BGIOSGA004403	XP_010911738.2 BURP domain-containing protein 6-like
TCONS_00011369	-0.11431	0.086854	1.76879	BGIOSGA013984	XP_015690040.1 ribosome-binding factor PSRP1, chloroplastic
TCONS_00013425	-0.114167	-0.484556	-0.523758	BGIOSGA032090	XP_006654522.1 40S ribosomal protein S23
TCONS_00031609	-0.114018	-0.0860405	-0.491347	BGIOSGA028008	XP_015697073.1 oxysterol-binding protein-related protein 1B-like
TCONS_00029053	-0.114003	0.745944	0.264443	BGIOSGA030801	XP_006660647.1PREDICTED: uncharacterized protein LOC102720455 isoform X1
TCONS_00035738	-0.113995	-0.305912	0.435782	BGIOSGA033263	XP_006644633.1PREDICTED: uncharacterized protein At4g10930-like isoform X1
TCONS_00019138	-0.113957	-0.262287	0.793109	BGIOSGA010985	XP_004962350.1lysophospholipid acyltransferase LPEAT1 isoform X2
TCONS_00036360	-0.113668	-0.157138	-0.0655406	BGIOSGA006666	XP_004963077.1calcineurin B-like protein 2
TCONS_00031191	-0.113579	0.278096	-0.902108	BGIOSGA021860	XP_006662378.2 soluble starch synthase 2-1, chloroplastic/amyloplastic isoform X2
TCONS_00021271	-0.113433	-0.319393	0.166075	BGIOSGA023497	XP_015693687.1 cell wall integrity protein scw1
TCONS_00027393	-0.113343	-1.40087	-1.3098	BGIOSGA011002	XP_002445102.1probable mixed-linked glucan synthase 6
TCONS_00036836	-0.113159	0.233284	-0.19075	BGIOSGA019707	XP_006663901.2PREDICTED: uncharacterized protein LOC102704378
TCONS_00023574	-0.11308	0.208555	-0.160628	BGIOSGA037714	XP_015695183.1PREDICTED: uncharacterized protein LOC102718006
TCONS_00024128	-0.112903	-0.617447	0.0728361	BGIOSGA006276	XP_015694731.1 serine/threonine-protein kinase SAPK2
TCONS_00002593	-0.112886	-1.77981	-1.58694	BGIOSGA023924	XP_006645351.1 abscisic stress-ripening protein 2-like
TCONS_00000164	-0.112881	-0.500661	-1.68329	BGIOSGA001801	XP_006643729.1PREDICTED: uncharacterized protein LOC102708932 isoform X2
TCONS_00017854	-0.112592	0.090312	-0.050147	BGIOSGA038889	YP_002000495.1ribulose-1,5-bisphosphate carboxylase/oxygenase large subunit (chloroplast)
TCONS_00010247	-0.112502	0.320486	-0.41879	BGIOSGA012810	XP_006650170.1 pre-mRNA-processing factor 17
TCONS_00037751	-0.112257	-0.27028	1.6465	BGIOSGA039198	XP_017696574.1 DNA-directed RNA polymerase III subunit RPC4-like
TCONS_00010494	-0.112227	-0.511342	-0.598004	BGIOSGA013103	XP_006650280.1PREDICTED: uncharacterized protein LOC102722752, partial
TCONS_00020824	-0.112226	0.629635	0.0424143	BGIOSGA022570	XP_006657017.1 uncharacterized membrane protein YuiD
TCONS_00000882	-0.112085	0.0612691	-0.136226	BGIOSGA007466	XP_006644156.1 glyoxysomal fatty acid beta-oxidation multifunctional protein MFP-a-like
TCONS_00016999	-0.112028	-0.451136	-1.24195	BGIOSGA029236	XP_003569001.1ACT domain-containing protein ACR9 isoform X2
TCONS_00018509	-0.112002	0.162982	0.431241	BGIOSGA018925	XP_006653983.2PREDICTED: uncharacterized protein LOC102721549
TCONS_00006207	-0.111802	0.743179	0.598854	BGIOSGA008370	XP_024317515.1uncharacterized protein LOC100843176
TCONS_00031895	-0.111772	0.971396	0.386725	BGIOSGA029874	XP_006661656.1 enolase
TCONS_00009934	-0.111575	0.830561	0.673502	BGIOSGA012484	XP_006651318.1PREDICTED: uncharacterized protein LOC102704504
TCONS_00020486	-0.111466	0.131055	0.754042	BGIOSGA015293	XP_006655985.1 ubiquitin receptor RAD23d-like
TCONS_00001061	-0.111436	1.09479	1.20062	BGIOSGA003677	XP_010231885.1FK506-binding protein 3 isoform X2
TCONS_00013393	-0.110949	-1.04946	-0.315002	BGIOSGA024615	XP_010228007.1nudix hydrolase 16, mitochondrial
TCONS_00034626	-0.110891	0.174906	0.744735	BGIOSGA008596	XP_006663345.2 acetolactate synthase small subunit 2, chloroplastic-like
TCONS_00001070	-0.110872	0.233775	0.382117	BGIOSGA025051	XP_015691873.1PREDICTED: uncharacterized protein LOC102699681
TCONS_00002661	-0.110828	-0.0797672	0.415809	BGIOSGA011993	XP_006643638.1 C-type lectin receptor-like tyrosine-protein kinase At1g52310 isoform X1
TCONS_00029793	-0.110538	-1.30058	-0.249811	BGIOSGA004250	XP_015696801.1 lipid phosphate phosphatase 2-like
TCONS_00036899	-0.110364	-0.0976459	0.994105	BGIOSGA025439	NP_001150181.1membrane steroid-binding protein 1
TCONS_00000929	-0.110232	0.0169773	-0.327282	BGIOSGA005043	XP_022682619.1probable serine/threonine-protein kinase At1g54610 isoform X2
TCONS_00007556	-0.110056	-1.27161	-0.508195	BGIOSGA006928	XP_006647042.2PREDICTED: uncharacterized protein LOC102708405
TCONS_00000763	-0.110056	0.557769	0.841803	BGIOSGA036953	XP_008655848.1E3 ubiquitin-protein ligase EL5
TCONS_00023086	-0.109971	0.168855	0.0618537	BGIOSGA025118	XP_012699449.1GIGYF family protein CG11148
TCONS_00024606	-0.109852	-0.541185	0.102515	BGIOSGA016456	XP_006657465.1 outer envelope protein 61
TCONS_00009757	-0.10963	0.301974	0.453716	BGIOSGA014455	XP_006649829.1 AT-hook motif nuclear-localized protein 22
TCONS_00008165	-0.109561	0.320077	0.601275	BGIOSGA006275	XP_004952835.1nascent polypeptide-associated complex subunit alpha, muscle-specific form
TCONS_00013572	-0.109464	0.281489	0.263296	BGIOSGA009403	XP_006650931.1 protein EXPORTIN 1A-like
TCONS_00009057	-0.109408	0.202512	-0.168849	BGIOSGA001699	XP_006648131.1 serine/threonine-protein phosphatase PP1
TCONS_00019957	-0.109313	0.178188	-0.0842255	BGIOSGA022098	XP_006655685.1PREDICTED: uncharacterized protein LOC102721280
TCONS_00031399	-0.109276	-1.51795	-1.40131	BGIOSGA014789	XP_006654334.1 AB hydrolase superfamily protein YfhM-like
TCONS_00014927	-0.109124	0.103647	-0.162742	BGIOSGA030915	XP_006652816.1 peroxisomal 2,4-dienoyl-CoA reductase
TCONS_00035888	-0.108937	-1.03746	-1.53821	BGIOSGA007558	XP_006663954.1 ribulose bisphosphate carboxylase small chain, chloroplastic-like
TCONS_00015810	-0.108902	-0.562886	0.134691	BGIOSGA015176	XP_015692242.1 WASH complex subunit strumpellin homolog isoform X1
TCONS_00011839	-0.108882	0.140651	-0.127569	BGIOSGA006318	XP_002468223.1protein SHORT HYPOCOTYL IN WHITE LIGHT 1

TCONS_00017957	-0.108793	-0.0679729	-0.130321	BGIOSGA022922	XP_010504143.1 histone H4
TCONS_00023349	-0.108755	0.455934	-0.739992	BGIOSGA003804	XP_004955807.1chloroplast stem-loop binding protein of 41 kDa a, chloroplastic
TCONS_00024885	-0.108746	-1.40202	-0.0508759	BGIOSGA001906	XP_015696506.1 protein argonaute 16
TCONS_00004439	-0.108651	0.211625	0.0140733	BGIOSGA000784	XP_006644741.1PREDICTED: uncharacterized protein LOC102702332
TCONS_00005849	-0.108332	-0.667419	-0.0264115	BGIOSGA021625	XP_006647169.1 probable LRR receptor-like serine/threonine-protein kinase At5g10290
TCONS_00009931	-0.108263	-0.750387	-0.647395	BGIOSGA017432	XP_006647285.1 DNA repair protein RAD50 isoform X1
TCONS_00020554	-0.108213	0.336213	-0.036146	BGIOSGA028724	XP_006656007.2 U-box domain-containing protein 35
TCONS_00006216	-0.108155	-0.989223	-0.92631	BGIOSGA034845	XP_006647351.2 digalactosyldiacylglycerol synthase 1, chloroplastic-like
TCONS_00006468	-0.108106	-0.634359	-0.510528	BGIOSGA015013	NP_001148658.2enzyme of the cupin superfamily
TCONS_00016780	-0.108096	0.00205368	-0.100704	BGIOSGA014185	XP_015691674.1 ribulose bisphosphate carboxylase/oxygenase activase, chloroplastic
TCONS_00013559	-0.107865	0.902502	1.12027	BGIOSGA003043	XP_006650919.1 protein BCCIP homolog
TCONS_00005228	-0.10782	1.07341	0.333724	BGIOSGA037972	XP_004951200.1uncharacterized protein LOC101766807
TCONS_00019378	-0.107818	0.702602	0.144404	BGIOSGA017970	XP_015692870.1 histone deacetylase 8-like
TCONS_00016945	-0.107789	0.624629	0.148582	BGIOSGA018997	XP_023157534.1zinc finger BED domain-containing protein RICESLEEPER 2-like
TCONS_00016890	-0.107786	0.167224	0.158833	BGIOSGA014072	XP_006653039.1 acyl-CoA-binding domain-containing protein 1-like
TCONS_00014455	-0.107688	-0.979125	0.17675	BGIOSGA016617	NP_001132121.1uncharacterized LOC100193538 precursor
TCONS_00020991	-0.107654	1.11595	0.804673	BGIOSGA015386	XP_003563668.1beta-glucuronosyltransferase GlcAT14A
TCONS_00012117	-0.107563	0.462969	1.64498	BGIOSGA029291	XP_006649956.1 acetate/butyrate--CoA ligase AAE7, peroxisomal
TCONS_00015161	-0.107531	0.0298246	-0.199306	BGIOSGA017341	XP_006653022.1 sacsin
TCONS_00027725	-0.107405	1.49635	0.453672	BGIOSGA028024	XP_006659267.1 tobamovirus multiplication protein 2A
TCONS_00025037	-0.106992	-0.25084	0.36901	BGIOSGA024369	XP_015695431.1 tetratricopeptide repeat protein 27 homolog
TCONS_00006201	-0.106953	1.26705	-0.107915	BGIOSGA030373	XP_006647344.1 beta-fructofuranosidase, insoluble isoenzyme 1
TCONS_00015248	-0.106904	-0.021617	-0.603467	BGIOSGA015734	XP_015691347.1 UMP-CMP kinase 2-like
TCONS_00012365	-0.106855	-0.257396	0.358224	BGIOSGA026180	XP_015690991.1 fibrous sheath CABYR-binding protein-like isoform X1
TCONS_00021430	-0.106701	-0.955599	-0.635969	BGIOSGA009320	XP_004966574.1signal peptide peptidase-like 5
TCONS_00034128	-0.106593	1.81214	-1.67065	BGIOSGA036792	XP_006662652.1 ferritin-1, chloroplastic-like
TCONS_00032484	-0.106531	0.326588	-0.242109	BGIOSGA001307	XP_008673420.1glycosyltransferase isoform X1
TCONS_00021043	-0.106391	0.0919473	0.00216817	BGIOSGA023274	XP_006657140.1PREDICTED: uncharacterized protein LOC102722226 isoform X2
TCONS_00007012	-0.106378	-0.869738	-0.212785	BGIOSGA030637	XP_015689589.1 nudix hydrolase 3 isoform X1
TCONS_00002418	-0.106247	0.359731	0.0250025	BGIOSGA005060	XP_003564936.1E3 ubiquitin-protein ligase complex slx8-rfp subunit slx8
TCONS_00006941	-0.106161	-2.9585	#NA	#N/A	#N/A
TCONS_00004996	-0.106074	-1.15204	-0.19798	BGIOSGA029470	XP_006660046.2 60S ribosomal protein L7a
TCONS_00032334	-0.106048	1.232	0.551715	BGIOSGA004325	XP_015698373.1 KRR1 small subunit processome component homolog
TCONS_00007009	-0.105966	0.637708	-0.821207	BGIOSGA026618	XP_003570283.1protein TIC 55, chloroplastic
TCONS_00008302	-0.10561	-2.26139	-0.616008	BGIOSGA006126	XP_006647453.2 rhodanese-like domain-containing protein 11, chloroplastic
TCONS_00000345	-0.105553	0.92177	0.232035	BGIOSGA015509	XP_004968473.1uncharacterized protein LOC101780552
TCONS_00029628	-0.105385	-1.6281	-1.23503	BGIOSGA020956	XP_006664888.1 DNA-binding protein SMUBP-2-like
TCONS_00023167	-0.105361	-0.278821	-0.288415	#N/A	#N/A
TCONS_00037538	-0.105273	1.8531	-0.195526	BGIOSGA031399	XP_015698578.1PREDICTED: uncharacterized protein LOC102703263
TCONS_00013391	-0.105229	-1.41917	0.360091	BGIOSGA009593	XP_006651942.2PREDICTED: uncharacterized protein LOC102707020, partial
TCONS_00017043	-0.105131	-3.16702	-0.681662	BGIOSGA014196	XP_004960359.1L-type lectin-domain containing receptor kinase VIII.2
TCONS_00008642	-0.104778	-2.18692	-1.27864	BGIOSGA016768	XP_015688380.1 V-type proton ATPase subunit G1-like isoform X2
TCONS_00024261	-0.104624	-1.1325	0.162192	BGIOSGA014893	XP_015695101.1 beta-glucosidase 26
TCONS_00016413	-0.104574	-1.6586	-1.35239	BGIOSGA037790	XP_015691539.1 calcium-dependent protein kinase 29-like
TCONS_00017231	-0.104497	-0.842874	-0.308805	BGIOSGA021947	XP_006654103.1 pre-mRNA-splicing factor 38B-like isoform X1
TCONS_00003925	-0.104376	-1.6951	-0.553908	BGIOSGA007821	XP_006646071.1 inorganic pyrophosphatase 3-like
TCONS_00019726	-0.104317	-0.474489	0.0376589	BGIOSGA017636	XP_021315696.1uncharacterized protein LOC110434946 isoform X3
TCONS_00037315	-0.104307	0.304809	0.0936179	BGIOSGA035053	XP_006664614.1 5'-adenylylsulfate reductase-like 6
TCONS_00029479	-0.104155	0.788693	0.72313	BGIOSGA031233	XP_021308931.1nicotinate-nucleotide pyrophosphorylase [carboxylating], chloroplastic
TCONS_00004312	-0.104036	1.3753	0.358424	BGIOSGA006837	XP_006656160.1 40S ribosomal protein S24-1-like
TCONS_00017426	-0.103918	-1.07081	-0.671147	BGIOSGA038406	XP_006654185.2 probable LRR receptor-like serine/threonine-protein kinase At1g56140 isoform X1
TCONS_00017531	-0.103851	-0.401903	0.214539	BGIOSGA019602	XP_006655191.1PREDICTED: uncharacterized protein LOC102719116
TCONS_00018203	-0.103687	0.595427	0.592354	BGIOSGA001317	XP_006655528.1 mitochondrial outer membrane protein porin 2
TCONS_00006921	-0.103603	-1.89743	-0.259753	BGIOSGA025264	XP_015693577.1 AP2/ERF and B3 domain-containing protein Os01g0141000-like

transcriptome

TCONS_00029978	-0.103575	-0.212037	-0.0803675	BGIOSGA029819	XP_006660595.1 enhanced ethylene response protein 5
TCONS_00031029	-0.103532	0.309822	1.1237	BGIOSGA032846	XP_006661735.1 alanine aminotransferase 2
TCONS_00000118	-0.103389	-0.0882829	-0.0844564	BGIOSGA010309	XP_006645454.1 factor of DNA methylation 1-like
TCONS_00007986	-0.103093	0.0892673	0.242263	BGIOSGA007095	XP_015689233.1PREDICTED: uncharacterized protein LOC102707936
TCONS_00032036	-0.102698	0.581	-0.158486	BGIOSGA032648	XP_006661705.1 protein DETOXIFICATION 16-like
TCONS_00012286	-0.102672	-0.267512	0.76903	BGIOSGA002058	XP_015690180.1 E3 ubiquitin-protein ligase SINAT3-like
TCONS_00028280	-0.102657	0.833241	0.994355	BGIOSGA024349	XP_015695661.1 squamosa promoter-binding-like protein 14
TCONS_00029299	-0.102507	-0.537504	0.913854	BGIOSGA031041	XP_006661409.1 cyclin-C1-1
TCONS_00008095	-0.102182	-1.05597	0.562256	BGIOSGA015157	XP_004952609.1COPII coat assembly protein SEC16
TCONS_00018366	-0.102182	1.03713	-0.940244	#N/A	#N/A
TCONS_00024048	-0.102135	0.401489	0.561405	BGIOSGA026085	XP_006657927.2 U3 small nucleolar RNA-associated protein 18 homolog isoform X1
TCONS_00037218	-0.102129	-0.167423	0.258508	BGIOSGA028595	XP_004962955.1translation initiation factor eIF-2B subunit alpha
TCONS_00000449	-0.102002	-0.144931	-0.16985	BGIOSGA017053	XP_006645645.2PREDICTED: uncharacterized protein LOC102714794
TCONS_00017279	-0.101969	-0.0360299	0.0641609	BGIOSGA018343	XP_006654139.1 lysine-specific demethylase JMJ703
TCONS_00018259	-0.101838	1.67609	0.195858	BGIOSGA009102	XP_004961326.1AP2/ERF and B3 domain-containing protein Os05g0549800
TCONS_00033585	-0.101704	0.201991	0.319158	BGIOSGA006509	XP_006663492.1PREDICTED: uncharacterized protein LOC102707074
TCONS_00025799	-0.101442	0.190697	-0.304951	BGIOSGA016652	XP_006659047.1 U-box domain-containing protein 4
TCONS_00035919	-0.101384	0.186509	-1.70287	BGIOSGA003613	XP_006664006.1 chloroplast stem-loop binding protein of 41 kDa b, chloroplastic
TCONS_00014414	-0.101324	0.462533	-0.480613	BGIOSGA027635	XP_015691491.1 probable RNA-dependent RNA polymerase 2
TCONS_00021153	-0.101169	-0.378981	-0.942745	BGIOSGA025368	XP_006656305.1 transcription initiation factor TFIID subunit 5
TCONS_00018040	-0.100854	0.478123	0.880762	BGIOSGA022536	XP_006654569.1 ABSCISIC ACID-INSENSITIVE 5-like protein 2
TCONS_00027445	-0.10078	-0.512009	-0.797301	BGIOSGA024536	XP_003571426.1phenolic glucoside malonyltransferase 1
TCONS_00021553	-0.10076	0.137202	-0.450138	BGIOSGA012239	XP_006655758.1 40S ribosomal protein S20
TCONS_00003320	-0.100753	0.099148	1.44923	BGIOSGA026501	XP_002457650.2calmodulin-binding protein 25
TCONS_00007910	-0.100711	-0.429414	-0.335964	BGIOSGA006568	XP_015689515.1PREDICTED: uncharacterized protein LOC102711407 isoform X1
TCONS_00028290	-0.100682	0.357471	0.377982	BGIOSGA029535	XP_006659579.1PREDICTED: uncharacterized protein LOC102711369
TCONS_00008296	-0.100503	0.0996016	-0.124074	BGIOSGA014880	XP_006647447.2 beta-1,4-mannosyl-glycoprotein 4-beta-N-acetylglucosaminyltransferase-like isoform X1
TCONS_00014366	-0.100474	-3.72793	-2.32846	BGIOSGA036203	XP_003581302.1sugar transport protein MST1
TCONS_00005393	-0.100437	-0.486201	0.384307	BGIOSGA035975	XP_002451521.1molybdopterin synthase catalytic subunit
TCONS_00032982	-0.100374	-0.682853	-0.140056	BGIOSGA034818	XP_008679418.1signal recognition particle 54 kDa protein, chloroplastic isoform X2
TCONS_00004272	-0.100278	0.0468129	-0.425831	BGIOSGA018279	XP_006644603.1 phospholipase A1-II 5 isoform X2
TCONS_00008327	-0.100106	0.9543	0.265207	BGIOSGA006099	XP_006647466.1 transcription factor GTE9-like
TCONS_00036074	-0.099945	-0.783146	-0.126013	BGIOSGA010054	XP_006664039.2 vacuolar protein sorting-associated protein 26A-like isoform X1
TCONS_00007794	-0.0998706	1.8158	0.282917	BGIOSGA035413	XP_003572542.1ABC transporter E family member 2 isoform X1
TCONS_00018568	-0.0996292	0.209368	0.102343	BGIOSGA018864	XP_003569073.1protein EMBRYO SAC DEVELOPMENT ARREST 30
TCONS_00013817	-0.0994026	-0.741589	-0.904259	BGIOSGA014014	XP_006652121.2PREDICTED: uncharacterized protein LOC102703480
TCONS_00002820	-0.0992436	0.415391	0.661577	BGIOSGA029493	XP_004294038.1 histone deacetylase HDT1-like
TCONS_00008505	-0.0992239	-0.404888	0.131278	BGIOSGA035269	XP_015689576.1 ELMO domain-containing protein A-like
TCONS_00010206	-0.0992118	-0.697251	-0.430242	BGIOSGA005049	XP_015691204.1 uncharacterized WD repeat-containing protein C2A9.03-like isoform X1
TCONS_00012971	-0.099072	-1.36723	1.16457	BGIOSGA010021	XP_015690023.1 tetratricopeptide repeat protein 38
TCONS_00036666	-0.0988327	0.449221	-0.071172	BGIOSGA005605	XP_004978649.1structure-specific endonuclease subunit slx1
TCONS_00010875	-0.0987358	-0.240386	-0.49445	BGIOSGA013498	XP_015691314.1PREDICTED: uncharacterized protein LOC102700881
TCONS_00005845	-0.0987297	-0.456228	-0.0957359	BGIOSGA007666	XP_009123854.1 protein phosphatase 2C and cyclic nucleotide-binding/kinase domain-containing protein
TCONS_00017092	-0.0986583	-2.16648	0.31271	BGIOSGA020063	XP_003566126.1cytochrome P450 714D1
TCONS_00005428	-0.0986175	0.268133	-0.555314	BGIOSGA007537	XP_015689253.1 centrosome-associated protein CEP250
TCONS_00009786	-0.0985858	-0.00563373	-0.655686	BGIOSGA012338	XP_006649857.1 trifunctional UDP-glucose 4,6-dehydratase/UDP-4-keto-6-deoxy-D-glucose 3,5-epimerase/UDP-4-keto-L-rhamnose-reductase RHM1-like
TCONS_00015046	-0.0985145	-0.21931	0.0495185	BGIOSGA006757	XP_006652903.1 probable cyclic nucleotide-gated ion channel 5
TCONS_00000139	-0.0984093	0.167459	-0.148526	BGIOSGA002713	XP_006643688.1 plastidic glucose transporter 4
TCONS_00022754	-0.0980271	0.103661	-0.322719	BGIOSGA004651	XP_015694080.1 tubulin beta-3 chain isoform X2
TCONS_00022828	-0.0979247	0.522329	-0.228866	BGIOSGA007551	XP_015694133.1 adagio-like protein 1
TCONS_00030817	-0.0978884	0.247682	0.308103	BGIOSGA004748	XP_006661663.1 V-type proton ATPase subunit a3-like

TCONS_00031470	-0.0978401	-0.62739	-0.483576	BGIOSGA011706	XP_006661963.1 molybdate-anion transporter
TCONS_00006361	-0.097723	-0.596759	-0.877153	BGIOSGA030090	XP_006647439.1 probable serine/threonine-protein kinase DDB_G0276461
TCONS_00036891	-0.0976765	-2.16409	-0.97669	BGIOSGA013813	XP_015698545.1 probable calcium-binding protein CML28
TCONS_00005652	-0.0976166	1.63535	1.80638	BGIOSGA005456	XP_006647062.1 probable ADP-ribosylation factor GTPase-activating protein AGD14 isoform X1
TCONS_00029604	-0.0975484	-1.90484	-0.622968	BGIOSGA036071	XP_006655836.1 DNA-directed RNA polymerase I subunit rpa43-like isoform X1
TCONS_00024578	-0.0973105	0.751638	0.544429	BGIOSGA009696	XP_006657449.2PREDICTED: uncharacterized protein LOC102708350
TCONS_00014872	-0.0972593	-0.885765	0.590978	BGIOSGA008874	XP_015691685.1 cell division cycle 20.2, cofactor of APC complex-like
TCONS_00004921	-0.0972171	0.549573	0.358043	BGIOSGA034421	XP_006645191.1 fructose-bisphosphate aldolase cytoplasmic isozyme-like
TCONS_00034652	-0.0972001	1.02133	1.09157	BGIOSGA003234	XP_006662909.1 RRP15-like protein isoform X2
TCONS_00029503	-0.0971618	-0.658695	-0.527998	BGIOSGA031257	XP_015696703.1 organic cation/carnitine transporter 7-like isoform X2
TCONS_00018443	-0.0970681	1.07058	-0.281204	BGIOSGA002542	XP_014754694.1paired amphipathic helix protein Sin3-like 4
TCONS_00018475	-0.0969359	0.756902	0.932407	BGIOSGA004186	NP_001150630.1pseudouridylate synthase/ transporter
TCONS_00014451	-0.0969031	0.311506	0.237393	BGIOSGA008120	XP_006653525.1 eukaryotic translation initiation factor 4B1-like
TCONS_00031355	-0.0966878	0.757032	0.773424	BGIOSGA033168	XP_015697418.1 probable prefoldin subunit 5
TCONS_00036080	-0.0966413	-0.453172	0.224452	BGIOSGA017454	XP_002442191.1eukaryotic translation initiation factor 5B
TCONS_00028220	-0.0966287	1.03236	-0.789433	BGIOSGA015096	XP_003574679.1UDP-glycosyltransferase 89B1
TCONS_00016568	-0.0966028	-0.270511	-1.04972	BGIOSGA013123	XP_006652779.1 calcium-transporting ATPase 8, plasma membrane-type-like isoform X1
TCONS_00001667	-0.0965819	-0.557857	0.131205	BGIOSGA004302	XP_006644590.1 probable magnesium transporter NIPA9
TCONS_00001661	-0.0963628	0.458004	0.366	BGIOSGA027568	XP_003569648.1uncharacterized protein LOC100838789
TCONS_00002966	-0.0963414	0.13545	0.0817519	BGIOSGA028858	XP_006645569.1 FRIGIDA-like protein 4a
TCONS_00035875	-0.0962315	-0.508074	-0.166149	BGIOSGA037249	XP_015698748.1 probable Ufm1-specific protease
TCONS_00009418	-0.0956491	1.26395	0.218064	BGIOSGA011972	XP_006649494.1 elongation factor 1-alpha
TCONS_00014446	-0.0956144	-1.03067	-2.02198	BGIOSGA004078	XP_015691861.1 BTB/POZ domain-containing protein At1g67900-like
TCONS_00014790	-0.0955121	1.06559	-0.110681	BGIOSGA037276	XP_002446969.1guard cell S-type anion channel SLAC1
TCONS_00013048	-0.0954559	0.161684	0.0171315	BGIOSGA009938	XP_006651740.1 phosphatidylinositol 4-kinase alpha 1
TCONS_00002336	-0.0953001	0.517709	0.109071	BGIOSGA004977	XP_006645165.1 60S ribosomal protein L5-1
TCONS_00015242	-0.0952409	-0.428356	0.145947	BGIOSGA015738	XP_006652062.1 rhomboid-like protein 19
TCONS_00009738	-0.0950578	-0.385452	0.626956	BGIOSGA012287	XP_002468129.2uncharacterized protein LOC8054288
TCONS_00032196	-0.0950578	0.936477	5.11524	BGIOSGA015061	XP_008776735.1 BURP domain-containing protein 12-like
TCONS_00004179	-0.0950472	-0.632545	0.608569	BGIOSGA007472	XP_015689583.1 O-acyltransferase WSD1-like
TCONS_00009508	-0.0948548	0.210709	-0.368797	BGIOSGA006680	XP_006663973.1 40S ribosomal protein S3a
TCONS_00036249	-0.0948332	-1.95412	-1.75734	BGIOSGA018709	XP_021301504.1probable boron transporter 2
TCONS_00005217	-0.0944595	-0.296713	-0.219272	BGIOSGA007329	XP_006648202.1 26S proteasome non-ATPase regulatory subunit 10-like
TCONS_00025940	-0.0943437	-0.625525	1.21694	BGIOSGA014578	XP_006659130.1 putative glycerol-3-phosphate transporter 1
TCONS_00015763	-0.0941489	0.796935	0.464508	BGIOSGA016236	XP_006652203.1 stearoyl-[acyl-carrier-protein] 9-desaturase 5, chloroplastic
TCONS_00004784	-0.0938963	1.80135	1.18992	BGIOSGA026957	XP_002458823.1G-box-binding factor 4
TCONS_00009751	-0.0937826	1.55177	0.261137	BGIOSGA028786	XP_008654057.1serine/threonine-protein phosphatase PP1 isoform X1
TCONS_00030444	-0.0937731	-0.881571	-1.20464	BGIOSGA028564	XP_006660908.1 SKP1-like protein 1B
TCONS_00032080	-0.093695	-0.92338	-1.10824	BGIOSGA028967	XP_015696973.1 probable protein phosphatase 2C 71
TCONS_00017173	-0.0934286	-0.637034	-0.092684	BGIOSGA027666	XP_015696101.1PREDICTED: uncharacterized protein LOC102719510
TCONS_00013164	-0.0932567	-1.6967	-1.2337	BGIOSGA032319	XP_004981799.1uncharacterized protein LOC101777146
TCONS_00017570	-0.0932517	-2.35733	-2.24428	BGIOSGA017870	XP_006654265.1 CBL-interacting protein kinase 18
TCONS_00025616	-0.0930852	0.0434123	0.141473	BGIOSGA023819	XP_006658892.1 transcription-associated protein 1-like
TCONS_00024302	-0.0930557	-0.386994	-0.943018	BGIOSGA020348	XP_024318711.1tubby-like F-box protein 6
TCONS_00006572	-0.0930387	0.540118	1.20647	BGIOSGA033883	XP_006648897.1PREDICTED: uncharacterized protein LOC102705603 isoform X1
TCONS_00009101	-0.0928959	0.419566	0.621459	BGIOSGA017183	XP_015689007.1 probable phospholipase A2 homolog 1
TCONS_00032371	-0.0926948	0.72178	1.13903	BGIOSGA031756	XP_006650259.2 psbP-like protein 1, chloroplastic isoform X2
TCONS_00000971	-0.092594	-0.40045	-0.343605	BGIOSGA017112	XP_006644189.1 LEC14B protein-like
TCONS_00004055	-0.0923496	0.742031	-0.809959	BGIOSGA026933	XP_015689374.1 CASP-like protein 4U1
TCONS_00004330	-0.092336	0.348569	0.257471	#N/A	#N/A
TCONS_00002346	-0.0923203	0.895907	0.0475246	BGIOSGA014827	XP_006645176.1 CMP-sialic acid transporter 3
TCONS_00020194	-0.0922875	0.559839	0.221457	BGIOSGA021342	XP_004964738.1proteasome subunit alpha type-4-2
TCONS_00011390	-0.0922778	0.245666	0.230424	BGIOSGA025008	XP_006661016.2PREDICTED: uncharacterized protein LOC102712932
TCONS_00006890	-0.0920242	0.256948	0.581731	BGIOSGA016882	XP_004953948.1ethylene-responsive transcription factor RAP2-4
TCONS_00025918	-0.0917526	-0.243893	-0.144692	BGIOSGA027993	XP_006659114.2 monodehydroascorbate reductase 5, mitochondrial
TCONS_00000328	-0.0917201	-0.380027	0.833562	BGIOSGA002904	XP_006643845.1 F-box protein At2g05970-like

TCONS_00028550	-0.0916708	0.56417	1.23287	BGIOSGA033364	XP_021312951.1uncharacterized protein LOC110433976
TCONS_00001172	-0.0915391	0.610739	0.386704	BGIOSGA020890	NP_001150382.1LIN1 protein
TCONS_00007305	-0.0915387	0.43728	0.230559	BGIOSGA007176	XP_015689396.1 structural maintenance of chromosomes protein 3
TCONS_00014873	-0.0913375	-0.59903	0.0778773	BGIOSGA004425	XP_006652753.1 clathrin interactor EPSIN 1-like
TCONS_00008526	-0.0912356	-0.185053	0.0118954	BGIOSGA000345	XP_006647633.1PREDICTED: uncharacterized protein LOC102719928 isoform X1
TCONS_00029548	-0.0912203	-1.99175	-1.05999	BGIOSGA031463	NP_001136855.1Retinol dehydrogenase 14
TCONS_00026669	-0.0908238	-0.539265	0.556275	BGIOSGA028735	XP_015695619.1 DCN1-like protein 4
TCONS_00021779	-0.0906573	0.21223	-0.342952	BGIOSGA009202	XP_006655875.1 transcription factor SPATULA-like isoform X1
TCONS_00012676	-0.0904691	1.09316	-0.824576	BGIOSGA016924	XP_002446942.2putative F-box protein At4g22170
TCONS_00029320	-0.0904504	-0.0579022	-0.42315	BGIOSGA003415	XP_004957303.1NAC domain-containing protein 41
TCONS_00027131	-0.0902705	-0.590392	-0.48174	BGIOSGA029173	XP_002445704.1NADH dehydrogenase [ubiquinone] iron-sulfur protein 5-B
TCONS_00028638	-0.0901576	0.423166	0.944869	BGIOSGA030393	XP_006660473.2 GBF-interacting protein 1-like isoform X1
TCONS_00032759	-0.0901078	-0.344697	-0.57172	BGIOSGA031353	XP_006662097.1 mitochondrial carnitine/acylcarnitine carrier-like protein
TCONS_00000919	-0.0900972	0.483615	0.766373	BGIOSGA003510	XP_006645888.2PREDICTED: uncharacterized protein LOC102701879
TCONS_00021189	-0.0900293	-0.712474	-0.570065	BGIOSGA023407	XP_006657211.2 probable homogentisate phytyltransferase 1, chloroplastic
TCONS_00016091	-0.089985	-0.0718338	0.228665	BGIOSGA025001	XP_003579963.12-oxoglutarate-dependent dioxygenase DAO
TCONS_00030520	-0.0899748	0.653588	0.658456	BGIOSGA029287	XP_006661527.1 cysteine--tRNA ligase, chloroplastic/mitochondrial
TCONS_00032820	-0.0897746	-0.379361	0.235626	BGIOSGA036859	XP_021301782.1protein DETOXIFICATION 44, chloroplastic isoform X3
TCONS_00015275	-0.0893953	-0.48339	-0.686912	BGIOSGA015708	XP_015691813.1 transcription factor E2FB-like
TCONS_00029471	-0.0892273	0.628989	0.337505	BGIOSGA029297	XP_015696862.1 25S rRNA (cytosine-C(5))-methyltransferase nop2 isoform X2
TCONS_00010427	-0.0891135	-1.59355	-1.30217	BGIOSGA013023	XP_006650305.1 peroxisomal membrane protein 2-like
TCONS_00027122	-0.0889066	-1.08394	-0.464922	BGIOSGA037087	XP_006659678.1 transmembrane 9 superfamily member 9-like
TCONS_00002713	-0.0888915	-1.26341	-0.415103	BGIOSGA002518	XP_015699285.1 probable receptor-like protein kinase At1g67000
TCONS_00008796	-0.0888257	0.72319	-0.558788	BGIOSGA027247	XP_002452564.1uncharacterized protein LOC8075719
TCONS_00022382	-0.0887929	0.427197	-0.825254	BGIOSGA036287	XP_006657037.1 CBL-interacting protein kinase 25-like
TCONS_00023045	-0.0887602	0.705642	0.361464	BGIOSGA007419	XP_006659404.1 eukaryotic translation initiation factor 3 subunit C-like
TCONS_00007509	-0.0887321	-0.389801	0.801948	BGIOSGA022688	XP_006647004.1 sodium-coupled neutral amino acid transporter 1-like
TCONS_00037526	-0.0886887	0.471079	0.286319	BGIOSGA022468	XP_015698719.1 auxin response factor 25
TCONS_00019231	-0.0886819	-1.00202	-0.587067	BGIOSGA026305	XP_003568536.1probable glycosyltransferase STELLO2
TCONS_00027311	-0.0885616	-0.376662	-0.494447	BGIOSGA024146	XP_006659812.1 transcription factor ILR3-like
TCONS_00009076	-0.0885385	0.848614	0.488591	BGIOSGA033230	XP_004954465.1uncharacterized protein LOC101774373
TCONS_00000670	-0.0884062	-0.584896	-0.431601	BGIOSGA003257	XP_006645769.1 50S ribosomal protein 5, chloroplastic
TCONS_00008633	-0.0883156	-2.06526	-1.9619	BGIOSGA001376	XP_006648955.1 pentatricopeptide repeat-containing protein At1g31920
TCONS_00031070	-0.0880125	0.405665	1.0112	BGIOSGA032889	XP_002467267.1protein HGH1 homolog
TCONS_00012955	-0.0878523	-2.05905	-1.26422	BGIOSGA010034	XP_006650416.1PREDICTED: uncharacterized protein LOC102714440
TCONS_00035766	-0.0877511	-2.4564	-2.34184	BGIOSGA038223	XP_006664438.1 2-alkenal reductase (NADP(+)-dependent)-like
TCONS_00031411	-0.087747	-0.177748	0.927652	BGIOSGA033224	XP_015697143.1 pentatricopeptide repeat-containing protein At2g20710, mitochondrial-like
TCONS_00035326	-0.0876734	0.491157	0.479773	BGIOSGA026370	XP_006663096.2PREDICTED: uncharacterized protein LOC102715982
TCONS_00023253	-0.0876624	0.304202	0.717772	BGIOSGA013742	XP_006658347.1 SEC12-like protein 1 isoform X1
TCONS_00022198	-0.0876043	-0.0245879	-0.167341	BGIOSGA005467	XP_008668705.1signal recognition particle 19 kDa protein
TCONS_00014190	-0.0872469	-0.626108	-0.694129	BGIOSGA010861	XP_015691999.1 neutral/alkaline invertase 3, chloroplastic-like
TCONS_00027142	-0.0871952	-0.470461	-0.293408	BGIOSGA004839	XP_021320832.160S ribosomal protein L10a isoform X1
TCONS_00028957	-0.0871749	-0.335282	0.0808904	BGIOSGA030713	XP_015696886.1PREDICTED: uncharacterized protein LOC107305020 isoform X4
TCONS_00017798	-0.0870158	0.218892	0.521241	BGIOSGA025694	XP_006654398.2 cysteine proteinase inhibitor 3-like
TCONS_00011505	-0.0869645	0.192746	-0.611491	BGIOSGA028357	XP_006651009.2 protein NLP1-like
TCONS_00016933	-0.0866168	-0.90307	-0.145323	BGIOSGA014031	XP_006653082.1 tubulin-folding cofactor B
TCONS_00011164	-0.0866095	-0.753791	-0.270075	BGIOSGA030649	XP_006651935.1 protochlorophyllide-dependent translocon component 52, chloroplastic-like
TCONS_00033822	-0.0865727	-1.82797	-0.43084	BGIOSGA035579	XP_006663039.1 putative disease resistance RPP13-like protein 3 isoform X1
TCONS_00031432	-0.0865652	0.0717734	-0.267443	BGIOSGA033247	XP_021306494.1leukocyte receptor cluster member 1 homolog
TCONS_00005049	-0.0865252	-0.193942	-0.202658	BGIOSGA000167	XP_006645293.2 spermatogenesis-associated protein 20 isoform X1
TCONS_00009870	-0.0864447	0.0747248	0.31154	BGIOSGA018246	XP_006649933.1 40S ribosomal protein S7
TCONS_00009197	-0.0863411	-0.480786	0.322643	BGIOSGA007417	XP_003558962.1cyclin-dependent kinase A-1
TCONS_00030103	-0.0862894	0.320551	1.40153	BGIOSGA029699	XP_006660652.1 E3 ubiquitin-protein ligase AIP2

TCONS_00004934	-0.0862672	-0.335779	-0.521514	BGIOSGA036314	XP_006645200.1 endoribonuclease Dicer homolog 3a isoform X5
TCONS_00022121	-0.0859598	0.729809	0.728886	BGIOSGA021384	XP_006656910.2 ERAD-associated E3 ubiquitin-protein ligase HRD1
TCONS_00015970	-0.085929	-0.141884	-0.0186457	BGIOSGA015008	XP_006652334.1 probable signal peptidase complex subunit 2
TCONS_00020690	-0.0856102	-0.0751347	-0.626067	BGIOSGA022884	XP_006661782.1 pheophytinase, chloroplastic
TCONS_00014816	-0.0854793	-0.794677	-1.75549	BGIOSGA016988	XP_006652694.1 pentatricopeptide repeat-containing protein At5g13770, chloroplastic
TCONS_00033629	-0.0854506	0.709221	0.611536	BGIOSGA006690	XP_006662991.1 ABC transporter E family member 2
TCONS_00026114	-0.0853829	-0.510738	-0.444109	BGIOSGA014361	XP_006659213.1 UDP-N-acetylglucosamine diphosphorylase 1-like
TCONS_00027721	-0.0853694	-1.07438	-1.04399	BGIOSGA027282	XP_003573659.2F-box protein At5g03100 isoform X2
TCONS_00023951	-0.0853568	-1.08325	-0.3459	BGIOSGA005858	XP_006657871.1 probable serine/threonine-protein kinase WNK1
TCONS_00000573	-0.0851224	1.00295	0.844018	BGIOSGA000971	XP_003566368.250S ribosomal protein L3-2, chloroplastic
TCONS_00020323	-0.0850082	0.0423884	0.0761275	BGIOSGA022515	XP_006656748.2 pantothenate kinase 1
TCONS_00012846	-0.0848536	-1.50645	0.0603867	BGIOSGA016232	XP_015691089.1 chlorophyll(ide) b reductase NOL, chloroplastic isoform X2
TCONS_00037589	-0.084639	-1.45848	-1.60922	BGIOSGA035852	XP_015698584.1PREDICTED: uncharacterized protein LOC102711115
TCONS_00016248	-0.084619	0.348841	-0.14718	BGIOSGA007591	XP_002448165.2putative disease resistance RPP13-like protein 1
TCONS_00005459	-0.0846034	0.556771	-0.89879	BGIOSGA032160	XP_006648324.2 thiosulfate sulfurtransferase 16, chloroplastic-like
TCONS_00034825	-0.0844769	#NA	-0.984853	BGIOSGA034019	XP_003560487.1anthocyanidin 3-O-glucosyltransferase
TCONS_00018574	-0.084442	0.478169	0.652118	BGIOSGA018860	XP_006654013.1 putative RNA polymerase II subunit B1 CTD phosphatase RPAP2 homolog
TCONS_00029678	-0.0843796	-1.45658	-0.769754	BGIOSGA031939	XP_021318578.1phospholipid-transporting ATPase 2 isoform X6
TCONS_00010749	-0.0841576	-0.0609312	0.458089	BGIOSGA033241	XP_006650434.1 valine--tRNA ligase, mitochondrial 1-like
TCONS_00033045	-0.0841389	-0.0480817	-0.125079	BGIOSGA015098	XP_006662764.1 RNA-binding protein FUS-like
TCONS_00016227	-0.0838913	-0.526809	0.400428	BGIOSGA014747	XP_006653589.2 general transcription factor IIH subunit 2
TCONS_00013050	-0.0838186	-0.186918	-0.112631	BGIOSGA009936	XP_006650487.1 coatomer subunit alpha-3 isoform X1
TCONS_00006669	-0.0837751	0.388097	0.623613	BGIOSGA017003	XP_006662363.1 probable purine permease 11
TCONS_00026111	-0.0837446	0.0726575	0.833155	BGIOSGA027463	XP_006659930.1 bifunctional purine biosynthesis protein PurH isoform X1
TCONS_00006144	-0.0837271	-1.14565	1.50713	BGIOSGA025074	XP_003575031.1glycerophosphodiester phosphodiesterase GDPD1, chloroplastic
TCONS_00009687	-0.083642	-0.257794	-0.394549	BGIOSGA012237	XP_006649744.1 peptidyl-tRNA hydrolase ICT1, mitochondrial
TCONS_00023365	-0.0835084	0.0120549	0.120396	BGIOSGA025407	XP_004955822.160S ribosomal protein L24
TCONS_00036903	-0.0834209	0.431394	1.89547	BGIOSGA033341	XP_006645389.1 probable glutathione S-transferase
TCONS_00009327	-0.0834133	-0.194681	-0.167173	BGIOSGA036374	XP_006651047.2 lysine-specific demethylase SE14-like
TCONS_00003440	-0.0834115	-0.0580045	-0.211956	BGIOSGA001785	XP_006645828.1 protein translocase subunit SECA1, chloroplastic
TCONS_00012782	-0.0833888	-3.44332	-1.1082	BGIOSGA035878	XP_015690817.1 protein NETWORKED 1A-like
TCONS_00003287	-0.0833811	0.182914	-0.145797	BGIOSGA036677	XP_015688142.1 probable peptide/nitrate transporter At3g43790
TCONS_00011936	-0.0833635	-3.9837	-1.98766	BGIOSGA011075	XP_004984959.1IST1-like protein
TCONS_00008749	-0.0831611	0.263978	0.23422	BGIOSGA005683	XP_006647841.1 rRNA-processing protein fcf2-like
TCONS_00016875	-0.0831463	0.115543	-0.414457	BGIOSGA001898	XP_006653027.1 pyruvate kinase, cytosolic isozyme
TCONS_00010556	-0.0830586	-0.241985	1.17551	BGIOSGA010207	XP_006650319.1 iron-sulfur protein NUBPL
TCONS_00018449	-0.0830401	-0.0819463	-0.221454	BGIOSGA008606	XP_006653930.1 50S ribosomal protein L28, chloroplastic
TCONS_00015767	-0.0829724	-0.900532	0.128469	BGIOSGA000233	XP_022684244.1dynamin-related protein 3A
TCONS_00022964	-0.0829522	-0.89461	-1.31088	BGIOSGA023470	XP_015695189.1 glutamate receptor 3.5-like
TCONS_00021546	-0.0828596	0.597082	0.01763	BGIOSGA000532	XP_004964437.1protein GPR107
TCONS_00024751	-0.0828184	-3.35704	-1.69694	BGIOSGA027158	XP_006657551.1 probable cellulose synthase A catalytic subunit 8 [UDP-forming]
TCONS_00013911	-0.0827869	-3.02096	-1.59787	BGIOSGA033693	XP_006652156.1 short-chain dehydrogenase TIC 32, chloroplastic-like
TCONS_00006030	-0.0827414	-0.493518	0.354903	BGIOSGA008182	XP_015688909.1PREDICTED: uncharacterized protein LOC107303569
TCONS_00026530	-0.0827324	0.29716	0.458032	BGIOSGA003034	XP_006660101.2 mitochondrial carrier protein MTM1-like
TCONS_00026636	-0.0825948	0.554608	1.37295	BGIOSGA033940	XP_004487626.1 probable histone H2A.3
TCONS_00028030	-0.0825877	-0.230031	0.219684	BGIOSGA021161	XP_006660138.1 methylcrotonoyl-CoA carboxylase beta chain, mitochondrial
TCONS_00024417	-0.0824096	1.82248	-0.990066	BGIOSGA038098	XP_021308816.1developmental and secondary metabolism regulator VEL1
TCONS_00019741	-0.0823607	0.188899	-0.48072	BGIOSGA038127	XP_015692688.1 probable receptor-like protein kinase At1g67000
TCONS_00037471	-0.0822441	-0.321755	0.508462	BGIOSGA009840	XP_006664711.1 E3 ubiquitin protein ligase DRIP1-like
TCONS_00025051	-0.0822351	-3.62253	-1.73897	BGIOSGA035476	XP_004956141.1uncharacterized protein LOC101768197
TCONS_00001229	-0.0822205	0.233599	-0.0216013	BGIOSGA017146	XP_006644316.1 serine/threonine-protein phosphatase 6 regulatory subunit 3-like isoform X2
TCONS_00028739	-0.0821003	0.523557	-0.258968	BGIOSGA030492	XP_006661107.1 putative disease resistance protein RGA3
TCONS_00011155	-0.0820656	-1.17795	-0.575895	BGIOSGA027058	XP_006650751.1PREDICTED: uncharacterized protein LOC102717971

TCONS_00005554	-0.081932	0.110383	0.361594	BGIOSGA007663	XP_006646967.1 ubiquitin receptor RAD23b isoform X2
TCONS_00008109	-0.081744	0.817562	0.673976	BGIOSGA019930	XP_006647332.1 probable glycosyltransferase 2
TCONS_00019436	-0.0817404	1.44822	0.317793	BGIOSGA004774	XP_003568309.1transcription factor MYB3R-2 isoform X2
TCONS_00033983	-0.0816885	1.07418	-0.893839	BGIOSGA033577	XP_015698951.1 calmodulin-binding protein 60 D-like
TCONS_00032120	-0.0815848	0.71453	0.592818	BGIOSGA032015	XP_015697159.1 eukaryotic initiation factor 4A-I
TCONS_00027785	-0.0813587	0.714246	0.0545916	BGIOSGA004740	XP_003573688.1protein RER1A
TCONS_00005236	-0.0812628	-0.3083	-0.0967151	BGIOSGA027652	XP_006648213.1 transmembrane protein 120 homolog
TCONS_00009739	-0.0811375	-0.602204	0.195532	BGIOSGA012291	XP_006649806.2 OTU domain-containing protein At3g57810 isoform X3
TCONS_00029896	-0.0808646	3.45613	1.42108	BGIOSGA029909	XP_006660562.1 probable LRR receptor-like serine/threonine-protein kinase At1g51820
TCONS_00034944	-0.0808593	0.829918	0.745112	BGIOSGA004060	XP_006662962.1 RNA polymerase II C-terminal domain phosphatase-like 3
TCONS_00010654	-0.0808449	-0.509351	-0.88888	BGIOSGA013283	XP_015690883.1 protein ROOT PRIMORDIUM DEFECTIVE 1
TCONS_00005553	-0.0805379	0.488506	0.129917	BGIOSGA004087	XP_006646965.1 HD domain-containing protein 2-like
TCONS_00013049	-0.0803753	0.338455	0.620854	BGIOSGA009938	XP_006650486.1 serine/threonine-protein kinase GRIK1-like
TCONS_00018677	-0.0803114	0.69055	0.895267	BGIOSGA018754	XP_004964745.3pre-mRNA-processing-splicing factor 8A
TCONS_00016735	-0.0800542	1.26009	-0.259026	BGIOSGA014229	NP_001340287.1ubiquitin carboxyl-terminal hydrolase 22
TCONS_00026277	-0.0800542	-1.18048	-0.259026	BGIOSGA028358	XP_015695817.1PREDICTED: uncharacterized protein LOC102707514
TCONS_00019189	-0.0799995	-0.91935	-0.345821	BGIOSGA014792	XP_006652477.1 protein CHROMOSOME TRANSMISSION FIDELITY 7
TCONS_00030964	-0.0799704	-0.605795	-0.146701	BGIOSGA032774	XP_015697333.1 F-box/kelch-repeat protein At1g22040-like
TCONS_00010292	-0.0799509	-0.509997	0.673211	BGIOSGA016721	XP_006650192.1PREDICTED: uncharacterized protein LOC102720778
TCONS_00024801	-0.0796142	0.104714	-0.488947	BGIOSGA021524	XP_006657578.1 PHD finger protein ALFIN-LIKE 2
TCONS_00027944	-0.0795676	0.315996	0.464616	BGIOSGA027052	XP_015696045.1PREDICTED: uncharacterized protein LOC102704273 isoform X1
TCONS_00030962	-0.079468	-1.35465	-1.29805	BGIOSGA018366	XP_015697314.1 acetyl-CoA carboxylase 1
TCONS_00034772	-0.0794071	-1.31488	-0.167549	BGIOSGA015987	XP_006663388.2 serine carboxypeptidase-like 18
TCONS_00025995	-0.0792608	-0.638346	0.117499	BGIOSGA000764	XP_004970074.1clp protease adapter protein ClpF, chloroplastic
TCONS_00004926	-0.0792261	0.0638712	0.420532	BGIOSGA000294	XP_006646582.1 UDP-N-acetylglucosamine--peptide N-acetylglucosaminyltransferase
TCONS_00006876	-0.0792241	-0.16409	0.470579	BGIOSGA021620	XP_015689585.1PREDICTED: uncharacterized protein LOC102705413
TCONS_00002489	-0.0791466	-0.60064	0.187587	BGIOSGA004267	XP_006645304.1 nascent polypeptide-associated complex subunit alpha-like protein 1
TCONS_00018676	-0.0789335	0.375315	-0.620285	BGIOSGA018756	XP_006654082.1 PHD finger protein ALFIN-LIKE 1
TCONS_00025956	-0.0787141	-1.18218	-1.26238	BGIOSGA030280	XP_003578830.1uncharacterized protein LOC100822076
TCONS_00004616	-0.0786295	-0.115173	-0.584519	BGIOSGA020083	XP_002458673.1uncharacterized protein At5g19025
TCONS_00010928	-0.0786295	1.82414	0.392761	BGIOSGA033114	XP_002466479.1PRA1 family protein B6
TCONS_00019370	-0.0786045	0.285198	-0.555574	BGIOSGA004862	XP_006654464.1 actin-1
TCONS_00000274	-0.0784253	-0.326936	0.0779363	BGIOSGA004592	XP_006643783.1 protein synthesis inhibitor II-like
TCONS_00028093	-0.0782572	0.870661	-0.607624	BGIOSGA010648	XP_003564855.14-coumarate--CoA ligase-like 5
TCONS_00028650	-0.0779479	-0.327234	-0.0759871	BGIOSGA030402	XP_006660475.1PREDICTED: uncharacterized protein LOC102716063 isoform X1
TCONS_00017133	-0.0779153	0.750642	-0.250121	BGIOSGA024257	XP_006654993.1 actin cytoskeleton-regulatory complex protein pan1-like
TCONS_00023072	-0.0778546	-0.547085	-0.192276	BGIOSGA009739	XP_006657431.1 probable LRR receptor-like serine/threonine-protein kinase At2g16250
TCONS_00007238	-0.0778106	-0.541052	-0.220323	BGIOSGA019080	XP_006659053.1 transcription factor IIIA-like
TCONS_00001710	-0.0776351	-0.531468	-2.00204	BGIOSGA004344	XP_006644630.1PREDICTED: uncharacterized protein LOC102715525
TCONS_00026811	-0.0775078	1.1519	-0.471839	BGIOSGA028871	XP_002445983.1uncharacterized protein LOC8067040
TCONS_00005693	-0.0773973	-0.08869	-0.00744348	BGIOSGA030511	XP_006647079.1 probable protein phosphatase 2C 12
TCONS_00019813	-0.0771969	-0.320832	-0.0637642	BGIOSGA031408	XP_004961181.150S ribosomal protein L12, chloroplastic
TCONS_00007219	-0.0771959	0.560404	-0.551294	BGIOSGA031105	XP_003571751.1protein KINESIN LIGHT CHAIN-RELATED 1
TCONS_00004776	-0.0771798	0.356449	0.652875	BGIOSGA000438	XP_006645071.1 uricase-2
TCONS_00032084	-0.076966	-0.233337	-1.51083	BGIOSGA024994	XP_006661601.1 protein NRT1/ PTR FAMILY 8.3-like
TCONS_00032765	-0.0768816	0.822952	0.085923	BGIOSGA031345	XP_004983558.1dnaJ homolog subfamily C GRV2
TCONS_00008407	-0.0767391	0.0937854	-0.018082	BGIOSGA035967	XP_003580128.160S ribosomal protein L14-1
TCONS_00020501	-0.076499	-0.646085	0.264437	BGIOSGA005685	XP_006656871.1 aldehyde dehydrogenase family 2 member B7, mitochondrial-like
TCONS_00030410	-0.0764599	-1.31449	0.235231	BGIOSGA029391	XP_007020909.1 vacuolar protein sorting-associated protein 22 homolog 1 isoform X3
TCONS_00012832	-0.0763772	-0.670559	0.869303	BGIOSGA010138	XP_006650355.2 probable E3 ubiquitin-protein ligase BAH1-like 1 isoform X1
TCONS_00019895	-0.0763551	-0.14924	-0.373866	BGIOSGA006173	XP_021316256.1uncharacterized protein LOC8055410
TCONS_00014227	-0.0762277	-3.35034	-2.61451	BGIOSGA008374	XP_015691448.1 2-Cys peroxiredoxin BAS1, chloroplastic
TCONS_00008750	-0.0757155	-0.270015	0.249664	BGIOSGA021478	XP_015689245.1 aspartic proteinase Asp1-like

TCONS_00024624	-0.0757067	-0.143833	-0.814878	BGIOSGA024760	XP_006657475.1PREDICTED: uncharacterized protein LOC102715316 isoform X2
TCONS_00023661	-0.0756763	0.273419	-0.479081	BGIOSGA013351	XP_006657691.1 arginine/serine-rich protein PNISR isoform X2
TCONS_00008789	-0.0753474	0.883589	0.622993	BGIOSGA005647	XP_006649036.1 nuclear-pore anchor
TCONS_00016865	-0.0752996	1.28571	0.83954	BGIOSGA010992	XP_006653023.1 dolichyl-diphosphooligosaccharide--protein glycosyltransferase subunit STT3B
TCONS_00018428	-0.0752684	0.271516	0.446363	BGIOSGA017146	XP_006654872.1 serine/threonine-protein phosphatase 6 regulatory subunit 3-like isoform X1
TCONS_00018204	-0.0751874	-1.65449	-0.4702	BGIOSGA018386	XP_006654709.1 ras-related protein Rab7-like
TCONS_00021686	-0.0750745	0.948017	1.33141	BGIOSGA018755	XP_021305127.1pre-mRNA-processing-splicing factor 8A
TCONS_00003630	-0.0746924	0.175317	0.286113	BGIOSGA007028	XP_002437838.1uncharacterized protein LOC8071317
TCONS_00020227	-0.074689	0.634171	0.784354	BGIOSGA022416	XP_003564221.1protein SRG1
TCONS_00026960	-0.0746696	-0.616125	-1.21585	BGIOSGA020942	XP_006659609.1PREDICTED: uncharacterized protein LOC102721010
TCONS_00035945	-0.0745857	0.434656	-0.166502	BGIOSGA009162	XP_006663997.2 E3 ubiquitin-protein ligase UPL2-like
TCONS_00005210	-0.0745473	0.336275	0.292438	BGIOSGA007322	XP_015689377.1PREDICTED: uncharacterized protein LOC102718059
TCONS_00020921	-0.0744889	0.416351	0.428851	BGIOSGA004246	XP_004966031.1serine/threonine-protein phosphatase PP2A-1 catalytic subunit
TCONS_00035621	-0.0744618	0.319225	0.277167	BGIOSGA034853	XP_006664361.1 transcription factor VIP1-like
TCONS_00015841	-0.0742718	#NA	-1.76396	BGIOSGA015144	XP_006652248.1PREDICTED: uncharacterized protein LOC102721263
TCONS_00033020	-0.0742061	-1.67329	-0.136076	BGIOSGA004354	XP_006662757.1 disease resistance protein RGA2-like
TCONS_00035796	-0.0741012	-0.175559	-0.18073	BGIOSGA037202	XP_006664454.2 fructose-bisphosphate aldolase-lysine N-methyltransferase, chloroplastic
TCONS_00005992	-0.0740728	-0.762847	-0.196382	BGIOSGA036886	XP_006664278.1 non-specific lipid-transfer protein 4
TCONS_00004629	-0.0740509	-1.41045	-0.75939	BGIOSGA006546	XP_006644912.1 synaptotagmin-2-like
TCONS_00005629	-0.0740181	0.289947	0.122846	BGIOSGA007506	XP_006648429.2 putative disease resistance RPP13-like protein 3
TCONS_00004845	-0.0739049	0.469646	0.109545	BGIOSGA014815	XP_015698070.1 NAC domain-containing protein 48-like
TCONS_00013012	-0.0737819	-0.362937	-0.0897319	BGIOSGA009976	XP_008665260.1elongation of fatty acids protein 3-like
TCONS_00015313	-0.0737267	-0.506436	-0.209669	BGIOSGA025570	XP_015692079.1 sterol 3-beta-glucosyltransferase UGT80A2-like isoform X1
TCONS_00024871	-0.0736793	-0.754872	-0.248885	BGIOSGA024508	XP_015694591.1PREDICTED: uncharacterized protein LOC102721466
TCONS_00030919	-0.0735229	-0.42156	0.0611879	BGIOSGA013553	XP_006648000.1 myosin-15-like
TCONS_00016200	-0.0733145	-0.323177	0.137336	BGIOSGA006053	XP_024311700.1leucine-rich repeat-containing G-protein coupled receptor 4 isoform X1
TCONS_00030506	-0.0731886	0.413615	0.306037	BGIOSGA029302	XP_006661518.1 nudix hydrolase 23, chloroplastic
TCONS_00008209	-0.0731097	-0.790835	-0.0346195	BGIOSGA015003	XP_006647400.1PREDICTED: uncharacterized protein LOC102700211
TCONS_00028326	-0.0728719	-0.457197	0.831187	#N/A	#N/A
TCONS_00031442	-0.0728485	-0.581868	-2.23249	BGIOSGA018965	XP_006661943.1 actin-2
TCONS_00011925	-0.0728182	-0.397726	-1.44711	BGIOSGA032183	XP_006649760.1 probable E3 ubiquitin-protein ligase LOG2
TCONS_00023430	-0.0726079	0.647528	-0.159871	BGIOSGA025462	XP_006657591.1PREDICTED: uncharacterized protein LOC102700911
TCONS_00016931	-0.0724551	1.02051	0.504511	BGIOSGA011679	XP_006653079.2 increased DNA methylation 1
TCONS_00018143	-0.0724499	-0.491894	0.0926524	BGIOSGA020201	XP_014754572.1alpha,alpha-trehalose-phosphate synthase [UDP-forming] 1
TCONS_00023793	-0.0724291	-1.04807	0.0419752	BGIOSGA021758	XP_006657772.2 glutamate receptor 3.4-like isoform X1
TCONS_00000265	-0.0723676	0.0638816	-0.146703	BGIOSGA007036	XP_008673678.2transcription initiation factor TFIID subunit 15b
TCONS_00012937	-0.0720507	-0.27413	-0.322779	BGIOSGA013510	XP_006650408.1 BEL1-like homeodomain protein 7
TCONS_00009744	-0.0720236	-1.22762	-0.35436	BGIOSGA019097	XP_015690888.1 LRR receptor-like serine/threonine-protein kinase FEI 1
TCONS_00013853	-0.0717957	1.54987	0.604393	BGIOSGA023484	XP_006652141.1 eukaryotic peptide chain release factor GTP-binding subunit ERF3A
TCONS_00035023	-0.0717794	0.640236	-0.174238	BGIOSGA020235	XP_002450905.1patatin-like phospholipase domain-containing protein 4
TCONS_00009856	-0.0716912	-0.4578	-1.1948	BGIOSGA012407	XP_015691164.1 6-phosphofructo-2-kinase/fructose-2,6-bisphosphatase-like isoform X1
TCONS_00007051	-0.0716327	0.142837	0.93029	BGIOSGA009233	XP_006649139.1 probable 18S rRNA (guanine-N(7))-methyltransferase
TCONS_00026016	-0.0716071	0.616094	0.192798	BGIOSGA028089	XP_006659179.1 BUD13 homolog
TCONS_00003465	-0.0715475	-0.240525	-0.280129	BGIOSGA001761	XP_008810079.1 protein GFS12
TCONS_00019533	-0.0715166	-0.12232	0.0472985	BGIOSGA017827	XP_006654565.1 E3 ubiquitin-protein ligase RING1-like
TCONS_00027356	-0.0712685	-1.05808	0.231468	BGIOSGA007347	XP_006659835.2 transmembrane protein 120 homolog
TCONS_00011784	-0.0711971	0.84288	0.612473	BGIOSGA011221	XP_006651166.1 DExH-box ATP-dependent RNA helicase DExH14
TCONS_00022726	-0.0711744	0.357919	0.680806	BGIOSGA020725	XP_024312552.1formin-like protein 3
TCONS_00025786	-0.0711216	0.915563	0.559724	BGIOSGA027848	XP_006649390.1 ribosome maturation protein SBDS
TCONS_00008485	-0.0706605	-0.219992	-0.199649	BGIOSGA031126	XP_015689428.1 kinesin-like protein NACK1
TCONS_00019416	-0.0706479	0.54548	2.05727	BGIOSGA017936	XP_006654495.1 protein GUCD1 isoform X1
TCONS_00028117	-0.0703977	-6.20946	-2.47988	BGIOSGA026872	XP_006659474.1PREDICTED: uncharacterized protein LOC102704354
TCONS_00005615	-0.0701967	0.574863	0.0747569	BGIOSGA022563	XP_006647021.2 zinc transporter ZIP12

TCONS_00002777	-0.070165	-0.685479	-0.159986	BGIOSGA002733	XP_015697568.1 probable serine/threonine-protein kinase At1g18390 isoform X2
TCONS_00002242	-0.0700291	-0.38124	-0.0182367	BGIOSGA004880	XP_004970721.1TLC domain-containing protein 2
TCONS_00036662	-0.069953	0.0496343	-0.867477	BGIOSGA023755	XP_021313359.1uncharacterized protein LOC8074102 isoform X2
TCONS_00007261	-0.069888	-0.529949	-0.695857	BGIOSGA007221	XP_006648254.1 protein DETOXIFICATION 46, chloroplastic
TCONS_00010772	-0.0697316	0.288665	0.584317	BGIOSGA007855	XP_006650447.1 suppressor of disruption of TFIIS-like
TCONS_00013130	-0.0696822	-0.703307	-0.834554	BGIOSGA009199	XP_006651788.1 glucose-1-phosphate adenylyltransferase large subunit
TCONS_00008617	-0.0696148	-1.43839	-0.0674854	BGIOSGA027900	XP_015689506.1 E3 ubiquitin-protein ligase Os04g0590900 isoform X1
TCONS_00034197	-0.0695739	-0.0790384	0.212155	BGIOSGA028217	XP_019078711.1 ethanolamine-phosphate cytidylyltransferase isoform X2
TCONS_00009573	-0.0693832	0.765344	0.606474	BGIOSGA012122	XP_006649640.1 probable glucan 1,3-alpha-glucosidase
TCONS_00031530	-0.0693076	-0.492751	-0.295827	BGIOSGA011545	XP_006662540.1 C-terminal binding protein AN-like
TCONS_00032436	-0.0692475	0.590472	0.377911	BGIOSGA031681	XP_006661868.1 double-stranded RNA-binding protein 6
TCONS_00027760	-0.0692264	0.770728	0.676575	BGIOSGA027239	XP_006659275.1 threonine--tRNA ligase, mitochondrial 1
TCONS_00028301	-0.0687669	1.07941	1.57867	BGIOSGA005135	XP_006659616.1 calcium-transporting ATPase 8, plasma membrane-type-like isoform X2
TCONS_00022806	-0.0687468	1.09528	0.180105	BGIOSGA021336	XP_006657272.1 T-complex protein 1 subunit eta
TCONS_00024733	-0.06871	1.1791	-0.151581	BGIOSGA027614	XP_015695375.1 DEAD-box ATP-dependent RNA helicase 52B
TCONS_00002573	-0.0685958	-0.211204	0.242061	BGIOSGA006140	XP_003565112.1DNA mismatch repair protein MLH1 isoform X2
TCONS_00025900	-0.0685886	-0.248537	0.296631	BGIOSGA030859	XP_006659106.1 beta-glucuronosyltransferase GlcAT14B-like
TCONS_00011824	-0.0685709	-1.9861	-0.0436657	BGIOSGA011186	XP_015693523.1 protein CREG2
TCONS_00006880	-0.0685293	0.127534	1.02338	BGIOSGA009059	XP_006647897.2 pentatricopeptide repeat-containing protein At4g39620, chloroplastic
TCONS_00009884	-0.0683227	-0.81706	-0.175359	BGIOSGA026261	XP_003558105.1probable galacturonosyltransferase-like 7
TCONS_00000758	-0.0682185	-0.184572	0.921029	BGIOSGA009511	NP_001182883.1putative lipase class 3 family protein precursor
TCONS_00020820	-0.0682002	-0.469516	0.134682	BGIOSGA023029	XP_015693724.1 spermine synthase-like
TCONS_00016651	-0.0681421	-0.44435	-0.429231	BGIOSGA014309	XP_006656106.1PREDICTED: uncharacterized protein LOC102702667 isoform X1
TCONS_00006810	-0.0679518	-1.33966	-0.928717	BGIOSGA009089	XP_006647845.1 protein CURVATURE THYLAKOID 1A, chloroplastic
TCONS_00006915	-0.067786	-0.238269	-0.493502	BGIOSGA012328	XP_006647952.1 cyclin-dependent kinase inhibitor 1-like
TCONS_00010044	-0.067777	0.700148	-0.0730941	BGIOSGA012596	XP_006651360.1 putative GTP diphosphokinase RSH1, chloroplastic
TCONS_00031958	-0.0677581	0.39637	0.200118	#N/A	#N/A
TCONS_00036893	-0.0676845	-0.462099	-0.278341	BGIOSGA034168	XP_006663917.1 chaperone protein ClpC2, chloroplastic
TCONS_00001602	-0.0676505	-0.203544	-0.472741	BGIOSGA004239	XP_015689623.1 protein ABHD17C-like
TCONS_00028371	-0.0676308	-0.921864	0.0282841	BGIOSGA026592	XP_010927726.1 COMM domain-containing protein 9
TCONS_00028107	-0.0675258	-0.113255	0.742137	BGIOSGA026884	XP_006659468.1 cyclin-dependent kinase C-3
TCONS_00017726	-0.0674987	-0.556588	-0.83884	BGIOSGA005202	XP_006655275.1 putative NAD kinase 3
TCONS_00015430	-0.0674823	-1.64762	-0.150078	BGIOSGA015342	XP_004961357.1jacalin-related lectin 19 isoform X2
TCONS_00018113	-0.0673488	-0.141374	-1.32095	BGIOSGA020169	XP_015692873.1 putative rRNA methylase YtqB
TCONS_00000010	-0.06733	0.227649	-0.386817	BGIOSGA006040	XP_024316020.1serine/arginine-rich splicing factor 11
TCONS_00011737	-0.0671704	0.568371	0.933728	BGIOSGA011270	XP_015690299.1 tetratricopeptide repeat protein 7B isoform X1
TCONS_00009347	-0.0670047	-0.59292	0.497454	BGIOSGA031850	XP_015689767.1 FH protein interacting protein FIP2-like
TCONS_00003042	-0.0667971	0.820557	0.265428	BGIOSGA031561	XP_006643882.2 aquaporin NIP1-2-like
TCONS_00004914	-0.0667718	-2.17787	-1.67815	BGIOSGA000307	NP_001140580.1Protein kinase superfamily protein
TCONS_00012565	-0.0667277	0.703189	1.23534	BGIOSGA016505	XP_004982540.1polyadenylate-binding protein RBP45
TCONS_00005642	-0.0666715	-1.07029	-0.773787	BGIOSGA020932	XP_006647055.1PREDICTED: uncharacterized protein LOC102712500 isoform X1
TCONS_00016847	-0.0665689	-0.670386	-0.961271	BGIOSGA007119	XP_015692160.1 phytosulfokine receptor 1-like
TCONS_00030374	-0.0664887	-0.952492	0.363921	BGIOSGA026550	XP_015696553.1PREDICTED: uncharacterized protein LOC102712469
TCONS_00018700	-0.0664841	0.392392	0.431428	BGIOSGA018729	XP_006654093.1PREDICTED: uncharacterized protein LOC102706471
TCONS_00006725	-0.0663892	-0.342544	-0.227106	BGIOSGA008895	XP_015689616.1 uridine-cytidine kinase C-like
TCONS_00011956	-0.0660245	0.588736	0.220398	BGIOSGA009526	XP_006651238.2 anthranilate synthase alpha subunit 2, chloroplastic
TCONS_00012952	-0.0658057	-0.106528	-0.0547824	BGIOSGA010037	XP_006650414.2 E3 ubiquitin-protein ligase UPL7
TCONS_00017332	-0.0656603	0.461491	0.522318	BGIOSGA010919	XP_006655099.1 abscisic acid receptor PYL8-like
TCONS_00000278	-0.0656558	0.228919	1.21198	BGIOSGA002855	XP_010230494.1RING finger and transmembrane domain-containing protein 2
TCONS_00027297	-0.0654717	0.234807	-0.146989	BGIOSGA011245	XP_006659093.1 BTB/POZ domain-containing protein At1g30440-like
TCONS_00021343	-0.0654263	-0.492284	-0.22054	BGIOSGA023570	XP_006657320.1 thylakoid lumenal 16.5 kDa protein, chloroplastic
TCONS_00035013	-0.0653353	-0.665343	0.384797	BGIOSGA033844	XP_006662992.1 glutamyl-tRNA(Gln) amidotransferase subunit B, chloroplastic/mitochondrial

TCONS_00018364	-0.0652012	0.358003	-0.360166	BGIOSGA020416	XP_006655617.1 translation initiation factor IF-2, chloroplastic
TCONS_00001027	-0.0649473	1.00741	0.885364	BGIOSGA024278	XP_015688348.1PREDICTED: uncharacterized protein LOC102715620
TCONS_00025328	-0.0649282	0.69083	0.143783	BGIOSGA014178	XP_015695254.1 probable serine/threonine-protein kinase abkC
TCONS_00037745	-0.0649047	-2.18158	#NA	#N/A	#N/A
TCONS_00018322	-0.0648042	0.51476	-0.0616509	BGIOSGA001073	XP_006654800.1PREDICTED: uncharacterized protein LOC102700994
TCONS_00033924	-0.0646932	0.184091	0.665741	BGIOSGA034056	XP_015698007.1 nuclear pore complex protein NUP107
TCONS_00025050	-0.0646302	0.0653744	0.937721	BGIOSGA024357	XP_006654002.2 glutamate--cysteine ligase B, chloroplastic
TCONS_00035602	-0.0645042	0.55567	-0.378838	BGIOSGA037016	XP_006663832.2 trithorax group protein osa-like
TCONS_00006757	-0.0644888	-0.177406	0.00344463	BGIOSGA008927	XP_006647799.1PREDICTED: uncharacterized protein LOC102721425
TCONS_00019250	-0.0642221	0.84754	0.872672	BGIOSGA018098	XP_015692591.1 ARM REPEAT PROTEIN INTERACTING WITH ABF2
TCONS_00019812	-0.0642072	1.22745	0.281074	BGIOSGA009273	XP_006655600.1 3-ketoacyl-CoA synthase 4-like
TCONS_00004844	-0.0641492	-1.25179	-0.458087	BGIOSGA010987	XP_006645134.1 probable methyltransferase PMT26
TCONS_00024687	-0.0641232	-0.460276	0.0544503	BGIOSGA024698	XP_006657518.1 glycine-rich RNA-binding protein RZ1C-like
TCONS_00016574	-0.0641004	0.603819	0.482741	BGIOSGA014747	XP_006652783.1 mannosyl-oligosaccharide 1,2-alpha-mannosidase MNS1-like
TCONS_00005452	-0.063942	-2.04376	-0.443614	BGIOSGA020625	XP_015688456.1PREDICTED: uncharacterized protein LOC102707005
TCONS_00024836	-0.0639035	0.323603	-0.16547	BGIOSGA007481	XP_006657588.1 protein transport protein SEC13 homolog B-like
TCONS_00036834	-0.0636859	-2.048	-1.35909	BGIOSGA020984	XP_006664413.2 protein SUPPRESSOR OF GENE SILENCING 3 homolog
TCONS_00002234	-0.0635257	-0.133485	0.194739	BGIOSGA004871	XP_015690472.1 sterol 3-beta-glucosyltransferase UGT80B1
TCONS_00007360	-0.0634929	0.21369	0.680375	BGIOSGA007125	XP_006648311.1 VIN3-like protein 2
TCONS_00004285	-0.063425	0.192859	0.200057	BGIOSGA028004	XP_002458398.1protein PLASTID MOVEMENT IMPAIRED 1-RELATED 1
TCONS_00004846	-0.0633422	-0.798525	-0.37254	BGIOSGA019894	XP_015698050.1 zinc finger CCCH domain-containing protein 19-like isoform X1
TCONS_00008934	-0.0632862	0.903194	0.387151	BGIOSGA022458	XP_004954152.126S proteasome regulatory subunit 7A
TCONS_00024047	-0.0632279	-0.104619	-0.42049	BGIOSGA000233	XP_006657925.1 histone deacetylase 5 isoform X1
TCONS_00013306	-0.0629593	0.268128	0.960923	BGIOSGA007229	XP_006651762.1PREDICTED: uncharacterized protein LOC102720419 isoform X1
TCONS_00012095	-0.0629312	0.247069	-0.0564944	BGIOSGA019642	XP_003558119.1uncharacterized protein LOC100845835
TCONS_00009501	-0.0628905	0.315587	0.422997	BGIOSGA003763	XP_006649567.1 serine--tRNA ligase
TCONS_00003796	-0.0626043	-0.0141806	-0.160002	#N/A	#N/A
TCONS_00007412	-0.062476	-0.248944	-0.960436	BGIOSGA007062	XP_015688467.1 peptidyl-prolyl cis-trans isomerase FKBP17-1, chloroplastic
TCONS_00009652	-0.0622106	0.154713	-1.07067	BGIOSGA012197	XP_006649700.1 anaphase-promoting complex subunit 6
TCONS_00005422	-0.0621376	0.484327	0.719223	BGIOSGA001292	XP_006646877.2 splicing factor 3B subunit 1
TCONS_00023205	-0.0621034	-0.540433	0.259726	BGIOSGA035519	XP_006658331.1 ETO1-like protein 1
TCONS_00035979	-0.0620325	#NA	-2.67776	BGIOSGA035291	XP_002442723.1cucumber peeling cupredoxin
TCONS_00000294	-0.0619864	1.68024	-0.27026	BGIOSGA023095	XP_015699259.1 ABC transporter C family member 3-like
TCONS_00007060	-0.0619684	1.40747	0.576442	BGIOSGA023119	XP_004954284.1GATA transcription factor 8
TCONS_00033703	-0.0618734	0.232717	0.02214	BGIOSGA035512	XP_006663539.1 prolycopene isomerase, chloroplastic
TCONS_00037524	-0.0617347	0.819158	0.163476	BGIOSGA035842	XP_015698393.1 histone-lysine N-methyltransferase ATXR7-like isoform X1
TCONS_00036761	-0.0615033	-2.37712	-2.00059	BGIOSGA007161	XP_015698834.1 CBS domain-containing protein CBSCBSPB3-like
TCONS_00018206	-0.0614766	-0.878784	0.239022	BGIOSGA032728	XP_021303107.1myosin-17 isoform X4
TCONS_00002793	-0.0613124	-0.227591	0.306662	BGIOSGA023090	XP_006643735.1 protein DAMAGED DNA-BINDING 2
TCONS_00008708	-0.0611818	0.0170391	-0.129292	BGIOSGA002744	XP_006647806.1 protein ASPARTIC PROTEASE IN GUARD CELL 2-like
TCONS_00033827	-0.0610649	-1.27062	-0.464252	BGIOSGA019356	XP_002449840.1RING-H2 finger protein ATL51
TCONS_00021342	-0.0610161	0.592155	-0.346834	BGIOSGA004046	XP_015693703.1 glycosyltransferase family 64 protein C4
TCONS_00027138	-0.0608739	0.534409	-0.294188	BGIOSGA031291	XP_006659687.1 histidine-containing phosphotransfer protein 1
TCONS_00031533	-0.0606773	0.34573	0.0238125	BGIOSGA028018	XP_006662544.2 mitogen-activated protein kinase 6
TCONS_00016879	-0.0605848	-0.324119	-0.0848704	BGIOSGA020130	XP_006653882.2 putative serine/threonine-protein kinase
TCONS_00010122	-0.0605492	0.406902	0.566808	BGIOSGA026779	XP_006651407.1 probable protein S-acyltransferase 17
TCONS_00019529	-0.0605436	0.433321	-0.127343	BGIOSGA025407	XP_006654560.1 60S ribosomal protein L24-like
TCONS_00013052	-0.0603037	0.916327	0.214137	BGIOSGA009937	XP_006650487.1 coatomer subunit alpha-3 isoform X1
TCONS_00021869	-0.0602288	1.66516	1.06625	BGIOSGA021651	XP_003564074.1serine/threonine-protein phosphatase 2A activator
TCONS_00032754	-0.0600398	-0.0169245	-2.7513	BGIOSGA033586	XP_006662095.2 AT-hook motif nuclear-localized protein 10-like
TCONS_00007256	-0.0600002	-0.379204	0.66113	BGIOSGA007227	XP_015688433.1 glutamate--tRNA ligase, chloroplastic/mitochondrial
TCONS_00001677	-0.0599609	-0.0895776	-0.903518	BGIOSGA021014	XP_015699086.1 V-type proton ATPase subunit B 2-like isoform X1
TCONS_00001863	-0.0596428	-1.01568	-0.17442	BGIOSGA004497	XP_006644745.2 malonate--CoA ligase isoform X1

TCONS_00008455	-0.0593323	-0.633958	-1.32005	BGIOSGA015984	XP_006647577.1 alcohol dehydrogenase-like 6
TCONS_00030664	-0.0592197	-4.65803	-2.67321	BGIOSGA008257	XP_002464781.1acyl transferase 1
TCONS_00020579	-0.0591805	-0.0669786	0.10796	BGIOSGA017093	XP_006649658.2 nuclear pore complex protein NUP155
TCONS_00022311	-0.0591151	-6.42387	-1.7677	BGIOSGA004708	XP_015694128.1 uncharacterized membrane protein At1g06890-like
TCONS_00030095	-0.0590914	-0.499831	-0.0966944	BGIOSGA029707	XP_006660649.1 beta-1,3-galactosyltransferase 6-like
TCONS_00017183	-0.0590373	-0.0787745	-0.110727	BGIOSGA013257	XP_004977101.1dihydrolipoyl dehydrogenase, mitochondrial
TCONS_00024223	-0.05903	-0.726633	-0.2824	BGIOSGA007081	NP_001278542.1COX VIIa-like protein
TCONS_00021055	-0.0588786	0.617267	0.221446	BGIOSGA005739	XP_006656264.1 AT-rich interactive domain-containing protein 5-like
TCONS_00002804	-0.0588325	-0.0967472	0.351744	BGIOSGA029066	XP_006643745.1PREDICTED: uncharacterized protein At3g49720-like isoform X1
TCONS_00009416	-0.0586458	-1.45154	-0.355945	BGIOSGA011969	XP_015689705.1PREDICTED: uncharacterized protein LOC102706542 isoform X3
TCONS_00037227	-0.0583791	-1.06207	-1.04208	BGIOSGA036147	XP_006664576.1PREDICTED: uncharacterized protein LOC102709234, partial
TCONS_00036499	-0.0581428	0.662543	-0.173371	BGIOSGA037868	XP_015698382.1PREDICTED: uncharacterized protein LOC102706435
TCONS_00014840	-0.0581173	-0.755846	0.0911436	BGIOSGA008840	XP_024318338.1uncharacterized protein LOC100830018
TCONS_00011792	-0.0579499	0.549827	0.219818	BGIOSGA037353	XP_006649638.1 pentatricopeptide repeat-containing protein At4g16390, chloroplastic
TCONS_00013488	-0.0578853	0.380312	1.2847	BGIOSGA009503	XP_006651990.1 probable RNA 3'-terminal phosphate cyclase-like protein
TCONS_00005156	-0.0578571	-2.33038	-0.567046	BGIOSGA018305	XP_021316190.1ATPase 10, plasma membrane-type isoform X2
TCONS_00012845	-0.057773	0.597432	-0.0276216	BGIOSGA010127	XP_012698366.1cationic amino acid transporter 2, vacuolar
TCONS_00012917	-0.0576941	-1.19685	0.624661	BGIOSGA032572	XP_006647973.2 separase
TCONS_00002462	-0.0576547	0.640332	0.490208	BGIOSGA005307	XP_002456892.1uncharacterized protein LOC8059401
TCONS_00023505	-0.0576511	-0.501647	0.5164	BGIOSGA031001	XP_006657624.1 nuclear pore complex protein NUP54
TCONS_00015743	-0.0573822	0.0788356	-0.0777917	BGIOSGA008264	XP_006653313.2 60S ribosomal protein L35
TCONS_00010346	-0.0573822	1.85474	1.04944	BGIOSGA011234	XP_003562767.1cysteine proteinase inhibitor 8
TCONS_00010132	-0.0573415	0.449953	0.556276	BGIOSGA007434	XP_003557839.1calcium/calmodulin-dependent serine/threonine-protein kinase 1 isoform X2
TCONS_00026635	-0.0571756	-0.365841	-0.269575	BGIOSGA011244	XP_006660144.2 vacuolar fusion protein CCZ1 homolog
TCONS_00017184	-0.0570687	-0.109795	0.201195	BGIOSGA021093	XP_015692488.1 macrophage erythroblast attacher
TCONS_00012281	-0.0568166	0.944872	0.571559	BGIOSGA009630	XP_015690283.1PREDICTED: uncharacterized protein LOC102701624
TCONS_00011451	-0.0568166	0.0693845	0.423731	BGIOSGA011573	XP_015689744.1 COP9 signalosome complex subunit 1
TCONS_00017605	-0.0566531	0.996208	0.336196	BGIOSGA019664	XP_006654284.1PREDICTED: uncharacterized protein LOC102717247
TCONS_00021438	-0.0565027	0.0672243	1.12675	BGIOSGA022113	XP_006656539.1 homogentisate 1,2-dioxygenase
TCONS_00018458	-0.0564841	0.27349	-0.404038	BGIOSGA002575	XP_006644461.1PREDICTED: uncharacterized protein LOC102713683
TCONS_00007928	-0.0563074	-0.814364	-0.520111	BGIOSGA006558	XP_015689509.1 vacuolar protein sorting-associated protein 45 homolog
TCONS_00007578	-0.0563022	0.089612	0.223119	BGIOSGA029561	XP_008645668.1UDP-glycosyltransferase 73C3
TCONS_00035577	-0.0560245	0.905322	1.78323	BGIOSGA034798	XP_006663817.1 DEAD-box ATP-dependent RNA helicase 52B
TCONS_00010083	-0.0557269	-0.477286	0.0261397	BGIOSGA012629	XP_006650088.1 ribosomal RNA-processing protein 17
TCONS_00004706	-0.0556539	-5.99601	-3.42714	BGIOSGA024987	XP_006644993.1 laccase-4
TCONS_00026855	-0.0555919	0.374566	0.87498	BGIOSGA028909	XP_006660246.1 elongator complex protein 2
TCONS_00025854	-0.0554156	0.0271586	-0.415934	BGIOSGA009278	XP_006646776.1 60S ribosomal protein L37-1-like
TCONS_00031376	-0.0550972	-0.947913	1.30066	BGIOSGA010707	XP_006661897.1 alpha-galactosidase
TCONS_00028951	-0.0548862	-1.35063	-0.0757493	BGIOSGA033375	XP_006660601.1 probable plastidic glucose transporter 1 isoform X2
TCONS_00026416	-0.0547029	-0.49013	0.136285	BGIOSGA003622	XP_006659315.2 protein ENHANCED DOWNY MILDEW 2-like isoform X1
TCONS_00030830	-0.0545343	-1.22682	-0.199242	BGIOSGA036596	XP_006662240.1PREDICTED: uncharacterized protein LOC102705752
TCONS_00005343	-0.0543217	-1.37214	0.402652	BGIOSGA018630	XP_023157549.1uncharacterized protein LOC103646630
TCONS_00008171	-0.0539614	-0.095855	0.527425	BGIOSGA036534	XP_006647364.1 probable L-ascorbate peroxidase 8, chloroplastic
TCONS_00002264	-0.0538633	0.7344	0.463108	BGIOSGA014027	XP_006645092.2 probable magnesium transporter NIPA6
TCONS_00021811	-0.0538305	0.890716	-0.113207	BGIOSGA021702	XP_006656744.2 mechanosensitive ion channel protein 10
TCONS_00019632	-0.0535696	-0.126376	0.258601	BGIOSGA000815	XP_006654646.1 transcription factor VOZ1-like
TCONS_00027053	-0.0533246	-2.10926	-2.70815	BGIOSGA029102	XP_015688705.1 shikimate O-hydroxycinnamoyltransferase-like
TCONS_00031043	-0.0532293	-0.41178	-0.0135399	BGIOSGA034004	XP_015696723.1 putative disease resistance protein RGA3
TCONS_00028074	-0.0531787	-0.683633	-0.632117	BGIOSGA026918	XP_006660166.1 omega-6 fatty acid desaturase, chloroplastic
TCONS_00017676	-0.0531264	-0.157178	0.311998	BGIOSGA002441	XP_024315197.1receptor-like cytoplasmic kinase 185 isoform X2
TCONS_00000632	-0.0531174	0.78602	0.914641	BGIOSGA026793	XP_015688185.1 methyltransferase-like protein 1
TCONS_00024493	-0.0530519	1.34983	0.991612	BGIOSGA032295	XP_022679705.1uncharacterized protein LOC111256259
TCONS_00002901	-0.0529797	-1.67425	-0.73232	BGIOSGA002318	XP_015694686.1 mini-chromosome maintenance complex-binding protein

TCONS_00013295	-0.0529458	-0.109733	-0.249659	BGIOSGA035815	XP_006650700.1 protein IQ-DOMAIN 1-like isoform X1
TCONS_00030324	-0.0529201	0.0277379	0.968918	BGIOSGA029484	XP_015696546.1PREDICTED: uncharacterized protein LOC102721954
TCONS_00019909	-0.0527865	0.691591	0.394951	BGIOSGA037444	XP_015692994.1 eukaryotic translation initiation factor 5B-like
TCONS_00012329	-0.0527376	-1.49081	0.340789	BGIOSGA026221	XP_006650123.1 novel plant SNARE 12-like isoform X2
TCONS_00010052	-0.0526133	1.01487	0.726875	BGIOSGA010714	XP_006650063.1 U3 small nucleolar RNA-associated protein 14-like
TCONS_00031380	-0.0524724	-1.83044	0.25051	#N/A	XP_015697222.1PREDICTED: uncharacterized protein LOC102708649 isoform X2
TCONS_00012187	-0.0524286	0.474579	0.334024	BGIOSGA010818	XP_015690293.1 U11/U12 small nuclear ribonucleoprotein 65 kDa protein
TCONS_00006956	-0.0523979	0.361006	0.259856	BGIOSGA009181	XP_006649094.2 isoleucine--tRNA ligase, chloroplastic/mitochondrial
TCONS_00015073	-0.0520886	0.661039	0.394149	BGIOSGA026901	XP_015691599.1 D-3-phosphoglycerate dehydrogenase 1, chloroplastic-like
TCONS_00009933	-0.0517694	-0.535093	0.260572	BGIOSGA019269	XP_006649975.1 probable enoyl-CoA hydratase 1, peroxisomal
TCONS_00011529	-0.0517607	-0.227155	0.374891	BGIOSGA011501	XP_006649353.2 F-box/LRR-repeat protein 10
TCONS_00017611	-0.0517428	1.42221	0.230619	BGIOSGA010932	XP_006654281.1 importin subunit beta-1-like
TCONS_00007532	-0.0516575	0.486328	0.569582	BGIOSGA002970	XP_003571257.1outer envelope protein 80, chloroplastic
TCONS_00005374	-0.0516435	0.344746	0.262423	BGIOSGA020570	XP_015689577.1PREDICTED: uncharacterized protein LOC102704209 isoform X1
TCONS_00021721	-0.0516134	0.375858	0.650186	BGIOSGA021800	XP_006656700.1 protein NUCLEAR FUSION DEFECTIVE 4
TCONS_00038464	-0.0513281	-3.2373	-2.97612	BGIOSGA030641	NP_001131192.1retrotransposon protein precursor
TCONS_00014955	-0.0512182	0.643576	0.537141	BGIOSGA017142	XP_006652836.1 OTU domain-containing protein 6B
TCONS_00003131	-0.0511513	1.60867	-1.81626	BGIOSGA033988	XP_015699148.1 mitochondrial arginine transporter BAC2
TCONS_00013067	-0.0511303	0.957067	1.57412	BGIOSGA009915	XP_003570580.1splicing factor SF3a60 homolog
TCONS_00012554	-0.0511014	1.31115	-0.0799618	BGIOSGA016743	XP_006650252.1 probable dolichyl-diphosphooligosaccharide--protein glycosyltransferase subunit 3
TCONS_00002583	-0.05104	-0.80265	0.606554	BGIOSGA005226	XP_024312259.1uncharacterized protein LOC100842716
TCONS_00028471	-0.050985	-0.0731765	0.58198	BGIOSGA026485	XP_015695874.1 dnaJ homolog subfamily C member 28-like
TCONS_00003564	-0.0506964	0.0754691	-0.157267	BGIOSGA001638	XP_006644171.1 serine/arginine repetitive matrix protein 5-like
TCONS_00025101	-0.0505828	0.363239	0.487729	BGIOSGA024299	XP_006657705.1PREDICTED: uncharacterized protein LOC102711274
TCONS_00009124	-0.0505721	-0.585208	-0.326586	BGIOSGA031095	XP_006649221.1 palmitoyl-protein thioesterase 1-like
TCONS_00001300	-0.0504235	-0.305545	-0.393388	BGIOSGA015786	XP_024318484.1 receptor-like protein EIX1
TCONS_00013269	-0.0503944	0.259587	-0.608908	BGIOSGA011132	XP_006650675.1 actin-depolymerizing factor 2
TCONS_00005290	-0.0501632	-0.575221	0.0529126	BGIOSGA037541	XP_015689664.1 metallocarboxypeptidase A-like protein ARB_03789
TCONS_00013588	-0.0499961	0.189414	-0.375959	BGIOSGA007532	XP_015690156.1 rhodanese-like domain-containing protein 8, chloroplastic
TCONS_00029321	-0.0499923	0.0363535	0.563551	BGIOSGA029064	XP_015696781.1 pyruvate dehydrogenase E1 component subunit beta-2, mitochondrial
TCONS_00004475	-0.0499726	-0.68416	-0.273129	BGIOSGA010097	XP_006644770.1 transmembrane protein 56-like
TCONS_00025042	-0.0498632	-0.500801	0.278928	BGIOSGA024364	XP_006658532.1PREDICTED: uncharacterized protein slr1919
TCONS_00026484	-0.0496965	0.13218	0.703626	BGIOSGA018629	XP_023157549.1uncharacterized protein LOC103646630
TCONS_00009372	-0.0496866	0.345609	0.321791	BGIOSGA011923	XP_015689774.1 putative ion channel POLLUX-like 2
TCONS_00008978	-0.0493475	0.215404	0.724531	BGIOSGA005428	XP_006648059.1PREDICTED: uncharacterized protein LOC102702632
TCONS_00018665	-0.0493233	-0.0368824	-0.292725	BGIOSGA007895	XP_006654078.1 GDSL esterase/lipase LIP-4-like
TCONS_00031396	-0.0491845	-1.934	-0.779583	BGIOSGA007810	XP_006661911.1 probable receptor-like protein kinase At2g42960
TCONS_00005004	-0.0491813	-0.0390254	0.247377	BGIOSGA000217	XP_006646634.2 transaldolase
TCONS_00005695	-0.048961	-0.0776956	0.937704	BGIOSGA007817	XP_006648470.1 xaa-Pro dipeptidase
TCONS_00027241	-0.0486486	0.727527	0.697954	BGIOSGA027762	XP_006659058.1 squamous cell carcinoma antigen recognized by T-cells 3
TCONS_00007126	-0.0486392	0.749643	1.91201	BGIOSGA009310	XP_006648138.1 mediator of RNA polymerase II transcription subunit 36a-like
TCONS_00004944	-0.0485408	-1.21991	-0.826251	BGIOSGA000277	XP_006646594.2 ABC transporter B family member 26, chloroplastic
TCONS_00029542	-0.048416	-0.773265	-0.577333	BGIOSGA007606	XP_015696570.1PREDICTED: uncharacterized protein LOC102708365 isoform X1
TCONS_00037339	-0.0479977	-0.646212	0.371499	BGIOSGA029804	XP_002444474.1protein trichome birefringence-like 16
TCONS_00032640	-0.0478521	0.21793	0.31443	BGIOSGA031472	XP_015697344.1 zinc finger protein GIS2-like
TCONS_00014134	-0.0478304	0.679453	-0.0964201	BGIOSGA025327	XP_021319649.1ACT domain-containing protein ACR6-like
TCONS_00009290	-0.0478125	1.07637	0.52269	BGIOSGA031734	XP_006649384.1 60S ribosomal protein L21-1
TCONS_00029480	-0.0476242	-1.33751	-1.67456	BGIOSGA031234	XP_006660953.1PREDICTED: uncharacterized protein LOC102718768
TCONS_00000851	-0.0474462	0.32125	0.393242	BGIOSGA008407	XP_006644146.1 protein disulfide isomerase-like 2-2
TCONS_00027552	-0.0474135	0.205477	-0.105055	BGIOSGA014776	XP_006659214.1 40S ribosomal protein S11-like
TCONS_00014889	-0.04736	0.991868	0.684893	BGIOSGA031510	XP_006652780.1 60S ribosomal protein L7-3-like
TCONS_00008161	-0.0473204	0.0248207	-0.328305	BGIOSGA006280	XP_024318176.1trafficking protein particle complex subunit 12

TCONS_00015433	-0.0472647	0.299049	-0.735347	BGIOSGA015598	XP_006652106.1 G-type lectin S-receptor-like serine/threonine-protein kinase RLK1
TCONS_00013569	-0.0471799	-2.15924	0.246204	BGIOSGA015775	XP_006652043.1 probable carbohydrate esterase At4g34215
TCONS_00015679	-0.047178	0.432118	0.342374	BGIOSGA008232	XP_006652189.2 40S ribosomal protein S8-like
TCONS_00016365	-0.0471751	-0.560325	-0.521311	BGIOSGA003050	XP_003580275.1putative kinase-like protein TMKL1
TCONS_00032854	-0.0470592	0.109525	0.215286	BGIOSGA034672	XP_015698025.1PREDICTED: uncharacterized protein LOC102711575 isoform X1
TCONS_00031990	-0.0470436	0.298453	0.323988	BGIOSGA012108	XP_020401151.1hornerin isoform X1
TCONS_00013176	-0.047016	0.218096	0.118398	BGIOSGA002654	XP_015690713.1 DExH-box ATP-dependent RNA helicase DExH3
TCONS_00037559	-0.0466486	-0.365591	-0.19424	BGIOSGA000219	XP_006664219.2 dihydrofolate synthetase
TCONS_00016185	-0.0464832	0.820076	0.757654	BGIOSGA023011	XP_015692106.1 eukaryotic translation initiation factor isoform 4G-1
TCONS_00018898	-0.0463671	-0.0317994	-0.78578	BGIOSGA018461	XP_003568712.1uncharacterized protein LOC100836950
TCONS_00035512	-0.0461758	-0.54855	0.420798	BGIOSGA010845	XP_006663783.1 alpha-L-arabinofuranosidase 1-like
TCONS_00006933	-0.045809	0.545016	-0.024551	BGIOSGA021698	XP_006647968.2PREDICTED: uncharacterized protein LOC102700684 isoform X1
TCONS_00027773	-0.045728	-2.15801	0.258593	BGIOSGA027224	NP_001130533.1lipid binding protein
TCONS_00020540	-0.0456122	0.0934973	0.695678	BGIOSGA022711	XP_015693549.1 DNA oxidative demethylase ALKBH2
TCONS_00009911	-0.045536	-0.269645	-0.3858	BGIOSGA026433	XP_006649964.2 probable glutamyl endopeptidase, chloroplastic
TCONS_00011502	-0.0454616	0.317912	0.612044	BGIOSGA011524	XP_006651005.1 epidermal growth factor receptor substrate 15-like
TCONS_00022137	-0.0452978	-0.265727	-0.682024	BGIOSGA029564	XP_015693789.1 putative BPI/LBP family protein At1g04970
TCONS_00034531	-0.045286	-0.273194	0.367099	BGIOSGA026237	XP_004963300.1xylulose kinase
TCONS_00011637	-0.0452405	-0.642021	0.451288	BGIOSGA030628	XP_003558721.1uncharacterized protein LOC100825547
TCONS_00037512	-0.0447581	-2.84037	-0.952476	BGIOSGA006114	XP_004963161.1patatin-like protein 3
TCONS_00017380	-0.0447327	0.21679	0.497176	BGIOSGA019441	XP_006654171.1 serine/threonine-protein kinase TOR
TCONS_00016673	-0.0443441	-0.00402135	0.145125	BGIOSGA012967	XP_010240474.1G-type lectin S-receptor-like serine/threonine-protein kinase At1g11330 isoform X1
TCONS_00025658	-0.0443278	-0.39445	-0.208002	BGIOSGA023764	XP_006658071.1PREDICTED: uncharacterized protein LOC102721379
TCONS_00035526	-0.0442625	-0.233513	-0.49567	BGIOSGA036907	XP_015697974.1 CBL-interacting protein kinase 32
TCONS_00019535	-0.0442591	-0.215539	0.675298	BGIOSGA025403	XP_015692748.1 ADP-ribosylation factor 2 isoform X1
TCONS_00024269	-0.044191	0.0567581	-0.366273	BGIOSGA026313	XP_006658913.1 thioredoxin reductase NTRC
TCONS_00004855	-0.0441518	-0.664366	0.562306	BGIOSGA024834	XP_015693211.1 keratin, type II cytoskeletal 5-like
TCONS_00015336	-0.0440311	0.157356	0.615142	BGIOSGA024499	XP_006652094.1 protein argonaute 4B
TCONS_00014284	-0.0440205	-0.246149	-0.244275	BGIOSGA016439	XP_015692311.1 F-box protein 7 isoform X1
TCONS_00011974	-0.0438226	-0.535857	0.211706	BGIOSGA011740	XP_003561787.1serine/threonine-protein kinase ATG1c isoform X1
TCONS_00001552	-0.0436766	-0.185287	0.737699	BGIOSGA004182	XP_006646192.1 non-canonical poly(A) RNA polymerase PAPD5
TCONS_00028465	-0.043645	-0.0981671	0.17833	BGIOSGA026490	XP_006659695.1 multiple myeloma tumor-associated protein 2 homolog
TCONS_00027629	-0.0435525	0.0841104	-0.675359	BGIOSGA027390	XP_015696083.1 afadin- and alpha-actinin-binding protein-like isoform X2
TCONS_00016336	-0.04353	-0.425452	-0.872611	BGIOSGA005930	XP_006653627.1 probable metal-nicotianamine transporter YSL9
TCONS_00009735	-0.0433911	0.199818	0.272447	BGIOSGA019953	XP_006649802.1 coronatine-insensitive protein homolog 2
TCONS_00022983	-0.0433738	0.107828	0.470822	BGIOSGA008138	XP_006657397.1 serine/arginine repetitive matrix protein 2
TCONS_00009971	-0.043256	-0.117977	-0.208706	BGIOSGA000583	XP_006650007.1 WRKY transcription factor 44-like
TCONS_00008620	-0.0432139	-1.15871	-2.12833	BGIOSGA013408	XP_006647719.1 uncharacterized membrane protein At4g09580
TCONS_00013328	-0.0430065	-0.331614	-1.12032	BGIOSGA008724	XP_010940064.1 glutamate dehydrogenase 1, mitochondrial isoform X2
TCONS_00009868	-0.0429752	-0.446567	-1.57169	BGIOSGA005021	XP_003558129.1mitoferrin isoform X1
TCONS_00018308	-0.042968	-4.28344	-2.16174	BGIOSGA002349	XP_015692701.1 fasciclin-like arabinogalactan protein 12
TCONS_00016215	-0.042935	-1.30397	-1.65899	BGIOSGA002157	XP_006652492.1 protein LOW PSII ACCUMULATION 1, chloroplastic isoform X2
TCONS_00013543	-0.0428818	-0.945405	-0.940972	BGIOSGA005094	XP_006650900.1 random slug protein 5-like
TCONS_00019627	-0.042759	-0.137518	-1.1859	BGIOSGA017731	XP_006655483.2 proline iminopeptidase
TCONS_00008961	-0.0426273	-0.0356976	-0.0462932	BGIOSGA023968	XP_006648044.2 formin-like protein 7
TCONS_00012195	-0.0422452	0.279164	0.340315	BGIOSGA021512	XP_006650027.1 endoglucanase 9
TCONS_00020191	-0.0421621	0.100637	0.422594	BGIOSGA005409	XP_006656686.2 AP-1 complex subunit gamma-2-like isoform X1
TCONS_00028298	-0.0419637	0.192471	0.62896	BGIOSGA026681	XP_015696231.1 protein CROWDED NUCLEI 3
TCONS_00024260	-0.0418545	-1.01573	-0.353912	BGIOSGA026302	XP_006658053.2 TVP38/TMEM64 family membrane protein slr0305
TCONS_00006096	-0.0418484	-0.59922	1.16001	BGIOSGA006439	XP_006648646.2 transcriptional activator DEMETER-like isoform X1
TCONS_00011666	-0.0418037	-0.998611	-1.19712	BGIOSGA020639	XP_004985506.1copper-transporting ATPase PAA2, chloroplastic
TCONS_00032718	-0.0417676	-0.805174	0.717153	BGIOSGA025202	XP_006662072.1 mediator of RNA polymerase II transcription subunit 31
TCONS_00019572	-0.0416654	-0.307992	0.338896	BGIOSGA017785	XP_016649378.1 B2 protein
TCONS_00007313	-0.0416028	-0.243435	0.00494861	BGIOSGA006577	XP_015689404.1 mitogen-activated protein kinase 13 isoform X1

TCONS_00005368	-0.0415396	0.0983684	0.395	BGIOSGA014171	XP_004951961.1myb-binding protein 1A-like protein
TCONS_00003967	-0.0413407	-3.2078	-2.7985	BGIOSGA008891	XP_002458146.1thrombospondin type-1 domain-containing protein 4
TCONS_00024972	-0.0411784	-1.15738	-0.652065	BGIOSGA024422	NP_001151489.1F-actin capping protein alpha subunit
TCONS_00032156	-0.0410081	0.501226	0.36977	BGIOSGA028040	XP_015697387.1 F-box/LRR-repeat protein 15
TCONS_00029854	-0.0407145	0.227491	0.287643	BGIOSGA002266	XP_004956611.1ABC transporter G family member 53
TCONS_00032581	-0.0407096	-0.146897	0.428496	BGIOSGA009182	XP_006661969.1 probable E3 ubiquitin-protein ligase XBOS33
TCONS_00004479	-0.0406898	0.250484	0.20654	BGIOSGA003162	XP_003569912.1F-box protein At4g18380
TCONS_00016598	-0.0406477	-0.251218	-0.460533	BGIOSGA007321	XP_003580529.1coatomer subunit epsilon-1
TCONS_00018205	-0.0403592	2.13081	0.722593	BGIOSGA017690	XP_006654710.1 probable WRKY transcription factor 26
TCONS_00024681	-0.0400625	1.60386	-0.071513	BGIOSGA024703	XP_024313011.1ylmG homolog protein 1-2, chloroplastic
TCONS_00008053	-0.0400302	0.582855	0.785868	BGIOSGA006402	XP_015698022.1 SART-1 family protein DOT2 isoform X1
TCONS_00027978	-0.0400241	1.79054	0.590506	BGIOSGA027018	XP_015695855.1 pre-mRNA cleavage factor Im 25 kDa subunit 1-like
TCONS_00037280	-0.039939	-0.380854	0.77013	BGIOSGA036093	XP_006664063.1PREDICTED: uncharacterized protein LOC102708390
TCONS_00001243	-0.0399144	0.106582	0.0606942	BGIOSGA035841	XP_006644322.1 single myb histone 2-like isoform X1
TCONS_00023552	-0.0398528	0.398516	-0.289892	BGIOSGA006754	XP_015695131.1 protein TSS-like
TCONS_00007921	-0.0398256	-0.272907	0.247708	BGIOSGA032925	NP_001151182.1protein transporter
TCONS_00015795	-0.0397092	0.545032	1.57822	BGIOSGA039198	XP_015691715.1 DNA-directed RNA polymerase III subunit rpc4
TCONS_00027464	-0.0393811	-0.541338	0.155487	BGIOSGA027550	XP_006659891.1 mRNA cap guanine-N7 methyltransferase 1
TCONS_00011709	-0.0391844	-0.0402772	-0.850827	BGIOSGA011301	XP_004985391.1probable mitochondrial adenine nucleotide transporter BTL3
TCONS_00034966	-0.0391361	0.00927571	-0.642801	BGIOSGA033884	XP_015697853.1PREDICTED: uncharacterized protein LOC102705960
TCONS_00007629	-0.0391071	-1.69164	-0.851737	BGIOSGA011717	XP_006661972.1 F-box protein PP2-A13-like
TCONS_00021064	-0.039085	1.16368	-0.0731793	BGIOSGA035191	XP_015693395.1PREDICTED: uncharacterized protein LOC102704621 isoform X1
TCONS_00008792	-0.0390729	0.496206	1.17247	BGIOSGA005644	XP_015688965.1 protein LSM12 homolog
TCONS_00020779	-0.0389211	0.58757	1.09739	BGIOSGA012884	XP_006660073.1 nucleolar complex protein 3 homolog
TCONS_00008258	-0.0388684	0.959005	0.195291	BGIOSGA014922	XP_015688650.1PREDICTED: uncharacterized protein LOC102716382
TCONS_00021832	-0.0388684	-0.51048	-0.104269	BGIOSGA021686	XP_004964897.1protein TIC 22, chloroplastic
TCONS_00013976	-0.0388174	0.646997	0.718726	BGIOSGA014629	XP_006660481.1 cyclin-P4-1-like
TCONS_00011480	-0.0386774	-0.333474	0.445163	BGIOSGA033347	XP_012698377.1C-terminal binding protein AN
TCONS_00000625	-0.0383826	0.646446	0.894516	BGIOSGA003208	XP_015698686.1 pre-mRNA-splicing factor SPF27 homolog
TCONS_00013868	-0.0382993	0.0989432	0.018503	BGIOSGA018578	XP_006652006.1 BTB/POZ domain-containing protein At2g13690-like
TCONS_00029063	-0.038173	-0.42371	-0.343178	BGIOSGA030810	XP_015696362.1 pentatricopeptide repeat-containing protein At5g10690
TCONS_00013482	-0.0381527	0.263961	1.27597	BGIOSGA009509	XP_015690138.1 phosphoribosylformylglycinamidine cyclo-ligase, chloroplastic/mitochondrial
TCONS_00016570	-0.0380816	-0.104764	0.408772	BGIOSGA028240	XP_006652780.1 60S ribosomal protein L7-3-like
TCONS_00022668	-0.0379415	-1.47092	0.152601	BGIOSGA000252	XP_004965715.1chaperone protein dnaJ 8, chloroplastic
TCONS_00018958	-0.0378921	0.499655	1.66155	BGIOSGA018405	XP_015692965.1PREDICTED: uncharacterized protein LOC102720606
TCONS_00007622	-0.0377679	0.00567954	0.186479	BGIOSGA023102	XP_015689436.1 glycine-rich RNA-binding protein 3, mitochondrial-like
TCONS_00026838	-0.0377436	-2.21234	0.362405	BGIOSGA027198	XP_004973769.1fasciclin-like arabinogalactan protein 1
TCONS_00028509	-0.0375811	-0.656626	-0.258129	BGIOSGA030262	XP_015696900.1PREDICTED: uncharacterized protein LOC102720903
TCONS_00009235	-0.0375368	-1.05019	-0.950436	BGIOSGA023834	XP_019709773.1 calcium-dependent protein kinase 5 isoform X2
TCONS_00022606	-0.0375022	-0.181162	-1.17453	BGIOSGA020832	XP_006656272.1 tryptophan synthase beta chain 2 isoform X1
TCONS_00004919	-0.0374768	-2.12522	-0.678903	BGIOSGA000304	XP_006645188.1 two-component response regulator ORR26
TCONS_00009618	-0.0374419	-0.00220393	-0.453943	BGIOSGA025345	XP_006649665.1 coatomer subunit gamma-1
TCONS_00010599	-0.0374318	0.621541	0.295065	BGIOSGA013221	XP_015690689.1 DNA-directed RNA polymerase II subunit RPB2 isoform X3
TCONS_00004765	-0.0374236	-0.364542	0.433517	BGIOSGA000450	XP_015699264.1 F-box/LRR-repeat protein At1g67190
TCONS_00037385	-0.0372968	1.03502	0.99872	BGIOSGA035986	XP_006664651.2 UNC93-like protein 3 isoform X1
TCONS_00032486	-0.0369142	-0.904453	0.538555	BGIOSGA019154	XP_006661896.2PREDICTED: uncharacterized protein LOC102708090
TCONS_00006979	-0.0368395	0.137125	0.0732618	BGIOSGA020339	XP_006648023.1 60S ribosomal protein L35a-1-like
TCONS_00004159	-0.036731	-0.0188924	-0.565754	BGIOSGA001088	XP_004969503.1ubiquitin-conjugating enzyme E2 36
TCONS_00011636	-0.0365959	-0.00619759	0.445176	BGIOSGA030832	XP_006649467.1 50S ribosomal protein L18
TCONS_00032143	-0.0365632	-0.31503	0.0335799	BGIOSGA031995	XP_015697358.1 signal peptide peptidase-like 1
TCONS_00017744	-0.0365337	-0.788335	-0.0155949	BGIOSGA019806	XP_015692586.1 mannosylglycoprotein endo-beta-mannosidase
TCONS_00030303	-0.0364054	-1.10134	0.117844	BGIOSGA035508	XP_004957254.1uncharacterized protein LOC101756298
TCONS_00033296	-0.0362679	-3.34984	-1.59745	BGIOSGA030469	XP_003577653.4protein NRT1/ PTR FAMILY 4.5
TCONS_00002481	-0.0362563	0.637072	-0.332429	BGIOSGA036737	XP_006645297.1 serine/threonine-protein kinase CDL1-like

TCONS_00028708	-0.0362288	0.711488	0.0805172	BGIOSGA006669	XP_006660509.1PREDICTED: uncharacterized protein LOC102702593 isoform X1
TCONS_00004607	-0.0359525	0.0508065	0.0360437	BGIOSGA037495	XP_006644889.1 vacuolar protein sorting-associated protein 36
TCONS_00018785	-0.035825	0.592589	0.711264	BGIOSGA022348	XP_006655076.1 probable beta-1,3-galactosyltransferase 12
TCONS_00025791	-0.035733	0.0111086	-0.0623821	BGIOSGA027855	XP_006659035.1 TOM1-like protein 2
TCONS_00022911	-0.0355633	0.850993	0.613317	BGIOSGA020532	XP_015693846.1 BRCA1-A complex subunit BRE
TCONS_00022939	-0.0353609	-0.104797	-0.26045	BGIOSGA015140	XP_006656510.2 1,4-alpha-glucan-branching enzyme, chloroplastic/amyloplastic
TCONS_00011106	-0.0353589	-2.12859	0.597851	BGIOSGA029210	XP_003559345.1probable glutamate carboxypeptidase PLA3 isoform X2
TCONS_00022810	-0.0353147	-1.36726	-0.407673	BGIOSGA023515	XP_015693689.1 putative cysteine-rich receptor-like protein kinase 20
TCONS_00035037	-0.0352777	0.00999956	1.007	BGIOSGA033819	XP_006662985.1PREDICTED: uncharacterized protein LOC102706141
TCONS_00021725	-0.035229	1.03241	-0.813778	BGIOSGA003238	XP_010227959.1hydroxyproline O-arabinosyltransferase 1
TCONS_00026470	-0.0351739	-2.63062	-1.22895	BGIOSGA009997	XP_015695839.1 protein tesmin/TSO1-like CXC 7
TCONS_00032465	-0.03512	0.784346	0.0685946	BGIOSGA010653	XP_006661882.1 E3 ubiquitin-protein ligase RING1-like
TCONS_00028166	-0.03512	0.722945	-2.39901	BGIOSGA024776	XP_004973676.1probable carboxylesterase 5
TCONS_00004376	-0.0350816	0.135816	0.155339	BGIOSGA000844	XP_022682565.1casein kinase 1-like protein HD16
TCONS_00026529	-0.0349982	0.267275	-0.165258	BGIOSGA022413	XP_004973352.2hydroxyproline O-galactosyltransferase HPGT1
TCONS_00014556	-0.0349844	-0.579465	-0.0884365	BGIOSGA008944	XP_015691514.1 calcium uptake protein 1 homolog, mitochondrial-like
TCONS_00036636	-0.0346975	0.201955	0.137009	BGIOSGA036634	XP_006662723.1 proteasome-associated protein ECM29 homolog isoform X2
TCONS_00017187	-0.0346341	0.73793	0.0916377	BGIOSGA018762	XP_015692956.1PREDICTED: uncharacterized protein LOC107304245
TCONS_00019622	-0.034505	0.275425	0.707989	BGIOSGA017735	XP_006655477.2 BAG family molecular chaperone regulator 8, chloroplastic
TCONS_00029780	-0.0344911	0.527715	-0.96761	BGIOSGA030035	XP_006660519.1 two pore potassium channel c-like
TCONS_00017125	-0.034467	-0.101171	0.4235	BGIOSGA019173	XP_006654043.1 protease Do-like 2, chloroplastic
TCONS_00011148	-0.0344651	0.797159	0.403903	BGIOSGA013847	XP_015690709.1 RNA-binding protein 28 isoform X1
TCONS_00017996	-0.0343694	-1.04667	-2.23095	BGIOSGA020044	XP_015692632.1 WEB family protein At2g38370-like
TCONS_00000437	-0.0343044	-0.658685	-0.055257	BGIOSGA014789	XP_015692833.1 protein DJ-1 homolog B-like
TCONS_00021001	-0.0342912	1.97443	0.710541	BGIOSGA035520	XP_003563656.1EIN3-binding F-box protein 1
TCONS_00009895	-0.0342097	-0.049041	0.12429	BGIOSGA001803	XP_004984650.1uncharacterized protein LOC101764593
TCONS_00001947	-0.0341798	0.768056	0.0905128	BGIOSGA004586	XP_015690165.1 pentatricopeptide repeat-containing protein At1g55890, mitochondrial-like
TCONS_00032683	-0.0339485	0.308338	0.63476	BGIOSGA036417	XP_006662050.1 CCR4-NOT transcription complex subunit 1
TCONS_00022524	-0.0338873	-0.253237	-0.22705	BGIOSGA020927	XP_006656215.1 heme oxygenase 1, chloroplastic
TCONS_00009201	-0.0338542	-1.89532	-0.00982844	BGIOSGA029108	XP_006649284.2 hydroxymethylglutaryl-CoA synthase-like
TCONS_00010889	-0.0338232	-5.53921	-0.331833	BGIOSGA008839	XP_015690053.1 serine carboxypeptidase-like 7
TCONS_00029831	-0.0337927	0.274529	0.540217	BGIOSGA004614	XP_020404942.1uncharacterized protein LOC109944582
TCONS_00019901	-0.0336942	-0.480482	0.182421	BGIOSGA003944	XP_006654862.1 AAA-ATPase At3g50940-like
TCONS_00012749	-0.0336942	1.64053	1.43035	BGIOSGA016262	XP_006650335.1 U3 snoRNP-associated protein-like YAOH
TCONS_00006077	-0.0335995	0.709534	-0.0298328	BGIOSGA015295	XP_006648644.2 40S ribosomal protein S8
TCONS_00004524	-0.0334921	1.53026	0.674642	BGIOSGA026827	XP_003564362.1universal stress protein PHOS32
TCONS_00017809	-0.0334677	-1.99534	0.181862	BGIOSGA004651	XP_004967662.1tubulin beta-5 chain
TCONS_00020760	-0.0334423	0.623538	-2.8182	BGIOSGA024257	XP_006656105.1 protein DETOXIFICATION 16-like
TCONS_00010581	-0.0334173	-0.449063	-0.0889675	BGIOSGA013196	XP_006659111.1 protein ALWAYS EARLY 2 isoform X3
TCONS_00023969	-0.033328	-1.41055	-1.59609	BGIOSGA026000	XP_015694700.1 glucan endo-1,3-beta-glucosidase 4-like
TCONS_00013929	-0.0332763	0.229694	0.0743096	BGIOSGA016104	XP_006652161.1 aminoacyl tRNA synthase complex-interacting multifunctional protein 1
TCONS_00012087	-0.0332281	1.1512	0.766232	BGIOSGA024669	XP_024313368.1probable mediator of RNA polymerase II transcription subunit 26c
TCONS_00011312	-0.0331375	0.858055	0.452884	BGIOSGA013918	XP_006650880.1 30S ribosomal protein S6 alpha, chloroplastic
TCONS_00015183	-0.0330436	-0.459072	-0.635836	BGIOSGA017365	XP_006653037.1 probable allantoinase
TCONS_00030810	-0.0330348	-1.73339	-0.0793839	BGIOSGA032618	NP_001150888.2CMV 1a interacting protein 1
TCONS_00025334	-0.0328645	-1.55709	-2.18478	BGIOSGA011584	XP_004958017.1uncharacterized protein LOC101785046
TCONS_00019332	-0.0327564	-0.674305	-2.08293	BGIOSGA036073	NP_001147922.2phenylalanine ammonia-lyase
TCONS_00016445	-0.0326962	0.325619	-0.820043	BGIOSGA000169	XP_006652658.1 homeobox-leucine zipper protein ROC4
TCONS_00023898	-0.0326772	0.665316	-0.183023	BGIOSGA025931	XP_015695124.1 eukaryotic translation initiation factor 4G isoform X1
TCONS_00026372	-0.0322574	-0.727782	0.797213	BGIOSGA028442	XP_015696134.1PREDICTED: uncharacterized protein LOC102719788 isoform X1
TCONS_00012397	-0.0320379	0.10866	-0.0232988	BGIOSGA026152	XP_015691130.1 protein WEAK CHLOROPLAST MOVEMENT UNDER BLUE LIGHT 1-like
TCONS_00008851	-0.0320028	-0.377607	0.441098	BGIOSGA001286	XP_006649069.1 cytoplasmic tRNA 2-thiolation protein 1
TCONS_00029290	-0.0317795	-0.501032	-0.0853353	BGIOSGA031033	XP_015696867.1 DNA topoisomerase 3-beta

TCONS_00000355	-0.031751	0.804328	-0.42861	BGIOSGA020219	XP_002457198.1ATP-dependent 6-phosphofructokinase 6 isoform X1
TCONS_00008227	-0.0317261	1.2092	-0.291235	BGIOSGA017915	XP_006647406.1 probable bifunctional riboflavin biosynthesis protein RIBA 2, chloroplastic
TCONS_00009461	-0.0314633	0.784255	0.953025	#N/A	XP_008651787.3dentin sialophosphoprotein
TCONS_00025606	-0.0313931	-0.433223	-0.628926	BGIOSGA030921	XP_004958497.1probable carboxylesterase 18
TCONS_00011947	-0.0311391	-0.300087	-0.402411	BGIOSGA016081	XP_006649786.1PREDICTED: uncharacterized protein LOC102720498
TCONS_00023153	-0.0310973	-1.6443	-0.391797	BGIOSGA024789	XP_012699389.2putative FBD-associated F-box protein At5g38570
TCONS_00036320	-0.0309857	-0.00764347	1.05808	BGIOSGA037683	XP_015698508.1PREDICTED: uncharacterized protein LOC102721131
TCONS_00008914	-0.0309035	0.259064	0.164519	BGIOSGA005308	XP_006649093.1 serine/threonine-protein kinase At5g01020
TCONS_00020695	-0.0308456	-0.476292	-0.0442842	BGIOSGA027859	XP_015696195.1 bromodomain-containing factor 1
TCONS_00035963	-0.0306863	-0.116516	-1.72367	BGIOSGA037327	XP_010237282.1disease resistance protein RGA2 isoform X1
TCONS_00022644	-0.030631	-0.344219	0.531078	BGIOSGA028425	XP_006656289.2 probable sucrose-phosphate synthase 3 isoform X1
TCONS_00030154	-0.0305083	0.775316	0.0501302	BGIOSGA027941	XP_006660685.1 thylakoidal processing peptidase 1, chloroplastic-like
TCONS_00019538	-0.0304993	0.0602539	0.951766	BGIOSGA017822	XP_015692642.1 protein kri1
TCONS_00001714	-0.0303986	-2.59061	-1.68248	BGIOSGA028644	XP_006644632.1 copper transport protein ATX1
TCONS_00026770	-0.030313	0.337549	0.640266	BGIOSGA028833	XP_006659492.1 glycoprotein 3-alpha-L-fucosyltransferase A-like
TCONS_00001441	-0.0302215	1.16713	-0.273446	BGIOSGA020588	XP_006644417.1 DEAD-box ATP-dependent RNA helicase 2
TCONS_00015590	-0.0301269	-1.34048	-1.06012	BGIOSGA006823	XP_006653257.2 phototropin-2
TCONS_00028278	-0.0301095	-3.99118	-1.00258	BGIOSGA029543	XP_003574750.1beta-glucosidase 28
TCONS_00006751	-0.029792	-3.09811	-1.82791	BGIOSGA026692	XP_006647792.1PREDICTED: uncharacterized protein LOC102719458
TCONS_00019290	-0.0297281	-0.304291	-1.34362	BGIOSGA019864	XP_006654400.1 protein NRT1/ PTR FAMILY 3.1-like
TCONS_00032915	-0.0295391	-0.663233	-1.02878	BGIOSGA001923	XP_015698006.1 hydroxymethylglutaryl-CoA lyase, mitochondrial-like
TCONS_00034643	-0.0294304	-0.975622	-0.203391	BGIOSGA031219	XP_015697622.1 protein NLP2
TCONS_00033626	-0.0294006	0.513118	-0.134836	BGIOSGA034477	XP_015698167.1PREDICTED: uncharacterized protein At3g49140
TCONS_00036722	-0.0290192	0.249677	0.31216	BGIOSGA036594	XP_004977339.1NDR1/HIN1-like protein 10
TCONS_00009703	-0.0288184	-1.01566	0.121237	BGIOSGA006609	XP_006649764.1PREDICTED: uncharacterized protein LOC102713884
TCONS_00001008	-0.0285592	-0.700983	0.368222	BGIOSGA003610	XP_006644216.1 ell-associated factor Eaf
TCONS_00032191	-0.0283917	0.0205727	0.438571	BGIOSGA028247	NP_001150714.1catalytic/ oxidoreductase, acting on NADH or NADPH
TCONS_00016599	-0.0282697	0.94536	0.996878	BGIOSGA014365	XP_003580534.1pentatricopeptide repeat-containing protein At1g79490, mitochondrial
TCONS_00017810	-0.0281639	-0.6506	-0.0560561	BGIOSGA035405	XP_006655322.2 isopentenyl-diphosphate Delta-isomerase I-like
TCONS_00010355	-0.028094	0.718207	-0.227083	BGIOSGA012300	XP_015698680.1 mechanosensitive ion channel protein 2, chloroplastic-like isoform X3
TCONS_00005563	-0.02805	-0.314159	0.577412	BGIOSGA016968	XP_006646981.1 ubiquitin fusion degradation protein 1 homolog
TCONS_00024322	-0.0277571	-3.45633	-4.0078	BGIOSGA006521	XP_006658093.1 flowering-promoting factor 1-like protein 5
TCONS_00027381	-0.0277305	0.161072	-0.0588604	BGIOSGA025859	XP_010234378.1NAC domain-containing protein 14
TCONS_00013858	-0.0276823	-0.541445	-0.287606	BGIOSGA022725	XP_006652144.1 UDP-glycosyltransferase 92A1-like
TCONS_00002478	-0.0275877	-0.926305	0.571517	BGIOSGA023739	XP_010921761.1 potassium transporter 7 isoform X2
TCONS_00024365	-0.0274987	-1.34452	0.359097	BGIOSGA009037	XP_010233812.1uncharacterized protein LOC100825692 isoform X3
TCONS_00001890	-0.0274484	0.70732	-0.288423	BGIOSGA006653	XP_003569904.1protein PAT1 homolog 1
TCONS_00020272	-0.0273974	-0.585535	-1.12305	BGIOSGA005480	XP_003564157.1ethylene-responsive transcription factor 1 isoform X1
TCONS_00025617	-0.0273866	-2.25764	-1.00934	BGIOSGA012663	XP_015694750.1PREDICTED: uncharacterized protein LOC102709668
TCONS_00012059	-0.0272165	1.9355	0.0443482	BGIOSGA010949	XP_006649901.1 lipid phosphate phosphatase gamma, chloroplastic
TCONS_00006436	-0.0272045	-0.927334	0.678095	BGIOSGA008599	XP_006647488.1PREDICTED: uncharacterized protein LOC102702253
TCONS_00016544	-0.0270565	0.673013	1.11269	BGIOSGA014423	XP_015692332.1PREDICTED: uncharacterized protein LOC102705530
TCONS_00028716	-0.0270258	-0.691515	-1.04323	BGIOSGA012541	XP_015696865.1 protein PHR1-LIKE 1-like isoform X2
TCONS_00009213	-0.026953	0.557934	0.928717	BGIOSGA011036	XP_006650987.1 serine/threonine-protein kinase ATG1a
TCONS_00014702	-0.0268835	-2.3055	-0.724366	BGIOSGA011649	XP_006652605.1 deSI-like protein At4g17486
TCONS_00020417	-0.0267006	0.150335	0.76241	BGIOSGA022602	XP_006655952.1 tRNA (guanine-N(7)-)-methyltransferase
TCONS_00007186	-0.0266934	1.01745	0.143819	BGIOSGA007312	XP_006646756.1 E3 ubiquitin-protein ligase UPL3
TCONS_00023845	-0.0265931	0.343786	0.285312	BGIOSGA016405	XP_006657793.1 putative receptor-like protein kinase At4g00960
TCONS_00009271	-0.0265708	0.154207	0.659491	BGIOSGA011226	XP_010229238.1CSC1-like protein At4g35870
TCONS_00005017	-0.0264759	-0.00772653	0.267971	BGIOSGA000204	XP_006645277.1 long chain base biosynthesis protein 2d
TCONS_00037564	-0.026456	-0.237612	-0.46704	BGIOSGA035795	XP_006650311.2 anaphase-promoting complex subunit 5
TCONS_00023043	-0.0264111	0.755532	-0.54384	BGIOSGA021712	XP_006658245.1 protein PHLOEM PROTEIN 2-LIKE A10-like
TCONS_00009216	-0.0263959	-0.403942	-0.047852	BGIOSGA022835	XP_006649300.1 33 kDa ribonucleoprotein, chloroplastic-like

TCONS_00026884	-0.0261701	-0.472969	-0.00187413	BGIOSGA028941	XP_006659555.1 phosphoribosylglycinamide formyltransferase, chloroplastic isoform X1
TCONS_00007462	-0.026085	-0.127082	-0.102092	BGIOSGA007021	XP_006646971.2 autophagy-related protein 11
TCONS_00022869	-0.0257097	0.423019	0.214551	BGIOSGA028929	XP_015693824.1PREDICTED: uncharacterized protein LOC102708538 isoform X1
TCONS_00003859	-0.0256741	-0.690727	-0.33754	BGIOSGA001358	XP_006644305.1 DNA repair protein RAD51 homolog 3
TCONS_00005272	-0.0256587	0.526691	0.809283	BGIOSGA007380	XP_006648227.1 RNA-binding protein NOB1
TCONS_00002407	-0.0253543	1.28771	1.55152	BGIOSGA005046	XP_006645227.1 glycine-rich RNA-binding protein 2, mitochondrial-like
TCONS_00015624	-0.0252608	-2.61293	-1.25981	BGIOSGA006027	XP_004975276.17-deoxyloganetin glucosyltransferase
TCONS_00010922	-0.0251246	0.592826	-0.0715905	BGIOSGA029750	XP_006650561.1 deoxyhypusine synthase-like
TCONS_00014963	-0.0248939	0.127998	0.21183	BGIOSGA020482	XP_004976847.1serine/threonine-protein phosphatase 6 regulatory subunit 3 isoform X1
TCONS_00031439	-0.0247354	-0.694552	0.172701	BGIOSGA033253	XP_015697342.1 endonuclease MutS2
TCONS_00002757	-0.0246685	-2.38545	-1.37304	BGIOSGA002459	XP_003565292.1protein NEGATIVE REGULATOR OF RESISTANCE
TCONS_00000228	-0.0245988	0.254448	0.773607	BGIOSGA002807	XP_015694044.1 probable LRR receptor-like serine/threonine-protein kinase At3g47570
TCONS_00011837	-0.0244613	-0.520614	-0.95082	BGIOSGA011176	XP_006649680.1 squalene monooxygenase-like
TCONS_00031562	-0.0243199	-3.4294	-0.644288	BGIOSGA016191	XP_003581111.3chloroplast envelope quinone oxidoreductase homolog
TCONS_00004266	-0.024281	-0.882478	0.0298893	BGIOSGA013381	XP_004969709.1probable cytokinin riboside 5'-monophosphate phosphoribohydrolase LOGL1
TCONS_00007421	-0.0241716	0.373256	1.1119	BGIOSGA020425	XP_015689486.1 nuclear transcription factor Y subunit C-2
TCONS_00014295	-0.0240753	-0.340049	-0.410388	BGIOSGA008418	XP_015692320.1 ultraviolet-B receptor UVR8-like
TCONS_00037163	-0.0240593	-4.86795	-0.766113	BGIOSGA014777	XP_012699834.1 leucine-rich repeat extensin-like protein 3
TCONS_00018154	-0.0240585	-1.86105	-0.246837	BGIOSGA020208	XP_024314855.1F-box protein At5g49610 isoform X1
TCONS_00033288	-0.0240155	#NA	-1.95967	BGIOSGA028249	XP_006659958.1 endo-1,3;1,4-beta-D-glucanase-like
TCONS_00036313	-0.0238665	1.1979	-1.1451	BGIOSGA003839	XP_006664146.2 probable protein phosphatase 2C 78
TCONS_00032521	-0.0238292	0.548668	0.621578	BGIOSGA027817	XP_002464409.1probable ascorbate-specific transmembrane electron transporter 1
TCONS_00008738	-0.0236521	-0.28201	-0.320627	BGIOSGA007700	XP_006647831.1 protein FAM32A
TCONS_00026528	-0.0232738	1.25399	-0.821115	BGIOSGA030346	XP_003574419.1probable WRKY transcription factor 53
TCONS_00013146	-0.0232317	0.557275	-0.0311971	BGIOSGA013731	XP_021313661.1E3 ubiquitin protein ligase DRIP2
TCONS_00010947	-0.0230916	-1.8022	#NA	BGIOSGA021907	XP_015690064.1PREDICTED: uncharacterized protein LOC107303805
TCONS_00033588	-0.0230738	1.13826	0.915353	BGIOSGA033865	XP_006663487.1 FBD-associated F-box protein At1g66310-like
TCONS_00004011	-0.0229687	1.73599	1.484	BGIOSGA007013	XP_015694570.1 S-type anion channel SLAH2-like
TCONS_00004540	-0.0228562	0.555339	0.713514	BGIOSGA011577	XP_006644829.1 RNA-binding protein PNO1-like
TCONS_00014595	-0.022796	-0.802515	-1.64084	BGIOSGA006476	XP_015691894.1 dCTP pyrophosphatase 1-like
TCONS_00031453	-0.0224318	1.28988	1.28431	BGIOSGA016343	XP_003574177.1cytochrome P450 89A2
TCONS_00028183	-0.0224079	-0.205748	-0.305068	BGIOSGA029622	XP_015695641.1 ubiquitin carboxyl-terminal hydrolase 17-like
TCONS_00016918	-0.0223949	-0.554604	-0.721359	BGIOSGA000012	XP_006653062.1 soluble inorganic pyrophosphatase
TCONS_00020215	-0.0223901	-0.132912	-0.153193	BGIOSGA023242	XP_004964721.126S proteasome regulatory subunit 6A homolog
TCONS_00000620	-0.0223166	1.23693	0.170452	BGIOSGA031613	XP_008662633.1protein STABILIZED1
TCONS_00011859	-0.022217	-0.556467	-0.446588	BGIOSGA004903	XP_015690512.1 cyclin-J18-like isoform X1
TCONS_00003024	-0.0220544	-0.767435	0.37632	BGIOSGA014356	XP_006643875.1 casein kinase I homolog 2-like
TCONS_00001386	-0.021775	0.353493	0.262467	BGIOSGA011435	XP_003569389.1cell division control protein 48 homolog C
TCONS_00010238	-0.0216831	1.1112	0.569566	BGIOSGA012803	XP_006650166.2PREDICTED: uncharacterized protein LOC102713252
TCONS_00023603	-0.0216681	-0.521377	0.00698215	BGIOSGA025625	XP_006658525.1 exonuclease mut-7 homolog
TCONS_00011089	-0.0215296	0.647215	0.565634	BGIOSGA013689	XP_006650699.2 trafficking protein particle complex subunit 8 isoform X1
TCONS_00005647	-0.021379	-0.304262	1.42974	BGIOSGA001119	XP_004951502.1plastidic ATP/ADP-transporter
TCONS_00022169	-0.02135	-0.00016953	0.199846	BGIOSGA013899	NP_001150415.1thioredoxin reductase 2
TCONS_00019974	-0.0211691	0.99467	0.367609	BGIOSGA040185	XP_006656555.1 cell division control protein 48 homolog C
TCONS_00020782	-0.0211552	-0.0430829	0.0826315	BGIOSGA029799	XP_015693590.1PREDICTED: uncharacterized protein LOC102712369, partial
TCONS_00011641	-0.0211475	-0.769403	-0.39874	BGIOSGA015776	XP_006649471.1 protein kinase 2B, chloroplastic-like
TCONS_00027202	-0.0210277	-0.547732	-0.891477	BGIOSGA024011	XP_006659025.1 ferredoxin-1, chloroplastic
TCONS_00011718	-0.021027	-0.828626	0.172317	BGIOSGA011292	XP_003558604.1FRIGIDA-like protein 3
TCONS_00000306	-0.0208206	0.258052	0.494424	BGIOSGA015724	XP_006645559.1 ubiquitin carboxyl-terminal hydrolase 14 isoform X1
TCONS_00022994	-0.0207851	-1.21855	-0.131338	#N/A	#N/A
TCONS_00027336	-0.0207769	-0.01889	0.677816	BGIOSGA027668	XP_015695492.1 lysine-specific histone demethylase 1 homolog 2-like
TCONS_00017896	-0.0205398	0.301236	-0.209762	BGIOSGA019944	XP_006654478.1 solute carrier family 35 member F2-like
TCONS_00006931	-0.0204039	0.0249596	0.446219	BGIOSGA009111	XP_006647966.1PREDICTED: uncharacterized protein LOC102700120
TCONS_00037055	-0.0203116	1.75494	0.756507	BGIOSGA020576	XP_004962571.1serine hydroxymethyltransferase 4
TCONS_00008502	-0.0203088	-1.45718	0.207151	BGIOSGA006309	XP_004953297.1protein ORANGE, chloroplastic
TCONS_00018530	-0.0201151	0.0700262	-0.488823	BGIOSGA023627	XP_003563269.1NADH dehydrogenase [ubiquinone] 1 beta subcomplex subunit 2

TCONS_00015184	-0.0199462	0.278111	0.391934	BGIOSGA017366	XP_015691620.1 endonuclease MutS2
TCONS_00010765	-0.0197079	-0.758586	-0.315241	BGIOSGA013390	XP_006650441.2 F-box protein At1g55000
TCONS_00009481	-0.0196699	-0.672508	-0.113172	BGIOSGA036188	XP_004985376.1putative yippee-like protein Os10g0369500
TCONS_00020140	-0.0194589	0.0344931	0.681492	BGIOSGA022197	XP_006656655.2 F-box/LRR-repeat MAX2 homolog
TCONS_00029244	-0.0186382	-0.0345072	2.12472	BGIOSGA030987	XP_022680062.1 heat shock protein 81-1-like
TCONS_00006906	-0.018623	-0.284774	0.212754	BGIOSGA011314	XP_006647937.2 transport inhibitor response 1-like protein
TCONS_00017711	-0.0185387	-0.713153	-0.757794	BGIOSGA005604	NP_001141942.1annexin-like protein RJ4
TCONS_00015913	-0.0185357	0.742895	-0.214737	BGIOSGA006274	XP_015692355.1PREDICTED: uncharacterized protein LOC102714282 isoform X2
TCONS_00003361	-0.0185083	-1.50868	-0.455813	BGIOSGA001208	XP_015688307.1 3'-N-debenzoyl-2'-deoxytaxol N-benzoyltransferase-like
TCONS_00024387	-0.0184363	-0.485396	0.348102	BGIOSGA026429	XP_006665015.1 manganese-dependent ADP-ribose/CDP-alcohol diphosphatase
TCONS_00019221	-0.0182997	-0.458315	0.510503	BGIOSGA018127	XP_006654348.1 protein N-terminal glutamine amidohydrolase isoform X2
TCONS_00016125	-0.0179142	-1.06918	-0.550072	BGIOSGA021390	XP_006653538.1 very-long-chain 3-oxoacyl-CoA reductase 1-like
TCONS_00024121	-0.0178344	0.37324	1.26298	BGIOSGA026156	XP_015695304.1 homeobox-DDT domain protein RLT3
TCONS_00018160	-0.0177229	0.772784	0.563101	BGIOSGA002932	XP_006654672.1 ATP-dependent 6-phosphofructokinase 6-like
TCONS_00023096	-0.0177052	0.383396	-0.332269	BGIOSGA005684	XP_006652223.1 U4/U6 small nuclear ribonucleoprotein Prp31
TCONS_00016763	-0.0176105	0.154767	0.712047	BGIOSGA014201	XP_006652932.1 protein FATTY ACID EXPORT 7-like
TCONS_00027032	-0.0175003	-2.22524	-0.498209	BGIOSGA013958	XP_012702477.1disease resistance protein RPM1
TCONS_00023891	-0.0173982	-0.932564	-0.518289	BGIOSGA028499	XP_006657820.1 mitochondrial pyruvate carrier 4-like
TCONS_00009168	-0.0173672	-0.920039	-0.0796332	BGIOSGA007048	XP_006650434.1 valine--tRNA ligase, mitochondrial 1-like
TCONS_00000628	-0.0173619	0.740417	1.16656	BGIOSGA003211	XP_015688103.1 la-related protein 1A isoform X2
TCONS_00009299	-0.0171495	-0.875283	-0.453739	BGIOSGA006036	XP_015689761.1PREDICTED: uncharacterized protein LOC102706550
TCONS_00027804	-0.0169061	0.00342695	0.27877	BGIOSGA027196	XP_006655101.1 signal recognition particle subunit SRP68
TCONS_00024558	-0.0168431	0.618512	0.38442	BGIOSGA013864	XP_006658271.1 DEAD-box ATP-dependent RNA helicase 53
TCONS_00010934	-0.0166767	1.81554	1.10686	BGIOSGA017813	XP_006650569.1 calmodulin-like protein 4
TCONS_00028496	-0.01655	0.273133	0.561044	BGIOSGA026460	XP_010236392.1bifunctional TH2 protein, mitochondrial
TCONS_00005134	-0.0164824	0.0692938	-0.0482495	BGIOSGA000033	XP_006645358.1 E3 ubiquitin-protein ligase RGLG1-like
TCONS_00006388	-0.0164357	-0.200687	-0.293562	BGIOSGA008550	XP_006647461.2 myosin-3
TCONS_00007880	-0.0162705	0.072207	0.638405	BGIOSGA011284	XP_006647224.1 F-box/kelch-repeat protein OR23 isoform X1
TCONS_00010565	-0.0159388	0.0973327	0.25087	BGIOSGA013180	XP_003562225.1F-box/LRR-repeat protein 17 isoform X1
TCONS_00023975	-0.0158448	-1.10217	-1.19	BGIOSGA026006	XP_006657882.1 major facilitator superfamily domain-containing protein 12-like
TCONS_00023757	-0.0157762	-0.734501	-0.114634	BGIOSGA035423	XP_006663516.2PREDICTED: uncharacterized protein LOC102714870
TCONS_00032556	-0.0157228	-2.26718	-0.754284	BGIOSGA027994	NP_001105021.1aquaporin NIP3-1
TCONS_00010472	-0.0156991	-0.469586	-1.13601	BGIOSGA012862	XP_015691101.1 ACT domain-containing protein ACR4-like isoform X1
TCONS_00021418	-0.0155936	0.742084	-0.0946185	BGIOSGA010045	XP_006656523.1 protein argonaute 1D
TCONS_00014644	-0.0155521	-0.0248582	-0.39663	BGIOSGA004756	XP_006652549.1 ABC transporter G family member 11-like
TCONS_00012462	-0.015171	-0.0202579	0.342008	BGIOSGA033170	XP_006651508.1 vacuolar protein sorting-associated protein 52 A isoform X2
TCONS_00010276	-0.0150174	0.263959	0.630098	BGIOSGA023908	XP_006650180.1 elongation factor 1-delta 2
TCONS_00020126	-0.0149766	-0.230646	0.600555	BGIOSGA021023	XP_006655790.1 cysteine synthase-like isoform X1
TCONS_00013906	-0.0149471	0.159181	0.0491613	BGIOSGA016081	XP_015691394.1 protein root UVB sensitive 1, chloroplastic
TCONS_00018486	-0.0149456	0.0136635	-0.210342	BGIOSGA002861	XP_015693068.1 probable serine/threonine-protein kinase NAK
TCONS_00001168	-0.014819	-0.145578	0.634136	BGIOSGA014717	XP_004969013.1casein kinase I
TCONS_00010343	-0.0147437	0.0813581	0.134261	BGIOSGA012919	XP_006650215.1 probable ubiquitin conjugation factor E4
TCONS_00022582	-0.0146431	0.477868	0.290983	BGIOSGA020861	XP_015694418.1 cell division cycle protein 27 homolog B isoform X1
TCONS_00031422	-0.0144373	-4.00889	-2.40918	BGIOSGA033236	XP_015697386.1 cingulin-like
TCONS_00024428	-0.0142625	-1.18731	-0.507467	BGIOSGA010422	XP_003557706.1tobamovirus multiplication protein 1
TCONS_00020746	-0.0142276	0.105859	0.296237	BGIOSGA002436	XP_015694195.1 tRNA(adenine(34)) deaminase, chloroplastic
TCONS_00006032	-0.013896	-1.61667	0.279872	BGIOSGA034048	XP_010235427.1protein MICRORCHIDIA 7 isoform X2
TCONS_00003773	-0.0138075	0.0185199	-0.144476	BGIOSGA001433	XP_015699102.1PREDICTED: uncharacterized protein LOC102707069
TCONS_00006205	-0.0136816	-0.22082	-0.0987495	BGIOSGA008368	XP_004952660.2uncharacterized protein LOC101786880
TCONS_00031941	-0.0135976	-0.479011	0.779498	BGIOSGA005102	XP_015697454.1 transcription initiation factor IIF subunit alpha-like
TCONS_00014019	-0.0134654	-0.645637	0.418937	BGIOSGA016165	XP_006652188.1 cell division cycle 5-like protein
TCONS_00005124	-0.0134542	-0.190783	0.364561	BGIOSGA000080	XP_024312562.1uncharacterized protein LOC104581991
TCONS_00027179	-0.0133873	0.670158	0.205099	BGIOSGA012829	XP_006659013.1 B3 domain-containing protein IDEF1-like

TCONS_00006794	-0.0133423	-0.0389212	-1.25721	BGIOSGA025621	XP_006647832.1 E3 ubiquitin-protein ligase CIP8-like
TCONS_00002100	-0.0131004	-1.2145	-0.115606	BGIOSGA029525	XP_006646443.1 cinnamoyl-CoA reductase 1-like
TCONS_00035671	-0.0129165	-1.47425	-0.363908	BGIOSGA032924	XP_006663868.2 aldehyde dehydrogenase family 3 member H1-like
TCONS_00029118	-0.0128043	0.46589	0.144759	BGIOSGA030869	XP_015696767.1PREDICTED: uncharacterized protein LOC102702971 isoform X2
TCONS_00005772	-0.0127501	-1.33431	-0.0173924	BGIOSGA001487	XP_015688536.1 putative callose synthase 8
TCONS_00028008	-0.0124821	0.390717	0.23707	BGIOSGA026978	XP_015696022.1 EVI5-like protein
TCONS_00005009	-0.0124678	-1.57893	0.0443897	BGIOSGA000210	XP_006645270.2PREDICTED: uncharacterized protein LOC102713964
TCONS_00017732	-0.0124589	-1.90253	0.594748	BGIOSGA016726	XP_006654350.1 AP2-like ethylene-responsive transcription factor At2g41710
TCONS_00033447	-0.0124005	0.384314	-0.359093	BGIOSGA025185	XP_006663437.1 ubiquitin carboxyl-terminal hydrolase 5
TCONS_00013238	-0.0120469	0.302228	0.799218	BGIOSGA038953	XP_006650660.1 cytochrome c biogenesis protein CCS1, chloroplastic
TCONS_00022441	-0.0120145	-0.0769973	0.344437	BGIOSGA011333	XP_006649500.1 UDP-glycosyltransferase TURAN
TCONS_00010608	-0.0119688	-0.023002	0.194944	BGIOSGA010131	XP_006650361.1 CCR4-NOT transcription complex subunit 3 isoform X1
TCONS_00001827	-0.0119344	-5.53622	-0.597618	BGIOSGA027158	XP_015697864.1 cellulose synthase A catalytic subunit 4 [UDP-forming] isoform X2
TCONS_00029773	-0.0117143	0.489106	-0.430648	BGIOSGA035876	XP_006660510.1 ubiquitin-conjugating enzyme E2 variant 1C-like
TCONS_00020036	-0.0116715	-2.96626	-2.34709	BGIOSGA022217	XP_015693420.1 BAG family molecular chaperone regulator 3-like
TCONS_00030876	-0.0115562	-1.54682	0.316095	BGIOSGA037136	XP_006661684.1PREDICTED: uncharacterized protein At1g10890-like
TCONS_00025054	-0.0115117	-1.40055	0.00865216	BGIOSGA025649	XP_006657673.1 acyl-coenzyme A thioesterase 13-like
TCONS_00031489	-0.0113447	-0.132648	0.909801	BGIOSGA033301	XP_006662527.1 flavonol synthase/flavanone 3-hydroxylase-like
TCONS_00000682	-0.011082	-1.39468	-0.758183	BGIOSGA003269	XP_004967668.1F-box protein At2g26850
TCONS_00029337	-0.011028	0.516207	0.32351	BGIOSGA016671	XP_004957335.1histidine protein methyltransferase 1 homolog
TCONS_00001621	-0.0109979	-0.706306	-1.30717	BGIOSGA023551	XP_006646232.1 putative clathrin assembly protein At2g01600
TCONS_00010989	-0.0107982	0.820629	-0.666132	BGIOSGA011455	XP_015690890.1 UDP-glucose 6-dehydrogenase 3
TCONS_00017688	-0.0107291	-1.40898	-0.71106	BGIOSGA002233	XP_006654329.1 cytokinin dehydrogenase 9 isoform X1
TCONS_00028525	-0.0106347	0.178495	-0.0170801	BGIOSGA030278	XP_024957980.1sodium/proton antiporter 1 isoform X2
TCONS_00021015	-0.0106144	0.157325	-1.8511	BGIOSGA034025	XP_006656232.1 fructose-bisphosphate aldolase, cytoplasmic isozyme 1-like
TCONS_00030375	-0.0106118	-2.23176	-0.186259	BGIOSGA016385	XP_006660854.1 protein LITTLE ZIPPER 2-like
TCONS_00031412	-0.0106106	0.41707	1.01286	BGIOSGA027116	XP_015697143.1 pentatricopeptide repeat-containing protein At2g20710, mitochondrial-like
TCONS_00001019	-0.0105845	-0.418319	0.0128154	BGIOSGA028482	XP_006644210.1 protein ENHANCED DOWNY MILDEW 2-like
TCONS_00012624	-0.0104595	0.547833	0.973539	BGIOSGA010349	XP_006650304.1 arabinosyltransferase XEG113
TCONS_00011471	-0.010361	0.631467	0.505597	BGIOSGA002691	XP_006649296.1 50S ribosomal protein L11
TCONS_00001996	-0.0101664	-0.191831	0.613554	BGIOSGA003541	XP_006644856.1 4-diphosphocytidyl-2-C-methyl-D-erythritol kinase, chloroplastic
TCONS_00000488	-0.0100652	-1.11721	-0.500078	BGIOSGA003064	XP_015697550.1 acyl-coenzyme A thioesterase 13-like isoform X3
TCONS_00021527	-0.00998065	-0.0796784	0.67788	BGIOSGA005707	XP_015694198.1PREDICTED: uncharacterized protein LOC102702856 isoform X3
TCONS_00002333	-0.00996713	-3.3651	-1.79608	BGIOSGA014525	XP_021312966.1protein IQ-DOMAIN 14
TCONS_00020338	-0.00980025	0.220379	-0.180372	BGIOSGA029299	XP_002436684.1mitochondrial phosphate carrier protein 3, mitochondrial
TCONS_00001612	-0.00973552	0.34427	0.148896	BGIOSGA027108	XP_003569601.1uncharacterized protein LOC100824080
TCONS_00015185	-0.00966167	0.901306	2.47618	BGIOSGA034926	XP_006653888.1 MLO-like protein 14
TCONS_00013025	-0.00960661	-1.57973	-3.25849	BGIOSGA009964	XP_006650462.1PREDICTED: uncharacterized protein LOC102704500 isoform X1
TCONS_00011675	-0.00949693	-0.125318	0.289969	BGIOSGA021019	XP_006649500.1 UDP-glycosyltransferase TURAN
TCONS_00001519	-0.00949507	-0.666125	-0.189062	BGIOSGA000351	XP_015695030.1 putative exosome complex component rrp40
TCONS_00000980	-0.00943978	0.50373	0.910335	BGIOSGA001607	XP_004968801.1protein DMP3
TCONS_00004500	-0.0093804	-0.0954074	0.00849889	BGIOSGA036998	XP_006646385.1 purple acid phosphatase 2
TCONS_00019131	-0.00933706	-0.164978	-0.0891057	BGIOSGA015886	XP_004962376.1protein trichome birefringence-like 28
TCONS_00007276	-0.0092777	0.24049	0.395356	BGIOSGA007207	XP_015688440.1 RNA polymerase II transcription factor B subunit 4
TCONS_00004550	-0.00907224	-1.96287	0.474794	BGIOSGA020116	XP_006644836.1PREDICTED: uncharacterized protein LOC102706984
TCONS_00014359	-0.00905915	-0.180283	0.149216	BGIOSGA008474	XP_015692202.1 cryptochrome-1 isoform X1
TCONS_00023379	-0.00894882	0.54388	1.13714	BGIOSGA013821	XP_010228213.1amino acid transporter ANT1
TCONS_00008908	-0.00803594	-0.208541	-1.00007	BGIOSGA023919	XP_006647991.2 nuclear transcription factor Y subunit A-4-like
TCONS_00013729	-0.00803594	0.501952	-0.192711	#N/A	#N/A
TCONS_00022072	-0.00786039	0.476698	0.705468	BGIOSGA014137	XP_006665104.1 phosphatidylinositol 4-kinase gamma 5-like
TCONS_00031001	-0.00783076	0.273456	0.431413	BGIOSGA005081	XP_006662301.1 calmodulin-binding transcription activator 3-like
TCONS_00004602	-0.00777398	-0.51023	-0.558225	BGIOSGA001903	XP_008451087.1 60S ribosomal protein L30
TCONS_00017848	-0.00776902	0.898717	1.00253	BGIOSGA036861	XP_006655343.1 protein trichome berefringence-like 7
TCONS_00013321	-0.00776551	1.31858	0.661509	BGIOSGA006954	XP_006651898.1 zinc finger A20 and AN1 domain-containing stress-associated protein 7-like

transcriptome

TCONS_00010658	-0.00772941	1.68214	0.169094	BGIOSGA019279	XP_020408369.1putative disease resistance RPP13-like protein 3
TCONS_00029340	-0.00771827	0.527981	0.389616	BGIOSGA031088	XP_006660844.1 transcription factor 25
TCONS_00013227	-0.00767006	0.0781448	0.596268	BGIOSGA009759	XP_015689841.1 transmembrane protein 147
TCONS_00035499	-0.00760634	-2.5133	-3.47665	BGIOSGA034717	XP_006662695.1PREDICTED: uncharacterized protein LOC102706327
TCONS_00037562	-0.00757212	-0.345209	-1.51068	BGIOSGA007809	XP_006664222.1 cysteine synthase
TCONS_00004861	-0.0074053	0.436522	1.09037	BGIOSGA019009	XP_015699178.1 probable phosphoribosylformylglycinamidine synthase, chloroplastic/mitochondrial
TCONS_00024151	-0.00737584	0.705148	0.358885	BGIOSGA026189	XP_006657987.1PREDICTED: uncharacterized protein LOC102720617
TCONS_00004584	-0.00731109	0.563772	0.457557	BGIOSGA000629	XP_006644873.1 rhomboid-like protein 20
TCONS_00023164	-0.00727353	-0.691043	0.0106183	BGIOSGA031389	XP_003572096.1mediator of RNA polymerase II transcription subunit 31
TCONS_00035639	-0.00722426	0.23393	-0.426866	BGIOSGA019609	XP_015698722.1 long chain acyl-CoA synthetase 9, chloroplastic isoform X1
TCONS_00036173	-0.00718153	-0.683552	-0.324125	BGIOSGA037530	XP_008662016.1riboflavin synthase alpha chain isoform X1
TCONS_00003837	-0.00715467	0.203757	0.519728	BGIOSGA005794	XP_006646018.2 IQ domain-containing protein IQM1
TCONS_00033240	-0.00712815	-0.204269	1.25385	BGIOSGA029101	XP_015697615.1 la-related protein 6B-like
TCONS_00019584	-0.00708734	-3.40614	-1.00243	BGIOSGA034564	XP_006654606.1 NAD(P)H dehydrogenase (quinone) FQR1-like
TCONS_00013322	-0.00699559	-1.04368	-0.562087	BGIOSGA011785	XP_006654944.1 probable glucuronosyltransferase Os05g0123100
TCONS_00016086	-0.00697846	-1.83433	-0.328868	BGIOSGA026304	XP_006652406.1 beta-glucosidase 13
TCONS_00002928	-0.00685996	-0.364306	-0.36879	BGIOSGA002294	XP_006645542.2 dol-P-Man:Man(5)GlcNAc(2)-PP-Dol alpha-1,3-mannosyltransferase
TCONS_00011847	-0.00684783	-0.475702	-0.0722906	BGIOSGA011162	XP_004987091.1mucin-7 isoform X1
TCONS_00028853	-0.00680933	-0.334212	-0.488551	BGIOSGA028627	XP_015696755.1PREDICTED: uncharacterized protein LOC102720628
TCONS_00033628	-0.00672147	0.13747	0.219943	BGIOSGA020099	XP_006662993.1 dr1-associated corepressor-like
TCONS_00022004	-0.0066775	-0.576083	0.545337	BGIOSGA021513	XP_015694178.1 THO complex subunit 4D isoform X2
TCONS_00014014	-0.00666492	-1.79184	-1.30197	BGIOSGA016161	XP_006652185.2 protein PRD1
TCONS_00026932	-0.00646546	0.134451	0.758521	BGIOSGA035356	XP_015695743.1 geranylgeranyl transferase type-2 subunit beta-like
TCONS_00032483	-0.00640527	0.129202	0.598796	BGIOSGA031634	XP_006662467.1 protein TIC110, chloroplastic
TCONS_00022632	-0.00628986	0.450838	0.387902	BGIOSGA020810	XP_015693890.1 elongator complex protein 4
TCONS_00020758	-0.00616663	-0.107531	-0.383375	BGIOSGA009251	XP_006656103.2 phosphate transporter PHO1-3-like
TCONS_00035411	-0.0060916	-0.638172	-1.72914	BGIOSGA033512	XP_015697867.1 ribulose bisphosphate carboxylase/oxygenase activase, chloroplastic
TCONS_00001578	-0.0060671	-0.138446	1.28455	BGIOSGA004210	XP_006646205.2 protein SDE2 homolog
TCONS_00009190	-0.00588791	-1.85673	-1.14573	BGIOSGA005701	XP_006649275.1 ARM REPEAT PROTEIN INTERACTING WITH ABF2
TCONS_00011932	-0.00585512	0.0131638	0.717682	BGIOSGA009792	XP_006649766.2 imidazole glycerol phosphate synthase hisHF, chloroplastic isoform X2
TCONS_00035486	-0.00577388	-1.14866	0.101155	BGIOSGA036899	XP_006662683.1PREDICTED: uncharacterized protein LOC102702887
TCONS_00002051	-0.00563798	-0.00535118	0.522483	BGIOSGA025407	XP_015688326.1 zinc finger protein VAR3, chloroplastic
TCONS_00004126	-0.00535488	-0.363928	-2.42178	BGIOSGA008700	XP_015689505.1 probable WRKY transcription factor 71
TCONS_00036672	-0.00532712	0.785557	0.18766	BGIOSGA001898	XP_006663816.1 pyruvate kinase 1, cytosolic
TCONS_00033739	-0.00523172	0.587131	0.476718	BGIOSGA012399	XP_006663004.2PREDICTED: uncharacterized protein LOC102711922
TCONS_00008515	-0.00521198	-0.282636	-0.0761014	BGIOSGA005913	XP_015689479.1 putative lipase YOR059C
TCONS_00014090	-0.00496153	0.254148	0.051181	BGIOSGA016248	XP_006652127.1 probable sodium/metabolite cotransporter BASS1, chloroplastic
TCONS_00037948	-0.00487361	1.78901	-0.649376	BGIOSGA005576	XP_006663677.1 wall-associated receptor kinase 5-like
TCONS_00016698	-0.00482853	2.00605	0.264976	BGIOSGA001936	XP_002448611.1UBP1-associated protein 2B
TCONS_00022457	-0.00479124	0.490677	0.517371	BGIOSGA007805	XP_015694101.1 elongation factor 1-gamma 3
TCONS_00017938	-0.00471725	-0.210372	0.0390071	BGIOSGA003812	XP_015692619.1 ATP-dependent zinc metalloprotease FTSH 8, mitochondrial
TCONS_00015226	-0.00464659	0.504511	-0.045312	BGIOSGA020949	XP_006653078.1 30S ribosomal protein S17, chloroplastic
TCONS_00033929	-0.0042743	-1.71855	-3.4208	BGIOSGA008289	XP_015697731.1 myosin-7-like
TCONS_00025955	-0.00427345	0.00181254	-0.669122	BGIOSGA026513	XP_006659860.1 transmembrane 9 superfamily member 2-like
TCONS_00037045	-0.00407583	-0.190762	-0.366049	BGIOSGA004918	XP_015698843.1PREDICTED: uncharacterized protein LOC102703739
TCONS_00021705	-0.00383464	0.0505007	0.956273	BGIOSGA021818	XP_006656691.1 fanconi-associated nuclease 1 homolog
TCONS_00018013	-0.00383205	0.0739206	-0.608185	BGIOSGA020061	XP_003626648.1NADH dehydrogenase [ubiquinone] 1 alpha subcomplex subunit 1
TCONS_00012651	-0.00382333	-0.576391	-1.17189	BGIOSGA019463	XP_006650296.2PREDICTED: uncharacterized protein LOC102703851 isoform X2
TCONS_00015942	-0.0036533	1.14413	0.716971	BGIOSGA015041	XP_006652316.1 regulator of nonsense transcripts UPF3 isoform X2
TCONS_00017098	-0.00359694	-0.387279	0.38218	BGIOSGA020375	XP_006654022.1 aspartic proteinase
TCONS_00009450	-0.00352098	0.0718933	1.1404	BGIOSGA011999	XP_015690392.1 putative dihydroflavonol-4-reductase
TCONS_00006108	-0.0035185	-4.41063	-4.01128	BGIOSGA019196	XP_003571092.1isoflavone 2'-hydroxylase
TCONS_00034316	-0.00343645	-0.710294	-2.42652	BGIOSGA037225	XP_006664468.1 pre-mRNA-splicing factor CWC22 homolog
TCONS_00029341	-0.00319954	0.55535	0.454679	BGIOSGA031089	XP_006660845.1 ATPase family AAA domain-containing protein At1g05910
TCONS_00021425	-0.00316215	-0.589203	-0.236692	BGIOSGA023650	XP_006656527.1 transmembrane protein 53-like
TCONS_00029676	-0.00307444	-1.1569	-2.36718	BGIOSGA020912	XP_006660469.1 SEC14 cytosolic factor-like

TCONS_00028616	-0.00304575	0.742064	0.686565	BGIOSGA030365	XP_015696490.1 exosome complex component RRP41 homolog isoform X3
TCONS_00011122	-0.0029869	-0.282015	0.379723	BGIOSGA014096	XP_006650721.1 pentatricopeptide repeat-containing protein At2g02980, chloroplastic
TCONS_00004369	-0.00292585	-0.107101	0.177731	BGIOSGA004368	XP_015699141.1 hexokinase-6
TCONS_00019956	-0.00274419	0.343285	0.51037	BGIOSGA035757	XP_015693831.1 protein arginine N-methyltransferase 7 isoform X1
TCONS_00016858	-0.00268838	-0.541322	-1.04225	BGIOSGA023855	XP_002448757.1small glutamine-rich tetratricopeptide repeat-containing protein 2
TCONS_00005469	-0.00220206	0.167208	0.559384	BGIOSGA007579	XP_003574897.2putative FBD-associated F-box protein At5g56390
TCONS_00015173	-0.002168	-1.41233	-2.01461	BGIOSGA029759	XP_004960064.1WD repeat-containing protein 44
TCONS_00013507	-0.00206841	-1.37882	-0.54802	BGIOSGA010394	XP_022679114.1wall-associated receptor kinase 2
TCONS_00013546	-0.00189969	1.10319	0.514702	BGIOSGA009429	XP_002463510.1elongation factor Tu, mitochondrial
TCONS_00018379	-0.00168383	-0.641556	-0.373938	BGIOSGA001160	XP_006654840.2 auxilin-like protein 1
TCONS_00019875	-0.00155464	-0.696887	-0.268886	BGIOSGA038132	XP_003576022.3putative disease resistance protein RGA3
TCONS_00018658	-0.00150194	0.5064	-0.0344205	BGIOSGA006264	XP_006654072.1 protein disulfide isomerase-like 2-1
TCONS_00034220	-0.00141678	0.675629	0.592431	BGIOSGA036703	XP_006663176.1 actin cytoskeleton-regulatory complex protein pan1-like
TCONS_00007233	-0.00128527	-0.661136	0.479938	BGIOSGA007255	XP_006646779.1 F-box/kelch-repeat protein SKIP4
TCONS_00018147	-0.00123487	-0.975969	0.28667	BGIOSGA002182	XP_015693148.1 mitogen-activated protein kinase kinase kinase 13-A-like
TCONS_00000322	-0.00106397	0.437255	0.385556	BGIOSGA002897	XP_006643839.2PREDICTED: uncharacterized protein LOC102717287 isoform X2
TCONS_00011710	-0.0010044	-0.189308	-0.0559875	BGIOSGA035144	XP_006649537.1PREDICTED: uncharacterized protein LOC102720124
TCONS_00004432	-0.000901371	-4.4862	-0.490162	BGIOSGA002848	XP_006646341.1PREDICTED: uncharacterized protein LOC102718327
TCONS_00022724	-0.000729097	-0.132074	0.0353728	BGIOSGA020728	XP_003577500.1probable Xaa-Pro aminopeptidase P
TCONS_00007426	-0.000602723	0.929482	0.487328	BGIOSGA007051	XP_006647411.1 F-box/kelch-repeat protein At1g22040-like
TCONS_00037140	-0.000234566	0.834379	0.112012	BGIOSGA036234	XP_006664540.2 iron-sulfur cluster co-chaperone protein HscB, mitochondrial
TCONS_00028547	-0.000153597	0.999479	0.49052	BGIOSGA009236	XP_004964736.1ribonucleoside-diphosphate reductase large subunit
TCONS_00005039	8.22606E-06	0.837626	0.130559	BGIOSGA004092	XP_004971179.1autophagy-related protein 18a
TCONS_00018817	0.00017305	0.718361	-0.506843	BGIOSGA018600	XP_015693365.1 mannosyl-oligosaccharide 1,2-alpha-mannosidase MNS3
TCONS_00015748	0.000311492	0.216869	0.59471	BGIOSGA015230	XP_015692084.1 eukaryotic translation initiation factor 3 subunit H-like isoform X1
TCONS_00004203	0.000479438	0.370069	0.243324	BGIOSGA020321	XP_003567006.2ras/Rap GTPase-activating protein SynGAP
TCONS_00023870	0.000479725	-0.0388338	-1.00521	BGIOSGA011174	XP_006657807.1 chitin-inducible gibberellin-responsive protein 1
TCONS_00008280	0.000773412	-2.55832	-1.81292	BGIOSGA006148	XP_004956139.1lecithin-cholesterol acyltransferase-like 1
TCONS_00006366	0.000866042	-1.65009	-2.84196	BGIOSGA008528	XP_006647444.1 inorganic phosphate transporter 2-1, chloroplastic
TCONS_00005209	0.000953728	-1.68115	-1.0798	BGIOSGA014366	XP_003570757.1NDR1/HIN1-like protein 26
TCONS_00026212	0.00102912	-0.58171	-3.07999	BGIOSGA032723	XP_006659244.1 probable LRR receptor-like serine/threonine-protein kinase At3g47570
TCONS_00014385	0.00145273	-0.964827	-0.830316	BGIOSGA010100	XP_006652369.2 protease Do-like 8, chloroplastic
TCONS_00034293	0.00166994	-0.40453	0.284459	BGIOSGA036643	XP_006664333.1 long chain acyl-CoA synthetase 6, peroxisomal-like
TCONS_00020811	0.00170084	-0.0958334	0.0388636	BGIOSGA006745	XP_006656129.1PREDICTED: uncharacterized protein LOC102709850
TCONS_00016627	0.00170305	-0.772728	-0.369721	BGIOSGA014336	XP_015691639.1 gamma-soluble NSF attachment protein
TCONS_00006265	0.00191394	-0.934557	0.262365	BGIOSGA016459	XP_006648736.1PREDICTED: uncharacterized protein LOC102721810
TCONS_00031670	0.00192812	-0.743111	-0.43831	BGIOSGA033491	XP_004983570.1uncharacterized protein At2g38710
TCONS_00008159	0.00216248	0.583431	0.0184645	BGIOSGA037527	XP_021310923.1coleoptile phototropism protein 1
TCONS_00008453	0.0021718	0.554354	-0.060495	BGIOSGA026163	XP_006647575.1 putative GEM-like protein 8
TCONS_00013487	0.00219511	0.342039	-0.300695	BGIOSGA009504	XP_006650854.1 squamosa promoter-binding-like protein 6
TCONS_00008457	0.00223191	0.15374	-0.382574	BGIOSGA005970	XP_006648871.1PREDICTED: uncharacterized protein LOC102720772 isoform X1
TCONS_00011129	0.00224172	-0.366847	0.764894	BGIOSGA006236	XP_004981425.1transcription factor UNE12
TCONS_00017881	0.00235168	0.0891316	0.307642	BGIOSGA006726	XP_006654458.1 protein LIKE COV 2-like
TCONS_00018012	0.00255578	-1.07949	0.181657	BGIOSGA000591	XP_006654550.1 probable LRR receptor-like serine/threonine-protein kinase At1g06840
TCONS_00027976	0.00263961	0.697093	-0.0900597	BGIOSGA014488	XP_015696021.1 ABC transporter A family member 2
TCONS_00006395	0.0026436	0.243057	0.435392	BGIOSGA008552	XP_015689345.1 telomere length regulation protein TEL2 homolog
TCONS_00006230	0.00281549	0.132129	0.204861	BGIOSGA008392	XP_006648718.1 WD repeat domain-containing protein 83
TCONS_00029788	0.00293405	0.375342	0.270854	BGIOSGA025431	XP_015696294.1 probable NAD(P)H dehydrogenase subunit CRR3, chloroplastic
TCONS_00003051	0.00296018	-0.173782	-1.75114	BGIOSGA023409	XP_004968566.1protein GRAVITROPIC IN THE LIGHT 1
TCONS_00003286	0.0030122	0.349806	-0.126712	BGIOSGA021271	XP_006649997.1 calmodulin
TCONS_00025449	0.00309402	0.214598	0.819228	BGIOSGA023980	XP_006657905.1 FRIGIDA-like protein 3
TCONS_00017878	0.00343722	0.297841	0.448075	BGIOSGA006601	XP_015692420.1 signal peptide peptidase 2-like
TCONS_00019207	0.00353164	-0.561163	-0.00838811	BGIOSGA018141	XP_006655269.2 AP-3 complex subunit mu

TCONS_00036425	0.00386794	-2.28655	-0.443813	BGIOSGA003109	XP_024312685.1wall-associated receptor kinase 5 isoform X1
TCONS_00024284	0.00416118	-1.0368	-0.916671	BGIOSGA026328	XP_006658074.1 4-alpha-glucanotransferase DPE2
TCONS_00017415	0.00424028	-1.8874	-0.0808275	BGIOSGA028165	XP_021321454.1probable LRR receptor-like serine/threonine-protein kinase At1g56130
TCONS_00017117	0.00425386	-0.649022	-0.761398	BGIOSGA001130	XP_006654030.1 mitogen-activated protein kinase 14
TCONS_00032391	0.00428375	0.777638	0.248567	BGIOSGA031732	XP_015697177.1 54S ribosomal protein L19, mitochondrial
TCONS_00036519	0.00428981	0.678564	1.0737	BGIOSGA005293	XP_008662745.1NEDD8-specific protease 1
TCONS_00003184	0.00436513	-1.04184	-0.797335	BGIOSGA019463	XP_004967339.1triose phosphate/phosphate translocator, chloroplastic
TCONS_00010703	0.00445109	0.865124	0.875147	BGIOSGA013327	XP_006650406.1 phosphomethylpyrimidine synthase, chloroplastic isoform X1
TCONS_00010284	0.00445271	-2.43447	-1.3029	#N/A	#N/A
TCONS_00007128	0.00458435	-0.275372	0.071848	BGIOSGA009312	XP_006648141.1 proline-rich receptor-like protein kinase PERK14
TCONS_00018643	0.0046251	0.450789	0.395839	BGIOSGA033114	XP_006655000.1 histidine--tRNA ligase, cytoplasmic
TCONS_00032589	0.00462578	0.144244	-0.215811	BGIOSGA031526	XP_006661973.1 post-GPI attachment to proteins factor 3-like
TCONS_00000459	0.00466147	-0.0337589	0.30323	BGIOSGA002717	XP_006645654.1 mitochondrial carrier protein MTM1-like
TCONS_00024148	0.0051153	0.383266	-0.1547	BGIOSGA026185	XP_015695220.1 E3 ubiquitin-protein ligase RKP
TCONS_00027045	0.00523736	0.0805611	0.0237647	#N/A	#N/A
TCONS_00009248	0.00526193	0.70131	0.00547993	BGIOSGA026900	XP_006649341.1 4-coumarate--CoA ligase-like 4
TCONS_00025612	0.00540047	0.472513	1.46092	BGIOSGA004230	XP_006658042.1 pre-mRNA-splicing factor SYF1
TCONS_00015233	0.00551449	0.0790626	0.729292	BGIOSGA017419	XP_006653923.2 farnesylcysteine lyase isoform X1
TCONS_00022860	0.00553435	1.01815	-1.4018	BGIOSGA020585	XP_003560515.1cation transporter HKT1
TCONS_00023338	0.00566578	1.18312	0.609079	BGIOSGA025379	XP_006657553.1 dolichyl-diphosphooligosaccharide--protein glycosyltransferase 48 kDa subunit
TCONS_00031638	0.00583228	-0.473602	0.0662507	BGIOSGA033454	XP_006662610.1 RNA-binding KH domain-containing protein PEPPER
TCONS_00015499	0.00598404	-0.300029	-1.94686	BGIOSGA016280	XP_006652131.1 heavy metal-associated isoprenylated plant protein 26-like
TCONS_00020371	0.00609019	-0.224936	0.114285	BGIOSGA021098	XP_006656789.1 5'-adenylylsulfate reductase-like 2
TCONS_00000443	0.00626305	-0.564485	-0.448325	BGIOSGA024948	XP_006643913.1 vesicle transport protein SFT2B
TCONS_00032604	0.00627799	-1.76649	-0.795684	BGIOSGA005168	XP_006661987.1 probable glutathione S-transferase GSTU6
TCONS_00019389	0.00639249	0.225381	0.275438	BGIOSGA016362	XP_004961931.1protein transport protein sec24
TCONS_00027286	0.00648323	-0.200537	0.253903	BGIOSGA000054	XP_006659086.1 actin-related protein 3-like
TCONS_00006758	0.00688551	0.827101	0.434616	BGIOSGA008928	XP_006648992.2 probable glucosamine 6-phosphate N-acetyltransferase 2
TCONS_00021572	0.00692212	1.28532	0.428855	BGIOSGA023979	XP_006655770.1PREDICTED: uncharacterized protein LOC102722126
TCONS_00002446	0.00711101	0.221719	0.295375	BGIOSGA005089	XP_006646631.1 delta(3,5)-Delta(2,4)-dienoyl-CoA isomerase, peroxisomal
TCONS_00016949	0.00731571	0.383996	0.776153	BGIOSGA003286	XP_004960938.1photosynthetic NDH subunit of lumenal location 5, chloroplastic
TCONS_00001449	0.00752714	0.479843	0.429287	BGIOSGA007662	XP_004969283.1HD domain-containing protein 2 homolog
TCONS_00030302	0.00752855	-3.4968	0.225719	BGIOSGA029506	XP_003578366.1protein MANNAN SYNTHESIS-RELATED 1 isoform X1
TCONS_00024886	0.00753803	-0.312696	0.659345	BGIOSGA015647	XP_015696506.1 protein argonaute 16
TCONS_00008768	0.00755717	0.394438	0.597732	BGIOSGA023562	XP_006649025.1 E3 ubiquitin protein ligase RIE1-like
TCONS_00017250	0.00766149	0.483891	0.59853	BGIOSGA016798	XP_006654117.1PREDICTED: uncharacterized protein LOC102713358
TCONS_00000309	0.00767643	-0.92958	-0.342537	BGIOSGA002884	XP_015696859.1 transmembrane protein 19
TCONS_00016195	0.00780711	0.678634	0.325789	BGIOSGA006056	XP_020401239.1enolase-phosphatase E1
TCONS_00012516	0.00788403	0.317872	-0.604756	BGIOSGA012067	XP_006650237.1 probable esterase D14L
TCONS_00003827	0.00789194	-0.780875	0.25264	BGIOSGA001388	XP_006644259.1PREDICTED: uncharacterized protein LOC102701212
TCONS_00027994	0.00791144	-0.177937	-1.4351	BGIOSGA022213	NP_001140351.1uncharacterized protein LOC100272399
TCONS_00024053	0.00816407	1.73121	0.946613	BGIOSGA026089	XP_004958238.1pentatricopeptide repeat-containing protein PNM1, mitochondrial
TCONS_00022898	0.00820747	-0.503816	0.612532	BGIOSGA008494	XP_006656482.1 MADS-box transcription factor 16
TCONS_00009350	0.00826699	0.833848	-0.0779771	BGIOSGA010658	XP_003558775.1phosphoserine aminotransferase 1, chloroplastic
TCONS_00008849	0.00838214	-0.108051	-0.579107	BGIOSGA004369	XP_015688940.1 putative 4-hydroxy-4-methyl-2-oxoglutarate aldolase 2
TCONS_00030763	0.00853175	-0.487937	-0.19745	BGIOSGA003728	XP_021306844.1disease resistance protein RPM1
TCONS_00008924	0.00872522	-0.596013	0.302013	BGIOSGA021098	XP_006648011.1PREDICTED: uncharacterized protein LOC102712504 isoform X1
TCONS_00004393	0.00895731	-0.306705	-0.943982	BGIOSGA004967	XP_006644703.1 serine/threonine-protein kinase TNNI3K-like
TCONS_00001498	0.00912264	-0.139461	-0.385635	BGIOSGA035444	XP_004969353.1long chain acyl-CoA synthetase 4 isoform X2
TCONS_00006856	0.00918598	0.553845	0.314806	BGIOSGA021603	XP_015689295.1 serine/threonine-protein kinase CTR1-like
TCONS_00009690	0.00920576	-0.311343	-0.678742	BGIOSGA017806	XP_003558346.1UDP-glucuronate 4-epimerase 3
TCONS_00001489	0.00923793	0.265241	0.458737	BGIOSGA011422	XP_004981468.1uncharacterized protein LOC101783491

TCONS_00013252	0.00935917	-1.44553	-1.37541	BGIOSGA009736	XP_006650668.1 5'-nucleotidase domain-containing protein 4 isoform X1
TCONS_00028982	0.00936201	-0.694683	-0.611646	BGIOSGA030741	XP_006661218.2 transcription initiation factor TFIID subunit 2 isoform X1
TCONS_00034672	0.00937067	-0.731129	-1.26924	BGIOSGA022950	XP_021302975.1probable serine/threonine-protein kinase PBL28
TCONS_00016762	0.00949458	0.969894	1.11307	BGIOSGA014202	XP_004960253.1peptidyl-tRNA hydrolase 2, mitochondrial
TCONS_00008229	0.00952589	0.892356	0.879986	BGIOSGA008410	XP_006648771.1 protein indeterminate-domain 13-like
TCONS_00011240	0.00954077	-0.377572	0.429381	BGIOSGA032009	XP_006650809.2 protein kinase APK1A, chloroplastic-like isoform X2
TCONS_00018825	0.00978108	0.495662	0.775447	BGIOSGA018593	XP_015693362.1 LRR receptor-like serine/threonine-protein kinase EFR
TCONS_00006738	0.00981395	-1.13523	-1.26521	BGIOSGA007345	XP_006648979.1PREDICTED: uncharacterized protein At1g26090, chloroplastic
TCONS_00022978	0.00994827	-3.56891	0.0118275	BGIOSGA034398	XP_006658206.2 dirigent protein 1-like
TCONS_00023785	0.00999547	-0.157236	0.13777	BGIOSGA013261	XP_006657768.1 protein LHCP TRANSLOCATION DEFECT
TCONS_00036232	0.0100926	0.0615822	1.15112	BGIOSGA037591	XP_004962771.1uncharacterized protein LOC101771063
TCONS_00024410	0.0103295	-0.242781	0.456278	BGIOSGA026449	XP_015695164.1 symplekin isoform X2
TCONS_00013173	0.0105189	0.984911	0.961594	BGIOSGA009811	XP_003559631.1protein NDH-DEPENDENT CYCLIC ELECTRON FLOW 5
TCONS_00025152	0.0106842	-0.306953	-0.487187	BGIOSGA024258	XP_021309726.1DSC E3 ubiquitin ligase complex subunit 1-like
TCONS_00017990	0.0107057	1.21574	0.193637	BGIOSGA012615	XP_006654538.2 protein RETICULATA-RELATED 5, chloroplastic-like
TCONS_00031840	0.0107579	0.107988	0.27674	BGIOSGA021723	XP_006662182.1 F-box/LRR-repeat protein 4-like
TCONS_00018708	0.0107752	0.445371	1.09928	BGIOSGA032637	XP_004962938.3F-box protein At5g07610
TCONS_00029933	0.0108636	-0.288858	0.397883	BGIOSGA029877	XP_015696473.1 putative F-box protein At4g21240
TCONS_00007044	0.0109531	1.28288	0.846071	BGIOSGA009228	XP_003570332.1protein diaphanous homolog 1
TCONS_00025172	0.0112424	0.953772	0.784472	BGIOSGA014371	XP_015695363.1 probable potassium transporter 14
TCONS_00009151	0.0112484	0.772829	-0.00880879	BGIOSGA006054	XP_004986049.1probable glucuronosyltransferase Os03g0107900
TCONS_00007114	0.0112786	-0.99664	0.659416	BGIOSGA038246	XP_006648122.1PREDICTED: uncharacterized protein Ytol-like isoform X2
TCONS_00013381	0.011289	0.429422	1.45323	BGIOSGA009604	XP_006650763.1PREDICTED: uncharacterized protein LOC102721533
TCONS_00021591	0.0114802	0.283272	0.443579	BGIOSGA022076	XP_006646013.2 protein EARLY FLOWERING 3-like isoform X1
TCONS_00010070	0.0116452	0.620578	0.266041	BGIOSGA023751	XP_002467862.1zinc finger protein CONSTANS-LIKE 13
TCONS_00001338	0.0116551	0.30895	-0.0143003	BGIOSGA003970	NP_001149665.1vacuolar ATP synthase 21 kDa proteolipid subunit
TCONS_00002011	0.0117032	0.0580939	0.736702	BGIOSGA004641	XP_006644868.1 psbP domain-containing protein 6, chloroplastic isoform X1
TCONS_00005889	0.0120367	-0.226249	0.289562	BGIOSGA037567	XP_006647192.1 eukaryotic translation initiation factor 1A-like
TCONS_00036503	0.0122258	0.185415	0.592116	BGIOSGA037872	XP_006664245.1 protein NUCLEAR FUSION DEFECTIVE 4
TCONS_00036215	0.0122448	-1.37913	0.386315	BGIOSGA030851	XP_002459541.1probable galactinol--sucrose galactosyltransferase 2
TCONS_00012853	0.0122456	-1.05356	-0.31105	BGIOSGA010120	XP_015690389.1 protein disulfide-isomerase LQY1, chloroplastic
TCONS_00010731	0.012307	2.02956	1.57491	BGIOSGA038809	XP_006651701.1 heterogeneous nuclear ribonucleoprotein A1, A2/B1 homolog
TCONS_00024668	0.0123517	-1.28743	0.722868	BGIOSGA009645	XP_006657502.1PREDICTED: uncharacterized protein LOC102722972
TCONS_00006619	0.0123826	0.608969	0.548478	BGIOSGA020414	XP_006647666.1 amino acid transporter ANTL1-like
TCONS_00034678	0.012498	-1.18452	-0.671273	BGIOSGA034152	XP_006662900.2 ATP-dependent (S)-NAD(P)H-hydrate dehydratase
TCONS_00007585	0.012715	-0.737783	0.263106	BGIOSGA034758	XP_015689065.1 caffeoylshikimate esterase-like
TCONS_00009725	0.012845	-0.372319	-0.266254	BGIOSGA029299	XP_004984704.1mitochondrial phosphate carrier protein 3, mitochondrial
TCONS_00010229	0.013168	-0.285961	0.123891	BGIOSGA023897	XP_015690206.1 ADP-ribosylation factor-related protein 1-like
TCONS_00029211	0.013225	1.59691	1.15559	BGIOSGA027972	XP_021309231.1pentatricopeptide repeat-containing protein At3g62890
TCONS_00026757	0.0133557	1.0903	0.642485	BGIOSGA030881	XP_015695633.1 mediator-associated protein 1-like
TCONS_00008657	0.0133568	-1.98145	-3.65783	BGIOSGA013424	XP_015689029.1 pheophytinase, chloroplastic
TCONS_00004572	0.0133882	-0.838656	0.146448	BGIOSGA005137	XP_015688170.1 probable glycerol-3-phosphate dehydrogenase [NAD(+)] 2, cytosolic isoform X1
TCONS_00019612	0.0134424	-1.11386	-1.16689	BGIOSGA023596	XP_006654626.1 general transcription factor IIF subunit 2-like
TCONS_00025331	0.0134486	-1.01001	-3.14471	BGIOSGA024098	XP_015694685.1 putative PAP-specific phosphatase, mitochondrial
TCONS_00025699	0.0134555	-0.743291	-0.307145	BGIOSGA005715	XP_024315124.1tyrosine--tRNA ligase 1, cytoplasmic isoform X1
TCONS_00006920	0.0135892	-0.308054	0.0504382	BGIOSGA009104	XP_015688744.1 galactoside 2-alpha-L-fucosyltransferase-like
TCONS_00001812	0.0137725	0.197003	0.334737	BGIOSGA004448	XP_015688182.1 nuclear pore complex protein NUP85
TCONS_00004154	0.0139281	0.0530064	0.0165898	BGIOSGA022076	XP_015699143.1 callose synthase 12-like
TCONS_00025559	0.0141759	-0.114788	-0.846794	BGIOSGA009047	XP_006657988.1 sphinganine C4-monooxygenase 1-like isoform X2
TCONS_00014799	0.0142005	-0.736594	0.0610347	BGIOSGA008813	XP_006652676.1 rho GTPase-activating protein 5-like
TCONS_00023582	0.0144608	-0.736921	0.0252021	BGIOSGA025558	XP_010230263.1F-box/FBD/LRR-repeat protein At1g13570

TCONS_00017012	0.0145827	0.183701	0.092039	BGIOSGA019058	XP_006654921.2 UDP-galactose/UDP-glucose transporter 5
TCONS_00029526	0.0146037	1.06909	0.0001347	BGIOSGA033775	XP_006661554.1 delta(14)-sterol reductase
TCONS_00027150	0.0146578	-0.172951	-2.261	BGIOSGA010019	XP_006659703.1 protein DMR6-LIKE OXYGENASE 1
TCONS_00010011	0.0148297	0.424868	0.0985471	BGIOSGA016565	XP_003557976.1receptor-like protein kinase FERONIA
TCONS_00005464	0.0148425	-0.0192422	-0.425509	BGIOSGA020204	XP_006646904.1PREDICTED: uncharacterized protein LOC102716841
TCONS_00011900	0.0151291	-0.480292	-0.32214	BGIOSGA022770	XP_006649742.1 autophagy-related protein 9-like
TCONS_00007222	0.01517	1.40118	0.689538	BGIOSGA007269	XP_006646772.2 pentatricopeptide repeat-containing protein At2g37230
TCONS_00003166	0.0153239	0.365944	-0.533885	BGIOSGA016842	XP_006643963.1 exportin-2
TCONS_00033174	0.0154743	-1.40594	0.471894	BGIOSGA034298	XP_015697973.1 serine/threonine-protein kinase TAO2-like
TCONS_00031968	0.0156336	-0.0833557	-0.0852442	BGIOSGA003551	XP_006662254.1 probable E3 ubiquitin-protein ligase LUL2
TCONS_00035735	0.0156347	-1.18345	-2.95715	BGIOSGA025191	XP_021321592.1zinc-finger homeodomain protein 3
TCONS_00025121	0.015649	-0.77906	-3.64197	BGIOSGA007428	XP_022680328.1monooxygenase 3-like
TCONS_00020759	0.0160114	-1.34037	-0.334286	BGIOSGA022957	XP_015693423.1 paired amphipathic helix protein Sin3-like 2
TCONS_00030367	0.016071	0.668871	0.204988	BGIOSGA040472	XP_006660849.1 acyl-coenzyme A thioesterase 9, mitochondrial
TCONS_00025532	0.0163879	0.0981742	0.401884	BGIOSGA030815	XP_006657960.1 alanine aminotransferase 2-like isoform X1
TCONS_00002270	0.0164545	-1.5771	0.632954	BGIOSGA000267	XP_015690541.1 dnaJ protein homolog 1-like
TCONS_00002890	0.0165499	-1.64545	-1.07981	BGIOSGA002328	XP_006645527.2 probable protein phosphatase 2C 1
TCONS_00036258	0.0165552	0.842354	0.479889	BGIOSGA010932	XP_006664116.1 importin subunit beta-1
TCONS_00021213	0.0166148	0.862968	0.461576	BGIOSGA027463	XP_006656351.1 nuclear transcription factor Y subunit C-2-like
TCONS_00024419	0.0166425	1.06652	0.995057	BGIOSGA038100	XP_015695217.1 telomeric repeat-binding factor 2-like
TCONS_00001597	0.0168194	2.03715	0.632047	BGIOSGA004233	XP_006644558.1 methionyl-tRNA formyltransferase
TCONS_00036109	0.0168194	0.329331	-0.660735	BGIOSGA012053	XP_006664586.1 protein CLT2, chloroplastic
TCONS_00031457	0.0168393	0.830161	0.320342	BGIOSGA025623	XP_004982612.1cytochrome P450 89A2
TCONS_00015106	0.016873	-0.185417	0.341704	BGIOSGA028463	XP_006660775.1 protein EMSY-LIKE 3-like isoform X1
TCONS_00006716	0.0169237	-0.160284	0.149878	BGIOSGA008885	XP_006647758.1 lipid phosphate phosphatase epsilon 1, chloroplastic-like
TCONS_00026983	0.0169257	-0.643906	1.47062	BGIOSGA029032	XP_015695814.1 ankyrin repeat and SAM domain-containing protein 6-like
TCONS_00013341	0.0169373	0.430258	0.749079	BGIOSGA009643	XP_006650731.1 light-inducible protein CPRF2-like
TCONS_00013135	0.0169453	-2.69265	0.246881	BGIOSGA014799	XP_006650552.1 endoglucanase 10
TCONS_00008489	0.0171823	-1.1995	-1.41067	BGIOSGA002861	XP_006647607.1 probable receptor-like protein kinase At4g10390
TCONS_00031312	0.0172459	0.76245	0.358762	BGIOSGA006974	XP_006661862.1 probable serine/threonine protein phosphatase 2A regulatory subunit B''delta
TCONS_00033610	0.0173804	0.00794248	0.376867	BGIOSGA037972	XP_008676643.1citrate synthase isoform X1
TCONS_00002232	0.0174003	-0.533897	0.408213	BGIOSGA019905	XP_004953713.1tyrosine--tRNA ligase 1, cytoplasmic
TCONS_00026556	0.0174032	0.193953	-0.0827949	BGIOSGA031747	XP_006659381.1 hypersensitive-induced response protein 1-like
TCONS_00008992	0.0175504	-0.0976514	0.175481	BGIOSGA005245	XP_006648068.1 vacuolar protein sorting-associated protein 55 homolog
TCONS_00035743	0.0175694	-2.35611	0.595421	BGIOSGA037154	XP_006663902.1 2-dehydro-3-deoxyphosphooctonate aldolase 1
TCONS_00010183	0.017634	1.84768	1.59526	BGIOSGA020069	XP_004984217.1probable apyrase 6
TCONS_00027807	0.0176732	0.549146	-0.456846	BGIOSGA023222	XP_006659301.1 rubredoxin
TCONS_00004529	0.0177126	-0.408759	-0.0900609	BGIOSGA029777	XP_006644815.1 transcription factor bHLH123-like
TCONS_00034651	0.017728	-1.25678	-0.303093	BGIOSGA034178	XP_006662911.1PREDICTED: uncharacterized protein LOC102706508
TCONS_00025169	0.0177514	0.792834	0.768186	BGIOSGA031778	XP_015695241.1 pre-mRNA-splicing factor ATP-dependent RNA helicase DEAH7
TCONS_00009061	0.0178915	-0.790413	0.771992	BGIOSGA005343	XP_015688929.1PREDICTED: uncharacterized protein LOC102722654
TCONS_00009100	0.017921	-0.46525	-0.282451	BGIOSGA013699	XP_015689290.1 protein argonaute 1C isoform X2
TCONS_00004224	0.0180502	1.03328	1.01543	BGIOSGA000980	XP_002458353.1threonine synthase, chloroplastic
TCONS_00027432	0.018077	-0.0571638	-0.0268323	BGIOSGA010867	XP_006659172.1 AP2-like ethylene-responsive transcription factor PLT2
TCONS_00024071	0.0181097	-0.493895	-1.8245	BGIOSGA022835	XP_002460999.1uncharacterized protein LOC8060668
TCONS_00018731	0.0181151	-0.247976	-0.156721	BGIOSGA027214	XP_006654109.2 phosphatidylinositol 3-kinase, root isoform
TCONS_00032675	0.0182997	-1.87898	-0.93105	BGIOSGA025033	XP_004983701.1flavin-containing monooxygenase FMO GS-OX-like 2
TCONS_00002871	0.0183825	-0.444367	-0.147334	BGIOSGA018753	XP_006643778.1 E3 ubiquitin-protein ligase MBR2-like
TCONS_00024943	0.0185008	0.0411465	0.234537	BGIOSGA022030	XP_006658485.1 endoplasmic reticulum metallopeptidase 1 isoform X1
TCONS_00013606	0.0186414	0.037387	0.0539746	BGIOSGA015760	XP_006652085.1 arginase 1, mitochondrial
TCONS_00030232	0.0186614	-0.260513	0.23314	BGIOSGA004722	XP_022680444.1probable WRKY transcription factor 2
TCONS_00008728	0.018763	0.829039	0.0235483	BGIOSGA005705	NP_001132348.1uncharacterized LOC100193791
TCONS_00019234	0.0187907	0.511622	0.126397	BGIOSGA018114	XP_006655283.1 E3 ubiquitin-protein ligase KEG

TCONS_00028989	0.0188104	-0.204065	0.15865	BGIOSGA006187	XP_003578110.1uncharacterized protein LOC100834851
TCONS_00000223	0.0188762	-0.614363	0.477808	BGIOSGA014973	XP_006645503.2PREDICTED: uncharacterized protein LOC102717201
TCONS_00017508	0.0188853	-0.615263	-0.119314	BGIOSGA019581	XP_006654245.1 transcription factor IIIB 60 kDa subunit-like isoform X2
TCONS_00007003	0.0189301	-0.504927	0.297511	BGIOSGA027337	XP_015689599.1 probable E3 ubiquitin-protein ligase XBOS32 isoform X2
TCONS_00001227	0.0189497	-0.804731	-0.469103	BGIOSGA003855	XP_010231954.1nitrate regulatory gene2 protein isoform X1
TCONS_00014649	0.0189913	-0.91497	0.073031	BGIOSGA037014	XP_006652554.1 syntaxin-81
TCONS_00012533	0.0192452	0.453599	-0.15475	BGIOSGA010448	XP_006650245.1 30S ribosomal protein S5
TCONS_00015300	0.0194234	0.373362	0.725136	BGIOSGA002841	XP_006653121.2 receptor-like protein 2
TCONS_00003104	0.0194322	0.204881	0.276083	BGIOSGA004136	XP_006643912.1 histone-lysine N-methyltransferase ATX5-like
TCONS_00028742	0.0195002	0.302857	-0.0554839	BGIOSGA030494	XP_015696545.1 peroxisome biogenesis protein 3-1-like
TCONS_00016176	0.0196771	0.896093	0.960295	BGIOSGA037172	XP_006652471.1 2-alkenal reductase (NADP(+)-dependent)-like
TCONS_00018200	0.0197402	-0.55235	0.271362	BGIOSGA017662	XP_006654704.1 tRNA(His) guanylyltransferase 1-like isoform X2
TCONS_00025400	0.0197829	1.20355	0.0515401	BGIOSGA024027	XP_003562833.1bet1-like protein At4g14600
TCONS_00024949	0.0198797	-1.16637	-1.44591	BGIOSGA000207	XP_015695303.1 aspartokinase 2, chloroplastic-like isoform X2
TCONS_00003284	0.0200068	-0.530129	0.72915	BGIOSGA001928	XP_015693298.1 cell number regulator 5
TCONS_00005105	0.020066	0.322222	0.0421883	BGIOSGA018133	XP_004971285.1PH, RCC1 and FYVE domains-containing protein 1
TCONS_00001818	0.0200884	-0.0318997	0.175219	BGIOSGA004455	XP_015688233.1 MACPF domain-containing protein CAD1
TCONS_00025548	0.0202082	-0.000513813	-0.456274	BGIOSGA014982	XP_006657979.1PREDICTED: uncharacterized protein LOC102718470
TCONS_00023160	0.0203008	0.109633	0.506956	BGIOSGA000898	XP_015695395.1 valine--tRNA ligase, chloroplastic/mitochondrial 2 isoform X1
TCONS_00000463	0.0204524	-0.277667	0.0279584	BGIOSGA003038	XP_006643931.2PREDICTED: uncharacterized protein LOC102721964
TCONS_00015091	0.0204542	-0.0288245	0.425947	BGIOSGA011068	XP_006652939.1 protein KAKU4
TCONS_00022401	0.0204838	0.260659	0.582495	BGIOSGA010692	XP_006643887.1 60S ribosomal protein L11
TCONS_00017322	0.0205177	-0.530886	-0.186964	BGIOSGA035199	XP_015692511.1 mitochondrial dicarboxylate/tricarboxylate transporter DTC-like
TCONS_00008077	0.0207345	-0.0608314	-1.06951	BGIOSGA013671	XP_006648672.1 WAS/WASL-interacting protein family member 3-like
TCONS_00032448	0.0208123	1.7968	1.48725	BGIOSGA031671	XP_004982826.1uncharacterized protein LOC101780267
TCONS_00015621	0.0208165	0.435943	0.569357	BGIOSGA012430	XP_006653269.2 pentatricopeptide repeat-containing protein At1g60770
TCONS_00006682	0.0208469	-0.75567	-0.380136	BGIOSGA036524	XP_006648952.2 E3 ubiquitin-protein ligase RNF14-like isoform X1
TCONS_00026321	0.0209633	-0.582765	-0.641228	BGIOSGA028404	XP_014758889.1putative disease resistance protein RGA3 isoform X2
TCONS_00018541	0.0209849	-0.124626	0.223389	BGIOSGA003052	XP_006654948.2PREDICTED: uncharacterized protein LOC102700148, partial
TCONS_00023631	0.0212018	-0.205893	-0.969899	BGIOSGA012037	XP_006658539.1 ABC transporter B family member 28
TCONS_00026604	0.0212625	-1.44001	0.18399	BGIOSGA028665	XP_015695601.1PREDICTED: uncharacterized protein LOC102718764
TCONS_00013389	0.0214576	0.627795	0.108486	BGIOSGA009595	XP_015690687.1 probable protein phosphatase 2C 55
TCONS_00011513	0.0217687	0.514795	1.54849	BGIOSGA007411	XP_015690163.1 putative HVA22-like protein g
TCONS_00023437	0.021798	-0.465828	-0.487366	#N/A	#N/A
TCONS_00005425	0.0218185	-0.534504	0.0179831	BGIOSGA000187	XP_006646880.1 DNA-binding protein BIN4
TCONS_00007374	0.0220038	-2.12902	-0.973315	BGIOSGA023514	XP_015689441.1 tyrosine-sulfated glycopeptide receptor 1-like
TCONS_00002653	0.0223414	-0.900714	-0.586072	BGIOSGA030202	XP_010912729.1 cinnamoyl-CoA reductase 1
TCONS_00027339	0.0223429	0.501237	0.289065	BGIOSGA019219	XP_006644248.1PREDICTED: uncharacterized protein LOC102721967 isoform X1
TCONS_00005110	0.0226394	-0.687552	-1.31031	BGIOSGA029369	XP_004971298.1uncharacterized protein LOC101786750
TCONS_00016389	0.0227429	0.0540057	-0.129997	BGIOSGA025702	XP_015692404.1PREDICTED: LOW QUALITY PROTEIN: uncharacterized protein LOC102716588
TCONS_00018445	0.0228657	0.331102	-0.130396	BGIOSGA018451	XP_006653928.1PREDICTED: uncharacterized protein At4g28440-like
TCONS_00025117	0.0229027	0.706258	-0.0749641	BGIOSGA024285	XP_006657716.1 COP9 signalosome complex subunit 7 isoform X2
TCONS_00007526	0.023149	-0.55456	-1.74065	BGIOSGA013393	XP_006648416.1 lipoxygenase 2.3, chloroplastic-like
TCONS_00005269	0.0231876	0.399648	-0.081669	BGIOSGA004552	XP_002439211.1uncharacterized protein LOC8071260
TCONS_00024741	0.0232386	0.250622	0.0205835	BGIOSGA024634	XP_006657547.1 probable Xaa-Pro aminopeptidase P
TCONS_00012085	0.023282	-0.245482	-0.638237	BGIOSGA010925	XP_006649925.1 gamma-interferon-inducible lysosomal thiol reductase-like isoform X2
TCONS_00001711	0.023356	0.19647	0.320375	#N/A	#N/A
TCONS_00035874	0.0234106	-1.03491	0.115613	BGIOSGA036065	XP_021321913.1uncharacterized protein LOC8074247
TCONS_00007746	0.023507	2.04916	1.13701	BGIOSGA006736	XP_006648524.2 U1 small nuclear ribonucleoprotein C-1
TCONS_00016041	0.0235904	0.238496	-0.0496321	BGIOSGA031211	XP_006653495.1 FBD-associated F-box protein At1g66310-like
TCONS_00030458	0.0241115	-0.0842951	0.658022	BGIOSGA029343	NP_001145190.2uncharacterized LOC100278440
TCONS_00031080	0.0241592	-0.812571	0.111197	#N/A	#N/A
TCONS_00009821	0.024255	0.767788	0.970958	BGIOSGA023662	XP_006649890.1 L-ascorbate peroxidase 1, cytosolic
TCONS_00013979	0.0244644	-0.173803	-0.469366	BGIOSGA013945	XP_006652174.1 FACT complex subunit SPT16

TCONS_00016093	0.0246743	1.47748	-0.181131	BGIOSGA006134	XP_006652412.2 protein NBR1 homolog
TCONS_00005256	0.024726	-0.358872	0.609857	BGIOSGA007369	XP_015689330.1 GPI mannosyltransferase 3
TCONS_00024404	0.0247617	-1.3058	-0.764811	BGIOSGA017574	XP_015695175.1 probable LRR receptor-like serine/threonine-protein kinase MRH1 isoform X2
TCONS_00009754	0.0248028	-0.653698	-0.249636	BGIOSGA023075	XP_006664426.2 aquaporin NIP3-2-like
TCONS_00009624	0.0249988	-0.341796	-0.130458	BGIOSGA012166	XP_002465601.1uncharacterized protein LOC8085242
TCONS_00009047	0.0250317	-0.377036	-0.335379	BGIOSGA012326	XP_006648121.1 telomere-binding protein 1
TCONS_00016572	0.0250705	-1.3104	-1.2323	BGIOSGA001448	XP_015688595.1 phenolic glucoside malonyltransferase 1-like
TCONS_00003657	0.0258564	-0.255377	0.20039	BGIOSGA001547	XP_006644218.1 transcription initiation factor TFIID subunit 6
TCONS_00022550	0.0260212	0.0603819	-1.84665	BGIOSGA004313	XP_006644607.1 glycine dehydrogenase (decarboxylating) 2, mitochondrial
TCONS_00015884	0.0261158	0.381484	0.751861	BGIOSGA015102	XP_002466218.1uncharacterized protein LOC8057075
TCONS_00007356	0.0263372	-2.25361	-1.89704	BGIOSGA016278	XP_006648310.1 endoglucanase 5
TCONS_00032204	0.0266862	0.658799	0.334411	BGIOSGA011933	XP_022678934.1carbon catabolite repressor protein 4 homolog 1
TCONS_00037403	0.0266988	1.15127	-0.00642311	BGIOSGA010279	XP_015698749.1 asparagine synthetase domain-containing protein 1-like
TCONS_00000773	0.0267014	-1.83959	-0.78422	BGIOSGA008017	XP_006645815.2 putative disease resistance RPP13-like protein 3
TCONS_00028061	0.0267326	-0.12488	0.327604	BGIOSGA001194	XP_006660154.1 polypyrimidine tract-binding protein homolog 1-like
TCONS_00010398	0.026776	0.663582	1.01964	BGIOSGA037226	XP_006650257.1 elongation factor G, mitochondrial
TCONS_00014119	0.0267774	0.0281556	-0.258514	BGIOSGA012779	XP_006652212.1 calmodulin-binding transcription activator 4
TCONS_00009681	0.0268454	-1.61137	0.499387	#N/A	#N/A
TCONS_00016072	0.0270212	-0.719876	-1.42344	BGIOSGA014908	XP_004975926.1WPP domain-interacting protein 1 isoform X3
TCONS_00012421	0.0270212	-1.28378	-0.0290999	BGIOSGA026243	XP_006658888.1 trafficking protein particle complex subunit 3
TCONS_00034534	0.0271124	-2.21418	-1.87638	BGIOSGA027872	XP_004979103.1NAD(P)H-quinone oxidoreductase subunit T, chloroplastic
TCONS_00007117	0.0271856	-0.54425	0.0802583	BGIOSGA029467	XP_006648124.1 probable protein S-acyltransferase 15
TCONS_00029766	0.0272318	-1.54757	0.221804	BGIOSGA030678	XP_015696286.1 bifunctional aspartokinase/homoserine dehydrogenase 2, chloroplastic
TCONS_00033085	0.027255	-0.238334	-0.857725	BGIOSGA034929	XP_006662784.1 short-chain dehydrogenase TIC 32, chloroplastic-like
TCONS_00007096	0.0273519	0.986944	0.51662	BGIOSGA024774	XP_006648100.1 glutathionyl-hydroquinone reductase YqjG
TCONS_00026958	0.0273596	0.218144	1.13196	#N/A	#N/A
TCONS_00011270	0.0274563	-0.226658	0.679734	BGIOSGA013874	XP_006650846.1 zinc finger matrin-type protein 2
TCONS_00007033	0.027773	0.240236	-0.517447	BGIOSGA023197	XP_006648060.1 probable protein phosphatase 2C 27
TCONS_00024442	0.0278363	0.202902	-0.783568	BGIOSGA024963	XP_015695121.1 glutamate--glyoxylate aminotransferase 2-like
TCONS_00032707	0.0279275	-0.00659875	0.117405	BGIOSGA029278	XP_006662063.1 putative dual specificity protein phosphatase DSP8
TCONS_00021748	0.0280651	0.45309	0.59814	BGIOSGA019284	XP_015694122.1 hydrophobic protein OSR8-like
TCONS_00037488	0.0282754	-0.0601125	0.564174	BGIOSGA017384	XP_006664176.1 ubiquitin-conjugating enzyme E2 variant 1C-like
TCONS_00031693	0.0282857	0.0378384	0.698035	BGIOSGA037905	XP_006662645.1 ankyrin repeat domain-containing protein 29
TCONS_00003283	0.0284644	-0.0805202	1.97901	BGIOSGA001929	XP_006644034.1PREDICTED: uncharacterized protein LOC102703908
TCONS_00009178	0.0284923	-0.423062	0.0336034	BGIOSGA031001	XP_006649260.1 serine/threonine-protein kinase OSR1-like isoform X1
TCONS_00028299	0.0286903	-0.142542	-0.827984	BGIOSGA037594	XP_008678366.1cyclin-dependent protein kinase inhibitor SMR1
TCONS_00018455	0.0287875	0.0993472	0.579089	BGIOSGA011842	XP_003569146.1translocon-associated protein subunit beta
TCONS_00025233	0.0288153	1.53107	1.52177	BGIOSGA001410	XP_006657780.1 isocitrate lyase
TCONS_00016948	0.0288685	0.13483	-0.252832	BGIOSGA002562	XP_006653935.1 charged multivesicular body protein 5-like
TCONS_00024061	0.0289528	-0.928847	-2.88038	BGIOSGA006672	XP_006657934.1 MADS-box transcription factor 18
TCONS_00008561	0.0291769	-0.85185	-1.67552	BGIOSGA016645	XP_015688118.1 pentatricopeptide repeat-containing protein At1g06140, mitochondrial-like
TCONS_00032490	0.029193	0.133361	0.240232	BGIOSGA004568	XP_015697397.1 katanin p80 WD40 repeat-containing subunit B1 homolog isoform X1
TCONS_00002968	0.0292014	-0.696445	0.220385	#N/A	#N/A
TCONS_00007264	0.0294711	0.96487	-0.243556	BGIOSGA007218	XP_002453222.1uncharacterized protein LOC8055043
TCONS_00025655	0.029573	-0.381139	0.382197	BGIOSGA018258	XP_004958592.1protein EARLY RESPONSIVE TO DEHYDRATION 15
TCONS_00036755	0.0296727	0.183732	-0.72968	BGIOSGA036560	XP_006663853.1 RNA polymerase II subunit A C-terminal domain phosphatase SSU72
TCONS_00031696	0.0296791	0.231822	-2.83453	BGIOSGA018401	XP_006657377.1 probable pyridoxal 5'-phosphate synthase subunit PDX1.1
TCONS_00016907	0.0297604	0.217026	0.399301	BGIOSGA014057	XP_006653902.1 exocyst complex component EXO70A1-like
TCONS_00027035	0.0298309	0.212509	0.738676	BGIOSGA016670	XP_006659646.1 probable E3 ubiquitin-protein ligase ARI8
TCONS_00002790	0.0298514	0.0836516	0.366516	BGIOSGA007576	XP_006643734.1 protein SUPPRESSOR OF K(+) TRANSPORT GROWTH DEFECT 1
TCONS_00016813	0.029853	-0.681471	0.456951	BGIOSGA014152	XP_006652974.1 cell division protein FtsZ homolog 1, chloroplastic
TCONS_00017293	0.0299605	-2.13856	-3.31537	BGIOSGA034240	XP_003566442.2lachrymatory-factor synthase

TCONS_00011571	0.0302116	1.42441	0.842318	BGIOSGA026279	XP_003558805.1uncharacterized protein LOC100825549
TCONS_00009349	0.0302235	-0.428685	0.215368	BGIOSGA031852	XP_006649429.1 protein RRP6-like 2 isoform X1
TCONS_00034174	0.0302338	0.44606	-0.316054	BGIOSGA000583	XP_006664281.1 probable WRKY transcription factor 54
TCONS_00027382	0.0302447	2.03417	0.199196	BGIOSGA022861	XP_004972796.1protein NTM1-like 9
TCONS_00006414	0.0307888	0.186826	0.333235	BGIOSGA008578	XP_006647486.1PREDICTED: uncharacterized protein LOC102701695
TCONS_00022059	0.0309097	-0.70718	-0.373974	BGIOSGA035797	XP_018506047.1PREDICTED: uncharacterized protein LOC103959960
TCONS_00010034	0.030958	0.588295	1.12899	BGIOSGA012587	XP_003566470.1protein pleiotropic regulatory locus 1
TCONS_00032454	0.0310408	-0.0798121	0.168531	BGIOSGA031663	XP_006661876.1 pre-mRNA-processing protein 40C isoform X2
TCONS_00011942	0.031048	-0.825023	0.250193	BGIOSGA011071	XP_004984943.1putative metallophosphoesterase At3g03305
TCONS_00008794	0.0311326	0.451529	0.640133	BGIOSGA005640	XP_004953885.1protease Do-like 9
TCONS_00026141	0.0311693	0.0887296	-0.47947	BGIOSGA034595	XP_006659222.1 ethanolamine-phosphate cytidylyltransferase-like
TCONS_00021556	0.0311856	-1.56767	-0.0353857	BGIOSGA017194	XP_022681616.1uncharacterized protein LOC101753518
TCONS_00011638	0.0313298	-0.230918	0.229802	BGIOSGA032908	XP_006649468.1PREDICTED: uncharacterized protein LOC102700034
TCONS_00028437	0.0313643	0.819026	1.70348	BGIOSGA002691	XP_015695776.1 myb-related protein Zm38-like
TCONS_00009145	0.0316126	-1.02782	-0.59602	BGIOSGA031467	XP_006649238.1 sporulation-specific protein 15-like
TCONS_00027350	0.0316128	-0.563097	-0.643177	BGIOSGA027648	XP_006659116.1 protein ENHANCED DISEASE RESISTANCE 2-like
TCONS_00034240	0.0317169	0.895083	0.561157	BGIOSGA036678	XP_006663183.1 glutamate-rich WD repeat-containing protein 1-like
TCONS_00034159	0.031766	0.564758	0.45075	BGIOSGA012580	XP_010237951.1uncharacterized protein LOC100826374
TCONS_00030193	0.0320068	-0.40418	-0.735535	BGIOSGA029614	NP_001131978.1Purine permease 3
TCONS_00019785	0.0321833	-0.00964993	-0.291696	BGIOSGA018227	XP_015692424.1 probable LRR receptor-like serine/threonine-protein kinase MRH1
TCONS_00026373	0.0322442	-0.333285	-1.08748	BGIOSGA014924	XP_006660036.1 indole-3-glycerol phosphate synthase, chloroplastic-like
TCONS_00015875	0.0322657	-0.212843	-0.212251	BGIOSGA015114	XP_006653389.2 protein FAM188A
TCONS_00019123	0.032293	0.645383	1.34493	BGIOSGA008589	XP_015693219.1 nuclear pore complex protein NUP50B-like
TCONS_00008845	0.0323925	-0.995391	-0.513929	BGIOSGA014372	XP_006647946.2 methionine aminopeptidase 1D, chloroplastic/mitochondrial
TCONS_00004775	0.0325772	-2.77268	-0.527402	BGIOSGA025243	XP_015699157.1 serine/threonine-protein kinase Nek5
TCONS_00016887	0.0325816	-1.22547	-0.445456	BGIOSGA014074	XP_006653038.1 choline transporter-like protein 2
TCONS_00026514	0.0326155	0.0223037	0.677693	BGIOSGA034915	XP_006660095.2 phosphoglucan phosphatase LSF1, chloroplastic
TCONS_00000666	0.0327543	-0.23193	0.466144	BGIOSGA003253	XP_004967687.1uncharacterized protein LOC101776220
TCONS_00026239	0.0328027	0.0489644	0.31997	#N/A	#N/A
TCONS_00013075	0.0328533	0.262603	0.208885	BGIOSGA009906	XP_006650506.1 phytochrome A
TCONS_00010850	0.0329161	-0.432651	0.069909	BGIOSGA033761	XP_006665115.1 probable F-box protein At4g22165
TCONS_00023752	0.03297	0.612294	0.960213	BGIOSGA027780	XP_006657752.2 ATP-dependent helicase rhp16
TCONS_00007224	0.0331642	-3.32404	-2.03934	BGIOSGA005308	XP_015689679.1 receptor-like serine/threonine-protein kinase NCRK
TCONS_00022196	0.0332291	0.188758	0.484238	BGIOSGA021300	XP_006656058.2PREDICTED: uncharacterized protein LOC102711627
TCONS_00033041	0.0332389	1.02442	0.613434	BGIOSGA036569	XP_022684934.1protein FAM135B
TCONS_00022655	0.0332947	-0.533277	-0.127986	BGIOSGA007674	XP_004965737.1homeobox protein knotted-1-like 11 isoform X1
TCONS_00021275	0.0335866	-0.405316	-0.191764	BGIOSGA017326	XP_006657273.1 EH domain-containing protein 1-like
TCONS_00012193	0.0337227	-0.750635	-0.410259	BGIOSGA009975	XP_015690189.1 probable apyrase 1
TCONS_00014269	0.0338052	-0.83309	0.528216	BGIOSGA016425	XP_006653397.2 ubiquitin carboxyl-terminal hydrolase 27
TCONS_00026971	0.0339258	0.317065	0.136039	BGIOSGA006480	XP_006660302.1 type I inositol polyphosphate 5-phosphatase 13-like
TCONS_00022381	0.0339593	0.349559	-0.538803	BGIOSGA009791	XP_006656146.1PREDICTED: uncharacterized protein LOC102715027
TCONS_00031587	0.0339598	-0.00717992	0.466512	BGIOSGA013510	XP_006662570.1PREDICTED: uncharacterized protein LOC102709683
TCONS_00032277	0.0340666	0.348364	-0.690812	BGIOSGA010128	XP_006662375.1 cationic amino acid transporter 3, mitochondrial-like
TCONS_00022368	0.0340876	0.721745	0.573874	BGIOSGA001614	XP_006656144.1PREDICTED: uncharacterized protein LOC102714019
TCONS_00001476	0.0341506	-0.558892	-0.418952	BGIOSGA001241	XP_006644440.1 GDSL esterase/lipase At1g28600-like isoform X2
TCONS_00000705	0.0342277	0.519013	0.673965	BGIOSGA029758	XP_006644083.1 putative L-cysteine desulfhydrase 2
TCONS_00005224	0.0342712	-1.03956	-0.0188321	BGIOSGA018608	XP_022678510.1uncharacterized protein LOC101765570
TCONS_00016459	0.0342998	-1.91274	-1.4025	BGIOSGA014512	XP_006652679.1 protein FATTY ACID EXPORT 4, chloroplastic
TCONS_00034406	0.0343908	-2.00292	-0.933981	#N/A	#N/A
TCONS_00000663	0.0344245	-1.31413	-1.21105	BGIOSGA011224	XP_021313762.1E3 ubiquitin-protein ligase RING1-like
TCONS_00016533	0.0344285	-0.087147	1.09259	BGIOSGA014435	XP_006652735.1 protein AE7 isoform X2
TCONS_00002805	0.0346087	-1.09556	-0.0279549	BGIOSGA013326	XP_006643749.1PREDICTED: uncharacterized protein LOC102714322
TCONS_00000024	0.0347533	0.410479	-0.396344	BGIOSGA012543	XP_004967932.1ricin B-like lectin R40G3

TCONS_00014192	0.0349403	-0.871905	-0.810875	BGIOSGA016347	XP_015691684.1PREDICTED: uncharacterized protein LOC102699394 isoform X3
TCONS_00023303	0.0351002	0.312811	-0.104353	BGIOSGA012163	XP_006658376.1 coatomer subunit gamma-2
TCONS_00009404	0.0352636	1.32737	1.44374	BGIOSGA011957	XP_006649480.1 nuclear pore complex protein NUP96
TCONS_00037941	0.0352994	-0.234659	0.162645	BGIOSGA003329	XP_015697609.1 disease resistance protein RPP13-like
TCONS_00037365	0.0353912	-1.40845	0.0403101	BGIOSGA029560	XP_004979741.1UDP-glycosyltransferase 91B1
TCONS_00033420	0.0354616	-0.311822	-0.248049	BGIOSGA011098	XP_006648080.1 long chain base biosynthesis protein 1c
TCONS_00029732	0.0355141	0.0299487	0.0833005	BGIOSGA008523	XP_006660487.1 AP2-associated protein kinase 1-like
TCONS_00036372	0.0355832	-1.04741	-0.0871231	BGIOSGA037738	XP_017631547.1 pyridoxal kinase
TCONS_00020414	0.0356849	0.0565319	-0.0675028	BGIOSGA022599	XP_003562759.2S phase cyclin A-associated protein in the endoplasmic reticulum
TCONS_00012497	0.0357267	-1.15895	0.00383687	BGIOSGA011798	XP_002467646.1probable amino-acid acetyltransferase NAGS2, chloroplastic
TCONS_00028505	0.0359636	-2.20694	-2.05977	BGIOSGA009410	XP_015698603.1PREDICTED: uncharacterized protein LOC102700483
TCONS_00003336	0.0361371	-2.82934	-0.882709	BGIOSGA013254	NP_001167651.1tubulin beta-6 chain
TCONS_00037177	0.0363019	-0.28582	0.294674	BGIOSGA016920	XP_006664017.2 protein S-acyltransferase 10
TCONS_00028508	0.0363824	-0.746355	-0.00825219	BGIOSGA027634	XP_006660408.1 triacylglycerol lipase 1 isoform X1
TCONS_00002135	0.0364297	-0.952235	-3.80477	BGIOSGA012351	XP_006646464.2PREDICTED: uncharacterized protein LOC102710086
TCONS_00017548	0.0365247	0.451415	0.653162	BGIOSGA009387	XP_004962449.1rhodanese-like domain-containing protein 7
TCONS_00014530	0.0365662	0.870177	1.25313	BGIOSGA016695	XP_006652474.1PREDICTED: uncharacterized protein LOC102717792
TCONS_00012982	0.0367613	-0.297873	0.016834	BGIOSGA037662	NP_001333999.1uncharacterized LOC103643836
TCONS_00001902	0.036852	0.495764	0.433917	BGIOSGA017746	XP_006644784.1 signal recognition particle 54 kDa protein 2
TCONS_00012079	0.0368807	0.164734	0.414223	BGIOSGA003631	XP_022678806.1ubiquitin-activating enzyme E1 3 isoform X1
TCONS_00016304	0.036925	-1.20064	-0.858467	BGIOSGA005611	XP_006653619.1 probable beta-D-xylosidase 6
TCONS_00008812	0.0369337	0.670378	0.385665	BGIOSGA005617	XP_006647894.1 exocyst complex component SEC6 isoform X1
TCONS_00024553	0.0369703	-0.59579	-0.149189	BGIOSGA021456	XP_022684001.1disease resistance protein RPM1
TCONS_00022591	0.0370018	0.108599	-0.114354	BGIOSGA020852	XP_010227485.1stromal processing peptidase, chloroplastic isoform X1
TCONS_00033468	0.0373318	0.221375	-1.1638	BGIOSGA000760	XP_021316333.1cytochrome P450 94B3-like
TCONS_00014798	0.0373419	-0.24649	0.300811	BGIOSGA029521	XP_004976563.1ubiquitin fusion degradation protein 1 homolog
TCONS_00013908	0.0377799	-0.150313	-1.57892	BGIOSGA034929	XP_006652156.1 short-chain dehydrogenase TIC 32, chloroplastic-like
TCONS_00020112	0.037924	1.38213	0.367276	BGIOSGA022297	XP_006656631.1 single-stranded DNA-binding protein WHY1, chloroplastic-like
TCONS_00025813	0.0380277	-2.036	-2.59705	BGIOSGA018174	XP_012702109.1beta-carotene isomerase D27, chloroplastic
TCONS_00028735	0.0381066	0.334701	-0.139169	BGIOSGA031849	XP_003576406.1uncharacterized protein LOC100829630
TCONS_00004752	0.0382465	0.812225	0.394413	BGIOSGA016807	XP_015699191.1 RNA polymerase II C-terminal domain phosphatase-like 2 isoform X1
TCONS_00030811	0.0386824	0.256521	-0.159533	BGIOSGA017773	XP_006662230.1 alanine--tRNA ligase
TCONS_00010557	0.0387222	0.413885	0.328741	BGIOSGA037730	XP_003562255.1flowering locus K homology domain
TCONS_00031621	0.0387472	-2.90094	-0.14935	BGIOSGA005905	XP_006662058.1 VAN3-binding protein-like
TCONS_00027029	0.0387485	-0.913846	-0.324836	BGIOSGA031077	XP_004974050.1E3 ubiquitin-protein ligase RFI2 isoform X1
TCONS_00015999	0.0387762	-0.220568	0.542631	BGIOSGA004525	XP_002447950.1lipoyl synthase, mitochondrial
TCONS_00013037	0.0388302	0.513228	-0.249953	BGIOSGA031968	XP_015690036.1 myosin-binding protein 2-like
TCONS_00029497	0.0388591	-1.14018	-0.65531	BGIOSGA031252	XP_006660969.1 trihelix transcription factor ASIL1-like
TCONS_00026965	0.0390086	0.804212	0.613444	BGIOSGA029016	XP_015696115.1 ranBP2-type zinc finger protein At1g67325
TCONS_00022791	0.0390827	-1.12289	0.0563204	BGIOSGA007584	XP_015694268.1 zinc finger CCCH domain-containing protein 13-like isoform X3
TCONS_00022891	0.039255	-5.21707	-2.90995	BGIOSGA032421	XP_006657332.1 benzyl alcohol O-benzoyltransferase-like
TCONS_00024950	0.0393557	0.176868	0.0651818	BGIOSGA004003	XP_010228166.1DEAD-box ATP-dependent RNA helicase 5
TCONS_00031437	0.0394558	-0.363046	0.367124	BGIOSGA033252	XP_006661938.1PREDICTED: uncharacterized protein LOC102720912
TCONS_00000015	0.0396569	1.29669	-0.100987	BGIOSGA036411	XP_006643632.1PREDICTED: uncharacterized protein LOC102702786
TCONS_00006899	0.0396912	0.363762	-1.12686	BGIOSGA017184	XP_021315253.1outer envelope protein 64, mitochondrial
TCONS_00008804	0.0397257	-0.156927	0.206475	BGIOSGA017225	XP_015689462.1 cullin-3A-like
TCONS_00024619	0.0398274	-0.440139	0.699321	BGIOSGA024767	XP_015695177.1 histone deacetylase 15-like
TCONS_00016372	0.0399661	-0.10616	-0.353432	BGIOSGA005906	XP_006652610.1PREDICTED: uncharacterized protein LOC102712248
TCONS_00011808	0.0399801	-0.290897	0.00757767	BGIOSGA010817	NP_001339006.1Outer arm dynein light chain 1 protein
TCONS_00007748	0.0401581	0.553747	0.2834	BGIOSGA020743	XP_015688882.1 basic leucine zipper 9-like isoform X1
TCONS_00018523	0.0402995	-0.286837	-0.00381416	BGIOSGA010963	XP_002440502.150S ribosomal protein L10, chloroplastic
TCONS_00013142	0.0403956	-0.814941	-0.766659	BGIOSGA009843	XP_004981843.1inositol 3-kinase isoform X1
TCONS_00013356	0.0404321	0.896179	0.656208	BGIOSGA024679	XP_006650742.1PREDICTED: uncharacterized protein LOC102715282
TCONS_00016963	0.0405022	-2.0039	-2.7578	BGIOSGA019014	XP_015692939.1 protein EMBRYO SAC DEVELOPMENT ARREST 3, chloroplastic isoform X2

TCONS_00007534	0.0405112	0.139357	0.276299	BGIOSGA021732	XP_003571262.13-oxoacyl-[acyl-carrier-protein] synthase, mitochondrial
TCONS_00005557	0.0405907	-0.619267	0.330152	BGIOSGA008557	XP_006646975.1 probable protein phosphatase 2C 11
TCONS_00018429	0.0406569	0.693185	-0.187449	BGIOSGA019816	XP_006654880.1 calcium permeable stress-gated cation channel 1-like
TCONS_00036284	0.0407057	0.0601103	-0.486707	BGIOSGA037645	XP_015698444.1PREDICTED: uncharacterized protein LOC102703916
TCONS_00032049	0.0407095	0.460481	0.290828	BGIOSGA032089	XP_006654523.1 probable apyrase 7
TCONS_00011477	0.0407407	0.816795	-1.80872	BGIOSGA011548	XP_021306961.1beta-carotene 3-hydroxylase, chloroplastic
TCONS_00000034	0.0408254	0.22008	0.661435	BGIOSGA002603	XP_015699194.1 serine/threonine-protein kinase ATM isoform X2
TCONS_00008719	0.0409057	0.415385	0.192966	BGIOSGA022746	XP_006647816.2 enhancer of mRNA-decapping protein 4-like
TCONS_00028186	0.0410455	-0.495101	-0.0170407	BGIOSGA029619	XP_015696223.1 glucose-6-phosphate isomerase 1, chloroplastic-like
TCONS_00010583	0.0411685	-1.0415	-0.402002	BGIOSGA013198	XP_004982347.1DNA repair protein recA homolog 1, chloroplastic
TCONS_00011549	0.0413219	-1.18359	-0.121632	BGIOSGA011470	XP_015690770.1PREDICTED: uncharacterized protein LOC102707106
TCONS_00009263	0.041389	-0.116776	0.138975	BGIOSGA017907	XP_006663594.1 phosphatidate phosphatase PAH2-like
TCONS_00036824	0.0414361	-1.21383	-0.395417	BGIOSGA036487	XP_024315750.1uncharacterized protein LOC100832377 isoform X1
TCONS_00022610	0.0415211	0.523028	-0.488738	BGIOSGA000230	XP_015693900.1 NEP1-interacting protein-like 2
TCONS_00036392	0.041525	0.53765	0.194969	BGIOSGA032938	XP_006664177.1 homocysteine S-methyltransferase 3
TCONS_00035050	0.0416927	-0.390854	-0.587264	BGIOSGA033804	XP_006662979.1 myb-related protein 306-like
TCONS_00014749	0.0417556	-0.301805	0.15002	BGIOSGA018660	XP_006652646.2 transport and Golgi organization 2 homolog
TCONS_00016867	0.0419192	-0.0169797	-0.58389	BGIOSGA014097	XP_006653024.2 plastid division protein CDP1, chloroplastic
TCONS_00005548	0.0420126	-0.465622	-0.415238	BGIOSGA019760	XP_021313837.1zinc finger protein CONSTANS-LIKE 3
TCONS_00004087	0.042161	-0.996744	0.387041	BGIOSGA001101	XP_006644452.2 membrane-bound transcription factor site-2 protease homolog
TCONS_00013480	0.0421656	0.731578	1.42465	BGIOSGA009510	XP_006651985.1 pumilio homolog 24
TCONS_00035137	0.0422688	-0.484338	-0.0143417	BGIOSGA021456	XP_006663025.1PREDICTED: uncharacterized protein LOC102718497
TCONS_00026547	0.0425976	-0.0212213	-0.627844	BGIOSGA037142	XP_006659379.1 AP-4 complex subunit sigma
TCONS_00025988	0.042619	-1.52465	-1.12042	BGIOSGA028063	XP_015696097.1 translation initiation factor IF-2 isoform X1
TCONS_00036594	0.0428104	0.176754	0.273593	BGIOSGA006117	XP_006664298.1 bifunctional aspartokinase/homoserine dehydrogenase 1 isoform X3
TCONS_00031542	0.0428657	0.232243	-1.73669	BGIOSGA038222	XP_006662551.1 protein CURVATURE THYLAKOID 1B, chloroplastic-like
TCONS_00004930	0.0429002	0.414565	0.433856	BGIOSGA016437	XP_015688276.1 magnesium transporter MRS2-F
TCONS_00034230	0.0429354	0.153513	0.507748	BGIOSGA036939	XP_006663786.1 aladin-like
TCONS_00001119	0.0431807	0.0205801	-1.3011	BGIOSGA003741	XP_004968940.1uncharacterized protein LOC101776372
TCONS_00009384	0.0432465	0.572823	0.489961	BGIOSGA031938	XP_006651071.1 carbon catabolite repressor protein 4 homolog 1-like
TCONS_00006591	0.0432958	0.462362	1.45695	BGIOSGA037784	XP_006647638.1 probable lipid phosphate phosphatase beta
TCONS_00021376	0.0434626	-0.0563589	-0.111208	BGIOSGA023604	XP_006656490.1PREDICTED: uncharacterized protein LOC102720530
TCONS_00035458	0.0435527	-2.47953	-1.33028	BGIOSGA006347	XP_015698002.1 serine/threonine-protein kinase At5g01020-like
TCONS_00037046	0.0437563	0.614288	0.465346	BGIOSGA006680	XP_006663971.1 40S ribosomal protein S3a-like
TCONS_00011704	0.0438904	-0.0327678	1.11481	BGIOSGA011306	XP_006649533.1 2-carboxy-1,4-naphthoquinone phytyltransferase, chloroplastic isoform X1
TCONS_00014934	0.0440021	0.0379959	0.0147431	BGIOSGA007260	XP_006652817.1 gamma-aminobutyrate transaminase 1, mitochondrial
TCONS_00018339	0.0441086	-0.548875	0.0405441	BGIOSGA001086	XP_006654808.1 putative glucose-6-phosphate 1-epimerase isoform X1
TCONS_00021723	0.0441303	-0.81406	-0.749264	BGIOSGA023197	XP_015693757.1 probable protein phosphatase 2C 54
TCONS_00012812	0.0441793	0.614398	1.12134	BGIOSGA010149	XP_006650339.1 ornithine aminotransferase, mitochondrial
TCONS_00008707	0.044318	-0.275883	-2.45251	BGIOSGA005725	XP_006648997.2 basic blue protein-like
TCONS_00025556	0.0443222	-0.119126	0.329042	BGIOSGA023877	XP_003562650.1putative MYST-like histone acetyltransferase 1
TCONS_00000955	0.0443789	0.841146	0.0510794	BGIOSGA024227	XP_004968778.1NPL4-like protein
TCONS_00006392	0.0444021	0.584769	0.242449	BGIOSGA026762	XP_006647462.1 26S proteasome non-ATPase regulatory subunit 6-like
TCONS_00032543	0.044535	-1.17946	-0.88319	BGIOSGA018965	XP_011004377.1 actin-7-like isoform X2
TCONS_00020100	0.0445711	-0.948008	-0.719598	BGIOSGA022281	XP_006655781.2 DNA-repair protein XRCC1
TCONS_00011481	0.0445937	0.264957	-1.30462	BGIOSGA011544	XP_015689699.1PREDICTED: uncharacterized protein LOC102721435
TCONS_00031685	0.0446011	0.934882	0.25336	BGIOSGA008862	XP_006662105.1 TOM1-like protein 2
TCONS_00007560	0.0447213	0.444998	0.725721	BGIOSGA006923	XP_006647045.1 DEAD-box ATP-dependent RNA helicase 41
TCONS_00017747	0.0447238	0.436503	1.18271	BGIOSGA019809	XP_006655285.1 cyclin-dependent kinase D-1
TCONS_00013348	0.0448648	-0.402103	-1.45389	BGIOSGA022384	XP_002463733.1PRA1 family protein E
TCONS_00012596	0.0448872	-0.382053	-1.06361	BGIOSGA023467	XP_006650268.1 probable beta-1,3-galactosyltransferase 2
TCONS_00003979	0.0449262	-0.534758	0.150919	BGIOSGA001213	XP_006646097.1 ubiquitin-like modifier-activating enzyme atg7
TCONS_00031663	0.0449401	-0.421514	0.375041	BGIOSGA013044	XP_006662087.1 NADH dehydrogenase [ubiquinone] 1 alpha subcomplex assembly factor 3-like

TCONS_00031402	0.0450764	0.444544	0.116329	BGIOSGA033214	XP_006662479.2 mediator of RNA polymerase II transcription subunit 16
TCONS_00013288	0.0451797	1.46	-1.45468	BGIOSGA031515	XP_006650693.1 probable glutathione S-transferase GSTU1
TCONS_00028214	0.0452123	0.292101	1.79498	BGIOSGA026168	XP_006659519.1 serine/arginine-rich splicing factor SC35
TCONS_00031535	0.0452446	0.319371	-0.11866	BGIOSGA021783	XP_006661997.1 desumoylating isopeptidase 2-like
TCONS_00010524	0.0453946	0.988022	0.723669	BGIOSGA020338	XP_015691206.1 T-complex protein 1 subunit beta
TCONS_00022916	0.0454187	2.01742	-0.0603926	BGIOSGA028131	XP_004966470.1protein EARLY-RESPONSIVE TO DEHYDRATION 7, chloroplastic
TCONS_00014018	0.0454198	1.39913	0.452696	BGIOSGA016164	XP_006652188.1 cell division cycle 5-like protein
TCONS_00022797	0.0454701	-0.208934	-0.309442	BGIOSGA005753	XP_015694284.1 external alternative NAD(P)H-ubiquinone oxidoreductase B1, mitochondrial
TCONS_00029598	0.0455589	-2.10109	0.19878	BGIOSGA004689	XP_006661031.1 PI-PLC X domain-containing protein At5g67130
TCONS_00032782	0.0455761	-0.180253	-0.32372	BGIOSGA007638	XP_006662107.1 choline-phosphate cytidylyltransferase 2-like
TCONS_00004604	0.0456959	-1.822	0.418507	BGIOSGA000613	XP_009386029.1 14 kDa zinc-binding protein
TCONS_00018226	0.0458544	-1.32159	-1.20083	BGIOSGA008016	XP_006654722.1 protein IQ-DOMAIN 1-like
TCONS_00011229	0.0459471	0.368849	0.629352	BGIOSGA009190	XP_015690129.1 NEDD8-activating enzyme E1 regulatory subunit-like
TCONS_00003934	0.0465063	2.27594	0.789927	BGIOSGA001585	XP_006644394.2 cytochrome P450 72A15-like
TCONS_00019999	0.0465131	-1.61542	-0.492125	BGIOSGA023825	XP_006656567.1 protein SMG9-like
TCONS_00016633	0.0465164	0.00436688	-0.397528	BGIOSGA014343	XP_006652838.1 F-box/WD-40 repeat-containing protein At5g21040
TCONS_00022417	0.0465884	-0.956126	-0.946911	BGIOSGA022118	XP_006656159.2 ABC transporter G family member 42-like
TCONS_00025072	0.0465909	-0.314765	-2.44409	BGIOSGA034755	XP_004957041.1tuliposide A-converting enzyme 1, chloroplastic
TCONS_00036544	0.0467436	0.30367	0.576791	BGIOSGA036776	XP_006664861.1PREDICTED: uncharacterized protein LOC102700676
TCONS_00017087	0.0471546	-0.366546	-0.310065	BGIOSGA009787	XP_006654810.1 pto-interacting protein 1-like
TCONS_00003138	0.0471724	-0.376232	-0.34171	BGIOSGA002083	XP_006643936.1 protein Mpv17
TCONS_00013539	0.0471845	0.222993	-0.0446572	BGIOSGA009437	XP_006650897.1 3-oxoacyl-[acyl-carrier-protein] reductase FabG isoform X2
TCONS_00025377	0.047236	-0.482382	0.382962	BGIOSGA004566	XP_006657864.2 zinc-metallopeptidase, peroxisomal-like isoform X1
TCONS_00000466	0.0474488	0.135196	-0.290429	BGIOSGA004415	NP_001333482.1reticulon-like protein B1
TCONS_00014730	0.0476914	0.454734	-0.847616	BGIOSGA034146	XP_002446921.1probable phospholipid hydroperoxide glutathione peroxidase 6, mitochondrial
TCONS_00019810	0.0477973	1.17915	0.62624	BGIOSGA029939	XP_006654803.1 DNA-3-methyladenine glycosylase 1-like
TCONS_00011763	0.0478113	0.167009	0.638703	BGIOSGA011242	XP_004985267.154S ribosomal protein L51, mitochondrial
TCONS_00033701	0.0480962	-0.158756	-0.346553	BGIOSGA035474	XP_015698034.1 replication factor C subunit 1 isoform X1
TCONS_00017058	0.0482218	-0.611066	0.171504	BGIOSGA019111	XP_006654953.1PREDICTED: uncharacterized protein LOC102701824
TCONS_00031677	0.0486802	0.26113	-0.412644	BGIOSGA033496	XP_006662101.1 NADH dehydrogenase [ubiquinone] 1 beta subcomplex subunit 10-B-like
TCONS_00010435	0.0486868	0.103029	-0.0837413	BGIOSGA021974	XP_015690752.1PREDICTED: LOW QUALITY PROTEIN: uncharacterized protein LOC102704410
TCONS_00011246	0.0487458	-0.304221	1.20614	BGIOSGA027931	XP_006650819.1 hemK methyltransferase family member 2
TCONS_00032842	0.0490634	0.621757	0.105776	BGIOSGA036733	XP_006663737.1PREDICTED: uncharacterized protein LOC102701873
TCONS_00034546	0.0492229	-0.725493	-0.241279	BGIOSGA015581	XP_002449362.1uncharacterized protein LOC8071686
TCONS_00029620	0.0493291	-1.83823	0.514023	BGIOSGA036325	XP_021304755.1ABC transporter G family member 11
TCONS_00023792	0.0495806	1.03843	0.214363	BGIOSGA012310	XP_006658622.1 ABC transporter G family member 43-like
TCONS_00017142	0.0497511	-0.0603536	-0.881118	BGIOSGA010749	XP_006654999.1 formin-like protein 7
TCONS_00020783	0.0500395	-0.546144	-0.296871	BGIOSGA001535	XP_024318165.1disease resistance protein RPM1
TCONS_00023820	0.0500709	-0.582184	0.412568	BGIOSGA025851	XP_015694906.1 protein ORAOV1 homolog
TCONS_00019395	0.0501151	-0.393341	0.817451	BGIOSGA003012	XP_006655367.2PREDICTED: uncharacterized protein LOC102707966
TCONS_00024381	0.0501495	2.70165	0.925249	BGIOSGA020734	XP_015694964.1 protein kinase 2A, chloroplastic-like
TCONS_00036070	0.050191	-0.569737	0.218657	BGIOSGA000593	XP_006664036.2PREDICTED: uncharacterized protein LOC102700197
TCONS_00016640	0.0503017	-0.218802	0.586255	BGIOSGA002990	XP_004987374.1transcription factor GTE7
TCONS_00000626	0.050493	-0.28368	-0.660565	BGIOSGA003209	NP_001310340.1geranylgeranyl diphosphate reductase, chloroplastic-like
TCONS_00036837	0.0505973	1.01387	-0.00874608	BGIOSGA032873	XP_006664417.1 probable protein phosphatase 2C 77
TCONS_00018979	0.0506847	-0.409452	-0.298469	BGIOSGA019146	XP_004962489.1acyl transferase 5
TCONS_00009068	0.0507043	-0.283333	-3.18986	BGIOSGA009554	XP_004954457.1zinc finger protein ZAT5
TCONS_00007195	0.0508019	0.00537726	-0.195071	BGIOSGA029555	XP_006660762.1 60S ribosomal protein L9
TCONS_00014081	0.0508538	0.512914	-0.0542343	BGIOSGA016234	XP_006652201.1 RPM1-interacting protein 4-like
TCONS_00029094	0.0508583	-0.0228745	-0.424554	BGIOSGA030844	XP_006660663.1 oryzain gamma chain
TCONS_00008289	0.0509389	0.715759	0.888529	BGIOSGA006141	NP_001149298.1alkaline phosphatase D precursor
TCONS_00001756	0.0509747	0.0802514	-0.107569	BGIOSGA029866	XP_003578837.1probable alpha,alpha-trehalose-phosphate synthase [UDP-forming] 7
TCONS_00027187	0.0511521	1.28805	0.824492	BGIOSGA027815	XP_006659731.1 nicalin
TCONS_00030040	0.0511782	0.270372	0.0401939	BGIOSGA028017	XP_004956809.160S ribosomal protein L34

TCONS_00004981	0.0511819	0.612581	0.0347709	BGIOSGA026597	XP_004971074.1CBS domain-containing protein CBSX6
TCONS_00006458	0.0511997	-3.58338	-3.07127	BGIOSGA007704	XP_020407052.1uncharacterized LOC100278596 isoform X1
TCONS_00030943	0.0514634	-1.1329	-0.59585	BGIOSGA013687	XP_006662250.1 probable tRNA (guanine(26)-N(2))-dimethyltransferase 2
TCONS_00029547	0.0517268	0.21727	-0.0649776	BGIOSGA031305	XP_006661005.2PREDICTED: uncharacterized protein LOC102709675
TCONS_00018430	0.0517641	-0.203562	0.102504	BGIOSGA020485	XP_006654882.1 sm-like protein LSM8
TCONS_00021554	0.0519331	-0.0120208	-0.0446081	BGIOSGA021997	XP_002437766.2tRNA 2'-phosphotransferase 1
TCONS_00028559	0.0519668	-0.444681	0.0985351	BGIOSGA038076	XP_006660423.1 calmodulin-binding receptor-like cytoplasmic kinase 3
TCONS_00001059	0.0519746	0.561593	0.413647	BGIOSGA003674	XP_006644245.1 protein AATF
TCONS_00007586	0.0519754	#NA	-3.86159	#N/A	#N/A
TCONS_00014275	0.0521873	-0.455734	-0.735141	BGIOSGA006968	XP_003579753.1probable serine/threonine-protein kinase At1g01540 isoform X1
TCONS_00000996	0.0523494	-1.52467	0.135856	BGIOSGA003595	XP_006644206.1 oxygen-evolving enhancer protein 1, chloroplastic
TCONS_00023598	0.0523501	0.775061	-0.70945	BGIOSGA005904	XP_015695105.1 probable aquaporin PIP2-1
TCONS_00006218	0.052452	0.311443	0.892806	BGIOSGA008381	XP_006647352.2 ribonuclease J isoform X1
TCONS_00008119	0.0524769	0.120005	-0.391988	BGIOSGA006316	XP_015688620.1PREDICTED: uncharacterized protein At5g03900, chloroplastic, partial
TCONS_00011012	0.0525215	-0.0322016	1.46489	BGIOSGA024936	XP_015690083.1 casein kinase II subunit alpha-like
TCONS_00007844	0.0526352	-0.937666	-0.788533	BGIOSGA022389	XP_015689528.1 putative disease resistance RPP13-like protein 3 isoform X1
TCONS_00022277	0.0530591	0.803477	-0.221374	BGIOSGA023101	XP_006656098.1 putative phospholipid-transporting ATPase 9 isoform X1
TCONS_00020617	0.0531461	0.476943	1.23724	#N/A	#N/A
TCONS_00035698	0.0532893	-2.04984	-0.96279	BGIOSGA037113	XP_006663881.1 photosystem I reaction center subunit N, chloroplastic
TCONS_00016275	0.0533114	0.211685	-0.214707	BGIOSGA015695	XP_006652525.1 endoplasmic reticulum metallopeptidase 1
TCONS_00010265	0.0534612	0.643332	0.590846	BGIOSGA012829	XP_006432385.1DNA-directed RNA polymerase III subunit 2 isoform X1
TCONS_00001888	0.0535952	0.330559	0.443169	BGIOSGA010095	XP_006644769.1 regulatory protein NPR3-like
TCONS_00026821	0.0539742	0.0824483	-0.030502	BGIOSGA028874	XP_015696016.1 copper-transporting ATPase PAA1, chloroplastic isoform X2
TCONS_00011931	0.0542419	3.40849	1.23447	BGIOSGA003170	XP_006651229.2PREDICTED: uncharacterized protein LOC102700696
TCONS_00013882	0.0543132	0.462619	0.058969	BGIOSGA016060	XP_015692182.1PREDICTED: uncharacterized protein LOC102717977
TCONS_00037535	0.0544105	0.484873	0.512044	BGIOSGA029774	XP_015698721.1 septin and tuftelin-interacting protein 1 homolog 1
TCONS_00031522	0.0546806	0.0264149	-1.76377	BGIOSGA014998	XP_006661986.1 probable glutathione S-transferase GSTU6
TCONS_00025382	0.0547167	-0.474289	-0.7036	BGIOSGA011168	XP_015695379.1 vacuole membrane protein KMS1-like
TCONS_00032962	0.0548245	0.273079	0.267277	BGIOSGA036986	XP_006663204.1PREDICTED: uncharacterized protein LOC102705296
TCONS_00001371	0.054944	-0.0614338	0.309861	BGIOSGA008529	XP_006646107.2 probable protein phosphatase 2C 7
TCONS_00013235	0.0551868	-0.0543046	0.169705	BGIOSGA004457	XP_015690088.1PREDICTED: uncharacterized protein LOC102702085
TCONS_00003686	0.0551999	-0.672751	0.00264636	BGIOSGA001515	XP_006644235.1 putative disease resistance protein RGA3 isoform X1
TCONS_00007396	0.0552628	0.911466	0.448494	BGIOSGA020678	XP_015689243.1 BEACH domain-containing protein B
TCONS_00029897	0.055301	-0.858687	-0.0780441	BGIOSGA033598	XP_024318534.1disease resistance protein RPP13-like
TCONS_00022025	0.0553685	-0.771876	-1.00552	BGIOSGA021491	XP_004965187.1protein CURVATURE THYLAKOID 1A, chloroplastic
TCONS_00008133	0.0555454	-0.714757	0.465619	BGIOSGA025643	XP_015689155.1 putative F-box/FBD/LRR-repeat protein At1g66290
TCONS_00010843	0.0555496	0.783011	-0.716916	BGIOSGA037877	XP_006650501.1 actin-1
TCONS_00010547	0.0556831	1.29979	0.330377	BGIOSGA013526	XP_010232509.2protein JINGUBANG
TCONS_00024490	0.0557581	-1.50446	-0.320942	BGIOSGA021093	XP_006658233.1PREDICTED: uncharacterized protein LOC102719504
TCONS_00021221	0.0557979	-0.797576	-0.24061	BGIOSGA023443	XP_006657238.1 peptidyl-prolyl cis-trans isomerase CYP59
TCONS_00027173	0.0558123	0.143538	0.31197	BGIOSGA029222	XP_015696224.1PREDICTED: uncharacterized protein LOC102714213
TCONS_00016262	0.055857	-0.554103	-2.38616	BGIOSGA022261	XP_006652519.1 probable low-specificity L-threonine aldolase 1
TCONS_00027532	0.0558723	1.03303	1.041	BGIOSGA027482	XP_006659206.1 KH domain-containing protein At4g18375
TCONS_00024551	0.056183	-0.752696	-0.354449	BGIOSGA024840	XP_015694468.1 oxygen-evolving enhancer protein 2, chloroplastic-like
TCONS_00015655	0.0562933	0.597372	0.139327	BGIOSGA002002	XP_006653274.1 aspartic proteinase-like protein 2
TCONS_00006412	0.0564986	-0.0357816	1.17978	BGIOSGA037734	XP_006648820.2 transcription factor bHLH128
TCONS_00027735	0.056834	-0.988129	-0.142253	BGIOSGA027267	XP_015696161.1 probable adenylate kinase 5, chloroplastic isoform X1
TCONS_00008772	0.0568808	0.855722	0.619217	BGIOSGA005662	XP_006647858.1 nicastrin isoform X1
TCONS_00030624	0.0569077	1.66481	-0.270759	BGIOSGA024238	XP_012703807.1UDP-glycosyltransferase 91C1
TCONS_00022837	0.0569223	-0.78135	-0.202589	BGIOSGA007550	XP_006656423.1 E3 ubiquitin-protein ligase arkadia-like
TCONS_00022045	0.0572089	0.502326	0.850087	BGIOSGA015551	XP_006663115.1 probable LRR receptor-like serine/threonine-protein kinase At3g47570
TCONS_00009171	0.0573573	0.351737	0.345081	BGIOSGA013744	XP_006650969.2PREDICTED: uncharacterized protein LOC102711416
TCONS_00029459	0.0573808	-1.47237	0.555007	BGIOSGA031212	XP_002445194.1putative F-box/LRR-repeat protein At5g41840 isoform X1
TCONS_00005427	0.0574051	0.402689	-1.0227	BGIOSGA023549	XP_006646882.1 protein CHLOROPLAST IMPORT APPARATUS 2

TCONS_00037268	0.0574272	-0.455665	-0.517645	BGIOSGA036403	XP_015698314.1 ABC transporter G family member 50-like
TCONS_00002611	0.0575153	-0.237266	0.211762	BGIOSGA000031	XP_024314581.1uncharacterized protein LOC100835523, partial
TCONS_00014412	0.0576284	-2.3672	-1.32773	BGIOSGA016575	XP_015691490.1 protein ALTERED XYLOGLUCAN 4-like
TCONS_00030358	0.0578095	0.107183	-0.499326	BGIOSGA034853	XP_015696550.1 transcription factor RF2a
TCONS_00010923	0.0581041	-0.0756433	0.556204	BGIOSGA013531	XP_006650562.1 CWF19-like protein 2
TCONS_00024199	0.0581172	-0.382263	0.33019	BGIOSGA034288	XP_006658030.1 xylulose kinase-like
TCONS_00013379	0.0581284	-2.27698	-1.00869	BGIOSGA013773	XP_015690117.1 protochlorophyllide-dependent translocon component 52, chloroplastic-like
TCONS_00009117	0.0581949	0.615964	0.316373	BGIOSGA020885	XP_006650939.1 ribose-phosphate pyrophosphokinase 1-like
TCONS_00025535	0.058371	0.107301	0.591886	BGIOSGA004017	XP_006657969.2 CDPK-related protein kinase-like
TCONS_00025166	0.0584783	0.359679	0.334049	BGIOSGA013582	XP_006657739.1 serine/threonine-protein kinase svkA-like
TCONS_00014016	0.0587375	0.619322	0.672499	BGIOSGA016162	XP_015691726.1PREDICTED: uncharacterized protein LOC102699399 isoform X2
TCONS_00004045	0.0588256	-0.39497	-0.282369	BGIOSGA001147	XP_015692399.1 protein CHROMATIN REMODELING 24-like
TCONS_00002391	0.0592779	0.00756361	0.338045	BGIOSGA005028	XP_004971016.1protein BLISTER
TCONS_00017168	0.0597366	-1.26827	-0.660671	BGIOSGA019214	XP_006655010.1PREDICTED: uncharacterized protein LOC102718837 isoform X1
TCONS_00033912	0.0598873	-0.432325	0.00460005	BGIOSGA035607	XP_006663072.1 protein FRA10AC1
TCONS_00027790	0.0599643	-0.915732	-0.109191	BGIOSGA030213	XP_003571630.1uncharacterized protein LOC100825830
TCONS_00021741	0.0600577	-1.18378	0.804847	BGIOSGA021780	XP_006655853.2PREDICTED: uncharacterized protein LOC102722683
TCONS_00027004	0.0601503	1.22789	1.61357	BGIOSGA029055	XP_010235340.1plant-specific TFIIB-related protein PTF2
TCONS_00002733	0.0601742	0.590929	0.484637	BGIOSGA002489	XP_015688104.1 ABC transporter G family member 7
TCONS_00025234	0.0602364	-0.627397	0.580901	BGIOSGA000657	XP_015695426.1 tyrosyl-DNA phosphodiesterase 1 isoform X1
TCONS_00001245	0.0604184	-0.236528	-0.634143	BGIOSGA003873	XP_006644326.1PREDICTED: uncharacterized protein LOC102721028
TCONS_00019707	0.0604356	1.06283	1.18282	BGIOSGA022322	XP_015693166.1 nucleosome assembly protein 1;2 isoform X1
TCONS_00021891	0.0607503	-0.291573	0.658733	BGIOSGA021631	XP_002454582.1transcription termination factor MTERF8, chloroplastic
TCONS_00024633	0.0607633	0.0911756	-1.68172	#N/A	#N/A
TCONS_00022720	0.061235	0.37661	0.379228	BGIOSGA029051	XP_006657223.1 squamosa promoter-binding-like protein 11
TCONS_00026961	0.0616353	0.490669	0.159407	BGIOSGA029511	XP_006659608.1 pumilio homolog 5
TCONS_00022296	0.0618707	0.0743684	0.179571	BGIOSGA021193	XP_006651498.1 dihydrolipoyllysine-residue acetyltransferase component 1 of pyruvate dehydrogenase complex, mitochondrial
TCONS_00023918	0.0618803	-0.760776	0.152161	BGIOSGA025951	XP_019701983.1 WASH complex subunit FAM21 isoform X1
TCONS_00014680	0.0620181	0.112091	-0.0043408	BGIOSGA033281	XP_015692174.1 sphingoid long-chain bases kinase 1-like
TCONS_00016602	0.0621678	-0.470237	-0.0691507	BGIOSGA034397	XP_015692329.1 cell differentiation protein RCD1 homolog
TCONS_00033551	0.0622505	0.589801	0.727633	BGIOSGA000204	XP_006662956.1 long chain base biosynthesis protein 2a
TCONS_00031811	0.0623704	0.398264	-0.593215	BGIOSGA007985	XP_024315700.1uncharacterized protein LOC100840606 isoform X1
TCONS_00030056	0.0623843	-3.40911	-0.785894	BGIOSGA006511	XP_015696346.1 probable GMP synthase [glutamine-hydrolyzing]
TCONS_00006913	0.062435	0.397767	0.216357	BGIOSGA009095	XP_006649067.1 presequence protease 1, chloroplastic/mitochondrial-like
TCONS_00024729	0.0626327	-1.40942	-0.271936	BGIOSGA017111	XP_010227266.2uncharacterized protein LOC100833242 isoform X2
TCONS_00014744	0.0627998	0.275052	0.425956	BGIOSGA011777	XP_006652641.1 coiled-coil domain-containing protein 124-like
TCONS_00022658	0.062908	-0.599883	0.560073	BGIOSGA034468	XP_006657192.1 lysosomal Pro-X carboxypeptidase
TCONS_00008112	0.0630103	0.460405	-0.520287	BGIOSGA006338	XP_006647336.1 pentatricopeptide repeat-containing protein At4g39530-like
TCONS_00007087	0.0633312	-1.04755	-1.47154	BGIOSGA009269	XP_015689525.1 F-box protein PP2-B1-like
TCONS_00021569	0.0633856	0.550881	1.46243	BGIOSGA021976	XP_006655766.1 ribosome production factor 1
TCONS_00014309	0.0634731	0.0381656	0.49858	BGIOSGA012541	XP_015691669.1 protein phosphatase methylesterase 1
TCONS_00036531	0.0635335	0.350259	-0.859126	BGIOSGA036795	XP_006663712.1 SAM50-like protein SPAC17C9.06
TCONS_00022439	0.0635669	-0.252809	-0.264584	BGIOSGA027055	XP_006656171.1 cysteine synthase-like
TCONS_00005726	0.0636409	-0.223061	-0.789001	BGIOSGA021058	XP_008677269.1zinc ion binding protein isoform X1
TCONS_00022915	0.0637632	0.462163	-0.70615	BGIOSGA017801	XP_004966469.1basic leucine zipper 23
TCONS_00035642	0.0638725	-0.130293	0.00606614	BGIOSGA003276	XP_006663861.1 gamma carbonic anhydrase 2, mitochondrial
TCONS_00035922	0.0640414	-2.36079	-0.771845	BGIOSGA040478	XP_006649309.1 histone-lysine N-methyltransferase SETD1A-like
TCONS_00021202	0.0640482	1.13312	-0.0991106	BGIOSGA034617	XP_006656342.1 vacuolar-sorting receptor 7
TCONS_00011201	0.064063	-0.486895	-0.183592	BGIOSGA001940	XP_015690632.1 ADP-ribosylation factor 1-like
TCONS_00018748	0.0640957	0.404474	0.37378	BGIOSGA004998	XP_006654126.1 30S ribosomal protein S31, chloroplastic-like
TCONS_00026473	0.0641932	1.11682	0.487673	BGIOSGA000169	XP_003571689.1pirin-like protein
TCONS_00016051	0.0642024	1.5961	0.102698	BGIOSGA006174	XP_006652391.1PREDICTED: uncharacterized protein LOC102716401
TCONS_00025623	0.0642403	-0.224122	-0.00410653	BGIOSGA023811	XP_004958524.139S ribosomal protein L45, mitochondrial
TCONS_00009663	0.0643492	-0.176251	-0.182988	BGIOSGA009740	XP_015690960.1 TSL-kinase interacting protein 1

TCONS_00007939	0.0643643	-1.35028	-1.29156	BGIOSGA000589	XP_006647240.1 synaptotagmin-2-like
TCONS_00019209	0.064396	-2.09943	-1.68861	BGIOSGA037761	XP_008649587.1ATPase WRNIP1
TCONS_00019671	0.0646695	-0.00017433	-0.463396	BGIOSGA002968	XP_004961422.1enoyl-CoA delta isomerase 2, peroxisomal
TCONS_00024202	0.0647409	-0.625126	-0.624523	BGIOSGA010567	XP_006658888.1 trafficking protein particle complex subunit 3
TCONS_00012160	0.064999	-0.0775886	0.19362	BGIOSGA010851	XP_006649993.1 glucose-6-phosphate 1-dehydrogenase 4, chloroplastic
TCONS_00020239	0.0650723	-1.02069	0.00735356	BGIOSGA022429	XP_006656708.1PREDICTED: uncharacterized protein LOC102719776, partial
TCONS_00012816	0.0650808	-1.82269	-0.373035	BGIOSGA024989	XP_015690289.1 proline transporter 1-like
TCONS_00030157	0.0651379	-0.0126672	-0.443393	BGIOSGA028826	XP_004956996.1junctophilin-1
TCONS_00002138	0.0651774	0.230774	0.129013	BGIOSGA008061	XP_006644978.1 coatomer subunit zeta-3-like
TCONS_00009153	0.0653131	-0.0767957	0.398999	BGIOSGA031471	XP_015691208.1 E3 ubiquitin-protein ligase RFWD3-like
TCONS_00035211	0.0654074	0.422882	0.630768	BGIOSGA033616	XP_006663051.1 mitochondrial inner membrane protease subunit 1
TCONS_00008499	0.0654107	-0.0619948	-0.505837	BGIOSGA032009	XP_006647611.1 probable receptor-like protein kinase At5g47070
TCONS_00014553	0.0656388	-0.0439589	0.438251	BGIOSGA016716	XP_006653574.2 trafficking protein particle complex II-specific subunit 120 homolog
TCONS_00005654	0.0658334	0.119213	1.13365	BGIOSGA007769	XP_015688509.1 sterol 3-beta-glucosyltransferase UGT80B1
TCONS_00021972	0.0661233	0.0664416	0.354077	BGIOSGA021542	XP_006656840.1 pyrophosphate--fructose 6-phosphate 1-phosphotransferase subunit beta
TCONS_00013560	0.0661837	0.206095	-0.0335578	BGIOSGA031346	XP_006652038.1 probable ADP-ribosylation factor GTPase-activating protein AGD9
TCONS_00028170	0.0662273	-0.963746	-0.47876	BGIOSGA024334	XP_002444515.1tuliposide A-converting enzyme 1, chloroplastic
TCONS_00007472	0.0669427	-2.44992	-0.412903	BGIOSGA026026	XP_015688832.1 probable receptor-like protein kinase At1g80640
TCONS_00006226	0.0671492	0.677752	1.22911	BGIOSGA018829	XP_006647354.1PREDICTED: uncharacterized protein LOC102710758
TCONS_00028558	0.0671516	0.918983	0.387408	BGIOSGA030303	XP_006661033.1 flowering time control protein FCA-like
TCONS_00016424	0.0672551	1.00454	0.466726	BGIOSGA014543	XP_006652645.2 Niemann-Pick C1 protein-like
TCONS_00017046	0.067322	0.276157	0.272185	BGIOSGA011863	XP_003561933.1uncharacterized protein LOC100834465
TCONS_00029207	0.0674493	0.0133405	-0.00786136	BGIOSGA033898	XP_004957095.1protein ROOT PRIMORDIUM DEFECTIVE 1
TCONS_00020052	0.0674674	0.155252	1.10953	BGIOSGA027570	XP_015694090.1 spastin isoform X2
TCONS_00033332	0.0675234	1.18741	1.0504	BGIOSGA035161	XP_006663372.1 pre-rRNA-processing protein TSR1 homolog
TCONS_00011215	0.067673	-2.40828	-1.37862	BGIOSGA013818	XP_015690432.1 alpha-ketoglutarate-dependent dioxygenase AlkB-like
TCONS_00002682	0.0677281	-4.1036	-1.11203	BGIOSGA002539	XP_012701813.1classical arabinogalactan protein 9
TCONS_00035908	0.0677592	-0.485207	0.678274	BGIOSGA040457	XP_006664874.2 biogenesis of lysosome-related organelles complex 1 subunit 1-like
TCONS_00032714	0.0678521	0.544614	0.89781	BGIOSGA031393	XP_006662065.2 protein ARABIDILLO 1-like
TCONS_00030295	0.068436	0.0063451	0.0971928	BGIOSGA016421	XP_015696733.1 uncharacterized ATP-dependent helicase C29A10.10c-like
TCONS_00012302	0.0684712	0.225146	0.228062	BGIOSGA010693	XP_006650102.1 ER membrane protein complex subunit 4
TCONS_00008923	0.0684864	-4.10456	-0.891538	BGIOSGA029691	XP_015688792.1 20 kDa chaperonin, chloroplastic-like
TCONS_00027783	0.0685187	1.43613	0.608561	BGIOSGA018691	XP_004955547.1eukaryotic translation initiation factor 3 subunit I
TCONS_00007515	0.0687383	-3.46941	-0.748015	BGIOSGA017741	XP_003571119.1glucomannan 4-beta-mannosyltransferase 1
TCONS_00006314	0.0687463	-0.397756	-0.635026	BGIOSGA036166	XP_015689138.1 sugar transport protein 5-like
TCONS_00000895	0.0690808	#NA	#NA	BGIOSGA000168	XP_006644154.1 protein SRG1-like
TCONS_00031463	0.0691549	0.811682	-0.0238695	BGIOSGA016850	XP_006661959.1 sphingoid long-chain bases kinase 1-like
TCONS_00011042	0.0696878	-0.037639	0.850546	BGIOSGA033351	XP_006650663.2 L-type lectin-domain containing receptor kinase IV.1-like
TCONS_00023537	0.0699012	-0.0101845	-0.158786	BGIOSGA015877	XP_006657630.1 ATG8-interacting protein 1
TCONS_00029881	0.0699791	0.693104	0.369521	BGIOSGA027570	XP_015696697.1 protein transport protein Sec61 subunit alpha-like isoform X1
TCONS_00016936	0.0701284	0.302918	0.0958169	BGIOSGA014029	XP_015692399.1 protein CHROMATIN REMODELING 24-like
TCONS_00017766	0.0701963	-0.474044	-1.90098	BGIOSGA028248	XP_006654369.1 endo-1,3;1,4-beta-D-glucanase-like
TCONS_00019801	0.0705192	-0.521175	0.446431	BGIOSGA017554	XP_006654797.1PREDICTED: uncharacterized protein ycf23-like
TCONS_00006124	0.0707875	-0.589639	0.441363	BGIOSGA016267	XP_015689027.1PREDICTED: uncharacterized protein LOC102719651, partial
TCONS_00010359	0.0709504	-2.52271	-0.120073	BGIOSGA012941	XP_006650221.1 uncharacterized isomerase BH0283-like
TCONS_00029748	0.0710602	0.388359	0.155682	BGIOSGA027147	XP_006661078.1PREDICTED: uncharacterized protein LOC102721193
TCONS_00012022	0.0712007	0.70058	1.35982	BGIOSGA003899	XP_006649864.2 zinc finger protein 652-A
TCONS_00014551	0.0715452	-0.649773	0.56102	BGIOSGA016714	XP_004976131.2gamma-tubulin complex component 2
TCONS_00006241	0.0716423	-0.263899	0.0297937	BGIOSGA016432	XP_006652294.1 40S ribosomal protein S10
TCONS_00021928	0.071813	-0.739528	-1.5917	BGIOSGA000583	XP_015693518.1 transcription factor bHLH155-like
TCONS_00016876	0.0718401	-1.72429	-1.11193	BGIOSGA004568	XP_006653028.1 katanin p80 WD40 repeat-containing subunit B1-like
TCONS_00011500	0.0719288	1.20309	1.06661	BGIOSGA011526	XP_003558900.1NADH dehydrogenase [ubiquinone] 1 beta subcomplex subunit 7
TCONS_00029686	0.0723962	0.414066	-0.708623	BGIOSGA030389	XP_004959299.1uncharacterized protein LOC101757514
TCONS_00018126	0.0724584	0.283017	1.02368	BGIOSGA017392	XP_003568116.1tubby-like F-box protein 9

TCONS_00011517	0.072621	0.760674	1.64617	BGIOSGA010892	XP_006649346.2 probable acyl-activating enzyme 1, peroxisomal
TCONS_00030068	0.0726511	0.659334	0.27739	BGIOSGA009198	XP_006660636.1 AUGMIN subunit 8-like
TCONS_00023144	0.0726522	0.601389	1.0126	BGIOSGA013222	XP_004955489.1myristoylated alanine-rich C-kinase substrate
TCONS_00009340	0.0727244	0.593399	-0.0790563	BGIOSGA012751	XP_006663032.1 40S ribosomal protein S9-2-like
TCONS_00028729	0.0728179	0.662784	-0.126868	BGIOSGA030482	XP_015696295.1 mediator of RNA polymerase II transcription subunit 25
TCONS_00001926	0.0729927	-1.73029	0.25621	BGIOSGA024055	XP_006644800.2 zinc-metallopeptidase, peroxisomal-like
TCONS_00016249	0.0732497	#NA	-1.12577	BGIOSGA034558	XP_003581423.2probable inactive beta-glucosidase 14 isoform X1
TCONS_00035144	0.0735354	-0.103201	0.0333861	BGIOSGA035534	XP_006663029.1 transcription factor bHLH47-like isoform X1
TCONS_00026136	0.0735478	-0.376228	0.500557	BGIOSGA014376	XP_015695528.1 protein strawberry notch homolog 1-like
TCONS_00020964	0.0736681	1.28466	0.348954	BGIOSGA023187	XP_015693421.1 UDP-galactose/UDP-glucose transporter 3-like
TCONS_00008940	0.0737361	0.264349	-0.328406	BGIOSGA005466	XP_004954172.1diacylglycerol kinase 7
TCONS_00009456	0.0737726	-0.489803	0.559073	BGIOSGA012004	XP_006651110.2 uroporphyrinogen-III synthase, chloroplastic
TCONS_00007484	0.0739446	-0.806587	-1.39753	BGIOSGA014346	XP_006648389.2 myosin heavy chain, clone 203
TCONS_00006613	0.0740062	0.865761	1.73034	BGIOSGA008779	XP_006647660.1 tRNA pseudouridine synthase A, mitochondrial-like
TCONS_00010014	0.0740534	-1.12253	-0.230144	BGIOSGA012564	XP_004984476.1photosystem II repair protein PSB27-H1, chloroplastic
TCONS_00022340	0.0741568	1.36041	-0.364823	BGIOSGA028889	NP_001145340.1uncharacterized protein LOC100278669
TCONS_00004131	0.074308	0.40049	0.435339	BGIOSGA000819	XP_006654794.1 60S ribosomal protein L18a-like
TCONS_00017602	0.0743794	0.900962	0.0131089	BGIOSGA003020	XP_006655220.2 chlorophyll synthase, chloroplastic
TCONS_00016549	0.0746335	1.15045	0.628778	BGIOSGA006676	XP_015692306.1 GPCR-type G protein COLD1
TCONS_00004490	0.0746444	-0.171367	0.657601	BGIOSGA000730	XP_006644787.1PREDICTED: uncharacterized protein LOC102716265
TCONS_00010773	0.0747998	0.294592	0.420311	BGIOSGA013398	XP_015690031.1PREDICTED: uncharacterized protein LOC102700606
TCONS_00027213	0.0753217	-1.03937	-0.801481	BGIOSGA012721	XP_004972417.1protein DMP3
TCONS_00030785	0.0753482	-0.354707	0.756336	BGIOSGA032594	XP_006661659.1 maspardin
TCONS_00020241	0.0754737	-0.514455	-0.292055	BGIOSGA023368	XP_006655852.1 two-component response regulator ORR22
TCONS_00017019	0.0755633	0.55981	0.311481	BGIOSGA019066	XP_006653977.1 E3 UFM1-protein ligase 1 homolog
TCONS_00024703	0.0756265	0.482301	0.343612	BGIOSGA026446	XP_006658363.1 DDT domain-containing protein DDR4-like
TCONS_00009569	0.0758057	-2.29711	0.199669	BGIOSGA012117	XP_006649637.1 microtubule-associated protein 70-4-like
TCONS_00025740	0.0761973	0.105051	0.309529	BGIOSGA026432	XP_006658987.1 aldehyde dehydrogenase 22A1
TCONS_00032281	0.0762016	-0.120172	0.661716	BGIOSGA037565	XP_015697153.1 FH protein interacting protein FIP2-like isoform X2
TCONS_00000516	0.0763454	0.344794	0.547714	BGIOSGA008052	XP_006643966.1 KH domain-containing protein At4g18375-like
TCONS_00032171	0.0763536	0.339278	0.604164	BGIOSGA031967	XP_006649068.1 phenylalanine--tRNA ligase alpha subunit, cytoplasmic
TCONS_00007300	0.0768489	0.401226	0.448718	BGIOSGA007183	XP_006648286.1 pentatricopeptide repeat-containing protein At4g04790, mitochondrial-like
TCONS_00030556	0.0768965	1.164	0.543781	BGIOSGA005053	XP_015696607.1 serine/threonine-protein kinase CTR1-like
TCONS_00024316	0.0771335	-1.66197	0.869906	BGIOSGA016077	XP_015695123.1PREDICTED: uncharacterized protein LOC102703991
TCONS_00002930	0.0771447	-0.397948	0.554537	BGIOSGA027158	XP_006645545.1PREDICTED: uncharacterized protein LOC102706803
TCONS_00016559	0.0771577	-1.1637	-1.1784	BGIOSGA028258	XP_006652770.1 E3 ubiquitin-protein ligase RLIM
TCONS_00002243	0.0772173	-1.11994	-4.72628	BGIOSGA019271	XP_002456704.1photosystem II 22 kDa protein, chloroplastic
TCONS_00002312	0.0772215	0.37807	0.130176	BGIOSGA004954	XP_006646550.1 signal recognition particle subunit SRP72
TCONS_00033077	0.0775113	-3.95838	-0.510543	BGIOSGA034918	XP_006662778.1 dirigent protein 22-like
TCONS_00024370	0.0777879	0.960568	-0.583934	BGIOSGA012543	XP_021310045.1ricin B-like lectin R40G3
TCONS_00023073	0.0778508	0.0458512	0.257845	BGIOSGA025103	XP_012704125.1transcription termination factor MTERF2, chloroplastic
TCONS_00010769	0.0779117	0.24314	0.0899481	BGIOSGA000202	XP_006650445.1 ethylene-insensitive protein 2-like
TCONS_00005831	0.0779146	1.2427	0.00495768	BGIOSGA007956	XP_015689407.1 probable plastidic glucose transporter 2
TCONS_00020972	0.0779783	0.514974	-0.254339	BGIOSGA005941	XP_015693624.1 palmitoyl-acyl carrier protein thioesterase, chloroplastic-like
TCONS_00003004	0.0783047	-1.34634	-0.529155	BGIOSGA021030	XP_006643866.1 pollen-specific protein SF21-like
TCONS_00024448	0.0783459	-0.470606	-0.0400149	#N/A	#N/A
TCONS_00024461	0.078395	#NA	-1.30519	BGIOSGA033951	XP_015694933.1 mavicyanin-like
TCONS_00036235	0.0785066	1.3097	0.115458	BGIOSGA023666	XP_021302165.1phosphoinositide phospholipase C 2
TCONS_00013254	0.0790787	-0.754611	0.297652	BGIOSGA036746	XP_015697991.1PREDICTED: uncharacterized protein LOC102704371 isoform X3
TCONS_00011190	0.0793037	0.514789	0.427248	BGIOSGA021815	XP_022679005.1probable galactinol--sucrose galactosyltransferase 2
TCONS_00013099	0.0793156	0.785659	-0.35203	BGIOSGA005913	XP_015690047.1PREDICTED: uncharacterized protein LOC102700607
TCONS_00004310	0.0793315	0.778307	0.300496	BGIOSGA001259	XP_002458418.1RGG repeats nuclear RNA binding protein C

TCONS_00018493	0.0793538	-0.34602	0.531937	BGIOSGA018940	XP_006653970.1PREDICTED: uncharacterized protein LOC102717986 isoform X1
TCONS_00023300	0.0793551	0.601085	0.208004	BGIOSGA024648	XP_004955762.1exocyst complex component EXO84B
TCONS_00001900	0.0795001	0.384625	0.0150793	BGIOSGA004534	XP_015699269.1 protein MON2 homolog isoform X1
TCONS_00026647	0.0795374	0.700674	0.456385	BGIOSGA028715	XP_006659426.1 NADH-ubiquinone oxidoreductase 20.9 kDa subunit
TCONS_00000203	0.0795547	0.336786	0.0739793	BGIOSGA002774	XP_006643753.1 protein IWS1 homolog
TCONS_00028019	0.0796241	0.70046	0.778499	BGIOSGA026972	XP_006660129.1 DEAD-box ATP-dependent RNA helicase 29
TCONS_00027489	0.079742	3.37758	1.23187	BGIOSGA011492	XP_015695984.1 putative aconitate hydratase, cytoplasmic
TCONS_00011970	0.0798766	-1.19094	-2.21057	BGIOSGA004865	XP_006649814.1 fructose-1,6-bisphosphatase, chloroplastic
TCONS_00001872	0.0799776	0.405147	-0.583411	BGIOSGA004505	XP_006644753.1 exocyst complex component EXO70B1-like
TCONS_00022955	0.0800948	1.33427	0.197353	BGIOSGA017360	XP_002437649.1clathrin light chain 2
TCONS_00036694	0.0800958	-0.630363	0.274063	BGIOSGA003265	XP_015698657.1 transcription factor TGA6-like
TCONS_00011504	0.0801352	-0.0577015	0.564879	BGIOSGA011522	XP_004985847.1cancer-related nucleoside-triphosphatase
TCONS_00035082	0.0804258	-0.409826	0.356173	BGIOSGA035516	XP_004985754.1uncharacterized protein LOC101779601
TCONS_00008703	0.0804365	0.551981	0.805127	BGIOSGA031003	XP_015688714.1 PRELI domain containing protein 3B
TCONS_00034739	0.0804385	-0.284772	0.26471	BGIOSGA030435	XP_004979204.1protein krasavietz
TCONS_00033987	0.080496	-1.63087	-1.49246	BGIOSGA024959	XP_015696687.1 polycomb group protein EMBRYONIC FLOWER 2
TCONS_00016903	0.0807746	-0.08087	0.614587	BGIOSGA013026	XP_006653899.1 ribosome biogenesis regulatory protein homolog
TCONS_00029393	0.0808135	0.743129	1.36362	BGIOSGA022661	XP_015696681.1 probable inactive purple acid phosphatase 29
TCONS_00002105	0.0808218	0.0121316	-0.82923	BGIOSGA019052	XP_006646447.1 internal alternative NAD(P)H-ubiquinone oxidoreductase A1, mitochondrial-like
TCONS_00021256	0.0809861	-0.894518	-1.81561	BGIOSGA011222	XP_010267846.1 putative beta-glucosidase 41 isoform X3
TCONS_00024006	0.0810432	-0.140429	-0.485581	BGIOSGA032095	XP_006657900.1 formin-like protein 13
TCONS_00031398	0.0813624	0.28079	0.592563	BGIOSGA016808	XP_015697181.1 lanC-like protein GCR2
TCONS_00002567	0.0815572	0.295329	0.556841	BGIOSGA005211	XP_021312544.1RRP12-like protein
TCONS_00022857	0.0818427	0.125949	0.0791773	BGIOSGA020587	XP_006657303.1 UDP-sugar pyrophosphorylase
TCONS_00007738	0.081849	-0.0045404	-0.0546123	BGIOSGA017219	XP_006647130.1PREDICTED: uncharacterized protein LOC102713982
TCONS_00003154	0.0818826	0.0162457	-0.259262	BGIOSGA004605	XP_006643957.2 salt stress root protein RS1-like
TCONS_00003379	0.0819125	1.34365	0.268129	BGIOSGA001838	XP_010230815.1probable tRNA N6-adenosine threonylcarbamoyltransferase, mitochondrial isoform X3
TCONS_00015736	0.0819488	0.822758	0.711265	BGIOSGA006431	XP_015692156.1 ras GTPase-activating protein-binding protein 1
TCONS_00029489	0.0821012	0.793836	-0.238038	BGIOSGA027234	XP_004957602.1protein PHOX1
TCONS_00000669	0.0821092	0.241574	-1.01103	BGIOSGA003256	XP_010237097.2putative protease Do-like 14
TCONS_00013077	0.0822187	-0.395214	0.0271511	BGIOSGA011159	XP_006650507.2 protein NRT1/ PTR FAMILY 8.3-like
TCONS_00024790	0.0823151	0.241315	0.824963	BGIOSGA025907	XP_015694572.1 F-box/FBD/LRR-repeat protein At4g00160-like
TCONS_00018294	0.0824139	-1.32961	-1.10668	BGIOSGA021697	XP_006654776.1 photosystem I reaction center subunit VI, chloroplastic
TCONS_00006721	0.0825987	0.444493	0.703969	BGIOSGA023242	XP_006647768.1 ATPase family AAA domain-containing protein 3C-like
TCONS_00004966	0.0827227	0.335817	0.96078	BGIOSGA000256	XP_004971045.1E3 ubiquitin-protein ligase Smurf1 isoform X1
TCONS_00034172	0.0828233	-0.17436	0.0378339	BGIOSGA032100	XP_006664280.1 vacuolar-sorting receptor 1-like
TCONS_00008815	0.0828831	0.0355659	-0.271548	BGIOSGA022570	XP_006647896.1 alkylated DNA repair protein alkB homolog 8
TCONS_00007817	0.0828993	0.212597	0.0696699	BGIOSGA006669	XP_015689378.1PREDICTED: uncharacterized protein LOC102705977
TCONS_00005972	0.0829173	-0.138433	-0.0627453	BGIOSGA008107	XP_006664824.1 AUGMIN subunit 6 isoform X2
TCONS_00025082	0.0833159	-0.131218	0.314899	BGIOSGA024320	XP_006647244.1 protein CTR9 homolog
TCONS_00004936	0.0834065	-0.373835	1.18515	BGIOSGA010790	XP_015694821.1 histone deacetylase HDT2-like
TCONS_00037563	0.0835316	-0.337781	1.2663	BGIOSGA036577	XP_006663847.1 deoxyhypusine hydroxylase-A
TCONS_00036294	0.0835463	2.75332	2.58414	BGIOSGA037656	XP_002466839.1mitochondrial import receptor subunit TOM5 homolog
TCONS_00015067	0.083772	-0.687412	-0.582491	BGIOSGA020610	XP_006652918.1 probable E3 ubiquitin-protein ligase HIP1 isoform X1
TCONS_00013414	0.0838308	-0.48765	-0.481175	BGIOSGA028213	XP_006650792.1 serine/threonine-protein kinase HT1
TCONS_00001023	0.0838473	-0.828809	0.700952	BGIOSGA003625	XP_006644224.1 AP-3 complex subunit delta
TCONS_00009258	0.08407	-1.68118	-2.54248	BGIOSGA037562	XP_006663045.1 patatin-like protein 1
TCONS_00031391	0.0840797	0.609	0.363233	BGIOSGA033203	XP_006661906.1PREDICTED: uncharacterized protein LOC102711185
TCONS_00022526	0.0841397	1.64355	1.38078	BGIOSGA008501	XP_006656216.1 DNA-directed RNA polymerase III subunit rpc6
TCONS_00006374	0.0842665	0.488497	0.610842	BGIOSGA004558	XP_006647451.1 eukaryotic translation initiation factor 4B1
TCONS_00000830	0.0844172	1.00021	0.420137	BGIOSGA007895	XP_006645847.1 GDSL esterase/lipase At1g09390-like
TCONS_00010953	0.0845283	0.717221	0.478185	BGIOSGA004205	XP_021302677.1YTH domain-containing family protein 1 isoform X2
TCONS_00019424	0.0845652	0.0107424	0.264238	BGIOSGA017930	XP_006654503.1PREDICTED: uncharacterized protein At2g39795, mitochondrial
TCONS_00011114	0.0845727	0.161078	0.0734101	BGIOSGA018634	XP_014752719.1casein kinase 1-like protein HD16

TCONS_00019443	0.0849672	-0.611495	-1.36786	BGIOSGA025900	XP_004961835.1probable histone H2A.4
TCONS_00031589	0.0849745	0.019754	-0.273535	BGIOSGA004605	XP_006662027.1 carotene epsilon-monooxygenase, chloroplastic
TCONS_00005672	0.0850318	-0.737993	0.392752	BGIOSGA007795	XP_015688512.1 probable 28S rRNA (cytosine-C(5))-methyltransferase
TCONS_00005838	0.0852103	1.33305	#NA	BGIOSGA024549	XP_021307881.1uncharacterized protein LOC8081771
TCONS_00000220	0.0853508	0.414516	-0.58714	BGIOSGA028022	XP_006645501.1 inner membrane protein ALBINO3, chloroplastic
TCONS_00005031	0.085489	0.331502	0.30146	BGIOSGA033850	XP_006645281.2 heparan-alpha-glucosaminide N-acetyltransferase-like
TCONS_00029181	0.08552	-1.94179	-1.75777	BGIOSGA021819	XP_006660714.1PREDICTED: uncharacterized protein LOC102717924
TCONS_00022749	0.0856612	0.775424	0.655912	BGIOSGA035743	XP_006656359.1 protein REVEILLE 6-like isoform X3
TCONS_00005608	0.0857483	-0.53136	-0.303779	BGIOSGA007721	XP_006646994.1 mediator of RNA polymerase II transcription subunit 18
TCONS_00021078	0.085942	-0.4889	0.616166	BGIOSGA023305	XP_003563591.1UPF0690 protein C1orf52 homolog isoform X2
TCONS_00016437	0.0861703	-0.203074	-0.389033	BGIOSGA007108	XP_006653681.1PREDICTED: uncharacterized protein LOC102712079
TCONS_00032192	0.0863652	0.168231	0.472306	BGIOSGA032892	XP_015696991.1 protein NEN1-like
TCONS_00013548	0.0865804	-0.112948	-0.0642527	BGIOSGA009427	XP_006650904.1 26S proteasome non-ATPase regulatory subunit 12 homolog A-like
TCONS_00005565	0.0865955	0.272778	0.067105	BGIOSGA020786	NP_001169973.1putative knotted-like transcription factor family protein
TCONS_00036776	0.0866015	0.959195	0.518553	BGIOSGA036540	XP_006663864.1 monothiol glutaredoxin-S12, chloroplastic
TCONS_00019205	0.0866439	-2.24342	-1.37482	BGIOSGA019010	XP_006654341.1 annexin D4
TCONS_00003633	0.0866788	0.213434	0.875921	BGIOSGA003435	XP_006644199.1 AMSH-like ubiquitin thioesterase 3
TCONS_00024641	0.0866962	1.1867	0.354067	BGIOSGA018969	XP_006657489.1PREDICTED: uncharacterized protein LOC102719216
TCONS_00029841	0.0869639	-0.247164	0.487836	BGIOSGA019011	XP_006660543.1 putative disease resistance protein RGA1 isoform X1
TCONS_00024177	0.0870786	-2.57898	0.25005	BGIOSGA010954	NP_001147403.2hydrophobic protein LTI6A
TCONS_00015148	0.0871508	-0.355071	-0.529715	BGIOSGA000480	XP_006652995.1 reticulon-like protein B11
TCONS_00018892	0.0872519	0.479741	0.00857664	BGIOSGA020447	XP_015690988.1 zinc finger BED domain-containing protein RICESLEEPER 2
TCONS_00019452	0.0873769	0.296652	0.322985	BGIOSGA017901	XP_006655387.1 E3 ubiquitin-protein ligase UPL4 isoform X1
TCONS_00028869	0.0874114	-0.259478	-0.52268	BGIOSGA017612	NP_001140205.1uncharacterized protein LOC100272233
TCONS_00016406	0.0877189	-0.11877	0.0250597	BGIOSGA033410	XP_015691538.1 branched-chain-amino-acid aminotransferase 5, chloroplastic-like
TCONS_00031896	0.0877411	-0.678436	-1.72375	BGIOSGA032246	XP_003573808.1deoxyribodipyrimidine photo-lyase
TCONS_00009246	0.0877722	0.814048	0.209711	BGIOSGA011776	XP_006649337.1 thiol-disulfide oxidoreductase LTO1 isoform X2
TCONS_00021143	0.0878144	0.869251	0.444581	BGIOSGA009164	XP_002437354.1probable LIM domain-containing serine/threonine-protein kinase DDB_G0286997
TCONS_00021921	0.0878531	0.506128	0.376456	BGIOSGA015052	XP_006656814.2 serine/threonine-protein kinase EDR1-like
TCONS_00014219	0.0878649	-3.11595	-1.94274	BGIOSGA004853	XP_008669437.1uncharacterized LOC103646490 isoform X1
TCONS_00002617	0.088001	-0.650887	0.584378	BGIOSGA020386	XP_015699275.1 BTB/POZ domain-containing protein FBL11-like
TCONS_00001299	0.0880219	-1.73495	-1.55752	BGIOSGA021813	XP_003569313.1transcription factor MYBS3
TCONS_00008211	0.0882275	-0.0220106	0.88916	BGIOSGA026838	NP_001152223.2salt tolerant protein
TCONS_00008213	0.0882354	0.88958	-0.692455	BGIOSGA006222	XP_010235574.1thioredoxin-like 4, chloroplastic isoform X3
TCONS_00026469	0.0882665	1.87397	0.943084	BGIOSGA011700	XP_021320072.1uncharacterized protein LOC8064003
TCONS_00010649	0.0883434	0.43447	-0.152486	BGIOSGA015052	XP_004982227.1ARF guanine-nucleotide exchange factor GNOM
TCONS_00002958	0.0883663	-0.143281	-0.38398	BGIOSGA016472	XP_006647617.1 GTP-binding protein YPTM2
TCONS_00014388	0.0886315	-0.839508	-0.891587	#N/A	XP_009401412.1PREDICTED: uncharacterized protein LOC103985441
TCONS_00023360	0.0886756	0.443812	-0.111346	BGIOSGA001940	XP_004955819.1ADP-ribosylation factor 1
TCONS_00014350	0.0887025	-0.765986	-0.931618	BGIOSGA016510	XP_006652344.1 FK506-binding protein 2-like
TCONS_00007671	0.0889338	0.0508232	-0.131644	BGIOSGA007862	XP_015692361.1 altered inheritance rate of mitochondria protein 25
TCONS_00031803	0.0891336	-0.380917	-0.389298	BGIOSGA012212	XP_006661622.1 uncharacterized GTP-binding protein At5g64813
TCONS_00030620	0.089384	-0.285011	-0.366643	BGIOSGA028363	XP_015696919.1 formin-like protein 3
TCONS_00028312	0.0895178	0.0839357	0.355627	BGIOSGA019016	XP_015695702.1 spindle pole body component 110-like
TCONS_00025087	0.0895523	-0.0871757	0.12295	BGIOSGA007257	XP_006657694.1 serine carboxypeptidase-like
TCONS_00021384	0.0896346	0.0348281	-0.0568005	BGIOSGA026520	XP_006656499.1 NEP1-interacting protein-like 1
TCONS_00020675	0.0898674	0.0623154	-0.0638388	BGIOSGA008023	XP_015694312.1 nicotianamine aminotransferase A-like isoform X2
TCONS_00009354	0.0900295	0.554213	-0.465179	BGIOSGA016744	XP_004985627.1probable serine/threonine-protein kinase PBL16
TCONS_00036921	0.0900998	-0.351742	-0.0514921	BGIOSGA036406	XP_006663927.1PREDICTED: uncharacterized protein LOC102712212 isoform X2
TCONS_00017819	0.0901389	0.790315	0.724331	BGIOSGA019879	XP_015692750.1 protease Do-like 10, mitochondrial
TCONS_00028425	0.0902365	-1.35402	-0.124428	BGIOSGA016943	XP_006659655.1 RHOMBOID-like protein 2
TCONS_00032600	0.090258	1.08849	1.70348	BGIOSGA005165	XP_006661977.1 glutathione S-transferase U17-like
TCONS_00019924	0.0903352	-0.253637	0.676433	BGIOSGA024281	XP_004960972.1nucleoside diphosphate kinase 3
TCONS_00036780	0.0904425	0.430654	0.651541	BGIOSGA037759	XP_006664178.1 eukaryotic translation initiation factor 2 subunit gamma

TCONS_00033660	0.0904656	-0.596274	0.702958	BGIOSGA035440	XP_006662981.1 endothelin-converting enzyme 2
TCONS_00009830	0.0905958	0.788065	1.50991	BGIOSGA012382	XP_010228758.1cytochrome c oxidase assembly protein cox16, mitochondrial
TCONS_00026096	0.0906744	-1.43624	-0.75092	BGIOSGA025886	XP_015695988.1 probable LRR receptor-like serine/threonine-protein kinase At1g56140
TCONS_00002173	0.0907776	1.43674	-0.140071	BGIOSGA017940	XP_006645017.1 phosphatidylinositol:ceramide inositolphosphotransferase
TCONS_00022886	0.0907957	1.09727	0.0841529	BGIOSGA023585	XP_002438989.1arogenate dehydrogenase 2, chloroplastic
TCONS_00004538	0.0908026	0.449142	0.76534	BGIOSGA011244	XP_006644826.1 disease resistance protein RPS2-like
TCONS_00021296	0.0910868	-0.183158	-0.159654	BGIOSGA020067	XP_006656418.1 nifU-like protein 3, chloroplastic
TCONS_00010019	0.091133	0.899136	0.317331	BGIOSGA036761	XP_006650048.1 vacuolar-sorting receptor 1-like
TCONS_00029536	0.0911619	0.998439	-0.0162912	BGIOSGA031294	XP_006661565.2 R3H domain-containing protein 1-like
TCONS_00023119	0.0913369	-0.299481	-0.503637	BGIOSGA024575	XP_006658281.2 40S ribosomal protein S12
TCONS_00033325	0.0914407	0.950913	0.745058	BGIOSGA035155	XP_006664857.1 mediator of RNA polymerase II transcription subunit 15-like
TCONS_00004297	0.0915254	-0.0140813	-0.149969	BGIOSGA000911	XP_006644623.1 probable choline kinase 2 isoform X1
TCONS_00015932	0.0915439	-0.347459	0.623357	BGIOSGA015053	XP_008662249.1OSJNBa0006B20.20-like protein isoform X1
TCONS_00023989	0.0915531	-1.13452	-0.108864	BGIOSGA012031	XP_006657890.1 putative yippee-like protein Os10g0369500
TCONS_00034380	0.0917271	0.36887	0.723813	BGIOSGA036558	XP_015698755.1 V-type proton ATPase 16 kDa proteolipid subunit-like
TCONS_00025205	0.0918256	0.570448	0.290103	BGIOSGA030818	XP_015694654.1 phospholipase A I
TCONS_00008226	0.0918793	0.804035	-0.300574	BGIOSGA028831	XP_015689153.1 E3 ubiquitin-protein ligase RING1-like
TCONS_00021974	0.0918968	-3.34932	-1.83457	BGIOSGA016278	XP_015694446.1 endoglucanase 16 isoform X3
TCONS_00015131	0.092148	0.256566	0.626557	BGIOSGA017310	XP_008661409.1uncharacterized LOC100193333 isoform X1
TCONS_00016466	0.0922358	0.858913	-0.449653	BGIOSGA005839	XP_006652686.1 scarecrow-like protein 8
TCONS_00018864	0.0922912	-0.0364712	-0.0689439	BGIOSGA018554	XP_006655109.1 DNA (cytosine-5)-methyltransferase 1-like
TCONS_00015762	0.0924943	0.00483786	-0.0997988	BGIOSGA000237	XP_006652203.1 stearoyl-[acyl-carrier-protein] 9-desaturase 5, chloroplastic
TCONS_00027879	0.0928405	0.088896	-0.338908	#N/A	#N/A
TCONS_00014374	0.0929592	-0.537445	-0.772156	BGIOSGA032833	NP_001150220.1wound/stress protein precursor
TCONS_00003215	0.0929861	#NA	0.23183	BGIOSGA002003	XP_006643989.2 receptor-like serine/threonine-protein kinase At1g78530
TCONS_00014320	0.0930728	-0.357986	-0.170413	BGIOSGA008513	XP_002447913.1trihelix transcription factor ASIL2
TCONS_00013426	0.0931619	-0.78694	-0.533208	BGIOSGA005285	XP_004981265.1protein SODIUM POTASSIUM ROOT DEFECTIVE 2
TCONS_00015760	0.0931734	-0.291794	0.0943079	BGIOSGA023009	XP_021308940.1uncharacterized protein LOC8054862
TCONS_00027665	0.0931966	0.802372	0.218099	BGIOSGA027346	XP_003573618.1uncharacterized protein LOC100831568
TCONS_00019102	0.0932498	0.2851	0.144643	BGIOSGA009563	XP_008649553.1CASP-like protein 4B4
TCONS_00020755	0.0934484	0.565236	1.35512	BGIOSGA007658	XP_002453821.1F-box/LRR-repeat protein 13
TCONS_00023456	0.093475	-0.0642134	-0.450066	BGIOSGA016259	XP_006658457.1PREDICTED: uncharacterized protein LOC102699882
TCONS_00018289	0.0935266	0.476994	0.045139	BGIOSGA027620	XP_006662577.1 branched-chain-amino-acid aminotransferase 5, chloroplastic-like
TCONS_00007108	0.093546	0.430121	0.179889	BGIOSGA005422	XP_006648113.2 NADH dehydrogenase [ubiquinone] 1 alpha subcomplex subunit 9, mitochondrial isoform X1
TCONS_00018137	0.093595	-0.0190603	0.600784	BGIOSGA030715	XP_015693179.1 probable alpha,alpha-trehalose-phosphate synthase [UDP-forming] 7
TCONS_00010616	0.093723	-0.0840582	0.631485	BGIOSGA013239	XP_006650366.1 3-isopropylmalate dehydrogenase 2, chloroplastic-like
TCONS_00015241	0.0937678	1.2616	1.01213	BGIOSGA015739	XP_015692082.1 eukaryotic translation initiation factor 3 subunit M-like
TCONS_00014353	0.0938222	-0.159943	0.31608	BGIOSGA016513	XP_015692398.1PREDICTED: uncharacterized protein LOC102715387 isoform X2
TCONS_00021985	0.0938222	-0.822908	0.24208	BGIOSGA031855	XP_015693906.1 BTB/POZ and MATH domain-containing protein 1-like
TCONS_00001576	0.0938407	-0.221612	-0.00910444	BGIOSGA004208	XP_006644524.1PREDICTED: uncharacterized protein LOC102708567
TCONS_00021797	0.0938473	0.277854	1.4416	BGIOSGA029856	XP_015693484.1 pentatricopeptide repeat-containing protein At3g18110, chloroplastic
TCONS_00016263	0.0938578	0.505564	-0.235723	BGIOSGA005283	XP_015692254.1 myb-related protein Myb4-like
TCONS_00006039	0.0938816	-1.14975	-0.258489	BGIOSGA021450	XP_006656881.1 DNA topoisomerase 1
TCONS_00016332	0.0939979	0.761834	0.578679	BGIOSGA002861	XP_006652593.1 probable receptor-like protein kinase At1g33260
TCONS_00036701	0.0940753	0.536859	0.473473	BGIOSGA006048	XP_006663828.1 UDP-galactose transporter 2-like
TCONS_00002903	0.0944989	-1.03153	0.303283	BGIOSGA008656	XP_006643793.1 uncharacterized membrane protein At1g06890
TCONS_00009564	0.0945174	-0.293483	0.345666	BGIOSGA029476	XP_015689815.1 probable inactive purple acid phosphatase 1
TCONS_00036707	0.0945455	-1.65849	-0.861851	BGIOSGA008181	XP_006663830.1 rho GTPase-activating protein 4-like
TCONS_00008883	0.0945491	1.00534	1.49721	BGIOSGA028623	XP_006665059.1 mediator-associated protein 1-like
TCONS_00016133	0.0945646	0.618366	0.249678	BGIOSGA027403	XP_006652433.1 F-box protein SKIP14
TCONS_00014629	0.0946341	0.497998	0.799494	BGIOSGA022587	XP_004976282.1protein RETICULATA-RELATED 1, chloroplastic
TCONS_00019558	0.0946391	-3.48923	#NA	BGIOSGA017800	XP_006655447.2 eukaryotic translation initiation factor 3 subunit J-like
TCONS_00035175	0.0949137	0.318676	0.997071	BGIOSGA011471	XP_006663044.1 SEC12-like protein 2

TCONS_00002758	0.0949282	0.134536	0.848181	BGIOSGA002458	XP_015692812.1PREDICTED: uncharacterized protein LOC102704932
TCONS_00021429	0.0949347	-0.157655	-0.377218	BGIOSGA023652	XP_006644463.1 coiled-coil domain-containing protein 12-like
TCONS_00031592	0.094992	0.0696019	0.136239	BGIOSGA024578	XP_006662028.1 mediator of RNA polymerase II transcription subunit 32
TCONS_00015754	0.0950955	-0.348043	0.50644	BGIOSGA015225	XP_006652199.1 dnaJ homolog subfamily C member 2
TCONS_00016015	0.0951442	-0.184538	0.267655	BGIOSGA035171	XP_006652371.1 probable ran guanine nucleotide release factor
TCONS_00026014	0.0953198	-0.108398	0.533095	BGIOSGA025888	XP_006659889.1 vacuolar protein sorting-associated protein 18 homolog
TCONS_00019331	0.0953664	0.474673	0.387784	BGIOSGA004910	XP_015693038.1 cleavage stimulation factor subunit 2 tau variant isoform X2
TCONS_00021718	0.0954598	-1.088	0.238022	BGIOSGA013442	XP_003561022.2probable glycosyltransferase At5g25310
TCONS_00022239	0.0954998	-0.334272	-0.141576	BGIOSGA021260	XP_006656085.1 probable WRKY transcription factor 19
TCONS_00012974	0.0955621	0.443633	0.198618	BGIOSGA029486	XP_015691103.1 scarecrow-like protein 9
TCONS_00034126	0.0957364	1.89811	-1.8222	BGIOSGA034644	XP_006662652.1 ferritin-1, chloroplastic-like
TCONS_00024258	0.0957598	-0.0959373	-0.111859	BGIOSGA026296	XP_006658053.2 TVP38/TMEM64 family membrane protein slr0305
TCONS_00004674	0.0958658	-3.9954	-0.788516	BGIOSGA000798	XP_014755194.1interactor of constitutive active ROPs 2, chloroplastic isoform X2
TCONS_00029813	0.0958658	-2.60309	-1.20355	BGIOSGA030006	XP_004956563.1glutathione S-transferase T3
TCONS_00009506	0.0958715	-1.77965	-0.938079	BGIOSGA030677	XP_006649573.1PREDICTED: uncharacterized protein LOC102705987
TCONS_00015353	0.0959406	0.200904	-0.244988	BGIOSGA020873	XP_006652098.1 protein YIF1B-like
TCONS_00027383	0.0960172	0.128528	-0.966888	BGIOSGA027621	NP_001140781.1putative FAD-binding Berberine family protein precursor
TCONS_00005125	0.0962044	0.00262044	0.138492	BGIOSGA000079	NP_001105732.1chloroplast srp54 receptor 1
TCONS_00020219	0.0963381	0.120972	0.419193	BGIOSGA022410	XP_006655845.1 cystathionine beta-lyase, chloroplastic
TCONS_00004890	0.0964162	-0.85521	-0.939651	BGIOSGA000331	XP_004970893.1uncharacterized protein LOC101764087
TCONS_00027647	0.0964927	-1.2413	-1.2051	BGIOSGA027367	XP_006659248.1 probable receptor-like serine/threonine-protein kinase At5g57670
TCONS_00018657	0.0965	1.33082	0.0810513	BGIOSGA003166	XP_006655009.1 importin subunit alpha-1b
TCONS_00005232	0.0965808	-0.0235837	-0.264982	BGIOSGA022106	XP_006648210.2 BAG family molecular chaperone regulator 7
TCONS_00006327	0.0967889	0.123	0.0576745	BGIOSGA008491	XP_002452338.1uncharacterized endoplasmic reticulum membrane protein YGL010W
TCONS_00025753	0.0970492	0.301948	0.277083	BGIOSGA023242	XP_006658170.1 26S protease regulatory subunit 4 homolog
TCONS_00016237	0.0970647	-0.469604	0.50555	BGIOSGA014737	XP_006653593.1PREDICTED: uncharacterized protein LOC102706841
TCONS_00028247	0.0971603	0.649349	0.561001	BGIOSGA037824	XP_003574712.1COP9 signalosome complex subunit 6a
TCONS_00014559	0.0971923	0.306149	0.696031	BGIOSGA005392	XP_006652482.1 calmodulin-binding receptor-like cytoplasmic kinase 3
TCONS_00011661	0.0973107	-1.39886	-2.57288	BGIOSGA010821	XP_015689784.1PREDICTED: uncharacterized protein LOC102702269, partial
TCONS_00018312	0.0973479	-0.0423458	0.471379	BGIOSGA001057	XP_022680692.1probable E3 ubiquitin-protein ligase ZFP1
TCONS_00009658	0.0973938	0.266109	0.419936	BGIOSGA014375	XP_006649705.1 protein OBERON 3-like
TCONS_00008680	0.0973975	0.606224	0.378271	BGIOSGA005750	XP_006647787.2 protease Do-like 7 isoform X1
TCONS_00036391	0.0974143	-0.0934107	0.680104	BGIOSGA012214	XP_015698360.1 probable ubiquitin-like-specific protease 2B
TCONS_00021967	0.097453	0.721475	0.766167	BGIOSGA021549	XP_006655961.2 alpha-mannosidase 2
TCONS_00012646	0.0975017	0.975258	0.85092	BGIOSGA010327	XP_006651579.2 DUF21 domain-containing protein At1g55930, chloroplastic-like
TCONS_00018837	0.0975508	-5.32243	-3.00933	BGIOSGA002110	XP_006654158.1 bidirectional sugar transporter SWEET3a
TCONS_00037517	0.0976279	-0.422895	-0.734744	BGIOSGA035848	XP_006664187.1 WPP domain-associated protein-like isoform X1
TCONS_00035891	0.0976896	-0.168065	0.439241	BGIOSGA037264	XP_006663956.1 WD repeat-containing protein 89 homolog isoform X1
TCONS_00020056	0.097724	0.592055	0.63555	BGIOSGA022238	XP_006656599.1 protein DECREASED SIZE EXCLUSION LIMIT 1
TCONS_00021273	0.0977809	-0.615548	-0.823161	BGIOSGA022635	XP_002437483.1protein IRX15-LIKE
TCONS_00003988	0.0981735	0.657409	1.31897	BGIOSGA017537	XP_006644371.1 CTP synthase-like
TCONS_00003298	0.098216	-1.19716	-0.183703	BGIOSGA032060	XP_006644043.1 glutamate--tRNA ligase, cytoplasmic-like
TCONS_00014691	0.098278	0.699668	-0.173873	BGIOSGA025432	XP_006652598.1 glutamate dehydrogenase A-like
TCONS_00024986	0.0983017	-1.32828	-0.3517	BGIOSGA024409	XP_006657637.1PREDICTED: uncharacterized protein LOC102713833 isoform X3
TCONS_00004615	0.0983647	-0.568311	-0.295106	BGIOSGA003893	XP_006644897.1 NADH--cytochrome b5 reductase 1
TCONS_00007094	0.0983751	-0.958823	-0.175789	BGIOSGA005145	XP_006648098.1 cytochrome c1 2, heme protein, mitochondrial-like isoform X1
TCONS_00034132	0.0987519	-0.32265	1.28147	BGIOSGA015908	XP_006663720.1PREDICTED: uncharacterized protein LOC102720754
TCONS_00034978	0.0989867	-3.59074	-1.73475	BGIOSGA032589	XP_006662967.1 chalcone synthase 1
TCONS_00030546	0.0990875	1.7492	0.474408	BGIOSGA030272	XP_015696457.1 28 kDa ribonucleoprotein, chloroplastic-like
TCONS_00033382	0.0992482	0.936891	1.67146	BGIOSGA017670	XP_002458100.1pentatricopeptide repeat-containing protein At3g49240
TCONS_00002273	0.099287	-0.729946	-0.0473466	BGIOSGA015756	XP_021313085.1phytolongin Phyl1.1
TCONS_00033245	0.0993656	0.152455	0.765797	BGIOSGA025013	XP_006662858.1 probably inactive leucine-rich repeat receptor-like protein kinase At5g48380
TCONS_00030900	0.0994077	0.549138	0.398694	BGIOSGA032704	XP_006644149.1 mevalonate kinase
TCONS_00020093	0.099451	-0.806093	-0.698616	BGIOSGA004551	XP_022685112.1uncharacterized protein LOC101754700 isoform X1
TCONS_00019633	0.0995014	-2.98655	-0.356009	BGIOSGA016471	XP_006654650.1 ras-related protein RIC2-like

TCONS_00009378	0.0995263	0.657525	0.347757	BGIOSGA011928	XP_006651066.1 2-dehydro-3-deoxygluconokinase
TCONS_00009925	0.0996316	0.411773	0.228067	BGIOSGA037754	XP_006647081.1 pentatricopeptide repeat-containing protein At3g06430, chloroplastic
TCONS_00004205	0.099906	0.614805	0.0572943	BGIOSGA029797	XP_002278705.1 zinc finger protein BRUTUS isoform X1
TCONS_00021653	0.10009	0.71301	0.293253	BGIOSGA021867	XP_006655815.1 rRNA-processing protein FCF1 homolog
TCONS_00016605	0.100133	-0.334242	-0.214275	BGIOSGA028186	XP_006653762.1 UDP-N-acetylglucosamine diphosphorylase 2-like
TCONS_00030050	0.100223	0.128158	-1.52804	BGIOSGA019646	XP_003578125.1WRKY transcription factor WRKY76
TCONS_00013639	0.100277	0.165086	0.746409	BGIOSGA015794	XP_006652078.1 amidase 1-like
TCONS_00031462	0.100306	-0.160885	0.989786	BGIOSGA033279	XP_006661958.2 electron transfer flavoprotein-ubiquinone oxidoreductase, mitochondrial isoform X1
TCONS_00033160	0.101093	#NA	#NA	BGIOSGA023267	XP_004951581.1TBCC domain-containing protein 1
TCONS_00009833	0.101115	0.464793	0.428456	BGIOSGA012385	XP_006649902.1 transmembrane protein 209
TCONS_00000656	0.10113	0.10955	0.191853	BGIOSGA010870	XP_003565639.1BSD domain-containing protein 1
TCONS_00006707	0.101203	-1.3788	-1.4077	BGIOSGA023422	XP_006647740.1 myb family transcription factor APL-like
TCONS_00029979	0.101257	0.380081	0.242238	BGIOSGA029829	XP_006660597.2 AUGMIN subunit 5
TCONS_00027628	0.101261	-0.237884	-0.72063	BGIOSGA027371	XP_006659236.1 ACT domain-containing protein DS12, chloroplastic isoform X2
TCONS_00006858	0.101565	-0.398611	-0.0258749	BGIOSGA021605	XP_006647877.1 NEP1-interacting protein-like 2
TCONS_00021321	0.101641	-0.913103	-0.639256	BGIOSGA017624	XP_006657301.1 putative clathrin assembly protein At5g57200
TCONS_00023281	0.101668	0.276166	1.08634	BGIOSGA025326	XP_006657528.1 basic proline-rich protein
TCONS_00010187	0.102195	-0.0724966	-0.292702	BGIOSGA033474	XP_006650137.1 probable methyltransferase PMT2
TCONS_00000316	0.102594	0.308391	0.461787	BGIOSGA011243	XP_006643831.2PREDICTED: uncharacterized protein LOC102715339
TCONS_00022234	0.102605	-0.0460088	-0.136527	BGIOSGA021267	XP_006656073.1PREDICTED: uncharacterized protein LOC102716222
TCONS_00000676	0.102656	-0.649373	-0.0387629	BGIOSGA031307	XP_015688222.1 probable anion transporter 1, chloroplastic
TCONS_00031021	0.102706	-0.652519	0.0416417	BGIOSGA032833	XP_006662311.2PREDICTED: uncharacterized protein LOC102722149
TCONS_00018460	0.102887	0.652087	1.13037	BGIOSGA018975	XP_006653944.1 eukaryotic translation initiation factor 3 subunit F
TCONS_00021416	0.103066	0.215013	0.414596	BGIOSGA023642	XP_015694182.1 epidermal growth factor receptor substrate 15-like 1 isoform X2
TCONS_00029544	0.10313	1.09998	0.625562	BGIOSGA034902	XP_006661001.1 U-box domain-containing protein 33-like
TCONS_00023325	0.10316	2.20025	0.768094	BGIOSGA025367	XP_006657543.1 cytochrome b-c1 complex subunit 7-like
TCONS_00026531	0.103246	-0.123221	0.3001	BGIOSGA028593	XP_006659360.1 golgin candidate 4-like
TCONS_00014346	0.103405	-0.262079	-1.63839	BGIOSGA016511	XP_006652342.1 UPF0483 protein AGAP003155-like
TCONS_00023397	0.103407	0.399729	0.159169	BGIOSGA025435	XP_006658423.1 protein RETICULATA, chloroplastic-like
TCONS_00007138	0.103476	0.598413	-0.217343	BGIOSGA031308	XP_015689461.1PREDICTED: LOW QUALITY PROTEIN: uncharacterized protein LOC102704866
TCONS_00013214	0.103544	1.11256	-0.84537	BGIOSGA020074	XP_006650630.1 probable protein phosphatase 2C 34
TCONS_00025598	0.103571	0.177776	0.417553	BGIOSGA017475	XP_002463267.1calcium/calmodulin-dependent serine/threonine-protein kinase 1
TCONS_00026808	0.103776	-0.0703507	0.670264	BGIOSGA025331	XP_015690776.1 carbon catabolite-derepressing protein kinase-like
TCONS_00026992	0.103879	0.331924	1.18542	BGIOSGA006714	XP_015696172.1 uridine kinase-like protein 1, chloroplastic
TCONS_00005734	0.103975	0.11329	0.682755	BGIOSGA007850	XP_006654147.1 60S ribosomal protein L11
TCONS_00034120	0.104034	-1.56772	-1.52481	BGIOSGA027711	XP_004961135.1protein CHUP1, chloroplastic
TCONS_00022084	0.104069	0.698136	0.357612	BGIOSGA025917	XP_006656003.1 sister chromatid cohesion protein PDS5 homolog A
TCONS_00016452	0.104263	0.25422	0.346169	BGIOSGA022225	XP_003580353.1probable methyltransferase PMT15
TCONS_00011220	0.104325	0.689174	1.16444	BGIOSGA013823	XP_003563748.1protein FLC EXPRESSOR
TCONS_00032672	0.104398	0.503145	-0.648507	BGIOSGA023704	XP_006662038.1 probable trehalose-phosphate phosphatase 2
TCONS_00003294	0.104568	-0.770565	-0.551527	BGIOSGA001919	XP_015692735.1 actin-related protein 6
TCONS_00004451	0.104935	0.115909	-0.697068	BGIOSGA008683	XP_004970055.1transcription factor PCF5
TCONS_00028887	0.104984	0.872774	0.0938802	BGIOSGA007204	XP_006659387.1 hexuronate transporter
TCONS_00027437	0.105184	-2.29677	-1.80225	BGIOSGA017213	XP_003573421.1uncharacterized protein LOC100823080
TCONS_00016706	0.105294	-1.01097	-0.592224	BGIOSGA014258	XP_015691839.1 putative E3 ubiquitin-protein ligase RING1a isoform X2
TCONS_00011163	0.10531	0.329509	-0.308513	BGIOSGA035823	XP_004981369.1serine/threonine-protein phosphatase PP2A-2 catalytic subunit
TCONS_00036078	0.105531	-0.903222	-0.113589	BGIOSGA037442	XP_006664044.1 nitrilase-like protein 2
TCONS_00029638	0.105622	0.240053	-0.255169	BGIOSGA029878	XP_015696827.1 serine/threonine-protein kinase 16-like isoform X1
TCONS_00003188	0.105649	-0.506092	-0.2112	BGIOSGA002028	XP_006643982.1 D-tyrosyl-tRNA(Tyr) deacylase
TCONS_00024620	0.105815	-0.266508	-0.206678	BGIOSGA024766	XP_006657473.1 syntaxin-132
TCONS_00030718	0.105819	-1.29432	0.495441	BGIOSGA018480	XP_015689722.1PREDICTED: uncharacterized protein LOC107303725 isoform X2
TCONS_00020519	0.105857	0.0710427	-0.0811658	BGIOSGA023592	XP_015694339.1 kanadaptin
TCONS_00012168	0.105898	0.126524	0.265663	BGIOSGA014979	XP_008643716.1uncharacterized protein LOC100382001 isoform X1
TCONS_00023754	0.1059	-0.813688	0.347665	BGIOSGA024780	XP_006657762.1 calcium-dependent protein kinase isoform 2
TCONS_00030508	0.106378	-0.745997	-1.54499	BGIOSGA022286	NP_001148281.2PRLI-interacting factor A

TCONS_00016053	0.106396	-0.00717554	0.153008	BGIOSGA014924	XP_006653504.1 poly(A)-specific ribonuclease PARN
TCONS_00013305	0.10642	0.553032	-0.260257	BGIOSGA024778	XP_010228727.1myosin-binding protein 7
TCONS_00002464	0.106422	-0.464231	-0.19023	BGIOSGA019719	XP_006646644.1 beta-glucosidase 5-like
TCONS_00010819	0.106517	-0.167629	0.24079	BGIOSGA004209	XP_006651745.1 dynamin-related protein 1C
TCONS_00007013	0.106912	-1.23825	-1.2088	BGIOSGA009195	XP_002452968.1probable steroid-binding protein 3
TCONS_00015118	0.107199	1.28449	2.77747	BGIOSGA017297	XP_015691605.1 molybdopterin biosynthesis protein CNX1
TCONS_00033017	0.107199	#NA	#NA	BGIOSGA037037	XP_015698033.1 transcription factor VIP1-like
TCONS_00017135	0.107199	-0.300475	#NA	#N/A	#N/A
TCONS_00007349	0.107386	-0.44313	-0.549131	BGIOSGA017551	XP_015689349.1 mitogen-activated protein kinase 3
TCONS_00021377	0.107434	0.456599	-0.0364855	BGIOSGA029015	XP_006656491.1 translocon-associated protein subunit alpha
TCONS_00016818	0.107524	0.485526	1.23353	BGIOSGA014146	XP_006653858.1 N-acylphosphatidylethanolamine synthase
TCONS_00021018	0.107682	0.0536782	-0.39384	BGIOSGA028236	XP_002437266.1subtilisin-like protease SBT5.3 isoform X2
TCONS_00009938	0.108131	0.659877	0.859664	BGIOSGA007567	XP_015689702.1 GPN-loop GTPase 1
TCONS_00008481	0.10822	-0.420009	0.158953	BGIOSGA023195	XP_006663091.1 palmitoyl-acyl carrier protein thioesterase, chloroplastic-like
TCONS_00020270	0.108401	0.415257	-1.48693	BGIOSGA022461	XP_008658694.1tac7077 isoform X1
TCONS_00005120	0.108425	0.0452775	-0.793092	BGIOSGA000083	XP_002459121.1U-box domain-containing protein 14
TCONS_00001655	0.108466	-0.261237	-0.3726	BGIOSGA028930	XP_006646251.1 chloride channel protein CLC-e
TCONS_00012370	0.108581	0.267943	1.23808	BGIOSGA037982	XP_006651450.1 probable ribosomal protein S11, mitochondrial
TCONS_00022854	0.108596	0.000295753	-0.227047	BGIOSGA007653	XP_006656441.1PREDICTED: uncharacterized protein LOC102705923
TCONS_00037299	0.108869	-0.519307	-0.286236	BGIOSGA014525	XP_015698319.1 bifunctional 3-dehydroquinate dehydratase/shikimate dehydrogenase, chloroplastic-like
TCONS_00019426	0.108929	#NA	-1.83874	BGIOSGA032431	XP_006654504.1 aldo-keto reductase family 4 member C10-like
TCONS_00007401	0.109135	0.537602	0.040721	BGIOSGA023459	XP_015689591.1PREDICTED: uncharacterized protein LOC102716758
TCONS_00023224	0.109483	0.518074	0.734848	BGIOSGA031055	XP_006657507.2 transcription factor UNE12-like
TCONS_00016220	0.109526	-2.00813	-0.133619	BGIOSGA014753	XP_006653586.1 2-phytyl-1,4-beta-naphthoquinone methyltransferase, chloroplastic isoform X1
TCONS_00033459	0.109569	0.807707	-0.302641	BGIOSGA000293	XP_015697758.1 TBC1 domain family member 13-like
TCONS_00015869	0.109579	-0.0135353	0.220672	BGIOSGA006296	XP_015691665.1 WD repeat-containing protein 44-like
TCONS_00005361	0.109647	1.0305	0.330359	BGIOSGA020334	XP_004951978.1proteasome subunit alpha type-1
TCONS_00006509	0.109676	0.973473	0.164875	BGIOSGA008619	XP_002452493.1uncharacterized protein LOC8066188
TCONS_00012346	0.110086	-0.769941	-0.112994	BGIOSGA010643	XP_015689916.1 cystathionine gamma-synthase 1, chloroplastic-like
TCONS_00016891	0.110305	-0.328242	-0.570079	BGIOSGA005526	XP_015692368.1 cysteine protease ATG4B-like
TCONS_00036847	0.110305	-0.409602	-0.169722	BGIOSGA036466	XP_002465541.1disease resistance protein RPS2
TCONS_00032392	0.110404	0.666581	0.670514	BGIOSGA017029	XP_006661845.2 pre-mRNA-processing factor 19
TCONS_00020634	0.110483	-1.33583	-2.25763	BGIOSGA034133	XP_002436975.1galactan beta-1,4-galactosyltransferase GALS1
TCONS_00018640	0.110488	-0.204573	-0.0820175	BGIOSGA018794	XP_006654053.1 double-stranded RNA-binding protein 2-like
TCONS_00005679	0.110609	0.295502	-0.192711	BGIOSGA019086	XP_006647076.1 tetraspanin-2-like
TCONS_00033026	0.110657	0.751419	-0.128773	BGIOSGA034451	XP_024310550.1putative disease resistance protein RGA4 isoform X1
TCONS_00027218	0.11106	-1.33383	-0.296999	BGIOSGA006850	XP_022683178.1pectinesterase inhibitor 28-like
TCONS_00030554	0.111116	0.699003	-0.799759	BGIOSGA012068	XP_006661559.1PREDICTED: uncharacterized protein LOC102715790
TCONS_00032696	0.111117	0.401134	-0.110556	BGIOSGA031412	XP_015697330.1PREDICTED: uncharacterized protein LOC102709312 isoform X3
TCONS_00031947	0.111128	0.406463	-0.829548	BGIOSGA005188	XP_015697795.1PREDICTED: uncharacterized protein LOC102706976 isoform X1
TCONS_00014539	0.111149	-0.307524	#NA	BGIOSGA004432	XP_015693948.1 (R)-mandelonitrile lyase-like
TCONS_00021579	0.111149	#NA	-2.40249	BGIOSGA011798	XP_004964501.1uncharacterized protein LOC101784438
TCONS_00012268	0.111172	0.773518	0.31848	BGIOSGA011790	XP_006651374.1 nuclear pore complex protein NUP93A-like
TCONS_00013468	0.111215	0.0274776	0.669873	BGIOSGA033212	XP_015690191.1 bifunctional epoxide hydrolase 2-like
TCONS_00007287	0.111393	0.868305	0.589481	BGIOSGA007195	XP_006648278.1 protein TRI1-like
TCONS_00009393	0.111418	-0.426245	-2.03848	BGIOSGA031945	NP_001148673.1ribosomal protein L32 containing protein
TCONS_00028372	0.111424	1.66299	1.26361	BGIOSGA026591	XP_006659618.1PREDICTED: uncharacterized protein LOC102699889
TCONS_00008745	0.111594	-2.70827	-2.23316	BGIOSGA005687	XP_015688987.1 calcium sensing receptor, chloroplastic
TCONS_00015801	0.111687	0.223679	0.696641	BGIOSGA015183	XP_006652226.1 poly(A) polymerase I-like
TCONS_00032313	0.111697	1.58526	0.821118	BGIOSGA006963	XP_004982996.1rubisco accumulation factor 1, chloroplastic-like
TCONS_00012283	0.111713	0.820626	0.137458	BGIOSGA010722	XP_006650091.1 probable diphthine methyl ester synthase
TCONS_00020336	0.111734	0.668267	0.0639869	BGIOSGA026688	XP_015693818.1 aminoacylase-1-like isoform X1
TCONS_00029407	0.111802	0.0954777	-0.0909579	BGIOSGA019674	NP_001169272.1ARO1-like protein 2
TCONS_00023913	0.111854	-0.245426	0.132347	BGIOSGA000699	XP_015694822.1 sphingosine kinase 1-like

TCONS_00018474	0.111917	-1.05085	-0.65419	BGIOSGA005243	XP_006653954.1 ABC transporter D family member 1
TCONS_00000364	0.11206	-0.852378	-0.561079	BGIOSGA013293	XP_008655733.1putative transcription factor bHLH041
TCONS_00008611	0.112106	0.840414	0.176118	BGIOSGA004883	XP_006647706.1 phosphatidylinositol glycan anchor biosynthesis class U protein-like
TCONS_00030421	0.112206	-1.9133	0.0592892	BGIOSGA034841	XP_006660883.1 two-component response regulator-like PRR95
TCONS_00036184	0.112232	-0.0398527	-1.21749	BGIOSGA001788	XP_004957251.1probable sarcosine oxidase
TCONS_00027079	0.112291	1.40786	1.24442	BGIOSGA009379	XP_006659664.1 proteasome subunit alpha type-7-A
TCONS_00039844	0.112533	-0.987765	0.312276	BGIOSGA031206	XP_004976668.1phosphoribulokinase, chloroplastic
TCONS_00000279	0.112644	0.477324	-0.610863	BGIOSGA019249	XP_015698988.1 autophagy-related protein 18d-like isoform X2
TCONS_00001470	0.112679	0.410071	0.621218	BGIOSGA004733	XP_006644438.1 malate dehydrogenase, mitochondrial-like
TCONS_00005569	0.112787	0.133771	0.306547	BGIOSGA028425	XP_006646988.1 probable sucrose-phosphate synthase 2
TCONS_00020363	0.11279	-3.39387	-1.20765	BGIOSGA005920	XP_006655916.1 blue copper protein-like
TCONS_00031684	0.112838	1.09169	0.551492	BGIOSGA033502	XP_015697455.1 glycerol-3-phosphate acyltransferase, chloroplastic
TCONS_00018836	0.112858	0.567724	-0.447853	#N/A	#N/A
TCONS_00008689	0.112868	-0.684105	0.759197	BGIOSGA028505	XP_006648988.1 pyrophosphate--fructose 6-phosphate 1-phosphotransferase subunit alpha-like
TCONS_00017281	0.112868	0.0972543	0.118526	BGIOSGA030717	XP_015692509.1 diacylglycerol O-acyltransferase 1-like
TCONS_00004313	0.112888	-0.419128	-0.759272	BGIOSGA014966	XP_006644646.1 putative ABC transporter B family member 8
TCONS_00002170	0.113007	0.750447	-0.166972	BGIOSGA030289	XP_015699217.1PREDICTED: uncharacterized protein LOC102710642 isoform X3
TCONS_00023926	0.11306	0.274109	1.1776	BGIOSGA003645	XP_015693233.1 probable manganese-transporting ATPase PDR2
TCONS_00033631	0.11307	0.537829	0.329567	BGIOSGA013429	XP_006662988.1 14-3-3-like protein GF14-D
TCONS_00020355	0.113273	0.87695	-0.0705837	BGIOSGA005572	XP_015693499.1PREDICTED: uncharacterized protein LOC102717445
TCONS_00000604	0.113287	-0.137708	0.127781	BGIOSGA019279	XP_010230731.1putative disease resistance protein RGA3
TCONS_00005163	0.113317	1.30379	0.444439	BGIOSGA006711	XP_006646728.2 proteinaceous RNase P 2
TCONS_00021466	0.113335	-2.79614	0.73911	BGIOSGA026749	XP_004964304.1E3 ubiquitin-protein ligase RHA1B
TCONS_00032467	0.11358	0.42385	1.05852	BGIOSGA031653	XP_014757242.1DNA ligase 1
TCONS_00001314	0.113727	0.511215	0.619578	BGIOSGA003947	XP_015689221.1PREDICTED: uncharacterized protein LOC102703543
TCONS_00023188	0.113752	-0.620741	-0.0608227	BGIOSGA025229	XP_006658317.1 folate-biopterin transporter 1, chloroplastic-like
TCONS_00006813	0.113795	0.532011	-0.0887538	BGIOSGA009273	XP_006647846.1 3-ketoacyl-CoA synthase 5-like
TCONS_00023625	0.113912	0.0600391	-0.0880862	BGIOSGA025649	XP_015694623.1 acyl-coenzyme A thioesterase 13-like
TCONS_00016026	0.11397	1.81072	1.29409	BGIOSGA022794	XP_002447973.1prohibitin-3, mitochondrial
TCONS_00005430	0.114075	0.368853	0.323526	BGIOSGA010036	XP_006646885.1 GATA transcription factor 17-like isoform X1
TCONS_00008950	0.114094	0.209525	0.443445	BGIOSGA005457	XP_006649120.1 autophagy-related protein 18b isoform X1
TCONS_00002907	0.114188	0.0759748	0.192671	BGIOSGA018735	XP_015691467.1 protein NETWORKED 4B-like
TCONS_00038002	0.114307	0.0615637	1.23037	BGIOSGA034459	XP_006653448.1 chaperonin 60 subunit beta 4, chloroplastic-like
TCONS_00023619	0.114477	0.16762	-0.338888	BGIOSGA006305	XP_021309522.1ultraviolet-B receptor UVR8
TCONS_00024980	0.114522	-1.26801	-1.03456	BGIOSGA015744	XP_006657631.1PREDICTED: uncharacterized protein LOC102713007 isoform X3
TCONS_00007888	0.114597	-0.0381145	-0.537255	BGIOSGA036403	XP_006648597.1 ABC transporter G family member 40 isoform X1
TCONS_00011678	0.114805	0.485168	0.434656	BGIOSGA011330	XP_006649509.1 eukaryotic translation initiation factor 3 subunit K
TCONS_00032605	0.114846	-1.24335	-2.47252	BGIOSGA031599	XP_006661987.1 probable glutathione S-transferase GSTU6
TCONS_00004342	0.114921	-2.31407	0.494813	BGIOSGA008289	XP_015699162.1 mitochondrial import receptor subunit TOM7-1-like
TCONS_00022907	0.114921	#NA	1.17289	BGIOSGA020536	XP_003563567.1uncharacterized protein LOC100837547
TCONS_00034649	0.115308	-0.231106	0.321737	BGIOSGA034183	XP_010237773.1F-box protein SKIP16 isoform X1
TCONS_00012238	0.115831	-0.942269	0.514042	BGIOSGA026380	XP_006650060.1 60S ribosomal protein L18-3-like
TCONS_00003103	0.116044	0.0325198	-0.217297	BGIOSGA002120	XP_006643911.2 ABC transporter D family member 2, chloroplastic
TCONS_00016260	0.116049	0.0613456	-0.0807655	BGIOSGA006015	XP_015688822.1 60S ribosomal protein L14-1-like
TCONS_00011304	0.116065	-1.51952	0.548784	#N/A	#N/A
TCONS_00010030	0.116099	-0.546383	-0.223093	BGIOSGA010772	XP_002467895.1uroporphyrinogen decarboxylase
TCONS_00030535	0.116152	-0.423771	-0.178167	BGIOSGA029271	XP_004957636.1uncharacterized protein LOC101772363
TCONS_00026009	0.11627	-1.34168	-0.511603	BGIOSGA028561	XP_002443946.2putative disease resistance RPP13-like protein 3
TCONS_00011775	0.116379	0.556051	-0.0607101	BGIOSGA030881	XP_015689812.1 glycine-rich cell wall structural protein 1.0-like
TCONS_00025743	0.116431	0.274251	0.560186	BGIOSGA010148	XP_006658168.1 lon protease homolog, mitochondrial
TCONS_00003871	0.116562	-0.942232	-0.353821	BGIOSGA001343	NP_001183642.1putative homeodomain-like transcription factor superfamily protein
TCONS_00011642	0.1166	-0.613987	-1.33504	BGIOSGA021515	XP_004985544.1uncharacterized protein LOC101768541

TCONS_00034493	0.116602	1.26583	0.646081	BGIOSGA034336	XP_010100144.1Putative disease resistance RPP13-like protein 1
TCONS_00022145	0.116735	-0.317182	-0.831696	#N/A	#N/A
TCONS_00005555	0.116883	0.691236	0.45897	BGIOSGA037467	XP_003571019.3heptahelical transmembrane protein ADIPOR2 isoform X1
TCONS_00002040	0.117411	-0.86309	0.250047	BGIOSGA017821	XP_006644887.1 F-box protein At4g18380-like
TCONS_00002344	0.117526	-2.4795	-0.815641	BGIOSGA004985	XP_006645174.1 patatin-like protein 2
TCONS_00004367	0.117553	1.67427	1.09118	BGIOSGA000851	XP_006644689.1 uncharacterized tRNA/rRNA methyltransferase slr0955
TCONS_00003425	0.117696	0.109958	0.50241	BGIOSGA001796	XP_006645814.2 lipoamide acyltransferase component of branched-chain alpha-keto acid dehydrogenase complex, mitochondrial
TCONS_00020464	0.117797	0.767359	-0.0731765	BGIOSGA028164	XP_015690956.1 glucose-6-phosphate isomerase, cytosolic B
TCONS_00009080	0.117833	0.84303	1.42551	BGIOSGA005323	NP_001031856.1structural maintenance of chromosomes-like protein, putative (DUF3531)
TCONS_00012772	0.118086	-0.396137	-0.746637	BGIOSGA002282	XP_004982373.2CBL-interacting protein kinase 7
TCONS_00011683	0.118251	0.429138	0.28457	BGIOSGA003918	XP_012698500.1ethylene-responsive transcription factor ERF073
TCONS_00011293	0.118305	-0.040014	-0.139432	BGIOSGA017034	XP_006651997.1 probable cellulose synthase A catalytic subunit 5 [UDP-forming]
TCONS_00030280	0.118473	0.185975	-0.485672	BGIOSGA020770	XP_006660780.1 cinnamoyl-CoA reductase 1-like
TCONS_00014075	0.118537	-0.512792	-0.0288032	BGIOSGA016226	XP_006652200.1 ankyrin repeat and SAM domain-containing protein 6-like
TCONS_00023351	0.118537	1.26482	0.163842	#N/A	#N/A
TCONS_00006240	0.118561	#NA	-0.943732	BGIOSGA013269	XP_004952846.1probable serine/threonine-protein kinase At1g01540 isoform X2
TCONS_00016124	0.118587	0.55067	0.544373	BGIOSGA014852	XP_024311292.1microtubule-associated protein futsch isoform X2
TCONS_00032241	0.118664	-0.204751	-0.163774	BGIOSGA011911	XP_006661783.1 protein NETWORKED 1D
TCONS_00027391	0.118825	0.894835	0.277663	BGIOSGA024643	XP_015696029.1 DEAD-box ATP-dependent RNA helicase 42
TCONS_00031585	0.119255	1.10189	1.53712	BGIOSGA028804	YP_008815731.1photosystem II protein D1 (plastid)
TCONS_00014807	0.119379	0.00104821	0.860806	BGIOSGA008817	XP_015692309.1 mechanosensitive ion channel protein 1, mitochondrial-like isoform X1
TCONS_00007392	0.119433	0.941184	1.88941	BGIOSGA031637	XP_012700817.1 polyubiquitin-like
TCONS_00003326	0.119598	0.355191	0.505163	BGIOSGA014054	XP_006645773.1 somatic embryogenesis receptor kinase 1
TCONS_00030053	0.119618	-0.158088	-0.790887	BGIOSGA003282	XP_006660629.1 cinnamoyl-CoA reductase 1-like
TCONS_00020235	0.119618	-0.107975	0.422401	BGIOSGA022426	XP_015694282.1PREDICTED: LOW QUALITY PROTEIN: uncharacterized protein LOC102718657
TCONS_00009430	0.119655	-0.77543	-0.80126	BGIOSGA011981	XP_015690724.1 polyadenylate-binding protein-interacting protein 7-like
TCONS_00014487	0.119767	-8.98436E-05	0.76833	BGIOSGA016646	XP_015691749.1PREDICTED: uncharacterized protein ycf20
TCONS_00015296	0.119922	0.0547655	0.114538	BGIOSGA014159	XP_004975019.1peptidyl-prolyl cis-trans isomerase Pin1
TCONS_00003127	0.119929	-2.48252	-0.319076	BGIOSGA000352	XP_006643928.2 G-type lectin S-receptor-like serine/threonine-protein kinase At2g19130
TCONS_00029941	0.119974	-3.64511	-0.728703	BGIOSGA028570	XP_015696874.1 serine/threonine-protein kinase CDL1-like
TCONS_00003019	0.120116	0.600884	0.187681	BGIOSGA029870	XP_022682630.1DEAD-box ATP-dependent RNA helicase 20
TCONS_00005947	0.120218	-0.423354	0.663719	BGIOSGA029185	XP_015689347.1 60S ribosomal protein L10a-like
TCONS_00006092	0.120344	0.209717	1.07717	BGIOSGA006441	XP_006647287.1PREDICTED: uncharacterized protein LOC102714806
TCONS_00014524	0.120375	-0.20014	-0.492751	BGIOSGA031219	XP_006652467.1 protein NLP2-like isoform X1
TCONS_00027399	0.120634	-0.0307527	-0.00532946	BGIOSGA027061	XP_006659154.1 dnaJ homolog subfamily B member 4
TCONS_00037256	0.12083	-3.42835	-1.20309	BGIOSGA013092	XP_015698689.1PREDICTED: uncharacterized protein LOC102705026
TCONS_00006853	0.120936	0.545947	-0.0833151	BGIOSGA009032	XP_015689266.1PREDICTED: uncharacterized protein LOC102719358
TCONS_00020043	0.120983	-0.445294	-0.484756	BGIOSGA030756	XP_015693439.1 probable methyltransferase PMT15
TCONS_00009409	0.121018	-1.05453	-0.131486	BGIOSGA035713	XP_006651086.2PREDICTED: uncharacterized protein LOC102700608 isoform X1
TCONS_00011258	0.121277	0.900785	0.587128	BGIOSGA024833	XP_004981197.1DEAD-box ATP-dependent RNA helicase 3B, chloroplastic
TCONS_00037454	0.121572	-0.180137	-0.0538212	BGIOSGA035903	XP_015698819.1 SPX and EXS domain-containing protein 5
TCONS_00023571	0.121759	-0.90627	-0.382624	BGIOSGA040468	XP_006657648.2 tyrosine decarboxylase 1
TCONS_00003635	0.121802	-0.304778	0.472056	BGIOSGA019815	XP_003567735.1nitrate regulatory gene2 protein
TCONS_00016797	0.122134	0.0093677	0.465755	BGIOSGA014169	XP_006652964.1 U3 small nucleolar ribonucleoprotein protein IMP3
TCONS_00021200	0.122175	0.594635	0.308244	BGIOSGA032116	XP_006656338.1 serine/threonine-protein kinase HT1
TCONS_00036159	0.122176	-0.296437	0.330125	BGIOSGA037511	XP_003575781.1uncharacterized protein LOC100843284
TCONS_00032023	0.122581	-0.515677	0.274992	BGIOSGA017531	XP_004983274.1alpha-L-fucosidase 2
TCONS_00009688	0.122623	0.304641	0.320628	BGIOSGA032595	XP_012698390.1chloride conductance regulatory protein ICln
TCONS_00003241	0.12264	0.298036	0.0918237	BGIOSGA005780	XP_006644008.1 syntaxin-22-like
TCONS_00023143	0.122699	-0.84384	0.660669	BGIOSGA028051	XP_006657457.1 putative deoxyribonuclease TATDN1
TCONS_00030443	0.122731	0.079078	-0.701164	BGIOSGA029359	XP_004957492.13-dehydroquinate synthase, chloroplastic
TCONS_00006689	0.122841	-2.34652	-0.722154	BGIOSGA022835	XP_004953564.1probable xyloglucan endotransglucosylase/hydrolase protein 30

transcriptome

TCONS_00023401	0.122958	-1.34667	0.0551639	BGIOSGA036961	XP_006657581.1 UDP-glycosyltransferase 76C2-like
TCONS_00007835	0.123087	-1.81982	-0.497263	BGIOSGA006649	XP_015688879.1 sirohydrochlorin ferrochelatase, chloroplastic isoform X1
TCONS_00008885	0.12311	-2.20151	-1.50208	BGIOSGA005156	XP_006647977.1 glucan endo-1,3-beta-glucosidase 12-like
TCONS_00028474	0.123238	0.650561	0.467142	BGIOSGA011539	XP_002444576.1NAC domain-containing protein 53
TCONS_00021679	0.123244	-0.964717	0.655533	BGIOSGA017267	XP_015693931.1 putative receptor protein kinase ZmPK1
TCONS_00008877	0.123263	0.314922	-0.186256	BGIOSGA021971	XP_006647970.1 proteasome subunit beta type-6-like
TCONS_00003279	0.123327	0.70336	0.282605	BGIOSGA001211	XP_015688136.1 UBP1-associated protein 2A-like
TCONS_00009810	0.123483	0.520912	0.4713	BGIOSGA012364	XP_015690453.1 protein MODIFIER OF SNC1 11
TCONS_00003734	0.123523	0.0218558	1.54562	BGIOSGA008526	XP_015688890.1PREDICTED: uncharacterized protein LOC102712132
TCONS_00018181	0.12356	-0.328387	0.566258	BGIOSGA000888	XP_006654695.1PREDICTED: uncharacterized protein LOC102719206
TCONS_00028362	0.12356	-0.0653523	0.15122	BGIOSGA002250	XP_008664221.1uncharacterized LOC100277175 isoform X1
TCONS_00004505	0.12356	#NA	#NA	BGIOSGA013826	XP_004970175.1uncharacterized protein LOC101755031
TCONS_00019063	0.12356	0.671613	1.47315	#N/A	#N/A
TCONS_00006878	0.123613	0.925527	0.0333613	BGIOSGA015654	XP_012704268.1transcription termination factor MTERF2, chloroplastic
TCONS_00025526	0.123666	-1.0462	-2.90183	BGIOSGA007873	XP_004968896.1SPX domain-containing protein 6
TCONS_00031207	0.123731	-0.466226	-1.06896	BGIOSGA024887	XP_012704300.1L-type lectin-domain containing receptor kinase IV.1
TCONS_00030449	0.123826	1.07851	0.851754	BGIOSGA028752	XP_006661487.1 mediator of RNA polymerase II transcription subunit 4
TCONS_00033815	0.123867	0.0809319	0.00174519	BGIOSGA028591	NP_001136713.1zinc finger, C3HC4 type family protein
TCONS_00014480	0.123906	-0.378234	0.0528144	BGIOSGA018024	XP_015691711.1 kelch repeat-containing protein At3g27220-like
TCONS_00016021	0.123944	-1.71421	-0.915954	BGIOSGA014955	XP_002447971.1uncharacterized protein LOC8073441 isoform X2
TCONS_00000900	0.123945	-0.0748907	-0.36232	BGIOSGA002906	XP_015693885.1 polynucleotide 5'-hydroxyl-kinase NOL9-like
TCONS_00028157	0.12399	0.587549	0.47654	BGIOSGA026827	XP_006659490.2 zinc finger protein ZPR1-like
TCONS_00027010	0.124106	-0.770058	0.647376	BGIOSGA030107	XP_015695993.1PREDICTED: uncharacterized protein LOC102703145
TCONS_00023637	0.124269	-0.00895886	0.275668	BGIOSGA013307	XP_006657676.1 growth-regulating factor 11
TCONS_00010214	0.124387	0.837744	0.614894	BGIOSGA016274	XP_006650150.2 calmodulin-binding transcription activator 3-like isoform X1
TCONS_00028798	0.124439	-1.47406	-0.948504	BGIOSGA030543	XP_006660549.1 delta(8)-fatty-acid desaturase 2-like
TCONS_00006362	0.124452	0.179007	0.192468	BGIOSGA026374	NP_001148018.1hypoxia induced protein conserved region containing protein
TCONS_00009894	0.124471	0.777929	0.639894	BGIOSGA023675	XP_021310192.1sarcoplasmic reticulum histidine-rich calcium-binding protein
TCONS_00013123	0.124697	0.739807	0.152233	BGIOSGA003643	XP_015690988.1 zinc finger BED domain-containing protein RICESLEEPER 2
TCONS_00013433	0.124738	0.502631	0.0518525	BGIOSGA009552	XP_002463627.1phosphatidylinositol/phosphatidylcholine transfer protein SFH2
TCONS_00005522	0.12483	-3.40699	-2.00411	BGIOSGA028081	XP_006648775.1 phosphopantothenate--cysteine ligase 1 isoform X2
TCONS_00007063	0.124873	-0.386108	0.0250757	BGIOSGA009244	XP_015689287.1 probable starch synthase 4, chloroplastic/amyloplastic
TCONS_00018283	0.125064	-0.0820353	0.808302	BGIOSGA001021	XP_015692845.1PREDICTED: uncharacterized protein LOC102702944
TCONS_00031683	0.125246	0.162427	-0.190295	BGIOSGA008859	XP_006662104.1 lysine-specific demethylase JMJ706 isoform X1
TCONS_00034655	0.125283	-1.62165	0.741403	BGIOSGA034174	XP_006663355.1 general transcription factor 3C polypeptide 3 isoform X1
TCONS_00003057	0.125458	0.139503	-0.0160109	BGIOSGA011945	XP_006643885.2 pentatricopeptide repeat-containing protein DOT4, chloroplastic
TCONS_00036252	0.125547	-0.419117	-0.0724153	BGIOSGA037611	XP_006664113.2 membrane magnesium transporter
TCONS_00016209	0.125585	-0.875527	-0.358	BGIOSGA014763	XP_006653580.1 F-box protein At1g47056-like
TCONS_00032507	0.125599	0.883183	0.265345	BGIOSGA031613	XP_006662478.1 protein STABILIZED1
TCONS_00025778	0.125742	-0.313387	-0.902342	BGIOSGA011937	XP_006659024.2 receptor homology region, transmembrane domain- and RING domain-containing protein 2-like
TCONS_00004660	0.126044	0.790863	-0.420717	BGIOSGA025958	XP_006644946.1 UPF0051 protein ABCI8, chloroplastic
TCONS_00016532	0.126056	-1.04643	0.300686	BGIOSGA009201	XP_006653725.1 UV-B-induced protein At3g17800, chloroplastic-like
TCONS_00016962	0.126118	-0.98677	-0.766048	BGIOSGA019013	XP_006653949.1PREDICTED: uncharacterized protein LOC102711430
TCONS_00011598	0.126232	0.0736605	-0.891803	BGIOSGA006455	XP_015689769.1 photosynthetic NDH subunit of subcomplex B 2, chloroplastic
TCONS_00037220	0.126237	0.198474	-0.475298	BGIOSGA037010	XP_020407159.1membrane-associated phospholipid phosphatase isoform X1
TCONS_00009678	0.126241	-4.37455	-0.437538	BGIOSGA032576	XP_006649732.1 filament-like plant protein 4
TCONS_00027759	0.126469	-0.066711	0.569078	BGIOSGA036394	XP_015696093.1 large proline-rich protein bag6 isoform X2
TCONS_00012957	0.126515	0.720175	1.09325	BGIOSGA010032	XP_006650422.1 angio-associated migratory cell protein
TCONS_00007290	0.126623	-0.0623421	0.608726	BGIOSGA007192	XP_015689490.1 BOI-related E3 ubiquitin-protein ligase 1-like
TCONS_00035638	0.126623	0.710191	1.05034	BGIOSGA022052	XP_006663854.2 chaperone protein DnaJ-like
TCONS_00021868	0.126627	0.163459	0.224774	BGIOSGA009083	XP_015693772.1 F-box protein FBW2-like

TCONS_00001837	0.126717	-0.344992	-0.101015	BGIOSGA004472	XP_006644717.1 reticulon-4-interacting protein 1, mitochondrial
TCONS_00002490	0.126726	1.25163	1.9508	BGIOSGA005134	XP_007134493.1hypothetical protein PHAVU_010G051900g
TCONS_00007908	0.126726	#NA	#NA	#N/A	#N/A
TCONS_00018991	0.126726	-1.33333	-0.856555	#N/A	#N/A
TCONS_00001134	0.126763	1.34547	0.798453	BGIOSGA003759	XP_006646000.1PREDICTED: uncharacterized protein At1g51745, partial
TCONS_00019143	0.126934	1.33391	0.236485	BGIOSGA018213	XP_006654299.1 phospholipase D zeta 1-like isoform X1
TCONS_00036463	0.127017	-0.349999	0.3752	BGIOSGA000840	XP_006664221.2 GATA transcription factor 2-like
TCONS_00018220	0.127226	0.072349	0.0834778	#N/A	XP_003565984.1uncharacterized protein ycf36
TCONS_00013063	0.127285	0.462503	0.48001	BGIOSGA036435	XP_006650498.1 vam6/Vps39-like protein
TCONS_00000219	0.127331	0.610264	-0.219926	BGIOSGA000907	XP_006643762.1PREDICTED: uncharacterized protein LOC102718117
TCONS_00008928	0.127728	0.173787	-0.177514	BGIOSGA022463	XP_006648015.1 ethylene-responsive transcription factor 1
TCONS_00010170	0.127853	-0.352436	-0.394073	BGIOSGA008841	XP_006651427.1 protein phosphatase 2C 32-like
TCONS_00010491	0.127871	-0.178341	-0.505772	BGIOSGA013095	XP_002461077.2uncharacterized protein LOC8079369
TCONS_00018961	0.127996	-0.000279891	0.0904644	BGIOSGA018402	XP_006654217.1 protein SAD1/UNC-84 domain protein 1-like
TCONS_00009471	0.128196	0.194049	0.448566	BGIOSGA017027	XP_006649530.1PREDICTED: uncharacterized protein LOC102717879 isoform X2
TCONS_00021216	0.128245	#NA	#NA	BGIOSGA003091	XP_006643967.2 tubby-related protein 1-like
TCONS_00004069	0.128419	-0.268543	-0.112528	BGIOSGA001119	XP_020398008.1 ADP,ATP carrier protein 2, chloroplastic
TCONS_00004422	0.128662	0.290073	0.796732	BGIOSGA000801	XP_006646336.1 la-related protein 6A
TCONS_00020311	0.128666	0.962018	0.593615	BGIOSGA022507	XP_006655894.1 ubiquitin-like protein ATG12
TCONS_00035416	0.128683	-0.312051	-0.732075	BGIOSGA024996	XP_006663696.1 methylsterol monooxygenase 2-2-like
TCONS_00007603	0.128695	0.815689	0.374059	BGIOSGA006872	XP_015689478.1 YLP motif-containing protein 1 isoform X2
TCONS_00019684	0.128716	-0.855053	-0.455352	BGIOSGA018669	XP_010915190.1 hexokinase-2
TCONS_00021637	0.12875	-0.231825	-0.117351	BGIOSGA033350	XP_025985265.1mitogen-activated protein kinase 6 isoform X3
TCONS_00019139	0.128764	0.355374	-0.64007	BGIOSGA018216	XP_002440945.1protein DEHYDRATION-INDUCED 19 homolog 2 isoform X1
TCONS_00021370	0.128794	0.281531	0.349817	BGIOSGA004017	XP_003563563.1CDPK-related kinase 3
TCONS_00019670	0.128866	0.390251	0.752905	BGIOSGA019929	XP_006654686.1 protein LIKE COV 1-like
TCONS_00029367	0.129114	0.105546	0.811172	BGIOSGA031118	XP_006660861.1 ribosomal RNA processing protein 36 homolog
TCONS_00013035	0.129145	0.943981	-0.347107	BGIOSGA009952	XP_002464017.1peptidyl-prolyl cis-trans isomerase FKBP17-2, chloroplastic
TCONS_00000821	0.129479	0.921984	0.171727	BGIOSGA017079	XP_024315496.1YTH domain-containing family protein 1
TCONS_00027371	0.129596	0.551965	-0.267051	BGIOSGA005953	XP_003573476.1vesicle-associated protein 1-2
TCONS_00005570	0.12967	0.246705	0.133619	BGIOSGA020802	XP_006646989.1PREDICTED: uncharacterized protein LOC102717037
TCONS_00001761	0.129714	-0.337894	0.551188	BGIOSGA000822	NP_001315573.1uncharacterized protein LOC103637583
TCONS_00037525	0.129726	0.355861	0.519192	BGIOSGA018193	XP_015698366.1 BTB/POZ domain-containing protein At1g30440-like
TCONS_00026685	0.129944	-1.24677	-0.130862	BGIOSGA028750	XP_015695837.1PREDICTED: uncharacterized protein LOC102722322
TCONS_00024092	0.129949	-0.359991	-0.905074	BGIOSGA026125	XP_006658807.1 protein STRICTOSIDINE SYNTHASE-LIKE 4-like
TCONS_00019688	0.130092	0.804392	-0.408273	BGIOSGA017671	XP_004961406.1probable serine acetyltransferase 5
TCONS_00014535	0.13021	0.645981	0.474202	BGIOSGA016700	XP_006653565.2 cell division control protein 48 homolog B isoform X1
TCONS_00010313	0.130308	0.447919	0.857747	BGIOSGA023938	XP_006650201.1 putative G3BP-like protein
TCONS_00006284	0.130411	0.546139	-0.0588581	BGIOSGA009559	XP_006647393.1 PHD finger protein ALFIN-LIKE 7
TCONS_00028855	0.130507	0.314402	0.514109	BGIOSGA022434	XP_015696467.1 zinc finger CCCH domain-containing protein 59
TCONS_00001003	0.130652	0.0889683	0.36733	BGIOSGA010873	XP_002455084.12-hydroxyacyl-CoA lyase
TCONS_00005069	0.130787	-0.680982	-0.425736	BGIOSGA004368	XP_006645311.1 hexokinase-3 isoform X1
TCONS_00023994	0.130864	0.917631	0.675196	BGIOSGA026040	XP_002460932.1U-box domain-containing protein 4
TCONS_00020260	0.130919	0.567137	0.915582	BGIOSGA022449	XP_006655869.1 DNA-dependent metalloprotease WSS1
TCONS_00004782	0.130958	0.223079	-0.208288	BGIOSGA000432	XP_002458820.1protein BOLA2
TCONS_00014410	0.131146	#NA	0.132674	BGIOSGA004057	XP_006647155.1 peroxisomal fatty acid beta-oxidation multifunctional protein
TCONS_00024714	0.131146	-1.34002	#NA	BGIOSGA018450	XP_002440849.1D-amino-acid transaminase, chloroplastic
TCONS_00030788	0.131146	2.8299	3.13267	BGIOSGA021998	XP_015690285.1 40S ribosomal protein S20-1-like
TCONS_00028206	0.131327	-0.733932	1.09705	BGIOSGA030944	XP_006660230.1 tRNA (cytosine(34)-C(5))-methyltransferase isoform X1
TCONS_00019754	0.131387	0.156861	0.232066	BGIOSGA017602	XP_015692881.1 65-kDa microtubule-associated protein 3-like
TCONS_00015891	0.131425	0.482165	0.405383	BGIOSGA011386	XP_006652291.1 ribonuclease P protein subunit p25-like protein
TCONS_00001018	0.131648	0.262349	0.568587	BGIOSGA003620	XP_006644211.1 mitogen-activated protein kinase kinase 1
TCONS_00004681	0.131651	-1.41241	-0.373232	BGIOSGA017892	XP_006646459.1 protein NETWORKED 2A-like
TCONS_00026749	0.131855	-0.104347	-0.386625	BGIOSGA013602	XP_006659484.2 cytochrome P450 76M5-like
TCONS_00026436	0.13186	0.33633	-0.178257	BGIOSGA017828	XP_015695721.1 protein PHR1-LIKE 1-like
TCONS_00004796	0.131898	-0.29281	0.325651	BGIOSGA028607	XP_021310983.1probable ion channel POLLUX

transcriptome

TCONS_00009980	0.132017	0.443885	0.686019	BGIOSGA026709	XP_006650014.1 ETHYLENE INSENSITIVE 3-like 1 protein
TCONS_00019276	0.132253	-1.14542	-0.11947	BGIOSGA033878	XP_006655309.1 cytochrome P450 734A1-like
TCONS_00020136	0.132518	1.65792	1.71426	BGIOSGA022335	XP_006655797.1 ubiquinone biosynthesis O-methyltransferase, mitochondrial
TCONS_00037655	0.132518	0.657925	1.12929	#N/A	#N/A
TCONS_00026579	0.132542	0.286063	0.391458	BGIOSGA028647	XP_015695594.1 tRNA modification GTPase MnmE
TCONS_00007363	0.132544	0.92458	-0.177406	BGIOSGA007121	XP_002453316.1transcription factor Sp8
TCONS_00029467	0.132678	-0.203488	-1.15566	BGIOSGA034185	XP_015696831.1 protein NLP1-like
TCONS_00020995	0.132726	0.636393	-0.277559	BGIOSGA007405	XP_006656220.1 phospholipase D alpha 2
TCONS_00005724	0.132894	-0.73613	0.55805	BGIOSGA020685	XP_015689406.1 probable LRR receptor-like serine/threonine-protein kinase At1g53440
TCONS_00025779	0.133049	0.448596	-0.0957686	BGIOSGA026950	XP_006659026.1 putative 1-phosphatidylinositol-3-phosphate 5-kinase FAB1D
TCONS_00003389	0.133101	0.602497	0.195105	BGIOSGA001819	XP_006645797.2 nodal modulator 1
TCONS_00008433	0.133319	0.65793	0.0235772	BGIOSGA014690	XP_006647558.1 regulator of nonsense transcripts UPF2
TCONS_00023906	0.133354	0.173314	0.534205	BGIOSGA019748	XP_003562876.1organic cation/carnitine transporter 2
TCONS_00017501	0.133384	0.519199	0.251974	BGIOSGA003212	XP_003568665.1TLC domain-containing protein At5g14285
TCONS_00006632	0.133564	0.352321	-0.535072	BGIOSGA029410	XP_006665019.1 F-box protein PP2-A13-like
TCONS_00020255	0.133609	0.528806	0.16546	BGIOSGA005462	XP_006655865.1 probable cyclic nucleotide-gated ion channel 17
TCONS_00028572	0.133634	-0.169784	0.436788	BGIOSGA030314	XP_003580623.1uncharacterized protein LOC100837954
TCONS_00011313	0.134172	0.267708	0.480662	BGIOSGA000420	XP_006652012.2 probable ion channel CASTOR isoform X1
TCONS_00031643	0.134293	-0.288845	0.0610765	#N/A	XP_015697167.1 metacaspase-1
TCONS_00022042	0.134485	-0.796042	-1.49267	BGIOSGA021302	XP_015694363.1 potassium transporter 24-like
TCONS_00024528	0.134567	1.23907	0.63395	BGIOSGA034120	XP_004972473.1succinate dehydrogenase [ubiquinone] flavoprotein subunit, mitochondrial
TCONS_00009460	0.13465	0.826557	1.08509	BGIOSGA012008	XP_006649525.1 zinc finger CCCH domain-containing protein 4
TCONS_00011030	0.134755	0.636952	0.0236086	BGIOSGA003470	XP_006650654.2 cold-regulated 413 plasma membrane protein 2
TCONS_00020306	0.135092	1.29178	0.799951	BGIOSGA009140	XP_006655892.1 fatty-acid-binding protein 2-like
TCONS_00002604	0.135124	0.824626	0.0736519	BGIOSGA036786	XP_006645346.2 ABC transporter D family member 1-like
TCONS_00018119	0.135449	-0.40679	-0.0855956	BGIOSGA034831	XP_012700334.1transcription factor PCF8 isoform X2
TCONS_00021573	0.135502	-0.451785	-0.0408495	BGIOSGA020417	XP_021302228.1putative F-box/FBD/LRR-repeat protein At4g03220 isoform X2
TCONS_00032867	0.135626	-0.288589	-0.206636	BGIOSGA034693	XP_015698438.1 bifunctional epoxide hydrolase 2-like isoform X1
TCONS_00001256	0.135687	0.717583	0.645965	BGIOSGA003883	XP_015689166.1PREDICTED: uncharacterized protein LOC102720665
TCONS_00009750	0.135895	0.378522	0.995171	BGIOSGA002419	XP_015690275.1 mitochondrial substrate carrier family protein B-like
TCONS_00034505	0.135983	1.05497	0.346021	BGIOSGA034317	XP_006662815.1 phosphatidylinositol 4-kinase beta 1-like
TCONS_00037340	0.136057	1.78054	1.06257	BGIOSGA036027	XP_006664088.1 trafficking protein particle complex II-specific subunit 130 homolog isoform X2
TCONS_00020325	0.136116	-0.412433	-0.377394	BGIOSGA012471	XP_015694382.1 protein trichome birefringence-like
TCONS_00032064	0.136423	-0.34768	0.119789	#N/A	#N/A
TCONS_00014449	0.136508	-1.51736	-0.7308	BGIOSGA005687	XP_023157790.1putative FBD-associated F-box protein At5g53635
TCONS_00029254	0.136522	-0.142943	0.550424	BGIOSGA028543	XP_002460441.1pirin-like protein At1g50590
TCONS_00004486	0.136537	0.0280615	-0.387055	BGIOSGA017744	XP_006644780.1 transcription initiation factor IIF subunit beta-like isoform X1
TCONS_00024880	0.136627	0.31188	0.694962	BGIOSGA009574	XP_006657610.1 prohibitin-2, mitochondrial
TCONS_00024380	0.13672	0.136995	0.314301	BGIOSGA030858	XP_006658154.2 RINT1-like protein MAG2L
TCONS_00033066	0.136758	0.553457	0.216181	BGIOSGA034909	XP_006663254.1 cleavage stimulating factor 64
TCONS_00029163	0.136831	0.676776	0.50103	BGIOSGA030915	XP_006661314.2 probable quinone oxidoreductase
TCONS_00028468	0.136945	0.0353837	1.23076	BGIOSGA026488	XP_006659698.1 peptidyl-prolyl cis-trans isomerase CYP18-2
TCONS_00015083	0.136976	0.0736523	0.361896	BGIOSGA029279	XP_006653834.1 NADPH--cytochrome P450 reductase-like
TCONS_00011980	0.137303	0.402417	1.09559	BGIOSGA003544	XP_006649825.1 tropinone reductase homolog At5g06060-like
TCONS_00000290	0.137446	-1.72907	-1.32634	BGIOSGA012711	XP_014754219.1peroxidase 25
TCONS_00000958	0.137574	-1.09752	-0.112086	BGIOSGA003562	XP_010228047.1putative F-box protein At1g50870 isoform X2
TCONS_00019685	0.137644	#NA	#NA	BGIOSGA009548	XP_006654697.1PREDICTED: uncharacterized protein LOC102719769
TCONS_00015463	0.137644	0.387576	0.853776	BGIOSGA014637	XP_006652118.2PREDICTED: uncharacterized protein LOC102702648
TCONS_00027047	0.137789	-0.127832	-0.648576	BGIOSGA029097	XP_006659649.1 acid sugar phosphatase isoform X1
TCONS_00009701	0.137805	-0.360848	-2.06019	BGIOSGA019252	XP_004984976.1patatin-like protein 7
TCONS_00002537	0.138156	0.236528	0.55555	BGIOSGA005181	XP_006646686.1 F-box protein At3g54460
TCONS_00006649	0.138235	1.04252	-0.591339	BGIOSGA034249	XP_015688888.1 protein LYK5-like
TCONS_00010312	0.138272	-0.898081	1.03537	BGIOSGA034577	XP_006650200.1 protein LONGIFOLIA 2-like
TCONS_00007630	0.13844	0.49362	0.166972	BGIOSGA006850	XP_015697195.1PREDICTED: uncharacterized protein LOC102707343
TCONS_00020812	0.138531	0.671208	0.874526	BGIOSGA013107	XP_006657012.1 protein trichome birefringence-like 24
TCONS_00003730	0.138833	-1.35102	1.11392	BGIOSGA005991	XP_006645972.1 DIBOA-glucoside dioxygenase BX6-like

TCONS_00031565	0.138833	-0.351018	#NA	BGIOSGA033376	XP_003563429.1uncharacterized protein LOC100845879
TCONS_00036083	0.138833	0.97091	-0.886076	#N/A	#N/A
TCONS_00024964	0.138842	0.520458	0.568737	BGIOSGA007341	XP_006664840.1 dihydrolipoyllysine-residue acetyltransferase component 3 of pyruvate dehydrogenase complex, mitochondrial-like
TCONS_00029808	0.138847	-0.171597	0.605627	BGIOSGA020435	XP_015696296.1 rab3 GTPase-activating protein non-catalytic subunit
TCONS_00015085	0.139127	-0.889887	-1.21504	BGIOSGA015857	XP_006652936.1 probable receptor-like serine/threonine-protein kinase At5g57670
TCONS_00021532	0.139252	-0.501152	-0.0601802	BGIOSGA022018	XP_006656590.1 ABC transporter B family member 25, mitochondrial-like
TCONS_00009550	0.139262	0.208248	-0.532875	BGIOSGA021314	XP_006649617.1 pentatricopeptide repeat-containing protein At3g29290
TCONS_00019358	0.139285	1.67245	0.401589	BGIOSGA013904	XP_004961969.1subtilisin-like protease SBT3.11
TCONS_00019217	0.139673	-2.42704	-1.24351	BGIOSGA010530	XP_006650604.2 COBRA-like protein 3 isoform X2
TCONS_00001240	0.139779	0.332644	0.661848	BGIOSGA003868	XP_006646051.2 NADH dehydrogenase [ubiquinone] complex I, assembly factor 7
TCONS_00003886	0.139822	-0.615239	-0.28788	BGIOSGA001328	XP_015691417.1 SNF1-related protein kinase regulatory subunit gamma-1-like
TCONS_00014149	0.139893	0.617661	0.175162	BGIOSGA015187	XP_006652219.1 dihydrolipoyllysine-residue succinyltransferase component of 2-oxoglutarate dehydrogenase complex 1, mitochondrial-like
TCONS_00021552	0.139981	0.490211	-0.946471	BGIOSGA016022	XP_006656605.2 transketolase, chloroplastic
TCONS_00002366	0.139989	1.23238	0.111128	BGIOSGA005003	XP_004970959.1uncharacterized protein LOC101753699
TCONS_00022834	0.139989	#NA	1.11113	#N/A	#N/A
TCONS_00029040	0.140016	0.0539492	0.532837	BGIOSGA008971	NP_001105285.1senescence-associated protein DH
TCONS_00029200	0.140022	1.22667	1.06559	BGIOSGA026778	XP_006660733.1 tRNA (cytosine(34)-C(5))-methyltransferase
TCONS_00028207	0.140093	0.296655	0.624623	BGIOSGA031387	XP_006659515.1 phospho-2-dehydro-3-deoxyheptonate aldolase 1, chloroplastic
TCONS_00019751	0.140296	0.256724	0.628755	BGIOSGA006734	XP_015692692.1 sulfhydryl oxidase 1
TCONS_00004858	0.14052	-0.058669	1.15753	BGIOSGA016480	XP_021311627.1PHD finger protein ALFIN-LIKE 6 isoform X1
TCONS_00009052	0.14073	-0.111344	0.329979	BGIOSGA005352	XP_015688928.1 protein N-lysine methyltransferase METTL21A
TCONS_00011368	0.141	0.60534	0.425894	BGIOSGA013986	XP_006650929.1 sucrose nonfermenting 4-like protein isoform X2
TCONS_00019973	0.141069	-4.45041	-1.95913	BGIOSGA022160	XP_004964307.1WAT1-related protein At3g30340
TCONS_00003221	0.141099	2.45327	3.56784	BGIOSGA001996	XP_006643993.1 DNA replication complex GINS protein PSF2
TCONS_00003240	0.141099	-0.354081	0.108413	BGIOSGA003170	XP_015688086.1PREDICTED: uncharacterized protein LOC107303456
TCONS_00013158	0.141099	0.645919	0.108413	BGIOSGA018725	NP_001141300.1Suppressor/enhancer of lin-12 protein 9 precursor
TCONS_00003504	0.141099	#NA	#NA	#N/A	#N/A
TCONS_00027541	0.141164	-0.406137	-0.291938	BGIOSGA027470	XP_014660873.1AUGMIN subunit 2
TCONS_00002335	0.141227	0.720237	0.120093	BGIOSGA004978	XP_006645165.1 60S ribosomal protein L5-1
TCONS_00009127	0.141231	-0.928787	0.178763	BGIOSGA011653	XP_006650947.2 formyltetrahydrofolate deformylase 2, mitochondrial-like
TCONS_00018652	0.141239	0.519304	-0.167236	BGIOSGA003476	XP_003568901.1E3 ubiquitin-protein ligase RFI2
TCONS_00006508	0.141282	0.452631	0.45424	BGIOSGA027286	XP_006648859.1 probable metal-nicotianamine transporter YSL14
TCONS_00026420	0.141317	0.105535	0.0145719	BGIOSGA030352	XP_004952139.1D-aminoacyl-tRNA deacylase
TCONS_00036994	0.141774	0.354452	-0.219939	BGIOSGA036385	XP_006663939.1 U-box domain-containing protein 43-like
TCONS_00022680	0.141854	0.661573	0.67011	BGIOSGA020761	XP_006657202.2 ubiquitin carboxyl-terminal hydrolase 18-like isoform X1
TCONS_00003518	0.141928	0.180179	0.435164	BGIOSGA036044	XP_015688216.1 auxilin-related protein 2-like
TCONS_00016648	0.142173	#NA	-0.894224	BGIOSGA008857	XP_022685093.1uncharacterized protein LOC101771785
TCONS_00017262	0.142173	-0.219104	0.386585	BGIOSGA029577	XP_002439406.1uncharacterized protein LOC8065892
TCONS_00021181	0.142173	0.644477	-0.894224	#N/A	#N/A
TCONS_00031288	0.142173	-1.67745	-0.63119	#N/A	#N/A
TCONS_00031927	0.142183	0.495103	0.215768	BGIOSGA032217	XP_015697443.1 probable disease resistance protein At1g58602 isoform X1
TCONS_00021875	0.142289	0.292866	0.270376	BGIOSGA009074	XP_006655922.1 oxidation resistance protein 1-like
TCONS_00007758	0.142298	0.261529	0.434143	BGIOSGA019929	XP_006647146.1 protein LIKE COV 1-like
TCONS_00002244	0.142419	-0.5513	-0.286973	BGIOSGA026164	XP_006645084.1 serine/threonine-protein kinase SAPK4
TCONS_00016905	0.142435	-0.96719	-0.502452	BGIOSGA015125	XP_006653056.1 NDR1/HIN1-Like protein 3-like
TCONS_00021294	0.142581	-0.364433	0.11933	BGIOSGA023523	XP_006656414.1PREDICTED: uncharacterized protein At2g39910
TCONS_00015857	0.142769	-0.842456	0.389734	BGIOSGA015132	XP_003581203.3ABC transporter B family member 29, chloroplastic
TCONS_00031578	0.142906	0.315917	0.158566	BGIOSGA020522	XP_015697371.1 probable protein phosphatase 2C 72
TCONS_00035249	0.14298	-0.698721	0.426293	BGIOSGA033656	XP_015698088.1 1-acyl-sn-glycerol-3-phosphate acyltransferase PLS1 isoform X2
TCONS_00009276	0.14322	0.643099	0.51826	BGIOSGA011814	XP_008672062.1uncharacterized protein LOC100278549 isoform X1
TCONS_00027877	0.14322	-0.356901	#NA	#N/A	#N/A
TCONS_00032622	0.143239	-0.428832	-0.0558106	BGIOSGA031488	XP_006662006.1PREDICTED: uncharacterized protein LOC102717375 isoform X1
TCONS_00031487	0.143368	0.153816	0.503931	BGIOSGA033301	XP_006662527.1 flavonol synthase/flavanone 3-hydroxylase-like

TCONS_00025877	0.143379	0.655201	0.620097	BGIOSGA036349	XP_015695752.1 E3 ubiquitin-protein ligase RGLG2-like
TCONS_00001791	0.143578	-0.144165	-0.179022	BGIOSGA004428	XP_003569759.1endoribonuclease YBEY, chloroplastic
TCONS_00037168	0.143623	-1.53726	-0.590844	BGIOSGA006676	XP_021301308.1uncharacterized protein LOC8064056
TCONS_00013074	0.143845	1.01263	0.233408	BGIOSGA004742	XP_006650505.1PREDICTED: uncharacterized protein LOC102716856
TCONS_00020864	0.143903	0.964048	0.972679	BGIOSGA002172	XP_015693609.1 protein BZR1 homolog 3-like
TCONS_00003794	0.14401	-0.0407253	-0.300056	BGIOSGA013829	XP_006644268.1 ras-related protein RIC1
TCONS_00012659	0.144069	0.138447	-0.144729	BGIOSGA032980	XP_004982554.1pentatricopeptide repeat-containing protein At3g53700, chloroplastic
TCONS_00017023	0.144229	1.22674	1.6857	BGIOSGA019070	XP_015692947.1PREDICTED: uncharacterized protein LOC107304241
TCONS_00008957	0.144483	-0.303802	-0.266157	BGIOSGA005450	XP_006649127.1 endochitinase A-like
TCONS_00009302	0.144686	-0.296029	0.00625951	BGIOSGA011843	XP_006649388.1 protein PIR
TCONS_00026429	0.144699	0.44303	0.19116	BGIOSGA016436	XP_006659341.1 histone deacetylase 6-like
TCONS_00004180	0.145114	1.5445	1.11331	BGIOSGA018867	XP_006644526.2 long chain acyl-CoA synthetase 4-like
TCONS_00000116	0.145204	-0.359498	-0.638633	BGIOSGA002473	XP_006645453.1 multicopper oxidase LPR1-like
TCONS_00006992	0.1455	-0.649882	-0.34632	BGIOSGA029989	XP_006648026.1 E3 ubiquitin-protein ligase At1g63170-like
TCONS_00021757	0.145545	-0.722008	0.0720317	BGIOSGA033901	XP_006655863.2 probable folate-biopterin transporter 2
TCONS_00013673	0.145568	-0.0570642	-0.6621	BGIOSGA015833	XP_010229311.1stress response protein NST1
TCONS_00009244	0.145641	-0.227965	-0.0209679	BGIOSGA011180	XP_006649331.1 serine/threonine-protein kinase CDL1-like isoform X4
TCONS_00028178	0.145945	-0.601372	0.761898	BGIOSGA026807	XP_006659499.1 sodium channel modifier 1-like
TCONS_00031704	0.146151	-0.134507	-0.376119	BGIOSGA003149	XP_004983488.1homeobox-leucine zipper protein HOX15
TCONS_00025764	0.146312	-0.225332	-0.126514	BGIOSGA004507	XP_006658180.2PREDICTED: uncharacterized protein LOC102703229, partial
TCONS_00016371	0.146451	0.18214	-0.0922091	BGIOSGA014597	XP_006652607.1 E3 ubiquitin-protein ligase BRE1-like 1
TCONS_00008800	0.146565	0.444512	-1.1872	BGIOSGA031315	XP_015689673.1 soluble starch synthase 2-2, chloroplastic/amyloplastic
TCONS_00012445	0.146781	0.181172	-0.762475	BGIOSGA026404	XP_003557723.1probable galacturonosyltransferase 11
TCONS_00003656	0.146898	-0.768418	0.563287	BGIOSGA031552	XP_015699203.1 protein FLUORESCENT IN BLUE LIGHT, chloroplastic-like
TCONS_00012684	0.147011	0.743225	0.346	BGIOSGA035962	XP_006651586.1 transcription initiation factor TFIID subunit 8-like
TCONS_00031255	0.147077	0.638116	-0.90626	BGIOSGA037352	XP_006662411.1 protein fluG
TCONS_00012805	0.147077	0.223079	#NA	#N/A	#N/A
TCONS_00007576	0.147228	2.51003	0.587548	BGIOSGA009273	XP_006647059.1 3-ketoacyl-CoA synthase 11-like
TCONS_00035799	0.147237	0.542757	0.692001	BGIOSGA021917	XP_006656637.1 mitogen-activated protein kinase kinase 2 isoform X1
TCONS_00005500	0.147878	-0.378804	-0.330792	BGIOSGA035867	XP_006646922.1 thiosulfate/3-mercaptopyruvate sulfurtransferase 1, mitochondrial-like isoform X2
TCONS_00003715	0.147911	0.520139	0.183246	BGIOSGA011731	XP_010462583.1 callose synthase 7-like
TCONS_00035408	0.147971	0.0155056	0.229051	BGIOSGA006740	XP_015697507.1PREDICTED: uncharacterized protein LOC102703627 isoform X2
TCONS_00035218	0.147971	#NA	0.091547	BGIOSGA017668	XP_015690560.1 universal stress protein YxiE-like isoform X3
TCONS_00018457	0.148028	0.154048	0.126197	BGIOSGA018978	XP_006653939.2 putative ankyrin repeat protein RF_0381
TCONS_00007627	0.148061	0.534451	0.397093	BGIOSGA031963	XP_015689537.1 serine/threonine protein phosphatase 2A 55 kDa regulatory subunit B beta isoform-like
TCONS_00002663	0.148153	-0.273584	0.155288	BGIOSGA002558	XP_006643642.1 light-mediated development protein DET1
TCONS_00026055	0.148469	0.599372	1.32266	BGIOSGA036477	XP_006662842.1 phosphoribosylamine--glycine ligase-like isoform X1
TCONS_00008450	0.148712	-0.267914	0.764727	BGIOSGA011203	XP_006647573.1 DEAD-box ATP-dependent RNA helicase 47A
TCONS_00025681	0.148719	-0.619982	0.380402	BGIOSGA023742	XP_006658089.1 xylose isomerase
TCONS_00000581	0.148802	1.50869	2.00262	BGIOSGA018778	XP_006644004.2 importin subunit alpha-1a
TCONS_00009578	0.148843	4.02824	3.41134	#N/A	#N/A
TCONS_00005626	0.1489	0.190395	0.445995	BGIOSGA007739	XP_006647034.1 DNA damage-inducible protein 1
TCONS_00035886	0.148971	-1.02343	-1.60561	BGIOSGA007558	XP_006663954.1 ribulose bisphosphate carboxylase small chain, chloroplastic-like
TCONS_00017054	0.148987	-1.26415	0.137228	BGIOSGA006773	XP_006654000.1 phosphoinositide phospholipase C 2-like
TCONS_00037067	0.149053	-0.798566	-2.67029	BGIOSGA011208	XP_015695738.1 inositol 2-dehydrogenase
TCONS_00012216	0.149309	1.02052	0.63796	BGIOSGA010791	XP_006650047.1 transmembrane protein 87B
TCONS_00021177	0.149499	0.344623	-0.226263	BGIOSGA007031	XP_006657208.1 CASP-like protein 2C1
TCONS_00010378	0.149547	-1.47601	-1.93498	BGIOSGA012965	XP_006651544.1 LON peptidase N-terminal domain and RING finger protein 1
TCONS_00016803	0.149685	-0.0756522	0.656314	BGIOSGA014163	XP_015692220.1 zinc finger CCCH domain-containing protein 30 isoform X1
TCONS_00036344	0.14969	2.21985	5.37275	BGIOSGA032863	NP_001143921.1uncharacterized protein LOC100276732
TCONS_00004172	0.14969	#NA	#NA	BGIOSGA037816	XP_006657459.2 ubiquitin carboxyl-terminal hydrolase 8
TCONS_00016765	0.14969	0.482885	#NA	#N/A	#N/A
TCONS_00030473	0.14969	0.634888	0.0873507	#N/A	#N/A
TCONS_00014910	0.149719	-1.05854	0.0252025	BGIOSGA011908	XP_006653754.1 serine/threonine-protein kinase CTR1
TCONS_00001931	0.149741	0.433043	1.38516	BGIOSGA004572	XP_006644803.1 vacuolar protein sorting-associated protein 28 homolog 1

TCONS_00036624	0.149968	0.532125	0.100033	BGIOSGA036678	XP_006663183.1 glutamate-rich WD repeat-containing protein 1-like
TCONS_00028776	0.149997	0.22752	0.156527	BGIOSGA020600	XP_015694184.1 eukaryotic translation initiation factor 5-like
TCONS_00011346	0.150044	0.0928806	0.677839	BGIOSGA031080	XP_006650905.2 U5 small nuclear ribonucleoprotein 40 kDa protein-like
TCONS_00004789	0.150133	0.312556	0.713152	BGIOSGA000220	XP_006645080.1 protein S-acyltransferase 8-like
TCONS_00001257	0.150243	0.628759	-0.103356	BGIOSGA003885	XP_010231976.1protein MARD1
TCONS_00008757	0.150286	0.128733	0.335393	BGIOSGA005675	XP_006650330.1 mediator of RNA polymerase II transcription subunit 23
TCONS_00035508	0.150327	-0.384117	-0.0938859	BGIOSGA028931	XP_014757318.1uncharacterized protein LOC100826700 isoform X3
TCONS_00030967	0.150369	-0.393158	1.09137	BGIOSGA037473	XP_015696968.1 mitochondrial import inner membrane translocase subunit TIM22-3
TCONS_00005157	0.150403	0.603237	0.288701	BGIOSGA000090	XP_006661738.1 5-oxoprolinase
TCONS_00011993	0.150508	-1.36611	1.8927	BGIOSGA000018	XP_003558241.1uncharacterized protein LOC100832493
TCONS_00029655	0.150508	-1.95107	0.0853427	BGIOSGA003798	XP_006660448.1 nitrile-specifier protein 5
TCONS_00002499	0.150827	-0.12096	0.161355	BGIOSGA022880	XP_006645312.2 glucan endo-1,3-beta-glucosidase, acidic isoform-like
TCONS_00008092	0.150854	0.22383	-0.0970283	BGIOSGA037753	XP_006664166.1 gamma-tubulin complex component 5-like isoform X1
TCONS_00012791	0.15099	0.509885	0.989544	BGIOSGA031175	XP_006650332.1 histone-binding protein MSI1 homolog
TCONS_00034592	0.151166	-0.0511068	0.241176	BGIOSGA036028	XP_006650254.1 protein trichome birefringence-like 14 isoform X1
TCONS_00014404	0.151201	1.4202	0.276659	BGIOSGA016568	XP_015691339.1 plant UBX domain-containing protein 4-like
TCONS_00007190	0.151234	0.704647	0.52658	BGIOSGA012206	XP_006649711.1 small nuclear ribonucleoprotein SmD3b-like
TCONS_00006493	0.1513	-0.367061	#NA	BGIOSGA008658	XP_021313868.1uncharacterized protein LOC110434333 isoform X1
TCONS_00035829	0.1513	#NA	#NA	BGIOSGA031017	XP_006660787.1 chitinase-like protein 1
TCONS_00010226	0.151646	-0.174642	-0.124036	BGIOSGA014071	XP_015692368.1 cysteine protease ATG4B-like
TCONS_00021318	0.15174	-0.348156	0.0151113	BGIOSGA007136	XP_006656437.1 mitogen-activated protein kinase 4
TCONS_00016750	0.151828	1.1013	-1.08337	BGIOSGA008814	XP_008668660.1glycosyltransferase isoform X1
TCONS_00005266	0.151937	0.17881	0.323591	BGIOSGA004552	XP_002439211.1uncharacterized protein LOC8071260
TCONS_00000622	0.152069	-1.36798	-0.918495	BGIOSGA000169	XP_015696264.1PREDICTED: uncharacterized protein LOC107304866
TCONS_00008854	0.152069	-0.367978	#NA	BGIOSGA018154	XP_006647955.1 uncharacterized calcium-binding protein B0563.7-like
TCONS_00023937	0.152069	1.95395	0.88886	#N/A	#N/A
TCONS_00028761	0.152069	0.632022	#NA	#N/A	#N/A
TCONS_00017523	0.152094	0.16828	0.740315	BGIOSGA000544	XP_015689608.1 coatomer subunit delta-1 isoform X1
TCONS_00015420	0.152156	0.587482	0.913672	BGIOSGA033798	XP_006653188.1 ABC transporter G family member 28-like
TCONS_00022494	0.152354	-0.198717	0.0605397	BGIOSGA020961	XP_003563704.1protein RMD5 homolog
TCONS_00024544	0.152369	0.708522	0.321522	BGIOSGA019943	XP_015694522.1PREDICTED: uncharacterized protein LOC102706862, partial
TCONS_00016590	0.152441	0.149297	0.0213949	BGIOSGA028211	XP_006652799.1 protein strawberry notch-like
TCONS_00023504	0.15257	-0.458651	-0.614137	BGIOSGA033493	XP_008651505.1regulatory protein isoform X1
TCONS_00012620	0.152681	-0.796031	-0.364202	BGIOSGA013022	XP_006651573.1 endoribonuclease Dicer homolog 2a
TCONS_00010010	0.152773	1.75664	0.844091	BGIOSGA030655	XP_008655361.1ribosome biogenesis protein BMS1 homolog
TCONS_00014951	0.152806	-0.050703	-0.985756	BGIOSGA027930	XP_015692097.1 probable UDP-arabinose 4-epimerase 2
TCONS_00001555	0.152815	1.80106	0.0796651	BGIOSGA004185	XP_004969515.1uncharacterized protein LOC101769093
TCONS_00010107	0.152848	-1.30195	-1.63536	BGIOSGA012660	XP_003557871.1transcription termination factor MTEF1, chloroplastic
TCONS_00018305	0.152854	#NA	-1.0707	BGIOSGA022664	XP_006654785.1 NAC domain-containing protein 73-like
TCONS_00020450	0.152862	0.341147	1.30518	BGIOSGA021098	XP_015693788.1 tRNA threonylcarbamoyladenosine dehydratase
TCONS_00009467	0.153177	2.9971	0.216932	BGIOSGA010403	XP_015690335.1 protein DETOXIFICATION 27-like
TCONS_00021781	0.153209	0.169615	0.0571769	BGIOSGA009348	XP_006655877.1 sucrose synthase 2
TCONS_00008973	0.153318	0.71862	0.301184	BGIOSGA008949	XP_015689445.1 transmembrane 9 superfamily member 11-like
TCONS_00020986	0.153377	-0.789565	0.485201	BGIOSGA026958	XP_006656208.1 protein OVEREXPRESSOR OF CATIONIC PEROXIDASE 3
TCONS_00015381	0.153521	0.0696933	1.00842	BGIOSGA029977	XP_021311222.1pentatricopeptide repeat-containing protein At2g15690
TCONS_00018669	0.153529	0.14892	0.21909	BGIOSGA003007	XP_015692956.1PREDICTED: uncharacterized protein LOC107304245
TCONS_00029318	0.153538	1.95221	-0.922127	#N/A	#N/A
TCONS_00022646	0.153999	0.367431	0.212005	BGIOSGA005696	XP_006656297.1 probable sodium-coupled neutral amino acid transporter 6
TCONS_00008290	0.154163	1.208	1.12237	BGIOSGA005217	XP_006647441.1 DNA mismatch repair protein PMS1
TCONS_00009801	0.154211	1.18446	0.558152	BGIOSGA014330	XP_002468067.1receptor-like protein kinase HERK 1
TCONS_00007443	0.154241	0.0934114	0.575701	BGIOSGA015278	XP_003570467.1transmembrane protein 230
TCONS_00007473	0.154241	-1.08423	-0.637566	BGIOSGA031885	NP_001144700.1uncharacterized protein LOC100277736
TCONS_00000934	0.154241	-0.0486076	-0.338908	BGIOSGA035642	XP_006644173.1 glutaredoxin-C1
TCONS_00020505	0.154241	0.214427	0.661092	#N/A	#N/A
TCONS_00023173	0.154241	0.214427	0.0761298	#N/A	#N/A
TCONS_00031183	0.154308	-0.635235	-0.534687	BGIOSGA009810	XP_004983051.1probable NAD(P)H dehydrogenase (quinone) FQR1-like 2

TCONS_00023610	0.154365	0.0827001	0.292576	BGIOSGA037193	XP_015695307.1 endo-1,4-beta-xylanase A-like
TCONS_00022405	0.154468	-0.323249	-0.523444	BGIOSGA014312	NP_001147080.1zinc ion binding protein
TCONS_00032591	0.154928	0.213638	0.0744347	#N/A	#N/A
TCONS_00023963	0.155232	-0.3314	-0.362292	BGIOSGA025398	XP_006657877.1 tubulin alpha-3 chain
TCONS_00011434	0.155595	1.62791	0.880137	BGIOSGA031588	XP_006661929.1 UPF0329 protein ECU05_1680/ECU11_0050-like isoform X1
TCONS_00033446	0.155595	1.79784	1.65774	#N/A	#N/A
TCONS_00010815	0.155711	0.255059	-0.44861	BGIOSGA032204	XP_006650493.1 phosphoglucomutase, cytoplasmic 2
TCONS_00017115	0.155867	-2.03645	0.0335703	BGIOSGA033493	XP_015692806.1PREDICTED: putative uncharacterized protein DDB_G0277003 isoform X1
TCONS_00005043	0.15596	0.189525	0.18569	BGIOSGA035969	XP_015699239.1 esterase PIR7B-like
TCONS_00009557	0.15599	0.368304	0.559879	BGIOSGA005244	XP_006651164.1 probable mitochondrial-processing peptidase subunit beta
TCONS_00008459	0.156063	0.566586	-0.615498	BGIOSGA005968	XP_015689089.1 ferredoxin-thioredoxin reductase, variable chain-like
TCONS_00021435	0.1561	-1.33614	-0.601012	BGIOSGA022117	XP_006655671.1 plastocyanin, chloroplastic
TCONS_00027117	0.156195	0.830385	0.825077	BGIOSGA026269	XP_015695451.1 probable rhamnogalacturonate lyase B
TCONS_00004094	0.156243	#NA	#NA	BGIOSGA020405	XP_010095709.1hypothetical protein L484_002759
TCONS_00027320	0.156243	0.627177	2.07118	BGIOSGA028484	XP_004977990.1UPF0481 protein At3g47200
TCONS_00037406	0.156243	-0.372823	0.0711755	BGIOSGA036151	XP_008465297.1 glycine-rich cell wall structural protein 1.0-like
TCONS_00009182	0.156559	0.770172	0.133523	BGIOSGA008273	XP_006650977.1 WD repeat-containing protein 44-like
TCONS_00030389	0.156665	-0.139554	-0.198316	BGIOSGA021964	XP_006660862.1 F-box protein PP2-A13
TCONS_00003765	0.156777	1.04961	0.44402	BGIOSGA001440	XP_006644280.1 ubiquitin carboxyl-terminal hydrolase 6
TCONS_00018567	0.156806	-0.170962	0.888667	BGIOSGA018865	XP_003569071.1DUF724 domain-containing protein 3 isoform X1
TCONS_00029161	0.156821	-1.14371	-1.4077	BGIOSGA030912	NP_001142366.1EF hand family
TCONS_00006645	0.157083	0.174752	0.0806921	BGIOSGA016969	XP_002454478.1rho GTPase-activating protein 5
TCONS_00015098	0.157087	0.66541	0.606559	BGIOSGA017280	XP_006652947.1PREDICTED: uncharacterized protein LOC102718262
TCONS_00003710	0.15712	0.699225	0.451983	BGIOSGA001495	XP_006644253.2 pre-mRNA-processing protein 40A isoform X1
TCONS_00002668	0.157196	0.929301	0.709128	BGIOSGA002554	XP_006643645.1 uncharacterized sugar kinase slr0537
TCONS_00011910	0.157272	-2.11858	-1.03512	BGIOSGA027463	XP_004984993.1nuclear transcription factor Y subunit C-2
TCONS_00021440	0.15736	-0.247263	-0.213427	BGIOSGA022111	XP_006655677.1 glutaminyl-peptide cyclotransferase
TCONS_00032750	0.157488	-0.0522922	0.238013	BGIOSGA024412	XP_015692448.1PREDICTED: uncharacterized protein LOC102708621
TCONS_00001087	0.157488	-0.37422	#NA	#N/A	#N/A
TCONS_00015113	0.157505	-0.841281	0.0259894	BGIOSGA017292	XP_006652955.1 probable UDP-arabinopyranose mutase 2
TCONS_00017796	0.157539	-0.064848	0.245977	BGIOSGA019856	XP_015693221.1 squamosa promoter-binding-like protein 9
TCONS_00033306	0.157564	-5.9794	-0.935141	BGIOSGA032722	XP_006653690.1 cytochrome P450 87A3-like
TCONS_00010425	0.157673	-0.0694119	-0.3244	BGIOSGA005422	XP_006650279.1 cyclin-B1-2
TCONS_00024640	0.157796	-0.804855	-0.168112	BGIOSGA035103	XP_015694802.1 2-oxoisovalerate dehydrogenase subunit beta 1, mitochondrial
TCONS_00021432	0.157812	0.462692	0.204707	BGIOSGA023655	XP_006656530.1 tetraspanin-18-like
TCONS_00036854	0.157846	0.941989	-0.757889	BGIOSGA004222	YP_899414.1ATP synthase CF1 beta subunit (chloroplast)
TCONS_00015515	0.157984	-1.60533	-0.928898	BGIOSGA020371	XP_006653226.2PREDICTED: uncharacterized protein LOC102699500
TCONS_00021244	0.158197	-2.40913	-2.64763	BGIOSGA021758	XP_006657254.1 glutamate receptor 3.5
TCONS_00031169	0.158206	-0.892419	0.125618	BGIOSGA032991	XP_006662369.1PREDICTED: uncharacterized protein LOC102716447
TCONS_00017109	0.1585	0.535917	-0.00954616	BGIOSGA006437	XP_003568971.1RNA-binding protein Y14A
TCONS_00012231	0.158651	0.17446	1.01872	BGIOSGA010777	XP_006651355.1 putative tRNA pseudouridine synthase
TCONS_00033795	0.158667	-0.375527	#NA	BGIOSGA000601	XP_015697905.1PREDICTED: uncharacterized protein LOC107305277
TCONS_00012116	0.158667	-3.18288	#NA	BGIOSGA003344	NP_001131487.1cAMP-regulated phosphoprotein 19-related protein
TCONS_00022113	0.158667	#NA	#NA	BGIOSGA033307	XP_015693555.1 very-long-chain 3-oxoacyl-CoA reductase-like protein At1g24470
TCONS_00009789	0.158747	-0.0016721	0.120634	BGIOSGA012342	XP_015689844.1PREDICTED: uncharacterized protein LOC102718159
TCONS_00011257	0.158855	-0.448177	0.631935	BGIOSGA032852	XP_006650832.1 SPX domain-containing protein 4
TCONS_00033140	0.158942	0.373573	-0.210397	BGIOSGA034982	XP_006663275.1PREDICTED: uncharacterized protein LOC102706048
TCONS_00023038	0.158954	0.189591	0.579864	BGIOSGA025069	XP_015694459.1 protein HGV2
TCONS_00010770	0.159014	-1.04849	-0.110704	BGIOSGA025172	XP_006650445.1 ethylene-insensitive protein 2-like
TCONS_00017458	0.159192	0.193666	0.121171	BGIOSGA022773	XP_015693346.1 60S ribosomal protein L15 isoform X3
TCONS_00034453	0.159194	0.341746	0.853495	BGIOSGA036521	XP_015697586.1 probable receptor-like protein kinase At5g56460
TCONS_00009032	0.159309	0.57466	-0.377891	BGIOSGA005376	XP_006649161.1 phospholipid--sterol O-acyltransferase
TCONS_00021286	0.159375	-1.02539	-0.7657	BGIOSGA007565	XP_006656407.1 tyrosine-sulfated glycopeptide receptor 1-like
TCONS_00015032	0.159453	1.09948	-0.0925082	BGIOSGA017214	XP_006652891.1 stress enhanced protein 2, chloroplastic
TCONS_00008314	0.159588	-0.537693	-0.0596603	BGIOSGA006101	XP_010235642.1protein HHL1, chloroplastic
TCONS_00002297	0.159788	-2.96171	-1.52257	BGIOSGA038922	XP_015692159.1 cytochrome c-like

transcriptome

TCONS_00022845	0.159907	1.06885	1.12105	BGIOSGA033266	XP_006656432.1 probable protein phosphatase 2C 59
TCONS_00015027	0.160044	0.91668	0.477375	BGIOSGA027564	XP_006653805.1 DNA polymerase I A, chloroplastic
TCONS_00012208	0.160074	0.0996877	0.0982007	BGIOSGA010798	XP_006650038.1PREDICTED: uncharacterized protein LOC102721997 isoform X2
TCONS_00019084	0.160263	0.49293	0.622389	BGIOSGA018270	NP_001143959.1uncharacterized protein LOC100276774
TCONS_00005135	0.160327	1.5402	1.41469	BGIOSGA000072	XP_006645357.2 mitochondrial import inner membrane translocase subunit tim16-like
TCONS_00036203	0.160327	0.944592	0.0610564	BGIOSGA036032	XP_006664626.1 universal stress protein YxiE-like
TCONS_00011558	0.160479	-0.0842722	0.434586	BGIOSGA011459	XP_006649393.1 ankyrin repeat and SOCS box protein 13
TCONS_00025023	0.160595	0.167231	-0.0217728	BGIOSGA024382	XP_006657658.1 probable E3 ubiquitin-protein ligase XBOS34
TCONS_00030201	0.160643	0.505701	-0.62064	BGIOSGA029605	XP_006660725.2 isoamylase 3, chloroplastic
TCONS_00031212	0.160798	1.05943	0.21123	BGIOSGA004174	XP_006662392.1 cell division cycle protein 48 homolog
TCONS_00008274	0.160853	-0.383685	0.891633	BGIOSGA014914	XP_006647432.1 small nuclear ribonucleoprotein Sm D1
TCONS_00007439	0.160879	0.67574	0.507175	BGIOSGA007042	XP_006646958.1 proline-rich protein PRCC
TCONS_00023873	0.16096	0.603225	0.319849	BGIOSGA033460	XP_002460828.1protein IQ-DOMAIN 14
TCONS_00005376	0.161132	1.23225	-0.231822	BGIOSGA020572	XP_006646863.2 probable histone acetyltransferase HAC-like 1 isoform X1
TCONS_00009172	0.16119	0.0245197	0.537492	BGIOSGA011699	XP_006649678.1 thymidine kinase-like
TCONS_00008901	0.161281	-1.32729	-0.171246	BGIOSGA031562	XP_006649091.1 kinesin-related protein 4
TCONS_00013754	0.161334	0.155733	-1.47187	BGIOSGA011440	XP_022683825.1probable inorganic phosphate transporter 1-5
TCONS_00034577	0.161365	1.20651	0.0584883	BGIOSGA020798	XP_015697608.1 probable sucrose-phosphate synthase 5
TCONS_00022919	0.161365	-0.030528	0.68652	BGIOSGA033392	XP_006656499.1 NEP1-interacting protein-like 1
TCONS_00016674	0.161371	-1.05623	-0.0736034	BGIOSGA012967	XP_006652874.1 G-type lectin S-receptor-like serine/threonine-protein kinase B120
TCONS_00032706	0.161402	-1.06171	-0.453756	BGIOSGA027973	XP_002464930.1transcription factor MYBS3
TCONS_00006452	0.161454	0.297926	1.0742	BGIOSGA025969	XP_006647511.1 protein TIC 20-I, chloroplastic-like isoform X2
TCONS_00024090	0.161516	0.0802299	-0.724775	BGIOSGA025868	XP_008653340.2serine/threonine-protein kinase RIPK
TCONS_00001365	0.161572	0.328874	0.811878	BGIOSGA017585	XP_003575377.139S ribosomal protein L22, mitochondrial
TCONS_00001570	0.161679	-0.190765	0.669201	BGIOSGA024943	XP_003569562.1DNA-directed RNA polymerase III subunit RPC3
TCONS_00030281	0.161731	-0.126905	-1.00793	BGIOSGA021761	XP_006660780.1 cinnamoyl-CoA reductase 1-like
TCONS_00003350	0.161866	-0.378981	#NA	#N/A	#N/A
TCONS_00013436	0.162134	-0.359189	0.513711	BGIOSGA017674	XP_006651480.1 dolichyl-phosphate beta-glucosyltransferase
TCONS_00015806	0.162369	1.406	0.783852	BGIOSGA008338	XP_006652229.2 chaperone protein ClpC1, chloroplastic
TCONS_00036117	0.162458	0.502733	0.629194	BGIOSGA011319	XP_006664064.1 mitochondrial import inner membrane translocase subunit TIM22-3-like
TCONS_00013180	0.162506	-0.998031	-0.26258	BGIOSGA036962	XP_003559512.1protein STRICTOSIDINE SYNTHASE-LIKE 3
TCONS_00023419	0.162704	-0.994219	-0.930697	BGIOSGA009502	NP_001140513.1putative cytidine/deoxycytidylate deaminase family protein
TCONS_00025176	0.162732	-2.89556	-1.64772	BGIOSGA031102	XP_006657749.1 anthocyanidin 3-O-glucosyltransferase 2-like
TCONS_00014614	0.162827	0.356972	1.27727	BGIOSGA015745	XP_006652527.2PREDICTED: uncharacterized protein LOC102710777
TCONS_00027289	0.162827	1.42736	-0.945118	BGIOSGA027712	XP_006659087.1PREDICTED: uncharacterized protein LOC102702399 isoform X2
TCONS_00027044	0.162827	-2.18735	0.0548821	BGIOSGA029099	XP_006660348.1 putative disease resistance protein RGA4
TCONS_00036611	0.162827	-0.0320702	0.180413	BGIOSGA034567	XP_006654114.1 transmembrane protein 230-like isoform X1
TCONS_00011054	0.163061	-0.419784	0.261083	BGIOSGA034375	XP_015690956.1 glucose-6-phosphate isomerase, cytosolic B
TCONS_00018129	0.163289	#NA	-1.85315	BGIOSGA000825	XP_015692663.1PREDICTED: uncharacterized protein LOC102699963
TCONS_00006500	0.163289	#NA	-0.94626	BGIOSGA008666	XP_014751434.1photosynthetic NDH subunit of lumenal location 3, chloroplastic
TCONS_00033645	0.163289	0.204484	1.37567	#N/A	#N/A
TCONS_00000068	0.163443	-3.65062	-1.37385	BGIOSGA017262	XP_006643667.1 leucine-rich repeat extensin-like protein 3
TCONS_00029123	0.163737	2.03409	0.789593	BGIOSGA030874	XP_015694263.1PREDICTED: uncharacterized protein LOC107304498
TCONS_00037464	0.163902	0.317635	1.57931	BGIOSGA035896	XP_006664708.1 probable ureidoglycolate hydrolase
TCONS_00001924	0.164037	0.689161	-0.0091999	BGIOSGA035727	XP_006654749.1 vacuole membrane protein KMS1
TCONS_00011460	0.164173	-0.381411	-0.685426	#N/A	#N/A
TCONS_00021834	0.164276	-0.0354416	0.146843	BGIOSGA017184	XP_006655907.1PREDICTED: uncharacterized protein LOC102715213
TCONS_00018646	0.164553	0.750059	1.33163	BGIOSGA003497	XP_006655002.1 translocase of chloroplast 159, chloroplastic
TCONS_00033326	0.164596	0.203103	-0.212556	BGIOSGA006550	XP_006649469.1PREDICTED: uncharacterized protein LOC102700314
TCONS_00024161	0.1647	0.667118	0.595794	BGIOSGA010644	XP_006657998.1 transmembrane emp24 domain-containing protein p24delta9-like
TCONS_00021056	0.16501	1.3712	0.207611	BGIOSGA023284	XP_006657145.1 bZIP transcription factor 60
TCONS_00023638	0.165304	-0.85571	0.386456	BGIOSGA009612	XP_015694474.1 probable protein S-acyltransferase 12 isoform X3

transcriptome

TCONS_00002268	0.165414	#NA	2.37036	BGIOSGA018011	XP_006645097.1PREDICTED: uncharacterized protein LOC102712313
TCONS_00001068	0.165414	-0.382726	-0.536529	#N/A	#N/A
TCONS_00009454	0.165809	1.17559	0.565915	BGIOSGA012002	XP_015690488.1PREDICTED: uncharacterized protein At5g64816
TCONS_00013382	0.165892	0.0505747	0.394677	BGIOSGA029786	XP_006659353.2 pentatricopeptide repeat-containing protein At1g19720
TCONS_00009785	0.166209	-0.252757	-1.05895	BGIOSGA020769	XP_006649856.1 UDP-glucuronic acid decarboxylase 6
TCONS_00008809	0.166399	-0.0287869	-0.0730294	BGIOSGA035533	XP_015689510.1 F-box/kelch-repeat protein At1g74510-like
TCONS_00036528	0.166419	-1.40082	-2.5382	BGIOSGA027711	XP_009356128.1 protein CHUP1, chloroplastic
TCONS_00025898	0.166456	0.627509	0.638683	BGIOSGA028879	XP_004972879.1vesicle-associated protein 4-2
TCONS_00009218	0.166549	0.439653	0.591856	BGIOSGA005268	XP_006650989.1 protein FIZZY-RELATED 2-like
TCONS_00020545	0.16657	-1.1913	-0.76181	BGIOSGA022713	XP_022681755.1putative disease resistance protein At1g50180 isoform X3
TCONS_00035459	0.166935	-0.15301	-0.893973	BGIOSGA034031	XP_002441776.1uncharacterized protein LOC8074828
TCONS_00024593	0.166935	-1.9693	0.781592	BGIOSGA035609	XP_004985802.1uncharacterized protein LOC101763926
TCONS_00009930	0.167087	0.783625	0.576849	BGIOSGA015244	XP_006647284.1 ras GTPase-activating protein-binding protein 1-like
TCONS_00023134	0.167158	0.0488436	1.35049	BGIOSGA028211	XP_020395849.1uncharacterized protein LOC100037820 isoform X1
TCONS_00022723	0.16721	0.347572	0.264353	BGIOSGA000523	NP_001148233.1FKBP-type peptidyl-prolyl cis-trans isomerase 4
TCONS_00005766	0.167264	-0.188413	0.567507	BGIOSGA007174	XP_006657996.1 transcription elongation factor 1 homolog
TCONS_00013041	0.167291	0.615284	0.043728	BGIOSGA020918	XP_006650480.1 chemocyanin-like
TCONS_00007688	0.167291	-1.96968	-1.54123	BGIOSGA035627	XP_006658807.1 protein STRICTOSIDINE SYNTHASE-LIKE 4-like
TCONS_00017601	0.167291	#NA	-0.541234	#N/A	#N/A
TCONS_00025306	0.167407	-1.01857	0.367559	BGIOSGA010658	XP_008651857.1kinase interacting kinase 1 isoform X1
TCONS_00019228	0.167413	0.801969	-0.301562	BGIOSGA020409	XP_003566295.1phospholipase A1-Igamma1, chloroplastic
TCONS_00005367	0.16757	0.469267	-0.203611	BGIOSGA034437	XP_006646850.2 protein O-linked-mannose beta-1,4-N-acetylglucosaminyltransferase 2-like
TCONS_00031708	0.167638	0.714448	0.235496	BGIOSGA015558	XP_006661596.2 notchless protein homolog
TCONS_00008549	0.167638	#NA	0.457888	BGIOSGA031177	XP_006647659.1PREDICTED: uncharacterized protein LOC102704488
TCONS_00006152	0.167664	0.727751	0.490888	BGIOSGA004827	XP_003569904.1protein PAT1 homolog 1
TCONS_00005796	0.167977	-1.38545	0.0419932	BGIOSGA002171	XP_015689205.1PREDICTED: uncharacterized protein LOC107303658
TCONS_00017684	0.168026	0.0178624	-0.0341318	BGIOSGA011739	XP_006654327.1 nascent polypeptide-associated complex subunit alpha-like protein
TCONS_00025073	0.168292	-0.719845	-2.36107	BGIOSGA030920	XP_004957035.1probable tuliposide A-converting enzyme b6, amyloplastic
TCONS_00006826	0.168307	2.31689	1.15436	BGIOSGA009001	XP_015688963.1PREDICTED: uncharacterized protein LOC102713443 isoform X1
TCONS_00019353	0.168307	-0.648842	1.15663	BGIOSGA017995	XP_006658042.1 pre-mRNA-splicing factor SYF1
TCONS_00003485	0.168307	0.508416	0.581259	BGIOSGA024654	XP_006644140.1 putative disease resistance RPP13-like protein 3
TCONS_00015097	0.168465	-0.884626	-0.0176109	BGIOSGA001086	XP_006652946.1 putative glucose-6-phosphate 1-epimerase
TCONS_00013558	0.168568	0.454759	0.616596	BGIOSGA020488	XP_006650917.1 PAX3- and PAX7-binding protein 1
TCONS_00025419	0.168629	0.185192	0.527458	BGIOSGA017876	XP_015695163.1 WRKY transcription factor SUSIBA2-like
TCONS_00007510	0.16863	-2.38616	0.36226	BGIOSGA018030	XP_015689193.1 basic leucine zipper 1-like
TCONS_00032076	0.168757	1.31443	1.03833	BGIOSGA032061	XP_006661720.1 glutamate--tRNA ligase, cytoplasmic-like
TCONS_00024993	0.168834	-2.53968	-0.856691	BGIOSGA024119	XP_006664834.1 probable cellulose synthase A catalytic subunit 3 [UDP-forming]
TCONS_00029448	0.168944	#NA	#NA	BGIOSGA029334	XP_006660936.1 auxin-responsive protein SAUR36-like
TCONS_00004986	0.169212	-0.4087	-0.288896	BGIOSGA004699	XP_004971088.1F-box protein At1g67340
TCONS_00004132	0.169242	-0.0769129	-0.0312193	BGIOSGA010966	XP_004969524.2protein ATAF2
TCONS_00030090	0.169252	#NA	#NA	BGIOSGA029710	XP_006661260.1 zinc finger protein 2-like
TCONS_00029429	0.169554	0.197805	1.54048	BGIOSGA029129	XP_015696635.1PREDICTED: uncharacterized protein LOC107304960
TCONS_00012149	0.169618	-0.429238	-0.450867	BGIOSGA017483	XP_015691133.1 protein REDUCED WALL ACETYLATION 1-like isoform X1
TCONS_00020669	0.169884	-0.0213875	-0.339763	BGIOSGA008018	XP_003563824.1eukaryotic translation initiation factor 1A
TCONS_00012581	0.169932	0.587781	-0.703156	BGIOSGA024966	XP_004982535.1protein DETOXIFICATION 40
TCONS_00008678	0.170138	0.612211	0.358424	BGIOSGA029415	XP_002452661.1uncharacterized protein LOC8073839
TCONS_00001372	0.17019	0.909475	0.997245	BGIOSGA024643	XP_006646108.1 DEAD-box ATP-dependent RNA helicase 25
TCONS_00026267	0.170422	0.781829	1.03578	BGIOSGA004920	XP_006659261.1 probable DNA replication complex GINS protein PSF3
TCONS_00009623	0.170422	1.07134	1.73622	#N/A	#N/A
TCONS_00030881	0.170616	-4.53078	-1.45592	BGIOSGA035935	XP_006661696.2 flavonoid 3'-monooxygenase-like
TCONS_00004370	0.170698	-1.12536	-0.286849	BGIOSGA030759	XP_003569775.2uncharacterized protein LOC100824998
TCONS_00014133	0.17097	2.41866	-0.550569	BGIOSGA007382	XP_003579626.1probable metal-nicotianamine transporter YSL6
TCONS_00026940	0.17097	-0.38869	-0.702572	BGIOSGA012463	XP_006659583.1 calvin cycle protein CP12-3, chloroplastic-like
TCONS_00007541	0.17097	#NA	1.25679	BGIOSGA017413	NP_001145103.1uncharacterized protein LOC100278318

TCONS_00021005	0.171399	0.192191	-0.536912	BGIOSGA023236	XP_006656224.2 glucan endo-1,3-beta-glucosidase 14
TCONS_00034791	0.171495	0.610739	-0.551895	BGIOSGA012000	XP_004972914.1DIMBOA UDP-glucosyltransferase BX8
TCONS_00020032	0.171495	1.93267	3.30609	BGIOSGA012251	XP_006655729.1PREDICTED: uncharacterized protein LOC102710500
TCONS_00023081	0.171495	-1.38926	-0.966933	#N/A	#N/A
TCONS_00031560	0.171749	0.48493	0.447463	BGIOSGA021607	XP_015697456.1 pentatricopeptide repeat-containing protein At1g08070, chloroplastic-like
TCONS_00015190	0.171998	0.800014	-0.110222	BGIOSGA008693	XP_006653041.1 elicitor-responsive protein 3-like
TCONS_00012519	0.171998	-1.97477	-0.553166	#N/A	#N/A
TCONS_00009223	0.172147	1.28557	0.221164	BGIOSGA012562	XP_006650992.1 probable receptor-like protein kinase At5g24010
TCONS_00030539	0.172211	-1.04768	-1.7697	BGIOSGA013107	NP_001148278.1thiol protease SEN102 precursor
TCONS_00004025	0.172241	-0.0115663	0.0784868	#N/A	#N/A
TCONS_00012408	0.172304	-0.413817	-0.581024	BGIOSGA017414	XP_006650173.1 1-phosphatidylinositol-3-phosphate 5-kinase FAB1A-like
TCONS_00004206	0.172478	0.527197	-0.139347	BGIOSGA000996	XP_006644552.2PREDICTED: uncharacterized protein LOC102717385 isoform X1
TCONS_00026948	0.172478	0.609659	1.03058	BGIOSGA008168	XP_022681835.1glutathione S-transferase T3-like
TCONS_00023795	0.172478	-0.0277708	0.223223	BGIOSGA024375	XP_004984875.160S ribosomal protein L44
TCONS_00004139	0.172478	-0.712269	1.40909	BGIOSGA035645	XP_003569532.1monothiol glutaredoxin-S5
TCONS_00002552	0.17271	-1.3906	-0.970013	BGIOSGA019782	XP_015697046.1PREDICTED: uncharacterized protein LOC107305075
TCONS_00030897	0.17271	1.51629	1.67384	#N/A	#N/A
TCONS_00011239	0.172937	#NA	-1.97059	BGIOSGA005135	XP_002443307.1putative disease resistance protein RGA1
TCONS_00037034	0.173137	0.921915	-0.401476	BGIOSGA037260	XP_006663954.1 ribulose bisphosphate carboxylase small chain, chloroplastic-like
TCONS_00039026	0.173159	0.193858	-0.386198	BGIOSGA010143	XP_015689993.1 GTPase LSG1-1-like
TCONS_00010436	0.17328	0.755619	0.875539	BGIOSGA013028	XP_015691311.1 DNA-binding protein SMUBP-2 isoform X2
TCONS_00035632	0.173568	0.574377	0.356188	BGIOSGA037052	XP_006664369.1 nuclear pore complex protein NUP98A
TCONS_00018358	0.173591	0.655715	0.490083	BGIOSGA000520	XP_006643766.1 histone H2B.5
TCONS_00006768	0.173631	1.0395	0.708721	BGIOSGA021368	XP_006648996.2 PHD finger protein At3g20280-like
TCONS_00020851	0.173633	1.22568	1.27653	BGIOSGA023058	XP_015693606.1 polyadenylate-binding protein-interacting protein 11-like
TCONS_00036426	0.173758	-0.301612	0.839408	BGIOSGA025218	XP_024312685.1wall-associated receptor kinase 5 isoform X1
TCONS_00002146	0.173938	0.713956	0.562815	BGIOSGA003060	XP_006644989.1 myb-related protein B
TCONS_00016790	0.173949	0.564845	0.524397	BGIOSGA009765	XP_006652956.1 MAP3K epsilon protein kinase 1-like isoform X2
TCONS_00011692	0.174103	-0.159768	-0.67568	BGIOSGA007056	NP_001306702.1MADS-box transcription factor 47
TCONS_00009838	0.174209	-0.84232	-0.300476	BGIOSGA012390	XP_006651273.1 rhodanese-like domain-containing protein 10
TCONS_00022699	0.174213	0.813349	0.588995	BGIOSGA020744	XP_006656331.1 V-type proton ATPase catalytic subunit A
TCONS_00035488	0.174407	-1.19987	0.0256734	BGIOSGA034697	XP_006663167.1 bis(5'-adenosyl)-triphosphatase-like
TCONS_00020666	0.174602	-2.71466	0.288217	BGIOSGA012501	XP_006656950.2PREDICTED: uncharacterized protein LOC102711358
TCONS_00002118	0.174602	1.2187	0.44022	BGIOSGA017891	XP_006644960.1PREDICTED: uncharacterized protein At4g08330, chloroplastic-like
TCONS_00032077	0.174602	-1.71466	-0.296746	BGIOSGA034221	XP_004985376.1putative yippee-like protein Os10g0369500
TCONS_00004279	0.174742	-0.667364	-1.14297	BGIOSGA000926	XP_006646257.2PREDICTED: uncharacterized protein LOC102714525
TCONS_00023516	0.17515	0.300985	0.26094	BGIOSGA025549	XP_006657641.1 F-box-like/WD repeat-containing protein TBL1XR1
TCONS_00006821	0.175162	-0.808408	-0.266825	BGIOSGA016319	XP_006647851.1 nudix hydrolase 21, chloroplastic
TCONS_00001672	0.17541	0.0761993	-0.346216	BGIOSGA004307	XP_006646255.2 5'-nucleotidase SurE
TCONS_00007315	0.175472	-0.153711	0.364894	BGIOSGA007169	XP_006646851.1PREDICTED: uncharacterized protein LOC102702068 isoform X2
TCONS_00033376	0.175507	-1.33086	-2.16978	BGIOSGA035199	XP_015697756.1 mitochondrial dicarboxylate/tricarboxylate transporter DTC-like
TCONS_00014520	0.175518	-0.393779	1.4379	BGIOSGA016683	XP_003580054.3uncharacterized protein LOC100842706
TCONS_00028602	0.17569	1.19098	1.02243	BGIOSGA028486	XP_003573407.1D-aminoacyl-tRNA deacylase isoform X3
TCONS_00010511	0.17569	-0.616372	0.384997	#N/A	#N/A
TCONS_00024152	0.17569	#NA	0.0224264	#N/A	#N/A
TCONS_00029658	0.175714	0.915741	0.566283	BGIOSGA030162	XP_006660451.1 serine/threonine-protein phosphatase 2A 65 kDa regulatory subunit A beta isoform isoform X1
TCONS_00016219	0.175756	-0.372488	0.380968	BGIOSGA009289	XP_015692099.1 CLIP-associated protein-like
TCONS_00013717	0.17586	0.70536	0.214642	BGIOSGA015878	XP_006653159.1 mitochondrial inner membrane protease subunit 2-like isoform X2
TCONS_00027630	0.17586	0.53871	-1.43743	BGIOSGA019780	XP_015696083.1 afadin- and alpha-actinin-binding protein-like isoform X2
TCONS_00017324	0.17586	0.383432	0.799604	BGIOSGA028567	XP_006654151.1 nudix hydrolase 15, mitochondrial-like
TCONS_00010588	0.175973	-1.25067	0.0411698	BGIOSGA013201	XP_006650338.1 mediator of RNA polymerase II transcription subunit 19a-like isoform X2
TCONS_00009642	0.176188	0.453437	-2.30077	BGIOSGA017720	XP_006651196.1 non-symbiotic hemoglobin 2-like
TCONS_00015805	0.176217	-0.308543	0.308821	BGIOSGA034171	XP_006652229.2 chaperone protein ClpC1, chloroplastic
TCONS_00007393	0.176299	1.348	0.31867	BGIOSGA026264	NP_001278542.1COX VIIa-like protein

TCONS_00004581	0.176348	0.54746	0.171246	BGIOSGA000633	XP_006644865.1 50S ribosomal protein L34, chloroplastic
TCONS_00030233	0.176505	1.43514	-0.564607	BGIOSGA029573	XP_002462551.1uncharacterized protein LOC8063765
TCONS_00007730	0.176505	-1.39493	-0.979645	BGIOSGA031913	XP_021317282.1uncharacterized protein LOC8074381 isoform X2
TCONS_00035455	0.176569	0.282455	0.473181	BGIOSGA036733	XP_006663737.1PREDICTED: uncharacterized protein LOC102701873
TCONS_00005064	0.176659	-4.56504	-1.14996	BGIOSGA008953	XP_015697232.1PREDICTED: uncharacterized protein LOC107305119
TCONS_00007158	0.1767	0.55032	0.36783	BGIOSGA019729	XP_015689288.1PREDICTED: uncharacterized protein LOC102706919
TCONS_00002218	0.177249	0.381797	0.381035	BGIOSGA002421	XP_003567296.1transcription factor DIVARICATA
TCONS_00034089	0.177249	-0.395811	#NA	BGIOSGA005460	XP_023157553.1E3 ubiquitin-protein ligase Os04g0590900
TCONS_00009546	0.177249	-3.03967	#NA	BGIOSGA012091	NP_001148446.1cp protein
TCONS_00030214	0.177249	-1.39581	-1.98154	BGIOSGA035772	XP_006660738.1 basic leucine zipper 43-like
TCONS_00014681	0.177325	-0.343407	-0.757163	BGIOSGA029853	XP_006652595.1 homeobox-leucine zipper protein HOX22-like
TCONS_00019014	0.17739	-0.495514	-0.801321	BGIOSGA028955	XP_015693254.1 zinc finger AN1 domain-containing stress-associated protein 15
TCONS_00025384	0.177537	-0.301052	-0.491716	BGIOSGA013190	XP_004958088.1organic cation/carnitine transporter 7
TCONS_00005800	0.177572	0.0877652	0.49475	BGIOSGA016804	XP_015688826.1PREDICTED: uncharacterized protein At5g41620-like
TCONS_00010968	0.177664	-0.0752354	0.586267	BGIOSGA013568	XP_015690757.1 protein TORNADO 1 isoform X1
TCONS_00007664	0.177694	0.104518	0.179641	BGIOSGA006822	XP_006647106.1 U2 small nuclear ribonucleoprotein A'
TCONS_00005320	0.17806	-2.64471	-2.64656	BGIOSGA035361	XP_006648266.2 cyclin-P4-1-like
TCONS_00006289	0.178313	-0.577658	0.513266	BGIOSGA008450	XP_006647397.1 probable nucleoside diphosphate kinase 5
TCONS_00006916	0.178313	-0.882513	0.0157661	BGIOSGA009518	XP_006647956.1 cell number regulator 1-like
TCONS_00012119	0.178313	#NA	2.60073	BGIOSGA020051	XP_015690560.1 universal stress protein YxiE-like isoform X3
TCONS_00027741	0.178359	-0.702948	0.303313	BGIOSGA027260	XP_015695558.1 aberrant root formation protein 4
TCONS_00013263	0.178557	0.602618	0.52972	BGIOSGA009724	XP_006650674.1PREDICTED: uncharacterized protein LOC102719752
TCONS_00013335	0.178568	0.565998	-0.139358	BGIOSGA009646	XP_006650725.1 BI1-like protein
TCONS_00002444	0.178625	0.839665	0.567188	BGIOSGA005087	XP_006646628.1 protein DEK-like
TCONS_00032592	0.17871	0.629203	0.459039	BGIOSGA031184	XP_015697363.1 phospholipase D gamma 1-like
TCONS_00008543	0.17891	1.60219	0.751222	BGIOSGA004372	XP_004960887.1NADP-dependent malic enzyme, chloroplastic
TCONS_00015000	0.17891	-0.39781	0.133555	BGIOSGA017191	XP_006653793.1 peroxisome biogenesis protein 22
TCONS_00014505	0.179273	0.822666	1.08144	BGIOSGA016670	XP_006652453.1 eIF-2-alpha kinase GCN2 isoform X1
TCONS_00000496	0.179393	0.500381	0.108592	BGIOSGA017448	XP_006643947.1 casein kinase I-like isoform X2
TCONS_00023760	0.179567	-1.84607	-0.0363103	BGIOSGA025789	XP_006646828.1 dnaJ homolog subfamily B member 1-like
TCONS_00017536	0.179615	0.813515	0.0839753	BGIOSGA034885	XP_015693213.1 long chain acyl-CoA synthetase 8
TCONS_00006606	0.179769	0.530899	-0.667454	BGIOSGA031177	XP_006647654.1 aquaporin PIP1-1
TCONS_00027347	0.179773	-0.176471	0.681932	BGIOSGA014082	XP_008680222.2probable chromatin-remodeling complex ATPase chain
TCONS_00015890	0.179972	1.09122	0.477242	BGIOSGA015099	XP_006652290.1 COP9 signalosome complex subunit 8
TCONS_00025365	0.180129	0.68031	0.565854	BGIOSGA010746	XP_006657855.1PREDICTED: uncharacterized protein LOC102706861
TCONS_00000764	0.180166	-0.261845	0.496518	BGIOSGA002775	XP_012701322.1RING-H2 finger protein ATL3
TCONS_00002646	0.18026	0.40789	-0.989147	BGIOSGA009557	XP_008674049.1protein SODIUM POTASSIUM ROOT DEFECTIVE 1
TCONS_00005988	0.18026	2.55473	0.332781	BGIOSGA016611	XP_003573046.1eukaryotic translation initiation factor 4B3
TCONS_00020010	0.18026	-1.62186	-0.796502	BGIOSGA029432	NP_001130976.1harpin-induced 1
TCONS_00005603	0.180468	0.591796	-0.0173924	#N/A	XP_015689301.1 methyl-CpG-binding domain-containing protein 9
TCONS_00016729	0.180536	#NA	-0.989841	BGIOSGA029257	XP_015691930.1 VQ motif-containing protein 31
TCONS_00010294	0.180666	-0.502495	0.130813	BGIOSGA039220	XP_006651499.1 glucose-6-phosphate 1-dehydrogenase, chloroplastic-like
TCONS_00011431	0.180771	-0.151782	0.226496	BGIOSGA011593	XP_006650967.2 folylpolyglutamate synthase-like
TCONS_00028104	0.180885	0.599759	1.00928	BGIOSGA029700	XP_006662238.1 AT-rich interactive domain-containing protein 4-like
TCONS_00033844	0.180885	-0.198607	0.210914	BGIOSGA035615	XP_015697532.1PREDICTED: uncharacterized protein LOC102701767
TCONS_00012735	0.180899	0.253436	0.222152	BGIOSGA010231	XP_003580705.1SEC1 family transport protein SLY1
TCONS_00020888	0.180969	#NA	-1.21332	BGIOSGA007902	XP_006656168.1 abscisic acid receptor PYL2
TCONS_00023181	0.181208	-1.2489	-0.453907	BGIOSGA025221	XP_006657488.1PREDICTED: uncharacterized protein LOC102718936, partial
TCONS_00025408	0.181294	-1.98571	#NA	#N/A	#N/A
TCONS_00011384	0.181362	-0.151209	0.240334	BGIOSGA009390	XP_006650935.1 transcriptional corepressor LEUNIG_HOMOLOG-like isoform X2
TCONS_00013355	0.181435	0.0717926	-1.16211	BGIOSGA017005	XP_006650741.1 thioredoxin H2-2
TCONS_00014057	0.181526	0.0139945	-0.136727	BGIOSGA015413	XP_006653303.1 reticulon-like protein B12
TCONS_00032053	0.181601	-2.9861	-0.577489	BGIOSGA031430	XP_004983255.1uncharacterized protein LOC101781197 isoform X2
TCONS_00009487	0.181601	-0.164098	-0.477954	BGIOSGA035939	XP_006649553.1 N-acetyltransferase 9-like protein isoform X2
TCONS_00013080	0.181601	-2.72307	-0.5071	#N/A	#N/A

TCONS_00000661	0.181609	0.410211	0.268586	BGIOSGA003249	XP_006644055.1 isocitrate dehydrogenase [NAD] catalytic subunit 5, mitochondrial-like
TCONS_00005279	0.181672	0.0378914	-0.131367	BGIOSGA007386	XP_006648233.1 protein LOW PSII ACCUMULATION 3, chloroplastic
TCONS_00019313	0.181893	-0.401504	0.743705	BGIOSGA025424	XP_006648657.2 coiled-coil domain-containing protein 177-like
TCONS_00038843	0.181893	-1.05358	-0.130765	BGIOSGA036031	XP_006663112.1 disease resistance protein RPM1-like isoform X2
TCONS_00016854	0.181893	3.10946	1.5917	#N/A	#N/A
TCONS_00035018	0.181948	-0.395862	0.192323	BGIOSGA006464	XP_015698155.1 centromere/kinetochore protein zw10 homolog
TCONS_00003399	0.181964	-0.401592	0.369131	BGIOSGA022008	XP_020405347.1PIT1 isoform X1
TCONS_00026056	0.182127	-1.25045	-0.67677	BGIOSGA028123	XP_015696079.1 starch synthase 3, chloroplastic/amyloplastic-like isoform X1
TCONS_00034258	0.182237	-0.209292	-1.21652	BGIOSGA034537	XP_006664852.2 phosphoglycerate mutase-like protein 4
TCONS_00028068	0.182237	-2.9869	-0.994126	#N/A	#N/A
TCONS_00016614	0.182303	1.18294	0.268742	BGIOSGA010113	XP_004976810.1uncharacterized protein At4g15545
TCONS_00002027	0.182368	0.45772	0.460109	BGIOSGA000417	XP_006644878.1PREDICTED: uncharacterized protein LOC102719437
TCONS_00024315	0.182368	1.19555	0.331896	BGIOSGA018180	XP_006658949.2PREDICTED: uncharacterized protein LOC102703417
TCONS_00005946	0.182433	-0.987147	-0.42204	BGIOSGA009442	XP_004952370.1B-cell receptor-associated protein 31
TCONS_00002279	0.182497	1.14522	0.764213	BGIOSGA004916	XP_003561922.1dirigent protein 8
TCONS_00002906	0.182744	-0.126943	-1.65836	BGIOSGA002313	XP_022682852.1uncharacterized protein LOC101753820
TCONS_00012543	0.182864	2.13332	0.267335	BGIOSGA010439	XP_003561920.2uncharacterized protein LOC100829561
TCONS_00024076	0.182864	0.174581	0.216845	BGIOSGA026111	XP_021308682.1stress-response A/B barrel domain-containing protein UP3
TCONS_00018257	0.182922	-0.372429	0.177485	BGIOSGA002450	XP_008649964.1peptidyl-tRNA hydrolase, mitochondrial isoform X2
TCONS_00004512	0.182922	0.394381	0.223163	BGIOSGA020136	XP_006644802.1 protein ULTRAPETALA 1-like isoform X2
TCONS_00036746	0.183054	0.822604	-0.0651905	BGIOSGA036568	XP_006663852.1 WD repeat-containing protein 48
TCONS_00024397	0.183205	1.31673	0.308299	BGIOSGA029631	XP_002461259.1probable protein phosphatase 2C 33 isoform X2
TCONS_00009503	0.183367	0.0913985	0.128569	BGIOSGA037468	XP_006651138.1 protein CLT2, chloroplastic-like isoform X1
TCONS_00033308	0.183524	-4.104	-2.11283	BGIOSGA026786	XP_006647933.2 uclacyanin-3-like
TCONS_00026818	0.183625	-0.0317244	0.869126	#N/A	#N/A
TCONS_00024430	0.183675	-0.0925547	-0.229058	BGIOSGA024978	XP_004987271.1DDRGK domain-containing protein 1
TCONS_00034656	0.183774	0.50301	-0.413016	BGIOSGA034172	XP_015698066.1PREDICTED: uncharacterized protein LOC102705200 isoform X2
TCONS_00010195	0.183822	0.110631	-0.142489	BGIOSGA012761	XP_006651443.2 probable F-actin-capping protein subunit beta
TCONS_00014874	0.183822	0.259023	-0.775707	BGIOSGA017810	XP_006652754.2 myb family transcription factor APL-like
TCONS_00033243	0.183822	0.454039	-0.538667	BGIOSGA019279	XP_004955354.1putative disease resistance RPP13-like protein 3
TCONS_00025642	0.183848	0.341351	-1.45055	BGIOSGA023722	XP_003559858.1ferredoxin-dependent glutamate synthase, chloroplastic
TCONS_00024471	0.183917	-1.47445	-1.5833	BGIOSGA024927	XP_006658228.1 probable N-acetyltransferase HLS1
TCONS_00003385	0.18401	-0.543156	-0.291351	BGIOSGA019123	XP_006644100.1PREDICTED: uncharacterized protein LOC102699440 isoform X1
TCONS_00031572	0.184234	-0.798127	-0.707666	BGIOSGA019155	XP_006662017.1 chitinase 8
TCONS_00020793	0.18432	-1.21193	0.410531	BGIOSGA006739	XP_006656122.1 plastid division protein PDV1-like
TCONS_00019672	0.18432	0.57728	0.322583	BGIOSGA015227	XP_002441426.1ER lumen protein-retaining receptor A
TCONS_00011360	0.184405	-0.40468	0.348367	BGIOSGA025981	XP_015690154.1PREDICTED: uncharacterized protein LOC102712522
TCONS_00021714	0.184475	0.127207	1.50993	BGIOSGA025459	XP_002437933.1vesicle-associated membrane protein 711
TCONS_00027708	0.184587	1.1547	0.963906	BGIOSGA036755	XP_015695556.1 probable WRKY transcription factor 32
TCONS_00007853	0.184879	0.372329	0.513834	BGIOSGA006628	XP_006647197.1PREDICTED: uncharacterized protein LOC102711222
TCONS_00013342	0.184952	-0.509208	0.84038	BGIOSGA009639	XP_015690257.1 tryptophan synthase alpha chain-like
TCONS_00017934	0.185096	0.209157	0.0861828	BGIOSGA034427	XP_003568316.1probable protein S-acyltransferase 16
TCONS_00002731	0.185165	-0.405641	-1.17138	BGIOSGA026844	XP_015688077.1 CAX-interacting protein 4
TCONS_00032290	0.185165	0.108932	-1.22385	BGIOSGA031841	XP_004983025.1transcription factor-like protein DPB
TCONS_00007136	0.185267	2.36607	-1.85586	BGIOSGA005904	XP_015689555.1 aquaporin PIP 1-3
TCONS_00027226	0.185301	-1.87148	-0.815378	BGIOSGA040494	XP_004955700.1probable nucleolar protein 5-1
TCONS_00001007	0.185367	-0.0887057	-0.232254	BGIOSGA003609	XP_002460837.1protein PGR
TCONS_00019388	0.185399	0.0301622	1.52521	BGIOSGA031062	XP_003568356.1NAC domain-containing protein 90
TCONS_00001198	0.185495	-1.76863	1.50022	BGIOSGA020255	XP_006644309.1 calcineurin B-like protein 9
TCONS_00002205	0.185558	1.55322	#NA	BGIOSGA024463	XP_003564666.1acidic endochitinase
TCONS_00020569	0.1856	0.0936853	0.0223684	BGIOSGA005712	XP_006656024.1 enhancer of mRNA-decapping protein 4-like
TCONS_00002274	0.185649	-1.54376	-0.877129	BGIOSGA004263	XP_006645107.1 RNA-binding protein 24-B-like isoform X1
TCONS_00033899	0.185768	#NA	0.914582	BGIOSGA015162	XP_006663615.1 adenylyl-sulfate kinase 3-like
TCONS_00005687	0.185768	1.78624	1.09658	BGIOSGA021020	XP_006647078.1 cysteine synthase-like
TCONS_00033281	0.185797	-1.05852	-0.00302794	BGIOSGA001169	XP_006662904.1 mitogen-activated protein kinase 15

TCONS_00018946	0.185883	-0.319085	0.903649	#N/A	#N/A
TCONS_00032736	0.185911	0.194868	0.920068	BGIOSGA031372	XP_015697176.1 VAN3-binding protein-like
TCONS_00022065	0.185939	#NA	-3.00338	BGIOSGA025003	XP_006662662.1 peroxidase 4-like
TCONS_00008532	0.185939	-3.40662	-0.418418	BGIOSGA025707	XP_006647637.1 probable trehalose-phosphate phosphatase 1
TCONS_00009620	0.185994	0.474668	-1.08152	BGIOSGA029831	XP_002436775.2U-box domain-containing protein 39
TCONS_00011284	0.18604	-0.12516	-0.734001	BGIOSGA037130	XP_015690143.1 translocase of chloroplast 159, chloroplastic-like
TCONS_00009734	0.186048	0.953032	0.778466	BGIOSGA012283	XP_006649801.1 50S ribosomal protein L4, chloroplastic
TCONS_00016529	0.186048	0.153959	-0.62514	BGIOSGA022173	XP_006652732.1 binding partner of ACD11 1-like isoform X1
TCONS_00004368	0.186101	0.818597	0.175539	BGIOSGA021382	XP_003569774.1probable 3-hydroxyisobutyrate dehydrogenase-like 1, mitochondrial
TCONS_00017446	0.186128	0.459877	1.66357	BGIOSGA022893	XP_006654218.1 ribonuclease 3-like protein 2
TCONS_00018044	0.186205	0.221077	-0.556584	BGIOSGA020092	XP_010231224.1putative disease resistance protein RGA3
TCONS_00011420	0.186221	-0.273264	-0.117989	BGIOSGA011604	XP_006649241.2 phosphoglucan phosphatase DSP4, amyloplastic isoform X1
TCONS_00024120	0.186231	1.26544	0.388211	BGIOSGA026155	NP_001151935.2calmodulin binding protein
TCONS_00008974	0.186281	1.38436	0.617257	BGIOSGA019802	XP_022679429.1uncharacterized protein LOC101771126
TCONS_00028128	0.186281	2.17791	1.4697	BGIOSGA039076	XP_006659478.2 putative F-box/LRR-repeat protein At3g18150
TCONS_00006090	0.186306	0.962152	0.658671	BGIOSGA015087	XP_002447851.1golgin subfamily A member 6-like protein 1
TCONS_00000098	0.186498	-0.813316	-0.117982	BGIOSGA019197	XP_022682352.1E3 ubiquitin-protein ligase SINA-like 10
TCONS_00013359	0.18659	1.93651	1.72083	BGIOSGA009625	XP_006650744.2 defective chorion-1 protein, FC106 isoform
TCONS_00032899	0.186657	-0.112068	-0.0722807	BGIOSGA007026	XP_014757318.1uncharacterized protein LOC100826700 isoform X3
TCONS_00016629	0.186679	-1.14452	-1.32715	BGIOSGA000636	NP_001170586.1uncharacterized LOC100384618
TCONS_00014632	0.186723	0.806518	0.480097	BGIOSGA040035	XP_020400622.1ATP-dependent Clp protease proteolytic subunit isoform X1
TCONS_00006267	0.186766	0.699254	-0.120913	BGIOSGA010979	XP_003572617.1tubulin-folding cofactor C
TCONS_00015008	0.186766	0.213827	1.10148	BGIOSGA032389	XP_015692241.1 anthocyanidin reductase-like
TCONS_00026521	0.186829	0.514257	0.916404	BGIOSGA028588	XP_024314338.1putative F-box protein At5g15660
TCONS_00000206	0.186891	1.46665	0.994253	BGIOSGA002779	XP_006451924.1ribosome biogenesis protein BRX1 homolog 1
TCONS_00017831	0.186932	0.411992	0.127802	BGIOSGA024039	XP_006654417.2 UPF0301 protein CT0663-like
TCONS_00012879	0.186952	1.68325	0.828092	BGIOSGA010101	XP_006650384.1 protein CDC73 homolog
TCONS_00017983	0.186952	-2.63029	-0.813252	BGIOSGA019328	XP_006654534.1 zinc transporter 5
TCONS_00032691	0.186972	0.53637	0.139483	BGIOSGA012950	XP_006662053.1 S-norcoclaurine synthase 1-like
TCONS_00004152	0.1872	0.591791	-0.328443	BGIOSGA022664	XP_015688316.1 NAC domain-containing protein 73-like
TCONS_00019646	0.187218	-1.99319	-1.36913	BGIOSGA019089	XP_022680618.1uncharacterized protein LOC101766057
TCONS_00018276	0.187221	-0.771345	2.50509	BGIOSGA001013	XP_015693010.1 glutamate synthase 2 [NADH], chloroplastic-like
TCONS_00004181	0.187237	0.729249	0.426355	BGIOSGA012257	XP_006644527.1 beclin-1-like protein
TCONS_00016838	0.187308	-0.0193017	0.666991	BGIOSGA014127	XP_006653000.2 OTU domain-containing protein 3 isoform X1
TCONS_00013744	0.18736	0.147984	-0.00691072	BGIOSGA027032	XP_004975140.21-aminocyclopropane-1-carboxylate oxidase homolog 1
TCONS_00012254	0.187511	0.591401	1.02834	BGIOSGA017653	XP_015690355.1 cell cycle checkpoint control protein RAD9A
TCONS_00015415	0.187559	0.969851	1.37111	BGIOSGA015958	XP_015691872.1 F-box protein At3g57580-like
TCONS_00031830	0.187575	0.828359	0.0920922	BGIOSGA018303	XP_006662178.1PREDICTED: uncharacterized protein LOC102703339, partial
TCONS_00022415	0.187591	-1.63109	-0.229875	BGIOSGA021048	XP_006656156.1 acidic repeat-containing protein-like
TCONS_00001553	0.187622	1.70277	1.25117	BGIOSGA003649	XP_006646168.1PREDICTED: uncharacterized protein LOC102709049
TCONS_00003703	0.187622	0.51726	-0.329488	BGIOSGA007575	XP_003565911.2F-box protein SKIP23
TCONS_00030234	0.187699	0.864182	0.69269	BGIOSGA020218	XP_006661362.1 plant UBX domain-containing protein 10-like
TCONS_00010286	0.187714	0.939068	0.117743	BGIOSGA001682	XP_024312641.1putative ubiquitin-like-specific protease 1B isoform X2
TCONS_00025008	0.187729	-1.40887	0.272283	BGIOSGA004101	XP_004956076.1probable indole-3-pyruvate monooxygenase YUCCA5 isoform X1
TCONS_00000658	0.187729	-2.5788	-0.370395	BGIOSGA035136	NP_001152938.2disulfide oxidoreductase/ monooxygenase/ oxidoreductase
TCONS_00020309	0.187802	-2.07193	-1.08601	BGIOSGA037023	XP_021310100.1F-box/FBD/LRR-repeat protein At1g13570-like
TCONS_00018258	0.187901	1.45646	1.1535	BGIOSGA008673	XP_015692686.1 threonine synthase, chloroplastic-like
TCONS_00029539	0.187911	-0.527904	-0.649554	BGIOSGA006315	XP_002458624.1uncharacterized protein At2g27730, mitochondrial
TCONS_00025641	0.187929	1.43415	0.991679	BGIOSGA013616	NP_001333427.330S ribosomal protein S9, chloroplastic
TCONS_00030158	0.187943	0.80031	0.799	BGIOSGA022020	XP_006660687.1 ESX-1 secretion-associated protein EspK-like
TCONS_00015154	0.187956	1.6052	0.633158	BGIOSGA008665	XP_006653011.1PREDICTED: uncharacterized protein At2g24330-like
TCONS_00022522	0.188023	0.949166	1.56759	BGIOSGA007750	XP_006656213.1 adenine nucleotide transporter BT1, chloroplastic/mitochondrial-like
TCONS_00033494	0.188126	0.0286176	0.258755	BGIOSGA035290	XP_015698124.1PREDICTED: uncharacterized protein LOC102716539
TCONS_00015316	0.188151	-3.21676	0.82763	BGIOSGA032416	XP_003566804.1serine decarboxylase 1

TCONS_00034645	0.188151	0.136029	0.103604	BGIOSGA034180	XP_010237773.1F-box protein SKIP16 isoform X1
TCONS_00035019	0.1882	0.105106	0.312935	BGIOSGA034120	XP_021317481.1ultraviolet-B receptor UVR8 isoform X1
TCONS_00022667	0.188237	0.318408	0.200371	BGIOSGA012704	XP_002438762.1magnesium transporter MRS2-B
TCONS_00000179	0.188249	-0.0375592	0.943839	BGIOSGA002752	XP_015688126.1 protein root UVB sensitive 5
TCONS_00001426	0.188249	0.569713	0.183533	BGIOSGA004055	XP_006644408.1 SNF1-related protein kinase regulatory subunit gamma-1-like
TCONS_00024310	0.188272	-0.207924	0.916829	BGIOSGA020890	XP_006658947.1 BTB/POZ domain-containing protein At3g05675
TCONS_00023932	0.188296	-1.28406	-0.339377	BGIOSGA021712	XP_004958062.1scarecrow-like protein 23
TCONS_00018392	0.188308	0.144334	-0.476384	BGIOSGA023790	XP_006654846.1 serine carboxypeptidase-like 50
TCONS_00004383	0.188342	-0.895072	-1.49477	BGIOSGA005549	XP_006644697.1 mannan endo-1,4-beta-mannosidase 2
TCONS_00016794	0.188342	-0.327183	0.295511	BGIOSGA014173	XP_015691658.1 inositol-pentakisphosphate 2-kinase IPK1 isoform X2
TCONS_00018389	0.188353	1.95957	-0.594334	BGIOSGA023790	XP_006654846.1 serine carboxypeptidase-like 50
TCONS_00005531	0.188549	-1.87557	0.435946	BGIOSGA007642	XP_006646948.2 ribosomal RNA large subunit methyltransferase I
TCONS_00009905	0.18859	0.0325616	0.319352	BGIOSGA012460	XP_006649959.1 peptide chain release factor PrfB2, chloroplastic
TCONS_00037425	0.18862	0.278061	-0.0100302	BGIOSGA003959	XP_006664147.1PREDICTED: uncharacterized protein LOC102709514, partial
TCONS_00036591	0.188659	1.11602	0.805447	BGIOSGA006482	XP_006664296.1 probable arabinosyltransferase ARAD1
TCONS_00000381	0.188659	1.19764	-0.943014	BGIOSGA028723	XP_003565468.1beta-glucuronosyltransferase GlcAT14B
TCONS_00009067	0.188698	-0.0222771	0.398056	BGIOSGA005337	XP_015689493.1 acyl-CoA-binding domain-containing protein 4 isoform X2
TCONS_00025153	0.188727	-0.0893069	-1.19271	BGIOSGA031408	XP_006657734.2 protein DETOXIFICATION 19-like
TCONS_00034881	0.188882	-0.211015	-2.76556	BGIOSGA031129	XP_004979279.1cytochrome P450 78A5
TCONS_00007043	0.188959	0.578664	0.229025	BGIOSGA028416	XP_006648066.2 probable monogalactosyldiacylglycerol synthase 3, chloroplastic
TCONS_00001419	0.188967	-1.1474	-1.33282	BGIOSGA032260	XP_006644403.1 probable receptor-like protein kinase At2g42960
TCONS_00023274	0.189024	1.03069	0.000651917	BGIOSGA026471	XP_006657527.1 AAA-ATPase At3g28580-like isoform X1
TCONS_00000846	0.189032	0.330032	0.100977	BGIOSGA040458	XP_006644144.1 AMSH-like ubiquitin thioesterase 3 isoform X1
TCONS_00011221	0.189049	0.987059	0.731414	BGIOSGA019715	XP_015691306.1 multiple inositol polyphosphate phosphatase 1
TCONS_00036118	0.189149	0.737786	-0.547391	BGIOSGA005583	XP_003575954.3dicarboxylate transporter 1, chloroplastic
TCONS_00026894	0.189149	0.744618	0.387211	BGIOSGA030378	XP_015696142.1 beta-(1,2)-xylosyltransferase
TCONS_00006104	0.189187	1.24706	-0.399833	BGIOSGA035935	XP_015689216.1 flavonoid 3'-monooxygenase-like
TCONS_00001308	0.189194	0.344172	0.688991	BGIOSGA014062	XP_002455911.1uncharacterized protein LOC8082509
TCONS_00032372	0.189246	-0.527329	0.762759	BGIOSGA027127	XP_006650259.2 psbP-like protein 1, chloroplastic isoform X2
TCONS_00006660	0.189266	0.820519	0.661209	BGIOSGA004740	XP_006647698.1 calcium-dependent protein kinase 26-like
TCONS_00022149	0.189335	-0.925466	-2.33372	BGIOSGA022821	XP_022681827.1salicylate carboxymethyltransferase
TCONS_00031350	0.189348	0.308982	0.222635	BGIOSGA033164	XP_006662461.1 nijmegen breakage syndrome 1 protein
TCONS_00006534	0.189446	-0.388312	0.0981123	BGIOSGA021893	XP_015689367.1 O-glucosyltransferase rumi homolog
TCONS_00016495	0.189538	0.283044	0.824852	BGIOSGA017726	XP_006653709.2 glutamate receptor 3.1
TCONS_00024679	0.189596	0.655893	-0.275476	BGIOSGA013092	XP_015694852.1 probable serine/threonine-protein kinase WNK3 isoform X2
TCONS_00036143	0.189596	0.942416	0.862027	BGIOSGA037499	XP_006664599.1 AP-5 complex subunit mu
TCONS_00004026	0.189658	-0.111253	1.08487	BGIOSGA035109	XP_006644399.1 mitogen-activated protein kinase 10 isoform X2
TCONS_00005540	0.189712	-1.73329	-1.74969	BGIOSGA020744	XP_006646955.1 V-type proton ATPase catalytic subunit A-like
TCONS_00007458	0.189754	-1.53072	-0.0359141	BGIOSGA030792	XP_015689648.1 protein ECERIFERUM 3-like
TCONS_00013394	0.18982	0.645832	1.08134	BGIOSGA017463	XP_006650773.2 mitochondrial Rho GTPase 1
TCONS_00006966	0.189882	-0.317894	0.93524	BGIOSGA003352	XP_006649096.2PREDICTED: uncharacterized protein LOC102721055
TCONS_00022199	0.189915	0.753664	0.961536	BGIOSGA021296	XP_021305112.1RNA-binding KH domain-containing protein RCF3
TCONS_00002115	0.189928	0.467056	1.07694	BGIOSGA004743	XP_004970460.2pentatricopeptide repeat-containing protein At5g39980, chloroplastic
TCONS_00026897	0.190012	1.13258	0.747345	BGIOSGA019961	XP_002445914.1nucleolin 1 isoform X1
TCONS_00006432	0.190025	-0.394411	0.336086	BGIOSGA034193	XP_006664907.1 acetolactate synthase small subunit 2, chloroplastic-like
TCONS_00008524	0.190054	0.0926771	-0.191908	BGIOSGA005902	XP_015688684.1PREDICTED: uncharacterized protein LOC102708416
TCONS_00004147	0.190135	0.425098	0.32532	BGIOSGA000812	XP_006644500.1 auxin response factor 2-like
TCONS_00018988	0.190169	0.726207	0.649112	BGIOSGA023019	XP_006659268.1 crooked neck-like protein 1
TCONS_00004528	0.190199	-0.455046	-1.55707	BGIOSGA014006	XP_015690164.1 receptor-like serine/threonine-protein kinase SD1-8
TCONS_00000924	0.190217	-0.948053	0.128047	BGIOSGA015816	XP_021311050.1wall-associated receptor kinase 5
TCONS_00035392	0.190305	-1.69245	0.226257	BGIOSGA035676	XP_015698052.1 myb-related protein MYBAS1-like isoform X1
TCONS_00035047	0.190417	-0.233913	0.229461	BGIOSGA020165	XP_006661557.2PREDICTED: uncharacterized protein LOC102715238

TCONS_00035420	0.1905	-0.348224	1.04946	BGIOSGA018538	XP_015698258.1PREDICTED: uncharacterized protein LOC102701320, partial
TCONS_00022558	0.1905	-0.720476	-0.545183	BGIOSGA019734	XP_015693926.1 protein QUIRKY-like
TCONS_00031314	0.190665	-0.125279	0.25203	BGIOSGA033130	XP_002467083.1exportin-4 isoform X1
TCONS_00004193	0.190679	-1.0458	0.022365	BGIOSGA001008	XP_006646213.1 DNA ligase 3 isoform X1
TCONS_00011789	0.190773	0.502607	0.270712	BGIOSGA011215	XP_006649632.1 nuclear receptor coactivator 6-like isoform X2
TCONS_00029879	0.190877	0.768414	0.723641	BGIOSGA017545	XP_002462259.1uncharacterized protein LOC8055553
TCONS_00006181	0.190961	0.825855	0.848136	BGIOSGA027158	XP_006647325.2 putative SWI/SNF-related matrix-associated actin-dependent regulator of chromatin subfamily A member 3-like 2
TCONS_00027808	0.191027	0.829262	0.747144	BGIOSGA003349	XP_006659300.1 ribonuclease E/G-like protein, chloroplastic isoform X1
TCONS_00034346	0.191038	0.656393	0.776776	BGIOSGA034458	XP_015698043.1PREDICTED: uncharacterized protein LOC102714235 isoform X1
TCONS_00022629	0.191071	0.982452	1.5113	BGIOSGA001690	XP_006657173.2 probable E3 ubiquitin ligase SUD1
TCONS_00012097	0.191206	0.033522	0.589563	BGIOSGA010914	XP_006649938.1 U2 small nuclear ribonucleoprotein B'' isoform X1
TCONS_00004022	0.191257	0.31935	-0.00969814	BGIOSGA001269	XP_006646125.1 cytochrome P450 72A14-like
TCONS_00005892	0.191262	0.862005	0.777008	BGIOSGA010512	XP_015688562.1 probable pre-mRNA-splicing factor ATP-dependent RNA helicase DEAH5
TCONS_00007091	0.191306	0.0329928	-0.411448	BGIOSGA031926	XP_021315271.1glutathione reductase, cytosolic
TCONS_00021373	0.19135	-0.676936	-0.293092	BGIOSGA008711	XP_003563566.1uncharacterized protein LOC100837238
TCONS_00013112	0.191601	-0.256691	-0.328331	BGIOSGA009875	XP_004981897.1prostamide/prostaglandin F synthase
TCONS_00000923	0.19161	1.36313	0.40641	BGIOSGA016114	XP_006645891.1 protein HASTY 1
TCONS_00025714	0.191683	0.421438	0.640072	BGIOSGA005100	XP_015695228.1 E3 ubiquitin-protein ligase SHPRH
TCONS_00005316	0.191694	0.980065	0.622222	BGIOSGA007426	XP_015689236.1 vacuolar protein sorting-associated protein 8 homolog
TCONS_00006285	0.192088	-0.437978	0.357566	BGIOSGA021586	XP_002452299.1NADH dehydrogenase [ubiquinone] 1 beta subcomplex subunit 3-B
TCONS_00028058	0.192222	0.316089	-0.42421	BGIOSGA013292	XP_006660157.2 myb family transcription factor APL-like
TCONS_00034169	0.192506	-1.09656	-0.523432	BGIOSGA000316	XP_006663744.1 probable non-specific lipid-transfer protein 3
TCONS_00035740	0.192602	0.176193	-0.638133	BGIOSGA037151	XP_006663904.1 glutathione S-transferase-like
TCONS_00032488	0.192756	-1.02533	0.0288007	BGIOSGA031630	XP_006662471.1PREDICTED: uncharacterized protein LOC102702787
TCONS_00007489	0.192881	-0.175111	-1.33406	BGIOSGA019309	XP_006647011.1 cinnamyl alcohol dehydrogenase 2
TCONS_00011342	0.19317	0.772861	0.494186	BGIOSGA031670	XP_002466094.1basic transcription factor 3
TCONS_00025371	0.193391	0.933484	0.684692	BGIOSGA025377	XP_012699680.1remorin 4.1
TCONS_00025502	0.193665	0.353463	0.69847	BGIOSGA012869	XP_002463160.1mitochondrial import inner membrane translocase subunit TIM17-2
TCONS_00024335	0.1937	0.172057	0.788805	BGIOSGA010760	XP_006658114.1 60S ribosomal protein L22-2
TCONS_00032569	0.194557	-0.308701	0.88525	BGIOSGA005932	XP_006661960.1 cytochrome b5
TCONS_00002609	0.194648	-0.133283	0.474411	BGIOSGA019021	XP_006645340.1PREDICTED: uncharacterized protein LOC102711847 isoform X1
TCONS_00020734	0.194743	0.0142388	-0.377813	BGIOSGA033878	XP_010228769.1uncharacterized protein LOC104581804
TCONS_00029328	0.196467	-0.480225	-0.154227	BGIOSGA029079	XP_004974050.1E3 ubiquitin-protein ligase RFI2 isoform X1
TCONS_00031856	0.196525	0.49345	0.796815	BGIOSGA019049	XP_006659016.1 vesicle-associated membrane protein 714
TCONS_00013282	0.196844	0.717861	0.279197	BGIOSGA006650	XP_006650687.2 cell differentiation protein RCD1 homolog
TCONS_00024715	0.19705	0.177518	-0.57507	BGIOSGA024662	XP_002445975.1NAD(P)H-quinone oxidoreductase subunit S, chloroplastic
TCONS_00003231	0.197133	-0.0636463	0.24122	BGIOSGA021952	XP_006644002.2 receptor-like protein kinase ANXUR1
TCONS_00032572	0.1973	0.276525	-0.987146	BGIOSGA030859	XP_002464362.1AAA-ATPase At2g46620
TCONS_00025941	0.197425	-0.153605	0.328384	BGIOSGA029768	XP_006659132.1 60S ribosomal protein L34-like
TCONS_00032739	0.197426	1.51905	0.239461	BGIOSGA031366	XP_006662084.1 eukaryotic translation initiation factor 3 subunit B-like
TCONS_00011428	0.197601	-1.65458	-0.568112	BGIOSGA011596	NP_001132515.1CemA-like proton extrusion protein-related
TCONS_00017232	0.198142	0.0224002	-0.153322	BGIOSGA029989	XP_015692736.1 E3 ubiquitin-protein ligase At1g63170-like isoform X1
TCONS_00018201	0.198679	-0.291715	0.308828	BGIOSGA001313	XP_015692871.1PREDICTED: uncharacterized protein LOC102723056
TCONS_00021396	0.19868	0.783589	0.711322	BGIOSGA029032	XP_002437607.2protein FREE1 isoform X1
TCONS_00004808	0.198807	-0.663294	0.375721	BGIOSGA000407	XP_006646523.1PREDICTED: uncharacterized protein LOC102705217
TCONS_00000498	0.199085	0.321231	0.26154	BGIOSGA013743	XP_006643951.2 activating signal cointegrator 1 complex subunit 1 isoform X1
TCONS_00024928	0.199115	0.0994041	0.31794	BGIOSGA003690	XP_006660440.1 trafficking protein particle complex subunit 6B
TCONS_00019827	0.199126	-0.059022	-0.0839689	BGIOSGA014210	XP_004961164.1uncharacterized protein LOC101785881 isoform X2
TCONS_00037775	0.199262	0.188007	0.0825359	BGIOSGA008642	XP_006653926.1 histone-lysine N-methyltransferase SUVR4-like isoform X1
TCONS_00019001	0.19931	0.293787	0.247399	BGIOSGA018364	XP_006654230.2 lactoylglutathione lyase isoform X1
TCONS_00018109	0.199467	0.109705	-0.216196	BGIOSGA033806	XP_006654635.1 transcription elongation factor SPT5-like
TCONS_00022717	0.199827	0.893811	0.559875	BGIOSGA036687	XP_006656337.1 serine/threonine-protein kinase At5g01020
TCONS_00018612	0.19983	0.465052	0.653241	BGIOSGA021779	XP_015693242.1 probable ATP-dependent DNA helicase CHR12
TCONS_00007617	0.199848	-0.204261	-0.127116	BGIOSGA025496	XP_004951449.1CASP-like protein 2D1

TCONS_00032721	0.199906	-1.6099	0.403826	BGIOSGA012138	XP_006662074.1 serine/threonine-protein kinase SAPK3
TCONS_00000578	0.199954	-0.0963226	-1.33379	BGIOSGA002775	NP_001130618.1uncharacterized protein LOC100191717
TCONS_00028048	0.200096	0.369308	1.16895	BGIOSGA026942	XP_006659424.1 ultraviolet-B receptor UVR8-like
TCONS_00019781	0.200182	0.710073	0.0743232	BGIOSGA001453	XP_004961249.1auxin-responsive protein IAA19
TCONS_00011112	0.200183	0.357038	0.333454	BGIOSGA028229	XP_014751919.1BTB/POZ and MATH domain-containing protein 4 isoform X1
TCONS_00004620	0.20022	0.547451	0.375906	BGIOSGA000596	XP_006646429.1 protein MOR1
TCONS_00012278	0.200279	0.935096	0.623707	BGIOSGA018160	XP_006650086.1 acyl carrier protein 2, mitochondrial-like
TCONS_00017234	0.200386	-0.0975624	0.0872699	BGIOSGA002889	XP_006654106.1 nucleolin-like
TCONS_00000418	0.200727	-1.25933	-1.51952	BGIOSGA019359	XP_006643904.1PREDICTED: uncharacterized protein LOC102713673
TCONS_00022257	0.201113	0.459476	0.0708989	BGIOSGA005770	XP_006656088.1 U-box domain-containing protein 6-like
TCONS_00017745	0.201215	1.09541	0.794302	BGIOSGA008534	XP_015692587.1 decapping nuclease DXO homolog, chloroplastic
TCONS_00032983	0.201235	0.886931	0.0506167	BGIOSGA034818	XP_015698067.1 signal recognition particle 54 kDa protein, chloroplastic-like
TCONS_00005158	0.201443	0.018497	0.285094	BGIOSGA024865	XP_004971382.1protein COFACTOR ASSEMBLY OF COMPLEX C SUBUNIT B CCB3, chloroplastic
TCONS_00001357	0.201718	-0.467732	0.364084	BGIOSGA003989	XP_006644367.1PREDICTED: uncharacterized protein At1g03900
TCONS_00001673	0.201956	0.661617	0.698509	BGIOSGA004308	XP_006644601.1 WD-40 repeat-containing protein MSI4 isoform X2
TCONS_00011553	0.202015	1.01774	0.06144	BGIOSGA011467	XP_006649392.1PREDICTED: uncharacterized protein LOC102701985
TCONS_00006520	0.202094	0.97799	0.0939928	BGIOSGA000463	XP_006647581.1 RNA polymerase II C-terminal domain phosphatase-like 1
TCONS_00004168	0.202245	-0.659521	-0.363039	BGIOSGA020345	XP_006644511.1 probable glucuronosyltransferase Os01g0675500
TCONS_00013428	0.20227	-3.28266	-1.45084	BGIOSGA020192	XP_006650802.1 chalcone--flavonone isomerase
TCONS_00013768	0.202279	0.457799	0.00459564	BGIOSGA015532	XP_006663938.1 vacuolar protein sorting-associated protein 41 homolog
TCONS_00008456	0.202329	0.287701	0.344894	BGIOSGA025480	XP_006648869.1PREDICTED: uncharacterized protein LOC102720212
TCONS_00037532	0.202509	-0.00417534	0.197328	BGIOSGA026102	XP_004963215.1putative hydrolase C777.06c
TCONS_00031576	0.202745	-0.238228	-0.0214047	BGIOSGA033391	XP_014755503.1DDB1- and CUL4-associated factor 8
TCONS_00018050	0.202798	0.8462	0.693752	BGIOSGA010140	XP_006654585.1 cytochrome P450 98A1
TCONS_00017312	0.202812	0.0363748	0.487182	BGIOSGA019343	XP_006654144.1 serine/threonine-protein phosphatase 5
TCONS_00015202	0.202871	0.352216	0.602052	BGIOSGA025968	XP_006653052.1 pentatricopeptide repeat-containing protein At5g04810, chloroplastic
TCONS_00030862	0.202983	-0.0195729	-0.645277	BGIOSGA032169	XP_004956162.1uncharacterized protein LOC101776137
TCONS_00026779	0.203427	0.48198	0.6498	BGIOSGA035204	XP_006659495.1 protein transport protein SEC23
TCONS_00013032	0.203679	-0.242052	0.17077	BGIOSGA025111	XP_015691036.1PREDICTED: uncharacterized protein LOC102707476 isoform X1
TCONS_00012940	0.203853	0.284165	0.319715	BGIOSGA025510	XP_015690016.1 serine/threonine protein phosphatase 2A 57 kDa regulatory subunit B' beta isoform-like
TCONS_00025946	0.204468	1.33366	1.11639	BGIOSGA027611	XP_015695502.1 stomatin-like protein 2, mitochondrial
TCONS_00006514	0.204647	-0.700926	-0.501255	BGIOSGA033672	XP_022685122.1receptor kinase-like protein Xa21
TCONS_00026398	0.204927	0.233203	1.09781	BGIOSGA030091	XP_006659310.1 protein EMSY-LIKE 3 isoform X2
TCONS_00027631	0.205016	-0.0876363	-0.00187262	BGIOSGA027387	XP_006659240.1 (DL)-glycerol-3-phosphatase 2 isoform X2
TCONS_00015035	0.205079	-0.564511	-0.541348	BGIOSGA014249	XP_006652892.1 uncharacterized aarF domain-containing protein kinase At1g71810, chloroplastic
TCONS_00030422	0.205203	-1.92166	-0.4889	BGIOSGA016820	XP_006660886.1 protein ODORANT1-like
TCONS_00024779	0.205448	0.984815	1.65697	BGIOSGA025767	XP_006657560.2 eukaryotic translation initiation factor 3 subunit E
TCONS_00010290	0.205671	-0.0166676	0.653938	BGIOSGA012854	XP_006651496.2 ATP-dependent Clp protease proteolytic subunit 6, chloroplastic isoform X2
TCONS_00012601	0.205685	1.9652	0.295551	BGIOSGA015033	XP_015689957.1 probable membrane metalloprotease ARASP2, chloroplastic
TCONS_00023492	0.206001	-0.276289	-0.163679	BGIOSGA025530	XP_006657615.1PREDICTED: uncharacterized protein LOC102708261 isoform X1
TCONS_00009407	0.206157	0.456917	0.710066	BGIOSGA010409	XP_006649483.1 polyadenylate-binding protein RBP47B'
TCONS_00028044	0.206158	0.308563	0.222181	BGIOSGA026946	XP_006659419.1PREDICTED: uncharacterized protein LOC102711722 isoform X1
TCONS_00007891	0.206188	0.0890715	-0.417211	BGIOSGA006585	XP_015688859.1 glutathionyl-hydroquinone reductase YqjG-like isoform X1
TCONS_00021534	0.206348	-0.474843	0.00010942	BGIOSGA022016	XP_006655740.1 BTB/POZ domain-containing protein At3g05675-like
TCONS_00024695	0.206414	0.939094	1.07362	BGIOSGA025679	XP_015695344.1PREDICTED: uncharacterized protein At4g14100-like
TCONS_00030132	0.206492	-0.486477	-0.174394	BGIOSGA029674	XP_006660669.1 phospholipid:diacylglycerol acyltransferase 1-like
TCONS_00025264	0.206551	1.24101	0.224891	BGIOSGA024030	XP_015695278.1 cysteine-rich receptor-like protein kinase 10
TCONS_00000042	0.206604	-0.261783	-0.0146478	BGIOSGA002610	XP_006648166.1PREDICTED: uncharacterized protein LOC102709903
TCONS_00009813	0.206755	-0.840761	-1.1332	BGIOSGA027395	XP_015689850.1 thioredoxin-like protein AAED1, chloroplastic

TCONS_00002305	0.206955	-0.121462	-0.311821	BGIOSGA002195	XP_021312164.1protein root UVB sensitive 6-like
TCONS_00001543	0.207024	0.353411	0.310295	BGIOSGA004652	XP_006644494.2PREDICTED: uncharacterized protein LOC102699448
TCONS_00025961	0.207332	0.312279	-1.71365	BGIOSGA028037	XP_015695734.1PREDICTED: uncharacterized protein LOC102699330 isoform X2
TCONS_00021161	0.207465	1.52214	0.13682	BGIOSGA020765	XP_006656315.1 probable GDP-L-fucose synthase 1
TCONS_00004032	0.207471	1.26282	0.0241468	BGIOSGA001162	NP_001136758.150S ribosomal protein L31
TCONS_00000600	0.207626	0.503204	0.074719	BGIOSGA031081	XP_006644013.1 serine/threonine-protein kinase CDL1-like isoform X2
TCONS_00001770	0.207666	-0.756021	-1.13937	BGIOSGA020004	XP_021312878.1respiratory burst oxidase homolog protein A
TCONS_00019045	0.207911	0.877776	0.707997	BGIOSGA018316	XP_015693185.1 pentatricopeptide repeat-containing protein At5g67570, chloroplastic-like
TCONS_00036458	0.207972	0.518579	-0.482674	BGIOSGA039527	XP_006664220.1 5-methyltetrahydropteroyltriglutamate--homocysteine methyltransferase 1
TCONS_00035405	0.207995	2.01353	0.638473	BGIOSGA010085	XP_006663687.2 glucan endo-1,3-beta-glucosidase-like
TCONS_00014452	0.208064	0.964708	0.382479	BGIOSGA016612	XP_006652420.2 SWI/SNF complex subunit SWI3A-like
TCONS_00012522	0.208252	-0.392655	-0.787473	BGIOSGA010460	XP_015690723.1 transcription initiation factor TFIID subunit 4b-like isoform X3
TCONS_00025122	0.208333	0.42847	-0.0722442	BGIOSGA031391	XP_006657719.1 nucleoside diphosphate kinase 1
TCONS_00038151	0.208473	0.193923	-0.904611	BGIOSGA037260	XP_006663954.1 ribulose bisphosphate carboxylase small chain, chloroplastic-like
TCONS_00028486	0.208483	0.792049	-0.214601	BGIOSGA026470	XP_006659714.1 zinc finger protein 593 homolog
TCONS_00035683	0.208535	-0.416793	0.029054	BGIOSGA014304	XP_015698423.1 amino acid permease 3
TCONS_00035969	0.208886	-0.472389	-1.2058	BGIOSGA010277	XP_015698744.1 UDP-glucose 6-dehydrogenase 5
TCONS_00010027	0.209053	0.0162704	0.67678	BGIOSGA036365	XP_006651352.1PREDICTED: uncharacterized protein LOC102715929
TCONS_00018192	0.209095	-0.355265	1.05907	BGIOSGA039709	XP_006655521.2 CLP protease regulatory subunit CLPX3, mitochondrial-like
TCONS_00011898	0.209115	1.5674	0.434793	BGIOSGA011117	XP_006649735.1 ATP-dependent Clp protease ATP-binding subunit CLPT1, chloroplastic-like
TCONS_00025579	0.209314	0.662751	-0.179021	BGIOSGA036000	XP_021307931.1uncharacterized protein LOC8079378
TCONS_00011174	0.209336	-1.07015	0.20361	BGIOSGA025979	XP_006650760.1PREDICTED: uncharacterized protein LOC102720690
TCONS_00020089	0.209425	0.146425	1.04309	BGIOSGA022265	XP_003557221.1coiled-coil domain-containing protein 97
TCONS_00010800	0.209621	-0.1936	0.131476	BGIOSGA024442	XP_003559518.1zinc finger protein VAR3, chloroplastic
TCONS_00022643	0.209659	0.0431384	0.403514	BGIOSGA015054	XP_015693859.1 formamidopyrimidine-DNA glycosylase-like
TCONS_00034535	0.20967	-0.422944	-1.06777	BGIOSGA018344	XP_006662827.1 uncharacterized aarF domain-containing protein kinase At4g31390, chloroplastic isoform X1
TCONS_00013444	0.20972	0.218513	0.288367	BGIOSGA037684	XP_006650817.1 nucleobase-ascorbate transporter 6-like
TCONS_00021080	0.210188	0.00440633	0.0778437	BGIOSGA023307	XP_006656270.1 papilin
TCONS_00019505	0.210487	0.230216	1.06209	BGIOSGA017853	XP_006654551.1 B3 domain-containing protein Os05g0481400-like
TCONS_00018976	0.210618	1.03714	0.591021	BGIOSGA018388	XP_006654207.2 peptide chain release factor 1 isoform X1
TCONS_00020065	0.210646	0.807535	0.557882	BGIOSGA022246	XP_003557242.13-phosphoshikimate 1-carboxyvinyltransferase 2
TCONS_00009434	0.210685	-1.11488	-1.03992	BGIOSGA032002	XP_015689789.1 protein TIFY 11b-like
TCONS_00007189	0.210925	1.302	1.16658	BGIOSGA007309	XP_003573697.1zinc finger CCCH domain-containing protein 13
TCONS_00003300	0.210983	0.513327	0.828522	BGIOSGA001913	XP_006644045.1PREDICTED: uncharacterized protein LOC102707253
TCONS_00021140	0.211056	1.05352	0.852712	BGIOSGA007008	XP_006656298.1 isoleucine--tRNA ligase, cytoplasmic-like
TCONS_00032383	0.211179	-0.172811	0.360737	BGIOSGA032168	XP_006647129.1 bifunctional riboflavin kinase/FMN phosphatase-like
TCONS_00016106	0.211199	1.74692	1.17549	BGIOSGA031359	XP_006653524.1PREDICTED: uncharacterized protein LOC102708054
TCONS_00014390	0.211308	-0.684128	0.717584	BGIOSGA016545	XP_006652367.1 cyclic pyranopterin monophosphate synthase accessory protein, mitochondrial
TCONS_00031571	0.21132	0.532575	0.223325	BGIOSGA035864	XP_006662016.2 probable serine/threonine-protein kinase At4g35230
TCONS_00009570	0.211493	0.785821	0.528147	BGIOSGA012201	XP_006651169.1 cysteine synthase 2
TCONS_00011156	0.211582	-0.398987	-1.02059	BGIOSGA011934	XP_015690116.1 protein YLS3
TCONS_00006600	0.211623	-0.577118	0.209865	BGIOSGA008767	XP_006647647.1 tripeptidyl-peptidase 2
TCONS_00006914	0.211706	0.016369	0.104599	BGIOSGA008494	XP_006649065.1 MADS-box transcription factor 22
TCONS_00024077	0.211715	0.365417	0.380805	BGIOSGA028956	XP_004958286.1stress-response A/B barrel domain-containing protein UP3
TCONS_00021036	0.211743	0.589057	0.117388	BGIOSGA032254	XP_003563624.1TBCC domain-containing protein 1
TCONS_00013036	0.211912	1.12857	0.217758	BGIOSGA027614	XP_006650476.2 DEAD-box ATP-dependent RNA helicase 21
TCONS_00006341	0.212002	-0.434444	1.00778	BGIOSGA008503	XP_006647426.2 protein PHYLLO, chloroplastic
TCONS_00015400	0.212067	-0.0896874	0.353767	BGIOSGA015973	XP_004975144.1electron transfer flavoprotein subunit beta, mitochondrial
TCONS_00010771	0.212158	0.949069	0.940853	BGIOSGA024034	XP_006650446.1 importin-5-like
TCONS_00018102	0.212259	-1.54661	-0.755075	BGIOSGA020157	XP_006644788.1 photosystem II reaction center W protein, chloroplastic
TCONS_00004751	0.212369	-0.18003	0.959103	BGIOSGA000465	XP_006645043.2 nuclear pore complex protein NUP62

TCONS_00009880	0.212395	0.0512742	0.808451	BGIOSGA012434	XP_006649940.2 probable LL-diaminopimelate aminotransferase, chloroplastic
TCONS_00008882	0.212457	-0.117989	-1.13566	BGIOSGA005530	XP_006647974.1 E3 ubiquitin-protein ligase COP1
TCONS_00006747	0.212803	1.18673	1.86281	BGIOSGA026594	XP_006647790.1 ribose-phosphate pyrophosphokinase 4
TCONS_00018461	0.212967	-0.377358	-0.281031	BGIOSGA027876	XP_006654897.2 E3 ubiquitin-protein ligase CHIP-like
TCONS_00025992	0.213121	0.222742	0.477618	BGIOSGA028066	XP_006659174.1PREDICTED: uncharacterized protein LOC102704722
TCONS_00033131	0.213329	0.663884	0.538786	BGIOSGA025493	XP_006663270.2 protein translocase subunit SECA2, chloroplastic
TCONS_00008545	0.213402	0.371689	0.458816	BGIOSGA015055	XP_006647655.1 mitogen-activated protein kinase kinase kinase YODA-like
TCONS_00002841	0.213602	0.268481	0.259653	BGIOSGA001568	XP_010230326.1histone H2B.3
TCONS_00006690	0.213658	0.275495	-1.27914	BGIOSGA033500	XP_006647728.1 lysine-specific demethylase JMJ706-like
TCONS_00030326	0.213727	1.13329	1.04023	BGIOSGA026611	XP_015696826.1 ubiquitin carboxyl-terminal hydrolase 2-like
TCONS_00018908	0.213989	0.0999324	0.618088	BGIOSGA018990	NP_001146919.1OB-fold nucleic acid binding domain containing protein
TCONS_00024708	0.214212	-0.716897	-0.512288	BGIOSGA022553	XP_015694803.1 probable beta-1,3-galactosyltransferase 18
TCONS_00005085	0.214225	-0.423978	-0.296112	BGIOSGA003961	XP_004971226.1amino-acid permease BAT1 homolog isoform X1
TCONS_00027447	0.21423	1.00098	0.988154	BGIOSGA001004	XP_015696125.1 IST1-like protein isoform X2
TCONS_00021531	0.214286	0.658123	0.353788	BGIOSGA022019	XP_006655738.1 LIMR family protein Os06g0128200
TCONS_00037427	0.214337	-0.168637	0.513078	BGIOSGA011239	XP_003579243.1metal transporter Nramp6
TCONS_00019879	0.214582	0.504702	-0.648751	BGIOSGA010862	XP_006654844.1 protein REDUCED WALL ACETYLATION 3-like
TCONS_00032719	0.214641	1.18246	0.511909	BGIOSGA006688	XP_002437450.160S ribosomal protein L27-3
TCONS_00017625	0.215017	0.397053	1.27835	BGIOSGA019687	XP_006654294.1 developmentally-regulated G-protein 2
TCONS_00015467	0.215507	0.707793	0.164935	BGIOSGA015553	XP_010239729.1uncharacterized protein LOC100830596 isoform X1
TCONS_00024227	0.215565	0.893443	0.304536	BGIOSGA026271	XP_006658903.1 mediator of RNA polymerase II transcription subunit 12-like
TCONS_00035086	0.215813	0.169036	0.81748	BGIOSGA034024	XP_021317268.1formin-like protein 14
TCONS_00025912	0.215993	0.981711	-0.913317	BGIOSGA036682	NP_001152070.1phloem-specific lectin
TCONS_00016268	0.216257	0.324266	0.745951	BGIOSGA022591	XP_014751391.1adenosine kinase 2
TCONS_00026876	0.216277	0.728448	0.498944	BGIOSGA004288	XP_002445942.1chloride channel protein CLC-f
TCONS_00002572	0.216292	-0.00851847	-0.0421986	BGIOSGA024758	XP_006645361.1 MACPF domain-containing protein At4g24290-like
TCONS_00022361	0.216379	-3.91751	-0.544107	BGIOSGA003005	XP_006656143.1 GDSL esterase/lipase At5g45910-like
TCONS_00027795	0.216991	0.643475	0.213027	BGIOSGA030237	XP_006660038.1 kynurenine formamidase
TCONS_00023163	0.217013	-0.0671857	0.0946324	BGIOSGA025201	XP_006650028.1 probable dolichyl pyrophosphate Man9GlcNAc2 alpha-1,3-glucosyltransferase
TCONS_00017206	0.21706	0.207524	-0.535795	BGIOSGA035020	XP_006659182.1 60S ribosomal protein L10-2-like
TCONS_00032639	0.217159	0.338505	0.789132	BGIOSGA009838	XP_015697472.1 putative magnesium transporter MRS2-G
TCONS_00025168	0.217255	-0.401662	0.428565	BGIOSGA024246	XP_006657741.1 serine/threonine-protein phosphatase 7 long form homolog
TCONS_00035964	0.217417	-1.65046	-1.188	BGIOSGA037328	XP_015698461.1PREDICTED: uncharacterized protein LOC102714335
TCONS_00029096	0.217468	0.517056	-0.0815874	BGIOSGA022355	XP_006660665.1 probable GTP diphosphokinase RSH3, chloroplastic
TCONS_00033856	0.217545	0.246319	0.152305	BGIOSGA035631	XP_006663052.1 monosaccharide-sensing protein 2-like
TCONS_00019651	0.217695	-0.223727	0.164013	BGIOSGA022332	XP_006654671.1 putative dual specificity protein phosphatase DSP8
TCONS_00010223	0.218079	0.156791	-0.174684	BGIOSGA012788	XP_006650155.1 cytochrome c oxidase subunit 6b-1-like
TCONS_00021924	0.218252	-0.270406	1.19899	BGIOSGA021599	XP_006655948.1 DCN1-like protein 2
TCONS_00024157	0.218294	0.92962	1.34667	BGIOSGA034875	XP_006658862.2 29 kDa ribonucleoprotein A, chloroplastic-like
TCONS_00012127	0.218448	-0.232497	0.218849	BGIOSGA010881	XP_006651312.1 histone-lysine N-methyltransferase EZ3 isoform X1
TCONS_00036231	0.218568	0.347238	0.351937	BGIOSGA011654	XP_006664099.1 DNA-directed RNA polymerases II, IV and V subunit 9B
TCONS_00023448	0.218661	-0.77068	-0.674207	BGIOSGA025480	XP_015694997.1PREDICTED: uncharacterized protein LOC102722879
TCONS_00022647	0.218755	-0.180364	0.746865	BGIOSGA020795	XP_006657185.1 protein OS-9 homolog
TCONS_00011973	0.219039	0.117285	0.660951	BGIOSGA011037	XP_015690712.1 rootletin
TCONS_00024270	0.219086	0.3464	-0.656457	BGIOSGA028795	XP_004958553.1uncharacterized protein LOC101761302
TCONS_00012560	0.2192	0.0468293	-0.0556754	BGIOSGA020564	XP_006656081.1PREDICTED: uncharacterized protein LOC102719021
TCONS_00000479	0.219281	-1.79502	-3.56533	BGIOSGA003045	XP_003565894.2 cytochrome P450 71A1-like
TCONS_00009165	0.219313	-0.375397	-0.335896	BGIOSGA033155	XP_006650965.1PREDICTED: uncharacterized protein LOC102710288
TCONS_00032623	0.219408	-0.0241226	0.427572	BGIOSGA031487	XP_015697335.1PREDICTED: uncharacterized protein LOC102717830 isoform X2
TCONS_00013114	0.21942	-0.173115	-2.84082	BGIOSGA014953	XP_008665348.1 lecithin-cholesterol acyltransferase-like 1
TCONS_00037217	0.219437	1.08776	1.43985	BGIOSGA011496	XP_003576164.1GRF1-interacting factor 3
TCONS_00001520	0.219688	-0.0151594	0.681638	BGIOSGA004150	XP_015691837.1 AMSH-like ubiquitin thioesterase 2
TCONS_00035625	0.219694	1.38708	1.08823	BGIOSGA037043	XP_006664363.1 nucleolar complex protein 2 homolog
TCONS_00033419	0.219999	0.218973	-0.322572	BGIOSGA028564	XP_006661910.1 SKP1-like protein 1A

TCONS_00025464	0.220205	-0.724165	-1.12007	BGIOSGA025427	XP_006657914.1 eukaryotic translation initiation factor 5A
TCONS_00018103	0.220243	0.687599	0.664083	BGIOSGA020158	XP_006655470.2 NADH dehydrogenase [ubiquinone] flavoprotein 2, mitochondrial
TCONS_00018292	0.220285	0.0241113	0.114392	BGIOSGA001030	XP_006654773.1 probable glucuronosyltransferase Os05g0559600 isoform X1
TCONS_00004635	0.220309	0.245237	0.433217	BGIOSGA000581	XP_006644918.1 methionine--tRNA ligase, cytoplasmic
TCONS_00027073	0.220312	0.643721	0.240051	BGIOSGA031147	XP_006659661.1 putative ER lumen protein-retaining receptor C28H8.4
TCONS_00028382	0.220441	-0.232009	0.873064	BGIOSGA003259	XP_015695959.1 probable protein S-acyltransferase 7 isoform X1
TCONS_00025164	0.220667	-0.0910979	0.259172	BGIOSGA014377	XP_006657737.1 probable protein phosphatase 2C 62
TCONS_00003236	0.220742	0.804068	-0.0664927	BGIOSGA011089	XP_006644007.1 topless-related protein 2 isoform X1
TCONS_00029333	0.221055	0.447519	0.264364	BGIOSGA008872	XP_006660839.1 PTI1-like tyrosine-protein kinase 3
TCONS_00034192	0.221202	-2.10015	-1.49208	BGIOSGA006347	XP_015698002.1 serine/threonine-protein kinase At5g01020-like
TCONS_00023945	0.2214	0.758514	1.23725	BGIOSGA013491	XP_006658720.1 COMPASS-like H3K4 histone methylase component WDR5B
TCONS_00037487	0.22149	-0.0232165	0.723363	BGIOSGA017747	XP_015698357.1 protein tesmin/TSO1-like CXC 2
TCONS_00023154	0.221726	-1.62673	-1.85118	BGIOSGA010955	XP_004981498.1fasciclin-like arabinogalactan protein 16
TCONS_00026801	0.221765	-0.461322	0.0162144	BGIOSGA028863	XP_015696222.1 structural maintenance of chromosomes protein 2-like
TCONS_00032958	0.221776	-1.01063	-0.0416688	BGIOSGA034789	XP_004978652.1R3H domain-containing protein 4
TCONS_00007196	0.221867	0.628947	-1.08013	BGIOSGA022091	XP_003575004.1ferredoxin--NADP reductase, leaf isozyme 1, chloroplastic
TCONS_00007511	0.222239	0.777882	0.395477	BGIOSGA033127	XP_024318021.1probable serine/threonine protein phosphatase 2A regulatory subunit B''delta
TCONS_00019734	0.222315	-0.584632	-0.507424	BGIOSGA017626	XP_006654742.1 uncharacterized membrane protein YuiD-like
TCONS_00007382	0.222325	1.06281	1.33866	BGIOSGA007094	NP_001145047.1uncharacterized protein LOC100278233
TCONS_00036432	0.222344	-0.0866668	0.0680494	BGIOSGA037801	XP_004963206.1protease Do-like 5, chloroplastic isoform X2
TCONS_00005609	0.222373	0.600818	0.919891	BGIOSGA007722	XP_006646993.2 SWI/SNF complex subunit SWI3B
TCONS_00031373	0.222399	-2.70121	-0.565923	BGIOSGA007384	XP_012704257.1probable inactive receptor kinase At3g02880 isoform X2
TCONS_00006347	0.222437	0.657405	-0.0556816	BGIOSGA032170	XP_006647431.2 ras-related protein Rab-2-B-like
TCONS_00002487	0.222468	-0.181518	-1.98767	BGIOSGA005132	XP_006645301.1 photosystem II reaction center PSB28 protein, chloroplastic
TCONS_00006473	0.223256	-0.139206	-0.138243	BGIOSGA017448	XP_006647534.1 casein kinase I-like
TCONS_00029112	0.223322	0.199952	0.63828	BGIOSGA030864	XP_015696700.1 formate--tetrahydrofolate ligase
TCONS_00014656	0.223361	0.480981	0.164431	BGIOSGA008699	XP_006652567.1 DEAD-box ATP-dependent RNA helicase 6-like
TCONS_00035950	0.223691	-2.02808	-0.073114	BGIOSGA037318	XP_006663995.2PREDICTED: uncharacterized protein LOC102712128, partial
TCONS_00008847	0.223707	-0.437128	-0.103141	BGIOSGA005569	XP_006647948.1 putative 4-hydroxy-4-methyl-2-oxoglutarate aldolase 2
TCONS_00023373	0.22375	0.0173393	-0.148082	BGIOSGA008928	XP_015695413.1PREDICTED: uncharacterized protein LOC102718937
TCONS_00026939	0.224117	-0.0647458	0.37954	BGIOSGA028990	XP_003573122.1disheveled-associated activator of morphogenesis 2
TCONS_00007744	0.224224	0.690725	0.134438	BGIOSGA006737	XP_015689675.1 vacuolar protein sorting-associated protein 29
TCONS_00018403	0.224242	-4.61586	-0.457521	BGIOSGA001183	XP_006655638.1 probable protein ABIL4
TCONS_00030183	0.224288	0.364515	0.127378	BGIOSGA026799	XP_006660715.1 glucose-6-phosphate isomerase 1, chloroplastic-like
TCONS_00008372	0.224395	0.55271	0.124635	BGIOSGA035892	XP_006648833.1PREDICTED: uncharacterized protein LOC102707653
TCONS_00031256	0.224466	1.62953	1.33853	BGIOSGA008935	XP_006661829.1 splicing factor 3B subunit 4
TCONS_00011767	0.224481	1.21912	-0.000922633	BGIOSGA011238	XP_015691194.1 mannose-1-phosphate guanyltransferase alpha
TCONS_00020335	0.224526	0.252526	-0.0589866	BGIOSGA030888	XP_006655901.1 transmembrane emp24 domain-containing protein p24delta3-like
TCONS_00013660	0.22453	-0.72459	-0.241125	BGIOSGA011417	XP_006664948.1PREDICTED: uncharacterized protein LOC102709138
TCONS_00012658	0.224871	0.645761	0.220787	BGIOSGA010307	XP_006650294.1 protein DEK-like
TCONS_00030524	0.225136	1.26368	0.460988	BGIOSGA023376	XP_006661529.1 cytochrome b561, DM13 and DOMON domain-containing protein At5g54830-like
TCONS_00015905	0.225215	0.705218	1.29052	BGIOSGA003439	XP_006653412.1 protein disulfide isomerase-like 5-2
TCONS_00017991	0.225528	0.0572004	0.132033	BGIOSGA020040	XP_021303489.1aldose reductase
TCONS_00011256	0.225608	1.12796	0.564698	BGIOSGA029456	XP_006650828.2 non-specific phospholipase C1
TCONS_00024665	0.226067	0.174201	0.844726	BGIOSGA021762	XP_015694784.1 ruvB-like protein 1
TCONS_00023628	0.226176	0.811381	0.196024	BGIOSGA014528	XP_004956145.1E3 ubiquitin-protein ligase At1g12760 isoform X2
TCONS_00002987	0.226729	-0.388309	0.385803	BGIOSGA015052	XP_006645587.1 transcription factor MYB1R1-like
TCONS_00009370	0.227023	0.18489	0.400376	BGIOSGA011922	XP_006649443.1 pentatricopeptide repeat-containing protein At3g23020
TCONS_00028557	0.227037	0.35968	0.165876	BGIOSGA030302	XP_006665031.1 CCR4-NOT transcription complex subunit 10
TCONS_00015495	0.227051	-0.0318318	-1.51794	BGIOSGA009376	XP_006664812.1 sedoheptulose-1,7-bisphosphatase, chloroplastic

TCONS_00030269	0.227111	0.543789	0.229253	BGIOSGA026696	XP_003578332.1uncharacterized protein LOC100825556
TCONS_00003568	0.227204	0.880217	0.49909	BGIOSGA003530	XP_015699158.1 cullin-1-like
TCONS_00008230	0.227277	-0.149393	-0.481772	BGIOSGA014948	XP_006648773.1 CMP-sialic acid transporter 5
TCONS_00025279	0.227551	-0.248164	-0.0476172	BGIOSGA027691	XP_004957935.1transcription factor ILR3 isoform X1
TCONS_00003475	0.22765	0.910506	1.01616	BGIOSGA001747	XP_006644133.1 solute carrier family 25 member 44-like
TCONS_00033627	0.227879	0.352611	1.36081	BGIOSGA017796	XP_015698135.1 HIRA-interacting protein 3 isoform X1
TCONS_00017321	0.227911	-0.382747	0.421719	BGIOSGA019354	XP_002458783.2probable ubiquitin-like-specific protease 2B
TCONS_00006577	0.228053	0.160707	-0.221852	BGIOSGA023466	XP_015689676.1 anaphase-promoting complex subunit 8-like
TCONS_00007590	0.228055	0.726985	0.494377	BGIOSGA021944	XP_015689229.1 coatomer subunit beta'-2-like
TCONS_00020992	0.228284	0.497106	1.1496	BGIOSGA006921	XP_003563670.1uncharacterized protein At2g34160
TCONS_00014909	0.228367	0.403122	1.10034	BGIOSGA025594	XP_015691572.1 oxygen-dependent coproporphyrinogen-III oxidase, chloroplastic
TCONS_00028655	0.228667	1.27443	0.10499	BGIOSGA033440	XP_006661064.1 hexose carrier protein HEX6-like
TCONS_00007469	0.228674	0.638168	0.239974	BGIOSGA017560	XP_006646983.1 two-component response regulator ORR24
TCONS_00035430	0.228751	0.494825	-0.344098	BGIOSGA036840	XP_004973930.1sericin 1
TCONS_00034961	0.229242	0.0754575	0.16086	BGIOSGA016610	XP_004979380.1uncharacterized protein LOC101755097
TCONS_00026606	0.229492	-0.449991	-1.70643	BGIOSGA030667	XP_006659400.1PREDICTED: uncharacterized protein LOC102706119
TCONS_00017789	0.22959	-0.51992	-0.75666	BGIOSGA028709	XP_015692596.1PREDICTED: uncharacterized protein At4g15545
TCONS_00019503	0.229626	-3.35814	0.0920989	BGIOSGA019120	XP_006654545.1 probable inactive receptor kinase At5g58300
TCONS_00013661	0.229676	0.907054	0.74457	BGIOSGA015821	XP_015698966.1 protein transport protein Sec24-like At3g07100
TCONS_00017838	0.229758	1.02267	-0.664683	BGIOSGA016546	NP_001136644.1putative NAC domain transcription factor superfamily protein
TCONS_00008868	0.229957	0.672236	0.876439	BGIOSGA030895	XP_006665092.1 mitochondrial phosphate carrier protein 3, mitochondrial-like
TCONS_00005026	0.230063	1.12197	1.12834	BGIOSGA000192	XP_008656895.1tafazzin homolog
TCONS_00017487	0.230113	0.827006	-0.540945	BGIOSGA040239	YP_008815741.1RNA polymerase beta subunit (plastid)
TCONS_00021676	0.230146	0.72259	1.02552	BGIOSGA021843	XP_006655822.1 E4 SUMO-protein ligase PIAL2-like
TCONS_00035140	0.230172	0.764939	-0.837642	BGIOSGA006807	XP_012704051.1hydroquinone glucosyltransferase
TCONS_00007163	0.230214	2.03932	1.34155	BGIOSGA009350	XP_002453098.1homoserine kinase
TCONS_00035035	0.230272	-0.0590228	-0.278303	BGIOSGA029913	XP_024319049.1putative disease resistance RPP13-like protein 3 isoform X1
TCONS_00012484	0.230349	0.267941	0.0614742	BGIOSGA026495	XP_015690245.1 probable uridine nucleosidase 2 isoform X2
TCONS_00005905	0.230389	-1.38926	-0.551895	BGIOSGA019109	XP_006647203.1 dnaJ homolog subfamily B member 4-like
TCONS_00036474	0.230544	-0.456072	0.676409	BGIOSGA026562	XP_006664225.1 methionine aminopeptidase 2B-like
TCONS_00016556	0.230579	0.696754	0.233724	BGIOSGA019865	XP_002448464.1maltose excess protein 1-like, chloroplastic
TCONS_00023222	0.230659	-0.809238	-1.14499	BGIOSGA021415	XP_006658336.1PREDICTED: uncharacterized protein LOC102706211
TCONS_00006851	0.230666	-0.925772	0.531372	BGIOSGA009028	XP_006649035.2 probable alpha-mannosidase I MNS5 isoform X1
TCONS_00028990	0.230731	-0.508407	0.22798	BGIOSGA009044	XP_006660621.1PREDICTED: uncharacterized protein LOC102712385
TCONS_00032928	0.230804	0.412983	0.380444	BGIOSGA036957	XP_006664846.1 heterogeneous nuclear ribonucleoprotein R-like
TCONS_00018096	0.230823	0.252129	0.350721	BGIOSGA001216	XP_021303864.1jacalin-related lectin 3
TCONS_00004546	0.230942	1.53529	1.19095	BGIOSGA030700	XP_006644833.1PREDICTED: uncharacterized protein LOC102706146 isoform X2
TCONS_00036310	0.231317	-0.19101	-0.169875	BGIOSGA030881	XP_015698548.1PREDICTED: uncharacterized protein LOC102708669
TCONS_00027363	0.231465	0.666772	0.119518	BGIOSGA023471	XP_015695499.1 transaldolase
TCONS_00011813	0.231515	1.13805	-1.98979	BGIOSGA011824	XP_003558468.1cytochrome P450 94B3
TCONS_00025808	0.231773	-0.903157	-0.12576	BGIOSGA018974	XP_006659758.1 E3 ubiquitin-protein ligase CHIP
TCONS_00023428	0.231872	0.191355	-0.252316	BGIOSGA009490	XP_006657590.1 mRNA-decapping enzyme-like protein
TCONS_00018190	0.231938	1.34647	0.196916	BGIOSGA024924	XP_015692866.1 NADH dehydrogenase [ubiquinone] iron-sulfur protein 7, mitochondrial
TCONS_00033465	0.232088	1.31953	0.52746	BGIOSGA035264	XP_006663446.2 protein transport protein Sec24-like CEF
TCONS_00032531	0.232118	-0.592673	0.653253	BGIOSGA033244	NP_197399.1ARM repeat superfamily protein
TCONS_00004302	0.23215	-0.283009	0.133144	BGIOSGA019255	XP_015699175.1 brassinosteroid LRR receptor kinase
TCONS_00035789	0.232277	-0.132064	0.189452	BGIOSGA037192	XP_015698526.1 FAS1 domain-containing protein SELMODRAFT_448915-like
TCONS_00035785	0.232474	0.165705	0.490808	BGIOSGA037188	XP_004960737.1adenylylsulfatase HINT3
TCONS_00009090	0.232592	-0.704521	0.351451	BGIOSGA016783	XP_006648170.2 probable cytosolic oligopeptidase A
TCONS_00034125	0.232811	1.76636	0.451252	BGIOSGA010930	XP_006662651.1 ubiquitin-activating enzyme E1 2
TCONS_00020563	0.232843	0.544161	0.920005	BGIOSGA029004	XP_006656908.2 transmembrane protein 245
TCONS_00004738	0.232992	0.847535	0.935892	BGIOSGA000477	XP_006645029.1 probable anion transporter 3, chloroplastic
TCONS_00028540	0.233031	0.0337616	0.17077	BGIOSGA016174	XP_006661028.1 kinesin-like protein BC2 isoform X2

transcriptome

TCONS_00003670	0.233245	0.656118	0.914157	BGIOSGA017752	XP_004951295.1ribosome production factor 2 homolog
TCONS_00010956	0.233377	0.250322	0.66351	BGIOSGA029308	XP_015690897.1 SRSF protein kinase 2-like
TCONS_00023217	0.233427	1.61836	0.934819	BGIOSGA020583	XP_015695296.1 pyruvate kinase isozyme A, chloroplastic
TCONS_00029939	0.233436	0.130169	-0.497892	BGIOSGA005088	XP_014758816.1uncharacterized protein LOC100844622
TCONS_00024263	0.233519	-3.67796	-1.39862	BGIOSGA020554	XP_006658910.1PREDICTED: uncharacterized protein LOC102714663, partial
TCONS_00001140	0.233565	0.958418	1.00897	BGIOSGA021527	XP_015689015.1 pentatricopeptide repeat-containing protein At1g10910, chloroplastic
TCONS_00018418	0.233624	1.2073	0.797877	BGIOSGA020249	XP_002440366.1RGG repeats nuclear RNA binding protein A
TCONS_00013181	0.233667	0.0933739	0.56348	BGIOSGA009803	XP_006651826.1 transcriptional adapter ADA2
TCONS_00029075	0.233939	-0.127696	0.224164	BGIOSGA011595	XP_004967752.1putative protease Do-like 14 isoform X1
TCONS_00007323	0.233971	-0.302088	0.292098	BGIOSGA023696	XP_006646868.1 protein PHR1-LIKE 1-like
TCONS_00037609	0.23403	0.460519	0.496141	BGIOSGA035752	XP_006664244.1 pentatricopeptide repeat-containing protein At1g74750-like
TCONS_00013597	0.234351	0.530293	-0.345779	BGIOSGA015750	XP_006652066.2 phosphoglycerate mutase-like protein 1
TCONS_00025511	0.23442	1.27114	-0.0164362	BGIOSGA036514	NP_001144833.2uncharacterized protein LOC100277918
TCONS_00004994	0.234483	0.785482	1.06905	BGIOSGA014021	XP_006646625.1 uncharacterized WD repeat-containing protein C17D11.16-like
TCONS_00023311	0.234734	0.834361	1.08603	BGIOSGA025354	XP_015694556.1PREDICTED: uncharacterized protein LOC102719882, partial
TCONS_00025677	0.234737	0.534273	0.926794	BGIOSGA023746	NP_001149561.1adenyl cyclase
TCONS_00024144	0.234766	-0.238051	0.153514	BGIOSGA026181	XP_015694737.1PREDICTED: uncharacterized protein LOC102720347
TCONS_00034411	0.234809	-1.5994	-2.02699	BGIOSGA025012	XP_003577776.1dirigent protein 21
TCONS_00009826	0.235004	-5.31573	-1.59271	BGIOSGA017809	XP_004984832.1probable glucuronosyltransferase Os03g0287800
TCONS_00005053	0.235062	0.148151	-0.0150802	BGIOSGA010119	XP_006645299.1 vesicle-associated protein 2-2-like
TCONS_00037017	0.235181	0.652359	0.18319	BGIOSGA015096	XP_003577844.1cysteine desulfurase 1, chloroplastic
TCONS_00007477	0.23533	-0.0735889	0.297182	BGIOSGA035426	XP_006648383.2 protein S-acyltransferase 24-like
TCONS_00012418	0.235331	-2.33832	0.437901	BGIOSGA009348	XP_015690747.1 sucrose synthase 1
TCONS_00030354	0.2354	0.10312	-0.488024	BGIOSGA029445	XP_006660846.1 proteasome subunit beta type-4-like
TCONS_00034790	0.235504	-0.625584	-0.604233	BGIOSGA026166	XP_015697653.1 DIMBOA UDP-glucosyltransferase BX8-like
TCONS_00015576	0.235578	-0.197777	-1.10784	BGIOSGA003049	XP_015691398.1 transcription factor bHLH35-like
TCONS_00007906	0.235756	0.1809	0.103543	BGIOSGA006571	XP_015688979.1PREDICTED: uncharacterized protein LOC102710561
TCONS_00035713	0.235841	-0.311655	0.136171	BGIOSGA037127	XP_015698177.1 tRNA pseudouridine synthase A
TCONS_00011790	0.236338	-0.661614	-0.806167	BGIOSGA007563	XP_006649633.1 GDP-mannose transporter GONST3 isoform X1
TCONS_00016149	0.236622	-0.376075	-2.41431	BGIOSGA030128	XP_006652446.1 phosphoglycolate phosphatase 1B, chloroplastic-like
TCONS_00007244	0.236712	-0.623091	-0.0586325	BGIOSGA007243	XP_003573378.1protein trichome birefringence-like 12 isoform X2
TCONS_00005970	0.236763	-0.100687	-0.324811	BGIOSGA008105	XP_006664825.1 fruit protein pKIWI502
TCONS_00018186	0.236891	-0.284674	0.393579	BGIOSGA028831	XP_008656482.1uncharacterized LOC103635877 isoform X1
TCONS_00023350	0.236914	-0.490341	0.518471	BGIOSGA036394	XP_015694828.1 nucleolar protein 58
TCONS_00030468	0.236957	-0.680489	-0.257121	BGIOSGA019449	XP_006660924.2 enoyl-CoA hydratase 2, peroxisomal-like
TCONS_00021949	0.237134	-0.652769	-0.912837	BGIOSGA003624	XP_006655954.2 chaperone protein dnaJ 16 isoform X1
TCONS_00020609	0.237185	-0.257142	-2.81992	BGIOSGA002943	NP_001148747.1carboxyl-terminal-processing protease
TCONS_00036992	0.237213	0.398163	0.376105	BGIOSGA010119	XP_006664489.1 U-box domain-containing protein 44-like
TCONS_00016884	0.237253	0.174089	0.229141	BGIOSGA014080	XP_003561788.1uncharacterized protein LOC100834768
TCONS_00005711	0.237399	0.188102	-0.534374	BGIOSGA009352	XP_015689247.1 monosaccharide-sensing protein 2-like
TCONS_00016491	0.237405	-0.0815216	0.220894	BGIOSGA014478	XP_002448399.1protein MULTIPLE CHLOROPLAST DIVISION SITE 1
TCONS_00014939	0.237408	0.539332	0.721321	BGIOSGA008003	XP_006652825.1 APO protein 1, chloroplastic
TCONS_00022309	0.237435	0.635223	0.323809	BGIOSGA021173	XP_006656123.1 HEAT repeat-containing protein 5B isoform X2
TCONS_00014878	0.237578	0.187336	-0.441694	BGIOSGA017056	XP_015692138.1 thylakoid lumenal 29 kDa protein, chloroplastic
TCONS_00012247	0.237939	0.722423	0.303533	BGIOSGA026376	XP_006650064.1 60S ribosomal protein L22-2
TCONS_00033173	0.237942	1.43195	0.622822	BGIOSGA034500	XP_006662826.1 pyruvate kinase 1, cytosolic-like
TCONS_00023915	0.238207	0.172106	0.677367	BGIOSGA011986	XP_006657838.1 protein FAR1-RELATED SEQUENCE 5-like isoform X1
TCONS_00035518	0.23827	0.809456	0.53673	BGIOSGA036683	XP_004975322.2F-box protein At2g02240
TCONS_00035281	0.238337	1.74782	0.802421	BGIOSGA022793	XP_006663078.1 sodium/hydrogen exchanger 2-like
TCONS_00004684	0.238375	1.68752	0.271197	BGIOSGA022005	XP_006644974.1 protein GPR107-like
TCONS_00014581	0.238762	0.499209	0.00916734	BGIOSGA016745	XP_015692228.1PREDICTED: uncharacterized protein LOC102701164 isoform X1
TCONS_00002795	0.238808	-0.0927786	0.404164	BGIOSGA021572	XP_006643737.2 probable zinc metalloprotease EGY2, chloroplastic isoform X1
TCONS_00010976	0.239019	-0.558338	-0.281596	BGIOSGA013579	XP_015690483.1 cytochrome b5 isoform A
TCONS_00014707	0.239175	-0.338575	0.559683	BGIOSGA019751	XP_021318866.1dnaJ protein homolog 2 isoform X1

transcriptome

TCONS_00012771	0.239264	-0.186216	1.47452	BGIOSGA010193	XP_006651617.2 CBL-interacting protein kinase 7
TCONS_00000905	0.239856	0.1473	0.0161149	BGIOSGA003494	XP_006644159.1 probable phosphoinositide phosphatase SAC9
TCONS_00032902	0.239937	-0.631163	1.01206	BGIOSGA010845	XP_006663783.1 alpha-L-arabinofuranosidase 1-like
TCONS_00007463	0.239979	0.457869	0.776002	BGIOSGA009314	XP_006646977.1 ubiquitin carboxyl-terminal hydrolase 2-like
TCONS_00024416	0.240451	0.880313	-0.148633	BGIOSGA003356	XP_004958813.1probable serine/threonine-protein kinase PBL7
TCONS_00023566	0.240638	0.288697	0.201546	BGIOSGA038908	XP_024312958.1histone-lysine N-methyltransferase family member SUVH2
TCONS_00020397	0.240695	1.01742	0.599582	BGIOSGA008486	XP_006655940.1 beta-1,3-galactosyltransferase 15
TCONS_00006028	0.240871	-0.720413	-0.424897	BGIOSGA008180	XP_015688588.1 phosphatidylcholine transfer protein-like
TCONS_00018172	0.240952	0.0179357	-0.370176	BGIOSGA031102	XP_006654678.2 anthocyanidin 5,3-O-glucosyltransferase-like
TCONS_00014180	0.240965	-0.0246394	-0.273856	BGIOSGA008338	XP_015691440.1 chaperone protein ClpD2, chloroplastic
TCONS_00025059	0.241149	-0.584457	-1.70572	BGIOSGA033261	XP_006658538.2 5-pentadecatrienyl resorcinol O-methyltransferase-like
TCONS_00029029	0.241191	0.591957	0.257988	BGIOSGA030782	XP_006661248.1 protein SPA, chloroplastic
TCONS_00008368	0.241359	1.26619	1.09683	BGIOSGA014778	XP_003575344.1muscle M-line assembly protein unc-89
TCONS_00010422	0.241623	-0.122436	0.0688711	BGIOSGA015602	XP_003562339.1uncharacterized protein LOC100821889
TCONS_00006448	0.241625	0.195984	1.13806	BGIOSGA016723	XP_006647508.1 trafficking protein particle complex subunit 2-like
TCONS_00007281	0.241696	-0.127205	0.302828	BGIOSGA007202	XP_021314708.1uncharacterized protein LOC8055087 isoform X1
TCONS_00003251	0.241987	0.953746	0.282074	BGIOSGA001965	XP_015688280.1 zinc finger CCCH domain-containing protein 4
TCONS_00000467	0.242191	-0.465476	0.420982	BGIOSGA003041	XP_006643934.1 U11/U12 small nuclear ribonucleoprotein 59 kDa protein
TCONS_00007398	0.242207	2.25092	1.99712	BGIOSGA030447	XP_021319296.1uncharacterized protein LOC8064466
TCONS_00019762	0.242262	0.744835	0.464	BGIOSGA013444	XP_006654761.1 dynamin-related protein 5A
TCONS_00010074	0.242263	-0.880222	0.419498	BGIOSGA012619	XP_006650084.2 SAC3 family protein C
TCONS_00014321	0.242377	-0.464418	-0.0694154	BGIOSGA016485	XP_006652331.1 sm-like protein LSM1B
TCONS_00023882	0.24239	-0.45364	-0.375343	BGIOSGA025913	XP_006657814.1 probable V-type proton ATPase subunit H
TCONS_00024626	0.242711	1.03675	1.32957	#N/A	#N/A
TCONS_00005401	0.242942	0.437071	0.409075	BGIOSGA007511	XP_015689514.1 nuclear pore complex protein NUP160
TCONS_00008651	0.242954	-0.0740095	-1.6436	BGIOSGA030195	XP_006647757.2 DNA-binding protein SMUBP-2-like
TCONS_00013923	0.243738	0.00626117	0.420892	BGIOSGA016100	XP_010239753.1protein TRANSPARENT TESTA 9 isoform X1
TCONS_00022853	0.243801	-0.0508152	0.410887	BGIOSGA008415	NP_001168499.1uncharacterized LOC100275318
TCONS_00010910	0.243863	0.826661	0.662415	BGIOSGA013523	XP_015690525.1 telomerase Cajal body protein 1
TCONS_00017240	0.243866	1.60128	1.48641	BGIOSGA002892	XP_006654112.1 heat shock 70 kDa protein 15-like
TCONS_00010059	0.24396	0.956737	0.0664634	BGIOSGA019712	XP_015690196.1 serine/arginine-rich-splicing factor SR34-like isoform X1
TCONS_00000765	0.244192	0.126195	0.640494	BGIOSGA003462	XP_006644118.1 dual specificity protein phosphatase PHS1-like
TCONS_00006113	0.244567	0.647389	0.392083	BGIOSGA008269	XP_015689097.1PREDICTED: uncharacterized protein LOC107303603
TCONS_00010507	0.244712	-0.220715	-0.158792	BGIOSGA013117	XP_006650276.1 protein tas
TCONS_00004071	0.244815	-0.497331	0.360909	#N/A	#N/A
TCONS_00022180	0.244937	0.946484	0.434235	BGIOSGA001783	XP_006656939.2 protein tesmin/TSO1-like CXC 7 isoform X2
TCONS_00004363	0.245278	1.19191	0.593906	BGIOSGA013704	XP_015692745.1 probable glutamate carboxypeptidase 2
TCONS_00021252	0.245468	0.00856641	0.447033	BGIOSGA023478	XP_006656388.1 uncharacterized vacuolar membrane protein YML018C
TCONS_00010554	0.245569	0.00921184	-0.620617	BGIOSGA022429	XP_015691147.1 protein DA1-related 1-like
TCONS_00011364	0.245578	2.69382	2.12129	BGIOSGA013978	XP_015691126.1 pentatricopeptide repeat-containing protein At5g46580, chloroplastic
TCONS_00002210	0.246057	-1.18577	1.29126	BGIOSGA004844	XP_008674794.1uncharacterized protein LOC103650976
TCONS_00017616	0.246097	1.01112	0.803337	BGIOSGA013496	XP_015692561.1 pentatricopeptide repeat-containing protein At3g62470, mitochondrial-like
TCONS_00011219	0.246126	-1.24152	0.087024	BGIOSGA024257	XP_015690543.1 amino acid transporter ANT1-like
TCONS_00003002	0.246127	0.0271352	-0.540473	BGIOSGA002421	XP_006643865.1 transcription factor MYB1R1-like
TCONS_00022540	0.246185	0.470148	0.937338	BGIOSGA000070	XP_006656235.1 E3 ubiquitin-protein ligase RGLG2-like
TCONS_00026247	0.246421	-0.326444	-0.385796	BGIOSGA028329	XP_006659266.1 lactation elevated protein 1-like
TCONS_00017594	0.246924	0.296484	0.80007	BGIOSGA025813	XP_006655213.1 sorting nexin 2B
TCONS_00012611	0.247206	1.32579	0.00853645	BGIOSGA010364	XP_006650274.1 secretory carrier-associated membrane protein 4
TCONS_00005197	0.247245	0.700732	0.57203	BGIOSGA000009	XP_025015759.1 vacuolar fusion protein MON1 homolog
TCONS_00029345	0.247261	-2.86447	-0.243263	BGIOSGA031094	XP_010069234.1PREDICTED: uncharacterized protein LOC104456191
TCONS_00009237	0.24735	-1.47007	-0.660836	BGIOSGA011767	XP_006649325.1 phosphatidylinositol-glycan biosynthesis class F protein
TCONS_00018723	0.247512	-0.890385	-1.01205	BGIOSGA016635	XP_010231650.1acyl transferase 7

transcriptome

TCONS_00024788	0.247695	-0.0882944	0.796877	BGIOSGA024578	XP_015694572.1 F-box/FBD/LRR-repeat protein At4g00160-like
TCONS_00034103	0.248057	0.590457	0.851995	BGIOSGA005292	XP_006663135.1 mitochondrial uncoupling protein 1-like
TCONS_00032677	0.248132	0.275124	0.876315	BGIOSGA031431	XP_006662043.1 putative pre-16S rRNA nuclease
TCONS_00021330	0.248134	-0.529601	-0.91573	BGIOSGA007152	XP_015693699.1 L-arabinokinase-like
TCONS_00035788	0.248178	0.581032	0.339526	BGIOSGA037191	XP_006663920.1 MAG2-interacting protein 2
TCONS_00006834	0.248355	0.869392	0.313754	BGIOSGA016577	XP_006647856.1 probable RNA-dependent RNA polymerase 1
TCONS_00010066	0.248422	0.357381	0.829445	BGIOSGA012609	XP_006650074.1 nuclear cap-binding protein subunit 1
TCONS_00000047	0.248428	1.03467	0.425295	BGIOSGA000169	XP_015699329.1PREDICTED: uncharacterized protein LOC102716464 isoform X2
TCONS_00035187	0.248491	0.355767	0.0339848	BGIOSGA007472	XP_006663049.1 proteasome subunit alpha type-5
TCONS_00001627	0.248504	0.124112	-0.246881	BGIOSGA004261	XP_015689667.1PREDICTED: uncharacterized protein LOC102706990, partial
TCONS_00004714	0.248739	-0.694445	-1.87454	BGIOSGA009301	XP_002458750.1probable protein S-acyltransferase 16 isoform X1
TCONS_00003898	0.248902	0.405653	-0.423093	BGIOSGA016764	XP_004969036.1cytokinin riboside 5'-monophosphate phosphoribohydrolase LOG isoform X1
TCONS_00036641	0.24898	-0.0763035	0.65077	BGIOSGA034534	XP_006664844.1 50S ribosomal protein L24-like
TCONS_00011294	0.248985	-1.13853	-0.852335	BGIOSGA016430	XP_006650867.1 nicotinate phosphoribosyltransferase 2-like
TCONS_00009038	0.249268	0.38159	0.606081	BGIOSGA009787	XP_015689570.1 receptor-like serine/threonine-protein kinase ALE2 isoform X1
TCONS_00000633	0.24938	0.726345	0.670255	BGIOSGA020576	XP_006644035.1 26S proteasome non-ATPase regulatory subunit 14 homolog
TCONS_00030536	0.249414	-0.228023	0.321397	BGIOSGA009729	XP_015696561.1PREDICTED: uncharacterized protein LOC102701201
TCONS_00031557	0.249696	-2.93315	0.554672	BGIOSGA024780	XP_006662014.1 calcium-dependent protein kinase isoform 1
TCONS_00032079	0.249872	-0.042112	-0.708305	BGIOSGA026022	XP_015697462.1 scarecrow-like protein 21
TCONS_00020894	0.249994	0.00193097	-0.556714	BGIOSGA001895	XP_015693615.1 probable phospholipid-transporting ATPase 4
TCONS_00004685	0.250351	0.506662	1.53103	BGIOSGA012354	XP_006646462.2 ankyrin repeat-containing protein At3g12360-like
TCONS_00023000	0.250535	1.86021	0.874528	BGIOSGA025031	XP_002461325.1probable inactive purple acid phosphatase 2
TCONS_00022008	0.250788	1.50808	-0.14267	BGIOSGA021510	XP_004965173.1ribonucleoside-diphosphate reductase small chain
TCONS_00022912	0.250896	0.656469	0.535132	BGIOSGA014027	XP_006656492.1 probable magnesium transporter NIPA6
TCONS_00014261	0.250984	0.248366	-0.179037	BGIOSGA016418	XP_015691337.1PREDICTED: uncharacterized protein LOC102709267
TCONS_00023006	0.2512	0.0113665	-0.947373	#N/A	#N/A
TCONS_00016960	0.251226	-0.0655433	-0.0912859	BGIOSGA031780	XP_006653946.1 ras-related protein RABA2a-like
TCONS_00033663	0.25138	1.16665	0.812555	BGIOSGA033803	XP_006662980.1 actin-related protein 2/3 complex subunit 5A-like
TCONS_00004544	0.25157	-0.330347	0.752166	BGIOSGA000663	XP_006644833.1PREDICTED: uncharacterized protein LOC102706146 isoform X2
TCONS_00035091	0.25189	0.70899	1.20152	BGIOSGA016888	XP_006646847.1 sec-independent protein translocase protein TATB, chloroplastic-like
TCONS_00007187	0.252016	-0.321351	0.904429	BGIOSGA007311	XP_015688971.1 kinesin-like protein KIF18A
TCONS_00024369	0.252273	1.49813	0.0237989	BGIOSGA012543	XP_004959898.1ricin B-like lectin R40G2
TCONS_00026565	0.252607	-0.570833	-0.0640313	BGIOSGA030601	XP_006659382.1PREDICTED: uncharacterized protein LOC102700820
TCONS_00034496	0.252609	0.411078	0.649801	BGIOSGA017645	XP_004978786.1F-box/LRR-repeat protein At3g26922
TCONS_00002012	0.25271	0.556889	-0.469362	BGIOSGA033307	XP_006644869.1 UDP-glycosyltransferase 87A1-like
TCONS_00008954	0.252807	0.37534	-0.0131338	BGIOSGA010446	XP_003570284.1probable serine incorporator
TCONS_00005007	0.252848	-2.23744	-0.943637	BGIOSGA000214	XP_004971138.1probable glucuronosyltransferase Os01g0926700
TCONS_00021265	0.252887	1.27359	0.85481	BGIOSGA023591	XP_015693827.1 lipid transfer-like protein VAS
TCONS_00012933	0.253135	-0.203221	0.4937	BGIOSGA027572	XP_006643748.1 glucose-induced degradation protein 4 homolog isoform X1
TCONS_00013285	0.25319	-0.739427	-0.132141	BGIOSGA006186	XP_023156179.1uncharacterized LOC103644136 isoform X3
TCONS_00007589	0.253196	0.178025	0.123796	BGIOSGA021944	XP_015689229.1 coatomer subunit beta'-2-like
TCONS_00026557	0.253452	0.568094	0.483236	BGIOSGA007797	XP_006660108.1 aminopeptidase M1-B
TCONS_00011547	0.25351	-0.0803369	0.36125	BGIOSGA033086	XP_006649385.1 cysteine--tRNA ligase, chloroplastic/mitochondrial-like
TCONS_00011509	0.253532	0.816093	0.637133	BGIOSGA011518	XP_006649333.1 poly(A) polymerase I-like isoform X1
TCONS_00006746	0.25358	0.195469	0.432174	BGIOSGA008914	XP_006648990.1 trafficking protein particle complex subunit 11
TCONS_00008911	0.254027	0.84779	0.481584	BGIOSGA020721	XP_006647995.1 replication protein A 70 kDa DNA-binding subunit A
TCONS_00034211	0.254325	0.353419	-0.0103181	BGIOSGA012537	XP_006664296.1 probable arabinosyltransferase ARAD1
TCONS_00028797	0.254414	0.906687	0.428707	BGIOSGA039316	XP_021309867.1cysteine desulfurase, mitochondrial
TCONS_00000543	0.254703	-0.204522	0.424845	BGIOSGA003125	XP_008672950.160S ribosomal protein L18A isoform X1
TCONS_00024114	0.254781	0.146909	0.708668	BGIOSGA026147	XP_006657962.2 protein STRUBBELIG-RECEPTOR FAMILY 7-like
TCONS_00028071	0.254813	0.491762	0.440754	BGIOSGA029760	XP_006659444.1 Golgi SNAP receptor complex member 1-1
TCONS_00002870	0.254958	#NA	-3.0995	BGIOSGA020359	XP_015693691.1 fasciclin-like arabinogalactan protein 11

TCONS_00033235	0.254959	0.980022	0.809023	BGIOSGA029210	XP_006662809.1PREDICTED: uncharacterized protein LOC102717289
TCONS_00009819	0.25516	2.02534	1.31083	BGIOSGA029361	XP_006649890.1 L-ascorbate peroxidase 1, cytosolic
TCONS_00022847	0.255264	1.05146	0.749758	BGIOSGA030521	XP_006656434.1 eukaryotic translation initiation factor 5-like
TCONS_00016827	0.25537	0.41024	1.27678	BGIOSGA021439	XP_006652988.2 phosphatidylinositol 4-kinase gamma 6-like
TCONS_00002817	0.255667	0.869268	0.899954	BGIOSGA002402	XP_006655910.1 factor of DNA methylation 1-like
TCONS_00016986	0.255821	0.963256	0.280817	BGIOSGA028730	NP_001132564.1NAD(P)-binding Rossmann-fold superfamily protein
TCONS_00010142	0.255822	0.132018	-0.0710836	BGIOSGA012700	XP_006650117.1 SWR1 complex subunit 6
TCONS_00012161	0.256789	0.17461	0.25322	BGIOSGA000624	XP_006649995.1 transcription factor HBP-1b(c38)-like
TCONS_00037368	0.256923	-0.71119	0.968737	BGIOSGA036001	XP_006664104.2 30S ribosomal protein S6
TCONS_00025586	0.256987	-0.411568	0.916488	BGIOSGA012719	XP_010234983.1meiosis regulator and mRNA stability factor 1
TCONS_00036879	0.256987	0.765971	-0.0478884	BGIOSGA035954	XP_006663912.1 diacylglycerol kinase 2
TCONS_00004440	0.257016	0.390595	0.464356	BGIOSGA037315	XP_006644742.1 aspartate aminotransferase, cytoplasmic
TCONS_00027158	0.257023	0.440443	-0.459445	BGIOSGA029203	XP_015695961.1 malate dehydrogenase [NADP] 1, chloroplastic
TCONS_00029707	0.257049	-1.14499	0.0559788	BGIOSGA030113	XP_004977388.1probable WRKY transcription factor 62 isoform X1
TCONS_00004700	0.257071	0.203767	0.526139	BGIOSGA019990	XP_006644985.1 60S ribosomal protein L36-3-like
TCONS_00020155	0.257706	0.0331778	0.143588	BGIOSGA022352	XP_010547553.1 succinate dehydrogenase assembly factor 4, mitochondrial
TCONS_00024837	0.257741	-0.0911059	0.811029	BGIOSGA013870	XP_015694587.1PREDICTED: uncharacterized protein LOC102716431
TCONS_00005133	0.257854	0.21295	0.368529	BGIOSGA020060	XP_015698512.1 probable LRR receptor-like serine/threonine-protein kinase At1g06840
TCONS_00031629	0.258116	1.69165	0.688428	BGIOSGA029853	XP_004983649.1homeobox-leucine zipper protein HOX1 isoform X2
TCONS_00009548	0.25812	-0.201726	-0.687522	BGIOSGA001035	XP_015689813.1PREDICTED: uncharacterized protein LOC107303742
TCONS_00031441	0.258234	-0.379372	-0.27743	BGIOSGA033256	XP_024316224.1probable plastid-lipid-associated protein 10, chloroplastic isoform X2
TCONS_00006519	0.258505	-0.284375	0.225524	BGIOSGA016806	XP_003569955.1WD repeat-containing protein 44
TCONS_00011357	0.258685	0.397768	0.825348	BGIOSGA013970	XP_006650921.1 RNA-binding protein 34
TCONS_00003725	0.258715	-0.614824	-0.530821	BGIOSGA031326	XP_006644294.1 calcium permeable stress-gated cation channel 1
TCONS_00000732	0.258743	#NA	-3.3313	BGIOSGA003333	XP_015688232.1 rab9 effector protein with kelch motifs-like isoform X3
TCONS_00009765	0.258953	1.00491	0.979969	BGIOSGA007134	NP_001136765.1putative RING zinc finger domain superfamily protein
TCONS_00013111	0.259063	0.0948066	-0.205247	BGIOSGA009874	XP_006650536.1 thioredoxin-like protein AAED1, chloroplastic
TCONS_00022244	0.259081	-0.371344	0.918203	BGIOSGA021252	XP_006656083.1 FKBP12-interacting protein of 37 kDa
TCONS_00037432	0.259201	-0.189362	0.130808	BGIOSGA035932	XP_006664685.1 GDP-Man:Man(3)GlcNAc(2)-PP-Dol alpha-1,2-mannosyltransferase
TCONS_00032723	0.25925	0.434989	0.767025	BGIOSGA031384	XP_015697316.1PREDICTED: uncharacterized protein LOC102700656
TCONS_00009220	0.259375	1.85549	0.230443	BGIOSGA018003	XP_004985910.1uncharacterized protein LOC101774065
TCONS_00019133	0.25958	-0.0194462	0.606621	BGIOSGA001808	XP_010231510.160S ribosomal protein L29-1
TCONS_00009767	0.259627	0.632384	0.0116978	BGIOSGA012319	XP_015691276.1 E3 ubiquitin-protein ligase SDIR1
TCONS_00025376	0.259639	-0.0928048	0.454753	BGIOSGA024056	XP_006657864.2 zinc-metallopeptidase, peroxisomal-like isoform X1
TCONS_00035626	0.259988	-0.262547	0.928119	BGIOSGA029659	XP_015698225.1 F-box protein At3g07870-like
TCONS_00029789	0.260162	-0.185835	0.618253	BGIOSGA015869	XP_015696687.1 polycomb group protein EMBRYONIC FLOWER 2
TCONS_00016658	0.260239	1.47253	1.26221	BGIOSGA014303	XP_006652860.1 ABC transporter I family member 1
TCONS_00032519	0.260455	0.321443	0.903943	BGIOSGA031596	NP_001140760.1putative DEAD-box ATP-dependent RNA helicase family protein
TCONS_00024568	0.260785	1.06734	0.30978	BGIOSGA009701	XP_015695273.1 WPP domain-associated protein isoform X2
TCONS_00016236	0.260827	0.999978	0.744004	BGIOSGA029294	XP_006652503.1PREDICTED: uncharacterized protein LOC102702840
TCONS_00017319	0.260898	0.61684	0.619153	BGIOSGA007850	XP_006643887.1 60S ribosomal protein L11
TCONS_00001185	0.261082	1.10694	1.19475	BGIOSGA000507	XP_006646022.1 ATP-dependent zinc metalloprotease FTSH 4, mitochondrial isoform X1
TCONS_00021505	0.261262	-0.0495931	-0.844689	BGIOSGA022048	XP_010228086.1uncharacterized protein LOC100841492 isoform X2
TCONS_00000652	0.26131	0.29071	0.465828	BGIOSGA011691	XP_006644044.2 NEDD8-activating enzyme E1 catalytic subunit
TCONS_00012770	0.261631	0.2989	0.869001	BGIOSGA016280	XP_004982374.1protein OSB2, chloroplastic
TCONS_00024636	0.261757	-0.148464	-0.401656	BGIOSGA009664	XP_006657485.1 zinc finger A20 and AN1 domain-containing stress-associated protein 9
TCONS_00002250	0.26176	-0.706094	0.409022	BGIOSGA026713	XP_006646516.1 embryogenesis-associated protein EMB8-like
TCONS_00026035	0.261803	-0.122915	-0.910159	BGIOSGA024203	XP_006659185.1 glucose-6-phosphate/phosphate translocator 2, chloroplastic-like
TCONS_00020856	0.261997	1.24975	1.1359	BGIOSGA033516	XP_015693607.1 50S ribosomal protein L1
TCONS_00015212	0.262213	0.110774	0.697246	BGIOSGA020180	XP_006653065.1 tubby-like F-box protein 7
TCONS_00015940	0.262237	0.954814	1.4777	BGIOSGA015043	XP_006653431.2 protein TIC 40, chloroplastic
TCONS_00021989	0.262438	1.04734	0.59527	BGIOSGA021526	XP_015693533.1 NF-X1-type zinc finger protein NFXL1

TCONS_00003661	0.262489	0.693478	0.190122	BGIOSGA012114	XP_006644222.1 26S proteasome non-ATPase regulatory subunit 13 homolog B-like
TCONS_00012246	0.262496	0.878897	1.02203	BGIOSGA032547	XP_006650062.1 auxin-repressed 12.5 kDa protein
TCONS_00035877	0.262565	0.908222	0.338962	BGIOSGA027851	XP_006665025.1 protein mago nashi homolog
TCONS_00032259	0.262646	0.747278	0.762066	BGIOSGA028966	XP_003573947.1serine/threonine-protein kinase EDR1
TCONS_00018290	0.262703	0.221402	0.0252554	BGIOSGA020343	XP_006655581.2 ninja-family protein Os05g0558800
TCONS_00009261	0.262771	-0.155302	0.619264	BGIOSGA021684	XP_015698128.1PREDICTED: uncharacterized protein LOC102717848
TCONS_00014922	0.262802	-2.33622	-1.93648	BGIOSGA007400	XP_006652808.1 polygalacturonase QRT3-like
TCONS_00011629	0.262805	0.673734	0.719053	BGIOSGA006870	XP_006649459.1 serine/threonine-protein phosphatase PP2A-4 catalytic subunit-like
TCONS_00006867	0.262829	0.471099	0.785921	BGIOSGA006429	XP_006647884.1 probable galacturonosyltransferase 9
TCONS_00001324	0.263248	-1.97275	-3.87039	BGIOSGA015195	XP_006644350.1 amino-acid permease BAT1 homolog isoform X1
TCONS_00001991	0.263294	-2.33684	-1.45227	BGIOSGA004627	XP_015691953.1 DNA-(apurinic or apyrimidinic site) lyase, chloroplastic-like isoform X1
TCONS_00022783	0.263337	-1.11635	-0.171799	BGIOSGA033706	XP_020403152.1DNAJ heat shock N-terminal domain-containing protein isoform X2
TCONS_00033704	0.263374	-0.589128	0.0668411	BGIOSGA035475	NP_001148055.1carotenoid isomerase 2
TCONS_00004161	0.26346	0.532135	0.882219	BGIOSGA014371	XP_006646194.1 trihelix transcription factor ASIL2-like
TCONS_00009087	0.263897	-0.139102	-0.0792108	BGIOSGA005316	XP_015689259.1 synaptotagmin-2
TCONS_00012355	0.263914	-0.32858	0.996689	BGIOSGA037352	NP_001143396.1uncharacterized protein LOC100276037
TCONS_00025761	0.263941	-4.57654	-1.36595	BGIOSGA012379	XP_015694979.1 probable glucuronosyltransferase Os07g0694400 isoform X1
TCONS_00019147	0.263979	0.192613	1.18164	BGIOSGA024448	XP_006654303.1 S-adenosylmethionine carrier 1, chloroplastic/mitochondrial-like
TCONS_00024727	0.264077	-0.184397	1.00627	BGIOSGA025344	XP_015694553.1 squalene synthase-like
TCONS_00016419	0.264111	-0.0330167	-0.650196	BGIOSGA014593	XP_014758891.1O-fucosyltransferase 6
TCONS_00035818	0.264136	-0.488417	0.875904	BGIOSGA037218	XP_022683950.1mediator of RNA polymerase II transcription subunit 15a isoform X1
TCONS_00036670	0.264239	-0.462581	0.464212	BGIOSGA032657	XP_006664333.1 long chain acyl-CoA synthetase 6, peroxisomal-like
TCONS_00018198	0.264583	-0.490459	0.907128	BGIOSGA033891	XP_004976560.1putative FBD-associated F-box protein At1g61330
TCONS_00011443	0.264605	0.145146	0.0714771	BGIOSGA011581	XP_006649259.1 probable RNA-binding protein EIF1AD
TCONS_00030888	0.264615	0.00756943	0.170794	BGIOSGA032691	XP_006661689.1 zinc finger RNA-binding protein-like
TCONS_00024494	0.264992	-0.0572418	0.414306	BGIOSGA024902	XP_015694854.1 plastid division protein PDV2
TCONS_00036561	0.265084	-0.167958	-1.62312	BGIOSGA000583	XP_006664281.1 probable WRKY transcription factor 54
TCONS_00007689	0.265126	-1.08814	-2.50156	BGIOSGA026857	XP_006657994.1 malate dehydrogenase, chloroplastic-like
TCONS_00011392	0.265203	-0.373019	0.746393	BGIOSGA011632	XP_006650940.1PREDICTED: uncharacterized protein LOC102702457
TCONS_00024045	0.265619	-3.6135	-0.724101	BGIOSGA029269	XP_006657923.1 PLASMODESMATA CALLOSE-BINDING PROTEIN 3-like
TCONS_00027638	0.266163	0.466014	0.905867	BGIOSGA027374	XP_024033861.1bifunctional dethiobiotin synthetase/7,8-diamino-pelargonic acid aminotransferase, mitochondrial isoform X2
TCONS_00012535	0.266471	-0.565202	0.222838	BGIOSGA005453	XP_006650244.1 probable serine incorporator
TCONS_00020413	0.26649	-1.92197	-0.644604	BGIOSGA002078	XP_006655950.2 rac-like GTP-binding protein 4 isoform X2
TCONS_00001201	0.266509	-1.18746	0.751062	BGIOSGA007369	XP_015689330.1 GPI mannosyltransferase 3
TCONS_00002909	0.266569	-0.370713	-0.247535	BGIOSGA018734	XP_015689829.1 thioredoxin H4-1
TCONS_00016895	0.266919	0.151733	0.120757	BGIOSGA014066	XP_006653044.1 K(+) efflux antiporter 2, chloroplastic
TCONS_00006467	0.266924	-1.16207	-0.675112	BGIOSGA008638	XP_003572720.1uncharacterized protein LOC100840025
TCONS_00024468	0.26694	-0.23845	0.143493	BGIOSGA006589	XP_006657406.1 casein kinase II subunit alpha
TCONS_00032614	0.266991	-2.4923	0.0001347	BGIOSGA000169	XP_004983824.1probable membrane-associated kinase regulator 6
TCONS_00033270	0.267101	-0.734257	-0.548051	BGIOSGA035578	XP_015698073.1 disease resistance protein RPM1-like
TCONS_00006692	0.267427	-0.0824737	0.638978	BGIOSGA008861	XP_004953560.1AF4/FMR2 family member 4
TCONS_00032129	0.267516	#NA	-2.88122	BGIOSGA011992	XP_015697244.1PREDICTED: uncharacterized protein LOC102704451
TCONS_00034304	0.267701	0.94141	0.253928	BGIOSGA034497	XP_006663207.1 DDT domain-containing protein PTM-like
TCONS_00009686	0.267777	-1.09317	-1.48934	BGIOSGA029548	XP_002489060.1IRK-interacting protein
TCONS_00024941	0.267991	-0.203673	0.13639	BGIOSGA024447	XP_006657623.1 UPF0235 protein At5g63440 isoform X1
TCONS_00001011	0.268096	-2.80536	-0.970952	BGIOSGA014892	XP_006644213.1 beta-glucosidase 1
TCONS_00029202	0.268164	0.418145	1.37083	BGIOSGA018917	XP_021308077.1ABC transporter G family member 11
TCONS_00003153	0.268472	0.482201	1.04407	BGIOSGA015927	XP_004967383.2glycosyltransferase-like KOBITO 1
TCONS_00025333	0.26851	-1.08809	-0.234962	BGIOSGA024095	XP_006657830.1 protein THYLAKOID FORMATION1, chloroplastic isoform X1
TCONS_00009595	0.268787	0.533734	-0.0681711	BGIOSGA012144	XP_015690704.1PREDICTED: uncharacterized protein LOC107303921
TCONS_00018446	0.268926	0.297749	0.593298	BGIOSGA002614	XP_006653929.2PREDICTED: uncharacterized protein LOC102706005 isoform X1
TCONS_00001825	0.26912	0.966909	0.37803	BGIOSGA017841	XP_006644708.1 probable sugar phosphate/phosphate translocator At1g06470
TCONS_00025893	0.269297	0.283512	1.54053	BGIOSGA027965	XP_004972891.1uncharacterized protein LOC101769116
TCONS_00014024	0.269328	-0.412049	-0.281173	BGIOSGA015296	XP_015691404.1 protein Rf1, mitochondrial-like

TCONS_00027964	0.269353	0.549112	-1.77128	BGIOSGA015508	XP_004979105.11-aminocyclopropane-1-carboxylate oxidase homolog 1
TCONS_00007288	0.269452	-0.568291	0.0347134	BGIOSGA007194	XP_021314502.1probable pyridoxal 5'-phosphate synthase subunit PDX2
TCONS_00023612	0.269624	-0.0180588	0.341829	BGIOSGA008835	XP_015694846.1PREDICTED: uncharacterized protein LOC102707884 isoform X1
TCONS_00031342	0.269699	0.591746	0.227861	BGIOSGA011692	NP_001150075.2lysM domain containing protein
TCONS_00023174	0.26976	0.352266	-0.856193	#N/A	#N/A
TCONS_00008340	0.269873	1.25895	0.549356	BGIOSGA006085	XP_006647479.2 tRNA (guanine(37)-N1)-methyltransferase
TCONS_00010969	0.270063	0.0500638	1.10476	BGIOSGA010720	XP_006650092.1 50S ribosomal protein L6, chloroplastic
TCONS_00035500	0.270204	0.311399	-0.646699	BGIOSGA001155	XP_006663173.1 mini zinc finger protein 1-like
TCONS_00011052	0.270226	-5.14064	-0.78911	BGIOSGA008209	XP_006650672.1PREDICTED: uncharacterized protein LOC102719188 isoform X2
TCONS_00034821	0.270406	-3.95629	-0.925565	BGIOSGA013434	XP_012703639.1rapid alkalinization factor 23
TCONS_00017128	0.270406	0.213638	0.659397	BGIOSGA019175	XP_006654044.1PREDICTED: uncharacterized protein LOC102716498
TCONS_00002844	0.270419	0.36005	0.554837	BGIOSGA001568	XP_003564576.1histone H2B.5
TCONS_00018189	0.270699	0.0392942	0.281051	BGIOSGA000900	XP_006655519.1 probable starch synthase 4, chloroplastic/amyloplastic
TCONS_00020463	0.270711	0.0265711	0.71078	BGIOSGA029238	XP_004965167.1uncharacterized protein LOC101778788
TCONS_00000920	0.270869	-1.20084	-1.90326	BGIOSGA010658	XP_004968732.3CASP-like protein 1E1
TCONS_00037774	0.271236	0.711803	0.541537	BGIOSGA017423	XP_006653088.1 dolichyl-diphosphooligosaccharide--protein glycosyltransferase subunit 1B
TCONS_00022944	0.271338	1.11021	-0.349626	BGIOSGA001315	XP_015694452.1 probable galacturonosyltransferase 3 isoform X2
TCONS_00011834	0.271385	-0.250042	-0.0886513	BGIOSGA011177	XP_006656251.2 maf-like protein DDB_G0281937
TCONS_00024424	0.271456	1.01851	0.594519	BGIOSGA026295	XP_006657384.1 BTB/POZ and MATH domain-containing protein 3-like
TCONS_00022027	0.271534	0.900005	0.258941	BGIOSGA010942	XP_006655987.1 asparagine synthetase [glutamine-hydrolyzing] 2
TCONS_00004093	0.271763	0.0991315	0.298004	BGIOSGA001095	XP_003569478.1zinc finger CCCH domain-containing protein 13
TCONS_00032532	0.271787	-0.214578	-0.173536	BGIOSGA014653	XP_006661932.1 vesicle-associated protein 1-2
TCONS_00013361	0.271914	0.328534	0.727488	BGIOSGA024675	XP_006650746.1 DDT domain-containing protein DDR4-like
TCONS_00002193	0.272089	0.70435	-0.231639	BGIOSGA017952	XP_006646490.2 pleckstrin homology domain-containing protein 1-like
TCONS_00008672	0.272231	0.410654	0.334477	BGIOSGA035563	XP_006647781.1 TBC1 domain family member 15
TCONS_00005300	0.272234	0.565449	0.735036	BGIOSGA022237	XP_006646801.1 peptidyl-prolyl cis-trans isomerase
TCONS_00011492	0.272288	-0.0800014	-1.16688	BGIOSGA011534	XP_006649321.1PREDICTED: uncharacterized protein LOC102703193
TCONS_00038004	0.272665	-0.667821	0.260363	BGIOSGA015018	XP_004975719.1COX assembly mitochondrial protein 2 homolog
TCONS_00028511	0.27297	1.14135	0.472695	BGIOSGA007638	XP_006660412.1 CAX-interacting protein 4
TCONS_00009155	0.272984	0.345151	1.38969	BGIOSGA011682	XP_006649246.1 DEAD-box ATP-dependent RNA helicase 50
TCONS_00000233	0.272995	0.618703	0.513672	BGIOSGA001370	XP_006645272.2 aspartate--tRNA ligase, chloroplastic/mitochondrial
TCONS_00012520	0.273136	0.197809	-1.37623	BGIOSGA010462	XP_015690524.1PREDICTED: uncharacterized protein ycf36 isoform X2
TCONS_00003551	0.273282	-0.663675	-1.77425	BGIOSGA001659	XP_006644169.2 acyltransferase-like protein At1g54570, chloroplastic
TCONS_00007143	0.273289	-0.275756	0.239433	BGIOSGA005324	XP_014755776.1putative F-box/LRR-repeat protein At5g02700
TCONS_00017170	0.273445	-0.791431	0.540581	BGIOSGA001731	XP_006654070.2 serine/threonine-protein phosphatase 4 regulatory subunit 3B-like isoform X2
TCONS_00002233	0.273522	0.263286	0.741329	BGIOSGA004869	XP_006646505.1PREDICTED: uncharacterized protein LOC102699925 isoform X2
TCONS_00001738	0.273589	-1.02375	-0.535395	BGIOSGA029007	XP_006646275.2 ribose-phosphate pyrophosphokinase 3, chloroplastic
TCONS_00001870	0.273769	-0.319619	0.388024	BGIOSGA004502	XP_002456390.1RHOMBOID-like protein 12, mitochondrial isoform X2
TCONS_00004810	0.273866	-0.0196336	0.716961	BGIOSGA020071	XP_006645093.1 deoxyguanosine kinase, mitochondrial-like
TCONS_00033971	0.27389	0.450746	-0.612278	BGIOSGA008933	XP_006663650.2 ADP,ATP carrier protein ER-ANT1 isoform X1
TCONS_00024760	0.274253	0.322987	0.160128	BGIOSGA024617	XP_004955805.1UPF0613 protein PB24D3.06c
TCONS_00021907	0.274308	0.843943	1.31298	BGIOSGA009046	XP_008648260.1DCD (Development and Cell Death) domain protein isoform X1
TCONS_00014890	0.274467	0.338729	0.959542	BGIOSGA017069	XP_015692218.1PREDICTED: uncharacterized protein LOC102708616
TCONS_00019628	0.274705	-4.44019	-2.04066	BGIOSGA030479	XP_006655485.2PREDICTED: uncharacterized protein LOC102699408
TCONS_00000677	0.274773	-0.165395	-0.426763	BGIOSGA036617	XP_015688196.1 transcription factor HBP-1b(c38) isoform X1
TCONS_00028528	0.274807	0.941743	0.634943	BGIOSGA030281	XP_006660419.1 DNA-directed RNA polymerases II, IV and V subunit 3-like
TCONS_00023927	0.274837	0.632295	0.979106	BGIOSGA002986	XP_004951320.1sodium/calcium exchanger NCL2
TCONS_00030229	0.275002	-1.15314	-0.71077	BGIOSGA037872	XP_004957141.2photosystem I reaction center subunit V, chloroplastic
TCONS_00006405	0.275047	0.167211	1.08587	BGIOSGA008567	XP_015688941.1 pentatricopeptide repeat-containing protein At3g04130, mitochondrial
TCONS_00003900	0.275204	-0.369054	0.774283	BGIOSGA032833	XP_006646054.1 pentatricopeptide repeat-containing protein At4g02750-like
TCONS_00025850	0.275594	0.636034	1.62538	BGIOSGA027917	XP_006659084.1 pre-mRNA-splicing factor SLU7

TCONS_00020258	0.275602	-2.08746	-0.423877	BGIOSGA026268	XP_002440417.1uncharacterized protein LOC8068642
TCONS_00022341	0.275614	-0.161478	-0.308431	BGIOSGA022885	XP_006657011.1 CMP-sialic acid transporter 1
TCONS_00023241	0.275663	1.31845	0.514569	BGIOSGA025282	XP_006657510.2 ATP-dependent DNA helicase 2 subunit KU70
TCONS_00002693	0.275869	0.283601	1.10632	BGIOSGA002527	XP_002455310.1U-box domain-containing protein 4
TCONS_00006797	0.27601	0.591791	0.702706	BGIOSGA008970	XP_006647835.1 eukaryotic translation initiation factor 5B
TCONS_00009721	0.276032	-0.402802	0.345191	BGIOSGA028721	NP_001130864.1harpin-induced protein
TCONS_00019661	0.276218	-1.24024	0.311564	BGIOSGA022722	XP_002441416.1anthocyanidin 5,3-O-glucosyltransferase
TCONS_00005011	0.276605	-0.0887222	0.42164	BGIOSGA012398	XP_006646639.1 aspartokinase 1, chloroplastic
TCONS_00030990	0.276684	0.68948	0.183091	BGIOSGA032796	XP_015697283.1PREDICTED: uncharacterized protein LOC107305138
TCONS_00022216	0.276715	0.209062	0.770176	BGIOSGA018369	XP_004969979.1uncharacterized protein LOC101783941
TCONS_00002380	0.277088	1.39628	1.0635	BGIOSGA036982	XP_015692217.1 BTB/POZ and TAZ domain-containing protein 1
TCONS_00034349	0.277354	0.689947	0.308759	BGIOSGA034455	XP_022684879.1uncharacterized protein LOC101775226 isoform X2
TCONS_00004603	0.277432	-0.0179701	-0.541312	BGIOSGA000614	XP_021313698.1protein RETICULATA-RELATED 3, chloroplastic-like
TCONS_00030676	0.277541	-0.0406562	0.117887	BGIOSGA032498	XP_006662165.1 caffeoylshikimate esterase-like
TCONS_00007550	0.277555	0.502385	0.06522	BGIOSGA023242	XP_006647038.1 26S protease regulatory subunit S10B homolog B
TCONS_00019303	0.277592	1.65144	0.520082	BGIOSGA020902	XP_006655330.1 MLO-like protein 1
TCONS_00003169	0.277612	-1.0067	-0.434052	BGIOSGA002052	XP_015698600.1 beta-amylase 3, chloroplastic-like
TCONS_00016726	0.277738	-1.48107	-2.32953	BGIOSGA014235	XP_006652910.1 haloalkane dehalogenase 2
TCONS_00033491	0.277951	-1.1422	-0.26368	BGIOSGA035287	XP_006663459.1 ABC transporter I family member 11, chloroplastic
TCONS_00030369	0.278005	-0.463911	0.252065	BGIOSGA022575	XP_015696797.1 inositol-tetrakisphosphate 1-kinase 6
TCONS_00022850	0.278064	0.1127	0.586679	BGIOSGA020595	XP_006656436.1 probable inactive purple acid phosphatase 28
TCONS_00021786	0.278181	1.30903	0.991545	BGIOSGA010143	XP_006655879.1 nucleolar GTP-binding protein 1-like
TCONS_00030034	0.278236	-0.176742	0.0247903	BGIOSGA024830	XP_006661225.2PREDICTED: uncharacterized protein At1g04910-like
TCONS_00001311	0.278402	-0.236147	-0.388899	BGIOSGA031550	XP_006644345.1 AAA-ATPase At3g50940-like
TCONS_00031460	0.278412	-0.0597569	-1.31174	BGIOSGA033278	XP_006661956.1 glycine cleavage system H protein, mitochondrial
TCONS_00023706	0.278487	0.215699	-0.0224999	BGIOSGA004441	XP_006657722.2 NAD-dependent malic enzyme 62 kDa isoform, mitochondrial
TCONS_00036079	0.278622	0.720994	0.957127	BGIOSGA018886	XP_006664045.1 DNAJ protein JJJ1 homolog
TCONS_00034945	0.278644	0.719583	-0.423731	BGIOSGA020687	XP_006662961.1 acyl carrier protein 1, chloroplastic-like
TCONS_00009942	0.278728	0.371647	0.757387	BGIOSGA012492	XP_006649979.1 adenylosuccinate lyase-like
TCONS_00002673	0.278957	-0.605105	-1.24787	BGIOSGA002548	XP_006643650.1 1-deoxy-D-xylulose 5-phosphate reductoisomerase, chloroplastic
TCONS_00004826	0.279044	0.844978	2.06864	BGIOSGA000391	XP_006646532.2 ribosomal lysine N-methyltransferase 3
TCONS_00003442	0.279139	0.381769	0.220457	BGIOSGA012049	XP_015688445.1 protein transport protein SEC23
TCONS_00001124	0.279394	-1.08926	-1.19324	BGIOSGA023484	XP_003569185.1probable protein phosphatase 2C 5 isoform X5
TCONS_00021670	0.279499	-1.01241	-0.359143	BGIOSGA027069	XP_015693936.1 carotenoid 9,10(9',10')-cleavage dioxygenase 1-like
TCONS_00013086	0.279768	0.33323	0.110264	BGIOSGA028762	XP_006650514.1 cyclase-associated protein 1
TCONS_00005954	0.279884	0.583248	0.0972915	BGIOSGA003944	XP_006647211.1 26S protease regulatory subunit 6B homolog
TCONS_00008677	0.27994	0.295046	0.420179	BGIOSGA005753	XP_003570219.1pentatricopeptide repeat-containing protein At1g77360, mitochondrial
TCONS_00008194	0.28008	0.689043	0.896595	BGIOSGA006244	XP_006647390.1 ankyrin repeat protein SKIP35-like
TCONS_00013225	0.280454	0.949935	3.17847	BGIOSGA026844	XP_004981649.1CCG-binding protein 1
TCONS_00004894	0.280587	-1.30091	-0.711034	BGIOSGA004786	XP_020405361.1ribonuclease 2 isoform X2
TCONS_00003838	0.28062	-1.1752	-0.355037	BGIOSGA001372	XP_015699214.1PREDICTED: uncharacterized protein LOC102713681
TCONS_00028053	0.280829	-0.0791999	-0.0173147	BGIOSGA026936	XP_006660152.1 GDT1-like protein 5
TCONS_00004719	0.280972	0.302218	0.397299	BGIOSGA005599	XP_006645001.1 probable methyltransferase PMT11
TCONS_00022360	0.280985	-1.2083	1.40995	BGIOSGA003415	XP_002438500.1GDSL esterase/lipase At5g45910
TCONS_00031836	0.280992	0.683348	-0.0568554	BGIOSGA034645	XP_020393135.1armadillo/beta-catenin-like repeat family protein isoform X1
TCONS_00009646	0.28104	0.130051	-0.145998	BGIOSGA012190	XP_006649695.1 glucose-induced degradation protein 8 homolog
TCONS_00017667	0.281345	0.929846	0.848652	BGIOSGA003505	XP_002439678.140S ribosomal protein S4
TCONS_00031633	0.281673	0.0820253	0.565781	BGIOSGA011650	XP_015697084.1 palmitoyl-protein thioesterase 1-like
TCONS_00029882	0.281802	1.0655	0.829623	BGIOSGA012106	XP_015696697.1 protein transport protein Sec61 subunit alpha-like isoform X1
TCONS_00001880	0.28192	0.518575	1.13185	BGIOSGA020165	NP_001149272.1BSD domain containing protein
TCONS_00007582	0.281929	0.812147	0.726442	BGIOSGA029561	XP_008645668.1UDP-glycosyltransferase 73C3
TCONS_00026218	0.282272	-0.334041	0.730724	BGIOSGA022571	XP_006659247.1 ADP-ribosylation factor 3
TCONS_00034252	0.282274	1.63663	1.1692	BGIOSGA016257	XP_004978608.1U3 small nucleolar ribonucleoprotein protein MPP10
TCONS_00000818	0.28234	-0.635099	-0.673622	BGIOSGA003406	XP_015692415.1PREDICTED: uncharacterized protein LOC102708380
TCONS_00000351	0.282516	-0.962303	0.457181	BGIOSGA013770	XP_015693304.1 auxin-responsive protein IAA2-like
TCONS_00010679	0.282673	-0.314624	1.12	BGIOSGA011758	XP_006650402.1 DUF21 domain-containing protein At2g14520 isoform X1

TCONS_00006576	0.282734	1.28474	0.641313	BGIOSGA032178	XP_006648900.2 cationic amino acid transporter 9, chloroplastic
TCONS_00002683	0.282737	-0.358444	-0.0554554	BGIOSGA017394	XP_006643660.1 proline-rich receptor-like protein kinase PERK8
TCONS_00016721	0.282843	1.14982	1.25953	BGIOSGA014241	XP_006652900.1 glutamyl-tRNA(Gln) amidotransferase subunit A, chloroplastic/mitochondrial isoform X1
TCONS_00020315	0.283021	0.50441	0.526571	BGIOSGA030392	XP_015693486.1PREDICTED: uncharacterized protein LOC102709938
TCONS_00013485	0.283214	-0.324446	0.20361	BGIOSGA009506	NP_001278470.1huntingtin interacting protein K
TCONS_00003753	0.283379	0.055785	-0.542476	BGIOSGA004758	XP_004977214.1putative pectate lyase 21
TCONS_00015431	0.283397	0.768484	0.364366	BGIOSGA004785	XP_015691709.1 pumilio homolog 3-like
TCONS_00024036	0.283601	0.758483	1.15917	BGIOSGA026074	XP_015694921.1 tRNA (guanosine(18)-2'-O)-methyltransferase
TCONS_00000477	0.283604	-0.796043	-1.06583	BGIOSGA007005	XP_006645668.1 cytochrome P450 71A1-like
TCONS_00018747	0.283895	-0.097076	0.732373	BGIOSGA018674	XP_006651564.1 pseudouridine kinase
TCONS_00016916	0.283932	0.766152	0.590992	BGIOSGA021314	XP_015691625.1 SKP1-interacting partner 15
TCONS_00016405	0.284462	-1.34151	-0.700266	BGIOSGA014561	XP_006652631.1PREDICTED: uncharacterized protein LOC102718823 isoform X1
TCONS_00023046	0.284691	0.608226	1.31493	BGIOSGA024641	XP_015695346.1 heterogeneous nuclear ribonucleoprotein A/B-like
TCONS_00016145	0.284707	0.246932	-0.186674	BGIOSGA004989	XP_015692351.1 sn1-specific diacylglycerol lipase beta isoform X1
TCONS_00012347	0.28471	1.07248	1.04589	BGIOSGA026194	XP_015690385.1 RNA-binding protein CP29B, chloroplastic-like
TCONS_00028404	0.284815	-0.180487	-0.224105	BGIOSGA024011	XP_006659647.1 serine/threonine-protein kinase Nek5-like
TCONS_00017828	0.28503	0.509548	0.384532	BGIOSGA024567	XP_004962059.1PHD finger protein ALFIN-LIKE 5
TCONS_00005964	0.285196	0.179891	0.264605	BGIOSGA013268	XP_006648590.2 ARF guanine-nucleotide exchange factor GNOM-like
TCONS_00016851	0.28573	0.514347	-0.0261325	BGIOSGA014113	XP_015692267.1PREDICTED: uncharacterized protein LOC102714176 isoform X3
TCONS_00013604	0.285963	0.48585	-1.49002	BGIOSGA015757	NP_001141064.1membrane protein
TCONS_00000121	0.28603	0.588505	1.6526	BGIOSGA014333	XP_015695730.1 uncharacterized lipoprotein syc1174_c-like
TCONS_00011280	0.286136	0.446756	1.6486	BGIOSGA028737	XP_006651991.1 flap endonuclease 1-B isoform X1
TCONS_00032537	0.286326	0.297302	0.546625	BGIOSGA031579	XP_015697383.1 tubulin-folding cofactor D
TCONS_00032039	0.286346	1.30491	1.65146	BGIOSGA017094	XP_006661708.1 vacuolar-sorting receptor 3-like
TCONS_00004824	0.286484	0.658916	0.243212	BGIOSGA000393	XP_006646530.1 extradiol ring-cleavage dioxygenase-like
TCONS_00028096	0.286499	-0.551588	-1.43773	BGIOSGA005795	XP_006659461.1 probable 4-coumarate--CoA ligase 5
TCONS_00000232	0.286683	1.76458	1.1296	BGIOSGA028894	XP_003564232.1histone H2B.1
TCONS_00022615	0.286985	0.0702557	1.44018	BGIOSGA022181	XP_006656565.1 pentatricopeptide repeat-containing protein At1g08070, chloroplastic-like
TCONS_00011457	0.287029	-0.0370256	0.171246	BGIOSGA019392	XP_006649286.1PREDICTED: uncharacterized protein LOC102717045
TCONS_00020777	0.287097	-0.757483	0.00746134	BGIOSGA022973	XP_015693579.1 alpha-glucan water dikinase, chloroplastic
TCONS_00001241	0.287191	-0.613437	0.0101786	BGIOSGA028054	XP_002455862.1uncharacterized protein LOC8082462
TCONS_00025545	0.287274	0.415298	0.525759	BGIOSGA023887	XP_006657976.1 multifunctional methyltransferase subunit TRM112-like protein At1g78190 isoform X3
TCONS_00035894	0.287466	-0.142202	0.463392	BGIOSGA029070	XP_002453153.2probable nucleolar protein 5-2
TCONS_00036233	0.287475	0.379961	0.320648	BGIOSGA025074	XP_004962768.1ocs element-binding factor 1
TCONS_00016144	0.287494	1.04099	-0.191514	BGIOSGA033495	XP_010240054.1serine/threonine-protein kinase OXI1
TCONS_00012434	0.287677	-0.584082	0.367341	BGIOSGA029066	XP_006650184.1PREDICTED: uncharacterized protein LOC102718528
TCONS_00005149	0.287699	-1.07962	-0.906691	BGIOSGA027714	XP_020395800.1actin-97 isoform X1
TCONS_00029506	0.287912	0.397667	0.0411989	BGIOSGA037089	XP_015696452.1 26S protease regulatory subunit 6B homolog
TCONS_00013830	0.287935	0.0878083	0.288861	BGIOSGA016010	XP_006652130.1 UPF0307 protein plu4061
TCONS_00007495	0.288027	0.196223	-0.0557795	BGIOSGA036567	XP_004951625.130S ribosomal protein S21, chloroplastic
TCONS_00029613	0.288082	0.454404	-0.84901	BGIOSGA030306	XP_015696597.1 ankyrin-1-like
TCONS_00033621	0.288165	-0.410007	-0.495482	BGIOSGA040498	XP_006663501.1 UPF0481 protein At3g47200-like
TCONS_00037515	0.288426	-0.0581737	0.650304	BGIOSGA018976	XP_021302550.1uncharacterized protein LOC8082998
TCONS_00026803	0.28901	0.314475	0.622384	BGIOSGA006207	XP_006659510.1 probable bifunctional riboflavin biosynthesis protein RIBA 1, chloroplastic
TCONS_00015921	0.289291	0.233974	-0.235307	BGIOSGA006268	XP_006652305.1 probable L-ascorbate peroxidase 7, chloroplastic
TCONS_00031260	0.289501	-0.00182825	-0.254308	BGIOSGA033078	XP_006661831.1 protein CHROMATIN REMODELING 20 isoform X1
TCONS_00016290	0.289719	-1.51813	0.00444951	BGIOSGA009848	XP_006652539.1 GEM-like protein 4
TCONS_00011327	0.289758	0.20508	0.735774	BGIOSGA013937	XP_006665037.1 myb-like protein X
TCONS_00017741	0.289953	-0.0112327	0.450301	BGIOSGA008237	XP_006654357.1 RNA polymerase II C-terminal domain phosphatase-like 4
TCONS_00024954	0.290059	-0.134536	-0.00486533	BGIOSGA008556	XP_006658492.1 glucose-6-phosphate 1-dehydrogenase, chloroplastic-like
TCONS_00010121	0.290079	-0.277254	0.762148	BGIOSGA029256	XP_006650106.2 serine/arginine-rich SC35-like splicing factor SCL28
TCONS_00024875	0.290138	-0.122553	0.042325	BGIOSGA016046	XP_006658455.1 BTB/POZ domain-containing protein At2g13690-like
TCONS_00007231	0.290174	-0.0117849	-0.544131	BGIOSGA008879	XP_006648225.1 serine carboxypeptidase 3
TCONS_00008185	0.290737	0.392044	-0.277075	BGIOSGA005988	XP_004952783.1two-component response regulator ORR2
TCONS_00003940	0.290737	0.35505	0.825023	BGIOSGA036679	XP_015689214.1 probable calcium-binding protein CML12

transcriptome

TCONS_00029298	0.290782	0.54783	0.89089	BGIOSGA019462	XP_006660590.1 leucine--tRNA ligase, cytoplasmic-like
TCONS_00032892	0.290875	-2.0545	-2.32642	BGIOSGA036922	XP_006662695.1PREDICTED: uncharacterized protein LOC102706327
TCONS_00027260	0.290939	2.29005	1.0783	BGIOSGA029883	XP_006659071.1 succinate dehydrogenase [ubiquinone] iron-sulfur subunit 1, mitochondrial isoform X1
TCONS_00034832	0.290945	-0.257195	0.289441	BGIOSGA037144	NP_001143653.1uncharacterized LOC100276375 precursor
TCONS_00029630	0.291042	0.478281	1.34091	BGIOSGA030191	XP_004962128.1upstream activation factor subunit UAF30
TCONS_00019235	0.291149	0.141039	0.156626	BGIOSGA018113	XP_006654358.1 peroxisomal adenine nucleotide carrier 1-like
TCONS_00019541	0.291265	0.600615	0.733314	BGIOSGA034571	XP_006654577.1 histone-lysine N-methyltransferase, H3 lysine-9 specific SUVH1-like
TCONS_00031751	0.291286	1.29137	-0.180786	BGIOSGA032367	XP_006661612.1 uncharacterized ATP-dependent helicase C29A10.10c-like
TCONS_00015123	0.291471	-0.531211	-0.000918801	BGIOSGA017302	XP_006652968.1 protein IQ-DOMAIN 32
TCONS_00022455	0.291521	-0.451925	0.55358	BGIOSGA021002	XP_015693779.1 histone deacetylase 2 isoform X2
TCONS_00025580	0.291794	-2.29836	-0.177617	BGIOSGA012321	XP_015694826.1 phosphate metabolism protein 8-like
TCONS_00021361	0.291795	-0.16786	0.747585	BGIOSGA011521	XP_006656480.1 pre-mRNA-splicing factor syf2
TCONS_00002548	0.291904	1.02343	-0.894098	BGIOSGA005192	XP_006645378.1PREDICTED: uncharacterized protein LOC102722445
TCONS_00027938	0.291916	-0.956634	-0.727686	BGIOSGA013757	XP_006650751.1PREDICTED: uncharacterized protein LOC102717971
TCONS_00001047	0.292143	0.0712033	-0.255168	BGIOSGA010136	XP_015693243.1PREDICTED: uncharacterized protein LOC102715308
TCONS_00006708	0.292145	-0.469382	-0.242474	BGIOSGA013935	XP_015689465.1 cyclin-dependent kinase F-4
TCONS_00009869	0.292188	-0.015677	0.468942	BGIOSGA006573	XP_006649933.1 40S ribosomal protein S7
TCONS_00009318	0.292237	-0.123833	1.0278	BGIOSGA033224	XP_006651039.1 protein SHOOT GRAVITROPISM 6
TCONS_00019939	0.292313	-1.49206	-0.509269	BGIOSGA019872	XP_006655670.1 glutathione gamma-glutamylcysteinyltransferase 1-like
TCONS_00021623	0.292448	-1.30575	-0.112422	BGIOSGA029762	XP_015694108.1 ATP-dependent 6-phosphofructokinase 3-like
TCONS_00028324	0.292633	0.807643	-0.424266	BGIOSGA035073	XP_004973917.1uncharacterized protein LOC101763435
TCONS_00022315	0.292684	0.204418	0.288893	BGIOSGA021164	XP_006656117.2 BTB/POZ domain-containing protein POB1-like
TCONS_00004107	0.29298	-1.25958	-0.59812	BGIOSGA033480	XP_003569488.1plastidial pyruvate kinase 2
TCONS_00011723	0.29319	-1.3302	-1.02463	BGIOSGA011286	XP_015690909.1 sulfate transporter 2.1-like isoform X3
TCONS_00024214	0.293235	-0.91941	0.693803	BGIOSGA037767	XP_021318591.1uncharacterized protein LOC8075867 isoform X1
TCONS_00015906	0.293344	-0.621639	-0.18404	BGIOSGA003439	XP_006652304.1PREDICTED: uncharacterized protein LOC102714005
TCONS_00019248	0.293505	0.415683	0.490958	BGIOSGA018100	XP_015692769.1 preprotein translocase subunit SCY2, chloroplastic isoform X2
TCONS_00002981	0.293619	-0.0409813	-0.59685	BGIOSGA018678	XP_006643850.1 glutamine--tRNA ligase-like
TCONS_00028790	0.294022	1.90642	-0.162403	BGIOSGA015622	XP_015696543.1 probable WRKY transcription factor 41
TCONS_00023784	0.294035	-1.07545	-0.814928	BGIOSGA005821	XP_024317889.1uncharacterized protein LOC100831688
TCONS_00002978	0.294041	1.10353	1.44833	BGIOSGA002241	XP_006645578.1 DEAD-box ATP-dependent RNA helicase 39
TCONS_00027837	0.294256	0.795921	0.260401	BGIOSGA027167	XP_015696230.1PREDICTED: uncharacterized protein LOC102706871
TCONS_00008068	0.294301	0.576232	0.310478	BGIOSGA006385	XP_006647258.1 lipopolysaccharide-induced tumor necrosis factor-alpha factor homolog
TCONS_00006369	0.294326	1.45078	0.231122	BGIOSGA023419	XP_015690303.1 serine/threonine-protein kinase HT1-like
TCONS_00011244	0.294502	-0.873223	-0.635709	BGIOSGA013844	XP_006650815.1 chaperone protein DnaJ-like
TCONS_00019447	0.294511	0.379319	0.0887379	BGIOSGA004755	XP_010231285.1SAC3 family protein A isoform X3
TCONS_00025760	0.294522	-2.3018	-1.24853	BGIOSGA004321	XP_006658177.2 peroxidase 21-like
TCONS_00019056	0.294553	0.176203	0.461887	BGIOSGA001597	XP_021321574.1uncharacterized protein LOC110437414 isoform X1
TCONS_00023262	0.294555	0.200507	0.785055	BGIOSGA009431	XP_015695344.1PREDICTED: uncharacterized protein At4g14100-like
TCONS_00019074	0.294627	-0.876825	0.0349458	BGIOSGA018265	XP_006656002.1 guanine nucleotide-binding protein alpha-1 subunit isoform X1
TCONS_00009778	0.294878	0.370816	0.596707	BGIOSGA035977	XP_006649845.1 protein spotted leaf 11-like isoform X2
TCONS_00028687	0.294901	0.666387	1.00178	BGIOSGA006869	XP_006660499.1 serine/threonine-protein phosphatase PP-X isozyme 2
TCONS_00017049	0.294912	0.138277	0.750504	BGIOSGA029449	XP_003561933.1uncharacterized protein LOC100834465
TCONS_00031583	0.295039	-0.281146	0.631696	BGIOSGA011602	XP_002467557.1arsenate reductase 2.1
TCONS_00027559	0.295176	-3.98637	0.556712	BGIOSGA032574	XP_021306439.1zinc finger BED domain-containing protein RICESLEEPER 2-like
TCONS_00012260	0.295251	0.778989	0.739322	BGIOSGA003167	XP_006650075.2PREDICTED: uncharacterized protein LOC102709821 isoform X1
TCONS_00034265	0.295301	0.483557	0.548706	BGIOSGA036663	XP_006664974.1 probable calcium-binding protein CML49
TCONS_00021355	0.295316	0.536898	0.612282	BGIOSGA007410	XP_006656475.1 peptidyl-prolyl cis-trans isomerase CYP20-1-like
TCONS_00033196	0.295325	0.697185	0.0974052	BGIOSGA034297	XP_015697521.1 putative receptor-like protein kinase At4g00960 isoform X2
TCONS_00026405	0.29553	1.47184	0.957925	BGIOSGA030083	XP_015695567.1 mediator of RNA polymerase II transcription subunit 14-like
TCONS_00032133	0.295735	0.56174	0.574439	BGIOSGA003756	XP_015696978.1 F-box protein At5g49610-like

TCONS_00023445	0.295956	-1.65153	2.13398	BGIOSGA020686	XP_015694877.1 metal transporter Nramp1 isoform X1
TCONS_00012381	0.295993	-0.228905	-0.773759	BGIOSGA013621	XP_006650154.1 serine/threonine-protein kinase SAPK1
TCONS_00014017	0.296004	-0.654204	0.275582	BGIOSGA016162	XP_004975311.1uncharacterized protein LOC101765475
TCONS_00029791	0.296127	0.73195	0.797807	BGIOSGA028752	XP_015696746.1 histone-lysine N-methyltransferase ASHH3-like
TCONS_00025385	0.296176	0.234873	1.16854	BGIOSGA009977	XP_002463061.1pre-mRNA-splicing factor 18
TCONS_00022879	0.296422	-2.36843	-1.02383	BGIOSGA002505	XP_004966377.1protein O-linked-mannose beta-1,4-N-acetylglucosaminyltransferase 2
TCONS_00016655	0.296481	-1.45496	-0.307948	BGIOSGA026517	XP_004979375.1putative FBD-associated F-box protein At1g61330 isoform X1
TCONS_00032181	0.296604	1.16642	0.388928	BGIOSGA029762	XP_006661761.1 ATP-dependent 6-phosphofructokinase 5, chloroplastic-like
TCONS_00005397	0.296658	-0.391827	-0.294721	BGIOSGA020580	XP_006646872.1 protein AIG2-like
TCONS_00004137	0.296689	-0.0138672	0.794104	BGIOSGA020365	XP_006644492.1 probable E3 ubiquitin-protein ligase HIP1 isoform X2
TCONS_00010818	0.296745	-0.170348	2.11704	BGIOSGA013443	XP_006650494.1 threonine dehydratase biosynthetic, chloroplastic
TCONS_00007311	0.297103	0.490568	-0.124803	BGIOSGA004995	XP_015688981.1 CASP-like protein 4A2
TCONS_00022243	0.297202	0.30695	0.605352	BGIOSGA021253	XP_006656084.1 mitogen-activated protein kinase kinase 3 isoform X1
TCONS_00033466	0.29725	0.160432	-0.694802	BGIOSGA011210	XP_015697813.1 pentatricopeptide repeat-containing protein At5g04780-like
TCONS_00020966	0.297362	-0.505177	-0.0419382	BGIOSGA023191	XP_006656200.1 3-hydroxyisobutyryl-CoA hydrolase-like protein 3, mitochondrial
TCONS_00025354	0.297576	1.05729	0.281995	BGIOSGA003373	XP_014753211.1caffeoylshikimate esterase
TCONS_00006431	0.297897	0.434044	0.474364	BGIOSGA008595	XP_006664904.2 protein FAR1-RELATED SEQUENCE 6-like isoform X2
TCONS_00037000	0.298314	-0.217755	0.331926	BGIOSGA011871	XP_006664493.1 lysine-specific demethylase REF6-like
TCONS_00001474	0.298993	-0.928082	-1.18043	BGIOSGA019840	XP_006644440.1 GDSL esterase/lipase At1g28600-like isoform X2
TCONS_00014711	0.299251	1.36165	0.781632	BGIOSGA008749	XP_015692170.1 acetylglutamate kinase-like
TCONS_00032960	0.299327	-0.468481	0.204896	BGIOSGA022517	XP_015697557.1 protein trichome birefringence-like 10
TCONS_00006568	0.299469	0.428525	0.789232	BGIOSGA008734	XP_006647620.1 probable enoyl-CoA hydratase 2, mitochondrial
TCONS_00015604	0.299538	-0.529563	-0.671934	BGIOSGA016116	XP_006652169.1 NADH dehydrogenase [ubiquinone] 1 alpha subcomplex subunit 2
TCONS_00006929	0.299665	0.595809	0.532125	BGIOSGA009109	XP_015689513.1 THO complex subunit 5A isoform X1
TCONS_00021620	0.29973	1.13588	-0.130518	BGIOSGA001950	XP_004964835.2anthranilate N-benzoyltransferase protein 1
TCONS_00006869	0.299882	-0.042813	0.377007	BGIOSGA004143	XP_003570655.1sphinganine C4-monooxygenase 1
TCONS_00007486	0.300006	-0.741871	0.222527	BGIOSGA006998	XP_021314992.1glycerophosphodiester phosphodiesterase GDPDL7 isoform X2
TCONS_00002428	0.300032	-0.734229	0.103053	BGIOSGA025961	XP_015690781.1 alpha-glucosidase 2-like
TCONS_00002957	0.300045	-0.836079	-2.32866	BGIOSGA026166	XP_012702012.2 crocetin glucosyltransferase, chloroplastic-like
TCONS_00008828	0.301263	0.266054	1.07006	BGIOSGA005597	XP_006647923.1 lysine-specific histone demethylase 1 homolog 1
TCONS_00020035	0.301273	0.664958	0.480481	BGIOSGA015390	XP_006655731.1 E3 ubiquitin ligase BIG BROTHER-like
TCONS_00021133	0.30179	-0.0621929	0.660384	BGIOSGA023354	XP_006656293.1 ER membrane protein complex subunit 7 homolog
TCONS_00017958	0.301832	0.177819	1.11106	BGIOSGA000547	XP_002439933.1CTD nuclear envelope phosphatase 1 homolog
TCONS_00033688	0.302049	-0.289866	0.708284	BGIOSGA037401	XP_006662977.1 probable prefoldin subunit 2
TCONS_00014358	0.302638	-1.27306	-2.39672	BGIOSGA027644	XP_015691939.1PREDICTED: uncharacterized protein LOC107304087
TCONS_00001022	0.302666	0.329593	1.12877	BGIOSGA021571	XP_006644223.1 chaperone protein dnaJ 15
TCONS_00010489	0.302752	0.124426	-0.54175	BGIOSGA009142	XP_004982450.1subtilisin-like protease SBT1.7
TCONS_00013136	0.30278	0.726102	0.587296	BGIOSGA027270	XP_006650555.1 putative rRNA methyltransferase YlbH
TCONS_00015819	0.302788	-0.44589	0.307649	BGIOSGA015167	XP_003579641.1sucrose nonfermenting 4-like protein isoform X1
TCONS_00015389	0.302852	0.481807	0.782128	BGIOSGA015982	XP_015692069.1 actin-related protein 9 isoform X2
TCONS_00008628	0.30304	-2.13774	-0.352599	BGIOSGA022028	XP_015689332.1 oligopeptide transporter 4
TCONS_00001448	0.303052	0.167349	0.156532	BGIOSGA013062	XP_004969282.1serine/threonine-protein kinase STY8
TCONS_00023415	0.303279	0.604331	0.944465	BGIOSGA025448	XP_006658433.1 transmembrane protein 184A-like
TCONS_00018573	0.303392	0.23932	1.00028	BGIOSGA002161	XP_015693099.1 shaggy-related protein kinase alpha
TCONS_00013408	0.303513	1.62049	1.83272	BGIOSGA009579	XP_006652009.1 prohibitin-1, mitochondrial-like
TCONS_00016587	0.303539	1.1487	0.654314	BGIOSGA013800	XP_006652796.1 serine/threonine-protein kinase HT1-like
TCONS_00025963	0.303683	-0.343306	0.983855	BGIOSGA010549	XP_015696099.1 MOB kinase activator-like 1A
TCONS_00003951	0.303688	0.391581	0.40057	BGIOSGA001243	XP_006644351.1 transcriptional corepressor LEUNIG-like isoform X2
TCONS_00029566	0.303727	0.828462	0.850845	BGIOSGA030253	XP_015696535.1PREDICTED: uncharacterized protein LOC102720352
TCONS_00029402	0.303748	0.286756	0.508189	BGIOSGA013489	XP_003578516.1protein XAP5 CIRCADIAN TIMEKEEPER
TCONS_00001289	0.3038	0.267489	0.570455	BGIOSGA033437	XP_006644338.1PREDICTED: uncharacterized protein LOC102701389
TCONS_00033169	0.303844	-3.27156	-1.44367	BGIOSGA021088	XP_006662825.1 dirigent protein 21-like
TCONS_00033970	0.303907	1.5874	0.893702	BGIOSGA020019	XP_006663095.1 auxilin-related protein 2-like
TCONS_00009692	0.303964	-0.53518	0.169482	BGIOSGA036627	XP_015690995.1 nucleolin isoform X3
TCONS_00036325	0.304008	-0.376151	0.138773	BGIOSGA001094	XP_003579224.1transcription factor HHO6
TCONS_00014060	0.304204	1.09237	1.40474	BGIOSGA036081	XP_010237221.1F-box/LRR-repeat protein 14

TCONS_00000698	0.304427	-0.0900959	0.128097	BGIOSGA003290	XP_006644079.1 protoporphyrinogen oxidase, chloroplastic
TCONS_00032439	0.304598	0.359615	0.108413	BGIOSGA031678	XP_006662448.2PREDICTED: uncharacterized protein LOC102718871
TCONS_00014618	0.304995	-0.516741	-0.268164	BGIOSGA024525	XP_006652529.1 IAA-amino acid hydrolase ILR1-like 5
TCONS_00006633	0.30501	0.556053	0.628187	BGIOSGA008798	XP_004953433.2lysophospholipid acyltransferase 1
TCONS_00030522	0.305094	0.614005	-1.02655	BGIOSGA006534	XP_015696261.1PREDICTED: uncharacterized protein At2g39795, mitochondrial-like isoform X1
TCONS_00035136	0.305302	-0.678826	0.509407	BGIOSGA035236	XP_003571252.1putative F-box protein At2g33200
TCONS_00015239	0.30534	0.361034	-0.125574	BGIOSGA015740	XP_006653094.1 fatty acid amide hydrolase isoform X1
TCONS_00019920	0.305345	-0.247333	0.195136	BGIOSGA017442	XP_006654881.1 26S proteasome regulatory subunit RPN13
TCONS_00011049	0.30535	0.886113	1.47873	BGIOSGA013647	XP_015690091.1 probable methyltransferase PMT28
TCONS_00036106	0.305404	#NA	-1.12568	BGIOSGA033779	XP_006664058.1 LIM domain-containing protein WLIM1
TCONS_00002068	0.305496	0.673752	-1.43205	BGIOSGA023345	XP_006646435.1PREDICTED: uncharacterized protein LOC102700298
TCONS_00006693	0.305552	-0.254776	-0.301074	BGIOSGA033503	XP_006647730.1 TOM1-like protein 2
TCONS_00017068	0.305736	#NA	-0.174658	#N/A	#N/A
TCONS_00003792	0.305957	-0.106336	0.267231	BGIOSGA001419	XP_006644269.1 RWD domain-containing protein 1
TCONS_00019900	0.305974	1.68568	0.859808	BGIOSGA017464	XP_006654861.1 paired amphipathic helix protein Sin3-like 5 isoform X2
TCONS_00025575	0.306256	0.589655	0.98928	BGIOSGA026319	XP_010235101.1DEAD-box ATP-dependent RNA helicase 36
TCONS_00037506	0.306447	-1.11198	-1.88936	BGIOSGA035862	XP_006664730.1 C-factor
TCONS_00006093	0.306496	-0.0577426	0.564566	BGIOSGA003800	XP_006647286.1 heat stress transcription factor A-5-like
TCONS_00023993	0.306573	0.725756	0.44176	BGIOSGA033112	XP_006657892.1 heterogeneous nuclear ribonucleoprotein 1
TCONS_00028180	0.306664	-0.0186811	0.42369	BGIOSGA026804	XP_006659501.2 IMPACT family member in pol 5'region
TCONS_00022536	0.306722	-0.28694	-1.67018	BGIOSGA021766	XP_006657119.1 phosphatidylinositol/phosphatidylcholine transfer protein SFH2-like
TCONS_00023187	0.307101	-0.0672749	0.770637	BGIOSGA023649	XP_006656526.1 DNA-binding protein RHL1
TCONS_00016987	0.307123	1.1431	1.04844	BGIOSGA019035	XP_006653963.1 DEAD-box ATP-dependent RNA helicase 17
TCONS_00016425	0.30728	0.419353	-0.45233	BGIOSGA004541	XP_006643834.1 aquaporin SIP1-1
TCONS_00023444	0.307361	0.851667	0.177762	BGIOSGA033900	XP_021310801.1uncharacterized protein LOC110433303 isoform X1
TCONS_00018905	0.307636	-1.71379	2.09763	BGIOSGA018990	XP_006655132.1 probable glucan 1,3-beta-glucosidase A
TCONS_00015796	0.307725	0.553221	0.560873	BGIOSGA016303	XP_006652220.1 zinc finger CCCH domain-containing protein 27
TCONS_00025517	0.308024	0.127371	-0.0689602	BGIOSGA012850	XP_002463169.1probable small nuclear ribonucleoprotein G
TCONS_00036656	0.308103	-0.250911	-0.471652	BGIOSGA028495	XP_006659342.1 transcription initiation factor IIE subunit alpha
TCONS_00025534	0.308426	-0.0986872	1.09074	BGIOSGA023899	XP_015694727.1 zinc finger CCCH domain-containing protein 62-like
TCONS_00029349	0.308568	-0.218523	-0.285642	BGIOSGA029560	XP_015696423.1 UDP-glycosyltransferase 74F2-like
TCONS_00007989	0.308577	1.15139	0.43874	BGIOSGA015335	XP_015689602.1 aspartic proteinase-like protein 2 isoform X1
TCONS_00014342	0.30874	0.101599	0.61343	BGIOSGA014738	XP_006652340.1 mitochondrial uncoupling protein 3
TCONS_00007131	0.309546	0.25488	-0.200371	BGIOSGA001488	XP_006648144.2 protein BTR1
TCONS_00024078	0.309632	#NA	0.126734	BGIOSGA019932	NP_001136465.1uncharacterized protein LOC100216576
TCONS_00033758	0.309745	0.0953489	-0.968819	BGIOSGA033689	XP_006663018.1 wheatwin-1-like
TCONS_00018121	0.309799	1.10936	0.922246	BGIOSGA020175	XP_006654638.1 cleavage stimulating factor 64-like
TCONS_00005237	0.30984	1.11258	0.747104	BGIOSGA013544	XP_015689265.1 DExH-box ATP-dependent RNA helicase DExH12
TCONS_00022446	0.310012	0.792986	0.639336	BGIOSGA012197	XP_006656173.1 transmembrane 9 superfamily member 7
TCONS_00004906	0.310178	-1.19248	0.434654	BGIOSGA000315	XP_015698577.1 OTU domain-containing protein At3g57810-like
TCONS_00008094	0.310199	0.391298	0.969445	BGIOSGA006357	XP_015688614.1 WD repeat-containing protein LWD1-like
TCONS_00035987	0.310469	0.0996946	0.436742	BGIOSGA020334	XP_006650655.1 CASP-like protein 5C1
TCONS_00018560	0.310479	0.377976	1.32336	BGIOSGA030850	XP_015692469.1 solute carrier family 40 member 3, chloroplastic-like
TCONS_00023954	0.310501	-0.235346	-0.129011	BGIOSGA025986	XP_006657874.1 adenylyl-sulfate kinase 3
TCONS_00021469	0.310558	0.217095	0.793412	BGIOSGA008573	XP_006656562.1 pentatricopeptide repeat-containing protein At5g27270
TCONS_00001063	0.310627	-0.735702	0.446179	BGIOSGA003679	XP_006644247.2 general transcription factor 3C polypeptide 5-like
TCONS_00003281	0.31066	0.631764	0.602825	BGIOSGA019055	XP_006644032.1 uncharacterized isomerase BH0283-like
TCONS_00025812	0.310717	-0.576142	-2.4905	BGIOSGA018174	XP_012702109.1beta-carotene isomerase D27, chloroplastic
TCONS_00005731	0.310826	-0.151845	-0.597216	BGIOSGA007846	XP_015688527.1PREDICTED: uncharacterized protein LOC102714621
TCONS_00020051	0.310946	-0.0738	1.13323	BGIOSGA026706	XP_006655747.1 uncharacterized ABC transporter ATP-binding protein C825.01
TCONS_00014283	0.31108	-0.162441	0.388578	BGIOSGA018676	XP_006653408.2PREDICTED: uncharacterized protein At4g26450
TCONS_00020037	0.31113	0.154169	0.360946	BGIOSGA003321	XP_015693792.1 peroxisomal membrane protein 11-5

TCONS_00009323	0.311312	1.2021	1.65566	BGIOSGA011867	XP_006649408.1 nephrocystin-3-like
TCONS_00004622	0.311328	0.255144	-0.926407	BGIOSGA033226	XP_003564479.1calcium uniporter protein 6, mitochondrial isoform X2
TCONS_00024630	0.312038	-0.335606	-0.615468	BGIOSGA019294	XP_004955561.1ubiquitin-conjugating enzyme E2 2
TCONS_00006491	0.312232	#NA	-3.46471	BGIOSGA002316	XP_015689603.1 uncharacterized membrane protein At1g06890-like
TCONS_00009743	0.312295	0.250165	0.203829	BGIOSGA031563	XP_006649811.1 SH3 domain-containing protein 2-like
TCONS_00015865	0.312339	-1.05979	-1.14304	BGIOSGA036595	XP_006653380.2 transcription factor E2FA-like isoform X2
TCONS_00013372	0.312442	1.88761	1.46077	BGIOSGA008326	XP_006652138.1 probable protein S-acyltransferase 14
TCONS_00031625	0.3126	-0.167035	-0.295696	BGIOSGA011626	XP_015697140.1 quinone oxidoreductase 1-like
TCONS_00001814	0.3126	-0.112587	-0.378158	BGIOSGA025735	XP_006646319.1 dihydroorotase, mitochondrial
TCONS_00001832	0.312638	-0.0444007	-0.068123	BGIOSGA004467	XP_006646327.1 tobamovirus multiplication protein 1
TCONS_00030552	0.312702	1.14514	0.520359	BGIOSGA027530	XP_015696608.1PREDICTED: uncharacterized protein LOC102714952
TCONS_00010339	0.31297	0.339866	0.191523	BGIOSGA012915	NP_001148102.1DNA binding protein
TCONS_00034515	0.312976	-2.86795	-1.14462	BGIOSGA011309	XP_006662819.1 alcohol dehydrogenase 2-like
TCONS_00026880	0.313015	-0.119361	0.397983	BGIOSGA020128	XP_006659546.2 F-box protein At-B
TCONS_00026743	0.313091	-0.387363	0.599154	BGIOSGA012071	XP_015696006.1 urease isoform X1
TCONS_00028842	0.313195	0.247845	0.445344	BGIOSGA020263	XP_006660565.1 mitochondrial outer membrane protein porin 1
TCONS_00025668	0.313205	-0.443852	0.173273	BGIOSGA011595	XP_002465724.1uncharacterized protein LOC8081911 isoform X2
TCONS_00034246	0.313249	0.0145302	-0.146683	BGIOSGA034556	XP_006662714.1 protein ZINC INDUCED FACILITATOR-LIKE 1-like isoform X3
TCONS_00015016	0.313347	1.03847	-0.467474	BGIOSGA027551	XP_006652870.1 transcription factor bHLH112-like
TCONS_00004449	0.313363	-1.01566	0.285031	BGIOSGA000773	XP_015695137.1PREDICTED: uncharacterized protein LOC102704847
TCONS_00002203	0.313429	-0.703912	0.162979	BGIOSGA004838	XP_006645054.1PREDICTED: uncharacterized protein YnbD-like isoform X1
TCONS_00018314	0.313763	0.266333	0.114744	BGIOSGA023694	XP_006655591.1 60S ribosomal protein L18a-like protein
TCONS_00002355	0.313769	-1.30586	-1.49951	BGIOSGA025518	XP_006646572.1 4-coumarate--CoA ligase-like 6
TCONS_00016258	0.313973	-3.45417	-1.35355	BGIOSGA007967	XP_006652513.1 casein kinase I-like isoform X1
TCONS_00003285	0.313984	1.39757	0.700875	BGIOSGA035578	XP_015693716.1 protein SAD1/UNC-84 domain protein 1-like
TCONS_00006472	0.31405	0.557412	0.544887	BGIOSGA008643	XP_006647533.1 succinyl-CoA ligase [ADP-forming] subunit beta, mitochondrial
TCONS_00033579	0.314262	0.608091	-0.301771	BGIOSGA021589	XP_006662965.1 succinate dehydrogenase assembly factor 2, mitochondrial
TCONS_00034254	0.314563	-0.115057	0.336873	BGIOSGA000455	XP_006664843.2 cytochrome P450 94B3-like
TCONS_00027670	0.314787	0.309936	0.841575	BGIOSGA013964	XP_015695548.1 tetraspanin-8-like
TCONS_00023465	0.315102	0.585634	0.748606	BGIOSGA026258	XP_015694894.1 protein MAK16 homolog A
TCONS_00034035	0.315333	-2.85436	-1.70211	BGIOSGA033565	XP_006663108.1PREDICTED: uncharacterized protein LOC102720559
TCONS_00021923	0.315349	-0.65744	0.58848	BGIOSGA012815	XP_003564036.1RING-H2 finger protein ATL46
TCONS_00015044	0.315356	1.23855	0.471857	BGIOSGA037921	XP_002448626.1V-type proton ATPase subunit D
TCONS_00019291	0.315689	0.419321	0.614206	BGIOSGA018060	XP_015692861.1PREDICTED: LOW QUALITY PROTEIN: uncharacterized protein Cbei_0202
TCONS_00005723	0.315802	0.39781	0.771967	BGIOSGA007840	XP_015689375.1 citrate synthase, glyoxysomal-like
TCONS_00013516	0.316026	-0.470239	0.0390576	BGIOSGA031780	XP_006650877.1 ras-related protein RABA2a-like
TCONS_00028656	0.316255	0.0586713	1.04891	BGIOSGA036218	XP_004966126.1disease resistance protein RPM1
TCONS_00017143	0.31626	-0.362413	-0.0815131	BGIOSGA019189	XP_006654056.1 bifunctional protein FolD 2
TCONS_00017061	0.316464	-0.955944	0.037372	BGIOSGA003709	XP_006654003.1 ocs element-binding factor 1-like
TCONS_00009107	0.316651	0.898063	0.561701	BGIOSGA005294	XP_006648184.1PREDICTED: uncharacterized protein LOC102715180
TCONS_00025525	0.316724	0.286275	1.02719	BGIOSGA026325	XP_015695410.1 elongation factor 1-delta 1
TCONS_00035109	0.317058	0.223946	-0.615229	BGIOSGA033702	XP_015697941.1 GDP-mannose 3,5-epimerase 2 isoform X2
TCONS_00008521	0.317227	-0.310075	0.44663	BGIOSGA014596	XP_015688682.1PREDICTED: uncharacterized protein LOC102707846
TCONS_00003644	0.317373	-0.0389254	-0.284702	BGIOSGA023577	XP_003567622.1protein DETOXIFICATION 12 isoform X1
TCONS_00034387	0.317515	1.22151	0.629657	BGIOSGA037310	XP_003577783.1calcium-dependent protein kinase 24
TCONS_00011269	0.317591	0.878324	0.0425891	BGIOSGA024533	XP_006650845.1 protein transport protein SEC13 homolog B-like
TCONS_00000517	0.317642	-0.506843	-2.60087	BGIOSGA017051	XP_003566154.1glutaredoxin domain-containing cysteine-rich protein CG12206
TCONS_00021659	0.317974	0.0677193	0.492022	BGIOSGA021862	XP_006655817.1 protein capicua homolog
TCONS_00028373	0.318376	0.623776	0.894539	BGIOSGA026590	XP_004961658.1glutamate-1-semialdehyde 2,1-aminomutase, chloroplastic
TCONS_00017078	0.318429	-2.85121	-0.883339	BGIOSGA005230	XP_015693223.1 peroxidase 2-like isoform X2
TCONS_00021897	0.318656	0.843065	0.77057	BGIOSGA009056	XP_004966817.1transcription termination factor MTERF2, chloroplastic
TCONS_00030070	0.318882	0.484326	-0.151989	BGIOSGA016698	XP_015696770.1 S-adenosylmethionine decarboxylase proenzyme-like
TCONS_00002477	0.318916	1.04894	1.8505	BGIOSGA005121	XP_006645289.1 putative cullin-like protein 2 isoform X1
TCONS_00005526	0.319124	-1.76342	-1.02852	BGIOSGA006201	XP_004952859.1sugar transport protein MST1 isoform X1
TCONS_00020776	0.319179	1.03173	0.144789	BGIOSGA022972	XP_004951891.1probable transcription factor At1g11510
TCONS_00022670	0.320054	-1.07648	-0.0527946	BGIOSGA012337	XP_006656310.1 anthocyanidin reductase-like

TCONS_00005369	0.320098	0.425833	0.353666	BGIOSGA024533	XP_006646853.1 protein transport protein SEC13 homolog B-like
TCONS_00016390	0.320336	-0.509062	0.0498	BGIOSGA014575	XP_015691760.1 serine racemase
TCONS_00020442	0.320386	0.339725	0.831489	BGIOSGA014117	XP_014752936.1probable inactive poly [ADP-ribose] polymerase SRO1 isoform X2
TCONS_00025412	0.320467	0.501781	0.189036	BGIOSGA039190	XP_003562826.1geranylgeranyl pyrophosphate synthase, chloroplastic
TCONS_00014120	0.320758	-0.36603	1.07657	BGIOSGA029054	XP_002446304.1protein NUCLEAR FUSION DEFECTIVE 4
TCONS_00001653	0.320767	-0.719789	0.300011	BGIOSGA004286	XP_006644580.2PREDICTED: uncharacterized protein LOC102701670
TCONS_00007100	0.32083	-1.24731	-2.9454	#N/A	#N/A
TCONS_00029889	0.321176	1.64567	0.88221	BGIOSGA022549	XP_015696318.1 bystin
TCONS_00003487	0.321226	0.164265	0.536687	BGIOSGA019216	XP_006644141.2 serine/threonine-protein phosphatase 4 regulatory subunit 3
TCONS_00030248	0.321237	0.0510218	0.0150129	BGIOSGA007026	XP_015696393.1 E3 ubiquitin-protein ligase UPL5
TCONS_00036239	0.321355	0.466727	0.678653	BGIOSGA037597	XP_006664103.1 probable prefoldin subunit 3
TCONS_00031053	0.321364	0.245655	0.859177	BGIOSGA032872	XP_015697426.1PREDICTED: uncharacterized protein C18orf8
TCONS_00006886	0.321784	1.56058	0.380606	BGIOSGA003306	XP_015689036.1 photosynthetic NDH subunit of lumenal location 4, chloroplastic
TCONS_00016369	0.321876	0.00195205	0.0612693	BGIOSGA014599	XP_015692199.1 probable isoaspartyl peptidase/L-asparaginase 3
TCONS_00016579	0.321941	-0.761575	-0.613672	BGIOSGA014390	XP_006652787.1 probable plastid-lipid-associated protein 8, chloroplastic
TCONS_00015258	0.32216	-3.5311	-0.726825	BGIOSGA013573	XP_008653318.2 ervatamin-B
TCONS_00002134	0.322408	0.119529	0.812147	BGIOSGA021762	XP_021313862.1ruvB-like protein 1
TCONS_00016988	0.322433	-1.68222	-0.738785	BGIOSGA019036	XP_002439135.2PGR5-like protein 1B, chloroplastic
TCONS_00020332	0.322564	2.02745	1.0692	BGIOSGA032057	XP_003564112.1STOREKEEPER protein
TCONS_00003874	0.322633	0.77509	1.31947	BGIOSGA022237	XP_006646034.1 peptidyl-prolyl cis-trans isomerase CYP57
TCONS_00030570	0.322838	1.02711	0.639672	BGIOSGA013292	XP_006659707.1 protein SRC2 homolog
TCONS_00010345	0.322944	0.0623112	0.887715	BGIOSGA020035	NP_001147667.1ATP-dependent protease Clp ATPase subunit
TCONS_00022700	0.322999	0.272014	0.135228	BGIOSGA006734	XP_004965642.1bZIP transcription factor RISBZ5
TCONS_00010220	0.323616	-2.89395	-1.70633	BGIOSGA014527	XP_006651457.1 phospho-2-dehydro-3-deoxyheptonate aldolase 1, chloroplastic
TCONS_00031523	0.323843	-0.103315	-0.374503	BGIOSGA025665	XP_006661985.1 probable glutathione S-transferase GSTU6
TCONS_00011463	0.323852	0.587232	0.598359	BGIOSGA010422	XP_006649291.1 tobamovirus multiplication protein 3-like isoform X1
TCONS_00024698	0.323954	0.0744921	0.736582	BGIOSGA009627	XP_006657522.1PREDICTED: uncharacterized protein LOC102704820 isoform X1
TCONS_00022560	0.324183	0.17741	-1.32715	BGIOSGA034480	XP_006657133.1 transcription factor TGA2-like isoform X1
TCONS_00026257	0.324266	1.03812	0.995421	BGIOSGA004347	XP_006659992.1 DNA-directed RNA polymerase III subunit RPC5 isoform X1
TCONS_00030483	0.324269	-1.72959	0.513938	BGIOSGA029325	XP_003578579.1probable flavin-containing monooxygenase 1
TCONS_00020329	0.324387	0.21565	1.56541	BGIOSGA029292	XP_003564116.1probable tyrosine-protein phosphatase DSP2
TCONS_00001412	0.324392	-0.0933329	-0.186919	BGIOSGA010862	XP_006644400.1 protein REDUCED WALL ACETYLATION 3-like
TCONS_00006212	0.324522	0.11927	0.237185	BGIOSGA016381	XP_004952670.12-Cys peroxiredoxin BAS1, chloroplastic
TCONS_00031127	0.324546	0.920161	0.572914	BGIOSGA011909	XP_010234743.1putative DNA helicase ino80
TCONS_00027245	0.32491	-0.9318	0.210448	BGIOSGA015524	XP_006659761.1 L-gulonolactone oxidase 2-like
TCONS_00011535	0.325036	0.302249	0.685645	BGIOSGA005200	XP_015690813.1 splicing factor U2af large subunit B-like
TCONS_00028304	0.325107	-1.0383	1.79272	BGIOSGA021183	XP_004973949.1methyltransferase-like protein 13
TCONS_00001383	0.325297	-0.0184381	-0.0579171	BGIOSGA004015	XP_004969201.1myosin IC heavy chain
TCONS_00007796	0.32584	0.888808	0.903962	BGIOSGA031388	XP_008663616.160S ribosomal protein L27-3
TCONS_00007095	0.325848	0.741067	0.190228	BGIOSGA011486	XP_010940715.1 60S ribosomal protein L23
TCONS_00023491	0.32588	0.760237	0.713039	BGIOSGA015575	XP_006658475.1 FBD-associated F-box protein At3g52670-like
TCONS_00035572	0.325978	0.727691	0.475755	BGIOSGA019906	XP_015698213.1 protein trichome birefringence-like 11
TCONS_00031467	0.32599	0.25798	0.832826	BGIOSGA029396	XP_006662514.1 small G protein signaling modulator 2
TCONS_00013154	0.326019	0.762201	0.215885	BGIOSGA028741	XP_006651806.2PREDICTED: uncharacterized protein LOC102710863
TCONS_00021758	0.326042	1.15082	1.03748	BGIOSGA004762	XP_003635231.2 ruvB-like 2
TCONS_00010806	0.326146	0.824952	0.547663	BGIOSGA013430	XP_006650485.2 protein CASP
TCONS_00007546	0.326512	0.724644	0.819693	BGIOSGA009859	XP_006647035.1 50S ribosomal protein L7/L12
TCONS_00029033	0.326512	-1.92506	-0.873486	BGIOSGA030785	XP_010914051.1 5'-nucleotidase domain-containing protein DDB_G0275467 isoform X2
TCONS_00014393	0.326656	0.341532	0.189398	BGIOSGA016548	XP_006652365.1 30S ribosomal protein 3, chloroplastic-like
TCONS_00010667	0.326714	0.0150567	0.37143	BGIOSGA013291	XP_015690009.1 GPI mannosyltransferase 1
TCONS_00027261	0.326921	0.603948	1.07133	BGIOSGA014855	XP_015695965.1PREDICTED: uncharacterized protein LOC102699707 isoform X2
TCONS_00034539	0.32705	0.690259	0.80235	BGIOSGA036490	XP_006662831.1 mRNA-capping enzyme-like
TCONS_00007858	0.327053	0.707914	0.585315	BGIOSGA014632	XP_006647202.1 myosin-13-like
TCONS_00026661	0.327314	-0.0488107	0.616834	BGIOSGA030749	XP_006660160.1PREDICTED: uncharacterized protein LOC102705837 isoform X1
TCONS_00007869	0.32737	0.256088	-0.658957	BGIOSGA006607	XP_006664943.1 ATP synthase mitochondrial F1 complex assembly factor 1
TCONS_00029070	0.327425	-1.14338	-0.867997	BGIOSGA000697	XP_006660653.1 ethylene-responsive transcription factor 1-like

TCONS_00012886	0.327483	-3.264	-0.53825	BGIOSGA000747	XP_015690864.1 transmembrane protein 56-B-like
TCONS_00014217	0.32756	-2.18754	-0.906415	BGIOSGA015690	XP_006652269.2 photosystem I subunit O
TCONS_00008830	0.327825	0.921293	1.16209	BGIOSGA024292	XP_006649062.1 7-deoxyloganetin glucosyltransferase-like
TCONS_00011212	0.327983	-0.598109	-0.396241	BGIOSGA004694	XP_004981292.1KH domain-containing protein SPIN1
TCONS_00036121	0.32836	1.68459	0.576405	BGIOSGA037480	XP_015698598.1PREDICTED: uncharacterized protein LOC107305428
TCONS_00030211	0.328562	0.417665	1.07384	BGIOSGA028876	NP_001123600.1methyl binding domain protein 117
TCONS_00029774	0.328736	0.865238	1.44788	BGIOSGA036597	NP_001105952.1plastid phosphate/phosphoenolpyruvate translocator 1
TCONS_00002176	0.328874	1.04303	-1.46419	BGIOSGA004812	XP_006646483.1 heme-binding-like protein At3g10130, chloroplastic
TCONS_00020981	0.328938	0.293973	-0.0846315	BGIOSGA022458	XP_006656207.1 26S protease regulatory subunit 8 homolog A-like
TCONS_00026936	0.328947	0.237743	1.58031	BGIOSGA028987	XP_006659582.1PREDICTED: uncharacterized protein LOC102712192
TCONS_00003880	0.328957	-0.521599	-1.63838	BGIOSGA001333	XP_004969401.1uncharacterized protein LOC101758396
TCONS_00008460	0.328971	1.5543	1.30605	BGIOSGA031623	XP_006647580.1 ALG-2 interacting protein X-like
TCONS_00004125	0.32919	0.458266	1.17425	BGIOSGA020372	XP_022682647.1CTD small phosphatase-like protein 2-A
TCONS_00027418	0.329358	0.945847	1.06758	BGIOSGA027587	XP_015696211.1 thyroid adenoma-associated protein homolog isoform X1
TCONS_00034705	0.329435	0.0424791	0.357007	BGIOSGA034128	XP_006662895.1 plant UBX domain-containing protein 11
TCONS_00001718	0.329648	0.521226	1.82593	BGIOSGA012000	XP_003569701.3probable serine acetyltransferase 1
TCONS_00002636	0.329877	0.840614	0.553068	BGIOSGA011835	XP_015688333.1 protein TRIGALACTOSYLDIACYLGLYCEROL 2, chloroplastic
TCONS_00029357	0.330003	0.563859	0.0975655	BGIOSGA012638	XP_006661447.1 probable 1-acylglycerol-3-phosphate O-acyltransferase
TCONS_00022181	0.330033	0.829717	0.432869	BGIOSGA021315	NP_001105880.1bundle sheath defective 2
TCONS_00031514	0.330047	0.544372	-0.280349	BGIOSGA018056	NP_001130938.1glutathione S-transferase GSTU6
TCONS_00032546	0.330068	0.777136	1.52572	BGIOSGA012688	XP_006661945.1 CDPK-related kinase 3-like
TCONS_00008513	0.330129	1.66763	0.793584	BGIOSGA037648	XP_015689401.1 ethylene-responsive transcription factor 1-like
TCONS_00011218	0.330354	-0.139946	0.121846	BGIOSGA013820	XP_006651955.1 SCAR-like protein 1 isoform X1
TCONS_00007580	0.330382	1.36921	0.508483	BGIOSGA029561	XP_004951506.1UDP-glycosyltransferase 73C1
TCONS_00021353	0.330439	1.62924	0.304961	BGIOSGA012552	XP_002437563.1protein RER1A
TCONS_00011253	0.330728	0.60223	0.414893	BGIOSGA013852	XP_006651977.1 protein DEFECTIVE IN EXINE FORMATION 1
TCONS_00011435	0.331137	-0.170963	-0.279077	#N/A	#N/A
TCONS_00020494	0.331207	0.30929	0.45032	BGIOSGA021039	XP_006655990.1 NAC domain-containing protein 8-like
TCONS_00013236	0.331474	0.160462	-0.222355	BGIOSGA009752	XP_002463825.130S ribosomal protein S9, chloroplastic
TCONS_00001689	0.331509	1.63011	0.42257	BGIOSGA010289	XP_006644609.2 glucan endo-1,3-beta-glucosidase GII-like
TCONS_00005729	0.331596	0.664264	0.323185	BGIOSGA007844	XP_015688526.1 U-box domain-containing protein 4-like
TCONS_00023149	0.331671	0.208748	0.284133	BGIOSGA037816	XP_006657459.2 ubiquitin carboxyl-terminal hydrolase 8
TCONS_00036855	0.332073	1.21362	0.602341	BGIOSGA029761	XP_015698243.1PREDICTED: uncharacterized protein ycf45
TCONS_00008168	0.332148	0.409251	0.577065	BGIOSGA006271	XP_004952824.1N-glycosylase/DNA lyase OGG1
TCONS_00010503	0.332189	0.095553	-0.093459	BGIOSGA013113	XP_015689969.1 serpin-ZXA
TCONS_00020330	0.332277	1.03416	1.08963	BGIOSGA013760	XP_015693491.1 lysM domain-containing GPI-anchored protein 1-like
TCONS_00029584	0.332306	0.782403	0.0908042	BGIOSGA012378	XP_006653073.1PREDICTED: uncharacterized protein LOC102708432
TCONS_00009056	0.332383	0.544834	1.06859	BGIOSGA005348	XP_006648130.2 DNL-type zinc finger protein
TCONS_00029579	0.33243	0.122093	1.23609	BGIOSGA030238	XP_006660418.1 pentatricopeptide repeat-containing protein At1g79540
TCONS_00000035	0.332467	0.961656	1.15636	BGIOSGA008896	XP_004967948.1RING-box protein 1
TCONS_00008806	0.332804	0.49443	0.00931866	BGIOSGA011170	XP_015689372.1 AP2-like ethylene-responsive transcription factor PLT1 isoform X1
TCONS_00033440	0.332949	1.51208	0.742193	BGIOSGA033757	XP_021317268.1formin-like protein 14
TCONS_00014171	0.333029	0.0701721	0.447963	BGIOSGA004332	XP_002446386.1probable alpha-amylase 2
TCONS_00009280	0.333294	-0.278531	-0.320265	BGIOSGA011819	XP_002468542.2antifreeze protein Maxi
TCONS_00033957	0.333519	0.335821	0.454545	BGIOSGA035647	XP_015698118.1 peptide chain release factor APG3, chloroplastic
TCONS_00034654	0.333539	-0.640665	0.4632	BGIOSGA034174	XP_015698100.1 general transcription factor 3C polypeptide 3 isoform X2
TCONS_00037128	0.334081	-0.0154108	-0.0126746	BGIOSGA036244	XP_006663983.2 fidgetin-like protein 1
TCONS_00015572	0.334088	-0.0978532	-0.194438	BGIOSGA015395	XP_015691395.1 metal tolerance protein C4
TCONS_00012330	0.334173	0.335113	-0.356528	BGIOSGA001986	XP_010228580.1probable transcription factor At3g04930
TCONS_00006524	0.334268	0.0844969	0.293766	BGIOSGA020754	XP_006647584.1 putative nuclear RNA export factor SDE5
TCONS_00006336	0.334355	0.553453	1.17869	BGIOSGA008499	XP_006648787.1PREDICTED: uncharacterized protein LOC102714988
TCONS_00013535	0.334438	0.439701	0.618271	BGIOSGA036998	XP_015690330.1 purple acid phosphatase 15
TCONS_00013202	0.334487	0.473654	0.573477	BGIOSGA009785	XP_006650611.1 transmembrane protein adipocyte-associated 1 homolog
TCONS_00004035	0.334504	0.0239518	0.756591	BGIOSGA035658	XP_006646132.1PREDICTED: uncharacterized protein LOC102721130

TCONS_00035716	0.334811	0.8815	0.413042	BGIOSGA035059	XP_015698239.1 CCR4-NOT transcription complex subunit 11-like
TCONS_00005200	0.334841	0.125505	0.54736	BGIOSGA000007	XP_006645413.1 2-methoxy-6-polyprenyl-1,4-benzoquinol methylase, mitochondrial
TCONS_00012474	0.334886	1.60203	1.21132	BGIOSGA010508	XP_006651510.1 erlin-2-B
TCONS_00022527	0.335242	-0.46942	0.485564	BGIOSGA013541	XP_006656217.1 SPX domain-containing protein 1
TCONS_00037232	0.335263	-0.40323	3.08131	BGIOSGA036142	XP_015698643.1 ureide permease 2-like
TCONS_00011081	0.335408	-0.296738	0.448831	BGIOSGA017683	XP_015690282.1 putative ALA-interacting subunit 2
TCONS_00014723	0.335423	-4.57336	-0.412131	BGIOSGA034174	XP_006652621.1 vesicle-associated protein 1-3-like isoform X1
TCONS_00003007	0.335451	1.55085	1.15992	BGIOSGA002210	XP_002455016.1uncharacterized protein LOC8084349 isoform X2
TCONS_00014501	0.335664	-0.0501984	0.55549	BGIOSGA031083	XP_006653555.1 probable E3 ubiquitin-protein ligase ARI8
TCONS_00025654	0.336064	0.704854	0.659748	BGIOSGA023771	XP_006658923.1 UDP-N-acetylglucosamine--dolichyl-phosphate N-acetylglucosaminephosphotransferase
TCONS_00019806	0.336168	0.305984	0.558209	BGIOSGA001991	XP_003567883.1uncharacterized protein LOC100843260
TCONS_00007428	0.33628	1.18044	0.9183	BGIOSGA007051	XP_015689365.1 urease accessory protein D-like
TCONS_00011097	0.336432	0.396141	-0.73232	BGIOSGA004651	XP_015690101.1 nuclear pore complex protein NUP1-like
TCONS_00023335	0.33645	-0.174727	-1.05411	BGIOSGA024059	XP_006657552.1 remorin-like
TCONS_00019019	0.336505	1.3384	0.587507	BGIOSGA034703	XP_015693109.1 ADP,ATP carrier protein 1, mitochondrial-like
TCONS_00033600	0.336532	1.60846	1.37453	BGIOSGA035386	XP_002449623.1uncharacterized protein LOC8064827
TCONS_00029570	0.336691	-1.11553	-0.585226	BGIOSGA030248	XP_015696498.1 DNA repair protein RAD51 homolog 4
TCONS_00037167	0.336795	0.262916	0.707822	BGIOSGA036204	XP_002442120.1uncharacterized protein LOC8063244
TCONS_00032872	0.337171	-0.359807	-0.120425	BGIOSGA036903	XP_015698516.1 bis(5'-adenosyl)-triphosphatase-like
TCONS_00018776	0.337237	-0.649653	-1.24555	BGIOSGA029380	XP_006654137.1 transcription factor MYB1R1-like
TCONS_00004656	0.337402	-3.57586	-0.586999	BGIOSGA000560	XP_006644941.1 GDSL esterase/lipase At1g58430-like
TCONS_00006485	0.337402	1.12458	0.413001	BGIOSGA005545	XP_002452476.1phosphoenolpyruvate carboxylase kinase 2
TCONS_00024974	0.337467	-1.57594	-0.00219693	BGIOSGA024420	XP_022680243.1fatty acyl-CoA reductase 1
TCONS_00006504	0.337677	-0.68945	0.183899	BGIOSGA005983	XP_006647560.1 ADP-ribosylation factor GTPase-activating protein AGD2-like isoform X2
TCONS_00005697	0.337769	-0.109138	-0.149475	BGIOSGA008052	XP_015696777.1 peroxisomal nicotinamide adenine dinucleotide carrier-like
TCONS_00016056	0.337838	-2.40234	-1.75393	BGIOSGA006169	XP_015691951.1PREDICTED: uncharacterized protein LOC107304097 isoform X2
TCONS_00009608	0.337867	0.112942	0.314293	BGIOSGA012153	XP_015691167.1 nuclear pore complex protein NUP133 isoform X2
TCONS_00030357	0.338458	-0.522556	0.164322	BGIOSGA029442	XP_002454783.2GTPase-activating protein gyp1 isoform X2
TCONS_00004044	0.338616	-3.57739	-3.17498	BGIOSGA005767	XP_006644410.1 epoxide hydrolase 3-like
TCONS_00002397	0.338702	-0.83229	-2.03698	BGIOSGA012162	XP_006645210.1 thioredoxin F, chloroplastic
TCONS_00009702	0.338764	0.345666	0.545723	BGIOSGA012252	XP_006649761.1 protein FAR1-RELATED SEQUENCE 9-like
TCONS_00023381	0.338913	0.0484571	0.153101	BGIOSGA025419	XP_006657577.1 cytochrome b5 domain-containing protein RLF
TCONS_00000001	0.338974	0.905451	0.546049	BGIOSGA025839	XP_006643619.1 small G protein signaling modulator 1-like isoform X2
TCONS_00003659	0.339077	2.11659	1.09422	BGIOSGA019679	XP_006645934.1 universal stress protein A-like protein
TCONS_00032648	0.339087	-1.56053	-0.410139	BGIOSGA031306	XP_024316763.1short-chain dehydrogenase TIC 32, chloroplastic
TCONS_00035628	0.339258	0.743726	0.215743	BGIOSGA017393	XP_004977309.1uncharacterized protein LOC101776555
TCONS_00032544	0.339363	1.37586	1.77736	BGIOSGA031573	XP_004986438.1protein ROOT PRIMORDIUM DEFECTIVE 1
TCONS_00006711	0.33953	2.97604	0.992676	BGIOSGA035907	XP_006647748.1 pentatricopeptide repeat-containing protein OTP51, chloroplastic
TCONS_00037871	0.339662	0.361451	-0.51623	#N/A	#N/A
TCONS_00037405	0.339915	-0.565794	-1.10445	BGIOSGA020656	XP_006664128.1PREDICTED: uncharacterized protein LOC102704577
TCONS_00030501	0.340052	0.268795	-1.50047	BGIOSGA013557	XP_006661515.1 SRSF protein kinase 1
TCONS_00016319	0.340173	0.0840158	0.128131	BGIOSGA033901	XP_006652582.1 vacuolar-processing enzyme beta-isozyme 1
TCONS_00032727	0.340267	-0.469728	-1.7389	BGIOSGA031378	XP_006662080.1 chlorophyllide a oxygenase, chloroplastic
TCONS_00005280	0.34039	0.998641	1.28005	BGIOSGA007387	XP_015692420.1 signal peptide peptidase 2-like
TCONS_00034737	0.340623	0.611604	0.842434	BGIOSGA035180	XP_006664993.1 protein KTI12 homolog
TCONS_00008878	0.340876	1.22712	1.65231	BGIOSGA009227	XP_006647971.2 protein CPR-5-like
TCONS_00005577	0.340995	0.373809	0.626481	BGIOSGA003815	XP_006646990.1 ent-cassadiene C2-hydroxylase-like
TCONS_00015992	0.341024	-0.183265	0.286222	BGIOSGA006203	XP_006652350.1 ubiquitin carboxyl-terminal hydrolase 4-like
TCONS_00004222	0.341036	-0.354052	-0.051022	BGIOSGA002747	NP_001151105.2DNA-binding protein RAV1
TCONS_00036635	0.341241	0.0631607	0.403481	BGIOSGA007345	XP_004978608.1U3 small nucleolar ribonucleoprotein protein MPP10
TCONS_00023361	0.341426	0.259236	0.149667	BGIOSGA006569	XP_004955819.1ADP-ribosylation factor 1
TCONS_00015588	0.341559	-0.251772	0.443227	BGIOSGA006506	XP_006652162.1 oligoribonuclease-like isoform X2
TCONS_00017877	0.341674	0.234952	0.254818	BGIOSGA032837	NP_001149606.1beta-hydroxyacyl-ACP dehydratase

TCONS_00032418	0.341678	-1.99628	-1.01263	BGIOSGA037430	XP_002464494.2long-chain-alcohol oxidase FAO1
TCONS_00005529	0.34181	0.951642	0.265291	BGIOSGA030264	XP_015689581.1 choline-phosphate cytidylyltransferase 2
TCONS_00035001	0.34225	0.406396	0.415344	BGIOSGA020728	XP_006662971.2 probable Xaa-Pro aminopeptidase P
TCONS_00023692	0.342335	-1.17247	-1.13504	BGIOSGA025724	XP_015695036.1 vacuolar protein sorting-associated protein 32 homolog 1-like
TCONS_00009549	0.342341	0.225279	-0.37684	BGIOSGA012095	XP_006649615.1 serine-rich adhesin for platelets-like
TCONS_00019085	0.342528	-1.2049	0.477618	BGIOSGA026577	XP_006659637.1 transcription factor bHLH137-like isoform X1
TCONS_00006115	0.342533	0.218113	0.736286	BGIOSGA008271	XP_002452138.1chromatin assembly factor 1 subunit A
TCONS_00015607	0.342789	1.20878	0.762103	BGIOSGA023600	XP_003579141.2protein WHAT'S THIS FACTOR 1
TCONS_00012122	0.342902	0.17664	0.74585	BGIOSGA010888	XP_015690884.1 PH-interacting protein-like
TCONS_00023166	0.343221	0.452076	0.65186	BGIOSGA025205	XP_006658307.1 proline--tRNA ligase, chloroplastic/mitochondrial
TCONS_00019357	0.343289	-0.523554	-0.012163	BGIOSGA009334	XP_003568383.1embryogenesis-associated protein EMB8
TCONS_00024145	0.343436	0.204686	-0.728764	BGIOSGA026182	XP_006657982.1 dnaJ-like protein MG002 homolog
TCONS_00013265	0.343669	-0.793695	-0.920979	BGIOSGA009722	XP_004981558.1photosystem I reaction center subunit III, chloroplastic
TCONS_00032684	0.343781	-0.958454	-0.181927	BGIOSGA031423	XP_002464938.1metalloendoproteinase 1-MMP
TCONS_00020003	0.343846	0.0569604	-0.453207	BGIOSGA033068	XP_015694371.1 protein ENHANCED DISEASE RESISTANCE 2-like
TCONS_00037377	0.343848	0.937577	0.590813	BGIOSGA035987	XP_003578816.1UNC93-like protein 3 isoform X2
TCONS_00005875	0.344478	-0.00776067	0.194722	BGIOSGA031736	XP_015689419.1 WD repeat-containing protein 26
TCONS_00010001	0.34483	-0.417903	0.212962	BGIOSGA012553	XP_004984501.1protein PHOSPHATE STARVATION RESPONSE 1
TCONS_00007015	0.344836	0.593909	0.518252	BGIOSGA017615	XP_006649128.1 flocculation protein FLO11-like
TCONS_00009268	0.344904	0.066271	-0.653844	BGIOSGA011805	XP_006649359.1PREDICTED: uncharacterized protein LOC102714990
TCONS_00018484	0.344907	0.690053	0.704508	BGIOSGA018949	NP_001278564.1RAB5-interacting protein
TCONS_00014894	0.344921	0.741858	0.522761	BGIOSGA017075	XP_014661073.1nuclear pore complex protein NUP88
TCONS_00025645	0.344926	0.154637	0.657359	BGIOSGA023781	XP_006658063.1 ankyrin repeat domain-containing protein 2A
TCONS_00017532	0.345133	1.98353	1.37763	BGIOSGA019605	XP_006654255.1 protein SSUH2 homolog
TCONS_00030026	0.345306	0.736134	0.69918	BGIOSGA007539	XP_006661212.1 dihydrolipoyllysine-residue acetyltransferase component 4 of pyruvate dehydrogenase complex, chloroplastic-like
TCONS_00009700	0.34544	-1.03924	-3.72499	BGIOSGA012250	XP_015689835.1PREDICTED: uncharacterized protein LOC102699852
TCONS_00030279	0.345443	-2.20463	-1.17419	BGIOSGA029528	XP_006660780.1 cinnamoyl-CoA reductase 1-like
TCONS_00026161	0.345785	-0.394176	-0.656075	#N/A	#N/A
TCONS_00019180	0.346171	-0.586885	-0.388566	BGIOSGA018173	XP_006654324.2 vesicle-associated protein 2-2-like
TCONS_00036646	0.346204	1.04533	0.494617	BGIOSGA034527	XP_006664317.2 cytochrome c-type biogenesis ccda-like chloroplastic protein 1
TCONS_00007968	0.346324	-1.36648	-0.862876	BGIOSGA006513	XP_015699051.1 extracellular ribonuclease-like
TCONS_00031205	0.346425	2.53421	1.53963	BGIOSGA008907	XP_021307373.1heavy metal-associated isoprenylated plant protein 3 isoform X2
TCONS_00036141	0.346533	0.281416	0.610539	BGIOSGA037497	XP_006664068.1 pentatricopeptide repeat-containing protein At4g02820, mitochondrial
TCONS_00018904	0.346637	-1.6516	-1.71165	BGIOSGA003204	XP_003566408.1probable glucan 1,3-beta-glucosidase A
TCONS_00013431	0.346736	0.495479	0.500705	BGIOSGA009553	XP_006650807.1 zinc finger protein GIS2-like
TCONS_00009790	0.346852	1.32951	0.824321	BGIOSGA030551	XP_006649859.1 histone H2B.1
TCONS_00017221	0.346863	0.603523	1.40825	BGIOSGA019261	XP_006648138.1 mediator of RNA polymerase II transcription subunit 36a-like
TCONS_00010967	0.346865	-0.0692713	-0.299037	BGIOSGA013567	XP_006650594.2 transmembrane protein 256 homolog isoform X2
TCONS_00017928	0.347051	0.517222	0.553049	BGIOSGA013615	XP_006655373.1 protein YIPF1 homolog
TCONS_00005643	0.347188	-0.687715	-0.633361	BGIOSGA020935	XP_006647058.1 serine-threonine kinase receptor-associated protein
TCONS_00026054	0.347443	0.511045	0.607662	BGIOSGA028120	XP_003565163.1protein PIN-LIKES 2
TCONS_00030393	0.347494	-0.754973	-0.325272	BGIOSGA016943	XP_015696720.1 RHOMBOID-like protein 2
TCONS_00023720	0.347549	-0.112177	-1.90654	BGIOSGA001476	XP_006658584.2PREDICTED: uncharacterized protein LOC102703050
TCONS_00001771	0.347688	0.488369	-2.78983	BGIOSGA022272	XP_006644666.1 anthocyanidin 5,3-O-glucosyltransferase-like
TCONS_00022949	0.347709	0.728523	0.554269	BGIOSGA020603	XP_006657370.2 photosystem II stability/assembly factor HCF136, chloroplastic
TCONS_00018918	0.347829	1.23497	0.67134	BGIOSGA023065	XP_006654194.1 xylanase inhibitor protein 2-like
TCONS_00032432	0.347847	-0.912252	-0.489168	#N/A	#N/A
TCONS_00037574	0.347856	-0.309163	-0.523834	BGIOSGA035785	XP_006664228.1 haloacid dehalogenase-like hydrolase domain-containing protein At3g48420
TCONS_00027979	0.347975	1.23186	1.60569	BGIOSGA028490	XP_015695438.1 elongation factor Ts, mitochondrial
TCONS_00005208	0.347995	-0.519561	-0.464589	BGIOSGA035799	XP_006655669.1 cysteine synthase-like
TCONS_00007884	0.348011	-0.417466	0.186639	BGIOSGA016888	XP_006648577.1PREDICTED: uncharacterized protein LOC102704397
TCONS_00006648	0.348174	-0.702622	-0.425946	BGIOSGA016977	XP_015689660.1 mechanosensitive ion channel protein 1, mitochondrial-like isoform X1
TCONS_00036782	0.348182	0.458487	0.712896	BGIOSGA006268	XP_004977222.1probable L-ascorbate peroxidase 6, chloroplastic/mitochondrial
TCONS_00030373	0.348427	-0.0768179	0.741078	#N/A	#N/A

TCONS_00017335	0.348514	0.0882225	0.0738437	BGIOSGA016391	XP_006654159.2 protein trichome birefringence-like 26
TCONS_00022863	0.349034	0.0261681	-0.637936	BGIOSGA035632	XP_006657312.1PREDICTED: uncharacterized protein LOC102707132
TCONS_00024588	0.349157	0.0744515	0.304875	BGIOSGA024805	XP_004955476.1uncharacterized protein LOC101765325
TCONS_00008046	0.349168	0.562265	-0.242119	BGIOSGA015216	XP_006647277.1 F-box/kelch-repeat protein At1g67480
TCONS_00020996	0.34922	0.188873	0.311564	BGIOSGA006926	XP_015693629.1 calcium-binding mitochondrial carrier protein SCaMC-1-like
TCONS_00008366	0.349286	0.683431	0.312025	BGIOSGA006059	XP_006647502.1 nuclear cap-binding protein subunit 2
TCONS_00026778	0.349343	-0.359909	-0.0377469	BGIOSGA028841	XP_003574622.1DPH4 homolog
TCONS_00031636	0.34938	0.917355	0.503749	BGIOSGA014859	XP_015697362.1 peptide methionine sulfoxide reductase A4, chloroplastic-like
TCONS_00025881	0.349732	0.215523	0.716839	BGIOSGA027951	XP_006659804.2 tryptophan synthase beta chain 1
TCONS_00008646	0.34991	0.201229	0.377282	BGIOSGA001973	XP_015689621.1 syntaxin-22-like isoform X2
TCONS_00017494	0.349954	0.526444	0.314607	BGIOSGA021467	XP_006655994.1 signal peptidase complex catalytic subunit SEC11A-like
TCONS_00032863	0.350153	-0.651271	-0.851321	BGIOSGA023430	XP_010931361.1 RING-H2 finger protein ATL74-like isoform X1
TCONS_00014513	0.350161	0.117018	0.152241	BGIOSGA016676	XP_006650349.1 protein IQ-DOMAIN 1-like
TCONS_00025425	0.350307	-0.0973744	-0.00554128	BGIOSGA001298	XP_015694887.1 A-kinase anchor protein 17A
TCONS_00003933	0.350463	-0.134838	-0.0494437	BGIOSGA001585	XP_006644394.2 cytochrome P450 72A15-like
TCONS_00018368	0.350527	0.748659	0.483909	BGIOSGA022897	XP_015692729.1 mitogen-activated protein kinase 17
TCONS_00011427	0.350652	0.40342	0.610321	BGIOSGA013953	XP_015690169.1 transcription factor BTF3 homolog 4-like isoform X2
TCONS_00016229	0.350705	0.356092	0.708535	BGIOSGA014745	XP_015692232.1 coiled-coil domain-containing protein 115 isoform X1
TCONS_00034302	0.350808	1.18869	0.260469	BGIOSGA035008	XP_006663205.2 pyruvate kinase 1, cytosolic-like
TCONS_00009671	0.351139	-0.230728	-0.991551	BGIOSGA012925	XP_015691224.1 calmodulin-lysine N-methyltransferase isoform X2
TCONS_00021804	0.351172	0.385613	-0.249786	BGIOSGA005340	XP_006655891.1 serine/threonine-protein kinase At5g01020-like
TCONS_00001715	0.351225	-0.431222	-0.978474	BGIOSGA023481	XP_006646268.1 fe-S cluster assembly factor HCF101, chloroplastic
TCONS_00030672	0.351301	0.877915	-0.0669002	BGIOSGA020112	XP_006662164.1PREDICTED: uncharacterized protein LOC102721866
TCONS_00017727	0.351392	0.926319	0.141829	BGIOSGA019789	XP_004962205.150S ribosomal protein L1, chloroplastic
TCONS_00017723	0.351494	0.996164	0.464266	BGIOSGA019783	XP_006654347.1 UDP-sulfoquinovose synthase, chloroplastic
TCONS_00023332	0.351586	0.427115	0.494872	BGIOSGA009436	XP_015698937.1 CTD small phosphatase-like protein 2
TCONS_00004726	0.351601	0.50566	-0.407752	BGIOSGA031780	XP_006645011.1 ras-related protein Rab2BV-like
TCONS_00002742	0.351631	1.54889	1.75675	BGIOSGA002484	XP_006643706.1 Bowman-Birk type bran trypsin inhibitor-like
TCONS_00003124	0.351695	-0.543713	-0.347284	BGIOSGA032855	XP_004977297.1GDSL esterase/lipase At5g45910
TCONS_00007386	0.351716	0.220507	1.14026	BGIOSGA007090	XP_015688463.1 protein SCO1 homolog 1, mitochondrial
TCONS_00000137	0.351889	-1.40159	-0.67151	BGIOSGA017615	XP_008673758.1uncharacterized protein LOC103649836
TCONS_00032520	0.352133	-1.2938	-1.87629	BGIOSGA018754	XP_006662494.1 remorin-like
TCONS_00035895	0.352165	-1.10298	0.172282	BGIOSGA037266	XP_002439072.1exosome complex component RRP42
TCONS_00002892	0.352406	-0.53065	-0.195166	BGIOSGA002326	XP_006643787.1PREDICTED: uncharacterized protein LOC102702045
TCONS_00020305	0.352729	0.954899	0.811804	BGIOSGA022162	XP_002444829.1UMP-CMP kinase 4
TCONS_00016754	0.352881	-0.380628	0.063057	BGIOSGA010709	XP_013461038.1alpha-N-acetylglucosaminidase family protein
TCONS_00011593	0.353021	-0.00175647	0.00563691	BGIOSGA028036	XP_015690235.1PREDICTED: uncharacterized protein LOC102713811
TCONS_00019194	0.353157	0.875583	1.76981	BGIOSGA020646	XP_006654336.2 probable methyltransferase PMT7
TCONS_00019689	0.353308	1.3876	1.64696	BGIOSGA035205	XP_006655522.1 pentatricopeptide repeat-containing protein At3g49240-like
TCONS_00017920	0.353345	-1.81847	0.172831	BGIOSGA016983	XP_006655371.2PREDICTED: uncharacterized protein LOC102709370
TCONS_00033870	0.353539	1.07502	2.19157	BGIOSGA005093	XP_006663062.1 E3 ubiquitin-protein ligase At3g02290-like
TCONS_00004916	0.353582	0.33663	0.627905	BGIOSGA015052	XP_004970961.1peptidyl serine alpha-galactosyltransferase
TCONS_00009732	0.353779	-0.441914	0.371512	BGIOSGA024992	XP_006649799.1 sulfoquinovosyl transferase SQD2-like
TCONS_00019043	0.353924	0.650514	1.14678	BGIOSGA000043	XP_006645341.1 small nuclear ribonucleoprotein Sm D2-like
TCONS_00010012	0.354534	0.332027	-0.192711	BGIOSGA012565	XP_006650040.1 eukaryotic translation initiation factor 2 subunit beta
TCONS_00024552	0.354543	-0.671941	0.493847	BGIOSGA024839	XP_015695045.1 classical arabinogalactan protein 9-like
TCONS_00010171	0.354831	1.36276	1.56033	BGIOSGA033535	XP_015690754.1 protein LTV1 homolog isoform X1
TCONS_00032768	0.354851	1.60806	0.468664	BGIOSGA031342	XP_015697495.1PREDICTED: uncharacterized protein LOC102706507
TCONS_00014695	0.355006	-0.300569	0.178879	BGIOSGA008737	XP_006652603.1 ubiquitin carboxyl-terminal hydrolase 3-like
TCONS_00035170	0.35509	-0.405641	0.168471	BGIOSGA036456	XP_006663043.1 putative 14-3-3-like protein GF14-H
TCONS_00027612	0.355722	0.841488	1.08443	BGIOSGA003844	NP_001344639.1putative WRKY DNA-binding domain superfamily protein

TCONS_00012014	0.355737	1.3703	0.551524	BGIOSGA010994	XP_006651253.1 probable ADP-ribosylation factor GTPase-activating protein AGD6
TCONS_00019738	0.355751	0.178487	0.640757	BGIOSGA035267	XP_015692813.1 lipid phosphate phosphatase 2-like
TCONS_00034288	0.355753	0.354567	0.753591	BGIOSGA036646	XP_006663811.1 suppressor of mec-8 and unc-52 protein homolog 2
TCONS_00035890	0.355818	1.03656	1.41913	#N/A	YP_008815731.1photosystem II protein D1 (plastid)
TCONS_00022716	0.355892	0.531946	0.903441	BGIOSGA020733	XP_006656336.2 tRNA pseudouridine synthase A
TCONS_00002952	0.355916	-0.669099	-0.061923	BGIOSGA002265	XP_015699174.1 aspartate aminotransferase
TCONS_00037311	0.35592	0.691373	1.58736	BGIOSGA036058	XP_006664608.1 rRNA-processing protein FYV7
TCONS_00032199	0.35628	1.34774	0.865949	BGIOSGA013329	XP_015697237.1 golgin subfamily A member 5
TCONS_00001580	0.356343	-0.387074	1.04117	BGIOSGA004213	XP_006644529.1 katanin p60 ATPase-containing subunit A1
TCONS_00019391	0.356398	0.486096	0.536662	BGIOSGA004842	NP_001169059.1uncharacterized LOC100382900
TCONS_00029026	0.356483	0.265988	0.00629398	BGIOSGA030779	XP_006660637.2 pentatricopeptide repeat-containing protein At4g30825, chloroplastic
TCONS_00011530	0.356556	1.12494	0.236377	BGIOSGA011500	XP_015691268.1 ankyrin repeat domain-containing protein, chloroplastic
TCONS_00025374	0.356911	-0.028942	-0.307118	BGIOSGA004562	XP_006658713.1 zinc-metallopeptidase, peroxisomal-like
TCONS_00023878	0.357107	-1.03895	-0.890992	BGIOSGA024132	XP_021307823.1putative F-box/FBD/LRR-repeat protein At4g03220
TCONS_00014985	0.357125	-0.0296969	0.25652	BGIOSGA028198	XP_006652854.1 BTB/POZ and MATH domain-containing protein 2-like
TCONS_00023765	0.357158	-0.0804611	1.14614	BGIOSGA025804	XP_006658611.1 reactive Intermediate Deaminase A, chloroplastic
TCONS_00026534	0.357162	0.319666	-0.19925	BGIOSGA028601	XP_003578003.1sulfite exporter TauE/SafE family protein 3
TCONS_00029302	0.357233	0.176619	0.385536	BGIOSGA031045	XP_010235251.1FHA domain-containing protein FHA2 isoform X2
TCONS_00010065	0.357578	1.5685	1.63622	BGIOSGA021015	XP_021307383.1metal tolerance protein 2 isoform X1
TCONS_00007837	0.357624	1.39852	1.27765	BGIOSGA006646	XP_015688961.1 putative aminoacrylate hydrolase RutD
TCONS_00024988	0.357654	1.29157	1.12146	BGIOSGA006781	XP_006653311.1PREDICTED: uncharacterized protein LOC102709459
TCONS_00023615	0.357727	-0.480049	0.0330208	BGIOSGA003090	XP_006657669.1 serine/threonine-protein kinase 19
TCONS_00004399	0.357744	-0.211829	0.293835	BGIOSGA004398	NP_001315573.1uncharacterized protein LOC103637583
TCONS_00026261	0.358011	0.163349	0.842642	BGIOSGA028346	XP_015695755.1 nuclear nucleic acid-binding protein C1D
TCONS_00002843	0.358101	0.529163	0.798423	BGIOSGA024911	XP_002459031.2uncharacterized protein LOC8059402
TCONS_00000147	0.358186	2.58594	0.28364	BGIOSGA002720	XP_006643685.1 protein SAR DEFICIENT 1-like
TCONS_00007058	0.35865	0.124516	0.479396	BGIOSGA009239	XP_003570347.1quinone oxidoreductase PIG3
TCONS_00013419	0.358832	-0.993961	-0.263833	BGIOSGA018257	XP_002463649.1CASP-like protein 4B3
TCONS_00036511	0.358856	-0.369652	-1.09999	BGIOSGA037874	XP_006664795.1 carotenoid 9,10(9',10')-cleavage dioxygenase
TCONS_00006996	0.359103	1.05951	0.331077	BGIOSGA033418	XP_006648167.1 eukaryotic translation initiation factor 3 subunit G-like
TCONS_00011510	0.359181	0.203701	-0.817415	BGIOSGA011517	XP_006649340.2 probable signal recognition particle 43 kDa protein, chloroplastic
TCONS_00015800	0.359249	0.865508	0.965935	BGIOSGA023659	XP_003581163.1histone H2A.Z-specific chaperone CHZ1
TCONS_00018387	0.359336	-1.69916	-3.7806	BGIOSGA020439	XP_015693060.1 solanesyl-diphosphate synthase 2, chloroplastic
TCONS_00028838	0.35942	0.334029	0.0115782	BGIOSGA001311	NP_001152515.1holocarboxylase synthetase
TCONS_00014074	0.359511	0.286417	0.085288	BGIOSGA027847	XP_015691690.1 TBC1 domain family member 13-like
TCONS_00031584	0.359575	2.16458	1.40359	BGIOSGA033399	XP_003574288.1cytochrome c-type biogenesis protein CcmE homolog, mitochondrial
TCONS_00024583	0.35958	0.130238	2.19147	#N/A	#N/A
TCONS_00000921	0.359587	-0.152362	0.793152	BGIOSGA002497	XP_004968116.1uncharacterized protein LOC101778809
TCONS_00003303	0.359629	0.764853	0.175284	BGIOSGA000812	XP_006644047.1 protein DELETION OF SUV3 SUPPRESSOR 1(I)-like
TCONS_00024321	0.359709	1.86495	0.750652	BGIOSGA022241	XP_004981248.160S ribosome subunit biogenesis protein NIP7 homolog
TCONS_00001855	0.359933	0.61801	0.208936	BGIOSGA017730	XP_015689932.1PREDICTED: LOW QUALITY PROTEIN: uncharacterized protein LOC102718611
TCONS_00005423	0.36004	1.03998	0.29554	BGIOSGA020588	XP_006646879.1 eukaryotic initiation factor 4A-3
TCONS_00002819	0.360044	0.115746	-0.0710075	BGIOSGA029371	XP_020406279.1triosephosphate isomerase, cytosolic-like
TCONS_00013486	0.360102	0.0855509	0.202446	BGIOSGA035847	XP_015691098.1PREDICTED: uncharacterized protein LOC102699671
TCONS_00027781	0.360243	-0.424199	0.60245	BGIOSGA018688	XP_010234573.1phosphatidylinositol 3-kinase, root isoform isoform X2
TCONS_00030241	0.360258	0.0561493	-0.0971637	BGIOSGA021366	XP_006661365.2 putative BPI/LBP family protein At1g04970
TCONS_00018136	0.360263	0.193319	0.597687	BGIOSGA020195	XP_006654651.1 protein LAZ1 homolog 1
TCONS_00036419	0.360357	1.74916	0.648676	BGIOSGA037030	XP_004976150.1suppressor protein SRP40
TCONS_00000127	0.360379	0.232594	0.199444	BGIOSGA013643	XP_015688259.1 endonuclease 2-like isoform X2
TCONS_00006676	0.360656	-0.0757437	0.402824	BGIOSGA001618	XP_006647712.1 acetylornithine deacetylase
TCONS_00014737	0.360862	0.303428	-0.054556	BGIOSGA016914	XP_015692265.1 15-cis-phytoene desaturase-like
TCONS_00013047	0.361081	2.3095	0.341635	BGIOSGA009939	XP_006651738.1 ACD11 homolog protein-like

TCONS_00015959	0.36124	0.393802	0.656599	BGIOSGA015025	XP_006652321.1PREDICTED: uncharacterized protein LOC102719198
TCONS_00012688	0.361422	-0.306361	0.440902	BGIOSGA014711	XP_015689966.1PREDICTED: uncharacterized protein LOC102711143
TCONS_00010336	0.361576	1.11711	1.05188	BGIOSGA012911	XP_008673581.1RAP domain-containing protein, chloroplastic
TCONS_00007249	0.361635	-0.00550621	0.14513	BGIOSGA028356	XP_006646793.1 syntaxin-52-like isoform X2
TCONS_00013467	0.361665	0.218031	0.495749	BGIOSGA012897	XP_015690368.1 acylpyruvase FAHD1, mitochondrial-like isoform X1
TCONS_00006372	0.361815	-1.10052	-0.797469	BGIOSGA008535	XP_021315408.1uncharacterized protein LOC8056473
TCONS_00029338	0.361819	-0.824312	0.393383	BGIOSGA031086	XP_006660842.1 protein farnesyltransferase/geranylgeranyltransferase type-1 subunit alpha
TCONS_00022171	0.361845	1.50164	0.699504	BGIOSGA001610	XP_002456895.1cytochrome P450 89A2
TCONS_00030725	0.362137	1.066	0.938053	BGIOSGA027162	XP_006659312.1 pre-mRNA-splicing factor ATP-dependent RNA helicase DEAH1-like
TCONS_00036626	0.362149	-1.84828	-1.37216	BGIOSGA025257	XP_006662713.1 protein ZINC INDUCED FACILITATOR-LIKE 1-like isoform X2
TCONS_00020180	0.362545	-3.71751	-1.008	BGIOSGA037599	XP_006655820.1PREDICTED: uncharacterized protein LOC102712638
TCONS_00010361	0.36262	0.570472	0.73521	BGIOSGA009538	XP_006650818.1 calmodulin-binding protein 60 A-like
TCONS_00004655	0.362674	2.03119	1.14372	BGIOSGA020037	XP_006644938.1 PRA1 family protein B2-like
TCONS_00004592	0.363132	0.409583	1.021	BGIOSGA000623	XP_015690212.1 ribonuclease III domain-containing protein RNC1, chloroplastic-like
TCONS_00030428	0.363293	-1.89095	-1.14494	BGIOSGA020533	XP_006661478.2 endoglucanase 24
TCONS_00013567	0.363343	-0.388396	0.166323	BGIOSGA029356	XP_006650928.1 acyl carrier protein 3, chloroplastic-like
TCONS_00037314	0.363445	0.366428	1.68571	BGIOSGA036054	XP_003575718.1tryptophan--tRNA ligase, cytoplasmic
TCONS_00016972	0.363685	-0.217848	-0.0693057	BGIOSGA005261	XP_015693045.1 transcription initiation factor IIA subunit 2
TCONS_00029608	0.364489	-1.03793	0.416701	BGIOSGA016636	XP_015692047.1 phospholipid-transporting ATPase 2-like isoform X3
TCONS_00009975	0.364781	0.647658	0.543769	BGIOSGA035269	XP_006650012.1 ELMO domain-containing protein A
TCONS_00027893	0.364905	1.73738	0.14936	BGIOSGA001657	XP_020407331.1co-chaperone protein SBA1 isoform X1
TCONS_00031531	0.364927	1.44266	1.15646	BGIOSGA011914	XP_003574248.1protein SAR DEFICIENT 4
TCONS_00024916	0.364991	0.558933	1.02225	BGIOSGA036017	XP_002461847.1F-box/LRR-repeat protein At3g26922
TCONS_00024608	0.365299	-0.875879	-3.35086	BGIOSGA026659	XP_003561407.13-oxo-5-alpha-steroid 4-dehydrogenase 2
TCONS_00008900	0.365627	0.388145	0.204767	BGIOSGA005509	XP_006647989.1 replication factor C subunit 3
TCONS_00023844	0.365718	0.532134	0.718742	BGIOSGA014818	XP_015694663.1 putative cysteine-rich receptor-like protein kinase 20
TCONS_00014345	0.365718	-0.467866	-0.203256	BGIOSGA015726	XP_004975780.1hepatoma-derived growth factor-related protein 2
TCONS_00009003	0.365748	-0.0031937	1.42542	BGIOSGA005404	XP_015688812.1 mRNA-decapping enzyme subunit 2
TCONS_00002616	0.366253	0.650457	1.32252	BGIOSGA021775	XP_015699275.1 BTB/POZ domain-containing protein FBL11-like
TCONS_00010163	0.366677	-2.51586	-1.59514	BGIOSGA009326	XP_015690516.1 cytochrome P450 709B2-like
TCONS_00002730	0.367437	2.65768	0.676777	BGIOSGA002492	XP_004968098.1uncharacterized protein LOC101772564
TCONS_00029395	0.368025	0.467274	1.00358	BGIOSGA031147	XP_006660889.2 putative ER lumen protein-retaining receptor C28H8.4
TCONS_00019975	0.36805	1.15924	0.449851	BGIOSGA022501	XP_015694252.1 UMP-CMP kinase 1
TCONS_00005686	0.368067	0.834278	0.893221	BGIOSGA001366	XP_021314660.1reticulocalbin-2
TCONS_00023649	0.368075	0.0147264	0.163641	BGIOSGA013557	XP_006658550.1 cyclin-dependent kinase-like 5
TCONS_00018148	0.368179	1.38483	1.25551	BGIOSGA020206	XP_022683244.1protein DJ-1 homolog C
TCONS_00005541	0.368179	0.554754	0.640177	BGIOSGA028219	XP_006646956.1PREDICTED: uncharacterized protein LOC102708028
TCONS_00009625	0.368335	-0.393979	0.0224264	BGIOSGA012170	XP_006649670.1 AP-3 complex subunit sigma
TCONS_00017191	0.368511	-0.767522	-3.84321	BGIOSGA018005	XP_006664988.1 probable GTP diphosphokinase CRSH2, chloroplastic
TCONS_00014135	0.368775	-0.486753	-0.70497	BGIOSGA001690	XP_010239812.1uncharacterized protein LOC100842192
TCONS_00023247	0.368881	-0.822544	0.193979	BGIOSGA025287	XP_006657514.1 C-Myc-binding protein homolog
TCONS_00006257	0.368948	-0.77601	-1.46886	BGIOSGA017850	XP_006647367.1 ultraviolet-B receptor UVR8
TCONS_00020443	0.36896	1.38385	-0.456914	BGIOSGA026660	XP_003564002.1U-box domain-containing protein 21
TCONS_00024957	0.369095	0.266203	0.808808	BGIOSGA020321	XP_010234546.1protein ROOT INITIATION DEFECTIVE 3 isoform X1
TCONS_00015299	0.369126	0.202069	0.168361	BGIOSGA007209	XP_006652076.1PREDICTED: uncharacterized protein LOC102712157
TCONS_00025196	0.369251	-1.27756	-0.776415	BGIOSGA011826	XP_003563024.1uncharacterized protein LOC100822827
TCONS_00011161	0.369435	1.42094	0.896181	BGIOSGA035491	XP_006650753.1 T-complex protein 1 subunit theta
TCONS_00037537	0.369456	-0.146793	0.618932	BGIOSGA036358	XP_006664207.1 50S ribosomal protein HLP, mitochondrial-like
TCONS_00011123	0.369793	0.59224	0.282051	BGIOSGA013723	XP_006650723.1 PITH domain-containing protein At3g04780
TCONS_00030699	0.369836	-0.191109	-0.2154	BGIOSGA032519	XP_015697123.1 queuine tRNA-ribosyltransferase subunit QTRTD1-like
TCONS_00018159	0.369894	-2.12516	-0.435254	BGIOSGA000856	XP_015693376.1PREDICTED: uncharacterized protein LOC102704983
TCONS_00009412	0.369897	-0.223818	-0.529746	BGIOSGA008412	XP_006649489.1 solute carrier family 25 member 44
TCONS_00021859	0.3699	-0.358412	-0.444818	BGIOSGA021660	XP_006656775.1 kinesin-like protein KCA2
TCONS_00028004	0.370043	0.436933	0.724833	BGIOSGA026983	XP_022683489.1disease resistance protein RPM1

TCONS_00031885	0.370077	0.33907	0.289564	BGIOSGA011113	XP_006661652.1 autophagy-related protein 9-like
TCONS_00011833	0.370387	0.948401	-0.112516	BGIOSGA013906	XP_002468217.2protein DETOXIFICATION 48
TCONS_00004556	0.370549	0.334163	0.615588	BGIOSGA022661	XP_006646405.1 phosphatidylglycerophosphatase GEP4, mitochondrial
TCONS_00025853	0.370762	0.699656	0.457581	BGIOSGA001680	XP_006659792.1PREDICTED: uncharacterized protein LOC102703606, partial
TCONS_00024467	0.370823	0.308838	0.917759	BGIOSGA019351	XP_004981678.1probable protein phosphatase 2C 34
TCONS_00031197	0.371235	0.372885	1.08942	BGIOSGA033017	XP_015697104.1 UPF0434 protein BRADO0313
TCONS_00031479	0.371396	-0.323032	-1.00595	BGIOSGA008355	XP_003558973.1uncharacterized protein LOC100824638 isoform X1
TCONS_00019723	0.371416	-1.7519	-0.921107	BGIOSGA037227	XP_006654732.1 farnesyl pyrophosphate synthase-like
TCONS_00021695	0.371661	-0.442052	0.725974	BGIOSGA006959	XP_012700848.1putative wall-associated receptor kinase-like 16
TCONS_00030188	0.371706	0.134398	-0.0327408	BGIOSGA004651	XP_006661329.2 serine/threonine-protein kinase PEPKR2
TCONS_00024029	0.372246	-0.531032	0.464345	BGIOSGA026068	XP_010228353.1protein UXT homolog
TCONS_00009229	0.372347	-0.57453	-0.448766	BGIOSGA007676	XP_004985894.1uncharacterized protein LOC101767597
TCONS_00033338	0.372467	-0.585725	-1.03354	BGIOSGA021587	XP_015697638.1 cycloeucalenol cycloisomerase
TCONS_00019047	0.372549	0.11065	0.526084	BGIOSGA018313	XP_006654254.1 putative zinc transporter At3g08650
TCONS_00007299	0.372727	0.143286	-0.720416	BGIOSGA023314	XP_015689573.1 ocs element-binding factor 1-like
TCONS_00009815	0.372732	0.654528	1.05487	BGIOSGA006416	XP_015690810.1 DNA topoisomerase 6 subunit A3
TCONS_00014624	0.372808	-0.0760038	0.456927	BGIOSGA016780	XP_015692197.1 psbQ-like protein 3, chloroplastic
TCONS_00015990	0.372877	0.24158	-0.411523	BGIOSGA024249	XP_006652347.1 probable protein phosphatase 2C 41
TCONS_00007167	0.372937	-0.647289	0.876919	BGIOSGA009355	XP_006649212.1 metal tolerance protein C2
TCONS_00028960	0.372991	-0.176222	0.85338	BGIOSGA030716	XP_006661200.1 cleavage and polyadenylation specificity factor subunit 3-II
TCONS_00027624	0.373026	1.2264	0.944318	BGIOSGA032931	XP_004973039.1putative glucose-6-phosphate 1-epimerase
TCONS_00012013	0.373172	0.501356	0.639464	BGIOSGA008351	NP_001145994.1THO complex subunit 4
TCONS_00008361	0.373332	0.310121	0.244073	BGIOSGA028752	XP_006647497.1 histone-lysine N-methyltransferase ASHR3-like
TCONS_00004882	0.373632	-1.21127	-0.325535	BGIOSGA000337	XP_006645158.2PREDICTED: uncharacterized protein LOC102705963 isoform X2
TCONS_00023987	0.37385	-1.21909	-0.482799	BGIOSGA030130	XP_015697304.1PREDICTED: uncharacterized protein LOC102708470
TCONS_00033294	0.373939	1.38883	0.833161	BGIOSGA001875	XP_006410289.1uncharacterized protein LOC18026827
TCONS_00023346	0.373974	-0.316005	-0.342475	BGIOSGA013794	XP_006658392.1 protein phosphatase 2C 70-like
TCONS_00008862	0.374174	1.47646	0.293324	BGIOSGA032685	XP_004954029.1bZIP transcription factor 23
TCONS_00035314	0.374305	-0.0859937	-0.313232	BGIOSGA001697	XP_024319288.1putative disease resistance RPP13-like protein 3
TCONS_00010119	0.374479	-0.0132461	1.01885	BGIOSGA022743	XP_015689905.1PREDICTED: uncharacterized protein HI_0077, partial
TCONS_00014852	0.37451	-1.10605	-2.79279	BGIOSGA000550	XP_006652729.1 S-norcoclaurine synthase-like
TCONS_00004617	0.374757	-0.959473	-1.37208	BGIOSGA033715	XP_006644898.1PREDICTED: uncharacterized protein LOC102701395
TCONS_00001807	0.375144	-3.2871	0.332716	BGIOSGA021597	XP_006644693.2 kinesin-4-like
TCONS_00034422	0.375205	-1.2097	-0.802292	BGIOSGA007095	XP_015697578.1 atherin-like
TCONS_00037463	0.375277	0.157982	0.89957	BGIOSGA000260	XP_015698352.1PREDICTED: LOW QUALITY PROTEIN: uncharacterized protein LOC102704583, partial
TCONS_00034382	0.375331	-0.10502	0.284264	BGIOSGA036555	XP_022685187.1filament-like plant protein 6
TCONS_00017856	0.375365	0.552711	0.353526	BGIOSGA000406	XP_015693142.1PREDICTED: uncharacterized protein LOC102716412
TCONS_00005691	0.375389	-0.62497	-0.895864	BGIOSGA027900	XP_015688520.1PREDICTED: uncharacterized protein LOC102709626
TCONS_00030340	0.375472	0.436084	0.408718	BGIOSGA012282	XP_015696777.1 peroxisomal nicotinamide adenine dinucleotide carrier-like
TCONS_00025470	0.375624	-0.257208	-0.786096	BGIOSGA023960	XP_006657917.1 filamin-A-interacting protein 1
TCONS_00006442	0.37576	0.465749	0.250201	BGIOSGA016698	XP_006647495.1 S-adenosylmethionine decarboxylase proenzyme-like
TCONS_00012018	0.375769	0.172815	-0.706759	BGIOSGA025323	XP_015690783.1 ATP synthase subunit b', chloroplastic
TCONS_00009852	0.375868	0.811452	-0.0377523	BGIOSGA012402	XP_015689856.1PREDICTED: LOW QUALITY PROTEIN: uncharacterized protein LOC102716306
TCONS_00009509	0.37593	-0.954678	0.492911	BGIOSGA005677	XP_004985315.1ADP-ribosylation factor-like protein 8a
TCONS_00014974	0.375945	-0.120963	0.654127	BGIOSGA025165	XP_006652849.1 peroxisomal (S)-2-hydroxy-acid oxidase GLO3
TCONS_00028728	0.375972	-0.452258	-0.636242	BGIOSGA040512	XP_006660520.1 ocs element-binding factor 1-like
TCONS_00022679	0.376095	0.82665	0.177603	BGIOSGA010489	XP_012489481.1 protein transport protein Sec61 subunit gamma-like
TCONS_00024787	0.376398	-0.439231	-0.922199	BGIOSGA024582	XP_015694571.1 uncharacterized aarF domain-containing protein kinase At5g05200, chloroplastic
TCONS_00004729	0.376419	0.0815461	0.573179	BGIOSGA000486	XP_015694193.1PREDICTED: uncharacterized protein LOC102715912, partial
TCONS_00009727	0.376775	-0.387026	-0.0220082	BGIOSGA012276	XP_004984700.1probable BOI-related E3 ubiquitin-protein ligase 2
TCONS_00027049	0.376888	0.93411	0.124458	BGIOSGA029101	XP_006660346.1 la-related protein 6B-like

TCONS_00002755	0.3771	0.897922	0.759463	BGIOSGA000527	XP_015699286.1 metal tolerance protein 7 isoform X1
TCONS_00019676	0.377222	0.306265	1.08125	BGIOSGA010774	XP_006654693.1 SNF1-related protein kinase catalytic subunit alpha KIN10-like
TCONS_00007723	0.377509	-0.0127471	0.675083	BGIOSGA002048	XP_006644252.1 40S ribosomal protein S15a-5-like
TCONS_00023678	0.377524	-0.627671	0.191906	BGIOSGA025703	XP_006658560.1 spindle and kinetochore-associated protein 1 homolog
TCONS_00024611	0.377545	-1.33682	-2.32646	BGIOSGA014206	XP_003561404.1probable carboxylesterase 15
TCONS_00012099	0.377859	1.54846	-0.198414	BGIOSGA012477	XP_015694032.1 wound-induced protein 1-like
TCONS_00025885	0.377921	-0.186263	0.336299	BGIOSGA014508	XP_006659807.1 actin-related protein 4
TCONS_00027539	0.377988	-0.635122	-1.51218	BGIOSGA013060	XP_006650520.1PREDICTED: uncharacterized protein LOC102721532
TCONS_00031634	0.378234	-0.561454	0.215672	BGIOSGA007108	XP_006662067.2PREDICTED: uncharacterized protein LOC102712849
TCONS_00037009	0.378448	1.11494	0.688051	BGIOSGA036363	XP_006664497.1 protein RETICULATA-RELATED 1, chloroplastic
TCONS_00028839	0.378598	-0.671772	0.409409	BGIOSGA007771	XP_021310476.1ABC transporter A family member 7 isoform X3
TCONS_00016458	0.378639	-0.288043	-0.740483	BGIOSGA014513	XP_006652677.1 uncharacterized metal-dependent hydrolase YcfH isoform X2
TCONS_00032945	0.378774	0.448303	0.527994	BGIOSGA016783	XP_006662727.1 two-component response regulator ORR9
TCONS_00032142	0.378899	0.631104	0.914743	BGIOSGA031996	XP_006661739.2 translation initiation factor eIF-2B subunit beta
TCONS_00009187	0.378976	-0.629505	0.18829	BGIOSGA016943	XP_006649269.1 RHOMBOID-like protein 2
TCONS_00016294	0.379	1.64348	1.18823	BGIOSGA014676	XP_008661938.1hus1-like protein isoform X1
TCONS_00012555	0.379138	-0.257465	1.26023	BGIOSGA011563	XP_004982553.1tobamovirus multiplication protein 1
TCONS_00029433	0.379174	0.0956138	0.709011	BGIOSGA031182	XP_022680422.1nitrate regulatory gene2 protein
TCONS_00028715	0.379512	0.197822	-0.274374	BGIOSGA008875	XP_004956514.1protein PHR1-LIKE 2 isoform X1
TCONS_00004164	0.379656	0.113146	0.352877	BGIOSGA010730	XP_015699224.1 tubby-like F-box protein 1
TCONS_00018286	0.37967	0.467923	0.140153	BGIOSGA001022	XP_004975378.160S ribosomal protein L37a-1
TCONS_00008837	0.38008	-0.622843	-0.357553	BGIOSGA005579	XP_006647940.1 peptidyl-prolyl cis-trans isomerase FKBP12
TCONS_00004851	0.380172	-0.705013	-1.00732	BGIOSGA037885	NP_001278609.160S ribosomal protein L37
TCONS_00000450	0.380312	-1.69883	-1.67794	BGIOSGA004417	XP_006643925.1 aluminum-activated malate transporter 9-like
TCONS_00017902	0.380585	0.0252268	-0.415662	BGIOSGA036996	XP_021303686.1protein BUD31 homolog 2
TCONS_00008244	0.380766	0.158831	-0.0942775	BGIOSGA006185	XP_006647415.2 photosynthetic NDH subunit of lumenal location 2, chloroplastic
TCONS_00027387	0.380906	2.0685	1.73809	BGIOSGA025102	XP_006659146.1 nucleolar GTP-binding protein 1
TCONS_00036038	0.380916	0.837606	1.85436	BGIOSGA034839	XP_006664024.1 cyclin-T1-4
TCONS_00001733	0.381056	-0.245678	0.761015	BGIOSGA020215	XP_015689773.1 hexokinase-9-like
TCONS_00006655	0.381109	-0.573298	-0.910109	BGIOSGA033362	XP_024318180.1tobamovirus multiplication protein 3
TCONS_00028100	0.381571	-0.0571124	1.30135	#N/A	#N/A
TCONS_00020431	0.381725	-4.80289	-0.316022	BGIOSGA005652	XP_010227794.1TORTIFOLIA1-like protein 3
TCONS_00035441	0.381764	-0.485748	-0.374725	BGIOSGA012069	XP_015697977.1 macrophage migration inhibitory factor homolog isoform X1
TCONS_00027689	0.381905	-0.851672	-0.558017	BGIOSGA036343	XP_015695554.1 F-box protein SKIP1-like
TCONS_00027863	0.382031	0.449523	0.960495	BGIOSGA030678	XP_015696042.1 bifunctional aspartokinase/homoserine dehydrogenase 1, chloroplastic isoform X1
TCONS_00023172	0.382126	-0.0971218	0.405466	BGIOSGA025213	XP_006657480.1 glutamine-dependent NAD(+) synthetase
TCONS_00001551	0.382304	0.525021	0.264314	BGIOSGA017567	XP_015699331.1 protein DEHYDRATION-INDUCED 19 homolog 3
TCONS_00019451	0.382373	1.37817	1.00729	BGIOSGA000540	XP_012700237.1nuclear transcription factor Y subunit B
TCONS_00023057	0.382476	-2.35784	-5.26383	BGIOSGA036164	XP_006657422.1 pathogenesis-related protein 1-like
TCONS_00005915	0.382639	0.548857	1.298	BGIOSGA000814	XP_006647204.1 OPA3-like protein
TCONS_00022914	0.382692	2.0754	0.51229	BGIOSGA026730	XP_006657350.1 endoplasmin homolog
TCONS_00015577	0.38272	-1.9623	-2.52391	BGIOSGA031696	XP_015691398.1 transcription factor bHLH35-like
TCONS_00011693	0.38272	0.35963	-0.938944	BGIOSGA032820	XP_015690955.1 protein CutA 1, chloroplastic-like isoform X2
TCONS_00009978	0.382755	0.571367	0.987924	BGIOSGA001902	XP_006650013.1 mitochondrial import receptor subunit TOM40-1-like
TCONS_00005810	0.382859	0.291612	0.0662072	BGIOSGA007931	XP_006647140.2 pentatricopeptide repeat-containing protein At3g16010
TCONS_00006536	0.382889	0.464145	0.411683	BGIOSGA022733	XP_004953271.1auxin-responsive protein SAUR36
TCONS_00002162	0.383003	-1.85731	-0.837439	BGIOSGA017926	XP_015690384.1 ultraviolet-B receptor UVR8
TCONS_00024631	0.383179	0.155918	0.0299315	BGIOSGA026295	XP_006657481.1 BTB/POZ and MATH domain-containing protein 4
TCONS_00029994	0.383454	-0.123615	-0.475811	BGIOSGA009908	XP_015696821.1 caffeoylshikimate esterase-like isoform X2
TCONS_00035827	0.383494	0.75633	0.867145	BGIOSGA034483	XP_006664468.1 pre-mRNA-splicing factor CWC22 homolog
TCONS_00031671	0.383523	-0.6805	-0.865361	BGIOSGA033489	XP_006662622.1 uncharacterized sugar kinase slr0537
TCONS_00001575	0.38353	1.83205	2.81707	BGIOSGA008934	XP_006644523.2 transcription initiation factor TFIID subunit 11
TCONS_00016351	0.383561	0.195948	0.901015	BGIOSGA017330	XP_006653633.2PREDICTED: uncharacterized protein LOC102721360
TCONS_00022729	0.38377	0.110511	0.104632	BGIOSGA005526	XP_006656347.1 haloacid dehalogenase-like hydrolase domain-containing protein 3
TCONS_00012717	0.383847	0.710729	0.56685	BGIOSGA030118	XP_006651600.2 50S ribosomal protein L25
TCONS_00009877	0.383943	0.537317	0.359834	BGIOSGA033025	XP_006649937.1 26S protease regulatory subunit 4 homolog

TCONS_00023485	0.38404	0.544705	0.635208	BGIOSGA024764	XP_006657613.2 putative aldehyde oxidase-like protein
TCONS_00029932	0.384059	-0.00693014	0.869413	BGIOSGA030347	XP_010238122.1serine/threonine-protein kinase 16 isoform X1
TCONS_00006515	0.3841	-0.0190648	0.271894	BGIOSGA008682	XP_002452499.1E2F-associated phosphoprotein
TCONS_00012475	0.384193	-0.459569	0.287997	BGIOSGA010504	XP_015689037.1 UDP-N-acetylglucosamine transferase subunit ALG14
TCONS_00017658	0.384257	0.597634	-0.107852	BGIOSGA014723	XP_006654314.1 beta-glucosidase 22-like
TCONS_00036645	0.384708	1.13686	0.465468	BGIOSGA036663	XP_006664974.1 probable calcium-binding protein CML49
TCONS_00011007	0.38522	-0.238638	0.489729	BGIOSGA026231	XP_006650635.2 peroxidase 51-like
TCONS_00035479	0.385747	-0.251179	0.179781	BGIOSGA029663	XP_006663754.1 cytochrome P450 714C3
TCONS_00013060	0.385764	0.239733	0.491222	BGIOSGA009923	XP_002463995.1NADH dehydrogenase [ubiquinone] iron-sulfur protein 1, mitochondrial
TCONS_00034183	0.385876	0.085629	0.825132	BGIOSGA036749	XP_006663163.2PREDICTED: uncharacterized protein LOC102714866
TCONS_00011153	0.38597	1.6071	1.04103	BGIOSGA009545	XP_004981386.1AAA-ATPase ASD, mitochondrial
TCONS_00019159	0.386079	-0.0884614	-0.015042	BGIOSGA002565	XP_020394134.1uncharacterized protein LOC100279052
TCONS_00031488	0.386115	0.311681	1.00212	BGIOSGA033301	XP_006662527.1 flavonol synthase/flavanone 3-hydroxylase-like
TCONS_00007806	0.386193	0.167986	0.811426	BGIOSGA012058	XP_021307558.140S ribosomal protein S3a-like
TCONS_00018370	0.386283	0.448009	0.59677	BGIOSGA020421	XP_015692791.1 peroxisomal membrane protein PEX14-like
TCONS_00023865	0.386405	-1.60585	-0.111	BGIOSGA013744	NP_001311123.1oxygen-evolving enhancer protein 3-1, chloroplastic
TCONS_00005781	0.386418	1.01793	1.21088	BGIOSGA002990	XP_015689451.1 transcription factor GTE4-like isoform X2
TCONS_00012068	0.386452	0.624686	0.15385	BGIOSGA010940	XP_006649912.1 ran-binding protein 1 homolog a-like
TCONS_00033896	0.386639	0.290431	0.25005	BGIOSGA009740	XP_019704954.1 40S ribosomal protein S29
TCONS_00012417	0.386657	0.549878	1.31982	BGIOSGA016276	XP_006650175.1PREDICTED: uncharacterized protein LOC102715926
TCONS_00022447	0.386757	0.296272	0.780763	BGIOSGA004310	XP_006656174.1 V-type proton ATPase subunit B 1
TCONS_00006759	0.386896	1.70722	0.702265	BGIOSGA008929	XP_003570252.1uncharacterized protein LOC100840317
TCONS_00025656	0.387233	1.2393	0.737888	BGIOSGA023767	XP_006658926.1 DDT domain-containing protein PTM
TCONS_00012314	0.387259	-0.195384	0.852073	BGIOSGA010682	XP_006650108.2PREDICTED: uncharacterized protein LOC102719468 isoform X1
TCONS_00036244	0.387574	-0.471441	0.332923	BGIOSGA025511	XP_006664107.1 psbP domain-containing protein 1, chloroplastic
TCONS_00030453	0.3876	1.2487	-0.325377	BGIOSGA029347	XP_006661489.2 trafficking protein particle complex subunit 2-like protein
TCONS_00031253	0.387744	0.876094	0.927488	BGIOSGA031079	XP_003573997.1uncharacterized protein LOC100821421
TCONS_00030126	0.387751	0.535781	0.483449	BGIOSGA014013	XP_006661281.2 probable receptor-like protein kinase At5g56460
TCONS_00003784	0.3879	-0.400947	0.629344	BGIOSGA001427	NP_001136640.1putative dynein light chain type 1 family protein
TCONS_00025175	0.387922	1.37714	-2.12832	BGIOSGA033880	NP_001105805.2adenosine 5'-phosphosulfate reductase-like 2
TCONS_00012996	0.387929	1.28907	1.45747	BGIOSGA013731	XP_006651721.1 putative ABC1 protein At2g40090
TCONS_00021919	0.388819	-0.402103	0.211994	BGIOSGA009038	XP_006656813.1 NEP1-interacting protein-like 2 isoform X2
TCONS_00011579	0.388884	0.482339	1.59034	BGIOSGA011436	XP_015690900.1 probable acetyltransferase NATA1-like
TCONS_00010367	0.389132	0.923091	0.445259	BGIOSGA020331	XP_006650233.1 somatic embryogenesis receptor kinase 2-like
TCONS_00018122	0.389331	1.11902	0.162031	BGIOSGA008253	XP_015693271.1 probable magnesium transporter NIPA3 isoform X2
TCONS_00026643	0.389479	0.581109	0.847152	BGIOSGA030697	XP_010235145.1probable ubiquitin receptor RAD23 isoform X1
TCONS_00005930	0.389523	-0.468492	-0.373957	BGIOSGA017912	XP_006647222.1 coatomer subunit zeta-2
TCONS_00016098	0.38968	-0.452735	0.303596	BGIOSGA006132	XP_004975964.1beta-1,4-mannosyl-glycoprotein 4-beta-N-acetylglucosaminyltransferase
TCONS_00034010	0.38994	0.676766	-0.910545	BGIOSGA011893	XP_014758377.1cell number regulator 13
TCONS_00006855	0.390007	0.271174	0.371954	BGIOSGA031387	XP_006647876.1 RING-H2 finger protein ATL46-like
TCONS_00030433	0.390212	-1.30559	-0.28732	BGIOSGA000096	XP_006660892.1PREDICTED: uncharacterized protein LOC102700085
TCONS_00024268	0.390422	-0.876784	-0.347076	BGIOSGA026312	XP_006658059.2 XIAP-associated factor 1-like
TCONS_00016000	0.390469	0.599932	0.121371	BGIOSGA010841	XP_006652356.1 endoplasmic reticulum-Golgi intermediate compartment protein 3-like
TCONS_00029304	0.390605	0.0616824	0.444921	BGIOSGA031047	XP_006660812.1 ribulose-phosphate 3-epimerase, cytoplasmic isoform
TCONS_00015828	0.390624	-0.762488	-0.486819	BGIOSGA015158	XP_015692103.1 probable protein phosphatase 2C 39
TCONS_00018265	0.390665	-0.0266673	0.337374	BGIOSGA020320	XP_021909473.1 zinc finger protein BRUTUS-like
TCONS_00030146	0.390701	-4.59251	-0.892516	BGIOSGA029656	XP_006660676.1 65-kDa microtubule-associated protein 6-like
TCONS_00013030	0.390712	-0.00383688	0.349686	BGIOSGA030742	NP_001149034.1protein binding protein
TCONS_00009174	0.390723	0.887132	0.558984	BGIOSGA033284	XP_004982595.1protein HLB1
TCONS_00019675	0.391054	0.459998	0.412744	BGIOSGA004390	NP_001144109.1uncharacterized protein LOC100276949
TCONS_00026642	0.391129	0.0298079	0.215482	BGIOSGA028710	XP_006659420.1 protein FLX-like 3
TCONS_00032834	0.391146	1.4263	0.558412	BGIOSGA019707	XP_006664864.2PREDICTED: uncharacterized protein LOC102701411

TCONS_00001535	0.391377	-0.329561	-2.25749	BGIOSGA004168	XP_006644485.1PREDICTED: uncharacterized protein LOC102720836
TCONS_00035736	0.3914	0.133394	0.593601	BGIOSGA037145	XP_006663906.1PREDICTED: uncharacterized protein LOC102706056
TCONS_00035528	0.391547	-0.405553	-0.242288	BGIOSGA034745	XP_006662718.1 L-aminoadipate-semialdehyde dehydrogenase-phosphopantetheinyl transferase-like
TCONS_00005005	0.391598	-1.56369	-0.832777	BGIOSGA023942	XP_004971136.1probable glucuronosyltransferase Os01g0926400
TCONS_00032078	0.391644	0.539167	0.77571	BGIOSGA001915	XP_006661720.1 glutamate--tRNA ligase, cytoplasmic-like
TCONS_00005546	0.391781	-0.903009	-0.901637	BGIOSGA023993	XP_006646961.1 probable 4-coumarate--CoA ligase 3
TCONS_00025174	0.391818	1.43986	-0.489402	BGIOSGA012454	NP_001147200.1peptidase S16, lon
TCONS_00037418	0.391887	0.545946	0.155688	BGIOSGA017205	XP_015698713.1 probable galacturonosyltransferase 13
TCONS_00019369	0.391946	1.61284	-0.962023	BGIOSGA017824	XP_010025545.1 histone H3.2
TCONS_00005141	0.392149	0.190823	-0.259708	BGIOSGA000062	XP_006645355.1 ubiquitin-fold modifier 1
TCONS_00005406	0.392232	1.00494	0.360419	BGIOSGA019170	XP_006646874.1 sucrose-phosphatase 2
TCONS_00004101	0.392463	0.140606	-0.507441	BGIOSGA001037	XP_008672795.1uncharacterized LOC100280571 isoform X1
TCONS_00027037	0.392806	0.0623419	0.543072	BGIOSGA018109	XP_006660342.1 probable RNA methyltransferase At5g51130
TCONS_00036208	0.393133	-0.462989	0.501265	BGIOSGA037484	XP_009629077.1 splicing regulatory glutamine/lysine-rich protein 1 isoform X1
TCONS_00029909	0.3936	-0.855227	-1.15832	BGIOSGA014631	XP_006660573.1 putative disease resistance protein RGA3
TCONS_00019542	0.393789	0.603219	0.734635	BGIOSGA004666	XP_012485277.1 proteasome subunit alpha type-3 isoform X2
TCONS_00011032	0.393892	0.357846	-0.385997	BGIOSGA037725	XP_015691165.1 BTB/POZ domain-containing protein At3g50780-like
TCONS_00022161	0.39394	0.085329	0.375344	BGIOSGA021337	XP_015693562.1 UPF0061 protein AZOSEA38000
TCONS_00008603	0.393953	1.05645	1.02975	BGIOSGA005823	XP_017188122.1 UPF0160 protein-like
TCONS_00027487	0.393955	-0.0966006	-0.173618	BGIOSGA027522	XP_004972671.1cysteine-rich PDZ-binding protein
TCONS_00023116	0.394005	-3.70133	0.258602	BGIOSGA025154	XP_006657444.1 187-kDa microtubule-associated protein AIR9
TCONS_00000113	0.394011	1.67617	0.995239	BGIOSGA002683	XP_015699325.1 66 kDa stress protein
TCONS_00007829	0.39405	1.09013	0.613322	BGIOSGA008309	XP_006647188.1 protein PAT1 homolog 1-like
TCONS_00024654	0.394165	-0.0218319	0.142629	BGIOSGA024734	XP_015694810.1 putative nuclease HARBI1
TCONS_00011948	0.394271	0.240494	0.291538	BGIOSGA011064	XP_006649787.1 eukaryotic translation initiation factor NCBP
TCONS_00005539	0.394369	0.764599	1.14892	BGIOSGA020743	XP_015688959.1 basic leucine zipper 9-like isoform X1
TCONS_00026006	0.39459	1.61809	1.09808	BGIOSGA029358	XP_003573418.1CRS2-associated factor 1, mitochondrial
TCONS_00009001	0.394634	0.994586	0.183184	BGIOSGA005406	XP_015689592.1PREDICTED: uncharacterized protein LOC102711234
TCONS_00014111	0.394691	0.133133	0.955646	BGIOSGA008281	XP_015691424.1PREDICTED: uncharacterized protein LOC102715299
TCONS_00014845	0.394758	0.677046	-0.289095	BGIOSGA017022	XP_003580216.1succinate dehydrogenase subunit 7, mitochondrial
TCONS_00036145	0.394849	1.03959	0.87504	BGIOSGA034413	XP_010228950.1uncharacterized protein LOC100821645
TCONS_00039931	0.395041	-0.525434	0.228204	BGIOSGA030524	XP_015696539.1 disease resistance protein RGA2-like
TCONS_00002766	0.395103	-1.3906	0.383624	BGIOSGA026793	XP_021310925.1mediator of RNA polymerase II transcription subunit 22a
TCONS_00014253	0.395187	-3.11058	-0.903403	BGIOSGA019333	XP_015691455.1 FAD-dependent urate hydroxylase-like
TCONS_00029027	0.395376	0.0591077	0.458702	BGIOSGA021591	XP_006661247.1 probable folate-biopterin transporter 6
TCONS_00032551	0.395585	0.136847	0.19515	BGIOSGA006748	XP_015696913.1 cytochrome P450 84A1
TCONS_00014867	0.395609	-0.220331	0.365164	BGIOSGA020224	XP_015692257.1 wall-associated receptor kinase 1-like
TCONS_00004458	0.395713	-0.376406	0.302716	BGIOSGA000763	XP_006644758.1 glutamate 5-kinase isoform X1
TCONS_00023483	0.395745	0.842915	0.680182	BGIOSGA013564	XP_006665056.2 4-coumarate--CoA ligase-like 7
TCONS_00033029	0.395898	-0.778044	-0.323491	BGIOSGA034870	XP_010237909.1cytochrome b-c1 complex subunit 6
TCONS_00035544	0.395903	0.111823	0.254514	BGIOSGA035053	XP_006663187.1 PP2A regulatory subunit TAP46-like
TCONS_00030170	0.395951	1.04778	0.337375	BGIOSGA026820	XP_015696379.1 probable BRI1 kinase inhibitor 1
TCONS_00031098	0.396704	0.93135	-0.0795419	BGIOSGA032916	XP_015697107.1 chitinase 2-like
TCONS_00011226	0.396816	0.650986	0.415643	BGIOSGA013827	XP_006650799.1 leukotriene A-4 hydrolase homolog
TCONS_00030455	0.397091	0.0931362	0.0445561	BGIOSGA028543	XP_006660915.2 probable 26S proteasome non-ATPase regulatory subunit 3
TCONS_00017444	0.397134	-0.584777	-1.15419	BGIOSGA040651	XP_006653251.1 type 1 phosphatases regulator YPI1
TCONS_00036823	0.397236	0.587288	0.166833	BGIOSGA013351	XP_006663896.1 mRNA-capping enzyme-like
TCONS_00012324	0.397555	0.481099	1.34569	BGIOSGA010670	XP_015690977.1 CST complex subunit STN1
TCONS_00019108	0.39785	0.690498	0.935964	BGIOSGA018253	XP_015693218.1 dehydration-responsive element-binding protein 2B-like isoform X4
TCONS_00029755	0.397975	-0.0856892	1.0652	BGIOSGA020411	XP_006647104.2 ycf20-like protein
TCONS_00036653	0.398082	0.506443	0.0195156	BGIOSGA012020	XP_010238743.1L-galactono-1,4-lactone dehydrogenase 2, mitochondrial
TCONS_00000033	0.398174	-0.272654	-0.312302	BGIOSGA027211	XP_006645429.1 protein RER1A-like
TCONS_00021013	0.398588	0.787017	0.230105	BGIOSGA036735	XP_006656230.1 vacuolar protein sorting-associated protein 32 homolog 2
TCONS_00027116	0.398592	0.402899	1.00636	BGIOSGA029160	XP_015696061.1 WD repeat-containing protein DWA2
TCONS_00000222	0.398618	-2.82572	-0.84145	BGIOSGA005340	XP_014660807.1glutathione hydrolase 3

TCONS_00007061	0.398824	0.579028	0.349435	BGIOSGA002075	XP_006648080.1 long chain base biosynthesis protein 1c
TCONS_00011563	0.398864	0.796511	1.37667	BGIOSGA033054	XP_015688185.1 methyltransferase-like protein 1
TCONS_00023966	0.398932	0.856647	-0.710686	BGIOSGA025997	XP_004958119.1probable homogentisate phytyltransferase 2, chloroplastic
TCONS_00035016	0.399031	-1.84919	-0.348361	BGIOSGA033839	XP_015692222.1 protein MRG1-like isoform X1
TCONS_00032913	0.39922	-1.46445	-1.06017	BGIOSGA034745	XP_006662718.1 L-aminoadipate-semialdehyde dehydrogenase-phosphopantetheinyl transferase-like
TCONS_00002488	0.399514	0.0432846	0.872835	BGIOSGA029376	XP_006645302.1 RNA-binding protein BRN1-like
TCONS_00017994	0.39958	1.52404	2.0131	BGIOSGA007951	XP_006655406.1 TOM1-like protein 2
TCONS_00019702	0.399668	-0.487424	-0.256829	BGIOSGA006536	XP_004976560.1putative FBD-associated F-box protein At1g61330
TCONS_00008544	0.399699	-0.626055	-0.0629891	BGIOSGA014563	XP_006648915.1PREDICTED: uncharacterized protein LOC102710954
TCONS_00001569	0.399938	0.429904	0.428736	BGIOSGA002722	XP_006644515.1 30S ribosomal protein S20, chloroplastic
TCONS_00024075	0.399951	0.731028	0.30724	BGIOSGA026109	XP_006658799.1 peroxisome biogenesis factor 10
TCONS_00023577	0.400228	0.0279095	0.052623	BGIOSGA005373	XP_015695298.1 protein PHR1-LIKE 1-like
TCONS_00034412	0.400323	-2.98159	0.431772	BGIOSGA034396	XP_003577776.1dirigent protein 21
TCONS_00017943	0.400624	0.222516	0.45686	BGIOSGA000519	XP_004961845.1ubiquitin-conjugating enzyme E2 7
TCONS_00020072	0.400679	0.498607	-0.205118	BGIOSGA022253	XP_015693867.1 protein cornichon homolog 4
TCONS_00015439	0.400679	-1.08636	0.701773	#N/A	#N/A
TCONS_00019710	0.400715	-0.00690173	-0.189424	BGIOSGA013884	XP_006655537.1 flap endonuclease 1-A
TCONS_00033380	0.400975	0.850795	0.775138	BGIOSGA032985	XP_006663404.1 signal peptide peptidase-like 2
TCONS_00002908	0.400977	-0.364569	-0.661558	BGIOSGA018735	XP_006643796.1 protein NETWORKED 4B-like
TCONS_00024931	0.401187	#NA	0.889017	BGIOSGA036106	NP_001141382.1uncharacterized LOC100273473
TCONS_00030431	0.401322	-0.201712	-0.141339	BGIOSGA004775	XP_003578514.1triosephosphate isomerase, chloroplastic
TCONS_00036600	0.401592	-0.87412	-1.11749	BGIOSGA030934	XP_024042344.1protein DETOXIFICATION 24 isoform X2
TCONS_00022672	0.401701	-0.205274	0.299219	BGIOSGA015572	XP_006656312.1 probable protein phosphatase 2C 58
TCONS_00010369	0.401855	-0.661517	0.235257	BGIOSGA009769	XP_006650231.1PREDICTED: uncharacterized protein LOC102707568
TCONS_00010950	0.401982	1.07102	0.991656	BGIOSGA025231	XP_006650578.1PREDICTED: uncharacterized protein LOC102716107
TCONS_00020188	0.402026	1.90125	1.25478	BGIOSGA006940	NP_001150644.1zinc finger, C2H2 type family protein
TCONS_00014504	0.402076	1.00109	0.136478	BGIOSGA031085	XP_006652451.1PREDICTED: uncharacterized protein LOC102711251
TCONS_00016969	0.402239	-0.645626	-0.58135	BGIOSGA014051	XP_006653953.1 E3 ubiquitin-protein ligase HERC2-like
TCONS_00028217	0.40224	0.368419	1.08136	BGIOSGA024846	XP_006659522.1 E3 ubiquitin-protein ligase CIP8-like
TCONS_00021339	0.402443	0.438737	0.832244	BGIOSGA020114	XP_006656458.1PREDICTED: uncharacterized protein LOC102711269
TCONS_00028291	0.402481	0.581554	0.454448	BGIOSGA014557	XP_006659580.1 aminoacylase-1
TCONS_00031436	0.402495	0.911161	1.52687	BGIOSGA033251	XP_006662500.1 methionine aminopeptidase 1A
TCONS_00005860	0.402874	-0.0310582	-0.402176	#N/A	#N/A
TCONS_00007352	0.402953	1.45759	0.371002	BGIOSGA023535	XP_006646888.1 DEAD-box ATP-dependent RNA helicase 35A
TCONS_00017137	0.403135	0.234053	1.05097	BGIOSGA019183	XP_006654995.1 probable serine/threonine-protein phosphatase 2A regulatory subunit B'' subunit TON2
TCONS_00033570	0.403181	-0.967865	0.49765	BGIOSGA000127	XP_006662958.2 chromophore lyase CRL, chloroplastic
TCONS_00015236	0.403248	-3.14263	-1.02475	BGIOSGA020098	XP_008669737.2cytochrome P450 93G1
TCONS_00011338	0.403513	-0.354376	0.473499	BGIOSGA000207	XP_006652023.1 aspartokinase 1, chloroplastic isoform X2
TCONS_00005392	0.403526	1.53805	1.42369	#N/A	#N/A
TCONS_00008199	0.40361	0.413424	-0.46682	BGIOSGA035513	XP_024317893.1uncharacterized protein LOC100832974
TCONS_00017703	0.403974	0.0530359	0.660655	BGIOSGA000584	XP_002439713.1serine/arginine repetitive matrix protein 1 isoform X1
TCONS_00035795	0.404013	0.0716892	-0.174926	BGIOSGA027838	XP_015696707.1 putative 1-phosphatidylinositol-3-phosphate 5-kinase FAB1D
TCONS_00006043	0.404225	-0.131375	-0.355112	BGIOSGA008200	XP_015688591.1 probable sodium/metabolite cotransporter BASS3, chloroplastic
TCONS_00011254	0.404297	-0.506927	-1.97685	BGIOSGA000497	XP_015690745.1PREDICTED: LOW QUALITY PROTEIN: uncharacterized protein LOC107303923
TCONS_00016062	0.404349	0.308234	0.393326	BGIOSGA003921	XP_006653508.2 pentatricopeptide repeat-containing protein At3g63370, chloroplastic
TCONS_00014837	0.404375	0.230864	1.07286	BGIOSGA017010	XP_006652715.1 protein SSUH2 homolog
TCONS_00008137	0.404889	-6.44666	-1.57974	BGIOSGA034464	XP_006648706.2 NDR1/HIN1-Like protein 3-like
TCONS_00006176	0.40495	-0.170034	0.355893	BGIOSGA016334	XP_006647322.1 acetyl-coenzyme A synthetase, chloroplastic/glyoxysomal
TCONS_00008722	0.405076	0.567126	1.27777	BGIOSGA005711	XP_015688968.1 DNA-directed RNA polymerases IV and V subunit 4 isoform X2
TCONS_00002598	0.405268	0.931419	0.854787	BGIOSGA016503	XP_014754790.1protein MITOFERRINLIKE 1, chloroplastic
TCONS_00002241	0.405427	0.599437	0.535303	BGIOSGA020774	XP_006646509.1 magnesium transporter MRS2-E
TCONS_00026423	0.405598	0.471308	-1.58152	BGIOSGA037134	XP_004972482.1probable serine/threonine-protein kinase At1g54610
TCONS_00030399	0.405608	-0.240026	0.798271	BGIOSGA018327	XP_006660866.1PREDICTED: uncharacterized protein LOC102716623
TCONS_00017169	0.405664	1.8151	0.52013	BGIOSGA021571	XP_006655011.1 dnaJ protein ERDJ3B

TCONS_00000951	0.405778	0.0805557	-0.0910582	BGIOSGA026631	XP_004968771.1bifunctional 3-dehydroquinate dehydratase/shikimate dehydrogenase, chloroplastic
TCONS_00014200	0.405813	0.0116044	-0.997094	BGIOSGA004836	NP_001344779.1catalytic/ hydrolase
TCONS_00011738	0.406087	-0.53591	-0.0573048	BGIOSGA005677	XP_004985315.1ADP-ribosylation factor-like protein 8a
TCONS_00010082	0.40619	0.653396	0.62112	BGIOSGA023769	XP_015690309.1 protein EARLY RESPONSIVE TO DEHYDRATION 15-like
TCONS_00028095	0.406318	0.388225	0.74516	BGIOSGA026898	XP_015695622.1 dentin sialophosphoprotein-like
TCONS_00007696	0.406633	-0.141852	0.375672	BGIOSGA023051	XP_006647119.1 T-complex protein 1 subunit gamma-like
TCONS_00011920	0.406797	-0.890107	0.585406	BGIOSGA011092	XP_006649758.1 probable mitochondrial import inner membrane translocase subunit TIM21
TCONS_00001407	0.40689	-0.429837	-0.843659	BGIOSGA001070	XP_006644397.1 CTD small phosphatase-like protein 2 isoform X2
TCONS_00001266	0.406978	1.67134	1.24427	BGIOSGA037494	XP_006646063.1PREDICTED: uncharacterized protein LOC102722356, partial
TCONS_00006409	0.407176	-2.15613	-0.735548	BGIOSGA019572	XP_015689145.1 protein phosphatase inhibitor 2-like
TCONS_00010994	0.407424	0.676991	1.52221	BGIOSGA022974	XP_015690074.1PREDICTED: LOW QUALITY PROTEIN: uncharacterized protein LOC102720317
TCONS_00013465	0.407518	0.112595	0.661766	BGIOSGA013859	XP_006650835.1 50S ribosomal protein L18, chloroplastic
TCONS_00010340	0.407596	-0.914815	-0.305154	BGIOSGA012916	XP_015690362.1 membrane-anchored ubiquitin-fold protein 1
TCONS_00016835	0.40773	0.740604	0.82918	BGIOSGA014129	XP_006652994.1 methylthioribose kinase 1
TCONS_00031532	0.407792	-5.21329	-1.00204	BGIOSGA032833	XP_021306961.1beta-carotene 3-hydroxylase, chloroplastic
TCONS_00023986	0.407926	0.29552	0.380428	BGIOSGA026020	XP_004958141.1transcription termination factor MTERF9, chloroplastic
TCONS_00032582	0.408129	0.541284	0.549852	BGIOSGA018613	XP_002465046.1shaggy-related protein kinase alpha isoform X1
TCONS_00021107	0.40814	-1.29284	-1.25422	BGIOSGA006997	XP_006657170.1 UPF0047 protein YjbQ
TCONS_00020343	0.408153	-0.317009	0.465516	BGIOSGA003499	XP_006655904.1 non-specific lipid-transfer protein-like 1
TCONS_00018405	0.408174	-1.00741	-0.951976	BGIOSGA020457	XP_006654849.1 jasmonic acid-amido synthetase JAR1
TCONS_00026860	0.408293	0.985009	0.103661	BGIOSGA028916	XP_006659535.2 cytochrome c oxidase assembly protein COX15
TCONS_00009392	0.40837	0.667806	0.815644	BGIOSGA011941	XP_006649463.1PREDICTED: uncharacterized protein LOC102722470, partial
TCONS_00023827	0.408384	0.512216	0.645392	BGIOSGA025856	XP_003562988.1pre-rRNA-processing protein ESF1
TCONS_00034303	0.408392	0.416635	0.3262	BGIOSGA036638	XP_003578924.1uncharacterized abhydrolase domain-containing protein DDB_G0269086
TCONS_00037419	0.408494	1.53178	0.581178	BGIOSGA028917	XP_004962991.1probable galacturonosyltransferase 13
TCONS_00008713	0.408494	-0.434229	0.801353	BGIOSGA028928	XP_015689331.1 uncharacterized WD repeat-containing protein C2A9.03-like
TCONS_00026633	0.40852	0.510682	0.470081	BGIOSGA027985	XP_006659413.1 annexin D5-like
TCONS_00001587	0.408932	0.76497	0.685253	BGIOSGA004222	XP_022682576.1ATP synthase subunit beta, mitochondrial-like
TCONS_00004426	0.408937	0.329583	0.110591	BGIOSGA000796	XP_015689921.1 bifunctional riboflavin kinase/FMN phosphatase-like
TCONS_00007269	0.40911	0.0535367	0.646041	BGIOSGA007214	XP_015688438.1 NADP-dependent D-sorbitol-6-phosphate dehydrogenase-like
TCONS_00013411	0.409289	0.349957	0.313976	BGIOSGA015474	XP_006651952.1 ethylene-responsive transcription factor RAP2-12-like
TCONS_00022269	0.409344	0.528379	-0.165838	BGIOSGA021226	XP_015693746.1 protein SAWADEE HOMEODOMAIN HOMOLOG 2-like
TCONS_00020251	0.409439	0.475423	0.483553	BGIOSGA022440	XP_015693749.1 AUGMIN subunit 3
TCONS_00021540	0.409472	-0.523317	0.13852	BGIOSGA022010	XP_004964427.1barley B recombinant-like protein D
TCONS_00012229	0.409507	0.732839	0.931671	BGIOSGA040269	XP_006651566.1 fumarate hydratase 1, mitochondrial-like
TCONS_00015745	0.409615	0.250364	0.700275	BGIOSGA037210	XP_015691418.1 3-oxoacyl-[acyl-carrier-protein] reductase 4
TCONS_00019623	0.409709	0.477963	0.202481	BGIOSGA015394	XP_006654636.1PREDICTED: uncharacterized protein LOC102702848 isoform X2
TCONS_00019665	0.410556	-1.99438	#NA	BGIOSGA032130	XP_002441419.2anthocyanidin 5,3-O-glucosyltransferase
TCONS_00019554	0.410607	1.45832	-0.190999	BGIOSGA001916	XP_015693172.1 cysteine proteinase inhibitor 2-like
TCONS_00006424	0.410794	0.675908	1.12767	BGIOSGA014809	XP_006647482.1PREDICTED: uncharacterized protein LOC102700591
TCONS_00003905	0.410992	1.29785	0.311948	BGIOSGA034723	XP_002441721.1uncharacterized protein LOC8071415 isoform X1
TCONS_00022195	0.411195	0.255206	0.689189	BGIOSGA002059	XP_006656057.1 phosphatidylinositol 4-kinase gamma 4-like
TCONS_00002550	0.411497	-1.04927	-1.35947	BGIOSGA005192	XP_006645378.1PREDICTED: uncharacterized protein LOC102722445
TCONS_00019747	0.411511	1.50116	0.954304	BGIOSGA015943	XP_015693370.1 CRM-domain containing factor CFM3, chloroplastic/mitochondrial
TCONS_00007581	0.411981	-0.102918	-1.18839	BGIOSGA025437	XP_004951504.1UDP-glycosyltransferase 73C3
TCONS_00023972	0.412206	-1.21088	-0.67098	BGIOSGA030566	XP_006657881.1 chlorophyll a-b binding protein 7, chloroplastic
TCONS_00023285	0.412261	1.27879	1.56978	BGIOSGA013755	XP_007034425.2 putative vesicle-associated membrane protein 726
TCONS_00016804	0.412424	0.0533167	1.05123	BGIOSGA018192	XP_017608293.1 PHD finger-like domain-containing protein 5B
TCONS_00016411	0.412855	0.972357	0.985424	BGIOSGA014556	NP_001148070.1flowering locus D
TCONS_00001465	0.412883	1.35016	0.140002	BGIOSGA004103	NP_001149398.1F-box domain containing protein

TCONS_00000639	0.412907	1.17623	0.918183	BGIOSGA003223	XP_006645748.2 DNA gyrase subunit B, chloroplastic/mitochondrial
TCONS_00026097	0.412918	-0.296908	-0.207375	BGIOSGA019490	XP_015695988.1 probable LRR receptor-like serine/threonine-protein kinase At1g56140
TCONS_00011344	0.412952	0.32165	-0.779575	BGIOSGA027713	XP_004981029.1monothiol glutaredoxin-S7, chloroplastic isoform X2
TCONS_00008020	0.413359	-0.354488	-0.362092	BGIOSGA008809	XP_006648643.1 U-box domain-containing protein 9-like
TCONS_00016666	0.413388	1.8993	1.41707	BGIOSGA028097	XP_006652864.1 helicase-like transcription factor CHR28
TCONS_00001899	0.413478	0.758898	0.245865	BGIOSGA002192	XP_003569928.1casein kinase 1-like protein HD16
TCONS_00006197	0.413581	-1.94315	0.355005	BGIOSGA016030	XP_006647342.1 alpha carbonic anhydrase 1, chloroplastic-like
TCONS_00036900	0.413641	0.124205	1.18137	BGIOSGA031602	XP_006661926.1 F-box/LRR-repeat protein At3g48880
TCONS_00031051	0.41418	0.413054	0.899668	BGIOSGA021160	XP_006661752.1 probable methionine--tRNA ligase
TCONS_00028456	0.414298	0.561199	0.144929	BGIOSGA026506	XP_006660379.1 phosphatidylinositol-3-phosphatase myotubularin-1-like
TCONS_00007902	0.414334	1.31101	1.84893	#N/A	#N/A
TCONS_00009861	0.414506	-0.333091	-0.0467275	BGIOSGA012412	XP_006649922.1 NADH dehydrogenase [ubiquinone] 1 alpha subcomplex subunit 8-B-like
TCONS_00020613	0.414774	0.911722	0.828307	BGIOSGA005728	XP_004965340.160S ribosomal protein L31
TCONS_00001787	0.414783	-0.304883	0.547731	BGIOSGA036243	XP_006644339.1 serine/threonine-protein phosphatase 7 long form homolog
TCONS_00013274	0.414901	-0.534874	-0.175268	BGIOSGA017702	XP_015691153.1 reticulon-like protein B8
TCONS_00027969	0.414914	#NA	1.81679	#N/A	#N/A
TCONS_00027577	0.41499	-0.154856	0.023745	BGIOSGA027443	XP_015696220.1 VIN3-like protein 2 isoform X1
TCONS_00018504	0.415361	-0.538281	-0.435261	BGIOSGA000008	XP_006653975.1 11S globulin seed storage protein 2-like
TCONS_00028849	0.415428	0.290136	-0.150099	BGIOSGA014624	XP_006660567.2 histone-lysine N-methyltransferase, H3 lysine-9 specific SUVH6-like
TCONS_00024059	0.415469	-0.630601	-1.22891	BGIOSGA010297	XP_006657933.1 UDP-arabinopyranose mutase 3
TCONS_00018146	0.415935	1.46382	0.430568	BGIOSGA002373	XP_006655495.1 VQ motif-containing protein 4-like
TCONS_00018042	0.416329	0.482172	0.582679	BGIOSGA010841	XP_006654573.1 endoplasmic reticulum-Golgi intermediate compartment protein 3-like
TCONS_00028374	0.416343	1.43584	0.661158	BGIOSGA024653	XP_015695940.1 nuclear transport factor 2 isoform X2
TCONS_00004307	0.416558	0.842013	0.793546	BGIOSGA008040	NP_001131270.1NADH-ubiquinone oxidoreductase subunit
TCONS_00016122	0.416686	1.03629	1.21936	BGIOSGA027740	XP_015692269.1 programmed cell death protein 4-like isoform X2
TCONS_00015779	0.416914	0.961056	0.862559	BGIOSGA016727	XP_006652211.2 2,3-dimethylmalate lyase-like
TCONS_00006422	0.417518	0.967809	1.36326	BGIOSGA009461	XP_015689639.1 U1 snRNP-associated protein usp106-like isoform X1
TCONS_00034141	0.417737	1.0773	0.405772	BGIOSGA018329	XP_015698917.1 regulatory-associated protein of TOR 2
TCONS_00000492	0.41781	-1.54794	-0.816481	BGIOSGA017578	XP_006643945.1 auxin-responsive protein IAA3
TCONS_00008008	0.417844	0.682459	0.847152	BGIOSGA006469	XP_006648640.1PREDICTED: uncharacterized protein LOC102712146
TCONS_00021813	0.418106	1.00978	0.638815	BGIOSGA009128	XP_015693828.1 probable cyclic nucleotide-gated ion channel 20, chloroplastic
TCONS_00004753	0.418125	0.228502	0.117114	BGIOSGA004830	NP_001150939.1undecaprenyl pyrophosphate synthetase
TCONS_00015681	0.418419	0.399382	0.0527931	BGIOSGA033725	XP_006653291.1 LRR receptor-like serine/threonine-protein kinase GSO1
TCONS_00002221	0.41849	1.80796	0.273397	BGIOSGA004856	XP_006645067.1PREDICTED: uncharacterized protein LOC102703079
TCONS_00020673	0.41863	-2.69402	0.0727825	BGIOSGA022858	XP_015694103.1PREDICTED: uncharacterized protein LOC102712736
TCONS_00021677	0.418917	0.24068	0.228491	BGIOSGA001699	XP_006655823.1 serine/threonine-protein phosphatase PP1
TCONS_00036965	0.419054	0.215401	-0.796351	BGIOSGA001610	XP_002440701.1cytochrome P450 71A1
TCONS_00001340	0.419068	0.844227	0.364329	BGIOSGA016414	XP_006644357.1 N-alpha-acetyltransferase 50
TCONS_00014013	0.41922	0.866756	0.588658	BGIOSGA016161	XP_003581104.1sister chromatid cohesion protein SCC4
TCONS_00009722	0.41939	0.552737	0.555194	BGIOSGA012272	XP_006649789.2 vacuolar protein sorting-associated protein 9A-like
TCONS_00006598	0.419426	1.07539	-0.499556	BGIOSGA017378	XP_015688900.1 probable phospholipid hydroperoxide glutathione peroxidase
TCONS_00022682	0.419675	-0.674347	-0.202724	BGIOSGA020758	XP_014753040.1D-3-phosphoglycerate dehydrogenase 3, chloroplastic
TCONS_00015298	0.419683	0.271758	0.148696	BGIOSGA007384	XP_015692108.1 serine/arginine-rich splicing factor RS31-like
TCONS_00014731	0.419727	0.321182	-0.213678	BGIOSGA016905	XP_006652627.1 sugar phosphatase YfbT
TCONS_00004740	0.419822	0.230787	0.741508	BGIOSGA035882	XP_015692458.1 AT-rich interactive domain-containing protein 2-like
TCONS_00036035	0.420192	1.95989	0.566137	BGIOSGA037872	XP_021301326.1uncharacterized membrane protein YMR155W
TCONS_00003140	0.420197	0.333123	-0.51674	BGIOSGA015765	XP_021313006.1hydroxyphenylpyruvate reductase
TCONS_00030679	0.4202	1.90847	-1.06264	BGIOSGA025520	XP_006650706.1 probable aldehyde oxidase 2
TCONS_00026113	0.420267	-1.92329	-1.00752	BGIOSGA029263	XP_006659210.1 putative potassium transporter 12 isoform X1
TCONS_00024309	0.420348	-0.970448	-1.28335	BGIOSGA035641	XP_006658084.1 lipase-like
TCONS_00008102	0.420487	-2.14034	-1.25015	BGIOSGA015148	XP_012698745.1uncharacterized protein At4g22758
TCONS_00027765	0.420646	1.83576	-0.014294	BGIOSGA031243	XP_010234557.1HSP-interacting protein
TCONS_00020568	0.420774	0.435165	0.787645	BGIOSGA022745	XP_006656025.1 serine/threonine-protein phosphatase 4 regulatory subunit 2

TCONS_00022463	0.420971	1.01012	1.25007	BGIOSGA020992	XP_015694225.1 transcriptional elongation regulator MINIYO
TCONS_00004121	0.421083	-0.479944	-0.230238	BGIOSGA021040	XP_015691232.1 aspartic proteinase oryzasin-1-like
TCONS_00021456	0.421142	-0.314797	0.214348	BGIOSGA007334	XP_015693759.1PREDICTED: uncharacterized protein LOC102721843
TCONS_00023990	0.421155	1.60549	1.09873	BGIOSGA017631	XP_006657891.1 probable serine/threonine-protein kinase WNK5
TCONS_00027646	0.42161	1.75233	0.972249	BGIOSGA027368	XP_003573607.1aspartate carbamoyltransferase, chloroplastic
TCONS_00000211	0.421729	1.41553	1.55077	BGIOSGA001568	XP_006643766.1 histone H2B.5
TCONS_00012164	0.421884	0.0789632	0.835683	BGIOSGA010846	NP_001105640.1aquaporin SIP2-1
TCONS_00021788	0.422757	0.376915	-0.414678	BGIOSGA021734	XP_002436639.1peroxiredoxin Q, chloroplastic
TCONS_00013396	0.422851	0.267482	0.920911	BGIOSGA000776	XP_006650774.1 triacylglycerol lipase SDP1-like
TCONS_00028688	0.422906	0.191802	1.11169	BGIOSGA028485	XP_015696604.1PREDICTED: uncharacterized protein LOC102699985 isoform X2
TCONS_00013819	0.422972	0.385328	0.990249	BGIOSGA037315	XP_021318603.1aspartyl protease family protein 1 isoform X1
TCONS_00016392	0.423105	1.8154	1.15039	BGIOSGA013368	XP_003580303.1putative cis-zeatin O-glucosyltransferase
TCONS_00018301	0.423124	-0.463061	-0.964072	BGIOSGA001041	XP_006655586.2 putative mediator of RNA polymerase II transcription subunit 26
TCONS_00022725	0.423351	0.578011	-0.55507	BGIOSGA020726	XP_002438805.1fructose-1,6-bisphosphatase, chloroplastic
TCONS_00003424	0.423407	0.409029	2.42527	BGIOSGA023501	XP_002448545.1uncharacterized protein LOC8074717 isoform X2
TCONS_00001669	0.42342	-0.338348	0.0953173	BGIOSGA004304	XP_006644593.1 trafficking protein particle complex subunit 5
TCONS_00018051	0.423846	-0.195087	0.250313	BGIOSGA035410	XP_006654586.1 dr1-associated corepressor-like isoform X1
TCONS_00008894	0.423995	0.307039	1.44991	BGIOSGA009631	XP_004954081.1uncharacterized protein LOC101763834
TCONS_00000188	0.424337	-0.258691	0.293384	BGIOSGA002763	XP_006643751.1PREDICTED: uncharacterized protein LOC102714864
TCONS_00003143	0.42437	1.03884	1.92926	BGIOSGA002221	XP_006643941.1 eukaryotic translation initiation factor 3 subunit L-like
TCONS_00031069	0.424532	-0.541068	-1.87182	BGIOSGA029500	XP_006660800.1 mimitin, mitochondrial
TCONS_00002580	0.424583	-1.05571	-0.87195	BGIOSGA005222	XP_010232844.1nitrate regulatory gene2 protein
TCONS_00026571	0.424698	-1.97607	-0.363478	BGIOSGA011433	XP_015695825.1 heavy metal-associated isoprenylated plant protein 26-like
TCONS_00018469	0.424698	-0.461493	0.543413	BGIOSGA026501	XP_024447371.1actin-7-like
TCONS_00037428	0.424788	-0.565358	0.0266878	BGIOSGA029710	XP_006664148.1 zinc finger protein 7-like
TCONS_00007714	0.4249	0.884053	-0.67971	BGIOSGA032871	NP_001151100.1VQ motif family protein
TCONS_00004183	0.4249	-0.530985	0.642218	#N/A	#N/A
TCONS_00018194	0.42514	-0.841225	-2.43212	BGIOSGA006665	XP_006654700.2 calcineurin B-like protein 4 isoform X2
TCONS_00002227	0.425585	0.941059	0.13483	BGIOSGA017824	XP_015691898.1 VAMP-like protein YKT61
TCONS_00007132	0.425632	0.487627	0.679969	BGIOSGA027881	XP_006648145.1 NAC domain-containing protein 53
TCONS_00035248	0.42613	0.947756	0.758667	BGIOSGA033657	XP_015698088.1 1-acyl-sn-glycerol-3-phosphate acyltransferase PLS1 isoform X2
TCONS_00019446	0.426189	1.76532	0.543448	BGIOSGA011800	XP_010231289.1phosphatidate phosphatase PAH1
TCONS_00034113	0.426751	0.500097	-0.138258	BGIOSGA006114	XP_015697535.1 mitochondrial substrate carrier family protein C-like
TCONS_00030760	0.426834	0.178487	0.822871	BGIOSGA024641	XP_006661647.1 F-box protein At2g26160-like
TCONS_00013270	0.426863	0.111337	0.859326	BGIOSGA009716	XP_006650679.1 alpha N-terminal protein methyltransferase 1
TCONS_00019887	0.426881	1.39644	0.537251	BGIOSGA037404	XP_015693052.1 calcium-dependent protein kinase 3-like
TCONS_00003077	0.427044	1.25541	0.500151	BGIOSGA024910	XP_006643896.1PREDICTED: uncharacterized protein LOC102710914
TCONS_00015018	0.427333	-0.570476	0.565214	BGIOSGA024122	XP_003580624.2G-type lectin S-receptor-like serine/threonine-protein kinase B120 isoform X1
TCONS_00025735	0.427388	2.45532	0.442983	BGIOSGA002157	XP_006658162.1 galactinol synthase 2-like
TCONS_00037057	0.42794	-2.54537	-0.00584774	BGIOSGA018557	XP_003575559.1ABC transporter G family member 11
TCONS_00007250	0.428227	0.26162	0.743462	BGIOSGA018877	XP_015688430.1 pentatricopeptide repeat-containing protein At2g17670-like
TCONS_00002426	0.428298	-1.16731	-2.14424	BGIOSGA011529	XP_006646619.1PREDICTED: uncharacterized protein At4g15970-like
TCONS_00014760	0.428358	1.18295	0.369137	BGIOSGA013241	XP_008790519.1 ORM1-like protein 3
TCONS_00024182	0.428449	-0.219227	-0.788068	BGIOSGA026220	XP_006658014.1 chaperone protein DnaJ
TCONS_00018998	0.428508	-2.01354	-1.10706	BGIOSGA018367	XP_015693295.1PREDICTED: uncharacterized protein LOC102709083 isoform X3
TCONS_00008654	0.428726	-0.121206	-1.40584	BGIOSGA000012	XP_015689467.1 soluble inorganic pyrophosphatase
TCONS_00004850	0.428784	-1.1325	0.165345	BGIOSGA019892	XP_004970825.1serine/arginine repetitive matrix protein 1 isoform X2
TCONS_00023171	0.428978	0.780484	0.745141	BGIOSGA025767	XP_006657560.2 eukaryotic translation initiation factor 3 subunit E
TCONS_00035791	0.42898	0.809506	0.074341	BGIOSGA037196	XP_006664450.1 argininosuccinate synthase, chloroplastic
TCONS_00009558	0.42922	1.43018	1.18542	BGIOSGA029934	XP_015696697.1 protein transport protein Sec61 subunit alpha-like isoform X1
TCONS_00006474	0.429233	-0.587064	-0.341686	BGIOSGA025612	XP_006647537.1 glycolipid transfer protein 1-like
TCONS_00008465	0.429312	0.693194	0.923605	BGIOSGA014666	XP_006648877.1 thioredoxin M1, chloroplastic
TCONS_00007431	0.429641	0.174624	0.382608	BGIOSGA017683	XP_006646949.1 ALA-interacting subunit 1
TCONS_00026581	0.429744	0.789591	0.705509	BGIOSGA028649	XP_006659386.1 uncharacterized WD repeat-containing protein C2A9.03
TCONS_00007434	0.429902	0.478399	0.270311	BGIOSGA007234	XP_006646952.1 serine/threonine-protein kinase HT1-like

TCONS_00004767	0.42997	-0.00582385	0.423138	BGIOSGA015452	XP_015695251.1PREDICTED: uncharacterized protein LOC107304671
TCONS_00030174	0.430093	0.684855	-0.913495	BGIOSGA029639	XP_003576610.2probable carboxylesterase 12
TCONS_00019691	0.430162	2.39549	0.863018	BGIOSGA004347	XP_004961397.1protein MLN51 homolog
TCONS_00012895	0.430448	0.67187	1.07182	BGIOSGA026319	XP_006651672.2 DEAD-box ATP-dependent RNA helicase 10
TCONS_00008551	0.430691	1.55873	0.404848	BGIOSGA033096	XP_012698797.1 heterodimeric geranylgeranyl pyrophosphate synthase small subunit, chloroplastic
TCONS_00010287	0.430707	1.047	-0.397841	BGIOSGA023917	XP_006650187.1 probable small nuclear ribonucleoprotein G
TCONS_00019963	0.430716	0.23866	-0.827576	BGIOSGA022151	XP_006655690.2 uncharacterized membrane protein YjcL
TCONS_00011429	0.43081	0.359858	0.334158	BGIOSGA030821	XP_022682441.1uncharacterized protein LOC101760608 isoform X2
TCONS_00029596	0.430914	0.450888	0.986796	BGIOSGA026273	XP_015695132.1PREDICTED: uncharacterized protein LOC102715406
TCONS_00030523	0.430937	0.250993	0.172292	BGIOSGA028493	XP_015696260.1 probable purine permease 5
TCONS_00002854	0.43099	-0.526607	-0.257227	BGIOSGA010978	XP_003565209.1protein Hook homolog 3 isoform X1
TCONS_00027729	0.431011	#NA	-2.27993	BGIOSGA028250	XP_003573587.1endo-1,3;1,4-beta-D-glucanase isoform X1
TCONS_00002520	0.431087	0.102458	0.741441	BGIOSGA005160	XP_006645395.1 ganglioside-induced differentiation-associated protein 2
TCONS_00006018	0.431113	0.865851	0.772388	BGIOSGA028234	XP_021315883.1uncharacterized protein LOC8077808
TCONS_00016059	0.431242	0.481721	-0.719402	BGIOSGA006159	XP_002447999.1heavy metal-associated isoprenylated plant protein 47
TCONS_00013283	0.431269	0.526269	1.49828	BGIOSGA009939	XP_006650688.1 accelerated cell death 11
TCONS_00016738	0.431319	0.188764	0.44942	BGIOSGA014224	XP_006652916.1 glycerol kinase
TCONS_00008198	0.431375	-0.650627	0.23017	BGIOSGA037716	XP_006647392.1 glutathione S-transferase Z1-like isoform X1
TCONS_00036129	0.431593	-0.226508	1.05357	BGIOSGA021094	XP_006664597.1 ribosomal protein S10, mitochondrial
TCONS_00001600	0.431664	-0.386157	0.040332	BGIOSGA017531	XP_003569589.1formin-like protein 15
TCONS_00037619	0.431995	0.270317	0.502837	BGIOSGA035747	XP_006664798.1 structural maintenance of chromosomes protein 1
TCONS_00016897	0.432074	1.11477	1.27605	BGIOSGA022207	XP_015691646.1 pre-mRNA cleavage factor Im 25 kDa subunit 2 isoform X1
TCONS_00008802	0.43241	1.65245	1.06838	BGIOSGA029419	XP_015688855.1 ATPase GET3-like
TCONS_00004515	0.433264	-0.829664	0.211081	BGIOSGA020135	XP_015688057.1 probable 1-acyl-sn-glycerol-3-phosphate acyltransferase 5
TCONS_00002532	0.433301	0.886548	0.909205	BGIOSGA017605	XP_015688273.1 uridine 5'-monophosphate synthase
TCONS_00035033	0.43406	0.234484	0.126085	BGIOSGA033824	XP_006662986.2 maf-like protein DDB_G0281937
TCONS_00027932	0.434095	-0.194152	-0.140326	BGIOSGA030505	XP_006659352.1 NEDD8-conjugating enzyme Ubc12-like
TCONS_00014187	0.434518	0.603788	0.995	BGIOSGA013985	XP_006652246.1 gibberellin 2-beta-dioxygenase 8-like
TCONS_00037301	0.434784	0.763313	0.481016	BGIOSGA009409	XP_004962851.1acyl carrier protein 1, chloroplastic
TCONS_00016097	0.435054	1.11545	1.38599	BGIOSGA006133	XP_006653523.1 NAC domain-containing protein 8-like
TCONS_00012488	0.435114	0.195008	1.47315	BGIOSGA026075	XP_015691218.1 EID1-like F-box protein 2
TCONS_00007959	0.435244	-1.55178	-2.34184	BGIOSGA026365	XP_003573212.1flowering-promoting factor 1-like protein 3
TCONS_00025437	0.43529	0.312768	0.237929	BGIOSGA019747	XP_006663705.1 eukaryotic peptide chain release factor subunit 1-3-like
TCONS_00036421	0.435486	0.356479	0.40068	BGIOSGA001750	XP_006664201.2 probable beta-1,3-galactosyltransferase 20
TCONS_00014883	0.43569	-0.204296	0.290092	BGIOSGA007035	XP_006652768.1 putative glucose-6-phosphate 1-epimerase
TCONS_00029533	0.435972	0.331612	0.133744	BGIOSGA029179	XP_006660991.1 histidine-containing phosphotransfer protein 2
TCONS_00028098	0.436209	0.289769	0.0612976	BGIOSGA008679	XP_006648864.1 proteasome subunit alpha type-2
TCONS_00007507	0.436252	-0.494622	1.24248	BGIOSGA006978	XP_006648402.1 biogenesis of lysosome-related organelles complex 1 subunit 2
TCONS_00032841	0.436343	-0.258794	0.314974	BGIOSGA036731	XP_006663736.1 stress response protein nst1-like
TCONS_00001604	0.436357	0.0810235	-0.340795	BGIOSGA000994	XP_024315008.1LEAF RUST 10 DISEASE-RESISTANCE LOCUS RECEPTOR-LIKE PROTEIN KINASE-like 2.4 isoform X1
TCONS_00010204	0.436372	1.17872	0.343981	BGIOSGA013706	XP_003557807.1non-specific lipid transfer protein-like 1
TCONS_00030371	0.436765	-2.44722	-1.40759	BGIOSGA029427	XP_004957380.1uncharacterized protein LOC101772217
TCONS_00010210	0.436978	1.0961	0.545204	BGIOSGA012773	XP_015689919.1 intron-binding protein aquarius
TCONS_00018149	0.437034	-0.0434879	0.927335	BGIOSGA020207	XP_006654659.2 probable transcriptional regulatory protein At2g25830 isoform X2
TCONS_00000329	0.437243	-2.71422	#NA	BGIOSGA019276	XP_021316038.1uncharacterized protein LOC110435051
TCONS_00023646	0.437927	-0.827881	0.498567	BGIOSGA025668	XP_006657681.2 protein SET DOMAIN GROUP 40
TCONS_00018427	0.437974	0.640596	0.751689	BGIOSGA020481	XP_006654871.1 DNA damage-binding protein 1a
TCONS_00003289	0.43811	1.24805	1.47856	BGIOSGA034747	XP_006644039.2 hydroxymethylglutaryl-CoA lyase, mitochondrial-like
TCONS_00036020	0.438291	0.552199	0.658875	BGIOSGA029518	XP_003575526.1NAC domain-containing protein 83
TCONS_00010180	0.438326	-0.465926	0.0425514	BGIOSGA012743	XP_010651562.1 protein DGS1, mitochondrial isoform X2
TCONS_00026679	0.438348	0.947212	0.910678	BGIOSGA028746	XP_004973553.1uncharacterized protein LOC101785423

TCONS_00011050	0.438654	-0.0337989	1.24215	BGIOSGA013648	XP_015691305.1 protein AE7-like 1 isoform X2
TCONS_00011454	0.438676	-0.282893	-0.249892	BGIOSGA022218	XP_006649278.1 peroxisomal membrane protein 11-1
TCONS_00012091	0.438757	0.814161	1.4591	BGIOSGA010918	XP_006651283.1 malonyl CoA-acyl carrier protein transacylase
TCONS_00003256	0.439066	0.1298	0.904139	BGIOSGA001959	XP_006644012.1 zinc finger CCCH domain-containing protein 5
TCONS_00001029	0.439118	0.809173	0.281314	BGIOSGA003632	XP_006645939.1 lisH domain and HEAT repeat-containing protein KIAA1468
TCONS_00018968	0.439191	0.732621	-0.263703	BGIOSGA040645	XP_006654198.1 B-cell receptor-associated protein 31-like
TCONS_00025516	0.439296	1.23361	0.0745279	BGIOSGA010379	XP_006657946.1 40S ribosomal protein S3-1-like
TCONS_00009711	0.439355	1.19128	0.676507	BGIOSGA012260	XP_006649775.1 ERAD-associated E3 ubiquitin-protein ligase component HRD3
TCONS_00010270	0.439486	0.477469	0.348179	BGIOSGA012833	XP_015691266.1 3beta-hydroxysteroid-dehydrogenase/decarboxylase isoform 1-like
TCONS_00004295	0.439539	-0.131897	-1.30157	BGIOSGA000912	XP_015695435.1 UPF0544 protein C5orf45 homolog isoform X2
TCONS_00020054	0.439837	1.10086	0.40616	BGIOSGA009886	XP_006656597.2 probable aminotransferase ACS12
TCONS_00006512	0.439958	0.349644	0.448816	BGIOSGA026895	XP_006648864.1 proteasome subunit alpha type-2
TCONS_00026376	0.440119	1.06694	0.948878	BGIOSGA017594	XP_006661015.1 dynamin-related protein 1E-like
TCONS_00024273	0.440331	0.367397	1.40458	BGIOSGA006661	XP_006658066.1 E3 ubiquitin-protein ligase SINAT5-like
TCONS_00023082	0.440465	-0.905109	-0.251196	#N/A	#N/A
TCONS_00036902	0.440523	0.328671	-0.427393	BGIOSGA031600	XP_015697413.1 folylpolyglutamate synthase-like
TCONS_00002195	0.440539	1.04318	0.105874	BGIOSGA004831	XP_015688350.1 nucleobase-ascorbate transporter 12
TCONS_00036459	0.440729	-0.131653	-0.216335	BGIOSGA039527	XP_006664220.1 5-methyltetrahydropteroyltriglutamate--homocysteine methyltransferase 1
TCONS_00005051	0.440814	0.414105	0.717571	BGIOSGA000165	XP_015690875.1 succinate dehydrogenase subunit 4, mitochondrial-like
TCONS_00023922	0.440894	0.639708	-1.49727	BGIOSGA032295	XP_015694961.1 internal alternative NAD(P)H-ubiquinone oxidoreductase A1, mitochondrial-like
TCONS_00017787	0.440962	-0.143941	0.578885	BGIOSGA029370	XP_006654382.1 peptide methionine sulfoxide reductase B3, chloroplastic-like
TCONS_00022184	0.441464	-0.364769	1.43519	BGIOSGA030767	XP_004965464.1cyclin-dependent kinase F-1
TCONS_00032228	0.441471	1.28084	0.693526	BGIOSGA016156	XP_006661780.2 heat stress transcription factor A-2c-like
TCONS_00024669	0.441476	0.560606	0.0139387	BGIOSGA033015	XP_010229183.1uncharacterized protein LOC100832216
TCONS_00033759	0.441626	-0.303996	0.532929	BGIOSGA032051	XP_024311160.1putative disease resistance RPP13-like protein 2 isoform X9
TCONS_00021402	0.442194	-0.738042	-1.84049	BGIOSGA018841	XP_004966497.1NADH dehydrogenase [ubiquinone] 1 beta subcomplex subunit 2
TCONS_00005177	0.442227	-0.522993	-0.982119	BGIOSGA012302	XP_006646733.1 protease HtpX homolog
TCONS_00017728	0.442494	1.92295	-0.23288	BGIOSGA005204	XP_006654349.1 protein RETICULATA-RELATED 4, chloroplastic-like
TCONS_00021557	0.442582	0.337877	1.24843	BGIOSGA021991	XP_002437769.1putative transferase At4g12130, mitochondrial
TCONS_00028173	0.442589	0.0944513	1.13752	BGIOSGA036668	XP_006660212.1 phosphoglycerate mutase-like protein 4
TCONS_00003563	0.442601	0.0254504	0.60517	BGIOSGA001637	XP_015693510.1PREDICTED: uncharacterized protein LOC102703266
TCONS_00023923	0.442695	-0.34825	0.891748	BGIOSGA034397	XP_015692329.1 cell differentiation protein RCD1 homolog
TCONS_00032751	0.442696	0.490117	0.409985	BGIOSGA031358	XP_015697328.1PREDICTED: uncharacterized protein LOC102719988
TCONS_00020226	0.44273	1.87482	0.320481	BGIOSGA022418	NP_001152138.1leucoanthocyanidin dioxygenase
TCONS_00022995	0.442777	-0.584934	-0.16297	#N/A	#N/A
TCONS_00025582	0.442908	-3.30588	-0.435631	BGIOSGA026223	XP_006658037.1 dirigent protein 5-like
TCONS_00017163	0.443256	0.397094	0.564718	BGIOSGA019209	XP_006654062.1 U1 small nuclear ribonucleoprotein A
TCONS_00001316	0.443388	1.27849	0.273653	BGIOSGA037095	XP_006644347.1 bidirectional sugar transporter SWEET6b
TCONS_00004364	0.44348	2.48783	2.01039	#N/A	#N/A
TCONS_00016788	0.44357	0.44819	1.08055	BGIOSGA014179	XP_014750930.1transcription factor IBH1
TCONS_00006967	0.443699	-0.885969	0.883748	BGIOSGA022655	XP_006649098.1 probable polygalacturonase
TCONS_00013662	0.444086	0.902113	0.62553	BGIOSGA015822	XP_004975033.1uncharacterized protein LOC101763724
TCONS_00024792	0.444259	-0.568943	0.524286	BGIOSGA024576	XP_003561285.2glycerol-3-phosphate dehydrogenase [NAD(+)], chloroplastic isoform X1
TCONS_00035470	0.444694	-1.44322	-1.10312	BGIOSGA036879	XP_006663744.1 probable non-specific lipid-transfer protein 3
TCONS_00007530	0.445068	1.09875	0.461332	BGIOSGA009663	XP_006647019.2 zinc finger A20 and AN1 domain-containing stress-associated protein 4
TCONS_00023799	0.445204	-1.72378	-0.730923	BGIOSGA025836	XP_006657776.1PREDICTED: uncharacterized protein LOC102707981
TCONS_00013072	0.445239	0.137945	0.475289	BGIOSGA009909	XP_006650504.1 putative 3,4-dihydroxy-2-butanone kinase isoform X1
TCONS_00023691	0.445463	-1.11825	-1.2677	BGIOSGA025723	NP_001146339.1uncharacterized LOC100279915
TCONS_00001115	0.445467	0.555996	1.04602	BGIOSGA003737	XP_006645991.1 F-box protein SKIP8
TCONS_00015158	0.445467	-0.0611472	-0.2868	BGIOSGA019310	XP_006653016.1 ubiquinone biosynthesis protein COQ4 homolog, mitochondrial
TCONS_00016320	0.445499	0.303616	-0.420188	BGIOSGA012986	XP_006653623.2 elongation factor G-2, chloroplastic

TCONS_00008780	0.445555	0.0599959	-0.0872173	BGIOSGA022620	XP_006647864.1 pyruvate dehydrogenase E1 component subunit alpha-1, mitochondrial
TCONS_00014462	0.445866	2.00891	0.382592	BGIOSGA008549	XP_006652429.1 uncharacterized membrane protein At3g27390-like isoform X2
TCONS_00010348	0.445876	1.22921	1.0689	BGIOSGA012571	XP_006650229.1 xanthine dehydrogenase
TCONS_00029281	0.446047	0.0704157	0.910129	BGIOSGA031023	XP_006660789.1 ER membrane protein complex subunit 3
TCONS_00016977	0.446221	-1.74422	0.64841	BGIOSGA019329	XP_010231853.1general transcription and DNA repair factor IIH subunit TFB5
TCONS_00026371	0.446221	0.798169	0.526049	BGIOSGA026750	XP_006659294.1 heterogeneous nuclear ribonucleoprotein 1
TCONS_00029391	0.446369	-1.68465	-0.947059	BGIOSGA031142	XP_006660884.1PREDICTED: uncharacterized protein LOC102721765 isoform X1
TCONS_00019008	0.446402	-0.0248546	0.38155	BGIOSGA003596	XP_008674833.1uncharacterized protein LOC103651017 isoform X3
TCONS_00011804	0.4465	0.190231	0.721349	BGIOSGA005977	XP_006647573.1 DEAD-box ATP-dependent RNA helicase 47A
TCONS_00033907	0.446518	0.510658	1.01231	BGIOSGA035600	XP_015698148.1 GTPase Der
TCONS_00019908	0.446751	-0.0705767	-0.319187	BGIOSGA017455	XP_006654869.1 ATP-dependent Clp protease proteolytic subunit-related protein 1, chloroplastic
TCONS_00003264	0.446773	1.11291	0.98702	BGIOSGA001951	XP_006644021.1 NAC domain-containing protein 74-like isoform X1
TCONS_00028829	0.446903	1.11808	-0.540893	BGIOSGA013642	XP_008651015.2putative receptor-like protein kinase family protein isoform X1
TCONS_00030804	0.446997	-0.318754	-0.84871	BGIOSGA025952	XP_003564044.1UDP-glycosyltransferase 73C5
TCONS_00007464	0.446998	-1.50366	-0.905344	BGIOSGA028747	XP_006646979.2 chorismate mutase 2-like
TCONS_00010473	0.446998	0.595879	1.48697	BGIOSGA035577	XP_006650290.1 E3 ubiquitin-protein ligase At3g02290-like
TCONS_00003982	0.447088	-0.544589	-0.816726	BGIOSGA001853	XP_014753532.1acyl transferase 5
TCONS_00036072	0.447179	-0.277452	1.03835	BGIOSGA008681	XP_004972657.1receptor kinase-like protein Xa21
TCONS_00030726	0.44718	-1.40435	-0.735874	BGIOSGA011174	XP_014660130.1F-box/LRR-repeat protein At2g29930
TCONS_00019028	0.44736	0.0216486	0.728977	BGIOSGA018331	XP_006654248.1PREDICTED: uncharacterized protein LOC102707586 isoform X1
TCONS_00007452	0.447569	0.445893	0.691649	BGIOSGA007031	XP_006648369.1 CASP-like protein 2C2
TCONS_00032534	0.447578	0.982858	1.33832	BGIOSGA031581	XP_006661933.1 chaperone protein dnaJ 13 isoform X2
TCONS_00020127	0.447601	0.00478013	-0.400619	BGIOSGA013553	XP_006656641.1 cysteine synthase-like
TCONS_00025605	0.447761	-0.267584	0.432599	BGIOSGA003276	XP_006663861.1 gamma carbonic anhydrase 2, mitochondrial
TCONS_00022232	0.447777	-1.13425	0.383488	BGIOSGA021882	XP_006656067.1 mitogen-activated protein kinase 11 isoform X1
TCONS_00005091	0.4478	-0.077971	0.262578	BGIOSGA000114	XP_006645394.1 pyrroline-5-carboxylate reductase
TCONS_00019292	0.447907	1.54889	1.36719	BGIOSGA026248	XP_006655320.2 bZIP transcription factor 17-like
TCONS_00037550	0.447951	-0.559349	-0.515149	BGIOSGA009687	XP_006664214.1 protein IQ-DOMAIN 1-like isoform X2
TCONS_00014708	0.448165	#NA	-0.738333	BGIOSGA002747	XP_006653648.1 ethylene-responsive transcription factor ERF039-like
TCONS_00000672	0.448174	-0.616786	-0.20104	BGIOSGA018469	XP_006644062.1 magnesium-protoporphyrin IX monomethyl ester [oxidative] cyclase, chloroplastic
TCONS_00021537	0.4482	0.272431	0.537215	BGIOSGA022013	XP_006655745.2 nudix hydrolase 14, chloroplastic-like
TCONS_00033510	0.448211	0.0708428	0.135641	BGIOSGA035304	XP_006662944.1 SUMO-activating enzyme subunit 1A-like
TCONS_00008525	0.448286	0.783665	1.1248	BGIOSGA005902	XP_015688810.1 F-box protein At5g51380-like
TCONS_00031216	0.448498	0.999974	0.508765	BGIOSGA033036	XP_004983002.1 translin
TCONS_00017254	0.448574	1.27334	0.854347	BGIOSGA015509	XP_006655064.2PREDICTED: uncharacterized protein LOC102713187, partial
TCONS_00029020	0.448959	-0.578744	-0.688608	BGIOSGA004090	XP_004956842.1ubiquitin-NEDD8-like protein RUB1
TCONS_00011760	0.448975	-1.26874	-0.775175	BGIOSGA035931	XP_015689809.1 BTB/POZ domain-containing protein At5g67385
TCONS_00001752	0.449188	-0.100786	0.657669	BGIOSGA004388	XP_006644657.1 uncharacterized membrane protein sll0875
TCONS_00001471	0.449232	0.259136	0.317762	BGIOSGA012682	XP_006644440.1 GDSL esterase/lipase At1g28600-like isoform X2
TCONS_00002674	0.449521	0.15883	0.0463877	BGIOSGA005869	XP_006643651.1 peroxisomal membrane protein PEX14 isoform X1
TCONS_00002649	0.449528	0.60134	0.156935	BGIOSGA006955	XP_006645415.1 dnaJ protein P58IPK homolog
TCONS_00032209	0.449602	0.0255484	0.921056	BGIOSGA031928	XP_006661771.1 ribosomal RNA small subunit methyltransferase I isoform X2
TCONS_00008698	0.449625	#NA	-1.74197	BGIOSGA033111	XP_015688382.1 protein NRT1/ PTR FAMILY 7.3-like isoform X2
TCONS_00004570	0.449736	0.150911	0.535293	BGIOSGA000644	XP_006644850.1 bifunctional endo-1,4-beta-xylanase XylA
TCONS_00035887	0.449872	0.516541	-1.04644	BGIOSGA007558	XP_006663954.1 ribulose bisphosphate carboxylase small chain, chloroplastic-like
TCONS_00006877	0.449967	-0.326888	0.215547	BGIOSGA021621	XP_015689672.1 shikimate kinase 1, chloroplastic-like
TCONS_00017491	0.450207	0.180259	-0.761996	BGIOSGA019560	XP_006655169.1PREDICTED: uncharacterized protein LOC102709369
TCONS_00011706	0.450235	-0.128101	0.186131	BGIOSGA014986	XP_006651118.1 ubiquitin carboxyl-terminal hydrolase 3-like
TCONS_00034706	0.450363	-0.039465	-0.065254	BGIOSGA032520	XP_006662892.1 E3 ubiquitin-protein ligase At3g02290
TCONS_00030559	0.450512	-1.48948	0.420891	BGIOSGA029257	XP_015696739.1 GDSL esterase/lipase At5g03610-like
TCONS_00019101	0.450659	-1.12711	0.816049	BGIOSGA012136	XP_006654274.1 protein EARLY RESPONSIVE TO DEHYDRATION 15-like
TCONS_00018470	0.450722	0.865895	0.777113	BGIOSGA027714	XP_020395801.1actin-97 isoform X2

TCONS_00031390	0.450953	0.392644	0.131735	BGIOSGA033201	XP_006661905.1 protochlorophyllide reductase B, chloroplastic
TCONS_00019561	0.451071	0.813132	0.543955	BGIOSGA034558	XP_006654594.1 metacaspase-5-like
TCONS_00032942	0.451114	0.885041	1.07185	BGIOSGA036963	XP_015698210.1 2-isopropylmalate synthase A-like
TCONS_00012670	0.451134	0.822124	0.147524	BGIOSGA010290	XP_006651583.1 peptidyl-prolyl cis-trans isomerase E
TCONS_00001898	0.45116	0.811151	1.09595	BGIOSGA004532	XP_006646377.1 coenzyme Q-binding protein COQ10 homolog B, mitochondrial
TCONS_00030088	0.451247	0.872834	0.95459	BGIOSGA013814	XP_021309876.1transcription initiation factor TFIID subunit 10 isoform X2
TCONS_00007247	0.451283	0.69257	0.376779	BGIOSGA007671	XP_006646790.1 pentatricopeptide repeat-containing protein At2g01860
TCONS_00030320	0.451399	-0.217029	-0.0512195	BGIOSGA014305	XP_015696667.1 F-box/LRR-repeat protein At4g14103-like
TCONS_00018752	0.451509	1.82285	1.91633	BGIOSGA030710	XP_006654122.2 hexokinase-7
TCONS_00025651	0.451604	1.80419	1.36861	BGIOSGA023774	XP_015694879.1 RNA pseudouridine synthase 6, chloroplastic isoform X1
TCONS_00009098	0.451799	1.06045	0.560769	BGIOSGA005306	XP_006648174.1 beta-catenin-like protein 1
TCONS_00003334	0.451951	0.852668	0.771646	BGIOSGA036276	XP_015688256.1 cell division cycle protein 123 homolog
TCONS_00008424	0.452052	-1.97695	-0.898282	BGIOSGA006001	XP_006647544.1 phenylalanine ammonia-lyase
TCONS_00029951	0.452097	1.02946	1.65829	BGIOSGA019462	XP_006660590.1 leucine--tRNA ligase, cytoplasmic-like
TCONS_00021288	0.452272	-2.7327	-2.01156	BGIOSGA020502	XP_006656410.1 probable receptor-like serine/threonine-protein kinase At5g57670
TCONS_00005127	0.452338	0.0250044	0.292625	BGIOSGA000077	XP_015698877.1 lipid droplet-associated hydrolase isoform X5
TCONS_00030736	0.452348	1.04819	1.80934	BGIOSGA032547	XP_023157674.1uncharacterized protein LOC100279122 isoform X3
TCONS_00031298	0.452368	1.18226	1.4532	BGIOSGA019107	XP_006661850.1 heterogeneous nuclear ribonucleoprotein 1-like
TCONS_00035666	0.452391	0.246025	0.648383	BGIOSGA031260	XP_004977230.1replication factor C subunit 2
TCONS_00036692	0.452431	-0.735155	0.316666	BGIOSGA036619	XP_006663822.2 ACT domain-containing protein ACR12-like
TCONS_00012561	0.452795	0.324066	0.584255	BGIOSGA023617	XP_004982546.1protein FATTY ACID EXPORT 5
TCONS_00036273	0.452816	0.091798	0.339929	BGIOSGA035980	XP_004982818.1mitochondrial import inner membrane translocase subunit Tim9
TCONS_00030418	0.453093	-0.294617	0.578761	BGIOSGA028457	XP_006660882.1 polygalacturonate 4-alpha-galacturonosyltransferase-like
TCONS_00035429	0.453101	1.85122	0.135776	BGIOSGA017974	XP_006664261.2 pentatricopeptide repeat-containing protein At3g09650, chloroplastic-like
TCONS_00025716	0.453136	-0.00165795	0.838769	BGIOSGA012565	XP_004958710.1eukaryotic translation initiation factor 2 subunit beta
TCONS_00037052	0.453173	-2.14887	-2.33571	BGIOSGA015844	XP_004977747.2uncharacterized protein LOC101761830
TCONS_00006818	0.454541	0.125704	1.21721	BGIOSGA008992	XP_006649018.1 APO protein 2, chloroplastic
TCONS_00033310	0.454694	1.25539	0.565281	BGIOSGA022558	XP_015689208.1 polyadenylate-binding protein 2-like isoform X1
TCONS_00031528	0.454771	1.52156	0.715648	BGIOSGA033345	XP_006661993.1 lysine-specific histone demethylase 1 homolog 3
TCONS_00027003	0.454806	0.413088	0.196725	BGIOSGA016275	XP_006659624.1 protein NUCLEAR FUSION DEFECTIVE 4
TCONS_00037531	0.455111	0.852445	-0.0068537	BGIOSGA035572	XP_006664745.1 2-methyl-6-phytyl-1,4-hydroquinone methyltransferase 2, chloroplastic
TCONS_00006647	0.456251	0.492855	0.69613	BGIOSGA008421	XP_006648936.1 pentatricopeptide repeat-containing protein At1g11290, chloroplastic-like
TCONS_00015257	0.456611	-1.1025	0.43127	BGIOSGA015764	XP_003580691.1glyoxylate/hydroxypyruvate reductase HPR3 isoform X4
TCONS_00031258	0.456924	0.393366	0.0534514	BGIOSGA032579	XP_006662414.1 3-ketoacyl-CoA thiolase 2, peroxisomal-like
TCONS_00018681	0.457444	0.404305	0.526945	BGIOSGA010094	XP_004977129.1zinc transporter 6
TCONS_00013968	0.457682	1.48973	1.44132	BGIOSGA001726	XP_006652172.1 tRNA (adenine(58)-N(1))-methyltransferase catalytic subunit TRMT61A
TCONS_00008559	0.457698	-0.137372	0.586434	BGIOSGA014546	XP_010555194.1 protein LAZ1 isoform X3
TCONS_00012032	0.457805	0.00290273	0.318338	BGIOSGA010974	XP_015689847.1 protein SIEL
TCONS_00020858	0.458406	0.901521	1.35244	BGIOSGA024882	XP_015694291.1 shaggy-related protein kinase eta-like
TCONS_00035276	0.458519	-1.07891	-0.785807	BGIOSGA012277	XP_003575753.2probable aminotransferase TAT2 isoform X1
TCONS_00026268	0.458547	-1.05106	0.510318	#N/A	XP_006659261.1 probable DNA replication complex GINS protein PSF3
TCONS_00005130	0.458635	1.03955	0.563861	BGIOSGA000072	XP_015699266.1 tRNA uridine 5-carboxymethylaminomethyl modification enzyme MnmG
TCONS_00006262	0.458777	0.493209	-0.490475	BGIOSGA012235	XP_004952809.1ACT domain-containing protein ACR3
TCONS_00028432	0.458811	-2.93296	-0.238444	BGIOSGA005938	XP_015696048.1 kinesin-like protein NACK2 isoform X1
TCONS_00027206	0.45894	0.920668	0.828806	BGIOSGA027794	XP_003571293.1PHD finger protein EHD3
TCONS_00005682	0.458943	1.00955	0.796862	BGIOSGA007805	XP_006647077.1 elongation factor 1-gamma 2
TCONS_00026387	0.458995	0.987201	1.17327	BGIOSGA028455	XP_015695566.1 GMP synthase [glutamine-hydrolyzing]
TCONS_00032576	0.459088	1.03303	0.436377	BGIOSGA021029	XP_006661964.1 plant UBX domain-containing protein 10
TCONS_00019666	0.459297	0.257111	-1.15293	BGIOSGA033858	XP_002441420.2respiratory burst oxidase homolog protein A
TCONS_00020157	0.459475	0.475429	0.715927	BGIOSGA001018	XP_006655812.1 long chain acyl-CoA synthetase 4-like
TCONS_00031354	0.459524	0.514615	0.195696	BGIOSGA033167	XP_015697417.1 pre-mRNA-processing ATP-dependent RNA helicase PRP5
TCONS_00007739	0.459885	-0.427699	0.486472	BGIOSGA006742	XP_006647131.1 50S ribosomal protein L21, chloroplastic

transcriptome

TCONS_00017215	0.460049	0.386464	0.286026	BGIOSGA020929	XP_004960817.1adenine nucleotide transporter BT1, chloroplastic/mitochondrial
TCONS_00010977	0.460058	1.13297	0.52469	BGIOSGA013580	XP_003559369.1solute carrier family 25 member 44 isoform X1
TCONS_00008300	0.460058	0.357012	0.170407	BGIOSGA014873	XP_006647450.1 isocitrate dehydrogenase [NAD] regulatory subunit 1, mitochondrial-like isoform X1
TCONS_00006244	0.460096	0.633275	-0.0555883	BGIOSGA020673	XP_006647357.1 midasin-like
TCONS_00025064	0.460114	0.861556	0.12471	BGIOSGA006063	XP_003563153.1CDGSH iron-sulfur domain-containing protein NEET
TCONS_00007414	0.460361	1.28587	0.591391	BGIOSGA007060	XP_002453366.1uncharacterized protein LOC8077174
TCONS_00006709	0.460502	0.231167	-0.218381	BGIOSGA016743	XP_003572855.1probable tocopherol O-methyltransferase, chloroplastic
TCONS_00008826	0.460795	-0.325565	0.162695	BGIOSGA035305	XP_006647922.1 5'-adenylylsulfate reductase-like 3
TCONS_00025434	0.460879	-0.65637	0.270037	BGIOSGA023994	XP_004958172.1arabinosyltransferase RRA3
TCONS_00012752	0.460906	-1.40907	-1.06962	BGIOSGA022961	XP_006650317.1 protein DETOXIFICATION 33
TCONS_00025981	0.461651	0.728101	0.619312	BGIOSGA024943	XP_015695506.1 protein CHLORORESPIRATORY REDUCTION 6, chloroplastic
TCONS_00028272	0.462632	0.0912579	1.13047	BGIOSGA029548	XP_006660265.1 IRK-interacting protein-like
TCONS_00012819	0.462638	1.44673	1.8288	BGIOSGA035826	XP_015689991.1 probable translation initiation factor eIF-2B subunit delta
TCONS_00011507	0.462717	1.91556	-0.756016	BGIOSGA038595	XP_006649332.1 catalase-1
TCONS_00022790	0.462762	1.38445	1.24353	BGIOSGA020663	XP_015693684.1 calcium homeostasis endoplasmic reticulum protein
TCONS_00017851	0.462835	1.84371	0.603539	BGIOSGA031837	XP_015693041.1 poly(A) RNA polymerase gld-2 homolog B-like
TCONS_00025892	0.463048	0.980553	1.59135	BGIOSGA027964	XP_015695489.1 pentatricopeptide repeat-containing protein At5g50390, chloroplastic
TCONS_00012395	0.463171	2.02173	1.65276	BGIOSGA010590	XP_002465170.1probable inactive heme oxygenase 2, chloroplastic
TCONS_00004077	0.463272	-2.07172	0.136791	BGIOSGA013510	XP_015689423.1 protein trichome birefringence-like 38
TCONS_00028712	0.463273	0.16395	0.785613	BGIOSGA010478	XP_006660513.1 THO complex subunit 3
TCONS_00013046	0.463432	0.23299	-0.569771	BGIOSGA010352	XP_003560805.1uncharacterized protein LOC100828553
TCONS_00022872	0.46361	1.00818	0.483698	BGIOSGA007487	XP_015693701.1 probable histone acetyltransferase HAC-like 2
TCONS_00009042	0.463715	-0.192978	-0.821823	BGIOSGA005363	XP_006648112.1PREDICTED: uncharacterized protein LOC102718148
TCONS_00033744	0.464111	-0.0478029	-0.240958	BGIOSGA033682	XP_006663010.2 glutathione S-transferase T1
TCONS_00006944	0.464132	1.82209	0.608313	BGIOSGA009125	XP_015689394.1 protein NRDE2 homolog
TCONS_00003186	0.464234	0.429566	0.49698	BGIOSGA002029	XP_006643979.1PREDICTED: uncharacterized protein LOC102712301
TCONS_00000298	0.464284	0.248931	0.989896	BGIOSGA029245	XP_006645556.2PREDICTED: uncharacterized protein LOC102709890
TCONS_00031860	0.464529	-0.407382	0.212344	BGIOSGA030275	XP_006661643.1 aldose 1-epimerase-like
TCONS_00033874	0.464571	-0.0864598	-0.0253248	BGIOSGA031878	XP_004974997.1BTB/POZ and MATH domain-containing protein 1
TCONS_00028034	0.464637	0.11653	0.0818229	BGIOSGA029808	XP_006659414.1PREDICTED: uncharacterized protein LOC102710043
TCONS_00010799	0.464703	-0.271376	-0.783316	BGIOSGA013423	XP_006650475.1PREDICTED: uncharacterized protein LOC102708320
TCONS_00009192	0.464877	-0.119976	0.511789	BGIOSGA024944	XP_006649279.1 riboflavin biosynthesis protein PYRD, chloroplastic
TCONS_00005227	0.464893	-0.0792374	0.450957	BGIOSGA007337	XP_002451348.1putative nitric oxide synthase isoform X1
TCONS_00030525	0.464896	0.270227	0.234517	BGIOSGA007666	XP_006660968.1 probable protein phosphatase 2C 70 isoform X2
TCONS_00024074	0.465615	0.925173	0.870813	BGIOSGA034217	XP_015695173.1 PHD finger protein ALFIN-LIKE 9
TCONS_00006443	0.465642	1.22298	0.262178	BGIOSGA023320	XP_006647498.1 pentatricopeptide repeat-containing protein At3g16010
TCONS_00014728	0.465892	0.340202	0.345137	BGIOSGA005684	XP_006652223.1 U4/U6 small nuclear ribonucleoprotein Prp31
TCONS_00035322	0.465916	-0.0410674	1.07157	BGIOSGA033590	XP_006663094.1 translationally-controlled tumor protein homolog
TCONS_00008212	0.465972	-0.40176	0.434537	BGIOSGA022945	NP_001158931.2uncharacterized LOC100303836
TCONS_00029923	0.46629	1.02837	0.651584	BGIOSGA029884	XP_006660578.1 protein SCO1 homolog 2, mitochondrial
TCONS_00005590	0.466459	-0.522874	0.594098	BGIOSGA014904	XP_006647012.2 protein TIC 21, chloroplastic-like
TCONS_00036473	0.466536	-0.755158	-1.49002	BGIOSGA018238	XP_008644627.2 atherin
TCONS_00025003	0.466773	-0.944585	-0.00859164	BGIOSGA005571	XP_006659938.2 methionine aminopeptidase 1B, chloroplastic-like isoform X2
TCONS_00010606	0.466778	-0.162626	0.695693	BGIOSGA013228	XP_006650356.1 glutamyl-tRNA(Gln) amidotransferase subunit C, chloroplastic/mitochondrial
TCONS_00028435	0.46691	0.18944	-0.816201	BGIOSGA022038	XP_002444625.1geraniol 8-hydroxylase
TCONS_00006717	0.466916	0.516734	1.31933	BGIOSGA008886	XP_006648967.1 ornithine carbamoyltransferase, chloroplastic
TCONS_00001782	0.466935	0.984897	0.901877	BGIOSGA004420	XP_006644673.1 pentatricopeptide repeat-containing protein At4g32450, mitochondrial-like
TCONS_00001889	0.467104	1.52833	0.822484	BGIOSGA004523	XP_006644771.2PREDICTED: uncharacterized protein LOC102712032
TCONS_00019109	0.467206	0.47819	1.23352	BGIOSGA006573	XP_006654278.1 40S ribosomal protein S7
TCONS_00005951	0.467279	0.103374	0.709757	BGIOSGA008088	XP_015688389.1 coiled-coil domain-containing protein 130
TCONS_00027192	0.467335	-0.86518	0.0232212	BGIOSGA034737	XP_015696197.1 protein S-acyltransferase 8-like isoform X1
TCONS_00023964	0.467569	-0.53782	-0.551483	BGIOSGA037243	XP_006657878.2 pheophytinase, chloroplastic
TCONS_00030255	0.467629	-0.157625	1.37258	BGIOSGA012100	XP_015696523.1 probable polygalacturonase

TCONS_00007141	0.467694	1.71854	-1.56888	BGIOSGA026130	XP_006649189.1 remorin
TCONS_00011545	0.4679	0.384242	1.0253	BGIOSGA026845	XP_006651028.1PREDICTED: uncharacterized protein LOC102705992
TCONS_00012298	0.467904	-0.451577	0.413142	BGIOSGA010698	XP_006651399.1 branched-chain-amino-acid aminotransferase-like protein 1
TCONS_00033089	0.468073	-1.03917	0.260328	BGIOSGA034933	XP_014758273.1protein CELLULOSE SYNTHASE INTERACTIVE 3 isoform X2
TCONS_00033804	0.468126	1.70047	-0.891202	BGIOSGA012751	XP_006663032.1 40S ribosomal protein S9-2-like
TCONS_00025620	0.468196	-0.533556	-0.0506139	BGIOSGA037767	XP_021310867.1uncharacterized protein LOC8082175
TCONS_00008998	0.468221	0.427143	0.353793	BGIOSGA029759	XP_015689350.1 transcription factor PIL1-like
TCONS_00005429	0.468405	0.295746	0.374941	BGIOSGA026672	XP_006646884.1PREDICTED: uncharacterized protein LOC102711040
TCONS_00002831	0.468774	0.118488	-0.0894327	BGIOSGA002388	XP_015688262.1 protein TRM32 isoform X1
TCONS_00020553	0.468806	-1.89552	-0.358147	BGIOSGA004012	XP_006656895.1 UDP-glycosyltransferase 708A6-like
TCONS_00037015	0.468965	0.744793	0.658585	BGIOSGA006979	XP_004962596.1protein ABHD17B
TCONS_00004961	0.468975	0.02209	0.318884	BGIOSGA010966	XP_002458980.1peptidyl-prolyl cis-trans isomerase FKBP15-1
TCONS_00031981	0.469005	-1.03996	-0.584331	BGIOSGA032667	XP_015697395.1PREDICTED: uncharacterized protein LOC107305181
TCONS_00030277	0.469192	-1.12214	0.118257	BGIOSGA003282	XP_006660780.1 cinnamoyl-CoA reductase 1-like
TCONS_00014942	0.469333	2.81047	2.27493	BGIOSGA017124	XP_006653765.2 protein argonaute 2-like
TCONS_00008484	0.469353	-0.804747	-0.151428	BGIOSGA023195	XP_006663091.1 palmitoyl-acyl carrier protein thioesterase, chloroplastic-like
TCONS_00003999	0.469478	0.195159	1.26091	BGIOSGA003134	XP_015691633.1 protein SEH1
TCONS_00018116	0.469478	-1.60484	-1.35881	BGIOSGA031705	XP_015692660.1PREDICTED: uncharacterized protein LOC102720710
TCONS_00014573	0.46961	0.601631	1.02453	BGIOSGA000017	XP_003581407.1isocitrate dehydrogenase [NADP]
TCONS_00031363	0.46986	-2.56951	#NA	BGIOSGA037599	XP_008663663.1putative ripening-related protein 5
TCONS_00020990	0.469963	0.435855	0.75444	BGIOSGA006921	XP_004965953.1uncharacterized protein At2g34160
TCONS_00019641	0.470031	2.10029	1.19752	BGIOSGA017716	XP_015693234.1 vesicle-fusing ATPase
TCONS_00025038	0.470643	0.204808	0.792721	BGIOSGA024368	XP_015695269.1 luc7-like protein 3
TCONS_00013167	0.470891	0.563593	0.617055	BGIOSGA009817	XP_006651815.1PREDICTED: uncharacterized protein LOC102713345
TCONS_00023069	0.471018	0.30007	0.0205436	BGIOSGA025099	XP_006657427.1 peptidyl-prolyl cis-trans isomerase FKBP19, chloroplastic
TCONS_00001978	0.471237	0.707272	0.252478	BGIOSGA021947	XP_006644843.2 protein CHLOROPLAST ENHANCING STRESS TOLERANCE, chloroplastic
TCONS_00020176	0.471365	0.0346737	0.994202	BGIOSGA004646	XP_015693459.1 protein SRG1
TCONS_00013553	0.4714	-3.57143	#NA	BGIOSGA029432	XP_006650911.1 mucin-1-like
TCONS_00037304	0.471411	0.686636	0.519404	BGIOSGA012381	XP_006649898.1PREDICTED: uncharacterized protein LOC102704684
TCONS_00016864	0.471645	0.510318	0.126679	BGIOSGA027827	XP_015692279.1PREDICTED: uncharacterized protein LOC102716962
TCONS_00025508	0.471743	0.310781	0.668839	BGIOSGA006176	NP_001147727.2nuclear transcription factor Y subunit B-3
TCONS_00006286	0.471761	-0.436882	-0.637197	BGIOSGA000027	XP_002452300.1CLP protease regulatory subunit CLPX1, mitochondrial
TCONS_00033262	0.471915	0.0339808	-0.485209	BGIOSGA035085	XP_015697783.1 F-box/LRR-repeat protein 12 isoform X1
TCONS_00029428	0.471947	-2.18553	-2.30656	BGIOSGA005878	XP_006660913.1 probable aquaporin PIP2-7
TCONS_00036445	0.472031	0.704682	0.962674	BGIOSGA001026	XP_006664758.1 ubiquitin carboxyl-terminal hydrolase 8-like
TCONS_00029824	0.472433	0.0335415	0.0998887	BGIOSGA009173	XP_006660535.1 E3 ubiquitin-protein ligase At1g63170-like isoform X1
TCONS_00013113	0.472785	0.153439	0.35626	BGIOSGA009280	NP_001147842.1BSD domain containing protein
TCONS_00013303	0.472972	0.0543445	0.742144	BGIOSGA033772	XP_006650704.1 E3 ubiquitin-protein ligase RHF2A-like
TCONS_00032407	0.473313	-0.127332	0.464185	BGIOSGA011812	XP_006661853.2 DExH-box ATP-dependent RNA helicase DExH1
TCONS_00035641	0.473375	-3.6754	-2.95548	BGIOSGA023247	XP_002442893.1fructose-bisphosphate aldolase, chloroplastic
TCONS_00025536	0.473407	-0.136883	-0.311994	BGIOSGA012794	XP_006657970.1 ADP-ribosylation factor-related protein 1-like
TCONS_00012834	0.473466	0.937782	1.0663	BGIOSGA010136	XP_006650360.1 protein PNS1-like isoform X1
TCONS_00011154	0.473471	1.22439	1.38394	BGIOSGA037943	XP_006650747.1 vesicle-associated membrane protein 721-like
TCONS_00035812	0.473516	-0.326177	#NA	BGIOSGA024755	XP_004960698.1TLC domain-containing protein At5g14285
TCONS_00000354	0.47359	0.532834	0.640392	BGIOSGA002930	XP_006643862.1 mitochondrial-processing peptidase subunit alpha-like
TCONS_00000990	0.473654	-0.604033	0.578542	BGIOSGA003590	XP_006644195.1 CRS2-associated factor 1, chloroplastic-like
TCONS_00024962	0.473751	0.61995	1.20358	BGIOSGA024432	XP_006657708.1 mitochondrial import inner membrane translocase subunit TIM44-2-like isoform X1
TCONS_00022267	0.473912	0.0103577	0.152447	BGIOSGA021223	XP_010227690.1protein SAWADEE HOMEODOMAIN HOMOLOG 2
TCONS_00036350	0.474162	1.36626	0.909705	BGIOSGA035901	XP_008382314.1 SKP1-like protein 1A
TCONS_00021439	0.474176	1.83593	1.43123	BGIOSGA018990	NP_001149564.1OB-fold nucleic acid binding domain containing protein
TCONS_00004634	0.474234	1.33188	0.151643	BGIOSGA019930	XP_006644917.2 protein FATTY ACID EXPORT 3, chloroplastic
TCONS_00005661	0.474385	1.06865	1.03879	BGIOSGA030634	XP_006651073.2 nuclear ribonuclease Z
TCONS_00025004	0.474394	-0.311126	-0.602428	BGIOSGA024395	XP_006659218.1 26S proteasome non-ATPase regulatory subunit 8 homolog A
TCONS_00000834	0.47446	-1.35482	-0.402179	BGIOSGA014682	XP_006644137.1 serine carboxypeptidase-like 33

TCONS_00002377	0.474536	0.704303	0.949106	BGIOSGA036374	XP_006645195.1 lysine-specific demethylase JMJ705-like
TCONS_00008988	0.474795	0.591578	0.618927	BGIOSGA028749	XP_006648063.1 calcineurin subunit B-like
TCONS_00000651	0.475044	1.1015	1.42301	BGIOSGA003235	XP_002455437.1RPII140-upstream gene protein
TCONS_00025307	0.475206	1.71262	0.974139	BGIOSGA000690	XP_004957966.1universal stress protein PHOS34
TCONS_00013533	0.475273	-0.928256	0.900077	BGIOSGA020605	XP_006665034.1 DNA-directed RNA polymerases II, IV and V subunit 8B-like
TCONS_00018721	0.475333	0.152702	0.0846564	BGIOSGA013925	XP_006654101.1 serine/threonine protein phosphatase 2A 57 kDa regulatory subunit B' iota isoform-like
TCONS_00009512	0.475466	-0.784753	0.662836	BGIOSGA012060	XP_015689805.1PREDICTED: uncharacterized protein LOC102707941
TCONS_00037407	0.475466	0.862945	0.279507	#N/A	#N/A
TCONS_00004459	0.475539	-1.19277	0.769983	BGIOSGA000762	XP_006644760.2 formamidase-like
TCONS_00011884	0.475763	-5.57694	-1.47365	BGIOSGA033514	XP_003558366.1thaumatin-like protein 1 isoform X1
TCONS_00020342	0.475813	0.592919	0.147709	BGIOSGA027710	XP_003564107.1bZIP transcription factor 46
TCONS_00021886	0.476001	-1.81433	-0.719252	BGIOSGA021638	XP_020399667.1protein CELLULOSE SYNTHASE INTERACTIVE 1 isoform X1
TCONS_00012368	0.476002	-0.251877	-0.260118	BGIOSGA010619	XP_006650142.1 zinc finger CCCH domain-containing protein 15 homolog
TCONS_00007122	0.476149	0.193569	0.599974	BGIOSGA032681	XP_006648134.1 bet1-like SNARE 1-1 isoform X1
TCONS_00032240	0.476186	-1.35508	0.487819	BGIOSGA031900	XP_012699917.1CCR4-NOT transcription complex subunit 7-like
TCONS_00018477	0.476398	-1.99278	0.731219	BGIOSGA028661	XP_021312951.1uncharacterized protein LOC110433976
TCONS_00006328	0.476557	0.00701838	-0.176066	BGIOSGA033132	XP_015688647.1 NAC domain-containing protein 92-like
TCONS_00012038	0.476651	-1.18575	0.467554	BGIOSGA010967	XP_015691241.1 protein starmaker isoform X1
TCONS_00027316	0.476757	0.215165	-0.621421	BGIOSGA004584	XP_006659104.1 probable NAD(P)H dehydrogenase (quinone) FQR1-like 1
TCONS_00033905	0.476832	1.08196	0.82325	BGIOSGA033112	XP_006663067.1 heterogeneous nuclear ribonucleoprotein 1
TCONS_00004358	0.476841	0.45832	0.760907	BGIOSGA005244	XP_006644677.1 mitochondrial-processing peptidase subunit alpha-like
TCONS_00006912	0.477034	-0.119113	0.910213	BGIOSGA031312	XP_015689460.1 peptidyl-prolyl cis-trans isomerase CYP40-like
TCONS_00030327	0.47722	0.594957	1.59236	BGIOSGA029478	XP_002462625.2oleoyl-acyl carrier protein thioesterase 1, chloroplastic
TCONS_00013291	0.477323	1.55037	1.28146	BGIOSGA030510	XP_003559354.1protein WHAT'S THIS FACTOR 1
TCONS_00004096	0.477491	-1.7995	-0.464545	BGIOSGA001092	XP_015698979.1 myosin heavy chain, cardiac muscle isoform-like
TCONS_00010376	0.477523	1.06087	0.274416	#N/A	#N/A
TCONS_00001664	0.477645	0.0217451	-0.158291	BGIOSGA001412	XP_006644586.1 vesicle transport v-SNARE 13
TCONS_00002994	0.477767	1.54982	0.820933	BGIOSGA019949	XP_006645556.2PREDICTED: uncharacterized protein LOC102709890
TCONS_00014968	0.478132	-1.60516	-0.760693	BGIOSGA017162	XP_015691580.1 putative disease resistance protein At3g14460
TCONS_00003671	0.47822	-0.455252	0.941292	BGIOSGA025538	XP_006644228.1 probable ribosome biogenesis protein RLP24
TCONS_00016210	0.478262	0.789144	-0.0103644	BGIOSGA025437	XP_006653582.2 probable leucine-rich repeat receptor-like protein kinase At5g63930
TCONS_00013200	0.4784	2.03498	0.966233	BGIOSGA034895	XP_006650609.1 LRR receptor-like serine/threonine-protein kinase RPK2
TCONS_00031870	0.478479	1.2875	-0.770967	BGIOSGA023892	XP_006661644.1 alcohol dehydrogenase-like 4
TCONS_00018060	0.478647	0.852979	1.20465	BGIOSGA020108	XP_015693020.1PREDICTED: uncharacterized protein LOC102714291
TCONS_00011415	0.478732	-0.393736	0.344224	BGIOSGA033410	XP_006650955.1 branched-chain-amino-acid aminotransferase 5, chloroplastic-like
TCONS_00034114	0.478802	0.948395	0.391539	BGIOSGA017337	XP_006664264.1 protein transport protein Sec61 subunit beta
TCONS_00025370	0.478911	0.141306	0.814303	BGIOSGA024060	XP_006657861.1PREDICTED: uncharacterized protein LOC102708909
TCONS_00029505	0.479311	0.844857	-0.0129554	BGIOSGA031259	XP_003576836.1transcription termination factor MTERF2, chloroplastic
TCONS_00029900	0.479325	0.687555	1.1899	BGIOSGA037321	XP_004956642.1probable protein arginine N-methyltransferase 1
TCONS_00020133	0.479335	0.551774	-0.446245	BGIOSGA032149	XP_006655796.1 profilin LP04
TCONS_00029243	0.479355	0.471649	-0.75003	BGIOSGA015776	XP_015696656.1 wall-associated receptor kinase 3-like
TCONS_00014739	0.479372	-1.24621	-0.385051	BGIOSGA016916	XP_006652639.1PREDICTED: uncharacterized protein LOC102720977
TCONS_00009462	0.479506	1.25629	0.560801	BGIOSGA012009	XP_020400923.1DDB1- and CUL4-associated factor 8 isoform X1
TCONS_00004736	0.479522	-0.0442182	-0.166896	BGIOSGA009780	XP_006645027.2 alpha-glucan phosphorylase, H isozyme
TCONS_00015071	0.479615	0.144219	-0.0193953	BGIOSGA031869	XP_004977052.1protein COFACTOR ASSEMBLY OF COMPLEX C SUBUNIT B CCB4, chloroplastic isoform X1
TCONS_00004405	0.479967	0.0374416	-0.0444913	BGIOSGA020190	XP_006644715.1 28 kDa heat- and acid-stable phosphoprotein
TCONS_00019195	0.479992	0.819837	0.608	BGIOSGA018155	XP_006655262.1 conserved oligomeric Golgi complex subunit 5
TCONS_00002621	0.480014	1.21008	#NA	BGIOSGA019022	XP_015691648.1 transcription initiation factor IIA subunit 2-like
TCONS_00000746	0.480014	#NA	-0.933393	BGIOSGA037323	XP_006644113.1PREDICTED: uncharacterized protein LOC102703072
TCONS_00001353	0.480074	-0.301979	0.836267	BGIOSGA003985	XP_006644364.1PREDICTED: uncharacterized protein LOC102709035
TCONS_00009123	0.480089	0.0154533	1.01837	BGIOSGA016876	XP_006650944.1 deSI-like protein At4g17486 isoform X1

transcriptome

TCONS_00033201	0.480214	0.932034	0.607454	BGIOSGA034264	XP_006662838.2 disease resistance protein RPM1-like
TCONS_00014549	0.480253	-1.03962	0.128437	BGIOSGA029754	XP_004976128.1uncharacterized protein LOC101782624
TCONS_00025913	0.480432	0.520867	0.795416	BGIOSGA027513	XP_006659107.1 tyrosine--tRNA ligase 1, cytoplasmic-like
TCONS_00002472	0.481051	0.230311	0.515682	BGIOSGA005116	XP_006646650.2PREDICTED: uncharacterized protein LOC102721046
TCONS_00003182	0.481428	0.667618	-0.33193	BGIOSGA034240	XP_015699268.1 probable transcription factor GLK2
TCONS_00021743	0.481796	1.20659	0.22723	BGIOSGA021778	XP_002437966.1protein RTF2 homolog
TCONS_00016299	0.481862	1.34431	0.711298	BGIOSGA026016	XP_006652551.1 probable polyol transporter 4
TCONS_00020643	0.481865	1.1925	0.854529	BGIOSGA020042	XP_006656045.1 target of Myb protein 1-like
TCONS_00037033	0.481883	0.46986	0.646486	BGIOSGA036343	XP_006663959.2 SAGA-associated factor 29-like
TCONS_00013613	0.482189	0.538811	0.833741	BGIOSGA005307	XP_004954520.1large ribosomal RNA subunit accumulation protein YCED homolog 1, chloroplastic
TCONS_00005508	0.482506	0.334183	0.793699	BGIOSGA007622	XP_006648352.2 porphobilinogen deaminase, chloroplastic
TCONS_00004116	0.482518	0.216371	0.368972	BGIOSGA020381	XP_008374426.1 iron-sulfur cluster assembly protein 1-like
TCONS_00021088	0.482764	-1.16734	0.800574	BGIOSGA023316	NP_001183612.1uncharacterized LOC100502206
TCONS_00019501	0.482781	#NA	-1.94024	BGIOSGA017856	XP_015692780.1 thioredoxin H5-like isoform X1
TCONS_00004024	0.482914	-0.697802	1.67678	BGIOSGA001170	XP_015693095.1 cytochrome P450 72A14-like
TCONS_00009849	0.483007	-0.302588	0.162759	BGIOSGA012399	XP_006649911.2 mitochondrial succinate-fumarate transporter 1
TCONS_00035417	0.483083	0.633894	0.396544	BGIOSGA023635	XP_006663134.1 transcription elongation factor B polypeptide 1
TCONS_00017900	0.483165	1.14916	0.108898	BGIOSGA002929	XP_006654480.1 60S acidic ribosomal protein P2B-like
TCONS_00019804	0.483287	-1.38554	-2.27848	BGIOSGA001130	XP_006654799.1 mitogen-activated protein kinase 7
TCONS_00019896	0.483665	-0.358546	0.63615	BGIOSGA017466	XP_006654856.1PREDICTED: uncharacterized protein LOC102719114
TCONS_00037367	0.483808	0.938919	1.24408	BGIOSGA023855	NP_001150742.1ribosomal protein S6 containing protein
TCONS_00003358	0.483829	1.15912	0.447947	BGIOSGA007733	XP_006644082.1 putative L-cysteine desulfhydrase 1
TCONS_00010013	0.484025	-0.168923	-0.562446	BGIOSGA010797	XP_006650040.1 eukaryotic translation initiation factor 2 subunit beta
TCONS_00032189	0.484281	-2.37949	-0.943946	BGIOSGA000252	XP_003558726.1uncharacterized protein LOC100827106
TCONS_00032790	0.484281	0.0566046	-0.100671	BGIOSGA031322	XP_006662110.1 probable NADH dehydrogenase [ubiquinone] 1 alpha subcomplex subunit 12
TCONS_00018235	0.484404	-0.69653	1.02912	BGIOSGA030434	XP_006654730.1 probable polygalacturonase At1g80170 isoform X2
TCONS_00011188	0.48465	0.930164	0.274259	BGIOSGA016994	XP_006650768.1 calcium-dependent protein kinase 32-like
TCONS_00019559	0.484788	-0.301191	-0.767097	BGIOSGA007623	XP_006654593.1 phosphoglycerate kinase, chloroplastic
TCONS_00002433	0.484911	0.483959	0.254714	BGIOSGA036535	XP_015690794.1 squamosa promoter-binding-like protein 2
TCONS_00023102	0.485575	-1.32203	-2.38733	BGIOSGA025136	XP_006657436.1 uncharacterized oxidoreductase At1g06690, chloroplastic-like isoform X2
TCONS_00027258	0.485662	0.593177	0.202666	BGIOSGA022657	XP_015695950.1 zinc finger protein CONSTANS-LIKE 4
TCONS_00010159	0.485788	0.93578	0.848243	BGIOSGA026028	XP_015689911.1PREDICTED: uncharacterized protein LOC102715002
TCONS_00021799	0.48582	-1.15025	-0.750953	BGIOSGA002188	NP_001142987.2uncharacterized protein LOC100275448
TCONS_00029149	0.48613	0.14481	-0.684495	BGIOSGA005551	XP_015696857.1 bZIP transcription factor TRAB1-like
TCONS_00024579	0.48632	0.743602	0.288478	BGIOSGA001476	XP_006657449.2PREDICTED: uncharacterized protein LOC102708350
TCONS_00029190	0.486378	-0.497229	0.199598	BGIOSGA026780	XP_006660723.1PREDICTED: uncharacterized protein LOC102720456
TCONS_00019329	0.486984	-2.48666	-0.280674	BGIOSGA015184	XP_006654430.1 UDP-glucuronate:xylan alpha-glucuronosyltransferase 1-like
TCONS_00008443	0.487326	0.177649	0.439238	BGIOSGA008057	XP_006647563.1PREDICTED: uncharacterized protein LOC102699931
TCONS_00006331	0.487737	0.424212	#NA	BGIOSGA016549	XP_006647418.1 MADS-box transcription factor 27
TCONS_00022412	0.487768	0.203983	0.238233	BGIOSGA019568	XP_006660340.1 solute carrier family 35 member F1-like
TCONS_00022645	0.487837	0.0843033	0.68739	BGIOSGA000720	XP_006656292.1 phosphoenolpyruvate phosphatase
TCONS_00019310	0.488087	0.161513	-0.11438	BGIOSGA018036	XP_008656278.1uncharacterized protein LOC103635650
TCONS_00000995	0.48821	0.521797	0.921725	BGIOSGA003594	XP_015688764.1 tyrosine--tRNA ligase, chloroplastic/mitochondrial
TCONS_00012889	0.488294	0.556583	0.133323	BGIOSGA004522	XP_006651667.2 regulatory protein NPR4-like
TCONS_00011157	0.4885	0.840227	0.272413	BGIOSGA006317	XP_006651928.1 exocyst complex component EXO84B-like
TCONS_00034163	0.488863	-0.296873	0.568188	BGIOSGA040457	XP_006664874.2 biogenesis of lysosome-related organelles complex 1 subunit 1-like
TCONS_00018881	0.489196	0.824928	1.00127	BGIOSGA027518	XP_004960657.1lactoylglutathione lyase GLX1
TCONS_00012197	0.489325	0.136056	0.746136	BGIOSGA017838	XP_006650029.1 probable galacturonosyltransferase 7
TCONS_00012252	0.489371	1.14892	0.555174	BGIOSGA010753	XP_004984415.1ATP-dependent Clp protease proteolytic subunit-related protein 3, chloroplastic

TCONS_00003924	0.489436	0.227188	0.266612	BGIOSGA020252	NP_001278722.1hydrogen-transporting ATP synthase, rotational mechanism
TCONS_00001339	0.489496	0.051324	1.20335	BGIOSGA003971	XP_004969131.1protein ANTI-SILENCING 1 isoform X1
TCONS_00019506	0.489566	0.784837	1.77982	#N/A	#N/A
TCONS_00007821	0.489567	1.45126	1.1395	BGIOSGA020255	XP_006647183.2PREDICTED: uncharacterized protein At4g37920, chloroplastic
TCONS_00025182	0.489914	0.101147	0.0919199	BGIOSGA014972	XP_015695135.1 tRNA wybutosine-synthesizing protein 2/3/4
TCONS_00005659	0.490122	0.828695	0.232757	BGIOSGA007781	XP_006650973.1 kinesin-II 85 kDa subunit
TCONS_00021627	0.490337	-0.518144	-0.17428	BGIOSGA021890	XP_010060731.1PREDICTED: uncharacterized protein LOC104448575
TCONS_00007419	0.490391	0.10665	1.20447	BGIOSGA009211	XP_015689144.1 CAX-interacting protein 4 isoform X2
TCONS_00009433	0.490461	0.535318	0.18553	BGIOSGA029357	XP_006649501.1 protein TIFY 11c-like
TCONS_00025557	0.491203	0.117718	0.272855	BGIOSGA023876	XP_004958389.1uncharacterized protein LOC101762384
TCONS_00010949	0.491406	1.96271	0.962239	BGIOSGA013551	XP_006650577.1PREDICTED: uncharacterized protein LOC102715832
TCONS_00003590	0.491427	0.00514822	0.849648	BGIOSGA035493	XP_006644181.1PREDICTED: uncharacterized protein LOC102699722
TCONS_00003076	0.491777	1.17558	-1.00505	BGIOSGA002145	XP_006643895.1 heme-binding protein 2-like
TCONS_00032517	0.492139	0.163369	-0.282911	BGIOSGA002021	XP_006661925.1 membrane steroid-binding protein 1-like
TCONS_00002802	0.492276	0.78857	1.63001	BGIOSGA002414	XP_006643744.1PREDICTED: uncharacterized protein At2g39795, mitochondrial-like
TCONS_00012227	0.492527	0.438219	0.737409	BGIOSGA010782	XP_006650497.1 GPN-loop GTPase 3
TCONS_00034820	0.493081	0.579384	0.536612	BGIOSGA034046	XP_006662925.1 adenosylhomocysteinase
TCONS_00028974	0.493264	-0.0390025	0.387033	BGIOSGA008172	XP_004956778.1probable ubiquitin receptor RAD23
TCONS_00017888	0.493355	-0.275216	0.632312	BGIOSGA035880	XP_015693162.1 serine/threonine-protein kinase Nek4 isoform X1
TCONS_00018996	0.493423	1.07017	1.20299	#N/A	#N/A
TCONS_00030309	0.493461	-3.99374	-2.47712	BGIOSGA028477	XP_002462614.2cytochrome b561 and DOMON domain-containing protein At3g25290
TCONS_00030246	0.493534	-0.175604	-0.0976391	BGIOSGA032130	XP_002441416.1anthocyanidin 5,3-O-glucosyltransferase
TCONS_00010718	0.493711	0.645728	-0.168264	BGIOSGA013342	XP_006660850.1PREDICTED: uncharacterized protein LOC102711458
TCONS_00001952	0.493926	0.639937	-0.0296036	BGIOSGA024187	XP_006644823.1PREDICTED: uncharacterized protein LOC102703077 isoform X1
TCONS_00024116	0.494301	-0.972094	-0.165194	BGIOSGA032014	XP_006657966.1 probable calcium-binding protein CML13
TCONS_00019838	0.49442	1.14048	1.00882	BGIOSGA017519	XP_004961152.1ketol-acid reductoisomerase, chloroplastic
TCONS_00011668	0.494526	-4.21456	-1.93742	BGIOSGA005806	NP_001152422.1catalytic/ hydrolase
TCONS_00019366	0.494617	1.14145	1.28764	BGIOSGA004872	XP_002456695.1protein YIPF6 homolog
TCONS_00016874	0.494977	-0.329419	0.291115	BGIOSGA009826	XP_006653879.1PREDICTED: uncharacterized protein LOC102705906
TCONS_00016036	0.495076	0.0499967	0.626477	BGIOSGA015943	XP_015691488.1 CRM-domain containing factor CFM2, chloroplastic
TCONS_00014841	0.495237	0.43178	1.07553	BGIOSGA017016	XP_006652716.1 alpha-1,3/1,6-mannosyltransferase ALG2
TCONS_00035815	0.495351	1.24087	1.12186	BGIOSGA035736	XP_015698710.1 stromal 70 kDa heat shock-related protein, chloroplastic-like
TCONS_00010134	0.495377	-0.298094	0.282857	BGIOSGA023835	XP_006650113.1PREDICTED: uncharacterized protein LOC102720777
TCONS_00001891	0.495395	0.484785	0.495807	BGIOSGA014980	XP_002456408.1lipoyl synthase, chloroplastic
TCONS_00007036	0.495519	1.98345	0.612703	BGIOSGA005425	XP_004954249.1uncharacterized protein LOC101761145
TCONS_00018037	0.495608	0.678294	1.63984	BGIOSGA020084	XP_004961693.1NADH--cytochrome b5 reductase 1
TCONS_00020623	0.495627	0.193456	-0.282987	BGIOSGA022807	XP_006659877.1PREDICTED: uncharacterized protein At2g17340-like
TCONS_00032910	0.495783	-0.0729774	-0.542826	BGIOSGA011542	XP_009410447.1 CBL-interacting protein kinase 32 isoform X3
TCONS_00008369	0.495901	2.15523	0.308809	BGIOSGA037106	XP_006647504.1 temperature-induced lipocalin-1-like
TCONS_00009540	0.495935	-0.276582	-0.236349	BGIOSGA012086	NP_001149706.2MFP1 attachment factor 1
TCONS_00008844	0.496079	0.868641	0.89242	BGIOSGA022548	XP_015688816.1PREDICTED: uncharacterized protein LOC102718147 isoform X2
TCONS_00024635	0.496563	0.88711	1.0994	BGIOSGA024246	XP_006657482.1 probable polyribonucleotide nucleotidyltransferase 1, chloroplastic
TCONS_00008935	0.49672	1.65145	0.978897	BGIOSGA005472	XP_015688923.1PREDICTED: uncharacterized protein LOC102715734
TCONS_00008202	0.496763	1.42596	1.51504	BGIOSGA000906	XP_003575177.1trihelix transcription factor ASIL1
TCONS_00011868	0.496803	0.208916	1.04156	BGIOSGA011141	XP_006649709.1 translocase of chloroplast 34, chloroplastic
TCONS_00008566	0.496945	-0.340222	0.0584755	BGIOSGA025983	XP_006647673.1 probable serine/threonine-protein kinase WNK4 isoform X1
TCONS_00026524	0.496972	0.830762	0.254279	BGIOSGA025905	XP_006659365.1 probable transmembrane ascorbate ferrireductase 2
TCONS_00001762	0.497303	0.95723	0.923152	BGIOSGA012180	XP_006644662.1 GTP-binding protein At2g22870
TCONS_00004667	0.497422	-0.37049	0.249384	BGIOSGA000549	XP_006644954.2 probable phytol kinase 2, chloroplastic
TCONS_00014239	0.497434	1.01569	1.09977	BGIOSGA016402	XP_012702173.1G-type lectin S-receptor-like serine/threonine-protein kinase At2g19130
TCONS_00037221	0.497737	0.217	0.691143	BGIOSGA036154	XP_006664570.1 cell division topological specificity factor homolog, chloroplastic
TCONS_00032409	0.497947	1.00353	0.804132	BGIOSGA022792	XP_012698159.1DExH-box ATP-dependent RNA helicase DExH1 isoform X1
TCONS_00021129	0.498181	0.152506	1.30608	BGIOSGA022181	XP_002436816.1ent-isokaurene C2-hydroxylase

TCONS_00025554	0.498276	0.890655	0.605717	BGIOSGA006937	XP_020396135.150S ribosomal protein L12-2 isoform X1
TCONS_00005433	0.498276	1.30569	1.72119	BGIOSGA015308	XP_010240005.1probable inactive ATP-dependent zinc metalloprotease FTSHI 3, chloroplastic
TCONS_00034716	0.498504	-0.220387	0.678159	BGIOSGA000313	XP_006662885.1 protein RecA-like
TCONS_00000273	0.498788	-0.429393	-0.24755	BGIOSGA002852	XP_015692433.1 proactivator polypeptide-like 1
TCONS_00025239	0.498999	-0.019897	0.468644	BGIOSGA006556	XP_006657790.1 glycerol-3-phosphate acyltransferase 3
TCONS_00010805	0.499163	0.614345	0.0501283	BGIOSGA028862	XP_015690686.1 14-3-3-like protein GF14-F
TCONS_00020123	0.499305	1.11625	0.720215	BGIOSGA017653	XP_006656640.2 nucleosome assembly protein 1;1
TCONS_00014817	0.499318	-2.39598	1.03801	BGIOSGA029505	XP_015691796.1PREDICTED: uncharacterized protein LOC102714284
TCONS_00024001	0.499464	0.549938	0.862499	BGIOSGA026036	XP_006657898.2 SUMO-activating enzyme subunit 2
TCONS_00024099	0.499474	1.65975	-0.954717	BGIOSGA007437	XP_021308118.1membrane protein PTM1 isoform X2
TCONS_00027919	0.499809	-0.479785	-0.205868	BGIOSGA027073	XP_006659348.1 actin-related protein 2
TCONS_00007027	0.49984	0.725167	0.744385	BGIOSGA009209	XP_006648055.2 aspartate aminotransferase, chloroplastic
TCONS_00016785	0.500156	0.709069	-0.113518	BGIOSGA009926	XP_015692095.1 glutamine synthetase, chloroplastic
TCONS_00028213	0.500262	0.96862	-1.30648	BGIOSGA026771	XP_003574671.1haloacid dehalogenase-like hydrolase domain-containing protein At4g39970 isoform X3
TCONS_00011655	0.500403	1.11543	1.16872	BGIOSGA011354	XP_006649486.1 adenylosuccinate synthetase, chloroplastic
TCONS_00030514	0.500411	-0.968209	-0.133905	BGIOSGA029293	XP_006660958.1 protein ACCUMULATION AND REPLICATION OF CHLOROPLASTS 3 isoform X1
TCONS_00035447	0.500795	0.886697	1.11793	BGIOSGA034658	XP_006663150.1 F-box/LRR-repeat protein 3-like
TCONS_00028567	0.501065	-0.398085	0.821041	#N/A	#N/A
TCONS_00014715	0.501629	1.43457	1.20173	BGIOSGA016889	XP_006652614.1 T-complex protein 1 subunit alpha
TCONS_00019868	0.501701	-0.0769357	-0.987919	BGIOSGA017545	NP_001132742.1uncharacterized protein LOC100194229
TCONS_00026235	0.501735	1.16709	0.849253	BGIOSGA028312	XP_015695543.1 preprotein translocase subunit SECY, chloroplastic
TCONS_00021198	0.501752	-0.0857354	1.27225	BGIOSGA023417	XP_015694444.1 diphthamide biosynthesis protein 3
TCONS_00004503	0.501953	0.475922	1.17592	BGIOSGA000717	XP_015694747.1 ultraviolet-B receptor UVR8 isoform X2
TCONS_00028513	0.501958	0.0770611	0.54723	BGIOSGA030267	XP_004956278.1PRKR-interacting protein 1
TCONS_00036311	0.501998	-2.39923	0.333261	BGIOSGA036124	XP_002442393.1uncharacterized protein LOC8068140
TCONS_00030379	0.502149	0.399264	0.756217	BGIOSGA029419	XP_006660858.1 ATPase ASNA1 homolog
TCONS_00017631	0.502188	0.922463	1.33278	BGIOSGA003358	XP_008649526.1E3 ubiquitin-protein ligase EL5
TCONS_00012284	0.502251	-2.44607	-2.27845	BGIOSGA010721	XP_006651386.1 protein TIC 62, chloroplastic
TCONS_00024283	0.502311	0.90974	1.38507	BGIOSGA000386	XP_006658928.2 putative pentatricopeptide repeat-containing protein At5g40405
TCONS_00013177	0.502577	0.793965	0.540195	BGIOSGA009807	XP_002463874.1serine/threonine-protein kinase TOUSLED isoform X2
TCONS_00000629	0.502641	-1.81506	-0.182932	BGIOSGA006481	XP_003565628.1E3 ubiquitin-protein ligase EL5
TCONS_00006656	0.503025	1.51973	1.18918	BGIOSGA008825	XP_006647695.2PREDICTED: uncharacterized protein LOC102715823, partial
TCONS_00016264	0.503059	0.329709	1.10787	BGIOSGA014709	XP_015692336.1 protein root UVB sensitive 2, chloroplastic isoform X1
TCONS_00005497	0.503142	0.921276	0.330377	#N/A	#N/A
TCONS_00006199	0.503529	0.183825	1.1774	BGIOSGA000447	XP_010236802.1uncharacterized protein LOC104584271
TCONS_00034609	0.50375	1.71406	0.328847	BGIOSGA006609	XP_012701223.1uncharacterized protein LOC101785537
TCONS_00012666	0.503882	-0.243054	-0.401185	BGIOSGA010294	XP_010938821.2 disease resistance protein RGA2-like
TCONS_00005443	0.503943	0.207809	0.614487	BGIOSGA007550	XP_015689028.1 disease resistance protein RGA2-like
TCONS_00029532	0.504274	0.86068	0.600187	BGIOSGA015161	XP_002459830.1flagellar radial spoke protein 5 isoform X1
TCONS_00033574	0.504296	1.80735	2.8129	BGIOSGA035367	XP_004979379.1uncharacterized protein LOC101754431
TCONS_00033267	0.50458	-0.170165	0.0685272	BGIOSGA026623	XP_006663352.1 dehydrogenase/reductase SDR family member 7
TCONS_00024960	0.504635	1.10921	1.63453	BGIOSGA006759	NP_001149286.1spermidine synthase 1
TCONS_00009002	0.50472	0.67155	0.0305344	BGIOSGA005405	XP_015689552.1PREDICTED: uncharacterized protein slr1919
TCONS_00008702	0.50476	0.330005	0.363198	BGIOSGA005728	XP_006647800.1 60S ribosomal protein L31
TCONS_00036154	0.505048	0.488432	0.923459	BGIOSGA037510	XP_021313664.1phenylalanine--tRNA ligase, chloroplastic/mitochondrial
TCONS_00016896	0.505507	1.04949	0.199934	BGIOSGA014066	XP_006653893.2 DNA mismatch repair protein MSH3
TCONS_00032406	0.505572	0.12691	0.996331	BGIOSGA020171	XP_006661853.2 DExH-box ATP-dependent RNA helicase DExH1
TCONS_00022909	0.505603	-1.08183	-0.997733	BGIOSGA026221	XP_006656488.1 novel plant SNARE 11
TCONS_00037191	0.505665	1.53584	1.72314	BGIOSGA002555	XP_015698497.1 iron-sulfur assembly protein IscA-like 1, mitochondrial
TCONS_00015276	0.505865	0.614609	1.09863	BGIOSGA015707	XP_006652081.1 tRNA (adenine(58)-N(1))-methyltransferase non-catalytic subunit trm6
TCONS_00002103	0.505892	1.64027	0.460978	BGIOSGA018226	NP_001151099.3electron transporter
TCONS_00000256	0.506029	0.1537	0.0407318	BGIOSGA019238	XP_015693112.1 putative transcription factor bHLH107
TCONS_00034934	0.506068	0.593781	0.833431	BGIOSGA009088	XP_015698141.1 transport inhibitor response 1-like protein Os11g0515500

TCONS_00007055	0.506248	1.40278	1.0551	BGIOSGA009238	XP_003570348.1proliferating cell nuclear antigen
TCONS_00003413	0.506416	2.9315	0.678309	BGIOSGA007337	XP_003567596.1putative methyltransferase DDB_G0268948
TCONS_00028930	0.506799	1.02485	0.641547	BGIOSGA029835	XP_006661187.1 DEAD-box ATP-dependent RNA helicase 22
TCONS_00033022	0.50688	-0.668407	-1.00092	BGIOSGA034868	XP_006662757.1 disease resistance protein RGA2-like
TCONS_00019258	0.507214	0.200269	-0.530322	BGIOSGA025293	XP_006654374.1 UPF0603 protein Os05g0401100, chloroplastic
TCONS_00017410	0.50736	1.54822	2.48974	BGIOSGA015304	XP_006654194.1 xylanase inhibitor protein 2-like
TCONS_00003875	0.507696	0.939372	1.91988	BGIOSGA001337	XP_006646036.1 UBP1-associated proteins 1C
TCONS_00013249	0.507806	0.198288	-0.370421	BGIOSGA004733	XP_006650666.1 malate dehydrogenase, glyoxysomal-like
TCONS_00020954	0.507811	0.915381	-2.00324	BGIOSGA013519	XP_006656195.1 syntaxin-125-like
TCONS_00015606	0.50845	-0.0272104	0.740598	BGIOSGA031963	XP_020409078.1putative DUF593 domain containing family protein isoform X1
TCONS_00013215	0.508496	1.73555	1.36429	BGIOSGA009772	XP_006650631.2 protein arginine N-methyltransferase 2
TCONS_00036613	0.508673	0.530965	-0.42042	BGIOSGA036939	XP_006663786.1 aladin-like
TCONS_00005685	0.508706	1.35598	-0.413696	BGIOSGA007807	XP_006649870.1PREDICTED: uncharacterized protein LOC102720688
TCONS_00027392	0.508725	-0.0846496	0.618612	BGIOSGA020395	XP_006659148.1 THO complex subunit 7B-like isoform X1
TCONS_00008128	0.508786	-0.0218745	0.605482	BGIOSGA006308	XP_006647346.2 cysteine and histidine-rich domain-containing protein RAR1
TCONS_00019441	0.509005	1.06719	0.522304	BGIOSGA008061	XP_015692734.1 coatomer subunit zeta-1
TCONS_00033689	0.509147	1.14636	1.81787	BGIOSGA035465	XP_006663534.1 THUMP domain-containing protein 1 homolog
TCONS_00018132	0.509271	0.144153	0.545673	BGIOSGA000812	XP_006654644.1 auxin response factor 14
TCONS_00010777	0.509356	0.141702	-0.566507	BGIOSGA013404	XP_006650456.1 probable sugar phosphate/phosphate translocator At1g48230
TCONS_00001173	0.509422	0.530021	1.76535	BGIOSGA008366	XP_006644301.1 heat stress transcription factor A-6a
TCONS_00006179	0.509642	1.44913	2.24014	BGIOSGA031071	XP_004952615.1protein ROOT PRIMORDIUM DEFECTIVE 1
TCONS_00019928	0.509787	0.175924	0.31378	BGIOSGA028945	XP_022681842.1zinc finger CCCH domain-containing protein 43-like
TCONS_00009918	0.509832	0.231425	0.590968	BGIOSGA012468	XP_006649968.1 ubiquitin-conjugating enzyme E2 32 isoform X1
TCONS_00027582	0.510054	0.175589	1.12635	BGIOSGA027439	XP_015695716.1 chloroplast envelope membrane protein
TCONS_00002493	0.510165	1.17545	-0.42412	BGIOSGA019747	XP_006645307.1 eukaryotic peptide chain release factor subunit 1-2-like
TCONS_00007800	0.510165	1.56777	-1.00908	BGIOSGA029415	XP_004954806.1NAC domain-containing protein 48
TCONS_00016329	0.510174	0.316741	0.697219	BGIOSGA024244	XP_006652590.1 SH3 domain-containing protein 2-like
TCONS_00022903	0.510276	-0.875452	-0.299041	BGIOSGA020540	XP_006656484.1 NADH dehydrogenase [ubiquinone] 1 beta subcomplex subunit 9
TCONS_00005701	0.510429	0.880767	1.14854	BGIOSGA004305	XP_006647082.2 mitochondrial substrate carrier family protein B-like
TCONS_00025108	0.510655	1.55335	-2.01029	BGIOSGA017693	XP_012699299.17-deoxyloganetic acid glucosyltransferase
TCONS_00008690	0.510683	1.19864	1.5746	BGIOSGA004518	XP_015689011.1 AT-rich interactive domain-containing protein 3-like
TCONS_00010092	0.510827	-1.18795	0.863749	BGIOSGA016833	XP_015689898.1 protein IQ-DOMAIN 14-like
TCONS_00015452	0.510893	0.313083	-0.095889	BGIOSGA016491	XP_015691377.1 ABC transporter C family member 4-like
TCONS_00016904	0.510948	2.17102	1.92824	BGIOSGA013893	XP_006653055.1 protein YLS9-like
TCONS_00016677	0.511215	0.0221269	0.836319	BGIOSGA014292	XP_006652874.1 G-type lectin S-receptor-like serine/threonine-protein kinase B120
TCONS_00027870	0.511235	-0.685803	-0.83033	BGIOSGA024540	XP_003573789.1glucose-1-phosphate adenylyltransferase small subunit, chloroplastic/amyloplastic
TCONS_00025423	0.511472	1.02625	0.972581	BGIOSGA010486	XP_004958159.1probable amino-acid acetyltransferase NAGS2, chloroplastic
TCONS_00015938	0.511519	-0.498677	-0.640459	BGIOSGA006256	XP_006652315.1 DDT domain-containing protein DDB_G0282237-like
TCONS_00025803	0.511654	0.869597	0.575719	BGIOSGA027870	NP_001144170.1uncharacterized protein LOC100277025
TCONS_00018981	0.511931	-0.831311	1.34571	#N/A	XP_015693118.1 tRNA pseudouridine synthase A 1 isoform X1
TCONS_00016707	0.512559	-0.303277	-0.306679	BGIOSGA014256	XP_015691728.1PREDICTED: uncharacterized protein LOC102705433 isoform X2
TCONS_00013043	0.512569	0.646363	0.487486	BGIOSGA029477	XP_015690761.1 serine/threonine-protein kinase prp4
TCONS_00009862	0.512586	0.394803	-0.22656	BGIOSGA012413	XP_015690823.1 rab3 GTPase-activating protein catalytic subunit isoform X2
TCONS_00003581	0.513734	1.05636	0.215854	BGIOSGA025712	XP_002463987.1asparagine--tRNA ligase, cytoplasmic 1
TCONS_00000998	0.513957	0.338818	0.125759	BGIOSGA003599	XP_004968811.1uncharacterized PKHD-type hydroxylase At1g22950
TCONS_00018011	0.514018	0.221602	-0.0400386	BGIOSGA013329	XP_015692429.1 probable acetyltransferase NSI
TCONS_00008444	0.514149	0.804262	0.955085	BGIOSGA036130	XP_006648860.2 replication protein A 32 kDa subunit B-like
TCONS_00026138	0.514312	1.02402	0.8573	BGIOSGA009568	XP_006659220.1 serine/threonine-protein kinase HT1-like
TCONS_00026995	0.514688	0.0447102	-0.0591437	BGIOSGA036248	XP_006659588.1 sulfite oxidase
TCONS_00009479	0.515044	0.987663	0.86506	BGIOSGA012029	XP_015690978.1 ubiquitin carboxyl-terminal hydrolase 26
TCONS_00035478	0.515602	-1.4886	-0.229133	BGIOSGA019678	XP_015698440.1 2-hydroxy-6-oxononadienedioate/2-hydroxy-6-oxononatrienedioate hydrolase-like isoform X3

TCONS_00028263	0.516069	0.935085	1.12323	BGIOSGA005728	XP_006659566.1 60S ribosomal protein L31
TCONS_00019867	0.516372	0.420708	-0.386842	BGIOSGA020379	XP_006654835.1 sugar transporter ERD6-like 4
TCONS_00011522	0.516402	0.483673	-0.0974204	BGIOSGA036157	XP_004979720.1GRF1-interacting factor 3
TCONS_00015465	0.516452	0.841231	0.934883	BGIOSGA000592	XP_006663002.2 pentatricopeptide repeat-containing protein At4g14820-like
TCONS_00027287	0.516706	1.16069	-0.148127	BGIOSGA029320	XP_010230621.1glycine-rich protein 2
TCONS_00022474	0.516822	1.40665	1.92049	BGIOSGA030840	XP_006656189.1 probable histone deacetylase 19
TCONS_00009689	0.517067	0.46405	0.504977	BGIOSGA021998	XP_015690285.1 40S ribosomal protein S20-1-like
TCONS_00028461	0.517158	0.0214522	-1.60196	BGIOSGA010497	XP_006659689.1 probable uridine nucleosidase 1
TCONS_00007893	0.517221	-0.78983	0.12513	BGIOSGA006584	XP_006647219.1 WD repeat-containing protein 25
TCONS_00015051	0.517241	0.997383	0.999898	BGIOSGA017234	XP_006653817.1 NEDD8 ultimate buster 1
TCONS_00010812	0.517944	-0.745562	-0.309816	BGIOSGA035876	XP_006650492.1 ubiquitin-conjugating enzyme E2 variant 1D-like
TCONS_00024763	0.518087	0.521548	0.618912	BGIOSGA024614	XP_015694816.1 pentatricopeptide repeat-containing protein At2g41720
TCONS_00026601	0.518427	-0.495514	-0.303822	BGIOSGA030654	XP_006659395.1PREDICTED: uncharacterized protein LOC102704725
TCONS_00003332	0.518445	1.32831	1.51116	BGIOSGA034416	XP_006644070.1 coatomer subunit beta-2
TCONS_00029807	0.518575	2.11791	1.15762	BGIOSGA002978	XP_006660531.2 phosphoenolpyruvate carboxylase 2
TCONS_00001559	0.518604	0.469017	0.302467	BGIOSGA005360	XP_006644512.1 auxin-responsive protein IAA5
TCONS_00017371	0.518832	0.477948	0.649629	BGIOSGA011482	XP_006654169.2 putative ubiquitin-conjugating enzyme E2 38
TCONS_00014303	0.51889	0.275618	-0.567883	BGIOSGA008426	XP_015691466.1PREDICTED: uncharacterized protein LOC102702004, partial
TCONS_00035480	0.518928	0.838051	0.790504	BGIOSGA034694	XP_015697819.1 cytochrome P450 714C2-like
TCONS_00000583	0.519143	0.756565	1.83263	BGIOSGA013128	XP_006644004.2 importin subunit alpha-1a
TCONS_00007000	0.519171	0.0705742	1.24507	BGIOSGA000827	XP_006648034.1 probable alpha,alpha-trehalose-phosphate synthase [UDP-forming] 8
TCONS_00020185	0.519444	0.726002	1.43218	#N/A	#N/A
TCONS_00029283	0.519496	0.399848	1.37678	BGIOSGA026669	XP_002460467.130S ribosomal protein S16-2, chloroplastic/mitochondrial
TCONS_00013034	0.519829	-0.628034	-0.0229334	BGIOSGA017877	XP_006651733.1 phytanoyl-CoA dioxygenase 1
TCONS_00016113	0.519866	1.36035	1.52378	BGIOSGA018595	XP_006653526.1 cytochrome P450 71A9-like
TCONS_00024496	0.520532	1.3578	0.368713	BGIOSGA024899	XP_006658243.2 phospholipase A-2-activating protein
TCONS_00005532	0.520772	-0.307825	0.116135	BGIOSGA007641	XP_004951749.1succinate-semialdehyde dehydrogenase, mitochondrial
TCONS_00009566	0.520951	0.65258	0.103707	BGIOSGA001543	XP_006644222.1 26S proteasome non-ATPase regulatory subunit 13 homolog B-like
TCONS_00016444	0.52096	0.0203726	0.789098	BGIOSGA025652	XP_004976528.1E3 ubiquitin-protein ligase At1g63170
TCONS_00032525	0.521003	-0.891229	0.111771	BGIOSGA025241	XP_002466001.1non-specific lipid-transfer protein 2P
TCONS_00029238	0.521258	0.131906	0.530907	BGIOSGA026739	XP_004957142.1WPP domain-interacting protein 2
TCONS_00008136	0.52179	0.0835413	0.502174	BGIOSGA006302	XP_020396895.1threonine--tRNA ligase, chloroplastic/mitochondrial 2
TCONS_00037485	0.521909	0.390426	0.436279	BGIOSGA033746	XP_006664175.1 RNA polymerase II-associated protein 3
TCONS_00021838	0.521951	0.300943	-0.548319	BGIOSGA021690	XP_021304637.1tuliposide A-converting enzyme 1, chloroplastic-like
TCONS_00019398	0.522026	0.83098	0.605894	BGIOSGA017954	XP_006654483.1 arginyl-tRNA--protein transferase 2-like isoform X1
TCONS_00010736	0.522045	0.531878	-0.939952	BGIOSGA009870	XP_015690729.1 plasma membrane ATPase 1-like
TCONS_00036039	0.522063	-0.852684	0.285835	BGIOSGA035464	XP_006664025.1 probable prefoldin subunit 2
TCONS_00020124	0.522158	0.235677	0.816325	BGIOSGA022323	XP_006655789.1PREDICTED: uncharacterized protein LOC102704065
TCONS_00019265	0.522163	1.83573	1.6974	BGIOSGA018085	XP_003568493.1transcription termination factor MTERF8, chloroplastic
TCONS_00019635	0.522216	0.130952	1.41513	BGIOSGA014547	XP_003568092.2gamma-glutamyl hydrolase 2
TCONS_00032760	0.522348	1.15443	0.726097	BGIOSGA018280	XP_010235028.160S ribosomal export protein NMD3
TCONS_00021398	0.522422	1.27199	0.73025	BGIOSGA023623	XP_015694212.1 serine/arginine-rich splicing factor SR45a
TCONS_00033555	0.522595	-1.52505	-0.542865	BGIOSGA033912	XP_006662957.1PREDICTED: uncharacterized protein LOC102721490 isoform X1
TCONS_00035606	0.522601	1.49728	1.69123	BGIOSGA028075	XP_006663834.1 mRNA-decapping enzyme-like protein
TCONS_00036243	0.52266	-0.172111	-0.15762	BGIOSGA029967	XP_006664106.1PREDICTED: uncharacterized protein LOC102722168
TCONS_00003936	0.523048	0.153362	0.854803	BGIOSGA023104	XP_006644341.1 plasminogen activator inhibitor 1 RNA-binding protein-like
TCONS_00006957	0.523139	1.01327	-0.0933224	BGIOSGA022501	XP_006648001.1 UMP-CMP kinase 4
TCONS_00009581	0.523304	0.449908	0.510933	BGIOSGA003092	XP_024312066.150S ribosomal protein L15, chloroplastic
TCONS_00006266	0.523643	-1.08468	-1.7505	BGIOSGA006257	XP_003575145.1protein MAINTENANCE OF PSII UNDER HIGH LIGHT 1
TCONS_00008439	0.523862	0.854141	1.18271	BGIOSGA031081	XP_022681476.1wall-associated receptor kinase 5-like isoform X2
TCONS_00003401	0.523887	0.856439	0.169293	BGIOSGA018220	XP_010231510.160S ribosomal protein L29-1
TCONS_00011508	0.523951	1.34931	0.909059	BGIOSGA008592	XP_004985838.1ubiquitin domain-containing protein DSK2a
TCONS_00023122	0.523963	1.84533	0.234955	BGIOSGA030757	XP_004955459.1probable inositol transporter 2
TCONS_00031504	0.524006	0.307089	0.701014	BGIOSGA005165	XP_002465034.1probable glutathione S-transferase GSTU6
TCONS_00025948	0.524122	0.0430021	0.548942	BGIOSGA033017	XP_015696151.1 tobamovirus multiplication protein 2A-like
TCONS_00005558	0.524149	0.573813	1.50948	BGIOSGA007667	XP_006646978.1 nucleoid-associated protein At2g24020, chloroplastic-like

TCONS_00035739	0.524215	-0.475414	-0.065096	BGIOSGA037151	XP_006664422.1 glutathione S-transferase-like
TCONS_00011568	0.524282	0.234807	0.633263	BGIOSGA011447	XP_006651042.1 protein sister of odd and bowel-like
TCONS_00004901	0.52429	0.201777	0.552778	BGIOSGA010495	XP_006645177.1 PTI1-like tyrosine-protein kinase 1
TCONS_00022176	0.524584	1.15886	0.771475	BGIOSGA021320	XP_022681568.1WD repeat-containing protein 7
TCONS_00022654	0.524613	-0.302037	-0.639674	BGIOSGA026984	XP_015693426.1 uncharacterized methyltransferase At2g41040, chloroplastic
TCONS_00008411	0.52503	0.305199	0.824478	BGIOSGA006009	XP_006647646.1 lysine--tRNA ligase, chloroplastic/mitochondrial
TCONS_00020283	0.525172	0.189152	0.202889	BGIOSGA022481	XP_004964583.1uncharacterized protein LOC101782131
TCONS_00030078	0.525251	-1.71468	-1.37494	BGIOSGA006377	XP_006661252.1 WAT1-related protein At4g30420-like
TCONS_00006688	0.525501	-0.419653	-0.330323	BGIOSGA014313	XP_014756494.1uncharacterized protein LOC100834713
TCONS_00003496	0.525588	1.00224	-1.12009	BGIOSGA001706	XP_004968704.1glycine, alanine and asparagine-rich protein
TCONS_00004394	0.525595	0.440402	0.880352	BGIOSGA009181	XP_006644707.1 probable alpha,alpha-trehalose-phosphate synthase [UDP-forming] 7
TCONS_00027074	0.525725	0.976467	0.693559	BGIOSGA036156	XP_006659662.1 E3 ubiquitin ligase BIG BROTHER-related-like
TCONS_00006932	0.525787	0.0501727	1.20121	BGIOSGA014304	XP_004954045.1soluble inorganic pyrophosphatase 6, chloroplastic
TCONS_00011964	0.52579	1.2961	0.951161	BGIOSGA011046	XP_006649808.1 cytosolic enolase 3
TCONS_00024439	0.525995	-0.357432	-1.26369	BGIOSGA040663	XP_015694502.1 dirigent protein 1-like
TCONS_00022542	0.526501	0.0732218	-0.300787	BGIOSGA007737	XP_015694124.1 probable calcium-binding protein CML21
TCONS_00009215	0.526635	0.44114	0.0237647	BGIOSGA014503	XP_006649299.1 SUMO-conjugating enzyme SCE1
TCONS_00006404	0.526928	1.70458	0.271621	BGIOSGA020719	XP_015689083.1 glyceraldehyde-3-phosphate dehydrogenase 3, cytosolic
TCONS_00020360	0.526948	-0.406583	0.844686	BGIOSGA022552	XP_006655914.1 cyclin-D3-1 isoform X1
TCONS_00007237	0.527199	2.80679	0.728962	BGIOSGA004771	XP_006648229.1 luminal-binding protein 2
TCONS_00037522	0.527225	-0.802571	-0.156304	BGIOSGA035844	XP_006664197.1 nuclear transcription factor Y subunit A-7-like isoform X2
TCONS_00021547	0.527386	0.312011	-0.0542496	#N/A	#N/A
TCONS_00011228	0.527457	-0.450609	0.567637	BGIOSGA001417	NP_001141136.1Ras protein ARA-3
TCONS_00013620	0.528089	-2.85878	-0.448341	BGIOSGA015773	XP_006652084.1PREDICTED: uncharacterized protein LOC102714366
TCONS_00006719	0.528283	2.15187	-0.110482	BGIOSGA008888	XP_003570192.1universal stress protein PHOS34
TCONS_00012748	0.528381	0.605847	0.819426	BGIOSGA035115	XP_006650313.1 exocyst complex component SEC3A-like
TCONS_00012228	0.528478	0.659973	-0.131603	BGIOSGA026364	XP_006650058.1 60S ribosomal protein L19-1-like
TCONS_00016152	0.528766	0.439123	0.407213	BGIOSGA014823	XP_015691787.1 protein NRT1/ PTR FAMILY 4.6-like isoform X1
TCONS_00018231	0.529137	-0.953194	-0.544092	BGIOSGA000941	XP_006654724.2 jacalin-related lectin 19-like isoform X2
TCONS_00007019	0.529213	-0.994399	-0.745898	BGIOSGA022375	XP_015688756.1 transcription factor bHLH66-like
TCONS_00034692	0.52952	0.0749967	0.628152	BGIOSGA013220	XP_006647932.1 LMBR1 domain-containing protein 2 homolog A-like isoform X1
TCONS_00023950	0.530365	0.287087	0.922632	BGIOSGA025982	XP_008670499.2O-fucosyltransferase 36
TCONS_00025788	0.530551	0.471428	-0.260631	BGIOSGA037250	XP_006665025.1 protein mago nashi homolog
TCONS_00033445	0.530609	0.743252	-0.0232237	BGIOSGA017685	XP_006662933.1 ER lumen protein-retaining receptor
TCONS_00027069	0.530717	1.20606	0.696851	BGIOSGA027107	XP_004974118.1RNA-binding protein 1
TCONS_00026526	0.530822	0.234858	0.638029	BGIOSGA033737	XP_004973343.1probable E3 ubiquitin ligase SUD1
TCONS_00030815	0.531118	0.854872	1.6542	BGIOSGA025777	XP_004951725.1ribosomal RNA small subunit methyltransferase
TCONS_00031477	0.531318	0.928957	1.79323	BGIOSGA035724	XP_015697055.1 mitochondrial inner membrane protein OXA1-like
TCONS_00019278	0.531496	-0.675338	0.504806	BGIOSGA018073	XP_022681358.1uncharacterized protein LOC101757009 isoform X5
TCONS_00001456	0.531506	0.0039972	0.707415	BGIOSGA004098	NP_001151674.1tat pathway signal sequence family protein
TCONS_00009867	0.531549	-0.0557699	1.2434	BGIOSGA012418	XP_006651281.1PREDICTED: uncharacterized protein LOC102717143
TCONS_00016573	0.531773	-1.90214	-0.416659	BGIOSGA006191	NP_001315062.1uncharacterized LOC100384454
TCONS_00036844	0.531793	0.384494	0.629813	BGIOSGA008095	XP_022683630.1putative methyltransferase At1g22800, mitochondrial
TCONS_00026554	0.531803	0.00235013	0.632376	BGIOSGA007771	XP_006659380.2 ABC transporter A family member 7-like
TCONS_00029415	0.532099	0.634486	0.639741	BGIOSGA023582	XP_006660897.1 peptidyl-prolyl cis-trans isomerase CYP21-1
TCONS_00001014	0.532332	-4.03842	-1.6227	BGIOSGA003617	XP_014754331.1NRR repressor homolog 1
TCONS_00034822	0.532408	-0.743094	-0.26037	BGIOSGA034044	XP_006662927.1 UV-B-induced protein At3g17800, chloroplastic-like
TCONS_00030929	0.532482	-0.0228558	0.147739	BGIOSGA023536	XP_015696959.1 protein SNOWY COTYLEDON 3-like
TCONS_00006868	0.532737	0.518717	1.14462	BGIOSGA021617	NP_001145244.1uncharacterized LOC100278526
TCONS_00020808	0.532811	0.401123	-0.321654	BGIOSGA017219	XP_006656131.1PREDICTED: uncharacterized protein LOC102710413
TCONS_00009543	0.532846	0.239712	0.513132	BGIOSGA012088	XP_006651159.1 methionine--tRNA ligase, chloroplastic/mitochondrial
TCONS_00035160	0.532992	-0.195451	0.614483	BGIOSGA019284	XP_006663038.2 acyl-coenzyme A oxidase 2, peroxisomal
TCONS_00013062	0.533054	0.68956	0.0627631	BGIOSGA009922	XP_006650496.1 binding partner of ACD11 1
TCONS_00019309	0.53329	0.0415631	-1.04635	BGIOSGA025413	XP_006654415.1 KH domain-containing protein At1g09660/At1g09670-like isoform X1

TCONS_00035353	0.533416	0.769218	1.5935	BGIOSGA037637	XP_010237400.1ribonucleases P/MRP protein subunit POP1
TCONS_00011381	0.533558	1.23081	0.434289	BGIOSGA023995	XP_006652056.1 DIBOA-glucoside dioxygenase BX6-like
TCONS_00002329	0.53371	0.490197	0.780663	BGIOSGA004970	XP_006645156.1 15 kDa selenoprotein
TCONS_00000183	0.533881	-0.955163	-0.590065	BGIOSGA038774	XP_006643739.1 sulfoquinovosyl transferase SQD2-like
TCONS_00014938	0.533887	0.94311	0.92873	BGIOSGA032507	XP_006652823.1 lecithin-cholesterol acyltransferase-like 4
TCONS_00032456	0.534254	-0.544616	-0.00435501	BGIOSGA031661	XP_022678727.1uncharacterized protein LOC111255797
TCONS_00021375	0.534448	2.21508	1.11362	BGIOSGA023603	XP_006657346.1PREDICTED: uncharacterized protein LOC102718850
TCONS_00024324	0.534468	-2.28185	0.236122	BGIOSGA028726	NP_001335716.1uncharacterized LOC100217024
TCONS_00024367	0.534773	0.342255	1.26284	BGIOSGA012543	XP_002461229.2ricin B-like lectin R40C1
TCONS_00010582	0.534775	-0.361875	0.566808	BGIOSGA033007	XP_004982351.1transcription factor PIF1 isoform X3
TCONS_00019477	0.534855	-0.900394	1.15157	BGIOSGA011956	XP_006654541.1 alanine--glyoxylate aminotransferase 2 homolog 3, mitochondrial-like
TCONS_00012602	0.534857	0.464376	-0.220202	BGIOSGA026364	XP_006650058.1 60S ribosomal protein L19-1-like
TCONS_00014445	0.534862	0.311165	-1.13326	BGIOSGA010743	XP_015691861.1 BTB/POZ domain-containing protein At1g67900-like
TCONS_00033275	0.535049	-0.854723	-1.16459	BGIOSGA035099	XP_006662906.1 endonuclease III homolog 1, chloroplastic-like
TCONS_00008000	0.535082	1.18624	0.33183	BGIOSGA029024	XP_006648637.1 type II inositol polyphosphate 5-phosphatase 15-like
TCONS_00001692	0.535413	1.76207	1.50198	BGIOSGA004326	XP_006644613.1PREDICTED: uncharacterized protein LOC102710920
TCONS_00010827	0.535708	0.297506	0.446238	BGIOSGA010782	XP_006650497.1 GPN-loop GTPase 3
TCONS_00002606	0.535999	0.064664	0.220091	BGIOSGA005245	XP_006645343.1 vacuolar protein sorting-associated protein 55 homolog isoform X1
TCONS_00015364	0.536136	1.30101	-0.468454	BGIOSGA030790	NP_001306701.1probable protein phosphatase 2C 37
TCONS_00026392	0.536254	-0.0617077	0.990685	BGIOSGA028458	XP_015695700.1 enoyl-[acyl-carrier-protein] reductase [NADH] 1, chloroplastic
TCONS_00000473	0.536552	-0.296634	-0.580382	BGIOSGA014024	XP_006643935.1 ras-related protein Rab7
TCONS_00033458	0.536593	0.226996	0.868157	BGIOSGA037371	XP_006663442.1 GPI transamidase component PIG-T
TCONS_00027267	0.536635	0.107886	0.459732	BGIOSGA027735	XP_002444971.1glycosyltransferase family 92 protein Os08g0121900
TCONS_00030235	0.536671	1.70216	2.19055	BGIOSGA020527	XP_004973824.2heat shock protein 81-1
TCONS_00019971	0.536977	-2.6149	-1.78168	BGIOSGA009420	XP_006656551.2 homeobox protein BEL1 homolog
TCONS_00011891	0.537073	0.45983	-0.181263	BGIOSGA010624	XP_003558380.13-ketoacyl-CoA synthase 1
TCONS_00014411	0.53746	-0.0756365	-0.132544	BGIOSGA016574	XP_004975885.1ankyrin repeat and SAM domain-containing protein 6
TCONS_00037263	0.537732	-0.615763	0.252034	BGIOSGA036114	XP_006664059.2 F-box protein At3g12350
TCONS_00000297	0.537855	0.214844	1.41713	BGIOSGA003689	XP_015699356.1 monothiol glutaredoxin-S4, mitochondrial
TCONS_00015195	0.53787	-3.87109	0.614726	BGIOSGA016827	XP_006653895.1 probable LRR receptor-like serine/threonine-protein kinase At1g63430
TCONS_00022532	0.537881	0.853277	1.11329	BGIOSGA000785	XP_006657117.2 CBL-interacting protein kinase 24
TCONS_00026963	0.538018	-0.882019	0.212061	BGIOSGA029014	XP_015696171.1 protein EMBRYO SAC DEVELOPMENT ARREST 3, chloroplastic
TCONS_00004708	0.538359	0.55456	-0.304016	BGIOSGA003811	XP_006644995.1 ATP-dependent zinc metalloprotease FTSH 3, mitochondrial
TCONS_00008311	0.538437	1.1098	0.983868	BGIOSGA035852	XP_004952992.1very-long-chain 3-oxoacyl-CoA reductase 1
TCONS_00019749	0.538503	0.160289	-0.616147	BGIOSGA019180	XP_006655565.1 beta-1,3-galactosyltransferase pvg3-like
TCONS_00030175	0.538608	1.47296	-0.236954	BGIOSGA034233	XP_006660708.1 tuliposide A-converting enzyme 1, chloroplastic-like
TCONS_00029795	0.539007	0.313039	1.06776	BGIOSGA030020	XP_006660525.1PREDICTED: uncharacterized protein LOC102707611
TCONS_00013552	0.539034	0.158067	0.201125	BGIOSGA009423	XP_006650909.1 derlin-2
TCONS_00031392	0.539282	-0.226331	1.17589	BGIOSGA033205	XP_015697038.1 1-acyl-sn-glycerol-3-phosphate acyltransferase 1, chloroplastic
TCONS_00005929	0.539415	1.48776	1.32349	BGIOSGA037308	XP_006648576.2PREDICTED: uncharacterized protein LOC102703558
TCONS_00025625	0.539699	0.793318	0.291234	BGIOSGA023807	XP_006658048.1 mitochondrial ubiquitin ligase activator of NFKB 1
TCONS_00008414	0.539817	-0.193143	-0.722693	BGIOSGA014706	XP_004953152.1adenosine kinase 2
TCONS_00014884	0.539819	0.465213	1.23664	#N/A	#N/A
TCONS_00007684	0.539893	0.860462	0.544724	BGIOSGA013491	XP_006647113.1 peroxisome biogenesis protein 7
TCONS_00010984	0.540069	0.213021	0.12917	BGIOSGA030285	NP_001152924.1PTD008 protein
TCONS_00020661	0.540201	1.54356	-0.437692	BGIOSGA027861	XP_006656946.1 50S ribosomal protein L30
TCONS_00028025	0.540369	1.5241	1.73328	BGIOSGA003380	XP_015695604.1 cyclin-D1-2-like
TCONS_00035578	0.540561	0.251773	-0.163135	BGIOSGA034799	XP_002448989.1phytosulfokines 5
TCONS_00030574	0.540628	1.59205	1.97383	BGIOSGA030021	XP_002438054.1chitin elicitor receptor kinase 1
TCONS_00011057	0.540837	1.65069	0.823561	BGIOSGA029518	XP_006651871.1 transcription factor JUNGBRUNNEN 1-like
TCONS_00000389	0.541006	0.678053	0.266993	BGIOSGA010692	XP_006643887.1 60S ribosomal protein L11
TCONS_00009622	0.541243	-0.2101	-0.0108256	BGIOSGA012169	XP_006649668.1 cytochrome P450 90B1
TCONS_00004261	0.541247	0.953548	-0.045276	BGIOSGA000943	XP_006644584.1 RNA polymerase II transcriptional coactivator KELP
TCONS_00022276	0.541257	-0.499325	0.00785079	BGIOSGA021217	XP_006656097.1 ubiquitin-like-specific protease 1D isoform X1
TCONS_00018464	0.541287	-0.183429	0.541293	BGIOSGA029978	XP_006653947.1 GTP-binding protein YPTM1-like

TCONS_00034484	0.5413	-0.315866	0.922132	BGIOSGA030690	XP_006663283.1 F-box/LRR-repeat protein At3g59190-like
TCONS_00036501	0.541506	0.304716	-0.07475	BGIOSGA027393	XP_006664787.1 plasma membrane ATPase 1
TCONS_00034209	0.541547	0.458012	0.620803	BGIOSGA036721	XP_006662694.1PREDICTED: uncharacterized protein LOC102706044
TCONS_00017024	0.541718	0.576834	0.164907	BGIOSGA028705	XP_015692955.1 UPF0014 membrane protein STAR2
TCONS_00010056	0.541753	1.55601	1.28219	BGIOSGA012600	XP_006651325.1 peter Pan-like protein
TCONS_00008959	0.541773	0.425317	0.928062	BGIOSGA005448	XP_002454694.1translation initiation factor IF3-4, chloroplastic
TCONS_00011100	0.541941	-0.349856	-0.559421	BGIOSGA037678	XP_006650280.1PREDICTED: uncharacterized protein LOC102722752, partial
TCONS_00003276	0.541995	1.09252	1.12011	BGIOSGA001942	XP_003566742.1probable envelope ADP,ATP carrier protein, chloroplastic
TCONS_00037582	0.542399	0.812118	0.836569	BGIOSGA017515	XP_006664772.1 malate dehydrogenase, glyoxysomal
TCONS_00006781	0.542856	0.135568	-0.0808921	BGIOSGA008954	XP_015693149.1 chromatin structure-remodeling complex protein BSH
TCONS_00009373	0.542951	1.34105	1.14476	BGIOSGA009272	XP_006649445.1 glutathione reductase, chloroplastic-like
TCONS_00031943	0.543417	-1.197	-0.780225	BGIOSGA013440	XP_004983081.1phosphoglucomutase, chloroplastic
TCONS_00019611	0.543455	0.251836	0.661356	BGIOSGA015273	XP_015692792.1 single-stranded DNA-binding protein, mitochondrial-like isoform X1
TCONS_00014098	0.543666	0.363169	0.733043	BGIOSGA019407	XP_021319681.1exocyst complex component EXO70B1
TCONS_00007017	0.544264	0.473368	0.718261	BGIOSGA021774	XP_003572898.1ubiquitin carboxyl-terminal hydrolase 23 isoform X1
TCONS_00002199	0.544344	0.647116	1.12851	BGIOSGA000453	XP_006645051.1PREDICTED: uncharacterized protein LOC102721883
TCONS_00006736	0.544388	0.663508	0.850925	BGIOSGA011197	XP_015689606.1 ATP-dependent DNA helicase homolog RECG, chloroplastic
TCONS_00007573	0.544672	0.820626	0.438324	BGIOSGA008333	XP_006647056.1 50S ribosomal protein L19, chloroplastic-like
TCONS_00010808	0.544705	0.909232	1.30952	BGIOSGA013432	XP_006650488.1 DNA-directed RNA polymerase I subunit RPA12-like
TCONS_00008960	0.544823	0.597979	-0.0282963	BGIOSGA005446	XP_006648044.2 formin-like protein 7
TCONS_00004403	0.54513	1.38724	0.87578	BGIOSGA001897	XP_015689575.1 glucosidase 2 subunit beta
TCONS_00006791	0.545166	1.54544	0.237543	BGIOSGA008966	XP_004953768.1protein TIC 20-v, chloroplastic
TCONS_00002098	0.545338	-0.884647	-0.701109	BGIOSGA004726	XP_006644940.1 exocyst complex component EXO70B1-like
TCONS_00033828	0.545439	1.33239	1.43473	BGIOSGA021206	XP_015697510.1 serine--tRNA ligase, chloroplastic/mitochondrial
TCONS_00004246	0.545504	0.00510652	0.0879423	BGIOSGA026025	XP_015689680.1 serine/threonine-protein kinase TAO3-like
TCONS_00022899	0.545601	1.30497	1.29516	BGIOSGA020544	XP_002439004.1tRNA-dihydrouridine(16/17) synthase [NAD(P)(+)]-like
TCONS_00025573	0.545714	0.0636689	0.733297	BGIOSGA000828	XP_015694739.1 serine/threonine-protein kinase STY8
TCONS_00021472	0.54572	1.48999	1.65566	BGIOSGA034370	XP_006656560.1 probable transcription factor KAN2
TCONS_00021846	0.545948	0.627947	0.0292821	BGIOSGA001628	XP_006656771.1 putative 12-oxophytodienoate reductase 4 isoform X1
TCONS_00012310	0.545949	-0.163288	0.0791553	BGIOSGA022478	XP_006651405.2 sugar transporter ERD6-like 16
TCONS_00003144	0.546145	-1.21098	0.195164	BGIOSGA006608	XP_006643944.1 rac-like GTP-binding protein 1
TCONS_00016644	0.546146	-0.529342	-0.234981	BGIOSGA024235	XP_015692344.1 autophagy-related protein 8B
TCONS_00023358	0.546488	0.936477	0.720065	BGIOSGA036272	XP_006657562.1 acyl carrier protein 1, mitochondrial
TCONS_00027215	0.54676	0.958162	0.717188	BGIOSGA009527	XP_006659745.1 GDP-mannose transporter GONST1-like
TCONS_00001822	0.546767	0.470675	0.642775	BGIOSGA004458	XP_006646323.1 50S ribosomal protein L13, chloroplastic
TCONS_00035524	0.546934	0.471692	0.870731	BGIOSGA036946	XP_022684237.1disease resistance protein RGA2-like
TCONS_00017837	0.547154	0.203482	0.157469	BGIOSGA019896	XP_006654421.1PREDICTED: uncharacterized protein LOC102709926
TCONS_00018385	0.547411	0.411904	-0.00064548	BGIOSGA008261	XP_004961108.1predicted GPI-anchored protein 58
TCONS_00022756	0.547449	-0.0427089	0.715468	BGIOSGA007158	XP_020394918.1protein G1-like1
TCONS_00011195	0.547486	-0.108344	-0.486258	BGIOSGA013797	XP_015690832.1 zerumbone synthase
TCONS_00007969	0.547555	1.39489	1.12929	BGIOSGA006512	XP_002453780.1uncharacterized protein LOC8081691
TCONS_00023127	0.547559	0.77281	-0.207104	BGIOSGA013685	XP_006657452.1 peroxisomal membrane protein 13 isoform X2
TCONS_00024264	0.54777	-0.770817	-0.948752	BGIOSGA031495	XP_006658910.1PREDICTED: uncharacterized protein LOC102714663, partial
TCONS_00032729	0.547969	0.108636	-0.00203699	BGIOSGA025891	NP_001142936.1uncharacterized LOC100275376
TCONS_00004208	0.548128	2.99596	0.775176	BGIOSGA000995	XP_015697631.1 probable serine/threonine-protein kinase At1g18390
TCONS_00026082	0.548159	1.54136	1.19014	BGIOSGA028140	XP_015695725.1 F-box/LRR-repeat protein At1g55660-like isoform X2
TCONS_00011913	0.548361	-1.67127	-1.69738	BGIOSGA023824	XP_006651222.1 probable carboxylesterase 9
TCONS_00029736	0.548589	1.18661	-0.323901	BGIOSGA032746	NP_001148769.1fiber protein Fb34 precursor
TCONS_00030586	0.548891	#NA	#NA	#N/A	#N/A
TCONS_00021606	0.548959	0.86085	0.345107	BGIOSGA021923	XP_006655784.1 iron-sulfur assembly protein IscA, chloroplastic isoform X1
TCONS_00028851	0.548992	0.985819	0.795691	BGIOSGA035901	XP_006661163.2 CRM-domain containing factor CFM3, chloroplastic/mitochondrial-like
TCONS_00033016	0.549021	0.739577	0.118728	BGIOSGA026208	XP_015698033.1 transcription factor VIP1-like
TCONS_00001650	0.549073	0.953439	1.00343	BGIOSGA017638	XP_015699274.1 AP-1 complex subunit mu-2
TCONS_00015229	0.549293	-0.30004	0.59167	BGIOSGA017415	XP_006653083.1PREDICTED: uncharacterized protein LOC102711253

TCONS_00032773	0.549399	1.22634	1.08517	BGIOSGA031336	XP_015697429.1PREDICTED: uncharacterized protein LOC102707618 isoform X1
TCONS_00016557	0.54942	0.24431	0.88744	BGIOSGA014410	XP_003580453.1alkylated DNA repair protein ALKBH8 homolog
TCONS_00036806	0.549658	-1.63046	-0.591199	BGIOSGA036507	XP_006663887.1 3'(2'),5'-bisphosphate nucleotidase-like
TCONS_00020898	0.549662	0.328057	0.0692675	BGIOSGA023106	XP_015693727.1 transmembrane protein 258
TCONS_00016783	0.549918	1.06305	0.42973	BGIOSGA014184	XP_004960144.1ribosomal RNA processing protein 1 homolog isoform X1
TCONS_00019418	0.550261	-0.0967569	0.0680494	BGIOSGA009489	XP_006654499.1PREDICTED: uncharacterized protein LOC102709564 isoform X2
TCONS_00002964	0.550314	0.804633	0.835008	BGIOSGA032692	XP_006645565.1 DNA mismatch repair protein MSH7
TCONS_00014248	0.550526	1.02295	0.387116	BGIOSGA016409	XP_008668184.1structural molecule isoform X2
TCONS_00015677	0.551066	0.128581	0.642352	BGIOSGA006454	XP_006653286.2 U-box domain-containing protein 9-like
TCONS_00032612	0.55114	0.0982743	0.558212	BGIOSGA031501	XP_006661996.1 protein PAM68, chloroplastic
TCONS_00026085	0.551221	0.773205	0.534362	BGIOSGA028158	XP_006659204.1 obg-like ATPase 1
TCONS_00002577	0.551227	0.459104	-0.151335	BGIOSGA005220	XP_004971333.1NAD(P)H-quinone oxidoreductase subunit O, chloroplastic
TCONS_00014507	0.551268	-0.573592	0.767384	BGIOSGA016669	XP_006652457.1 uncharacterized CRM domain-containing protein At3g25440, chloroplastic
TCONS_00022087	0.551287	-4.80243	-1.6477	BGIOSGA004012	XP_006656895.1 UDP-glycosyltransferase 708A6-like
TCONS_00021349	0.551306	0.0304316	0.336132	BGIOSGA025518	XP_015694183.1 protein DETOXIFICATION 16-like isoform X1
TCONS_00011412	0.551536	1.8965	1.23655	BGIOSGA011614	XP_002466026.1UPF0187 protein At3g61320, chloroplastic
TCONS_00001005	0.551891	1.1037	1.26834	BGIOSGA004677	XP_024316138.1pentatricopeptide repeat-containing protein At5g11310, mitochondrial-like
TCONS_00030032	0.552066	0.530012	0.284788	BGIOSGA029775	XP_003576520.1fructose-bisphosphate aldolase-lysine N-methyltransferase, chloroplastic
TCONS_00031641	0.552198	-0.19653	1.42962	BGIOSGA025742	XP_003574352.1casein kinase II subunit beta-1
TCONS_00021201	0.552298	-0.374155	0.298234	BGIOSGA031074	XP_006656340.1 receptor-like cytosolic serine/threonine-protein kinase RBK1
TCONS_00027136	0.552334	1.82561	1.72205	BGIOSGA007755	XP_015696024.1 peptidyl-prolyl cis-trans isomerase CYP71
TCONS_00022131	0.552409	0.439987	0.956025	BGIOSGA008943	XP_003563917.1protein-tyrosine-phosphatase IBR5
TCONS_00023682	0.552843	0.750598	0.91953	BGIOSGA001620	XP_006658561.1 asparagine--tRNA ligase, chloroplastic/mitochondrial
TCONS_00011171	0.553136	0.335073	1.20702	BGIOSGA013776	XP_006650758.2 5'-adenylylsulfate reductase-like 5
TCONS_00010331	0.553685	0.334109	0.791173	BGIOSGA001051	XP_015689940.1 putative receptor protein kinase ZmPK1
TCONS_00026435	0.553707	0.0261197	0.237602	BGIOSGA024652	XP_006659337.2 myb family transcription factor APL-like
TCONS_00023352	0.553911	0.8677	0.565386	BGIOSGA023341	XP_006647334.1 ent-cassadiene C2-hydroxylase-like
TCONS_00026474	0.553958	0.00268765	1.09907	BGIOSGA028542	XP_015695579.1PREDICTED: uncharacterized protein LOC107304721, partial
TCONS_00023771	0.554319	-1.81015	0.0163811	BGIOSGA013733	XP_003560151.118.8 kDa class V heat shock protein
TCONS_00027537	0.554455	0.310622	-0.31567	BGIOSGA030015	XP_006659321.1 GDP-mannose transporter GONST4-like
TCONS_00005507	0.554618	-0.343498	0.339499	BGIOSGA007621	XP_008674599.1protein-O-fucosyltransferase 1 isoform X1
TCONS_00026896	0.554623	0.320999	0.708295	BGIOSGA028956	XP_004973850.1thylakoid lumenal 19 kDa protein, chloroplastic
TCONS_00022259	0.554867	0.305876	1.57472	BGIOSGA021234	XP_008672768.1thioredoxin superfamily protein isoform X1
TCONS_00026998	0.554964	-3.52634	0.310182	BGIOSGA029049	XP_006659619.1 65-kDa microtubule-associated protein 8
TCONS_00009580	0.55572	-0.804743	0.538534	BGIOSGA012133	XP_004985169.1sprT-like domain-containing protein Spartan
TCONS_00018004	0.555935	0.795692	0.457666	BGIOSGA020052	XP_015692634.1 mucin-1
TCONS_00031109	0.555978	0.561992	-0.474868	BGIOSGA001197	XP_015697450.1 probable aldo-keto reductase 1
TCONS_00011489	0.556056	0.447616	-0.266424	BGIOSGA023094	XP_015691235.1 K(+) efflux antiporter 5 isoform X2
TCONS_00027248	0.55615	0.86535	1.21473	BGIOSGA027753	XP_002444926.1polyadenylate-binding protein-interacting protein 12
TCONS_00025497	0.55619	1.40718	1.70588	BGIOSGA012882	XP_006657926.1 putative G3BP-like protein
TCONS_00020077	0.556279	-0.0917097	0.472027	BGIOSGA022257	XP_015693444.1 transcription initiation factor TFIID subunit 14b
TCONS_00000681	0.556441	1.22342	1.86876	BGIOSGA036552	XP_006644067.2 pentatricopeptide repeat-containing protein At1g80270, mitochondrial-like
TCONS_00022636	0.556566	-0.358076	0.665849	BGIOSGA010100	XP_004965769.1ent-cassadiene C2-hydroxylase
TCONS_00020240	0.557152	0.324164	0.196938	BGIOSGA037725	XP_006655851.1 protein DA1-related 1-like
TCONS_00030224	0.557759	-3.26512	-3.06637	BGIOSGA008961	XP_006661352.1 cellulose synthase-like protein E6
TCONS_00010198	0.557813	-1.53547	-0.424968	BGIOSGA029421	NP_001152498.2acyltransferase precursor
TCONS_00022122	0.558077	0.509941	0.758752	BGIOSGA021382	XP_006656911.1 protein FATTY ACID EXPORT 1, chloroplastic-like
TCONS_00000366	0.558115	1.36227	0.927923	BGIOSGA002020	XP_006645601.1 ectonucleotide pyrophosphatase/phosphodiesterase family member 3-like
TCONS_00005140	0.558655	-0.133211	1.27872	BGIOSGA000063	XP_006645356.2PREDICTED: uncharacterized protein LOC102716178
TCONS_00035573	0.558686	0.725402	0.728031	BGIOSGA034795	XP_006664335.1 peptidyl-prolyl cis-trans isomerase FKBP42
TCONS_00026847	0.558776	0.539658	1.15908	BGIOSGA023243	XP_006659530.1 C2 and GRAM domain-containing protein At1g03370-like isoform X1
TCONS_00011249	0.558877	-1.02786	0.0415126	BGIOSGA013848	XP_006650820.1 dolichol-phosphate mannosyltransferase subunit 1

TCONS_00019334	0.559151	-2.68829	-0.187041	BGIOSGA014332	XP_015692437.1 dnaJ homolog subfamily B member 13-like
TCONS_00001112	0.559575	0.431058	0.542052	BGIOSGA003736	XP_006644282.1 DEAD-box ATP-dependent RNA helicase 15
TCONS_00007894	0.559637	0.118449	0.331127	BGIOSGA008072	XP_006647217.1PREDICTED: uncharacterized protein LOC102717678 isoform X1
TCONS_00000022	0.559804	0.925888	0.117254	BGIOSGA005442	XP_004967927.1protein NCA1
TCONS_00035113	0.560317	1.16126	1.12184	BGIOSGA030000	XP_006663020.1 CDK5RAP1-like protein
TCONS_00020817	0.560381	0.611527	1.28481	BGIOSGA027351	XP_015694208.1 cyclic nucleotide-gated ion channel 1-like isoform X1
TCONS_00016576	0.560696	0.672333	1.13106	BGIOSGA028232	XP_006652784.1 uncharacterized membrane protein At1g16860-like
TCONS_00036417	0.560843	0.81098	0.641007	BGIOSGA018136	XP_004977833.2L10-interacting MYB domain-containing protein
TCONS_00010822	0.561375	-1.06169	0.184873	BGIOSGA013447	XP_015691007.1PREDICTED: uncharacterized protein LOC102715652 isoform X2
TCONS_00026240	0.561382	-0.524077	-0.210903	BGIOSGA028318	XP_006659978.1PREDICTED: uncharacterized protein LOC102719609, partial
TCONS_00005176	0.561426	-1.3371	0.226977	BGIOSGA000030	XP_006646732.1 DEAD-box ATP-dependent RNA helicase 58, chloroplastic
TCONS_00015011	0.561662	-0.140056	0.726049	BGIOSGA031690	XP_006652868.1 anthocyanidin reductase-like
TCONS_00026501	0.561717	0.319305	-0.0705563	BGIOSGA019358	XP_015695581.1 nudix hydrolase 15, mitochondrial-like
TCONS_00010656	0.561879	0.774681	0.940422	BGIOSGA010084	XP_021301928.1uncharacterized protein LOC8064631
TCONS_00010297	0.562131	0.316891	0.0580758	BGIOSGA013075	XP_006650194.1 ACT domain-containing protein ACR4
TCONS_00026915	0.562131	1.41412	1.47425	BGIOSGA036965	XP_006659570.1 pre-rRNA-processing protein TSR2-like
TCONS_00035402	0.562268	1.43614	0.355208	BGIOSGA022937	XP_002451314.1vacuolar protein sorting-associated protein 2 homolog 1
TCONS_00034134	0.562382	-3.17388	-0.938753	BGIOSGA003354	XP_006662656.1 probable receptor-like protein kinase At5g18500
TCONS_00017761	0.56246	0.709647	1.10734	BGIOSGA019067	XP_006654366.1 kinesin-like protein KIF22
TCONS_00013995	0.562613	-2.14126	-0.51337	BGIOSGA016142	XP_003621683.2aldo/keto reductase family oxidoreductase
TCONS_00013449	0.563406	-0.375518	-0.714634	BGIOSGA020078	XP_013457837.1transmembrane protein, putative
TCONS_00018238	0.563536	0.0799633	-0.0903704	BGIOSGA020296	XP_006654731.1 WD repeat-containing protein 82
TCONS_00014574	0.563986	0.085035	0.00960304	BGIOSGA008632	XP_006652495.2 glutaredoxin-C6
TCONS_00019925	0.563997	-0.336705	0.204773	BGIOSGA017437	XP_006654885.2PREDICTED: uncharacterized protein LOC102703123
TCONS_00013493	0.564197	1.3457	0.747222	BGIOSGA004862	XP_006650864.1 actin-3
TCONS_00035548	0.564464	1.28208	1.27071	BGIOSGA034775	XP_015698210.1 2-isopropylmalate synthase A-like
TCONS_00001143	0.564822	0.225021	-0.513446	#N/A	#N/A
TCONS_00033492	0.564901	0.740891	-0.0356986	BGIOSGA012523	XP_006662941.1 probable WRKY transcription factor 56
TCONS_00004158	0.565658	0.321974	1.05886	BGIOSGA036411	XP_015688144.1 katanin p60 ATPase-containing subunit A-like 2
TCONS_00033700	0.565726	-0.384968	0.495773	BGIOSGA030496	XP_006662975.2 protein WAVE-DAMPENED 2-like
TCONS_00027895	0.565763	-0.619182	0.236678	BGIOSGA003763	XP_006660084.2 chloroplastic group IIA intron splicing facilitator CRS1, chloroplastic
TCONS_00032214	0.565905	-0.067088	1.44856	BGIOSGA011926	XP_006661774.1 exocyst complex component SEC15A-like
TCONS_00021235	0.565909	-0.11061	0.779018	BGIOSGA023461	XP_006657246.1 geranylgeranyl transferase type-2 subunit alpha
TCONS_00030285	0.566036	-0.548936	1.01143	BGIOSGA026682	XP_015696665.1 tetraketide alpha-pyrone reductase 1-like
TCONS_00009504	0.566087	-0.560683	0.577519	BGIOSGA012054	XP_006649570.1 UPF0496 protein 1
TCONS_00013196	0.566119	-1.76654	-0.567203	BGIOSGA023934	XP_015690777.1 COBRA-like protein 3 isoform X1
TCONS_00002086	0.566198	0.465419	-0.224586	BGIOSGA024813	XP_004970402.1CBL-interacting protein kinase 11
TCONS_00019579	0.566337	1.2179	2.66836	BGIOSGA017779	NP_001152707.1allyl alcohol dehydrogenase-like protein
TCONS_00017499	0.566628	-0.397382	-0.00277495	BGIOSGA008571	XP_006654236.1 protein phosphatase inhibitor 2-like
TCONS_00014347	0.566675	0.19465	1.33593	BGIOSGA016508	XP_003579864.1pentatricopeptide repeat-containing protein At5g09450, mitochondrial
TCONS_00016201	0.567039	-0.272375	0.776794	BGIOSGA014774	XP_006652483.1 dol-P-Glc:Glc(2)Man(9)GlcNAc(2)-PP-Dol alpha-1,2-glucosyltransferase isoform X2
TCONS_00018990	0.567099	-0.780636	-0.815181	BGIOSGA018373	XP_015693241.1 protein plastid transcriptionally active 16, chloroplastic
TCONS_00030803	0.567283	1.07002	0.117768	BGIOSGA024546	XP_004981982.1uncharacterized protein LOC101784562
TCONS_00035645	0.567306	0.58621	0.686888	BGIOSGA037066	XP_008662338.14-alpha-L-fucosyltransferase isoform X1
TCONS_00023695	0.567384	-2.41031	-0.873152	BGIOSGA030669	XP_006658616.2 cytochrome P450 716B1-like
TCONS_00013512	0.567468	0.0421318	0.50695	BGIOSGA016415	XP_006652009.1 prohibitin-1, mitochondrial-like
TCONS_00020243	0.567512	0.17449	0.348926	BGIOSGA028363	XP_006655856.1 formin-like protein 6
TCONS_00001556	0.567572	0.0341577	0.940314	BGIOSGA004186	XP_006646195.1 pentatricopeptide repeat-containing protein At1g30610, chloroplastic isoform X1
TCONS_00000695	0.567586	0.784825	1.41919	BGIOSGA003286	XP_003567459.1peptidyl-prolyl cis-trans isomerase
TCONS_00007633	0.567908	-0.363715	-1.36923	BGIOSGA006848	XP_006648471.1 peroxisomal membrane protein PMP22-like
TCONS_00009559	0.567977	0.115844	1.09098	BGIOSGA012107	XP_008658422.1stellacyanin isoform X1
TCONS_00001541	0.568121	0.461462	0.0488586	BGIOSGA004172	XP_002467510.1nudC domain-containing protein 2 isoform X1

TCONS_00005837	0.568176	0.218984	1.56892	BGIOSGA007963	XP_006648541.1 tobamovirus multiplication protein 2B-like
TCONS_00021564	0.568665	-0.394747	-0.394283	BGIOSGA022245	XP_024316062.1uncharacterized protein LOC112271179
TCONS_00027231	0.56867	1.33516	1.03006	BGIOSGA020163	XP_006659048.2 phospholipase SGR2
TCONS_00022642	0.568705	0.869862	0.272747	BGIOSGA001181	XP_002453412.1proteasome subunit beta type-3
TCONS_00030838	0.568842	-0.116031	0.16689	BGIOSGA033607	XP_006661676.1 probable cinnamyl alcohol dehydrogenase 1
TCONS_00006440	0.569173	-1.0273	-0.626526	BGIOSGA023312	XP_002452430.1zinc finger protein CONSTANS-LIKE 3
TCONS_00002919	0.569258	1.26943	-1.43737	BGIOSGA017102	XP_006643804.2 galactinol--sucrose galactosyltransferase-like
TCONS_00008116	0.569279	-1.9555	1.19161	#N/A	#N/A
TCONS_00021075	0.569279	#NA	-1.50883	#N/A	#N/A
TCONS_00013069	0.569311	0.0819937	0.374	BGIOSGA004992	NP_001170515.1cytochrome c oxidase assembly protein ctaG
TCONS_00031259	0.569633	1.0837	0.386769	BGIOSGA033078	XP_003571839.1protein CHROMATIN REMODELING 20 isoform X2
TCONS_00007567	0.56967	0.296222	-1.56307	BGIOSGA002288	XP_015688502.1 transcription factor HY5-like
TCONS_00024131	0.569708	0.558218	0.876087	BGIOSGA010613	XP_015694733.1 copper-transporting ATPase PAA1, chloroplastic-like
TCONS_00024745	0.569742	0.186082	-0.1152	BGIOSGA009601	XP_015698938.1 cycloartenol-C-24-methyltransferase 1
TCONS_00003302	0.569859	-0.0243194	1.22763	BGIOSGA018523	XP_015688164.1PREDICTED: uncharacterized protein LOC102702619, partial
TCONS_00033336	0.570052	-1.99713	-0.553453	BGIOSGA019008	XP_004979190.1receptor protein kinase-like protein ZAR1
TCONS_00013534	0.570337	0.770731	0.33248	BGIOSGA009448	XP_004987364.1uncharacterized protein LOC101778812
TCONS_00004907	0.570455	-0.175818	0.523616	BGIOSGA000314	XP_006645182.1 U-box domain-containing protein 45
TCONS_00025621	0.570583	1.72637	0.87918	BGIOSGA033238	XP_021318592.1uncharacterized protein LOC8075867 isoform X2
TCONS_00009185	0.570633	-0.372088	-1.51218	BGIOSGA013826	XP_006649268.1PREDICTED: uncharacterized protein LOC102711510 isoform X1
TCONS_00032708	0.570681	-0.1327	0.875842	BGIOSGA001636	XP_006662064.1 serine/arginine repetitive matrix protein 1-like
TCONS_00021777	0.57071	0.175424	0.99798	BGIOSGA035175	XP_006655872.1 ubiquitin-conjugating enzyme E2 34-like
TCONS_00013463	0.570739	0.61311	0.148285	BGIOSGA028235	XP_004963535.1G-type lectin S-receptor-like serine/threonine-protein kinase LECRK3
TCONS_00001704	0.5708	-0.511713	-0.490955	BGIOSGA019250	XP_006644626.1 tryptophan aminotransferase-related protein 4-like
TCONS_00023539	0.570868	1.64091	-0.459285	BGIOSGA012723	XP_006658008.1 cytochrome P450 709B2-like
TCONS_00035286	0.57094	-3.1045	0.623152	BGIOSGA009029	XP_022680199.1uncharacterized protein LOC101767362 isoform X2
TCONS_00025204	0.570956	-0.250313	0.197423	BGIOSGA024215	XP_006658617.1 microtubule-associated protein TORTIFOLIA1
TCONS_00022846	0.571356	-0.0750365	-0.347657	BGIOSGA008383	XP_021305234.1protein ENHANCED DISEASE RESISTANCE 2-like isoform X2
TCONS_00022570	0.571454	0.125477	0.428724	BGIOSGA020881	XP_015693639.1 VQ motif-containing protein 25
TCONS_00013033	0.571872	0.530863	0.921146	BGIOSGA009954	XP_006650474.1 outer envelope protein 64, chloroplastic
TCONS_00028603	0.572437	-2.87841	-1.20058	BGIOSGA027198	XP_006660446.1 fasciclin-like arabinogalactan protein 2
TCONS_00029140	0.572762	0.143817	0.442791	BGIOSGA014130	XP_006660686.1 probable N-succinyldiaminopimelate aminotransferase DapC
TCONS_00004260	0.572836	0.857039	0.328807	BGIOSGA036755	XP_006644583.1 protein PLASTID TRANSCRIPTIONALLY ACTIVE 7 isoform X2
TCONS_00007052	0.573061	0.551026	0.1271	BGIOSGA017168	XP_006649140.2PREDICTED: uncharacterized protein LOC102710100
TCONS_00015493	0.573068	0.531875	1.15778	BGIOSGA008773	XP_006664813.1 probable aquaporin PIP2-6
TCONS_00007083	0.573123	-0.436285	1.13986	BGIOSGA009264	XP_006648090.2 translation initiation factor eIF-2B subunit epsilon
TCONS_00030429	0.573151	1.21463	-0.534365	BGIOSGA019728	XP_006660888.1 seipin-2-like
TCONS_00001510	0.573284	1.96803	-0.925773	BGIOSGA024463	XP_006644469.1 acidic endochitinase-like
TCONS_00036691	0.573471	1.152	0.954504	BGIOSGA036620	XP_014757281.1pentatricopeptide repeat-containing protein At1g22960, mitochondrial isoform X1
TCONS_00028005	0.573504	0.359707	1.42933	BGIOSGA026981	XP_006659391.1 MKI67 FHA domain-interacting nucleolar phosphoprotein-like
TCONS_00005819	0.573705	-1.96049	-0.519797	BGIOSGA004493	XP_015689117.1 dynein light chain LC6, flagellar outer arm-like
TCONS_00005432	0.573705	#NA	1.06517	#N/A	#N/A
TCONS_00024171	0.57381	#NA	-0.384001	BGIOSGA018909	XP_004958441.1transcription factor MYB61
TCONS_00022201	0.573969	1.09416	0.0189701	BGIOSGA025442	XP_006656061.1 anthocyanidin 5,3-O-glucosyltransferase-like
TCONS_00017278	0.574273	-0.376148	1.93823	BGIOSGA024260	XP_012701226.1disease resistance protein RPP13
TCONS_00030398	0.574348	2.40689	0.246932	BGIOSGA040485	XP_006661460.1 UDP-glucose 4-epimerase 3
TCONS_00011577	0.574616	0.245485	0.319649	BGIOSGA033039	XP_006649411.1 protein GDAP2 homolog
TCONS_00016715	0.575166	-1.29189	-0.195252	BGIOSGA014247	XP_015691327.1 binding partner of ACD11 1 isoform X1
TCONS_00027772	0.575289	-0.0308316	0.673788	BGIOSGA017238	XP_006660026.1 synaptotagmin-5-like
TCONS_00032370	0.575313	-0.140494	0.321393	BGIOSGA033084	XP_004957650.1tetratricopeptide repeat domain-containing protein PYG7, chloroplastic
TCONS_00008305	0.57573	0.201807	0.260218	BGIOSGA006123	XP_006647455.1 vacuolar protein sorting-associated protein 20 homolog 2-like
TCONS_00033025	0.575833	1.86661	-0.710941	BGIOSGA001826	XP_006662757.1 disease resistance protein RGA2-like
TCONS_00031652	0.575882	0.313662	0.866292	BGIOSGA033468	XP_004983603.1SH2 domain-containing protein B isoform X1

TCONS_00017290	0.575939	-0.203916	-0.711202	BGIOSGA002945	XP_003566445.1cytochrome P450 90D2
TCONS_00009846	0.576024	0.620289	0.249598	BGIOSGA012398	XP_003558154.1probable protein phosphatase 2C 32
TCONS_00030933	0.576131	0.583319	0.421625	BGIOSGA032740	XP_006662283.1 zinc finger protein-like 1
TCONS_00002370	0.576365	0.933053	1.33214	BGIOSGA020760	XP_006645189.1 exocyst complex component EXO70A1-like
TCONS_00029509	0.576416	0.506775	0.683181	BGIOSGA031264	XP_006660977.1 mitochondrial amidoxime reducing component 2-like
TCONS_00025235	0.576575	1.0762	0.636384	BGIOSGA016050	XP_006658634.1PREDICTED: uncharacterized protein LOC102720895
TCONS_00011026	0.576996	-3.15504	1.35683	BGIOSGA013628	XP_021316952.1uncharacterized protein LOC8086185
TCONS_00026189	0.577372	-0.059951	0.213727	BGIOSGA003631	XP_015694103.1PREDICTED: uncharacterized protein LOC102712736
TCONS_00029738	0.577755	1.30476	0.943873	BGIOSGA025162	XP_004956467.1mediator of RNA polymerase II transcription subunit 14 isoform X1
TCONS_00023955	0.577759	0.740621	0.463576	BGIOSGA033110	XP_006658722.1 protein FREE1
TCONS_00030403	0.577905	0.686537	0.272521	BGIOSGA025571	XP_006660876.1 mediator of RNA polymerase II transcription subunit 10b-like
TCONS_00029496	0.577968	-0.320147	-0.51423	BGIOSGA023380	XP_002460652.1hydrophobic protein OSR8
TCONS_00002933	0.57799	0.417544	-0.00472898	BGIOSGA023218	XP_006643815.1PREDICTED: uncharacterized protein At2g34160-like
TCONS_00004741	0.578089	-0.808349	0.61558	BGIOSGA000474	XP_006645031.1PREDICTED: uncharacterized protein LOC102716268
TCONS_00014930	0.578309	0.471845	0.566201	BGIOSGA003571	XP_006654389.1 LEC14B protein-like
TCONS_00026562	0.578319	-0.192778	-0.401873	BGIOSGA028624	XP_010236682.2histone-lysine N-methyltransferase, H3 lysine-9 specific SUVH6 isoform X2
TCONS_00018786	0.578335	0.778804	0.310835	BGIOSGA018636	XP_015692781.1 thioredoxin-like 2, chloroplastic isoform X2
TCONS_00007528	0.579079	0.206963	0.456095	BGIOSGA006956	XP_024317460.1protein ABHD18 isoform X2
TCONS_00006724	0.579143	0.563373	0.955472	BGIOSGA008894	XP_006647772.1 carbamoyl-phosphate synthase small chain, chloroplastic
TCONS_00027315	0.57963	1.1268	1.9449	BGIOSGA035977	XP_015695790.1 multiple organellar RNA editing factor 9, chloroplastic-like
TCONS_00028046	0.580139	0.468922	-0.717283	BGIOSGA028862	XP_015695937.1 14-3-3-like protein GF14-C
TCONS_00026969	0.580231	1.17125	1.62337	BGIOSGA017636	XP_015695670.1 chaperone protein dnaJ 10-like
TCONS_00028707	0.580397	-0.427858	-1.03288	BGIOSGA035064	XP_002459987.1chlorophyll a-b binding protein of LHCII type 1
TCONS_00012998	0.580946	0.337529	0.580926	BGIOSGA009988	XP_006650440.1 alkaline ceramidase 3
TCONS_00006294	0.581082	1.08394	0.986831	BGIOSGA016497	XP_006647401.1PREDICTED: uncharacterized protein LOC102700496
TCONS_00007263	0.581118	1.24576	1.64365	BGIOSGA007219	XP_003573630.1protein ACCUMULATION AND REPLICATION OF CHLOROPLASTS 6, chloroplastic
TCONS_00035440	0.581181	-0.0883732	0.689806	BGIOSGA034648	XP_021301782.1protein DETOXIFICATION 44, chloroplastic isoform X3
TCONS_00006565	0.581466	0.639619	0.245251	BGIOSGA013829	XP_006647617.1 GTP-binding protein YPTM2
TCONS_00028858	0.581937	0.874153	0.74239	BGIOSGA030608	XP_002460129.1GTP-binding protein BRASSINAZOLE INSENSITIVE PALE GREEN 2, chloroplastic
TCONS_00002743	0.582003	0.474566	0.70255	BGIOSGA002475	XP_015692431.1PREDICTED: uncharacterized protein LOC102700196
TCONS_00014250	0.582073	-2.27803	-0.208858	BGIOSGA017391	XP_015691838.1 WAT1-related protein At1g09380-like
TCONS_00028204	0.58211	0.333305	-0.473661	BGIOSGA030937	XP_015695449.1PREDICTED: uncharacterized protein LOC102716059
TCONS_00017640	0.582328	-1.38472	1.36566	#N/A	#N/A
TCONS_00032821	0.582352	-0.433128	0.111169	BGIOSGA012069	XP_006662655.1 macrophage migration inhibitory factor homolog isoform X2
TCONS_00021755	0.582691	-0.771573	-0.654441	BGIOSGA016309	XP_006655861.1 serine carboxypeptidase II-2 isoform X1
TCONS_00008417	0.58293	-0.165464	1.59936	BGIOSGA006000	XP_006647542.2 phenylalanine ammonia-lyase
TCONS_00034301	0.582937	0.343024	0.725322	BGIOSGA016109	XP_006663203.1 cation/calcium exchanger 5
TCONS_00016247	0.583014	0.532086	-0.127932	BGIOSGA006018	XP_021318113.1eukaryotic translation initiation factor 5B
TCONS_00028376	0.583029	-0.0548288	0.0160834	BGIOSGA026587	NP_001150532.1lipid transfer protein precursor
TCONS_00018749	0.583345	-3.97077	-1.73645	BGIOSGA018672	XP_006654125.1 pseudo histidine-containing phosphotransfer protein 2
TCONS_00030010	0.583657	0.952008	0.582191	BGIOSGA013186	XP_003578091.1plant UBX domain-containing protein 2
TCONS_00026725	0.58367	1.52714	0.275115	BGIOSGA005389	XP_015696212.1PREDICTED: uncharacterized protein LOC102703511
TCONS_00007099	0.584118	1.32838	-0.506415	BGIOSGA009608	XP_004954347.128 kDa ribonucleoprotein, chloroplastic
TCONS_00029541	0.584142	0.885426	-0.750065	BGIOSGA024018	XP_015696458.1 beta-amylase 2, chloroplastic-like
TCONS_00011680	0.584404	0.419459	1.58179	BGIOSGA018232	XP_006649510.2 ethylene-responsive transcription factor 1-like
TCONS_00028023	0.584597	-1.40676	-0.0707663	BGIOSGA015022	XP_015695836.1 probable 2-oxoglutarate-dependent dioxygenase AOP1
TCONS_00019609	0.584665	0.956641	1.29634	BGIOSGA004535	XP_006654623.2 signal recognition particle 54 kDa protein 2-like
TCONS_00010764	0.58487	0.49138	1.16882	BGIOSGA013389	XP_006651726.1 adenylosuccinate synthetase 2, chloroplastic
TCONS_00010412	0.584991	0.602631	0.852548	BGIOSGA016476	XP_006652325.1 26S proteasome non-ATPase regulatory subunit 11 homolog
TCONS_00037188	0.585087	0.681625	0.985446	BGIOSGA012031	XP_006664021.1 protein yippee-like At4g27745
TCONS_00024697	0.585176	0.86855	0.258888	BGIOSGA019154	XP_015695157.1 thioredoxin H2-1-like
TCONS_00014664	0.585226	0.732466	0.565334	BGIOSGA016828	XP_003581460.3calcium-dependent protein kinase 27
TCONS_00000843	0.585284	1.3	1.53724	BGIOSGA003432	XP_004968693.1dihydrolipoyl dehydrogenase 1, chloroplastic

TCONS_00018872	0.585592	1.61925	1.4254	BGIOSGA000017	XP_006654164.1 eukaryotic translation initiation factor 3 subunit L-like
TCONS_00018735	0.585736	0.142118	-0.549884	BGIOSGA018681	NP_001335912.1late embryogenesis abundant protein
TCONS_00026596	0.585961	1.47194	0.440421	BGIOSGA029045	XP_002444325.1uncharacterized protein LOC8070074
TCONS_00037049	0.586007	0.348275	1.25679	BGIOSGA011317	XP_006656125.1 agamous-like MADS-box protein AGL62
TCONS_00003848	0.586248	0.686321	0.662101	BGIOSGA009681	XP_006646020.1 calumenin-like
TCONS_00009513	0.586346	1.54928	0.32257	BGIOSGA012061	XP_015690347.1 pentatricopeptide repeat-containing protein At2g16880-like
TCONS_00019761	0.586371	0.341398	-0.304136	BGIOSGA017595	XP_006654759.1 photosynthetic NDH subunit of subcomplex B 3, chloroplastic
TCONS_00010933	0.586384	0.393231	0.895445	BGIOSGA032925	XP_004981815.1probable histone H2A variant 3
TCONS_00031465	0.586385	0.257681	-0.00953728	BGIOSGA033282	XP_006662512.1 pyridoxal reductase, chloroplastic
TCONS_00004007	0.586499	1.29969	1.20122	BGIOSGA019256	XP_006644381.1 uroporphyrinogen decarboxylase 1, chloroplastic
TCONS_00026705	0.586632	1.2023	0.80227	BGIOSGA029792	XP_006659465.1 1-phosphatidylinositol-3-phosphate 5-kinase FAB1B-like
TCONS_00033657	0.586728	-3.16807	-0.541426	BGIOSGA024896	XP_015697919.1 phytosulfokines-like
TCONS_00014126	0.58675	0.218564	0.923893	BGIOSGA033025	XP_015692216.1 neurofilament medium polypeptide-like
TCONS_00019039	0.586769	1.01525	-0.478038	BGIOSGA030989	XP_006654252.1 reticulon-like protein B1
TCONS_00021144	0.587058	0.638394	0.321063	BGIOSGA014555	XP_006656301.1 ATP synthase subunit O, mitochondrial
TCONS_00001955	0.587279	-0.859563	-0.968819	BGIOSGA026589	XP_006644825.1 nuclear transport factor 2-like
TCONS_00022450	0.587378	0.487864	1.08461	BGIOSGA033270	XP_006656175.1 ent-sandaracopimaradiene 3-hydroxylase-like
TCONS_00025424	0.587419	0.606839	-0.21207	BGIOSGA023398	XP_006657896.1 NADH dehydrogenase [ubiquinone] iron-sulfur protein 4, mitochondrial
TCONS_00031044	0.587516	0.179975	1.42913	BGIOSGA015758	XP_006661745.1 proteasome assembly chaperone 3
TCONS_00011656	0.587748	0.6094	-1.55498	BGIOSGA011353	XP_008660489.1uncharacterized protein LOC103639524
TCONS_00020087	0.587748	1.48387	1.90446	#N/A	#N/A
TCONS_00035921	0.587919	1.13348	-0.487388	BGIOSGA037305	XP_004962578.1photosystem I reaction center subunit XI, chloroplastic
TCONS_00019545	0.587975	1.16139	0.176107	BGIOSGA013543	XP_006654579.1 calmodulin-2
TCONS_00003864	0.588159	-0.774418	-1.04786	BGIOSGA020277	XP_006644310.1PREDICTED: uncharacterized protein LOC102717014 isoform X2
TCONS_00029253	0.588197	1.02393	0.0288398	#N/A	#N/A
TCONS_00026595	0.588231	0.0817596	0.82814	BGIOSGA028661	XP_006660127.1 5'-adenylylsulfate reductase-like 4
TCONS_00004768	0.588354	0.195087	0.67003	BGIOSGA025888	NP_001151279.1uncharacterized LOC100284912
TCONS_00029715	0.588415	0.60865	0.76525	BGIOSGA029060	XP_022679479.1uncharacterized protein LOC101761678 isoform X2
TCONS_00002201	0.588526	0.731886	1.2165	BGIOSGA016353	XP_006645052.1PREDICTED: uncharacterized protein LOC102722165 isoform X2
TCONS_00032387	0.58853	0.162249	0.697797	BGIOSGA031736	XP_015697396.1 WD repeat-containing protein 26-like
TCONS_00024600	0.588751	-0.627074	0.650902	BGIOSGA025190	XP_003579406.1putative F-box/LRR-repeat protein At3g28410
TCONS_00033031	0.588763	0.181862	0.556544	BGIOSGA036575	XP_006662760.1PREDICTED: uncharacterized protein LOC102703623
TCONS_00022425	0.588795	-0.0658779	1.28221	BGIOSGA002221	XP_015693407.1 acid phosphatase 1-like
TCONS_00024288	0.588876	-0.587575	2.81953	BGIOSGA013880	XP_015695027.1 momilactone A synthase-like
TCONS_00006188	0.589044	-1.39206	1.02669	BGIOSGA030890	XP_008783998.1 zinc finger A20 and AN1 domain-containing stress-associated protein 5
TCONS_00010963	0.589252	0.975282	0.140811	BGIOSGA025518	XP_006650589.1 protein-lysine N-methyltransferase N6amt2
TCONS_00012439	0.589556	-0.849248	-0.281562	BGIOSGA010544	XP_006651494.1PREDICTED: uncharacterized protein LOC102714635
TCONS_00003397	0.589838	-0.0447318	-0.139973	BGIOSGA006341	XP_006644110.1 calvin cycle protein CP12-1, chloroplastic
TCONS_00036769	0.590013	1.32944	0.210562	BGIOSGA011870	XP_021302175.1uncharacterized protein LOC8076284
TCONS_00018545	0.590108	1.13391	0.573003	BGIOSGA018888	XP_015693292.1 metal tolerance protein 1
TCONS_00031657	0.590127	1.73994	-0.26655	BGIOSGA033472	XP_015697208.1 haloacid dehalogenase-like hydrolase domain-containing protein Sgpp isoform X1
TCONS_00030770	0.5902	-0.978333	-0.561198	BGIOSGA028952	XP_021304755.1ABC transporter G family member 11
TCONS_00016589	0.590269	0.209555	0.487656	BGIOSGA024249	XP_006652798.1 probable protein phosphatase 2C 44
TCONS_00016316	0.59038	0.794051	1.71137	BGIOSGA014651	XP_006652578.1 histidine triad nucleotide-binding protein 3
TCONS_00032233	0.590387	0.876802	1.09296	BGIOSGA026802	XP_006662362.2 alpha-ketoglutarate-dependent dioxygenase alkB homolog 6
TCONS_00019330	0.590462	-0.179627	0.336673	BGIOSGA034973	XP_015693030.1 probable beta-1,3-galactosyltransferase 8 isoform X3
TCONS_00026177	0.590679	0.27113	0.643791	BGIOSGA031950	NP_001150714.1catalytic/ oxidoreductase, acting on NADH or NADPH
TCONS_00025615	0.590841	-5.90666	-1.28626	BGIOSGA023820	XP_006658043.1 uncharacterized GPI-anchored protein At5g19250-like
TCONS_00001976	0.591007	0.0515762	1.06527	BGIOSGA015096	XP_006644842.1 ethylene-responsive transcription factor 11
TCONS_00004309	0.591122	-0.471354	1.17416	BGIOSGA020250	XP_006646271.1 probable starch synthase 4, chloroplastic/amyloplastic
TCONS_00027732	0.591226	0.342406	1.02116	#N/A	#N/A
TCONS_00025501	0.591385	0.342218	2.02075	BGIOSGA023932	XP_003516788.1mitochondrial import inner membrane translocase subunit TIM17-2
TCONS_00036789	0.59145	0.786246	1.00408	BGIOSGA006063	XP_006663869.2 dynamin-like protein ARC5
TCONS_00013950	0.591471	-0.515481	-0.509876	BGIOSGA029174	XP_015692366.1 cysteine proteinase 1-like

TCONS_00012185	0.591716	0.424236	0.422501	BGIOSGA010820	XP_006650018.1PREDICTED: uncharacterized protein LOC102715277
TCONS_00015156	0.591848	1.86774	0.867575	BGIOSGA024374	XP_006653015.1 two-component response regulator ORR6-like
TCONS_00004671	0.592143	1.34133	-0.343741	BGIOSGA000545	XP_019709579.1 ATP-dependent Clp protease adapter protein CLPS1, chloroplastic-like
TCONS_00024062	0.592437	-0.721341	-2.22976	BGIOSGA023928	XP_024312789.1protein EGG APPARATUS-1-like
TCONS_00021360	0.592497	2.04517	0.611082	BGIOSGA023588	XP_004966407.1WAS/WASL-interacting protein family member 1
TCONS_00006282	0.592503	-0.00370744	-0.0604815	BGIOSGA008441	XP_006647391.1 glyoxylate/succinic semialdehyde reductase 1
TCONS_00008319	0.592556	1.41337	0.851203	BGIOSGA014843	XP_006647464.1 cytosolic Fe-S cluster assembly factor NBP35-like
TCONS_00026489	0.592836	1.62738	0.502495	BGIOSGA014371	XP_006659350.1 iron-sulfur assembly protein IscA-like 2, mitochondrial
TCONS_00025552	0.592836	0.01857	0.432106	BGIOSGA024293	XP_006658848.1 non-specific lipid transfer protein-like 1
TCONS_00015087	0.593011	0.44836	0.607945	BGIOSGA017266	XP_006652935.1 COP9 signalosome complex subunit 5b-like
TCONS_00034322	0.593016	-0.381411	0.905689	BGIOSGA036616	XP_006663219.1PREDICTED: uncharacterized protein OB11G0041L07_1
TCONS_00007562	0.593294	0.433584	1.51946	BGIOSGA023216	XP_006647046.1PREDICTED: uncharacterized protein At2g34160-like
TCONS_00021209	0.59335	-2.98205	-1.5692	BGIOSGA000577	XP_021306004.1RING-H2 finger protein ATL74
TCONS_00030363	0.593654	0.610983	-0.161013	BGIOSGA034507	XP_022679893.1PHD finger protein rhinoceros
TCONS_00026681	0.594051	0.222895	0.834444	BGIOSGA005420	XP_006659453.2 calcineurin subunit B-like
TCONS_00031362	0.5945	-2.56841	#NA	BGIOSGA037599	XP_008663663.1putative ripening-related protein 5
TCONS_00020429	0.594522	1.21263	1.45135	BGIOSGA035978	XP_006656838.1PREDICTED: uncharacterized protein LOC102716225
TCONS_00022803	0.594633	-0.130912	0.773132	#N/A	#N/A
TCONS_00015175	0.594708	0.186224	1.95094	BGIOSGA017357	XP_015691670.1 probable N-acetylglucosaminyl-phosphatidylinositol de-N-acetylase isoform X2
TCONS_00010160	0.594708	0.823654	0.427375	BGIOSGA027788	XP_004984268.1protein DMP2
TCONS_00023716	0.594861	0.689634	0.63744	BGIOSGA025754	XP_006657728.1PREDICTED: uncharacterized protein LOC102717449
TCONS_00004885	0.595543	0.529523	0.57907	BGIOSGA000334	NP_001130281.1IM30 protein-like protein
TCONS_00005732	0.595573	-0.241544	0.205415	BGIOSGA007847	XP_006647107.1 actin-related protein 2/3 complex subunit 3
TCONS_00005851	0.595662	1.82248	0.424972	BGIOSGA007977	XP_015689559.1 protein FAR1-RELATED SEQUENCE 5-like isoform X2
TCONS_00037507	0.595662	-2.24791	-0.0725277	BGIOSGA027060	XP_004963131.1auxin-responsive protein SAUR71
TCONS_00021635	0.595716	1.20188	0.300917	BGIOSGA016300	XP_006655798.1 proteasome subunit beta type-5-A-like
TCONS_00011173	0.596006	1.16669	1.483	BGIOSGA022989	XP_006650758.2 5'-adenylylsulfate reductase-like 5
TCONS_00023024	0.596111	0.769921	0.642458	BGIOSGA009647	XP_004955369.1putative F-box/LRR-repeat protein At4g15060
TCONS_00005544	0.596244	0.0489717	-0.451246	BGIOSGA007654	NP_001150164.1fiber protein Fb15
TCONS_00012569	0.596248	1.61806	0.963084	BGIOSGA010407	XP_006647303.1 DNA/RNA-binding protein KIN17
TCONS_00034262	0.596252	-1.13762	-0.198002	BGIOSGA022288	XP_006664849.1 mitochondrial acidic protein MAM33
TCONS_00034214	0.596313	0.0360936	0.4203	BGIOSGA036715	XP_006664298.1 bifunctional aspartokinase/homoserine dehydrogenase 1 isoform X3
TCONS_00033317	0.59641	-0.663885	-0.99195	BGIOSGA011300	XP_006662894.1PREDICTED: uncharacterized protein LOC102700469
TCONS_00018800	0.596524	0.347289	-0.293919	BGIOSGA018620	XP_006655086.1PREDICTED: uncharacterized protein LOC102721554
TCONS_00023424	0.596684	1.32719	1.2998	BGIOSGA025457	XP_006657589.1 putative S-adenosyl-L-methionine-dependent methyltransferase MAV_4873
TCONS_00006763	0.597013	1.06078	0.576665	BGIOSGA003635	XP_006647803.1 fimbrin-4-like
TCONS_00006532	0.597043	1.29853	0.354782	BGIOSGA008699	XP_004953257.1DEAD-box ATP-dependent RNA helicase 8
TCONS_00000011	0.597499	0.32863	-1.48729	BGIOSGA002582	XP_006643629.1 chaperone protein dnaJ 20, chloroplastic-like isoform X1
TCONS_00024674	0.597623	0.538022	0.693481	BGIOSGA009639	XP_006657506.1 tryptophan synthase alpha chain-like
TCONS_00036827	0.59806	-0.481135	0.23036	BGIOSGA034277	XP_006663894.1 DNA-directed RNA polymerases I and III subunit RPAC2
TCONS_00015548	0.598075	0.263628	0.596441	BGIOSGA015424	XP_015691329.1PREDICTED: uncharacterized protein LOC102717513
TCONS_00029711	0.598265	-0.766056	-0.301113	BGIOSGA030112	XP_006660480.1 nucleoporin GLE1-like
TCONS_00029185	0.598434	0.692913	0.882369	BGIOSGA021768	XP_006660719.1 phosphoribosylaminoimidazole-succinocarboxamide synthase, chloroplastic
TCONS_00023642	0.598487	-0.632782	1.44064	BGIOSGA011918	XP_023157558.1mitochondrial fission regulator 1-like
TCONS_00023565	0.598492	-1.29993	0.0445549	BGIOSGA025595	XP_006658507.1 photosystem I reaction center subunit IV, chloroplastic-like
TCONS_00004853	0.598554	0.450774	0.670398	BGIOSGA019891	XP_015692262.1 UPF0301 protein Cag_1601-like
TCONS_00023475	0.598635	1.06142	0.88389	BGIOSGA013925	XP_010228177.1serine/threonine protein phosphatase 2A 57 kDa regulatory subunit B' theta isoform
TCONS_00007575	0.598666	0.796432	0.904101	BGIOSGA011435	XP_006647057.1 26S protease regulatory subunit 8 homolog A
TCONS_00000019	0.599525	-0.182392	0.585199	BGIOSGA002230	XP_015694374.1PREDICTED: uncharacterized protein LOC102710794
TCONS_00016537	0.599607	1.60143	0.853644	BGIOSGA033797	XP_006652741.1 secretory carrier-associated membrane protein 6

TCONS_00012892	0.599612	0.217962	0.363282	BGIOSGA010091	XP_004976546.1nucleoporin NSP1 isoform X1
TCONS_00007135	0.600353	0.654855	0.472051	BGIOSGA023653	XP_006648148.1 signal peptide peptidase-like 4
TCONS_00008840	0.600402	-1.06357	0.326698	BGIOSGA036737	XP_021308134.1putative leucine-rich repeat receptor-like serine/threonine-protein kinase At2g04300
TCONS_00027589	0.600404	2.25707	0.872513	BGIOSGA015988	XP_015695871.1 BTB/POZ and MATH domain-containing protein 2-like
TCONS_00006097	0.600721	0.584838	0.470399	BGIOSGA007466	XP_006659718.1 mitochondrial import receptor subunit TOM9-2-like
TCONS_00013608	0.601393	1.46733	-0.589378	BGIOSGA015727	XP_006664931.1 glyoxylate/hydroxypyruvate reductase HPR3-like
TCONS_00007485	0.601393	1.09536	0.995584	BGIOSGA023333	XP_004951637.1probable glycosyltransferase At5g03795
TCONS_00021495	0.601489	0.979758	1.32465	BGIOSGA022055	XP_006655726.1 syntaxin-43-like
TCONS_00035662	0.601582	-3.57731	-3.17481	BGIOSGA019700	XP_004977234.1myb-related protein Zm38
TCONS_00009526	0.601583	1.59625	1.08768	BGIOSGA012074	XP_006649592.1 DNA repair protein UVH3 isoform X1
TCONS_00023884	0.6017	-0.0226124	0.864626	BGIOSGA011733	XP_010504143.1 histone H4
TCONS_00002748	0.601739	0.122935	1.14673	BGIOSGA007609	XP_006643700.1 transcription repressor MYB4-like
TCONS_00005461	0.601803	-1.83216	-1.03401	BGIOSGA007570	XP_004951835.1probable arabinose 5-phosphate isomerase
TCONS_00006582	0.601846	0.0947859	-0.590504	BGIOSGA016885	XP_006647628.1 acetylglutamate kinase-like
TCONS_00008798	0.601949	1.88166	1.28371	BGIOSGA022590	NP_001141133.1putative HSP20-like chaperone domain family protein
TCONS_00024253	0.60213	1.10498	0.821007	BGIOSGA022503	XP_006658050.2 BTB/POZ and MATH domain-containing protein 1-like
TCONS_00021749	0.602612	0.508858	0.822632	BGIOSGA038193	XP_002437974.1pentatricopeptide repeat-containing protein At3g25210, mitochondrial
TCONS_00008595	0.602662	-1.21367	-0.850649	BGIOSGA014487	XP_004953470.1uncharacterized protein LOC101766680
TCONS_00015987	0.603007	1.07827	0.0470735	BGIOSGA033400	XP_003579865.1RING-H2 finger protein ATL52
TCONS_00033236	0.603345	0.742401	1.28025	BGIOSGA021834	XP_021317386.1putative protease Do-like 14 isoform X1
TCONS_00032601	0.603399	1.33621	0.154418	BGIOSGA005166	XP_006661977.1 glutathione S-transferase U17-like
TCONS_00004749	0.603687	1.62392	1.00697	BGIOSGA029105	XP_006645040.1 nematode resistance protein-like HSPRO1
TCONS_00027048	0.604149	0.389089	0.961789	BGIOSGA034060	XP_006659650.1 ankyrin repeat-containing protein At5g02620-like
TCONS_00016787	0.60461	0.579955	0.328279	BGIOSGA007546	XP_022680914.1probable protein phosphatase 2C 45 isoform X2
TCONS_00012124	0.604707	0.196401	0.547071	BGIOSGA010885	XP_006649962.2 bifunctional TENA2 protein
TCONS_00011017	0.605099	1.23583	1.50358	BGIOSGA026164	XP_006651844.1 serine/threonine-protein kinase SAPK8
TCONS_00019904	0.605229	0.204704	1.96184	BGIOSGA028172	XP_015693153.1 inactive protein kinase SELMODRAFT_444075-like
TCONS_00024618	0.605731	0.767976	0.778404	BGIOSGA024768	XP_006657468.1 small RNA 2'-O-methyltransferase-like
TCONS_00011981	0.606	0.016363	0.303082	BGIOSGA033070	XP_006649826.1 tropinone reductase homolog At5g06060-like
TCONS_00016104	0.606158	0.431672	0.568079	BGIOSGA014874	XP_006652418.1 protein FLX-like 2
TCONS_00009453	0.606241	1.161	1.69142	BGIOSGA027984	XP_006651108.1PREDICTED: uncharacterized protein LOC102707853, partial
TCONS_00019556	0.607224	-0.00472235	-0.518115	BGIOSGA031655	XP_004970279.1probable E3 ubiquitin-protein ligase RHC1A
TCONS_00031480	0.607283	-0.47299	-0.633929	BGIOSGA016354	NP_001148225.1catalytic/ hydrolase
TCONS_00009039	0.607312	0.40306	-0.328945	BGIOSGA005367	XP_002454776.1haloacid dehalogenase-like hydrolase domain-containing protein At2g33255 isoform X3
TCONS_00001834	0.607472	0.821923	0.537179	BGIOSGA018721	XP_006644712.1 FBD-associated F-box protein At4g10400-like
TCONS_00013521	0.607593	0.265365	0.483072	BGIOSGA036821	XP_015690928.1 PTI1-like tyrosine-protein kinase 1
TCONS_00002927	0.608254	0.0636225	-1.46762	BGIOSGA007462	XP_015691981.1PREDICTED: uncharacterized protein LOC102710060
TCONS_00026276	0.608274	0.689215	1.46499	BGIOSGA007237	XP_015695703.1 syntaxin-52-like
TCONS_00004842	0.608555	1.87075	0.823097	BGIOSGA026745	XP_006645133.1 MADS-box transcription factor 2
TCONS_00016920	0.608792	1.18834	1.58118	BGIOSGA011560	XP_006653068.2 cationic peroxidase SPC4-like
TCONS_00031561	0.609022	0.257938	1.82655	BGIOSGA033379	XP_003563429.1uncharacterized protein LOC100845879
TCONS_00029876	0.609092	-0.19389	0.274862	BGIOSGA000871	XP_006660552.1 ubiquitin domain-containing protein 1-like
TCONS_00035734	0.609476	0.12605	-0.737251	BGIOSGA028614	XP_006663907.1 AP-2 complex subunit sigma
TCONS_00022747	0.609505	0.322385	-0.290879	BGIOSGA014928	XP_015694048.1 ATP-dependent zinc metalloprotease FTSH 2, chloroplastic
TCONS_00015995	0.610047	0.269215	0.61776	BGIOSGA019588	XP_003576120.1uncharacterized protein LOC100829625
TCONS_00011583	0.610355	0.960221	0.536863	BGIOSGA026897	XP_015689765.1 4-coumarate--CoA ligase-like 1
TCONS_00002125	0.610407	-0.809873	-1.88125	BGIOSGA033858	XP_006646457.1 putative respiratory burst oxidase homolog protein H
TCONS_00005048	0.610412	0.198449	-0.378483	BGIOSGA010948	XP_006645290.1 protein SRG1-like
TCONS_00013753	0.611006	0.798552	0.971524	BGIOSGA015516	XP_006652112.1 probable E3 ubiquitin-protein ligase HIP1
TCONS_00002985	0.611219	1.36326	0.566148	BGIOSGA037254	XP_002455034.1serine/threonine-protein kinase tricorner
TCONS_00012550	0.611813	0.197329	0.0695137	BGIOSGA012976	XP_015691034.1 magnesium-chelatase subunit ChlI, chloroplastic
TCONS_00004910	0.611847	1.67885	1.88746	BGIOSGA000311	XP_004970953.1protein PLASTID TRANSCRIPTIONALLY ACTIVE 10
TCONS_00027240	0.612069	0.478714	0.00366606	BGIOSGA013603	XP_015695712.1 succinate dehydrogenase subunit 6, mitochondrial

TCONS_00016848	0.612078	0.913286	0.564648	BGIOSGA022626	XP_006653008.1 inactive poly [ADP-ribose] polymerase RCD1-like isoform X2
TCONS_00028438	0.612203	0.303064	-0.329444	BGIOSGA023006	XP_015696191.1 probable L-ascorbate peroxidase 4 isoform X2
TCONS_00028677	0.612258	-0.492784	-1.04772	BGIOSGA030426	XP_006660493.1 scyllo-inositol 2-dehydrogenase (NADP(+))
TCONS_00007127	0.612305	0.38098	0.491529	BGIOSGA009311	XP_006648139.1 ATG8-interacting protein 1-like
TCONS_00020781	0.61269	1.29296	0.933415	BGIOSGA016458	XP_006656111.1 GTP-binding protein BRASSINAZOLE INSENSITIVE PALE GREEN 2, chloroplastic-like
TCONS_00000901	0.612874	0.451408	0.490877	BGIOSGA025478	XP_006660454.1 pentatricopeptide repeat-containing protein At4g14850-like
TCONS_00028583	0.613601	0.672673	0.62069	BGIOSGA030328	XP_004970801.1protein CLMP1
TCONS_00015141	0.613768	0.35795	0.724552	BGIOSGA011760	XP_008437332.1 heavy metal-associated isoprenylated plant protein 3
TCONS_00011355	0.613874	1.06835	0.956126	BGIOSGA013967	XP_006650918.1PREDICTED: uncharacterized protein LOC102719753
TCONS_00030741	0.614256	-1.95473	-0.332775	BGIOSGA015511	XP_010234589.1probable amidase At4g34880
TCONS_00035780	0.614423	0.845795	-2.49125	BGIOSGA020552	NP_001152518.110-deacetylbaccatin III 10-O-acetyltransferase
TCONS_00028122	0.614663	-1.20026	-0.35604	BGIOSGA026866	XP_006660189.1 cytokinin dehydrogenase 11
TCONS_00017604	0.614665	1.88259	0.760946	BGIOSGA010940	XP_006654285.1 ran-binding protein 1 homolog c
TCONS_00009167	0.614706	-2.02129	-0.64377	BGIOSGA011693	XP_006649252.1PREDICTED: uncharacterized protein LOC102706920
TCONS_00011318	0.614968	0.773383	0.0444617	BGIOSGA025510	XP_006650885.1 serine/threonine protein phosphatase 2A 57 kDa regulatory subunit B' theta isoform-like
TCONS_00026357	0.615039	1.46408	0.606165	BGIOSGA028210	XP_006659305.1 50S ribosomal protein L15
TCONS_00025951	0.615141	1.86374	1.13615	#N/A	#N/A
TCONS_00027190	0.615172	0.209427	1.17696	BGIOSGA025359	XP_015696147.1 serine/threonine-protein kinase STY8-like
TCONS_00010395	0.615357	-1.01507	-0.699875	BGIOSGA012984	XP_006651553.1 haloacid dehalogenase-like hydrolase domain-containing protein At3g48420
TCONS_00008083	0.615365	0.673567	1.12762	BGIOSGA014172	XP_004960168.1protein HEAT INTOLERANT 4
TCONS_00012860	0.615365	#NA	-2.78265	BGIOSGA032721	XP_006650369.2 cytochrome P450 87A3-like
TCONS_00024615	0.615429	0.827726	0.0547137	BGIOSGA024772	XP_015695342.1 D-lactate dehydrogenase [cytochrome], mitochondrial
TCONS_00000267	0.615476	0.0484863	0.520187	BGIOSGA002326	XP_006643786.1 dehydration-responsive element-binding protein 2A
TCONS_00023656	0.61584	-0.102256	0.192309	BGIOSGA000751	XP_015694631.1PREDICTED: uncharacterized protein At4g14100-like, partial
TCONS_00015152	0.615917	0.873776	0.918891	BGIOSGA017332	XP_003580790.1plant UBX domain-containing protein 7
TCONS_00011315	0.616483	-0.025053	-3.31928	BGIOSGA033366	XP_006652014.1 probable thiol methyltransferase 2
TCONS_00003426	0.61652	0.157236	0.301104	BGIOSGA001792	XP_006644121.1 malignant T-cell-amplified sequence 1 homolog
TCONS_00014073	0.616613	0.565116	1.00807	BGIOSGA000293	XP_004975358.1TBC1 domain family member 13
TCONS_00020031	0.616737	0.0448319	1.13178	BGIOSGA022029	XP_020395337.1succinate dehydrogenase subunit 8A, mitochondrial
TCONS_00002498	0.616783	-0.133471	0.147274	BGIOSGA009736	XP_006646669.1 putative tyrosine-protein phosphatase OCA1
TCONS_00034579	0.616876	0.913049	0.117141	BGIOSGA031884	XP_015697812.1 mini-ribonuclease 3-like
TCONS_00007767	0.617014	0.239189	0.426158	BGIOSGA006718	XP_015688548.1PREDICTED: uncharacterized protein LOC102709816
TCONS_00030889	0.617138	0.343605	0.0363167	BGIOSGA032692	XP_006661688.1 probable chromo domain-containing protein LHP1
TCONS_00014640	0.617269	1.63118	1.55879	BGIOSGA024158	XP_006652543.1PREDICTED: uncharacterized protein LOC102715655
TCONS_00002265	0.617549	-1.45884	-0.226917	BGIOSGA016295	XP_002456719.1myb-related protein 308
TCONS_00028383	0.617765	-0.240542	1.11644	BGIOSGA026579	XP_002444685.1mitochondrial import inner membrane translocase subunit TIM8
TCONS_00007001	0.618181	-0.97141	0.593276	BGIOSGA020196	XP_006648034.1 probable alpha,alpha-trehalose-phosphate synthase [UDP-forming] 8
TCONS_00024221	0.618329	-0.476181	1.21254	BGIOSGA007293	XP_015695392.1 calcium-binding protein 39-like
TCONS_00037230	0.618455	-0.204424	-0.739241	BGIOSGA033670	XP_003576098.1phosphoserine phosphatase, chloroplastic
TCONS_00024196	0.618539	0.0662497	0.168476	BGIOSGA037064	XP_006658029.1 eukaryotic translation initiation factor 6-2
TCONS_00002339	0.618626	1.09575	1.07136	BGIOSGA004981	XP_006646563.1 pentatricopeptide repeat-containing protein At3g02650, mitochondrial
TCONS_00014803	0.618677	0.358784	-1.08745	BGIOSGA016973	XP_006653699.1PREDICTED: uncharacterized protein LOC102717895
TCONS_00005761	0.618694	0.171489	0.912161	BGIOSGA007873	XP_006648496.1 probable splicing factor 3A subunit 1
TCONS_00004813	0.618842	0.697151	1.06075	BGIOSGA000402	XP_006645098.1 ubiquitin-like domain-containing CTD phosphatase
TCONS_00036222	0.619281	-0.344031	0.126021	BGIOSGA027306	XP_004968528.1uncharacterized protein LOC101768011
TCONS_00016786	0.619724	-0.0863729	0.627292	BGIOSGA014181	XP_004963393.1uncharacterized protein LOC101785360
TCONS_00002863	0.619759	0.710127	0.382984	BGIOSGA013655	XP_015694902.1 mitochondrial import inner membrane translocase subunit TIM14-1-like
TCONS_00012450	0.619787	-0.292317	0.000750517	BGIOSGA012866	XP_015690581.1PREDICTED: uncharacterized protein LOC102721626
TCONS_00004980	0.619968	0.709933	0.510378	BGIOSGA015218	XP_006645239.1 stearoyl-[acyl-carrier-protein] 9-desaturase 2, chloroplastic
TCONS_00020000	0.619969	0.656022	0.437881	BGIOSGA014797	XP_008647952.1ATOZI1 isoform X1
TCONS_00008088	0.620072	1.17032	0.703993	BGIOSGA032223	XP_015689512.1 probable glucuronosyltransferase Os02g0520750
TCONS_00017165	0.62019	0.0705411	1.55228	BGIOSGA019211	XP_006654066.1 nifU-like protein 4, mitochondrial
TCONS_00004251	0.620816	2.18542	2.14573	BGIOSGA027113	XP_015693534.1 bifunctional nuclease 1

TCONS_00024647	0.621126	0.464845	0.917729	BGIOSGA024740	XP_006657491.1 DNA-directed RNA polymerases II, IV and V subunit 11-like
TCONS_00022157	0.621133	1.3279	0.447005	BGIOSGA019369	XP_004985587.1protein PAM68, chloroplastic
TCONS_00004695	0.621528	1.04283	1.06739	BGIOSGA001568	XP_004970520.1histone H2B.11
TCONS_00026519	0.621542	-1.16823	0.182294	BGIOSGA028585	XP_006659366.1 peptidyl-prolyl cis-trans isomerase, chloroplastic
TCONS_00018360	0.621569	1.53488	0.915994	BGIOSGA033173	XP_015692708.1 pentatricopeptide repeat-containing protein At1g15510, chloroplastic
TCONS_00004233	0.621637	1.20925	-0.0254244	BGIOSGA019930	XP_006644533.2 ABC transporter B family member 4-like
TCONS_00037306	0.621724	-0.203151	0.300287	BGIOSGA036060	XP_006664077.1 probable 2,3-bisphosphoglycerate-independent phosphoglycerate mutase
TCONS_00002059	0.622022	-0.62334	0.785463	BGIOSGA034601	XP_004970372.1protein LURP-one-related 8
TCONS_00028075	0.622323	-1.13157	-0.959634	BGIOSGA032209	XP_002445566.1cinnamoyl-CoA reductase 1
TCONS_00032066	0.622394	-0.352531	-0.131936	BGIOSGA032072	XP_015697332.1 myosin-11 isoform X2
TCONS_00022226	0.622406	0.525037	0.0758741	BGIOSGA001926	XP_015689417.1 probable peroxygenase 5
TCONS_00002167	0.622484	0.573982	-2.02281	BGIOSGA017929	XP_006645007.1 aldo-keto reductase family 4 member C9-like
TCONS_00032449	0.622589	0.702928	1.03161	BGIOSGA013953	XP_002464470.1basic transcription factor 3
TCONS_00028198	0.622712	0.390427	0.57676	BGIOSGA030940	XP_015695708.1 dicarboxylate transporter 2.1, chloroplastic-like
TCONS_00007248	0.622937	0.23232	0.200434	BGIOSGA007238	XP_006646792.2 AUGMIN subunit 4
TCONS_00008303	0.623102	1.23195	1.48446	BGIOSGA006125	XP_006647454.2 protein GLE1
TCONS_00004129	0.623355	0.617356	1.26335	BGIOSGA001064	XP_006644484.1PREDICTED: uncharacterized protein At4g18257
TCONS_00019027	0.623495	0.33828	0.284262	BGIOSGA018332	XP_006655180.1PREDICTED: uncharacterized protein LOC102715210
TCONS_00027011	0.623726	-0.0969706	1.21219	BGIOSGA029059	XP_006659631.1PREDICTED: uncharacterized protein LOC102703605
TCONS_00007933	0.623861	0.616413	1.12077	BGIOSGA006554	XP_003573060.2probable diaminopimelate decarboxylase, chloroplastic
TCONS_00011756	0.62443	-1.68974	0.29554	BGIOSGA025281	XP_015689808.1 QWRF motif-containing protein 2
TCONS_00031970	0.624499	0.848645	0.369005	BGIOSGA016873	XP_006662256.1 probable glucuronosyltransferase Os10g0205300
TCONS_00014670	0.624552	-0.111354	0.169398	BGIOSGA012640	XP_015691324.1 indole-3-acetic acid-induced protein ARG7
TCONS_00028607	0.624738	0.172399	-0.632859	BGIOSGA030358	XP_006662124.2 ferredoxin-thioredoxin reductase catalytic chain, chloroplastic
TCONS_00026769	0.6249	1.08728	0.76004	BGIOSGA026419	XP_004973666.1bZIP transcription factor TRAB1
TCONS_00007831	0.62492	1.16607	0.583192	BGIOSGA004524	XP_006647188.1 protein PAT1 homolog 1-like
TCONS_00010951	0.625215	0.0238665	-0.408745	BGIOSGA031053	XP_006650581.1 cysteine synthase
TCONS_00002183	0.625491	0.712565	0.598865	BGIOSGA029234	XP_006645033.1 leucine-rich repeat extensin-like protein 3
TCONS_00016975	0.625795	0.00898561	0.529206	BGIOSGA025983	XP_010231854.1probable serine/threonine-protein kinase WNK2
TCONS_00029606	0.625941	0.356174	0.922031	BGIOSGA020802	XP_006660421.1PREDICTED: uncharacterized protein LOC102700645
TCONS_00035601	0.626037	0.228809	-0.123466	BGIOSGA006048	XP_006663828.1 UDP-galactose transporter 2-like
TCONS_00015147	0.626068	0.830981	-0.224668	BGIOSGA031049	XP_004960228.1uncharacterized protein LOC101760494
TCONS_00003030	0.62623	0.524053	0.379263	BGIOSGA031410	XP_006662599.1 autophagy-related protein 3
TCONS_00023194	0.62637	-1.62883	-1.81038	BGIOSGA026002	XP_006657880.1 glutamic acid-rich protein-like
TCONS_00009403	0.626573	1.21254	-0.451897	BGIOSGA022524	XP_006649479.2 alanine--glyoxylate aminotransferase 2 homolog 1, mitochondrial
TCONS_00004583	0.626712	0.995974	1.22798	BGIOSGA020095	XP_015699200.1 stomatal closure-related actin-binding protein 1-like isoform X1
TCONS_00006427	0.626907	-0.0642713	1.26364	BGIOSGA008591	XP_006648822.1 nicotinamidase 1
TCONS_00006191	0.62701	-0.63444	0.0384969	BGIOSGA008354	XP_006647338.2 probable sodium/metabolite cotransporter BASS4, chloroplastic
TCONS_00018540	0.627026	1.67334	1.21138	BGIOSGA018897	XP_006654947.1PREDICTED: uncharacterized protein LOC102699867
TCONS_00003313	0.627071	1.19471	0.860299	BGIOSGA012532	XP_006644056.1 mitochondrial import receptor subunit TOM40-1-like
TCONS_00034462	0.627289	-0.567421	-0.604516	BGIOSGA016215	XP_006662803.1 probable magnesium transporter NIPA4
TCONS_00023390	0.627331	-0.334957	-0.146301	BGIOSGA033522	XP_006657579.1 vacuolar protein sorting-associated protein 2 homolog 1-like
TCONS_00009189	0.627443	0.259478	0.463511	BGIOSGA037765	XP_006649272.2 F-box protein At4g00755-like
TCONS_00037275	0.628306	0.294805	-0.513734	BGIOSGA032551	XP_006664062.1 polyol transporter 5-like
TCONS_00021070	0.628544	1.3833	0.941035	BGIOSGA030286	XP_004965828.1peroxiredoxin-2E-1, chloroplastic
TCONS_00015842	0.628646	-0.968054	0.254664	BGIOSGA015142	XP_004975537.1DNA-binding protein S1FA2
TCONS_00011798	0.628968	-0.265526	-0.418312	BGIOSGA011207	XP_015690350.1PREDICTED: uncharacterized protein LOC107303832
TCONS_00018411	0.629182	0.236121	0.927779	BGIOSGA020463	XP_006654853.2 protein PLASTID TRANSCRIPTIONALLY ACTIVE 14
TCONS_00007014	0.629392	0.589755	0.50405	BGIOSGA028758	XP_006649126.1PREDICTED: uncharacterized protein LOC102706173
TCONS_00034162	0.629527	-0.95705	0.362289	BGIOSGA036771	XP_006664867.1PREDICTED: uncharacterized protein LOC102702156
TCONS_00009000	0.63007	0.685017	0.550701	BGIOSGA004605	XP_015689503.1 PAP-specific phosphatase HAL2-like
TCONS_00007557	0.630401	-0.0667624	-0.643678	BGIOSGA021469	XP_004951536.1uncharacterized protein LOC101765845
TCONS_00021381	0.630478	1.91662	0.408708	BGIOSGA020527	XP_006657350.1 endoplasmin homolog
TCONS_00024682	0.630518	-2.78118	-0.964528	BGIOSGA019111	NP_001334047.1uncharacterized LOC100275286
TCONS_00017885	0.630563	1.39903	0.387982	BGIOSGA011140	XP_006654466.1 U-box domain-containing protein 29-like
TCONS_00035581	0.6307	-0.0598997	0.353118	BGIOSGA019079	XP_006663080.2PREDICTED: uncharacterized protein LOC102711651, partial

TCONS_00010047	0.630723	-0.403359	1.59118	BGIOSGA014208	XP_012698589.1alpha-N-acetylglucosaminidase isoform X1
TCONS_00006373	0.631057	1.04717	0.156896	BGIOSGA004471	XP_006648805.1 elongation factor Tu, chloroplastic
TCONS_00022954	0.631391	-0.00299229	0.660218	BGIOSGA020493	XP_006657375.2PREDICTED: uncharacterized protein LOC102704258
TCONS_00018174	0.631546	0.842616	0.459941	BGIOSGA027374	XP_006654685.1 50S ribosomal protein L9
TCONS_00011068	0.631582	1.52681	2.096	BGIOSGA013663	XP_004981553.1inosine-5'-monophosphate dehydrogenase
TCONS_00032472	0.631783	0.43856	0.932174	BGIOSGA000910	XP_010234854.1uncharacterized protein LOC100846112
TCONS_00029805	0.632003	0.00680603	0.604875	BGIOSGA030014	XP_015696500.1 probable protein phosphatase 2C 67
TCONS_00000409	0.632027	-0.298713	-0.952954	BGIOSGA004165	XP_006643898.2 phospho-N-acetylmuramoyl-pentapeptide-transferase homolog
TCONS_00013412	0.632306	0.291524	1.25796	BGIOSGA024577	XP_006650790.1 transcription elongation factor TFIIS
TCONS_00017330	0.632324	-4.05397	-0.484202	BGIOSGA017468	XP_006655095.1 TPR repeat-containing thioredoxin TTL1-like
TCONS_00026841	0.632591	0.466877	1.43823	BGIOSGA014919	XP_004968247.1histone H2B.3
TCONS_00008613	0.632879	0.589493	-0.124412	BGIOSGA020778	XP_008646075.1uncharacterized protein LOC103627570
TCONS_00020090	0.632891	1.30452	-0.139621	BGIOSGA002221	XP_006656617.1 acid phosphatase 1
TCONS_00027799	0.63304	0.53009	1.3885	BGIOSGA014479	XP_024316228.1pentatricopeptide repeat-containing protein At5g66520
TCONS_00034511	0.633054	0.899627	0.683817	BGIOSGA032264	XP_006662818.1 alcohol dehydrogenase 1
TCONS_00028472	0.633144	-0.656621	-0.255861	BGIOSGA026484	XP_021306676.1photosystem I reaction center subunit II, chloroplastic-like
TCONS_00031640	0.633371	0.873357	1.27155	BGIOSGA036580	XP_006662075.1 calcineurin B-like protein 1
TCONS_00029538	0.633413	0.902473	0.817941	BGIOSGA031296	XP_015696519.1PREDICTED: uncharacterized protein LOC107304926
TCONS_00024386	0.633841	0.34229	0.710639	BGIOSGA019942	XP_015695229.1 transcription factor HBP-1b(c38)-like
TCONS_00029091	0.633905	1.15051	0.0215536	BGIOSGA029664	XP_006665048.1 cytochrome P450 71A1-like
TCONS_00011669	0.63391	-3.8667	-2.46409	BGIOSGA018039	XP_006651096.1 4,5:9,10-diseco-3-hydroxy-5,9,17-trioxoandrosta-1(10),2-diene-4-oate hydrolase-like
TCONS_00000618	0.634116	-3.86696	0.535402	BGIOSGA025002	XP_006644025.1 peroxidase 72-like
TCONS_00025350	0.634811	-0.254473	-0.27994	BGIOSGA004569	NP_001158996.1SC3 protein
TCONS_00002941	0.635091	0.591933	1.271	BGIOSGA002283	XP_006645555.2 acyl-protein thioesterase 2
TCONS_00007979	0.635232	-0.476044	-0.297441	BGIOSGA000948	XP_004969679.1F-box/kelch-repeat protein At1g23390
TCONS_00026806	0.635402	-0.168135	-0.145858	BGIOSGA024867	XP_002440130.1E3 ubiquitin-protein ligase ATL31
TCONS_00014686	0.635891	0.313014	-0.49701	BGIOSGA031777	XP_006652596.1 poly(U)-specific endoribonuclease-B
TCONS_00011447	0.635987	1.34014	1.81617	BGIOSGA011576	XP_021303123.1pentatricopeptide repeat-containing protein At3g53170 isoform X1
TCONS_00030288	0.636021	0.86232	0.376963	BGIOSGA022307	XP_015696398.1 protein lojap, chloroplastic
TCONS_00020196	0.636393	0.742803	1.68184	BGIOSGA022387	XP_006655831.1 mitochondrial outer membrane import complex protein METAXIN
TCONS_00030461	0.636696	2.23832	1.2839	BGIOSGA018109	XP_006660919.1 pentatricopeptide repeat-containing protein At1g20300, mitochondrial
TCONS_00008291	0.636756	1.05815	0.704786	BGIOSGA006139	XP_006647443.1PREDICTED: uncharacterized protein LOC102713796
TCONS_00029521	0.636961	0.851932	1.3663	BGIOSGA009394	XP_006661543.1 ruBisCO large subunit-binding protein subunit alpha-like
TCONS_00010421	0.636961	1.16864	1.0475	BGIOSGA034282	XP_004976818.1multiple organellar RNA editing factor 3, mitochondrial
TCONS_00033264	0.637285	0.855089	0.449188	BGIOSGA035087	XP_006663350.1 ER membrane protein complex subunit 10
TCONS_00026672	0.637492	0.45667	-0.2731	BGIOSGA006055	XP_006659445.1 temperature-induced lipocalin-1
TCONS_00004466	0.637606	1.5642	0.482777	BGIOSGA030854	XP_015692505.1 E3 ubiquitin-protein ligase RNF5-like isoform X3
TCONS_00005363	0.637619	-0.326545	-1.30584	BGIOSGA007474	XP_015688447.1 phosphopantetheine adenylyltransferase 2
TCONS_00020249	0.637737	-0.947308	-0.104094	BGIOSGA012652	XP_006656719.1 putative glutathione peroxidase 7, chloroplastic
TCONS_00029751	0.637799	0.756929	1.32191	BGIOSGA029567	XP_006660500.1 sodium/hydrogen exchanger 6-like
TCONS_00023645	0.638046	1.25444	0.75725	BGIOSGA025667	XP_015694629.1 serine/threonine-protein kinase/endoribonuclease IRE1b
TCONS_00022769	0.638234	0.0906711	0.440238	BGIOSGA007114	XP_015694230.1 putative serine/threonine-protein kinase
TCONS_00004892	0.638251	0.612493	-0.727248	BGIOSGA011725	XP_006645166.1 cyclin-dependent kinase B1-1
TCONS_00018769	0.638342	0.061622	1.18418	BGIOSGA019430	XP_021317282.1uncharacterized protein LOC8074381 isoform X2
TCONS_00026430	0.638395	-0.649663	0.303349	BGIOSGA025924	XP_006659340.1 mitochondrial pyruvate carrier 4-like
TCONS_00011139	0.638563	0.69254	1.12653	BGIOSGA017652	XP_006650739.1 protein RTE1-HOMOLOG
TCONS_00013511	0.638824	0.993191	0.252123	BGIOSGA009468	XP_006652010.2 prolycopene isomerase 1, chloroplastic
TCONS_00011858	0.639179	-0.299399	0.113597	BGIOSGA011150	XP_006649703.1 65-kDa microtubule-associated protein 7-like
TCONS_00025129	0.639229	0.888371	0.598124	BGIOSGA024274	XP_006658574.1 ATP synthase subunit delta', mitochondrial
TCONS_00033816	0.639479	-0.241958	-0.11293	BGIOSGA002669	XP_006663036.1 thioredoxin domain-containing protein PLP3A-like
TCONS_00011183	0.639534	0.236283	-0.874439	BGIOSGA024058	XP_003564066.1chaperonin-like RbcX protein 2, chloroplastic
TCONS_00021313	0.639704	0.051024	-0.200819	BGIOSGA032300	XP_004966130.1exocyst complex component EXO70A1
TCONS_00021444	0.639758	1.27803	0.736777	BGIOSGA017454	NP_001150057.1IQ calmodulin-binding motif family protein

TCONS_00000949	0.639766	1.0122	0.227673	BGIOSGA032183	XP_006644177.2 probable E3 ubiquitin-protein ligase LUL2
TCONS_00029179	0.639923	1.29779	1.65193	BGIOSGA004260	XP_015696383.1 WAS/WASL-interacting protein family member 3-like
TCONS_00008556	0.639965	3.28212	0.392823	BGIOSGA002547	XP_015689539.1 dehydrin COR410
TCONS_00029903	0.640145	1.80261	1.3392	#N/A	#N/A
TCONS_00025715	0.640481	0.635805	0.528578	BGIOSGA025331	XP_004958712.1protein ENHANCED DISEASE RESISTANCE 4
TCONS_00016310	0.640482	-0.013361	-0.0447462	BGIOSGA005957	XP_006652568.1 remorin-like
TCONS_00010458	0.64067	-2.61798	-0.338163	BGIOSGA026003	XP_006664981.1 chlorophyll a-b binding protein, chloroplastic
TCONS_00019075	0.640877	2.38679	0.696869	BGIOSGA018283	XP_015693176.1 chaperone protein DnaJ-like
TCONS_00024592	0.641222	0.532822	1.04373	#N/A	#N/A
TCONS_00002190	0.641295	1.11006	1.94131	BGIOSGA004825	XP_015690412.1 cell division control protein 6 homolog
TCONS_00026999	0.641322	0.22391	0.756199	BGIOSGA029050	NP_001168939.1uncharacterized LOC100382753
TCONS_00003694	0.64159	0.375489	1.10642	BGIOSGA022886	XP_008648688.1uncharacterized LOC100193223 isoform X1
TCONS_00015678	0.641616	1.31875	1.2327	BGIOSGA015301	XP_006653289.2 U11/U12 small nuclear ribonucleoprotein 48 kDa protein
TCONS_00011663	0.64167	0.179718	-0.66579	BGIOSGA011346	XP_006651093.1PREDICTED: uncharacterized protein LOC102702547
TCONS_00017038	0.642196	-1.24367	-1.2398	BGIOSGA017726	XP_006654944.1 probable glucuronosyltransferase Os05g0123100
TCONS_00031511	0.642236	2.2412	-0.188196	BGIOSGA020018	XP_002467615.1probable glutathione S-transferase GSTU6
TCONS_00027040	0.642439	0.845591	0.493024	BGIOSGA029089	XP_002445769.1peptidyl-prolyl cis-trans isomerase FKBP16-3, chloroplastic
TCONS_00029124	0.642769	-0.54003	0.150074	BGIOSGA002010	XP_015694263.1PREDICTED: uncharacterized protein LOC107304498
TCONS_00000673	0.642796	1.28874	0.964695	BGIOSGA003261	XP_006644063.2 COP9 signalosome complex subunit 2
TCONS_00005281	0.642956	0.613097	-0.43366	BGIOSGA037813	XP_015688795.1 peptidyl-prolyl cis-trans isomerase FKBP18, chloroplastic
TCONS_00032160	0.643073	0.840307	1.94437	BGIOSGA031980	XP_006661750.1 delta(24)-sterol reductase
TCONS_00037565	0.64325	-1.23083	-2.73054	BGIOSGA006987	XP_002442578.1phytoene synthase 2, chloroplastic
TCONS_00003194	0.643301	0.502888	0.32371	BGIOSGA012487	XP_003565568.1transcription factor bHLH150
TCONS_00022039	0.643344	-0.0886788	0.331286	BGIOSGA014531	XP_003563969.1aluminum-activated malate transporter 9
TCONS_00008267	0.643512	-1.69681	1.20411	BGIOSGA016565	XP_003575244.1heavy metal-associated isoprenylated plant protein 47
TCONS_00023547	0.643526	-0.0299502	0.454959	BGIOSGA025579	XP_006658498.1 alpha-glucosidase 2-like
TCONS_00034738	0.643599	-0.0321532	0.52345	BGIOSGA002464	XP_004979211.1protein CASPARIAN STRIP INTEGRITY FACTOR 2
TCONS_00006831	0.643843	0.289838	0.766382	BGIOSGA009005	XP_006649024.1 ankyrin repeat-containing protein At2g01680
TCONS_00008637	0.644419	1.07652	0.574309	BGIOSGA014425	XP_006647738.1PREDICTED: uncharacterized protein LOC102704308
TCONS_00009940	0.645166	1.16428	1.34234	BGIOSGA012491	XP_006649978.1 probable NADH dehydrogenase [ubiquinone] 1 alpha subcomplex subunit 5, mitochondrial
TCONS_00028405	0.645833	0.728098	0.46275	BGIOSGA037845	XP_004974072.1methionine aminopeptidase 2B
TCONS_00007926	0.646176	0.845707	0.894621	BGIOSGA012223	XP_006647231.1 probable 4-hydroxy-tetrahydrodipicolinate reductase 1, chloroplastic
TCONS_00003505	0.64623	0.546829	0.99872	BGIOSGA005347	XP_006644155.1 serine/threonine-protein phosphatase PP1-like
TCONS_00032653	0.646313	1.66765	0.821506	BGIOSGA031456	XP_006662031.1 UDP-N-acetylmuramoyl-L-alanyl-D-glutamate--2,6-diaminopimelate ligase
TCONS_00028968	0.646375	-1.14109	1.07375	BGIOSGA021880	XP_006660609.2 probable cinnamyl alcohol dehydrogenase 8C
TCONS_00022918	0.64652	1.80139	2.16369	BGIOSGA020523	XP_006656497.1 RNA pseudouridine synthase 2, chloroplastic
TCONS_00017065	0.646577	0.863017	1.39166	BGIOSGA019115	XP_006654954.1 acetylornithine aminotransferase, mitochondrial
TCONS_00008635	0.646704	2.72098	1.80066	BGIOSGA014376	XP_006648958.1PREDICTED: uncharacterized protein LOC102701336
TCONS_00012559	0.646792	1.00006	1.54392	BGIOSGA030640	XP_004982548.1transcription factor SOX-4
TCONS_00029346	0.64725	-1.44079	-0.477376	BGIOSGA005257	XP_004957365.1formin-like protein 15
TCONS_00002505	0.647431	-0.130999	1.08754	BGIOSGA018592	XP_004951877.1endoglucanase 5
TCONS_00016213	0.647493	-0.130753	-0.733231	BGIOSGA006036	XP_015692137.1PREDICTED: uncharacterized protein LOC102722484 isoform X3
TCONS_00001778	0.647545	0.678105	-0.0701781	BGIOSGA004416	XP_006644672.1 polynucleotide 3'-phosphatase ZDP
TCONS_00028580	0.64767	-0.377044	0.604995	BGIOSGA025150	XP_006661040.2 N-acetyl-D-glucosamine kinase-like
TCONS_00013246	0.647762	0.564374	0.0902209	BGIOSGA020068	XP_003558723.1uncharacterized protein LOC100826176
TCONS_00009202	0.64797	1.09261	1.26319	BGIOSGA011730	XP_006649284.2 hydroxymethylglutaryl-CoA synthase-like
TCONS_00035015	0.648458	0.443483	0.519866	BGIOSGA010066	XP_002453153.2probable nucleolar protein 5-2
TCONS_00026370	0.648836	0.645569	0.658549	BGIOSGA028440	XP_006659295.1 tyrosine--tRNA ligase 1, cytoplasmic-like
TCONS_00016119	0.649344	0.362864	-0.310466	BGIOSGA033452	XP_015691503.1 peptide methionine sulfoxide reductase A2-1
TCONS_00024312	0.649582	0.972748	1.01594	BGIOSGA026355	XP_006658090.1 probable GTP-binding protein OBGC1, chloroplastic
TCONS_00000360	0.649688	1.87335	1.30786	BGIOSGA002937	XP_006643869.1 protein ALWAYS EARLY 3-like isoform X1
TCONS_00010262	0.649841	0.454136	-0.582327	BGIOSGA021732	XP_006651481.1 3-oxoacyl-[acyl-carrier-protein] synthase II, chloroplastic-like
TCONS_00034191	0.650023	1.34061	-0.464095	BGIOSGA034031	XP_002441776.1uncharacterized protein LOC8074828

TCONS_00022372	0.650198	-2.5155	-0.368799	BGIOSGA021180	XP_015693604.1PREDICTED: uncharacterized protein LOC102704713
TCONS_00004585	0.650469	-0.146339	0.607863	BGIOSGA000628	XP_004970298.1F-box protein At2g41170 isoform X2
TCONS_00005491	0.65073	0.610454	0.689539	BGIOSGA004048	XP_006646920.1 putative serine/threonine-protein kinase isoform X1
TCONS_00002272	0.651011	1.14644	1.26142	BGIOSGA018018	XP_006645104.1 splicing regulator RBM11-like isoform X1
TCONS_00020362	0.65103	0.756376	0.693189	BGIOSGA012011	XP_003564084.1GLABRA2 expression modulator
TCONS_00020920	0.651647	0.687848	0.932034	BGIOSGA023125	XP_006656183.1 (S)-coclaurine N-methyltransferase-like
TCONS_00008980	0.65168	0.856175	1.33489	BGIOSGA037481	XP_022681499.1F-box/LRR-repeat protein 14
TCONS_00006803	0.651869	-0.27571	-0.00404317	BGIOSGA021102	XP_006649012.1 E3 ubiquitin-protein ligase ATL41-like
TCONS_00013360	0.651887	1.24674	1.96072	BGIOSGA022177	XP_006651920.1 DEAD-box ATP-dependent RNA helicase 27
TCONS_00023977	0.652149	0.334619	-0.208691	BGIOSGA026008	XP_006658734.1 5-formyltetrahydrofolate cyclo-ligase, mitochondrial
TCONS_00011084	0.652215	1.02969	1.17486	BGIOSGA025164	XP_006650694.1 peroxisomal membrane protein 13-like
TCONS_00000135	0.652233	0.300211	0.7951	BGIOSGA002709	XP_006643692.1 probable signal peptidase complex subunit 3
TCONS_00016534	0.652609	0.839492	0.909905	BGIOSGA014086	XP_015692191.1 HBS1-like protein isoform X5
TCONS_00004801	0.653008	0.441249	0.748308	BGIOSGA000416	XP_020394452.1uncharacterized protein LOC100274722 isoform X2
TCONS_00000132	0.653054	1.02153	0.880479	BGIOSGA002706	XP_015690200.1PREDICTED: uncharacterized protein LOC102705209
TCONS_00029564	0.653122	1.56107	0.838379	BGIOSGA030257	XP_004974953.1serine/arginine repetitive matrix protein 1 isoform X3
TCONS_00000513	0.653137	-0.847129	-1.4825	BGIOSGA003087	XP_015688095.1PREDICTED: uncharacterized protein LOC102704381
TCONS_00029631	0.653709	0.679701	1.03335	BGIOSGA030190	XP_006660432.1 tropinone reductase-like 3
TCONS_00036172	0.653755	1.54857	1.00972	BGIOSGA009496	XP_015698396.1 acyl-CoA-binding domain-containing protein 4-like
TCONS_00030388	0.653892	-2.91016	-2.0929	BGIOSGA020673	XP_003578469.1BAG family molecular chaperone regulator 1
TCONS_00009636	0.653933	1.38725	1.26168	BGIOSGA004399	XP_006649684.2 GTP-binding protein At2g22870-like
TCONS_00031365	0.654074	-0.176384	0.525616	BGIOSGA033179	XP_004982768.1reactive oxygen species modulator 1
TCONS_00000642	0.65414	1.01152	0.414489	BGIOSGA003223	XP_006645750.1 rhomboid-like protein 20
TCONS_00019736	0.654174	1.22353	0.979059	BGIOSGA023551	XP_006655551.1 putative clathrin assembly protein At2g01600
TCONS_00002531	0.654587	-3.91104	-0.509662	BGIOSGA018146	XP_015692392.1 probable ADP-ribosylation factor GTPase-activating protein AGD11
TCONS_00005440	0.65481	0.359345	1.09753	BGIOSGA037224	XP_006648308.1 apoptosis-inducing factor 2-like
TCONS_00030178	0.655294	0.373461	-0.672003	BGIOSGA029624	XP_006660713.1 beta carbonic anhydrase 5, chloroplastic-like isoform X2
TCONS_00009411	0.655524	1.46861	1.22498	BGIOSGA013646	XP_006649485.1 tRNA-specific 2-thiouridylase MnmA
TCONS_00030924	0.655898	0.94847	0.177931	BGIOSGA027383	XP_006662277.1PREDICTED: uncharacterized protein LOC102706505
TCONS_00008204	0.656216	-0.107143	0.717179	BGIOSGA015009	XP_015688639.1 probable signal peptidase complex subunit 2
TCONS_00017420	0.656232	-0.39781	0.817564	BGIOSGA014849	XP_006663036.1 thioredoxin domain-containing protein PLP3A-like
TCONS_00022135	0.656795	1.42009	1.56525	BGIOSGA008941	XP_006648996.2 PHD finger protein At3g20280-like
TCONS_00032855	0.657141	0.396676	0.524315	BGIOSGA036731	XP_006663736.1 stress response protein nst1-like
TCONS_00017821	0.657571	1.14198	2.25327	BGIOSGA019881	XP_006654411.1 RPA-interacting protein A
TCONS_00005783	0.657718	0.151459	0.012338	BGIOSGA007896	XP_006647122.1 probable inactive dual specificity protein phosphatase-like At4g18593
TCONS_00014665	0.657766	0.679491	-0.063557	BGIOSGA016829	XP_006652577.1 peroxisomal membrane protein 11-4
TCONS_00028519	0.657873	-1.78821	-0.387967	BGIOSGA017934	XP_015698949.1 golgin-84
TCONS_00021383	0.658096	0.212875	-0.927218	BGIOSGA023615	XP_006656498.1PREDICTED: uncharacterized protein LOC102722776
TCONS_00028316	0.658205	0.313786	0.448236	BGIOSGA026134	XP_006659606.1 oligouridylate-binding protein 1B-like
TCONS_00011822	0.658364	-0.0123423	-1.29252	#N/A	#N/A
TCONS_00022961	0.658419	0.933085	0.737202	BGIOSGA024996	XP_015695422.1 methylsterol monooxygenase 2-1-like
TCONS_00005169	0.658744	1.53407	0.393104	BGIOSGA000034	XP_006645336.1PREDICTED: uncharacterized protein LOC102710745
TCONS_00021251	0.658872	-3.7498	-0.925872	BGIOSGA019132	XP_006656386.1 peroxidase 3-like
TCONS_00022585	0.659718	0.243071	0.617382	BGIOSGA027541	XP_015693736.1 cinnamoyl-CoA reductase 1-like
TCONS_00033036	0.660219	-2.71488	#NA	BGIOSGA034865	XP_006663240.2 horcolin-like
TCONS_00033314	0.661131	2.24205	1.95284	BGIOSGA029336	XP_006663367.1 gem-associated protein 2-like
TCONS_00011490	0.661214	0.558581	0.909721	BGIOSGA021782	XP_006649317.1 callose synthase 3-like isoform X1
TCONS_00006739	0.661286	4.63262	1.948	BGIOSGA007446	XP_006647786.1 17.8 kDa heat shock protein-like
TCONS_00018741	0.661306	-0.219894	1.23836	BGIOSGA005924	XP_006654120.1 probable WRKY transcription factor 51
TCONS_00015191	0.661327	0.192527	-0.440794	BGIOSGA014518	XP_006653042.2 phosphomannomutase
TCONS_00017919	0.66147	0.700917	-0.506725	BGIOSGA008965	XP_010231313.2O-fucosyltransferase 19
TCONS_00011461	0.6615	0.125682	0.124179	BGIOSGA017683	XP_015690329.1 ALA-interacting subunit 5-like
TCONS_00017825	0.661543	0.914716	0.159905	BGIOSGA024491	XP_006654244.1 guanosine nucleotide diphosphate dissociation inhibitor 2-like
TCONS_00025469	0.661719	1.06498	1.65499	BGIOSGA023961	XP_006658770.1 pentatricopeptide repeat-containing protein At4g21705, mitochondrial

TCONS_00016844	0.661802	1.16193	0.580156	BGIOSGA033685	XP_006653863.1 protein DJ-1 homolog D
TCONS_00004292	0.661926	1.00359	0.553815	BGIOSGA031498	XP_006644618.1PREDICTED: uncharacterized protein LOC102712307
TCONS_00012102	0.661954	2.13508	1.99145	BGIOSGA001357	XP_015690372.1 programmed cell death protein 2
TCONS_00011330	0.662086	0.300881	0.915266	BGIOSGA013940	XP_010927687.1 actin-related protein 2/3 complex subunit 4 isoform X2
TCONS_00004950	0.662386	-1.13243	-0.187251	BGIOSGA020241	NP_001143833.1uncharacterized LOC100276614 precursor
TCONS_00019259	0.662572	-0.113133	-0.490205	BGIOSGA022538	XP_015692811.1PREDICTED: uncharacterized protein LOC102721274
TCONS_00018139	0.662779	-0.195826	0.465373	BGIOSGA009865	NP_001338701.1uncharacterized LOC100856921 precursor
TCONS_00022173	0.662819	0.0925672	0.758439	BGIOSGA022713	XP_024318165.1disease resistance protein RPM1
TCONS_00032034	0.662906	0.528158	-1.18083	BGIOSGA010400	XP_004983271.1protein DETOXIFICATION 16
TCONS_00006609	0.663125	-0.493178	0.210944	BGIOSGA023963	XP_006647657.1 purine-uracil permease NCS1
TCONS_00000963	0.663201	0.34462	-0.107096	BGIOSGA003566	XP_003565905.1F-box protein At5g49610
TCONS_00018639	0.663256	0.265695	1.60449	BGIOSGA000104	XP_006654997.2 proline synthase co-transcribed bacterial homolog protein
TCONS_00002972	0.663321	0.659519	1.20385	BGIOSGA023854	XP_006643835.1 TSL-kinase interacting protein 1
TCONS_00028778	0.663362	-0.535673	-1.00771	BGIOSGA007228	XP_006660541.1 bifunctional protein FolD 1, mitochondrial-like
TCONS_00012833	0.663477	0.819926	0.331989	BGIOSGA010137	XP_006650357.1 rhomboid-like protein 15
TCONS_00012511	0.663727	0.663634	-0.335153	BGIOSGA026082	XP_006665039.2 3-oxo-Delta(4,5)-steroid 5-beta-reductase
TCONS_00010486	0.663824	1.01398	0.515393	BGIOSGA030618	XP_006650281.1 presenilin-like protein At2g29900
TCONS_00010722	0.663984	1.33727	0.721788	BGIOSGA013346	XP_006651698.1 ferredoxin-2
TCONS_00005963	0.664084	0.795493	0.423544	BGIOSGA008099	XP_004952262.1uncharacterized protein LOC101782043
TCONS_00033460	0.664113	-0.310923	0.440126	BGIOSGA035093	XP_015697758.1 TBC1 domain family member 13-like
TCONS_00024278	0.664139	0.965344	1.1236	BGIOSGA020992	XP_006658924.1 ubiquitin carboxyl-terminal hydrolase 24
TCONS_00027349	0.664522	0.128596	0.33717	BGIOSGA027653	XP_006659115.2PREDICTED: uncharacterized protein LOC102711087
TCONS_00007451	0.664614	0.570944	0.577797	BGIOSGA035648	XP_006646960.1 probable small nuclear ribonucleoprotein F
TCONS_00015238	0.664964	-0.372226	0.552792	BGIOSGA015741	XP_006652065.1PREDICTED: uncharacterized protein LOC102708426
TCONS_00026411	0.665062	1.32382	1.30571	BGIOSGA000017	XP_015694050.1 putative acyl-activating enzyme 19 isoform X1
TCONS_00012956	0.665176	2.14183	1.10791	BGIOSGA010033	XP_015690020.1PREDICTED: uncharacterized protein C6C3.02c
TCONS_00005671	0.665209	0.518414	0.226248	BGIOSGA023376	XP_003571680.1protein CLP1 homolog
TCONS_00035678	0.6653	1.18598	0.859824	BGIOSGA010355	XP_017970016.1 DNA-directed RNA polymerases I, II, and III subunit RPABC5 isoform X1
TCONS_00004456	0.665449	-0.712281	-1.20223	BGIOSGA000765	XP_006644755.1 chorismate mutase 3, chloroplastic-like
TCONS_00036375	0.665497	#NA	1.14884	BGIOSGA019301	XP_006664715.1 auxin-responsive protein IAA31-like
TCONS_00019116	0.66578	0.538359	0.815802	BGIOSGA004105	XP_006654280.1 transcription initiation factor TFIID subunit 7-like
TCONS_00028906	0.665853	-0.121018	-0.475936	BGIOSGA036200	XP_004956706.1uncharacterized protein LOC101767387
TCONS_00018377	0.665872	1.4483	-2.31154	BGIOSGA020429	XP_006654839.1 anaphase-promoting complex subunit 10 isoform X1
TCONS_00016577	0.665977	0.962783	0.181411	BGIOSGA028231	XP_015691567.1PREDICTED: uncharacterized protein LOC102716404, partial
TCONS_00026249	0.666356	1.58523	0.759574	BGIOSGA028333	XP_004973121.1uncharacterized protein LOC101762210
TCONS_00010944	0.666467	0.252714	0.797274	BGIOSGA013547	XP_006650574.1 pentatricopeptide repeat-containing protein At3g22670, mitochondrial-like
TCONS_00025880	0.667453	-0.00223817	0.391521	BGIOSGA027950	XP_006659097.1 zinc finger CCCH domain-containing protein 55-like
TCONS_00034601	0.667558	0.344102	0.571979	BGIOSGA015015	XP_006662852.1 PHD finger protein ALFIN-LIKE 8
TCONS_00001212	0.667664	1.73557	1.38439	BGIOSGA003836	XP_003569261.1protein ANTAGONIST OF LIKE HETEROCHROMATIN PROTEIN 1
TCONS_00017257	0.667923	0.413311	1.0618	BGIOSGA010196	XP_003568784.1auxin-responsive protein IAA16 isoform X1
TCONS_00030406	0.668048	-0.396813	-0.23271	BGIOSGA021278	XP_006660873.2 probable 6-phosphogluconolactonase 4, chloroplastic
TCONS_00009553	0.668231	0.275419	1.14195	BGIOSGA022721	XP_010228985.2plant intracellular Ras-group-related LRR protein 7
TCONS_00040304	0.66829	-0.724656	0.267335	BGIOSGA038011	XP_015691560.1 protein NRT1/ PTR FAMILY 8.2-like
TCONS_00016636	0.668766	-1.3595	0.956314	BGIOSGA014327	XP_003580576.1RPM1-interacting protein 4
TCONS_00017129	0.66897	-1.99454	-1.07986	BGIOSGA019176	XP_003572753.1senescence-associated protein OSA15, chloroplastic isoform X2
TCONS_00032671	0.669042	1.13521	0.576676	BGIOSGA018909	XP_015697072.1 3-hydroxyisobutyryl-CoA hydrolase-like protein 2, mitochondrial
TCONS_00036191	0.669101	0.349063	1.72507	BGIOSGA037545	XP_002442297.1nucleoside diphosphate kinase 2, chloroplastic
TCONS_00001728	0.669102	1.18121	0.680339	BGIOSGA014237	XP_015693954.1 probable acyl-[acyl-carrier-protein]--UDP-N-acetylglucosamine O-acyltransferase, mitochondrial isoform X1
TCONS_00021953	0.669102	1.44424	1.96574	BGIOSGA030119	NP_001152287.1TMV response-related protein
TCONS_00012600	0.669498	-0.181507	-0.27569	BGIOSGA010377	XP_006650269.1PREDICTED: uncharacterized protein LOC102719001 isoform X1
TCONS_00006617	0.669498	0.0462885	-0.885741	BGIOSGA027819	XP_006648924.1 HMG1/2-like protein

TCONS_00015580	0.669642	0.766512	-0.342353	BGIOSGA028343	XP_004975238.1beta-glucuronosyltransferase GlcAT14A
TCONS_00026198	0.669661	1.52154	0.263905	BGIOSGA028270	XP_006659238.2 4'-phosphopantetheinyl transferase HetI-like
TCONS_00017075	0.66979	1.79701	0.678621	BGIOSGA019127	XP_006654009.1 DNA (cytosine-5)-methyltransferase DRM1-like
TCONS_00024033	0.669997	1.26742	1.47189	BGIOSGA026072	XP_006658766.1 probable mitochondrial saccharopine dehydrogenase-like oxidoreductase At5g39410
TCONS_00002399	0.670706	1.1496	1.25039	BGIOSGA005035	XP_006646601.1 pentatricopeptide repeat-containing protein At1g05600-like
TCONS_00018485	0.670728	0.545278	0.791758	BGIOSGA000017	XP_015692897.1 probable kinetochore protein SPC25
TCONS_00032981	0.671158	-0.520013	0.0317316	BGIOSGA024436	XP_003578894.1rho GTPase-activating protein 5
TCONS_00006727	0.671246	0.375879	0.488999	BGIOSGA008898	XP_006647777.1 histone-lysine N-methyltransferase SUVR5 isoform X1
TCONS_00022623	0.671393	0.330313	0.674623	BGIOSGA030830	XP_004965791.1transcription factor MYB44
TCONS_00001452	0.671782	-0.407143	-0.00441592	BGIOSGA017500	XP_021313274.1probable isoprenylcysteine alpha-carbonyl methylesterase ICMEL2 isoform X2
TCONS_00008099	0.672061	-1.85965	-0.0388271	#N/A	#N/A
TCONS_00017628	0.67215	0.270465	-0.742295	BGIOSGA019690	XP_004962336.1uncharacterized protein LOC101777552
TCONS_00018512	0.672294	1.14442	-0.271752	#N/A	#N/A
TCONS_00033033	0.672438	2.35756	-3.32797	BGIOSGA037048	XP_004977309.1uncharacterized protein LOC101776555
TCONS_00001479	0.672477	-0.560022	1.19549	BGIOSGA004119	XP_022682886.1putative pentatricopeptide repeat-containing protein At5g52630
TCONS_00018543	0.672663	1.1278	-1.0066	BGIOSGA014464	XP_002440516.1probable 2-oxoglutarate-dependent dioxygenase At3g111800 isoform X1
TCONS_00001521	0.672698	-0.184149	0.00414672	BGIOSGA017542	XP_004961181.150S ribosomal protein L12, chloroplastic
TCONS_00021397	0.67297	0.0284237	0.877158	BGIOSGA022683	XP_021304905.1protein FREE1 isoform X2
TCONS_00006870	0.673002	0.339781	0.518625	BGIOSGA009048	XP_010236118.2uncharacterized protein LOC100822345 isoform X1
TCONS_00019667	0.673186	0.77613	0.395494	BGIOSGA004405	XP_006654684.1 50S ribosomal protein L20-like
TCONS_00034066	0.673502	-2.05317	-1.15004	BGIOSGA037610	XP_006663116.1 ATP-citrate synthase alpha chain protein 2
TCONS_00021885	0.673736	-0.804016	-1.55124	BGIOSGA030618	XP_002438085.1proline-rich receptor-like protein kinase PERK13
TCONS_00001794	0.67378	-0.924233	0.36914	BGIOSGA028042	XP_010232287.1uncharacterized protein LOC100822437
TCONS_00012431	0.673803	1.96882	1.22323	BGIOSGA008904	XP_015690190.1 serine/threonine-protein kinase At5g01020-like
TCONS_00003800	0.673886	0.649982	0.770152	BGIOSGA004299	XP_006644264.1 vesicle transport v-SNARE 13-like
TCONS_00005812	0.673912	1.45807	1.31223	BGIOSGA033972	XP_006648526.1 chloroplast processing peptidase
TCONS_00034423	0.67407	-1.04418	0.255268	BGIOSGA015169	XP_008673747.1putrescine hydroxycinnamoyltransferase 1
TCONS_00010108	0.674548	0.43227	0.359906	BGIOSGA019850	XP_006650103.1 peptide methionine sulfoxide reductase B5
TCONS_00005916	0.674613	1.97207	0.971826	BGIOSGA033864	XP_006664945.1 apoptosis inhibitor 5
TCONS_00020592	0.67473	-0.843719	-0.0258016	BGIOSGA022777	XP_006656079.1 E3 ubiquitin-protein ligase SINA-like 7
TCONS_00000474	0.674789	-0.100586	-2.01186	BGIOSGA021834	XP_002457087.2cytochrome P450 71A1
TCONS_00009885	0.675382	1.96684	0.539214	BGIOSGA023673	XP_015689862.1 COBRA-like protein 7
TCONS_00032485	0.675413	-1.45053	-0.472953	BGIOSGA031634	XP_006662467.1 protein TIC110, chloroplastic
TCONS_00025138	0.675415	2.02806	1.41357	BGIOSGA024268	XP_006657729.1 large proline-rich protein BAG6 isoform X1
TCONS_00018691	0.675581	0.433869	-0.793089	BGIOSGA018738	XP_012699727.1 5-amino-6-(5-phospho-D-ribitylamino)uracil phosphatase, chloroplastic
TCONS_00011073	0.675588	1.16247	0.57153	BGIOSGA013673	XP_006650683.1 actin-related protein 7
TCONS_00003693	0.67566	-0.05421	0.746021	BGIOSGA001504	XP_006645957.1 probable RNA-dependent RNA polymerase SHL2
TCONS_00005809	0.675662	0.640814	0.143104	BGIOSGA022987	XP_006647139.1 reticulon-like protein B2
TCONS_00019423	0.675952	0.120968	1.0628	BGIOSGA017931	XP_006655372.2 uracil phosphoribosyltransferase
TCONS_00018737	0.675985	1.1286	0.616741	BGIOSGA018680	XP_003568801.1protein SLOW GREEN 1, chloroplastic
TCONS_00006368	0.676034	-0.201923	1.20988	BGIOSGA013285	XP_006650783.1 ribosomal RNA-processing protein 8
TCONS_00000831	0.676779	0.992737	0.180543	BGIOSGA019225	XP_015689057.1 Golgi apparatus membrane protein-like protein ECHIDNA
TCONS_00021548	0.677124	1.02042	0.197261	BGIOSGA022003	XP_002436414.1magnesium protoporphyrin IX methyltransferase, chloroplastic
TCONS_00028516	0.677272	0.558067	-0.168492	#N/A	#N/A
TCONS_00022404	0.677323	1.26345	1.00803	BGIOSGA031780	NP_001132839.1Ras protein RIC2
TCONS_00016003	0.677348	-1.01374	0.085187	BGIOSGA038471	XP_002447953.1short-chain dehydrogenase cctT
TCONS_00004668	0.677509	1.02673	1.32653	BGIOSGA020008	XP_015699279.1 selT-like protein
TCONS_00011995	0.677802	-0.16194	0.00386484	BGIOSGA000182	XP_006650184.1PREDICTED: uncharacterized protein LOC102718528
TCONS_00030116	0.677938	-0.191084	-0.460463	BGIOSGA030468	XP_006661275.1 transmembrane protein 87B-like
TCONS_00013384	0.67808	0.793052	1.61373	BGIOSGA030777	XP_006650766.2 probable cellulose synthase A catalytic subunit 2 [UDP-forming]
TCONS_00017867	0.678191	0.54462	0.437464	BGIOSGA019919	XP_006654446.1 transmembrane protein 18-like
TCONS_00001984	0.678577	0.756886	0.226007	BGIOSGA017791	XP_006644845.1 metacaspase-4-like isoform X1
TCONS_00004722	0.678633	0.904303	0.728544	BGIOSGA019972	XP_006645008.1 PRA1 family protein A1-like
TCONS_00011147	0.678646	0.920481	0.97071	BGIOSGA013747	XP_006651917.2 vacuolar protein sorting-associated protein 35B

TCONS_00027059	0.678658	0.298202	0.388105	BGIOSGA011729	XP_006659653.1 hydroxymethylglutaryl-CoA synthase-like
TCONS_00000619	0.67873	0.966617	0.759965	BGIOSGA003202	XP_004967773.1mediator of RNA polymerase II transcription subunit 27
TCONS_00006320	0.679517	-0.1586	0.240039	BGIOSGA022582	XP_006648780.1 beta-1,3-galactosyltransferase 15-like
TCONS_00021379	0.679642	-2.34671	-0.121168	BGIOSGA011126	XP_004966466.1chaperone protein dnaJ 11, chloroplastic
TCONS_00027025	0.679941	1.17506	-0.426066	BGIOSGA029075	XP_006659641.1PREDICTED: uncharacterized protein LOC102706312
TCONS_00021566	0.680101	-0.622328	0.724252	BGIOSGA029874	XP_015693768.1 enolase 1
TCONS_00025048	0.68013	-0.378145	0.467996	BGIOSGA024359	XP_002452586.2uncharacterized protein LOC8075741
TCONS_00021356	0.680234	0.482487	1.08378	BGIOSGA023584	XP_003563539.1uncharacterized protein LOC100828468
TCONS_00024427	0.680285	0.780067	0.482289	BGIOSGA031499	XP_006657387.2 uncharacterized GPI-anchored protein At1g61900
TCONS_00027943	0.680308	1.22296	1.21291	BGIOSGA006218	XP_006660092.2 thioredoxin-like protein CITRX, chloroplastic
TCONS_00028672	0.680847	-0.617356	-1.4246	BGIOSGA017908	XP_008240942.1PREDICTED: uncharacterized protein LOC103339425
TCONS_00014515	0.681075	-0.403629	-0.625513	BGIOSGA009689	NP_001140927.1expp1 protein precursor
TCONS_00007474	0.681396	1.09588	2.10128	BGIOSGA007010	XP_015689535.1 BRCA1-associated protein
TCONS_00037952	0.681411	1.06162	0.0142561	BGIOSGA008334	XP_006661977.1 glutathione S-transferase U17-like
TCONS_00024132	0.681604	2.17079	0.488255	BGIOSGA010614	XP_015695167.1 serine/arginine-rich splicing factor SC35-like isoform X1
TCONS_00009764	0.682173	0.870388	1.10099	BGIOSGA007740	XP_015690589.1PREDICTED: uncharacterized protein LOC102709820
TCONS_00029303	0.682195	0.554218	-0.0708098	BGIOSGA029040	XP_004957283.1proteasome subunit beta type-1
TCONS_00033840	0.682273	1.74409	0.334009	#N/A	#N/A
TCONS_00015848	0.682807	0.610611	0.878878	BGIOSGA017458	XP_006652252.1PREDICTED: uncharacterized protein LOC102722388
TCONS_00009864	0.683083	1.38503	0.734373	BGIOSGA023669	XP_006649930.1 eukaryotic translation initiation factor 2 subunit alpha-like
TCONS_00021968	0.683085	-0.23765	0.36667	BGIOSGA025507	XP_006655963.1PREDICTED: uncharacterized protein LOC102707968
TCONS_00015886	0.683489	-1.36849	-0.922995	BGIOSGA015104	XP_003579736.2RNA-binding motif protein, X-linked-like-3
TCONS_00019253	0.684048	0.415632	0.487228	BGIOSGA018096	XP_003568498.1cytochrome b-c1 complex subunit 9
TCONS_00026013	0.684331	-0.0923453	0.0192535	BGIOSGA028086	XP_006659178.1 sm-like protein LSM7
TCONS_00010937	0.68438	-0.86366	-2.44362	BGIOSGA013545	XP_006650572.1PREDICTED: uncharacterized protein LOC102714273
TCONS_00017765	0.684457	0.0524082	0.329609	BGIOSGA028250	XP_006654368.1 endo-1,3;1,4-beta-D-glucanase-like
TCONS_00010080	0.684712	1.12678	0.688573	BGIOSGA012625	XP_015698612.1PREDICTED: uncharacterized protein LOC107305446
TCONS_00008494	0.684712	0.174089	0.731641	BGIOSGA031548	XP_006648885.1 cytochrome b5-like
TCONS_00035754	0.685093	1.60416	0.570717	BGIOSGA002317	XP_006663910.1 receptor-like protein 12
TCONS_00027690	0.685244	-0.817615	-0.580362	BGIOSGA036343	XP_015695554.1 F-box protein SKIP1-like
TCONS_00007102	0.685432	0.450223	-0.0391725	BGIOSGA023891	XP_010235960.1alcohol dehydrogenase class-3
TCONS_00026532	0.68559	1.30944	0.673123	BGIOSGA007658	XP_006647227.1 T-complex protein 1 subunit delta
TCONS_00018692	0.685723	-0.389194	-1.13047	BGIOSGA008949	XP_006647311.1 transmembrane 9 superfamily member 12-like
TCONS_00019238	0.686027	0.0876207	1.15066	BGIOSGA018109	XP_003566273.1pentatricopeptide repeat-containing protein At1g09900
TCONS_00012730	0.686033	0.611239	-1.55284	BGIOSGA005991	XP_006650308.1 protein DMR6-LIKE OXYGENASE 2-like
TCONS_00019799	0.686222	1.61536	-1.17124	BGIOSGA009466	XP_006654793.1 ras-related protein RABA1f
TCONS_00035851	0.686977	1.35945	1.32665	BGIOSGA022903	XP_006656800.1 pentatricopeptide repeat-containing protein OGR1, mitochondrial
TCONS_00029019	0.68731	1.45325	0.927645	BGIOSGA022813	XP_011461813.1 cytochrome c oxidase copper chaperone 1-like
TCONS_00009910	0.687683	1.15921	0.266491	BGIOSGA012464	XP_006649963.1PREDICTED: uncharacterized protein LOC102722941
TCONS_00029681	0.687942	0.190673	0.169122	BGIOSGA008736	XP_006660470.1 serine/threonine-protein kinase D6PK-like
TCONS_00035615	0.688021	-2.13718	-0.849548	#N/A	#N/A
TCONS_00003506	0.688098	1.02899	2.2031	BGIOSGA012303	XP_006644155.1 serine/threonine-protein phosphatase PP1-like
TCONS_00024318	0.688439	0.652276	1.2943	BGIOSGA034412	XP_004958643.1protein SRC2
TCONS_00010437	0.68853	1.56205	0.368624	BGIOSGA013029	XP_006650302.1 inositol monophosphatase 3
TCONS_00030850	0.688553	0.93179	1.093	BGIOSGA032657	XP_006665096.1PREDICTED: uncharacterized protein LOC102702249
TCONS_00032705	0.688743	-0.224275	0.988606	BGIOSGA031404	XP_003572074.2O-fucosyltransferase 30
TCONS_00029651	0.688885	0.893327	1.58572	BGIOSGA030168	XP_006660447.1 FRIGIDA-like protein 3
TCONS_00026311	0.689335	0.150855	0.679823	BGIOSGA028397	XP_006659279.1 peptidyl-prolyl cis-trans isomerase CYP28, chloroplastic isoform X2
TCONS_00003958	0.689357	1.12541	-0.0665538	BGIOSGA001234	XP_006646084.1 ABC transporter G family member 36
TCONS_00015549	0.689626	0.381656	1.39013	BGIOSGA000507	XP_004954267.126S proteasome regulatory subunit 6A homolog
TCONS_00008825	0.689968	0.696727	1.21515	BGIOSGA029665	XP_006647920.2 histidine--tRNA ligase, chloroplastic/mitochondrial
TCONS_00001678	0.690042	0.504364	1.30639	BGIOSGA012104	XP_003569667.1probable mitochondrial-processing peptidase subunit beta, mitochondrial
TCONS_00008869	0.690419	0.335594	0.872869	BGIOSGA005543	XP_006665070.1 prolyl-tRNA synthetase associated domain-containing protein 1
TCONS_00008927	0.690467	1.33113	1.97562	BGIOSGA028777	XP_006648013.1 chaperone protein DnaJ-like
TCONS_00011027	0.690499	-2.40978	-0.122828	BGIOSGA034920	XP_010229320.1uncharacterized protein LOC104581898

TCONS_00023182	0.690635	0.633718	0.841803	BGIOSGA028447	NP_001151302.1F-box domain containing protein
TCONS_00037037	0.69088	-0.343579	0.0202188	BGIOSGA036339	XP_006664505.1 telomerase reverse transcriptase
TCONS_00030884	0.691265	0.499177	0.48804	BGIOSGA032684	XP_006665051.1 ATP synthase subunit gamma, mitochondrial
TCONS_00037410	0.692159	1.38456	0.489906	BGIOSGA029476	XP_015698397.1 probable inactive purple acid phosphatase 1
TCONS_00026341	0.692162	-1.30047	-0.807494	#N/A	#N/A
TCONS_00035574	0.692162	#NA	0.192506	#N/A	#N/A
TCONS_00036979	0.692504	-0.329511	-1.37558	BGIOSGA018502	XP_021321955.1disease resistance protein RPM1
TCONS_00017459	0.692633	-3.20414	1.01641	BGIOSGA019533	XP_006654213.1PREDICTED: uncharacterized protein LOC102718927
TCONS_00013410	0.693033	0.306178	-0.52175	BGIOSGA009572	XP_006650787.1 probable methylenetetrahydrofolate reductase
TCONS_00031123	0.693045	-0.103793	0.619066	BGIOSGA032939	XP_015696998.1 pentatricopeptide repeat-containing protein At5g61370, mitochondrial-like
TCONS_00008176	0.693176	1.02146	2.05261	BGIOSGA006263	XP_006647371.1 GPN-loop GTPase 2
TCONS_00008239	0.693683	1.02086	0.745126	BGIOSGA014963	XP_006647413.1 NAP1-related protein 2
TCONS_00020412	0.693765	1.30711	0.598204	BGIOSGA022597	XP_006655949.1 protease Do-like 9
TCONS_00020735	0.694036	0.825489	0.388278	BGIOSGA022927	XP_006656093.1 protein BOLA4, chloroplastic/mitochondrial
TCONS_00016528	0.694232	-3.07126	-1.0847	BGIOSGA007316	XP_006653723.1 myb-related protein Zm1-like
TCONS_00006203	0.694233	1.66357	1.33445	BGIOSGA016367	XP_006647345.1 mitochondrial fission protein ELM1-like
TCONS_00033930	0.694427	-7.0705	-0.631	BGIOSGA024969	XP_003561781.1dirigent protein 1
TCONS_00031059	0.694535	0.971978	-0.0233352	BGIOSGA030656	XP_006662337.1 uncharacterized oxidoreductase At4g09670-like
TCONS_00024123	0.694659	1.40933	0.472243	BGIOSGA032032	XP_008650908.1AT hook-containing MAR binding 1-like protein isoform X1
TCONS_00019911	0.694932	0.851329	0.564911	BGIOSGA017451	XP_015693011.1 V-type proton ATPase subunit C isoform X2
TCONS_00014721	0.694934	1.32928	0.409549	BGIOSGA016895	XP_004976449.1protein TRIGALACTOSYLDIACYLGLYCEROL 1, chloroplastic
TCONS_00004940	0.695289	0.704018	0.153916	BGIOSGA000281	XP_006645204.1PREDICTED: uncharacterized protein LOC102719341
TCONS_00013233	0.695319	1.8065	0.688196	BGIOSGA011889	XP_015690184.1 thioredoxin-like protein HCF164, chloroplastic
TCONS_00012794	0.695525	0.617702	0.786409	BGIOSGA029383	XP_003562016.1kxDL motif-containing protein 1
TCONS_00005904	0.695542	0.879762	-0.213325	BGIOSGA028777	XP_006647203.1 dnaJ homolog subfamily B member 4-like
TCONS_00011479	0.696062	1.36582	0.738265	BGIOSGA011546	XP_004985893.1anthranilate phosphoribosyltransferase, chloroplastic
TCONS_00013422	0.696286	-4.52378	-1.70713	BGIOSGA017466	XP_015690126.1 protein trichome birefringence-like 33
TCONS_00012167	0.69629	1.05626	1.0045	BGIOSGA014979	XP_006650004.1 endoplasmic reticulum-Golgi intermediate compartment protein 3-like
TCONS_00026652	0.69681	1.94336	1.29805	BGIOSGA030745	XP_006659428.1PREDICTED: uncharacterized protein LOC102714113
TCONS_00007206	0.696859	-2.10946	-1.46063	BGIOSGA028353	XP_004951214.1probable receptor-like protein kinase At1g33260
TCONS_00030829	0.697045	0.660658	0.382886	BGIOSGA031468	XP_015696938.1 UTP:RNA uridylyltransferase 1-like
TCONS_00026607	0.697778	0.0118767	2.43131	BGIOSGA028668	XP_015695758.1 protein CHAPERONE-LIKE PROTEIN OF POR1, chloroplastic-like
TCONS_00036029	0.697781	-0.370798	0.56516	BGIOSGA037393	NP_001316335.1uncharacterized LOC103633003
TCONS_00033178	0.698124	0.404461	0.439138	BGIOSGA035013	XP_006662830.2 glucuronokinase 1-like
TCONS_00036521	0.698336	0.314553	0.397504	BGIOSGA036816	XP_015698842.1 mitochondrial substrate carrier family protein C-like
TCONS_00010067	0.698347	-0.0413106	0.654099	BGIOSGA020490	XP_015690240.1 CRS2-like protein, chloroplastic
TCONS_00022050	0.698368	-0.0840864	0.156821	BGIOSGA021466	XP_006655994.1 signal peptidase complex catalytic subunit SEC11A-like
TCONS_00002724	0.698374	0.697626	1.0701	BGIOSGA002499	XP_015688119.1 leucine--tRNA ligase, chloroplastic/mitochondrial
TCONS_00011222	0.698995	0.781279	-0.00488654	BGIOSGA029525	XP_015690980.1 cinnamoyl-CoA reductase 1
TCONS_00009597	0.699414	-2.62762	#NA	BGIOSGA020466	XP_015689819.1 probable inactive receptor kinase At1g48480
TCONS_00025028	0.699562	1.45019	1.16126	BGIOSGA025924	XP_006657664.1 mitochondrial pyruvate carrier 4-like
TCONS_00009048	0.699703	2.04633	-1.14973	BGIOSGA005357	NP_001306640.1protein LUTEIN DEFICIENT 5, chloroplastic
TCONS_00013452	0.699884	0.422896	0.172885	BGIOSGA024541	XP_004981213.1mitochondrial import inner membrane translocase subunit TIM10
TCONS_00019766	0.700173	1.09089	1.402	BGIOSGA004197	XP_015692699.1PREDICTED: uncharacterized protein LOC102704064, partial
TCONS_00015251	0.700302	1.35733	1.08977	BGIOSGA015731	XP_006652086.2PREDICTED: uncharacterized protein LOC102714910
TCONS_00020042	0.700312	0.786279	-1.22528	BGIOSGA022053	XP_006656574.2 glutamate formimidoyltransferase
TCONS_00014590	0.700649	1.4255	0.535905	BGIOSGA013026	XP_002446782.1uncharacterized protein At4g13200, chloroplastic
TCONS_00031963	0.700674	-5.53611	-3.54811	BGIOSGA011093	XP_003571721.1uncharacterized protein LOC100840322
TCONS_00020287	0.700977	1.8729	0.8126	BGIOSGA022485	XP_006655888.1 G-protein coupled receptor 1
TCONS_00004056	0.701274	0.370028	0.822958	BGIOSGA001140	XP_015699222.1 dual-specificity RNA methyltransferase RlmN
TCONS_00030059	0.701584	-0.0998478	-0.228437	BGIOSGA029750	XP_006650561.1 deoxyhypusine synthase-like
TCONS_00032434	0.701698	-0.317182	-0.831696	BGIOSGA026430	XP_006663109.1 transcription factor MYB108-like
TCONS_00000606	0.701898	0.727673	0.727942	BGIOSGA003191	XP_006645732.1 transcription factor Pur-alpha 1

TCONS_00014763	0.702214	0.503015	0.655719	BGIOSGA006296	XP_006652653.1 WD repeat-containing protein 44-like
TCONS_00012080	0.702226	0.984263	0.234446	BGIOSGA009501	XP_002463438.1uncharacterized protein LOC8084976
TCONS_00029734	0.702375	2.12736	1.86259	BGIOSGA001769	XP_006660491.1 30S ribosomal protein 2, chloroplastic
TCONS_00030971	0.703083	0.927473	1.12723	BGIOSGA033336	XP_006661719.1 probable glutathione S-transferase GSTU6
TCONS_00014069	0.703309	1.58986	0.352252	BGIOSGA000755	XP_015692410.1 protein DETOXIFICATION 16-like
TCONS_00007146	0.703608	-0.746569	0.379383	BGIOSGA009332	XP_006649190.2 methionine adenosyltransferase 2 subunit beta
TCONS_00008448	0.703845	0.280263	0.222045	BGIOSGA013390	XP_004953202.1protein PEROXIN-4
TCONS_00020075	0.704025	0.147494	0.796759	BGIOSGA022255	XP_015693722.1 ATP-dependent Clp protease proteolytic subunit-related protein 2, chloroplastic
TCONS_00013217	0.7042	0.0229475	0.18456	BGIOSGA013616	XP_006650634.1 casein kinase II subunit alpha
TCONS_00029398	0.70461	-1.10494	-0.503458	BGIOSGA031150	XP_002460450.2OTU domain-containing protein At3g57810 isoform X1
TCONS_00014736	0.704948	0.389008	0.996422	BGIOSGA016913	XP_006652636.2 thioredoxin-like 3-3
TCONS_00036562	0.705311	-1.34243	0.229494	BGIOSGA007670	XP_004978523.1probable WRKY transcription factor 62
TCONS_00012189	0.705792	1.94442	1.17604	BGIOSGA012542	XP_003558000.1ricin B-like lectin R40C1
TCONS_00032693	0.705857	-0.469594	-0.0652334	BGIOSGA030579	XP_006662053.1 S-norcoclaurine synthase 1-like
TCONS_00031579	0.706305	0.74959	-0.334153	BGIOSGA033394	NP_001145066.1uncharacterized LOC100278262 precursor
TCONS_00018269	0.706668	0.800078	1.28761	BGIOSGA030586	XP_006654755.1 syntaxin-71
TCONS_00011398	0.706759	0.494476	0.460475	BGIOSGA033439	XP_006649223.2 quinone oxidoreductase 1-like isoform X2
TCONS_00028085	0.706896	0.718642	0.477222	BGIOSGA026907	NP_001328050.1Splicing factor 3B subunit 5/RDS3 complex subunit 10
TCONS_00002267	0.707204	0.778257	0.0759142	BGIOSGA022876	XP_002456723.1serine hydroxymethyltransferase 7
TCONS_00011064	0.707366	2.0667	2.09271	BGIOSGA021674	XP_006650677.1 inosine-5'-monophosphate dehydrogenase-like
TCONS_00013489	0.708039	-0.609728	0.434024	BGIOSGA025452	XP_006650858.1 guanine deaminase-like
TCONS_00013068	0.708289	0.0974133	-0.585323	BGIOSGA014945	XP_006650500.1 anthranilate synthase beta subunit 2, chloroplastic
TCONS_00024216	0.708299	0.732161	0.15007	BGIOSGA020512	XP_006658044.1 NADH dehydrogenase [ubiquinone] flavoprotein 1, mitochondrial
TCONS_00000538	0.708327	0.559712	0.556657	BGIOSGA032111	XP_006643978.1PREDICTED: uncharacterized protein LOC102712021
TCONS_00024198	0.708545	0.325042	-0.215805	BGIOSGA004281	XP_006658024.1 NADH dehydrogenase [ubiquinone] 1 alpha subcomplex subunit 6
TCONS_00009116	0.708693	-0.781059	-0.21555	BGIOSGA011642	XP_015699039.1PREDICTED: uncharacterized protein LOC102712417
TCONS_00001815	0.708932	-0.0923879	-0.732665	BGIOSGA004452	XP_015699290.1 putative RNA-binding protein YlmH isoform X2
TCONS_00011141	0.709182	1.42916	0.828752	BGIOSGA013741	XP_015690111.1 probable GTP-binding protein OBGC2
TCONS_00027958	0.70945	0.565433	-0.0091999	BGIOSGA027038	XP_003574414.1uncharacterized protein LOC100846834
TCONS_00007329	0.709993	-2.6802	-1.04088	BGIOSGA021229	XP_012704165.1achilleol B synthase isoform X1
TCONS_00008506	0.71001	0.495636	0.221442	BGIOSGA000160	XP_006647615.1 vesicle-associated protein 2-1
TCONS_00000614	0.710169	-0.846409	0.309008	BGIOSGA003197	XP_006644022.2 transmembrane protein 136-like
TCONS_00023943	0.710172	0.151087	0.788016	BGIOSGA025975	XP_006658715.1 ribosome-recycling factor, chloroplastic
TCONS_00020844	0.710414	1.43891	1.29209	BGIOSGA024073	XP_015694259.1 T-complex protein 1 subunit gamma
TCONS_00005769	0.710707	0.362232	0.664527	BGIOSGA009950	XP_006647117.1 prefoldin subunit 6
TCONS_00005385	0.710772	0.0857122	1.28904	BGIOSGA008357	XP_006648298.2 glucan endo-1,3-beta-glucosidase 6-like
TCONS_00003941	0.710859	1.67899	1.28335	BGIOSGA020469	XP_006644343.1PREDICTED: uncharacterized protein LOC102702791
TCONS_00019719	0.710905	-0.176471	-0.155888	BGIOSGA004291	NP_001148662.1uncharacterized protein LOC100282278
TCONS_00008993	0.710964	0.42861	0.308229	BGIOSGA015423	XP_006648069.2 26S protease regulatory subunit 6A homolog
TCONS_00009480	0.711019	0.249909	0.102921	BGIOSGA026022	XP_015690825.1 scarecrow-like protein 21
TCONS_00014336	0.711022	1.33748	1.40213	BGIOSGA008458	XP_006652339.1PREDICTED: uncharacterized protein LOC102700888
TCONS_00015043	0.711195	0.473676	0.680989	BGIOSGA005627	XP_006659173.1 cullin-3A-like
TCONS_00010991	0.711618	0.38672	0.600129	BGIOSGA021445	XP_006650618.1 26S proteasome non-ATPase regulatory subunit 10
TCONS_00021661	0.71187	1.20768	-0.21058	BGIOSGA033011	XP_015694288.1 soluble starch synthase 1, chloroplastic/amyloplastic
TCONS_00023190	0.712702	0.837175	0.540955	BGIOSGA013552	XP_015695227.1PREDICTED: uncharacterized protein LOC102700914
TCONS_00001384	0.712725	0.694794	0.685628	BGIOSGA023017	XP_015699310.1PREDICTED: uncharacterized protein LOC102713682
TCONS_00010841	0.713109	1.29972	0.571356	BGIOSGA031952	XP_015690042.1 probable histidine kinase 4
TCONS_00036610	0.713207	1.2493	1.9471	BGIOSGA034570	XP_015697769.1 mitochondrial import inner membrane translocase subunit TIM8
TCONS_00025367	0.71336	1.14199	1.04676	BGIOSGA016994	XP_006657859.1 calcium-dependent protein kinase 8-like
TCONS_00035853	0.71342	-0.0192323	-0.428125	BGIOSGA019417	XP_006663941.1 WEB family protein At5g55860
TCONS_00032029	0.713499	0.367422	0.96314	BGIOSGA032113	XP_015697424.1 CASP-like protein 5A2
TCONS_00007111	0.714277	-0.0802106	0.341754	BGIOSGA009295	XP_006648116.1PREDICTED: uncharacterized protein LOC102718989
TCONS_00020134	0.714335	0.591933	0.535657	BGIOSGA023597	XP_015693451.1 B-box zinc finger protein 22-like
TCONS_00001680	0.714402	0.260108	-1.90011	BGIOSGA020897	XP_004969722.3 glycine dehydrogenase (decarboxylating), mitochondrial-like

TCONS_00005501	0.714605	-1.42206	0.295162	BGIOSGA004491	XP_006448872.1tubulin beta chain isoform X2
TCONS_00023996	0.714741	1.29323	1.57799	BGIOSGA011361	XP_006657895.1 putative MO25-like protein At5g47540
TCONS_00003728	0.714749	-0.404463	0.737837	BGIOSGA014293	XP_012701442.1cysteine-rich receptor-like protein kinase 15
TCONS_00017031	0.714916	0.0450752	0.552414	BGIOSGA019079	XP_006653985.1PREDICTED: uncharacterized protein LOC102722116
TCONS_00021966	0.715124	-0.115432	-1.16999	BGIOSGA002433	XP_006644049.1PREDICTED: uncharacterized protein LOC102708379
TCONS_00034175	0.715503	-1.25341	0.529501	BGIOSGA007670	XP_004978523.1probable WRKY transcription factor 62
TCONS_00016638	0.71561	0.331412	0.150723	BGIOSGA001116	XP_006652846.1 transcription factor GTE2-like
TCONS_00014234	0.716115	1.1353	0.248678	BGIOSGA016387	XP_006652276.2 succinate dehydrogenase subunit 5, mitochondrial
TCONS_00031476	0.716164	0.231177	0.527855	BGIOSGA033291	XP_006662520.1PREDICTED: uncharacterized protein LOC102718398
TCONS_00024385	0.716224	1.34261	1.30839	BGIOSGA026427	XP_006658985.1 rhodanese-like/PpiC domain-containing protein 12, chloroplastic
TCONS_00012662	0.716312	0.93143	-1.08549	BGIOSGA017254	XP_006650293.1 isoaspartyl peptidase/L-asparaginase 1
TCONS_00018982	0.716626	-0.882213	-1.02739	BGIOSGA020538	XP_006654201.1 ras-related protein RGP2
TCONS_00021574	0.716648	0.356969	0.321176	BGIOSGA005534	XP_006647970.1 proteasome subunit beta type-6-like
TCONS_00035792	0.716692	0.348842	0.340342	BGIOSGA034093	XP_006664452.1 adenylate kinase 3
TCONS_00009835	0.716747	0.951658	0.897793	BGIOSGA025331	XP_015690776.1 carbon catabolite-derepressing protein kinase-like
TCONS_00005339	0.717227	0.0523458	-0.269543	BGIOSGA007447	NP_001314799.1uncharacterized LOC103626562
TCONS_00013128	0.717406	2.20362	1.86872	BGIOSGA032644	XP_006650548.1 folate transporter 1, chloroplastic
TCONS_00020389	0.718074	1.06527	1.2821	BGIOSGA019094	XP_006655935.1 transcription factor TCP14-like
TCONS_00028617	0.718288	0.76692	0.281572	BGIOSGA002627	XP_015696872.1 acetyl-CoA acetyltransferase, cytosolic 1-like
TCONS_00024452	0.718343	-0.00746166	0.678297	#N/A	#N/A
TCONS_00022612	0.718646	-0.117263	-0.456214	BGIOSGA007825	XP_015694454.1 nudix hydrolase 2
TCONS_00032900	0.719245	1.09724	0.014845	BGIOSGA001894	XP_015699121.1 calmodulin-binding protein 25-like
TCONS_00010668	0.719478	1.54629	2.6934	BGIOSGA019389	XP_015698376.1 glycine-rich RNA-binding protein GRP1A-like
TCONS_00002348	0.719548	1.95819	1.29459	BGIOSGA004989	XP_015692088.1PREDICTED: uncharacterized protein LOC102712314 isoform X2
TCONS_00026419	0.719772	-0.269127	0.99737	BGIOSGA030441	XP_003573802.1uncharacterized protein LOC100836363
TCONS_00003863	0.72033	0.00147398	-0.488276	BGIOSGA020277	XP_006644311.1PREDICTED: uncharacterized protein LOC102717292 isoform X2
TCONS_00031318	0.720383	1.32936	0.716247	BGIOSGA033134	XP_006664816.1 malate dehydrogenase, cytoplasmic
TCONS_00016688	0.720429	-1.38161	0.508642	BGIOSGA020657	XP_006652880.1PREDICTED: uncharacterized protein LOC102721919
TCONS_00037159	0.720487	0.347888	0.723381	BGIOSGA038148	XP_015698920.1 5-methylthioadenosine/S-adenosylhomocysteine deaminase-like isoform X1
TCONS_00034161	0.720541	-4.72671	-1.32169	BGIOSGA036775	XP_006664873.1 BTB/POZ domain-containing protein At3g56230-like
TCONS_00006513	0.720863	1.29748	0.515772	BGIOSGA008680	XP_006647571.1 bifunctional nitrilase/nitrile hydratase NIT4
TCONS_00018607	0.721121	0.176252	1.14385	BGIOSGA018827	XP_006654982.2PREDICTED: uncharacterized protein LOC102710786
TCONS_00018843	0.721344	0.929239	1.25655	BGIOSGA037015	XP_015693245.1 UDP-galactose transporter 2-like
TCONS_00037222	0.72147	0.444791	0.198758	BGIOSGA015207	XP_003576139.1NAD(P)H-quinone oxidoreductase subunit U, chloroplastic
TCONS_00016342	0.721941	-0.052927	0.455509	BGIOSGA023488	XP_024311400.1cyclin-P4-1
TCONS_00033796	0.722071	1.79966	0.597452	BGIOSGA002494	XP_006663028.2PREDICTED: uncharacterized protein LOC102719334
TCONS_00006710	0.722432	1.31529	1.09986	BGIOSGA024671	XP_002454428.13-dehydrosphinganine reductase TSC10A
TCONS_00018570	0.722595	1.68464	0.650696	BGIOSGA018862	XP_015692471.1 nitrogen regulatory protein P-II homolog
TCONS_00023162	0.722679	-0.331206	1.1023	BGIOSGA025200	NP_001338687.1DUF3148 family protein
TCONS_00027132	0.72358	0.884162	-0.329253	BGIOSGA014213	XP_006659684.1 KDEL-tailed cysteine endopeptidase CEP1
TCONS_00030291	0.723732	1.01839	1.01363	BGIOSGA023556	XP_006660788.1 methyl-CpG-binding domain-containing protein 2-like
TCONS_00014687	0.723796	3.17254	1.11392	#N/A	#N/A
TCONS_00033490	0.723884	-3.73093	0.0623116	BGIOSGA002287	XP_004979290.1protein LAZY 1
TCONS_00020656	0.724154	-1.56135	0.669326	BGIOSGA025978	XP_015693567.1 primary amine oxidase-like
TCONS_00036932	0.72512	0.378845	0.484856	BGIOSGA036395	XP_006663931.1 54S ribosomal protein L37, mitochondrial
TCONS_00001579	0.725512	-0.624707	0.307118	BGIOSGA004212	XP_015688282.1 D-glycerate 3-kinase, chloroplastic isoform X2
TCONS_00000406	0.725521	0.740058	1.44654	BGIOSGA016551	XP_015692411.1 14-3-3-like protein GF14-G
TCONS_00027128	0.725645	-1.08198	0.758636	BGIOSGA031944	XP_006659680.1 dihydroneopterin aldolase 2-like
TCONS_00012897	0.725731	0.591463	0.941391	BGIOSGA010086	XP_004982216.1guanine nucleotide-binding protein subunit beta
TCONS_00001622	0.72601	-0.0540007	0.462697	BGIOSGA017625	XP_006644536.1 phosphoethanolamine N-methyltransferase 1-like
TCONS_00000388	0.726061	0.230881	#NA	BGIOSGA002162	NP_001151708.1rho termination factor, N-terminal domain containing protein
TCONS_00034488	0.726144	1.02112	0.419298	BGIOSGA026286	XP_006662809.1PREDICTED: uncharacterized protein LOC102717289
TCONS_00009194	0.726242	0.649944	0.240831	BGIOSGA001970	XP_006649280.1 protein-ribulosamine 3-kinase, chloroplastic isoform X1

TCONS_00026781	0.726735	1.18674	1.31297	BGIOSGA024331	XP_006659495.1 protein transport protein SEC23
TCONS_00004887	0.727024	1.87137	1.22363	BGIOSGA025454	XP_006646560.1 calreticulin-3-like
TCONS_00007772	0.727062	0.90449	1.22095	BGIOSGA006711	XP_015688549.1 proteinaceous RNase P 1, chloroplastic/mitochondrial-like
TCONS_00011245	0.727135	-0.618557	0.00624025	BGIOSGA013845	XP_006651967.1PREDICTED: uncharacterized protein LOC102714280
TCONS_00027143	0.727135	0.316902	-0.764941	BGIOSGA028055	NP_001314999.1uncharacterized protein LOC100502427 isoform 2
TCONS_00011439	0.727313	#NA	-2.46481	BGIOSGA030655	XP_004986014.1probable receptor-like protein kinase At4g10390
TCONS_00001871	0.727752	0.285104	0.322122	BGIOSGA013088	XP_003569879.2 glycerophosphodiester phosphodiesterase GDPD6
TCONS_00035012	0.728116	-0.53742	0.0787917	BGIOSGA033845	XP_015690236.1 GDT1-like protein 2, chloroplastic isoform X1
TCONS_00021621	0.728167	0.347897	0.329046	BGIOSGA021897	XP_014753236.1protein WHI3 isoform X2
TCONS_00030902	0.728959	-1.29829	0.404399	BGIOSGA032706	XP_002457536.1nodulin-related protein 1
TCONS_00025600	0.729192	2.22674	1.6857	BGIOSGA023832	XP_015694490.1PREDICTED: uncharacterized protein LOC107304516 isoform X1
TCONS_00022848	0.729192	1.44913	2.27066	#N/A	#N/A
TCONS_00026023	0.729283	0.207386	0.873089	BGIOSGA028095	XP_006659890.2PREDICTED: uncharacterized protein LOC102713016
TCONS_00027454	0.729621	0.800501	0.898241	BGIOSGA004062	XP_006659177.1 protein RAE1-like
TCONS_00027602	0.729649	0.744705	1.12435	BGIOSGA027413	XP_008661708.1uncharacterized LOC100275707 isoform X1
TCONS_00029993	0.729658	0.290025	0.890565	BGIOSGA026957	XP_006660603.1 type IV inositol polyphosphate 5-phosphatase 9-like
TCONS_00020181	0.729725	-0.782638	-0.300864	BGIOSGA022372	XP_006655821.1 protein disulfide isomerase-like 1-5
TCONS_00006547	0.729855	0.0295859	0.649676	BGIOSGA027645	XP_006647604.1 monothiol glutaredoxin-S6
TCONS_00021372	0.73007	1.51917	1.13323	BGIOSGA028671	XP_006656486.1 ATPase family AAA domain-containing protein 1-like isoform X1
TCONS_00006833	0.730373	0.566491	0.481809	BGIOSGA037876	XP_006647855.1 microtubule-associated protein 70-2
TCONS_00018651	0.730614	0.128783	0.481214	BGIOSGA018784	XP_006655007.2 gamma-tubulin complex component 4
TCONS_00036589	0.730655	0.5105	0.784979	BGIOSGA034585	XP_006662694.1PREDICTED: uncharacterized protein LOC102706044
TCONS_00023812	0.730711	0.88355	1.05034	BGIOSGA022137	XP_014753308.1protein ACCELERATED CELL DEATH 6 isoform X1
TCONS_00007179	0.730903	1.11837	0.696232	BGIOSGA007318	XP_006648192.1PREDICTED: uncharacterized protein LOC102717217
TCONS_00028259	0.730908	0.288445	0.974892	BGIOSGA026717	XP_006660257.2 pseudouridine-5'-phosphate glycosidase
TCONS_00031410	0.73104	1.57557	1.76322	BGIOSGA022219	XP_021306530.1monothiol glutaredoxin-S11 isoform X1
TCONS_00011740	0.731047	1.10944	0.0222639	BGIOSGA011267	XP_006649582.1 yrdC domain-containing protein, mitochondrial-like
TCONS_00025547	0.731113	0.246397	0.407449	BGIOSGA023885	XP_006657978.1 transcription elongation factor SPT4 homolog 2-like
TCONS_00024634	0.731117	1.54618	0.903358	#N/A	#N/A
TCONS_00014289	0.731176	-0.406922	0.650246	BGIOSGA016444	XP_006653417.1 protein EI24 homolog
TCONS_00004401	0.731335	-0.591058	-0.122246	BGIOSGA000820	XP_006646328.1 3-hydroxyisobutyryl-CoA hydrolase-like protein 5
TCONS_00031669	0.731384	0.322156	-0.282717	BGIOSGA033491	XP_006662622.1 uncharacterized sugar kinase slr0537
TCONS_00006062	0.731722	-1.0798	-0.891286	BGIOSGA016972	XP_015688595.1 phenolic glucoside malonyltransferase 1-like
TCONS_00017171	0.731903	0.691912	1.33766	BGIOSGA019390	XP_006654073.1 DNA-directed RNA polymerase V subunit 7-like
TCONS_00023877	0.732263	1.32979	0.614797	BGIOSGA024134	XP_015694677.1 pentatricopeptide repeat-containing protein At5g42310, mitochondrial-like
TCONS_00029144	0.732305	1.87461	0.211115	BGIOSGA005544	XP_006660689.1 mitochondrial phosphate carrier protein 3, mitochondrial-like
TCONS_00021453	0.732608	0.888065	0.803597	BGIOSGA022098	XP_006655686.1 protein CWC15 homolog
TCONS_00008184	0.732656	1.42538	1.27211	BGIOSGA006254	XP_006647382.1 eukaryotic translation initiation factor 2D
TCONS_00027761	0.73271	-0.95825	-1.02768	BGIOSGA000355	XP_006659275.1 threonine--tRNA ligase, mitochondrial 1
TCONS_00021857	0.732801	0.781107	-0.271547	BGIOSGA019900	XP_006655913.1 MADS-box transcription factor 55
TCONS_00002507	0.73295	1.3826	1.47307	BGIOSGA025801	XP_015697990.1PREDICTED: uncharacterized protein LOC102704371 isoform X2
TCONS_00036415	0.732983	-0.406058	0.384747	BGIOSGA037781	XP_006664185.1 beta-1,4-mannosyl-glycoprotein 4-beta-N-acetylglucosaminyltransferase
TCONS_00036316	0.733201	1.08536	1.22869	BGIOSGA037680	XP_004963014.1CSC1-like protein HYP1 isoform X1
TCONS_00021999	0.733238	-1.34736	-0.814954	BGIOSGA023252	XP_003563993.11-aminocyclopropane-1-carboxylate oxidase homolog 4
TCONS_00028584	0.733374	2.02578	1.36867	BGIOSGA005528	XP_006660436.2 probable tyrosine-protein phosphatase At1g05000 isoform X2
TCONS_00025539	0.733653	0.933314	0.601701	BGIOSGA023894	XP_015694730.1 ATP-dependent RNA helicase DEAH11, chloroplastic-like
TCONS_00019224	0.733805	-0.0421528	-0.910583	BGIOSGA022596	XP_004962200.1sm-like protein LSM5
TCONS_00017468	0.734119	1.43122	1.37649	BGIOSGA003155	XP_006654202.1 E3 ubiquitin-protein ligase hel2
TCONS_00029507	0.734441	0.893584	1.42982	BGIOSGA009917	XP_006660975.2 ultraviolet-B receptor UVR8
TCONS_00019128	0.734553	-2.45015	-0.313826	BGIOSGA037857	XP_004962397.1probable inactive receptor-like protein kinase At3g56050 isoform X2
TCONS_00011048	0.734997	0.307925	0.776373	BGIOSGA033210	XP_015690091.1 probable methyltransferase PMT28
TCONS_00008253	0.735788	0.850573	0.527417	BGIOSGA008969	XP_006647425.1 ASC1-like protein 1 isoform X1
TCONS_00004247	0.736067	0.843813	0.196521	BGIOSGA026025	XP_014754126.1mitogen-activated protein kinase kinase kinase 17

TCONS_00031485	0.736382	0.0504825	0.454422	BGIOSGA033299	XP_006662525.1 protein COFACTOR ASSEMBLY OF COMPLEX C SUBUNIT B CCB2, chloroplastic
TCONS_00006200	0.736407	0.389627	1.55047	BGIOSGA008362	XP_006660598.1 N-carbamoylputrescine amidase
TCONS_00001186	0.736612	0.795529	1.22491	BGIOSGA019984	XP_015699228.1 ATP-dependent zinc metalloprotease FTSH 5, mitochondrial isoform X1
TCONS_00011020	0.73689	0.447786	1.31483	BGIOSGA013623	XP_015690323.1PREDICTED: uncharacterized protein LOC102700979
TCONS_00025724	0.737439	-0.311171	0.0611652	BGIOSGA011007	XP_006658977.1 cyclic dof factor 1-like isoform X1
TCONS_00004760	0.737464	-0.13566	0.613579	BGIOSGA000453	XP_006645050.1 transcription initiation factor TFIID subunit 12
TCONS_00031450	0.737981	1.0108	0.749557	BGIOSGA033268	XP_006661953.1 UPF0496 protein 4
TCONS_00015477	0.738118	0.501752	0.776424	BGIOSGA014067	XP_015692357.1PREDICTED: uncharacterized protein LOC102708331
TCONS_00001944	0.738367	0.101671	0.880087	BGIOSGA010683	XP_015697855.1 nodulation protein H-like
TCONS_00018971	0.739029	0.997405	0.395202	BGIOSGA023406	XP_002437396.1pentatricopeptide repeat-containing protein At2g18940, chloroplastic
TCONS_00015153	0.739085	0.826778	-0.186663	BGIOSGA017333	XP_006653010.1 peroxisome biogenesis protein 22-like
TCONS_00034548	0.739179	-0.903354	-0.62827	BGIOSGA033029	XP_004979091.1uncharacterized protein LOC101753208
TCONS_00021460	0.739183	0.188977	-1.36053	BGIOSGA007302	XP_006655693.1 ferredoxin--NADP reductase, leaf isozyme, chloroplastic
TCONS_00010542	0.739204	1.21443	2.07613	#N/A	#N/A
TCONS_00009809	0.739349	1.00732	0.759993	BGIOSGA012363	XP_006649878.1PREDICTED: uncharacterized protein LOC102722940
TCONS_00024104	0.739586	0.626805	0.825098	BGIOSGA007754	XP_015695235.1PREDICTED: uncharacterized protein LOC102709758
TCONS_00035385	0.739891	#NA	#NA	BGIOSGA032669	XP_003577269.1probable LRR receptor-like serine/threonine-protein kinase At3g47570
TCONS_00024753	0.739999	2.05595	0.440361	BGIOSGA022232	NP_001147550.2alkaline alpha galactosidase 1
TCONS_00013665	0.740194	1.17618	1.64006	BGIOSGA015824	XP_006652089.2 protein AAR2 homolog isoform X1
TCONS_00026118	0.740558	0.627912	2.39471	BGIOSGA030215	XP_006659933.1 GTP-binding protein ERG
TCONS_00019267	0.740922	1.31934	0.685261	BGIOSGA018081	XP_006654383.1PREDICTED: uncharacterized protein LOC102699685
TCONS_00009541	0.74115	0.438179	0.195342	BGIOSGA026064	XP_006650427.1 vesicle transport protein GOT1B-like isoform X2
TCONS_00023727	0.741396	1.61356	0.541929	BGIOSGA006807	XP_015694643.1 anthocyanidin 3-O-glucosyltransferase 2-like
TCONS_00011117	0.74155	0.530872	-0.379979	BGIOSGA019772	XP_003557676.1uncharacterized protein LOC100842786
TCONS_00036742	0.74216	1.03941	1.03481	BGIOSGA019367	XP_015698464.1 pentatricopeptide repeat-containing protein At4g15720-like
TCONS_00032408	0.74245	0.947708	0.653051	BGIOSGA031707	XP_024316154.1protein NPGR2-like
TCONS_00014639	0.743048	1.32556	-0.933393	BGIOSGA031071	XP_006652542.1 mitochondrial intermembrane space import and assembly protein 40
TCONS_00036280	0.743048	0.210078	-0.933393	#N/A	#N/A
TCONS_00028181	0.743847	0.696155	0.368728	BGIOSGA026803	XP_006659502.1 ATP synthase subunit d, mitochondrial
TCONS_00008857	0.744018	-0.169998	-1.27924	BGIOSGA005558	XP_006647960.2 photosystem I chlorophyll a/b-binding protein 5, chloroplastic
TCONS_00023357	0.744206	-0.418115	1.06424	BGIOSGA025399	XP_006649646.1 superoxide dismutase [Cu-Zn] 2-like
TCONS_00007871	0.744252	1.64698	0.974698	BGIOSGA002027	XP_006647207.2 C2 domain-containing protein At1g53590-like
TCONS_00008784	0.74461	1.89319	1.40677	BGIOSGA005651	XP_015688369.1 protein MSP1
TCONS_00010137	0.744712	-1.73627	-0.891635	BGIOSGA012725	XP_002467808.1flavonoid 3',5'-hydroxylase 1
TCONS_00014104	0.744751	-2.37675	-0.352647	BGIOSGA017717	NP_001144230.1uncharacterized protein LOC100277095 precursor
TCONS_00008892	0.745049	-0.945771	-0.34502	BGIOSGA005518	XP_006647985.2 putative D-cysteine desulfhydrase 1, mitochondrial
TCONS_00024401	0.745101	1.01813	0.80918	BGIOSGA004480	XP_006658171.1PREDICTED: uncharacterized protein LOC102700724
TCONS_00001439	0.745248	0.0872095	-1.51311	BGIOSGA035541	XP_006644416.2 scopoletin glucosyltransferase-like
TCONS_00008718	0.745544	0.386348	0.318711	BGIOSGA007738	XP_015688808.1 ADP-ribosylation factor GTPase-activating protein AGD12-like isoform X1
TCONS_00022391	0.745565	1.50185	1.87842	BGIOSGA021069	XP_021304348.1berberine bridge enzyme-like 18
TCONS_00016836	0.745608	1.93952	1.18213	BGIOSGA003068	XP_006652994.1 methylthioribose kinase 1
TCONS_00037821	0.745815	0.20706	-0.940244	BGIOSGA022951	XP_015697880.1PREDICTED: uncharacterized protein LOC107305270 isoform X1
TCONS_00010892	0.745994	-0.0969136	-0.508813	BGIOSGA013506	XP_006650539.1 photosystem I reaction center subunit N, chloroplastic
TCONS_00006589	0.74621	0.338103	1.3623	BGIOSGA000443	XP_006647635.2PREDICTED: uncharacterized protein LOC102720677, partial
TCONS_00014571	0.746257	-0.0707059	0.955933	BGIOSGA005945	XP_015692150.1 diphthine methyltransferase homolog
TCONS_00006799	0.746328	1.20651	#NA	BGIOSGA006991	XP_004951624.1mediator of RNA polymerase II transcription subunit 11
TCONS_00018354	0.746328	0.621549	0.380416	BGIOSGA012861	XP_006654820.1 nuclear transcription factor Y subunit B-4 isoform X1
TCONS_00019814	0.74645	0.843071	-0.0263713	BGIOSGA017541	XP_006655603.1 protease Do-like 1, chloroplastic
TCONS_00032676	0.746682	-3.07124	-0.554137	BGIOSGA033951	XP_015697353.1 QWRF motif-containing protein 7-like
TCONS_00037366	0.746744	0.908045	0.701847	BGIOSGA036004	XP_006664641.1 solute carrier family 40 member 2, chloroplastic
TCONS_00006389	0.747051	0.610831	1.34594	BGIOSGA008552	XP_015689345.1 telomere length regulation protein TEL2 homolog

TCONS_00028057	0.747259	0.557902	0.17992	BGIOSGA004108	XP_006659432.1 malate dehydrogenase, chloroplastic
TCONS_00023912	0.747315	-0.264017	0.345561	BGIOSGA025945	XP_015695037.1PREDICTED: uncharacterized protein LOC102718290
TCONS_00003108	0.74758	-1.44446	-0.437428	BGIOSGA018591	XP_015698084.1PREDICTED: uncharacterized protein LOC102717836
TCONS_00001072	0.747706	0.14459	0.646138	BGIOSGA024462	XP_006660440.1 trafficking protein particle complex subunit 6B
TCONS_00001104	0.747848	1.24922	1.06446	BGIOSGA003727	XP_006644285.2 jmjC domain-containing protein 4
TCONS_00019178	0.74815	0.00392458	0.488079	BGIOSGA026352	XP_006654322.1 lipase-like
TCONS_00024663	0.748251	1.20448	1.05374	#N/A	#N/A
TCONS_00029146	0.748394	0.949794	0.47815	BGIOSGA006926	XP_006660691.2 NF-kappa-B-activating protein
TCONS_00018319	0.748537	-0.537268	0.0624707	BGIOSGA001070	XP_022682647.1CTD small phosphatase-like protein 2-A
TCONS_00003146	0.748785	1.45078	2.29851	BGIOSGA017247	XP_006645675.1 putative E3 ubiquitin-protein ligase LIN
TCONS_00015120	0.748875	1.01251	0.859116	BGIOSGA024498	XP_006652963.1 26S proteasome non-ATPase regulatory subunit 7 homolog A
TCONS_00031623	0.748956	1.56122	1.16984	BGIOSGA033437	XP_004986091.1sphingomyelin phosphodiesterase 4
TCONS_00025659	0.748999	0.815394	0.674497	BGIOSGA023763	XP_006658927.1 pentatricopeptide repeat-containing protein At1g01970
TCONS_00013333	0.749011	0.458309	-0.746194	BGIOSGA023584	XP_003557501.1leucine-rich repeat extensin-like protein 6
TCONS_00007418	0.749042	0.131834	0.61621	BGIOSGA007058	XP_006646937.2 pentatricopeptide repeat-containing protein At2g35130
TCONS_00018694	0.749155	0.173576	0.805395	BGIOSGA002311	XP_006654090.1 protein NETWORKED 4B-like
TCONS_00007240	0.749206	0.239863	0.492756	BGIOSGA018462	XP_004952164.1transcription factor ILR3
TCONS_00009667	0.749902	0.00214798	0.33819	BGIOSGA012212	XP_015690263.1 uncharacterized GTP-binding protein At5g64813-like isoform X2
TCONS_00004968	0.749973	0.93963	0.0494435	BGIOSGA007561	XP_003567400.1uncharacterized protein LOC100844366
TCONS_00037860	0.749973	-1.3823	1.37137	#N/A	XP_015697160.1PREDICTED: uncharacterized protein LOC107305099
TCONS_00017367	0.750121	-0.846978	0.168833	BGIOSGA035890	XP_006654167.1 auxin-responsive protein IAA17
TCONS_00006255	0.750717	-0.624119	-0.374901	BGIOSGA024176	XP_006647366.1 RNA polymerase II transcriptional coactivator KIWI-like
TCONS_00027892	0.751532	0.423408	-0.954455	BGIOSGA030021	XP_010234623.1lipid phosphate phosphatase 2
TCONS_00008145	0.751571	1.63205	0.237169	BGIOSGA010691	XP_015688831.1 abscisic stress-ripening protein 3-like
TCONS_00001135	0.752253	0.937212	1.62869	BGIOSGA003760	XP_014754301.1uncharacterized protein LOC100828486
TCONS_00006910	0.752253	0.937212	0.043728	BGIOSGA031878	XP_004977682.2BTB/POZ and MATH domain-containing protein 3
TCONS_00012288	0.752364	0.179304	0.381035	BGIOSGA000106	XP_006650094.1 mitochondrial fission 1 protein A-like
TCONS_00025298	0.75244	1.84667	1.08438	BGIOSGA023611	XP_006657811.1 probable proteasome inhibitor
TCONS_00029840	0.752446	-0.627252	-0.925651	BGIOSGA037843	XP_006660542.2 putative glucose-6-phosphate 1-epimerase
TCONS_00005068	0.75247	1.23643	0.892798	BGIOSGA019060	XP_004971213.1mediator of DNA damage checkpoint protein 1
TCONS_00022464	0.7526	-1.38509	-0.957149	BGIOSGA019146	XP_021314222.1uncharacterized protein LOC110434461
TCONS_00008570	0.752797	0.902132	0.104517	BGIOSGA018842	XP_022683328.1dof zinc finger protein 2 isoform X2
TCONS_00003252	0.752939	#NA	-0.958007	BGIOSGA037535	XP_002457590.1rapid alkalinization factor
TCONS_00016326	0.753516	1.04727	0.410324	BGIOSGA015555	NP_001168161.1required to maintain repression 2
TCONS_00033596	0.753544	1.71359	2.60287	BGIOSGA005513	XP_006663128.1 heat shock cognate 70 kDa protein 2
TCONS_00004908	0.753653	0.422607	1.1227	BGIOSGA000130	XP_006645183.1 DNA repair protein recA homolog 3, mitochondrial
TCONS_00008357	0.753779	0.552775	1.27018	BGIOSGA014796	XP_004953057.1la protein 1
TCONS_00029335	0.753788	0.0648527	0.16372	BGIOSGA029085	XP_015696499.1 probable E3 ubiquitin-protein ligase ARI5
TCONS_00013256	0.754343	0.871512	1.48428	BGIOSGA004935	XP_003558631.1pentatricopeptide repeat-containing protein At2g22070
TCONS_00036967	0.754443	1.10574	0.667482	BGIOSGA010192	NP_001152192.1oxygen-independent coproporphyrinogen III oxidase-like protein sll1917
TCONS_00005216	0.754812	-1.97244	1.15271	BGIOSGA007328	XP_006648201.2 rhodanese-like domain-containing protein 19, mitochondrial
TCONS_00036176	0.755914	1.20282	0.83132	BGIOSGA037533	XP_004962824.1uncharacterized protein LOC101759704
TCONS_00035228	0.756001	0.409136	0.39119	BGIOSGA036145	XP_006663065.1 phosphoserine phosphatase, chloroplastic
TCONS_00008178	0.756242	0.640232	0.438254	BGIOSGA015055	XP_015688806.1 mitogen-activated protein kinase kinase kinase YODA-like isoform X2
TCONS_00011080	0.756257	0.729685	0.594645	BGIOSGA013680	XP_006650685.1 endoplasmic reticulum-Golgi intermediate compartment protein 3
TCONS_00007262	0.756418	1.25338	1.1784	BGIOSGA007220	XP_006646806.1 eukaryotic translation initiation factor 3 subunit J-A-like
TCONS_00032423	0.757456	0.379705	0.143138	BGIOSGA010174	XP_003574056.1vacuolar protein sorting-associated protein 2 homolog 3
TCONS_00025680	0.757541	0.648801	0.404814	BGIOSGA033808	XP_006658086.2 serine/threonine-protein kinase CDL1
TCONS_00028443	0.757605	0.379186	0.00178161	BGIOSGA005460	XP_006660366.1 NEP1-interacting protein-like 1
TCONS_00011028	0.75821	1.3138	1.56021	BGIOSGA013630	XP_006650650.1 proteasome assembly chaperone 2
TCONS_00023593	0.758456	0.185082	0.628694	BGIOSGA025615	XP_006657661.1 phosphomannomutase/phosphoglucomutase isoform X1

TCONS_00001424	0.758537	0.569687	0.878603	BGIOSGA019202	XP_006644407.1 protein INVOLVED IN DE NOVO 2-like
TCONS_00011013	0.7589	0.216712	-0.279163	BGIOSGA035627	XP_004981660.1probable N-acetyltransferase HLS1-like
TCONS_00003821	0.759171	0.514604	0.964774	BGIOSGA014252	NP_001142674.1uncharacterized protein LOC100274969
TCONS_00021157	0.759178	2.26664	-0.00482983	BGIOSGA023379	XP_006656311.1PREDICTED: uncharacterized protein LOC102717163
TCONS_00000398	0.760305	1.41378	1.02331	#N/A	#N/A
TCONS_00009394	0.760555	0.253096	-0.275599	BGIOSGA011944	XP_006649465.1 ribulose-phosphate 3-epimerase, chloroplastic
TCONS_00008103	0.760599	0.120962	1.00409	BGIOSGA033288	XP_002453922.1heat stress transcription factor A-3
TCONS_00024658	0.761338	0.596013	-0.906831	BGIOSGA016111	XP_003557503.1probable ribose-5-phosphate isomerase 3, chloroplastic
TCONS_00007303	0.761456	0.318165	0.925923	BGIOSGA007181	XP_006648287.1 cyclin-T1-1-like
TCONS_00037164	0.761622	1.51178	0.827318	#N/A	#N/A
TCONS_00008354	0.761782	1.08751	0.169075	BGIOSGA006073	XP_002454154.1outer envelope pore protein 37, chloroplastic
TCONS_00035558	0.761922	1.1895	1.0972	BGIOSGA034511	XP_010238795.1E3 ubiquitin-protein ligase RNF181
TCONS_00033644	0.762068	1.18932	-0.0880862	BGIOSGA035427	NP_001147401.1tubulin alpha-6 chain
TCONS_00021009	0.762311	1.00573	0.747392	BGIOSGA016957	XP_006647038.1 26S protease regulatory subunit S10B homolog B
TCONS_00008763	0.762353	1.41138	2.01811	BGIOSGA027747	XP_006649020.1 probable calcium-binding protein CML20
TCONS_00031832	0.762491	1.06329	0.825109	BGIOSGA014570	XP_022685460.1uncharacterized protein LOC101771470, partial
TCONS_00011118	0.762761	-1.7184	-1.30486	BGIOSGA026653	XP_002466308.2uncharacterized protein LOC8081268
TCONS_00020821	0.763177	1.58333	1.07708	BGIOSGA023031	XP_015694351.1 putative E3 ubiquitin-protein ligase UBR7
TCONS_00036814	0.763447	0.0583291	-0.988396	BGIOSGA036500	XP_006663877.1 psbP domain-containing protein 4, chloroplastic
TCONS_00000500	0.76352	-0.812419	-1.98485	BGIOSGA021615	NP_001105641.1aquaporin TIP4-4
TCONS_00005451	0.76352	-0.0754538	1.26307	BGIOSGA037257	XP_006646894.1 ribulose bisphosphate carboxylase small chain clone 512-like
TCONS_00014293	0.763873	1.35708	1.01426	BGIOSGA016448	XP_006652306.1 psbP domain-containing protein 2, chloroplastic
TCONS_00007205	0.764099	-2.39808	-1.98631	BGIOSGA005298	XP_008645149.1RING-H2 finger protein ATL79
TCONS_00027353	0.764425	-0.0359122	0.375435	#N/A	#N/A
TCONS_00027325	0.764979	2.02503	0.485352	BGIOSGA020655	XP_015696206.1 external alternative NAD(P)H-ubiquinone oxidoreductase B2, mitochondrial-like
TCONS_00022883	0.765215	0.96751	1.86044	BGIOSGA017152	XP_012702682.2putative disease resistance protein At3g14460
TCONS_00008249	0.765408	1.44161	1.06928	BGIOSGA005673	XP_002454066.1protein TsetseEP
TCONS_00029062	0.76548	0.120162	-0.143694	BGIOSGA030808	XP_015696359.1 glutamate receptor 2.9-like
TCONS_00034286	0.765515	0.867099	1.226	BGIOSGA034508	XP_006664328.1 crocetin glucosyltransferase, chloroplastic-like
TCONS_00035955	0.765587	1.30052	0.331863	BGIOSGA007026	XP_004962556.1E3 ubiquitin-protein ligase ATL6
TCONS_00013354	0.765587	1.23235	-1.31199	BGIOSGA009629	NP_001307021.1uncharacterized LOC100273825
TCONS_00011217	0.765735	1.20307	-0.261048	BGIOSGA030651	XP_006651955.1 SCAR-like protein 1 isoform X1
TCONS_00018626	0.76596	1.60149	1.17205	BGIOSGA018808	XP_006654042.1 T-complex protein 1 subunit zeta 1
TCONS_00006840	0.765962	0.582295	0.856267	BGIOSGA000228	XP_015689554.1 probable histidine kinase 6
TCONS_00009417	0.766214	1.2315	1.11825	BGIOSGA011970	XP_015690254.1 mitochondrial ubiquitin ligase activator of NFKB 1
TCONS_00034139	0.766256	0.41741	0.760321	BGIOSGA018329	XP_006665213.1 double-stranded RNA-binding protein 7-like
TCONS_00016984	0.766486	1.10091	1.71205	BGIOSGA019032	XP_006654903.1 E3 ubiquitin-protein ligase RING1-like
TCONS_00010753	0.766926	1.68587	1.707	BGIOSGA031311	XP_002466626.1uncharacterized protein LOC8062954
TCONS_00015354	0.766998	0.338007	-2.63447	BGIOSGA015620	XP_024311606.1uncharacterized protein LOC100820859
TCONS_00022905	0.767266	1.26094	-2.57925	BGIOSGA004173	XP_006656487.1 ras-related protein RABA3-like
TCONS_00024105	0.767394	0.791561	0.12969	BGIOSGA021298	XP_015695289.1 peroxisomal (S)-2-hydroxy-acid oxidase GLO4-like
TCONS_00007624	0.767453	4.35606	-3.16141	BGIOSGA033699	XP_015688519.1 cytochrome P450 711A1-like
TCONS_00001437	0.767459	2.1827	0.590183	BGIOSGA010658	XP_006644414.2 myb-related protein 340-like
TCONS_00034948	0.767664	-0.474358	-0.250908	BGIOSGA022712	XP_006660464.1 RPM1-interacting protein 4-like
TCONS_00006702	0.768147	-0.236754	0.551641	BGIOSGA008869	XP_002454434.1uncharacterized protein LOC8073172
TCONS_00028464	0.768222	0.753709	0.120573	BGIOSGA025459	XP_006659694.1 vesicle-associated membrane protein 727
TCONS_00007690	0.768223	0.67133	1.28248	BGIOSGA006799	XP_021308676.1defective in cullin neddylation protein 1 isoform X2
TCONS_00003579	0.768496	3.11024	-0.188018	BGIOSGA000766	XP_004968765.1probable glutathione S-transferase GSTF1
TCONS_00026301	0.768784	1.55025	0.00190107	#N/A	#N/A
TCONS_00014940	0.768832	#NA	0.586744	BGIOSGA002233	XP_002447096.2myristoylated alanine-rich C-kinase substrate
TCONS_00003150	0.76901	1.45351	1.00591	BGIOSGA013743	XP_006656446.1 60S acidic ribosomal protein P3
TCONS_00014818	0.769018	1.47491	0.685815	BGIOSGA031846	XP_006652695.1 expansin-A10
TCONS_00019617	0.769044	1.37952	1.68923	BGIOSGA017740	XP_006654632.1 carnosine N-methyltransferase-like isoform X1
TCONS_00025503	0.76924	0.817874	1.32269	BGIOSGA019138	XP_002457708.1peroxidase 5
TCONS_00031529	0.769283	-3.40457	-1.99934	BGIOSGA004738	XP_004983834.1transcription repressor OFP8
TCONS_00019526	0.769662	0.640019	1.21356	BGIOSGA031326	XP_006654558.1 protein KRTCAP2 homolog

TCONS_00017882	0.769728	0.710341	1.45898	BGIOSGA010397	XP_006655359.2 G-box-binding factor 4-like
TCONS_00028674	0.769742	-0.308454	-0.623559	BGIOSGA030526	XP_006660490.1 rhodanese-like domain-containing protein 9, chloroplastic
TCONS_00010634	0.770023	-0.183116	0.2212	BGIOSGA013256	XP_006655024.2PREDICTED: uncharacterized protein LOC102699508
TCONS_00010264	0.770299	0.339425	0.525986	BGIOSGA005822	XP_002467721.1uncharacterized protein LOC8080433
TCONS_00004186	0.770525	0.642023	1.3541	BGIOSGA001012	XP_015688245.1 glycosylphosphatidylinositol anchor attachment 1 protein isoform X1
TCONS_00000780	0.770672	0.9156	1.45662	BGIOSGA003376	XP_003567617.1caffeoylshikimate esterase
TCONS_00018969	0.771437	0.365295	0.695726	BGIOSGA013855	XP_006654297.2 bifunctional epoxide hydrolase 2-like
TCONS_00011881	0.771515	0.737703	0.354713	BGIOSGA032570	XP_006649722.1 26S proteasome non-ATPase regulatory subunit 4 homolog
TCONS_00034361	0.771575	2.21702	1.57991	BGIOSGA034446	XP_006663236.1PREDICTED: uncharacterized protein LOC102714508
TCONS_00027337	0.771728	0.914267	0.579526	BGIOSGA005913	XP_006659824.1 putative lipase YOR059C
TCONS_00036669	0.771812	0.292673	1.74924	BGIOSGA036642	XP_022684373.1meiotic recombination protein DMC1 homolog isoform X1
TCONS_00031694	0.771846	1.00469	1.50678	BGIOSGA037906	XP_006662115.2 ATP-dependent Clp protease proteolytic subunit 4, chloroplastic
TCONS_00016470	0.771894	-0.729798	-2.00585	BGIOSGA004933	XP_006653701.1 protein DMR6-LIKE OXYGENASE 1-like
TCONS_00008669	0.771954	-0.0156279	-0.0991055	BGIOSGA026130	XP_003570207.2uncharacterized protein LOC100827381
TCONS_00003409	0.771993	0.872113	0.382949	BGIOSGA002734	XP_006644115.1PREDICTED: uncharacterized protein LOC102703625
TCONS_00032994	0.772051	2.24059	1.81394	BGIOSGA009203	XP_006663223.1 mRNA-decapping enzyme-like protein
TCONS_00017069	0.772101	-0.528306	-0.860259	#N/A	#N/A
TCONS_00008810	0.772217	-0.600922	0.578313	BGIOSGA012641	XP_006647893.1PREDICTED: uncharacterized protein At5g39865-like
TCONS_00017775	0.772537	-0.194555	0.18159	BGIOSGA004111	XP_006654372.1 GDSL esterase/lipase At1g54790 isoform X1
TCONS_00009822	0.772706	1.14692	1.67021	BGIOSGA023663	XP_006649892.1 arogenate dehydratase/prephenate dehydratase 2, chloroplastic-like
TCONS_00016401	0.772822	1.42746	0.354422	BGIOSGA022423	XP_010933314.1 cactin
TCONS_00027193	0.772836	-0.631448	0.354387	BGIOSGA006095	XP_014756554.1PI-PLC X domain-containing protein At5g67130
TCONS_00021109	0.773076	1.31311	0.254257	BGIOSGA000159	XP_006648392.1 probable glycosyltransferase At5g03795
TCONS_00002942	0.773187	1.6349	2.4828	BGIOSGA013141	XP_021316975.1ankyrin repeat, PH and SEC7 domain containing protein secG
TCONS_00000327	0.773896	0.267684	1.05958	BGIOSGA033436	XP_004968446.1alkane hydroxylase MAH1
TCONS_00002044	0.773994	0.534594	-0.315402	BGIOSGA008868	XP_004304633.1 ADP-ribosylation factor 1 isoform X1
TCONS_00018499	0.774228	0.704671	1.1583	BGIOSGA018934	XP_006653974.1 RNA-binding protein 34
TCONS_00028624	0.774297	-0.230321	-0.52637	BGIOSGA029305	XP_006660467.1PREDICTED: uncharacterized protein LOC102713845
TCONS_00033920	0.77444	#NA	-1.69022	BGIOSGA004808	XP_015697928.1 putative laccase-19
TCONS_00016695	0.774547	0.825833	0.703794	BGIOSGA014268	XP_006652886.2 MICOS complex subunit MIC60
TCONS_00000503	0.775013	1.31231	1.35088	BGIOSGA003076	XP_006643955.1 VHS domain-containing protein At3g16270
TCONS_00036803	0.775013	2.23207	2.30837	BGIOSGA026941	XP_002442933.1dihydrolipoyllysine-residue acetyltransferase component 5 of pyruvate dehydrogenase complex, chloroplastic
TCONS_00013390	0.775128	1.74334	2.14143	BGIOSGA009594	XP_006651940.1 dentin sialophosphoprotein
TCONS_00017674	0.775311	1.6753	0.94918	BGIOSGA024304	XP_015693240.1 protein QUIRKY-like
TCONS_00007366	0.775349	0.26586	1.08995	BGIOSGA007102	XP_022684508.1tyrosine-sulfated glycopeptide receptor 1-like
TCONS_00014735	0.777345	2.19413	0.7627	BGIOSGA003074	XP_015691897.1 probable aquaporin PIP1-2 isoform X1
TCONS_00016547	0.778033	-0.205886	0.737955	BGIOSGA014420	XP_006653734.1PREDICTED: uncharacterized protein LOC102705815
TCONS_00015497	0.778036	#NA	-1.04105	BGIOSGA008776	XP_006652133.1 E3 ubiquitin-protein ligase RHA2A-like
TCONS_00009932	0.778087	-0.922979	-1.26907	BGIOSGA006435	XP_009113385.1 NHL repeat-containing protein 2 isoform X2
TCONS_00011533	0.77876	-0.188997	0.869373	BGIOSGA011194	XP_006651018.2 protein CHAPERONE-LIKE PROTEIN OF POR1, chloroplastic
TCONS_00006118	0.7789	0.668084	0.771887	BGIOSGA028788	XP_024316982.1exocyst complex component EXO70B1
TCONS_00028423	0.779618	-4.78535	-1.63081	BGIOSGA028566	XP_015695690.1 BAG family molecular chaperone regulator 1
TCONS_00019016	0.780512	0.740439	0.890129	BGIOSGA005123	XP_006654238.1 cytochrome c1-2, heme protein, mitochondrial-like isoform X2
TCONS_00033630	0.780824	0.63516	0.437156	BGIOSGA033267	XP_015697688.1 transmembrane protein 87B
TCONS_00036945	0.780956	0.168255	1.01224	BGIOSGA018541	XP_015688074.1 chaperone protein dnaJ 50
TCONS_00001858	0.781144	0.279255	0.0674709	BGIOSGA007615	XP_015691811.1 soluble scavenger receptor cysteine-rich domain-containing protein SSC5D
TCONS_00009256	0.781291	1.0135	1.58543	BGIOSGA011786	XP_015689755.1 ATP phosphoribosyltransferase, chloroplastic
TCONS_00025936	0.781297	0.34599	0.628356	BGIOSGA027632	XP_004972813.1vesicle-associated protein 1-3
TCONS_00019176	0.781804	0.523798	1.17501	BGIOSGA000165	XP_006655251.1 succinate dehydrogenase subunit 4, mitochondrial-like isoform X1
TCONS_00011528	0.78215	0.964386	0.195699	BGIOSGA011502	XP_006649352.1 glutathione S-transferase F11-like
TCONS_00023446	0.782771	0.419089	1.06584	BGIOSGA025477	XP_015694839.1 BTB/POZ domain-containing protein At4g08455
TCONS_00000182	0.782827	0.635386	1.22319	BGIOSGA002755	XP_006643738.1 EKC/KEOPS complex subunit TPRKB
TCONS_00001155	0.783223	0.174551	-0.0117162	BGIOSGA037078	NP_001151680.1protein transport protein Sec61 beta subunit

TCONS_00031233	0.783315	-0.344245	-0.338271	BGIOSGA033052	XP_006661803.1 ubiquitin-conjugating enzyme E2 4
TCONS_00002340	0.783493	-1.47251	-1.30413	BGIOSGA014722	XP_006645170.1 beta-glucosidase 4
TCONS_00019422	0.783654	0.0698101	0.884447	BGIOSGA004801	XP_006654501.1 delta-1-pyrroline-5-carboxylate synthase
TCONS_00013554	0.783675	1.27903	1.37388	BGIOSGA018233	XP_006650912.1PREDICTED: uncharacterized protein LOC102718067
TCONS_00031407	0.783707	0.991233	1.91934	BGIOSGA005008	XP_003574136.1E3 ubiquitin-protein ligase AIRP2 isoform X1
TCONS_00022937	0.784011	0.640858	0.00325955	BGIOSGA021611	XP_021305305.1ATP-dependent zinc metalloprotease FTSH 1, chloroplastic
TCONS_00018859	0.784852	0.539993	0.798308	BGIOSGA000824	XP_006664910.1 coiled-coil domain-containing protein 25
TCONS_00025871	0.785093	0.494079	1.665	BGIOSGA033388	XP_006659800.2 plant cysteine oxidase 3-like
TCONS_00013402	0.786191	0.326556	-0.166966	BGIOSGA002607	XP_010229598.3NAC domain-containing protein 67
TCONS_00033084	0.786496	0.673719	-0.0909943	BGIOSGA023525	XP_006662783.1 nifU-like protein 1, chloroplastic
TCONS_00001505	0.786513	0.0758004	0.564497	BGIOSGA002105	XP_006644467.1 V-type proton ATPase subunit E-like
TCONS_00011586	0.786742	1.50125	1.56721	BGIOSGA021125	XP_015690915.1 RNA-binding protein 39-like isoform X2
TCONS_00010106	0.786946	0.111	0.368562	BGIOSGA026262	XP_006650101.1 putative MO25-like protein At4g17270
TCONS_00028047	0.787197	-1.23382	-1.92557	BGIOSGA021491	XP_015695938.1 protein CURVATURE THYLAKOID 1A, chloroplastic-like
TCONS_00019829	0.787804	0.393957	0.833788	BGIOSGA029488	XP_004953250.1uncharacterized protein LOC101783675
TCONS_00025498	0.788188	-2.06273	0.330267	BGIOSGA012881	XP_006658777.1 protein LONGIFOLIA 2-like
TCONS_00030270	0.788538	-0.713232	0.190704	BGIOSGA026689	XP_006660776.1PREDICTED: uncharacterized protein LOC102712842
TCONS_00021890	0.788542	0.68435	0.840398	BGIOSGA025748	XP_004966817.1transcription termination factor MTERF2, chloroplastic
TCONS_00006997	0.789153	2.20248	0.825574	BGIOSGA002960	XP_015689674.1 protein CYPRO4-like
TCONS_00007090	0.789629	0.449853	1.0472	BGIOSGA009270	XP_015689525.1 F-box protein PP2-B1-like
TCONS_00021290	0.78978	1.26757	1.19041	BGIOSGA023520	XP_006656412.1 cytoplasmic 60S subunit biogenesis factor REI1 homolog 1
TCONS_00018245	0.789966	1.72591	2.47225	BGIOSGA009184	XP_015693381.1 FHA domain-containing protein DDL
TCONS_00001528	0.790145	0.734404	0.313831	BGIOSGA017549	XP_006644478.1PREDICTED: uncharacterized protein LOC102719055
TCONS_00021576	0.790257	0.208304	0.947161	BGIOSGA021969	XP_006655772.2 tRNA wybutosine-synthesizing protein 4 isoform X1
TCONS_00008659	0.790273	0.698769	0.667586	BGIOSGA021239	XP_006647764.1 U-box domain-containing protein 5-like
TCONS_00023151	0.790314	0.352608	0.604228	BGIOSGA025188	XP_006657464.1PREDICTED: uncharacterized protein LOC102712645
TCONS_00012973	0.790678	-0.713987	1.02669	#N/A	#N/A
TCONS_00026200	0.790759	-0.0994422	-0.846077	BGIOSGA028270	XP_006659239.2 NADPH--cytochrome P450 reductase-like
TCONS_00003014	0.790989	-4.34437	-1.82483	BGIOSGA011837	XP_002455008.1NDR1/HIN1-like protein 6
TCONS_00033518	0.791043	-2.05178	0.157552	BGIOSGA025077	XP_015697765.1 11-beta-hydroxysteroid dehydrogenase 1B-like
TCONS_00010071	0.791752	-0.917543	-0.122218	BGIOSGA000386	XP_008660155.1inactive beta-amylase 9
TCONS_00004847	0.792006	0.12121	-0.497549	BGIOSGA000372	NP_001338755.1uncharacterized protein LOC100280094
TCONS_00004817	0.792178	0.89284	0.743424	BGIOSGA000399	XP_004970761.1SUN domain-containing protein 4
TCONS_00001517	0.792487	-0.0932798	0.41586	BGIOSGA004148	XP_002456086.1uncharacterized protein LOC8060135
TCONS_00031678	0.792809	1.33516	0.718129	BGIOSGA008261	XP_006662102.1 cinnamoyl-CoA reductase 1-like
TCONS_00005354	0.793259	-0.362111	0.939592	BGIOSGA007465	XP_006646835.1 probable ATP synthase 24 kDa subunit, mitochondrial
TCONS_00004675	0.793435	1.6135	0.583849	BGIOSGA005701	XP_006644967.1 nuclear transcription factor Y subunit B-2
TCONS_00017204	0.793468	1.52643	0.90255	BGIOSGA019247	XP_006654089.1PREDICTED: uncharacterized protein LOC102705342
TCONS_00023940	0.793851	-0.441924	0.547019	BGIOSGA029832	XP_006657863.1 zinc finger AN1 and C2H2 domain-containing stress-associated protein 16
TCONS_00021490	0.793958	1.35172	1.47324	BGIOSGA000430	XP_006656571.2 superoxide dismutase [Fe] 1, chloroplastic-like
TCONS_00020118	0.794594	-3.2152	0.508776	BGIOSGA013343	XP_015694380.1 GDSL esterase/lipase APG
TCONS_00001074	0.794717	0.319915	0.6926	BGIOSGA003691	XP_006644249.1 protein usf
TCONS_00028067	0.794809	0.843424	1.21606	BGIOSGA015096	XP_004973533.1zinc-finger homeodomain protein 10
TCONS_00003619	0.794944	-0.934314	1.90786	BGIOSGA001263	XP_015694204.1 cytochrome P450 734A6
TCONS_00028652	0.795713	0.74751	0.0617593	BGIOSGA036735	XP_010237994.1vacuolar protein sorting-associated protein 32 homolog 2
TCONS_00036901	0.795815	-0.41934	-0.159544	BGIOSGA014659	XP_015697415.1 LIM domain-containing protein PLIM2b-like
TCONS_00032853	0.796449	0.378032	0.326588	BGIOSGA034671	XP_006662663.2PREDICTED: uncharacterized protein LOC102720274 isoform X1
TCONS_00005863	0.796565	1.02439	0.862131	BGIOSGA007996	XP_015689034.1 ribosomal RNA small subunit methyltransferase nep-1
TCONS_00021605	0.797112	-0.0063468	-0.819476	BGIOSGA021924	XP_015693876.1PREDICTED: uncharacterized protein LOC102717905, partial
TCONS_00015824	0.7974	2.01528	0.937359	BGIOSGA025454	XP_006652238.1 calnexin homolog
TCONS_00002395	0.79813	0.542939	-0.229987	BGIOSGA005031	NP_001136449.1Thioredoxin F-type chloroplastic
TCONS_00034982	0.798283	0.321387	1.31489	BGIOSGA033869	XP_015697770.1 ATP-dependent DNA helicase At3g02060, chloroplastic
TCONS_00014225	0.798317	0.618415	-0.549217	BGIOSGA004097	XP_002446447.1protein FAM210B isoform X2
TCONS_00023273	0.798331	1.68214	2.49552	BGIOSGA019907	XP_003557437.1tRNA ligase 1

TCONS_00010723	0.798609	0.44949	1.44677	BGIOSGA013347	XP_006650417.1 uncharacterized tRNA/rRNA methyltransferase YsgA
TCONS_00031876	0.798784	0.0209929	-0.75564	BGIOSGA032257	XP_021901068.1uncharacterized protein LOC110816989
TCONS_00004431	0.798944	0.59619	0.273243	BGIOSGA015194	XP_006644736.1 phosphatidate cytidylyltransferase 1-like
TCONS_00021582	0.799151	-0.712269	-1.05256	BGIOSGA006914	XP_015694392.1 uncharacterized RNA-binding protein C17H9.04c-like isoform X1
TCONS_00030085	0.799527	0.837808	1.24606	#N/A	#N/A
TCONS_00011713	0.799586	0.570417	0.991129	BGIOSGA011297	XP_006649539.1 5'-3' exoribonuclease
TCONS_00026002	0.799844	-0.15339	-0.37208	BGIOSGA026166	XP_004972723.1phenolic glucoside malonyltransferase 1
TCONS_00003274	0.799875	0.471974	0.584271	BGIOSGA005610	XP_015688290.1 random slug protein 5-like isoform X3
TCONS_00003739	0.800005	-1.23264	0.570332	BGIOSGA008526	XP_004968878.1uncharacterized protein LOC101786743
TCONS_00002356	0.80004	0.510287	0.59665	BGIOSGA021712	XP_006646573.2 putative polyketide hydroxylase
TCONS_00010807	0.800067	-0.996914	-0.575	BGIOSGA014097	XP_004982006.1zinc finger protein CONSTANS-LIKE 16
TCONS_00003378	0.800132	0.863808	0.493329	BGIOSGA024808	XP_015688327.1 protein BRASSINOSTEROID INSENSITIVE 1-like
TCONS_00022946	0.800319	1.18997	0.938774	BGIOSGA007885	XP_006657366.1 callose synthase 3-like
TCONS_00030408	0.800448	-0.724305	-0.277545	BGIOSGA029393	XP_015696764.1PREDICTED: uncharacterized protein LOC102708648
TCONS_00013251	0.80071	0.399328	0.173267	BGIOSGA009737	XP_006651861.1 NADH-ubiquinone oxidoreductase subunit 8-like
TCONS_00019730	0.800996	-0.0735829	0.00276887	BGIOSGA037952	XP_006654740.1 DNA-binding protein DDB_G0278111
TCONS_00012757	0.801197	0.928158	0.670517	BGIOSGA010205	XP_004982381.1probable prefoldin subunit 4
TCONS_00024740	0.801456	-0.330591	0.684226	BGIOSGA024636	XP_015694818.1 5'-nucleotidase SurE
TCONS_00016732	0.801479	0.390876	1.48786	BGIOSGA036996	XP_006645047.1 protein BUD31 homolog 1
TCONS_00014787	0.801597	0.409433	0.657395	BGIOSGA016959	XP_006652671.1 xyloglucan galactosyltransferase KATAMARI1 homolog
TCONS_00008285	0.80176	0.85989	0.989567	BGIOSGA006145	XP_006648796.1PREDICTED: uncharacterized protein LOC102718342 isoform X1
TCONS_00029417	0.801805	1.3988	1.12547	BGIOSGA011334	XP_006659664.1 proteasome subunit alpha type-7-A
TCONS_00012814	0.802229	0.0253107	0.360377	BGIOSGA003964	XP_006663280.1 pentatricopeptide repeat-containing protein At4g18520
TCONS_00011730	0.802403	-1.32552	-0.718293	BGIOSGA006944	XP_003558588.1plant-specific TFIIB-related protein 1
TCONS_00010733	0.802709	0.113518	-0.872875	BGIOSGA029447	XP_006651702.1 calcium-dependent protein kinase 10-like
TCONS_00000286	0.803194	1.89673	0.796994	BGIOSGA002862	XP_004968344.1universal stress protein PHOS32
TCONS_00002911	0.803262	-1.50698	0.336037	BGIOSGA023913	XP_004968331.1uncharacterized protein LOC101759345
TCONS_00008089	0.803301	0.944708	0.364433	BGIOSGA016315	XP_004975464.1cytochrome b-c1 complex subunit Rieske, mitochondrial
TCONS_00010602	0.803401	-2.39781	-0.763352	BGIOSGA014650	XP_006650352.1 protein YABBY 2
TCONS_00034879	0.80385	-0.438742	0.880428	BGIOSGA033982	XP_015698151.1PREDICTED: uncharacterized protein LOC102715344
TCONS_00029652	0.803933	0.911176	1.09439	BGIOSGA030169	XP_006660447.1 FRIGIDA-like protein 3
TCONS_00034121	0.803944	-0.480149	-0.137575	BGIOSGA036808	XP_006662647.2 probable phytol kinase 2, chloroplastic
TCONS_00028130	0.804352	-0.518205	1.47379	BGIOSGA026858	XP_006659478.2 putative F-box/LRR-repeat protein At3g18150
TCONS_00018667	0.804459	-4.31584	-1.91486	BGIOSGA002993	XP_006655015.1 GDSL esterase/lipase At1g09390-like
TCONS_00028447	0.804516	0.80381	-1.16865	BGIOSGA026515	XP_015695713.1PREDICTED: uncharacterized protein ycf39, partial
TCONS_00027960	0.804529	-0.51313	-1.00258	BGIOSGA004933	XP_006652056.1 DIBOA-glucoside dioxygenase BX6-like
TCONS_00018270	0.804694	1.21833	0.47293	BGIOSGA001009	XP_006655572.1 probable histone chaperone ASF1A
TCONS_00012342	0.804718	-1.71761	-3.98154	BGIOSGA015937	XP_006664886.1PREDICTED: uncharacterized protein LOC102708677
TCONS_00003466	0.804772	-0.994304	-0.215197	BGIOSGA001754	XP_022682343.1F-box protein SKIP23-like
TCONS_00009973	0.804794	0.697771	0.383751	BGIOSGA023437	XP_006650010.1 14 kDa zinc-binding protein isoform X1
TCONS_00007380	0.805164	0.911338	0.443109	BGIOSGA007096	XP_006646903.1 single-stranded DNA-binding protein WHY2, mitochondrial
TCONS_00030442	0.805415	-0.34385	0.0950723	BGIOSGA006546	XP_006660906.1 synaptotagmin-1-like
TCONS_00032351	0.805465	-0.243564	1.09645	BGIOSGA030655	XP_006662410.2 plant intracellular Ras-group-related LRR protein 8
TCONS_00023148	0.805498	0.418942	0.300991	BGIOSGA018909	XP_004955496.1uncharacterized protein At5g39865
TCONS_00030069	0.805544	1.38718	0.586279	BGIOSGA028416	XP_006660638.2 probable monogalactosyldiacylglycerol synthase 1, chloroplastic
TCONS_00021571	0.806216	0.959408	2.19482	BGIOSGA021975	XP_002437788.1peptide methionine sulfoxide reductase A5 isoform X1
TCONS_00019647	0.806302	2.31977	0.440175	BGIOSGA037278	XP_015692667.1 receptor-like protein kinase 5
TCONS_00013020	0.806688	-1.39341	1.20758	BGIOSGA003613	XP_006651730.2 beta-glucosidase 7
TCONS_00026570	0.806696	0.436238	1.40154	BGIOSGA028632	XP_024318248.1pentatricopeptide repeat-containing protein At5g12100, mitochondrial isoform X1
TCONS_00029708	0.80706	1.67179	1.31126	BGIOSGA020692	XP_003577911.1sufE-like protein 1, chloroplastic/mitochondrial
TCONS_00010648	0.807235	0.98974	1.74157	BGIOSGA013267	XP_002466700.1uncharacterized protein LOC8056751 isoform X1
TCONS_00005846	0.807536	0.0627294	0.996348	BGIOSGA007970	XP_006647167.1 F-box protein At3g58530
TCONS_00019724	0.807669	0.847496	0.199967	BGIOSGA029517	XP_006654734.1 chaperone protein dnaJ 10-like

TCONS_00004857	0.808592	1.83092	1.89197	BGIOSGA000362	XP_006645144.2 peptide chain release factor 1
TCONS_00002114	0.809101	0.687568	0.906657	BGIOSGA014409	XP_006644957.1 dual specificity tyrosine-phosphorylation-regulated kinase 2-like isoform X1
TCONS_00014398	0.809176	0.92334	0.342253	BGIOSGA014531	XP_024312041.1protein FLOURY 1-like
TCONS_00029390	0.809334	0.645427	0.935303	BGIOSGA031143	XP_015696580.1 pentatricopeptide repeat-containing protein At4g01400, mitochondrial-like
TCONS_00010577	0.809428	1.36634	2.07616	BGIOSGA013062	XP_015690929.1 serine/threonine-protein kinase HT1-like
TCONS_00001264	0.809525	1.60455	1.29097	BGIOSGA036929	XP_004969063.1protein MLN51 homolog
TCONS_00015551	0.809576	-0.717448	0.538773	BGIOSGA029541	XP_015691391.1 translation initiation factor IF-2-like
TCONS_00017105	0.809615	#NA	1.04564	BGIOSGA031688	XP_006654025.1 transcription factor MYB46-like
TCONS_00018330	0.809896	1.70983	-0.117054	BGIOSGA001079	XP_009405654.1 iron-sulfur cluster assembly protein 1-like
TCONS_00002474	0.810017	1.07503	1.60251	BGIOSGA005118	XP_004971181.1mitochondrial thiamine diphosphate carrier 2 isoform X1
TCONS_00008814	0.810042	-0.361884	0.0937405	#N/A	#N/A
TCONS_00003235	0.810186	1.76648	0.615297	BGIOSGA033678	NP_001105262.2uncharacterized LOC542172
TCONS_00003315	0.810317	0.293964	1.30676	BGIOSGA001899	XP_006645764.1 histidine biosynthesis bifunctional protein hisIE, chloroplastic
TCONS_00024011	0.810317	2.07465	1.31883	BGIOSGA026047	XP_006657902.1 UPF0553 protein-like isoform X1
TCONS_00014977	0.810331	0.210636	-1.36776	BGIOSGA004829	XP_006653781.1 aminomethyltransferase, mitochondrial
TCONS_00000505	0.810835	0.820433	1.43471	BGIOSGA003078	XP_015699134.1 histidinol dehydrogenase, chloroplastic
TCONS_00013610	0.810966	0.708183	-0.341748	BGIOSGA037023	XP_004974991.1glyoxylate/hydroxypyruvate reductase HPR3
TCONS_00004696	0.811142	0.390487	0.305718	BGIOSGA019991	XP_004970524.1ubiquitin-conjugating enzyme E2 7 isoform X2
TCONS_00022390	0.811396	0.971773	1.10299	BGIOSGA007852	XP_006656149.2 aspartate aminotransferase, mitochondrial-like
TCONS_00008332	0.812284	1.47477	-0.121276	BGIOSGA006093	XP_006647487.1 4-hydroxy-3-methylbut-2-en-1-yl diphosphate synthase (ferredoxin), chloroplastic
TCONS_00028240	0.812629	0.846417	1.12986	BGIOSGA008949	XP_021320796.1transmembrane 9 superfamily member 12 isoform X3
TCONS_00022531	0.812984	1.30512	1.12461	BGIOSGA010548	XP_006657115.1 transcription factor GAMYB-like
TCONS_00026924	0.812984	1.49275	1.1835	BGIOSGA030091	XP_022683053.1protein EMSY-LIKE 3 isoform X2
TCONS_00008771	0.813108	1.13026	1.3569	BGIOSGA005663	XP_006647857.1 dihydropyrimidine dehydrogenase (NADP(+)), chloroplastic
TCONS_00019263	0.814335	0.9235	0.32016	BGIOSGA018087	XP_006654376.1 BI1-like protein
TCONS_00009859	0.81493	0.288162	1.12844	BGIOSGA003683	XP_006649920.2 ethylene-overproduction protein 1
TCONS_00036299	0.814993	-1.31785	-1.05375	BGIOSGA010009	XP_006664138.1PREDICTED: uncharacterized protein LOC102707081
TCONS_00037842	0.815286	1.17669	-0.144153	BGIOSGA023144	XP_022681911.1uncharacterized protein LOC101760546 isoform X2
TCONS_00020234	0.815974	0.30135	1.6615	BGIOSGA022425	XP_006655850.1PREDICTED: uncharacterized protein LOC102721559, partial
TCONS_00011397	0.816414	0.131701	0.264126	BGIOSGA011627	XP_015690785.1PREDICTED: uncharacterized protein LOC102722191
TCONS_00021204	0.816797	1.13076	1.04187	BGIOSGA031262	XP_006656345.1 ALA-interacting subunit 1-like
TCONS_00000608	0.816929	-0.65139	-0.0437631	BGIOSGA007201	XP_004967793.1protein CASPARIAN STRIP INTEGRITY FACTOR 1
TCONS_00013320	0.816929	0.821583	-0.378586	BGIOSGA009661	XP_015690815.1 zinc finger A20 and AN1 domain-containing stress-associated protein 6
TCONS_00011317	0.816973	0.452098	1.61721	BGIOSGA023755	XP_021302153.1uncharacterized protein LOC8064571 isoform X1
TCONS_00008195	0.817056	1.45199	1.33689	BGIOSGA007734	XP_006648751.1 MLO-like protein 1
TCONS_00020602	0.817087	1.20266	0.564017	#N/A	#N/A
TCONS_00030697	0.817303	-0.761558	0.163831	BGIOSGA032518	XP_015697194.1PREDICTED: uncharacterized protein LOC102717552 isoform X1
TCONS_00034690	0.817346	0.987682	0.616285	BGIOSGA009087	XP_015688363.1 pentatricopeptide repeat-containing protein At1g73400, mitochondrial-like
TCONS_00015866	0.817391	-3.00063	-1.15638	BGIOSGA014058	XP_006653383.2 protein YLS9-like
TCONS_00010877	0.817783	1.10292	1.39528	BGIOSGA024894	XP_006650532.1PREDICTED: uncharacterized protein LOC102701709
TCONS_00027141	0.818009	-0.418778	-1.79141	BGIOSGA037411	XP_006659693.1PREDICTED: uncharacterized protein LOC102721386
TCONS_00008670	0.818455	0.389518	0.0682159	BGIOSGA014973	XP_006647779.1 histidinol-phosphate aminotransferase, chloroplastic-like
TCONS_00028094	0.818718	2.47671	1.99305	BGIOSGA009171	XP_006660174.1 protein ecdysoneless homolog
TCONS_00027876	0.819193	0.219624	1.2173	BGIOSGA027127	XP_006650258.2 psbP-like protein 1, chloroplastic isoform X1
TCONS_00032230	0.820203	1.16953	0.114944	BGIOSGA006684	XP_003571791.2chlorophyllase-2, chloroplastic
TCONS_00014584	0.820281	0.285045	1.25962	BGIOSGA013996	XP_015692314.1 transcriptional corepressor LEUNIG_HOMOLOG isoform X2
TCONS_00017116	0.820537	0.846134	1.2273	BGIOSGA019166	XP_006654983.1 DNA replication complex GINS protein SLD5
TCONS_00000845	0.820677	0.890877	0.846432	BGIOSGA003434	XP_015693784.1 transcription initiation factor TFIID subunit 13
TCONS_00004811	0.820883	1.37791	1.77983	BGIOSGA000404	XP_006645096.1 5'-3' exonuclease
TCONS_00016214	0.820989	0.302643	0.328766	BGIOSGA014757	XP_024311971.1DNA mismatch repair protein MSH1, mitochondrial isoform X2
TCONS_00013315	0.821232	1.32441	1.27226	BGIOSGA028430	XP_015690871.1 25.3 kDa vesicle transport protein
TCONS_00037412	0.82144	1.15003	0.970459	BGIOSGA012913	XP_015698771.1 diacylglycerol kinase 1-like isoform X1
TCONS_00021904	0.821536	0.841313	0.724213	BGIOSGA000885	XP_006655932.1 HVA22-like protein a isoform X1
TCONS_00021656	0.82163	0.39045	-0.447449	BGIOSGA036312	XP_010025545.1 histone H3.2

TCONS_00013825	0.821705	0.667824	0.709202	BGIOSGA016001	XP_003581591.1prostaglandin E synthase 2
TCONS_00000862	0.821918	0.42529	0.000236683	BGIOSGA018778	XP_020227577.1importin subunit alpha-1a-like
TCONS_00028991	0.821928	0.00809624	0.384199	BGIOSGA030750	XP_006661228.1PREDICTED: uncharacterized protein LOC102704449
TCONS_00020979	0.822178	2.62694	1.58769	BGIOSGA021494	XP_015694331.1 3-ketoacyl-CoA synthase 11-like
TCONS_00020471	0.822346	1.6342	-2.17076	BGIOSGA027658	XP_006655982.1 myb-related protein 308-like
TCONS_00017461	0.822561	0.378674	0.41367	BGIOSGA037837	XP_010231753.1uncharacterized abhydrolase domain-containing protein DDB_G0269086
TCONS_00015208	0.822581	0.786475	1.03131	BGIOSGA017388	XP_006653903.1 nudix hydrolase 26, chloroplastic-like
TCONS_00024336	0.822775	-3.19575	0.397649	BGIOSGA010762	XP_006658115.1 phylloplanin-like
TCONS_00025084	0.822926	0.213869	0.581079	BGIOSGA024317	XP_006644184.1 peptidyl-prolyl cis-trans isomerase CYP21-4
TCONS_00007142	0.823064	-0.0810792	0.291441	BGIOSGA015836	XP_006648155.1 V-type proton ATPase subunit F
TCONS_00007459	0.823079	-1.25425	0.63477	BGIOSGA013348	XP_015688472.1 gamma-glutamyl peptidase 5-like
TCONS_00005568	0.823322	1.46755	0.705092	BGIOSGA007677	XP_006648384.1 prostatic spermine-binding protein-like isoform X1
TCONS_00023503	0.824341	1.00719	0.682258	BGIOSGA025543	XP_006658483.1 pentatricopeptide repeat-containing protein At1g71490
TCONS_00001080	0.825251	1.47954	1.15053	BGIOSGA003699	XP_006644295.2PREDICTED: uncharacterized protein LOC102712026
TCONS_00006186	0.825255	0.825489	0.709845	BGIOSGA003418	XP_006647331.1 neutral/alkaline invertase 3, chloroplastic
TCONS_00008474	0.825308	0.657827	0.136555	BGIOSGA031583	XP_006647598.1 vesicle-associated protein 1-2-like
TCONS_00014458	0.825557	0.554233	-0.487311	BGIOSGA021475	XP_006653528.1PREDICTED: uncharacterized protein LOC102709460, partial
TCONS_00013918	0.825792	-0.0580654	-1.53008	BGIOSGA016727	XP_006653251.1 type 1 phosphatases regulator YPI1
TCONS_00008175	0.826249	1.22718	0.989477	BGIOSGA020102	XP_004952813.2protein disulfide isomerase-like 1-2
TCONS_00001777	0.826318	1.26786	-0.160566	BGIOSGA026122	XP_006644671.1 reticulon-like protein B1
TCONS_00008699	0.82687	-2.58095	-0.374625	BGIOSGA031434	XP_006647794.1 protein NRT1/ PTR FAMILY 7.3-like isoform X1
TCONS_00009854	0.828171	-3.91136	-1.18836	BGIOSGA012406	XP_006649917.1 protein CHUP1, chloroplastic
TCONS_00021607	0.828425	2.7979	3.36315	BGIOSGA023145	XP_004964545.1uncharacterized protein LOC101767447
TCONS_00010864	0.828463	0.715092	0.0952628	BGIOSGA013485	XP_006650519.1 phosphatidylinositol transfer protein PDR17
TCONS_00007471	0.828555	0.974976	0.168143	BGIOSGA001181	XP_002453412.1proteasome subunit beta type-3
TCONS_00012035	0.8286	0.34503	0.0911213	BGIOSGA010972	XP_015690288.1PREDICTED: uncharacterized protein LOC102711054
TCONS_00013377	0.828789	1.26495	0.957522	BGIOSGA009607	XP_010923350.1 lipid phosphate phosphatase delta-like isoform X1
TCONS_00018333	0.828814	-2.11175	-1.08331	BGIOSGA004078	XP_006655602.1 putative cryptochrome DASH, mitochondrial
TCONS_00034320	0.829326	-0.618203	0.21111	BGIOSGA006319	XP_006662737.1 CEN-like protein 2
TCONS_00009847	0.829606	2.11843	1.79536	BGIOSGA012399	XP_006649911.2 mitochondrial succinate-fumarate transporter 1
TCONS_00006273	0.830001	0.192528	1.14975	BGIOSGA008431	XP_003575150.1NADPH-dependent pterin aldehyde reductase isoform X2
TCONS_00002959	0.830754	-0.0476524	-2.66029	BGIOSGA002257	XP_015688224.1 probable serine incorporator isoform X3
TCONS_00008501	0.830924	-0.299261	0.297052	BGIOSGA005927	XP_006647613.2 U6 snRNA phosphodiesterase
TCONS_00031607	0.831489	-0.114633	0.505847	BGIOSGA012183	XP_015697466.1PREDICTED: uncharacterized protein LOC102704739
TCONS_00021196	0.831629	-0.0954554	0.472326	BGIOSGA023415	XP_006656327.1 ubiquitin-conjugating enzyme E2 22
TCONS_00011729	0.831734	1.11263	0.956445	BGIOSGA011281	XP_006649561.1 30S ribosomal protein S10, chloroplastic
TCONS_00019581	0.83185	-2.20622	-1.79527	BGIOSGA027686	XP_006654606.1 NAD(P)H dehydrogenase (quinone) FQR1-like
TCONS_00004353	0.832146	0.378378	1.11087	BGIOSGA020051	XP_006646300.1 RING-H2 finger protein ATL67-like
TCONS_00018025	0.832205	1.06786	0.434674	BGIOSGA020072	XP_002467921.1uncharacterized protein LOC8083674
TCONS_00008782	0.83352	-1.60395	0.0748552	BGIOSGA022616	XP_002452577.1TORTIFOLIA1-like protein 3
TCONS_00033873	0.833709	1.66719	1.04636	BGIOSGA035095	XP_015698082.1 putative disease resistance RPP13-like protein 3
TCONS_00020222	0.833793	1.10954	1.1127	BGIOSGA028592	XP_015693468.1 probable beta-1,3-galactosyltransferase 10
TCONS_00017939	0.834247	0.112721	-0.801312	BGIOSGA035974	XP_006654511.1PREDICTED: uncharacterized protein LOC102712810
TCONS_00003820	0.834315	1.39271	-0.250753	BGIOSGA001396	XP_006644261.1 UPF0587 protein C1orf123
TCONS_00006750	0.835042	0.525801	0.466262	BGIOSGA008919	XP_015688849.1 short-chain dehydrogenase/reductase family 42E member 1
TCONS_00015058	0.835368	0.634553	-0.280919	BGIOSGA017453	XP_006652913.1 protein TSS-like
TCONS_00031699	0.83556	2.77959	1.06809	BGIOSGA008839	XP_006662126.1 serine carboxypeptidase-like 6
TCONS_00004492	0.835784	-0.949434	0.0645262	BGIOSGA020157	XP_006644788.1 photosystem II reaction center W protein, chloroplastic
TCONS_00023477	0.835946	0.343232	0.32371	BGIOSGA005008	NP_001152052.1RNA-binding protein
TCONS_00008755	0.836178	-0.0791997	0.108119	BGIOSGA011269	XP_002452602.1ADP-ribosylation factor-like protein 8c
TCONS_00022757	0.836439	0.431875	0.737515	BGIOSGA020698	XP_015693731.1PREDICTED: uncharacterized protein LOC107304379
TCONS_00009510	0.837029	0.645254	1.94661	BGIOSGA012059	XP_006649578.1PREDICTED: uncharacterized protein LOC102707381
TCONS_00027314	0.837232	1.10456	2.01068	BGIOSGA010992	XP_006659102.1 glycine-rich RNA-binding protein 4, mitochondrial-like
TCONS_00002447	0.837848	0.0831622	-0.0313049	BGIOSGA037382	XP_015692758.1 NAC domain-containing protein 83-like

TCONS_00003280	0.837874	0.654102	0.821885	BGIOSGA001935	XP_006644031.2 translin-associated protein X
TCONS_00027244	0.838333	1.73982	1.27594	BGIOSGA027759	XP_015696209.1 molybdate-anion transporter
TCONS_00035631	0.838368	-1.52831	-0.766094	BGIOSGA037053	XP_006664369.1 nuclear pore complex protein NUP98A
TCONS_00029210	0.838795	-0.84593	0.103659	BGIOSGA028882	XP_004957100.1uncharacterized protein LOC101761820
TCONS_00005231	0.839028	1.17707	-0.398215	BGIOSGA029843	XP_006646766.1 L-lactate dehydrogenase A
TCONS_00002306	0.839107	0.42191	0.19426	BGIOSGA004947	XP_004970838.12-C-methyl-D-erythritol 4-phosphate cytidylyltransferase, chloroplastic
TCONS_00021732	0.839146	0.0877047	0.260143	BGIOSGA005298	XP_021305816.1E3 ubiquitin-protein ligase RLIM-like isoform X2
TCONS_00027262	0.839203	1.78453	0.508243	BGIOSGA037061	XP_006659075.1 fructose-bisphosphate aldolase cytoplasmic isozyme-like
TCONS_00007782	0.839362	-0.0679826	0.0974699	BGIOSGA006702	XP_015688901.1 probable inactive nicotinamidase At3g16190
TCONS_00011746	0.839454	0.580073	0.673978	BGIOSGA011259	XP_006649585.1 vicilin-like seed storage protein At2g18540
TCONS_00011603	0.839651	-1.21227	0.574684	BGIOSGA028533	XP_006649433.1PREDICTED: uncharacterized protein LOC102713804
TCONS_00023103	0.840148	-0.101973	-1.05708	BGIOSGA025136	XP_006657437.1 uncharacterized oxidoreductase At1g06690, chloroplastic-like
TCONS_00032610	0.840191	2.45956	-0.151538	BGIOSGA011760	XP_021314843.1heavy metal-associated isoprenylated plant protein 39
TCONS_00033713	0.840289	0.81291	0.643054	BGIOSGA013227	XP_004979533.2myosin-binding protein 7
TCONS_00001610	0.84041	0.245278	0.583666	BGIOSGA023128	XP_006644549.1 phytochrome-associated serine/threonine-protein phosphatase
TCONS_00009059	0.840508	1.30645	0.0899712	BGIOSGA009821	XP_006649178.1 coumaroyl-CoA:anthocyanidin 3-O-glucoside-6''-O-coumaroyltransferase 2-like
TCONS_00003638	0.840744	-0.76733	0.380834	BGIOSGA001569	XP_010230919.1sec-independent protein translocase protein TATC, chloroplastic
TCONS_00019663	0.840841	1.0304	0.76722	BGIOSGA035541	XP_002441416.1anthocyanidin 5,3-O-glucosyltransferase
TCONS_00020722	0.840928	-0.44917	-1.8976	BGIOSGA028098	XP_004987307.2peptide methionine sulfoxide reductase B1, chloroplastic isoform X2
TCONS_00018195	0.841357	0.768882	0.154989	BGIOSGA004680	XP_006654703.1 UBA domain-containing protein 3-like isoform X1
TCONS_00021141	0.841594	-0.99532	0.277182	BGIOSGA019255	XP_006656299.1PREDICTED: uncharacterized protein LOC102713371
TCONS_00004020	0.84164	1.10744	0.673242	BGIOSGA000868	XP_006644392.1 mitochondrial import receptor subunit TOM7-1-like
TCONS_00002725	0.842574	1.14227	0.229368	BGIOSGA038682	XP_006643716.1 alanine--tRNA ligase isoform X1
TCONS_00009816	0.842653	-0.861716	-0.777185	BGIOSGA012369	XP_015690811.1PREDICTED: uncharacterized protein LOC107303928
TCONS_00002620	0.84274	1.04897	0.121634	BGIOSGA005260	XP_004971406.1eukaryotic translation initiation factor 4E-1
TCONS_00011584	0.842774	-0.19664	-0.941969	BGIOSGA011431	XP_015690311.1PREDICTED: uncharacterized protein LOC102708595
TCONS_00001225	0.843881	-0.897716	0.184951	BGIOSGA003853	XP_015694155.1PREDICTED: uncharacterized protein LOC102705538
TCONS_00026653	0.844557	1.06097	0.0997943	BGIOSGA018543	XP_022683074.1uncharacterized protein LOC101767369
TCONS_00026433	0.844638	1.92607	1.02407	BGIOSGA028504	XP_015695574.1PREDICTED: uncharacterized protein LOC102716896
TCONS_00009154	0.844844	0.207728	0.591182	BGIOSGA024311	XP_006649245.1 vacuolar protein sorting-associated protein 24 homolog 1-like
TCONS_00009967	0.844936	1.76604	1.19955	BGIOSGA012521	XP_006650003.1 membrane steroid-binding protein 2-like
TCONS_00028013	0.845007	0.101091	1.04463	#N/A	#N/A
TCONS_00015833	0.845162	1.32732	2.5848	BGIOSGA019877	XP_004975523.1uncharacterized protein LOC101781948
TCONS_00031661	0.845644	0.0229717	1.19787	BGIOSGA033473	XP_006662083.2PREDICTED: uncharacterized protein LOC102717376, partial
TCONS_00018659	0.845712	1.07942	1.07094	BGIOSGA011667	XP_006654071.1 tubulin gamma-2 chain
TCONS_00009530	0.84577	0.262266	0.54637	BGIOSGA032113	XP_006649596.2 CASP-like protein 5A1
TCONS_00006332	0.846474	0.767112	0.453583	BGIOSGA011837	XP_006647421.1 14-3-3-like protein GF14-E isoform X1
TCONS_00025170	0.84651	0.918337	0.345262	BGIOSGA014635	XP_006657745.1 SH3 domain-containing protein 3
TCONS_00013358	0.846585	1.59126	1.85304	BGIOSGA009626	XP_006651918.2 FAD synthetase, chloroplastic
TCONS_00009606	0.846808	2.06415	1.72695	BGIOSGA025247	XP_015690243.1 heat stress transcription factor A-9
TCONS_00014157	0.847766	1.04363	0.893018	BGIOSGA007688	XP_006652230.2PREDICTED: uncharacterized protein LOC102716118, partial
TCONS_00023005	0.847943	0.235577	0.289651	BGIOSGA025427	XP_021308281.1eukaryotic translation initiation factor 5A-like
TCONS_00021616	0.84813	0.606331	1.26158	BGIOSGA021903	XP_004964841.1uncharacterized protein LOC101785241
TCONS_00025366	0.848151	0.902306	0.834082	BGIOSGA025968	XP_006657857.1 HVA22-like protein k
TCONS_00036251	0.848688	1.53103	0.689241	BGIOSGA035708	XP_006664112.1 ATP-citrate synthase alpha chain protein 3
TCONS_00026582	0.850013	0.218903	0.0916885	BGIOSGA011326	XP_003574464.1ethylene-responsive transcription factor RAP2-13
TCONS_00029735	0.85082	-0.400346	-0.128436	BGIOSGA030086	XP_015696568.1 meiotic nuclear division protein 1 homolog
TCONS_00020082	0.851017	0.169127	0.554864	BGIOSGA035281	XP_015694264.1 transcription factor GTE1
TCONS_00010574	0.851091	-2.40937	0.213584	BGIOSGA035756	XP_006650326.1 putative receptor protein kinase CRINKLY4
TCONS_00024056	0.851318	1.79545	-0.00937129	BGIOSGA033209	XP_006658783.1 COBRA-like protein 1 isoform X1
TCONS_00024326	0.851324	1.46129	-0.046282	BGIOSGA018789	XP_006658100.1 stress-related protein
TCONS_00014996	0.852307	1.36169	1.79554	BGIOSGA037414	XP_006653790.1 pentatricopeptide repeat-containing protein At3g20730
TCONS_00019471	0.852398	0.430286	1.04685	BGIOSGA007233	XP_015693378.1 probable methyltransferase PMT23

TCONS_00031325	0.852537	1.05492	0.546293	BGIOSGA033138	XP_006661864.1 mitochondrial import inner membrane translocase subunit Tim16-like isoform X2
TCONS_00012792	0.853701	2.79829	2.49023	BGIOSGA031553	XP_006650332.1 histone-binding protein MSI1 homolog
TCONS_00008742	0.854337	1.92584	0.82907	BGIOSGA022681	XP_004953784.1H/ACA ribonucleoprotein complex subunit 2-like protein
TCONS_00029597	0.854547	0.473726	0.20149	BGIOSGA014003	XP_006660420.1 CBS domain-containing protein CBSX1, chloroplastic-like
TCONS_00036719	0.855321	-2.71774	1.39698	BGIOSGA032640	NP_001142857.1uncharacterized protein LOC100275251
TCONS_00008387	0.855393	-2.68032	-0.0358435	BGIOSGA014754	XP_006647520.1 CLIP-associated protein-like
TCONS_00012954	0.855845	1.20323	0.903	BGIOSGA009569	XP_015690307.1 protein SAAL1 isoform X2
TCONS_00010168	0.856035	-0.564733	-0.605289	#N/A	#N/A
TCONS_00024612	0.856442	-0.223015	0.0591306	BGIOSGA030919	XP_015694533.1 probable carboxylesterase 15
TCONS_00023339	0.856508	0.740268	1.01545	BGIOSGA026354	XP_006658087.2 DNA polymerase zeta catalytic subunit
TCONS_00006299	0.856629	1.74012	0.278181	BGIOSGA022347	XP_003575200.1uncharacterized protein LOC100831971
TCONS_00033438	0.857045	0.146093	0.597206	BGIOSGA030203	XP_020406526.1uncharacterized protein LOC109945126
TCONS_00008944	0.857048	1.43778	1.12116	BGIOSGA005463	XP_006649117.1 vacuolar amino acid transporter 1
TCONS_00002803	0.857231	-0.0334978	0.98958	BGIOSGA019909	XP_012461032.1PREDICTED: uncharacterized protein LOC105781011
TCONS_00004565	0.857454	1.40766	1.10848	BGIOSGA030255	XP_004985802.1uncharacterized protein LOC101763926
TCONS_00024597	0.857484	1.57385	1.09359	BGIOSGA028908	XP_006658290.1 33 kDa ribonucleoprotein, chloroplastic
TCONS_00009489	0.857743	1.44926	0.012338	BGIOSGA009834	XP_010230226.1cytochrome b561 and DOMON domain-containing protein At5g47530
TCONS_00016476	0.857776	1.9761	2.27327	BGIOSGA031487	XP_021318233.1helicase sen1
TCONS_00010632	0.85788	-1.39834	-0.368699	BGIOSGA004651	XP_006650378.1 tubulin beta-8 chain
TCONS_00028040	0.857945	1.44901	1.01183	#N/A	#N/A
TCONS_00020738	0.858011	0.465207	1.47554	BGIOSGA022931	XP_006656096.1 formate dehydrogenase 1, mitochondrial
TCONS_00004569	0.858373	1.91958	0.880945	BGIOSGA000645	XP_015692605.1PREDICTED: uncharacterized protein LOC102710070
TCONS_00022931	0.858526	0.612249	0.330317	BGIOSGA021106	XP_006656504.2PREDICTED: uncharacterized protein LOC102700628
TCONS_00001614	0.858603	1.26643	0.61644	BGIOSGA002737	XP_015693873.1 lipid phosphate phosphatase 2-like isoform X1
TCONS_00007101	0.858839	-1.51242	-0.000225142	BGIOSGA009282	XP_006648102.1PREDICTED: uncharacterized protein LOC102715735
TCONS_00008946	0.858861	1.36795	1.31255	BGIOSGA006069	XP_006648031.1 serine/arginine-rich splicing factor RSZ21 isoform X1
TCONS_00004735	0.859015	1.78238	0.82789	BGIOSGA008342	XP_006645025.2 putative ataxin-3 homolog
TCONS_00019937	0.860105	2.49448	1.63347	BGIOSGA003251	XP_015694423.1 E3 ubiquitin-protein ligase RING1-like isoform X4
TCONS_00003879	0.860136	0.257767	-0.0812517	BGIOSGA022935	XP_006646038.2 myosin-11-like
TCONS_00026745	0.860185	-0.807424	0.908425	BGIOSGA028803	XP_015695766.1 protein EMBRYO SAC DEVELOPMENT ARREST 3, chloroplastic
TCONS_00009680	0.860285	0.466615	0.785544	BGIOSGA015550	XP_006649736.1PREDICTED: uncharacterized protein LOC102705512
TCONS_00017331	0.860346	0.68281	1.19834	BGIOSGA026327	XP_006655097.1 pentatricopeptide repeat-containing protein At3g49170, chloroplastic
TCONS_00022987	0.8611	0.607474	-0.202733	BGIOSGA013594	XP_006658212.2 two pore potassium channel b-like
TCONS_00018663	0.861149	-0.967859	1.13989	BGIOSGA018772	NP_001136908.14Fe-4S ferredoxin, iron-sulfur binding protein
TCONS_00008667	0.861325	1.58151	2.06014	BGIOSGA005763	XP_006647770.1 universal stress protein A-like protein
TCONS_00004213	0.861973	0.452683	1.28304	BGIOSGA000989	XP_006644550.1PREDICTED: uncharacterized protein LOC102716543 isoform X1
TCONS_00024580	0.862129	#NA	0.884236	BGIOSGA024812	XP_004955458.1uncharacterized protein LOC101758435
TCONS_00014235	0.862435	2.78651	0.61722	BGIOSGA030245	XP_006648710.1 U-box domain-containing protein 27
TCONS_00031413	0.862531	1.26229	1.18812	BGIOSGA033224	XP_015697143.1 pentatricopeptide repeat-containing protein At2g20710, mitochondrial-like
TCONS_00011043	0.86256	0.521215	0.722703	BGIOSGA009742	XP_006650664.1 cytochrome c oxidase subunit 6a, mitochondrial
TCONS_00029687	0.863373	0.678252	-0.00179158	BGIOSGA026161	XP_002463491.1phosphoglycolate phosphatase 2 isoform X1
TCONS_00008547	0.863532	0.810472	0.271863	BGIOSGA005881	XP_006647658.1 transmembrane protein 208 homolog
TCONS_00034732	0.863692	0.403636	-1.44152	#N/A	#N/A
TCONS_00019444	0.864031	-3.82915	-3.70349	BGIOSGA012351	XP_006654519.1PREDICTED: uncharacterized protein LOC102715759
TCONS_00020229	0.864097	-0.804452	0.357211	BGIOSGA006303	XP_004964695.1pyrophosphate-energized vacuolar membrane proton pump
TCONS_00022012	0.864378	0.592919	0.843703	BGIOSGA029558	XP_002438190.1uncharacterized protein At1g66480
TCONS_00016158	0.86452	-1.44902	-1.0713	BGIOSGA023597	XP_006652456.1 B-box zinc finger protein 24-like
TCONS_00007883	0.864523	1.68584	1.25838	BGIOSGA027506	XP_010907847.1 putative F-box/LRR-repeat protein 23
TCONS_00014766	0.864639	1.27066	0.673129	BGIOSGA016942	XP_006652657.1 replication factor C subunit 4
TCONS_00020510	0.865037	1.33472	1.49833	BGIOSGA005691	XP_002436854.1H/ACA ribonucleoprotein complex subunit 2-like protein
TCONS_00029477	0.865048	1.24049	0.947084	BGIOSGA003204	XP_006661517.1 UTP--glucose-1-phosphate uridylyltransferase
TCONS_00033356	0.865191	0.395376	0.656481	BGIOSGA037198	XP_004979201.1adenylate kinase 4
TCONS_00012128	0.865483	1.45135	1.67103	BGIOSGA022686	XP_006651312.1 histone-lysine N-methyltransferase EZ3 isoform X1

TCONS_00035564	0.866028	1.00588	0.63931	BGIOSGA035837	XP_015697554.1 protein TRAUCO-like
TCONS_00036496	0.866591	0.982239	0.718509	BGIOSGA027604	XP_006664238.1 E3 ubiquitin-protein ligase RNF5-like
TCONS_00018176	0.866596	0.352017	0.0610564	BGIOSGA029943	XP_004961420.1ubiquitin domain-containing protein 2
TCONS_00004976	0.866971	0.882437	1.28206	BGIOSGA005533	XP_006646613.2 protein CPR-5-like
TCONS_00003450	0.867104	1.45343	0.847594	BGIOSGA001776	XP_006645831.1 suppressor of mec-8 and unc-52 protein homolog 1
TCONS_00017238	0.867375	1.86359	-0.370411	BGIOSGA019280	XP_006655056.1PREDICTED: uncharacterized protein LOC102710126
TCONS_00024389	0.867685	1.75868	1.30944	BGIOSGA014768	XP_015694848.1 nuclear poly(A) polymerase 3
TCONS_00028457	0.869294	1.34096	0.830061	BGIOSGA027053	XP_015695967.1 28 kDa ribonucleoprotein, chloroplastic-like
TCONS_00004848	0.869618	-0.072828	-0.498581	BGIOSGA000371	NP_001145171.1uncharacterized LOC100278407
TCONS_00006695	0.870079	1.08855	0.483587	BGIOSGA004569	XP_006647732.2 secretory carrier-associated membrane protein 5
TCONS_00001623	0.870227	1.86091	0.832973	BGIOSGA004257	XP_021312654.1uncharacterized protein LOC8063107 isoform X1
TCONS_00036956	0.870352	0.0984045	-0.798327	BGIOSGA011073	XP_006663942.1 isoflavone reductase homolog
TCONS_00022359	0.870382	0.893237	1.45233	BGIOSGA021113	XP_006657021.2 protein DJ-1 homolog A
TCONS_00031301	0.871924	-0.682371	0.380855	BGIOSGA036096	XP_006661852.1 cyanate hydratase
TCONS_00032731	0.872027	0.65765	0.979983	BGIOSGA017594	XP_015697447.1 dynamin-related protein 1E-like
TCONS_00004795	0.872485	-0.861284	-0.131461	BGIOSGA000421	XP_025983439.1AUGMIN subunit 7
TCONS_00014415	0.872585	1.27073	1.097	BGIOSGA017773	XP_021318694.1uncharacterized protein LOC8073555
TCONS_00002440	0.872652	0.813913	-0.129702	BGIOSGA005083	XP_004971106.150S ribosomal protein L27, chloroplastic
TCONS_00027966	0.873008	1.67263	-0.263957	BGIOSGA014455	XP_004979105.11-aminocyclopropane-1-carboxylate oxidase homolog 1
TCONS_00009113	0.873022	1.44498	-0.274068	BGIOSGA030319	XP_004966492.1GDSL esterase/lipase At1g71691
TCONS_00003594	0.873238	0.816119	0.321522	BGIOSGA019170	XP_006644185.1 probable sucrose-phosphatase 1
TCONS_00021159	0.873331	1.62678	1.14456	BGIOSGA030174	XP_006656314.2 DNA-directed RNA polymerase 3B, chloroplastic-like
TCONS_00030012	0.87343	1.90037	1.11969	BGIOSGA029793	XP_006661206.1 HMG-Y-related protein A-like
TCONS_00024604	0.873443	-0.822661	-1.27632	BGIOSGA024785	XP_006657461.1PREDICTED: uncharacterized protein YwkD
TCONS_00022046	0.873807	1.79525	0.960005	BGIOSGA022679	XP_015693544.1 protein trichome birefringence-like 19
TCONS_00010461	0.873845	-1.59515	0.00475241	BGIOSGA035064	XP_006664981.1 chlorophyll a-b binding protein, chloroplastic
TCONS_00006829	0.874267	1.72971	0.800912	BGIOSGA009003	XP_006649023.1 mitochondrial metalloendopeptidase OMA1
TCONS_00001895	0.874447	-1.8071	0.19661	BGIOSGA028790	XP_015692976.1 copper transporter 2-like
TCONS_00017610	0.874668	0.586538	-0.21924	BGIOSGA036924	XP_004962393.1pentatricopeptide repeat-containing protein At2g30100, chloroplastic
TCONS_00028065	0.87513	0.648256	0.637125	BGIOSGA028017	XP_004972805.160S ribosomal protein L34
TCONS_00026641	0.87521	1.97539	0.117704	BGIOSGA022508	XP_015695609.1 protein ODORANT1
TCONS_00015931	0.875298	0.355559	1.69478	BGIOSGA006480	XP_004975673.1stress response protein NST1 isoform X2
TCONS_00018585	0.875597	1.40989	0.942682	BGIOSGA018907	XP_003568974.1hydrophobic protein LTI6B
TCONS_00024676	0.875646	0.279399	1.22342	BGIOSGA010196	XP_015694538.1 auxin-responsive protein IAA24-like
TCONS_00016739	0.876146	0.82591	0.887912	BGIOSGA018796	XP_015691594.1 BRCA1-associated protein-like
TCONS_00015385	0.876293	-4.57944	-2.59405	BGIOSGA034071	XP_006653168.2 serine carboxypeptidase-like 18 isoform X1
TCONS_00022786	0.876299	-1.39418	0.481428	BGIOSGA007588	XP_015693425.1 CRIB domain-containing protein RIC10-like
TCONS_00018751	0.87712	2.61428	0.047765	BGIOSGA003130	XP_006654124.1 NADP-dependent malic enzyme, chloroplastic-like
TCONS_00005989	0.877408	1.60817	1.18333	BGIOSGA008121	XP_006648594.2 eukaryotic translation initiation factor 4B3-like
TCONS_00010651	0.87748	1.14174	0.790448	BGIOSGA033027	XP_006650388.1 ras-related protein RABF2b-like
TCONS_00006338	0.878556	-1.71682	-0.433708	BGIOSGA008501	XP_003575236.1photosystem II 5 kDa protein, chloroplastic
TCONS_00031982	0.879138	-0.355523	1.10578	BGIOSGA011366	XP_015697395.1PREDICTED: uncharacterized protein LOC107305181
TCONS_00022940	0.87937	2.1364	1.41427	BGIOSGA005866	XP_015694207.1 protein LAZ1 isoform X1
TCONS_00012702	0.879425	0.100413	0.238822	BGIOSGA028888	XP_003562291.1zinc finger protein 7
TCONS_00008634	0.879664	0.111833	0.315893	BGIOSGA005793	XP_015688967.1PREDICTED: uncharacterized protein LOC102701061 isoform X2
TCONS_00032541	0.87968	0.120749	-3.02507	BGIOSGA004831	XP_004982643.1protein RESPONSE TO LOW SULFUR 2-like
TCONS_00020209	0.880861	0.74709	1.64618	BGIOSGA022397	XP_006655840.1 pentatricopeptide repeat-containing protein At5g48730, chloroplastic-like
TCONS_00023175	0.880894	1.5232	2.06689	BGIOSGA035878	XP_006657483.1 probable glutathione S-transferase GSTU6
TCONS_00013149	0.880952	-1.03675	0.556549	BGIOSGA023196	XP_006650567.1 auxin-responsive protein IAA13
TCONS_00017675	0.881	0.078915	0.452509	BGIOSGA020576	XP_006654320.1 26S proteasome non-ATPase regulatory subunit 14 homolog
TCONS_00001700	0.881309	1.39379	1.11682	BGIOSGA003870	XP_006644622.1 putative methyltransferase DDB_G0268948
TCONS_00002627	0.881644	1.65979	2.42309	BGIOSGA012316	XP_003569088.1E3 ubiquitin-protein ligase RDUF2
TCONS_00001992	0.881704	0.594447	1.16311	BGIOSGA004626	XP_015688269.1 DNA-(apurinic or apyrimidinic site) lyase, chloroplastic-like
TCONS_00022624	0.881842	0.690488	0.90416	BGIOSGA007960	XP_015693652.1 protein TIC 21, chloroplastic-like
TCONS_00013910	0.881966	-0.401043	0.467095	BGIOSGA016083	XP_006652156.1 short-chain dehydrogenase TIC 32, chloroplastic-like

TCONS_00024112	0.882933	-0.277703	-1.23036	BGIOSGA018539	XP_004984090.1F-box only protein 13
TCONS_00027376	0.88297	-0.53208	-1.08925	BGIOSGA036346	XP_006659136.1 flavone 3'-O-methyltransferase 1
TCONS_00007257	0.883142	1.08111	1.2139	BGIOSGA021391	XP_015689222.1 methyltransferase-like protein 16
TCONS_00036783	0.883499	0.406625	-0.00574705	BGIOSGA015065	XP_004977222.1probable L-ascorbate peroxidase 6, chloroplastic/mitochondrial
TCONS_00005516	0.884202	0.506194	-0.0643081	BGIOSGA016636	XP_006646946.2 glyceraldehyde-3-phosphate dehydrogenase GAPCP1, chloroplastic-like
TCONS_00021410	0.884426	1.3034	1.15591	BGIOSGA007252	XP_006656514.1 catalase isozyme B
TCONS_00002798	0.884803	0.999096	1.17047	BGIOSGA002415	NP_001152571.1mitochondrial glycoprotein
TCONS_00005097	0.885063	1.53431	0.895904	BGIOSGA037746	XP_002459097.1probable calcium-binding protein CML10
TCONS_00020192	0.885421	0.330563	1.42355	BGIOSGA026103	XP_015694433.1 dehydrodolichyl diphosphate synthase 6
TCONS_00002726	0.885836	-0.30795	-0.340443	BGIOSGA003514	XP_015692350.1PREDICTED: uncharacterized protein LOC102722630, partial
TCONS_00009626	0.886669	-0.636859	-0.349649	BGIOSGA012171	XP_006649671.1PREDICTED: uncharacterized protein LOC102710957
TCONS_00020356	0.886696	2.61535	0.69627	BGIOSGA011934	XP_010227862.1probable carboxylesterase 12
TCONS_00009555	0.887381	0.67629	0.457701	BGIOSGA012101	XP_015690427.1 SNAP25 homologous protein SNAP33-like
TCONS_00035114	0.887599	1.23239	0.259837	BGIOSGA033758	XP_003577400.1uncharacterized protein LOC100821647
TCONS_00006268	0.887999	1.40167	0.943718	BGIOSGA016465	XP_006647376.1 protein AIG1-like
TCONS_00019613	0.888168	1.17609	0.692615	BGIOSGA017743	XP_006654627.1 outer envelope protein 80, chloroplastic
TCONS_00009267	0.888368	1.06741	1.27212	BGIOSGA011804	XP_006649358.1PREDICTED: uncharacterized protein LOC102714711
TCONS_00027903	0.889178	0.115214	0.159809	BGIOSGA027098	XP_003572142.4iron-sulfur assembly protein IscA-like 2, mitochondrial
TCONS_00008265	0.889808	-0.537535	0.0548821	BGIOSGA014619	XP_004952921.1heavy metal-associated isoprenylated plant protein 47
TCONS_00037600	0.88994	-0.0642687	0.164173	BGIOSGA029665	XP_003579038.2probable ubiquitin-conjugating enzyme E2 18
TCONS_00008963	0.890236	0.85665	0.715763	BGIOSGA002594	XP_004967927.1protein NCA1
TCONS_00016553	0.890471	-0.127599	0.200325	BGIOSGA005784	XP_006652762.1 V-type proton ATPase subunit G1-like
TCONS_00015627	0.890719	0.427768	-0.271499	BGIOSGA000683	XP_015692400.1 probable protein phosphatase 2C 38
TCONS_00030383	0.890913	-0.451929	0.266757	BGIOSGA005915	XP_004957403.1dehydration-responsive element-binding protein 1A
TCONS_00029323	0.891407	1.67441	1.46631	BGIOSGA031067	XP_006660827.1 cilia- and flagella-associated protein 20
TCONS_00014854	0.891457	1.38097	1.7486	BGIOSGA017033	XP_006653722.1 E3 ubiquitin-protein ligase RNF14-like
TCONS_00025829	0.891476	1.31447	0.844617	BGIOSGA027896	XP_006659069.2 COP9 signalosome complex subunit 3
TCONS_00015228	0.891843	0.0165024	-0.829262	BGIOSGA001878	XP_015691631.1 1-phosphatidylinositol-3-phosphate 5-kinase FAB1B-like
TCONS_00013003	0.891894	-1.95629	0.0744347	BGIOSGA022107	XP_015690659.1PREDICTED: uncharacterized protein LOC107303889
TCONS_00023685	0.891912	1.4516	1.21503	BGIOSGA025715	XP_015695291.1 uncharacterized Rho GTPase-activating protein At5g61530 isoform X2
TCONS_00022900	0.892095	1.81186	0.248481	BGIOSGA020543	XP_006657339.1 protein FMP32, mitochondrial
TCONS_00026059	0.892169	-0.160223	-1.86718	BGIOSGA028126	XP_015694828.1 nucleolar protein 58
TCONS_00019589	0.892243	1.12957	0.406555	BGIOSGA004569	XP_006654610.1 secretory carrier-associated membrane protein 3
TCONS_00003115	0.892898	-0.640985	0.628059	BGIOSGA002108	XP_006645644.2 ABC transporter F family member 4-like
TCONS_00003233	0.893018	1.53882	0.256618	BGIOSGA001979	XP_006644005.1 replication protein A 14 kDa subunit
TCONS_00035297	0.893275	0.916984	-0.226163	BGIOSGA007571	XP_006663083.1 CASP-like protein 1U2
TCONS_00014400	0.893808	0.206669	1.26903	BGIOSGA016563	XP_021318613.1L10-interacting MYB domain-containing protein
TCONS_00028793	0.893839	0.041504	1.94408	#N/A	#N/A
TCONS_00025160	0.894217	0.469777	0.485203	BGIOSGA024252	XP_006657736.1 transmembrane protein 184 homolog DDB_G0279555-like
TCONS_00001826	0.894751	-2.98664	-0.915611	BGIOSGA018386	XP_006644709.1 ras-related protein RIC2-like
TCONS_00006847	0.895051	1.12762	-0.933393	BGIOSGA009024	XP_006647867.1PREDICTED: uncharacterized protein LOC102717681
TCONS_00011008	0.896082	-0.165168	-0.292015	BGIOSGA005232	XP_015690204.1 peroxidase 51-like
TCONS_00035724	0.896343	0.353278	1.20789	BGIOSGA022215	XP_015698745.1 probable serine/threonine-protein kinase At1g54610 isoform X1
TCONS_00019349	0.896362	0.659141	0.254591	BGIOSGA032039	XP_002441151.1DAR GTPase 3, chloroplastic
TCONS_00007488	0.897575	0.165522	0.868039	BGIOSGA023331	XP_008799157.1 UPF0047 protein YjbQ
TCONS_00032764	0.897603	0.952783	0.75818	BGIOSGA006155	XP_006662627.1 probable ADP-ribosylation factor GTPase-activating protein AGD8
TCONS_00008309	0.898517	1.03193	1.05545	BGIOSGA006119	XP_006647457.1 ultraviolet-B receptor UVR8-like
TCONS_00030006	0.898581	-0.82455	0.87757	BGIOSGA031873	XP_006660609.2 probable cinnamyl alcohol dehydrogenase 8C
TCONS_00005648	0.898597	0.091964	0.952606	BGIOSGA007763	XP_015689282.1 serine/threonine-protein kinase prpf4B
TCONS_00027137	0.898718	1.86239	0.0550776	BGIOSGA005765	XP_006659686.1 monodehydroascorbate reductase-like
TCONS_00016171	0.898956	0.596435	0.881965	BGIOSGA021329	XP_004976086.1homeobox protein SIX3 isoform X2
TCONS_00034527	0.89955	2.55629	1.2299	BGIOSGA039627	XP_015697970.1 putative disease resistance protein RGA4
TCONS_00033064	0.899605	0.653518	1.52932	BGIOSGA027732	XP_015697789.1 E3 ubiquitin-protein ligase CHFR isoform X4
TCONS_00023427	0.899833	-0.123585	-0.997608	BGIOSGA026491	XP_002459621.2vesicle-associated membrane protein 724

TCONS_00028495	0.899882	0.299534	1.28353	BGIOSGA026463	XP_015695884.1 histone-lysine N-methyltransferase family member SUVH9-like
TCONS_00030934	0.900703	-0.380951	-0.947373	BGIOSGA032740	XP_006662283.1 zinc finger protein-like 1
TCONS_00027431	0.901027	0.781025	1.81232	BGIOSGA005627	XP_013466991.1cullin 3B
TCONS_00036650	0.901617	0.589655	0.885444	BGIOSGA002945	XP_010238746.1cytochrome P450 90A4
TCONS_00008492	0.90164	-0.266069	-1.85067	BGIOSGA005933	XP_022684399.1embryogenesis-associated protein EMB8
TCONS_00005896	0.901968	0.619011	1.43444	BGIOSGA006624	XP_022678564.1nicotianamine aminotransferase A isoform X2
TCONS_00014106	0.901976	#NA	2.55194	#N/A	#N/A
TCONS_00025703	0.90232	0.254608	0.56772	BGIOSGA002159	XP_006658129.1 CBL-interacting protein kinase 2
TCONS_00019721	0.902774	-2.96811	-0.537515	BGIOSGA004290	XP_015692951.1PREDICTED: uncharacterized protein LOC107304243
TCONS_00029344	0.903641	0.238759	1.66978	BGIOSGA004814	XP_022679919.1uncharacterized protein C227.17c isoform X2
TCONS_00022024	0.904256	3.31572	2.26612	BGIOSGA021492	XP_014753035.1uncharacterized protein LOC100837969
TCONS_00022538	0.905277	1.81354	1.1496	BGIOSGA020910	XP_006657122.1 116 kDa U5 small nuclear ribonucleoprotein component-like
TCONS_00024267	0.905295	1.72458	0.853884	BGIOSGA017716	NP_001310269.1uncharacterized LOC103647908
TCONS_00007107	0.905593	0.427891	0.313503	BGIOSGA009291	XP_002453036.1tubulin-folding cofactor A
TCONS_00022233	0.90591	1.5521	0.454566	#N/A	#N/A
TCONS_00022476	0.90601	2.49502	2.51545	BGIOSGA030840	XP_006656189.1 probable histone deacetylase 19
TCONS_00011720	0.90669	-0.241243	0.159974	BGIOSGA000096	XP_015690253.1PREDICTED: uncharacterized protein LOC102699480
TCONS_00012649	0.906812	0.206424	-0.36285	BGIOSGA010321	XP_006650243.1PREDICTED: uncharacterized protein LOC102711137
TCONS_00017372	0.906815	1.02756	1.45227	BGIOSGA011068	XP_010234256.2uncharacterized protein LOC104583529
TCONS_00009367	0.907104	-0.972752	#NA	BGIOSGA017343	XP_004985604.1probable histone H2A variant 1
TCONS_00013143	0.907793	0.841699	1.18111	BGIOSGA009842	XP_015691290.1PREDICTED: uncharacterized protein LOC102707480
TCONS_00001404	0.90796	0.6043	1.50701	BGIOSGA001171	XP_003566917.1cytochrome P450 72A15
TCONS_00011124	0.90807	0.854161	0.525239	BGIOSGA023100	XP_006650726.1 heat stress transcription factor A-2e
TCONS_00004574	0.908201	-0.388979	0.448761	BGIOSGA020102	XP_021313058.1uncharacterized protein LOC8055259
TCONS_00022510	0.908302	#NA	-4.09697	BGIOSGA003221	XP_006656201.1 methanol O-anthraniloyltransferase-like
TCONS_00014382	0.908448	-2.28898	0.793523	#N/A	#N/A
TCONS_00018125	0.908867	1.67488	1.24909	BGIOSGA036553	XP_015692662.1 CBL-interacting protein kinase 19
TCONS_00001402	0.909049	1.14923	0.264795	BGIOSGA001171	XP_004969235.1cytochrome P450 72A15
TCONS_00019888	0.909444	0.346625	0.0305777	#N/A	#N/A
TCONS_00011607	0.90967	0.856065	0.346951	#N/A	#N/A
TCONS_00029392	0.910372	1.03315	0.603896	BGIOSGA031144	XP_006660887.2 pantothenate kinase 2
TCONS_00020039	0.910657	1.86755	1.96447	BGIOSGA030508	XP_002436388.1nuclear speckle RNA-binding protein A
TCONS_00036537	0.910943	-2.31395	-0.806432	BGIOSGA003354	XP_006662656.1 probable receptor-like protein kinase At5g18500
TCONS_00017485	0.911109	-0.688695	-1.01161	BGIOSGA037390	XP_006654233.1 high-light-induced protein, chloroplastic
TCONS_00036120	0.913102	0.134632	0.0803925	BGIOSGA037480	XP_015698598.1PREDICTED: uncharacterized protein LOC107305428
TCONS_00002556	0.913307	-2.61203	-0.0749559	BGIOSGA011489	XP_015690813.1 splicing factor U2af large subunit B-like
TCONS_00012991	0.91347	0.827461	0.373992	BGIOSGA010000	NP_001130854.1uncharacterized LOC100191958
TCONS_00018629	0.91347	2.26803	2.1672	#N/A	#N/A
TCONS_00021889	0.913605	2.58812	2.14496	BGIOSGA021634	XP_006655927.1PREDICTED: uncharacterized protein LOC102721085
TCONS_00018917	0.914256	2.18568	1.2606	BGIOSGA001006	XP_006654194.1 xylanase inhibitor protein 2-like
TCONS_00001998	0.914484	-0.261068	0.0565052	BGIOSGA001916	XP_006644859.1 cysteine proteinase inhibitor 1
TCONS_00024979	0.914494	0.826248	1.01775	BGIOSGA000239	XP_022679495.1probable alpha-1,6-mannosyltransferase MNN10 isoform X1
TCONS_00033802	0.914746	-0.366114	0.358918	BGIOSGA000885	XP_015697825.1 receptor expression-enhancing protein 3-B-like
TCONS_00023526	0.915153	1.9567	1.65351	BGIOSGA025559	XP_006664801.1 adenylate kinase isoenzyme 6 homolog
TCONS_00009531	0.915367	0.0178191	0.999737	BGIOSGA012078	XP_003558542.1probable E3 ubiquitin-protein ligase RHY1A
TCONS_00001769	0.915402	0.0178024	0.430491	BGIOSGA017691	XP_003569741.1uncharacterized protein LOC100841435
TCONS_00016979	0.915666	0.363624	-0.29584	BGIOSGA005265	XP_006653959.1 cytochrome b5
TCONS_00008790	0.916102	-1.98305	0.487617	BGIOSGA031952	XP_015689053.1PREDICTED: uncharacterized protein LOC102702077 isoform X1
TCONS_00006570	0.916279	1.04673	0.738783	BGIOSGA014144	XP_006647623.1 ubiquitin carboxyl-terminal hydrolase 3-like
TCONS_00019206	0.91634	1.05563	0.509935	BGIOSGA000106	XP_006654342.1 mitochondrial fission 1 protein A-like
TCONS_00015843	0.917027	0.111653	0.133811	BGIOSGA015143	XP_006652250.1 DNA-binding protein S1FA2
TCONS_00035778	0.917334	0.342828	0.145996	BGIOSGA036379	XP_006663937.1 cystinosin homolog
TCONS_00015770	0.917411	#NA	-2.15954	BGIOSGA029163	XP_006652208.1PREDICTED: uncharacterized protein LOC102709832
TCONS_00004669	0.917505	1.61358	0.744296	BGIOSGA020007	XP_004970462.1carboxy-terminal domain RNA polymerase II polypeptide A small phosphatase 1
TCONS_00037539	0.918099	-1.98551	0.00865216	BGIOSGA024803	XP_004964201.1protein LATE FLOWERING
TCONS_00007270	0.918125	0.293399	-0.236549	BGIOSGA007213	XP_006646813.1 probable CDP-diacylglycerol--inositol 3-phosphatidyltransferase 2

TCONS_00025711	0.918436	1.90226	0.740568	BGIOSGA023421	XP_006658133.1 vacuolar-sorting receptor 1-like
TCONS_00016291	0.918567	-1.1381	0.560014	BGIOSGA014680	XP_006652540.1 GEM-like protein 4
TCONS_00000686	0.91866	0.684432	0.585618	BGIOSGA023828	XP_006644073.1 gamma carbonic anhydrase 1, mitochondrial
TCONS_00007278	0.918706	0.463055	1.0015	BGIOSGA027990	XP_006646822.1 small nuclear ribonucleoprotein E-like
TCONS_00027368	0.919482	0.647487	1.25383	BGIOSGA016665	XP_006659128.1 DNA-directed RNA polymerase II subunit 1
TCONS_00028328	0.919648	0.61015	2.1189	BGIOSGA032895	XP_002445877.1chitinase-like protein 1
TCONS_00031513	0.920333	-0.159441	-0.0354357	BGIOSGA024085	XP_002465034.1probable glutathione S-transferase GSTU6
TCONS_00000727	0.920463	0.950604	1.39636	BGIOSGA032536	XP_006644099.2PREDICTED: uncharacterized protein LOC102722988
TCONS_00018703	0.92069	0.612856	-0.0220254	BGIOSGA032330	NP_001151326.1uvrB/uvrC motif family protein
TCONS_00013847	0.92069	-1.98878	-0.582819	#N/A	#N/A
TCONS_00025467	0.921328	1.18034	2.32248	BGIOSGA025814	XP_004957834.1ankyrin repeat domain-containing protein 13B
TCONS_00016030	0.921446	1.02438	0.672409	BGIOSGA006178	XP_003575233.1cellulose synthase-like protein D2
TCONS_00013456	0.921535	-1.66792	-1.263	BGIOSGA012378	XP_006649895.1PREDICTED: uncharacterized protein LOC102703849
TCONS_00027055	0.921654	1.54605	0.950827	BGIOSGA031107	XP_006660351.1 transcription factor RF2a-like
TCONS_00025383	0.921731	-1.9901	-0.585419	BGIOSGA024049	XP_003562904.1fatty-acid-binding protein 1
TCONS_00028056	0.922041	0.612197	0.807801	BGIOSGA026933	XP_002445546.1uncharacterized endoplasmic reticulum membrane protein YGL010W
TCONS_00006752	0.922202	0.829918	0.320673	BGIOSGA023188	XP_004953688.1outer envelope membrane protein 7
TCONS_00037546	0.922932	0.330313	-1.58841	BGIOSGA010142	XP_015698542.1 nuclear transcription factor Y subunit A-10-like
TCONS_00025427	0.92296	-1.1843	1.05538	BGIOSGA003415	XP_006657897.1 GDSL esterase/lipase At3g26430-like
TCONS_00021203	0.923222	0.762907	0.732796	BGIOSGA028279	XP_015693963.1 myb family transcription factor APL-like
TCONS_00028377	0.923369	0.693637	0.472627	BGIOSGA029471	XP_006659626.1 transmembrane emp24 domain-containing protein p24beta2-like
TCONS_00031344	0.923737	0.104084	0.955753	BGIOSGA033158	XP_006661880.1PREDICTED: uncharacterized protein YpgQ
TCONS_00000812	0.923836	0.707684	0.868773	BGIOSGA014038	XP_006644128.1 peroxidase 1-like
TCONS_00015129	0.92409	1.26778	1.1683	BGIOSGA017308	XP_015691814.1PREDICTED: uncharacterized protein LOC102701814
TCONS_00028105	0.924516	-0.182052	1.59704	BGIOSGA029700	XP_006662238.1 AT-rich interactive domain-containing protein 4-like
TCONS_00035062	0.924796	1.25396	1.75273	BGIOSGA030484	XP_006663533.1 cell division cycle-associated 7-like protein
TCONS_00008051	0.924817	0.370366	1.04203	BGIOSGA015197	XP_002447708.1U6 snRNA-associated Sm-like protein LSm6
TCONS_00021790	0.924839	1.70642	1.61631	BGIOSGA006950	XP_015694384.1 3-oxoacyl-[acyl-carrier-protein] synthase I, chloroplastic-like
TCONS_00028854	0.924881	0.706369	1.57668	BGIOSGA030602	XP_015696698.1 putative hydrolase C777.06c
TCONS_00013262	0.925386	3.39761	2.33624	BGIOSGA009725	XP_006650673.1 protein JASON-like
TCONS_00028644	0.925692	1.58987	0.831008	BGIOSGA039660	YP_762326.1ATPase subunit 1 (mitochondrion)
TCONS_00003082	0.925721	4.31473	1.90717	BGIOSGA002141	XP_006645627.1 cytochrome P450 710A1-like
TCONS_00023702	0.925812	0.352696	0.564536	BGIOSGA025741	XP_006657720.1 probable (S)-ureidoglycine aminohydrolase
TCONS_00009264	0.92583	1.18531	1.45171	BGIOSGA011801	XP_015698036.1 DNA-directed RNA polymerase I subunit rpa49
TCONS_00017547	0.925839	1.43124	1.32345	BGIOSGA019250	XP_004962450.1probable WRKY transcription factor 70
TCONS_00007697	0.926396	1.05135	-0.124199	BGIOSGA012812	XP_015688539.1 RING-H2 finger protein ATL39-like
TCONS_00021020	0.926545	-0.0818916	1.85195	BGIOSGA023252	XP_004965907.1serine/arginine repetitive matrix protein 2
TCONS_00004582	0.926861	0.24513	0.767929	BGIOSGA021882	XP_015699233.1PREDICTED: uncharacterized protein LOC102716174
TCONS_00002430	0.926918	1.06082	2.22081	BGIOSGA005073	XP_006646622.1 probable mitochondrial import receptor subunit TOM20
TCONS_00009458	0.927234	1.14907	0.706688	BGIOSGA010396	XP_012704214.1vacuolar protein-sorting-associated protein 37 homolog 1 isoform X1
TCONS_00022284	0.927403	-0.250922	-1.19591	BGIOSGA021202	XP_021305782.1uncharacterized protein LOC110431243
TCONS_00005192	0.927487	-3.04463	-1.43782	BGIOSGA019042	XP_006645404.1 pectin acetylesterase 5-like
TCONS_00031510	0.927591	1.731	-0.0152762	BGIOSGA000988	XP_010234937.1probable glutathione S-transferase GSTU6
TCONS_00014701	0.928551	-1.19132	0.524747	BGIOSGA016875	XP_006652604.1 nudix hydrolase 8-like
TCONS_00008897	0.928942	1.8532	1.18513	BGIOSGA038929	XP_006647988.1 heat shock 70 kDa protein, mitochondrial
TCONS_00023665	0.929747	0.894545	0.659147	BGIOSGA025690	XP_006657693.1 mannose-P-dolichol utilization defect 1 protein homolog 2
TCONS_00007747	0.930385	0.487121	0.887288	BGIOSGA006735	XP_006647141.1 protein CbxX, chromosomal
TCONS_00028185	0.930427	0.309364	-0.957089	BGIOSGA026800	XP_004973706.1mitochondrial uncoupling protein 5
TCONS_00003319	0.931072	1.24319	0.885169	BGIOSGA010821	XP_006645766.1 phospholipid-transporting ATPase 1-like
TCONS_00015967	0.932188	1.5389	2.07963	BGIOSGA015012	XP_015691721.1 3-oxoacyl-[acyl-carrier-protein] synthase I, chloroplastic
TCONS_00019769	0.933746	1.82325	1.77153	BGIOSGA002067	XP_006654769.1 50S ribosomal protein L21, mitochondrial
TCONS_00009364	0.934037	0.667641	-0.0591612	BGIOSGA016156	XP_006649438.1 1,2-dihydroxy-3-keto-5-methylthiopentene dioxygenase 2
TCONS_00011233	0.934104	0.634203	0.604168	BGIOSGA014576	XP_006650806.1 actin-depolymerizing factor 4
TCONS_00008503	0.93481	-0.0707986	-0.536465	BGIOSGA005925	XP_020394218.1cyclin-P4-1
TCONS_00017223	0.9356	-0.335661	-0.275297	BGIOSGA011209	XP_006654098.2PREDICTED: uncharacterized protein LOC102708154 isoform X2

TCONS_00010341	0.935707	2.5442	1.34816	BGIOSGA007016	XP_004983984.1chaperone protein ClpB2, chloroplastic
TCONS_00008668	0.93574	-1.35864	0.169383	BGIOSGA007272	XP_004953634.1protein KINESIN LIGHT CHAIN-RELATED 2
TCONS_00014652	0.935761	0.739065	0.895238	BGIOSGA016814	XP_006652560.1 tRNA-dihydrouridine(20) synthase [NAD(P)+]-like
TCONS_00012861	0.935951	1.03338	0.225651	BGIOSGA010115	XP_022679162.1uncharacterized protein LOC101755397
TCONS_00020528	0.935991	0.587886	1.25126	BGIOSGA022703	XP_006656883.2PREDICTED: uncharacterized protein LOC102709185, partial
TCONS_00018636	0.936335	0.282129	0.157294	BGIOSGA021330	XP_004960278.11-aminocyclopropane-1-carboxylate oxidase
TCONS_00006553	0.936651	-1.83743	-0.0666383	BGIOSGA014634	XP_006647606.2 aldehyde dehydrogenase family 3 member F1-like
TCONS_00030237	0.936842	1.35702	1.64696	BGIOSGA031963	XP_004973824.2heat shock protein 81-1
TCONS_00016516	0.936946	1.36745	1.11813	BGIOSGA014452	XP_006652723.1 31 kDa ribonucleoprotein, chloroplastic-like
TCONS_00001887	0.937632	0.916691	1.10308	BGIOSGA004521	XP_015690006.1 DExH-box ATP-dependent RNA helicase DExH6
TCONS_00001467	0.937776	0.872956	0.168685	BGIOSGA004105	XP_003569454.1transcription initiation factor TFIID subunit 7
TCONS_00002832	0.938101	0.648059	0.562851	BGIOSGA002387	XP_006643760.1 very-long-chain enoyl-CoA reductase
TCONS_00019696	0.938501	0.467333	0.717636	BGIOSGA017665	XP_006654706.1PREDICTED: uncharacterized protein LOC102722771
TCONS_00015934	0.938851	1.2683	1.00155	BGIOSGA006480	XP_004975673.1stress response protein NST1 isoform X2
TCONS_00018388	0.93906	-0.172029	-0.701921	BGIOSGA004162	XP_006654845.1 mitogen-activated protein kinase 9
TCONS_00025284	0.939234	0.698336	1.51073	BGIOSGA000326	XP_006658666.2 formin-like protein 11
TCONS_00014341	0.939734	-0.467462	-0.200924	BGIOSGA016501	XP_015691478.1 zeaxanthin epoxidase, chloroplastic
TCONS_00009285	0.94047	-0.925233	0.541203	BGIOSGA004822	XP_006651022.1 cytochrome P450 86B1-like
TCONS_00014567	0.940515	-4.6577	0.742481	BGIOSGA024730	XP_006653579.1PREDICTED: uncharacterized protein At4g06744-like
TCONS_00008426	0.940716	2.0353	0.332837	BGIOSGA027531	XP_005646670.1ubiquitin-like protein
TCONS_00008451	0.940917	0.64324	1.54947	BGIOSGA038588	XP_015688951.1 F-box/LRR-repeat protein 2
TCONS_00006483	0.941133	0.601223	1.79058	BGIOSGA016517	XP_006647540.1 cryptochrome-1
TCONS_00004641	0.941209	#NA	1.44795	BGIOSGA036880	XP_006644921.1 non-specific lipid-transfer protein 1-like
TCONS_00031750	0.941295	1.04729	1.60905	BGIOSGA032368	XP_015697349.1 probable ascorbate-specific transmembrane electron transporter 1
TCONS_00025528	0.942084	-0.41069	0.366583	BGIOSGA037590	XP_006658815.2 DNA-directed RNA polymerases II, IV and V subunit 9A-like
TCONS_00009909	0.943067	-0.428337	0.365128	BGIOSGA028991	XP_015689870.1 calvin cycle protein CP12-1, chloroplastic-like
TCONS_00019853	0.9432	0.982205	1.15491	BGIOSGA004099	XP_003567840.1uncharacterized protein LOC100830726
TCONS_00014794	0.943269	0.289803	1.89647	BGIOSGA016964	XP_002451498.2uncharacterized protein LOC8057530
TCONS_00036219	0.943366	1.0754	1.14231	BGIOSGA037576	XP_015698823.1 protein DEHYDRATION-INDUCED 19 homolog 4-like isoform X2
TCONS_00033177	0.943417	0.583575	-0.0122211	BGIOSGA035012	XP_006662829.1 methylthioribose-1-phosphate isomerase
TCONS_00017258	0.94368	0.571817	0.698704	BGIOSGA019302	XP_006647264.1 proteasome subunit beta type-7-A-like
TCONS_00014882	0.944934	0.828306	0.480161	BGIOSGA017059	XP_002468559.1probable protein-S-isoprenylcysteine O-methyltransferase
TCONS_00015412	0.94547	-0.441833	0.752992	BGIOSGA015961	XP_006652110.1PREDICTED: uncharacterized protein LOC102699580
TCONS_00002638	0.945591	-3.68343	-1.60192	#N/A	#N/A
TCONS_00008983	0.945701	0.868278	0.602383	BGIOSGA013021	XP_006650279.1 cyclin-B1-2
TCONS_00015134	0.946069	-0.986818	-0.107773	BGIOSGA017314	XP_006653857.1 probable plastid-lipid-associated protein 13, chloroplastic
TCONS_00009089	0.946188	0.179497	0.725359	BGIOSGA005314	XP_002454840.1protein BRICK1
TCONS_00032425	0.946788	0.893947	0.798353	BGIOSGA028831	XP_006662441.2 pentatricopeptide repeat-containing protein At1g08610-like
TCONS_00036374	0.946887	-0.622518	1.03581	BGIOSGA037740	XP_006664714.2 putative uridine kinase C227.14 isoform X2
TCONS_00019247	0.947118	1.16914	0.794502	BGIOSGA018101	XP_015692590.1 probable E3 ubiquitin-protein ligase LUL3
TCONS_00022851	0.947658	-0.420887	0.731324	BGIOSGA036884	XP_006656438.1 macrophage migration inhibitory factor homolog
TCONS_00025110	0.947989	1.0687	1.36475	BGIOSGA025722	XP_015695325.1 peptidyl-prolyl cis-trans isomerase FKBP20-2, chloroplastic isoform X1
TCONS_00005624	0.948031	0.275807	-0.509352	BGIOSGA020905	XP_006647029.1 probable calcium-binding protein CML21
TCONS_00008330	0.948693	1.38006	1.33125	BGIOSGA005936	XP_006648819.1 leucine-rich repeat receptor-like protein FASCIATED EAR2
TCONS_00012545	0.948955	2.38316	1.14041	BGIOSGA010435	NP_001143046.1uncharacterized protein LOC100275514
TCONS_00034317	0.949019	1.16236	-1.07485	BGIOSGA003738	XP_006663217.1 purple acid phosphatase 22-like
TCONS_00021057	0.949398	2.11325	1.10816	BGIOSGA011119	XP_010227491.1brefeldin A-inhibited guanine nucleotide-exchange protein 1
TCONS_00023903	0.950111	0.737894	1.5486	BGIOSGA027969	XP_006658690.1 vesicle-associated protein 4-2-like
TCONS_00003295	0.950172	1.43231	0.719117	BGIOSGA021869	XP_006644040.1 putative disease resistance RPP13-like protein 3
TCONS_00009315	0.950575	2.50108	0.910577	BGIOSGA020776	XP_010907453.1 cyclin-dependent kinases regulatory subunit 1
TCONS_00022675	0.950902	0.179067	0.483471	BGIOSGA008554	XP_006656312.1 probable protein phosphatase 2C 58
TCONS_00022213	0.952248	0.145063	1.91646	BGIOSGA021288	XP_015694012.1 translation initiation factor IF-2-like

TCONS_00035589	0.952279	-4.72956	-0.239848	BGIOSGA037009	NP_001140569.1uncharacterized LOC100272635
TCONS_00024107	0.952416	0.462772	0.314521	BGIOSGA025502	XP_020396116.1senescence-associated-like protein isoform X1
TCONS_00012201	0.952763	0.559088	-0.435851	BGIOSGA006084	XP_006650031.1 probable calcium-binding protein CML27
TCONS_00019845	0.952998	-0.600915	-0.709219	BGIOSGA009118	XP_006655615.2PREDICTED: uncharacterized protein LOC102715763
TCONS_00010686	0.954523	-0.0689284	0.481563	BGIOSGA013310	NP_001341858.1uncharacterized protein LOC100502302
TCONS_00007882	0.955791	0.559629	1.01177	BGIOSGA027495	XP_006647223.1 putative F-box/LRR-repeat protein 23
TCONS_00025018	0.956044	1.96534	2.53003	#N/A	#N/A
TCONS_00001670	0.956231	1.2343	1.62553	BGIOSGA007822	XP_006644595.1 mitochondrial substrate carrier family protein P-like
TCONS_00016923	0.956569	-0.960726	1.61092	BGIOSGA003397	XP_006653070.1 cationic peroxidase SPC4-like
TCONS_00010908	0.956619	0.997567	1.40061	BGIOSGA013521	XP_006651796.1 cyclin-H1-1 isoform X1
TCONS_00032952	0.956684	1.52239	0.55852	BGIOSGA013519	XP_015697554.1 protein TRAUCO-like
TCONS_00037189	0.956737	0.46031	1.00905	BGIOSGA001838	XP_006644093.1 probable tRNA N6-adenosine threonylcarbamoyltransferase, mitochondrial isoform X1
TCONS_00007857	0.956854	1.92371	1.11294	BGIOSGA006623	XP_006647199.2PREDICTED: uncharacterized protein LOC102711771
TCONS_00004909	0.957178	0.490362	-0.294908	BGIOSGA016638	XP_006645184.1 uncharacterized membrane protein YuiD-like
TCONS_00006242	0.957748	0.323661	1.10287	BGIOSGA000276	XP_021310923.1coleoptile phototropism protein 1
TCONS_00006099	0.95841	-0.82171	-0.349938	BGIOSGA008254	XP_006647282.1 ethylene-responsive transcription factor-like protein At4g13040
TCONS_00016908	0.958501	1.3483	1.87116	BGIOSGA028654	XP_015691624.1PREDICTED: uncharacterized protein LOC102713185
TCONS_00029880	0.958803	0.148539	1.07978	BGIOSGA029938	XP_006660559.1 protein SAWADEE HOMEODOMAIN HOMOLOG 1 isoform X2
TCONS_00007533	0.958872	0.706525	1.33294	BGIOSGA006951	XP_006647024.1 fumarylacetoacetase
TCONS_00016394	0.959056	1.56873	1.36214	BGIOSGA003738	XP_006652626.1 cis-zeatin O-glucosyltransferase 1-like
TCONS_00012875	0.95971	1.1832	1.19886	BGIOSGA033514	XP_008644626.1protein P21
TCONS_00007410	0.959976	1.83494	1.65762	BGIOSGA012222	XP_008659894.1probable transcription factor KAN4
TCONS_00013824	0.960104	1.44598	1.45072	BGIOSGA016000	XP_006652132.1 prostaglandin E synthase 2
TCONS_00009886	0.961164	0.552372	0.0431992	BGIOSGA012440	XP_015689863.1 pentatricopeptide repeat-containing protein At5g50990-like
TCONS_00021319	0.961395	0.767887	-0.546398	BGIOSGA007536	XP_015693696.1 protein CHLOROPLAST IMPORT APPARATUS 2
TCONS_00009206	0.961596	0.214427	0.883485	BGIOSGA025915	XP_010504143.1 histone H4
TCONS_00022132	0.962042	-0.536571	0.650879	BGIOSGA023257	XP_006656018.1 65-kDa microtubule-associated protein 1
TCONS_00014159	0.962177	0.787758	0.362604	BGIOSGA014605	XP_010239814.1ethylene-responsive transcription factor ERF113
TCONS_00030446	0.962801	0.660474	-0.0607915	BGIOSGA036399	XP_006660909.1 acyl carrier protein 2, chloroplastic-like
TCONS_00031668	0.963399	1.97678	1.9677	BGIOSGA034489	XP_006662091.1 plant intracellular Ras-group-related LRR protein 5
TCONS_00023254	0.963447	1.85359	1.12051	BGIOSGA005583	NP_001152028.2nitrate and chloride transporter
TCONS_00023688	0.963574	1.76327	1.58151	BGIOSGA024287	XP_003563089.1ubiquinol-cytochrome-c reductase complex assembly factor 1
TCONS_00015567	0.964229	2.0859	2.15707	BGIOSGA018401	XP_015691799.1 probable ribonuclease P/MRP protein subunit POP5 isoform X2
TCONS_00022904	0.964235	1.37317	0.423268	BGIOSGA026583	XP_006656485.1 auxin-responsive protein SAUR32-like
TCONS_00014593	0.964464	-0.179645	0.697886	BGIOSGA016753	XP_004976208.1protein disulfide isomerase pTAC5, chloroplastic
TCONS_00010018	0.964619	2.66732	0.646032	BGIOSGA030346	XP_004984468.1probable WRKY transcription factor 70 isoform X2
TCONS_00013633	0.964651	-1.3676	1.0737	BGIOSGA006114	XP_006652079.1 very-long-chain 3-oxoacyl-CoA reductase 1-like
TCONS_00011379	0.964834	-6.29345	-0.501952	BGIOSGA013992	XP_006652054.2PREDICTED: uncharacterized protein LOC102717512
TCONS_00022221	0.964898	0.975572	1.71709	BGIOSGA025411	XP_015693586.1 xylanase inhibitor protein 1-like
TCONS_00029540	0.965247	1.07561	-0.0212168	BGIOSGA031299	XP_006660999.1 sugar phosphatase YfbT-like
TCONS_00018193	0.965256	0.000872148	-0.0759151	BGIOSGA034398	XP_002440134.1uncharacterized protein LOC8068977 isoform X1
TCONS_00008904	0.96579	0.747426	1.21175	#N/A	#N/A
TCONS_00019984	0.966334	1.24195	1.70591	BGIOSGA003633	XP_015692289.1 pentatricopeptide repeat-containing protein At1g11290, chloroplastic
TCONS_00013157	0.966959	0.684889	0.978769	BGIOSGA037096	NP_001141300.1Suppressor/enhancer of lin-12 protein 9 precursor
TCONS_00019079	0.967096	-0.00727882	0.184222	BGIOSGA018281	XP_006654268.1 proactivator polypeptide-like 1 isoform X1
TCONS_00032296	0.967452	0.588468	1.86609	BGIOSGA031838	XP_006661798.1 phosphoglycerate kinase-like
TCONS_00004723	0.967513	1.48148	2.62238	BGIOSGA011319	XP_006646479.1 pentatricopeptide repeat-containing protein At3g46790, chloroplastic
TCONS_00000574	0.968798	1.20052	0.656847	BGIOSGA019540	XP_006645714.1 E3 ubiquitin-protein ligase hel2
TCONS_00035584	0.969862	0.385998	1.01053	BGIOSGA006196	XP_006663819.1 protein BUD31 homolog 3
TCONS_00023270	0.970099	1.57659	0.861269	BGIOSGA013749	XP_008670937.1pentatricopeptide repeat-containing protein At1g59720, chloroplastic/mitochondrial
TCONS_00009959	0.971214	2.03665	1.11052	BGIOSGA012511	XP_009384967.1 sigma factor binding protein 1, chloroplastic-like
TCONS_00023206	0.971579	1.06559	0.823294	BGIOSGA012151	XP_004955620.1heat stress transcription factor A-2b
TCONS_00011211	0.97184	1.30406	1.10156	BGIOSGA035136	XP_006650788.1 50S ribosomal protein L17
TCONS_00034096	0.972073	0.211797	-0.354462	BGIOSGA037630	XP_004980075.1metallothionein-like protein 1A

TCONS_00000806	0.972318	-0.693448	-0.457531	BGIOSGA033399	XP_006645835.1 peroxidase 1-like
TCONS_00022278	0.972529	1.31357	1.58816	BGIOSGA021214	XP_006656099.1 G patch domain-containing protein TGH homolog
TCONS_00032656	0.972745	0.71303	1.00576	BGIOSGA031451	XP_015698309.1PREDICTED: uncharacterized protein LOC102708114, partial
TCONS_00001046	0.973223	1.83592	-0.000360126	BGIOSGA003653	XP_015693243.1PREDICTED: uncharacterized protein LOC102715308
TCONS_00011903	0.973513	1.39147	1.1962	BGIOSGA029874	XP_003558351.1enolase 2
TCONS_00010272	0.973797	0.973531	0.817807	BGIOSGA012837	XP_015689931.1 probable nucleoredoxin 1-2
TCONS_00016038	0.97429	1.93759	0.851981	#N/A	#N/A
TCONS_00036695	0.974646	1.61528	#NA	BGIOSGA030709	XP_006663825.1PREDICTED: uncharacterized protein LOC102705767
TCONS_00000323	0.974676	0.479421	0.16108	BGIOSGA000911	XP_006643841.1 probable choline kinase 2
TCONS_00031690	0.975302	-0.625133	-0.0120236	BGIOSGA005731	XP_006662644.2 protein NRT1/ PTR FAMILY 8.3-like
TCONS_00004041	0.975302	0.216333	-0.0748344	BGIOSGA026798	XP_010232067.1zinc-finger homeodomain protein 5
TCONS_00018672	0.975467	-0.112465	0.0581833	BGIOSGA033359	XP_004977108.1peroxidase 5
TCONS_00006897	0.975727	0.363813	0.85665	BGIOSGA009075	XP_006647918.1 50S ribosomal protein L29, chloroplastic
TCONS_00015898	0.976205	0.969441	0.443368	BGIOSGA008610	XP_006652297.1 protein GrpE-like
TCONS_00034611	0.976471	0.234628	1.19412	BGIOSGA034206	XP_015697946.1 hemolysin A
TCONS_00008995	0.977412	2.20345	1.09459	BGIOSGA012143	XP_015689600.1 chaperone protein DnaJ
TCONS_00001969	0.977711	0.662106	-0.185777	#N/A	#N/A
TCONS_00017085	0.977819	-5.88339	-0.837313	BGIOSGA023839	XP_006654966.1 peroxidase 5-like
TCONS_00007461	0.978383	1.91906	1.38539	BGIOSGA017738	XP_021315643.1uncharacterized protein LOC8077621
TCONS_00034639	0.978787	1.52079	1.81182	BGIOSGA006722	XP_006662913.1 subtilisin-like protease SBT3.9
TCONS_00017496	0.979088	0.741967	1.11453	BGIOSGA032304	XP_015692757.1 solute carrier family 35 member F1-like
TCONS_00009664	0.97985	2.24541	1.61653	BGIOSGA032464	XP_006649713.1 NHP2-like protein 1
TCONS_00032466	0.979892	-0.532288	-0.930937	BGIOSGA031654	XP_006661887.1 putative GEM-like protein 8
TCONS_00036652	0.980399	1.35777	0.787904	BGIOSGA007988	XP_006663193.1 DNA-directed RNA polymerase V subunit 5A
TCONS_00029934	0.980551	0.398745	0.525556	BGIOSGA026979	XP_006660585.1 mitochondrial pyruvate carrier 1-like
TCONS_00031279	0.980618	-0.408043	1.2162	BGIOSGA029218	XP_010234799.1ketohexokinase isoform X3
TCONS_00004163	0.980713	1.16203	0.841574	BGIOSGA031604	XP_015688208.1PREDICTED: uncharacterized protein LOC102703076
TCONS_00017820	0.980735	0.620873	1.36353	BGIOSGA019880	XP_004962075.1thioredoxin-like fold domain-containing protein MRL7L homolog, chloroplastic
TCONS_00035017	0.981585	0.0484211	0.286157	BGIOSGA033840	XP_015692222.1 protein MRG1-like isoform X1
TCONS_00019945	0.982118	1.0755	-0.594868	BGIOSGA024835	XP_004964259.1uncharacterized acetyltransferase At3g50280
TCONS_00026587	0.982508	0.437577	1.2917	BGIOSGA030631	XP_006659387.1 hexuronate transporter
TCONS_00025538	0.983158	0.332179	1.70782	#N/A	#N/A
TCONS_00034653	0.98338	-1.39437	0.82893	BGIOSGA035579	XP_015698073.1 disease resistance protein RPM1-like
TCONS_00005337	0.983843	1.8796	1.61185	BGIOSGA007445	XP_006646826.1PREDICTED: uncharacterized protein LOC102718334
TCONS_00011432	0.984884	#NA	-0.304174	BGIOSGA009327	XP_006649253.1 remorin-like
TCONS_00010174	0.985318	0.971525	0.618824	BGIOSGA024341	XP_021306416.1transmembrane protein 64
TCONS_00018623	0.985542	3.5338	1.71652	#N/A	#N/A
TCONS_00032839	0.986151	0.734429	0.595499	BGIOSGA036728	XP_006662663.2PREDICTED: uncharacterized protein LOC102720274 isoform X1
TCONS_00008624	0.98663	1.33208	0.759026	BGIOSGA005800	XP_006647721.1 histone acetyltransferase MCC1 isoform X1
TCONS_00030172	0.98683	#NA	-0.623041	BGIOSGA013162	XP_003578241.2cyclin-dependent kinase inhibitor 6
TCONS_00023150	0.986922	-0.813649	-0.987401	BGIOSGA014650	XP_006657460.1 protein YABBY 1
TCONS_00011478	0.986949	0.263896	1.14761	BGIOSGA011547	NP_001315323.1uncharacterized LOC103631233
TCONS_00035547	0.986992	-1.99328	0.853085	BGIOSGA009804	XP_006663808.1 protein STRICTOSIDINE SYNTHASE-LIKE 10-like
TCONS_00022345	0.987327	-0.591754	1.09904	BGIOSGA009826	NP_001148396.1avr9/Cf-9 rapidly elicited protein 194
TCONS_00010484	0.987608	-0.0126103	-0.88527	BGIOSGA004504	XP_006650282.2 glycerophosphodiester phosphodiesterase GDPD6-like
TCONS_00014319	0.987615	0.77046	1.33278	BGIOSGA016483	XP_006652329.1PREDICTED: uncharacterized protein LOC102721441
TCONS_00030946	0.987891	-0.399806	2.09762	BGIOSGA009322	XP_015697407.1PREDICTED: uncharacterized protein LOC102721865
TCONS_00025524	0.989248	1.40585	1.0942	BGIOSGA011334	XP_006658809.1 proteasome subunit alpha type-6-like
TCONS_00032103	0.989349	0.416883	0.470216	BGIOSGA002173	XP_006661727.1 IRK-interacting protein-like
TCONS_00031270	0.989634	2.04779	2.66647	BGIOSGA031760	XP_002467125.2uncharacterized protein LOC8059268 isoform X1
TCONS_00009628	0.989993	3.19214	1.36868	BGIOSGA011298	XP_006651191.1 probable inactive poly [ADP-ribose] polymerase SRO3
TCONS_00036807	0.990259	0.683378	0.170937	BGIOSGA023617	XP_006663886.1 3'(2'),5'-bisphosphate nucleotidase
TCONS_00004482	0.990722	2.91856	1.70348	BGIOSGA000739	XP_006644774.1PREDICTED: uncharacterized protein LOC102712946
TCONS_00003977	0.990906	0.987501	0.510534	BGIOSGA001214	XP_008672662.1uncharacterized LOC100278657 isoform X1
TCONS_00016057	0.99093	1.98869	1.32445	BGIOSGA014922	XP_015691494.1PREDICTED: uncharacterized protein LOC107304021
TCONS_00000589	0.991506	-0.259966	0.984969	BGIOSGA003173	XP_006644009.1 bifunctional epoxide hydrolase 2-like
TCONS_00005352	0.99161	0.257632	1.05982	BGIOSGA007467	XP_006648283.1PREDICTED: uncharacterized protein LOC102720491
TCONS_00025300	0.991632	1.79935	2.29144	BGIOSGA024126	XP_006658674.1 pentatricopeptide repeat-containing protein At3g46870-like

TCONS_00019399	0.99193	1.56249	1.11308	BGIOSGA006658	XP_004961917.1phosphoglucan, water dikinase, chloroplastic
TCONS_00000285	0.992043	1.91689	1.48516	BGIOSGA002862	XP_006643810.2PREDICTED: uncharacterized protein LOC102708838
TCONS_00024294	0.992082	-3.99005	-3.58532	BGIOSGA026355	XP_015695027.1 momilactone A synthase-like
TCONS_00035545	0.992146	1.038	1.6769	BGIOSGA022398	XP_006663807.1 probable calcium-binding protein CML36
TCONS_00034309	0.992196	0.594768	1.90625	BGIOSGA036632	XP_015697559.1 RING-H2 finger protein ATL38-like
TCONS_00012239	0.992417	-0.119002	0.9869	BGIOSGA026380	XP_015689885.1 Fanconi anemia group I protein
TCONS_00003554	0.992451	2.33141	2.69916	BGIOSGA001652	XP_003565749.1hydroxyethylthiazole kinase
TCONS_00019842	0.992737	1.34834	0.91791	BGIOSGA017515	XP_006654824.1 malate dehydrogenase, mitochondrial-like
TCONS_00030314	0.992767	0.880959	0.787498	BGIOSGA000261	XP_006661403.1 peptidyl-prolyl cis-trans isomerase FKBP15-1-like
TCONS_00012040	0.993294	0.178344	0.388937	BGIOSGA018766	XP_015689849.1 cardiolipin synthase (CMP-forming), mitochondrial
TCONS_00000615	0.994287	-0.822908	0.164077	BGIOSGA003198	XP_006644024.2PREDICTED: uncharacterized protein LOC102701030
TCONS_00014846	0.994878	1.22029	0.708106	BGIOSGA035743	XP_006652721.1 scarecrow-like protein 9
TCONS_00023694	0.995038	0.449164	0.546876	BGIOSGA013759	XP_004957739.1protein VASCULAR ASSOCIATED DEATH 1, chloroplastic
TCONS_00032136	0.995074	1.40365	2.01974	#N/A	#N/A
TCONS_00011731	0.995098	1.03629	0.816108	BGIOSGA011278	XP_015691055.1 COP9 signalosome complex subunit 4
TCONS_00009465	0.995489	0.804901	1.08796	BGIOSGA012012	XP_006649527.1 bet1-like protein At1g29060
TCONS_00030517	0.996266	0.752094	0.691447	BGIOSGA029290	XP_006660960.1 signal peptidase complex subunit 3B-like
TCONS_00024589	0.996396	1.05687	0.706573	BGIOSGA024804	XP_006657454.1 F-box/kelch-repeat protein At1g30090
TCONS_00017933	0.996403	2.40965	0.943107	BGIOSGA017247	XP_002439911.1glycerol-3-phosphate acyltransferase 5
TCONS_00001010	0.996405	0.844103	0.651523	BGIOSGA003612	XP_006644214.1 ATP-dependent Clp protease proteolytic subunit 3, chloroplastic
TCONS_00032216	0.996851	1.91083	1.6993	BGIOSGA031923	XP_004983185.1histone acetyltransferase GCN5
TCONS_00011715	0.996948	0.973957	0.356661	BGIOSGA011295	XP_006649540.1 NADH dehydrogenase [ubiquinone] 1 alpha subcomplex subunit 13-B-like
TCONS_00024018	0.997124	1.73831	1.03153	BGIOSGA023406	XP_006658757.1 pentatricopeptide repeat-containing protein At5g02860
TCONS_00025089	0.997448	-0.800325	0.25057	BGIOSGA024310	XP_015694492.1PREDICTED: uncharacterized protein LOC102708262 isoform X1
TCONS_00028611	0.997488	-0.539803	-1.51579	BGIOSGA030361	XP_024312582.1protein ACTIVITY OF BC1 COMPLEX KINASE 7, chloroplastic
TCONS_00012274	0.99786	1.1697	0.823168	BGIOSGA006674	XP_024318726.1divinyl chlorophyllide a 8-vinyl-reductase, chloroplastic
TCONS_00012002	0.99859	0.907592	1.31304	BGIOSGA011006	XP_006649849.1PREDICTED: uncharacterized protein LOC102714542
TCONS_00008664	0.998963	0.455525	-0.13182	BGIOSGA029182	XP_015688709.1 monodehydroascorbate reductase 4, peroxisomal-like
TCONS_00019076	1.00021	1.69021	0.451534	BGIOSGA018283	XP_015693176.1 chaperone protein DnaJ-like
TCONS_00004425	1.00141	-0.339969	0.707382	BGIOSGA000797	XP_015688342.1 pentatricopeptide repeat-containing protein At4g21190
TCONS_00020405	1.00149	0.416101	0.939996	BGIOSGA014706	XP_015693517.1 probable fructokinase-6, chloroplastic
TCONS_00009298	1.00294	0.917807	1.14399	BGIOSGA011837	XP_006649386.1 PHD finger protein ING1 isoform X2
TCONS_00033175	1.00424	0.00568852	0.208679	#N/A	#N/A
TCONS_00034259	1.00505	0.829232	1.68719	BGIOSGA036666	XP_006664844.1 50S ribosomal protein L24-like
TCONS_00022864	1.00533	-0.0122932	-0.773872	BGIOSGA007507	XP_006657314.2 protein AIG2-like
TCONS_00035112	1.00567	1.26532	1.23756	BGIOSGA017612	XP_006663566.1 probable galacturonosyltransferase 6 isoform X1
TCONS_00012156	1.00601	0.984177	0.731115	BGIOSGA026158	XP_003558047.130S ribosomal protein S1, chloroplastic
TCONS_00028657	1.00694	-0.0731645	0.356349	BGIOSGA016161	XP_002459936.1glucan endo-1,3-beta-glucosidase 5
TCONS_00026226	1.00718	2.29473	0.75847	BGIOSGA028304	XP_015695458.1 thioredoxin domain-containing protein 9 homolog isoform X3
TCONS_00008187	1.0072	2.05218	0.928163	BGIOSGA006251	XP_015689469.1PREDICTED: uncharacterized protein LOC102701060
TCONS_00008584	1.00777	0.632999	-0.017438	BGIOSGA005843	XP_002452756.22-C-methyl-D-erythritol 2,4-cyclodiphosphate synthase, chloroplastic
TCONS_00027946	1.0081	2.41788	0.228609	BGIOSGA004756	XP_006659368.1 2-methylene-furan-3-one reductase
TCONS_00016643	1.00834	0.219444	1.74972	BGIOSGA014318	XP_015691678.1 exonuclease DPD1, chloroplastic/mitochondrial
TCONS_00004771	1.00835	0.828081	0.692146	BGIOSGA019956	XP_006645064.1PREDICTED: uncharacterized protein LOC102702232
TCONS_00000016	1.00851	0.31312	-0.0113112	BGIOSGA002587	XP_006643633.1 probable inactive shikimate kinase like 1, chloroplastic isoform X3
TCONS_00011991	1.00871	1.34569	1.78199	BGIOSGA011019	XP_006649832.2PREDICTED: uncharacterized protein LOC102709256
TCONS_00005967	1.00892	1.69309	0.722014	BGIOSGA030364	XP_006647209.1 GTPase activating protein 1
TCONS_00002479	1.00905	0.386563	0.760395	BGIOSGA018345	XP_011000911.1 cytochrome c1-2, heme protein, mitochondrial-like
TCONS_00019862	1.00912	1.42525	1.47671	BGIOSGA017496	XP_006654832.1PREDICTED: uncharacterized protein LOC102711434
TCONS_00016802	1.00928	-0.112446	0.46479	BGIOSGA014164	XP_006652967.1 repressor of RNA polymerase III transcription MAF1 homolog isoform X2
TCONS_00025634	1.0098	2.69936	1.42362	BGIOSGA023793	XP_012699642.1CTL-like protein DDB_G0274487
TCONS_00021523	1.01112	1.21755	0.403465	BGIOSGA017662	XP_010090590.1Oligopeptide transporter 7

TCONS_00011639	1.01129	0.104948	2.37567	BGIOSGA029695	XP_006649469.1PREDICTED: uncharacterized protein LOC102700314
TCONS_00001131	1.01137	1.67139	2.01837	BGIOSGA018054	XP_021308602.1uncharacterized protein LOC8059909
TCONS_00022671	1.0119	1.44396	2.4389	BGIOSGA033295	XP_006657197.1PREDICTED: uncharacterized protein LOC102716791, partial
TCONS_00022794	1.01216	0.644717	0.717678	BGIOSGA020659	XP_006656392.1 50S ribosomal protein L24, chloroplastic
TCONS_00008714	1.01238	1.87927	1.82447	BGIOSGA022396	XP_015689331.1 uncharacterized WD repeat-containing protein C2A9.03-like
TCONS_00029629	1.01335	2.09056	1.53829	BGIOSGA017054	XP_002465768.1multiple organellar RNA editing factor 8, chloroplastic/mitochondrial
TCONS_00019656	1.01394	2.15399	0.919319	BGIOSGA001432	NP_001147295.1harpin-induced protein
TCONS_00017833	1.01394	1.49456	1.00581	BGIOSGA038922	XP_006654419.1 cytochrome c
TCONS_00037571	1.01456	1.46829	0.852907	BGIOSGA023512	XP_006664226.1 thaumatin-like protein
TCONS_00007592	1.01469	0.840104	0.66816	BGIOSGA020437	XP_002451757.2uncharacterized protein LOC8071670
TCONS_00013935	1.01492	-0.169401	-0.470278	BGIOSGA034501	XP_004959425.1UDP-glycosyltransferase 90A1
TCONS_00013447	1.01537	2.37752	2.60007	BGIOSGA009192	XP_006651972.1 pentatricopeptide repeat-containing protein At1g74850, chloroplastic
TCONS_00010211	1.016	0.740008	1.17281	BGIOSGA014982	XP_006650147.1PREDICTED: uncharacterized protein LOC102708135
TCONS_00010054	1.01628	0.593046	-0.0865053	BGIOSGA004365	XP_004954455.1caffeic acid 3-O-methyltransferase
TCONS_00003957	1.01726	2.72714	0.611417	BGIOSGA025831	XP_006646084.1 ABC transporter G family member 36
TCONS_00003435	1.01732	0.859635	0.860806	BGIOSGA001787	XP_006644123.2PREDICTED: uncharacterized protein LOC102705856
TCONS_00010714	1.0174	-0.00555973	-0.73932	BGIOSGA013339	XP_006650409.1 protein LST8 homolog
TCONS_00003943	1.01797	1.52476	1.49545	BGIOSGA005493	XP_024312682.1DNL-type zinc finger protein-like
TCONS_00010188	1.01827	0.116314	1.23209	BGIOSGA012750	XP_006650138.1 transcription factor bHLH47-like
TCONS_00014314	1.01853	0.311836	0.835648	BGIOSGA013003	XP_006652325.1 26S proteasome non-ATPase regulatory subunit 11 homolog
TCONS_00002147	1.01868	1.59588	1.87737	BGIOSGA029371	XP_006644990.1 triosephosphate isomerase, cytosolic-like
TCONS_00035757	1.01926	-0.143182	0.264808	BGIOSGA005265	XP_006663911.1 cytochrome b5-like
TCONS_00021365	1.0196	0.0819074	0.756406	BGIOSGA030976	XP_006657336.1 polygalacturonate 4-alpha-galacturonosyltransferase-like
TCONS_00010621	1.0196	0.876092	1.47984	BGIOSGA031868	XP_006651580.1 TATA-binding protein 2-like
TCONS_00030832	1.01974	2.28502	1.25487	BGIOSGA012829	XP_010234663.3uncharacterized protein LOC100839410
TCONS_00022613	1.0202	-1.99685	0.275757	BGIOSGA011993	XP_006657163.2 protein STRUBBELIG-RECEPTOR FAMILY 8-like
TCONS_00007563	1.02024	0.0226931	1.10991	BGIOSGA035073	XP_002453506.1uncharacterized protein LOC8082925
TCONS_00002864	1.02056	0.311969	-0.105599	BGIOSGA024190	XP_021313686.1pentatricopeptide repeat-containing protein At3g46870
TCONS_00019809	1.02062	0.758418	1.16782	BGIOSGA038246	XP_015692802.1 GTPase Era
TCONS_00002938	1.02091	-0.406653	0.541985	BGIOSGA002288	XP_003569450.1NADH-cytochrome b5 reductase-like protein
TCONS_00022167	1.02132	1.15553	1.11347	BGIOSGA035006	XP_015695863.1 AT-hook motif nuclear-localized protein 10-like
TCONS_00017543	1.02253	-3.34849	-1.53339	BGIOSGA027272	XP_006654258.2 probable receptor-like protein kinase At1g80640
TCONS_00031765	1.02273	1.7783	1.11624	BGIOSGA009263	XP_006662187.2 putative F-box protein At5g38390
TCONS_00010271	1.02278	1.12545	0.580731	BGIOSGA012835	XP_006651485.2 ubiquinone biosynthesis protein COQ9, mitochondrial
TCONS_00031346	1.02279	-0.129913	1.0247	BGIOSGA027998	XP_002512354.2cation/calcium exchanger 5
TCONS_00001523	1.02295	-0.755188	-0.00465485	BGIOSGA004155	XP_003569506.1pterin-4-alpha-carbinolamine dehydratase 2, mitochondrial isoform X2
TCONS_00011797	1.0233	-1.47499	-1.21336	BGIOSGA005059	XP_006651171.1 protein DETOXIFICATION 42-like
TCONS_00011299	1.0233	-1.03332	-0.584586	BGIOSGA032907	XP_015690146.1PREDICTED: uncharacterized protein LOC102700516
TCONS_00015478	1.02346	0.737928	0.943108	BGIOSGA040585	XP_006653210.1 glycerol-3-phosphate dehydrogenase SDP6, mitochondrial
TCONS_00016645	1.02348	0.698025	-0.637308	BGIOSGA028122	XP_006652851.1 starch synthase 3, chloroplastic/amyloplastic-like
TCONS_00034985	1.02383	-0.298528	0.582817	BGIOSGA033865	XP_021302228.1putative F-box/FBD/LRR-repeat protein At4g03220 isoform X2
TCONS_00013504	1.02386	0.437583	0.147995	BGIOSGA009478	XP_021320644.1transcription factor TFIIIB component B''
TCONS_00036760	1.02398	-1.95366	-1.01612	BGIOSGA034424	XP_006663860.1 filament-like plant protein 3
TCONS_00011268	1.02465	-4.93612	0.709297	BGIOSGA013872	XP_006650844.1PREDICTED: uncharacterized protein LOC102721821
TCONS_00013175	1.02478	1.63287	0.603569	BGIOSGA009809	XP_006650586.1 protein NAR1 isoform X1
TCONS_00020097	1.02489	1.06176	1.37622	BGIOSGA022277	XP_015694456.1 superoxide dismutase [Fe] 2, chloroplastic
TCONS_00030130	1.02508	1.18254	1.21301	BGIOSGA029676	XP_004956948.1mediator of RNA polymerase II transcription subunit 20a
TCONS_00013126	1.02539	1.28209	0.503533	BGIOSGA006937	XP_006650547.1 50S ribosomal protein L7/L12-like
TCONS_00031691	1.02565	0.943781	0.502925	BGIOSGA004678	XP_004983514.1ADP-ribosylation factor
TCONS_00006098	1.02613	0.845282	-0.215624	BGIOSGA020176	XP_015689416.1 probable magnesium transporter NIPA2 isoform X1
TCONS_00006789	1.02626	1.90884	1.49567	BGIOSGA008964	NP_001281105.1BSD domain containing protein
TCONS_00030402	1.02644	-0.270004	0.212607	BGIOSGA037146	XP_006660878.1 B-box zinc finger protein 19
TCONS_00027743	1.02655	1.23489	0.780682	BGIOSGA027258	XP_006659281.1PREDICTED: uncharacterized protein LOC102715501
TCONS_00013220	1.02715	0.289304	0.384767	BGIOSGA019993	XP_006650639.1 zinc finger protein 1-like
TCONS_00028477	1.02745	1.99026	1.19042	BGIOSGA027834	XP_006659707.1 protein SRC2 homolog
TCONS_00023868	1.02776	1.71953	0.9596	BGIOSGA023949	XP_010241030.1 protein H2A.7

TCONS_00028757	1.02777	-0.398864	0.860078	BGIOSGA027062	XP_003577985.1NEDD8-conjugating enzyme Ubc12
TCONS_00033007	1.02787	0.449009	-1.3101	BGIOSGA023553	XP_022684826.1putative pentatricopeptide repeat-containing protein At3g13770, mitochondrial
TCONS_00011524	1.02862	1.098	1.36752	BGIOSGA011494	XP_006649360.1 pentatricopeptide repeat-containing protein At4g35130, chloroplastic
TCONS_00011410	1.02886	0.965793	1.55388	BGIOSGA031660	XP_006650953.1 AT-hook motif nuclear-localized protein 9-like
TCONS_00016060	1.03043	0.597816	-0.731584	BGIOSGA014918	XP_002447999.1heavy metal-associated isoprenylated plant protein 47
TCONS_00023007	1.03071	1.43873	0.805518	BGIOSGA024953	XP_006648343.1PREDICTED: uncharacterized protein LOC102716192
TCONS_00004818	1.03109	1.52298	1.32565	BGIOSGA019041	XP_004970765.1DNA repair RAD52-like protein 1, mitochondrial
TCONS_00011232	1.03114	1.69636	-0.511729	BGIOSGA025702	XP_006650805.1 actin-depolymerizing factor 3
TCONS_00002973	1.03226	0.277923	0.839073	BGIOSGA002246	XP_015697198.1 protein GIGANTEA
TCONS_00021862	1.03234	1.96524	1.72646	BGIOSGA021658	XP_015693754.1 chaperone protein dnaJ 1, mitochondrial isoform X1
TCONS_00022416	1.03244	-0.726661	-2.32159	BGIOSGA002191	XP_008659681.1uncharacterized protein LOC103638588
TCONS_00011329	1.03417	-1.69412	-0.393877	BGIOSGA005768	XP_003562420.1CRIB domain-containing protein RIC4
TCONS_00004065	1.03452	#NA	-0.741795	BGIOSGA036168	XP_006646144.1 zinc finger CCCH domain-containing protein 9
TCONS_00014770	1.03456	0.518588	0.921056	BGIOSGA016945	XP_015691803.1 E3 ubiquitin-protein ligase RLIM-like isoform X1
TCONS_00030963	1.03472	1.27046	1.13217	BGIOSGA026908	XP_015697313.1 pentatricopeptide repeat-containing protein At4g26680, mitochondrial
TCONS_00009947	1.0354	0.864111	0.639976	BGIOSGA009782	XP_006649983.1 nifU-like protein 1, chloroplastic
TCONS_00009635	1.03546	-0.281399	-0.396773	BGIOSGA024896	XP_006649683.1 phytosulfokines
TCONS_00004386	1.03556	0.969851	1.67067	BGIOSGA000835	XP_006644699.1 pentatricopeptide repeat-containing protein At2g27800, mitochondrial
TCONS_00033230	1.03558	-1.51023	0.0223092	BGIOSGA003922	XP_006662849.1 chlorophyll a-b binding protein CP26, chloroplastic
TCONS_00020896	1.03559	0.143841	0.407555	BGIOSGA010092	XP_015693616.1 zinc transporter 10-like
TCONS_00007284	1.03736	1.6698	1.70202	BGIOSGA010719	XP_003573967.1E3 ubiquitin-protein ligase SINAT2
TCONS_00027380	1.03796	0.507397	0.159488	BGIOSGA027624	XP_015695453.1PREDICTED: uncharacterized protein LOC102700172
TCONS_00000487	1.039	1.75694	1.97202	BGIOSGA003062	XP_006643943.1PREDICTED: uncharacterized protein LOC102701944
TCONS_00015159	1.03921	2.01299	1.60215	BGIOSGA014105	XP_006653870.1 anamorsin homolog 1
TCONS_00035786	1.03938	1.21576	0.852525	BGIOSGA037189	XP_006663919.1PREDICTED: uncharacterized protein LOC102710254 isoform X1
TCONS_00018704	1.04164	2.395	0.400369	BGIOSGA002291	XP_006654096.1 phospholipase D alpha 1-like
TCONS_00034355	1.04174	-0.272688	0.138939	BGIOSGA008598	XP_008647952.1ATOZI1 isoform X1
TCONS_00001960	1.04322	1.28643	0.185123	BGIOSGA023737	XP_003564377.1pentatricopeptide repeat-containing protein At1g61870, mitochondrial
TCONS_00012850	1.04324	1.0315	0.90623	BGIOSGA010123	XP_006650364.1 membrin-11-like
TCONS_00010352	1.04359	-0.240575	-0.689591	#N/A	#N/A
TCONS_00028837	1.04372	0.285787	0.643299	BGIOSGA030587	XP_006661157.1 histidine protein methyltransferase 1 homolog
TCONS_00013211	1.04412	1.23115	1.6127	BGIOSGA009776	XP_006650628.1 TIP41-like protein
TCONS_00010761	1.04414	0.226242	0.906224	BGIOSGA013386	XP_006650439.1 protein yippee-like
TCONS_00010856	1.04432	2.41988	1.35916	BGIOSGA011918	XP_008644553.1probable histone H2AXa
TCONS_00014566	1.04442	-0.15831	0.465366	BGIOSGA016728	XP_006652487.1 filament-like plant protein 7
TCONS_00010072	1.04541	0.772721	-0.349463	BGIOSGA012616	XP_015691168.1 superoxide dismutase [Cu-Zn] 1
TCONS_00024699	1.04582	0.831784	1.28731	BGIOSGA002357	XP_015694902.1 mitochondrial import inner membrane translocase subunit TIM14-1-like
TCONS_00001930	1.04695	0.829051	0.617854	BGIOSGA008864	XP_006644801.1 secretory carrier-associated membrane protein 2
TCONS_00001534	1.04732	0.467002	1.49553	BGIOSGA004165	XP_006644483.1 high mobility group B protein 14
TCONS_00035487	1.04762	0.319651	0.207219	BGIOSGA036822	XP_015698516.1 bis(5'-adenosyl)-triphosphatase-like
TCONS_00009768	1.04813	-0.588832	-0.0281657	BGIOSGA012320	XP_006649836.2PREDICTED: uncharacterized protein LOC102710675
TCONS_00025576	1.04904	-1.58477	-0.383347	BGIOSGA033071	XP_003562619.1uncharacterized protein LOC100829568
TCONS_00015396	1.04907	0.554802	0.387752	BGIOSGA033044	XP_006652116.1PREDICTED: uncharacterized protein LOC102701532
TCONS_00001013	1.04926	-1.39295	1.0247	BGIOSGA003615	XP_002439670.1NRR repressor homolog 1
TCONS_00020266	1.05001	0.413929	0.853837	BGIOSGA036042	XP_023157105.1calcium uniporter protein 2, mitochondrial-like
TCONS_00021220	1.05074	1.67828	1.19937	BGIOSGA016936	XP_002437427.1probable transcription factor KAN4
TCONS_00026289	1.05082	1.98268	1.28497	BGIOSGA028374	XP_006659269.1 alpha-soluble NSF attachment protein 2
TCONS_00010103	1.05113	1.22286	0.774851	BGIOSGA022438	XP_003557888.1probable glutathione peroxidase 4
TCONS_00022153	1.0513	1.8475	0.956965	BGIOSGA000270	XP_006656929.1 rho GDP-dissociation inhibitor 1-like
TCONS_00028919	1.0513	0.717103	1.585	BGIOSGA008205	XP_004956719.1protein CHAPERONE-LIKE PROTEIN OF POR1, chloroplastic
TCONS_00006518	1.0518	0.402513	0.674975	BGIOSGA008686	XP_006647578.1PREDICTED: uncharacterized protein LOC102704121 isoform X1
TCONS_00030152	1.05233	1.00403	0.92488	BGIOSGA006224	XP_006660682.1 transmembrane protein 50 homolog
TCONS_00020953	1.05294	1.65759	-0.272746	BGIOSGA023177	XP_006656194.2 dehydrogenase/reductase SDR family member 12
TCONS_00025922	1.05323	1.50516	-0.0760497	BGIOSGA023345	XP_006659834.1 cytochrome P450 89A2-like
TCONS_00023454	1.05376	0.311897	0.837181	BGIOSGA025486	XP_006657609.2 hydroxypyruvate reductase

TCONS_00031352	1.05439	0.548153	-0.287745	BGIOSGA027680	NP_001278609.160S ribosomal protein L37
TCONS_00013153	1.05458	1.09262	0.793933	BGIOSGA015713	XP_006650570.1 ATP-sulfurylase 3, chloroplastic
TCONS_00001862	1.0546	-0.673516	-1.10992	BGIOSGA004496	XP_006646347.1 protein CURVATURE THYLAKOID 1C, chloroplastic-like
TCONS_00035565	1.05564	0.073466	-0.167149	BGIOSGA034791	XP_004978652.1R3H domain-containing protein 4
TCONS_00016090	1.05582	0.516793	1.01636	BGIOSGA014889	XP_006652408.1 pentatricopeptide repeat-containing protein At1g62350-like
TCONS_00011907	1.05697	-1.68101	1.15829	BGIOSGA011105	XP_006649750.1 HVA22-like protein i
TCONS_00003816	1.05737	0.459683	0.231533	BGIOSGA001396	XP_006644261.1 UPF0587 protein C1orf123
TCONS_00036671	1.05741	-0.639032	-1.09312	BGIOSGA034502	XP_006663814.1 stem-specific protein TSJT1
TCONS_00008752	1.05754	1.13233	1.44328	BGIOSGA005680	XP_015688725.1 pentatricopeptide repeat-containing protein At3g22690-like
TCONS_00027850	1.05809	0.612033	0.721745	BGIOSGA027156	XP_006659316.1 growth hormone-regulated TBC protein 1
TCONS_00032552	1.05824	0.695655	1.2195	BGIOSGA034281	NP_001132805.1CRS1 / YhbY domain containing protein
TCONS_00013834	1.05833	1.23528	0.998339	BGIOSGA014516	XP_006652137.2 4-hydroxy-tetrahydrodipicolinate synthase 1, chloroplastic
TCONS_00037482	1.0587	-0.989426	0.87534	BGIOSGA028216	XP_006664721.2 VAN3-binding protein-like
TCONS_00003914	1.05912	2.3676	1.62433	BGIOSGA019517	XP_015694297.1 50S ribosomal protein L25
TCONS_00023822	1.05989	0.911934	1.01612	BGIOSGA021484	XP_006657784.1 probable pyruvate, phosphate dikinase regulatory protein, chloroplastic
TCONS_00023502	1.06021	1.06747	1.54695	BGIOSGA001536	XP_006644228.1 probable ribosome biogenesis protein RLP24
TCONS_00020585	1.06094	2.29315	1.21768	BGIOSGA012703	XP_006656020.1 peroxidase 1-like
TCONS_00012170	1.06127	0.86305	2.06998	BGIOSGA010839	XP_006650008.1 octanoyltransferase
TCONS_00026250	1.06144	1.91401	0.315981	BGIOSGA028334	XP_003573628.1nuclear pore complex protein NUP43
TCONS_00033409	1.06174	-0.185907	0.523821	BGIOSGA033485	XP_006662920.1 short integuments 2, mitochondrial
TCONS_00020965	1.06234	-1.19419	-0.768611	BGIOSGA022562	XP_002437220.1crocetin glucosyltransferase, chloroplastic
TCONS_00003070	1.06293	1.70715	0.999169	BGIOSGA025757	XP_006643894.1 polyadenylate-binding protein-interacting protein 9-like
TCONS_00011657	1.06357	0.707333	1.09362	BGIOSGA023919	XP_006649488.1 nuclear transcription factor Y subunit A-7
TCONS_00025551	1.06394	1.44353	-0.0580227	BGIOSGA022501	XP_006657980.1 UMP-CMP kinase 3
TCONS_00024086	1.06405	0.891417	1.54897	BGIOSGA026119	XP_015694993.1 glutamic acid-rich protein-like
TCONS_00014440	1.06472	0.58796	0.793303	BGIOSGA025577	XP_003579964.1F-box protein At1g70590 isoform X1
TCONS_00004216	1.06472	1.79819	1.52693	BGIOSGA031679	XP_004969618.1probable glutathione S-transferase GSTU6
TCONS_00014231	1.06476	2.40016	1.33499	BGIOSGA034845	XP_006653384.1 digalactosyldiacylglycerol synthase 1, chloroplastic-like
TCONS_00023474	1.06478	1.42531	0.908465	BGIOSGA018909	XP_008651557.1B12D protein isoform X1
TCONS_00030362	1.0648	0.0402025	0.0725688	BGIOSGA029436	XP_015696730.1 probable hydroxyacylglutathione hydrolase 2, chloroplastic
TCONS_00012178	1.0655	1.96549	2.44346	BGIOSGA028321	XP_006651334.1 putative disease resistance protein RGA4
TCONS_00002441	1.06677	-0.507499	-0.152856	BGIOSGA001288	XP_006645256.1PREDICTED: uncharacterized protein LOC102709973
TCONS_00011821	1.06692	1.28198	1.28616	BGIOSGA013727	XP_006651188.2PREDICTED: uncharacterized protein LOC102710289
TCONS_00034956	1.06759	0.488296	1.01133	BGIOSGA033896	XP_015699016.1 N-alpha-acetyltransferase MAK3
TCONS_00010055	1.06845	2.1598	1.12939	BGIOSGA028820	NP_001131008.1photolyase/blue-light receptor PHR2
TCONS_00021363	1.06976	1.52945	-0.0414879	BGIOSGA026745	XP_003560546.1uncharacterized protein LOC100835592
TCONS_00021922	1.07033	0.0863409	0.242818	BGIOSGA021601	XP_006656816.1 glutathione S-transferase DHAR3, chloroplastic-like
TCONS_00002081	1.07064	-1.50321	-4.86795	BGIOSGA004711	XP_006644926.1PREDICTED: uncharacterized protein LOC102710071
TCONS_00019695	1.07097	0.800262	1.16585	BGIOSGA013259	XP_015693263.1 type I inositol polyphosphate 5-phosphatase 1-like isoform X1
TCONS_00017757	1.07108	-1.21771	0.0778734	#N/A	#N/A
TCONS_00027474	1.07138	1.19863	1.05823	BGIOSGA018487	XP_003581244.1uncharacterized protein LOC100842205
TCONS_00035312	1.0714	0.848282	1.35863	BGIOSGA033600	XP_006664861.1PREDICTED: uncharacterized protein LOC102700676
TCONS_00000541	1.07152	-0.397235	-0.791901	BGIOSGA000814	XP_006643981.1 optic atrophy 3 protein homolog
TCONS_00002374	1.07165	-0.305164	0.0684988	BGIOSGA033219	XP_003564869.1E3 ubiquitin-protein ligase AIRP2
TCONS_00010782	1.07167	1.34572	1.14279	BGIOSGA013409	XP_006650465.1 30S ribosomal protein S13, chloroplastic
TCONS_00037375	1.0717	-0.208407	0.448761	BGIOSGA035995	XP_006664110.1PREDICTED: uncharacterized protein LOC102699736
TCONS_00000085	1.07176	2.01025	1.61037	BGIOSGA002654	XP_002455301.1fructose-bisphosphate aldolase 1, chloroplastic isoform X1
TCONS_00007005	1.07277	1.92346	0.905158	BGIOSGA001789	XP_006648037.1 UDP-glucuronate 4-epimerase 1
TCONS_00035958	1.07362	1.58941	0.680672	BGIOSGA005446	XP_015698506.1 putative leucine aminopeptidase 1
TCONS_00016302	1.07425	0.0328615	1.28447	BGIOSGA005963	XP_006652556.1 E3 ubiquitin-protein ligase RMA1H1-like isoform X2
TCONS_00028234	1.07476	-1.20859	0.369856	BGIOSGA002813	XP_021320677.1NDR1/HIN1-like protein 10
TCONS_00003889	1.07497	1.15519	0.702155	BGIOSGA001323	XP_006644318.1 probable V-type proton ATPase subunit d
TCONS_00036285	1.07545	2.28301	2.50334	BGIOSGA010017	XP_006664663.2 scarecrow-like protein 9
TCONS_00011214	1.07632	-0.979138	-0.714969	BGIOSGA010658	XP_015690533.1PREDICTED: uncharacterized protein LOC107303854

TCONS_00032545	1.07666	0.100454	0.775145	BGIOSGA008704	XP_004982635.1auxin-responsive protein SAUR32
TCONS_00022663	1.07702	-1.99424	0.323957	BGIOSGA022217	XP_002438758.1uncharacterized protein LOC8058425
TCONS_00016601	1.07727	0.774144	-0.126634	BGIOSGA014363	XP_006652809.1PREDICTED: uncharacterized protein LOC102699397
TCONS_00037048	1.07762	1.53302	1.77294	BGIOSGA023057	XP_015698849.1 uncharacterized mitochondrial protein ymf11
TCONS_00036357	1.07785	1.99696	2.83605	BGIOSGA027007	XP_004963060.1uncharacterized protein LOC101786433
TCONS_00035504	1.07832	-0.62812	-1.22402	BGIOSGA012529	XP_015698179.1 ELMO domain-containing protein C-like
TCONS_00004196	1.07879	2.34091	-1.2252	BGIOSGA018441	XP_006644559.1 acidic endochitinase-like
TCONS_00008650	1.079	-0.74813	-0.466458	BGIOSGA013214	XP_006647756.1 zeaxanthin 7,8(7',8')-cleavage dioxygenase, chromoplastic
TCONS_00008793	1.07912	0.160172	-0.345036	BGIOSGA002078	XP_006647874.1 rac-like GTP-binding protein 3
TCONS_00002226	1.07974	1.44933	1.22759	BGIOSGA036573	XP_006645073.1 actin-7
TCONS_00023411	1.07987	-0.183787	-1.10006	BGIOSGA018791	XP_004955881.1uncharacterized protein LOC101759795
TCONS_00003192	1.08015	0.428458	0.471858	BGIOSGA002304	XP_015694317.1 GDSL esterase/lipase At4g10955-like
TCONS_00025876	1.08016	0.908441	1.19998	BGIOSGA027941	XP_006659801.1PREDICTED: uncharacterized protein LOC102706676 isoform X2
TCONS_00012713	1.08021	2.0037	1.88675	BGIOSGA000785	XP_004982436.1serine/threonine-protein kinase SAPK10
TCONS_00028267	1.08058	0.416337	2.14494	BGIOSGA033197	XP_015695810.1 putative tRNA (cytidine(34)-2'-O)-methyltransferase
TCONS_00018487	1.08086	0.878002	0.632115	BGIOSGA008938	XP_002440458.1mitochondrial import inner membrane translocase subunit Tim9
TCONS_00017059	1.08113	0.746986	-0.0194048	BGIOSGA002217	XP_002439217.2zinc finger CCCH domain-containing protein 33
TCONS_00029432	1.08121	1.687	2.50816	BGIOSGA031181	XP_004957509.1uncharacterized protein LOC101754806
TCONS_00001408	1.08151	1.91218	2.2275	BGIOSGA020441	XP_006644398.1 serine carboxypeptidase-like 50
TCONS_00011581	1.08283	0.757096	-0.0684573	BGIOSGA033027	XP_006649414.1 ras-related protein RABF1
TCONS_00032933	1.08296	1.38075	1.35766	BGIOSGA000087	XP_006663807.1 probable calcium-binding protein CML36
TCONS_00015882	1.08348	-1.96134	-1.06077	BGIOSGA015104	XP_003579736.2RNA-binding motif protein, X-linked-like-3
TCONS_00033224	1.08371	1.0757	1.15399	BGIOSGA037131	XP_006662845.2 CCR4-NOT transcription complex subunit 11-like
TCONS_00019869	1.08512	0.325908	-0.896777	BGIOSGA025611	XP_006654838.1 protein RADIALIS-like 3
TCONS_00005194	1.08521	0.163128	1.47848	BGIOSGA000011	XP_013470439.1transmembrane protein, putative
TCONS_00028661	1.08521	#NA	#NA	#N/A	#N/A
TCONS_00030633	1.08585	-0.555733	1.26355	BGIOSGA012208	XP_003573741.1NHP2-like protein 1
TCONS_00004982	1.08698	0.753062	0.780294	BGIOSGA000235	XP_006645241.1 transcription and mRNA export factor SUS1
TCONS_00027065	1.08736	-1.46589	0.172294	BGIOSGA029111	XP_006660355.1 traB domain-containing protein
TCONS_00008337	1.08746	0.615901	0.787553	BGIOSGA006089	XP_015689352.1PREDICTED: uncharacterized protein LOC102701141
TCONS_00009437	1.08829	1.69419	1.03327	BGIOSGA021631	XP_004985484.13-ketoacyl-CoA synthase 10
TCONS_00028283	1.08835	-0.0553162	0.601834	BGIOSGA016787	XP_006660269.1 estradiol 17-beta-dehydrogenase 8-like
TCONS_00036954	1.0897	-0.460925	-0.535407	BGIOSGA018532	XP_006663942.1 isoflavone reductase homolog
TCONS_00017056	1.09004	0.311211	0.596223	BGIOSGA011943	XP_004960371.1dnaJ homolog subfamily B member 4
TCONS_00024887	1.09034	-0.437085	0.95093	BGIOSGA023001	XP_004960178.126S proteasome non-ATPase regulatory subunit 7 homolog A
TCONS_00019586	1.09071	1.63647	0.112325	BGIOSGA017772	XP_006654608.2PREDICTED: uncharacterized protein LOC102719015
TCONS_00012285	1.09086	0.909242	0.901207	BGIOSGA013569	XP_006650092.1 50S ribosomal protein L6, chloroplastic
TCONS_00023606	1.09132	-0.389261	0.578502	BGIOSGA025631	XP_006655793.1 trafficking protein particle complex subunit 4
TCONS_00017248	1.09187	0.720989	1.28903	BGIOSGA009668	XP_003568799.1ubiquitin-conjugating enzyme E2 2
TCONS_00025441	1.09206	0.842286	0.805901	BGIOSGA020130	XP_006658756.2PREDICTED: uncharacterized protein LOC102714574
TCONS_00031626	1.09208	1.01171	-0.271222	BGIOSGA025094	XP_006662062.1 hexose carrier protein HEX6-like
TCONS_00014999	1.0925	-0.279096	-0.51135	BGIOSGA025936	XP_015691585.1 cyclin-P1-1
TCONS_00006049	1.0928	0.563043	1.29437	BGIOSGA008205	XP_003574973.1uncharacterized protein LOC100839623
TCONS_00031194	1.0945	1.3183	1.16703	BGIOSGA025143	XP_006661791.1 SKP1-like protein 11
TCONS_00009352	1.09461	2.74116	1.74611	BGIOSGA006824	XP_015691195.1 DEAD-box ATP-dependent RNA helicase 38
TCONS_00037261	1.09495	1.49861	0.498721	#N/A	#N/A
TCONS_00016608	1.09504	0.723989	0.962843	#N/A	#N/A
TCONS_00009136	1.09547	-1.56655	-0.831262	BGIOSGA023353	XP_015690260.1 pEARLI1-like lipid transfer protein 1
TCONS_00006254	1.09566	1.17845	0.475997	BGIOSGA008415	XP_006647365.2 tetratricopeptide repeat protein 33
TCONS_00012558	1.09625	2.1677	1.12522	BGIOSGA010416	XP_006644417.1 DEAD-box ATP-dependent RNA helicase 2
TCONS_00016047	1.09667	1.33888	-0.156239	BGIOSGA022257	XP_015691834.1PREDICTED: uncharacterized protein LOC102715199
TCONS_00022006	1.09766	0.859762	0.768007	BGIOSGA021511	XP_006655979.1 signal recognition particle 9 kDa protein
TCONS_00032862	1.09794	0.27759	-0.0362058	BGIOSGA034679	XP_006663744.1 probable non-specific lipid-transfer protein 3
TCONS_00022002	1.09808	-0.859142	1.13407	BGIOSGA011585	XP_006655976.1 nudix hydrolase 21, chloroplastic-like

TCONS_00011208	1.09842	0.677762	0.64671	BGIOSGA013809	NP_001136636.1uncharacterized LOC100216764 precursor
TCONS_00026057	1.09846	0.979679	2.3938	BGIOSGA028130	XP_006659201.1 pentatricopeptide repeat-containing protein At3g59040
TCONS_00013555	1.09948	0.303543	0.612589	BGIOSGA009420	XP_006650914.1 pentatricopeptide repeat-containing protein At4g25270, chloroplastic-like
TCONS_00023372	1.10073	2.06442	1.17191	BGIOSGA004694	XP_006657570.1 KH domain-containing protein SPIN1-like isoform X2
TCONS_00018068	1.10077	0.2017	1.33246	BGIOSGA022600	XP_020396393.1uncharacterized protein LOC103631732
TCONS_00031348	1.10121	1.23445	1.83526	BGIOSGA033169	XP_015697172.1 tetratricopeptide repeat protein 1
TCONS_00004102	1.10144	0.371677	1.15391	BGIOSGA001087	XP_006644466.1 vacuolar protein sorting-associated protein 25
TCONS_00011090	1.10251	0.262945	0.891322	BGIOSGA029275	NP_001150776.2syntaxin 121
TCONS_00016049	1.1031	2.08094	0.447585	BGIOSGA020710	XP_022684202.1probable inactive ATP-dependent zinc metalloprotease FTSHI 3, chloroplastic isoform X1
TCONS_00022519	1.10329	-0.782134	-0.668177	BGIOSGA007757	XP_008658130.1uncharacterized LOC100193920 isoform X1
TCONS_00019153	1.10339	0.866391	0.946135	BGIOSGA018199	XP_006654308.1 chaperone protein dnaJ 49
TCONS_00011674	1.10373	0.596435	0.759916	BGIOSGA031166	XP_006649499.1 proteasome subunit alpha type-6
TCONS_00020588	1.10414	0.919289	1.27224	BGIOSGA022772	XP_006656019.1 trigger factor-like protein TIG, Chloroplastic
TCONS_00030777	1.10502	1.31709	1.05719	BGIOSGA027437	XP_006661654.1 geraniol 8-hydroxylase-like
TCONS_00028702	1.1056	1.19436	-1.10752	BGIOSGA021171	XP_015696850.1 ubiquitin-conjugating enzyme E2-17 kDa-like
TCONS_00003990	1.10603	2.14816	1.72724	BGIOSGA014210	XP_015696505.1 protein ROOT PRIMORDIUM DEFECTIVE 1
TCONS_00011051	1.1063	0.204132	1.80263	BGIOSGA027991	XP_015691125.1PREDICTED: uncharacterized protein At1g01500
TCONS_00000379	1.10661	-1.07757	-0.949445	BGIOSGA005280	XP_021894753.1metallothionein-like protein type 3
TCONS_00027156	1.1072	1.28449	#NA	#N/A	#N/A
TCONS_00030364	1.10721	0.990786	1.01572	BGIOSGA008586	XP_006661438.2 C2 and GRAM domain-containing protein At1g03370
TCONS_00020607	1.10747	0.946619	1.99861	BGIOSGA031706	XP_021306087.1BTB/POZ domain-containing protein At2g04740 isoform X1
TCONS_00009733	1.10769	1.00738	0.842561	BGIOSGA007210	XP_006651240.1 peroxisomal nicotinamide adenine dinucleotide carrier
TCONS_00011830	1.1088	-4.26064	-4.1408	BGIOSGA028301	XP_004985109.1uncharacterized protein At1g01500
TCONS_00018788	1.10961	2.707	2.09974	BGIOSGA004533	XP_002440770.1casein kinase 1-like protein HD16 isoform X1
TCONS_00023084	1.10975	0.421206	0.986663	#N/A	#N/A
TCONS_00024041	1.11046	1.63542	1.79051	BGIOSGA002124	XP_006657919.1 pentatricopeptide repeat-containing protein At1g26460, mitochondrial
TCONS_00019843	1.11051	0.673625	0.0940262	BGIOSGA009454	XP_006654825.1 GTP-binding nuclear protein Ran-2
TCONS_00035111	1.11051	-0.545427	-0.145727	BGIOSGA010624	XP_003577407.13-ketoacyl-CoA synthase 20
TCONS_00009878	1.11065	-0.0674678	0.188602	BGIOSGA010019	XP_003558117.1sex determination protein tasselseed-2
TCONS_00027687	1.11139	-2.76396	-0.410519	BGIOSGA027316	XP_003573634.1glycine-rich protein 5
TCONS_00003105	1.11229	1.18441	0.3767	BGIOSGA004136	XP_006643912.1 histone-lysine N-methyltransferase ATX5-like
TCONS_00015205	1.11243	1.38631	1.9954	BGIOSGA030760	XP_015691871.1 probable CCR4-associated factor 1 homolog 11
TCONS_00036149	1.11374	0.604453	-1.29165	BGIOSGA011380	XP_006664601.1 myosin-16-like
TCONS_00032453	1.11414	1.96912	1.42412	BGIOSGA036904	XP_004982810.1pentatricopeptide repeat-containing protein At5g16420, mitochondrial
TCONS_00000250	1.11466	1.40969	0.397877	BGIOSGA002828	XP_006643772.1 cell number regulator 8
TCONS_00002033	1.11522	1.03051	0.769871	BGIOSGA017816	XP_006644881.1 proteasome subunit alpha type-3
TCONS_00001766	1.11564	-2.17494	0.201347	BGIOSGA018579	XP_015688100.1 BURP domain-containing protein 3
TCONS_00026981	1.11604	-0.236206	-0.0236316	BGIOSGA016651	XP_015696123.1 PGR5-like protein 1B, chloroplastic
TCONS_00009827	1.11854	-0.394147	-0.158086	BGIOSGA035979	XP_006649897.1 metallothionein-like protein 1B
TCONS_00005276	1.11876	-0.0838157	0.0990172	BGIOSGA026333	XP_006646784.2 retinol dehydrogenase 12-like
TCONS_00003783	1.11917	-0.167777	0.745984	BGIOSGA001428	XP_006644275.1PREDICTED: uncharacterized protein LOC102706231
TCONS_00031317	1.11977	1.79567	1.4012	BGIOSGA033133	XP_006664817.1 transportin-3 isoform X1
TCONS_00003015	1.12014	0.868468	1.46761	BGIOSGA000017	XP_002455007.1translation machinery-associated protein 22
TCONS_00008656	1.12024	0.561103	1.27323	BGIOSGA005772	XP_015689351.1 probable pinoresinol-lariciresinol reductase 3
TCONS_00011072	1.12024	-0.656628	0.268152	BGIOSGA022521	XP_012704122.1transcription factor PIF4 isoform X2
TCONS_00018372	1.12031	0.775929	0.925806	BGIOSGA030202	XP_003567812.1cinnamoyl-CoA reductase-like SNL6
TCONS_00018066	1.12072	1.10124	0.33482	BGIOSGA024189	XP_006654596.1 protein translation factor SUI1 homolog
TCONS_00017030	1.12148	0.902779	1.52542	BGIOSGA023446	XP_015692960.1 UPF0769 protein C21orf59 homolog
TCONS_00002829	1.12219	1.11507	1.21629	BGIOSGA035283	XP_015694044.1 probable LRR receptor-like serine/threonine-protein kinase At3g47570
TCONS_00003343	1.12225	-2.12928	-0.861998	BGIOSGA022649	XP_006644076.1 transcription factor MYB86
TCONS_00035968	1.12295	0.374627	-0.996034	BGIOSGA016983	XP_015689643.1 UDP-glucose 6-dehydrogenase 4
TCONS_00004593	1.12327	1.56988	1.64072	BGIOSGA000622	XP_004970308.1ribonuclease III domain-containing protein RNC1, chloroplastic
TCONS_00024174	1.12344	2.24229	0.497097	BGIOSGA021748	XP_006658008.1 cytochrome P450 709B2-like
TCONS_00028524	1.12356	1.25658	2.47315	#N/A	#N/A

TCONS_00017071	1.1236	1.57393	1.39866	BGIOSGA019123	XP_006654005.1PREDICTED: uncharacterized protein LOC102705341
TCONS_00013205	1.12389	-0.4615	-1.93598	BGIOSGA029560	XP_024313641.1UDP-glycosyltransferase 83A1-like
TCONS_00004757	1.12469	0.562884	1.55333	BGIOSGA036996	XP_006645047.1 protein BUD31 homolog 1
TCONS_00012222	1.1261	0.704415	0.626241	BGIOSGA011527	XP_006650052.1 reticulon-like protein B16
TCONS_00012104	1.12636	2.41787	2.49989	BGIOSGA011539	XP_004955571.1uncharacterized protein LOC101769811
TCONS_00005770	1.12673	0.66667	2.14345	BGIOSGA016295	XP_006663096.2PREDICTED: uncharacterized protein LOC102715982
TCONS_00027465	1.12673	#NA	#NA	#N/A	#N/A
TCONS_00013059	1.1279	-0.71722	-0.30235	BGIOSGA003890	XP_003559532.1uncharacterized protein LOC100838339
TCONS_00020819	1.12824	0.664339	1.7247	#N/A	#N/A
TCONS_00025693	1.12904	-1.41344	-0.700932	BGIOSGA015858	XP_002463377.1uncharacterized protein LOC8078539
TCONS_00003121	1.12936	1.02865	2.6843	BGIOSGA002103	XP_015688080.1PREDICTED: uncharacterized protein LOC102715905
TCONS_00015759	1.12971	-1.33789	0.136151	#N/A	#N/A
TCONS_00016670	1.13002	-7.34296	-0.217221	BGIOSGA010904	XP_006653796.1 kinesin-5-like
TCONS_00014857	1.13115	#NA	1.71764	BGIOSGA002958	XP_015691936.1PREDICTED: uncharacterized protein LOC107304086
TCONS_00010676	1.13115	-0.340022	0.132674	BGIOSGA013301	XP_021306794.1uncharacterized protein LOC8056728
TCONS_00007059	1.13128	0.531072	1.5993	BGIOSGA009240	XP_006648076.1PREDICTED: uncharacterized protein LOC102707841
TCONS_00030439	1.13131	-1.25704	1.97603	BGIOSGA032062	XP_015696847.1 myb-related protein Hv1-like
TCONS_00017786	1.13163	1.20296	0.845126	BGIOSGA001050	XP_004962137.1transcription termination factor MTERF4, chloroplastic
TCONS_00021485	1.13194	0.287173	0.579276	BGIOSGA008598	XP_010228076.1uncharacterized protein LOC100841062
TCONS_00032177	1.13252	3.65792	#NA	#N/A	#N/A
TCONS_00022678	1.13277	0.815249	1.20504	BGIOSGA020763	XP_006656317.1 U5 small nuclear ribonucleoprotein 40 kDa protein
TCONS_00006832	1.13312	1.10544	1.55739	BGIOSGA004700	XP_006647854.1 pentatricopeptide repeat-containing protein At2g13600-like
TCONS_00010899	1.13385	3.61017	2.58545	BGIOSGA033889	XP_002466510.1uncharacterized protein LOC8059942
TCONS_00017213	1.13443	-0.987749	-1.0022	BGIOSGA030042	XP_015692496.1 phosphoenolpyruvate/phosphate translocator 3, chloroplastic-like
TCONS_00004929	1.13451	0.471571	0.615915	BGIOSGA000292	XP_002458950.1uncharacterized protein LOC8078704
TCONS_00016407	1.13516	-1.34589	1.70782	BGIOSGA007044	XP_015691538.1 branched-chain-amino-acid aminotransferase 5, chloroplastic-like
TCONS_00024499	1.13516	0.560997	0.444787	BGIOSGA012179	XP_015695391.1 phytosulfokines 4-like
TCONS_00017373	1.13613	0.952601	1.60893	BGIOSGA017555	XP_006576121.1B3 domain-containing protein At3g25182
TCONS_00000796	1.13663	0.610854	1.59209	BGIOSGA028078	XP_006645833.2 CRS2-associated factor 2, chloroplastic
TCONS_00025821	1.13727	2.44108	1.54856	BGIOSGA027813	XP_015689043.1 RNA-binding protein CP29B, chloroplastic
TCONS_00001100	1.13764	0.972538	0.11681	BGIOSGA012381	NP_001130329.1uncharacterized protein LOC100191424
TCONS_00028681	1.13764	2.65061	0.11681	BGIOSGA018386	XP_006660497.1 ras-related protein RGP1
TCONS_00018662	1.13781	0.477454	0.806257	BGIOSGA019962	XP_006654074.1 universal stress protein A-like protein
TCONS_00023647	1.13858	2.63758	0.63605	BGIOSGA014534	XP_006657683.2 protein argonaute 18-like
TCONS_00029132	1.13863	0.0420213	0.478062	BGIOSGA003942	XP_003578208.1STOREKEEPER protein
TCONS_00013110	1.13879	0.848431	0.0777023	BGIOSGA009876	XP_004981899.1uncharacterized protein LOC101786698
TCONS_00020826	1.13883	-0.351018	0.113924	BGIOSGA035562	NP_001151100.1VQ motif family protein
TCONS_00009331	1.13892	1.52565	1.32157	BGIOSGA011875	XP_015690315.1 ribosomal large subunit pseudouridine synthase B isoform X2
TCONS_00036912	1.13926	0.697902	1.11502	BGIOSGA016258	XP_004960722.1probable aspartyl aminopeptidase
TCONS_00011287	1.13959	1.55069	1.29399	BGIOSGA017959	XP_006650863.1 ferredoxin-3, chloroplastic-like isoform X2
TCONS_00035957	1.13999	#NA	#NA	BGIOSGA003344	XP_006663988.2PREDICTED: uncharacterized protein LOC102710357 isoform X2
TCONS_00007651	1.13999	0.64742	#NA	BGIOSGA003742	XP_010234075.1uncharacterized protein LOC100842455 isoform X2
TCONS_00000634	1.14017	2.85246	-0.0111675	BGIOSGA027484	XP_022682015.1putative serpin-Z12
TCONS_00006259	1.1408	0.196215	-0.124376	BGIOSGA006729	XP_003579789.1uncharacterized protein LOC100839050
TCONS_00022000	1.14083	0.0626788	0.246417	BGIOSGA023252	XP_003563993.11-aminocyclopropane-1-carboxylate oxidase homolog 4
TCONS_00023117	1.14096	-2.31739	-0.256683	BGIOSGA025153	XP_006657445.1 photosystem I reaction center subunit psaK, chloroplastic
TCONS_00024262	1.14129	1.43602	1.63378	BGIOSGA026773	XP_004958542.1probable glycosyltransferase STELLO1
TCONS_00003314	1.14138	1.40391	1.90597	BGIOSGA013090	XP_006644057.2 presenilin-like protein At1g08700
TCONS_00010291	1.1414	-0.0746975	0.980209	BGIOSGA012855	XP_006650190.1PREDICTED: uncharacterized protein LOC102720218
TCONS_00035543	1.14152	1.58712	0.613762	BGIOSGA036960	XP_021317106.1uncharacterized protein LOC8085629
TCONS_00006017	1.14176	0.367357	0.765427	BGIOSGA008166	XP_006647310.1 prefoldin subunit 1
TCONS_00006584	1.14217	2.64448	2.10578	BGIOSGA003742	XP_021314032.1protein NUCLEAR FUSION DEFECTIVE 6, chloroplastic/mitochondrial isoform X1
TCONS_00009675	1.14326	0.205864	-0.391378	BGIOSGA006560	XP_006649727.1 probable 4-hydroxy-tetrahydrodipicolinate reductase 2, chloroplastic
TCONS_00029166	1.14362	0.619909	-0.950821	BGIOSGA029627	XP_015696813.1 2-hydroxyisoflavanone dehydratase-like

TCONS_00022833	1.14406	1.43619	0.458547	BGIOSGA020614	XP_015693911.1PREDICTED: LOW QUALITY PROTEIN: uncharacterized protein LOC102699872
TCONS_00004120	1.14423	4.54866	#NA	BGIOSGA004060	XP_002458271.1protein RADIALIS-like 3
TCONS_00016138	1.14427	2.55406	1.57021	BGIOSGA000630	XP_015691668.1 DNA-directed RNA polymerases II and IV subunit 5A-like isoform X2
TCONS_00011985	1.14485	0.86696	0.149727	BGIOSGA002448	XP_002443019.1glucuronoxylan 4-O-methyltransferase 1
TCONS_00006445	1.14492	2.08453	1.61261	BGIOSGA015089	XP_006647500.2 protein GrpE-like
TCONS_00009903	1.14493	1.48121	2.2863	BGIOSGA012458	XP_006649958.1 argininosuccinate lyase, chloroplastic
TCONS_00020060	1.1452	-0.359498	0.0983329	BGIOSGA005386	XP_003557140.1uncharacterized protein LOC100833108
TCONS_00018098	1.14568	-0.02498	0.31534	BGIOSGA020152	XP_006654619.1 thioredoxin H-type-like
TCONS_00034593	1.14586	1.01532	-0.394323	BGIOSGA034226	XP_006663327.1PREDICTED: uncharacterized protein LOC102703438, partial
TCONS_00027600	1.14615	0.639286	2.1159	#N/A	#N/A
TCONS_00019282	1.14669	1.28581	1.5083	BGIOSGA035760	XP_015693272.1 lipase-like
TCONS_00015829	1.14693	-1.20153	-0.971513	BGIOSGA006831	XP_004952609.1COPII coat assembly protein SEC16
TCONS_00028315	1.14701	1.48739	0.522878	BGIOSGA026658	XP_002445860.1pentatricopeptide repeat-containing protein At3g42630
TCONS_00025953	1.14756	0.544954	0.964906	BGIOSGA028029	XP_006659152.1 NADH dehydrogenase [ubiquinone] iron-sulfur protein 6, mitochondrial
TCONS_00013446	1.14797	0.636994	0.091547	BGIOSGA010474	XP_021307183.1mitotic-spindle organizing protein 1B
TCONS_00019280	1.14797	-0.363006	#NA	#N/A	#N/A
TCONS_00031285	1.14797	0.221956	0.676509	#N/A	#N/A
TCONS_00006194	1.14816	0.536882	1.10298	BGIOSGA007658	XP_004954965.1protein BIC1
TCONS_00024169	1.1488	0.268259	0.634593	BGIOSGA003693	XP_006658004.1 ras-related protein RABA5a
TCONS_00033474	1.14956	1.49185	1.45076	BGIOSGA036259	XP_022680191.1bifunctional dihydrofolate reductase-thymidylate synthase isoform X1
TCONS_00030137	1.14969	1.88282	0.894706	BGIOSGA023727	XP_003572276.1counting factor 45-1
TCONS_00008487	1.15051	1.95582	1.08534	BGIOSGA008719	XP_006647605.1 aldehyde dehydrogenase family 3 member F1-like
TCONS_00008514	1.15084	0.54242	0.99551	BGIOSGA013173	XP_015689402.1 3-isopropylmalate dehydratase small subunit 3-like
TCONS_00012739	1.1513	-0.367061	1.0834	BGIOSGA010224	XP_010232525.1uncharacterized protein LOC100826416 isoform X1
TCONS_00015010	1.1513	-0.367061	0.668358	BGIOSGA026791	XP_015691687.1 anthocyanidin reductase-like
TCONS_00032769	1.1513	-0.030026	0.294899	BGIOSGA031341	XP_022685583.1protein SAMBA
TCONS_00002827	1.15175	0.801999	0.548726	BGIOSGA037630	XP_006643757.1 putative disease resistance RPP13-like protein 1
TCONS_00012148	1.15207	#NA	-0.333533	BGIOSGA010864	XP_003558058.1uncharacterized protein LOC100826796
TCONS_00025088	1.15281	0.331674	0.332868	BGIOSGA011681	XP_004957682.1vacuolar protein sorting-associated protein 24 homolog 1
TCONS_00031486	1.15303	2.49572	0.64301	BGIOSGA025907	XP_021306985.1U-box domain-containing protein 15 isoform X2
TCONS_00006927	1.15348	1.2527	1.18587	BGIOSGA034214	XP_006649072.1PREDICTED: uncharacterized protein LOC102713247
TCONS_00001111	1.15354	3.53718	2.07787	BGIOSGA003736	XP_024314393.1DEAD-box ATP-dependent RNA helicase 15 isoform X1
TCONS_00003001	1.15512	2.30896	1.5776	BGIOSGA019112	XP_015688255.1 zinc finger CCCH domain-containing protein 2-like
TCONS_00000976	1.1556	0.627912	2.07278	BGIOSGA010029	XP_006651971.2 protein NRT1/ PTR FAMILY 5.6-like
TCONS_00018410	1.15572	2.48997	2.036	BGIOSGA020462	XP_006655644.2 pentatricopeptide repeat-containing protein At4g01030, mitochondrial
TCONS_00001582	1.15624	0.423644	0.697717	BGIOSGA010645	XP_015692049.1 ER membrane protein complex subunit 6 isoform X1
TCONS_00007538	1.15624	-1.76285	0.160625	BGIOSGA022800	XP_006647025.1 chlorophyll a-b binding protein 8, chloroplastic
TCONS_00017134	1.15648	1.84466	0.400317	BGIOSGA000168	XP_004960278.11-aminocyclopropane-1-carboxylate oxidase
TCONS_00009662	1.15687	1.69686	1.65457	BGIOSGA018172	XP_004951184.1small nuclear ribonucleoprotein SmD3b
TCONS_00032210	1.1569	2.39141	1.74678	BGIOSGA024672	XP_004983172.1probable prolyl 4-hydroxylase 7
TCONS_00035710	1.15697	2.69853	1.73625	BGIOSGA017530	XP_004967389.2uncharacterized protein LOC101762180
TCONS_00011717	1.15743	3.67771	0.805306	BGIOSGA032789	XP_006651124.1 inositol-3-phosphate synthase
TCONS_00028293	1.15809	0.277193	1.58118	BGIOSGA012463	XP_006659583.1 calvin cycle protein CP12-3, chloroplastic-like
TCONS_00020055	1.15829	1.95515	1.50439	BGIOSGA021664	XP_015693866.1 peptidyl-prolyl cis-trans isomerase CYP18-1
TCONS_00006860	1.1588	1.20801	1.31981	BGIOSGA009040	XP_015688731.1 methyltransferase-like protein 7A
TCONS_00020934	1.15979	1.0383	-0.135979	BGIOSGA023148	XP_015694366.1PREDICTED: uncharacterized protein LOC102705259 isoform X2
TCONS_00005470	1.15995	0.828194	2.20292	BGIOSGA020656	XP_006646906.1PREDICTED: uncharacterized protein LOC102717676
TCONS_00026477	1.16033	1.62266	2.13145	BGIOSGA005567	XP_006661190.1 protein ELF4-LIKE 4-like
TCONS_00021087	1.16042	0.319872	1.35574	BGIOSGA022688	XP_006656275.1 sodium-coupled neutral amino acid transporter 2-like
TCONS_00020532	1.16085	0.399705	0.0597561	BGIOSGA013062	XP_015693548.1 dof zinc finger protein DOF3.1-like
TCONS_00020034	1.16096	1.53298	1.35474	BGIOSGA015078	XP_015693438.1 pentatricopeptide repeat-containing protein At5g08510
TCONS_00025845	1.16107	2.10308	1.3271	BGIOSGA016539	XP_006659081.1 glyceraldehyde-3-phosphate dehydrogenase 1, cytosolic
TCONS_00007516	1.16109	2.18995	0.469918	BGIOSGA001967	XP_006648407.1 peroxiredoxin-2E-2, chloroplastic
TCONS_00004384	1.16127	0.707405	0.619267	BGIOSGA020205	XP_006646318.1 serine/threonine-protein kinase HT1-like

TCONS_00020684	1.16137	-1.70038	-0.941512	BGIOSGA028801	XP_006656957.1 GDSL esterase/lipase EXL1-like
TCONS_00000447	1.16159	0.751278	0.210505	BGIOSGA022097	XP_006643922.1PREDICTED: uncharacterized protein LOC102718874
TCONS_00013001	1.16162	1.03466	0.869584	BGIOSGA009985	XP_006651727.1 N-alpha-acetyltransferase 20
TCONS_00023696	1.16187	1.62102	3.14472	BGIOSGA018057	XP_006653490.1 pentatricopeptide repeat-containing protein At4g39952, mitochondrial
TCONS_00026755	1.16187	2.42837	1.64222	#N/A	#N/A
TCONS_00033960	1.1621	2.47788	1.7914	BGIOSGA034666	XP_006664864.2PREDICTED: uncharacterized protein LOC102701411
TCONS_00020217	1.16227	0.173476	0.528056	BGIOSGA018242	XP_006655843.1 sulfiredoxin, chloroplastic/mitochondrial
TCONS_00004975	1.16272	0.580899	0.821033	BGIOSGA000244	XP_006645234.1 small ubiquitin-related modifier 1
TCONS_00005483	1.16283	1.94193	3.96177	#N/A	#N/A
TCONS_00035370	1.16307	-0.628624	-0.306087	BGIOSGA035681	XP_015698038.1 beta-glucosidase-like SFR2, chloroplastic
TCONS_00029531	1.16386	1.71181	0.327982	BGIOSGA031289	XP_015696715.1 monodehydroascorbate reductase
TCONS_00004791	1.16398	0.598038	1.25503	BGIOSGA000425	XP_006646508.1PREDICTED: uncharacterized protein LOC102701050
TCONS_00011960	1.1646	2.31858	0.635441	BGIOSGA019949	XP_006649800.1 josephin-like protein
TCONS_00029672	1.16481	0.346268	-1.10043	BGIOSGA014924	XP_006660466.1 indole-3-glycerol phosphate synthase, chloroplastic-like
TCONS_00027133	1.16509	0.774209	0.777982	BGIOSGA014864	XP_003574920.1protein-L-isoaspartate O-methyltransferase 1 isoform X2
TCONS_00011259	1.16581	0.616857	0.0474479	BGIOSGA013865	XP_014751942.1uncharacterized protein LOC100831283
TCONS_00015641	1.16581	-2.12011	1.67972	BGIOSGA040713	XP_022685469.1uncharacterized protein LOC101776219
TCONS_00022158	1.16645	-0.326035	0.692892	BGIOSGA007023	XP_006656041.1 thioredoxin-like protein Clot
TCONS_00036178	1.16657	#NA	-0.954455	BGIOSGA026618	XP_004962821.1rapid alkalinization factor
TCONS_00036260	1.16685	2.28474	0.962345	BGIOSGA019672	XP_006664116.1 importin subunit beta-1
TCONS_00022816	1.16737	-0.596926	0.358964	BGIOSGA031468	XP_006656408.1 probable calcium-binding protein CML29
TCONS_00026331	1.16785	0.264975	0.385115	BGIOSGA028412	XP_006659271.1 protein MEMO1 isoform X1
TCONS_00005952	1.16798	#NA	0.0419932	BGIOSGA003241	XP_004952376.1protein FON2 SPARE1
TCONS_00001647	1.16798	1.19951	1.04199	BGIOSGA021863	XP_015691557.1 nuclear poly(A) polymerase 2-like
TCONS_00026458	1.16831	-1.38581	-1.95885	BGIOSGA028527	XP_004983194.1uncharacterized protein LOC101754068
TCONS_00016669	1.16863	0.13076	0.549029	BGIOSGA014295	XP_006652865.1 thioredoxin-like protein CXXS1
TCONS_00004577	1.16863	1.93577	2.04033	#N/A	#N/A
TCONS_00017286	1.17014	-0.387789	0.036496	BGIOSGA008713	NP_001152623.1Grx_A2 - glutaredoxin subgroup III
TCONS_00035093	1.17015	1.2505	1.3462	BGIOSGA033747	XP_015697705.1 E3 ubiquitin-protein ligase RNF181-like
TCONS_00014173	1.17054	-1.92678	-1.70387	BGIOSGA016331	NP_001142209.13-beta hydroxysteroid dehydrogenase/isomerase family protein
TCONS_00002408	1.1707	1.19657	0.772044	#N/A	#N/A
TCONS_00017649	1.17079	2.69813	2.72253	BGIOSGA023731	XP_015693318.1 serine/arginine-rich-splicing factor SR34-like isoform X2
TCONS_00037457	1.17175	2.55799	2.43452	BGIOSGA012625	XP_015698612.1PREDICTED: uncharacterized protein LOC107305446
TCONS_00008495	1.17248	-0.197696	3.00458	BGIOSGA014629	XP_015689610.1 metal-nicotianamine transporter YSL2
TCONS_00015432	1.17267	1.77742	-0.457915	BGIOSGA014315	XP_015691680.1 vacuolar amino acid transporter 1-like
TCONS_00003828	1.17294	1.77907	2.61437	BGIOSGA018218	XP_006644258.1 secretory carrier-associated membrane protein 5
TCONS_00029173	1.17294	2.45045	1.14488	BGIOSGA030921	XP_015696382.1 2-hydroxyisoflavanone dehydratase-like
TCONS_00033244	1.17316	2.19386	1.6138	#N/A	#N/A
TCONS_00017752	1.17359	1.96704	1.35757	BGIOSGA019814	XP_006655288.1 protein PALE CRESS, chloroplastic
TCONS_00027621	1.17367	1.46908	2.2409	BGIOSGA022636	XP_006659230.1 cytosolic sulfotransferase 8-like
TCONS_00008843	1.17421	-1.12925	0.0261742	BGIOSGA005573	XP_002454591.1sprouty-related, EVH1 domain-containing protein 3
TCONS_00000180	1.17447	1.71104	1.72117	BGIOSGA025077	XP_004968155.1AP2/ERF and B3 domain-containing protein Os01g0141000
TCONS_00007709	1.17534	1.41378	1.02331	BGIOSGA012235	XP_014660900.1E3 ubiquitin-protein ligase EL5
TCONS_00000983	1.17569	-0.130945	0.507853	BGIOSGA032514	XP_015698079.1 tRNA (guanine(37)-N1)-methyltransferase 1
TCONS_00020045	1.17635	0.605253	0.480186	BGIOSGA022227	NP_001147665.139S ribosomal protein L47
TCONS_00019631	1.17711	1.41171	2.34076	BGIOSGA000816	XP_015692968.1 28 kDa heat- and acid-stable phosphoprotein-like
TCONS_00015772	1.17711	-1.13261	1.60379	#N/A	#N/A
TCONS_00007173	1.17742	0.0393948	0.449957	BGIOSGA020178	XP_022683865.1rac-like GTP-binding protein 5
TCONS_00016089	1.17767	2.51058	1.60237	BGIOSGA014890	XP_015691737.1PREDICTED: uncharacterized protein LOC102721636
TCONS_00017214	1.1778	-1.39647	-0.982931	BGIOSGA002867	XP_010231664.1embryo-specific protein ATS3A
TCONS_00028260	1.1778	0.478002	0.754034	BGIOSGA026718	XP_006660257.2 pseudouridine-5'-phosphate glycosidase
TCONS_00016792	1.1778	4.33145	1.60203	BGIOSGA027488	XP_015694711.1 probable WRKY transcription factor 3
TCONS_00013140	1.17831	2.03587	-0.7212	BGIOSGA034068	XP_006650557.1 magnesium-dependent phosphatase 1-like
TCONS_00035798	1.17868	2.03543	2.33677	BGIOSGA021915	XP_006656638.1 RNA-binding protein 2-like
TCONS_00030057	1.17868	2.05093	1.42988	BGIOSGA029753	XP_015696878.1 26S proteasome non-ATPase regulatory subunit 9
TCONS_00009413	1.17915	0.517968	1.51574	BGIOSGA011967	XP_006651090.2 omega-amidase, chloroplastic
TCONS_00020912	1.17987	0.71649	1.51433	BGIOSGA006859	XP_003563721.1GATA transcription factor 21

transcriptome

TCONS_00014764	1.17987	1.45187	0.111363	BGIOSGA016940	XP_021318118.1uncharacterized protein LOC110436155
TCONS_00009390	1.17987	0.688475	0.0118275	BGIOSGA031943	XP_006651075.1 F-box/kelch-repeat protein At1g55270-like
TCONS_00025282	1.18071	1.22321	1.21539	BGIOSGA024143	XP_006657802.1 54S ribosomal protein L24, mitochondrial
TCONS_00016652	1.18097	0.719393	1.5278	BGIOSGA014310	XP_006656109.1PREDICTED: uncharacterized protein LOC102702667 isoform X4
TCONS_00028952	1.18099	-0.989799	0.492616	BGIOSGA030708	XP_006660604.1 annexin D5-like
TCONS_00007037	1.18122	1.80122	1.95727	BGIOSGA009222	XP_006649136.1 uncharacterized GTP-binding protein YjiA-like
TCONS_00008120	1.18122	1.75202	3.41077	BGIOSGA025817	NP_001148627.1ATPase inhibitor isoform 1
TCONS_00033043	1.18145	-0.400947	-0.477571	BGIOSGA000058	XP_006662762.2 60S ribosomal protein L3-like
TCONS_00024855	1.18145	3.27148	0.81521	BGIOSGA013892	XP_003561273.1NDR1/HIN1-like protein 10
TCONS_00032476	1.18145	0.184015	0.329783	BGIOSGA016773	XP_004986461.1putative ripening-related protein 4
TCONS_00034485	1.18182	1.50548	1.70736	BGIOSGA003294	XP_006663283.1 F-box/LRR-repeat protein At3g59190-like
TCONS_00013089	1.18243	2.08967	2.7333	BGIOSGA032661	XP_002463963.1SKI family transcriptional corepressor 2
TCONS_00019564	1.18298	0.239231	1.9109	BGIOSGA029416	NP_001146973.1ethylene-responsive transcription factor 4
TCONS_00003091	1.18304	1.26948	1.39618	BGIOSGA014738	XP_003565491.3RING-H2 finger protein ATL39
TCONS_00006466	1.18309	1.09948	0.878192	BGIOSGA008637	XP_003575374.1protein MIS12 homolog isoform X1
TCONS_00018036	1.18326	1.01181	-0.581655	BGIOSGA020083	XP_024315322.1uncharacterized protein At5g19025-like
TCONS_00008871	1.18331	-0.403299	2.00317	BGIOSGA005541	XP_006649077.1PREDICTED: uncharacterized protein LOC102714624
TCONS_00031408	1.18331	1.0308	1.66775	BGIOSGA033220	XP_015697122.1PREDICTED: LOW QUALITY PROTEIN: uncharacterized protein LOC102706415
TCONS_00010851	1.18347	0.181463	1.00278	BGIOSGA002218	XP_006650510.1 transcription factor MYB44-like
TCONS_00003752	1.18363	3.18127	1.70283	BGIOSGA017578	XP_006644286.1 pentatricopeptide repeat-containing protein At4g38150
TCONS_00016314	1.18372	0.333147	0.380656	BGIOSGA031583	XP_006652575.1 vesicle-associated protein 1-2-like
TCONS_00003791	1.18372	3.40354	2.11762	BGIOSGA033322	XP_002455769.1probable glutathione S-transferase GSTU6
TCONS_00023405	1.18382	0.988376	0.171826	BGIOSGA029560	XP_003557348.1DIMBOA UDP-glucosyltransferase BX8
TCONS_00005447	1.18382	2.57588	1.35554	BGIOSGA037423	XP_004958778.1probable protein phosphatase 2C 33
TCONS_00014316	1.18401	0.239676	0.116908	BGIOSGA000361	XP_006652327.1 PHD finger protein ALFIN-LIKE 4-like
TCONS_00006477	1.1841	1.28376	2.46064	BGIOSGA012222	XP_006647539.1 myb-related protein Myb4-like
TCONS_00027890	1.18419	1.07534	1.52282	BGIOSGA004250	XP_004973277.1lipid phosphate phosphatase 2
TCONS_00014989	1.18428	0.469951	1.32269	BGIOSGA027926	XP_006652854.1 BTB/POZ and MATH domain-containing protein 2-like
TCONS_00034152	1.18465	1.86803	1.41487	BGIOSGA036910	XP_006663735.1 phosphoglucan phosphatase LSF2, chloroplastic-like
TCONS_00021246	1.18477	1.11843	0.806899	#N/A	#N/A
TCONS_00019713	1.18499	2.21125	1.89945	BGIOSGA024580	XP_006655539.1PREDICTED: uncharacterized protein LOC102715310
TCONS_00030044	1.18527	1.56106	0.445751	BGIOSGA001222	XP_012698877.1zinc-finger homeodomain protein 9
TCONS_00022892	1.18537	2.19402	1.52161	BGIOSGA011232	XP_002438996.1formin-like protein 3
TCONS_00013445	1.18594	0.107955	0.511193	BGIOSGA012944	XP_004981226.1calmodulin-binding protein 60 A isoform X1
TCONS_00027583	1.18638	1.74483	2.03505	BGIOSGA032837	XP_008781484.1 3-hydroxyacyl-[acyl-carrier-protein] dehydratase FabZ-like
TCONS_00028169	1.18643	-2.08531	-1.41963	BGIOSGA013832	XP_004973678.1tuliposide A-converting enzyme 1, chloroplastic
TCONS_00004537	1.18659	1.63695	0.802355	BGIOSGA014933	XP_006644822.1 peroxidase 1-like isoform X2
TCONS_00030560	1.18661	1.24461	1.88803	BGIOSGA026495	XP_006660994.2 probable uridine nucleosidase 2
TCONS_00005293	1.18666	0.518475	1.19647	BGIOSGA010367	XP_006646796.1 GDSL esterase/lipase At5g55050-like
TCONS_00032725	1.18691	0.945792	0.524717	BGIOSGA018418	XP_021311758.1BOI-related E3 ubiquitin-protein ligase 1 isoform X1
TCONS_00016580	1.18725	1.08111	1.5597	BGIOSGA007653	XP_006652788.1PREDICTED: uncharacterized protein LOC102717240 isoform X1
TCONS_00024449	1.18738	0.524592	0.944261	BGIOSGA025029	XP_006648343.1PREDICTED: uncharacterized protein LOC102716192
TCONS_00017207	1.18748	1.34633	1.38511	BGIOSGA002856	XP_006655036.1 autophagy-related protein 18d-like
TCONS_00015494	1.18752	1.39409	1.4261	BGIOSGA008773	XP_015698867.1 probable aquaporin PIP2-6
TCONS_00000032	1.18756	1.26941	2.42556	BGIOSGA017200	XP_004967934.1transcription factor bHLH110 isoform X1
TCONS_00037123	1.18776	-0.167903	0.620133	BGIOSGA029046	XP_006664537.1 sulfite oxidase-like
TCONS_00026879	1.18832	1.79202	0.990714	BGIOSGA012996	XP_015695783.1 pentatricopeptide repeat-containing protein At1g80150, mitochondrial
TCONS_00002965	1.18907	2.01265	1.17328	BGIOSGA002251	XP_015696117.1 DDT domain-containing protein DDR4-like
TCONS_00005573	1.18908	1.03	1.73607	BGIOSGA017000	XP_006646991.2 premnaspirodiene oxygenase-like
TCONS_00032458	1.18913	-0.785027	0.742078	BGIOSGA025921	XP_004982787.1cytochrome P450 86B1
TCONS_00008687	1.1892	1.23313	1.44796	BGIOSGA009821	XP_004953674.13-hydroxy-3-methylglutaryl-coenzyme A reductase 1
TCONS_00009576	1.18924	1.04865	1.09276	BGIOSGA020258	XP_015696667.1 F-box/LRR-repeat protein At4g14103-like
TCONS_00037417	1.18941	1.69335	2.05841	BGIOSGA020936	XP_006664140.2 pentatricopeptide repeat-containing protein At3g29230-like

TCONS_00004250	1.1895	1.32586	1.17542	BGIOSGA008123	XP_015688088.1 phosphatidylinositol/phosphatidylcholine transfer protein SFH6-like
TCONS_00023319	1.1906	#NA	-0.474974	BGIOSGA013779	XP_024313019.1uncharacterized protein LOC112269860
TCONS_00007409	1.19211	2.24853	1.0498	BGIOSGA007064	XP_002453359.14-hydroxyphenylpyruvate dioxygenase
TCONS_00017505	1.19364	2.03037	1.0831	BGIOSGA011003	XP_006654412.1 guanosine nucleotide diphosphate dissociation inhibitor 2
TCONS_00010224	1.19429	0.218588	0.473515	BGIOSGA014991	XP_010491788.1 histone H3.3
TCONS_00029372	1.19621	0.609965	1.09259	BGIOSGA019011	XP_015696474.1 ras-related protein RABA3-like
TCONS_00028482	1.19665	-0.101067	0.370301	BGIOSGA026474	XP_021321181.1bet1-like SNARE 1-1
TCONS_00003785	1.19884	0.550738	1.20645	BGIOSGA001426	XP_006644273.1 peptide deformylase 1A, chloroplastic
TCONS_00028809	1.19934	1.07794	1.01158	BGIOSGA030558	XP_006660550.1PREDICTED: uncharacterized protein LOC102715047
TCONS_00002768	1.19941	0.362987	0.555027	BGIOSGA020313	XP_006645469.1 chloroplastic group IIB intron splicing facilitator CRS2, chloroplastic
TCONS_00010726	1.19954	0.949318	0.997064	BGIOSGA013350	XP_003561186.1uncharacterized protein LOC100820864
TCONS_00011742	1.19975	-0.902684	-1.26359	BGIOSGA011264	XP_006651143.1 adenylyl-sulfate kinase 3
TCONS_00020616	1.20097	-0.826223	0.899242	BGIOSGA006945	XP_004965344.1chlorophyll a-b binding protein 1B-21, chloroplastic
TCONS_00016361	1.20325	1.29352	0.569488	BGIOSGA016313	XP_002448280.1ethylene-responsive transcription factor 2
TCONS_00017268	1.2044	0.121882	0.813741	BGIOSGA017339	XP_006653016.1 ubiquinone biosynthesis protein COQ4 homolog, mitochondrial
TCONS_00020220	1.20526	1.3221	0.363832	BGIOSGA022411	XP_020399691.1cytochrome b-c1 complex subunit 8
TCONS_00021471	1.2056	0.366806	0.270251	BGIOSGA022078	XP_006655706.1 bark storage protein A-like
TCONS_00003299	1.20705	0.962686	1.02055	BGIOSGA001914	XP_006645756.2 ATP-dependent Clp protease proteolytic subunit-related protein 4, chloroplastic
TCONS_00000067	1.20717	-1.94175	0.102234	BGIOSGA002531	XP_015695577.1PREDICTED: uncharacterized protein LOC107304718
TCONS_00005148	1.2103	1.11983	1.08787	BGIOSGA005021	XP_006645350.1 VAMP-like protein YKT61
TCONS_00033488	1.21059	0.976911	0.228668	BGIOSGA021834	XP_006663457.1PREDICTED: uncharacterized protein LOC102717196
TCONS_00014642	1.21096	0.73153	1.60524	BGIOSGA016799	XP_006652546.1 UPF0426 protein At1g28150, chloroplastic isoform X1
TCONS_00023911	1.21167	-0.930437	-0.177271	BGIOSGA014976	XP_006657834.1 chlorophyll a-b binding protein of LHCII type III, chloroplastic
TCONS_00033353	1.21168	-0.0627878	0.851083	BGIOSGA034092	XP_003576076.1exosome complex component RRP43
TCONS_00016306	1.21302	2.39571	1.26135	BGIOSGA025974	XP_006652561.2 L-type lectin-domain containing receptor kinase IV.1-like
TCONS_00028787	1.21469	-0.563295	-0.561198	BGIOSGA013431	XP_006661132.1 submaxillary gland androgen-regulated protein 3A-like
TCONS_00005145	1.21529	0.044731	0.937187	BGIOSGA034879	XP_006645352.1 syntaxin-61
TCONS_00036909	1.21541	0.283937	-0.118384	BGIOSGA029552	XP_015698760.1PREDICTED: uncharacterized protein LOC102711391
TCONS_00036763	1.21557	0.209651	1.15812	BGIOSGA036551	XP_006664375.2 probable sodium/metabolite cotransporter BASS1, chloroplastic
TCONS_00033807	1.21588	0.0123095	0.886038	BGIOSGA002418	XP_015698131.1 peptidyl-prolyl cis-trans isomerase CYP23
TCONS_00026431	1.21619	0.297362	1.51985	BGIOSGA019256	XP_002462138.2phosphoenolpyruvate/phosphat e translocator 1, chloroplastic
TCONS_00000753	1.21634	1.63799	2.13366	BGIOSGA027194	XP_015696176.1PREDICTED: uncharacterized protein At4g37920, chloroplastic
TCONS_00028051	1.21859	0.527611	-0.687629	BGIOSGA026938	XP_002445542.1ATP-dependent Clp protease adapter protein CLPS1, chloroplastic isoform X1
TCONS_00002450	1.2189	0.582551	0.921692	BGIOSGA035577	XP_006645264.1 E3 ubiquitin-protein ligase At3g02290-like isoform X1
TCONS_00002358	1.2192	1.29542	0.831141	BGIOSGA004996	XP_006646576.1 probable thimet oligopeptidase
TCONS_00004166	1.21932	0.371023	0.203227	BGIOSGA023299	XP_006644510.1 peroxiredoxin-2C
TCONS_00013876	1.22013	1.75392	0.725718	BGIOSGA016055	XP_003579514.1CASP-like protein 2A1
TCONS_00011061	1.22047	1.36919	2.0643	BGIOSGA013659	XP_010229022.1calmodulin-binding protein 60 D isoform X2
TCONS_00002328	1.22079	0.697136	0.231811	BGIOSGA038187	XP_006645155.1 putative metallophosphoesterase At3g03305 isoform X1
TCONS_00030685	1.22096	2.16426	1.45199	BGIOSGA032505	XP_006660834.1 UPF0664 stress-induced protein C29B12.11c
TCONS_00010863	1.22186	1.74862	1.86732	BGIOSGA027574	XP_006650517.1 BTB/POZ domain-containing protein At1g63850-like
TCONS_00020254	1.22271	2.41553	0.976581	BGIOSGA013385	XP_015694416.1 serine/arginine-rich splicing factor RSZ21A isoform X1
TCONS_00032817	1.22286	-3.80268	-0.155152	BGIOSGA002203	XP_006663716.1 protein trichome birefringence-like 34
TCONS_00020408	1.22294	-0.712633	0.634717	BGIOSGA012085	XP_024313389.1uncharacterized protein LOC100833226
TCONS_00004912	1.22353	1.65269	1.5278	BGIOSGA037224	XP_003564861.1probable carboxylesterase 16
TCONS_00030014	1.22382	0.201132	0.286024	BGIOSGA032873	XP_004956770.1putative 1-phosphatidylinositol-3-phosphate 5-kinase FAB1C
TCONS_00006122	1.22399	-0.0654112	-0.622267	BGIOSGA015071	NP_001147349.1drought-induced protein 1 precursor
TCONS_00023403	1.22417	2.73577	1.02577	BGIOSGA028193	XP_006658427.1 UDP-glycosyltransferase 76F1-like
TCONS_00009966	1.22469	2.05379	1.89471	BGIOSGA012520	XP_015689872.1 guanylate kinase 2, chloroplastic/mitochondrial
TCONS_00022191	1.22525	1.13756	1.1704	BGIOSGA021307	XP_004965459.1translation initiation factor eIF-2B subunit gamma
TCONS_00035471	1.22572	-0.187184	-0.283814	BGIOSGA023430	XP_009411192.2 RING-H2 finger protein ATL73
TCONS_00025362	1.22644	0.016953	0.630358	BGIOSGA024068	XP_006658708.1 cytochrome c6, chloroplastic
TCONS_00031861	1.22836	0.459351	1.03693	BGIOSGA030275	XP_006661643.1 aldose 1-epimerase-like

TCONS_00027155	1.2284	1.22497	1.15756	BGIOSGA029201	XP_006659705.1 superoxide dismutase [Cu-Zn], chloroplastic
TCONS_00035699	1.22866	1.33354	0.279931	BGIOSGA037114	XP_003576984.1uncharacterized protein LOC100835766
TCONS_00005221	1.22886	2.57203	1.91959	BGIOSGA022066	XP_021315832.1ruBisCO large subunit-binding protein subunit beta, chloroplastic-like
TCONS_00023209	1.22972	1.325	1.46003	BGIOSGA025250	XP_006658334.1 pentatricopeptide repeat-containing protein At4g20740
TCONS_00029246	1.22976	1.04008	1.98776	BGIOSGA030988	XP_006660753.1PREDICTED: uncharacterized protein LOC102705839
TCONS_00002746	1.23172	0.996261	1.89787	BGIOSGA002471	XP_015692585.1 protein ABCI7, chloroplastic
TCONS_00033737	1.23203	0.871244	1.49647	BGIOSGA037408	XP_015697706.1 50S ribosomal protein L4
TCONS_00021208	1.23208	0.948202	1.89166	BGIOSGA035307	XP_006657229.1 VQ motif-containing protein 22-like
TCONS_00028579	1.23225	0.882865	0.512441	BGIOSGA018932	XP_006661039.1 probable plastid-lipid-associated protein 2, chloroplastic
TCONS_00011548	1.23234	2.56228	2.40604	BGIOSGA017649	XP_006651029.1 chaperonin CPN60-1, mitochondrial-like
TCONS_00005705	1.23362	2.48406	1.41473	BGIOSGA007826	XP_023155958.1endoribonuclease YBEY, chloroplastic isoform X1
TCONS_00012729	1.23476	1.76602	1.23198	BGIOSGA033194	XP_006650307.1 probable N-acetyl-gamma-glutamyl-phosphate reductase, chloroplastic
TCONS_00006696	1.2358	-0.0763458	-0.607608	BGIOSGA014433	XP_006647733.1 phosphoribulokinase, chloroplastic
TCONS_00009706	1.23741	0.548717	1.47722	BGIOSGA012255	XP_006649771.1PREDICTED: uncharacterized protein DDB_G0275933
TCONS_00031440	1.23923	0.481308	1.12232	BGIOSGA037819	XP_021302625.1uncharacterized protein LOC8065181
TCONS_00011954	1.23958	1.91633	0.998633	BGIOSGA011057	XP_006651237.1 ASC1-like protein 3
TCONS_00001628	1.23979	0.273504	0.241882	BGIOSGA006576	XP_006644563.2 ABC transporter B family member 4-like
TCONS_00010498	1.23985	1.50833	0.756955	BGIOSGA013108	XP_009621401.1 serine protease HTRA1-like isoform X3
TCONS_00017551	1.2409	0.81016	0.948508	BGIOSGA019625	XP_006654262.1 superoxide dismutase [Mn], mitochondrial
TCONS_00009119	1.24092	1.91463	0.879809	BGIOSGA004785	XP_006649220.2PREDICTED: uncharacterized protein LOC102721622
TCONS_00027085	1.24136	0.681699	-0.0396067	#N/A	#N/A
TCONS_00023420	1.24154	1.83272	1.30702	BGIOSGA015163	XP_006658437.1 calreticulin
TCONS_00007492	1.24173	2.16102	-0.858391	BGIOSGA025531	XP_003571042.4putative UDP-glucose glucosyltransferase
TCONS_00024064	1.24182	2.92455	1.29261	BGIOSGA037241	XP_006658788.2 flavonol sulfotransferase-like
TCONS_00000401	1.24228	2.94584	0.906192	BGIOSGA027082	XP_006643893.2 SCAR-like protein 2
TCONS_00006264	1.24273	0.974573	-0.288658	BGIOSGA008425	XP_006664957.1PREDICTED: uncharacterized protein At1g32220, chloroplastic
TCONS_00032812	1.24336	-4.1108	0.610747	BGIOSGA009561	XP_006663716.1 protein trichome birefringence-like 34
TCONS_00002332	1.24362	2.11847	1.32157	BGIOSGA004973	XP_015699273.1 RHOMBOID-like protein 13
TCONS_00027198	1.24428	1.90227	1.20591	BGIOSGA025826	XP_006659023.1 transmembrane protein 18-like
TCONS_00000384	1.24495	-0.0522922	0.875443	#N/A	#N/A
TCONS_00031381	1.24562	1.75601	0.91063	BGIOSGA004069	XP_010234860.1WAT1-related protein At3g02690, chloroplastic isoform X1
TCONS_00008007	1.24608	2.06545	1.74489	BGIOSGA006471	XP_015689014.1PREDICTED: uncharacterized protein LOC102718711 isoform X2
TCONS_00032887	1.24737	#NA	-1.59463	BGIOSGA010812	XP_015698595.1 probable apyrase 3
TCONS_00001894	1.24774	3.01971	2.19452	BGIOSGA004528	XP_006646367.2 protein PLASTID TRANSCRIPTIONALLY ACTIVE 12
TCONS_00033309	1.24895	-1.71931	0.393659	BGIOSGA005584	XP_002452518.1uncharacterized protein LOC8066205
TCONS_00013526	1.2492	-0.101067	-2.00708	BGIOSGA009456	XP_015690491.1 30S ribosomal protein S1
TCONS_00032126	1.25046	1.69824	0.758606	BGIOSGA015024	XP_024315508.1red chlorophyll catabolite reductase isoform X1
TCONS_00006678	1.25055	0.23292	0.974565	BGIOSGA008847	XP_002454446.1uncharacterized protein LOC8073893
TCONS_00013442	1.25159	0.0670458	0.707498	BGIOSGA022659	XP_006651971.2 protein NRT1/ PTR FAMILY 5.6-like
TCONS_00033684	1.25184	#NA	-1.46722	BGIOSGA001262	XP_006663675.1 chalcone synthase-like
TCONS_00037350	1.25393	1.77295	1.48182	BGIOSGA036018	XP_014758823.1putative pentatricopeptide repeat-containing protein At3g16890, mitochondrial
TCONS_00021368	1.2563	1.07884	-0.325239	BGIOSGA005853	XP_003563555.1B-box zinc finger protein 20
TCONS_00007789	1.25665	1.54	0.524391	BGIOSGA036421	XP_004952236.1lactoylglutathione lyase GLX1
TCONS_00031951	1.25801	0.854295	1.06283	#N/A	XP_006662246.2PREDICTED: uncharacterized protein LOC102708835
TCONS_00018253	1.25867	1.07585	1.57576	BGIOSGA010573	XP_006655549.2 pentatricopeptide repeat-containing protein At1g07740, mitochondrial
TCONS_00028202	1.25899	1.09261	1.90353	BGIOSGA004605	XP_006660227.1 29 kDa ribonucleoprotein A, chloroplastic-like
TCONS_00002626	1.2602	0.603253	1.17085	BGIOSGA037164	XP_006645329.1 cytochrome b5-like
TCONS_00018343	1.26056	0.637184	0.879532	BGIOSGA027613	XP_006659149.1 THO complex subunit 7B-like isoform X2
TCONS_00018320	1.26138	1.78799	1.75539	BGIOSGA020373	XP_006654798.1PREDICTED: uncharacterized protein LOC102700432
TCONS_00026453	1.26195	0.311311	-1.15862	BGIOSGA028523	XP_006660082.2 thylakoid membrane protein slr0575
TCONS_00014328	1.26236	0.839308	1.339	BGIOSGA006948	XP_021319005.1pentatricopeptide repeat-containing protein At1g09900 isoform X1
TCONS_00004605	1.26475	0.229796	1.77889	BGIOSGA022536	XP_022682969.1ABSCISIC ACID-INSENSITIVE 5-like protein 2
TCONS_00009974	1.2658	-0.248837	0.742754	BGIOSGA037773	XP_006650011.1PREDICTED: uncharacterized protein LOC102713059
TCONS_00004400	1.26599	-0.119687	1.24788	BGIOSGA002024	XP_006644711.1 GDSL esterase/lipase At4g10955-like
TCONS_00028458	1.26638	0.380972	0.764221	BGIOSGA000183	XP_008678430.1manganese-dependent ADP-ribose/CDP-alcohol diphosphatase

TCONS_00004478	1.26706	1.35027	0.403928	BGIOSGA007536	XP_022678517.1tyrosine decarboxylase 1 isoform X2
TCONS_00026414	1.26753	0.269662	0.929895	BGIOSGA028481	XP_015696095.1PREDICTED: uncharacterized protein LOC102702586 isoform X1
TCONS_00019463	1.26805	1.17879	0.302346	BGIOSGA017888	XP_002451615.2uncharacterized protein LOC8076115
TCONS_00024811	1.2693	-0.0732922	0.633416	BGIOSGA008351	XP_015694577.1 THO complex subunit 4B-like
TCONS_00018842	1.26976	2.31162	2.6857	#N/A	#N/A
TCONS_00024644	1.2715	1.97972	1.99129	BGIOSGA024743	XP_006658314.1 ADP-glucose phosphorylase
TCONS_00018273	1.27218	1.11714	1.90306	BGIOSGA020181	XP_006655573.2PREDICTED: uncharacterized protein LOC102701270
TCONS_00026632	1.27234	0.652116	1.46814	BGIOSGA012082	XP_003572204.1chaperonin-like RBCX protein 1, chloroplastic isoform X1
TCONS_00020092	1.27332	-0.0845837	0.456261	BGIOSGA022272	XP_006656619.1 homeobox-leucine zipper protein HOX2-like
TCONS_00027622	1.27364	1.17804	2.20547	BGIOSGA027396	XP_004972490.1AUGMIN subunit 1
TCONS_00033761	1.27375	1.45122	1.00157	BGIOSGA032051	XP_015697708.1 GTPase HflX
TCONS_00008110	1.2758	0.944592	-0.201978	BGIOSGA019946	XP_006647333.1 60S acidic ribosomal protein P2A-like
TCONS_00001442	1.27668	3.50326	1.04982	BGIOSGA020384	XP_006644418.1PREDICTED: uncharacterized protein LOC102701034
TCONS_00019970	1.27693	-1.20333	0.0181064	BGIOSGA022158	XP_006656551.2 homeobox protein BEL1 homolog
TCONS_00020685	1.27746	-2.32625	-0.174871	BGIOSGA022879	XP_006656957.1 GDSL esterase/lipase EXL1-like
TCONS_00004047	1.27808	1.12315	1.08142	BGIOSGA001147	XP_015693482.1 peptide deformylase 1B, chloroplastic
TCONS_00030807	1.27961	3.3005	1.89569	BGIOSGA032615	XP_003564521.1thioredoxin-like fold domain-containing protein MRL7 homolog, chloroplastic
TCONS_00030273	1.28035	3.09086	3.30074	BGIOSGA005513	XP_006661382.1 heat shock 70 kDa protein, mitochondrial-like
TCONS_00020079	1.28067	2.21836	1.78626	BGIOSGA022259	XP_004964480.1probable ribosome-binding factor A, chloroplastic
TCONS_00013556	1.28123	1.25444	1.83785	BGIOSGA009419	XP_006650916.2 cleavage and polyadenylation specificity factor subunit 3-I
TCONS_00015112	1.28173	0.904807	0.363773	BGIOSGA017292	XP_010240553.1probable inactive UDP-arabinopyranose mutase 2 isoform X2
TCONS_00035760	1.28296	0.0742349	1.12532	BGIOSGA037171	XP_003578767.1outer envelope pore protein 37, chloroplastic isoform X2
TCONS_00025053	1.28404	0.89776	1.50731	BGIOSGA024354	XP_006657672.1 DNA-directed RNA polymerases II, IV and V subunit 6A-like
TCONS_00031426	1.2841	0.504569	0.350253	BGIOSGA033240	XP_006662497.1 valine--tRNA ligase, mitochondrial 1-like
TCONS_00014476	1.28437	2.11576	1.33357	BGIOSGA007628	XP_006652435.1 glyceraldehyde-3-phosphate dehydrogenase 2, cytosolic
TCONS_00002434	1.28485	1.77339	-0.01976	BGIOSGA026745	XP_004971093.1MADS-box transcription factor 51
TCONS_00033139	1.28562	-1.38779	0.451534	BGIOSGA037103	XP_006663273.1PREDICTED: uncharacterized protein LOC102705477
TCONS_00022334	1.28608	2.24469	0.888198	BGIOSGA025174	XP_006656114.1 peroxidase P7-like
TCONS_00005535	1.287	0.927066	2.07625	BGIOSGA007644	XP_015688469.1 secreted acidic protein 1A-like
TCONS_00029379	1.28737	-0.621764	0.360947	BGIOSGA023605	XP_006660868.1 rhodanese-like domain-containing protein 14, chloroplastic isoform X2
TCONS_00017244	1.28748	1.93212	2.44683	BGIOSGA011365	XP_015692502.1PREDICTED: uncharacterized protein LOC107304167
TCONS_00019864	1.28942	0.846327	1.34325	BGIOSGA022559	XP_006654833.1PREDICTED: uncharacterized protein LOC102711702
TCONS_00007616	1.29144	1.28435	1.49265	BGIOSGA006861	XP_006648460.1 putative GPI-anchor transamidase
TCONS_00017930	1.29285	-0.434107	0.651779	BGIOSGA030356	XP_006654505.1 CASP-like protein 5B2 isoform X1
TCONS_00019268	1.29325	0.586511	0.918267	BGIOSGA018079	XP_006654384.1 methyl-CpG-binding domain-containing protein 1-like
TCONS_00028462	1.29349	2.3593	1.26165	BGIOSGA001257	XP_006659692.1 probable calcium-binding protein CML48
TCONS_00029044	1.29372	1.28597	1.34912	BGIOSGA023388	XP_003578148.1protein ECERIFERUM 3
TCONS_00001823	1.29706	0.57294	1.07109	BGIOSGA011916	XP_006644706.1 heat stress transcription factor A-4b
TCONS_00029068	1.29832	1.21115	1.62523	BGIOSGA014467	NP_001183875.1uncharacterized LOC100275491
TCONS_00024610	1.30034	1.47446	-0.768036	BGIOSGA034236	XP_022679921.1probable carboxylesterase 15 isoform X1
TCONS_00018337	1.30216	1.45251	1.929	BGIOSGA020388	XP_003567857.1uncharacterized protein LOC100835838
TCONS_00020958	1.3037	0.294522	0.724557	#N/A	#N/A
TCONS_00028064	1.30407	1.29412	1.18305	#N/A	#N/A
TCONS_00008219	1.30452	1.7594	1.6519	BGIOSGA015504	XP_006647334.1 ent-cassadiene C2-hydroxylase-like
TCONS_00010007	1.30467	0.16442	-0.0737432	BGIOSGA012557	XP_021310391.1protein transport protein yos1
TCONS_00016712	1.30477	-2.15027	-0.613269	BGIOSGA009055	XP_006652893.1 shikimate kinase 3, chloroplastic
TCONS_00004974	1.30482	0.548676	0.61018	BGIOSGA000243	XP_006645232.1 small ubiquitin-related modifier 1-like isoform X1
TCONS_00035344	1.30492	1.88114	1.48279	BGIOSGA036020	XP_006663101.1 transmembrane protein 205-like
TCONS_00018695	1.30581	1.19915	0.78634	BGIOSGA002310	XP_006654091.1 thioredoxin H4-2
TCONS_00011200	1.3067	1.42227	1.51472	BGIOSGA013801	XP_006650779.1 splicing factor 3B subunit 6-like protein
TCONS_00027288	1.30687	0.513358	1.10949	BGIOSGA027712	XP_006659087.1PREDICTED: uncharacterized protein LOC102702399 isoform X2
TCONS_00028219	1.30687	0.0731887	0.658137	BGIOSGA029710	XP_003574677.1alpha/beta hydrolase domain-containing protein 17A
TCONS_00019789	1.30695	0.582808	2.04363	BGIOSGA017570	XP_006654782.1 3-deoxy-manno-octulosonate cytidylyltransferase, mitochondrial
TCONS_00037568	1.30695	-0.308844	-0.660663	BGIOSGA024204	XP_002443621.1thaumatin-like protein

TCONS_00033774	1.30915	0.889934	0.12809	BGIOSGA030424	XP_015697712.1 probable plastid-lipid-associated protein 6, chloroplastic
TCONS_00016873	1.30949	2.494	0.127077	BGIOSGA027376	XP_004960074.1NDR1/HIN1-like protein 6
TCONS_00023660	1.30989	0.900522	0.254065	BGIOSGA031387	XP_006657689.1 thioredoxin-like protein CDSP32, chloroplastic
TCONS_00017502	1.31052	2.83519	1.71922	BGIOSGA005513	XP_008644594.1stromal 70 kDa heat shock-related protein, chloroplastic
TCONS_00003995	1.31075	0.0211822	1.36021	BGIOSGA016144	XP_006644373.2 probable aldo-keto reductase 1
TCONS_00013748	1.311	0.823983	2.13037	BGIOSGA015511	XP_006652113.1 putative amidase C869.01
TCONS_00003211	1.31191	0.525712	1.43091	BGIOSGA002005	XP_006643988.1 protein NLP2-like
TCONS_00022738	1.31267	1.45463	1.50919	BGIOSGA007624	XP_006656352.1 glycine cleavage system H protein 2, mitochondrial-like isoform X2
TCONS_00004474	1.31285	2.37196	1.16081	BGIOSGA029215	XP_024311908.1exocyst complex component EXO70B1-like
TCONS_00024095	1.3134	0.181893	-0.416435	BGIOSGA009804	XP_006658807.1 protein STRICTOSIDINE SYNTHASE-LIKE 4-like
TCONS_00026990	1.31353	1.02138	1.26611	BGIOSGA031046	XP_006659590.1 proteasome subunit beta type-1-like
TCONS_00005836	1.31372	2.59055	1.23205	BGIOSGA007002	XP_003581127.1premnaspirodiene oxygenase
TCONS_00036933	1.31594	0.766264	1.09952	BGIOSGA012378	XP_006664463.2 putative ribosome biogenesis protein slx9-like
TCONS_00023737	1.31595	-1.13472	0.226536	BGIOSGA025770	XP_015695140.1 filament-like plant protein 3
TCONS_00014054	1.31606	1.74012	1.39366	BGIOSGA025331	XP_006647576.1 putative ripening-related protein 2
TCONS_00008470	1.31813	0.278155	2.27297	BGIOSGA031595	XP_006648879.1 remorin-like isoform X2
TCONS_00024861	1.32089	-0.855962	1.49484	BGIOSGA006475	XP_022685294.1uncharacterized protein LOC111258351
TCONS_00000795	1.32147	#NA	-0.541545	BGIOSGA009996	XP_006644125.1 probable receptor-like protein kinase At5g18500
TCONS_00000230	1.32151	1.73332	1.26447	BGIOSGA032805	XP_006645505.1 putative receptor-like protein kinase At3g47110
TCONS_00021636	1.32174	-0.141429	1.76641	BGIOSGA021884	XP_003564316.1probable thiol methyltransferase 2
TCONS_00004304	1.32192	1.68797	3.35013	BGIOSGA031779	XP_006644631.1 E3 ubiquitin-protein ligase MIEL1-like
TCONS_00001071	1.32309	1.90933	1.63492	BGIOSGA001360	XP_015699356.1 monothiol glutaredoxin-S4, mitochondrial
TCONS_00035764	1.32431	#NA	-0.520114	BGIOSGA037175	XP_015698837.1 2-alkenal reductase (NADP(+)-dependent)-like
TCONS_00008467	1.32636	-1.92486	-0.873108	BGIOSGA033592	XP_006647586.1 membrane steroid-binding protein 2
TCONS_00030256	1.32835	0.435328	-0.827243	BGIOSGA001199	XP_015696615.1 glucosamine 6-phosphate N-acetyltransferase 1
TCONS_00003899	1.32859	1.37571	1.62787	BGIOSGA020261	XP_015689148.1 30S ribosomal protein S1-like
TCONS_00018995	1.329	0.76809	1.03144	BGIOSGA005656	XP_006654232.1 pentatricopeptide repeat-containing protein At5g50280, chloroplastic
TCONS_00002122	1.32989	1.728	1.5052	BGIOSGA004750	XP_006646456.2 zinc finger CCCH domain-containing protein 11
TCONS_00017217	1.33222	1.01878	2.99834	BGIOSGA016843	NP_001149571.1lactoylglutathione lyase
TCONS_00013125	1.33408	2.75503	1.75928	BGIOSGA009860	XP_015690057.1 endoplasmic reticulum oxidoreductin-1
TCONS_00037378	1.33426	1.51638	1.44591	BGIOSGA023596	XP_015698738.1 UNC93-like protein 3 isoform X2
TCONS_00008811	1.33482	1.00705	1.38588	BGIOSGA005618	XP_002466372.1dolichol-phosphate mannosyltransferase subunit 3
TCONS_00021854	1.33656	1.97449	1.02328	BGIOSGA012744	XP_006656773.1 peptidyl-prolyl cis-trans isomerase CYP40-like
TCONS_00024707	1.33672	3.3007	1.32925	BGIOSGA019069	XP_003557425.1hydroxyproline O-galactosyltransferase GALT5
TCONS_00028499	1.33696	0.613664	1.92039	BGIOSGA026458	XP_006660404.2 exocyst complex component EXO84C-like
TCONS_00018090	1.33704	1.68171	0.617253	BGIOSGA002618	XP_006654613.2 protein SULFUR DEFICIENCY-INDUCED 1-like
TCONS_00032128	1.33843	-1.74505	0.595725	BGIOSGA003356	XP_006661732.1 protein STRUBBELIG-RECEPTOR FAMILY 7-like
TCONS_00026127	1.33868	0.325377	1.04882	#N/A	#N/A
TCONS_00027402	1.33978	-3.11978	-1.07585	BGIOSGA022061	XP_006659159.1 acyl-CoA-binding protein
TCONS_00036308	1.33984	1.82828	1.90518	BGIOSGA031868	XP_008805368.1 TATA-box-binding protein
TCONS_00012990	1.33992	1.00593	0.28122	BGIOSGA000064	XP_006651717.1 collagen alpha-2(I) chain-like
TCONS_00005528	1.34006	2.57904	1.47188	BGIOSGA034053	XP_003570281.1calmodulin-binding protein 25
TCONS_00023142	1.34093	-1.90511	-0.421121	#N/A	#N/A
TCONS_00012859	1.34266	-8.22271	-0.799348	BGIOSGA036357	XP_006650369.2 cytochrome P450 87A3-like
TCONS_00010620	1.34277	2.20126	1.83399	BGIOSGA004844	XP_006651651.2 N utilization substance protein B homolog
TCONS_00004825	1.34305	-4.97527	-0.483406	BGIOSGA012548	XP_006646530.1 extradiol ring-cleavage dioxygenase-like
TCONS_00007310	1.34426	2.41081	2.22372	BGIOSGA007878	XP_009381199.1 transcription elongation factor 1 homolog
TCONS_00031619	1.34433	1.92942	2.1956	BGIOSGA026016	XP_006664062.1 polyol transporter 5-like
TCONS_00036741	1.34453	1.41462	1.72562	BGIOSGA034861	XP_015698804.1PREDICTED: uncharacterized protein LOC102711209
TCONS_00009478	1.34485	-1.33313	-0.359649	BGIOSGA032057	XP_015690893.1 probable protein phosphatase 2C BIPP2C1
TCONS_00006460	1.34551	1.06756	-0.898267	BGIOSGA020782	XP_015689140.1 glutaredoxin-C4, chloroplastic
TCONS_00028460	1.34587	2.05316	1.25218	BGIOSGA019233	XP_006659685.1 putative low molecular weight protein-tyrosine-phosphatase slr0328
TCONS_00007579	1.34619	-0.444946	-0.391886	BGIOSGA004479	XP_015689906.1 transcription factor PCF7-like
TCONS_00011863	1.3464	1.60335	1.60153	BGIOSGA032373	XP_006649704.2PREDICTED: uncharacterized protein LOC102720214
TCONS_00011817	1.34755	1.05926	0.689025	BGIOSGA021312	XP_003558451.1mitogen-activated protein kinase kinase 9
TCONS_00017199	1.34772	-0.0179559	0.39558	BGIOSGA005460	XP_006655023.1 E3 ubiquitin-protein ligase ATL41

TCONS_00003170	1.34898	1.06876	0.984437	BGIOSGA002051	XP_006643971.1 probable 2-carboxy-D-arabinitol-1-phosphatase
TCONS_00029904	1.35013	0.518865	0.961173	BGIOSGA029902	XP_006660566.1PREDICTED: uncharacterized protein At4g33100
TCONS_00008955	1.35056	1.43265	1.43915	BGIOSGA009053	XP_006649124.1 zinc finger CCCH domain-containing protein 18
TCONS_00009071	1.35211	0.469461	-0.200884	#N/A	#N/A
TCONS_00019797	1.3534	0.403856	2.00278	BGIOSGA017563	XP_015689720.1 E3 SUMO-protein ligase MMS21-like
TCONS_00002944	1.35402	-1.46471	-1.0663	BGIOSGA018709	XP_006643819.1 boron transporter 4-like
TCONS_00007023	1.35536	2.02431	1.69832	BGIOSGA030760	XP_002452981.1probable CCR4-associated factor 1 homolog 7
TCONS_00014067	1.35605	0.00818119	0.934749	BGIOSGA034355	XP_015691738.1 probable magnesium transporter NIPA2 isoform X1
TCONS_00025611	1.35616	0.902522	1.5355	BGIOSGA035873	XP_006658890.1 ATP synthase mitochondrial F1 complex assembly factor 2
TCONS_00035810	1.35656	1.29294	1.31682	BGIOSGA011623	XP_002442071.1tropomyosin-1, isoforms 33/34
TCONS_00019233	1.35658	2.45046	1.5798	BGIOSGA033171	XP_004962186.1uncharacterized protein LOC101784684
TCONS_00030275	1.35667	1.23622	1.5286	BGIOSGA031916	XP_006660780.1 cinnamoyl-CoA reductase 1-like
TCONS_00017224	1.35694	2.67949	1.31588	BGIOSGA023659	XP_002464354.1F-box protein At3g49450
TCONS_00000476	1.3572	2.5459	1.31523	BGIOSGA023345	XP_004967424.1cytochrome P450 71A1
TCONS_00019807	1.35754	-0.705159	0.277515	BGIOSGA017548	XP_004961194.1cold-regulated 413 inner membrane protein 1, chloroplastic
TCONS_00013290	1.35776	0.590998	1.53624	BGIOSGA001453	XP_003558095.1DNA-binding protein HEXBP
TCONS_00003904	1.35814	0.290957	1.1609	BGIOSGA034723	XP_002441721.1uncharacterized protein LOC8071415 isoform X1
TCONS_00015922	1.35817	1.33733	1.21283	BGIOSGA015063	XP_006652308.1 glutathione S-transferase TCHQD
TCONS_00006507	1.35838	2.67768	2.19984	BGIOSGA020314	XP_006647564.1 pentatricopeptide repeat-containing protein At4g36680, mitochondrial-like
TCONS_00011875	1.35865	3.65008	1.55264	BGIOSGA020241	XP_003558360.1protein EARLY-RESPONSIVE TO DEHYDRATION 7, chloroplastic
TCONS_00012773	1.35901	2.20414	0.310761	BGIOSGA001222	XP_002464205.1pentatricopeptide repeat-containing protein At2g42920, chloroplastic
TCONS_00001560	1.35958	2.40206	1.7126	BGIOSGA017614	XP_008675201.1uncharacterized abhydrolase domain-containing protein DDB_G0269086
TCONS_00027284	1.36075	2.06957	2.65658	BGIOSGA031689	XP_015695483.1PREDICTED: uncharacterized protein LOC102702775, partial
TCONS_00019092	1.36092	0.927152	1.12166	BGIOSGA018265	XP_006655209.1 ras-related protein Rab-21-like
TCONS_00008471	1.36106	1.32613	1.51045	BGIOSGA027817	XP_006647595.1 probable ascorbate-specific transmembrane electron transporter 1
TCONS_00029077	1.36153	0.98623	-0.566133	BGIOSGA030823	XP_015696522.1PREDICTED: uncharacterized protein LOC102719231
TCONS_00036791	1.36246	0.899779	1.06877	BGIOSGA036526	XP_015698848.1 NAD-dependent protein deacylase SRT2
TCONS_00001757	1.36343	0.636773	0.729814	BGIOSGA017684	XP_002456291.1protein CAJ1
TCONS_00025960	1.36531	0.0493576	0.967645	BGIOSGA011423	NP_001143378.1uncharacterized LOC100276012
TCONS_00037497	1.36617	2.60986	2.01701	BGIOSGA022578	XP_006664180.2 thiosulfate/3-mercaptopyruvate sulfurtransferase 2-like
TCONS_00014876	1.36641	0.908935	2.23634	BGIOSGA027685	XP_003580460.1multiple organellar RNA editing factor 2, chloroplastic
TCONS_00011870	1.3666	2.36819	1.10902	BGIOSGA036721	XP_006649710.1PREDICTED: uncharacterized protein LOC102721815
TCONS_00016830	1.36771	-0.134492	1.07143	BGIOSGA021494	NP_001148189.2ethylene-responsive transcription factor 3
TCONS_00026365	1.36803	0.276343	-0.149759	BGIOSGA030223	XP_015696177.1 CBS domain-containing protein CBSX1, chloroplastic-like
TCONS_00023022	1.36868	0.558909	0.79313	BGIOSGA025051	XP_015694981.1PREDICTED: uncharacterized protein LOC102719598
TCONS_00015351	1.36874	#NA	0.000655194	BGIOSGA024204	XP_004975059.1thaumatin-like protein 1b
TCONS_00015163	1.36967	0.95265	1.07535	BGIOSGA017345	XP_015691795.1 thioredoxin X, chloroplastic
TCONS_00014116	1.3712	0.164497	1.62283	BGIOSGA016269	XP_014751692.1MADS-box transcription factor 51
TCONS_00029976	1.37132	3.72586	2.56504	BGIOSGA025973	XP_010238141.1zinc finger AN1 domain-containing stress-associated protein 17
TCONS_00036773	1.37254	0.469143	1.25551	BGIOSGA034389	XP_006664386.2 protein-tyrosine-phosphatase PTP1-like isoform X1
TCONS_00009896	1.37346	0.65232	1.70475	BGIOSGA025557	XP_014755546.1anaphase-promoting complex subunit 11
TCONS_00009718	1.37421	1.07413	1.44359	BGIOSGA012267	XP_006649782.1 dnaJ homolog subfamily C member 8
TCONS_00027014	1.37542	2.09519	1.41045	BGIOSGA031063	XP_006660328.2 pyruvate dehydrogenase E1 component subunit beta-1, mitochondrial isoform X1
TCONS_00017583	1.37663	1.76939	2.11566	BGIOSGA010916	XP_006654273.1PREDICTED: uncharacterized protein LOC102714375
TCONS_00029237	1.37675	1.18067	0.363773	BGIOSGA011227	XP_006660748.1 caffeoyl-CoA O-methyltransferase-like
TCONS_00023512	1.37688	0.45752	0.459354	BGIOSGA000047	XP_008651270.1uncharacterized protein LOC100278270 isoform X1
TCONS_00011928	1.37699	1.91517	2.50205	BGIOSGA011084	XP_015690296.1 protein CHAPERONE-LIKE PROTEIN OF POR1, chloroplastic
TCONS_00017093	1.37727	0.946631	-1.12938	BGIOSGA015989	XP_004960419.1acyl transferase 4
TCONS_00028002	1.37762	0.796488	0.930022	BGIOSGA033493	XP_021320671.1uncharacterized methyltransferase At1g78140, chloroplastic isoform X2
TCONS_00034324	1.37966	1.91267	-0.333728	BGIOSGA033385	NP_001145027.2uncharacterized protein LOC100278201
TCONS_00007105	1.37975	-1.75216	0.238915	BGIOSGA033374	XP_006648108.2 CLIP-associated protein-like isoform X4
TCONS_00032802	1.37997	-0.565837	-0.161387	BGIOSGA017396	XP_002460024.1uncharacterized protein LOC8071893

TCONS_00001567	1.38099	0.663993	0.246442	BGIOSGA004200	XP_003569557.1uncharacterized protein LOC100836458
TCONS_00018848	1.38106	-0.375527	2.18064	BGIOSGA004017	XP_006645800.1 universal stress protein A-like protein
TCONS_00001793	1.38218	2.33609	3.44715	BGIOSGA008160	XP_006644680.1 glucan endo-1,3-beta-glucosidase 13 isoform X1
TCONS_00009791	1.38325	3.32254	1.28215	BGIOSGA025900	XP_003547405.1histone H2A
TCONS_00008259	1.38412	1.32952	0.223859	BGIOSGA014920	XP_003572663.1uncharacterized protein LOC100844997
TCONS_00035189	1.3855	1.27407	0.767316	#N/A	#N/A
TCONS_00011728	1.38776	0.519143	0.581113	BGIOSGA021330	XP_010229038.1probable serine acetyltransferase 4
TCONS_00008779	1.38896	1.60062	0.682474	BGIOSGA016812	NP_001130472.1putative galacturonosyltransferase-like 9 precursor
TCONS_00000146	1.39023	0.00252222	0.112754	BGIOSGA002719	XP_006645471.2 delta(7)-sterol-C5(6)-desaturase-like
TCONS_00016768	1.39134	0.783427	0.361457	#N/A	#N/A
TCONS_00013607	1.39167	-0.28805	1.15616	BGIOSGA040505	XP_006664926.1 glyoxylate/hydroxypyruvate reductase HPR3-like
TCONS_00024004	1.39244	2.0014	1.20293	BGIOSGA026028	XP_002460943.1U-box domain-containing protein 4
TCONS_00013014	1.39538	0.34808	0.516745	BGIOSGA009974	XP_006650450.1PREDICTED: uncharacterized protein LOC102701429
TCONS_00026913	1.39539	2.68358	1.04362	BGIOSGA032653	XP_004973644.1cytochrome P450 76M5
TCONS_00012111	1.39619	0.0232105	1.02721	#N/A	#N/A
TCONS_00008942	1.39681	2.15332	2.47984	BGIOSGA000813	XP_008643505.1uncharacterized LOC100280095 isoform X1
TCONS_00006453	1.39684	-3.62722	0.502402	BGIOSGA029132	XP_006647515.1 filament-like plant protein 7
TCONS_00035733	1.397	0.114922	0.693075	BGIOSGA021314	XP_004960785.2L10-interacting MYB domain-containing protein isoform X1
TCONS_00007092	1.39799	0.982425	-1.00983	BGIOSGA021497	XP_003570371.13-ketoacyl-CoA synthase 12
TCONS_00001330	1.3985	1.63521	1.101	BGIOSGA003964	XP_015689235.1 pentatricopeptide repeat-containing protein At5g55740, chloroplastic-like
TCONS_00020977	1.39863	0.124556	-0.808936	BGIOSGA023200	XP_015693626.1PREDICTED: uncharacterized protein LOC102710506
TCONS_00034028	1.3989	#NA	#NA	BGIOSGA033557	XP_015697907.1PREDICTED: uncharacterized protein LOC102719718
TCONS_00005509	1.39943	2.19883	2.29402	BGIOSGA039286	XP_006646936.1 phosphoglycerate kinase, cytosolic
TCONS_00004521	1.39983	1.51817	1.53627	BGIOSGA027553	XP_015699166.1 diacylglycerol kinase 5-like
TCONS_00031697	1.39995	2.31958	1.57537	BGIOSGA029431	XP_006662125.2 serine carboxypeptidase-like 13 isoform X1
TCONS_00036989	1.40057	1.08619	1.57158	BGIOSGA018492	XP_003563208.1uncharacterized protein LOC100828168
TCONS_00019673	1.40122	1.81633	0.491988	BGIOSGA004393	XP_003568045.1chaperone protein dnaJ C76, chloroplastic
TCONS_00009464	1.40146	2.08476	0.692427	BGIOSGA005974	XP_006649526.1 GEM-like protein 1
TCONS_00029884	1.40169	-0.309631	0.67266	BGIOSGA032097	XP_015696316.1 probable LRR receptor-like serine/threonine-protein kinase At1g05700
TCONS_00016686	1.40187	#NA	0.599351	BGIOSGA028054	XP_006652878.1PREDICTED: uncharacterized protein LOC102721356
TCONS_00018645	1.40213	2.29051	-0.047128	BGIOSGA011921	XP_002440610.1REF/SRPP-like protein OsI_017815
TCONS_00023368	1.40236	0.718597	0.878764	BGIOSGA035061	XP_015694929.1 NAC domain-containing protein 67-like
TCONS_00008884	1.40311	1.33694	1.00971	BGIOSGA037301	XP_003564116.1probable tyrosine-protein phosphatase DSP2
TCONS_00006535	1.40315	1.22196	0.739174	BGIOSGA008703	XP_004953270.1thylakoid lumenal protein TL20.3, chloroplastic
TCONS_00006907	1.40319	0.978807	-0.862181	BGIOSGA008985	XP_015688741.1 protein CURVATURE THYLAKOID 1D, chloroplastic-like
TCONS_00001962	1.40421	2.18355	1.22931	#N/A	YP_002000494.1ATP synthase CF1 beta subunit (chloroplast)
TCONS_00030035	1.40466	0.574564	0.902498	BGIOSGA017499	XP_006660617.1 calmodulin-like protein 2
TCONS_00016292	1.40514	1.40032	1.72299	BGIOSGA034104	XP_024311358.1mitochondrial carrier protein MTM1
TCONS_00010912	1.40516	1.08538	-0.174948	BGIOSGA005553	XP_015690826.1 serine hydroxymethyltransferase, mitochondrial
TCONS_00014212	1.40586	1.71198	2.22517	BGIOSGA000542	XP_006652266.1 insulinoma-associated protein 1-like
TCONS_00020163	1.40671	3.09793	2.80801	BGIOSGA021847	XP_006656663.1 hsp70-Hsp90 organizing protein 1-like
TCONS_00009329	1.40769	1.51173	1.15021	BGIOSGA011873	XP_006651049.1 transcription factor-like protein DPB
TCONS_00033552	1.40824	1.22546	1.29997	BGIOSGA035342	XP_004962904.1uncharacterized protein LOC101758071 isoform X2
TCONS_00004337	1.40857	0.558313	0.773628	BGIOSGA017063	XP_004969837.1probable WRKY transcription factor 65
TCONS_00033057	1.4097	1.14654	0.994788	BGIOSGA034890	XP_004963245.1receptor kinase-like protein Xa21
TCONS_00031995	1.41028	2.42786	1.86341	BGIOSGA032153	XP_006661695.1 glycine-rich RNA-binding protein 2-like
TCONS_00001171	1.41075	1.5497	1.24697	BGIOSGA004305	XP_006644298.1 graves disease carrier protein homolog
TCONS_00016991	1.41093	-0.583956	0.244014	BGIOSGA019039	XP_015692882.1 metallothionein-like protein 2C
TCONS_00028112	1.41108	1.63722	-0.216651	BGIOSGA005545	XP_006659472.1 serine/threonine-protein kinase At5g01020
TCONS_00016653	1.41148	0.406837	0.482464	BGIOSGA014308	XP_006653788.1 chloroplastic lipocalin
TCONS_00012830	1.41156	-0.0696511	-1.33332	BGIOSGA012010	XP_004955502.1flavonoid 3'-monooxygenase CYP75B3
TCONS_00013277	1.41495	1.10094	0.166075	BGIOSGA018806	XP_003558244.1AP-1 complex subunit sigma-1
TCONS_00028060	1.41537	-0.918388	0.171941	BGIOSGA026004	XP_006659431.1 chlorophyll a-b binding protein P4, chloroplastic
TCONS_00028771	1.41581	0.741802	-1.19864	BGIOSGA025070	XP_003577991.1probable protein phosphatase 2C 68

TCONS_00033469	1.41607	1.62339	1.41374	BGIOSGA025468	NP_001148486.1DNA binding protein
TCONS_00024102	1.4164	2.44662	2.94512	BGIOSGA033615	XP_015695394.1 oligouridylate-binding protein 1-like isoform X2
TCONS_00017001	1.41658	-0.141935	-1.14256	BGIOSGA005890	XP_006653969.1 actin-depolymerizing factor 7
TCONS_00020789	1.42015	1.65896	1.04169	BGIOSGA013379	XP_006656127.1 reticulon-like protein B2
TCONS_00014158	1.42039	0.661433	1.5368	BGIOSGA004975	XP_015691718.1 centromere protein S
TCONS_00028335	1.421	1.48547	2.19163	BGIOSGA035636	XP_006659601.1 signal recognition particle receptor subunit beta-like isoform X2
TCONS_00023600	1.42112	1.41171	0.832142	BGIOSGA024375	XP_004984875.160S ribosomal protein L44
TCONS_00005523	1.42121	0.0609562	0.218596	BGIOSGA015161	XP_004952866.1uncharacterized protein LOC101763013
TCONS_00001438	1.42328	2.17473	0.223964	BGIOSGA011413	XP_006644415.1 scopoletin glucosyltransferase-like
TCONS_00000778	1.4233	-0.513934	0.218223	BGIOSGA024077	XP_003567617.1caffeoylshikimate esterase
TCONS_00008362	1.42336	1.65597	0.956096	BGIOSGA018165	XP_006647499.1 enhancer of rudimentary homolog
TCONS_00023501	1.42356	1.70954	1.78938	BGIOSGA025536	XP_006658482.1 protein TIC 22-like, chloroplastic
TCONS_00031524	1.42522	1.60871	0.219752	BGIOSGA031599	XP_006661985.1 probable glutathione S-transferase GSTU6
TCONS_00024183	1.42907	-0.0456515	0.851817	BGIOSGA020534	XP_006658016.1 novel plant SNARE 13
TCONS_00009726	1.42933	2.53444	1.29432	BGIOSGA012275	XP_006649794.1 selenoprotein K
TCONS_00006459	1.4295	1.79942	0.526166	BGIOSGA008631	XP_006647522.1 39S ribosomal protein L41-A, mitochondrial-like
TCONS_00035911	1.42997	1.8173	1.30766	#N/A	#N/A
TCONS_00016168	1.43049	0.807109	1.49692	BGIOSGA019832	XP_006652462.1 chitinase 5-like
TCONS_00012242	1.43137	2.41379	1.69616	BGIOSGA018317	XP_015689885.1 Fanconi anemia group I protein
TCONS_00015814	1.4317	#NA	-1.99798	BGIOSGA022286	XP_004975477.1protein PYRICULARIA ORYZAE RESISTANCE 21
TCONS_00019664	1.43262	-3.07306	-0.0882265	BGIOSGA007865	XP_002441416.1anthocyanidin 5,3-O-glucosyltransferase
TCONS_00022225	1.43284	1.17964	0.906059	BGIOSGA021272	XP_004953839.1cysteine-rich repeat secretory protein 60-like
TCONS_00009211	1.43381	1.59345	0.166684	BGIOSGA016876	XP_015690340.1 B3 domain-containing protein Os03g0120900
TCONS_00003655	1.43387	1.03713	-0.0657748	BGIOSGA001549	XP_004969790.1uncharacterized protein LOC101776777
TCONS_00030171	1.43656	0.98231	1.51351	BGIOSGA001540	XP_006660705.1 probable protein phosphatase 2C 69
TCONS_00003275	1.43925	-2.1065	-1.70272	BGIOSGA020575	XP_004967765.1ADP-ribosylation factor 2
TCONS_00004651	1.44201	1.99856	-0.594075	BGIOSGA026724	XP_015688247.1 sodium-coupled neutral amino acid transporter 5-like isoform X1
TCONS_00015204	1.44364	1.56777	1.69613	BGIOSGA033153	XP_006653054.1 ubiquitin-conjugating enzyme E2 variant 1C-like
TCONS_00029160	1.4451	2.74339	2.76084	BGIOSGA024937	NP_001142366.1EF hand family
TCONS_00011876	1.44553	2.58566	0.185793	#N/A	#N/A
TCONS_00016551	1.44657	0.822144	0.546864	BGIOSGA023325	NP_001150571.1EF hand family protein
TCONS_00032242	1.44868	-3.72818	0.675412	BGIOSGA009727	XP_020402849.1protein MARD1
TCONS_00021550	1.4488	1.27167	2.76258	BGIOSGA022001	NP_001149448.1rad1-like protein
TCONS_00018120	1.44997	2.5556	3.05593	BGIOSGA000774	XP_006655478.1 mitochondrial import inner membrane translocase subunit TIM50-like
TCONS_00011885	1.45164	#NA	1.45808	BGIOSGA029269	XP_006651212.1 pathogenesis-related protein 5-like
TCONS_00032613	1.45171	2.17489	2.42278	BGIOSGA016388	XP_015697173.1 probable serine/threonine-protein kinase At1g01540
TCONS_00025599	1.45558	1.37025	1.74203	BGIOSGA023833	XP_006658031.1 protein N-methyltransferase nnt1
TCONS_00018431	1.45595	3.25504	1.8453	BGIOSGA027057	XP_006654883.2 UDP-glucose 4-epimerase 1
TCONS_00009239	1.45694	0.357626	-0.243427	BGIOSGA030174	XP_004985853.1benzoate--CoA ligase, peroxisomal
TCONS_00027492	1.45939	1.33778	1.28119	BGIOSGA018486	XP_006659200.1 lactoylglutathione lyase
TCONS_00002163	1.46076	1.64898	-0.886076	BGIOSGA017927	XP_004970564.1uncharacterized protein LOC101771906
TCONS_00001137	1.46082	1.74681	1.90972	BGIOSGA003762	XP_021313209.1PITH domain-containing protein 1
TCONS_00032931	1.46154	2.2815	1.55219	BGIOSGA036960	XP_021317106.1uncharacterized protein LOC8085629
TCONS_00028424	1.46187	2.60057	2.68392	BGIOSGA029408	XP_006659654.1 plant UBX domain-containing protein 8-like isoform X1
TCONS_00033980	1.46218	0.704799	1.26164	BGIOSGA035666	XP_015698951.1 calmodulin-binding protein 60 D-like
TCONS_00004774	1.4632	2.59182	1.14123	BGIOSGA000440	XP_006646501.1 protein YLS9-like
TCONS_00037566	1.46323	0.470786	-1.51569	BGIOSGA005890	XP_006664223.1 actin-depolymerizing factor 11
TCONS_00016624	1.46326	1.48999	1.36615	BGIOSGA018529	XP_003580557.2trinucleotide repeat-containing gene 18 protein
TCONS_00028596	1.4652	-0.393979	0.437464	BGIOSGA030342	XP_006660439.1 E3 ubiquitin-protein ligase RNF181-like
TCONS_00026684	1.46531	-0.21304	0.361031	BGIOSGA029432	XP_002439828.1putative clathrin assembly protein At1g03050
TCONS_00032141	1.46647	0.52207	0.803472	BGIOSGA024807	XP_012704263.1protein TIFY 11d
TCONS_00015165	1.46722	0.0324812	1.39581	BGIOSGA020335	XP_004960079.1uncharacterized protein LOC101771182
TCONS_00025332	1.46753	-1.12121	-0.0284487	BGIOSGA030566	XP_006657829.1 chlorophyll a-b binding protein CP29.1, chloroplastic-like
TCONS_00025781	1.46957	-0.449402	0.363391	BGIOSGA027792	XP_015695470.1 indole-2-monooxygenase-like
TCONS_00030724	1.47162	1.63489	0.672313	BGIOSGA009509	XP_004967583.1uncharacterized protein LOC101770560
TCONS_00036480	1.47162	2.1185	1.02984	BGIOSGA033027	XP_006664229.1 ras-related protein RABF2b
TCONS_00027531	1.47338	0.804694	0.113626	BGIOSGA027483	XP_006659205.1 photosystem II 10 kDa polypeptide, chloroplastic
TCONS_00020324	1.474	-1.36798	#NA	BGIOSGA013573	XP_015693983.1PREDICTED: uncharacterized protein LOC107304429

TCONS_00033890	1.47474	0.2161	0.664628	BGIOSGA021165	XP_002448565.1putative disease resistance protein At1g50180
TCONS_00014527	1.47497	2.2001	2.0697	BGIOSGA016691	XP_006652468.1 splicing factor U2AF-associated protein 2
TCONS_00000229	1.47575	1.54202	1.93151	BGIOSGA002805	XP_006645505.1 putative receptor-like protein kinase At3g47110
TCONS_00004215	1.47593	1.54177	2.18945	BGIOSGA031679	XP_004969619.1probable glutathione S-transferase GSTU6
TCONS_00024361	1.47728	1.16161	1.76935	BGIOSGA018323	XP_015694771.1 mediator of RNA polymerase II transcription subunit 33A-like
TCONS_00001649	1.47764	0.196334	0.107881	BGIOSGA000974	XP_006644578.1 farnesyl pyrophosphate synthase
TCONS_00028683	1.47779	1.74243	0.62823	BGIOSGA025977	XP_021310179.1uncharacterized protein LOC8063475 isoform X2
TCONS_00017086	1.4783	0.983228	1.1827	BGIOSGA001769	XP_006654018.1 S-adenosylmethionine synthase 1
TCONS_00031515	1.47855	1.79576	1.21672	BGIOSGA018056	XP_004983851.1probable glutathione S-transferase GSTU6
TCONS_00013120	1.4788	1.54356	1.4692	BGIOSGA019686	XP_006650542.1PREDICTED: uncharacterized protein LOC102704785
TCONS_00002602	1.47965	0.721123	0.000980655	BGIOSGA007416	XP_006645348.1 probable Histone-lysine N-methyltransferase ATXR5
TCONS_00004864	1.48001	0.740593	1.65157	BGIOSGA010193	XP_015695199.1PREDICTED: uncharacterized protein LOC102702898
TCONS_00021707	1.48025	0.868724	2.52752	BGIOSGA024622	XP_015693741.1 probable galactinol--sucrose galactosyltransferase 6
TCONS_00009320	1.48226	-0.377336	0.0610564	BGIOSGA011864	XP_006649402.1 mitochondrial inner membrane protease subunit 2-like
TCONS_00018180	1.48322	2.04085	1.21272	BGIOSGA030901	XP_006654692.1 heat stress transcription factor A-4d-like
TCONS_00014478	1.485	1.63465	1.33841	BGIOSGA003890	NP_001146954.1acid phosphatase/vanadium-dependent haloperoxidase related
TCONS_00029097	1.48566	0.0824071	0.740689	BGIOSGA030847	NP_001143544.1uncharacterized LOC100276234
TCONS_00000588	1.4858	1.10888	1.12331	BGIOSGA018039	XP_006644009.1 bifunctional epoxide hydrolase 2-like
TCONS_00011056	1.48618	3.15474	3.00432	BGIOSGA034482	XP_019082114.1 mitochondrial import inner membrane translocase subunit TIM14-3 isoform X2
TCONS_00009666	1.48927	2.07803	1.9307	BGIOSGA012210	XP_015690365.1 cell cycle checkpoint protein RAD17
TCONS_00034753	1.49263	3.6116	1.03508	BGIOSGA017668	XP_010237735.1protein PEP-RELATED DEVELOPMENT ARRESTED 1 homolog, chloroplastic isoform X2
TCONS_00004114	1.49345	0.0223303	0.839508	BGIOSGA020384	NP_001144147.2uncharacterized LOC100277000
TCONS_00025662	1.49464	0.931329	0.0299867	#N/A	#N/A
TCONS_00010965	1.49634	0.809123	0.5111	#N/A	#N/A
TCONS_00008486	1.49738	-3.99358	-0.19997	BGIOSGA014634	XP_006647605.1 aldehyde dehydrogenase family 3 member F1-like
TCONS_00014911	1.49758	0.314838	-0.389716	BGIOSGA025263	XP_004976772.1protein ECERIFERUM 26-like
TCONS_00011820	1.49779	-0.979138	-0.978003	BGIOSGA017720	XP_006649662.1 non-symbiotic hemoglobin 2
TCONS_00012024	1.49918	4.27661	3.92536	BGIOSGA027688	XP_006649860.1 glycine-rich RNA-binding protein 4, mitochondrial
TCONS_00003052	1.50019	3.11542	1.87802	BGIOSGA002127	XP_003565227.1protein IWS1 homolog A
TCONS_00001291	1.50048	-1.52188	0.816546	BGIOSGA005595	XP_015697347.1 chlorophyll a-b binding protein 2, chloroplastic
TCONS_00032849	1.50049	3.40997	3.01515	BGIOSGA036873	XP_014757503.1Holliday junction resolvase MOC1, chloroplastic isoform X2
TCONS_00007519	1.5015	1.50828	1.71304	BGIOSGA000733	XP_004951601.1uncharacterized protein LOC101757461
TCONS_00001939	1.50209	1.22214	1.59605	BGIOSGA004578	XP_015692278.1 frataxin, mitochondrial isoform X2
TCONS_00030450	1.5033	2.68661	2.32998	BGIOSGA029352	XP_006660912.2 la-related protein 1B
TCONS_00031512	1.50461	-3.4025	-0.995248	BGIOSGA005170	XP_002467616.1probable glutathione S-transferase GSTU6
TCONS_00002097	1.50474	2.08638	1.80526	BGIOSGA004725	XP_002456567.1exocyst complex component EXO70B1
TCONS_00024415	1.50593	-2.0826	-0.563184	BGIOSGA010964	XP_006659007.2 two-component response regulator-like PRR37 isoform X1
TCONS_00033952	1.50602	1.886	2.15087	BGIOSGA000164	XP_006663643.2 lipase-like
TCONS_00034145	1.50717	2.31834	2.49291	BGIOSGA033600	XP_015697826.1 RNA-directed DNA methylation 4-like isoform X1
TCONS_00024164	1.50858	2.18066	1.97871	BGIOSGA010652	XP_021309721.1serine/arginine-rich SC35-like splicing factor SCL33 isoform X2
TCONS_00031152	1.50894	2.11559	2.02958	BGIOSGA015076	XP_006662368.1 BTB/POZ and MATH domain-containing protein 1-like
TCONS_00020421	1.50922	2.34657	1.31519	BGIOSGA019733	XP_003560867.1metalloendoproteinase 3-MMP
TCONS_00018014	1.50952	2.46577	1.31445	BGIOSGA020062	XP_015692801.1 pentatricopeptide repeat-containing protein At1g11900-like
TCONS_00001377	1.51041	1.49706	1.63416	BGIOSGA004007	XP_006646110.1 chitinase domain-containing protein 1
TCONS_00008182	1.51102	0.619946	-1.55274	BGIOSGA014906	XP_003575145.1protein MAINTENANCE OF PSII UNDER HIGH LIGHT 1
TCONS_00005716	1.51485	2.18294	1.61184	BGIOSGA007833	XP_006647096.1 protein phosphatase 1 regulatory subunit pprA
TCONS_00003109	1.51515	0.790808	0.774594	BGIOSGA005908	XP_015698093.1PREDICTED: uncharacterized protein LOC107305334
TCONS_00004551	1.51599	0.984559	0.255363	BGIOSGA024189	NP_001150984.1GOS2 homolog
TCONS_00029225	1.51821	1.89044	1.80924	BGIOSGA030967	XP_015696676.1 ribosomal RNA small subunit methyltransferase B isoform X1
TCONS_00032378	1.51998	1.44378	2.37964	BGIOSGA002504	XP_006662421.1PREDICTED: uncharacterized protein LOC102709961
TCONS_00022992	1.52071	0.738074	1.24183	BGIOSGA030827	XP_006655880.1 2Fe-2S ferredoxin-like isoform X1

TCONS_00030240	1.52125	1.35496	1.76265	BGIOSGA007782	XP_006660751.1 ribonuclease Z, chloroplastic
TCONS_00023249	1.5213	2.4799	1.70784	BGIOSGA028692	XP_006657517.1 chromatin remodeling protein EBS-like isoform X2
TCONS_00030912	1.52189	0.71029	1.12365	BGIOSGA011371	XP_006661700.1PREDICTED: uncharacterized protein LOC102718110
TCONS_00011671	1.52379	1.30236	1.37702	BGIOSGA011337	XP_004985495.1cation/calcium exchanger 4
TCONS_00003795	1.52383	2.1795	1.19684	BGIOSGA001416	XP_015694338.1PREDICTED: LOW QUALITY PROTEIN: uncharacterized protein LOC102701407
TCONS_00019729	1.52388	1.03101	0.505402	BGIOSGA032579	XP_004963626.1mitogen-activated protein kinase kinase kinase 17
TCONS_00026677	1.524	0.40742	-0.153872	BGIOSGA004740	XP_003574538.1CBL-interacting protein kinase 6
TCONS_00023744	1.52401	2.60781	1.37062	BGIOSGA025778	XP_015694648.1 methionine aminopeptidase 1B, chloroplastic
TCONS_00035841	1.52467	-0.0976798	1.86154	#N/A	#N/A
TCONS_00032161	1.52492	2.7267	1.78825	BGIOSGA002207	XP_006302487.1solute carrier family 25 member 44
TCONS_00010465	1.52682	0.11387	-0.495209	BGIOSGA021192	XP_010232929.1ent-cassadiene C2-hydroxylase
TCONS_00018738	1.52723	1.60841	1.76471	BGIOSGA018679	NP_001147566.1bifunctional protein tilS/hprT
TCONS_00024186	1.5287	2.39373	0.412414	BGIOSGA011658	XP_002461097.1uncharacterized protein LOC8063537
TCONS_00025373	1.52888	-0.923604	1.80747	BGIOSGA038015	XP_006657862.1 L-type lectin-domain containing receptor kinase IX.1
TCONS_00024355	1.53249	2.76619	3.0312	BGIOSGA025968	XP_015695358.1 alpha-galactosidase 3-like
TCONS_00000014	1.53382	-0.470678	-0.506118	BGIOSGA003461	XP_006643631.1PREDICTED: uncharacterized protein LOC102702501
TCONS_00015413	1.53729	-0.54628	0.0464852	#N/A	#N/A
TCONS_00029713	1.54037	1.03139	0.0841827	BGIOSGA033493	XP_004956437.1heavy metal-associated isoprenylated plant protein 46
TCONS_00032319	1.54171	0.882023	-0.208706	#N/A	#N/A
TCONS_00012176	1.54281	1.00156	0.90293	BGIOSGA010833	XP_008655209.1chaperone protein dnaJ 20, chloroplastic
TCONS_00017455	1.54323	1.50632	1.51809	BGIOSGA019530	XP_006655161.1 DNA mismatch repair protein MSH2
TCONS_00009762	1.54336	2.55693	2.86537	BGIOSGA034614	XP_006649831.1 protein TOC75, chloroplastic
TCONS_00034424	1.54345	-0.0376706	0.902364	BGIOSGA015174	XP_021311172.1putrescine hydroxycinnamoyltransferase 1
TCONS_00024027	1.54466	2.72119	0.801071	BGIOSGA032725	XP_006657908.1 vesicle transport protein GOT1B-like
TCONS_00004308	1.54595	1.73688	0.229026	BGIOSGA001283	XP_006644635.1 inorganic pyrophosphatase 2
TCONS_00027002	1.54756	0.0729623	-1.45567	BGIOSGA008983	XP_012702155.1MADS-box transcription factor 51
TCONS_00010865	1.54777	2.85305	1.89155	BGIOSGA013486	XP_004981930.1maf-like protein DDB_G0281937
TCONS_00010483	1.54941	2.31862	2.47319	BGIOSGA013087	XP_006650283.1 fructokinase-like 2, chloroplastic
TCONS_00013677	1.54952	0.222171	0.522923	BGIOSGA009328	XP_006648155.1 V-type proton ATPase subunit F
TCONS_00008643	1.5504	1.26897	0.65157	BGIOSGA030840	XP_015688706.1 trihelix transcription factor GT-3b-like
TCONS_00001475	1.55093	-0.646044	0.563831	BGIOSGA004111	XP_006644440.1 GDSL esterase/lipase At1g28600-like isoform X2
TCONS_00022945	1.55149	-0.134196	-0.184461	BGIOSGA005828	XP_006656522.1 protein REVEILLE 1-like
TCONS_00029753	1.55181	0.536306	2.2159	BGIOSGA011963	XP_015696560.1 ethylene-responsive transcription factor RAP2-2-like
TCONS_00014323	1.55232	1.51296	1.53787	BGIOSGA016487	XP_006652333.1PREDICTED: uncharacterized protein LOC102722570
TCONS_00005378	1.55299	-0.504316	-1.81602	BGIOSGA020573	XP_004951946.1uncharacterized protein LOC101760180
TCONS_00000489	1.5559	0.188984	0.866103	BGIOSGA025184	XP_015697561.1 acyl-coenzyme A thioesterase 13-like
TCONS_00009314	1.55596	0.670901	0.295731	BGIOSGA014024	XP_006649395.1 ras-related protein RABC2a
TCONS_00003621	1.55787	1.43815	0.0537761	BGIOSGA001580	XP_006644194.1 dual specificity protein phosphatase 1B-like isoform X2
TCONS_00037279	1.56005	2.97212	2.2223	BGIOSGA029594	XP_015698315.1 heat shock protein 90-6, mitochondrial
TCONS_00027857	1.56038	2.11173	2.31698	BGIOSGA027148	XP_010234639.1grpE protein homolog 2, mitochondrial isoform X1
TCONS_00024734	1.56056	0.90124	1.80018	BGIOSGA033161	XP_004982785.1thioredoxin-like protein YLS8
TCONS_00020949	1.56068	-5.02229	-0.160554	BGIOSGA023171	XP_006657094.1PREDICTED: uncharacterized protein LOC102705731
TCONS_00033771	1.56075	2.17337	0.791219	BGIOSGA028020	XP_015697712.1 probable plastid-lipid-associated protein 6, chloroplastic
TCONS_00001349	1.56119	0.224248	0.318396	BGIOSGA003981	XP_006644363.2 nascent polypeptide-associated complex subunit alpha, muscle-specific form-like
TCONS_00023905	1.56292	-0.256581	-1.50857	BGIOSGA039436	XP_006658311.1 short-chain type dehydrogenase/reductase
TCONS_00002767	1.563	0.732719	2.26327	BGIOSGA026063	XP_002457427.1zinc finger protein GIS3
TCONS_00027225	1.56356	1.25034	-0.540384	BGIOSGA027779	XP_006659039.1 probable adenylate kinase 2, chloroplastic
TCONS_00002669	1.56467	-3.72882	-0.518488	BGIOSGA002047	XP_006643646.2 isoflavone reductase homolog IRL-like
TCONS_00012235	1.56478	-0.0837218	2.12212	BGIOSGA002159	XP_006650055.1 CBL-interacting protein kinase 10
TCONS_00008990	1.56517	2.35801	1.13234	BGIOSGA035136	XP_017700379.1PREDICTED: uncharacterized protein LOC103715899
TCONS_00010999	1.56518	2.72448	1.44768	BGIOSGA001610	XP_002466416.2isoflavone 2'-hydroxylase
TCONS_00031649	1.56552	0.855064	1.75949	BGIOSGA008471	XP_008658496.1RING-H2 finger protein ATL5
TCONS_00011559	1.567	2.3131	2.30303	BGIOSGA021615	XP_006649396.1 probable aquaporin TIP1-1
TCONS_00014115	1.56772	0.609881	2.31478	BGIOSGA016106	XP_008806429.1 MADS-box transcription factor 34-like isoform X3

TCONS_00026898	1.56774	0.304535	0.948791	BGIOSGA006687	XP_015695656.1 zinc finger A20 and AN1 domain-containing stress-associated protein 11-like
TCONS_00023956	1.57063	-0.631813	2.53247	BGIOSGA018731	NP_001152099.1carboxyl-terminal peptidase precursor
TCONS_00000696	1.57226	0.79397	2.09499	BGIOSGA028986	XP_004971532.1uncharacterized protein LOC101785548
TCONS_00023871	1.57251	1.33736	0.979586	BGIOSGA025903	XP_015694673.1 pentatricopeptide repeat-containing protein At4g18975, chloroplastic
TCONS_00024203	1.57296	1.87833	1.06064	BGIOSGA032046	XP_003559895.1putative SWI/SNF-related matrix-associated actin-dependent regulator of chromatin subfamily A member 3-like 1
TCONS_00012787	1.57506	0.0568545	2.98188	BGIOSGA031695	XP_015689985.1 vacuolar protein sorting-associated protein 2 homolog 2
TCONS_00010748	1.57589	0.622097	1.28215	BGIOSGA014352	XP_006650433.1 50S ribosomal protein L18-like
TCONS_00006753	1.57611	1.15814	1.02669	BGIOSGA008922	NP_001339112.1uncharacterized LOC100193276
TCONS_00027887	1.57674	0.541816	1.78922	BGIOSGA023451	XP_006659328.1 bifunctional nuclease 2 isoform X1
TCONS_00031653	1.57723	2.19136	2.49507	BGIOSGA033469	XP_006662081.1 uncharacterized mitochondrial protein ymf11
TCONS_00021558	1.57745	2.58699	1.57682	BGIOSGA021990	XP_015693416.1PREDICTED: uncharacterized protein LOC102719302 isoform X1
TCONS_00004619	1.57842	1.10097	1.10474	BGIOSGA004951	XP_015693359.1 NAC domain-containing protein 68-like
TCONS_00010025	1.58005	1.36765	2.23716	BGIOSGA015701	XP_006650051.2 macro domain-containing protein VPA0103
TCONS_00012186	1.58064	0.441539	0.102676	BGIOSGA025370	XP_006651339.1 thioredoxin-like 1-2, chloroplastic
TCONS_00018755	1.58073	1.83898	1.40994	BGIOSGA018666	XP_006654127.1 derlin-1
TCONS_00005923	1.58133	2.4475	1.38133	BGIOSGA023512	XP_003573011.1aspartyl protease family protein 2
TCONS_00010058	1.58145	-1.31075	-0.216732	#N/A	#N/A
TCONS_00019427	1.58178	1.27544	0.157808	BGIOSGA004795	XP_021312038.1ultraviolet-B receptor UVR8 isoform X3
TCONS_00006526	1.5819	1.05774	0.812407	BGIOSGA008694	XP_015689380.1PREDICTED: uncharacterized protein LOC102706813
TCONS_00037276	1.58245	1.55497	1.04497	BGIOSGA024011	XP_010237508.1stress-associated endoplasmic reticulum protein 2
TCONS_00011365	1.58276	1.04823	1.10458	BGIOSGA013981	XP_015690155.1PREDICTED: uncharacterized protein LOC107303360
TCONS_00033456	1.58334	0.614192	0.456191	BGIOSGA009628	XP_006662935.1 TPD1 protein homolog 1B-like
TCONS_00018349	1.58668	1.5402	1.4222	BGIOSGA031185	XP_015692706.1 protein ROOT PRIMORDIUM DEFECTIVE 1
TCONS_00018058	1.58711	1.03754	1.65997	BGIOSGA000642	XP_006655445.2 probable glycerol-3-phosphate dehydrogenase [NAD(+)] 3, cytosolic
TCONS_00006807	1.5872	2.58524	2.22134	BGIOSGA008982	XP_015688938.1 sex-lethal homolog
TCONS_00000643	1.58733	2.51484	2.43895	BGIOSGA003228	XP_003566861.1Holliday junction resolvase MOC1, chloroplastic
TCONS_00033757	1.58797	1.19411	1.61437	BGIOSGA033690	XP_006663017.1 barwin-like
TCONS_00002539	1.58897	1.58582	1.66639	BGIOSGA024374	XP_006645383.1 two-component response regulator ORR4-like
TCONS_00021092	1.58904	0.344906	1.24908	BGIOSGA005527	XP_015697608.1 probable sucrose-phosphate synthase 5
TCONS_00002732	1.59026	1.47139	0.279996	BGIOSGA002490	XP_006643707.1PREDICTED: uncharacterized protein LOC102701761
TCONS_00015894	1.59141	-4.21821	-0.118644	BGIOSGA030777	XP_006653401.1 putative cellulose synthase-like protein H3
TCONS_00017802	1.59254	2.64647	0.439081	BGIOSGA024777	XP_004962100.1probable carboxylesterase 17
TCONS_00028730	1.59323	-5.15182	-0.215242	BGIOSGA030483	XP_006660522.1 protein TPX2
TCONS_00026893	1.59338	0.844955	0.781444	BGIOSGA001444	XP_006659564.1 tonoplast dicarboxylate transporter-like
TCONS_00012204	1.59362	1.74219	1.15266	BGIOSGA010803	XP_006650035.1 hydroxyacylglutathione hydrolase cytoplasmic
TCONS_00018272	1.59489	0.696043	1.50917	BGIOSGA002655	XP_015693335.1 CDP-diacylglycerol--serine O-phosphatidyltransferase 2 isoform X1
TCONS_00001391	1.5953	2.65008	1.72631	BGIOSGA003628	XP_006644387.1 protein SGT1 homolog
TCONS_00031012	1.59651	1.52798	2.13028	BGIOSGA011316	XP_006662309.2 protein CutA 1, chloroplastic
[truncated: 157,834 more chars]
